# Comprehensive analysis of a necroptosis-associated diagnostic signature for myelodysplastic syndromes based on single-cell RNA-seq and bulk RNA-seq

**DOI:** 10.1186/s41065-024-00335-x

**Published:** 2024-10-15

**Authors:** Huimin Zhang, Li Zhang, Xiaoning Liang, Lihong Zhang, Bing Ma, Yuexian Li, Jianying Wang, Yang Shen, Yuhui Pang, Jianjun Xiong

**Affiliations:** 1 Department of Hematology, the First Hospital of Hebei Medical University, Shijiazhuang, China; 2 Department of Hematology, Shijiazhuang Ping’an Hospital, Shijiazhuang, China

**Keywords:** Myelodysplastic syndromes, Necroptosis, Immune cell infiltration, Prognosis

## Abstract

**Background:**

Myelodysplastic syndromes (MDS) are heterogeneous and clonal hematological disorders. The role and mechanism of necroptosis in MDS remain poorly understood.

**Methods:**

mRNA expression profiles and single-cell RNA-sequencing (scRNA-seq) data were sourced from the GEO database. ScRNA-seq data were processed using the “Seurat” package. After cell annotation, necroptosis-related scores (NRscores) for each cell were calculated using the “UCell” package. Differentially expressed genes (DEGs) and their associated biological functions in NRscore-related cell populations were identified. Additionally, DEGs and necroptosis-related genes (DE-NRGs) between MDS patients and healthy controls were identified. Consensus clustering was employed to classify MDS patients into distinct subclusters based on DE-NRGs. The biological functions and immune characteristics of these classifications were analyzed. Prognostic gene signatures were determined using LASSO and SVM-RFE analyses, and a nomogram was constructed based on the prognostic gene signature.

**Results:**

A total of 12 cell types were identified in MDS and healthy controls. NRscore was found to be elevated in monocytes and common lymphoid precursors (CLPs). Enrichment analysis revealed that monocytes and CLPs with high NRscore were associated with mitochondria-related and immune-related pathways. Eleven DEGs in monocytes and CLPs between MDS patients and healthy controls were identified. Additionally, 13 DE-NRGs were identified from 951 DEGs between MDS and healthy controls. MDS patients were classified into two distinct subclusters based on these 13 DE-NRGs, revealing several immune-related processes and signaling pathways. Differences in immune subpopulations between the two subclusters were observed. A necroptosis-related diagnostic gene signature (IRF9, PLA2G4A, MLKL, BAX, JAK2, and STAT3) was identified as predictive of MDS prevalence.

**Conclusion:**

Necroptosis plays a role in MDS progression by inducing inflammation. A novel necroptotic gene signature has been developed to distinguish and diagnose MDS at early stages of the disease.

**Supplementary Information:**

The online version contains supplementary material available at 10.1186/s41065-024-00335-x.

## Introduction

Myelodysplastic syndromes (MDS) are classified as myeloid neoplasms and are characterized by clonal proliferation of hematopoietic stem cells, recurrent genetic abnormalities, myelodysplasia, disorders of hematopoiesis, peripheral cytopenia, and a high risk of progression to acute myeloid leukemia (AML) [1]. MDS is traditionally categorized into primary MDS, which occurs without a known history of therapy, and therapy-related MDS, which is a serious complication arising from chemotherapy [2, 3].

Most MDS cases are associated with structural chromosomal alterations and somatic mutations in hematopoietic stem cells and progenitor cells [1, 4, 5]. Several mutation-deriver genes contribute to the pathological processes of MDS and account for its heterogeneity [6–10]. Bone marrow failure and cytopenia in at least one hematological cell line are hallmarks of MDS. The revised International Prognostic Scoring System (R-IPSS) in 2012, is commonly used to classify MDS patients into high-risk and low-risk groups [11]. Current treatment strategies focus on improving the quality of life and preventing cytopenia for low-risk MDS while aiming to delay disease progression for high-risk patients [12, 13]. Despite the discovery of several promising drugs for MDS, the heterogeneity of MDS can complicate therapeutic outcomes. Consequently, there is a need to identify more sensitive and effective diagnostic and therapeutic markers for MDS.

MDS is complex and heterogeneous myeloid neoplasms with mechanisms leading to ineffective hematopoiesis that remain not fully understood. However, inflammation and excessive programmed cell death (PCD) have been identified in the progression of MDS [14]. Two prominent features of MDS include increased levels of inflammatory cytokines such as TNF-а, IFN-γ, TGF-β, IL-6, and IL-8 [15, 16], and increased inflammation-related bone marrow cell death [17, 18]. Previous studies have shown that hematopoietic cell death plays a significant role in the pathological process of low-risk MDS [19, 20]. Furthermore, inflammatory cytokines promote the proliferation and PCD of hematopoietic progenitors in MDS, indicating that immune disorder and inflammatory process act as pathogenic drivers of the disease [16, 21–23]. However, the immune characteristics of the bone marrow in MDS remain unknown.

In recent years, a novel inflammation-related form of programmed cell death called necroptosis has been identified in the context of immune response and autoimmunity. Necroptosis is triggered following the activation of the tumor necrosis receptor (TNFR1) by TNFα [24, 25]. This activation leads to the formation of a complex involving receptor-interacting protein kinase 1 (RIPK1), RIPK3, and mixed lineage kinase domain-like protein (MLKL), ultimately inducing necroptosis [26].

Necroptosis has been implicated in myelodysplasia and bone marrow failure, contributing to the pathologic progression of MDS [27–31]. However, the potential role and regulatory mechanism of necroptosis in MDS remain unclear.

Single-cell RNA sequencing (scRNA-seq) is a powerful strategy used to explore cell heterogeneity and elucidate the cellular dynamic processes of human hematopoiesis [32]. In the present study, we utilized transcriptomic data to explore the cellular landscape and analyze the expression of necroptosis-related genes (NRGs) in MDS and their related molecular classification. Furthermore, we explored the molecular function and immune characteristics of NRGs-related molecular classification and assessed the prognosis and predictive values of NRGs in MDS using LASSO, SVM-RFE, and nomogram models.

## Methods

### Data acquisition and processing

The expression profiles and corresponding clinical data for MDS patients and healthy controls from bone marrow CD34 cells were obtained from the Gene Expression Omnibus (GEO). The GSE58831 dataset includes 159 MDS patients and 17 health controls. The GSE19429 dataset includes 183 MDS patients and 17 health controls. The GSE4619 dataset includes 55 MDS patients and 11 health controls. All datasets were generated by Affymetrix Human Genome U133 Plus 2.0 Array. Additionally, scRNA-seq data for 8 MDS patients and 4 health controls were obtained from the GSE135194 datasets, which include bone marrow CD34 + hematopoietic stem and progenitor cells (HSPCs). The scRNA-seq data were generated using Illumina HiSeq 3000 (Homo sapiens).

Furthermore, a total of 159 NRGs were obtained from the Kyoto Encyclopedia of Genes and Genomes (KEGG, https://www.kegg.jp/kegg/) using the “KEGGREST” package in R.

### scRNA-seq data processing

The Seurat” R package was utilized for the analysis of scRNA-seq data. Cells were included in the analysis after filtering based on the following criteria: nCount_RNA > 1,000 and < 50,000, or nFeature_RNA > 1,000 and < 5,500, or percent.rb < 80%, or percent. mt < 10%. To mitigate the batch effect of sample identification, the “Harmony” R package was employed. The FindClusters function was applied to stratify all cells into distinct clusters, and cell clusters were annotated using the FindAllMarkers Function.

### Calculation of NRscore

The “UCell” R package was performed to calculate the necroptosis-related score (NRscore) for each cell. Subsequently, cells were classified into high- and low-NRscore groups based on the median NRscore value. The cell populations with significant NRscores were selected for further analysis. We identified the DEGs between MDS patients and health controls in high-NRscore cell populations using a threshold of |log_2_ FC| > 0.5. Additionally, we explored the gene ontology (GO) enrichment of these cell populations using the “clusterprofiler”. A zscore > 0 indicates the activation of the pathways in MDS patients compared to health controls.

### Screening of NRGs in MDS

The DEGs between MDS and health controls were screened using the “Limma” package in R with |log_2_FC| > 0.585 and *P*-value < 0.05. The differentially expressed NRGs (DE-NRGs) were then identified by overlapping the DEGs and 159 NRGs.

### Consensus clustering analysis

Consensus clustering was performed based on DE-NRGs using the “ConsensusClusterPlus” package in R [33]. The similarity within each group was measured by Euclidean distance with 1000 times repetitions. The optimal number of clusters (k) was determined using the cumulative distribution function (CDF) plot. The CDF plot indicated the number of consensuses and the stability of clustering, which was further verified using principal component analysis (PCA) and T-distributed neighbor embedding (T-SNE). Single sample Gene Set Enrichment Analysis (ssGSEA) was performed to detect the NRscore differences between clusters.

### GO and KEGG enrichment analysis

The GO annotation, including biological process, BP; molecular function, MF; cellular component, CC, as well as KEGG enrichment analysis, were performed using the “ClusterProfiler” package in R. The parameters were set as follows: *P*-value cutoff = 0.05, P adjusted Methods = “BH”, and q-value cutoff = 0.2.

### Immune cell infiltration analysis

Immune cell infiltration was analyzed using the “Immune-Oncology-Biological-Research (IOBR)” package in R, which integrates MCPcounter, TIMER, EPIC, CIBERSORT, xCELL, and ssGSEA methods [34]. The “IOBR” R package was used to analyze the differences in infiltrating immune cells between two MDS subclusters [35].

### Generation and validation of LASSO and SVM-RFE model models for feature gene selection

Two machine-learning algorithms were conducted to identify the diagnostic variables in MDS. The least absolute shrinkage and selection operator (LASSO) was performed using the “glmnet” package in R [36]. A support vector machine-recursive feature elimination (SVM‐RFE) model was developed using a “caret” package in R, incorporating 10-fold cross-validations as outlined by Sanz et al. [37]. For the SVM-FRE algorithm, the MDS samples in the GSE58831 dataset were divided into training and test sets in a 3:1 ratio to develop and validate the diagnostic model.

Subsequently, the candidate genes were obtained by overlapping the DEGs from scRNA-seq data analysis, and signature both from the LASSO and SVM-RFE algorithms. The diagnostic ability of the candidate genes was assessed using the receiver operating characteristic (ROC) curves, specifically by measuring the area under the curve (AUC). Furthermore, validation of the expression of the overlapping genes was performed using external datasets GSE19429 and GSE4619.

### Development of a diagnostic nomogram

A diagnostic model was developed using logistic regression analysis and visualized as a nomogram using the “rms” package in R. The calibration curve was drawn to evaluate the predictive accuracy. The decision curve was drawn using the “ggDCA” package in R to assess whether the decisions generated from the model were beneficial to the patients.

## Results

### Identification of the NRscore and relevant enriched pathways at the single-cell level

**Fig. 1:**
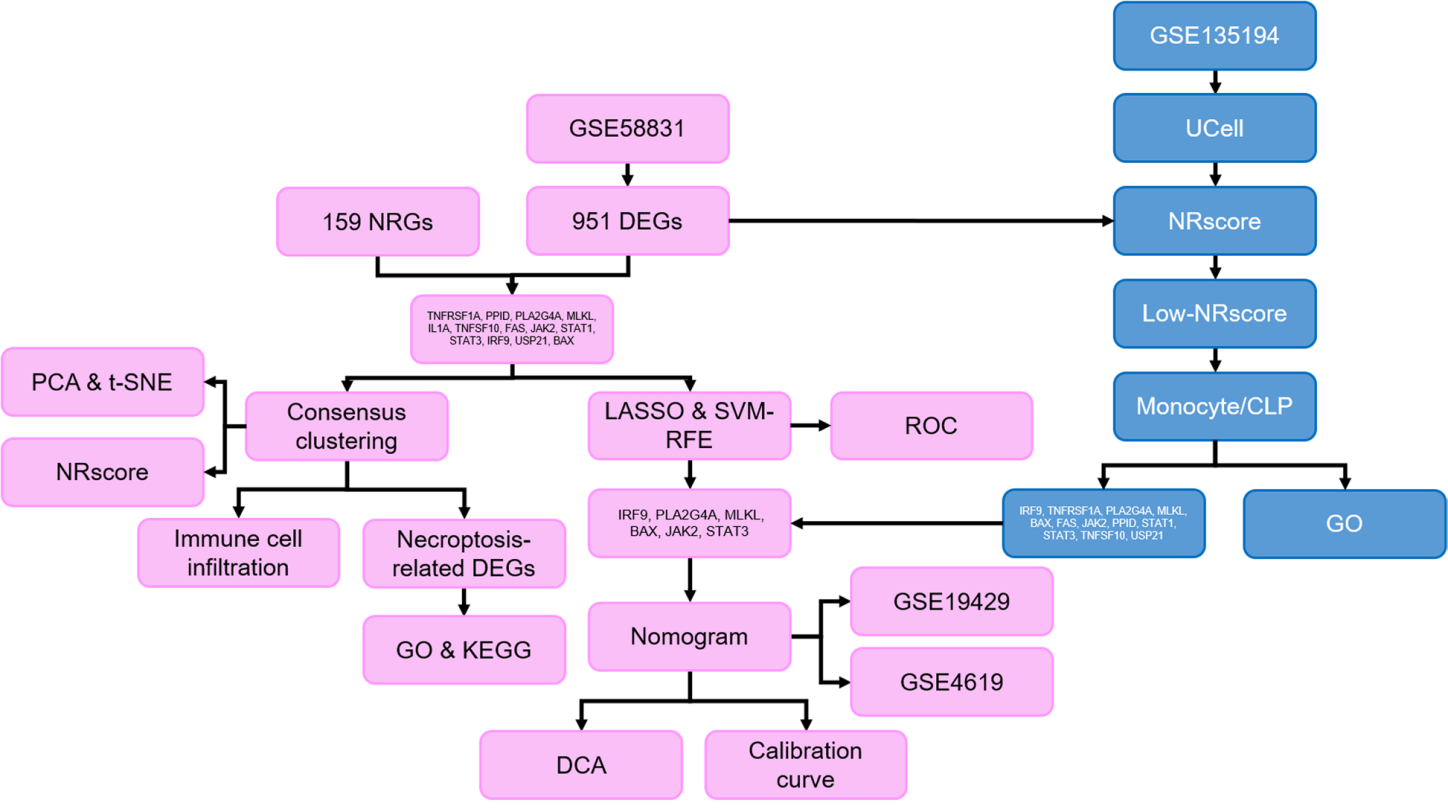
Workflow showed the analysis processes in the present study. Pink represents the analysis process based on transcriptome data, and blue represents the analysis process based on scRNA-seq data

**Fig. 2:**
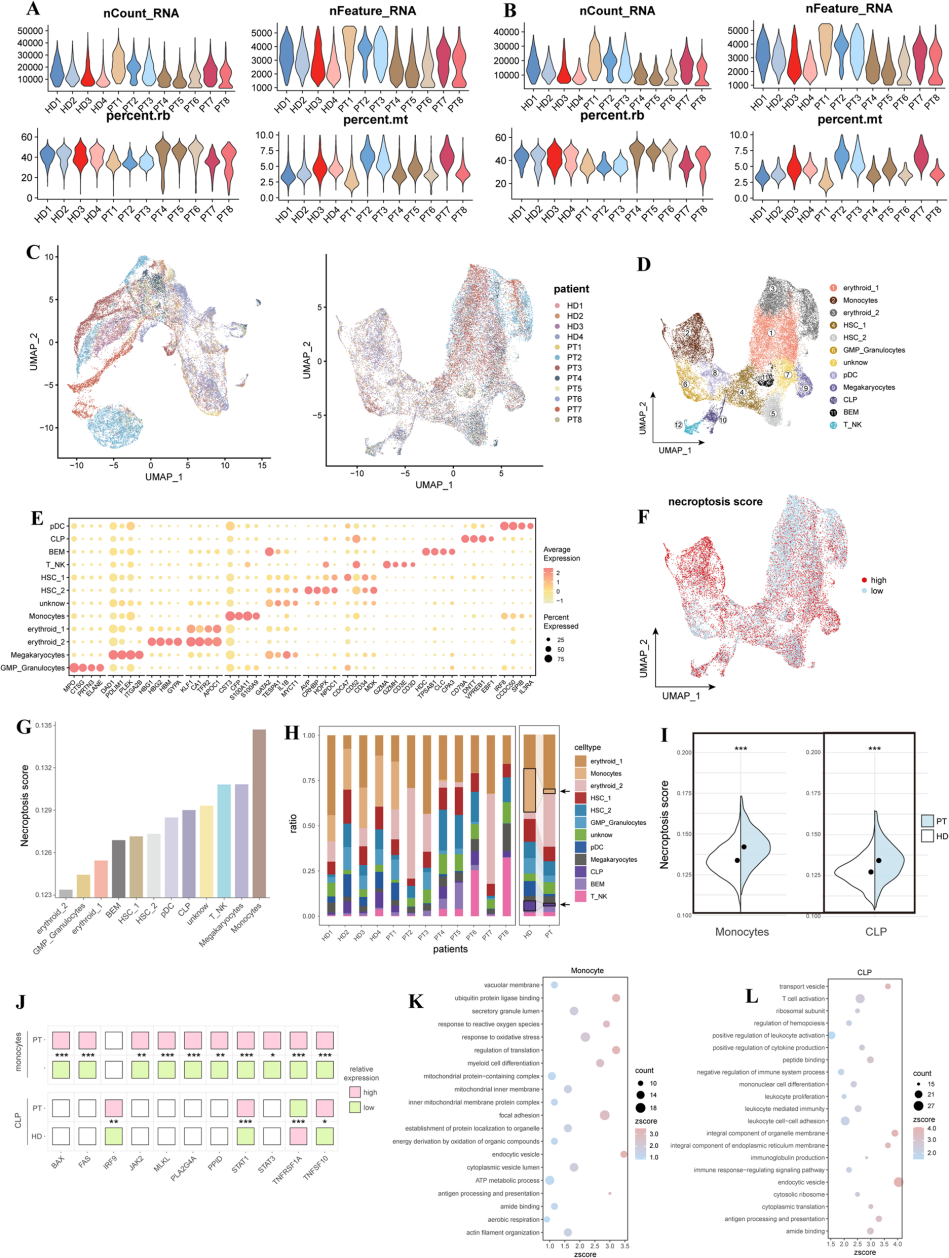
Identification of the NRscore and relevant enriched pathways at the single-cell level. **A, B** Violin plots show the total molecular (nCount), unique gene (nFeature), percentage of ribosomal cells (percent.rb), and the percentage of mitochondrial genes (percent.mt) for each sample in the GSE135194 dataset before and after quality control. **C** UMAP shows the clusters of all samples before and after harmony. **D** UMAP shows the 12 annotated cell populations, including erythroid cells (erythroid_1 and erythroid_2), hematopoietic stem cells (HSC_1 and HSC_2), monocytes, common lymphoid precursor (CLP), granulocyte-monocyte progenitors (GMP), pDC, megakaryocytes, basophil eosinophil-mast cell progenitor cells (BEM), T/NK (T_NK) cells, and unknown population. **E **Bubble plot shows the top 4 markers for each cell cluster. **F **UMAP shows the distribution of the cell clusters with NRscores. **G **Column shows the NRscore for each cell population. **H **Column shows the cell distributions between MDS patients and health controls. **I **Violin plots of the NRscore between MDS patients and health controls in monocytes and CLPs. **J** Heatmap shows the DEGs between MDS patients and health controls in monocytes and CLPs. **K, L** Bubble plots show the biological function enrichment in monocyte and CLP clusters

The analysis processes of this study are shown in the workflow in Fig. 1. ScRNA-seq data from the 4 health controls (HD1-4) and 8 MDS (PT1-8) were processed. After quality control and normalization (Fig. 2A-C), all cells were classified into 12 cell populations based on the expression of canonical markers and previous studies (Fig. 2D, E) [32, 38–44], including erythroid cells (erythroid_1 and erythroid_2), hematopoietic stem cells (HSC_1 and HSC_2), monocytes, common lymphoid precursor (CLP), granulocyte-monocyte progenitors (GMP), pDC, megakaryocytes, basophileosinophil-mast cell progenitor cells (BEM), T/NK (T_NK) cells, and unknown population.

The NRscore for each cell population was calculated using the “UCell” R package (Fig. 2F), we found monocyte and CLP with the highest NRscore than other cell types (Fig. 2G). Additionally, we found the decreased abundances of monocytes and CLPs in MDS patients compared with in health control (Fig. 2H). As expected, monocytes and CLPs in MDS patients with higher NRscore compared with those in health control (Fig. 2I). It suggests that decreased abundances of monocytes and CLPs in MDS patients are linked to necroptosis.

Therefore, we further explore the molecular function of the monocytes and CLPs. A total of 11 DEGs (BAX, FAS, IRF9, JAK2, MLKL, PLA2G4A, PPID, STAT1, STAT3, TNFRSF1A, and TNFSF10) were identified in the monocytes and CLPs between MDS patients and health controls (Fig. 2J). These DEGs in monocytes were involved in mitochondria-related pathways, such as ubiquitin protein ligase binding, mitochondrial-protein-containing complex, mitochondrial inner membrane, inner mitochondrial membrane protein complex, and ATP metabolic process (Fig. 2K). In addition, these DEGs in CLPs were involved in the T cell activation and proliferation, positive regulation of leukocyte activation, leukocyte cell-cell adhesion, immune response-regulating signaling pathway, and endocytic vesicle (Fig. 2L).

### Identification of DE-NRGs in MDS

**Fig. 3:**
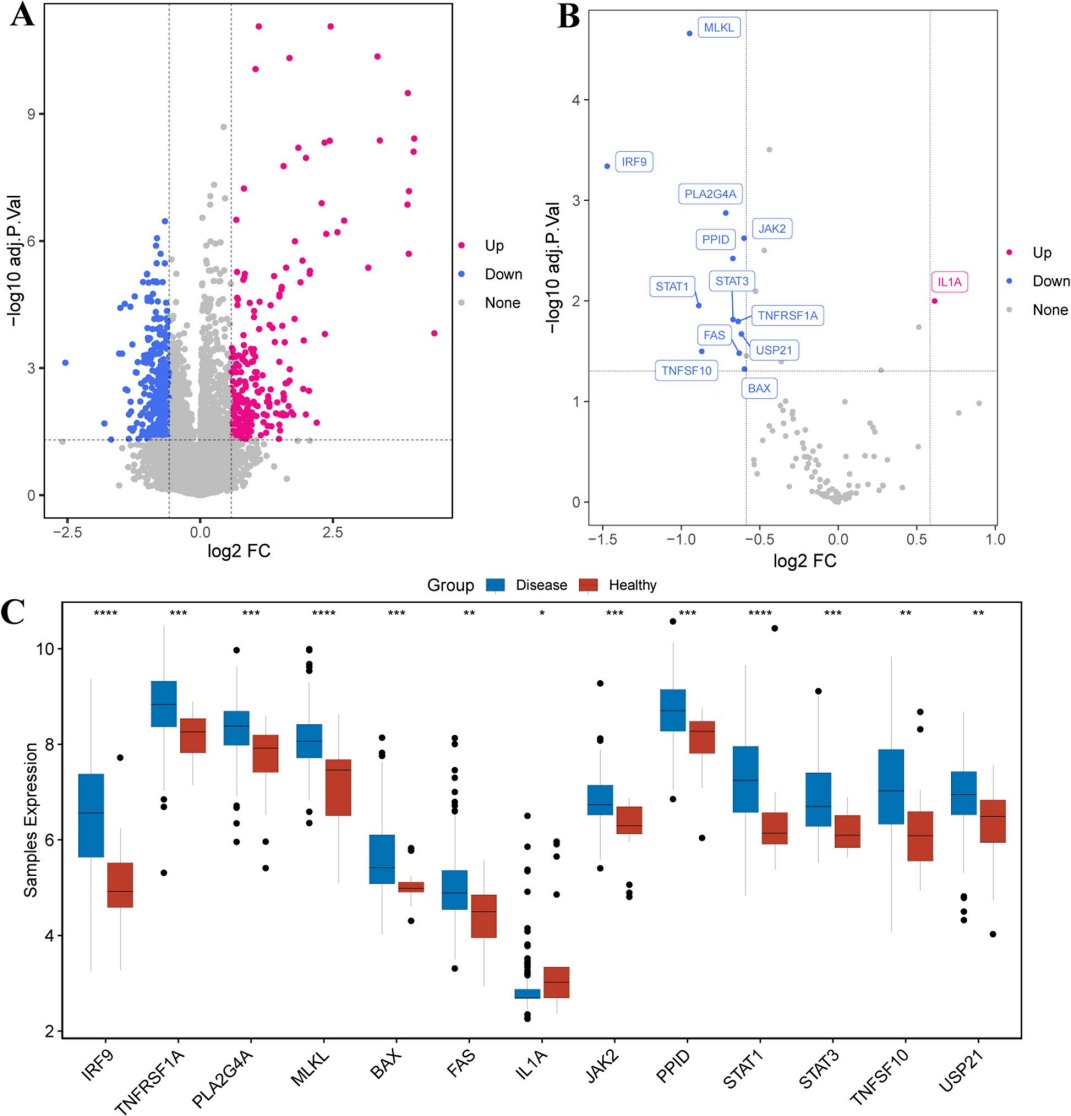
Identification of NRGs in MDS. **A **Volcano plot shows the DEGs between MDS and health controls with |log_2_FC| > 0.585 and *P*-value < 0.05. **B** Volcano plot shows 13 DE-NRGs between MDS and health controls with |log2FC| > 0.585 and *P*-value < 0.05 C. Boxplot shows 13 DE-NRGs between MDS and health controls using the Wilcoxon test

We also identified the DE-NRGs at the transcriptome level. A total of 951 DEGs (299 upregulated and 652 downregulated DEGs) between MDS and health controls were identified according to |log2FC| > 0.585 and *P*-value < 0.05 (Fig. 3A, Table S1). 13 DE-NRGs (1 upregulated and 12 downregulated DE-NRGs) were subsequently selected by overlapping 951 DEGs and 159 NRGs (Table S2). 13 DE-NRGs were shown in Fig. 3B, C, including TNFRSF1A, PPID, PLA2G4A, MLKL, IL1A, TNFSF10, FAS, JAK2, STAT1, STAT3, IRF9, USP21, BAX.

### Development of two distinct necroptosis-related clusters in MDS

**Fig. 4:**
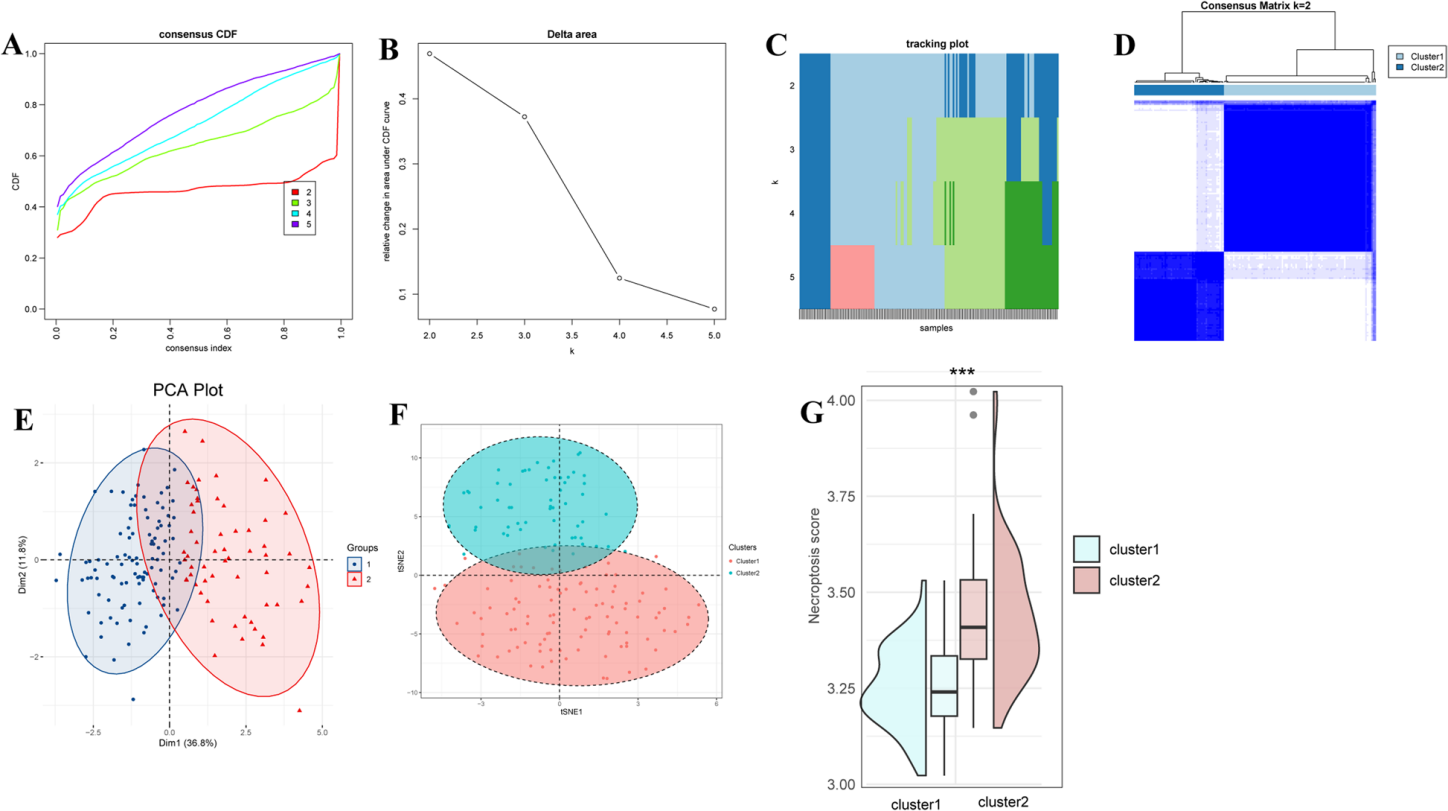
Development of two distinct necroptosis-related clusters in MDS. **A** The cumulative distribution function (CDF) of consensus clustering for k from 2 to 5. **B** The changes in the area under the CDF for k from 2 to 5. **C** The tracking plot shows the principal component for k from 2 to 5. **D** Consensus clustering of 13 DE-NRGs for k = 2. **F** t-SNE scatter plot shows the clustering results based on 13 DE-NRGs. **G** Violin plots show the NRscore between two clusters

Consensus clustering was conducted to classify MDS patients into different subclusters based on 13 DE-NRGs. The CDF plot indicated a slight increase in CDF but a sharp decrease in cluster consensus when k was 2 (Fig. 4A). The change in area under the CDF curve was highest when k was 2 (Fig. 4B, C, Figure S1). Thereby, 159 MDS patients were divided into two molecular clusters, 100 patients in cluster 1 and 59 patients in cluster 2 (Fig. 4D). The classification results were validated by PCA and t-SNE (Fig. 4E, F). SsGSEA was used to calculate the NRscore of two clusters, resulting in a higher NRscore in cluster 2 than in cluster 1 (Fig. 4G).

### Functional Enrichment Analysis

**Fig. 5:**
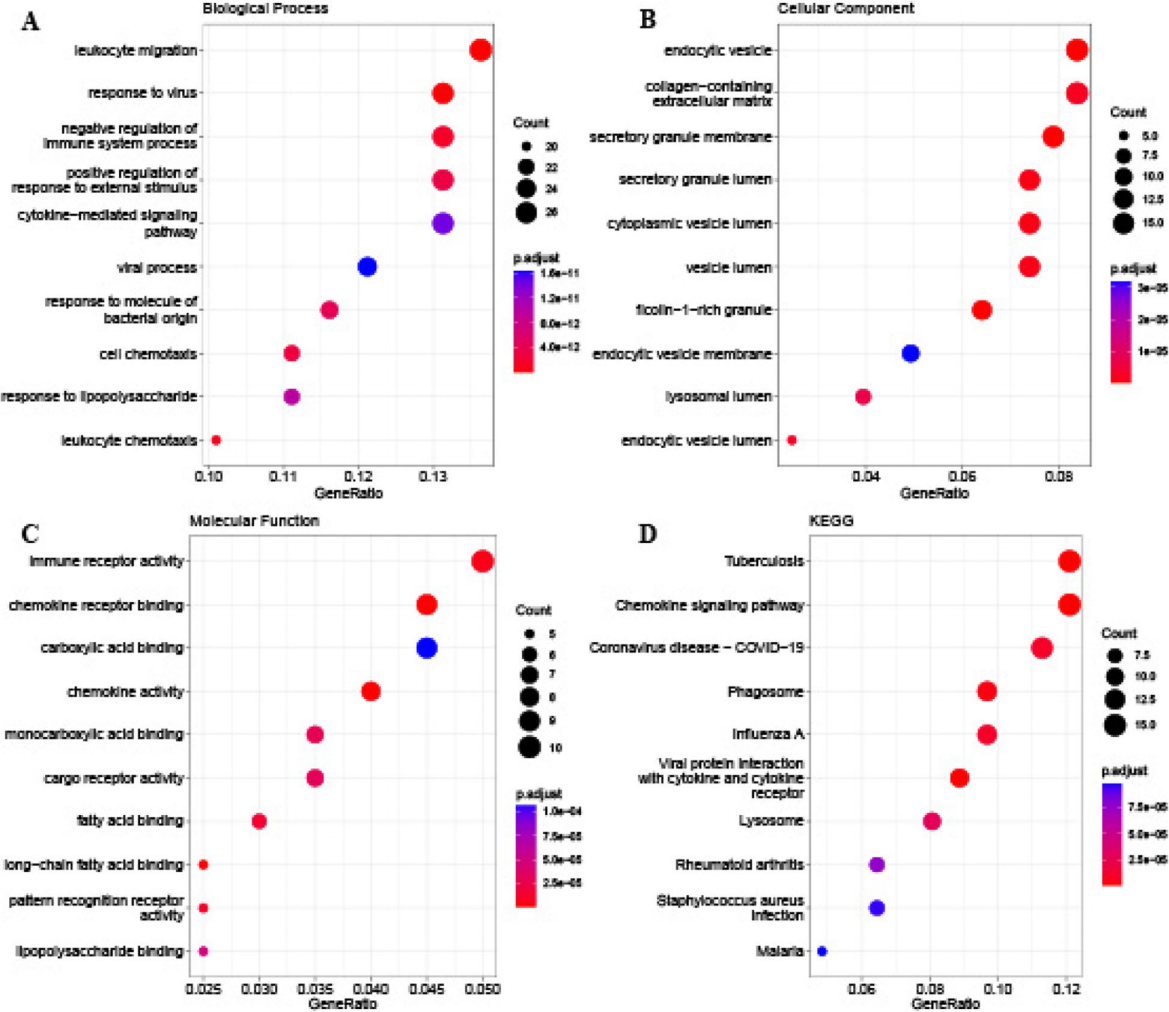
Functional enrichment analysis.  **A**-**C** Bubble plots show the GO annotation analysis, including top 10 BP, top 10 CC, and top 10 MF terms. **D** Bubble plots show the top 10 KEGG pathway enrichment

We then identified 831 DEGs (754 upregulated and 77 downregulated) between two subclusters with |log2FC| > 0.585 and *P*-value < 0.05 (Table S3). GO and KEGG analyses were performed to explore the potential biological functions and signaling pathways. We found that 942 GO terms, including 782 BP terms, 68 CC terms, and 92 MF terms, were enriched (Table S4). The top 10 BP terms included several immune-related processes, such as leukocyte migration/chemotaxis, response to the virus, negative regulation of immune system process, positive regulation response to external stimulus, cytokine-mediated signaling pathway, viral process, response to molecule of bacteria origin, cell chemotaxis, and response to lipopolysaccharide (Fig. 5A). The top 10 CC terms included endocytic vesicle, collagen-containing extracellular matrix, secretory granule membrane, secretory granule lumen, cytoplasmic vesicle lumen, vesicle lumen, ficolin-1rick granule, endocytic vesicle membrane, lysosomal lumen, and endocytic vesicle lumen (Fig. 5B). The top 10 MF terms included immune receptor activity, chemokine receptor binding, carboxylic acid binding, chemokine activity, monocarboxylic acid binding, cargo receptor activity, fatty acid binding, long-chain fatty acid binding, pattern recognition receptor activity, lipopolysaccharide-binding (Fig. 5C). Furthermore, 45 significant pathways were identified, such as the chemokine signaling pathway, phagosome, viral protein interaction with cytokine and cytokine receptor, and lysosome (Table S5, Fig. 5D).

### The landscape of immune cell infiltration in two necroptosis-related clusters

**Fig. 6:**
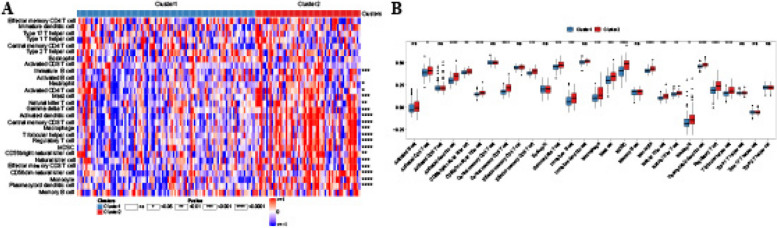
The landscape of immune cell infiltration in two necroptosis-related clusters. **A** Heatmap shows the differences in infiltrated immune cells between two clusters using ssGSEA analysis. **B** Boxplots show the differences in infiltrated immune cells between two clusters using the Wilcoxon test

In the present study, we calculated the difference in immune cell infiltration ratio in the two subclusters using ssGSEA. We found most of the immune cells were decreased in cluster 1 compared with cluster 2, including activated dendritic cell (DC), central memory CD8 T cell, myeloid-derived suppressor cell (MDSC), monocyte, plasmacytoid dendritic cell (DC), regulatory T cell (Tregs), T follicular helper cell (Tfh), CD56dim natural killer (NK) cell, effector memory CD8 T (TEFF) cell, Macrophage, gamma delta T cell, immature B cell, mast cell, natural killer cell (NK), CD56bright natural killer cell (NK), natural killer T (NKT) cell, neutrophil, and activated CD4 T cell (Fig. 6A, B). These findings suggested that higher immune infiltrating in cluster 2 compared with cluster 1.

### Generation and validation of the diagnostic NRGs in MDS

**Fig. 7:**
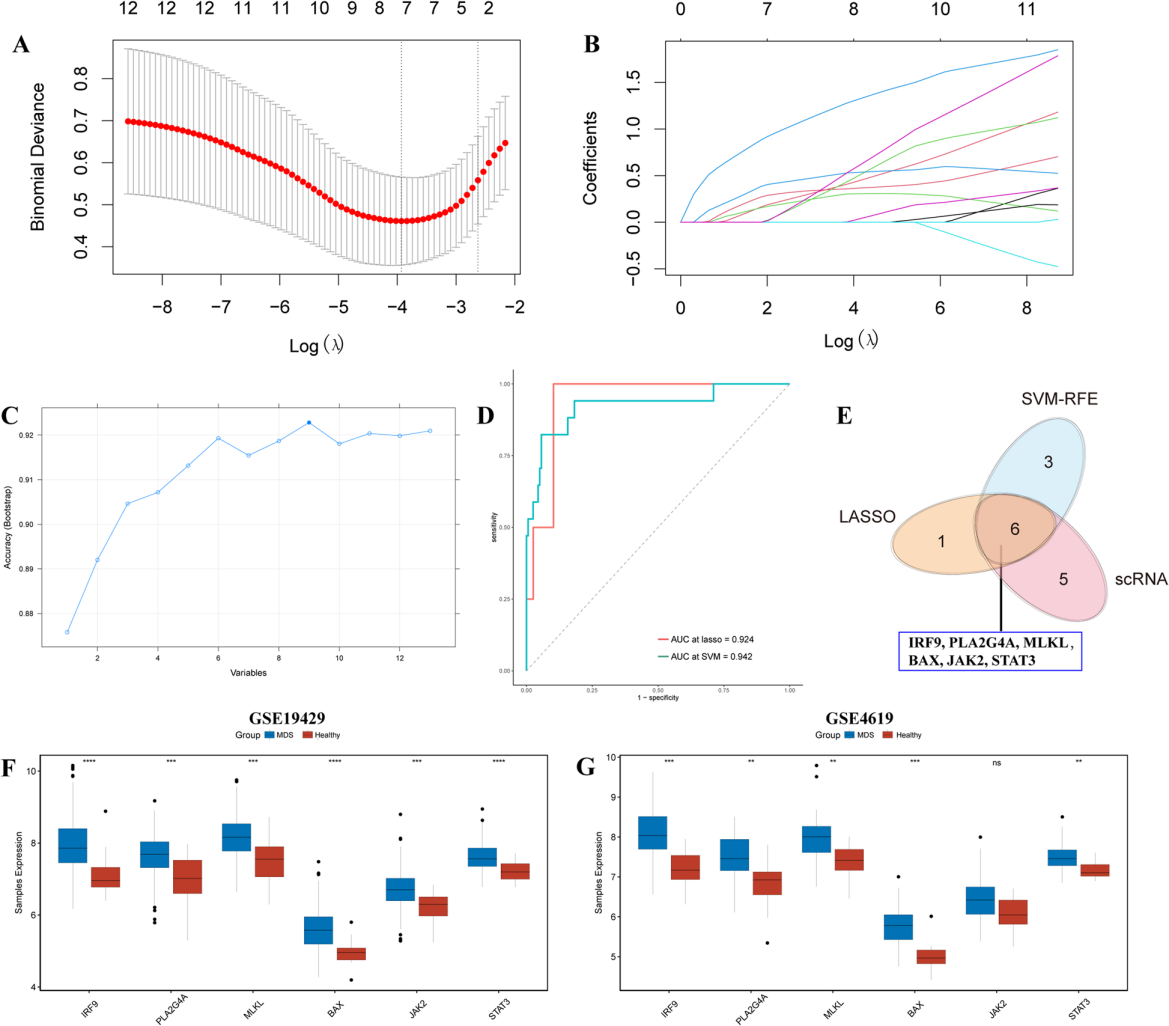
Generation and validation of the diagnostic NRGs in MDS. **A** The distribution plots of the partial likelihood deviation of the LASSO coefficients. **B** The distribution plots of the LASSO coefficients. **C** SVE-RFE algorithm was used to identify the candidate genes. **D** ROC curves show the performance of LASSO and SVM-RFE models. **E** Venn plot shows the candidate genes obtained by overlapping the feature genes from two machine algorithms and DEGs from the scRNA-seq data analysis. **F**-**G** Columns show the expression of IRF9, PLA2G4A, MLKL, BAX, JAK2, and STAT3 between MDS patients and health controls in GSE19429 and GSE4619 datasets

To explore whether NRGs could serve as indicators for MDS diagnosis, the 13 DE-NRGs were simultaneously identified using LASSO and SVM-RFE algorithms based on the expression profiles from the GSE58831 dataset. LASSO regression was performed to narrow the NRGs, resulting in 7 NRGs (PPID, PLA2G4A, MLKL, JAK2, STAT3, IRF9, and BAX) being identified as the candidates for MDS diagnosis (Fig. 7A, B). In addition, the samples of the GSE58831 dataset were divided into a training set for model development (3/4, *n* = 119) and a test set for model validation (1/4, *n* = 40), SVE-RFE algorithm was conducted to select 9 candidates (MLKL, STAT1, IRF9, BAX, JAK2, PLA2G4A, STAT3, FAS, TNFSF10) for MDS (Fig. 7C). Furthermore, ROC curves were plotted to validate the diagnostic model (LASSO and SVM-RFE models), indicating that the AUC values of LASSO and SVM-RFE models were 0.924 and 0.942, respectively (Fig. 7D). The results indicated excellent accuracy both in LASSO and SVM-RFE models. Thereby, 6 key candidates, IRF9, PLA2G4A, MLKL, BAX, JAK2, and STAT3, were selected as diagnostic markers for MDS based on the scRNA-seq data and bulk RNA-freq data (Fig. 7E). Finally, we also explored the expression of 6 diagnostic markers in external datasets, resulting in IRF9, PLA2G4A, MLKL, BAX, and STAT3 significantly increased in MDS compared with healthy controls (Fig. 7F, G).

### Development of a diagnostic nomogram

**Fig. 8:**
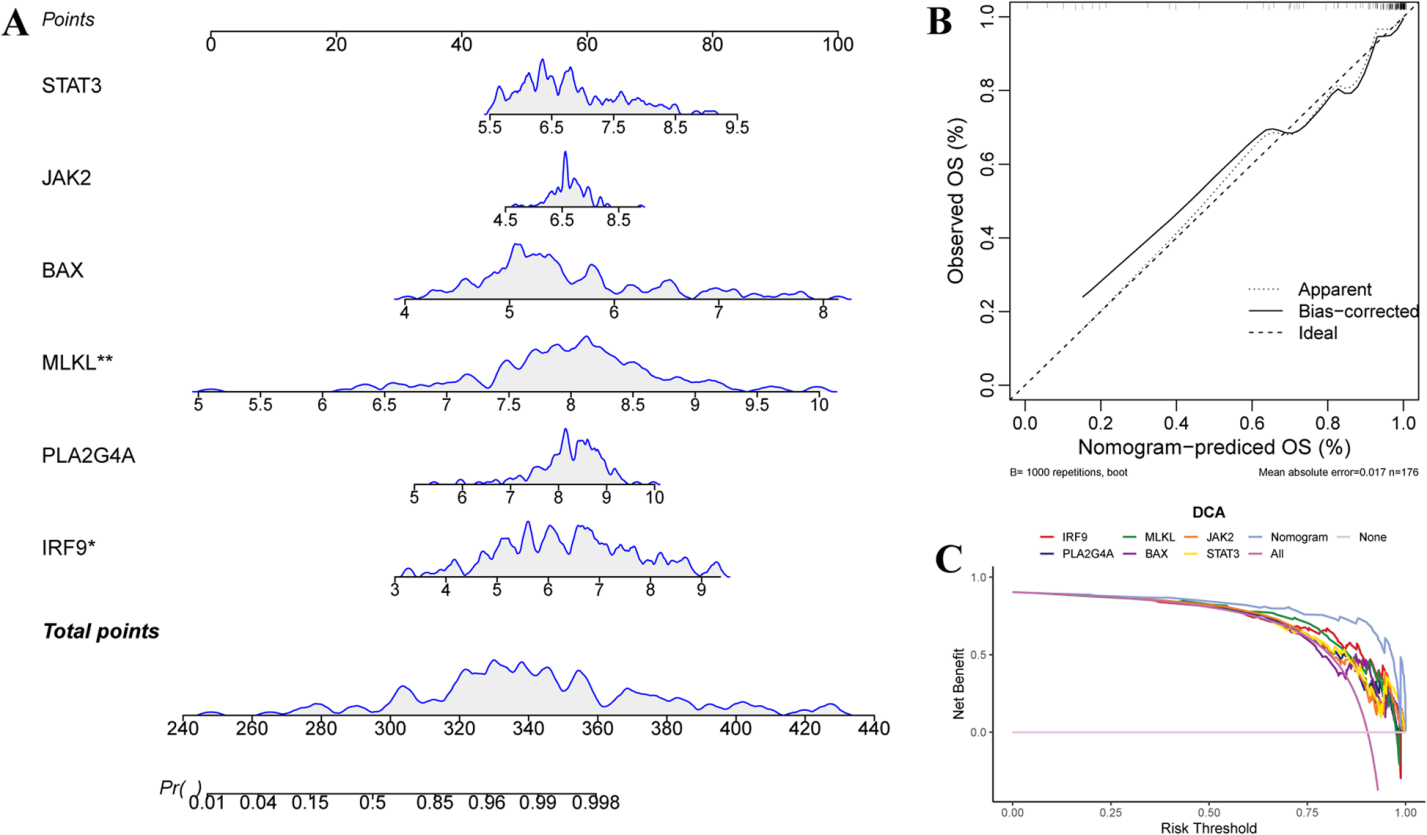
Development of a diagnostic nomogram. **A** Nomogram model for MDS predicting based on the six diagnostic markers. **B** Calibration curve estimated the predictive ability of the nomogram model. **C** Decision curves based on the nomogram model assessed the benefit of MDS patients

A diagnostic nomogram model was developed based on 6 prognostic markers to predict the prevalence of MDS patients (Fig. 8A). Calibration curves indicated the accuracy of the prognostic model (Fig. 8B). Moreover, the DCA curves show the lines of each marker and nomogram higher than the rose and pink lines from 0 to 1, indicating the decisions based on the six prognostic biomarkers and prognostic model may benefit the MDS patients (Fig. 8C).

## Discussion

MDS is a heterogeneous and clonal hematological disorder [45]. Owning to the next-generation sequencing (NGS) techniques, significant advancements have been made in understanding the molecular mechanisms involved in the initiation and progression of MDS [46–52]. NGS is extensively utilized for various applications in MDS, including distinguishing MDS from other diseases, classifying subgroups of MDS patients, identifying effective therapeutic targets, providing prognostic information, and monitoring disease progression or treatment failure [53].

Necroptosis is a prominent model of PCD in the bone marrow microenvironment of MDS, leading to inflammation through the release of cellular contents [29]. Necroptosis exacerbates both cell death and inflammation within the bone marrow. However, the role and molecular mechanisms of necroptosis in pathological processes remain incompletely understood. In the present study, we reanalyzed the scRNA-seq data to illustrate the characteristics of bone marrow in the MDS. We identified 13 distinct cell populations, including erythroid_1, erythroid_2, HSC_1, HSC_2, monocytes, CLP, GMP, pDC, megakaryocytes, BEM, T/NK (T_NK) cells, and unknown population. Among these cells, monocyte and CLP exhibited significant differences between MDS patients and health controls and were involved in several pathways associated with mitochondria-related pathways and immune-related pathways. Mitochondria plays a central role in linking cell death to inflammation [54, 55], underscoring their importance in inflammatory processes associated with cell death. Interestingly, MDS patients showed a higher NRscore than health controls, suggesting that mitochondrial-related pathways are involved in the necroptosis of MDS.

Based on the bulk RNA-seq data analysis, 13 DE-NRGs were screened out between MDS and health controls, including TNFRSF1A, PPID, PLA2G4A, MLKL, IL1A, TNFSF10, FAS, JAK2, STAT1, STAT3, IRF9, USP21, BAX. Notably, IL1A expression was increased in MDS patients compared to health controls, while TNFRSF1A, PPID, PLA2G4A, MLKL, TNFSF10, FAS, JAK2, STAT1, STAT3, IRF9, USP21, and BAX expression levels were decreased. Based on those NRGs, 159 MDS patients were divided into two clusters. The classification was validated by PCA and t-SNE. SsGSEA revealed a higher NRscore in cluster 2 compared to cluster 1. Functional analyses of the DEGs between two subclusters revealed enrichment in several immune-related biological processes and functions. For example, leukocyte migration/chemotaxis, negative regulation of immune system process, positive regulation response to external stimulus, cytokine-mediated signaling pathway, response to molecule of bacteria origin, cell chemotaxis, and response to lipopolysaccharide. Besides, several signaling pathways were enriched, including the chemokine signaling pathway, phagosome, viral protein interaction with cytokine and cytokine receptor, and lysosome.

Previous research highlights the key role of leukocyte migration or chemotaxis in the surveillance in MDS surveillance, with reduced leukocyte migration indicating high-risk MDS [56]. Over the past decade, studies have shown that the deregulation of innate immune and inflammatory signaling drives MDS pathogenesis by activating bone marrow hematopoietic stem cells and progenitor cells, as well as affecting other immune system cells and the bone marrow microenvironment [57, 58]. We speculated that the process of necroptosis exacerbates bone marrow inflammation by inducing abnormal immune cell population dynamics.

We further investigated the landscape of immune cell infiltration in two subclusters. The results showed that activated DC, central memory CD8 T cell, MDSC, monocyte, plasmacytoid DC, Tregs, Tfh, CD56dim NK cell, TEFF cell, Macrophage, gamma delta T cell, immature B cell, mast cell, NK, CD56bright NK cell, NKT cell, neutrophil, and activated CD4 T cell were decreased in cluster 1 compared to cluster 2. The higher NRscore and decreased immune cell infiltration were observed in cluster 1, indicating MDS patients in cluster 1 were linked to necroptosis. Previous study reveals that disorders of immune populations drive abnormal clonal cells to escape immune surveillance in MDS [59]. For example, NK cell is a key population in innate immunity with heterogeneity and contribute against malignant cells [60, 61]. It has been found that decreased mature NK cells are associated with poor survival in MDS [62]. Consistent with it, the significantly reduced NK cells (D56dim NK cells, CD56bright NK cells, NK cells, and NKT cells) in cluster 1 showed a poor prognosis. Furthermore, the disorder of immune response contributes to bone marrow insufficiency and disease progression in MDS [63]. Previous studies have found that higher counts of cytotoxic T cells and lower counts of Tregs relate to low-risk MDS [64, 65]. MDSC functions as an inflammatory and immunosuppressive effector cell. MDSC are markedly expanded in the bone marrow to reduce T cell proliferation and function in MDS [59]. Here, the decreased immune cell populations in cluster 1 might link to immune escapes.

Finally, a necroptosis-related diagnostic gene signature (IRF9, PLA2G4A, MLKL, BAX, JAK2, and STAT3) was identified, and these models showed good performance both in training and validation sets. Subsequently, a nomogram model was developed based on IRF9, PLA2G4A, MLKL, BAX, JAK2, and STAT3 to predict the prevalence of MDS patients and the decisions benefited the MDS. Several studies have indicated IRF9 plays an important role in inflammation [66], we first found the key role of IRF9 in MDS. PLA2G4A also is an inflammation-related gene, a previous study indicates that high PLA2G4A expression is associated with poor overall survival of MDS patients [67]. Several studies have demonstrated that upregulated MLKL expression in MDS may be associated with necrosis of the MDS cell line [30, 68]. Previous study reveals that BAX expression relates to high-risk MDS [69]. JAK-STAT activating is a specific phenotype in MDS, against JAK-STAT signaling that can inhibit inflammatory cytokines and myeloproliferative [70, 71]. Our findings are consistent with previous research, that high IRF9, PLA2G4A, MLKL, BAX, JAK2, and STAT3 expression in MDS compared to health controls and is associated with poor prognosis.

## Conclusion

In conclusion, we identified a necroptosis-associated diagnostic signature for MDS by integrating scRNA-seq and bulk RNA-seq data. This study elucidates the regulatory mechanisms of NRGs in MDS, highlights primary cell populations affected by necroptosis, and demonstrates that MDS patients with high NRscores exhibit poor prognosis. These findings suggest that targeting necroptosis could represent a novel therapeutic strategy for MDS. Our results provide potential biomarkers for the diagnosis and treatment of MDS.

## Supplementary Information

Supplementary Material 1: Table S1. The DEG between MDS and health in the GSE58831 dataset. Table S2. The DE-NRGs between MDS and health in the GSE58831 dataset. Table S3. The DEGs between two clusters in the GSE58831 dataset. Table S4. The GO annotation analysis of DEGs. Table S5. The KEGG enrichment analysis of DEGs.

Supplementary Material 2: Supplementary Figure S1. Consensus clustering of the 13 DE-NRGs for k from 2 to 5. 

## Data Availability

The raw data that support the finding have been deposited in the Gene Expression Omnibus (GEO) database under the accession number GSE58831, GSE19429, and GSE4619. All the other data generated in this study are included in the article and supplementary files.
