# Supplementary material for: Comprehensive analysis of a necroptosis-associated diagnostic signature for myelodysplastic syndromes based on single-cell RNA-seq and bulk RNA-seq
Source: Hereditas. 2024 Oct 15;161:38. doi: 10.1186/s41065-024-00335-x (PMC11481600; doi:10.1186/s41065-024-00335-x)
Supplement: Supplementary file 1 — Supplementary Material 1: Table S1. The DEG between MDS and health in the GSE58831 dataset. Table S2. The DE-NRGs between MDS and health in the GSE58831 dataset. Table S3. The DEGs between two clusters in the GSE58831 dataset. Table S4. The GO annotation analysis of DEGs. Table S5. The KEGG enrichment analysis of DEGs. [file 41065_2024_335_MOESM1_ESM.pdf]

Table S1 The differentially expressed genes (DEGs) between MDS and health in GSE58831 dataset.
Table S2 The differentially expressed NRGs between MDS and health in GSE58831 dataset.
Table S3 The DEGs between two clusters in GSE58831 dataset.
Table S4 The GO annoation analysis of DEGs.
Table S5 The KEGG enrichment analysis of DEGs.

Table S1 The differentially expressed genes (DEGs) between MDS and health in GSE58831 dataset.

	symbol	log2FC	pvalue	adj.P.Val	sig
1	OR7A5	2.456355	6.82E-16	8.57E-12	Up
2	RP11-357(	1.102544	7.92E-16	8.57E-12	Up
3	IGHV5-78	3.339046	6.08E-15	4.39E-11	Up
4	ARPP21	1.680972	8.81E-15	4.77E-11	Up
5	GPR176	1.041912	2.01E-14	8.71E-11	Up
6	AKAP12	3.91089	8.90E-14	3.21E-10	Up
7	BEST3	0.440143	6.53E-13	2.02E-09	None
8	MME	4.030662	1.41E-12	3.81E-09	Up
9	RAG2	3.383277	1.76E-12	4.22E-09	Up
10	SMAD7	2.440635	1.98E-12	4.29E-09	Up
11	RAG1	2.343122	2.41E-12	4.75E-09	Up
12	NPCDR1	1.847915	3.49E-12	6.29E-09	Up
13	P2RY14	4.019132	4.67E-12	7.78E-09	Up
14	LDLRAD4	1.991904	7.05E-12	1.09E-08	Up
15	DUSP26	1.570418	1.18E-11	1.70E-08	Up
16	LOC28600!	0.260786	3.48E-11	4.71E-08	None
17	INO80C	0.823392	4.52E-11	5.75E-08	Up
18	VPREB1	3.931933	5.53E-11	6.66E-08	Up
19	LINC00928	0.19073	7.67E-11	8.75E-08	None
20	NPTX1	0.468475	9.06E-11	9.81E-08	None
21	LOC10012!	2.286717	1.24E-10	1.28E-07	Up
22	VPREB3	3.904263	1.40E-10	1.38E-07	Up
23	PENK	0.186828	1.47E-10	1.38E-07	None
24	LECT2	0.041277	3.13E-10	2.83E-07	None
25	PRCD	0.680187	3.65E-10	3.16E-07	Up
26	LINC01013	2.710904	3.96E-10	3.30E-07	Up
27	SMIM20	-0.66454	4.26E-10	3.42E-07	Down
28	BACH2	2.584623	8.02E-10	6.20E-07	Up
29	NPY	2.374845	9.11E-10	6.80E-07	Up
30	CSK	-0.81446	1.19E-09	8.57E-07	Down
31	SH2D4B	1.783642	1.46E-09	1.02E-06	Up
32	WNT9A	0.193236	1.52E-09	1.03E-06	None
33	VASN	0.381048	1.64E-09	1.07E-06	None
34	RP11-69I8.	0.286819	1.82E-09	1.16E-06	None
35	LOC10050	0.114126	2.07E-09	1.28E-06	None
36	APOOL	-0.83322	2.15E-09	1.29E-06	Down
37	MTR	-0.74611	3.50E-09	2.01E-06	Down
38	EBF1	3.928094	3.52E-09	2.01E-06	Up
39	CCDC134	-0.54212	4.93E-09	2.74E-06	None
40	C11orf92	0.113581	5.48E-09	2.91E-06	None
41	HMHB1	1.926213	5.51E-09	2.91E-06	Up
42	PGPEP1	-0.66745	6.55E-09	3.36E-06	Down
43	ND2	-0.82084	6.67E-09	3.36E-06	Down
44	L1CAM	0.122405	6.89E-09	3.39E-06	None
45	LEF1-AS1	1.618122	9.05E-09	4.27E-06	Up
46	CD24	3.1651	9.07E-09	4.27E-06	Up
47	AC092660.	0.298503	9.93E-09	4.58E-06	None
48	CTD-2639I	0.449372	1.10E-08	4.96E-06	None
49	SLCO1C1	0.16496	1.14E-08	4.99E-06	None
50	CYP4A11	0.204798	1.15E-08	4.99E-06	None
51	NID2	2.066563	1.19E-08	5.07E-06	Up
52	BLK	0.693486	1.29E-08	5.39E-06	Up
53	LYL1	-1.00079	1.47E-08	5.93E-06	Down
54	SERPINA12	0.374789	1.48E-08	5.93E-06	None
55	HEY1	0.839693	1.51E-08	5.94E-06	Up
56	SNX3	-0.45034	1.54E-08	5.95E-06	None

57	CXCR4	2.062957	1.57E-08	5.95E-06	Up
58	FES	-1.00348	1.65E-08	6.14E-06	Down
59	C10orf10	1.391196	1.83E-08	6.73E-06	Up
60	FAM43A	0.829652	2.02E-08	7.29E-06	Up
61	CCR7	0.81194	2.32E-08	8.23E-06	Up
62	TTLL11-IT1	0.25407	2.52E-08	8.81E-06	None
63	AC002059.	0.374569	2.58E-08	8.88E-06	None
64	ID2B	0.353168	2.68E-08	8.98E-06	None
65	SUPT7L	-0.83357	2.73E-08	8.98E-06	Down
66	GTF2H1	-0.88353	2.74E-08	8.98E-06	Down
67	MGAT4B	-1.07713	2.88E-08	9.31E-06	Down
68	TUBGCP4	-0.70805	2.95E-08	9.34E-06	Down
69	TPP1	-0.84503	2.98E-08	9.34E-06	Down
70	PAX5	1.888488	3.09E-08	9.44E-06	Up
71	MCUR1	-0.8091	3.09E-08	9.44E-06	Down
72	HEBP1	-0.97341	3.21E-08	9.65E-06	Down
73	DMRT2	0.039336	3.38E-08	1.00E-05	None
74	ERGIC3	0.576391	3.50E-08	1.02E-05	None
75	C15orf62	0.03583	3.82E-08	1.10E-05	None
76	SHISA2	1.535795	4.25E-08	1.21E-05	Up
77	SKIL	1.528482	4.92E-08	1.38E-05	Up
78	DENND2D	-0.89013	6.39E-08	1.77E-05	Down
79	FAM3C	1.491581	6.69E-08	1.83E-05	Up
80	ALDH3B1	-1.23347	7.55E-08	2.04E-05	Down
81	ZC3H12D	1.491584	7.62E-08	2.04E-05	Up
82	HOXD1	0.078101	7.87E-08	2.08E-05	None
83	CMBL	-0.9932	8.36E-08	2.18E-05	Down
84	MLKL	-0.94685	8.56E-08	2.19E-05	Down
85	AC024560.	0.695289	8.60E-08	2.19E-05	Up
86	RP11-334J	0.161778	9.40E-08	2.36E-05	None
87	WWP2	0.428599	9.48E-08	2.36E-05	None
88	RNF39	0.202823	9.68E-08	2.38E-05	None
89	RANBP2	0.709651	9.84E-08	2.39E-05	Up
90	FAM167A	0.172472	1.07E-07	2.58E-05	None
91	TEX19	0.117866	1.08E-07	2.58E-05	None
92	ZSCAN26	-0.99242	1.10E-07	2.58E-05	Down
93	CALHM1	0.366072	1.11E-07	2.58E-05	None
94	LINC01231	0.163059	1.13E-07	2.61E-05	None
95	SLC29A3	-0.9211	1.18E-07	2.66E-05	Down
96	RUSC1	-0.79932	1.18E-07	2.66E-05	Down
97	CRMP1	1.118163	1.22E-07	2.72E-05	Up
98	CDC16	-0.45682	1.26E-07	2.78E-05	None
99	PPP1R11	-0.64228	1.31E-07	2.86E-05	Down
100	RPL7AL2	0.933021	1.34E-07	2.90E-05	Up
101	LOC28439	0.34382	1.40E-07	3.01E-05	None
102	CHCHD10	-1.42094	1.43E-07	3.04E-05	Down
103	FMN1	0.060278	1.50E-07	3.15E-05	None
104	CSMD2-AS	0.18963	1.53E-07	3.19E-05	None
105	ABHD13	0.700834	1.58E-07	3.26E-05	Up
106	FAIM3	1.325086	1.61E-07	3.30E-05	Up
107	SPTBN1	0.127035	1.63E-07	3.30E-05	None
108	RP11-454K	0.054588	1.79E-07	3.57E-05	None
109	NFE2	-1.31574	1.80E-07	3.57E-05	Down
110	KCNK5	-1.50513	1.93E-07	3.80E-05	Down
111	MYOM3	0.175717	1.96E-07	3.82E-05	None
112	TAC1	0.156473	2.13E-07	4.12E-05	None
113	MC2R	0.037977	2.30E-07	4.40E-05	None
114	S1PR1	1.014795	2.38E-07	4.49E-05	Up

115	TRAF3	-0.62687	2.38E-07	4.49E-05	Down
116	ZMIZ1-AS:	0.143256	2.50E-07	4.67E-05	None
117	SNAP29	-0.836	2.56E-07	4.74E-05	Down
118	FLII	-0.50868	3.19E-07	5.86E-05	None
119	LOC10192'	0.250741	3.33E-07	6.07E-05	None
120	BCOR	1.012495	3.49E-07	6.26E-05	Up
121	SMCO3	0.207424	3.50E-07	6.26E-05	None
122	MED16	-0.81313	3.60E-07	6.39E-05	Down
123	C11orf68	-0.63272	3.80E-07	6.69E-05	Down
124	TRAM2	-0.78546	3.84E-07	6.71E-05	Down
125	NAA60	-0.74101	3.90E-07	6.71E-05	Down
126	ZSCAN9	-0.61202	3.92E-07	6.71E-05	Down
127	TIMM9	-0.55704	3.93E-07	6.71E-05	None
128	SYT13	0.156937	4.09E-07	6.90E-05	None
129	BNIP3	-0.78447	4.11E-07	6.90E-05	Down
130	TFCP2	-0.79077	4.20E-07	6.92E-05	Down
131	LINC01215	1.776946	4.24E-07	6.92E-05	Up
132	5-Mar	0.502637	4.25E-07	6.92E-05	None
133	UBE2G1	-0.51683	4.25E-07	6.92E-05	None
134	KLHL21	-0.8646	4.28E-07	6.92E-05	Down
135	JAG2	0.396428	4.52E-07	7.25E-05	None
136	MUS81	-0.57179	4.82E-07	7.66E-05	None
137	PDDC1	-0.61646	4.84E-07	7.66E-05	Down
138	WDTC1	-0.60871	5.02E-07	7.87E-05	Down
139	AC018816.	0.284578	5.46E-07	8.51E-05	None
140	NCLN	0.559565	5.94E-07	9.18E-05	None
141	NPTX2	1.306378	6.25E-07	9.61E-05	Up
142	IL18BP	-0.25026	6.46E-07	9.84E-05	None
143	CHST15	1.546437	6.66E-07	0.000101	Up
144	OVOL1	0.174308	7.00E-07	0.000105	None
145	FAM73B	-0.62716	7.22E-07	0.000108	Down
146	C11orf21	-0.75449	7.32E-07	0.000109	Down
147	LOC10013:	0.068899	7.65E-07	0.000113	None
148	HHIP	1.36154	7.73E-07	0.000113	Up
149	ZNF280B	-0.99377	7.83E-07	0.000114	Down
150	NLRP10	0.104278	8.25E-07	0.000119	None
151	CTD-2083I	1.110275	8.35E-07	0.00012	Up
152	PTPN6	-0.89919	8.40E-07	0.00012	Down
153	SLC52A2	-0.80276	8.50E-07	0.00012	Down
154	IPMK	0.458858	8.51E-07	0.00012	None
155	COL11A2	0.287415	1.00E-06	0.00014	None
156	SCAMP1-A	-0.67721	1.01E-06	0.000141	Down
157	SLC24A2	0.039769	1.04E-06	0.000143	None
158	PEX5	-0.30581	1.10E-06	0.000151	None
159	DNTT	4.409611	1.11E-06	0.000151	Up
160	ACSBG2	0.288398	1.12E-06	0.000152	None
161	EMC3	-0.74244	1.15E-06	0.000154	Down
162	KRTAP4-2	0.179148	1.18E-06	0.000157	None
163	KIAA0226L	2.348009	1.18E-06	0.000157	Up
164	E2F6	-0.7617	1.22E-06	0.000161	Down
165	RBMS3	0.158543	1.24E-06	0.000162	None
166	PIP4K2C	-0.67664	1.24E-06	0.000162	Down
167	PIGP	-0.58313	1.25E-06	0.000162	None
168	OCEL1	-0.73309	1.26E-06	0.000163	Down
169	MAP3K11	-0.40028	1.31E-06	0.000167	None
170	ZHX2	0.815033	1.31E-06	0.000167	Up
171	RPS6KC1	-0.608	1.36E-06	0.000173	Down
172	SDCBP2-A	0.460315	1.43E-06	0.00018	None

173	LRRC18	0.176917	1.46E-06	0.000183	None
174	CEP19	-0.51416	1.50E-06	0.000186	None
175	TBC1D13	-0.64347	1.55E-06	0.000192	Down
176	ABHD17A	-0.98653	1.57E-06	0.000194	Down
177	PKNOX2	0.072466	1.63E-06	0.000199	None
178	SPNS1	-0.94712	1.63E-06	0.000199	Down
179	AC079741.	0.131874	1.68E-06	0.000203	None
180	MTUS1	0.309871	1.72E-06	0.000206	None
181	MEN1	-0.97952	1.80E-06	0.000216	Down
182	RP11-164F	0.198794	1.88E-06	0.000223	None
183	PAG1	1.950714	1.89E-06	0.000223	Up
184	ACTR3BP2	0.146579	2.06E-06	0.000243	None
185	FOXO1	1.404661	2.08E-06	0.000243	Up
186	PIGF	-0.64904	2.10E-06	0.000245	Down
187	PPP1R16B	1.591258	2.13E-06	0.000246	Up
188	RNF135	-0.62437	2.13E-06	0.000246	Down
189	PLCB2	-0.40421	2.15E-06	0.000246	None
190	FAM71D	0.15201	2.16E-06	0.000246	None
191	PDCD2	-0.66333	2.27E-06	0.000257	Down
192	MAVS	-0.64301	2.29E-06	0.000259	Down
193	SLC43A1	-0.82189	2.33E-06	0.000259	Down
194	TLE3	-0.57051	2.33E-06	0.000259	None
195	REN	0.323312	2.34E-06	0.000259	None
196	SCAP	-0.62058	2.34E-06	0.000259	Down
197	ZNF252P	-0.38585	2.38E-06	0.000261	None
198	ZNF844	-0.4903	2.39E-06	0.000262	None
199	CDV3	-0.70437	2.49E-06	0.000271	Down
200	HOXD11	0.112768	2.51E-06	0.000271	None
201	CLDN10-A	0.11646	2.52E-06	0.000271	None
202	PRR14	-0.80928	2.54E-06	0.000273	Down
203	BOLA1	-0.86301	2.61E-06	0.000277	Down
204	TNRC6C-A	-0.8171	2.62E-06	0.000277	Down
205	LIAS	-1.27799	2.62E-06	0.000277	Down
206	HMCES	0.822388	2.74E-06	0.000288	Up
207	PREPL	-0.82251	2.81E-06	0.000294	Down
208	PRPH	0.330455	2.86E-06	0.000298	None
209	CSNK2A2	-0.57369	2.88E-06	0.000298	None
210	BIN2	-0.83205	2.89E-06	0.000298	Down
211	ZNF692	-0.83046	3.05E-06	0.000313	Down
212	STAT6	-0.43842	3.06E-06	0.000313	None
213	MED22	-0.84586	3.22E-06	0.000326	Down
214	C6orf136	-0.73529	3.23E-06	0.000326	Down
215	KRTAP19-3	0.095495	3.26E-06	0.000329	None
216	LINC01192	0.131463	3.32E-06	0.000333	None
217	STMN1	0.596011	3.38E-06	0.000337	Up
218	IL11	0.421013	3.43E-06	0.000341	None
219	EXTL2	-0.75335	3.47E-06	0.000343	Down
220	HSPG2	-0.63917	3.49E-06	0.000344	Down
221	LCP2	-0.62457	3.59E-06	0.000352	Down
222	MEOX2	0.050024	3.62E-06	0.000352	None
223	CMTM7	0.956559	3.63E-06	0.000352	Up
224	TMEM108-	0.263341	3.65E-06	0.000352	None
225	UBE2NL	0.336939	3.76E-06	0.000358	None
226	SCYL1	-0.2946	3.77E-06	0.000358	None
227	TMEM243	1.011645	3.78E-06	0.000358	Up
228	TJAP1	-0.41029	3.78E-06	0.000358	None
229	PIK3C2G	0.026202	3.79E-06	0.000358	None
230	EIF2AK3	0.424409	3.81E-06	0.000358	None

231	RP3-522P1	0.611457	3.97E-06	0.000371	Up
232	ZDHHC4	-0.49998	3.98E-06	0.000371	None
233	LOC10192	0.15432	4.03E-06	0.000374	None
234	RITA1	-0.92306	4.13E-06	0.000382	Down
235	KCNJ14	0.243672	4.30E-06	0.000396	None
236	LCN10	0.987469	4.59E-06	0.000421	Up
237	LOC22194	0.033507	4.64E-06	0.000423	None
238	LMAN2L	-0.74823	4.65E-06	0.000423	Down
239	RP11-613M	0.85136	4.71E-06	0.000427	Up
240	LOC10013	0.059927	4.87E-06	0.000438	None
241	CDK10	-0.56582	4.87E-06	0.000438	None
242	PXN	-0.43013	4.94E-06	0.000442	None
243	GSE1	-0.87065	5.03E-06	0.000448	Down
244	GNPDA1	-1.52723	5.12E-06	0.000455	Down
245	PRR5	-0.89107	5.17E-06	0.000457	Down
246	GNG7	0.998876	5.23E-06	0.000458	Up
247	NMT1	-0.77789	5.26E-06	0.000458	Down
248	BAG4	-0.89859	5.27E-06	0.000458	Down
249	IRF9	-1.47157	5.28E-06	0.000458	Down
250	GPR132	0.51276	5.29E-06	0.000458	None
251	ELFN2	0.023757	5.35E-06	0.000461	None
252	MTERF2	-0.82774	5.36E-06	0.000461	Down
253	COA6	-0.52069	5.55E-06	0.000475	None
254	CD22	0.62497	5.68E-06	0.000484	Up
255	WDR81	-0.53015	5.75E-06	0.000484	None
256	PRRC2A	-1.00523	5.76E-06	0.000484	Down
257	EMC1	-0.91429	5.76E-06	0.000484	Down
258	USP20	-0.88637	5.76E-06	0.000484	Down
259	OR51B6	0.026518	5.83E-06	0.000487	None
260	LHFPL3-AS	0.179911	6.09E-06	0.000505	None
261	ELF2	0.527083	6.15E-06	0.000505	None
262	FAM213B	-1.05726	6.15E-06	0.000505	Down
263	UNC119	-0.51142	6.16E-06	0.000505	None
264	WFS1	0.726089	6.17E-06	0.000505	Up
265	CBR1	-0.9825	6.19E-06	0.000505	Down
266	VWA5A	-0.94172	6.20E-06	0.000505	Down
267	ZNF585B	-0.4086	6.42E-06	0.000521	None
268	CDHR3	0.02605	6.45E-06	0.000522	None
269	TPST1	0.79968	6.69E-06	0.000538	Up
270	ZNF670	1.036815	6.70E-06	0.000538	Up
271	ZSCAN16	0.666485	6.80E-06	0.000542	Up
272	DDX31	-0.58544	6.83E-06	0.000542	Down
273	AKNA	-0.50624	6.83E-06	0.000542	None
274	LIN37	-0.37443	6.89E-06	0.000545	None
275	SMOC1	0.112514	6.97E-06	0.000549	None
276	ANXA4	-0.54167	7.33E-06	0.000574	None
277	RIPPLY3	0.141923	7.35E-06	0.000574	None
278	DYRK2	0.591473	7.46E-06	0.000581	Up
279	KCNN4	-0.93159	7.66E-06	0.000593	Down
280	ZMAT3	-1.0481	7.67E-06	0.000593	Down
281	RP11-263K	0.122059	8.02E-06	0.000618	None
282	C2orf76	-0.69614	8.05E-06	0.000618	Down
283	MTMR6	0.792214	8.09E-06	0.000619	Up
284	FLJ32255	-0.9914	8.22E-06	0.000626	Down
285	LOC10050	0.111098	8.49E-06	0.000645	None
286	RP11-21L2	0.05986	8.57E-06	0.000649	None
287	HSD17B1	-0.31469	8.82E-06	0.000666	None
288	DFFB	-0.74198	9.10E-06	0.000684	Down

289	TCF4	0.993432	9.35E-06	0.0007	Up
290	ICK	0.330199	9.39E-06	0.000701	None
291	KIAA0319L	-0.45806	9.44E-06	0.000701	None
292	NR3C1	0.843941	9.45E-06	0.000701	Up
293	TREM1	1.490442	9.64E-06	0.000712	Up
294	CARM1	-1.06856	9.72E-06	0.000715	Down
295	RET	0.217126	9.74E-06	0.000715	None
296	ZNF717	-0.35939	9.78E-06	0.000715	None
297	ZNF367	0.970477	9.85E-06	0.000718	Up
298	GTF2H4	-1.17841	9.97E-06	0.000725	Down
299	MED12	-0.9841	1.01E-05	0.000733	Down
300	GPATCH1	-0.59917	1.02E-05	0.000735	Down
301	DIS3L	-0.72779	1.03E-05	0.000738	Down
302	GGPS1	-0.50848	1.04E-05	0.000743	None
303	RGS5	0.274589	1.05E-05	0.000749	None
304	LOC10192	0.570408	1.05E-05	0.000749	None
305	PTH2R	-2.541	1.06E-05	0.000749	Down
306	LENEP	0.19037	1.07E-05	0.000757	None
307	PPP1R26	-1.0134	1.07E-05	0.000757	Down
308	FUK	-0.57881	1.09E-05	0.000768	None
309	ANKMY2	-0.51392	1.10E-05	0.000769	None
310	TOP2B	0.683096	1.12E-05	0.000785	Up
311	MKL2	0.19964	1.13E-05	0.000787	None
312	ANXA10	0.035424	1.14E-05	0.000793	None
313	H2AFZ	0.380864	1.15E-05	0.000793	None
314	VPS35	-0.60015	1.17E-05	0.000807	Down
315	SMAD1	0.892281	1.17E-05	0.000807	Up
316	IL15RA	-0.90758	1.18E-05	0.000807	Down
317	CYBRD1	-0.68595	1.20E-05	0.000821	Down
318	PSMG3	-0.6296	1.21E-05	0.000826	Down
319	PPP6R1	-0.70041	1.24E-05	0.000839	Down
320	COX4I2	0.24601	1.26E-05	0.00085	None
321	COG7	-0.72296	1.27E-05	0.000859	Down
322	SF3A1	-0.51274	1.30E-05	0.000873	None
323	KPNA6	-0.50126	1.30E-05	0.000873	None
324	ELK3	0.571748	1.33E-05	0.000888	None
325	GAB1	0.727024	1.34E-05	0.00089	Up
326	TFB1M	-0.50352	1.34E-05	0.000893	None
327	RNF24	-0.74913	1.35E-05	0.000895	Down
328	RANBP10	-0.43893	1.36E-05	0.000895	None
329	ZCCHC5	0.119003	1.36E-05	0.000897	None
330	WIZ	-0.23882	1.42E-05	0.000932	None
331	DCP1B	-0.67224	1.43E-05	0.000937	Down
332	SYTL3	0.508447	1.45E-05	0.000945	None
333	OR1Q1	0.050702	1.47E-05	0.000955	None
334	TRAPPC12	-0.6668	1.50E-05	0.000975	Down
335	NUPL1	0.632094	1.52E-05	0.000981	Up
336	TESK1	-0.66328	1.53E-05	0.000987	Down
337	CANT1	-0.73225	1.57E-05	0.001008	Down
338	ZKSCAN3	-0.33128	1.57E-05	0.001008	None
339	LINC00886	0.479935	1.58E-05	0.001008	None
340	LOC10192	0.165197	1.58E-05	0.001008	None
341	APEX2	-0.54271	1.66E-05	0.001056	None
342	FLJ38379	1.686344	1.69E-05	0.001068	Up
343	FAM127A	-0.79356	1.74E-05	0.001096	Down
344	ADAD1	0.109688	1.76E-05	0.001106	None
345	RELL1	0.543575	1.78E-05	0.00112	None
346	TMEM8A	-0.45801	1.79E-05	0.001121	None

347	AC068138.	0.031472	1.80E-05	0.001125	None
348	NGB	0.145803	1.84E-05	0.001143	None
349	TRIM68	-0.8996	1.84E-05	0.001144	Down
350	NRTN	0.269917	1.86E-05	0.001148	None
351	FRMD8	0.19357	1.87E-05	0.001153	None
352	DUSP23	-0.65581	1.94E-05	0.001191	Down
353	GOLPH3L	-0.59582	1.94E-05	0.001191	Down
354	TTC31	-0.56833	2.00E-05	0.001221	None
355	SMIM7	-0.45683	2.01E-05	0.001226	None
356	ODF4	0.17139	2.02E-05	0.001227	None
357	LOC10192	0.099454	2.05E-05	0.001245	None
358	LTB	1.031573	2.06E-05	0.001246	Up
359	ADCY5	0.069804	2.07E-05	0.001246	None
360	LOC10272	0.767111	2.08E-05	0.001246	Up
361	GTF3A	-0.32546	2.08E-05	0.001246	None
362	SIN3B	-0.55325	2.08E-05	0.001246	None
363	IGF2BP3	-0.71191	2.10E-05	0.001252	Down
364	CREM	1.065597	2.11E-05	0.001252	Up
365	CENPBD1	-0.82655	2.12E-05	0.001255	Down
366	ZNF783	-0.59017	2.13E-05	0.001257	Down
367	NRROS	-1.21437	2.13E-05	0.001257	Down
368	LOC10192	0.586485	2.14E-05	0.001257	Up
369	JOSD1	0.677728	2.14E-05	0.001257	Up
370	PLEKHA8P	-0.81776	2.18E-05	0.001278	Down
371	LOC28492	-1.19824	2.19E-05	0.00128	Down
372	ZNF101	0.587582	2.28E-05	0.001324	Up
373	PLA2G4A	-0.71667	2.30E-05	0.001334	Down
374	ARAP1	-0.71	2.33E-05	0.001351	Down
375	SHC1	-0.36675	2.44E-05	0.001405	None
376	GPHA2	0.065622	2.44E-05	0.001405	None
377	LOC10192	-0.30658	2.45E-05	0.001408	None
378	RP11-5C2	-0.77587	2.48E-05	0.001419	Down
379	CLEC14A	0.484252	2.49E-05	0.001424	None
380	LOC10050	0.2049	2.52E-05	0.001436	None
381	FAM19A4	0.024276	2.53E-05	0.001438	None
382	SCML2	0.3654	2.56E-05	0.00145	None
383	RP1L1	0.026682	2.59E-05	0.001463	None
384	PMEPA1	1.174446	2.64E-05	0.001487	Up
385	ZNF467	-0.47827	2.68E-05	0.001507	None
386	MTERF3	-0.62757	2.71E-05	0.001522	Down
387	SPSB1	0.411841	2.74E-05	0.001534	None
388	CYLC1	0.09715	2.75E-05	0.001536	None
389	ALPI	0.038808	2.80E-05	0.001561	None
390	POLR3A	-0.58986	2.86E-05	0.001586	Down
391	DHRS4-AS	-0.36333	2.87E-05	0.001586	None
392	ZBTB8B	0.02368	2.87E-05	0.001586	None
393	CTDNEP1	-0.60342	2.93E-05	0.001612	Down
394	DNAL1	0.327908	2.94E-05	0.001612	None
395	LAMTOR1	-0.78294	2.94E-05	0.001612	Down
396	ASB7	-0.48272	2.95E-05	0.001613	None
397	LOC40216	0.075195	2.99E-05	0.001632	None
398	PPP1R21	-0.37346	3.01E-05	0.001637	None
399	HGH1	-0.33246	3.03E-05	0.001642	None
400	DGKK	0.148557	3.03E-05	0.001643	None
401	ARRB1	-0.63316	3.05E-05	0.001648	Down
402	RP5-1007H	0.534196	3.06E-05	0.001648	None
403	MLLT6	-0.97178	3.08E-05	0.001653	Down
404	SLC30A8	0.077308	3.16E-05	0.00169	None

405	MUC17	0.132156	3.16E-05	0.00169	None
406	MON1A	-0.74147	3.17E-05	0.00169	Down
407	CD79A	1.060292	3.18E-05	0.001694	Up
408	DCLRE1A	-0.77303	3.25E-05	0.001726	Down
409	DBH-AS1	0.486897	3.30E-05	0.001747	None
410	ZBTB21	0.973439	3.34E-05	0.001761	Up
411	LOC10050(	0.028588	3.36E-05	0.001771	None
412	PIAS3	-0.75284	3.40E-05	0.001788	Down
413	OTOP2	0.075404	3.43E-05	0.0018	None
414	RSAD1	-0.7082	3.45E-05	0.001805	Down
415	LINC01278	-0.32086	3.46E-05	0.001807	None
416	PARP4	-0.70142	3.48E-05	0.001811	Down
417	ZDHHC22	0.097942	3.60E-05	0.001867	None
418	SYT4	0.392225	3.62E-05	0.001875	None
419	IFITM3	-0.91227	3.68E-05	0.001901	Down
420	STK10	-0.61853	3.70E-05	0.001908	Down
421	BCDIN3D-.	0.122052	3.72E-05	0.001915	None
422	PLD6	0.080398	3.80E-05	0.001948	None
423	SYT6	0.147718	3.80E-05	0.001948	None
424	LOC10050'	-1.03798	3.86E-05	0.00197	Down
425	ARID5A	0.74536	3.88E-05	0.001978	Up
426	LOC10013(	1.68217	3.90E-05	0.001983	Up
427	RP11-33O4	-0.62713	3.93E-05	0.001992	Down
428	DENND4B	-0.63548	3.98E-05	0.002011	Down
429	ND4	-0.31269	3.98E-05	0.002011	None
430	MFSD1	-0.59529	4.02E-05	0.00202	Down
431	ARRDC3-A	0.173098	4.02E-05	0.00202	None
432	KPNB1	-0.44322	4.05E-05	0.002031	None
433	FAM181A	0.050553	4.07E-05	0.002033	None
434	LOC10050!	0.108748	4.07E-05	0.002033	None
435	PIM1	-0.81727	4.12E-05	0.002051	Down
436	BATF	-0.63668	4.14E-05	0.002056	Down
437	RPL10L	0.617381	4.16E-05	0.002058	Up
438	RP11-764E	0.146069	4.17E-05	0.002058	None
439	RP1-31B8.	0.235148	4.18E-05	0.002058	None
440	PKN1	-0.87485	4.19E-05	0.002058	Down
441	TTBK1	0.160133	4.19E-05	0.002058	None
442	TBX22	0.014719	4.22E-05	0.002067	None
443	PRR13	-0.46047	4.26E-05	0.002082	None
444	SPANXB1	0.840678	4.27E-05	0.002084	Up
445	MTFR1L	-0.52028	4.29E-05	0.002085	None
446	GALNT13	0.049371	4.34E-05	0.002107	None
447	INO80E	-0.75329	4.35E-05	0.002107	Down
448	LOC10192!	0.039896	4.37E-05	0.002112	None
449	ARHGAP25	-0.91778	4.40E-05	0.002121	Down
450	FLOT2	-0.50427	4.41E-05	0.002121	None
451	TNIP2	-0.57862	4.42E-05	0.002121	None
452	FDXR	-0.83334	4.46E-05	0.002133	Down
453	DMRTC2	0.038468	4.46E-05	0.002133	None
454	CX3CL1	0.109161	4.52E-05	0.002154	None
455	CTSC	-0.80474	4.60E-05	0.002188	Down
456	LOC28489!	0.10789	4.61E-05	0.002188	None
457	EDEM1	0.728172	4.63E-05	0.002195	Up
458	PPP2CB	-0.26769	4.67E-05	0.002204	None
459	IFITM2	-0.78464	4.67E-05	0.002204	Down
460	ZNF580	-0.61746	4.69E-05	0.002206	Down
461	LARP4B	-0.4733	4.70E-05	0.002207	None
462	TYK2	-0.51514	4.75E-05	0.002223	None

463	PDE4B	1.641719	4.75E-05	0.002223	Up
464	OCR1	0.043208	4.78E-05	0.002233	None
465	MYOC	0.353564	4.83E-05	0.00225	None
466	HSF1	-0.21712	4.90E-05	0.002278	None
467	ISG20	1.60633	4.95E-05	0.002297	Up
468	POLR3B	0.474269	4.97E-05	0.002298	None
469	NLRX1	-0.36989	4.99E-05	0.002304	None
470	ATG4C	-0.67512	5.00E-05	0.002304	Down
471	NDFIP1	-1.11295	5.01E-05	0.002305	Down
472	IRF2	-0.69497	5.12E-05	0.002347	Down
473	ZGPAT	-0.72231	5.18E-05	0.002373	Down
474	JAK2	-0.60028	5.21E-05	0.002379	Down
475	ZNF213	-0.4052	5.25E-05	0.002393	None
476	OK/SW-CL	0.683346	5.26E-05	0.002395	Up
477	SMYD4	-0.66346	5.38E-05	0.002441	Down
478	SC5D	0.341477	5.42E-05	0.002454	None
479	NUDT6	-0.79732	5.48E-05	0.002477	Down
480	DPP10-AS:	0.035209	5.50E-05	0.002481	None
481	CD19	1.042629	5.53E-05	0.002489	Up
482	USP54	-0.68163	5.56E-05	0.002498	Down
483	E4F1	-0.45781	5.61E-05	0.002515	None
484	ELFN1-AS:	0.36193	5.64E-05	0.002521	None
485	LOC64648:	0.303009	5.65E-05	0.002524	None
486	LIG4	0.857215	5.67E-05	0.002524	Up
487	COMMD3	-0.46441	5.68E-05	0.002524	None
488	PARP16	-0.49702	5.75E-05	0.00255	None
489	TGIF2LY	0.074441	5.80E-05	0.00257	None
490	FUT11	-0.45286	5.89E-05	0.002603	None
491	CNP	-0.25285	5.97E-05	0.002635	None
492	PBX2	-0.50631	5.99E-05	0.002635	None
493	TRMT61B	-0.50544	6.00E-05	0.002635	None
494	MKKS	-0.4311	6.02E-05	0.002641	None
495	UBE2E3	-0.4849	6.04E-05	0.002642	None
496	PLP1	0.091447	6.15E-05	0.002685	None
497	JAKMIP3	0.083937	6.19E-05	0.002695	None
498	C12orf65	-0.7395	6.37E-05	0.00277	Down
499	FBXO4	0.343272	6.50E-05	0.002821	None
500	RP5-890E1	0.092993	6.59E-05	0.002846	None
501	RIMS3	0.855681	6.60E-05	0.002846	Up
502	UBL3	0.479257	6.60E-05	0.002846	None
503	CDAN1	-0.31617	6.64E-05	0.002855	None
504	NDUFB5	-0.39083	6.66E-05	0.002855	None
505	CCDC64	0.150761	6.67E-05	0.002855	None
506	UBE3C	0.352677	6.67E-05	0.002855	None
507	GFI1B	-1.03291	6.70E-05	0.002863	Down
508	SLC23A3	0.22544	6.85E-05	0.002918	None
509	KDM4D	0.117714	6.87E-05	0.002922	None
510	ABHD14B	-0.9107	6.89E-05	0.002926	Down
511	QPCTL	0.267349	6.90E-05	0.002926	None
512	USB1	-0.66585	6.96E-05	0.002941	Down
513	XPO6	-0.32201	6.97E-05	0.002941	None
514	DCUN1D5	0.575153	7.00E-05	0.002944	None
515	DHRS13	-0.74924	7.00E-05	0.002944	Down
516	RNF138	0.5853	7.01E-05	0.002944	Up
517	ARRDC1	-0.42662	7.13E-05	0.002988	None
518	VRK3	-0.33822	7.17E-05	0.002997	None
519	TMC8	-0.65421	7.20E-05	0.003005	Down
520	LOC28466!	0.2214	7.22E-05	0.003006	None

521	LINC01212	0.095615	7.34E-05	0.00305	None
522	BC034788	0.065647	7.37E-05	0.003057	None
523	NR4A2	1.940778	7.52E-05	0.003115	Up
524	CDK15	0.112336	7.59E-05	0.003135	None
525	SHARPIN	-0.47148	7.64E-05	0.003151	None
526	CHTF8	-0.50827	7.74E-05	0.003181	None
527	IL6	1.326546	7.76E-05	0.003181	Up
528	DEAF1	-0.48691	7.76E-05	0.003181	None
529	GIMAP6	-1.4053	7.78E-05	0.003181	Down
530	LOC10050(	0.113755	7.78E-05	0.003181	None
531	MAMDC2-	0.202528	7.87E-05	0.003209	None
532	TGDS	-0.40613	7.93E-05	0.003225	None
533	TRIM35	-0.43529	7.95E-05	0.003225	None
534	PCAT4	0.047733	7.99E-05	0.003225	None
535	RYK	-0.598	7.99E-05	0.003225	Down
536	KAT2A	-0.5121	7.99E-05	0.003225	None
537	CSRP1	-0.55987	8.00E-05	0.003225	None
538	HHIP-AS1	1.161588	8.01E-05	0.003225	Up
539	FAM13B	0.546295	8.06E-05	0.003233	None
540	TTLL12	-0.74534	8.06E-05	0.003233	Down
541	LOC10028	-0.75048	8.09E-05	0.003235	Down
542	ACSS2	-0.58187	8.10E-05	0.003235	None
543	NAT16	0.102134	8.18E-05	0.003264	None
544	FEZ2	-0.60194	8.26E-05	0.003285	Down
545	SAV1	-0.62677	8.27E-05	0.003285	Down
546	APBA3	-0.23511	8.33E-05	0.003302	None
547	FAM195A	-0.65421	8.41E-05	0.003329	Down
548	TAPT1	0.793647	8.50E-05	0.003361	Up
549	VASH1	0.454895	8.52E-05	0.003362	None
550	LOC28348!	0.073156	8.54E-05	0.003364	None
551	NETO2	-1.3724	8.56E-05	0.003364	Down
552	NLRP9	0.073262	8.66E-05	0.003398	None
553	MRI1	-0.39829	8.83E-05	0.003454	None
554	HMGA1	-0.50873	8.85E-05	0.003454	None
555	BRE-AS1	2.048503	8.85E-05	0.003454	Up
556	LCN6	0.501507	8.91E-05	0.003461	None
557	MAN1B1	-0.53759	8.92E-05	0.003461	None
558	MAGEF1	-0.56632	8.92E-05	0.003461	None
559	ZNF322	-0.69442	8.98E-05	0.003479	Down
560	NACA2	0.357026	9.04E-05	0.003496	None
561	LINC01365	0.097219	9.07E-05	0.003502	None
562	AVPR2	0.160336	9.13E-05	0.003514	None
563	ERCC2	-0.27638	9.14E-05	0.003514	None
564	ZBTB45	-0.2634	9.15E-05	0.003514	None
565	CCNE2	0.625301	9.19E-05	0.003518	Up
566	RP4-612B1	0.070651	9.20E-05	0.003518	None
567	AP2B1	-0.65119	9.23E-05	0.003521	Down
568	RP11-589F	-0.84567	9.24E-05	0.003521	Down
569	ABCC10	-0.52081	9.30E-05	0.003536	None
570	TALDO1	-0.29566	9.31E-05	0.003536	None
571	ERRFI1	1.087486	9.46E-05	0.003587	Up
572	MLC1	-0.82835	9.48E-05	0.003587	Down
573	RP11-48B3	1.080047	9.54E-05	0.003604	Up
574	CLCN6	-0.56921	9.57E-05	0.003608	None
575	KEAP1	-0.63984	9.58E-05	0.003608	Down
576	ZNF74	-0.42829	9.60E-05	0.003609	None
577	BLOC1S3	-0.34299	9.64E-05	0.003618	None
578	SLC46A3	-0.71765	9.71E-05	0.003637	Down

579	ACO1	-0.3375	9.77E-05	0.003655	None
580	NAGPA	-0.63472	9.95E-05	0.003716	Down
581	PPID	-0.67124	0.000102	0.003783	Down
582	PARP9	-0.79526	0.000102	0.003783	Down
583	EZR	1.100829	0.000102	0.003783	Up
584	TMEM132E	0.046508	0.000102	0.003797	None
585	CDIP1	-0.53929	0.000104	0.003847	None
586	GALNS	-0.27293	0.000104	0.003853	None
587	ARMC10	-0.55803	0.000106	0.003899	None
588	PLAUR	1.567552	0.000108	0.003976	Up
589	FAM160A2	-0.55962	0.000109	0.00402	None
590	HSBP1L1	-0.67549	0.00011	0.00403	Down
591	LOC101928	-1.05553	0.000111	0.004051	Down
592	TM7SF3	-0.74077	0.000111	0.004054	Down
593	RP11-218F	0.120195	0.000111	0.004054	None
594	TRIM21	-0.61703	0.000113	0.004124	Down
595	EIF4ENIF1	-0.27056	0.000114	0.004129	None
596	COX18	-0.20216	0.000114	0.004129	None
597	GRWD1	-0.5012	0.000114	0.004129	None
598	ARHGAP22	-0.50273	0.000114	0.00413	None
599	C15orf61	-0.53227	0.000115	0.004145	None
600	PCGF2	0.148264	0.000116	0.004164	None
601	MVP	-0.6978	0.000116	0.004164	Down
602	FSCN1	-0.69067	0.000116	0.004164	Down
603	VTA1	-0.34304	0.000116	0.004164	None
604	TEX41	0.054585	0.000117	0.004199	None
605	KLK5	0.036803	0.000119	0.004274	None
606	FCRL1	0.728201	0.000122	0.004355	Up
607	E2F7	0.6429	0.000122	0.004355	Up
608	CTRL	0.174397	0.000122	0.004355	None
609	CLDN17	0.028363	0.000122	0.004355	None
610	SF3A2	-0.95505	0.000123	0.004355	Down
611	TIGD5	-0.45583	0.000124	0.004393	None
612	CCDC38	0.307957	0.000125	0.004412	None
613	TMEM216	-0.56025	0.000126	0.00445	None
614	PNMA6A	-0.72933	0.000127	0.004464	Down
615	TIGD3	0.538205	0.000127	0.004464	None
616	RP1-74M1	0.121361	0.000127	0.004464	None
617	RAI2	0.122087	0.000128	0.004485	None
618	DGAT1	-0.75438	0.000128	0.004485	Down
619	NPRL3	-0.60627	0.00013	0.004563	Down
620	ZFYVE26	-0.44446	0.000131	0.004569	None
621	LOC10050	0.470323	0.000132	0.004612	None
622	GABPA	0.395021	0.000132	0.004612	None
623	CTDSP1	-0.43299	0.000133	0.004638	None
624	ZNF689	-0.54779	0.000134	0.004638	None
625	SERPINB9	1.278972	0.000134	0.004639	Up
626	SERINC2	0.111927	0.000134	0.004639	None
627	NME6	-0.87044	0.000137	0.004748	Down
628	OSBPL10	0.104995	0.000138	0.004749	None
629	LOC28584	0.019632	0.000138	0.004749	None
630	LOC10050	0.025287	0.000138	0.004753	None
631	RP11-65D2	0.054597	0.000138	0.004753	None
632	IVD	-0.54814	0.000139	0.004767	None
633	TMEM41A	-0.46257	0.000139	0.004767	None
634	CTGF	1.57698	0.00014	0.004795	Up
635	PTER	-0.68137	0.000141	0.004801	Down
636	C4orf32	0.442188	0.000142	0.004817	None

637	PTGES	0.094085	0.000142	0.004817	None
638	RCSD1	0.750551	0.000143	0.004838	Up
639	GRHL2	0.044739	0.000144	0.004886	None
640	COQ6	-0.63225	0.000146	0.004936	Down
641	SLC17A7	0.174145	0.000146	0.004936	None
642	ACTR10	-0.39615	0.000146	0.004936	None
643	MARK4	-0.59282	0.000147	0.004936	Down
644	RFNG	-0.65379	0.000147	0.004936	Down
645	RP1-101G1	0.183244	0.000147	0.00495	None
646	F11-AS1	0.131372	0.000149	0.004997	None
647	CPTP	-0.39112	0.000149	0.005	None
648	RP4-613A2	0.259832	0.00015	0.005001	None
649	CORO1B	-0.78431	0.000151	0.005032	Down
650	EXT2	-0.44886	0.000152	0.005067	None
651	FDX1L	-0.72762	0.000153	0.005081	Down
652	HPS4	0.776444	0.000155	0.005129	Up
653	TMEM109	-0.59015	0.000155	0.005129	Down
654	SELPLG	-0.60555	0.000155	0.005129	Down
655	HGS	-0.43015	0.000155	0.005137	None
656	HOXD9	0.117816	0.000158	0.005208	None
657	SLC8A1-AS	0.757068	0.000158	0.005215	Up
658	COL5A1	1.283225	0.000159	0.005225	Up
659	INTS5	-0.47171	0.000159	0.005225	None
660	SDHC	-0.69184	0.00016	0.005257	Down
661	WWC3	-0.70017	0.000161	0.005282	Down
662	CTD-2008I	0.12885	0.000162	0.005283	None
663	WFDC12	0.114963	0.000162	0.005287	None
664	MOAP1	0.442831	0.000162	0.005287	None
665	PNPLA7	0.221958	0.000162	0.005287	None
666	PRR9	0.058678	0.000163	0.005309	None
667	CTDSPL2	0.436799	0.000164	0.005327	None
668	C9orf69	-0.59704	0.000164	0.005327	Down
669	LINC00312	0.142133	0.000165	0.005339	None
670	LOC10192	0.027829	0.000169	0.005446	None
671	KRTAP4-12	0.07289	0.000169	0.005446	None
672	DUSP18	-0.45206	0.000171	0.005515	None
673	SEC31A	-0.35636	0.000172	0.005515	None
674	AKIRIN2	-0.66484	0.000172	0.005515	Down
675	PI15	0.047224	0.000172	0.005519	None
676	KRTAP7-1	0.148833	0.000173	0.00552	None
677	LOC10272	0.107033	0.000173	0.00552	None
678	TET1	-0.8691	0.000173	0.005536	Down
679	CYP4F62P	0.036185	0.000175	0.005588	None
680	ANKRD52	-0.39419	0.000176	0.005596	None
681	ABCB8	0.128704	0.000176	0.005596	None
682	METRNL	1.573819	0.000176	0.005601	Up
683	IMPDH1	-0.61005	0.000178	0.005626	Down
684	RP3-333B1	0.019822	0.000178	0.005626	None
685	EFCAB2	0.877225	0.000179	0.005657	Up
686	CORT	0.432919	0.000179	0.005657	None
687	FAM98C	-0.63313	0.00018	0.005662	Down
688	VPS37A	-0.42924	0.00018	0.005677	None
689	ANKS6	-0.35353	0.000184	0.005798	None
690	ANKRD37	0.40335	0.000185	0.005807	None
691	ING3	0.656173	0.000186	0.005807	Up
692	TRMT1	-0.70073	0.000186	0.005807	Down
693	DNASE1L1	-0.61335	0.000186	0.005823	Down
694	GALNT10	-0.74054	0.000189	0.005897	Down

695	ZNF124	0.858573	0.00019	0.005918	Up
696	AC003973.	0.057073	0.000194	0.00604	None
697	CPXM2	0.113126	0.000195	0.00605	None
698	FAM217B	-0.72601	0.000195	0.00605	Down
699	DGKD	0.551852	0.000196	0.006074	None
700	LOC72950(	0.158558	0.000197	0.006087	None
701	LOC10192	0.241112	0.000199	0.006144	None
702	PRKCD	-1.12945	0.0002	0.006164	Down
703	C19orf25	-0.42562	0.0002	0.006164	None
704	ZNF331	0.888758	0.0002	0.006164	Up
705	FOXR2	0.077035	0.000201	0.006169	None
706	RGL2	-0.58116	0.000201	0.006169	None
707	MAST3	-0.73588	0.000202	0.006173	Down
708	PLD1	-0.74195	0.000202	0.006173	Down
709	IL2RB	0.724984	0.000205	0.00624	Up
710	ARL5B	0.876669	0.000205	0.00624	Up
711	ANKRD10	0.35473	0.000205	0.00624	None
712	IFITM1	-1.31095	0.000206	0.006255	Down
713	IGHV1-69	1.005787	0.000206	0.006267	Up
714	TMEM154	-0.80518	0.000207	0.006279	Down
715	CD209	0.048369	0.000207	0.00628	None
716	OR10H1	0.217097	0.000208	0.006283	None
717	RP11-126(	0.12809	0.000208	0.006283	None
718	RASD1	1.257455	0.000208	0.006288	Up
719	RNF123	-0.52996	0.000212	0.006375	None
720	RPAP2	-0.49066	0.000212	0.006375	None
721	SLC39A3	-0.71101	0.000214	0.006412	Down
722	RP11-884k	-0.35705	0.000214	0.006412	None
723	CMTM8	1.451205	0.000216	0.006455	Up
724	AP3M2	0.454301	0.000217	0.006499	None
725	LOC10192!	0.152365	0.000218	0.006499	None
726	HRSP12	-0.4333	0.000218	0.006499	None
727	RAB2B	-0.48612	0.000219	0.006529	None
728	SCAMP2	-0.4033	0.00022	0.006543	None
729	CTC-425F1	0.818731	0.000221	0.006573	Up
730	C6orf195	0.198798	0.000223	0.0066	None
731	UBQLN4	-0.30886	0.000223	0.0066	None
732	CAMTA2	-0.30899	0.000223	0.0066	None
733	CRLF2	0.362763	0.000224	0.006603	None
734	ZNF473	-0.54292	0.000225	0.006629	None
735	IL18	-0.76926	0.000225	0.006643	Down
736	GEMIN6	-0.47414	0.000226	0.006663	None
737	TMEM180	-0.43308	0.000227	0.006665	None
738	MIR3682	0.712013	0.00023	0.006737	Up
739	SLC2A3	1.303659	0.000231	0.006755	Up
740	FAM206A	-0.43036	0.000231	0.006755	None
741	KCNB1	0.02563	0.000231	0.00676	None
742	DSTNP2	-0.41803	0.000232	0.006768	None
743	FAM63B	-0.48995	0.000233	0.006768	None
744	ALS2	-0.86574	0.000233	0.006768	Down
745	KRT3	0.026221	0.000233	0.006768	None
746	SUMF1	-0.51283	0.000234	0.006796	None
747	RHBDD3	-0.34292	0.000235	0.00681	None
748	MED20	-0.34573	0.000235	0.00681	None
749	SLC22A18	-0.67725	0.000236	0.006814	Down
750	PPP2R2C	0.019046	0.000236	0.006814	None
751	SPRR1A	0.10492	0.000237	0.006822	None
752	LY6E	-0.93403	0.000237	0.006822	Down

753	DHX32	-0.3976	0.000238	0.006827	None
754	GGA3	-0.27695	0.000238	0.006827	None
755	PLOD1	-0.62252	0.000239	0.006845	Down
756	C19orf54	-0.52321	0.000239	0.006845	None
757	CBX6	-0.36808	0.000241	0.006899	None
758	ZNF326	0.545473	0.000241	0.006899	None
759	C2orf54	0.248007	0.000243	0.006927	None
760	NIT1	-0.57604	0.000245	0.006974	None
761	MGA	-0.74632	0.000247	0.007014	Down
762	STON2	-0.81611	0.000247	0.007014	Down
763	LOC28348	0.031142	0.000247	0.007017	None
764	RP11-67L3	0.09997	0.000248	0.007036	None
765	SMCHD1	0.523581	0.000249	0.007036	None
766	PRRX1	0.059582	0.000249	0.007036	None
767	MPHOSPH	-0.48171	0.00025	0.007045	None
768	GOLGA7	-0.40038	0.00025	0.007046	None
769	MICU1	-0.64073	0.00025	0.007046	Down
770	RBM33	0.536794	0.000252	0.007083	None
771	CUL4A	-0.4429	0.000252	0.007083	None
772	ARHGAP17	-0.61465	0.000253	0.007093	Down
773	SPR	-0.60206	0.000255	0.007153	Down
774	VPS72	-0.45869	0.000256	0.007153	None
775	RFX1	-0.27394	0.000256	0.007166	None
776	ZNF236	0.47962	0.000258	0.00719	None
777	TBC1D24	-0.70142	0.000259	0.007209	Down
778	FBXO6	-0.95926	0.00026	0.007246	Down
779	OLR1	0.918324	0.000261	0.007256	Up
780	TLR8-AS1	0.021238	0.000262	0.00726	None
781	SPC25	1.03739	0.000265	0.007339	Up
782	MTX2	-0.55826	0.000265	0.007342	None
783	LOC10050	0.657171	0.000267	0.007397	Up
784	UBE2L3	-0.44264	0.000269	0.007417	None
785	TRHR	0.03008	0.000269	0.007417	None
786	PLBD2	-0.36481	0.000271	0.007479	None
787	TCTN3	-0.45904	0.000272	0.00749	None
788	RAB14	-0.7269	0.000274	0.00752	Down
789	NOC3L	-0.51664	0.000275	0.007535	None
790	PUS3	-0.45185	0.000276	0.007576	None
791	MIB2	-0.25566	0.000277	0.007583	None
792	METTL13	-0.60634	0.000278	0.007589	Down
793	LOC40062	0.063874	0.000278	0.007597	None
794	LOC64307	-0.51826	0.000279	0.007597	None
795	FLNA	-0.81457	0.00028	0.007624	Down
796	GALNT6	-0.79956	0.000281	0.007635	Down
797	BCLAF1	0.537459	0.000282	0.007666	None
798	TRIM24	0.477372	0.000284	0.00769	None
799	PFKFB4	-0.36816	0.000284	0.00769	None
800	GATA6-AS	0.285025	0.000285	0.007722	None
801	CASP7	0.53772	0.000289	0.007822	None
802	RP11-499E	-0.63132	0.000292	0.007885	Down
803	MFF	-0.32869	0.000294	0.007909	None
804	URI1	-0.41477	0.000294	0.007909	None
805	NCOA1	-0.405	0.000294	0.007909	None
806	ETV3	1.079724	0.000295	0.00792	Up
807	LOC14547	1.665128	0.000295	0.007922	Up
808	LINC00521	0.034875	0.000296	0.007932	None
809	TMPRSS11	0.094399	0.000297	0.007943	None
810	PEX19	-0.35714	0.000297	0.00795	None

811	TICAM1	-0.52622	0.000301	0.008026	None
812	MIEF1	-0.48285	0.000307	0.008181	None
813	ZNF574	-0.55438	0.000308	0.008192	None
814	LOC10012ᵢ	1.301729	0.000308	0.008192	Up
815	NSF	0.336792	0.000313	0.008311	None
816	ABR	-0.41832	0.000314	0.008328	None
817	HEATR6	-0.58074	0.000314	0.008334	None
818	C8orf82	-0.85074	0.000316	0.00835	Down
819	CCDC137	-0.58479	0.000316	0.00835	None
820	GOLGA1	-0.62415	0.000316	0.008352	Down
821	SREBF1	-0.33684	0.000318	0.008377	None
822	MON1B	-0.57898	0.000318	0.008377	None
823	FUT8	-0.62803	0.000318	0.008377	Down
824	FBXO46	-0.5039	0.000319	0.008385	None
825	APOBEC3C	-0.49698	0.000322	0.008458	None
826	LINC01169	0.079795	0.000325	0.008509	None
827	XYLT2	-0.28208	0.000325	0.008509	None
828	C6orf123	0.027404	0.000327	0.008542	None
829	C18orf15	0.063242	0.000327	0.008542	None
830	TUBGCP6	-0.25958	0.000328	0.008563	None
831	GNG5	-0.40846	0.000333	0.008685	None
832	FZD2	-0.61658	0.000334	0.0087	Down
833	LOC10050!	0.227763	0.000337	0.008757	None
834	GBGT1	-0.88815	0.000343	0.008882	Down
835	TOR1B	-0.44622	0.000343	0.008882	None
836	FHIT	0.887381	0.000343	0.008882	Up
837	NR1H3	-1.02665	0.000345	0.008919	Down
838	OSM	0.719222	0.000345	0.008919	Up
839	EZH2	0.640458	0.000346	0.00894	Up
840	NAPRT	-0.67471	0.000347	0.00894	Down
841	RGS3	-0.35644	0.000349	0.008992	None
842	POLR1A	-0.49137	0.00035	0.009011	None
843	ENTPD1-A	-0.48175	0.000351	0.00902	None
844	ARPC4	-0.65284	0.000352	0.009028	Down
845	TMEM182	-0.29689	0.000355	0.009105	None
846	SLC9B2	-0.4023	0.000359	0.009181	None
847	MAPKAPK3	-0.44757	0.000363	0.009272	None
848	ARMCX5	-0.33411	0.000363	0.009272	None
849	CCDC102A	-0.56804	0.000364	0.009293	None
850	WDR83OS	-0.40619	0.000368	0.00936	None
851	LINC01243	0.03205	0.000368	0.00936	None
852	NKX6-3	0.174183	0.00037	0.009397	None
853	DSCR3	-0.34735	0.00037	0.009397	None
854	PDLIM2	-0.38967	0.000371	0.009415	None
855	TRMT1L	-0.60735	0.000373	0.009444	Down
856	PRR24	-0.5772	0.000377	0.009526	None
857	CAP1	-0.35362	0.000377	0.009526	None
858	PP7080	-0.75301	0.000378	0.009547	Down
859	RP1-217P2	0.187559	0.000382	0.009621	None
860	ZBTB25	-0.62906	0.000383	0.009642	Down
861	ZP4	0.01981	0.000384	0.009656	None
862	PRKAG3	0.134957	0.000386	0.009703	None
863	RALBP1	-0.30425	0.000392	0.009841	None
864	ZNF605	-0.316	0.000394	0.009863	None
865	LOC10192!	-0.62535	0.000394	0.009864	Down
866	GANAB	-0.63892	0.000394	0.009864	Down
867	CCDC53	-0.50474	0.000395	0.009876	None
868	HIPK1-AS1	0.333999	0.000397	0.009903	None

869	SIGIRR	-0.6364	0.0004	0.009976	Down
870	NKIRAS2	-0.43321	0.000402	0.009999	None
871	RP11-24P1	0.011223	0.000403	0.010007	None
872	DEK	0.392895	0.000404	0.010011	None
873	IL1A	0.613528	0.000404	0.010011	Up
874	GRASP	0.74545	0.000405	0.010011	Up
875	EVA1B	-0.73252	0.000405	0.010011	Down
876	TCL1B	0.247551	0.000405	0.010011	None
877	C17orf98	0.029349	0.000407	0.010039	None
878	LINC00276	0.077272	0.000407	0.010046	None
879	LRRC47	-0.34234	0.000408	0.010054	None
880	LOC10013:	0.285713	0.000416	0.010228	None
881	FBXO3	0.360023	0.000416	0.010228	None
882	C1orf50	-0.42429	0.000417	0.010228	None
883	ASB8	-0.37189	0.000417	0.010228	None
884	RP11-38C1	0.125089	0.000419	0.010265	None
885	OSBPL2	-0.40272	0.00042	0.010275	None
886	ANKIB1	-0.57848	0.000421	0.010288	None
887	CSRNP1	0.903776	0.000424	0.010343	Up
888	DAXX	-0.38415	0.000425	0.010367	None
889	MORC2	-0.63311	0.000426	0.010379	Down
890	SCRIB	-0.52864	0.000427	0.010388	None
891	KCNC3	0.130978	0.000428	0.010402	None
892	OR12D3	0.030164	0.00043	0.010417	None
893	TAF4	-0.25213	0.00043	0.010417	None
894	PHACTR4	-0.52412	0.000432	0.01046	None
895	DVL2	-0.29093	0.000434	0.010496	None
896	MRPS31	-0.7564	0.000436	0.010535	Down
897	ESRRA	-0.24837	0.000436	0.010535	None
898	TIPARP	0.865748	0.000438	0.010537	Up
899	TRAPPC2L	-0.6325	0.000438	0.010537	Down
900	RNF220	-0.45713	0.000438	0.010537	None
901	MLYCD	-0.3117	0.000438	0.010537	None
902	INTS3	-0.31401	0.000439	0.010541	None
903	FAM132B	-1.30186	0.000442	0.010591	Down
904	RAB6A	0.439129	0.000442	0.010591	None
905	ERLIN2	-0.60093	0.000443	0.010607	Down
906	LOC10050(	0.17282	0.000451	0.010781	None
907	C1orf100	0.097234	0.000452	0.010782	None
908	RBMX2	-0.32106	0.000453	0.010814	None
909	ADCK3	-0.32003	0.000457	0.010881	None
910	METTL8	-0.64919	0.000458	0.010881	Down
911	TMEM63A	-0.35156	0.000458	0.010881	None
912	BTG1	0.754845	0.000458	0.010886	Up
913	PAQR7	-0.53625	0.000459	0.010888	None
914	SIPA1	-0.81425	0.00046	0.010896	Down
915	FAM110A	-0.44826	0.000462	0.010924	None
916	ARHGEF9	-0.48554	0.000462	0.010924	None
917	RGS1	1.718009	0.000466	0.010995	Up
918	SSBP4	-0.53251	0.000466	0.010995	None
919	POSTN	0.010459	0.000468	0.011021	None
920	STAG3	0.969614	0.00047	0.011065	Up
921	LOC10192!	0.032512	0.000471	0.011077	None
922	LOC10192!	0.194781	0.000473	0.011111	None
923	BORA	-0.70047	0.000474	0.011111	Down
924	STAT1	-0.88816	0.000474	0.011117	Down
925	ZC3H10	0.308517	0.000475	0.011127	None
926	CNPY3	-0.46229	0.000479	0.011196	None

927	DUSP4	0.561646	0.000479	0.011196	None
928	ZNF142	-0.39933	0.000483	0.011265	None
929	EN2	0.065322	0.000484	0.011289	None
930	ST13	-0.34855	0.000486	0.01131	None
931	PVRIG	0.592229	0.000487	0.01131	Up
932	CMTR1	-0.4486	0.000487	0.01131	None
933	ANKLE1	-1.08442	0.000487	0.01131	Down
934	POLR2C	-0.54029	0.000488	0.01131	None
935	CARD6	-0.63513	0.000488	0.011313	Down
936	SCYL3	-0.42991	0.000492	0.011383	None
937	MSRB1	-0.70935	0.000494	0.011414	Down
938	THTPA	-0.66347	0.000498	0.01149	Down
939	SLC10A1	0.251313	0.000499	0.011491	None
940	IL21R	0.251994	0.000499	0.011491	None
941	IGF2BP2	-0.38483	0.000501	0.011521	None
942	RMND1	-0.63321	0.000501	0.011525	Down
943	LOC10029:	0.553372	0.000506	0.011627	None
944	NFIC	-0.51362	0.000507	0.011631	None
945	FAM3A	-0.39058	0.000508	0.011632	None
946	RGMA	0.160852	0.000508	0.011637	None
947	SERPING1	-0.8162	0.00051	0.011645	Down
948	TMEM219	-0.38037	0.00051	0.011645	None
949	MAFF	1.554235	0.000512	0.011682	Up
950	NAPB	-0.45983	0.000515	0.011736	None
951	CHCHD7	-0.6517	0.000515	0.011738	Down
952	AGPAT1	-0.76807	0.000516	0.01174	Down
953	ADIPOQ	0.164969	0.000517	0.01174	None
954	IL7R	1.427509	0.000518	0.011755	Up
955	RBM43	-0.4008	0.000518	0.011755	None
956	TMED3	-0.36727	0.00052	0.011783	None
957	BLOC1S2	-0.51513	0.000521	0.011783	None
958	EXOC1	-0.38071	0.000521	0.011783	None
959	RPL23AP3:	0.637012	0.000525	0.011853	Up
960	BCL6B	0.186185	0.000525	0.011853	None
961	LINC00466	0.046292	0.000528	0.011899	None
962	ASS1	-0.69664	0.00053	0.011927	Down
963	VPS11	-0.38804	0.000532	0.011948	None
964	UBFD1	-0.47473	0.000532	0.011948	None
965	UCMA	0.095969	0.000535	0.011997	None
966	POLR3F	0.34341	0.000535	0.012	None
967	SF3B4	-0.59576	0.000542	0.012133	Down
968	TRIM6	-0.96794	0.000542	0.012133	Down
969	NXF3	-0.65696	0.000545	0.012167	Down
970	PAXIP1OS	-0.3722	0.000545	0.012167	None
971	PLEKHG4	-0.8883	0.000546	0.012187	Down
972	WDR5B	-0.75314	0.000548	0.012197	Down
973	COL18A1	-0.62519	0.000548	0.012197	Down
974	GNPTG	-0.49258	0.000549	0.012197	None
975	ASNA1	-0.6664	0.000549	0.012197	Down
976	LINC00327	0.147258	0.000551	0.012219	None
977	SPG21	-0.19255	0.000552	0.012219	None
978	GEM	1.196076	0.000552	0.012219	Up
979	RBMS1	-0.54749	0.000554	0.012253	None
980	OXLD1	-0.51825	0.000557	0.012301	None
981	CBY1	-0.31758	0.000558	0.012307	None
982	RAD23A	-0.69075	0.00056	0.012329	Down
983	MRM1	-0.35816	0.00056	0.012329	None
984	GPBP1L1	-0.58756	0.000561	0.012329	Down

985	ACAP1	-0.72431	0.000561	0.012329	Down
986	NCKIPSD	0.308769	0.000562	0.012336	None
987	N4BP3	0.167132	0.000562	0.012336	None
988	MS4A12	0.0328	0.000563	0.012336	None
989	ANAPC13	-0.16832	0.000564	0.012352	None
990	LINC01206	0.100911	0.000565	0.012352	None
991	LINC01351	0.015386	0.000567	0.0124	None
992	YDJC	-0.45621	0.000569	0.012422	None
993	FYB	-1.34371	0.000571	0.012442	Down
994	C1orf194	0.059945	0.000571	0.012446	None
995	DDIT4	1.499743	0.000572	0.012449	Up
996	NUP210	-0.70723	0.000573	0.012453	Down
997	IER3	1.989315	0.000575	0.012475	Up
998	ZBTB9	-0.50667	0.000575	0.012475	None
999	PLOD3	-0.5097	0.00058	0.012574	None
1000	ICOSLG	0.215895	0.000583	0.01263	None
1001	TEX261	-0.35582	0.000585	0.012654	None
1002	MRPL46	-0.47305	0.000585	0.012654	None
1003	MUC3	0.103951	0.000587	0.012673	None
1004	RRM2B	0.574045	0.000588	0.012673	None
1005	RGS16	0.891854	0.000588	0.012677	Up
1006	CCL20	1.75279	0.000589	0.012686	Up
1007	MICALL1	-0.5421	0.000591	0.012705	None
1008	RASD2	0.034487	0.000594	0.01275	None
1009	RP11-108K	0.092642	0.000595	0.01276	None
1010	GATAD1	-0.63987	0.0006	0.012835	Down
1011	GTF2F1	-0.63837	0.000601	0.012835	Down
1012	ENG	-0.4471	0.000601	0.012835	None
1013	E2F8	1.006669	0.000602	0.012835	Up
1014	FAM127B	-0.36563	0.000602	0.012835	None
1015	LOC28493	0.116371	0.000602	0.012835	None
1016	GPR183	1.620344	0.000602	0.012835	Up
1017	ZDHHC9	-0.43498	0.000603	0.012839	None
1018	FAM84B	-0.46866	0.000605	0.012855	None
1019	RBCK1	-0.37343	0.000605	0.012855	None
1020	HOXD4	0.058977	0.000606	0.012855	None
1021	JMJD8	-0.40855	0.000606	0.012855	None
1022	B3GALT5	0.007888	0.000607	0.012855	None
1023	CISD1	-0.67024	0.00061	0.012916	Down
1024	ZNF609	-0.57701	0.000611	0.012916	None
1025	PCNA	0.525294	0.000612	0.012923	None
1026	ERAL1	-0.65893	0.000612	0.012923	Down
1027	CTD-2292I	-0.90275	0.000616	0.012989	Down
1028	LOC10192	0.024362	0.000618	0.013013	None
1029	DEF8	-0.50349	0.000619	0.013019	None
1030	PTPN23	-0.21731	0.00062	0.013019	None
1031	ZNF747	-0.48716	0.00062	0.013019	None
1032	SLC16A9	-1.1246	0.00062	0.013019	Down
1033	DLG5	-0.58978	0.000622	0.013048	Down
1034	RP1-8B22.	0.166239	0.000623	0.013051	None
1035	ANKRD44	0.586239	0.000626	0.013094	Up
1036	AMOTL1	0.236198	0.000628	0.013118	None
1037	SCN3A	1.558243	0.00063	0.013166	Up
1038	GADD45B	1.251984	0.000632	0.013186	Up
1039	PROC	0.524897	0.000642	0.013378	None
1040	C11orf30	0.274947	0.000645	0.013428	None
1041	IQCK	0.165408	0.000646	0.013428	None
1042	RPL7L1	-0.36748	0.000646	0.013428	None

1043	GTPBP2	-0.38785	0.000647	0.013433	None
1044	MED11	-0.65512	0.000649	0.013459	Down
1045	LHX4-AS1	-0.65513	0.000653	0.01353	Down
1046	RAB8A	-0.63285	0.000656	0.01358	Down
1047	SARS	-0.4713	0.000657	0.01358	None
1048	TGFBR1	1.060522	0.000657	0.01358	Up
1049	CRIM1	0.827945	0.000659	0.013613	Up
1050	LOC33980:	-0.61569	0.000662	0.013657	Down
1051	FAM120AC	-0.45627	0.000663	0.013664	None
1052	ZNF107	0.539033	0.000667	0.013712	None
1053	LOC10050	0.100291	0.000667	0.013712	None
1054	LMO2	-0.73223	0.000667	0.013712	Down
1055	DVL1	-0.56676	0.000669	0.013729	None
1056	MOB2	-0.46583	0.000669	0.013729	None
1057	PTCH1	0.013955	0.000673	0.01379	None
1058	GALR3	0.034742	0.000678	0.013875	None
1059	AP2A2	-0.679	0.000679	0.013875	Down
1060	CASP5	0.163734	0.000681	0.013909	None
1061	RP11-45M	0.122009	0.000682	0.013909	None
1062	GIP	0.095849	0.000682	0.013911	None
1063	CFL2	-0.60149	0.000686	0.013981	Down
1064	REPS2	-0.46688	0.000691	0.014055	None
1065	ELMO1	-0.99534	0.000693	0.014094	Down
1066	KIAA1033	0.65513	0.000694	0.014094	Up
1067	SUOX	-0.43335	0.000695	0.0141	None
1068	KLF10	0.97894	0.000696	0.014117	Up
1069	HELZ	-0.72045	0.000697	0.014117	Down
1070	SH3TC1	0.651117	0.000701	0.014181	Up
1071	CCZ1	0.211061	0.000703	0.014213	None
1072	GPR56	-0.49601	0.000707	0.014286	None
1073	SAFB	-0.72836	0.000709	0.0143	Down
1074	NAA35	-0.34914	0.000711	0.014334	None
1075	ZNF485	-0.54481	0.000712	0.014335	None
1076	MPV17	-0.51839	0.000712	0.014335	None
1077	PRPSAP1	-0.17063	0.000714	0.014361	None
1078	CSF2	0.160605	0.000715	0.014369	None
1079	IDH1	-0.74935	0.000719	0.014423	Down
1080	BFAR	-0.40966	0.000727	0.014584	None
1081	OARD1	-0.39815	0.000729	0.014598	None
1082	KL	0.626919	0.000731	0.014628	Up
1083	VRK2	-0.44662	0.000732	0.014628	None
1084	RRNAD1	-0.37783	0.000734	0.014655	None
1085	CIZ1	-0.67658	0.000736	0.014673	Down
1086	PAQR3	0.338973	0.000736	0.014673	None
1087	SFI1	-0.37261	0.000737	0.01468	None
1088	CHST12	-0.52938	0.000743	0.014788	None
1089	TAF8	-0.43783	0.000745	0.014795	None
1090	OR7E104P	0.145906	0.000745	0.014795	None
1091	ZNRF1	-0.65452	0.000745	0.014795	Down
1092	BTBD3	0.621125	0.000747	0.014822	Up
1093	ARRB2	-0.66236	0.000748	0.014829	Down
1094	ADNP	0.312863	0.000749	0.014831	None
1095	LOC10192	0.101531	0.000758	0.014981	None
1096	SLC12A2	0.56823	0.000758	0.014984	None
1097	SUCO	0.384808	0.000761	0.015026	None
1098	STAG2	0.680308	0.000764	0.015062	Up
1099	CTB-113P1	0.0452	0.000771	0.015197	None
1100	AGAP3	0.061085	0.000773	0.015197	None

1101	DCTN1	-0.44282	0.000773	0.015197	None
1102	NMT2	0.601011	0.000773	0.015197	Up
1103	PDGFC	-0.73396	0.000775	0.015208	Down
1104	TRAM2-AS	-0.6398	0.000776	0.015221	Down
1105	LINC00667	-0.36314	0.000777	0.015234	None
1106	NODAL	0.095672	0.000778	0.015238	None
1107	CRTC2	-0.47926	0.000779	0.015238	None
1108	LINC00656	0.105323	0.00078	0.015238	None
1109	DLG3	0.092578	0.000784	0.01528	None
1110	TMEM39B	-0.61432	0.000784	0.01528	Down
1111	VAPB	-0.57936	0.000785	0.01528	None
1112	CLK2	-0.44916	0.000785	0.01528	None
1113	ATP13A1	-0.54486	0.000785	0.01528	None
1114	STAT3	-0.67011	0.000787	0.015286	Down
1115	KAZALD1	0.162036	0.000787	0.015286	None
1116	ANGEL1	-0.32956	0.000788	0.015299	None
1117	SLC25A1	-0.66408	0.000792	0.015349	Down
1118	ESPNL	0.050123	0.000792	0.015349	None
1119	PNKD	-0.69661	0.000795	0.015391	Down
1120	KIAA0753	-0.55473	0.000802	0.015512	None
1121	ZBTB48	-0.60121	0.000803	0.015512	Down
1122	SUN1	0.346939	0.000804	0.015522	None
1123	FAM186B	0.136384	0.000807	0.015542	None
1124	POGLUT1	-0.39609	0.000807	0.015542	None
1125	WDR45B	0.444968	0.000807	0.015542	None
1126	GPR17	0.013852	0.000812	0.015621	None
1127	RBM4B	-0.61755	0.000819	0.015737	Down
1128	LYPLA2	-0.35374	0.000823	0.015804	None
1129	CKAP2	0.738733	0.000825	0.015817	Up
1130	C1orf35	-0.42726	0.000826	0.015827	None
1131	ACAN	0.118839	0.000827	0.015838	None
1132	LOC10029	0.540882	0.000829	0.015843	None
1133	RAD17	-0.47014	0.000829	0.015843	None
1134	ALDH5A1	-0.48047	0.00083	0.015843	None
1135	ANKS1A	-0.48369	0.000832	0.015874	None
1136	ANKS3	-0.391	0.000835	0.015912	None
1137	FAM13A	-0.60495	0.000837	0.015934	Down
1138	TECPR1	-0.37169	0.000839	0.015934	None
1139	AEBP2	0.310971	0.000839	0.015934	None
1140	DDX17	0.780394	0.000839	0.015934	Up
1141	CEP164	-0.28056	0.00084	0.015935	None
1142	PSMG2	-0.33127	0.00084	0.015935	None
1143	MAP7D1	-0.71072	0.000843	0.015971	Down
1144	TNFRSF1A	-0.6377	0.000845	0.015986	Down
1145	HEATR1	-0.38529	0.000847	0.016018	None
1146	THAP10	-0.60132	0.00085	0.016066	Down
1147	MAPK3	-0.27448	0.000853	0.016104	None
1148	REG3G	0.09941	0.000854	0.016105	None
1149	OSR2	0.324968	0.000855	0.016105	None
1150	HNRNPA0	0.47611	0.000856	0.016126	None
1151	LOC10192	0.140332	0.00086	0.016179	None
1152	UBAP2	-0.38694	0.000867	0.01629	None
1153	ATP6V1E1	-0.37673	0.00087	0.016329	None
1154	KLHDC8B	-0.93681	0.00087	0.016329	Down
1155	UPF2	-0.43087	0.000874	0.016367	None
1156	LOC10272	0.169514	0.000874	0.016367	None
1157	TPRN	-0.15753	0.000874	0.016367	None
1158	C10orf113	0.042363	0.00088	0.016451	None

1159	MXD3	0.483438	0.000883	0.016502	None
1160	KRT78	0.133655	0.000886	0.016522	None
1161	LDLRAP1	-0.54035	0.000886	0.016522	None
1162	OXR1	-0.6202	0.000887	0.016522	Down
1163	MRPS6	-0.42584	0.00089	0.016569	None
1164	CPN2	0.196076	0.000892	0.016588	None
1165	LOC10192	0.048181	0.000892	0.016588	None
1166	RAB40A	0.132153	0.000896	0.016626	None
1167	NR4A1	0.876683	0.000896	0.016626	Up
1168	LNPEP	0.695886	0.000898	0.016626	Up
1169	FLYWCH1	-0.448	0.000898	0.016626	None
1170	EIF4H	0.356244	0.000898	0.016626	None
1171	SEPN1	-0.76668	0.000906	0.016748	Down
1172	ZNF598	-0.25049	0.000907	0.016762	None
1173	NIPAL3	-0.50259	0.00091	0.016797	None
1174	LOC10192	0.122079	0.000912	0.016823	None
1175	MAP3K8	0.951974	0.000915	0.016868	Up
1176	DDX19A	-0.78509	0.000921	0.016967	Down
1177	ANP32A	0.297827	0.000925	0.017017	None
1178	OR52D1	0.087188	0.000929	0.017077	None
1179	AKAP3	0.016067	0.000933	0.017129	None
1180	TXLNA	-0.29261	0.000937	0.01717	None
1181	TMEM17	-0.60539	0.000937	0.01717	Down
1182	GTF3C6	-0.35246	0.000937	0.01717	None
1183	PAK2	-0.41269	0.00094	0.017201	None
1184	FAM189B	-0.1651	0.000942	0.01722	None
1185	SEC23IP	-0.41	0.000945	0.01727	None
1186	PNPO	-0.65835	0.000947	0.017286	Down
1187	SLA	-0.68349	0.000949	0.017306	Down
1188	HECTD3	-0.5121	0.00095	0.017315	None
1189	BET1L	-0.31807	0.000952	0.017341	None
1190	PACRGL	-0.43945	0.000962	0.017504	None
1191	YAE1D1	-0.66865	0.000964	0.017528	Down
1192	ME2	-0.61867	0.000965	0.017532	Down
1193	SP2	0.04731	0.000966	0.017532	None
1194	IQCD	0.178227	0.000969	0.017566	None
1195	ATP6V1B2	-0.52505	0.000969	0.017567	None
1196	MPP5	-0.43993	0.000971	0.017572	None
1197	RCCD1	-0.31161	0.000977	0.017683	None
1198	RNF152	0.119434	0.000979	0.017703	None
1199	NUP50	-0.5303	0.000987	0.01783	None
1200	C12orf79	0.192813	0.000989	0.017855	None
1201	15-Sep	0.363578	0.000992	0.017879	None
1202	PPAT	-0.49671	0.000995	0.017911	None
1203	RP11-143k	0.0832	0.000995	0.017911	None
1204	POC5	-0.54116	0.000997	0.017911	None
1205	NRF1	0.425977	0.000997	0.017911	None
1206	GTF3C1	-0.4088	0.001	0.017964	None
1207	SPP1	0.610644	0.001003	0.01799	Up
1208	DAPK1	-0.35145	0.001004	0.017991	None
1209	ULK3	-0.51299	0.001005	0.017991	None
1210	FGFR1OP2	-0.69749	0.001005	0.017991	Down
1211	TSPAN32	-0.65553	0.001007	0.018016	Down
1212	CTSZ	-0.56563	0.001014	0.018116	None
1213	C3orf52	0.385644	0.001016	0.018137	None
1214	LINC00424	0.056386	0.00102	0.018187	None
1215	MAPK8	0.51353	0.001021	0.018192	None
1216	LINC00094	-0.70814	0.001022	0.018192	Down

1217	FLJ10038	0.575788	0.001024	0.018205	None
1218	SNX25	0.076715	0.001025	0.018205	None
1219	ANKEF1	-0.30967	0.001025	0.018205	None
1220	TPMT	-0.41338	0.001027	0.018233	None
1221	ZNF787	-0.29456	0.001029	0.01825	None
1222	RP11-350F	-0.45351	0.001039	0.018421	None
1223	ARHGEF38	0.038257	0.001043	0.018462	None
1224	LAD1	0.06555	0.001045	0.018483	None
1225	HIP1R	0.371109	0.001051	0.018581	None
1226	FUNDC2	0.190204	0.001052	0.018581	None
1227	HLTF	-0.54586	0.001055	0.01862	None
1228	TRIM58	-0.73517	0.001058	0.018653	Down
1229	AKIP1	-0.35906	0.001059	0.018653	None
1230	BRD1	0.43531	0.001064	0.018718	None
1231	JAM2	0.862331	0.001064	0.018718	Up
1232	THAP7	-0.40474	0.001071	0.018822	None
1233	RBM18	-0.43563	0.001072	0.018822	None
1234	ACOT13	-0.7576	0.001074	0.01885	Down
1235	RABEP2	-0.39357	0.001076	0.018873	None
1236	MBNL3	0.506007	0.001081	0.018945	None
1237	SOCS2-AS	1.192668	0.001087	0.019021	Up
1238	RIMKLB	0.586216	0.001092	0.019093	Up
1239	BPIFA1	0.081553	0.001092	0.019093	None
1240	MED8	-0.50627	0.001097	0.019158	None
1241	DALRD3	-0.38711	0.001101	0.019208	None
1242	VIPAS39	-0.37871	0.001103	0.019224	None
1243	DAOA	0.066977	0.001104	0.019238	None
1244	OSGEPL1	-0.46157	0.00111	0.019321	None
1245	PLK3	0.57784	0.001112	0.019344	None
1246	MORN3	0.131782	0.001116	0.019399	None
1247	GNPAT	-0.26908	0.001122	0.019487	None
1248	BLNK	2.194734	0.001124	0.019511	Up
1249	APOLD1	0.914157	0.001133	0.019648	Up
1250	LAPTM5	0.468604	0.001143	0.019788	None
1251	BACE2	-1.19175	0.001143	0.019788	Down
1252	ZNF513	-0.53709	0.001145	0.019812	None
1253	ZAK	-0.50571	0.001147	0.019815	None
1254	WDR75	-0.371	0.001151	0.019874	None
1255	HMGB3P1	0.467032	0.001153	0.019899	None
1256	OR5AK4P	0.109784	0.001155	0.019914	None
1257	LOC28569:	0.007721	0.001157	0.019924	None
1258	SPRTN	0.533123	0.00116	0.019968	None
1259	EXD2	-0.57262	0.001175	0.020203	None
1260	LINC00535	0.149684	0.001176	0.020217	None
1261	RNF146	-0.48223	0.001181	0.020274	None
1262	BHLHE40	0.723012	0.001184	0.020321	Up
1263	DPP3	-0.47974	0.001187	0.020328	None
1264	LOC10272	0.165568	0.001187	0.020328	None
1265	EZH1	0.294507	0.001187	0.020328	None
1266	COBL	0.428478	0.00119	0.020349	None
1267	IFIT1	-1.80473	0.001191	0.020356	Down
1268	LOC10013:	0.101728	0.001192	0.020356	None
1269	RHOG	-0.60373	0.001193	0.020356	Down
1270	IL17RC	0.124278	0.001195	0.020377	None
1271	KATNAL1	0.417748	0.001209	0.020601	None
1272	CLDN4	0.050279	0.00121	0.020601	None
1273	CLN5	-0.53167	0.001215	0.020657	None
1274	PIAS4	-0.35287	0.001216	0.020657	None

1275	HAUS3	0.425598	0.001216	0.020657	None
1276	HTATIP2	-0.68069	0.001224	0.020768	Down
1277	LOC10050(	-0.21107	0.001229	0.020839	None
1278	SLC25A38	-0.43812	0.001231	0.02086	None
1279	AGXT2	0.059192	0.001232	0.02086	None
1280	DEXI	-0.6427	0.001233	0.02086	Down
1281	SLC16A6	0.678326	0.001234	0.020866	Up
1282	LONP1	-0.54142	0.001237	0.020901	None
1283	RNF167	-0.33802	0.001244	0.020999	None
1284	GZF1	0.480124	0.001249	0.021057	None
1285	PGAP3	-0.38161	0.001252	0.021089	None
1286	KLHL24	0.597725	0.001252	0.021089	Up
1287	ZNF432	0.496278	0.001254	0.021094	None
1288	MRPL40	-0.49639	0.001257	0.021132	None
1289	FAM117A	-0.68983	0.001261	0.02117	Down
1290	BRE	-0.2874	0.001261	0.02117	None
1291	CLDN2	0.15323	0.001265	0.021191	None
1292	EGFLAM-A	0.042325	0.001265	0.021191	None
1293	BICD2	-0.58472	0.001265	0.021191	None
1294	CLEC2D	0.37623	0.001266	0.021193	None
1295	GRAMD1A	-0.60862	0.001269	0.021217	Down
1296	SAMD9L	-0.83228	0.001271	0.021229	Down
1297	ARHGEF19	-0.36744	0.001274	0.021246	None
1298	LOC28374!	0.014228	0.001274	0.021246	None
1299	EMC10	-0.28237	0.001274	0.021246	None
1300	USP21	-0.61684	0.001277	0.021275	Down
1301	FXN	-0.41574	0.001281	0.021302	None
1302	TCTEX1D2	0.640763	0.001281	0.021302	Up
1303	RP11-308D	0.138314	0.001288	0.0214	None
1304	RASA2	0.710401	0.001291	0.021447	Up
1305	RSPO1	0.118551	0.001294	0.021475	None
1306	SOS1	-1.0887	0.001298	0.021518	Down
1307	HAS1	0.964413	0.001301	0.021552	Up
1308	MTRR	-0.34847	0.001305	0.02161	None
1309	SLC48A1	-0.87669	0.001313	0.021729	Down
1310	GH1	0.221386	0.001315	0.021734	None
1311	LOC10050	0.067923	0.001318	0.021734	None
1312	HPS1	-0.39214	0.001318	0.021734	None
1313	SLC16A14	1.490509	0.001318	0.021734	Up
1314	ATP6V0C	-0.72328	0.00132	0.021734	Down
1315	CTSA	-0.85238	0.001321	0.021734	Down
1316	NBPF1	-0.42523	0.001321	0.021734	None
1317	ZKSCAN4	-0.56003	0.001323	0.021747	None
1318	NMB	-0.4199	0.001328	0.02182	None
1319	CCDC115	-0.38114	0.001331	0.021841	None
1320	EP300-AS1	0.49811	0.001331	0.021841	None
1321	LGALS9	-0.62036	0.001333	0.021852	Down
1322	PFKFB3	0.970203	0.001335	0.021875	Up
1323	ZMYM1	-0.59408	0.001338	0.021907	Down
1324	PMM2	-0.44228	0.001341	0.021926	None
1325	PIP5K1B	-0.35653	0.001363	0.022282	None
1326	ZNF839	-0.21223	0.001367	0.022321	None
1327	ISCU	-0.50686	0.001368	0.022321	None
1328	MEGF8	-0.4612	0.001369	0.02233	None
1329	SMARCAL1	-0.3985	0.001371	0.022336	None
1330	ZBED8	-0.85044	0.001374	0.022366	Down
1331	NPEPPS	-0.46469	0.001376	0.022386	None
1332	ZNF579	-0.37879	0.00138	0.022434	None

1333	ATG5	-0.51283	0.001388	0.022547	None
1334	DNAJC22	0.073392	0.001399	0.022713	None
1335	SPRYD3	-0.43044	0.001401	0.022714	None
1336	PIGZ	-0.44505	0.001401	0.022714	None
1337	RP11-326I	0.164832	0.001404	0.022737	None
1338	ACTN4	-0.6134	0.001407	0.022776	Down
1339	MMP3	0.0276	0.00141	0.022796	None
1340	EGFL7	-0.84351	0.001412	0.02282	Down
1341	FAM120B	-0.42059	0.001416	0.022866	None
1342	SLC26A7	0.02346	0.00142	0.022901	None
1343	ATXN2	-0.54821	0.001421	0.022901	None
1344	RP11-469N	0.805687	0.001421	0.022901	Up
1345	IKBKB	-0.67644	0.001423	0.022917	Down
1346	EEF1A1	0.089793	0.001437	0.023108	None
1347	MORN1	0.025141	0.001437	0.023108	None
1348	LOC10012	0.175584	0.001445	0.023218	None
1349	RAB17	0.162359	0.00145	0.023259	None
1350	SLC10A3	-0.54154	0.00145	0.023259	None
1351	C15orf48	1.268513	0.001452	0.023272	Up
1352	PQLC3	-0.24242	0.001453	0.023272	None
1353	EHBP1L1	-0.55664	0.001455	0.023282	None
1354	ZNF674	0.036815	0.001457	0.023282	None
1355	ZNF704	0.23605	0.001457	0.023282	None
1356	SUPT20H	-0.36733	0.001464	0.023342	None
1357	LOC10192	0.013143	0.001464	0.023342	None
1358	LHFPL2	-0.61823	0.001465	0.023342	Down
1359	GPC2	0.440268	0.001465	0.023342	None
1360	LCE1E	0.027833	0.001466	0.023342	None
1361	MXI1	-0.72044	0.001467	0.023348	Down
1362	OPCML	0.037457	0.001469	0.02335	None
1363	KRBOX4	-0.38496	0.001471	0.023378	None
1364	NAA30	-0.20066	0.001475	0.023406	None
1365	ZFAND2B	-0.40301	0.001475	0.023406	None
1366	C9orf78	-0.25888	0.00148	0.023459	None
1367	PMS2P5	0.908692	0.001489	0.023588	Up
1368	TMEM184(	-0.36426	0.00149	0.023592	None
1369	PHF12	-0.28829	0.001496	0.023635	None
1370	GTF3C2	-0.30996	0.001496	0.023635	None
1371	ELF4	-0.42969	0.001496	0.023635	None
1372	ARFGAP1	-0.39751	0.001498	0.023649	None
1373	NR4A3	1.339755	0.001502	0.023674	Up
1374	CEP131	-0.35585	0.001502	0.023674	None
1375	TAMM41	-0.34026	0.001515	0.023865	None
1376	SMARCC2	-0.68552	0.001519	0.023913	Down
1377	PHYHIPL	0.067677	0.001524	0.023973	None
1378	YWHAQ	0.298073	0.00153	0.024037	None
1379	QDPR	-0.25019	0.001533	0.024074	None
1380	MMS19	-0.4367	0.001536	0.02408	None
1381	LOC10192	0.064376	0.001536	0.02408	None
1382	SDS	0.128364	0.001538	0.02408	None
1383	ADAR	-0.32377	0.001539	0.02408	None
1384	SF3B6	-0.32783	0.00154	0.02408	None
1385	BZW1	0.288309	0.001541	0.02408	None
1386	FOPNL	-0.27301	0.001541	0.02408	None
1387	S100A6	-0.48716	0.001548	0.024176	None
1388	CACNG6	0.082659	0.001559	0.024327	None
1389	NASP	0.459515	0.001562	0.024347	None
1390	LOC10192	0.071462	0.001572	0.024485	None

1391	PLXND1	-0.57929	0.001577	0.024551	None
1392	WIF1	0.473505	0.001579	0.024555	None
1393	LOC93622	-0.43995	0.00158	0.024555	None
1394	TMUB2	-0.35706	0.001584	0.024608	None
1395	UCN	-0.45515	0.001586	0.024611	None
1396	PRDM4	-0.27873	0.001587	0.024611	None
1397	PCBD1	-0.76409	0.001588	0.024622	Down
1398	METTL10	-0.64836	0.001593	0.024679	Down
1399	FW340027	0.1467	0.001605	0.024818	None
1400	CLDN15	-0.4904	0.001607	0.024818	None
1401	UBAP2L	-0.42238	0.001607	0.024818	None
1402	SARM1	-0.50906	0.001607	0.024818	None
1403	SUSD1	-0.52322	0.001608	0.024818	None
1404	ARF3	-0.60102	0.001612	0.024857	Down
1405	LZTS2	-0.27376	0.001615	0.024889	None
1406	ERC1	-0.40814	0.001617	0.024903	None
1407	PIK3CA	0.456303	0.001619	0.024914	None
1408	ATXN7L1	-0.32945	0.00162	0.024921	None
1409	MADCAM1	0.202943	0.001625	0.024974	None
1410	RP11-410E	0.097405	0.001633	0.025074	None
1411	SOAT2	0.124351	0.001635	0.025085	None
1412	NXPE1	0.098835	0.001636	0.025085	None
1413	KRTAP5-A	0.038704	0.001638	0.025105	None
1414	SLMO1	-0.55775	0.001652	0.0253	None
1415	KIAA1279	-0.66358	0.00166	0.025397	Down
1416	SP1	-0.51513	0.001662	0.025414	None
1417	TPRA1	-0.58866	0.001663	0.025419	Down
1418	C9orf114	-0.36808	0.001668	0.025466	None
1419	CHFR	-0.33024	0.001669	0.025466	None
1420	COA7	-0.54869	0.001671	0.025466	None
1421	LINC00244	0.042294	0.001672	0.025466	None
1422	MRPL45	-0.40949	0.001672	0.025466	None
1423	C7orf43	-0.33349	0.001673	0.025467	None
1424	TBC1D5	-0.44513	0.001675	0.025469	None
1425	KLHL36	-0.43748	0.001681	0.025539	None
1426	WDR91	-0.60275	0.001688	0.025631	Down
1427	PPFIA4	0.088974	0.001689	0.025635	None
1428	RP11-18F1	0.026145	0.001693	0.02568	None
1429	GBAP1	-0.45076	0.001702	0.025793	None
1430	MARS2	-0.7368	0.001705	0.025814	Down
1431	PPIL2	-0.35522	0.001723	0.026072	None
1432	DHRSX	-0.44123	0.001729	0.026148	None
1433	RNF214	-0.3454	0.001733	0.026191	None
1434	RPRD1B	0.250013	0.001739	0.026267	None
1435	TPD52L1	-0.9511	0.001743	0.026296	Down
1436	ACAP3	-0.30101	0.001749	0.026373	None
1437	DHRS9	-1.25009	0.001751	0.026384	Down
1438	ATP1B1	-0.79357	0.001752	0.026386	Down
1439	PGK1	0.364786	0.001759	0.026468	None
1440	SEPHS2	-0.28056	0.001768	0.026589	None
1441	TIMMDC1	-0.37423	0.001771	0.026589	None
1442	OR2S2	0.057565	0.001771	0.026589	None
1443	DPEP2	-0.7489	0.001773	0.026606	Down
1444	CTRC	0.145886	0.001776	0.026627	None
1445	ABCA7	-0.4567	0.00178	0.026677	None
1446	ZFYVE21	-0.58894	0.001784	0.02672	Down
1447	C8orf37	-0.38973	0.001788	0.026752	None
1448	C20orf195	0.008834	0.001791	0.026763	None

1449	KCNS3	0.351761	0.001791	0.026763	None
1450	RAB10	-0.43357	0.001793	0.026763	None
1451	COCH	-1.25386	0.001795	0.026763	Down
1452	PABPC4L	-1.31905	0.001795	0.026763	Down
1453	NUP98	-0.42671	0.001796	0.026763	None
1454	RASSF7	-0.18595	0.001797	0.026763	None
1455	PCYOX1	-0.58444	0.001804	0.026846	None
1456	ATOH8	0.078028	0.001807	0.02687	None
1457	FCRLB	-0.47198	0.001808	0.02687	None
1458	NOP10	-0.74142	0.001821	0.027025	Down
1459	RPS6KA1	-0.46942	0.001822	0.027025	None
1460	SLC35A3	0.462978	0.001823	0.027025	None
1461	SDSL	-0.54254	0.001823	0.027025	None
1462	UFSP2	-0.47798	0.001825	0.027025	None
1463	PRKAG2	-0.44195	0.001831	0.027097	None
1464	LOC10028	-0.20254	0.001848	0.027334	None
1465	ORC2	-0.37533	0.001853	0.027369	None
1466	TBC1D9B	-0.25356	0.001854	0.027369	None
1467	LOC14700	0.073502	0.001854	0.027369	None
1468	ZNRF2P1	0.11017	0.001857	0.027394	None
1469	ZXDA	0.744511	0.001861	0.02743	Up
1470	ERI1	-0.33632	0.001862	0.02743	None
1471	DXO	-0.39546	0.001867	0.027488	None
1472	PIK3R1	0.531968	0.001871	0.027527	None
1473	RP4-560B	0.169824	0.001878	0.027615	None
1474	MITF	-0.92431	0.001881	0.027638	Down
1475	C15orf27	0.07229	0.001883	0.027647	None
1476	TMEM261	-0.40326	0.001887	0.027682	None
1477	KCNE3	-0.54656	0.001893	0.027754	None
1478	KRTAP3-2	0.096686	0.001903	0.02788	None
1479	A2M-AS1	-0.83104	0.001904	0.027884	Down
1480	FAM95A	0.125863	0.001908	0.027914	None
1481	CHIC1	0.224538	0.001912	0.027957	None
1482	SEMA4G	0.079215	0.001914	0.027973	None
1483	ALDH9A1	-0.3437	0.001916	0.027977	None
1484	ZNF616	-0.49841	0.001924	0.028076	None
1485	TBC1D2	-0.40361	0.001929	0.028123	None
1486	BET1	-0.5847	0.001932	0.028148	None
1487	FAM129C	0.86383	0.001936	0.028175	Up
1488	SEMA4D	-0.34153	0.001936	0.028175	None
1489	REXO4	-0.35436	0.001937	0.028177	None
1490	LOC15391	0.034324	0.00194	0.028181	None
1491	KCNQ3	0.069075	0.001941	0.028181	None
1492	TULP4	-0.29813	0.001942	0.028181	None
1493	LOC10042	0.094838	0.001945	0.028217	None
1494	THNSL1	-0.71968	0.001969	0.028534	Down
1495	LINC0002	0.063393	0.001972	0.028562	None
1496	CHST14	-0.44978	0.001974	0.028564	None
1497	HTRA2	-0.40219	0.001975	0.028564	None
1498	RP1-30M3	-0.51873	0.001976	0.028564	None
1499	GPN3	-0.3725	0.001977	0.028564	None
1500	HEG1	0.480736	0.001982	0.028606	None
1501	UPF1	-0.35457	0.001983	0.028606	None
1502	MRPL18	-0.42116	0.001985	0.028619	None
1503	C16orf62	-0.17504	0.001995	0.028739	None
1504	RP11-382	-0.88798	0.002006	0.028863	Down
1505	PSTPIP2	-0.53349	0.002007	0.028863	None
1506	CDIPT	-0.28122	0.002008	0.028863	None

1507	ARHGAP18	-0.89712	0.002009	0.028863	Down
1508	WIPF2	-0.35838	0.002014	0.028918	None
1509	TMPO	0.739114	0.002016	0.028925	Up
1510	LOC10192	0.02914	0.002023	0.029008	None
1511	BTN3A3	-0.51812	0.002029	0.029073	None
1512	PPHLN1	-0.43272	0.002031	0.029087	None
1513	AGPAT3	-0.64272	0.002032	0.029087	Down
1514	OBFC1	-0.29764	0.002036	0.029116	None
1515	C20orf173	0.038995	0.002041	0.029172	None
1516	WAPAL	-0.35807	0.002075	0.029644	None
1517	EIF2B1	-0.23144	0.002078	0.029665	None
1518	UBE2R2	-0.29635	0.002085	0.02974	None
1519	SAT2	-0.50212	0.002088	0.029761	None
1520	LOC14569	0.079077	0.002093	0.029782	None
1521	MS4A3	1.506083	0.002093	0.029782	Up
1522	APOBEC4	0.056033	0.002093	0.029782	None
1523	TMEM263	0.527409	0.002106	0.029948	None
1524	WASF3	-1.27427	0.002118	0.030092	Down
1525	MAN2B2	-0.48727	0.002125	0.030167	None
1526	RAD51D	-0.30516	0.002127	0.030167	None
1527	C17orf47	0.050443	0.002127	0.030167	None
1528	NYNRIN	-0.75295	0.002131	0.03019	Down
1529	MAMSTR	0.163656	0.002133	0.03019	None
1530	PDZRN3-A	0.168383	0.002133	0.03019	None
1531	ZC4H2	0.360622	0.002135	0.030204	None
1532	SOAT1	0.613343	0.002152	0.030417	Up
1533	C11orf24	-0.28506	0.002159	0.030492	None
1534	DCAKD	0.258044	0.002162	0.030502	None
1535	SLC32A1	0.132869	0.002162	0.030502	None
1536	RP11-216L	-0.34193	0.002169	0.030579	None
1537	COPS7A	-0.50337	0.002172	0.030606	None
1538	SEC24C	-0.39862	0.002176	0.030625	None
1539	SPATA20	-0.71913	0.002178	0.030625	Down
1540	KIAA1671	0.13356	0.002179	0.030625	None
1541	ASCC2	-0.56076	0.002179	0.030625	None
1542	COX11	-0.30666	0.002181	0.030628	None
1543	ATP6V1C1	-0.55382	0.002192	0.030757	None
1544	NUDT17	-0.21165	0.002193	0.030757	None
1545	C2orf88	-1.08679	0.002197	0.030787	Down
1546	TNFAIP8L2	-0.77612	0.002202	0.030844	Down
1547	HPDL	-0.9778	0.002206	0.030877	Down
1548	STIL	0.672842	0.002207	0.030879	Up
1549	LOC91548	-0.23757	0.002213	0.030913	None
1550	CDC42EP2	0.091369	0.002214	0.030913	None
1551	SLC2A11	-0.32567	0.002214	0.030913	None
1552	UHRF1	0.958804	0.002225	0.031042	Up
1553	ZNF668	-0.21118	0.002227	0.031049	None
1554	DIAPH2-A	0.038646	0.002228	0.031049	None
1555	RBM19	-0.32033	0.002235	0.031111	None
1556	EIF4G1	-0.53526	0.002235	0.031111	None
1557	FAM214A	0.504238	0.002238	0.031127	None
1558	HACL1	0.537848	0.00224	0.031127	None
1559	TMEM5	-0.38573	0.002245	0.031183	None
1560	C11orf95	-0.34519	0.002252	0.031267	None
1561	MGC20647	0.095858	0.002258	0.031306	None
1562	RPL22	0.205497	0.002258	0.031306	None
1563	TSPAN17	-0.30428	0.002262	0.031324	None
1564	KIAA1191	-0.34225	0.002262	0.031324	None

1565	KSR2	0.053317	0.002264	0.031325	None
1566	OCRL	-0.17564	0.002266	0.031338	None
1567	MAGEA8	0.073191	0.002268	0.031345	None
1568	LOC33966(	0.215662	0.002275	0.031412	None
1569	FXR2	-0.31474	0.002276	0.031417	None
1570	EFHD2	-0.51196	0.002278	0.031425	None
1571	TSKU	0.243351	0.002284	0.031478	None
1572	THEM6	-0.55699	0.002289	0.031529	None
1573	RSG1	-0.19045	0.002303	0.031703	None
1574	TNFSF10	-0.86939	0.002306	0.031726	Down
1575	CTNNAL1	0.778701	0.002311	0.031777	Up
1576	EIF2S2	-0.39216	0.002315	0.031803	None
1577	C2CD2	-0.56091	0.002317	0.031812	None
1578	ZBTB7B	-0.07825	0.002319	0.031825	None
1579	TOMM40L	-0.47954	0.002323	0.031857	None
1580	TMC6	-0.45302	0.002328	0.031901	None
1581	NUDT3	-0.54999	0.002331	0.031924	None
1582	AARS2	-0.34621	0.002332	0.031924	None
1583	TCTA	-0.41132	0.002341	0.03203	None
1584	IWS1	-0.40121	0.002347	0.032072	None
1585	BBS2	-0.48745	0.002349	0.032072	None
1586	KCNAB2	-0.49443	0.002349	0.032072	None
1587	GRSF1	-0.35728	0.00235	0.032072	None
1588	SLC45A4	-0.63355	0.002356	0.032133	Down
1589	DHRS11	-0.78242	0.002358	0.032141	Down
1590	ZNF347	-0.2738	0.002366	0.032223	None
1591	ZNF284	-0.34396	0.00237	0.032258	None
1592	DKFZP586I	0.475092	0.002372	0.032258	None
1593	CEP104	-0.43856	0.002379	0.032338	None
1594	KRR1	0.360696	0.002392	0.032497	None
1595	DYRK4	-0.52739	0.002404	0.032637	None
1596	ECHDC3	-0.53003	0.002409	0.03268	None
1597	ALOXE3	0.011793	0.002414	0.032729	None
1598	PRAMEF12	0.15676	0.002417	0.032743	None
1599	SYAP1	0.628393	0.002418	0.032743	Up
1600	OR5L2	0.079513	0.002419	0.032743	None
1601	TADA3	-0.32847	0.002421	0.032746	None
1602	FAM64A	0.857094	0.002423	0.032756	Up
1603	NFRKB	-0.33157	0.002426	0.032756	None
1604	DENND1A	-0.52112	0.002426	0.032756	None
1605	STK38	0.49602	0.002431	0.032794	None
1606	SPSB2	-0.50006	0.002432	0.032794	None
1607	CNEP1R1	-0.42579	0.002434	0.0328	None
1608	CHIAP2	0.019757	0.002437	0.032821	None
1609	IPO13	-0.40952	0.002444	0.032897	None
1610	GCDH	-0.60555	0.002451	0.032962	Down
1611	ANGPTL6	-0.56204	0.002456	0.033007	None
1612	FAS	-0.63101	0.002462	0.033076	Down
1613	AASDH	-0.46251	0.002467	0.033114	None
1614	MYO1F	-0.54638	0.002468	0.033114	None
1615	CTNS	-0.61743	0.002481	0.033269	Down
1616	FLJ42627	-0.57779	0.002485	0.033299	None
1617	NSUN5P1	-0.52618	0.002501	0.033499	None
1618	UTP20	-0.56193	0.002504	0.033518	None
1619	BTN3A1	-0.36923	0.002509	0.033563	None
1620	RP11-680F	0.025374	0.002511	0.033565	None
1621	CYTH1	0.241056	0.002515	0.033604	None
1622	MYPOP	-0.42853	0.002518	0.033612	None

1623	CSGALNAC	1.241598	0.002533	0.033793	Up
1624	ELL3	0.457431	0.002547	0.033957	None
1625	FUBP3	0.339449	0.002559	0.034106	None
1626	HIGD2A	-0.34929	0.002561	0.034107	None
1627	EFNB3	0.113294	0.002563	0.034107	None
1628	LOC14937	0.265013	0.002569	0.034148	None
1629	AC009502.	0.019967	0.002569	0.034148	None
1630	E2F5	0.519327	0.002573	0.034181	None
1631	PCYOX1L	-0.61894	0.002574	0.034181	Down
1632	OR5V1	0.027297	0.002579	0.034209	None
1633	CPQ	-0.65442	0.00258	0.034209	Down
1634	TNFSF15	0.244311	0.002588	0.03428	None
1635	EID3	0.688293	0.00259	0.03428	Up
1636	NEURL4	-0.51149	0.002591	0.03428	None
1637	CD9	0.143283	0.002591	0.03428	None
1638	PPP1R16A	-0.35116	0.002593	0.03428	None
1639	LINC00691	0.143526	0.002604	0.034403	None
1640	OSBPL5	-0.47086	0.002615	0.034529	None
1641	LOC28576	0.026881	0.002627	0.034666	None
1642	CAD	-0.30023	0.002629	0.03467	None
1643	HR	0.073654	0.00263	0.03467	None
1644	PTK2B	-0.38838	0.002633	0.034678	None
1645	TMEM126E	-0.21159	0.002641	0.034762	None
1646	CD151	-0.48294	0.002646	0.034801	None
1647	EPHA8	0.105081	0.002647	0.034801	None
1648	ARID1A	-0.77556	0.002651	0.034828	Down
1649	RWDD2B	-0.60774	0.002654	0.034855	Down
1650	CDC42EP4	-0.45938	0.00266	0.034913	None
1651	CA14	0.210954	0.002672	0.035035	None
1652	CHRNB4	0.027446	0.002675	0.035035	None
1653	CREG1	-0.44389	0.002676	0.035035	None
1654	PTDSS2	-0.38509	0.002676	0.035035	None
1655	ERMAP	-0.64393	0.002679	0.035049	Down
1656	FASN	-0.31868	0.002684	0.035081	None
1657	LOC73142	0.414779	0.002685	0.035081	None
1658	TRADD	-0.36595	0.002686	0.035081	None
1659	METTL21C	0.045561	0.002688	0.035085	None
1660	FTL	-0.5848	0.002695	0.035149	None
1661	GYS1	-0.54342	0.002697	0.035149	None
1662	F12	-0.36549	0.002698	0.035149	None
1663	RP11-73K9	0.251902	0.002699	0.035149	None
1664	PPFIBP2	-0.93638	0.002709	0.035257	Down
1665	CIDEB	-0.57936	0.002713	0.035291	None
1666	CORO1C	-0.46903	0.002722	0.035387	None
1667	8-Sep	-1.00989	0.002727	0.035425	Down
1668	NPIPA1	-0.61318	0.002732	0.035464	Down
1669	ACOT4	-0.73842	0.002742	0.035583	Down
1670	HK1	-0.51039	0.00275	0.035665	None
1671	SETDB1	-0.37512	0.002754	0.035692	None
1672	WDR92	-0.52493	0.002764	0.035766	None
1673	ZBED6	-0.4609	0.002764	0.035766	None
1674	LOC10192	0.008837	0.002765	0.035766	None
1675	POLL	-0.40589	0.002778	0.035917	None
1676	TNFSF13	-0.56479	0.002784	0.035971	None
1677	LINC00382	0.018529	0.002789	0.036019	None
1678	CSTF2T	0.41732	0.002803	0.036171	None
1679	SHCBP1	0.904827	0.002809	0.036224	Up
1680	UBE2G2	-0.35885	0.002821	0.036367	None

1681	AIF1	-0.56701	0.002825	0.036391	None
1682	PRB3	0.345282	0.002832	0.036457	None
1683	NOL8	-0.42581	0.00284	0.036526	None
1684	EXOC6B	-0.31709	0.002841	0.036526	None
1685	COL22A1	0.014363	0.002842	0.036526	None
1686	KLHL12	-0.35415	0.002848	0.036575	None
1687	MAPKAPK!	-0.43252	0.002849	0.036575	None
1688	CSTB	-0.49258	0.002857	0.036626	None
1689	AP1G2	-0.26367	0.002858	0.036626	None
1690	MLF2	-0.43722	0.002859	0.036626	None
1691	CMC4	-0.37521	0.00286	0.036626	None
1692	FZD7	-0.80627	0.00287	0.036686	Down
1693	CRTC3	-0.37512	0.00287	0.036686	None
1694	MAGEB2	0.037307	0.00287	0.036686	None
1695	HSBP1	-0.24982	0.00288	0.036775	None
1696	LOC10012!	0.187548	0.00288	0.036775	None
1697	CSPG5	0.093163	0.002882	0.036775	None
1698	STARD4	0.764393	0.002886	0.036806	Up
1699	RB1	0.376582	0.002889	0.036828	None
1700	HCRTR1	0.015228	0.002898	0.03691	None
1701	EIF4EBP2	-0.35517	0.002902	0.036939	None
1702	MFSD10	-0.69504	0.00292	0.037151	Down
1703	ZNF514	-0.81911	0.002922	0.037156	Down
1704	COA5	-0.27015	0.002928	0.037209	None
1705	INPP5E	-0.42642	0.002945	0.037409	None
1706	ZMAT4	0.09393	0.002949	0.037436	None
1707	NABP2	-0.47572	0.002952	0.037443	None
1708	CLDN19	0.143039	0.002956	0.037472	None
1709	MTNR1A	0.010943	0.00296	0.037502	None
1710	LOC10013:	0.162469	0.002963	0.037503	None
1711	RP11-649/	0.273268	0.002963	0.037503	None
1712	MRPL49	-0.39578	0.002968	0.037536	None
1713	F8	-0.69348	0.002972	0.037567	Down
1714	LOC10050'	-0.36341	0.002976	0.037575	None
1715	C11orf1	-0.54458	0.002977	0.037575	None
1716	ZFP82	-0.37813	0.002978	0.037575	None
1717	GABBR2	0.024702	0.00298	0.037589	None
1718	LOC10013(	0.211959	0.002983	0.037604	None
1719	LOC10192!	0.019188	0.002988	0.037641	None
1720	DUSP3	-0.56933	0.003	0.037773	None
1721	LOC10192(	0.136702	0.003004	0.037775	None
1722	SEC16B	0.010209	0.003004	0.037775	None
1723	CTPS2	-0.30034	0.003008	0.037801	None
1724	CD79B	0.28554	0.003027	0.038021	None
1725	WDSUB1	-0.34334	0.003038	0.03814	None
1726	ECHS1	-0.34074	0.003044	0.038194	None
1727	SLITRK5	-1.09931	0.003046	0.038199	Down
1728	HDAC11	-0.23027	0.003048	0.038199	None
1729	NCAPG2	0.683096	0.003056	0.038218	Up
1730	MAD2L2	0.588716	0.003057	0.038218	Up
1731	SDF4	-0.33385	0.003058	0.038218	None
1732	OGFOD1	-0.43156	0.003059	0.038218	None
1733	TIMM10B	-0.2967	0.003059	0.038218	None
1734	CCAR1	0.377718	0.00306	0.038218	None
1735	ZBTB3	-0.39385	0.003063	0.038228	None
1736	ADRM1	-0.83901	0.003067	0.038263	Down
1737	HMG20A	-0.33435	0.00307	0.038277	None
1738	TRPV2	-0.2677	0.00308	0.038376	None

1739	BBS1	0.072355	0.003083	0.038388	None
1740	LOC28424(	0.015196	0.003087	0.038413	None
1741	MCEE	-0.36205	0.003099	0.038541	None
1742	ASTE1	-0.40537	0.0031	0.038541	None
1743	RPF2	-0.42376	0.003104	0.038567	None
1744	MEIS3P1	-0.91613	0.003115	0.038673	Down
1745	RP11-550I:	0.016891	0.00312	0.038689	None
1746	PREX1	-0.49822	0.00312	0.038689	None
1747	NAV2	0.00706	0.003121	0.038689	None
1748	PCDH9	1.140053	0.003126	0.038697	Up
1749	TXNDC8	0.029003	0.003128	0.038697	None
1750	PHF23	-0.37652	0.003128	0.038697	None
1751	HDHD3	-0.60292	0.003129	0.038697	Down
1752	PPP3R1	-0.77258	0.003145	0.038877	Down
1753	SEMA6A	0.231852	0.003149	0.038889	None
1754	CYB5R1	-0.3461	0.00315	0.038889	None
1755	POU4F3	0.015956	0.003155	0.03893	None
1756	PLGRKT	-0.66066	0.003164	0.039016	Down
1757	NBEAL2	-0.26177	0.003169	0.039042	None
1758	KCTD13	-0.23897	0.003172	0.039042	None
1759	LINC00893	0.62001	0.003173	0.039042	Up
1760	MX2	-0.95833	0.003173	0.039042	Down
1761	TMEM260	-0.39507	0.003177	0.039069	None
1762	INE1	0.276057	0.00318	0.039078	None
1763	RHOT1	-0.25867	0.003186	0.039129	None
1764	ERCC4	-0.31625	0.003188	0.03913	None
1765	RRN3P2	-0.91631	0.003191	0.039155	Down
1766	MPG	-0.47754	0.003204	0.039283	None
1767	MAN2A2	-0.39852	0.003223	0.039493	None
1768	DHX9	0.723183	0.003228	0.039516	Up
1769	GKN2	0.061213	0.003229	0.039516	None
1770	VASH2	0.573968	0.003231	0.039516	None
1771	STK40	-0.31442	0.003232	0.039516	None
1772	EVC2	-0.38706	0.003243	0.039603	None
1773	ADCK1	-0.47649	0.003244	0.039603	None
1774	GORASP2	-0.3097	0.003246	0.039603	None
1775	HRASLS	-0.99624	0.003248	0.039603	Down
1776	BARD1	0.539137	0.003248	0.039603	None
1777	TCF12	0.397959	0.00325	0.03961	None
1778	RP1-6P5.2	0.219173	0.003256	0.039655	None
1779	LRRC71	0.123696	0.003276	0.039873	None
1780	ESCO2	0.349054	0.003277	0.039873	None
1781	PDCD6	0.76492	0.003281	0.039894	Up
1782	LASP1	-0.33226	0.003292	0.040009	None
1783	SOCS1	0.106097	0.003296	0.040033	None
1784	ZNF618	-0.6041	0.003299	0.040051	Down
1785	TRAF7	-0.26676	0.003308	0.040116	None
1786	SPATA2	-0.36271	0.00331	0.040116	None
1787	PPWD1	0.384435	0.00331	0.040116	None
1788	CLN3	-0.45738	0.003316	0.040159	None
1789	TPRXL	0.069089	0.003323	0.040225	None
1790	POU4F1	0.646664	0.003329	0.040278	Up
1791	TMED8	-0.49804	0.003331	0.040279	None
1792	NUDT16	-0.2558	0.003342	0.040367	None
1793	OCA2	0.263103	0.003342	0.040367	None
1794	SLC5A5	0.140873	0.003346	0.040387	None
1795	POLI	-0.53074	0.003353	0.040456	None
1796	SLC7A6OS	-0.27997	0.003365	0.040576	None

1797	KCNJ1	0.024416	0.003367	0.040576	None
1798	TAS2R1	0.099082	0.003369	0.040576	None
1799	PEX26	0.184566	0.003377	0.040654	None
1800	SMIM3	0.715673	0.003386	0.040735	Up
1801	KATNBL1	-0.56348	0.003393	0.040796	None
1802	LOC10192!	0.011469	0.003404	0.040908	None
1803	MBD1	-0.29004	0.003406	0.04091	None
1804	NCK1-AS1	-0.72841	0.003415	0.040993	Down
1805	ERO1LB	0.585125	0.003427	0.041104	Up
1806	MAST4	-0.62746	0.00343	0.041104	Down
1807	HIF1A	0.447655	0.00343	0.041104	None
1808	KLF7	0.573888	0.003432	0.041104	None
1809	COX1	-0.1183	0.003434	0.041113	None
1810	GPD1L	-0.58801	0.003452	0.041306	Down
1811	NDUFA3	-0.55767	0.003454	0.041306	None
1812	C8orf66	0.037379	0.003461	0.041367	None
1813	RP11-66N:	0.048079	0.003479	0.041553	None
1814	SNX8	0.12649	0.003487	0.041624	None
1815	PIGW	0.58713	0.003495	0.041692	Up
1816	ANGPTL1	0.01297	0.003498	0.041692	None
1817	RP11-73M	-0.38118	0.003498	0.041692	None
1818	ARHGEF10	0.169075	0.003515	0.041865	None
1819	GOLM1	-0.75506	0.003521	0.041898	Down
1820	CWC27	-0.36808	0.003521	0.041898	None
1821	LINC00588	0.054332	0.003525	0.041924	None
1822	MINA	-0.40289	0.003537	0.042033	None
1823	MEDAG	0.110549	0.003538	0.042033	None
1824	RNF185	-0.41436	0.003548	0.042105	None
1825	LINC01186	0.044008	0.00355	0.042105	None
1826	TDG	0.390044	0.00355	0.042105	None
1827	LYN	-0.44631	0.003563	0.042228	None
1828	AK026905	0.226423	0.003572	0.042274	None
1829	CXCR1	0.194401	0.003572	0.042274	None
1830	C19orf66	-0.56643	0.003572	0.042274	None
1831	NAV2-AS4	0.170104	0.003576	0.042296	None
1832	SUPT4H1	-0.41232	0.003584	0.04236	None
1833	PNPLA6	-0.53807	0.003587	0.04236	None
1834	RILPL2	0.444467	0.003588	0.04236	None
1835	APTR	-0.2473	0.003592	0.04236	None
1836	NNT-AS1	-0.73583	0.003595	0.04236	Down
1837	AOX2P	0.197096	0.003595	0.04236	None
1838	ZBTB18	0.288712	0.003595	0.04236	None
1839	ZDHHC5	-0.2399	0.003603	0.042431	None
1840	AKR1C6P	0.123677	0.003608	0.042463	None
1841	LGI3	0.050308	0.003624	0.042622	None
1842	LOC10013(	0.416038	0.003627	0.042645	None
1843	GTSF1L	0.146507	0.003632	0.042679	None
1844	ZNF558	-0.70374	0.003639	0.04271	Down
1845	VTI1B	-0.41387	0.00364	0.04271	None
1846	APOPT1	-0.23536	0.003643	0.04271	None
1847	CPSF7	-0.26186	0.003643	0.04271	None
1848	ZNF512B	-0.43602	0.003648	0.042729	None
1849	HIATL1	0.268659	0.003648	0.042729	None
1850	AARS	-0.45676	0.003658	0.042805	None
1851	DNAJA3	-0.45702	0.00366	0.042805	None
1852	UQCRC2	0.344574	0.003662	0.042805	None
1853	RGSL1	0.096161	0.003665	0.042805	None
1854	CRY1	0.466861	0.003666	0.042805	None

1855	SPNS2	-0.617	0.003667	0.042805	Down
1856	BRAP	-0.26153	0.003671	0.042805	None
1857	GFOD1	0.626669	0.003671	0.042805	Up
1858	LOC10192!	0.213448	0.003673	0.042805	None
1859	ZNF607	-0.38471	0.003677	0.04283	None
1860	METTL21B	-0.59606	0.00368	0.042837	Down
1861	RRAGD	-0.50545	0.003681	0.042837	None
1862	FGF16	0.036922	0.003688	0.042888	None
1863	MRS2	-0.42277	0.003695	0.042936	None
1864	RTP4	-0.8023	0.003696	0.042936	Down
1865	PHF21A	-0.26168	0.0037	0.042942	None
1866	ERAP1	0.923653	0.0037	0.042942	Up
1867	SDAD1	-0.53622	0.003717	0.043114	None
1868	NOD1	-0.28131	0.003726	0.04312	None
1869	C5orf56	0.636129	0.003726	0.04312	Up
1870	MMACHC	-0.57109	0.003728	0.04312	None
1871	FERMT3	-0.47138	0.003728	0.04312	None
1872	IFRD2	-0.44469	0.003728	0.04312	None
1873	MBOAT1	-0.54806	0.003736	0.0432	None
1874	HRK	0.411843	0.003739	0.043201	None
1875	MZF1	-0.46338	0.003742	0.043201	None
1876	MARVELD1	-0.21451	0.003744	0.043201	None
1877	SREK1IP1	-0.33666	0.003746	0.043201	None
1878	IRF3	-0.42681	0.003747	0.043201	None
1879	GLG1	-0.29719	0.003749	0.043201	None
1880	LINC00881	0.104124	0.003751	0.043201	None
1881	MARK3	-0.30131	0.003756	0.043215	None
1882	RNF8	-0.19979	0.003756	0.043215	None
1883	IFFO1	-0.45887	0.003764	0.043283	None
1884	AGO3	-0.55396	0.003768	0.043305	None
1885	HMX2	0.093242	0.003787	0.043511	None
1886	DNAH10	0.046585	0.003793	0.043543	None
1887	LOC10192	0.091675	0.003794	0.043543	None
1888	LOC10099	0.779343	0.003798	0.043559	Up
1889	ITGB8	0.097878	0.003802	0.043565	None
1890	KIAA1522	-0.52831	0.003802	0.043565	None
1891	POP1	-0.58141	0.003809	0.043605	None
1892	AKAP1	-0.4299	0.00381	0.043605	None
1893	LZIC	-0.31095	0.003813	0.043619	None
1894	NICN1	-0.37982	0.003815	0.043623	None
1895	NT5DC1	-0.35765	0.003823	0.043687	None
1896	LOC10099	0.085168	0.003828	0.043717	None
1897	SCML1	0.454611	0.003833	0.043752	None
1898	C4orf45	0.015886	0.003835	0.043759	None
1899	VPS52	-0.27097	0.003839	0.043778	None
1900	LENG8	-0.86802	0.003845	0.043823	Down
1901	SMEK2	0.484583	0.00385	0.043859	None
1902	OSTM1	-0.57422	0.003867	0.044014	None
1903	ORAI1	-0.56359	0.003868	0.044014	None
1904	LOC10192	0.010284	0.003872	0.044043	None
1905	ATP6AP1	-0.4645	0.003875	0.044046	None
1906	SRXN1	-0.77834	0.003879	0.044075	Down
1907	GGA1	-0.38053	0.003887	0.044134	None
1908	EIF3J-AS1	-0.37951	0.003895	0.044192	None
1909	COMMD6	-0.36222	0.003896	0.044192	None
1910	MESDC2	-0.39736	0.003902	0.044244	None
1911	ATXN7L3	-0.32826	0.003907	0.044276	None
1912	LINC00572	0.029101	0.003911	0.044299	None

1913	IL7	-0.93082	0.003915	0.044313	Down
1914	RAB29	-0.58347	0.003917	0.044313	None
1915	SELE	0.55255	0.003924	0.04436	None
1916	HTRA4	0.425144	0.003925	0.04436	None
1917	SLCO6A1	0.016568	0.00394	0.044509	None
1918	TNS4	0.114969	0.003951	0.044606	None
1919	ARHGEF3	-0.56952	0.003953	0.044606	None
1920	SLC1A4	0.495467	0.003961	0.044669	None
1921	IL27RA	-0.59571	0.003973	0.044758	Down
1922	IL25	0.040031	0.003974	0.044758	None
1923	COPS7B	-0.40696	0.003975	0.044758	None
1924	CAND2	-0.37659	0.003996	0.044971	None
1925	GCLM	-0.62472	0.003998	0.044976	Down
1926	AK8	0.128434	0.004005	0.045032	None
1927	ZNF324B	0.109405	0.004008	0.045038	None
1928	CCDC178	0.076433	0.004019	0.045119	None
1929	GCLC	-0.47562	0.004021	0.045119	None
1930	PSAT1	-0.79909	0.004023	0.045119	Down
1931	SPATA5L1	-0.34894	0.004024	0.045119	None
1932	SLC9A3R1	-0.41025	0.004025	0.045119	None
1933	KCNA5	0.448997	0.004029	0.045128	None
1934	DPH3	-0.37837	0.00403	0.045128	None
1935	SYT9	0.006966	0.004039	0.045197	None
1936	SLC16A5	-0.48661	0.004041	0.045197	None
1937	ZNF709	0.250359	0.004043	0.045203	None
1938	LOC10050	0.486308	0.004051	0.045261	None
1939	HDHD2	-0.31366	0.004061	0.045349	None
1940	TMEM198	-0.44531	0.004063	0.045349	None
1941	PREP	-0.33541	0.004065	0.045349	None
1942	ZNF318	-0.48444	0.004078	0.045476	None
1943	RCAN1	0.203812	0.004086	0.04554	None
1944	LCE2B	0.077667	0.004095	0.045611	None
1945	GMPS	-0.46982	0.004098	0.045611	None
1946	STIM2	0.354285	0.004099	0.045611	None
1947	PPP2R5D	-0.22303	0.004115	0.045766	None
1948	IZUMO4	0.093383	0.004118	0.045777	None
1949	TAF1D	0.583753	0.004124	0.045817	None
1950	HDGF	-0.37068	0.004134	0.045881	None
1951	ZNF593	-0.68117	0.004134	0.045881	Down
1952	RP11-349	0.35415	0.004138	0.045902	None
1953	C7orf31	-0.71909	0.004142	0.045924	Down
1954	CNPPD1	-0.24768	0.004149	0.045982	None
1955	CSNK1G1	-0.31613	0.004167	0.046153	None
1956	RNF216	-0.33143	0.004176	0.046228	None
1957	ZNF23	-0.43283	0.004184	0.046286	None
1958	HSPA2	-0.7495	0.004185	0.046286	Down
1959	PAPPA2	0.100056	0.004191	0.046317	None
1960	HEATR5B	-0.38886	0.004193	0.046317	None
1961	SNX6	-0.46676	0.004194	0.046317	None
1962	ZNF682	0.368265	0.004201	0.046356	None
1963	SLC17A4	0.069577	0.004204	0.046356	None
1964	LMAN1	0.324688	0.004207	0.046356	None
1965	PSMG3-A	-0.40535	0.004208	0.046356	None
1966	RNF13	-0.3868	0.004209	0.046356	None
1967	LOC10192	0.01194	0.004212	0.046371	None
1968	FLJ33534	0.132022	0.004225	0.046493	None
1969	RMDN3	-0.37414	0.004235	0.046578	None
1970	ZNF501	0.289316	0.004258	0.046802	None

1971	MYBL1	0.437403	0.00426	0.046807	None
1972	MT2A	-1.16321	0.00427	0.046891	Down
1973	EGR3	1.482616	0.004274	0.046912	Up
1974	OXNAD1	-0.50648	0.004278	0.046921	None
1975	C16orf46	-0.20461	0.004279	0.046921	None
1976	TMEM140	-0.99739	0.004292	0.047038	Down
1977	SLC45A2	0.025541	0.004301	0.047075	None
1978	LMNB1	0.782478	0.004301	0.047075	Up
1979	CCL27	0.072121	0.004303	0.047075	None
1980	NFS1	-0.25063	0.004305	0.047075	None
1981	NTPCR	-0.37717	0.004312	0.047075	None
1982	TMEM161/	-0.37027	0.004313	0.047075	None
1983	TLE4	0.84271	0.004313	0.047075	Up
1984	DDX55	-0.4892	0.004315	0.047075	None
1985	DLK1	-1.34455	0.004315	0.047075	Down
1986	ADIG	0.150678	0.004319	0.047097	None
1987	ZSCAN21	-0.49255	0.004333	0.047221	None
1988	FHAD1	0.009293	0.004338	0.047258	None
1989	KIAA0232	0.386509	0.004348	0.047332	None
1990	PRAM1	-0.90578	0.00435	0.047332	Down
1991	CYB561D1	-0.22335	0.004357	0.047392	None
1992	BTN3A2	-0.59608	0.004365	0.047457	Down
1993	LOC34034(	0.006014	0.00437	0.047479	None
1994	BAX	-0.5975	0.004377	0.047534	Down
1995	SYNE3	0.11399	0.00439	0.047647	None
1996	LRP3	-0.234	0.004404	0.047777	None
1997	RP11-646E	0.085262	0.004428	0.048013	None
1998	ACOX1	-0.41455	0.00443	0.048013	None
1999	ESM1	0.369893	0.004433	0.048021	None
2000	PSME3	-0.57942	0.004437	0.048043	None
2001	SLIRP	-0.50578	0.004441	0.048056	None
2002	VANGL1	-0.60101	0.004446	0.048067	Down
2003	DDX18	-0.24904	0.004446	0.048067	None
2004	ABLIM2	0.103503	0.004457	0.048158	None
2005	EXOSC2	-0.74301	0.004464	0.048209	Down
2006	FBXL15	-0.35944	0.004466	0.048209	None
2007	MALT1	0.576538	0.004487	0.048404	None
2008	WBP1L	-0.37799	0.004488	0.048404	None
2009	RNF126	-0.36415	0.004513	0.048642	None
2010	GPAM	-0.44951	0.004518	0.048676	None
2011	MAD1L1	-0.66488	0.004523	0.048709	Down
2012	GLDC	0.16941	0.00453	0.04873	None
2013	FAM210B	-0.54417	0.00453	0.04873	None
2014	IFI27	-1.67879	0.004535	0.048757	Down
2015	HMGB1	0.272176	0.004545	0.048827	None
2016	NDUFV2	-0.63465	0.004546	0.048827	Down
2017	CCDC113	-0.79336	0.004553	0.04886	Down
2018	SETDB2	-0.38105	0.004553	0.04886	None
2019	RP11-521E	0.106693	0.004575	0.049055	None
2020	NDUFC1	-0.2927	0.004578	0.049055	None
2021	TCERG1	0.924341	0.004578	0.049055	Up
2022	LOC15165	0.013747	0.004582	0.049075	None
2023	FBLL1	0.172579	0.004585	0.049075	None
2024	PREB	-0.48739	0.004587	0.049075	None
2025	LINC0100C	-0.54845	0.004597	0.049154	None
2026	ROCK2	-0.22542	0.004614	0.049314	None
2027	IFI44	-0.81034	0.004626	0.049422	Down
2028	EHBP1	-0.29761	0.004629	0.049429	None

2029	HOXC6	0.191995	0.004633	0.049438	None
2030	DPY30	-0.38198	0.004636	0.049438	None
2031	SEC62	-0.3094	0.004637	0.049438	None
2032	NACC2	-0.41829	0.004639	0.049438	None
2033	OVOL2	0.043424	0.004642	0.049447	None
2034	KCNH5	0.015567	0.004656	0.049567	None
2035	LOC10192	0.012137	0.004658	0.049569	None
2036	SLC7A3	0.323042	0.004672	0.049675	None
2037	ALDH3A2	-0.5229	0.004675	0.049675	None
2038	GIPC1	-0.29158	0.004675	0.049675	None
2039	PEX11B	-0.37487	0.004678	0.049685	None
2040	ARFIP1	-0.38723	0.004692	0.049803	None
2041	HELLS	0.590758	0.00471	0.049941	Up
2042	MAP2K5	-0.45732	0.004712	0.049941	None
2043	C3orf18	-0.40202	0.004713	0.049941	None
2044	TRAPPC13	0.690232	0.004714	0.049941	Up
2045	AL022344.	0.075391	0.004731	0.050093	None
2046	PCED1A	-0.3024	0.004736	0.050122	None
2047	STARD8	-0.54045	0.004744	0.050186	None
2048	THAP11	-0.31106	0.004746	0.050186	None
2049	RP4-575N	0.247249	0.004754	0.050245	None
2050	FBXO10	0.053347	0.004782	0.050489	None
2051	TNC	0.077793	0.004782	0.050489	None
2052	ABI1	0.3604	0.004797	0.050623	None
2053	RP11-757F	0.089	0.004806	0.050682	None
2054	CTD-2002	0.094206	0.004807	0.050682	None
2055	PARVB	-0.60319	0.004819	0.050739	Down
2056	TUBA1A	0.399002	0.004819	0.050739	None
2057	CCL2	1.056083	0.00482	0.050739	Up
2058	LARS2	-0.31755	0.00484	0.050917	None
2059	TMEM214	-0.39232	0.004841	0.050917	None
2060	SLC35D1	0.531166	0.004853	0.05099	None
2061	PSEN2	-0.53651	0.004853	0.05099	None
2062	TRIM41	-0.62487	0.004861	0.051048	Down
2063	RAD1	-0.41243	0.004868	0.051085	None
2064	CHD8	-0.46287	0.004869	0.051085	None
2065	ARRDC2	0.598809	0.004877	0.05114	Up
2066	PRRC2B	-0.50048	0.004882	0.051169	None
2067	C12orf43	-0.39235	0.00489	0.051235	None
2068	PXMP4	-0.40353	0.004896	0.051268	None
2069	SNORD89	0.512081	0.004912	0.051414	None
2070	MFSD5	-0.50116	0.004915	0.051414	None
2071	KRI1	-0.39458	0.004928	0.051527	None
2072	PWRN2	0.134723	0.00493	0.051527	None
2073	KCNK13	0.00849	0.004937	0.051571	None
2074	RHOU	-0.77208	0.004946	0.051642	Down
2075	ZFPM1	-0.31712	0.004954	0.051682	None
2076	LINC01410	-0.8168	0.004955	0.051682	Down
2077	PRG2	2.064621	0.004961	0.051723	Up
2078	CLIC2	-0.88179	0.004967	0.051761	Down
2079	LINC00674	0.242314	0.004979	0.051841	None
2080	COX10	-0.3315	0.004979	0.051841	None
2081	ELANE	1.833643	0.005011	0.052141	Up
2082	LOC10192	0.137386	0.005022	0.052194	None
2083	LOC28402	-0.47761	0.005022	0.052194	None
2084	APBA2	-0.68099	0.005023	0.052194	Down
2085	LOC34017	0.043972	0.00503	0.052246	None
2086	ZNF343	-0.27799	0.005057	0.052493	None

2087	RP11-1137	0.068183	0.005059	0.052497	None
2088	MSMB	0.043178	0.005071	0.05258	None
2089	PRR7-AS1	0.033316	0.005072	0.05258	None
2090	DKK4	0.047035	0.005082	0.052659	None
2091	LEPROT	-0.40946	0.00509	0.052718	None
2092	LRRTM4	0.015033	0.005099	0.052785	None
2093	FAM193A	-0.31292	0.005106	0.052827	None
2094	CNBD2	0.015185	0.005111	0.05286	None
2095	RNF111	-0.34602	0.005119	0.052915	None
2096	PRODH2	0.107274	0.005125	0.052944	None
2097	HSPBAP1	-0.33657	0.005127	0.052944	None
2098	ASCL2	-0.39702	0.005143	0.053082	None
2099	GDE1	-0.50917	0.005147	0.053103	None
2100	MFSD8	-0.6697	0.005158	0.053193	Down
2101	USP7	0.21112	0.005162	0.053207	None
2102	ZNF441	0.209525	0.005166	0.053222	None
2103	LHFPL3-AS	0.008407	0.005177	0.053306	None
2104	TESPA1	-0.61519	0.00519	0.053414	Down
2105	PHKA1	-0.43792	0.005205	0.053515	None
2106	STK32B	0.691417	0.005207	0.053515	Up
2107	LINC00567	0.081464	0.005207	0.053515	None
2108	SNAPC4	-0.34115	0.005225	0.053675	None
2109	PPP3CA	-0.26482	0.005235	0.053734	None
2110	G3BP1	0.473312	0.005236	0.053734	None
2111	CCDC109B	0.959037	0.005242	0.053773	Up
2112	SIAH1	0.348093	0.005246	0.053792	None
2113	ACP6	-0.53177	0.005258	0.053857	None
2114	LOC10192	0.010783	0.005259	0.053857	None
2115	ZSWIM8	-0.28337	0.005261	0.053857	None
2116	ARSI	0.049079	0.005263	0.053857	None
2117	ETFA	-0.37504	0.005269	0.053885	None
2118	DPP10-AS	-0.82786	0.00527	0.053885	Down
2119	RNF103	0.471946	0.005275	0.053907	None
2120	ALKBH6	-0.37326	0.005283	0.053959	None
2121	GTPBP10	0.009727	0.005286	0.053964	None
2122	AC004692.	0.403098	0.005293	0.054014	None
2123	KCNS1	0.173311	0.0053	0.054037	None
2124	RP1-181J2	0.080754	0.0053	0.054037	None
2125	PDHA2	0.039694	0.005309	0.054074	None
2126	NPHS2	0.047675	0.005309	0.054074	None
2127	TSTA3	-0.58304	0.005322	0.054184	None
2128	ZNF202	-0.36508	0.005325	0.054192	None
2129	MMP28	0.492575	0.005328	0.054192	None
2130	LOC10192	0.177347	0.005346	0.054354	None
2131	CLU	-0.89566	0.005364	0.054513	Down
2132	ZKSCAN1	-0.58191	0.005371	0.054554	None
2133	BBS12	-0.66354	0.005375	0.054567	Down
2134	TNFRSF13	0.052756	0.005382	0.054617	None
2135	KIR2DS3	0.077176	0.005388	0.054648	None
2136	SLC2A10	-0.89893	0.005407	0.054816	Down
2137	RP11-579	0.00801	0.005416	0.054881	None
2138	C12orf45	-0.53749	0.005419	0.054882	None
2139	FBXW5	-0.41805	0.005424	0.054916	None
2140	HLA-DQB1	-2.59747	0.005427	0.05492	Down
2141	TMEM230	-0.383	0.005431	0.05493	None
2142	STAM2	-0.36467	0.005439	0.054984	None
2143	GLCE	-0.59302	0.005467	0.055248	Down
2144	GJB2	0.390696	0.005478	0.05531	None

2145	GMIP	-0.21696	0.005479	0.05531	None
2146	NDUFB2-A	-0.16791	0.005482	0.055321	None
2147	FAM212A	-0.39159	0.005489	0.055336	None
2148	C10orf55	0.147551	0.005489	0.055336	None
2149	DEDD2	-0.37355	0.005497	0.055391	None
2150	P4HTM	-0.40989	0.005505	0.055446	None
2151	LINC01359	0.046377	0.00551	0.055475	None
2152	GAS2L1	-0.82598	0.005514	0.055489	Down
2153	DPF2	-0.3555	0.005522	0.055517	None
2154	DGKH	0.270106	0.005522	0.055517	None
2155	NAA40	-0.40303	0.005534	0.055597	None
2156	TRPC3	0.085248	0.005537	0.055597	None
2157	NCOR1	-0.3364	0.005538	0.055597	None
2158	BTNL3	0.319288	0.005553	0.055725	None
2159	SMARCD2	0.412888	0.005556	0.055725	None
2160	CCDC15	-0.66809	0.005559	0.055732	Down
2161	SPATS2	-0.33546	0.005568	0.055795	None
2162	LACTB	-0.71736	0.0056	0.056092	Down
2163	EEF1A1P42	0.150101	0.005603	0.056092	None
2164	PRSS58	0.085154	0.005606	0.056098	None
2165	LARP1	-0.81521	0.005624	0.05623	Down
2166	RP11-3L8.3	0.063036	0.005624	0.05623	None
2167	SLC34A3	0.036556	0.005635	0.056285	None
2168	RP11-471M	0.011366	0.005635	0.056285	None
2169	TOR1AIP2	0.17682	0.005674	0.056645	None
2170	ALDH16A1	-0.45855	0.005685	0.056711	None
2171	ZNF671	-0.48561	0.005688	0.056711	None
2172	ZBTB17	-0.2203	0.005688	0.056711	None
2173	DPAGT1	-0.48738	0.005692	0.056721	None
2174	SLC39A4	-0.70475	0.005696	0.05674	Down
2175	VASP	-0.64756	0.005704	0.056786	Down
2176	RP9P	-0.28888	0.005744	0.057159	None
2177	ISG20L2	-0.32119	0.005746	0.057159	None
2178	AKR1B10	0.07883	0.005757	0.057171	None
2179	ACACA	-0.31481	0.005758	0.057171	None
2180	SRPRB	-0.47537	0.005758	0.057171	None
2181	ZNF114	-0.45419	0.00576	0.057171	None
2182	WDR41	-0.35867	0.005761	0.057171	None
2183	RP5-944M	0.010345	0.005777	0.05728	None
2184	STXBP2	-0.25242	0.005777	0.05728	None
2185	ZFP90	-0.39028	0.00578	0.05728	None
2186	C1orf216	-0.54683	0.005784	0.057295	None
2187	DESI1	-0.40527	0.005801	0.057436	None
2188	EXTL3-AS1	0.267281	0.005809	0.057494	None
2189	BTAF1	0.381893	0.005815	0.057525	None
2190	NUP62	-0.64128	0.005821	0.057558	Down
2191	SLC17A6	0.047651	0.005824	0.057565	None
2192	RP11-421E	0.269977	0.005854	0.05783	None
2193	SLC30A3	0.118796	0.005867	0.057939	None
2194	RARA-AS1	0.465633	0.005877	0.058008	None
2195	VPS37C	-0.45244	0.005892	0.058132	None
2196	PPP3CC	0.223682	0.0059	0.058155	None
2197	ERVH48-1	0.091858	0.0059	0.058155	None
2198	DBT	-0.35753	0.005914	0.058261	None
2199	MED19	-0.25337	0.005939	0.058483	None
2200	LAT2	0.301255	0.005943	0.0585	None
2201	PEX6	-0.72945	0.005956	0.058595	Down
2202	ADAMTS13	0.067541	0.005972	0.058734	None

2203	COQ5	-0.35108	0.005994	0.058919	None
2204	CPA2	0.139057	0.006	0.05895	None
2205	FSCB	0.01103	0.006003	0.058958	None
2206	NCEH1	-0.88727	0.006012	0.059019	Down
2207	ABL1	-0.45323	0.006037	0.059236	None
2208	ZNF500	-0.52142	0.006056	0.059395	None
2209	ZNF436	-0.31072	0.006066	0.05944	None
2210	MAPKAP1	-0.3884	0.006066	0.05944	None
2211	RNF125	0.581103	0.006081	0.059562	None
2212	LOC44146:	0.263182	0.006087	0.059588	None
2213	CD274	-0.56378	0.006112	0.059797	None
2214	SOX17	0.031536	0.006114	0.059797	None
2215	ARHGEF1	0.394851	0.006123	0.059862	None
2216	WDR12	-0.50313	0.006127	0.059877	None
2217	RHOT2	-0.35018	0.006131	0.059884	None
2218	ARL4C	1.058679	0.006142	0.059968	Up
2219	RP5-1119/	0.201107	0.006161	0.060127	None
2220	EIF5	-0.38331	0.006169	0.060175	None
2221	KRT10	0.250825	0.006193	0.060342	None
2222	WDR35	0.386215	0.006194	0.060342	None
2223	NECAP2	-0.33518	0.006194	0.060342	None
2224	ST3GAL4-/	-0.60065	0.00621	0.06047	Down
2225	FLJ21369	0.077377	0.006215	0.060477	None
2226	LOC10192	0.294256	0.006217	0.060477	None
2227	IFITM10	0.042876	0.006234	0.06062	None
2228	RPL32P3	-0.38785	0.006258	0.060827	None
2229	LOC10192	0.144425	0.006263	0.06084	None
2230	CLDN16	0.058823	0.006265	0.06084	None
2231	C21orf33	-0.4679	0.006294	0.061096	None
2232	ERMN	0.702068	0.006297	0.061096	Up
2233	PLD3	-0.96018	0.006312	0.061213	Down
2234	PCIF1	0.571713	0.006321	0.061273	None
2235	LAMB2P1	-0.38349	0.00633	0.061316	None
2236	DCTD	-0.32269	0.006331	0.061316	None
2237	SYNPO	0.195824	0.006337	0.061337	None
2238	SMG5	-0.28898	0.006339	0.061337	None
2239	SEC31B	-0.66922	0.006355	0.061461	Down
2240	ANXA2R	0.517423	0.006368	0.061538	None
2241	PTTG1IP	-0.32471	0.006368	0.061538	None
2242	TCAIM	-0.57777	0.006393	0.061751	None
2243	TRAF3IP3	-0.59014	0.006397	0.061761	Down
2244	PRKCSH	-0.39925	0.006403	0.061771	None
2245	METTL5	-0.22479	0.006404	0.061771	None
2246	STRA6	0.016307	0.006411	0.061815	None
2247	TSPAN5	-0.68094	0.006419	0.061858	Down
2248	LOC64869:	0.020723	0.006429	0.061928	None
2249	MADD	-0.37143	0.006438	0.061987	None
2250	LINC00115	-0.46654	0.006446	0.062026	None
2251	SGTA	-0.3036	0.006448	0.062026	None
2252	FSIP1	0.074044	0.006451	0.062031	None
2253	NSAP11	0.049535	0.006471	0.06217	None
2254	AC073283.	0.644422	0.006471	0.06217	Up
2255	INF2	-0.189	0.006474	0.062175	None
2256	PKN3	-0.49401	0.006484	0.062237	None
2257	YWHAE	-0.43319	0.006487	0.062237	None
2258	TMEM170E	0.792217	0.006492	0.062256	Up
2259	KDM4B	-0.26584	0.006497	0.062279	None
2260	B4GALNT1	0.018636	0.006552	0.062785	None

2261	ADCY7	-0.26143	0.006559	0.062816	None
2262	LOC10013(	0.03595	0.006564	0.062844	None
2263	GBP3	-0.78777	0.006569	0.062863	Down
2264	IGLL1	1.198529	0.006583	0.062967	Up
2265	ULBP1	0.048682	0.006589	0.062982	None
2266	NSUN5	-0.39751	0.006591	0.062982	None
2267	USP51	-0.27705	0.006596	0.063005	None
2268	MAP4K2	-0.61895	0.006602	0.063035	Down
2269	ITFG3	-0.377	0.006614	0.063113	None
2270	FAM124B	-0.7047	0.00662	0.063113	Down
2271	LOC10013:	0.080922	0.006621	0.063113	None
2272	HEATR5A	-0.76688	0.006622	0.063113	Down
2273	EIF4EBP1	-0.71124	0.006626	0.063123	Down
2274	ABHD5	-0.57594	0.006634	0.063174	None
2275	ARX	0.060129	0.006646	0.063259	None
2276	RASL11A	0.009396	0.006664	0.06339	None
2277	RP11-554D	0.126428	0.006665	0.06339	None
2278	LTB4R	-0.59229	0.006678	0.063456	Down
2279	NAGLU	-0.27525	0.006681	0.063456	None
2280	NOC4L	-0.52843	0.006681	0.063456	None
2281	DGKZ	-0.28571	0.006697	0.06358	None
2282	HSPH1	-0.71739	0.006701	0.063593	Down
2283	APH1B	-0.30999	0.006705	0.063598	None
2284	NUDT22	-0.21075	0.00672	0.063699	None
2285	RP11-506(	0.299544	0.006721	0.063699	None
2286	SMIM15	-0.29972	0.006766	0.064085	None
2287	MAL	-1.19676	0.006768	0.064085	Down
2288	ATAT1	-0.24651	0.006772	0.064099	None
2289	HSD3B7	-0.65636	0.006783	0.064168	Down
2290	SH2B1	-0.42773	0.006806	0.064362	None
2291	FAHD1	-0.36964	0.006819	0.064435	None
2292	TRIM56	-0.57185	0.006822	0.064435	None
2293	EIF3F	0.589548	0.006825	0.064435	Up
2294	SUGP1	-0.29926	0.006826	0.064435	None
2295	INPP5D	0.606328	0.006839	0.064533	Up
2296	GPR35	0.325682	0.006843	0.064539	None
2297	IRF5	-0.73081	0.006878	0.064838	Down
2298	ZNF658	-0.57337	0.006882	0.064851	None
2299	CDH24	0.043556	0.006887	0.064869	None
2300	C1orf122	-0.34169	0.006913	0.065083	None
2301	ADAD2	0.239211	0.00694	0.065313	None
2302	RP3-486D:	0.297142	0.006946	0.065331	None
2303	MT1E	-1.1404	0.006948	0.065331	Down
2304	PODXL2	-0.07543	0.006952	0.065345	None
2305	DNM1	-0.69718	0.006959	0.065369	Down
2306	SYNPR-AS	0.006982	0.006961	0.065369	None
2307	TSHZ1	-0.38051	0.006968	0.065378	None
2308	HIST1H2BE	-0.73965	0.006968	0.065378	Down
2309	SKP2	0.539639	0.006972	0.065386	None
2310	SMARCD3	-0.39	0.007006	0.065673	None
2311	BRWD1-IT	0.199768	0.007014	0.065722	None
2312	ADORA2B	-0.73167	0.007017	0.065722	Down
2313	ICAM4	-0.78606	0.00703	0.065813	Down
2314	1-Dec	0.01103	0.007038	0.065862	None
2315	ZNF19	0.079368	0.007046	0.065907	None
2316	CD1D	-0.76864	0.00706	0.06601	Down
2317	LINC00955	0.011674	0.007065	0.066035	None
2318	SENP8	-0.33645	0.00707	0.066047	None

2319	STAU2-AS	0.008351	0.007084	0.066151	None
2320	FAM96B	-0.31569	0.007095	0.066202	None
2321	KAZN	-0.32695	0.007096	0.066202	None
2322	RP11-68I3.	0.288412	0.0071	0.066218	None
2323	GPX2	0.070496	0.007112	0.066302	None
2324	ZNF691	-0.41675	0.007116	0.066304	None
2325	L2HGDH	-0.28454	0.00712	0.066316	None
2326	SMARCD1	-0.16101	0.007153	0.066598	None
2327	IDI1	0.337075	0.007157	0.066607	None
2328	COPS5	-0.28045	0.007174	0.066733	None
2329	LIPT1	-0.36619	0.007201	0.066956	None
2330	SECISBP2L	0.454498	0.007219	0.067079	None
2331	FRAT2	-0.42981	0.007221	0.067079	None
2332	ENO2	-0.70324	0.007229	0.067106	Down
2333	ACTR3B	-0.2403	0.00723	0.067106	None
2334	CPA5	0.078802	0.007239	0.06716	None
2335	CNST	-0.48376	0.007242	0.067161	None
2336	CCDC42	-1.30624	0.007245	0.067162	Down
2337	SCO1	-0.44608	0.007255	0.067228	None
2338	NDUFB10	-0.45688	0.007262	0.067267	None
2339	XIRP1	0.238657	0.007267	0.067282	None
2340	MDM4	0.377691	0.007276	0.067333	None
2341	PI4KB	-0.65789	0.00728	0.067341	Down
2342	AX747191	0.064029	0.007284	0.067348	None
2343	SIM2	0.035445	0.007289	0.067358	None
2344	STARD9	0.733566	0.007294	0.067358	Up
2345	GSDMD	-0.34073	0.007296	0.067358	None
2346	NAA11	0.0195	0.007298	0.067358	None
2347	ADAM5	0.035335	0.0073	0.067358	None
2348	CCDC185	0.095122	0.007305	0.067375	None
2349	EMR1	-1.06762	0.007314	0.067424	Down
2350	LRRK1	-0.33965	0.007321	0.067463	None
2351	PINK1	-0.41567	0.007336	0.067573	None
2352	SIK1	0.547161	0.007339	0.067573	None
2353	SNCG	0.043715	0.007348	0.067579	None
2354	TANGO2	-0.28571	0.00735	0.067579	None
2355	EP400	-0.39654	0.007351	0.067579	None
2356	NSUN4	-0.27517	0.007352	0.067579	None
2357	RAD54B	-0.67111	0.007359	0.067589	Down
2358	SMARCB1	-0.28523	0.00736	0.067589	None
2359	B3GNT4	0.113221	0.007366	0.067614	None
2360	KBTBD2	0.547348	0.007371	0.067614	None
2361	GALNTL5	0.006254	0.007372	0.067614	None
2362	ADK	0.428769	0.007378	0.067641	None
2363	CS	-0.41465	0.007383	0.06765	None
2364	UCP2	-0.60557	0.007386	0.06765	Down
2365	JMJD1C	0.378145	0.007388	0.06765	None
2366	XPO4	-0.3706	0.007404	0.067769	None
2367	SLC29A1	-0.46112	0.007412	0.067786	None
2368	LOC64285:	-0.37896	0.007412	0.067786	None
2369	BC039673	0.008666	0.007433	0.067926	None
2370	LOC10050(	0.115942	0.007434	0.067926	None
2371	LOC10192(	0.027793	0.007457	0.068103	None
2372	SLC4A7	0.318328	0.007465	0.068126	None
2373	RP11-29H:	-0.47353	0.007465	0.068126	None
2374	PRKDC	-1.0734	0.007479	0.068222	Down
2375	AP5S1	-0.33889	0.007502	0.0684	None
2376	CNOT3	-0.45324	0.007527	0.068586	None

2377	ZNF581	-0.59125	0.007529	0.068586	Down
2378	PIM2	-0.44971	0.007536	0.068623	None
2379	CASP10	-0.10508	0.007539	0.068623	None
2380	PDCD6IP	-0.24515	0.007547	0.068672	None
2381	ZNF30	-0.55441	0.007562	0.068777	None
2382	BMP2K	-0.60027	0.007583	0.068934	Down
2383	PSMD5	-0.15416	0.00759	0.068969	None
2384	TLR6	-0.23774	0.007593	0.068969	None
2385	RBM12	0.260106	0.007618	0.069167	None
2386	FKBP4	-0.37586	0.007633	0.069272	None
2387	MED15	-0.40742	0.007646	0.069366	None
2388	EFNA3	-0.16624	0.007655	0.069419	None
2389	MT1F	-0.97563	0.007661	0.069435	Down
2390	RELA	-0.35745	0.007666	0.069435	None
2391	PRKCZ	-0.77366	0.007669	0.069435	Down
2392	TWIST2	0.064016	0.00767	0.069435	None
2393	PDPN	0.012409	0.007678	0.069484	None
2394	TIMP1	-0.78676	0.007685	0.069517	Down
2395	HAAO	0.159685	0.007694	0.069564	None
2396	ALMS1P	0.157482	0.007697	0.069565	None
2397	FADS3	0.531567	0.007721	0.069752	None
2398	LSP1	-0.59854	0.007734	0.069837	Down
2399	RAD51AP1	0.644705	0.007758	0.070021	Up
2400	APOL3	0.53159	0.00776	0.070021	None
2401	ZSWIM3	-0.38244	0.007765	0.070031	None
2402	ABHD2	-0.3229	0.00778	0.070139	None
2403	PPP2R5E	-0.29731	0.007785	0.070142	None
2404	TSPAN3	0.141349	0.007794	0.070142	None
2405	CIDECP	0.118829	0.007795	0.070142	None
2406	ENTPD6	-0.19435	0.007796	0.070142	None
2407	PPP1R9B	-0.24398	0.007798	0.070142	None
2408	TEC	-0.60797	0.0078	0.070142	Down
2409	CAHM	-0.45158	0.00781	0.070189	None
2410	BUB3	0.303524	0.007818	0.070189	None
2411	NR1D2	0.526499	0.00782	0.070189	None
2412	FFAR1	0.11396	0.00782	0.070189	None
2413	PPP1R3D	-0.25302	0.007821	0.070189	None
2414	FLJ31713	0.043591	0.00783	0.070222	None
2415	FBXW4	-0.36071	0.007832	0.070222	None
2416	LINC00961	0.217278	0.007835	0.070222	None
2417	LOC64626[illegible]	0.057416	0.007843	0.07027	None
2418	CHD1	0.471534	0.007858	0.070372	None
2419	COL4A5	-0.91353	0.007861	0.070376	Down
2420	C10orf67	0.051314	0.007872	0.070443	None
2421	LOC10272[illegible]	0.019245	0.007897	0.070632	None
2422	TRAPPC3	-0.34014	0.007904	0.070666	None
2423	DPP9	-0.34338	0.007937	0.070912	None
2424	ZNRD1	0.077246	0.00794	0.070912	None
2425	MCOLN3	0.012992	0.007941	0.070912	None
2426	TDRD7	-0.6416	0.007948	0.070939	Down
2427	NABP1	-0.79102	0.007951	0.070939	Down
2428	TBPL1	-0.39767	0.00796	0.070995	None
2429	ARMC6	-0.37333	0.007984	0.071178	None
2430	NRXN2	-0.69638	0.00799	0.071201	Down
2431	ZNF468	0.468831	0.007994	0.071207	None
2432	CLDN20	0.025425	0.008003	0.071258	None
2433	IL5RA	0.099938	0.008007	0.071265	None
2434	SLC38A7	-0.35274	0.008026	0.071405	None

2435	ELP3	-0.32734	0.008041	0.071508	None
2436	VAMP8	-0.33882	0.00805	0.071545	None
2437	RFESD	-1.01283	0.008052	0.071545	Down
2438	ZFAT	-0.10926	0.008066	0.071641	None
2439	C10orf76	-0.31866	0.00808	0.071699	None
2440	ZBTB22	-0.26928	0.008083	0.071699	None
2441	GOSR2	-0.33463	0.008084	0.071699	None
2442	POP5	-0.42745	0.008085	0.071699	None
2443	CARHSP1	0.217817	0.008105	0.071828	None
2444	TFDP2	-0.33149	0.008107	0.071828	None
2445	DENND1C	-0.45148	0.00811	0.071828	None
2446	RNF10	-0.42727	0.008121	0.071897	None
2447	CHL1	0.33943	0.008142	0.072051	None
2448	HEATR2	-0.42684	0.008146	0.07206	None
2449	LOC10050(	-0.36402	0.008168	0.072219	None
2450	LOC73010:	-0.30298	0.008171	0.072219	None
2451	GSK3B	-0.44511	0.008198	0.07243	None
2452	ACSF2	-0.41013	0.008205	0.072459	None
2453	PZP	0.058723	0.008209	0.072464	None
2454	ARTN	0.127434	0.008214	0.072464	None
2455	HNF1B	0.128939	0.008215	0.072464	None
2456	RMDN1	-0.31323	0.008222	0.07248	None
2457	MAP3K12	-0.27693	0.008224	0.07248	None
2458	OLAH	0.004997	0.008227	0.07248	None
2459	RP11-96K1	0.327469	0.008238	0.072549	None
2460	OXCT2	0.184498	0.00825	0.072622	None
2461	FAM111B	0.455677	0.008255	0.072639	None
2462	ZMIZ2	-0.14124	0.00826	0.072649	None
2463	ZNF330	-0.19879	0.00827	0.072715	None
2464	MRPL10	-0.3213	0.008281	0.072756	None
2465	STEAP3	-0.62846	0.008284	0.072756	Down
2466	TOPORS-A	-0.34701	0.008285	0.072756	None
2467	PTCD2	-0.39893	0.008291	0.072777	None
2468	TMCO4	-0.23596	0.008312	0.072932	None
2469	SNUPN	-0.30396	0.008338	0.07313	None
2470	LRFN4	-0.18908	0.008342	0.073137	None
2471	FBXL6	-0.18511	0.008356	0.073214	None
2472	C1orf52	-0.27223	0.008358	0.073214	None
2473	FAM102A	-0.21347	0.008391	0.073476	None
2474	GPR52	0.226743	0.008417	0.073671	None
2475	DISP1	-0.17954	0.008433	0.073783	None
2476	LOC10192	0.118423	0.008447	0.073878	None
2477	CAPRIN2	-0.38722	0.008498	0.074291	None
2478	NAT14	-0.41363	0.008505	0.074322	None
2479	SLC30A5	0.288782	0.008513	0.074322	None
2480	TSPAN9	0.110831	0.008514	0.074322	None
2481	MRPS18B	-0.42085	0.008515	0.074322	None
2482	NUP88	0.280796	0.008531	0.07443	None
2483	FHOD1	-0.50542	0.008544	0.074515	None
2484	ZKSCAN8	-0.21648	0.008564	0.07466	None
2485	ORAI3	-0.43097	0.008574	0.074718	None
2486	ZMYM2	0.494725	0.008601	0.07492	None
2487	KCNJ3	0.015464	0.008619	0.075047	None
2488	TOB2	-0.53209	0.008626	0.075067	None
2489	MEG9	0.018721	0.008628	0.075067	None
2490	SMIM19	-0.38588	0.008632	0.075073	None
2491	MYOCD	0.00981	0.008648	0.075177	None
2492	FGFR3	-0.31126	0.008651	0.075177	None

2493	NUCB1	-0.45804	0.008661	0.075229	None
2494	CCDC170	0.096818	0.008665	0.075234	None
2495	LOC101928	0.088521	0.008669	0.075241	None
2496	CDK17	0.359142	0.008682	0.075327	None
2497	DACT3-AS	0.029509	0.008705	0.075494	None
2498	SFN	0.372927	0.008714	0.075537	None
2499	ZHX1	-0.30526	0.008723	0.075584	None
2500	GARS	-0.3776	0.008726	0.075584	None
2501	FBXO25	-0.51223	0.008741	0.075687	None
2502	LOC101927	0.102941	0.008752	0.075753	None
2503	TIPRL	-0.28455	0.008769	0.075855	None
2504	RP11-109C	0.181397	0.008771	0.075855	None
2505	RP11-498C	0.293648	0.008806	0.076128	None
2506	ANKRD1	0.021955	0.008819	0.07621	None
2507	OR7E24	0.027487	0.008823	0.07621	None
2508	TWF2	-0.31737	0.008857	0.076438	None
2509	HIST1H1D	0.926909	0.00886	0.076438	Up
2510	LINC00644	0.007954	0.008862	0.076438	None
2511	SGCD	0.0057	0.008863	0.076438	None
2512	SH3BGRL3	-0.83883	0.008869	0.076454	Down
2513	MOSPD2	-0.41003	0.008887	0.076583	None
2514	PRKAG2-A	-0.55789	0.008913	0.07677	None
2515	NRBF2	-0.40137	0.008925	0.076818	None
2516	ACSL5	-0.29899	0.008925	0.076818	None
2517	ATP6V0E1	-0.95378	0.008933	0.076858	Down
2518	DTX3L	-0.37426	0.009001	0.077411	None
2519	WDR47	0.362268	0.009018	0.077521	None
2520	TRIM27	-0.39418	0.009036	0.077649	None
2521	RBM14	-0.65101	0.009058	0.077785	Down
2522	CHMP6	-0.48375	0.009059	0.077785	None
2523	AZGP1P1	0.006287	0.009083	0.077961	None
2524	FLVCR1-AS	-0.68057	0.009101	0.07808	Down
2525	DRC1	0.115624	0.009107	0.078092	None
2526	UNC13A	0.073646	0.009109	0.078092	None
2527	IFI44L	-1.47132	0.009116	0.078122	Down
2528	EPDR1	-0.60523	0.009131	0.078191	Down
2529	RP3-428L1	0.458159	0.009132	0.078191	None
2530	ZNF526	-0.35243	0.009173	0.078517	None
2531	AFF3	0.084227	0.00918	0.078545	None
2532	LINC01339	0.200014	0.00919	0.078594	None
2533	C12orf49	0.391296	0.009207	0.078715	None
2534	SH3RF2	0.006437	0.009214	0.078744	None
2535	FAM76A	0.238524	0.009238	0.078917	None
2536	FAM162A	-0.35367	0.009255	0.079028	None
2537	TULP3	-0.28519	0.009274	0.07913	None
2538	ID3	0.91321	0.009275	0.07913	Up
2539	MLLT1	-0.14773	0.009278	0.07913	None
2540	MBD6	-0.6615	0.009285	0.079155	Down
2541	AIM2	-0.87952	0.009288	0.079155	Down
2542	PRDM15	-0.32687	0.009303	0.07925	None
2543	MIS12	0.298618	0.00931	0.079279	None
2544	UACA	-0.28155	0.009322	0.079318	None
2545	FAM58A	0.246485	0.009322	0.079318	None
2546	TTC4	-0.27347	0.009326	0.079318	None
2547	CCNG1	-0.28354	0.009339	0.079403	None
2548	WDR17	0.465515	0.009351	0.07946	None
2549	PFAS	-0.45701	0.009353	0.07946	None
2550	SLC6A17	0.078729	0.009362	0.079504	None

2551	BRAT1	-0.37431	0.009376	0.079588	None
2552	LOC10028	0.039966	0.009386	0.079643	None
2553	MAPK14	-0.47914	0.00939	0.079647	None
2554	TMEM151E	0.066838	0.009397	0.07966	None
2555	DDX46	0.320394	0.009399	0.07966	None
2556	METTL22	-0.24629	0.009411	0.079709	None
2557	RPARP-AS	-0.33129	0.009412	0.079709	None
2558	ZNF133	-0.42724	0.009416	0.07971	None
2559	MZB1	0.978613	0.009437	0.079808	Up
2560	RAB39A	0.307523	0.009439	0.079808	None
2561	SH3BP2	-0.52871	0.009441	0.079808	None
2562	IMMT	-0.26649	0.009442	0.079808	None
2563	ELFN1	0.12043	0.009457	0.079897	None
2564	SPATA19	0.043741	0.009462	0.079897	None
2565	MRPS35	-0.26445	0.009464	0.079897	None
2566	LACTB2	-0.56662	0.009472	0.079937	None
2567	BC022047	0.421586	0.009487	0.079968	None
2568	HINFP	-0.43785	0.009488	0.079968	None
2569	HLF	1.077738	0.009491	0.079968	Up
2570	YTHDF1	-0.38137	0.009491	0.079968	None
2571	SUMO3	-0.19891	0.009496	0.079986	None
2572	TMEM60	-0.39489	0.009504	0.079999	None
2573	IFT46	-0.32341	0.009509	0.079999	None
2574	ARL5C	0.0314	0.00951	0.079999	None
2575	RPAP1	-0.33803	0.009513	0.079999	None
2576	GRM6	0.117429	0.009519	0.079999	None
2577	GATC	-0.26445	0.00952	0.079999	None
2578	ADPRHL1	0.038998	0.009533	0.080079	None
2579	METTL14	-0.30115	0.009554	0.080183	None
2580	LOC10099	0.054978	0.009556	0.080183	None
2581	AUTS2	0.45902	0.009557	0.080183	None
2582	RCL1	-0.48119	0.009574	0.080273	None
2583	TBL3	-0.24999	0.009579	0.080273	None
2584	LOC10192	0.084002	0.009585	0.080273	None
2585	PEPD	-0.5015	0.009586	0.080273	None
2586	UNC13B	-0.57444	0.009586	0.080273	None
2587	CDC34	-0.28346	0.009606	0.080409	None
2588	LRRC14	-0.19048	0.009624	0.080526	None
2589	SSH1	-0.2579	0.009641	0.080618	None
2590	ZG16B	-0.56323	0.009642	0.080618	None
2591	PDZD7	0.070965	0.00965	0.080651	None
2592	FPGT	-0.50762	0.009662	0.080688	None
2593	KRTAP5-8	0.196637	0.009664	0.080688	None
2594	ZNF780A	-0.24841	0.009665	0.080688	None
2595	2-Mar	-0.7738	0.009697	0.080922	Down
2596	RPL41	0.055351	0.009711	0.081002	None
2597	OSTF1	-0.59661	0.009716	0.081014	Down
2598	LOC10192	0.010814	0.009731	0.081106	None
2599	GNAS	0.301671	0.00975	0.081196	None
2600	VWCE	-0.47931	0.00975	0.081196	None
2601	SLC35C1	-0.21835	0.009754	0.081196	None
2602	ASB12	0.478571	0.009756	0.081196	None
2603	CPEB4	-0.78375	0.009793	0.081468	Down
2604	GINM1	-0.35896	0.0098	0.081479	None
2605	PPAPDC2	-0.71476	0.009802	0.081479	Down
2606	FMO2	0.389561	0.009808	0.0815	None
2607	TGFB3	0.06234	0.009812	0.081503	None
2608	CCDC126	-0.36932	0.009816	0.081503	None

2609	PDE4D	0.324091	0.009841	0.081684	None
2610	CYP3A5	0.068786	0.009856	0.081775	None
2611	PCNX	0.432669	0.009891	0.082023	None
2612	ZNF562	-0.13313	0.009893	0.082023	None
2613	CABP2	0.054277	0.009898	0.08203	None
2614	LOC10192!	0.01107	0.009904	0.082048	None
2615	ORC4	-0.31406	0.009925	0.082191	None
2616	ABHD6	-0.28769	0.009936	0.082246	None
2617	LINC01137	-0.55792	0.009944	0.082285	None
2618	ACVR1	-0.51511	0.009971	0.082475	None
2619	CC2D2B	0.132347	0.009983	0.082517	None
2620	LOC10192!	0.039988	0.009984	0.082517	None
2621	FIBIN	0.015602	0.01	0.082615	None
2622	PTPRE	0.679274	0.010003	0.082615	Up
2623	TGFB1	-0.54321	0.01002	0.082722	None
2624	XPC	-0.29992	0.010025	0.08273	None
2625	GBP5	-0.71887	0.01004	0.082822	Down
2626	GDPD5	-0.24488	0.01005	0.082877	None
2627	ARNTL2	-0.45185	0.010057	0.082901	None
2628	EIF4A3	0.346726	0.010075	0.08302	None
2629	L3MBTL1	-0.05473	0.010085	0.08305	None
2630	BAG6	-0.40142	0.010089	0.08305	None
2631	TEX26	0.029748	0.010092	0.08305	None
2632	ZNF582-A!	-0.35222	0.010094	0.08305	None
2633	CHST13	-0.33787	0.010101	0.083071	None
2634	PARP3	-0.39795	0.010107	0.083092	None
2635	KIAA0141	-0.41385	0.010133	0.083274	None
2636	SH3GLB1	-0.34208	0.010137	0.083275	None
2637	KCNB2	0.027891	0.010143	0.083278	None
2638	ELAC2	-0.18378	0.010145	0.083278	None
2639	DLX4	0.042962	0.010168	0.083432	None
2640	ARL2BP	-0.42598	0.010175	0.083463	None
2641	HRASLS5	0.061603	0.010184	0.083503	None
2642	PLEKHA3	0.389829	0.010213	0.083705	None
2643	ZFYVE19	-0.23562	0.010216	0.083705	None
2644	GATA1	-0.64242	0.010224	0.083738	Down
2645	MYBBP1A	-0.27567	0.010238	0.083783	None
2646	PSG5	0.077699	0.010238	0.083783	None
2647	KIFC1	0.516516	0.010241	0.083783	None
2648	TSHZ2	0.010423	0.010253	0.083815	None
2649	GPD2	-0.41454	0.010256	0.083815	None
2650	FNTA	-0.38741	0.010259	0.083815	None
2651	PHRF1	-0.1783	0.010261	0.083815	None
2652	PSEN1	0.320381	0.010268	0.083846	None
2653	NUP37	-0.32517	0.010275	0.083848	None
2654	DKFZp564I	0.038848	0.010276	0.083848	None
2655	AFAP1L1	-0.38451	0.01031	0.084089	None
2656	CTSD	-0.76567	0.010324	0.084135	Down
2657	MAFG	-0.4372	0.010325	0.084135	None
2658	NFATC2IP	-0.2524	0.010327	0.084135	None
2659	PLEKHO2	-0.62088	0.010353	0.084318	Down
2660	PHOX2A	0.141249	0.01038	0.084503	None
2661	ZNF818P	-0.46538	0.010398	0.084616	None
2662	LOC15506(	-0.38505	0.010416	0.084733	None
2663	PRIMPOL	-0.3837	0.010424	0.084733	None
2664	HSPB1	-0.82501	0.010425	0.084733	Down
2665	ARFGEF2	0.206198	0.010428	0.084733	None
2666	AKT3	0.469026	0.010436	0.084768	None

2667	HOOK1	-0.86413	0.010448	0.084832	Down
2668	CLPP	-0.40315	0.010481	0.085069	None
2669	TRPC2	-0.37513	0.010491	0.085114	None
2670	ZNF706	-0.24425	0.010494	0.085114	None
2671	ADAP1	-0.32411	0.010506	0.085173	None
2672	PPP1CB	0.373937	0.010514	0.085207	None
2673	CHSY1	0.304747	0.01053	0.085271	None
2674	PIK3C2A	-0.50746	0.010533	0.085271	None
2675	FRRS1L	0.005958	0.010533	0.085271	None
2676	ZNF212	-0.29205	0.010598	0.085765	None
2677	FCRL4	0.005712	0.010616	0.085874	None
2678	DDR1-AS1	0.08735	0.010636	0.086003	None
2679	DHX30	0.286468	0.010666	0.086215	None
2680	C14orf169	-0.38268	0.010672	0.086229	None
2681	TMEM86B	-0.27235	0.010686	0.08631	None
2682	DDX10	-0.17723	0.010759	0.08685	None
2683	C8orf33	-0.29757	0.01076	0.08685	None
2684	TARBP2	-0.36161	0.010772	0.08691	None
2685	CAST	-0.58827	0.010791	0.087033	Down
2686	LOC72819(	0.033749	0.010821	0.087243	None
2687	DHX37	-0.29485	0.010832	0.087284	None
2688	KMT2B	-0.29404	0.010842	0.087284	None
2689	CIAO1	-0.39903	0.010844	0.087284	None
2690	LCN8	0.042513	0.010845	0.087284	None
2691	OST4	-0.53186	0.010846	0.087284	None
2692	TXNIP	-0.71589	0.01086	0.087357	Down
2693	NPDC1	-0.70109	0.010905	0.087692	Down
2694	FAM104B	-0.15858	0.010924	0.087806	None
2695	BRD3	-0.28646	0.010928	0.087812	None
2696	MAST2	-0.18122	0.010957	0.088006	None
2697	MED4	0.330009	0.010973	0.088106	None
2698	TAAR3	0.01683	0.011002	0.088221	None
2699	VGLL4	-0.26784	0.011007	0.088221	None
2700	DCAF6	-0.32756	0.011009	0.088221	None
2701	LIMA1	-0.44539	0.011009	0.088221	None
2702	NISCH	-0.23766	0.011012	0.088221	None
2703	C14orf178	0.041116	0.011012	0.088221	None
2704	CHRNG	0.152252	0.011046	0.088463	None
2705	CHRM4	0.024355	0.01106	0.088542	None
2706	CEBPZOS	-0.25483	0.011073	0.088587	None
2707	UCHL5	0.227611	0.011074	0.088587	None
2708	RPP40	-0.37989	0.011115	0.088885	None
2709	SMR3A	0.051879	0.011144	0.089056	None
2710	RP11-384L	0.142933	0.011145	0.089056	None
2711	BCAS3	-0.40778	0.011155	0.089103	None
2712	FBXW4P1	0.592081	0.011161	0.089116	Up
2713	RPS19BP1	-0.379	0.011185	0.089282	None
2714	AGGF1	-0.3252	0.011196	0.089333	None
2715	LINC01304	0.038136	0.011209	0.089401	None
2716	AMBN	0.020127	0.011244	0.089648	None
2717	SEMA4C	-0.38431	0.011254	0.089684	None
2718	WDR96	0.004712	0.011257	0.089684	None
2719	NRL	0.073422	0.011297	0.089974	None
2720	CPPED1	-0.54684	0.01132	0.090125	None
2721	XXYLT1	-0.56481	0.011338	0.090232	None
2722	RFXANK	-0.34113	0.01139	0.090614	None
2723	TMEM91	-0.50335	0.0114	0.090632	None
2724	ZNF189	-0.33095	0.011404	0.090632	None

2725	RMND5B	-0.23089	0.011405	0.090632	None
2726	TXN	-0.34247	0.011415	0.090655	None
2727	B3GNT8	-0.38111	0.011416	0.090655	None
2728	PRPF8	-0.33525	0.011426	0.0907	None
2729	ZFAND3	-0.32225	0.011434	0.090729	None
2730	LOC10013(	0.117816	0.011458	0.090884	None
2731	C1orf198	-0.57738	0.011472	0.090905	None
2732	LOC10192(	0.087239	0.011475	0.090905	None
2733	XPNPEP2	0.186207	0.011475	0.090905	None
2734	KIAA0513	-0.37469	0.011477	0.090905	None
2735	SLC25A35	-0.14467	0.011482	0.090911	None
2736	GSR	-0.44106	0.0115	0.091008	None
2737	XAF1	-0.66152	0.011509	0.091008	Down
2738	MT1HL1	-1.03657	0.011509	0.091008	Down
2739	PIP	0.05773	0.011511	0.091008	None
2740	ATP7B	-0.90743	0.011531	0.09104	Down
2741	RP11-395E	-0.42849	0.011531	0.09104	None
2742	DCAF5	-0.31467	0.011532	0.09104	None
2743	SPCS1	-0.31107	0.011536	0.09104	None
2744	IKZF4	-0.30088	0.011536	0.09104	None
2745	INO80	0.318643	0.011549	0.091107	None
2746	SRSF3	0.339845	0.011558	0.091143	None
2747	IL17B	0.027681	0.011624	0.091634	None
2748	ORC5	-0.53554	0.011642	0.09174	None
2749	ATMIN	-0.23214	0.011653	0.091799	None
2750	RAB6B	-0.58604	0.011689	0.092049	Down
2751	SI	0.011392	0.01173	0.092319	None
2752	CXorf40A	0.269211	0.011732	0.092319	None
2753	HEIH	-0.25862	0.011738	0.092332	None
2754	MPST	-0.52096	0.011762	0.092483	None
2755	C2orf43	-0.42839	0.01177	0.092485	None
2756	SLC44A1	0.396787	0.01177	0.092485	None
2757	MRPL15	-0.33505	0.01183	0.092912	None
2758	ALG3	-0.39654	0.011835	0.092912	None
2759	BLOC1S6	-0.44397	0.011838	0.092912	None
2760	SLC25A12	-0.29937	0.011853	0.092996	None
2761	CDCA7L	0.356165	0.011859	0.092996	None
2762	KDELR1	-0.42848	0.011861	0.092996	None
2763	CCNJL	0.047712	0.011867	0.093007	None
2764	GAL3ST4	0.201356	0.011876	0.093043	None
2765	SLC25A20	-0.38494	0.011885	0.093059	None
2766	RNMT	0.35233	0.011887	0.093059	None
2767	B4GALNT4	0.054279	0.011928	0.093352	None
2768	RAB27B	-0.79484	0.011956	0.093535	Down
2769	LOC10192(	0.020816	0.011967	0.093586	None
2770	GRAP	0.160399	0.012014	0.093925	None
2771	SPARCL1	0.955462	0.012027	0.093965	Up
2772	SMPDL3A	-0.68461	0.012028	0.093965	Down
2773	MFSD12	-0.27443	0.012036	0.093967	None
2774	SLC25A19	-0.36231	0.012037	0.093967	None
2775	ZNF137P	-0.53901	0.012051	0.094043	None
2776	GANC	-0.22783	0.012064	0.094107	None
2777	TMEM19	-0.27033	0.012076	0.094166	None
2778	MTMR2	-0.34209	0.01209	0.094246	None
2779	ZNF511	-0.35992	0.012096	0.094257	None
2780	LRP5L	0.526944	0.012105	0.09429	None
2781	TMEM72-/	0.071955	0.012127	0.094423	None
2782	CEP85L	0.266969	0.012133	0.094423	None

2783	DYSF	-0.94922	0.012135	0.094423	Down
2784	GAB2	0.432965	0.01214	0.094427	None
2785	ACP2	-0.68768	0.012156	0.09452	Down
2786	ANKRD13E	-0.19147	0.012196	0.094794	None
2787	PPDPF	-0.48151	0.012213	0.094844	None
2788	PEX13	-0.34749	0.012223	0.094844	None
2789	ZNF70	0.193104	0.012224	0.094844	None
2790	LINC00216	0.025194	0.012224	0.094844	None
2791	RSBN1	0.335125	0.012225	0.094844	None
2792	SESN1	-0.41451	0.012228	0.094844	None
2793	VAV1	-0.38736	0.012246	0.094928	None
2794	ADAM18	0.01232	0.012253	0.094928	None
2795	ZNF385D	-0.54218	0.012255	0.094928	None
2796	SLC43A3	-0.45759	0.012259	0.094928	None
2797	MBLAC1	0.298065	0.012261	0.094928	None
2798	RP11-379H	0.450499	0.012311	0.095277	None
2799	GHDC	-0.46268	0.012326	0.095359	None
2800	ARRDC3	0.781687	0.012336	0.095404	Up
2801	LOC10028!	0.3483	0.012346	0.095448	None
2802	IRAK1	-0.1517	0.012352	0.09545	None
2803	DAND5	0.084575	0.012355	0.09545	None
2804	RPL9	0.082516	0.01236	0.095453	None
2805	B3GNTL1	-0.31191	0.012365	0.09546	None
2806	KLK7	0.09751	0.01237	0.095464	None
2807	CCDC47	0.372003	0.012391	0.095576	None
2808	TEX2	-0.33134	0.012393	0.095576	None
2809	KDM4A	-0.27067	0.01241	0.095673	None
2810	LEPREL4	-0.42558	0.012417	0.095692	None
2811	PRMT9	0.37972	0.01243	0.095754	None
2812	SPATA24	0.213687	0.012444	0.095828	None
2813	STAG1	0.289268	0.01245	0.095833	None
2814	RCVRN	0.040644	0.012453	0.095833	None
2815	STIM1	-0.29584	0.012472	0.095946	None
2816	SLC35A1	-0.30249	0.012488	0.09603	None
2817	IFIT3	-0.87498	0.012503	0.096114	Down
2818	LAMP2	-0.44421	0.012556	0.096462	None
2819	CIPC	-0.52477	0.012557	0.096462	None
2820	OIP5-AS1	-0.35387	0.012589	0.096657	None
2821	MFAP3	-0.3991	0.012592	0.096657	None
2822	SLX4IP	-0.34379	0.012599	0.096677	None
2823	TARSL2	-0.17658	0.012603	0.096677	None
2824	DCLRE1B	-0.35656	0.012635	0.096885	None
2825	LOC44017:	0.128513	0.012639	0.096885	None
2826	HOXD8	0.175139	0.012679	0.097136	None
2827	RC3H1	0.489188	0.012684	0.097136	None
2828	HSPBP1	-0.44828	0.012685	0.097136	None
2829	CDK16	-0.32104	0.012704	0.097245	None
2830	RAVER2	-0.26116	0.012715	0.097291	None
2831	DHTKD1	-0.34175	0.012738	0.097439	None
2832	ULK2	-0.35087	0.012748	0.097478	None
2833	WDYHV1	-0.37332	0.012759	0.097527	None
2834	BAK1	-0.30972	0.012764	0.097533	None
2835	NLN	-0.33606	0.012777	0.097599	None
2836	TMEM135	-0.15952	0.012792	0.097676	None
2837	GPN2	-0.38088	0.012833	0.097926	None
2838	ZBED3	-0.37146	0.012834	0.097926	None
2839	RP5-963E2	0.011962	0.012847	0.097994	None
2840	CLCF1	0.214623	0.012866	0.098039	None

2841	WFDC10A	0.025074	0.012868	0.098039	None
2842	MTHFR	0.187141	0.012868	0.098039	None
2843	HSPA13	0.376427	0.012871	0.098039	None
2844	MNAT1	-0.24455	0.012909	0.098293	None
2845	COLEC12	-1.10006	0.012919	0.098337	Down
2846	GOLGA5	-0.23386	0.01294	0.098433	None
2847	ACTN1	-0.53605	0.012941	0.098433	None
2848	POLR3D	-0.34996	0.01299	0.098768	None
2849	CTSH	-1.07599	0.013	0.098811	Down
2850	RP11-338N	0.094083	0.013015	0.098874	None
2851	ZNF746	-0.26132	0.013017	0.098874	None
2852	BAG1	-0.35767	0.013045	0.099048	None
2853	ATXN3	-0.27919	0.013052	0.099066	None
2854	SRSF11	0.47167	0.01306	0.099096	None
2855	RIPK3	-0.33583	0.013066	0.099108	None
2856	LOC15357	0.57395	0.01308	0.099173	None
2857	RNASEL	-0.33604	0.01309	0.099194	None
2858	CLCN3	-0.47923	0.013092	0.099194	None
2859	ALKBH4	-0.29378	0.01311	0.099301	None
2860	LINC00202	0.10718	0.013117	0.099316	None
2861	ADA	0.583548	0.013122	0.099322	None
2862	ADAM21	0.039497	0.013129	0.099341	None
2863	TADA1	-0.22706	0.013158	0.099471	None
2864	DNAJB5	0.097146	0.013159	0.099471	None
2865	DOK1	-0.28513	0.013162	0.099471	None
2866	NUP214	-0.36239	0.013165	0.099471	None
2867	LOC10050(	-0.55427	0.013179	0.099507	None
2868	EMP3	-0.80634	0.013179	0.099507	Down
2869	RBM5	0.374335	0.013207	0.099682	None
2870	BNIP3L	-0.44336	0.013225	0.099789	None
2871	TAB3	0.331399	0.01326	0.100019	None
2872	BCKDK	-0.40857	0.013281	0.100139	None
2873	ITPR3	0.165327	0.013304	0.100225	None
2874	HTT	-0.31786	0.013314	0.100225	None
2875	RPS6KB2	-0.45839	0.013316	0.100225	None
2876	C6orf203	-0.41827	0.013318	0.100225	None
2877	GPR6	0.038557	0.013319	0.100225	None
2878	CRELD2	0.426377	0.01332	0.100225	None
2879	SERTAD4-/	0.090079	0.013334	0.100265	None
2880	LMBRD2	0.159902	0.013335	0.100265	None
2881	SOCS2	1.10196	0.013345	0.100307	Up
2882	ADAT1	-0.30698	0.01336	0.100386	None
2883	MDS2	0.043363	0.013376	0.10047	None
2884	TRAK2	-0.50275	0.013387	0.100487	None
2885	SEMA5B	0.164181	0.013387	0.100487	None
2886	BCAS4	0.234707	0.013393	0.100491	None
2887	JAK3	0.042057	0.0134	0.100512	None
2888	SRM	-0.56117	0.013406	0.10052	None
2889	CEP55	0.748438	0.013434	0.100698	Up
2890	RP11-803D	-0.50601	0.013444	0.100735	None
2891	ITPR2	-0.41539	0.013448	0.100735	None
2892	SOCS6	0.403914	0.013464	0.100817	None
2893	IL12RB2	0.666242	0.013493	0.100996	Up
2894	RP11-456(	0.080558	0.013513	0.101118	None
2895	CBR3	0.487643	0.013522	0.101134	None
2896	SCAND1	0.378561	0.013525	0.101134	None
2897	CCDC25	-0.26206	0.013551	0.101297	None
2898	PSMC3IP	0.353828	0.013561	0.101333	None

2899	CDRT1	0.005467	0.013572	0.101379	None
2900	LOC10272	0.046368	0.013639	0.101845	None
2901	ZBED6CL	-0.38291	0.013653	0.101892	None
2902	MT3	0.139795	0.013655	0.101892	None
2903	LILRA4	0.712783	0.013691	0.102103	Up
2904	DTWD2	0.305935	0.013692	0.102103	None
2905	FBXO45	-0.47507	0.013701	0.102109	None
2906	GCNT7	0.198409	0.013703	0.102109	None
2907	CCR4	0.006442	0.013718	0.102188	None
2908	HNRNPA2I	0.330897	0.01373	0.102242	None
2909	LINC00888	-0.26309	0.013759	0.102422	None
2910	C10orf54	-0.44445	0.013767	0.10245	None
2911	LPAR5	-0.48348	0.013798	0.102642	None
2912	REV1	-0.25952	0.013811	0.102704	None
2913	CDC23	0.345979	0.01383	0.102808	None
2914	NPL	-0.84876	0.013849	0.102891	Down
2915	MICB	0.322453	0.01385	0.102891	None
2916	NRAP	0.006382	0.013877	0.103057	None
2917	ZBTB40	-0.44742	0.013911	0.103274	None
2918	KDM5B	-0.39383	0.013922	0.103315	None
2919	LINC01268	-0.95197	0.013938	0.103401	Down
2920	C1orf131	-0.39406	0.013946	0.103427	None
2921	TBC1D23	-0.28505	0.01397	0.10357	None
2922	ZNF492	0.600216	0.013979	0.103601	Up
2923	GALNT12	-0.47628	0.013986	0.103613	None
2924	WDR61	0.264188	0.014002	0.103657	None
2925	RNF175	-1.11333	0.014005	0.103657	Down
2926	MCEMP1	1.008808	0.014006	0.103657	Up
2927	UPB1	0.102743	0.014024	0.103727	None
2928	HDDC3	-0.37313	0.014025	0.103727	None
2929	LOC10192	0.076438	0.014043	0.103823	None
2930	SREBF2	0.353841	0.014059	0.10391	None
2931	HIST1H2BI	-0.77186	0.014069	0.103949	Down
2932	ARL14EP	-0.29451	0.014104	0.10413	None
2933	TNFAIP3	0.897388	0.014108	0.10413	Up
2934	GPSM3	-0.51368	0.014108	0.10413	None
2935	ATP6V0A4	0.009701	0.014116	0.104135	None
2936	ESX1	0.043315	0.014119	0.104135	None
2937	RPLP0	0.092072	0.014141	0.104263	None
2938	LOC10272	0.105097	0.014149	0.104286	None
2939	MYO3A	0.011202	0.014157	0.104309	None
2940	ELP4	-0.3789	0.014165	0.104317	None
2941	TWISTNB	-0.30442	0.014167	0.104317	None
2942	ARID4A	0.304775	0.014173	0.104323	None
2943	SLC22A5	-0.23245	0.014187	0.104388	None
2944	MCCC2	-0.34332	0.014196	0.10442	None
2945	GPSM1	0.207181	0.014203	0.104435	None
2946	GTPBP4	0.347725	0.014214	0.104482	None
2947	AFMID	-0.33103	0.014223	0.104511	None
2948	ZNF316	-0.11865	0.014238	0.104584	None
2949	PRMT2	-0.29257	0.014258	0.104697	None
2950	TMEM44-/	-0.42255	0.014284	0.104797	None
2951	ANO5	-0.77017	0.014286	0.104797	Down
2952	KLHDC7B	-0.44189	0.014286	0.104797	None
2953	TMEM123	0.497442	0.014295	0.104828	None
2954	NDUFA8	-0.39583	0.014345	0.10516	None
2955	BTBD6	0.583082	0.014366	0.105253	None
2956	KLF17	0.042204	0.014367	0.105253	None

2957	ZDHHC3	-0.32618	0.014391	0.105345	None
2958	ACPT	0.190718	0.014393	0.105345	None
2959	ENPP7	0.329073	0.014395	0.105345	None
2960	DES	0.072731	0.014417	0.105473	None
2961	C11orf71	-0.24807	0.014425	0.105485	None
2962	API5	0.425549	0.014432	0.105485	None
2963	HILPDA	-0.51481	0.014433	0.105485	None
2964	RGS19	-0.42061	0.014439	0.10549	None
2965	LOXL4	-0.33737	0.014457	0.105587	None
2966	DOLK	-0.45532	0.014471	0.10565	None
2967	CCL7	0.342411	0.014533	0.106067	None
2968	PSG6	0.009161	0.014568	0.106291	None
2969	LOC10192	0.020374	0.014575	0.106308	None
2970	DNAJC16	-0.53918	0.014611	0.106515	None
2971	SMAD4	0.550984	0.014614	0.106515	None
2972	PPP6R2	-0.24057	0.014655	0.106782	None
2973	SLC25A48	0.069712	0.014664	0.106808	None
2974	PACS2	-0.22537	0.014682	0.106908	None
2975	ZYX	-0.81233	0.014703	0.107021	Down
2976	HAB1	0.11697	0.01471	0.107035	None
2977	OLFML2B	-0.55437	0.014719	0.107064	None
2978	RGS10	-0.42683	0.014742	0.1072	None
2979	CNNM4	-0.25218	0.014747	0.1072	None
2980	TAF1L	0.14154	0.014809	0.107616	None
2981	TUB	-0.0684	0.014815	0.107624	None
2982	RNF26	-0.27678	0.014849	0.107835	None
2983	GATA2	-0.45887	0.014854	0.107835	None
2984	RAB5C	-0.68176	0.01488	0.107974	Down
2985	MIR4435-1	-0.23127	0.014886	0.107974	None
2986	TIMELESS	0.256466	0.014889	0.107974	None
2987	MYH9	-0.40842	0.014898	0.107985	None
2988	HTRA3	-0.43638	0.0149	0.107985	None
2989	KATNA1	-0.30837	0.014923	0.108082	None
2990	CD300LF	-0.55176	0.014923	0.108082	None
2991	ZNF366	0.05033	0.014957	0.108288	None
2992	DUS1L	-0.36283	0.014966	0.108315	None
2993	GRIP1	-0.28949	0.014979	0.108377	None
2994	TXNL4B	-0.31501	0.014986	0.108389	None
2995	KIF21B	-0.40026	0.015012	0.108504	None
2996	COG4	-0.33368	0.015012	0.108504	None
2997	FAM120A	-0.29961	0.015017	0.108505	None
2998	GAL	-0.40937	0.015025	0.108531	None
2999	LOC10050	0.117242	0.015063	0.108757	None
3000	HPCAL1	-0.39657	0.015072	0.108757	None
3001	C9orf53	0.066443	0.015072	0.108757	None
3002	GFOD2	-0.20737	0.01509	0.108849	None
3003	LINC01208	0.254333	0.015096	0.10886	None
3004	LOC10193	0.054524	0.01513	0.109068	None
3005	CHMP2A	-0.36861	0.015185	0.109394	None
3006	KLK3	0.20243	0.015185	0.109394	None
3007	RNF43	-0.24859	0.015204	0.109492	None
3008	TPI1	-0.39578	0.015231	0.109621	None
3009	FATE1	0.115868	0.015232	0.109621	None
3010	FAM173A	-0.42817	0.015244	0.109669	None
3011	LOXL3	-0.38505	0.015259	0.109709	None
3012	MSI1	0.128489	0.015267	0.109709	None
3013	LLGL1	0.203632	0.015272	0.109709	None
3014	ZNF222	-0.45998	0.015275	0.109709	None

3015	PTOV1	-0.40593	0.015276	0.109709	None
3016	DCHS1	-0.40328	0.015281	0.109709	None
3017	MILR1	0.625956	0.015285	0.109709	Up
3018	ZNF397	-0.27242	0.015298	0.109769	None
3019	SLC22A24	0.02213	0.015325	0.109922	None
3020	MAGEA6	0.00489	0.015336	0.109969	None
3021	FAM134B	0.351383	0.015343	0.109985	None
3022	MTFR1	-0.55877	0.015357	0.110043	None
3023	NIPSNAP1	-0.40003	0.015375	0.110138	None
3024	ZNF185	-0.78644	0.015384	0.110139	Down
3025	FAM107B	0.78565	0.015385	0.110139	Up
3026	LINC00334	0.019561	0.015409	0.110268	None
3027	RNF40	-0.30857	0.015421	0.11031	None
3028	LOC34008!	0.336525	0.015425	0.11031	None
3029	MFNG	-0.52666	0.015432	0.110329	None
3030	PAX1	0.023446	0.015442	0.110361	None
3031	GNPNAT1	-0.36052	0.015448	0.11037	None
3032	NELFB	-0.3416	0.015488	0.110616	None
3033	COIL	-0.25527	0.015507	0.110716	None
3034	LOC10192!	0.054036	0.015531	0.110825	None
3035	LINC01260	0.077415	0.015533	0.110825	None
3036	SH3BGR	-0.36127	0.015538	0.110825	None
3037	SLC25A15	-0.52782	0.015543	0.110825	None
3038	PDK3	-0.58175	0.015561	0.110919	None
3039	CCDC130	-0.28982	0.015608	0.111214	None
3040	PGRMC2	0.305573	0.015622	0.111279	None
3041	RNF157-A!	0.010886	0.015696	0.11177	None
3042	2-Mar	-0.77003	0.01571	0.111832	Down
3043	PPP1R12B	-0.20642	0.015747	0.11206	None
3044	DEFB121	0.005779	0.015764	0.112143	None
3045	ZCCHC14	0.291399	0.015777	0.112198	None
3046	STAMBPL1	-0.44672	0.015783	0.112207	None
3047	DLEU1	-0.51941	0.015798	0.112261	None
3048	SRP14	0.212538	0.015805	0.112261	None
3049	ZNHIT3	-0.39892	0.015806	0.112261	None
3050	H2AFY2	-0.6704	0.015821	0.112331	Down
3051	EARS2	-0.30541	0.015835	0.112391	None
3052	LOC43993!	0.020379	0.015875	0.112636	None
3053	AHSG	0.091845	0.015918	0.11291	None
3054	LNX2	-0.29566	0.015947	0.113079	None
3055	ZNF486	0.257709	0.015956	0.113106	None
3056	AZIN1	-0.2339	0.015984	0.113265	None
3057	TTTY7	0.028819	0.016003	0.113317	None
3058	CD101	0.138521	0.016003	0.113317	None
3059	RP11-54K1	-0.26623	0.016007	0.113317	None
3060	HMGN2	0.184202	0.01602	0.113343	None
3061	WFDC1	-0.67896	0.016021	0.113343	Down
3062	SRD5A2	0.052143	0.016048	0.113482	None
3063	C1orf85	-0.54575	0.016051	0.113482	None
3064	STIP1	-0.51307	0.016068	0.113495	None
3065	TECPR2	-0.36249	0.016069	0.113495	None
3066	IFT22	0.242911	0.016078	0.113495	None
3067	TBC1D10A	-0.15231	0.016079	0.113495	None
3068	LYG2	0.048056	0.01608	0.113495	None
3069	SLC36A4	0.362853	0.016105	0.113635	None
3070	ACADS	-0.15725	0.016126	0.11375	None
3071	TIMM44	-0.37204	0.01616	0.113949	None
3072	USP9X	0.238355	0.016212	0.114248	None

3073	LARP7	0.517906	0.016216	0.114248	None
3074	ASNS	-0.26161	0.01622	0.114248	None
3075	ANAPC4	-0.25226	0.016224	0.114248	None
3076	LOC10192!	0.181369	0.016228	0.114248	None
3077	ANKRD27	-0.34306	0.016238	0.114263	None
3078	MCM6	0.307539	0.016241	0.114263	None
3079	MFSD9	-0.19178	0.016249	0.114283	None
3080	CCDC88C	-0.41935	0.016268	0.114378	None
3081	NUP153	0.334633	0.016284	0.114436	None
3082	SOD2	0.561333	0.016287	0.114436	None
3083	SH2D1B	0.111205	0.016296	0.114464	None
3084	MOCS3	-0.29121	0.016304	0.114479	None
3085	VAMP7	-0.21676	0.016312	0.114502	None
3086	BMP6	0.056016	0.01641	0.115097	None
3087	HIF1AN	-0.2634	0.016411	0.115097	None
3088	DHDDS	-0.40267	0.016413	0.115097	None
3089	CORO2A	-0.36567	0.016457	0.115347	None
3090	COTL1	-0.74413	0.016459	0.115347	Down
3091	LOC10050!	0.154748	0.016475	0.115424	None
3092	CERS2	-0.2865	0.016494	0.115516	None
3093	RNF44	-0.31516	0.016535	0.115764	None
3094	CSF3R	-0.7392	0.016565	0.115905	Down
3095	LOC10192	0.03465	0.016566	0.115905	None
3096	ZNF398	-0.3827	0.016587	0.116017	None
3097	VWA8	-0.26363	0.016596	0.11604	None
3098	FOXO3	-0.31253	0.016612	0.116119	None
3099	OGFR	-0.24816	0.016618	0.116121	None
3100	ZKSCAN7	-0.31728	0.016634	0.116198	None
3101	DCTN2	0.317156	0.01664	0.116198	None
3102	PTPLB	-0.33279	0.016645	0.116198	None
3103	MIR4755	0.126951	0.016677	0.116382	None
3104	XPO5	-0.44101	0.016705	0.116487	None
3105	MGME1	-0.30142	0.016708	0.116487	None
3106	HDAC9	0.22019	0.016708	0.116487	None
3107	CCDC86	-0.48335	0.016716	0.116487	None
3108	LINC00623	-0.36564	0.016719	0.116487	None
3109	CARD8	-0.30913	0.016742	0.116615	None
3110	VPS13A-A!	0.1039	0.016754	0.116628	None
3111	DPP10	-1.25785	0.016755	0.116628	Down
3112	MAGOH2	0.229687	0.016771	0.116701	None
3113	TCF3	0.291958	0.016787	0.116741	None
3114	RP4-813F1	-0.33952	0.016787	0.116741	None
3115	SLC45A3	-0.21	0.016806	0.11679	None
3116	HDGFL1	0.194444	0.016814	0.11679	None
3117	TRIM25	-0.23321	0.016819	0.11679	None
3118	RECQL	0.484677	0.016819	0.11679	None
3119	HCFC1R1	-0.24778	0.016821	0.11679	None
3120	TRAF3IP2-	0.1594	0.016894	0.117256	None
3121	PLCXD1	-0.252	0.016945	0.117574	None
3122	CXorf57	0.499846	0.016979	0.117736	None
3123	RAB3IL1	0.289343	0.016979	0.117736	None
3124	SLC22A3	0.204911	0.016996	0.117815	None
3125	THOP1	-0.38399	0.017002	0.117815	None
3126	CDH13	0.05793	0.017007	0.117817	None
3127	ZDHHC14	-0.19449	0.017028	0.117887	None
3128	PDP2	-0.25955	0.017034	0.117887	None
3129	ZNF843	0.058298	0.017038	0.117887	None
3130	LOC10192(	0.473496	0.017039	0.117887	None

3131	FBXO33	0.346533	0.017071	0.11804	None
3132	CD72	1.046091	0.017072	0.11804	Up
3133	NPLOC4	-0.32332	0.017083	0.118077	None
3134	ASPM	0.740896	0.01709	0.118084	Up
3135	MT1H	-1.08501	0.017096	0.118091	Down
3136	RP11-254F	0.016872	0.017109	0.11814	None
3137	SELO	0.389858	0.017118	0.118169	None
3138	SERPINB2	1.248179	0.017135	0.11825	Up
3139	GPR161	-0.24324	0.01716	0.118341	None
3140	DNAJC24	-0.1943	0.017161	0.118341	None
3141	MTL5	-0.21949	0.017183	0.118341	None
3142	MBD3	-0.31132	0.017184	0.118341	None
3143	SEPW1	-0.37185	0.01719	0.118341	None
3144	CD2AP	0.149168	0.017194	0.118341	None
3145	ODF3	0.145618	0.017197	0.118341	None
3146	SAR1B	-0.58762	0.017197	0.118341	Down
3147	DGKB	0.016846	0.017198	0.118341	None
3148	LINC01197	0.006643	0.017224	0.118484	None
3149	ATG2B	-0.24057	0.017236	0.118525	None
3150	IL5	0.060402	0.017241	0.118525	None
3151	TOM1L2	-0.13814	0.017265	0.118653	None
3152	SLC27A1	-0.25072	0.017277	0.118697	None
3153	GPR37L1	0.07158	0.017308	0.118852	None
3154	PLAC8L1	0.167438	0.017313	0.118852	None
3155	COX7A2	0.187222	0.017316	0.118852	None
3156	PIGK	-0.38679	0.017326	0.118882	None
3157	PRSS55	0.019849	0.017337	0.11892	None
3158	LOC57399	0.047716	0.017349	0.118937	None
3159	HCST	-0.5626	0.01735	0.118937	None
3160	NUP160	0.523077	0.017382	0.119086	None
3161	CSTF3-AS1	0.077502	0.017388	0.119086	None
3162	CTC-338M	-0.30514	0.017389	0.119086	None
3163	KLHL26	-0.2226	0.017403	0.119146	None
3164	PRKCQ	-0.48179	0.017424	0.11925	None
3165	PIGV	-0.36305	0.017434	0.119252	None
3166	IL17RD	0.029749	0.017435	0.119252	None
3167	TARS	-0.18914	0.017458	0.119372	None
3168	SLC22A4	0.896545	0.017474	0.119446	Up
3169	TBC1D14	-0.29013	0.017489	0.119482	None
3170	GUCA1C	0.005586	0.017498	0.119482	None
3171	SWI5	-0.38089	0.017502	0.119482	None
3172	RSU1P2	0.050041	0.017506	0.119482	None
3173	SYNGAP1	-0.10756	0.017507	0.119482	None
3174	ARL6IP1	0.225293	0.017527	0.119583	None
3175	TMEM138	-0.28136	0.017534	0.119592	None
3176	YY1AP1	-0.25344	0.017542	0.119604	None
3177	LOC10050(	-0.37705	0.017567	0.11974	None
3178	IL19	0.004697	0.017578	0.119779	None
3179	BC015159	0.006193	0.017586	0.119792	None
3180	CLEC4C	0.494793	0.017636	0.120097	None
3181	PCDHAC1	0.0181	0.017678	0.120347	None
3182	GDI1	-0.35742	0.017686	0.120364	None
3183	HADHB	-0.1689	0.017713	0.120492	None
3184	NUFIP1	-0.39362	0.017716	0.120492	None
3185	PIK3R6	-0.36191	0.017743	0.120591	None
3186	BCS1L	-0.44827	0.017751	0.120591	None
3187	PTP4A3	-0.45535	0.017758	0.120591	None
3188	ZNF789	-0.12849	0.017761	0.120591	None

3189	UCK1	-0.17462	0.017764	0.120591	None
3190	ZNF80	0.069154	0.017764	0.120591	None
3191	FLJ11292	0.02832	0.017798	0.12075	None
3192	WDR6	-0.23562	0.017802	0.12075	None
3193	SIPA1L3	-0.08828	0.017804	0.12075	None
3194	PAQR8	-0.41148	0.017817	0.120799	None
3195	MIAT	-0.44826	0.017834	0.120877	None
3196	PCGF1	-0.33372	0.017853	0.120965	None
3197	ZDHHC6	-0.21551	0.017874	0.121073	None
3198	TTPAL	-0.24607	0.01789	0.12114	None
3199	LINC01142	0.049423	0.017912	0.121254	None
3200	SF3B2	-0.11184	0.017937	0.12138	None
3201	LOC10192[illegible]	0.156253	0.017971	0.121573	None
3202	IARS	-0.22907	0.017989	0.121639	None
3203	FBXO9	-0.41476	0.017992	0.121639	None
3204	PVRL2	-0.28303	0.018	0.121657	None
3205	LXN	-0.56452	0.018007	0.121669	None
3206	CNNM3	-0.41637	0.018027	0.121761	None
3207	RP11-292[illegible]	0.017472	0.018034	0.121774	None
3208	COASY	-0.26725	0.018042	0.121778	None
3209	DCUN1D4	-0.41968	0.018046	0.121778	None
3210	LINC00452	0.095486	0.018052	0.121783	None
3211	MRPL42	-0.27075	0.018062	0.121807	None
3212	SLC5A6	-0.41498	0.018088	0.121883	None
3213	LINC0142[illegible]	-0.28136	0.018089	0.121883	None
3214	TTC1	-0.39125	0.01809	0.121883	None
3215	MEF2C	0.420499	0.018104	0.121943	None
3216	CHN2	-0.1881	0.018122	0.122022	None
3217	RBP3	0.076711	0.018134	0.122065	None
3218	OR8B8	0.020994	0.01814	0.122072	None
3219	ZGRF1	0.209702	0.01816	0.122152	None
3220	DQX1	0.025643	0.018164	0.122152	None
3221	WBP2	-0.53115	0.01817	0.122158	None
3222	SERPINH1	-0.59365	0.018216	0.122432	Down
3223	GLUD1	-0.35191	0.018245	0.122575	None
3224	CYFIP1	-0.34309	0.018249	0.122575	None
3225	PARP12	-0.49089	0.018271	0.122666	None
3226	ZNF565	-0.38516	0.018274	0.122666	None
3227	ETNK2	0.112202	0.018281	0.122673	None
3228	ZMYND19	-0.38912	0.018299	0.122758	None
3229	MED25	-0.10434	0.018309	0.12277	None
3230	DPM1	0.248111	0.018312	0.12277	None
3231	USP40	-0.27667	0.018319	0.12278	None
3232	ECHDC2	-0.69143	0.018348	0.122925	Down
3233	AP2M1	-0.36605	0.018356	0.122925	None
3234	HPS5	-0.2685	0.018358	0.122925	None
3235	SULT1A1	-0.51612	0.018364	0.122925	None
3236	HEYL	-0.11589	0.018379	0.122981	None
3237	TMEM97	-0.42374	0.018383	0.122981	None
3238	LOC28605[illegible]	-0.49245	0.018394	0.122998	None
3239	RP1-130G[illegible]	0.387981	0.018401	0.122998	None
3240	FRMD3	-0.94957	0.018403	0.122998	Down
3241	FAM98A	-0.33608	0.018409	0.122998	None
3242	FAM83H	-0.18853	0.018417	0.123016	None
3243	TMEM38A	0.07729	0.01845	0.1232	None
3244	C11orf49	-0.14542	0.018469	0.123257	None
3245	ZNF831	0.0575	0.01847	0.123257	None
3246	TRBV27	-1.17781	0.018477	0.123265	Down

3247	LOC28612:	0.009124	0.018493	0.123298	None
3248	ERGIC2	-0.34988	0.018494	0.123298	None
3249	ENOPH1	-0.25625	0.018504	0.123298	None
3250	C14orf119	-0.39985	0.018505	0.123298	None
3251	MRPS9	-0.37272	0.018533	0.123449	None
3252	TP53	-0.35871	0.018539	0.123449	None
3253	RP3-508I1!	0.307965	0.018568	0.123608	None
3254	TMEM185/	-0.37795	0.018578	0.123636	None
3255	WRN	0.36509	0.018593	0.123698	None
3256	SPATA7	-0.46359	0.018623	0.123856	None
3257	FAM129A	-0.4796	0.018654	0.123976	None
3258	ZNF555	0.232813	0.018657	0.123976	None
3259	LOC10050(	0.023336	0.018658	0.123976	None
3260	SLC27A4	-0.27317	0.018669	0.124012	None
3261	COMMD7	-0.11516	0.018681	0.124021	None
3262	C20orf202	0.083777	0.018682	0.124021	None
3263	STK11IP	-0.44801	0.018689	0.124021	None
3264	SAFB2	-0.24273	0.018693	0.124021	None
3265	CYGB	0.13484	0.018723	0.124161	None
3266	PAXIP1-AS	-0.3413	0.018726	0.124161	None
3267	AGT	0.126763	0.018735	0.124176	None
3268	PDCD11	-0.29934	0.018743	0.124176	None
3269	MTUS2-AS	0.098323	0.018747	0.124176	None
3270	BOLA3-AS	-0.3664	0.018751	0.124176	None
3271	BC069004	0.103373	0.018763	0.124197	None
3272	LOC10192(	0.021456	0.018767	0.124197	None
3273	CHTOP	-0.22911	0.018771	0.124197	None
3274	FAM219B	-0.36202	0.018798	0.124332	None
3275	ZFPL1	-0.26754	0.018813	0.124398	None
3276	LINC01103	0.062356	0.01883	0.124448	None
3277	TRIML2	0.021145	0.018835	0.124448	None
3278	NBR1	-0.18336	0.018844	0.124448	None
3279	TRMT12	0.275801	0.018844	0.124448	None
3280	CBX3	0.304487	0.018865	0.124548	None
3281	CEND1	0.072352	0.018885	0.124642	None
3282	NATD1	-0.41817	0.0189	0.124702	None
3283	ZDHHC16	-0.34399	0.018909	0.124724	None
3284	GLRX2	-0.3231	0.018927	0.12477	None
3285	PCDHB12	0.047309	0.01893	0.12477	None
3286	TBC1D9	-0.69986	0.018933	0.12477	Down
3287	PDZD11	-0.52542	0.018953	0.124819	None
3288	BC041363	-1.06881	0.018953	0.124819	Down
3289	FMO4	-0.42961	0.018963	0.124819	None
3290	CD99L2	-0.11647	0.018963	0.124819	None
3291	GSTM4	-0.34414	0.018987	0.124905	None
3292	SYCP2	-0.54519	0.018988	0.124905	None
3293	UBE2O	-0.2652	0.019014	0.125037	None
3294	LGR6	0.239009	0.019065	0.125315	None
3295	VIMP	0.369625	0.019068	0.125315	None
3296	SLC17A5	-0.32994	0.019167	0.125903	None
3297	CSTF2	-0.30241	0.019169	0.125903	None
3298	CAPN1	-0.29075	0.019184	0.125913	None
3299	NDUFAF3	-0.43164	0.019184	0.125913	None
3300	PPOX	-0.34584	0.019188	0.125913	None
3301	CD82	-0.39879	0.019205	0.125971	None
3302	HCK	-1.03283	0.019208	0.125971	Down
3303	RP11-127E	0.482736	0.019232	0.126087	None
3304	SLC25A28	-0.50106	0.019286	0.126402	None

3305	MLLT3	-0.62212	0.019299	0.126452	Down
3306	ANKRD17	-0.25069	0.019308	0.126472	None
3307	HERC2	-0.29139	0.019317	0.126489	None
3308	TOR1AIP1	-0.4296	0.019331	0.126545	None
3309	LOC10192ǃ	0.058398	0.01935	0.126632	None
3310	EIF3H	-0.28681	0.019403	0.126938	None
3311	RIT2	0.010992	0.019433	0.127083	None
3312	MYO1D	-0.27715	0.019437	0.127083	None
3313	EGFLAM-A	0.009139	0.019447	0.127112	None
3314	LOC28617ǃ	0.060801	0.019469	0.127181	None
3315	EPHA10	0.025252	0.019469	0.127181	None
3316	SATB1	0.570351	0.019478	0.1272	None
3317	FKRP	-0.20001	0.019491	0.127211	None
3318	KCNH6	0.017731	0.019491	0.127211	None
3319	RPS6KA2	-0.5506	0.019498	0.127214	None
3320	SPATA1	0.143324	0.019512	0.127268	None
3321	LIN54	0.177146	0.019545	0.127417	None
3322	CDK2AP2	-0.60716	0.019547	0.127417	Down
3323	TAF1C	-0.21976	0.019558	0.127417	None
3324	NAPG	0.27357	0.019558	0.127417	None
3325	PPCS	-0.28459	0.019581	0.127487	None
3326	MID1IP1	-0.4808	0.019581	0.127487	None
3327	CAPS	-0.118	0.019601	0.127582	None
3328	OS9	-0.33837	0.019617	0.127643	None
3329	MYSM1	0.308736	0.019639	0.12775	None
3330	PSMD14	-0.27165	0.019667	0.127842	None
3331	TPR	0.498512	0.019668	0.127842	None
3332	CNR2	0.070897	0.019671	0.127842	None
3333	DSC2	-0.6683	0.019677	0.127847	Down
3334	OAS1	-0.96818	0.019718	0.128069	Down
3335	SSPN	-0.35317	0.019738	0.128162	None
3336	DEFB124	0.155391	0.019754	0.128226	None
3337	PDP1	0.374868	0.019806	0.128531	None
3338	IQSEC2	0.121509	0.019828	0.128631	None
3339	WFDC6	0.029817	0.019849	0.128658	None
3340	LAMC1	-0.59848	0.019853	0.128658	Down
3341	MAP4K1	-0.45136	0.019856	0.128658	None
3342	HDAC7	-0.22529	0.019856	0.128658	None
3343	NXPH3	0.080423	0.019892	0.128856	None
3344	WFDC8	0.064502	0.019903	0.128884	None
3345	GMPR	-0.84224	0.01991	0.128891	Down
3346	LOC10192	-0.32571	0.019924	0.128943	None
3347	CCDC27	0.046679	0.019955	0.129085	None
3348	DPCR1	0.02455	0.019957	0.129085	None
3349	LOC10013	0.064421	0.019976	0.129169	None
3350	TMA16	0.614379	0.020031	0.129486	Up
3351	SLC27A6	-0.7057	0.020042	0.129514	Down
3352	C2orf47	-0.20803	0.020048	0.129514	None
3353	AP1G1	-0.28032	0.020054	0.129519	None
3354	CNOT11	-0.29619	0.020087	0.129689	None
3355	BRPF3	0.045431	0.02012	0.129744	None
3356	ICMT	-0.39939	0.020122	0.129744	None
3357	LRIF1	-0.62207	0.020123	0.129744	Down
3358	LZTR1	-0.22466	0.020129	0.129744	None
3359	CEP112	0.020239	0.02013	0.129744	None
3360	GPATCH11	-0.23226	0.020131	0.129744	None
3361	ZNF595	0.725165	0.020145	0.129795	Up
3362	C11orf74	-0.3517	0.020158	0.129816	None

3363	RP4-781L3	-0.38684	0.02016	0.129816	None
3364	SPRR3	0.049645	0.020169	0.129819	None
3365	SORD	-0.43428	0.020173	0.129819	None
3366	CRK	-0.43926	0.020181	0.129819	None
3367	BIRC3	0.767177	0.020185	0.129819	Up
3368	CLN8	0.354245	0.020193	0.129835	None
3369	PLCG2	0.187486	0.020211	0.129913	None
3370	C1orf162	-0.52198	0.020221	0.129935	None
3371	PNMA3	-0.23883	0.020237	0.130002	None
3372	ZNF883	-0.86207	0.020275	0.130208	Down
3373	DHPS	0.449528	0.020298	0.13026	None
3374	LOC10192	0.117599	0.0203	0.13026	None
3375	NDUFV1	0.194215	0.020301	0.13026	None
3376	GLTP	-0.36227	0.020342	0.130444	None
3377	CHRD	0.008355	0.020342	0.130444	None
3378	KIAA0907	-0.24922	0.020355	0.130485	None
3379	NT5C1A	0.078756	0.020399	0.130712	None
3380	ABCB6	-0.27021	0.020404	0.130712	None
3381	D2HGDH	-0.53745	0.020408	0.130712	None
3382	PLAGL2	-0.36616	0.02043	0.130812	None
3383	NDUFA5	0.407215	0.020446	0.130877	None
3384	PCAT6	-0.27928	0.020481	0.131062	None
3385	RAB1B	-0.32312	0.020556	0.131478	None
3386	PSMD6	-0.29387	0.020558	0.131478	None
3387	SNTB1	-0.23172	0.020574	0.131542	None
3388	CCL17	0.111391	0.020589	0.131574	None
3389	UTP18	-0.09861	0.020591	0.131574	None
3390	FCGR2A	-0.48041	0.020602	0.131604	None
3391	SH3BP5L	-0.19677	0.02064	0.131805	None
3392	TIAL1	-0.2178	0.02071	0.132215	None
3393	HS3ST6	0.01951	0.020745	0.132403	None
3394	MAZ	-0.24527	0.020763	0.132475	None
3395	DHODH	-0.33944	0.020786	0.132573	None
3396	PXDN	1.017365	0.02079	0.132573	Up
3397	ADTRP	0.095539	0.020847	0.132897	None
3398	LINC00612	0.012602	0.020889	0.13312	None
3399	LYRM2	-0.33242	0.020912	0.133192	None
3400	PTGR1	-0.51149	0.020912	0.133192	None
3401	C11orf94	0.11156	0.020924	0.133228	None
3402	STAT5A	-0.29292	0.020933	0.133246	None
3403	NOL9	-0.36128	0.020951	0.133291	None
3404	PPP2R5A	-0.2004	0.020952	0.133291	None
3405	CDC37L1	-0.38746	0.020989	0.133488	None
3406	NFE2L1	-0.26266	0.021032	0.133722	None
3407	CSGALNAC	0.562282	0.02107	0.133924	None
3408	CHIC2	-0.65268	0.021103	0.134013	Down
3409	SLC13A5	0.078873	0.021106	0.134013	None
3410	STH	0.051593	0.021107	0.134013	None
3411	RNASE2	0.86819	0.021112	0.134013	Up
3412	FAM214B	-0.35657	0.021115	0.134013	None
3413	BAMBI	-0.78472	0.021123	0.134013	Down
3414	C1RL	-0.37527	0.021134	0.134013	None
3415	MAGEA11	0.006685	0.021137	0.134013	None
3416	MYO1A	0.065243	0.021146	0.134013	None
3417	POLR3C	0.3256	0.021146	0.134013	None
3418	XRCC1	-0.2975	0.021173	0.134144	None
3419	LDHA	0.164969	0.021186	0.134189	None
3420	CHAMP1	-0.60537	0.021208	0.134255	Down

3421	HOXA1	-0.74154	0.021209	0.134255	Down
3422	MRPS28	-0.52633	0.021239	0.134403	None
3423	CCT8	-0.25686	0.021268	0.134525	None
3424	MOCS2	-0.3375	0.021273	0.134525	None
3425	TPST2	-0.43361	0.021277	0.134525	None
3426	KIF3C	-0.28663	0.021283	0.134527	None
3427	CACNA1I	0.03481	0.021297	0.134573	None
3428	NUP210P1	0.122694	0.021311	0.134625	None
3429	LOC28637(	0.011081	0.021329	0.134697	None
3430	NRGN	-0.94152	0.021337	0.13471	Down
3431	ZNF256	0.488304	0.021349	0.134743	None
3432	WBP11	-0.46244	0.021365	0.13481	None
3433	ZNF626	0.234235	0.021396	0.134964	None
3434	SH3BGRL2	-0.6653	0.021412	0.135025	Down
3435	PRRC2C	-0.45745	0.021429	0.135096	None
3436	SSBP1	0.460621	0.021455	0.135189	None
3437	TMOD1	-0.7954	0.021457	0.135189	Down
3438	CRHBP	0.982635	0.021482	0.135276	Up
3439	GABARAP	-0.27027	0.021491	0.135276	None
3440	ACIN1	-0.42957	0.021491	0.135276	None
3441	ALKBH8	-0.31858	0.021495	0.135276	None
3442	UBL4A	-0.39138	0.021522	0.135403	None
3443	NKRF	-0.15983	0.021551	0.135548	None
3444	PTCSC1	0.109021	0.021561	0.13557	None
3445	HBEGF	0.433183	0.02159	0.135711	None
3446	TSLP	-0.85158	0.021597	0.135717	Down
3447	TMEM200E	-0.37816	0.021617	0.135802	None
3448	LEO1	-0.37662	0.02163	0.135831	None
3449	RBM6	0.77283	0.021634	0.135831	Up
3450	MYBL2	0.621976	0.021641	0.135838	Up
3451	ZNF569	0.253971	0.021648	0.135843	None
3452	CPNE8	-0.30947	0.021715	0.136222	None
3453	AUH	-0.35647	0.021739	0.136335	None
3454	ATXN10	-0.24468	0.021747	0.136343	None
3455	RUSC1-AS	-0.30839	0.02176	0.136384	None
3456	PUS7	-0.48313	0.021791	0.136462	None
3457	HSD17B11	-0.34968	0.021798	0.136462	None
3458	STK4-AS1	0.14909	0.0218	0.136462	None
3459	RP5-1074L	-0.65025	0.021801	0.136462	Down
3460	CHP1	-0.24304	0.021807	0.136462	None
3461	MOGS	-0.43756	0.02181	0.136462	None
3462	CCNH	0.289759	0.021817	0.136465	None
3463	SLC4A9	0.074527	0.021844	0.136509	None
3464	ZNF829	0.180042	0.021845	0.136509	None
3465	IFI6	-0.88572	0.021849	0.136509	Down
3466	SYTL2	0.511563	0.021849	0.136509	None
3467	TNFAIP2	-0.39832	0.021881	0.13667	None
3468	TMEM121	-0.24783	0.021911	0.136819	None
3469	ZBTB42	-0.37635	0.021923	0.136852	None
3470	ERMP1	-0.40956	0.021932	0.136871	None
3471	MGC24103	0.008867	0.021949	0.136935	None
3472	RPL15	0.250049	0.021981	0.137062	None
3473	GALE	0.199092	0.021982	0.137062	None
3474	MGAT5	0.174229	0.022004	0.137119	None
3475	CASC15	0.111413	0.022004	0.137119	None
3476	ASXL1	0.314863	0.022014	0.137119	None
3477	FUT10	0.152182	0.022022	0.137119	None
3478	GNL3L	-0.20194	0.022033	0.137119	None

3479	FGG	0.020449	0.02204	0.137119	None
3480	PCBP1	-0.277	0.022043	0.137119	None
3481	SBF2	0.709889	0.022048	0.137119	Up
3482	TK2	-0.35075	0.022053	0.137119	None
3483	FYTTD1	0.218796	0.022054	0.137119	None
3484	ARMC7	-0.21007	0.022112	0.137431	None
3485	CCBE1	0.008641	0.022118	0.137431	None
3486	GCSAM	0.465131	0.022125	0.137431	None
3487	FLJ38773	0.067881	0.022132	0.137431	None
3488	SLC15A4	0.307393	0.022136	0.137431	None
3489	HOXB3	-0.42524	0.022143	0.137431	None
3490	GJA4	-0.44854	0.022164	0.137523	None
3491	FAM129B	-0.41188	0.02217	0.137523	None
3492	PTCHD4	0.004924	0.022182	0.13756	None
3493	XXyac-YX1	-0.30815	0.022206	0.137668	None
3494	CRB3	0.124629	0.022221	0.137705	None
3495	LINC00847	-0.22646	0.022225	0.137705	None
3496	SMG6	-0.07894	0.022242	0.137772	None
3497	AP000525.	-0.76392	0.022291	0.138038	Down
3498	RCN2	0.260095	0.022301	0.138057	None
3499	YEATS4	0.53575	0.022358	0.13837	None
3500	MRPL32	-0.29018	0.022364	0.13837	None
3501	LINC00563	0.07698	0.022379	0.138422	None
3502	RP11-396F	-0.14248	0.022398	0.138498	None
3503	CSH1	0.081831	0.022431	0.138665	None
3504	ATP11C	0.123778	0.02249	0.138993	None
3505	XAGE-4	0.389946	0.02253	0.139197	None
3506	LOC10192	0.148381	0.022569	0.139311	None
3507	ZNF721	0.341344	0.022572	0.139311	None
3508	RTN1	-0.63645	0.022581	0.139311	Down
3509	DDN	0.040449	0.022589	0.139311	None
3510	PHLDA1	0.516725	0.02259	0.139311	None
3511	MRPS30	-0.33767	0.022591	0.139311	None
3512	CMAHP	-0.55953	0.022593	0.139311	None
3513	BTBD10	-0.24169	0.02263	0.139499	None
3514	TOP3A	-0.26841	0.022644	0.139546	None
3515	MANEAL	-0.58093	0.022656	0.139552	None
3516	ABHD4	-0.34268	0.022658	0.139552	None
3517	NXNL2	0.059829	0.022687	0.139648	None
3518	MRTO4	-0.40967	0.022687	0.139648	None
3519	RP11-173M	-0.43114	0.022702	0.139705	None
3520	TBK1	-0.29126	0.022712	0.139708	None
3521	HAND2	0.031261	0.022722	0.139708	None
3522	FECH	-0.60335	0.022722	0.139708	Down
3523	RAB42	-0.98621	0.022761	0.139906	Down
3524	AC005256.	0.054538	0.022782	0.139931	None
3525	RPS6KA4	-0.17566	0.022783	0.139931	None
3526	SNX27	-0.26126	0.022784	0.139931	None
3527	EPS15L1	-0.24713	0.022804	0.139985	None
3528	TOMM5	-0.32198	0.022811	0.139985	None
3529	C9orf40	-0.52188	0.022813	0.139985	None
3530	TRIM26	-0.37087	0.022861	0.140241	None
3531	ARHGAP26	0.04617	0.022915	0.140534	None
3532	SARAF	0.27548	0.022926	0.140561	None
3533	LINC00520	0.404949	0.022964	0.140754	None
3534	IL3RA	0.397349	0.022978	0.140802	None
3535	PHEX-AS1	0.040913	0.022988	0.14082	None
3536	CRAT	-0.22557	0.022998	0.140845	None

3537	CMIP	-0.43467	0.023017	0.140888	None
3538	SPEN	-0.43028	0.023018	0.140888	None
3539	ROBO3	-0.41373	0.023054	0.141064	None
3540	PIN1P1	0.158706	0.02308	0.141187	None
3541	PRR15	0.500505	0.023093	0.141227	None
3542	DDX27	0.285296	0.023151	0.141543	None
3543	MEGF6	-0.44421	0.023161	0.14156	None
3544	GCN1L1	-0.28554	0.023193	0.141693	None
3545	RGS14	-0.24808	0.023205	0.141693	None
3546	POLR1E	-0.54553	0.023205	0.141693	None
3547	TCN2	-0.36788	0.023214	0.141693	None
3548	HCG4	-0.79856	0.023215	0.141693	Down
3549	CA3	-0.41901	0.023262	0.141936	None
3550	ZNF165	0.634577	0.023269	0.141939	Up
3551	ARMC1	-0.29189	0.023297	0.142073	None
3552	ARNT2	-0.72053	0.023304	0.142073	Down
3553	STX4	-0.31163	0.02332	0.142132	None
3554	TTLL3	0.157187	0.023405	0.142612	None
3555	CARTPT	0.033864	0.02342	0.142626	None
3556	POLR3H	-0.27465	0.023421	0.142626	None
3557	C6orf106	-0.4455	0.023464	0.142846	None
3558	EGR2	0.830133	0.023514	0.143092	Up
3559	GBP4	0.493579	0.023517	0.143092	None
3560	ECD	-0.27171	0.023555	0.143279	None
3561	ZNF438	-0.24783	0.023585	0.143422	None
3562	PCGF5	-0.52541	0.023632	0.143669	None
3563	LOC10050(	-0.47658	0.023644	0.143693	None
3564	ALG12	-0.20706	0.023649	0.143693	None
3565	ZNF502	0.279939	0.023664	0.143707	None
3566	SAMD15	0.019316	0.023688	0.143707	None
3567	ARID1B	0.266932	0.02369	0.143707	None
3568	RDH13	-0.36684	0.023692	0.143707	None
3569	SLC35G1	-0.30364	0.023694	0.143707	None
3570	GATAD2A	-0.14714	0.023696	0.143707	None
3571	TMC1	0.015543	0.023698	0.143707	None
3572	TTC5	-0.22282	0.023705	0.143707	None
3573	ADNP2	-0.31392	0.023713	0.14372	None
3574	LOC93463	0.050919	0.023736	0.143818	None
3575	LOC15840:	-0.49343	0.023743	0.143818	None
3576	ANKRD18A	-0.50915	0.023757	0.143864	None
3577	B3GNT7	0.338745	0.023784	0.143922	None
3578	HOXB2	-0.52299	0.023786	0.143922	None
3579	ACLY	-0.32731	0.023787	0.143922	None
3580	MRPS33	-0.29785	0.023806	0.14397	None
3581	OXSM	-0.3447	0.023808	0.14397	None
3582	SHOC2	0.263056	0.023833	0.14405	None
3583	HYOU1	-0.67015	0.023834	0.14405	Down
3584	CAPZB	-0.32248	0.023865	0.144195	None
3585	ADIPOR1	-0.49761	0.02391	0.144428	None
3586	CTD-2012I	0.212373	0.023988	0.144858	None
3587	CTSG	1.130764	0.023995	0.144859	Up
3588	DDX11L2	-0.67747	0.024042	0.145105	Down
3589	WASF2	-0.3535	0.02406	0.145168	None
3590	MYC	-0.65463	0.024066	0.145168	Down
3591	DFFA	-0.27697	0.024078	0.145198	None
3592	PPAPDC1A	-0.50073	0.024101	0.145297	None
3593	DIEXF	-0.38992	0.024154	0.145575	None
3594	DISC1	0.014011	0.024165	0.145599	None

3595	SMA4	-0.45051	0.024202	0.145785	None
3596	SH2D2A	0.135043	0.024223	0.145871	None
3597	LOC39949:	-0.39667	0.024233	0.14589	None
3598	MORC4	-0.34135	0.024305	0.146281	None
3599	RASAL3	-0.36114	0.024354	0.146483	None
3600	ID2	0.984543	0.024362	0.146483	Up
3601	TAF9B	0.485298	0.024367	0.146483	None
3602	KYNU	-0.85455	0.024371	0.146483	Down
3603	CST2	0.059791	0.024372	0.146483	None
3604	SLC4A1AP	-0.16127	0.024428	0.146778	None
3605	ARFIP2	-0.34955	0.024449	0.146863	None
3606	C4orf46	0.381416	0.024474	0.146975	None
3607	PPM1B	0.330428	0.02449	0.147031	None
3608	C10orf2	-0.45205	0.024504	0.147069	None
3609	AL132709.8	-0.69984	0.024564	0.147389	Down
3610	NDUFA7	-0.23825	0.024589	0.147501	None
3611	NSMCE1	-0.22577	0.024623	0.147664	None
3612	MRPS26	-0.21663	0.024642	0.147734	None
3613	SPAG9	0.373963	0.024659	0.147796	None
3614	HSF5	-0.68911	0.024672	0.147796	Down
3615	LOC10192(	0.350214	0.024673	0.147796	None
3616	C9orf16	-0.58943	0.024738	0.148144	Down
3617	KRTAP10-1	0.103824	0.024744	0.148144	None
3618	GPR101	0.010029	0.024754	0.148164	None
3619	PLEKHB2	-0.39618	0.02478	0.148275	None
3620	UBASH3A	-0.73822	0.024807	0.148396	Down
3621	NTS	0.085623	0.024835	0.148521	None
3622	LINC00598	0.045713	0.024862	0.148597	None
3623	ZFX	0.224468	0.024862	0.148597	None
3624	CEP85	-0.21339	0.024868	0.148597	None
3625	MUC1	-0.52744	0.024878	0.148618	None
3626	TRAF2	-0.28674	0.024906	0.148743	None
3627	LINC00343	0.006847	0.024923	0.148802	None
3628	RXRA	-0.47804	0.024985	0.149101	None
3629	ACTR1B	-0.22513	0.024987	0.149101	None
3630	RP11-369(	0.044474	0.025016	0.149232	None
3631	CFP	-0.67915	0.02503	0.149276	Down
3632	UBN2	-0.53228	0.02504	0.149294	None
3633	DLGAP4	-0.14549	0.025048	0.149304	None
3634	TMEM45A	-0.89187	0.025079	0.149446	Down
3635	PDCD2L	-0.45561	0.025091	0.149478	None
3636	CR1L	-0.87942	0.02511	0.149545	Down
3637	SERPINA4	0.005903	0.02512	0.149566	None
3638	GBA2	-0.19033	0.025137	0.149624	None
3639	C1orf180	0.049741	0.025147	0.149645	None
3640	GAS5	-0.23965	0.025179	0.14977	None
3641	CHCHD4	-0.41211	0.025182	0.14977	None
3642	LOC10192	-0.14045	0.0252	0.149808	None
3643	RP11-805I:	0.181033	0.025211	0.149808	None
3644	AC018755.	0.014276	0.025216	0.149808	None
3645	LOC34018	0.066629	0.02522	0.149808	None
3646	LOC10050	-0.49357	0.025223	0.149808	None
3647	WDR45	-0.31977	0.025255	0.149959	None
3648	TCEB1	0.259076	0.02529	0.150123	None
3649	PLA2R1	-0.05146	0.025298	0.150129	None
3650	PRR5L	-0.36172	0.02535	0.150401	None
3651	DDA1	-0.27082	0.025363	0.15041	None
3652	CADM3	0.028996	0.025366	0.15041	None

3653	TRIM47	-0.52513	0.02538	0.150454	None
3654	PHF13	-0.42103	0.025408	0.150578	None
3655	ZNF33A	-0.20249	0.025417	0.15059	None
3656	UBE2M	-0.87386	0.025446	0.150695	Down
3657	MRPL20	0.074027	0.025449	0.150695	None
3658	LINC01016	0.038111	0.025457	0.1507	None
3659	LINC00942	0.043558	0.025486	0.150802	None
3660	HOXA4	-0.324	0.025488	0.150802	None
3661	KAT7	-0.44606	0.025547	0.15107	None
3662	PKHD1	0.017688	0.025553	0.15107	None
3663	LOC10272	0.200048	0.025554	0.15107	None
3664	LOC14841	-0.29983	0.025568	0.151082	None
3665	C15orf40	-0.19338	0.025575	0.151082	None
3666	PTGIR	-0.15511	0.025578	0.151082	None
3667	DCAF12	-0.37516	0.025584	0.151082	None
3668	NOTCH2	-0.22166	0.025634	0.151335	None
3669	LOC15896	-0.40598	0.025682	0.151579	None
3670	S100A11	-0.94099	0.025713	0.151718	Down
3671	LAT	-0.62855	0.025726	0.151754	Down
3672	HILS1	0.069479	0.025736	0.151776	None
3673	HPSE	-0.88534	0.025815	0.152199	Down
3674	HCLS1	-0.31116	0.025826	0.1522	None
3675	ZNF785	-0.62606	0.025829	0.1522	Down
3676	PHACTR1	0.403781	0.025852	0.152279	None
3677	PCYT1B	-0.27687	0.025863	0.152279	None
3678	ZNF106	-0.30755	0.02587	0.152279	None
3679	LOC10099	0.628456	0.025871	0.152279	Up
3680	HOXB-AS3	-0.37599	0.025896	0.152362	None
3681	LY6G6C	0.068114	0.025899	0.152362	None
3682	SRCRB4D	-0.24302	0.025907	0.152362	None
3683	KMT2E	0.368426	0.025913	0.152362	None
3684	NCSTN	-0.17831	0.025931	0.152426	None
3685	C2orf44	-0.41372	0.025951	0.152464	None
3686	ZNF415	-0.79033	0.025955	0.152464	Down
3687	DGAT2	-0.3599	0.025965	0.152464	None
3688	LINC00473	0.049815	0.025966	0.152464	None
3689	RAB37	-0.45646	0.025989	0.152533	None
3690	SCAMPER	0.014551	0.025992	0.152533	None
3691	CDC42BPB	-0.24757	0.026025	0.152686	None
3692	FSD1L	0.271962	0.026052	0.152805	None
3693	GRIN2C	0.022857	0.026065	0.152838	None
3694	NIF3L1	-0.41647	0.026083	0.152905	None
3695	LINC00628	0.053407	0.026107	0.152969	None
3696	RGS18	-0.86146	0.026108	0.152969	Down
3697	KLF11	-0.39742	0.026133	0.153071	None
3698	NTNG2	-0.47587	0.02614	0.153071	None
3699	MDN1	0.54222	0.026158	0.153125	None
3700	RP11-248J	0.407323	0.026163	0.153125	None
3701	FYCO1	-0.16893	0.026184	0.153207	None
3702	MT1G	-1.01414	0.026198	0.153244	Down
3703	SMURF2	0.429919	0.026222	0.153304	None
3704	ADD1	-0.17591	0.026222	0.153304	None
3705	RBM12B	-0.42107	0.026233	0.153324	None
3706	NAT1	-0.30142	0.026265	0.153472	None
3707	SIGLEC10	-0.33902	0.026276	0.153493	None
3708	CXorf40B	0.230781	0.026285	0.153505	None
3709	PDK1	-0.35334	0.026302	0.153533	None
3710	ABAT	-0.50553	0.026307	0.153533	None

3711	NOS1AP	0.064039	0.026311	0.153533	None
3712	BNIP2	0.236577	0.026332	0.153616	None
3713	KIAA0922	0.38309	0.026341	0.153629	None
3714	IQGAP1	0.515396	0.026355	0.153664	None
3715	RACGAP1	0.484361	0.026387	0.153814	None
3716	ATG4A	-0.30568	0.026404	0.15387	None
3717	RPL31	-0.51708	0.026419	0.153916	None
3718	GAPDH	0.176426	0.026427	0.153923	None
3719	LINC0112C	0.035482	0.026462	0.154059	None
3720	MALL	-0.32166	0.026472	0.154059	None
3721	HMGN1	0.195174	0.026472	0.154059	None
3722	CIRBP	-0.41625	0.02648	0.154061	None
3723	FASTK	-0.30502	0.026515	0.154224	None
3724	KBTBD12	-0.78934	0.026545	0.154345	Down
3725	LCA5L	0.06404	0.02655	0.154345	None
3726	TASP1	0.223129	0.026559	0.154356	None
3727	CD53	0.543857	0.026591	0.154505	None
3728	TMEM79	-0.18678	0.026617	0.154611	None
3729	PRKAB2	-0.41885	0.02663	0.154613	None
3730	CCNK	-0.11573	0.026632	0.154613	None
3731	SMPDL3B	-0.20136	0.026649	0.154675	None
3732	NPRL2	-0.15715	0.026656	0.154675	None
3733	GS1-166A:	-0.34581	0.026712	0.154955	None
3734	LSM12	-0.28842	0.02674	0.155073	None
3735	CHML	0.137916	0.026771	0.155216	None
3736	PCNP	-0.38293	0.026802	0.155355	None
3737	VANGL2	-0.34004	0.026844	0.155547	None
3738	COPB2	-0.176	0.02685	0.155547	None
3739	THBS3	-0.35909	0.026863	0.15556	None
3740	MAN2B1	-0.42318	0.026876	0.15556	None
3741	AKAP10	-0.36866	0.02688	0.15556	None
3742	RTKN2	0.144594	0.026881	0.15556	None
3743	KLHL14	0.004132	0.026903	0.155649	None
3744	TOMM70A	-0.23226	0.026943	0.155834	None
3745	NDUFV3	-0.32839	0.026953	0.155855	None
3746	TIMP3	-0.52991	0.026988	0.156015	None
3747	GSTM3	-0.57159	0.027003	0.156059	None
3748	TRIM11	-0.2894	0.027011	0.156065	None
3749	LRRN4CL	0.065476	0.027027	0.156101	None
3750	ZC3H4	-0.11487	0.027032	0.156101	None
3751	TTC28	-0.4502	0.027054	0.156185	None
3752	CHPF2	-0.35622	0.027075	0.156268	None
3753	GIMAP1	-0.62311	0.027119	0.156476	Down
3754	SNX5	-0.27719	0.02713	0.156499	None
3755	RANBP3	-0.20355	0.027139	0.156512	None
3756	HSF2BP	0.091352	0.027147	0.156514	None
3757	SBF2-AS1	-0.14922	0.027269	0.157164	None
3758	RHBDF1	-0.24607	0.027274	0.157164	None
3759	ZNF589	-0.28839	0.027298	0.157218	None
3760	FEN1	0.467458	0.027298	0.157218	None
3761	KIAA1549	-0.5272	0.027308	0.157236	None
3762	XPA	-0.30256	0.027341	0.157381	None
3763	RP11-251C	-0.22411	0.027373	0.157522	None
3764	MYD88	-0.24428	0.02738	0.157522	None
3765	BBS4	-0.42453	0.027422	0.157723	None
3766	AKT1	-0.40659	0.027433	0.157746	None
3767	RSPH3	-0.23167	0.027457	0.157841	None
3768	MROH6	-0.34905	0.027516	0.158127	None

3769	DBH	0.027062	0.027522	0.158127	None
3770	CALML3	0.022275	0.027559	0.158299	None
3771	ZNF628	-0.44071	0.027624	0.158603	None
3772	IDS	0.378161	0.027626	0.158603	None
3773	CYTL1	-0.7281	0.027662	0.158766	Down
3774	ISCA2	-0.34638	0.027732	0.159123	None
3775	METTL21EI	0.030619	0.02774	0.15913	None
3776	MRPL13	-0.40843	0.027756	0.159144	None
3777	KIAA0430	-0.15812	0.027757	0.159144	None
3778	LRFN2	0.04377	0.02778	0.159231	None
3779	DBP	-0.25285	0.027823	0.159414	None
3780	S100Z	-0.67141	0.027827	0.159414	Down
3781	ALKBH5	-0.62079	0.027842	0.159458	Down
3782	LOC10192	0.009272	0.027865	0.159458	None
3783	ZNF239	-0.43104	0.027866	0.159458	None
3784	SIAH2	0.403691	0.027869	0.159458	None
3785	SYNCRIP	-0.25182	0.027871	0.159458	None
3786	PITPNM1	-0.18776	0.027961	0.159932	None
3787	LINC00868	0.107749	0.027975	0.159953	None
3788	KDM1B	-0.24462	0.02798	0.159953	None
3789	DKFZP434I	-0.39561	0.028	0.160028	None
3790	TFE3	-0.2039	0.028012	0.160052	None
3791	CCDC43	-0.23949	0.028062	0.160295	None
3792	ICT1	-0.34839	0.028069	0.160295	None
3793	GSDMC	0.060118	0.028087	0.160357	None
3794	ZBTB37	-0.16634	0.028109	0.160427	None
3795	TNFRSF25	-0.27737	0.02812	0.160427	None
3796	GGA2	-0.33643	0.028122	0.160427	None
3797	BAIAP2-AS	-0.32436	0.028136	0.160467	None
3798	DAPP1	-0.57457	0.028222	0.160911	None
3799	LINC01442	0.035356	0.028241	0.160951	None
3800	KRTAP1-1	0.034312	0.028254	0.160951	None
3801	B3GALNT2	0.092975	0.028255	0.160951	None
3802	NIPAL2	0.109027	0.028258	0.160951	None
3803	HNRNPA3	0.215038	0.028314	0.161224	None
3804	PPP1R37	0.038956	0.028323	0.161235	None
3805	LOC10099	0.088352	0.028367	0.161445	None
3806	CYFIP2	0.538846	0.028379	0.161471	None
3807	C8B	0.009986	0.028441	0.161776	None
3808	CYP4F12	0.477986	0.028455	0.161816	None
3809	FBXO42	-0.20426	0.028496	0.162003	None
3810	RP11-432J	0.105165	0.028529	0.162125	None
3811	DPM2	-0.34339	0.028535	0.162125	None
3812	FGD4	0.320043	0.028549	0.162125	None
3813	ANXA3	-0.85233	0.02855	0.162125	Down
3814	PRR3	-0.49606	0.028554	0.162125	None
3815	LSM1	-0.28183	0.028572	0.162183	None
3816	CALB2	0.066942	0.028612	0.16233	None
3817	ATP11B	0.264707	0.028613	0.16233	None
3818	CMYA5	0.062925	0.028668	0.162601	None
3819	RFWD2	-0.26827	0.028686	0.162658	None
3820	TH	0.028933	0.028703	0.162705	None
3821	ZNF615	-0.314	0.028717	0.162705	None
3822	TDP2	-0.24737	0.028721	0.162705	None
3823	ZNF696	-0.19131	0.028724	0.162705	None
3824	TYW5	0.284548	0.028732	0.162706	None
3825	GAK	-0.2056	0.028745	0.16274	None
3826	NKIRAS1	-0.41583	0.028775	0.162846	None

3827	ADRA2A	-0.79402	0.028779	0.162846	Down
3828	HOMER2	-0.31026	0.028805	0.162926	None
3829	RP11-1094	0.325397	0.028808	0.162926	None
3830	ABCF3	-0.34146	0.028845	0.163088	None
3831	C10orf95	-0.19703	0.028864	0.163154	None
3832	LUZP2	0.086175	0.028872	0.163161	None
3833	FOSL2	0.829315	0.028916	0.163362	Up
3834	RP11-226L	-0.47534	0.028933	0.163417	None
3835	TMEM55A	-0.46891	0.02894	0.163417	None
3836	ST3GAL4	-0.16347	0.028994	0.163676	None
3837	LINC00263	-0.1913	0.02902	0.163708	None
3838	SLC25A39	-0.69428	0.029023	0.163708	Down
3839	ARHGEF39	-0.15054	0.029025	0.163708	None
3840	TIA1	0.264483	0.029037	0.163708	None
3841	GNS	-0.61567	0.029037	0.163708	Down
3842	ZNF587B	0.337688	0.029045	0.163708	None
3843	GPR63	-0.23201	0.029072	0.163819	None
3844	FXYD6	-0.96545	0.029087	0.16386	Down
3845	SLC25A5	0.202559	0.029106	0.163925	None
3846	NRIP1	0.484306	0.029158	0.164173	None
3847	C10orf88	-0.30828	0.029182	0.164238	None
3848	CABIN1	-0.35623	0.029187	0.164238	None
3849	HIST1H2BH	-0.66737	0.0292	0.164238	Down
3850	LINC00954	-0.43926	0.029201	0.164238	None
3851	IQSEC3	0.020519	0.029207	0.164238	None
3852	MEA1	-0.32337	0.029219	0.164261	None
3853	CYP2R1	-0.32556	0.029228	0.164268	None
3854	LGALS8	-0.31208	0.029245	0.164322	None
3855	KIF3B	-0.21442	0.02933	0.164757	None
3856	LOC101928	-0.28269	0.02938	0.164998	None
3857	C9orf156	0.413614	0.029389	0.165	None
3858	CFLAR	-0.34803	0.029396	0.165	None
3859	C1orf61	0.074154	0.029417	0.165045	None
3860	TERF2IP	-0.23817	0.029419	0.165045	None
3861	EVL	-0.33414	0.029454	0.165184	None
3862	DNAAF1	0.123279	0.029466	0.165184	None
3863	PTPLAD2	-0.15931	0.029467	0.165184	None
3864	HCG11	-0.50134	0.029475	0.165184	None
3865	CDK5RAP2	-0.23987	0.029515	0.165368	None
3866	KCTD15	-0.28387	0.029528	0.165399	None
3867	EIF2AK1	-0.31179	0.029541	0.165428	None
3868	FITM2	0.202392	0.029587	0.165622	None
3869	YIPF3	-0.46059	0.029592	0.165622	None
3870	AMPD2	-0.2839	0.029602	0.165622	None
3871	MCOLN1	-0.84122	0.029606	0.165622	Down
3872	CEL	-0.18053	0.029643	0.165786	None
3873	IRF8	1.066677	0.029735	0.166255	Up
3874	FLJ33544	0.052292	0.029742	0.166255	None
3875	ZC3H14	-0.27124	0.029857	0.16681	None
3876	OR3A3	0.047064	0.029857	0.16681	None
3877	MFHAS1	-0.50775	0.029886	0.166926	None
3878	FCHSD2	-0.2305	0.029911	0.166993	None
3879	FCHO2	-0.51237	0.029913	0.166993	None
3880	REST	0.226172	0.02994	0.167102	None
3881	ZZZ3	-0.30312	0.029971	0.16721	None
3882	CHCHD6	-0.37939	0.029984	0.16721	None
3883	PPRC1	-0.41503	0.029989	0.16721	None
3884	NELL1	0.064326	0.029991	0.16721	None

3885	CRY2	-0.16067	0.030029	0.16738	None
3886	MITD1	-0.43225	0.030048	0.167447	None
3887	PDS5B	0.226597	0.030073	0.167542	None
3888	ST3GAL1	0.357136	0.030088	0.16758	None
3889	NAV1	0.412349	0.030112	0.167634	None
3890	MBD5	-0.25952	0.030113	0.167634	None
3891	C16orf58	-0.40658	0.030124	0.16765	None
3892	CENPU	0.507756	0.03018	0.16792	None
3893	DRD2	0.054339	0.030188	0.16792	None
3894	PROKR2	0.030086	0.030205	0.167971	None
3895	LINC00294	-0.34848	0.030232	0.16808	None
3896	KIF11	0.55239	0.03024	0.168084	None
3897	FEM1A	-0.15543	0.030355	0.168675	None
3898	FUT1	-0.63509	0.030481	0.169334	Down
3899	LOC10192	0.028447	0.030543	0.169549	None
3900	CXorf38	-0.29575	0.030545	0.169549	None
3901	ACER3	-0.33292	0.03055	0.169549	None
3902	RBP7	-1.07572	0.030551	0.169549	Down
3903	RABL6	-0.45587	0.030576	0.169647	None
3904	LOC25715	-0.29228	0.030606	0.169769	None
3905	UBE2Q2L	-0.22771	0.030637	0.169894	None
3906	HYAL2	-0.39777	0.030707	0.170209	None
3907	LOC43991	0.428951	0.030709	0.170209	None
3908	TINF2	0.199567	0.030722	0.170234	None
3909	NQO2	-0.30958	0.030784	0.170536	None
3910	FNDC1	0.053369	0.030812	0.170649	None
3911	C12orf60	-0.21431	0.030884	0.171003	None
3912	ADAM17	0.260562	0.030932	0.171225	None
3913	ARL6IP6	0.33205	0.030943	0.171244	None
3914	PUSL1	-0.41315	0.030959	0.171272	None
3915	TEX14	0.256867	0.030964	0.171272	None
3916	C17orf75	0.321597	0.030974	0.171283	None
3917	ASB16-AS	-0.3831	0.031057	0.171654	None
3918	ZNF10	0.331371	0.031057	0.171654	None
3919	C10orf35	-0.45573	0.031117	0.171942	None
3920	SLC20A1	0.331293	0.031131	0.171972	None
3921	SF3B3	-0.22917	0.031196	0.172278	None
3922	LOC28514	-0.22439	0.031202	0.172278	None
3923	STX7	-0.31408	0.031214	0.172299	None
3924	FBXO28	0.263269	0.031226	0.172324	None
3925	MATN1-A	-0.24328	0.031251	0.172419	None
3926	FUOM	-0.33376	0.031293	0.172595	None
3927	INO80B	-0.19642	0.031299	0.172595	None
3928	PIK3C2B	0.541467	0.031329	0.172714	None
3929	VAT1L	0.227099	0.031341	0.172736	None
3930	AP3B2	0.061499	0.031354	0.172766	None
3931	DHCR24	-0.65385	0.031404	0.172997	Down
3932	RP11-488L	0.556646	0.03142	0.17304	None
3933	CBX7	-0.39612	0.031434	0.173073	None
3934	FAM114A1	-0.32417	0.031443	0.17308	None
3935	RP11-53B2	0.112441	0.031465	0.173141	None
3936	ZNF516	-0.5471	0.031475	0.173141	None
3937	ATG9A	-0.3566	0.031478	0.173141	None
3938	RUSC2	-0.15936	0.031524	0.173325	None
3939	CASC17	0.009792	0.031527	0.173325	None
3940	C8orf59	0.356215	0.031575	0.173542	None
3941	ST3GAL6	0.345033	0.031614	0.173653	None
3942	LOC10050	-0.43477	0.031627	0.173653	None

3943	MAP7	-0.37913	0.031629	0.173653	None
3944	PATE1	0.060384	0.031633	0.173653	None
3945	GAB3	-0.45327	0.031635	0.173653	None
3946	MYH7B	0.047627	0.031738	0.174143	None
3947	BYSL	-0.32407	0.031741	0.174143	None
3948	UCHL1	-0.53331	0.031784	0.174337	None
3949	ARFRP1	-0.19311	0.031828	0.174535	None
3950	SQSTM1	-0.4393	0.03186	0.174667	None
3951	PHC2	-0.16041	0.031903	0.174857	None
3952	ATOX1	-0.56034	0.031921	0.174894	None
3953	TMEM14C	-0.35564	0.031926	0.174894	None
3954	IGF1R	-0.38961	0.031944	0.174949	None
3955	C17orf59	-0.20094	0.032053	0.175501	None
3956	AC007389.	0.209291	0.032066	0.175528	None
3957	SH3BP4	0.338287	0.032121	0.175786	None
3958	MPV17L2	-0.14798	0.032157	0.175903	None
3959	MTF1	-0.25304	0.032159	0.175903	None
3960	C15orf39	-0.36463	0.032215	0.176131	None
3961	ZNF518B	-0.24625	0.032222	0.176131	None
3962	AX747826	0.751068	0.032225	0.176131	Up
3963	TMEM184E	-0.28797	0.032234	0.176136	None
3964	APOF	0.022986	0.032264	0.176244	None
3965	LINC01361	0.021331	0.03227	0.176244	None
3966	SOD1	-0.22462	0.03228	0.176254	None
3967	RARS	-0.22169	0.032294	0.176287	None
3968	SOX12	-0.2089	0.032346	0.176521	None
3969	GUCA1B	-0.16252	0.032353	0.176521	None
3970	RAB5B	-0.33835	0.032388	0.176648	None
3971	SMG9	-0.27014	0.032393	0.176648	None
3972	TGM6	0.054568	0.032444	0.17688	None
3973	MIIP	-0.27062	0.032474	0.176976	None
3974	POU3F1	0.079313	0.032487	0.176976	None
3975	SECTM1	-0.44876	0.032492	0.176976	None
3976	TMEM165	-0.19954	0.032494	0.176976	None
3977	TCEAL2	1.028575	0.03251	0.176995	Up
3978	C4orf33	0.076725	0.032514	0.176995	None
3979	PRRX2-AS:	0.047527	0.032536	0.177074	None
3980	PLEKHM2	-0.2958	0.032654	0.177654	None
3981	ZBTB6	0.078939	0.032659	0.177654	None
3982	C21orf58	-0.08502	0.032692	0.177781	None
3983	FAN1	-0.30306	0.032699	0.177781	None
3984	CCDC132	-0.31848	0.032708	0.177782	None
3985	ONECUT1	0.018538	0.032769	0.178069	None
3986	NUDT11	0.851597	0.032787	0.178121	Up
3987	C9orf142	-0.2569	0.032797	0.178132	None
3988	CD34	-0.62492	0.032843	0.178316	Down
3989	THUMPD1	0.327803	0.032849	0.178316	None
3990	TMEM159	-0.49909	0.032855	0.178316	None
3991	OTUD5	-0.26696	0.032875	0.178381	None
3992	COX7C	-0.34178	0.032909	0.178473	None
3993	PHGDH	0.681984	0.03291	0.178473	Up
3994	FTCD	0.017177	0.032928	0.178473	None
3995	FTSJ2	-0.32357	0.032936	0.178473	None
3996	GRINA	-0.40127	0.032941	0.178473	None
3997	STK25	-0.31372	0.032942	0.178473	None
3998	LDLRAD3	-0.69144	0.032953	0.178484	Down
3999	GAA	-0.55487	0.03296	0.178484	None
4000	MSN	-0.34812	0.032976	0.178517	None

4001	MRPL37	-0.33193	0.032984	0.178517	None
4002	GRIN2B	0.00645	0.032996	0.178517	None
4003	CYTIP	0.563632	0.033012	0.178517	None
4004	IGFBP2	-0.77015	0.033014	0.178517	Down
4005	RTDR1	0.035111	0.033022	0.178517	None
4006	BMP2	0.324732	0.033024	0.178517	None
4007	SRD5A1	-0.28992	0.033037	0.178543	None
4008	SFSWAP	-0.27694	0.033076	0.178706	None
4009	ENGASE	-0.38315	0.033105	0.178819	None
4010	KIF13B	-0.30392	0.033123	0.178865	None
4011	MOGAT1	0.048856	0.03313	0.178865	None
4012	COLGALT1	-0.23436	0.033147	0.17891	None
4013	CLEC12A	0.346693	0.033208	0.179195	None
4014	KIAA1432	0.192681	0.033243	0.179343	None
4015	LST1	-0.60602	0.03329	0.179517	Down
4016	MOB3C	-0.26825	0.033304	0.179517	None
4017	CARD16	-0.32723	0.033305	0.179517	None
4018	STX12	-0.34789	0.033309	0.179517	None
4019	XIRP2	-0.56967	0.033335	0.179567	None
4020	LOC101928	0.126671	0.033335	0.179567	None
4021	DCAF16	-0.42658	0.033356	0.179592	None
4022	KCTD9	0.339476	0.033356	0.179592	None
4023	PIPOX	-0.49527	0.033484	0.18024	None
4024	OTUD6B-A	0.281897	0.033515	0.180316	None
4025	LYRM5	-0.31226	0.033515	0.180316	None
4026	TRAF5	0.221084	0.033601	0.180732	None
4027	GEN1	0.394273	0.033617	0.180775	None
4028	BIRC7	0.235041	0.033739	0.181386	None
4029	KIF15	0.60802	0.033765	0.18148	Up
4030	KIAA0586	-0.28092	0.033774	0.181483	None
4031	ZC3H15	-0.30788	0.033807	0.181618	None
4032	NSMAF	0.468694	0.033845	0.181774	None
4033	RAB33A	0.613757	0.033887	0.181933	Up
4034	GINS3	0.409001	0.033907	0.181933	None
4035	1-Mar	-0.78185	0.033915	0.181933	Down
4036	TRAPPC1	-0.50014	0.033918	0.181933	None
4037	LINC01254	0.037529	0.033924	0.181933	None
4038	ADGB	0.010569	0.033925	0.181933	None
4039	CASP4	-0.36372	0.033954	0.182041	None
4040	KCNIP2-AS	0.193919	0.033976	0.182086	None
4041	DYNLL1-A	-0.10135	0.033979	0.182086	None
4042	CLASP2	-0.29736	0.034014	0.182184	None
4043	CX3CR1	-0.48203	0.034014	0.182184	None
4044	BC047484	0.295478	0.034057	0.182297	None
4045	ACSBG1	-0.29594	0.034058	0.182297	None
4046	CYB561	-0.27161	0.03406	0.182297	None
4047	ZDHHC8	-0.08311	0.034079	0.182353	None
4048	DDX6	0.725963	0.034104	0.182361	Up
4049	PRPF31	-0.45732	0.034104	0.182361	None
4050	PET112	-0.30613	0.034106	0.182361	None
4051	FAXDC2	-0.58715	0.034131	0.18245	Down
4052	BLVRB	-0.76335	0.034186	0.1827	Down
4053	LOC10272	-0.33784	0.034211	0.182785	None
4054	ZNF766	-0.23514	0.03424	0.182896	None
4055	CCDC6	-0.2182	0.034254	0.182929	None
4056	TMEM212	0.13147	0.034304	0.183149	None
4057	OR6A2	0.003713	0.034314	0.183155	None
4058	PM20D2	0.58234	0.034347	0.183272	None

4059	CCDC85C	-0.13344	0.034352	0.183272	None
4060	FBXL19	-0.20329	0.034377	0.18335	None
4061	SORBS1	-0.45448	0.034384	0.18335	None
4062	MANBA	-0.27115	0.034414	0.183466	None
4063	EIF3L	0.123163	0.034435	0.1835	None
4064	PPM1A	0.248913	0.034438	0.1835	None
4065	SAP18	0.373378	0.034464	0.183599	None
4066	WDR46	-0.32359	0.034474	0.183605	None
4067	CEP170B	0.096746	0.034508	0.183742	None
4068	KIAA1467	0.049749	0.034524	0.183783	None
4069	NT5C	-0.32245	0.034552	0.183847	None
4070	DCTN5	-0.39897	0.034554	0.183847	None
4071	LOC10192[illegible]	0.066801	0.034573	0.183877	None
4072	RPA3OS	0.371299	0.034576	0.183877	None
4073	SNX2	0.373576	0.034634	0.184139	None
4074	ZNF517	0.033234	0.034645	0.184155	None
4075	ARHGAP1[illegible]	-0.20663	0.034666	0.184216	None
4076	CSDE1	-0.21307	0.034694	0.18432	None
4077	NCOA7	-0.20568	0.034706	0.184339	None
4078	INA	0.06592	0.03475	0.184497	None
4079	RGS2	0.589969	0.034753	0.184497	Up
4080	ATHL1	-0.32974	0.034766	0.184497	None
4081	ERVW-1	0.087909	0.034777	0.184497	None
4082	SMARCC1	-0.43099	0.034793	0.184497	None
4083	CASKIN2	-0.23407	0.034794	0.184497	None
4084	RXFP1	-0.72339	0.034795	0.184497	Down
4085	PAPD7	0.334958	0.034842	0.184703	None
4086	URM1	-0.15922	0.034866	0.184781	None
4087	LOC10192[illegible]	-0.31478	0.034887	0.18482	None
4088	LY6G5C	-0.4477	0.03489	0.18482	None
4089	MCAM	0.1626	0.034962	0.185068	None
4090	RRM2	0.565247	0.034963	0.185068	None
4091	PTGDS	0.167754	0.034963	0.185068	None
4092	C20orf27	-0.36387	0.035004	0.18524	None
4093	TMEM169	0.042235	0.035067	0.185488	None
4094	LOC64601[illegible]	0.529035	0.035068	0.185488	None
4095	NTN1	-0.13961	0.035086	0.18554	None
4096	SMYD5	-0.37758	0.035097	0.185554	None
4097	SLC25A37	-0.6712	0.035114	0.185597	Down
4098	RPUSD1	-0.21621	0.035132	0.185645	None
4099	CTPS1	0.439534	0.035165	0.185779	None
4100	MKI67	0.581112	0.03522	0.18602	None
4101	MAP9	0.399947	0.035241	0.186045	None
4102	SCOC-AS1	-0.12639	0.035242	0.186045	None
4103	PTPN3	0.025036	0.035256	0.186067	None
4104	TNRC6A	-0.29355	0.035263	0.186067	None
4105	ZNF276	-0.18775	0.035282	0.186124	None
4106	NPW	-0.57935	0.035297	0.186145	None
4107	AMZ1	0.077943	0.035304	0.186145	None
4108	DDX39A	0.287513	0.035349	0.186341	None
4109	ITSN2	-0.32664	0.035365	0.186378	None
4110	RP11-629[illegible]	0.061071	0.035391	0.186432	None
4111	PAM16	-0.37944	0.035392	0.186432	None
4112	HSPB6	0.107628	0.035438	0.186558	None
4113	ZNF134	0.332113	0.03544	0.186558	None
4114	UBALD2	-0.27179	0.035442	0.186558	None
4115	SLC4A2	-0.50641	0.035467	0.186641	None
4116	KRIT1	-0.39509	0.035519	0.186873	None

4117	LINC0056C	0.017343	0.035567	0.187057	None
4118	LOC10192{	0.068124	0.035572	0.187057	None
4119	ZNF786	-0.4596	0.035662	0.187487	None
4120	AL833181	0.722656	0.035699	0.187637	Up
4121	JADE3	0.275826	0.035717	0.187683	None
4122	LOC72848!	-0.35043	0.035733	0.187722	None
4123	BNC1	0.021474	0.035762	0.187829	None
4124	ZNF662	-0.48188	0.035799	0.187979	None
4125	COA4	-0.21238	0.035833	0.188114	None
4126	RP11-326I.	-0.26785	0.03586	0.188186	None
4127	DCPS	-0.37857	0.035871	0.188186	None
4128	TAF7	0.302283	0.035873	0.188186	None
4129	SLC35A4	-0.42879	0.035899	0.188274	None
4130	POMGNT2	-0.46023	0.035915	0.188313	None
4131	RP11-731J	0.008713	0.035927	0.188331	None
4132	ATP5D	-0.39831	0.035936	0.188332	None
4133	DICER1-AS	-0.14156	0.035957	0.188365	None
4134	GRN	-0.5005	0.035959	0.188365	None
4135	SMC2	0.350861	0.035993	0.188489	None
4136	ERG	0.43228	0.036001	0.188489	None
4137	GALK2	-0.4071	0.036066	0.188787	None
4138	PALB2	-0.33547	0.036088	0.188836	None
4139	OR52A1	0.015503	0.036093	0.188836	None
4140	SLITRK4	-0.69876	0.036129	0.188932	Down
4141	PARD3	-0.55236	0.036129	0.188932	None
4142	ANK1	-0.74108	0.036152	0.189006	Down
4143	FHL3	-0.23192	0.036204	0.189234	None
4144	C12orf66	-0.39175	0.036226	0.189275	None
4145	AP006222.	-0.24956	0.036229	0.189275	None
4146	POU4F2	0.010292	0.036305	0.189624	None
4147	ATXN1L	0.216196	0.036351	0.189806	None
4148	ATP6V1A	-0.31263	0.036357	0.189806	None
4149	SUPT5H	-0.23151	0.036384	0.1899	None
4150	SUPT6H	-0.31783	0.036415	0.189981	None
4151	LOC10192!	0.017734	0.036417	0.189981	None
4152	RND2	0.060016	0.036465	0.19015	None
4153	TMEM150/	-0.34683	0.036471	0.19015	None
4154	DUSP7	-0.1789	0.036476	0.19015	None
4155	ITIH5	0.051944	0.036486	0.19016	None
4156	HCG18	0.213439	0.036501	0.190189	None
4157	MPDU1	-0.33622	0.036522	0.190252	None
4158	IGSF11-AS	0.006002	0.036622	0.190661	None
4159	ALAS1	-0.31176	0.036623	0.190661	None
4160	SPECC1L	-0.2829	0.036627	0.190661	None
4161	RHBDL2	0.022916	0.03665	0.190738	None
4162	MAMDC2	-1.24164	0.036681	0.190809	Down
4163	RAP2B	-0.49656	0.036682	0.190809	None
4164	LOC64535!	0.03179	0.036726	0.190997	None
4165	TRUB1	0.065075	0.036736	0.191003	None
4166	BAP1	-0.23437	0.036795	0.19126	None
4167	ARL8A	-0.21705	0.036816	0.191326	None
4168	PGM3	-0.33574	0.036867	0.191535	None
4169	SNX30	0.351499	0.036883	0.191535	None
4170	TOR3A	0.216163	0.036894	0.191535	None
4171	SEC24B	0.262016	0.036899	0.191535	None
4172	LOC10028	-0.14214	0.036907	0.191535	None
4173	AKAP5	0.08717	0.036917	0.191535	None
4174	CORO6	0.198117	0.036918	0.191535	None

4175	VPS4A	-0.20985	0.036956	0.191647	None
4176	RP11-355E	0.530142	0.036965	0.191647	None
4177	MCHR2	0.078831	0.036974	0.191647	None
4178	VGF	0.070514	0.036981	0.191647	None
4179	CD69	0.723579	0.036998	0.191647	Up
4180	H2BFS	-0.81135	0.037001	0.191647	Down
4181	LETMD1	-0.32362	0.037002	0.191647	None
4182	ZC3H3	-0.14967	0.037011	0.191647	None
4183	ERVV-1	0.064842	0.037036	0.191731	None
4184	GXYLT1	-0.30312	0.037077	0.1919	None
4185	PEAR1	-0.56446	0.037105	0.191999	None
4186	PYGO2	-0.09027	0.037121	0.192036	None
4187	C3orf70	-0.3069	0.037196	0.192375	None
4188	COQ9	-0.17927	0.037254	0.192631	None
4189	TCF20	0.122663	0.037289	0.192763	None
4190	MYO15A	0.084999	0.037319	0.192872	None
4191	NDUFS4	-0.25443	0.037369	0.193085	None
4192	ZNF311	-0.20124	0.037387	0.193095	None
4193	ENOSF1	-0.29797	0.037389	0.193095	None
4194	ENOX2	-0.21507	0.037411	0.193165	None
4195	UBE3D	0.266275	0.03749	0.193526	None
4196	SUPT16H	0.31613	0.037518	0.193611	None
4197	ABHD3	0.277195	0.037524	0.193611	None
4198	SERTAD4	0.005299	0.037561	0.193705	None
4199	MTURN	-0.53811	0.037563	0.193705	None
4200	RASL11B	0.03059	0.037569	0.193705	None
4201	SEMA4B	-0.30573	0.037626	0.193954	None
4202	LOC10192	0.034092	0.037674	0.194151	None
4203	DCAF10	-0.27227	0.037697	0.194224	None
4204	NFAT5	-0.47865	0.037742	0.194412	None
4205	FAAH	0.444999	0.037778	0.194549	None
4206	LGALS13	0.056559	0.037866	0.194959	None
4207	CCS	-0.36742	0.037876	0.194961	None
4208	SPTSSB	0.059792	0.037933	0.19521	None
4209	XIAP	-0.41849	0.037954	0.195272	None
4210	ZNF749	0.16435	0.037966	0.195285	None
4211	LOC10192	-0.31398	0.037986	0.195311	None
4212	NFATC3	-0.19005	0.037989	0.195311	None
4213	RANBP6	0.307078	0.038	0.19532	None
4214	CCDC149	-0.14902	0.038028	0.195417	None
4215	RP11-304	0.047808	0.038062	0.195549	None
4216	PDCD4	0.68009	0.038086	0.195605	Up
4217	COMMD5	-0.16735	0.038091	0.195605	None
4218	GTF2A1	-0.18351	0.038109	0.195651	None
4219	LOC10192	-0.43443	0.038139	0.195716	None
4220	LOC10050	0.262859	0.03814	0.195716	None
4221	RAD9A	-0.26663	0.038156	0.195753	None
4222	TSC2	-0.23429	0.038174	0.195796	None
4223	PAM	0.448337	0.038212	0.195946	None
4224	SUN2	0.384519	0.038233	0.196008	None
4225	DPY19L4	-0.36488	0.038246	0.196027	None
4226	AMICA1	0.700749	0.038309	0.196303	Up
4227	SBNO1	-0.33795	0.038356	0.196497	None
4228	RCAN3	0.499939	0.038446	0.196914	None
4229	LTBP3	-0.30333	0.038461	0.196941	None
4230	POU3F2	0.030281	0.038479	0.196959	None
4231	THAP9-AS	0.274098	0.038484	0.196959	None
4232	OSBPL6	-0.57609	0.0385	0.196959	None

4233	NME4	-0.41658	0.038501	0.196959	None
4234	OAF	-0.17782	0.03861	0.197389	None
4235	TMPRSS5	0.058995	0.038631	0.197389	None
4236	MAP3K6	0.009921	0.038635	0.197389	None
4237	ARNTL	-0.26167	0.038635	0.197389	None
4238	PARS2	-0.36067	0.038638	0.197389	None
4239	LOC10192	0.052819	0.038644	0.197389	None
4240	PCDHGA4	-0.58539	0.038648	0.197389	Down
4241	MANSC1	-0.61534	0.038668	0.197444	Down
4242	ADORA1	0.074637	0.038682	0.197467	None
4243	ST14	-0.46787	0.038716	0.197595	None
4244	SPATC1L	-0.5485	0.038734	0.197641	None
4245	EXOSC10	-0.12627	0.038774	0.197795	None
4246	HMGB2	0.103205	0.038804	0.197907	None
4247	ZNF675	0.232345	0.038816	0.197918	None
4248	HBBP1	-0.64345	0.038855	0.198069	Down
4249	ZNF582	0.165806	0.038865	0.198076	None
4250	TRANK1	-0.34875	0.038889	0.198119	None
4251	PER2	0.189238	0.038892	0.198119	None
4252	TRIM13	-0.34567	0.038995	0.198599	None
4253	MPLKIP	-0.21755	0.039047	0.198816	None
4254	FAM193B	-0.26126	0.039116	0.199121	None
4255	CREB3L2	-0.29243	0.039152	0.199255	None
4256	RAB32	-0.28807	0.039172	0.199288	None
4257	URGCP	-0.18306	0.039177	0.199288	None
4258	C12orf29	-0.26044	0.039232	0.199522	None
4259	GPR155	-0.40061	0.039248	0.199552	None
4260	NFAM1	-0.22121	0.039256	0.199552	None
4261	LRRC37A3	0.246012	0.039319	0.199823	None
4262	TRPC7	0.006747	0.039368	0.200027	None
4263	RNPEPL1	-0.13637	0.039412	0.200183	None
4264	SLAIN1	0.73198	0.039417	0.200183	Up
4265	C1orf174	-0.33587	0.039479	0.200435	None
4266	BIRC2	0.232657	0.039485	0.200435	None
4267	FANCG	0.303348	0.039543	0.200682	None
4268	MED23	0.35219	0.039564	0.200742	None
4269	TOP2A	0.59469	0.039658	0.201168	Up
4270	SPTLC2	-0.32828	0.039699	0.201329	None
4271	R3HDM2	-0.2295	0.039736	0.201471	None
4272	RRAGC	-0.26788	0.039825	0.201875	None
4273	GPRC5A	0.005447	0.039836	0.201883	None
4274	SLC7A8	-0.56394	0.039883	0.202072	None
4275	GPR111	0.007479	0.039892	0.202072	None
4276	BTBD19	0.188331	0.039944	0.202241	None
4277	PIP4K2B	-0.1493	0.039944	0.202241	None
4278	NPIPB15	0.305936	0.039957	0.202258	None
4279	RHAG	-0.77631	0.039978	0.20232	Down
4280	LOC10192	0.015591	0.040001	0.202388	None
4281	LOC44120	-0.17888	0.040037	0.202516	None
4282	METTL1	-0.32822	0.040045	0.202516	None
4283	NIFK-AS1	-0.25937	0.040081	0.202652	None
4284	CINP	-0.32216	0.040119	0.202797	None
4285	SPATA32	0.115872	0.040151	0.202904	None
4286	MDH2	-0.25682	0.040159	0.202904	None
4287	PPARGC1B	-0.18104	0.040178	0.202954	None
4288	HDHD1	-0.47074	0.040191	0.202954	None
4289	KLHL20	0.202645	0.040198	0.202954	None
4290	ZNF575	-0.165	0.040207	0.202954	None

4291	HDAC10	0.119187	0.040224	0.202961	None
4292	AK021537	0.118884	0.040227	0.202961	None
4293	C16orf95	0.233531	0.0403	0.203242	None
4294	DCAF17	-0.31819	0.040301	0.203242	None
4295	C22orf31	0.09393	0.040319	0.203247	None
4296	PIK3R3	-0.38168	0.040321	0.203247	None
4297	RP11-84D1	0.043559	0.040342	0.203307	None
4298	DNM1L	-0.26185	0.040361	0.203353	None
4299	RSBN1L	-0.22189	0.040375	0.203379	None
4300	STRIP2	-0.3078	0.040405	0.203433	None
4301	MCMBP	0.339495	0.040411	0.203433	None
4302	LOC28635	0.048509	0.040414	0.203433	None
4303	PGBD1	-0.34958	0.040432	0.203467	None
4304	CD248	0.104871	0.04044	0.203467	None
4305	FNDC3A	-0.21797	0.040456	0.2035	None
4306	FBXO11	0.312347	0.040516	0.203757	None
4307	SETMAR	-0.32932	0.040549	0.203873	None
4308	PDE3B	-0.55297	0.040576	0.203961	None
4309	ZSCAN16-	-0.21876	0.040597	0.203987	None
4310	G6PD	-0.46946	0.0406	0.203987	None
4311	TP53I13	-0.3429	0.040624	0.204031	None
4312	PTPN18	-0.36436	0.040627	0.204031	None
4313	ATP2B4	-0.40004	0.040649	0.20407	None
4314	SLC25A40	0.231634	0.040654	0.20407	None
4315	C10orf40	0.010065	0.040677	0.20414	None
4316	TONSL	-0.11274	0.040718	0.204283	None
4317	CDH19	0.009598	0.040727	0.204283	None
4318	TERF2	0.249361	0.040741	0.204283	None
4319	SAMD9	-0.35322	0.040752	0.204283	None
4320	SNX29P2	0.193844	0.04076	0.204283	None
4321	LOC10272	-0.49791	0.040762	0.204283	None
4322	MKNK1	-0.41002	0.040778	0.204313	None
4323	PAN2	-0.30624	0.040793	0.204344	None
4324	TMEM133	-0.45885	0.040866	0.204661	None
4325	HIGD1B	0.038363	0.040895	0.204757	None
4326	RAB21	0.257792	0.040914	0.204759	None
4327	TBC1D4	-0.6176	0.04092	0.204759	Down
4328	TMEM175	-0.20008	0.040923	0.204759	None
4329	RP11-111M	-0.53584	0.040952	0.204855	None
4330	ALAD	-0.48549	0.040962	0.204855	None
4331	FAM171A1	0.944234	0.040977	0.204884	Up
4332	SLC15A2	-0.65546	0.041	0.204954	Down
4333	CADM2	0.012566	0.041093	0.20537	None
4334	RBM4	0.512594	0.041118	0.20545	None
4335	SMARCA2	-0.49947	0.041146	0.205539	None
4336	POLG2	-0.29076	0.041197	0.205746	None
4337	RBBP5	-0.42407	0.041221	0.205821	None
4338	INPP4A	-0.20752	0.041238	0.205847	None
4339	CGA	0.020034	0.041245	0.205847	None
4340	ADAMTS12	0.05776	0.041272	0.205932	None
4341	SHBG	0.085003	0.041283	0.20594	None
4342	FAM78B	0.030456	0.04136	0.206276	None
4343	PRAMEF10	0.033433	0.041378	0.206316	None
4344	TMEM99	-0.5223	0.041422	0.206396	None
4345	LSM10	-0.33832	0.041423	0.206396	None
4346	UBP1	-0.24497	0.041423	0.206396	None
4347	PDCD5	-0.34838	0.041432	0.206396	None
4348	ZNF823	-0.39321	0.041461	0.20648	None

4349	CAV2	-0.7314	0.041471	0.20648	Down
4350	PPA1	-0.2762	0.041477	0.20648	None
4351	MESP1	0.094582	0.041497	0.206532	None
4352	MRPL19	0.42005	0.041507	0.206534	None
4353	TAF13	0.236525	0.041523	0.206566	None
4354	CIRBP-AS1	-0.06575	0.041566	0.206733	None
4355	2-Sep	0.339708	0.04163	0.207004	None
4356	LOC40075(	0.16716	0.041652	0.207067	None
4357	CTD-3092/	0.386688	0.041683	0.207169	None
4358	MTMR4	-0.22537	0.041714	0.207279	None
4359	ZNF688	-0.08187	0.041731	0.207317	None
4360	CCDC147	0.096359	0.041752	0.207329	None
4361	DDX19B	0.403475	0.041753	0.207329	None
4362	NEURL2	-0.35539	0.041812	0.207575	None
4363	DACT1	0.472192	0.041834	0.207591	None
4364	RABGAP1	-0.38818	0.041835	0.207591	None
4365	PRKD1	-0.30145	0.041855	0.207643	None
4366	NUSAP1	0.523687	0.041895	0.207755	None
4367	NCF4	-0.54728	0.041896	0.207755	None
4368	LDB1	-0.37205	0.041986	0.208152	None
4369	GAPDHS	0.014228	0.042009	0.20821	None
4370	NKAIN3	0.089128	0.042017	0.20821	None
4371	GPR137B	-0.86908	0.042031	0.208232	Down
4372	NUBP2	-0.28436	0.042091	0.208447	None
4373	TXN2	-0.36531	0.042094	0.208447	None
4374	GOPC	-0.25179	0.042112	0.20849	None
4375	LOC10106(	0.243193	0.042156	0.208553	None
4376	ISPD-AS1	0.014253	0.042157	0.208553	None
4377	ICOS	0.167576	0.042163	0.208553	None
4378	NUP43	-0.13782	0.042169	0.208553	None
4379	HLCS	-0.07663	0.042187	0.208553	None
4380	AKT1S1	-0.12561	0.042189	0.208553	None
4381	STK11	-0.11662	0.042197	0.208553	None
4382	MOB4	0.223035	0.042202	0.208553	None
4383	THOC6	-0.51139	0.042216	0.208578	None
4384	CES2	-0.17871	0.04223	0.208596	None
4385	NECAB3	-0.15091	0.042264	0.208706	None
4386	LINC01089	-0.20007	0.042273	0.208706	None
4387	ANGEL2	-0.2565	0.042281	0.208706	None
4388	STXBP1	-0.62777	0.042342	0.208918	Down
4389	ABCA3	-0.20314	0.042343	0.208918	None
4390	DGKQ	-0.16234	0.042401	0.209156	None
4391	LOC10050	-0.17308	0.042433	0.209266	None
4392	RMI1	-0.38424	0.042465	0.209374	None
4393	CCDC136	-0.21891	0.042491	0.209451	None
4394	TFDP3	0.069333	0.0425	0.209451	None
4395	ACSL3	-0.31417	0.042554	0.20967	None
4396	ALDH1B1	-0.24114	0.042607	0.209886	None
4397	RRAS	-0.39469	0.04264	0.209998	None
4398	CLEC4F	0.010845	0.042666	0.21008	None
4399	ANXA2P3	0.064255	0.042743	0.210386	None
4400	DKK2	0.056639	0.042748	0.210386	None
4401	CBR4	-0.31401	0.042773	0.210465	None
4402	CLC	1.391902	0.04284	0.210735	Up
4403	HOXC8	0.168076	0.042848	0.210735	None
4404	MAP3K7	0.300833	0.042901	0.210878	None
4405	ISY1	-0.29494	0.042904	0.210878	None
4406	POMT2	-0.12508	0.042914	0.210878	None

4407	MKRN3	0.148687	0.042916	0.210878	None
4408	TIMP2	-0.52327	0.043008	0.211281	None
4409	ACTA2	-0.23816	0.04306	0.211493	None
4410	MYLK-AS1	0.410216	0.043094	0.211609	None
4411	ABCB9	-0.13563	0.043122	0.211698	None
4412	PPP2CA	0.431142	0.043141	0.211729	None
4413	ERP44	0.265214	0.043162	0.211729	None
4414	LOC10050(	-0.64404	0.043164	0.211729	Down
4415	ZNF680	0.330499	0.043167	0.211729	None
4416	CHRM5	0.115644	0.043198	0.211834	None
4417	WDFY1	-0.27118	0.04325	0.212032	None
4418	AF520793	0.429455	0.043258	0.212032	None
4419	HBB	-0.86137	0.043307	0.212222	Down
4420	SSH3	-0.21265	0.043317	0.212225	None
4421	EPB41L2	-0.70827	0.043328	0.21223	Down
4422	DOK2	-0.7126	0.043348	0.212233	Down
4423	SULT1A2	-0.38165	0.043348	0.212233	None
4424	RP11-410(	0.061158	0.043371	0.212297	None
4425	NAT6	-0.39995	0.043398	0.21238	None
4426	PPAPDC3	-0.30295	0.043424	0.212462	None
4427	ORC3	-0.21871	0.043445	0.212499	None
4428	LIX1L	-0.10952	0.043458	0.212499	None
4429	KLHL10	0.044357	0.043461	0.212499	None
4430	CCIN	0.04625	0.043513	0.212703	None
4431	GNA12	0.22788	0.043532	0.212749	None
4432	ERCC5	-0.19909	0.043542	0.212751	None
4433	ATP10B	0.038048	0.043565	0.212811	None
4434	MFI2-AS1	-0.09447	0.04358	0.21284	None
4435	6-Sep	-0.34583	0.043628	0.213023	None
4436	ZBED1	-0.37921	0.043667	0.213165	None
4437	HOXB5	-0.33409	0.04374	0.213475	None
4438	BRCC3	-0.30419	0.043774	0.213562	None
4439	PRMT1	-0.37226	0.043791	0.213562	None
4440	NAT9	-0.23892	0.043792	0.213562	None
4441	RBM42	-0.32066	0.043797	0.213562	None
4442	DEFB108B	0.023579	0.043849	0.213765	None
4443	SFXN3	-0.39294	0.043875	0.213843	None
4444	PAN3	0.252197	0.043904	0.213938	None
4445	GALT	-0.29971	0.043917	0.213956	None
4446	RP11-493L	0.018264	0.043942	0.214028	None
4447	ZNF248	-0.27823	0.043954	0.214036	None
4448	GATSL3	-0.11659	0.043999	0.214209	None
4449	RNGTT	0.278853	0.04405	0.214411	None
4450	PROP1	0.033554	0.044085	0.214529	None
4451	UBALD1	-0.30574	0.04412	0.214652	None
4452	CTD-2554(	-0.18832	0.044151	0.214753	None
4453	TEAD4	-0.25648	0.044179	0.214841	None
4454	OR1J2	0.016971	0.04424	0.215085	None
4455	GNRHR2	0.023484	0.044263	0.215085	None
4456	FANCI	0.37299	0.044264	0.215085	None
4457	FASTKD3	-0.24788	0.044269	0.215085	None
4458	GPAA1	-0.2747	0.044287	0.215125	None
4459	RFTN1	0.145968	0.044307	0.215132	None
4460	ZNF394	0.265621	0.044317	0.215132	None
4461	PKN2	0.346079	0.044318	0.215132	None
4462	TCOF1	-0.29852	0.044338	0.215173	None
4463	GEMIN5	-0.49893	0.044346	0.215173	None
4464	PIP5K1C	-0.28271	0.044384	0.215307	None

4465	SARNP	0.178448	0.044436	0.215511	None
4466	RP3-525N1	-0.61462	0.044484	0.215699	Down
4467	ARCN1	-0.20845	0.04456	0.216017	None
4468	CTTNBP2	-0.35457	0.044576	0.216047	None
4469	NBN	-0.2872	0.044592	0.216077	None
4470	CHKB-AS1	-0.12718	0.044603	0.21608	None
4471	C21orf91	-0.28216	0.04462	0.216112	None
4472	PRKAG1	-0.44836	0.044644	0.216183	None
4473	PDXDC1	0.178883	0.04467	0.216211	None
4474	BCL7C	-0.41894	0.044685	0.216211	None
4475	AFF2	0.018024	0.044688	0.216211	None
4476	EXOC6	-0.4025	0.04469	0.216211	None
4477	EHD2	-0.4054	0.04473	0.216357	None
4478	TGFB1I1	-0.68703	0.044779	0.216487	Down
4479	SKAP2	-0.49217	0.044784	0.216487	None
4480	WDR55	-0.2043	0.044787	0.216487	None
4481	FKBP7	-0.27913	0.044881	0.216894	None
4482	SKAP1	-0.56489	0.044942	0.217016	None
4483	NRSN2	0.049469	0.04496	0.217016	None
4484	GHRH	0.005943	0.044965	0.217016	None
4485	ADAM8	-0.28331	0.044969	0.217016	None
4486	LOC10192	0.015045	0.044976	0.217016	None
4487	CTSF	-0.47987	0.044976	0.217016	None
4488	TSG101	-0.19129	0.044977	0.217016	None
4489	ATAD3B	-0.24842	0.045008	0.217118	None
4490	CDC42SE2	0.310475	0.045018	0.217119	None
4491	STRADB	-0.44867	0.045028	0.217121	None
4492	PTPRN	0.060976	0.04504	0.21713	None
4493	CCDC3	-0.52063	0.045094	0.21734	None
4494	RP11-774	-0.2137	0.045127	0.217437	None
4495	EXOC7	-0.37691	0.045134	0.217437	None
4496	LOC64648	0.006447	0.045168	0.21755	None
4497	APOL4	-0.20302	0.045264	0.217966	None
4498	WDR25	-0.29891	0.0453	0.218044	None
4499	ASAP1-IT2	-0.425	0.0453	0.218044	None
4500	LINC0131	0.056102	0.045325	0.218115	None
4501	STAB1	-0.36717	0.045345	0.218126	None
4502	SACS-AS1	0.051154	0.045357	0.218126	None
4503	GGTLC1	-0.2107	0.045361	0.218126	None
4504	GPANK1	-0.18157	0.045368	0.218126	None
4505	METTL7B	-0.60684	0.045399	0.218212	Down
4506	PRKCQ-AS	-0.39141	0.045406	0.218212	None
4507	EHD3	-0.55425	0.045442	0.21828	None
4508	CARF	-0.20423	0.045447	0.21828	None
4509	C9orf72	0.448416	0.04545	0.21828	None
4510	BHLHB9	-0.32546	0.045472	0.218338	None
4511	MS4A7	-1.35473	0.045509	0.218442	Down
4512	ARHGEF10	-0.30742	0.045514	0.218442	None
4513	REEP3	0.219547	0.045599	0.218745	None
4514	CDKN2AIP	0.27682	0.045606	0.218745	None
4515	GNB2	-0.43767	0.045608	0.218745	None
4516	FAM81B	-0.52091	0.045621	0.21876	None
4517	NFYC	-0.39393	0.045649	0.218845	None
4518	TRMT2A	-0.33094	0.045678	0.218919	None
4519	NANOG	0.12877	0.045684	0.218919	None
4520	C16orf59	-0.39909	0.045712	0.218973	None
4521	C3orf30	0.019229	0.045716	0.218973	None
4522	AAR2	0.253268	0.045756	0.219116	None

4523	MGLL	-0.45471	0.045795	0.219255	None
4524	DDAH1	-0.2521	0.045824	0.219347	None
4525	NUDT16P1	-0.12733	0.045835	0.219351	None
4526	ESYT1	-0.3079	0.045857	0.219405	None
4527	SLAMF8	-0.66421	0.045888	0.219479	Down
4528	AAAS	-0.37714	0.045892	0.219479	None
4529	PMPCB	0.16515	0.045926	0.21959	None
4530	LINC00116	-0.49924	0.045952	0.219631	None
4531	CTD-2310I	0.032664	0.045961	0.219631	None
4532	LINC00595	0.114266	0.045965	0.219631	None
4533	PRR18	0.012613	0.045979	0.219652	None
4534	PRICKLE2-.	0.060403	0.046006	0.219712	None
4535	AAK1	0.163737	0.046012	0.219712	None
4536	NOX4	0.005471	0.04603	0.219748	None
4537	SKIDA1	-0.14571	0.046046	0.219775	None
4538	HES6	-0.82211	0.046067	0.219807	Down
4539	CACNG4	0.020262	0.046073	0.219807	None
4540	COX2	-0.32109	0.046106	0.219884	None
4541	BMS1P5	-0.29107	0.046109	0.219884	None
4542	BRD2	-0.32996	0.046164	0.220099	None
4543	ETF1	0.274638	0.046262	0.220482	None
4544	B3GNT6	0.066112	0.046265	0.220482	None
4545	NRDE2	-0.13262	0.046293	0.220564	None
4546	DAK	-0.18618	0.046321	0.220631	None
4547	ZNF532	-0.61772	0.04633	0.220631	Down
4548	XK	-0.98955	0.046337	0.220631	Down
4549	RASSF5	0.15075	0.046372	0.220683	None
4550	LOC10050!	-0.24627	0.046374	0.220683	None
4551	LOC10050(	0.026802	0.046379	0.220683	None
4552	L3MBTL2	-0.23892	0.046425	0.220857	None
4553	PTGES2	-0.39427	0.046458	0.220962	None
4554	CHMP4B	-0.33535	0.046491	0.221074	None
4555	RP11-539L	-0.41397	0.046606	0.22157	None
4556	CABLES1	0.462465	0.046683	0.221889	None
4557	SSX1	0.059555	0.04674	0.222109	None
4558	DGKE	0.24628	0.046823	0.222457	None
4559	PTK7	-0.07419	0.046885	0.222699	None
4560	TSGA10	-0.09601	0.046921	0.222826	None
4561	SRGN	0.499092	0.046945	0.222888	None
4562	ABCD1	0.079698	0.047041	0.223297	None
4563	FAHD2CP	-0.24516	0.047108	0.223539	None
4564	CHCHD3	0.155789	0.047122	0.223539	None
4565	IRF2BPL	0.332429	0.047123	0.223539	None
4566	MGC16025	0.01851	0.047146	0.223584	None
4567	MERTK	-0.41203	0.047163	0.223584	None
4568	LOC10192	0.076028	0.047164	0.223584	None
4569	LOC10192	0.349623	0.047212	0.223762	None
4570	C20orf203	-0.14605	0.047274	0.224007	None
4571	ACBD4	-0.13454	0.047327	0.224211	None
4572	LOC10013	0.013486	0.04737	0.224367	None
4573	CLTA	-0.18524	0.047393	0.224397	None
4574	PGAP2	-0.25709	0.047397	0.224397	None
4575	TRMT61A	-0.14896	0.04741	0.224408	None
4576	ZWINT	0.366409	0.047458	0.224586	None
4577	SETD8	-0.23744	0.047476	0.224621	None
4578	EIF5A	-0.51117	0.0476	0.225132	None
4579	TUT1	-0.18644	0.047608	0.225132	None
4580	CLPTM1	-0.21789	0.047615	0.225132	None

4581	C20orf62	0.015195	0.047638	0.225192	None
4582	RPGR	0.334535	0.047684	0.225359	None
4583	PFN4	0.04301	0.047756	0.22565	None
4584	SNAPIN	-0.26108	0.047781	0.225721	None
4585	AX747031	0.005916	0.047809	0.225766	None
4586	GIGYF2	-0.3024	0.047831	0.225766	None
4587	NARS2	-0.27152	0.047831	0.225766	None
4588	TMEM209	0.217595	0.047833	0.225766	None
4589	TFPT	-0.35189	0.047848	0.22579	None
4590	RBM38	0.45801	0.047899	0.225914	None
4591	WDR54	0.356981	0.047907	0.225914	None
4592	TRAF3IP2	-0.19163	0.047907	0.225914	None
4593	CAPN15	-0.16328	0.047925	0.225914	None
4594	SASH1	-0.76353	0.047926	0.225914	Down
4595	VEGFB	-0.32768	0.047975	0.226044	None
4596	DDX59	-0.29993	0.047975	0.226044	None
4597	CHDH	0.043102	0.048001	0.226073	None
4598	KNOP1	-0.31609	0.048002	0.226073	None
4599	LINC00865	0.636429	0.048018	0.2261	Up
4600	NDE1	-0.1866	0.048036	0.22613	None
4601	TUBG2	-0.21344	0.048046	0.22613	None
4602	PAFAH1B2	-0.27865	0.048071	0.226202	None
4603	LOC10192	0.009073	0.048091	0.226247	None
4604	MFAP5	0.178659	0.048108	0.226275	None
4605	UQCR10	-0.3874	0.04812	0.226284	None
4606	ADCY6	-0.26325	0.048168	0.226459	None
4607	PLEKHS1	0.020749	0.048239	0.226714	None
4608	BRD4	-0.26938	0.048243	0.226714	None
4609	GALNT3	0.317019	0.048267	0.226778	None
4610	ZNF219	-0.08684	0.048298	0.226854	None
4611	SLC25A23	-0.14878	0.048304	0.226854	None
4612	TGFBRAP1	-0.31877	0.048334	0.226946	None
4613	TRUB2	-0.2841	0.048371	0.227071	None
4614	NPEPL1	-0.19088	0.048422	0.227261	None
4615	PCMTD1	-0.34419	0.048473	0.227377	None
4616	LUC7L2	0.255979	0.048475	0.227377	None
4617	ZNF512	-0.30605	0.048478	0.227377	None
4618	ADH1B	0.052271	0.048489	0.227379	None
4619	CR2	-0.17219	0.048504	0.227397	None
4620	STK36	-0.15648	0.04853	0.22747	None
4621	ING4	-0.2489	0.0486	0.227751	None
4622	SPIC	-1.08733	0.048617	0.227782	Down
4623	RASSF3	-0.32932	0.048648	0.227858	None
4624	ABCC1	-0.29979	0.048655	0.227858	None
4625	GPR180	-0.22794	0.048699	0.228018	None
4626	CELF6	-0.23134	0.048716	0.228045	None
4627	KIAA1107	-0.24036	0.048741	0.228113	None
4628	SAC3D1	-0.39737	0.048781	0.228254	None
4629	HYI	0.269219	0.048822	0.228346	None
4630	SPRYD7	-0.19941	0.048826	0.228346	None
4631	TMBIM4	-0.16454	0.048833	0.228346	None
4632	ZNF564	-0.3107	0.048873	0.228447	None
4633	DNA2	0.376539	0.048875	0.228447	None
4634	NEURL3	0.133388	0.048968	0.228828	None
4635	DHX58	-0.27644	0.048982	0.228828	None
4636	TANC2	-0.22156	0.04899	0.228828	None
4637	XKR8	-0.22305	0.048999	0.228828	None
4638	RP11-196(	-0.37351	0.049027	0.228908	None

4639	LOC10099(	0.091314	0.049075	0.229085	None
4640	C17orf62	-0.32912	0.049091	0.229108	None
4641	HIATL2	-0.30149	0.049123	0.229172	None
4642	IST1	-0.21481	0.049126	0.229172	None
4643	CD3G	0.168491	0.049169	0.22928	None
4644	GLIPR2	-0.39123	0.04917	0.22928	None
4645	CACNG7	0.03682	0.049204	0.229371	None
4646	TAF2	0.211584	0.049217	0.229371	None
4647	LOC10192!	0.021372	0.049221	0.229371	None
4648	CROT	-0.29249	0.049305	0.229713	None
4649	LYSMD3	0.355976	0.049335	0.229804	None
4650	NUDT7	-0.31982	0.049382	0.229944	None
4651	MFSD3	-0.32598	0.049387	0.229944	None
4652	OGFOD2	-0.22412	0.049442	0.23015	None
4653	UBXN10	-0.34673	0.049494	0.230343	None
4654	CD33	-0.5583	0.049513	0.230384	None
4655	C7orf26	-0.3182	0.049558	0.230534	None
4656	LAMB3	0.068249	0.049567	0.230534	None
4657	ZNF141	-0.26675	0.049587	0.23058	None
4658	SLC29A2	-0.03381	0.0496	0.230591	None
4659	ATP5SL	-0.21977	0.049628	0.230671	None
4660	MT1X	-0.92298	0.049647	0.230703	Down
4661	THAP4	-0.23248	0.049656	0.230703	None
4662	LOC10272:	0.041588	0.049686	0.230793	None
4663	EPSTI1	-0.57495	0.049717	0.230888	None
4664	PROSER2	-0.3091	0.049758	0.231013	None
4665	PPP2R5C	0.271649	0.049777	0.231013	None
4666	ZNF736	0.277671	0.04978	0.231013	None
4667	TMEM201	-0.18569	0.049787	0.231013	None
4668	PTBP3	-0.3729	0.049863	0.231277	None
4669	STXBP6	-0.41426	0.049873	0.231277	None
4670	EID1	0.284087	0.049876	0.231277	None
4671	ALDH7A1	0.416929	0.049895	0.231316	None
4672	LSM6	-0.27727	0.04993	0.231428	None
4673	LAMA5	-0.36675	0.049957	0.231433	None
4674	N6AMT2	-0.31159	0.04996	0.231433	None
4675	MFAP4	-0.52548	0.049963	0.231433	None
4676	CALR	-0.67869	0.049978	0.231439	Down
4677	ARHGEF18	-0.316	0.049999	0.231439	None
4678	GJB1	0.142414	0.049999	0.231439	None
4679	LINC01049	0.066756	0.050007	0.231439	None
4680	PTPLA	-0.21674	0.050045	0.231563	None
4681	TSSK3	0.021866	0.050094	0.231743	None
4682	KEL	-0.46717	0.050172	0.232054	None
4683	THAP8	-0.19081	0.050187	0.232074	None
4684	ZCCHC7	0.437944	0.05022	0.232176	None
4685	AOAH	-0.56876	0.050246	0.232245	None
4686	AREG	0.888634	0.05027	0.23226	None
4687	CNTROB	-0.09143	0.05027	0.23226	None
4688	STK24	0.273653	0.050285	0.232277	None
4689	NR2C2AP	-0.35507	0.0503	0.232296	None
4690	TP53I11	0.138657	0.050398	0.232614	None
4691	NDUFB8	0.281448	0.050401	0.232614	None
4692	STRBP	0.322728	0.050408	0.232614	None
4693	CEP97	0.367965	0.050412	0.232614	None
4694	ZNF496	-0.20084	0.050439	0.232614	None
4695	CYB5D1	-0.30555	0.05044	0.232614	None
4696	STUB1	-0.3	0.050443	0.232614	None

4697	ANKRD20/	-0.34585	0.050466	0.23267	None
4698	PRDM6	0.03593	0.050501	0.23278	None
4699	LOC10013:	0.004338	0.050554	0.232975	None
4700	SARS2	-0.36179	0.050588	0.23308	None
4701	NCKAP1L	-0.27685	0.050607	0.233112	None
4702	EBAG9	-0.21146	0.050616	0.233112	None
4703	LINGO1	-0.11869	0.05065	0.233182	None
4704	LINC00642	0.015847	0.050653	0.233182	None
4705	NPVF	0.037613	0.050668	0.233203	None
4706	LOC10050!	0.079637	0.05071	0.233345	None
4707	ITGAL	-0.49799	0.050776	0.233598	None
4708	ABCC4	-0.4993	0.050827	0.233739	None
4709	LINC00951	0.136335	0.050832	0.233739	None
4710	C16orf72	-0.43844	0.050856	0.233739	None
4711	TMEM253	0.010733	0.050859	0.233739	None
4712	ACSF3	-0.28688	0.05086	0.233739	None
4713	LOC10050!	0.069807	0.050933	0.234023	None
4714	ARV1	-0.16276	0.050965	0.234117	None
4715	ARSK	0.195113	0.050975	0.234117	None
4716	ADAM33	0.131457	0.051094	0.234525	None
4717	TFF2	0.092337	0.051096	0.234525	None
4718	TMEM131	-0.17072	0.051096	0.234525	None
4719	PPFIA1	-0.21812	0.051125	0.234575	None
4720	LOC10013:	0.083087	0.051135	0.234575	None
4721	ATXN7	-0.35256	0.05114	0.234575	None
4722	ICAM2	-0.28487	0.051167	0.234651	None
4723	TTC21A	0.064639	0.051216	0.234826	None
4724	RLIM	0.269207	0.051244	0.234865	None
4725	HSPA4L	-0.45741	0.051252	0.234865	None
4726	SCFD2	-0.26456	0.051282	0.234865	None
4727	CDC42EP3	-0.4844	0.051287	0.234865	None
4728	MISP	0.084888	0.051297	0.234865	None
4729	TNFAIP8L1	-0.26766	0.0513	0.234865	None
4730	TXNDC17	-0.17608	0.051301	0.234865	None
4731	KIAA1328	-0.1011	0.051325	0.23493	None
4732	NLRC4	-0.31303	0.051401	0.235108	None
4733	RHBDD1	-0.17863	0.051403	0.235108	None
4734	ZBTB1	0.221892	0.051406	0.235108	None
4735	CACNB1	0.069694	0.05141	0.235108	None
4736	RNU2-22P	-0.37647	0.05145	0.235108	None
4737	SLC38A11	0.009079	0.051457	0.235108	None
4738	LOC44117!	0.021619	0.05146	0.235108	None
4739	MAK16	-0.27949	0.051466	0.235108	None
4740	ZNF79	-0.2534	0.051468	0.235108	None
4741	UVSSA	0.068941	0.051476	0.235108	None
4742	OPTC	0.139488	0.051484	0.235108	None
4743	LPAR2	-0.25435	0.051548	0.235307	None
4744	FGF9	0.334176	0.051558	0.235307	None
4745	PEA15	-0.27159	0.05156	0.235307	None
4746	DTYMK	0.225213	0.051602	0.23545	None
4747	GMPPA	-0.34131	0.05166	0.235664	None
4748	RP11-357(	0.048304	0.05168	0.235703	None
4749	CTC-428G:	0.301981	0.05169	0.235703	None
4750	AB488780	-0.27509	0.051716	0.235741	None
4751	CCDC23	-0.31167	0.05172	0.235741	None
4752	DEPDC4	-0.0481	0.051793	0.236019	None
4753	STX1A	0.071201	0.051807	0.236019	None
4754	RP11-932(	0.007118	0.051814	0.236019	None

4755	DPH3P1	0.037045	0.051994	0.236743	None
4756	TOR4A	-0.32121	0.051996	0.236743	None
4757	CTNNA1	-0.32079	0.052016	0.236743	None
4758	ZIC4	0.006456	0.052017	0.236743	None
4759	LINC01225	0.089502	0.052042	0.236808	None
4760	LINC00997	-0.17612	0.052064	0.236857	None
4761	BTBD2	-0.1306	0.052122	0.237071	None
4762	ZNF350	0.367453	0.052189	0.237293	None
4763	DEGS2	-0.2417	0.052192	0.237293	None
4764	OSBPL1A	-0.52378	0.052227	0.237402	None
4765	ATG7	-0.28667	0.052262	0.237509	None
4766	TFAP2A-A:	0.091615	0.052279	0.237537	None
4767	FAM174A	0.359904	0.05232	0.237673	None
4768	ZBTB34	-0.29088	0.052342	0.237723	None
4769	FLJ90680	0.011731	0.052354	0.237726	None
4770	RPP25	-0.29135	0.052396	0.237868	None
4771	TRNT1	-0.30999	0.052445	0.238043	None
4772	PMS2P4	0.066496	0.052491	0.238175	None
4773	KLHL15	0.326543	0.052505	0.238175	None
4774	EXOSC3	-0.19145	0.052507	0.238175	None
4775	NBPF3	-0.24444	0.052571	0.238338	None
4776	NUCKS1	0.229251	0.052576	0.238338	None
4777	RAB9B	-0.30412	0.052579	0.238338	None
4778	OCIAD1	0.134428	0.052587	0.238338	None
4779	GTF2H2B	-0.61605	0.052632	0.238487	None
4780	FAM20B	-0.23078	0.05265	0.238487	None
4781	PHKG2	0.044373	0.052653	0.238487	None
4782	LOC10192	0.00811	0.052744	0.238849	None
4783	RTN2	-0.28048	0.052783	0.238973	None
4784	LITAF	-0.47555	0.052826	0.239093	None
4785	CCDC129	0.039571	0.05284	0.239093	None
4786	SCO2	-0.34353	0.052842	0.239093	None
4787	LINC00339	-0.48818	0.052951	0.239534	None
4788	LINC00441	0.043605	0.05298	0.239597	None
4789	BMS1	0.275211	0.052998	0.239597	None
4790	PLA2G2F	0.042014	0.053008	0.239597	None
4791	RP11-480D	0.095205	0.053012	0.239597	None
4792	LINC00410	0.080727	0.053035	0.239597	None
4793	UBXN8	-0.25411	0.053039	0.239597	None
4794	C17orf96	-0.24077	0.053042	0.239597	None
4795	RP5-894D1	0.037419	0.053142	0.239965	None
4796	TNK2-AS1	0.066269	0.053146	0.239965	None
4797	SCAPER	-0.34532	0.053331	0.240752	None
4798	MST1	-0.30613	0.053381	0.240882	None
4799	GRAMD1B	0.337763	0.053382	0.240882	None
4800	SMARCE1	0.233336	0.053404	0.240928	None
4801	FRMPD4	0.019378	0.053422	0.240963	None
4802	JMJD6	0.258202	0.053444	0.240997	None
4803	ADD2	-0.46809	0.053452	0.240997	None
4804	CCPG1	-0.22686	0.05353	0.241259	None
4805	NAGK	-0.58359	0.053533	0.241259	None
4806	TP53I3	-0.5613	0.053548	0.241276	None
4807	CCP110	0.22739	0.053592	0.241424	None
4808	AMN	-0.36302	0.053699	0.241856	None
4809	CNPY2	0.307275	0.053714	0.241873	None
4810	DDX23	-0.27956	0.053736	0.241924	None
4811	PEBP1	-0.26501	0.053774	0.242043	None
4812	EIF3E	0.239555	0.053799	0.242108	None

4813	SERPINA9	0.050573	0.053815	0.242127	None
4814	MRGPRX3	0.029705	0.053866	0.242307	None
4815	NIFK	-0.20313	0.053967	0.242711	None
4816	HMGCL	-0.35718	0.053982	0.242727	None
4817	CECR2	0.027517	0.054013	0.242758	None
4818	INTS8	-0.21367	0.054019	0.242758	None
4819	TMEM55B	-0.30818	0.054022	0.242758	None
4820	USO1	-0.23698	0.054044	0.242758	None
4821	WAS	-0.12016	0.054045	0.242758	None
4822	TMEM203	-0.24349	0.054143	0.243124	None
4823	SH3D19	-0.30061	0.054149	0.243124	None
4824	LINC01149	0.034999	0.054187	0.24323	None
4825	SIRT4	-0.09113	0.054195	0.24323	None
4826	RPS27L	-0.37343	0.054215	0.243272	None
4827	USF2	-0.2508	0.054239	0.243308	None
4828	ARL11	-0.28537	0.054251	0.243308	None
4829	NAA16	0.276658	0.054257	0.243308	None
4830	CISD3	-0.18601	0.054268	0.243308	None
4831	MSL1	-0.24336	0.054314	0.243464	None
4832	CASP1	-0.48124	0.054386	0.24369	None
4833	LSR	-0.1903	0.054387	0.24369	None
4834	WDR11	0.296083	0.054474	0.243979	None
4835	DHRS12	0.145416	0.054477	0.243979	None
4836	RUNDC1	-0.1696	0.054486	0.243979	None
4837	GPR160	-0.55398	0.054501	0.243997	None
4838	CTA-292E1	-0.36456	0.05457	0.244255	None
4839	PPIP5K1	-0.19251	0.054595	0.24432	None
4840	RP11-18I14	0.104395	0.054607	0.244321	None
4841	MTA1	-0.20031	0.054669	0.244546	None
4842	UGT3A1	0.002872	0.054695	0.244611	None
4843	ATF6	-0.16275	0.054715	0.244611	None
4844	MBOAT2	-0.43288	0.054717	0.244611	None
4845	LEAP2	0.447025	0.054745	0.244615	None
4846	EIF2A	0.242081	0.054747	0.244615	None
4847	SMIM1	-0.49855	0.054758	0.244615	None
4848	VRK1	0.266561	0.054763	0.244615	None
4849	TYW1	-0.24376	0.054832	0.244875	None
4850	FCER1G	-0.90309	0.05485	0.244901	None
4851	LCK	0.437059	0.054862	0.244906	None
4852	MYO9B	-0.40954	0.054905	0.245049	None
4853	MGST3	0.091755	0.054933	0.245123	None
4854	SLC11A2	0.325637	0.055005	0.245394	None
4855	RILPL1	-0.43564	0.055041	0.245503	None
4856	CCDC104	-0.30209	0.055078	0.245603	None
4857	TMEM144	-0.20191	0.055086	0.245603	None
4858	TEFM	-0.25991	0.055136	0.245713	None
4859	RP11-12M	0.020014	0.05515	0.245713	None
4860	KRBA2	-0.56809	0.05516	0.245713	None
4861	DHX16	-0.22752	0.055165	0.245713	None
4862	RNF114	-0.25864	0.055168	0.245713	None
4863	SNAPC1	0.33258	0.055188	0.245729	None
4864	FAM86A	-0.13876	0.055203	0.245729	None
4865	PITRM1	0.104874	0.055205	0.245729	None
4866	LRRC27	-0.08173	0.055257	0.245903	None
4867	DOCK3	-0.57402	0.055267	0.245903	None
4868	CRTC1	-0.08801	0.055294	0.245961	None
4869	LANCL3	-0.52004	0.055303	0.245961	None
4870	AC005306.	-0.22681	0.055325	0.246007	None

4871	MIRLET7D	-0.49938	0.055406	0.246318	None
4872	ZDHHC12	-0.22092	0.05552	0.246776	None
4873	APOBR	-0.3089	0.055593	0.24705	None
4874	ALMS1	0.301933	0.05562	0.247116	None
4875	HHEX	-0.33978	0.055648	0.247191	None
4876	TNKS2	0.25676	0.055674	0.247238	None
4877	HPCA	-0.0754	0.055681	0.247238	None
4878	TSPAN33	-0.45962	0.055749	0.247487	None
4879	CDYL	-0.30276	0.055775	0.247489	None
4880	WTAP	0.291113	0.05579	0.247489	None
4881	IKZF5	0.232857	0.055793	0.247489	None
4882	RSF1	0.187483	0.055795	0.247489	None
4883	LYPD4	0.011415	0.055855	0.247705	None
4884	PATL1	-0.18792	0.055906	0.247803	None
4885	ZSWIM1	-0.11309	0.055909	0.247803	None
4886	RILP	-0.29824	0.05592	0.247803	None
4887	ATG16L2	-0.4648	0.055923	0.247803	None
4888	LOC64676:	-0.24591	0.055948	0.247862	None
4889	CASC7	0.413826	0.055968	0.2479	None
4890	AC012531.	0.074438	0.055982	0.24791	None
4891	NKAP	-0.20354	0.055993	0.24791	None
4892	BC130595	0.076174	0.05601	0.247936	None
4893	C5orf42	0.147188	0.056072	0.24816	None
4894	C1orf53	-0.26436	0.056098	0.248222	None
4895	GEMIN8	-0.26573	0.056109	0.248222	None
4896	RABAC1	0.407248	0.056133	0.248275	None
4897	LOC10192	-0.61101	0.056169	0.248386	None
4898	DDAH2	0.528754	0.0562	0.248469	None
4899	PPP1CA	0.304181	0.056232	0.24856	None
4900	LETM1	0.177241	0.056346	0.24898	None
4901	WBP4	-0.18874	0.05635	0.24898	None
4902	UBE3B	0.154448	0.056376	0.249047	None
4903	LOC28385	0.004685	0.056425	0.24921	None
4904	RAD52	-0.1645	0.056475	0.249383	None
4905	CYCS	-0.50667	0.056547	0.249622	None
4906	AKAP17A	-0.44986	0.056553	0.249622	None
4907	C10orf32	-0.25961	0.056571	0.249653	None
4908	HNRNPH3	0.188238	0.056613	0.249787	None
4909	BIVM	-0.29205	0.056628	0.249801	None
4910	EDC4	-0.23922	0.056661	0.249899	None
4911	MYBPC3	0.141983	0.056677	0.249918	None
4912	NREP	0.446987	0.056698	0.249948	None
4913	LRRC75A	-0.31192	0.056707	0.249948	None
4914	LZTS3	0.318533	0.056769	0.250154	None
4915	TNS1	-0.38683	0.056777	0.250154	None
4916	CST9	0.015439	0.056819	0.250275	None
4917	LINC00597	0.524999	0.056828	0.250275	None
4918	OTOS	0.021385	0.056919	0.250625	None
4919	SAP30L	-0.20713	0.056944	0.250684	None
4920	GPSM2	-0.29626	0.056964	0.250716	None
4921	GATAD2B	-0.25729	0.056983	0.250716	None
4922	NDUFB3	-0.17634	0.056986	0.250716	None
4923	BCAT1	-0.32092	0.057045	0.250874	None
4924	ACP1	-0.27248	0.057045	0.250874	None
4925	CSTA	0.818356	0.057056	0.250874	None
4926	ITGB2-AS1	-0.42086	0.057068	0.250875	None
4927	WRAP73	-0.28228	0.057121	0.251057	None
4928	BPIFA3	0.141452	0.057224	0.251432	None

4929	RP11-486(	-0.38708	0.057236	0.251432	None
4930	FCHSD1	-0.10208	0.057241	0.251432	None
4931	RNF213	-0.28371	0.057262	0.251473	None
4932	PPP1R12A	-0.32901	0.057285	0.25152	None
4933	LOC10272:	-0.27913	0.05732	0.251623	None
4934	NVL	-0.24152	0.057444	0.252117	None
4935	PQLC2	-0.23662	0.057476	0.252165	None
4936	VPS37D	0.102497	0.057478	0.252165	None
4937	OR2M4	0.014567	0.05752	0.252296	None
4938	DNAJC19	-0.22147	0.05754	0.252337	None
4939	LINC01107	0.029237	0.057563	0.252384	None
4940	LOC10050	-0.18227	0.057584	0.252423	None
4941	TUBD1	-0.35477	0.057626	0.252543	None
4942	BRF1	0.084229	0.057636	0.252543	None
4943	UROS	-0.38559	0.057646	0.252543	None
4944	PLEKHA8	0.130216	0.057667	0.252587	None
4945	DDX43	-0.67506	0.057689	0.252632	None
4946	FANCM	-0.25524	0.057735	0.252683	None
4947	NDNF	0.055911	0.05774	0.252683	None
4948	CLCC1	0.322953	0.057743	0.252683	None
4949	TXNDC12	-0.19386	0.057751	0.252683	None
4950	CMC1	-0.24454	0.057759	0.252683	None
4951	PYCARDO:	-0.23311	0.057784	0.252738	None
4952	CRP	0.003209	0.057833	0.252886	None
4953	BMP7	0.060971	0.057841	0.252886	None
4954	NUF2	0.478515	0.057857	0.252903	None
4955	ACRBP	-0.63781	0.057946	0.253244	None
4956	ZNF346	-0.33003	0.058013	0.253485	None
4957	CCDC169	-0.11205	0.058036	0.253533	None
4958	CYMP	0.029073	0.058061	0.253555	None
4959	ZNF543	0.041354	0.058064	0.253555	None
4960	ASTN2	-0.10901	0.058114	0.25372	None
4961	UAP1L1	-0.35608	0.058155	0.253847	None
4962	LPHN1	-0.22812	0.058216	0.254049	None
4963	THNSL2	-0.12668	0.058234	0.254049	None
4964	LIME1	-0.21732	0.058236	0.254049	None
4965	DZANK1	-0.16688	0.05828	0.254192	None
4966	SSBP2	0.444217	0.058298	0.254216	None
4967	LOC10192	0.008632	0.058379	0.254518	None
4968	SYMPK	-0.14197	0.058429	0.254603	None
4969	B4GALT2	-0.26529	0.05844	0.254603	None
4970	TMEM56	-0.7833	0.058451	0.254603	None
4971	GPR3	0.090918	0.058453	0.254603	None
4972	PGAP1	0.451052	0.058459	0.254603	None
4973	MGC34796	0.099099	0.058469	0.254603	None
4974	PWWP2A	-0.31693	0.05849	0.254645	None
4975	KCTD2	-0.23926	0.058602	0.255061	None
4976	PIGH	-0.29968	0.058609	0.255061	None
4977	OR7E37P	-0.46332	0.058661	0.255234	None
4978	MTIF3	-0.18783	0.058706	0.25535	None
4979	LPCAT4	-0.15137	0.058711	0.25535	None
4980	CARKD	-0.31756	0.058775	0.255541	None
4981	MYH8	0.034627	0.058778	0.255541	None
4982	SLC30A7	0.272306	0.058865	0.255851	None
4983	MAN2A1	0.307637	0.058874	0.255851	None
4984	LINC00635	0.02668	0.058934	0.25606	None
4985	NT5M	-0.356	0.058956	0.256107	None
4986	ALAS2	-0.71414	0.058983	0.256175	None

4987	HSCB	-0.28556	0.05916	0.256889	None
4988	ZNF317	-0.13427	0.059208	0.257046	None
4989	NT5C3A	-0.32144	0.059268	0.257254	None
4990	WNT1	0.02231	0.059284	0.257273	None
4991	KRTCAP3	-0.35342	0.059511	0.258207	None
4992	CAPN2	-0.22672	0.059559	0.258352	None
4993	EMC8	-0.22648	0.05958	0.258352	None
4994	ALDH4A1	-0.14615	0.05958	0.258352	None
4995	CYTH2	-0.33436	0.059631	0.258488	None
4996	SIGLEC7	-0.44944	0.059635	0.258488	None
4997	RP11-389(	-0.57459	0.059762	0.258971	None
4998	RPP14	-0.24027	0.059771	0.258971	None
4999	L3HYPDH	-0.38511	0.059811	0.259048	None
5000	MED30	0.426408	0.059812	0.259048	None
5001	RP11-1078	0.055519	0.059828	0.259065	None
5002	AC025442.	0.065475	0.059898	0.259315	None
5003	TGOLN2	-0.30217	0.060001	0.259708	None
5004	RP11-548M	0.197636	0.060027	0.259767	None
5005	NRIP2	0.0404	0.060057	0.259807	None
5006	CACNB4	-0.30762	0.06006	0.259807	None
5007	FCF1	-0.2295	0.060126	0.260043	None
5008	MMD	-0.40302	0.060145	0.26007	None
5009	C17orf77	0.059958	0.060163	0.260099	None
5010	UBE2J1	0.27639	0.060189	0.260158	None
5011	ST6GAL2	0.631544	0.060264	0.260387	None
5012	SPRYD4	-0.31586	0.060266	0.260387	None
5013	MRO	-0.731	0.06034	0.260653	None
5014	LOC10192	0.047067	0.060395	0.260841	None
5015	SSBP3	-0.30169	0.060507	0.261273	None
5016	MYF5	0.026534	0.060546	0.2613	None
5017	PPP1R26-/	-0.08741	0.060549	0.2613	None
5018	PSMF1	-0.2251	0.06055	0.2613	None
5019	EPOR	-0.54281	0.060587	0.261409	None
5020	MEG3	-0.39329	0.060622	0.26151	None
5021	GTF2E1	-0.29115	0.060661	0.261622	None
5022	SMUG1	-0.1672	0.060686	0.261669	None
5023	CYP2C8	-0.20102	0.0607	0.261669	None
5024	ARHGAP5-	-0.20261	0.060708	0.261669	None
5025	HMBOX1	-0.27307	0.060733	0.261726	None
5026	LOC10192	0.015764	0.060753	0.261726	None
5027	UBQLN1	-0.27163	0.060757	0.261726	None
5028	GLRB	-0.32756	0.060803	0.261828	None
5029	ZNF204P	-0.56622	0.060815	0.261828	None
5030	ACSM5	-0.6956	0.060817	0.261828	None
5031	ZDHHC11	-0.22623	0.060856	0.261924	None
5032	RBMY3AP	0.084708	0.060864	0.261924	None
5033	HARS2	-0.33042	0.060893	0.261949	None
5034	RIC8B	-0.20303	0.060905	0.261949	None
5035	MUC3B	0.064462	0.060906	0.261949	None
5036	TSPAN14	0.380399	0.061004	0.262321	None
5037	BACE1	-0.30413	0.061033	0.262393	None
5038	SLC37A4	-0.28386	0.061091	0.26259	None
5039	RAB7B	-0.57784	0.061129	0.262699	None
5040	TMEM47	0.154766	0.061179	0.262864	None
5041	DCK	0.347742	0.06122	0.262902	None
5042	RP11-365H	0.156755	0.061224	0.262902	None
5043	SLC12A1	0.062592	0.061229	0.262902	None
5044	FABP3	-0.9191	0.061237	0.262902	None

5045	RNF215	0.009434	0.06134	0.263271	None
5046	TMEM248	-0.21596	0.061347	0.263271	None
5047	PYCR1	-0.18525	0.061416	0.263516	None
5048	LOC10013	-0.31277	0.061454	0.263628	None
5049	ANKHD1	0.416893	0.061513	0.263829	None
5050	ZNF57	-0.40749	0.061567	0.264008	None
5051	RP4-742J2	0.108077	0.061604	0.26408	None
5052	OR10H3	0.009378	0.061609	0.26408	None
5053	IFT81	0.15837	0.061671	0.264295	None
5054	HS3ST2	-1.04842	0.061705	0.264388	None
5055	VIM	0.411858	0.061731	0.264447	None
5056	MPO	0.830737	0.06177	0.264564	None
5057	RB1CC1	0.327213	0.061802	0.264648	None
5058	EPB41	-0.21668	0.061866	0.264735	None
5059	RP11-819(	0.35188	0.061867	0.264735	None
5060	STARD5	-0.29028	0.061868	0.264735	None
5061	RELT	-0.33255	0.061871	0.264735	None
5062	AL109706	-0.17111	0.061896	0.264786	None
5063	WDR34	0.386342	0.06195	0.264968	None
5064	SH3GL1	-0.26299	0.061984	0.26506	None
5065	ACTL9	0.018246	0.062012	0.265128	None
5066	SUGT1	-0.24163	0.062057	0.265269	None
5067	FAM83A	-0.43759	0.062103	0.265411	None
5068	ZACN	0.070948	0.06215	0.265559	None
5069	PARK7	0.162969	0.062182	0.265611	None
5070	MN1	-0.61524	0.062186	0.265611	None
5071	ERC2-IT1	0.067911	0.062228	0.265738	None
5072	PPP4R2	0.352669	0.062247	0.265766	None
5073	NR1H2	-0.31968	0.062369	0.266234	None
5074	MRPS34	-0.39058	0.062396	0.266297	None
5075	CCL13	-0.34922	0.062413	0.266317	None
5076	SETD9	-0.16872	0.06245	0.266421	None
5077	NMU	-1.25306	0.062477	0.266484	None
5078	KIF12	0.070547	0.062504	0.266547	None
5079	CPA4	0.06068	0.062537	0.266636	None
5080	CAPN7	-0.21317	0.062636	0.267006	None
5081	HLA-DRB6	0.143415	0.062653	0.267026	None
5082	SPRED3	-0.18906	0.062759	0.267396	None
5083	LRRTM2	0.171234	0.062765	0.267396	None
5084	NUP133	-0.12488	0.062821	0.267564	None
5085	LINC00884	-0.14153	0.062833	0.267564	None
5086	PDZK1IP1	-0.76545	0.062841	0.267564	None
5087	DOCK7	-0.20749	0.062905	0.267783	None
5088	SLC37A1	-0.15143	0.062945	0.267855	None
5089	MAP1S	-0.38973	0.062947	0.267855	None
5090	REL	0.442918	0.062996	0.268013	None
5091	NUP62CL	-0.25209	0.063054	0.268161	None
5092	BCAT2	-0.15408	0.063074	0.268161	None
5093	MKL1	0.080269	0.063081	0.268161	None
5094	ATP13A2	-0.38479	0.063102	0.268161	None
5095	ZNF645	0.023341	0.063105	0.268161	None
5096	FAH	-0.15925	0.063105	0.268161	None
5097	ENPP5	-0.34709	0.063145	0.268275	None
5098	DENND6A	0.207692	0.063173	0.268294	None
5099	ATE1	0.131219	0.063184	0.268294	None
5100	GZMB	0.63551	0.063212	0.268294	None
5101	ADAMTS1	0.013362	0.063221	0.268294	None
5102	LINC00557	0.005354	0.063234	0.268294	None

5103	PACS1	-0.1405	0.063238	0.268294	None
5104	F11R	-0.31664	0.063247	0.268294	None
5105	RP11-212F	0.193632	0.063251	0.268294	None
5106	CCDC14	0.10847	0.063261	0.268294	None
5107	ZNF879	-0.38678	0.063351	0.268623	None
5108	LRMP	-0.22454	0.063429	0.268904	None
5109	IPP	-0.15015	0.063475	0.269044	None
5110	TXNRD2	-0.17074	0.063543	0.269282	None
5111	ZNF600	-0.26667	0.063558	0.269291	None
5112	COPG1	-0.54645	0.063572	0.269297	None
5113	CTIF	-0.11354	0.063596	0.269347	None
5114	KHDRBS2	0.113452	0.063621	0.2694	None
5115	NDUFB7	-0.20366	0.063642	0.269436	None
5116	STC1	0.033658	0.063829	0.270135	None
5117	STRADA	-0.1372	0.063832	0.270135	None
5118	POLR2H	-0.25125	0.063845	0.270139	None
5119	RNPEP	-0.30816	0.063902	0.270324	None
5120	KRTAP8-1	0.053613	0.063929	0.270333	None
5121	AP3D1	-0.43946	0.063931	0.270333	None
5122	USP31	-0.37868	0.063953	0.270333	None
5123	DUSP14	-0.28691	0.063954	0.270333	None
5124	MCOLN2	0.332614	0.063976	0.270375	None
5125	STK35	-0.1585	0.06401	0.270466	None
5126	C5orf45	-0.13172	0.064026	0.270481	None
5127	TLE1	0.551375	0.064039	0.270484	None
5128	LPCAT2	0.350326	0.06413	0.270815	None
5129	SLC2A8	-0.18336	0.06416	0.270855	None
5130	LDOC1L	0.234269	0.06418	0.270855	None
5131	CTC-429P9	-0.24119	0.06419	0.270855	None
5132	TMPRSS3	0.04224	0.064195	0.270855	None
5133	LSM11	-0.20031	0.064202	0.270855	None
5134	CYB5R3	-0.19195	0.064217	0.270866	None
5135	SUCLG1	-0.10304	0.064235	0.270887	None
5136	UBE2J2	-0.23157	0.064271	0.270968	None
5137	KHDRBS3	-0.35662	0.064279	0.270968	None
5138	LRSAM1	-0.08435	0.064303	0.271017	None
5139	ZNF646	-0.1462	0.064322	0.271033	None
5140	TBC1D10B	-0.32133	0.064332	0.271033	None
5141	PARP2	-0.21064	0.064358	0.271088	None
5142	RP11-348E	0.007817	0.064379	0.271127	None
5143	VPS45	-0.14461	0.064423	0.271241	None
5144	CACNG8	-0.08959	0.064431	0.271241	None
5145	TMEM213	0.013466	0.064469	0.27133	None
5146	P2RX3	0.056555	0.064478	0.27133	None
5147	FBXO21	0.247238	0.064507	0.271403	None
5148	ALOX5AP	0.647224	0.064523	0.271414	None
5149	ARHGAP35	-0.22632	0.064766	0.272384	None
5150	HEXDC	-0.12904	0.064864	0.272743	None
5151	PEX12	-0.40925	0.064911	0.272839	None
5152	TAP1	-0.29323	0.064916	0.272839	None
5153	TNFSF8	0.317028	0.064925	0.272839	None
5154	XAB2	-0.25574	0.064965	0.272955	None
5155	CCNC	0.229378	0.06503	0.273178	None
5156	MRPS23	-0.25806	0.06505	0.273207	None
5157	LOC10192	0.003246	0.0651	0.273366	None
5158	GGCT	-0.22793	0.065146	0.273496	None
5159	FKBP9	-0.564	0.065157	0.273496	None
5160	LYPLAL1	-0.36037	0.06522	0.273707	None

5161	HRH3	0.050962	0.065258	0.273815	None
5162	GLB1L	-0.57798	0.065302	0.273948	None
5163	RAC2	-0.35898	0.065325	0.273957	None
5164	DYNC2LI1	-0.22235	0.06533	0.273957	None
5165	LOC10272:	-0.5336	0.065347	0.273976	None
5166	PLEK	-0.30317	0.065394	0.274068	None
5167	LOC10013(	0.008007	0.065394	0.274068	None
5168	LYRM4	-0.24675	0.065417	0.274088	None
5169	MTRF1	-0.20674	0.065428	0.274088	None
5170	NUCB2	0.275702	0.065437	0.274088	None
5171	KRT25	0.058681	0.065478	0.274197	None
5172	USP28	-0.25204	0.065488	0.274197	None
5173	MASTL	0.453216	0.065508	0.27422	None
5174	JADE2	0.38748	0.065519	0.27422	None
5175	BTNL8	0.130706	0.065547	0.274283	None
5176	LINC00899	-0.31101	0.065579	0.274366	None
5177	IL1RAPL2	0.042445	0.065646	0.27458	None
5178	SURF6	-0.29073	0.065656	0.27458	None
5179	B3GAT3	-0.22407	0.065698	0.274692	None
5180	MRPS21	-0.17259	0.065708	0.274692	None
5181	FUCA1	-0.38995	0.065723	0.274693	None
5182	SH3GL1P1	0.0244	0.065739	0.274693	None
5183	UBA2	0.211413	0.065746	0.274693	None
5184	TMEM173	-0.42237	0.065787	0.27481	None
5185	SNX13	0.084169	0.065812	0.274838	None
5186	RTN3	-0.20795	0.065819	0.274838	None
5187	DAAM2	-0.57235	0.065835	0.274853	None
5188	RPRD2	-0.26929	0.065863	0.2749	None
5189	CCDC127	-0.31177	0.065872	0.2749	None
5190	LRRC37A2	0.254974	0.065935	0.275112	None
5191	CPLX3	0.056988	0.06596	0.275161	None
5192	ING1	0.211051	0.065997	0.275263	None
5193	PBX3	-0.45344	0.066027	0.275335	None
5194	SPTA1	0.678787	0.066098	0.275578	None
5195	NEU4	0.21956	0.066117	0.275605	None
5196	ATP6V0A1	-0.14569	0.066134	0.275623	None
5197	EMILIN1	-0.55274	0.066261	0.276057	None
5198	TAF4B	-0.25103	0.066281	0.276057	None
5199	ARHGEF37	-0.27766	0.066298	0.276057	None
5200	PPAPDC1B	-0.20643	0.066299	0.276057	None
5201	ZMYM4	-0.21457	0.066302	0.276057	None
5202	CYP2A6	0.019331	0.06636	0.27619	None
5203	GPR114	-0.26467	0.066361	0.27619	None
5204	TAGLN2	-0.49732	0.066372	0.27619	None
5205	DNAJB9	0.426761	0.066415	0.276314	None
5206	PPM1N	-0.19096	0.066428	0.276317	None
5207	CCT5	-0.28506	0.066571	0.276846	None
5208	FDX1	0.178561	0.066581	0.276846	None
5209	ADORA3	-0.88197	0.066625	0.276976	None
5210	PGAM5	-0.11925	0.066653	0.27704	None
5211	GUCY1A3	-0.47794	0.066749	0.277386	None
5212	STX2	-0.24796	0.066789	0.277497	None
5213	KIAA2013	-0.14557	0.066847	0.277685	None
5214	BOLA3	-0.41012	0.066875	0.277748	None
5215	LOC10192	0.02081	0.066891	0.277759	None
5216	C10orf11	-0.37025	0.066951	0.277959	None
5217	THY1	0.172773	0.066984	0.278025	None
5218	CCDC92	-0.38243	0.066998	0.278025	None

5219	HNRNPUL1	-0.28239	0.067017	0.278025	None
5220	POU2F2	-0.18857	0.067019	0.278025	None
5221	KCTD3	0.417751	0.0671	0.278309	None
5222	ZNF3	0.121999	0.067134	0.278395	None
5223	DPYSL2	0.488424	0.067158	0.278443	None
5224	ITPRIPL2	-0.45582	0.067201	0.278561	None
5225	FLG-AS1	-0.0949	0.067223	0.278561	None
5226	ENC1	0.327567	0.067225	0.278561	None
5227	SERTAD2	-0.23747	0.067275	0.278716	None
5228	HCRP1	-0.46598	0.067296	0.278746	None
5229	BOLA2	-0.16244	0.067331	0.27884	None
5230	TTF1	-0.23277	0.067374	0.278917	None
5231	S100A13	-0.20737	0.067376	0.278917	None
5232	GLYCTK-A	0.018373	0.067393	0.278937	None
5233	CCDC51	-0.29891	0.067447	0.279105	None
5234	LOC10272	0.00914	0.067474	0.279165	None
5235	HN1	-0.30909	0.067603	0.279645	None
5236	LOC15368	-0.29597	0.067631	0.279709	None
5237	GEMIN4	-0.33713	0.067651	0.279735	None
5238	RPP25L	-0.2541	0.067683	0.279816	None
5239	GDPD1	-0.25998	0.067709	0.279869	None
5240	FASTKD1	-0.26628	0.067759	0.280022	None
5241	KIAA1147	-0.35292	0.067823	0.280233	None
5242	CD300A	-0.42557	0.067851	0.280258	None
5243	TXK	-0.39716	0.067861	0.280258	None
5244	MARVELD2	-0.33394	0.067867	0.280258	None
5245	EPB42	-0.91782	0.067882	0.280262	None
5246	TTC23L	0.041947	0.067994	0.280673	None
5247	KCNH1	0.006853	0.068012	0.280693	None
5248	C1QC	-1.15061	0.068036	0.280741	None
5249	NLRP3	0.509703	0.068077	0.280848	None
5250	RAN	0.145858	0.068094	0.280848	None
5251	B3GNT2	-0.45653	0.068101	0.280848	None
5252	KRTAP4-8	0.086426	0.068122	0.280878	None
5253	TP53TG1	-0.28915	0.068138	0.280891	None
5254	LINC00574	0.008213	0.068224	0.281195	None
5255	RAB20	-0.34867	0.068269	0.281324	None
5256	SIKE1	0.264021	0.068289	0.281353	None
5257	RP11-53O1	-0.32716	0.068335	0.281491	None
5258	NRBP1	-0.32824	0.068442	0.281876	None
5259	SESN2	-0.28609	0.068522	0.282155	None
5260	DNMT3B	-0.32812	0.068566	0.282279	None
5261	GK2	0.044847	0.068592	0.28233	None
5262	LOC72839	0.569017	0.068604	0.28233	None
5263	RRN3	0.239209	0.068629	0.282378	None
5264	PPIE	-0.27113	0.068686	0.282559	None
5265	ANAPC7	-0.15393	0.068707	0.282593	None
5266	ARL16	-0.30279	0.06872	0.282594	None
5267	TTC7B	-0.47696	0.06874	0.282609	None
5268	PTPN7	-0.27986	0.068769	0.282609	None
5269	ZMAT1	0.280071	0.068789	0.282609	None
5270	CLINT1	0.248555	0.068792	0.282609	None
5271	ESAM	-0.49579	0.068798	0.282609	None
5272	APEH	-0.17478	0.068802	0.282609	None
5273	PHACTR2	-0.3428	0.06882	0.282629	None
5274	CTSW	-0.5601	0.06889	0.2828	None
5275	ZNF225	-0.18531	0.068892	0.2828	None
5276	AES	-0.43072	0.068901	0.2828	None

5277	LILRA1	-0.23056	0.068988	0.283104	None
5278	ATIC	-0.23308	0.069006	0.283106	None
5279	PELP1	-0.24698	0.069015	0.283106	None
5280	MROH7	-0.40772	0.069041	0.28316	None
5281	MRPS36	-0.31113	0.069115	0.283411	None
5282	TXNRD1	-0.08055	0.069169	0.283559	None
5283	UBE2QL1	0.159128	0.069201	0.283559	None
5284	C20orf194	-0.32256	0.069207	0.283559	None
5285	ERCC3	0.216376	0.069207	0.283559	None
5286	DNAJB4	-0.35364	0.069217	0.283559	None
5287	TFEC	-0.65435	0.069299	0.283778	None
5288	UFL1	0.503363	0.069303	0.283778	None
5289	OSR1	0.022688	0.06931	0.283778	None
5290	FAM160A1	0.063041	0.069346	0.283875	None
5291	ZFYVE1	-0.21035	0.069369	0.283913	None
5292	LRRC16A	-0.17178	0.069398	0.283976	None
5293	ZNF354A	-0.2772	0.069505	0.284364	None
5294	SWAP70	0.281659	0.069552	0.284501	None
5295	ZBTB4	-0.18195	0.069592	0.284612	None
5296	FAM50A	-0.3995	0.069637	0.284733	None
5297	FLJ45482	-0.42944	0.069648	0.284733	None
5298	PURG	-0.1768	0.069702	0.284897	None
5299	TCAM1P	-0.61014	0.069731	0.284964	None
5300	TRAPPC9	-0.08339	0.069761	0.285034	None
5301	SNHG22	0.094929	0.069785	0.285051	None
5302	NPHP3	-0.4157	0.069792	0.285051	None
5303	FAM50B	-0.36794	0.069846	0.285219	None
5304	LOC10050!	0.024189	0.069908	0.285418	None
5305	TRIAP1	-0.20825	0.069946	0.285493	None
5306	LRRC61	-0.24064	0.069958	0.285493	None
5307	TMEM239	0.04847	0.06997	0.285493	None
5308	LOC10028!	0.076363	0.069979	0.285493	None
5309	C15orf57	-0.31847	0.070064	0.285787	None
5310	FPR2	0.31455	0.070145	0.28602	None
5311	ANKRD6	-0.42921	0.070148	0.28602	None
5312	COG3	0.158086	0.070188	0.286129	None
5313	MRGPRX1	0.021554	0.070261	0.286373	None
5314	AC016831.	-0.31585	0.070286	0.286419	None
5315	TIMM8B	-0.25798	0.070375	0.286696	None
5316	LOC10050(	0.03643	0.07038	0.286696	None
5317	LOC25739(	-0.34575	0.070428	0.286837	None
5318	CEACAM5	0.029541	0.070459	0.286903	None
5319	TNS3	-0.18061	0.070472	0.286903	None
5320	TMEM161E	0.243032	0.070484	0.286903	None
5321	SNX11	-0.28143	0.070511	0.28696	None
5322	FAM222B	-0.2284	0.070574	0.287163	None
5323	AXIN1	-0.15823	0.070587	0.287163	None
5324	AGAP6	-0.19261	0.070611	0.287205	None
5325	SYK	0.304233	0.070636	0.287255	None
5326	KPNA2	0.268692	0.070655	0.287259	None
5327	MEIS1	-0.46774	0.070675	0.287259	None
5328	LINC00184	0.075515	0.070677	0.287259	None
5329	PRUNE	-0.24639	0.070724	0.287381	None
5330	DNMT3A	-0.33402	0.07074	0.287381	None
5331	TPM4	0.430274	0.07076	0.287381	None
5332	C4orf29	-0.32809	0.07076	0.287381	None
5333	EFHD1	0.023996	0.070787	0.2874	None
5334	PCM1	0.225024	0.070791	0.2874	None

5335	RTEL1	-0.08036	0.0709	0.287787	None
5336	ZPBP	0.016777	0.071004	0.288154	None
5337	DPH5	0.279159	0.071039	0.28824	None
5338	LOC10192	0.012532	0.071107	0.288463	None
5339	FCAMR	0.002945	0.071146	0.288563	None
5340	UNC50	-0.15966	0.071158	0.288563	None
5341	ST20	-0.27646	0.071185	0.288585	None
5342	PPME1	-0.27307	0.07119	0.288585	None
5343	AHCYL2	0.131196	0.071203	0.288585	None
5344	HMGB3	0.366163	0.071237	0.288669	None
5345	ARL6IP5	-0.25752	0.071276	0.288771	None
5346	NSDHL	-0.20298	0.071401	0.289224	None
5347	BLOC1S4	-0.21063	0.071423	0.28926	None
5348	GCH1	0.498207	0.071467	0.289383	None
5349	LOC38919	0.097741	0.071511	0.289505	None
5350	CAPZA1	0.187421	0.071637	0.289949	None
5351	AADAT	-0.27171	0.071647	0.289949	None
5352	CCRL2	0.58519	0.071719	0.290186	None
5353	SZT2	-0.19826	0.071787	0.290407	None
5354	GRAMD1C	0.532144	0.071803	0.290417	None
5355	ZNF763	-0.20424	0.071828	0.290422	None
5356	NIPSNAP3	0.372583	0.071831	0.290422	None
5357	GCSAML	-0.64801	0.071887	0.290494	None
5358	ABHD10	-0.22763	0.071887	0.290494	None
5359	CA4	0.179462	0.071889	0.290494	None
5360	ST3GAL2	-0.20579	0.071916	0.290528	None
5361	IRF7	-0.47303	0.071934	0.290528	None
5362	HOOK3	0.225017	0.071938	0.290528	None
5363	TNFRSF10/	-0.21844	0.071955	0.290545	None
5364	ABCD3	0.247994	0.072027	0.290779	None
5365	TMEM87B	-0.2516	0.072046	0.290801	None
5366	RP11-669(	0.090957	0.072062	0.290814	None
5367	MTMR3	-0.11111	0.072098	0.290905	None
5368	LONRF3	0.160331	0.072141	0.291022	None
5369	SLC27A3	0.340077	0.072155	0.291022	None
5370	TOM1L1	-0.37425	0.072167	0.291022	None
5371	MRRF	-0.28843	0.072241	0.291248	None
5372	RP11-203E	0.037076	0.07225	0.291248	None
5373	AX747730	-0.40386	0.072342	0.291561	None
5374	APTX	-0.27623	0.072395	0.291699	None
5375	PPCDC	-0.32389	0.072403	0.291699	None
5376	FOXJ1	-0.11965	0.072436	0.291777	None
5377	MRPL4	-0.39932	0.07247	0.291863	None
5378	TBC1D8B	-0.35046	0.07256	0.292168	None
5379	PHTF1	0.29385	0.072628	0.29239	None
5380	RP4-710M	0.244777	0.072656	0.292446	None
5381	SERPINB8	0.246117	0.072684	0.292504	None
5382	COX15	-0.26537	0.072732	0.292645	None
5383	TSTD1	-0.27006	0.072821	0.292946	None
5384	NFATC2	-0.15519	0.072856	0.292962	None
5385	LOC10192	0.049323	0.07286	0.292962	None
5386	TMEM176E	-0.36511	0.072871	0.292962	None
5387	IKBKG	-0.1179	0.072879	0.292962	None
5388	ZFAND5	-0.36906	0.07294	0.293106	None
5389	LPP	-0.40863	0.072947	0.293106	None
5390	TOR2A	-0.10614	0.072955	0.293106	None
5391	ZNF197	-0.18362	0.073009	0.293228	None
5392	CLEC16A	-0.13515	0.073016	0.293228	None

5393	TTYH3	-0.36855	0.073026	0.293228	None
5394	ATP8B2	0.226484	0.073074	0.293256	None
5395	SCG2	0.155914	0.073079	0.293256	None
5396	CIDEA	0.021347	0.073094	0.293256	None
5397	RPA2	-0.25509	0.073098	0.293256	None
5398	JKAMP	-0.1741	0.073101	0.293256	None
5399	AIM1	0.598796	0.073156	0.293424	None
5400	TNPO2	-0.25696	0.07317	0.293424	None
5401	APOBEC3D	0.064552	0.073254	0.293692	None
5402	WARS2	-0.25937	0.073274	0.293692	None
5403	RP11-339E	-0.21217	0.073296	0.293692	None
5404	ACAA1	-0.20421	0.0733	0.293692	None
5405	ZNF268	-0.32512	0.073304	0.293692	None
5406	NFATC1	0.165663	0.073403	0.294032	None
5407	FAM69B	0.153743	0.073453	0.294181	None
5408	PRPF4	-0.26815	0.073485	0.294231	None
5409	ZNF77	-0.17184	0.073493	0.294231	None
5410	PPFIBP1	0.464081	0.073516	0.294269	None
5411	DIRAS3	-0.60594	0.073564	0.294355	None
5412	FGF13	-0.90412	0.073565	0.294355	None
5413	ITPKC	-0.13138	0.073619	0.294518	None
5414	LAG3	-0.40037	0.073672	0.294675	None
5415	HBE1	-0.22505	0.073694	0.294703	None
5416	PPP2R4	-0.13162	0.073706	0.294703	None
5417	MGC12916	0.15283	0.073774	0.294918	None
5418	PLEKHA7	-0.31385	0.073788	0.294921	None
5419	PTDSS1	-0.22778	0.0739	0.295314	None
5420	MYL9	-0.26583	0.073925	0.295358	None
5421	PSAP	-0.45193	0.073989	0.295559	None
5422	KBTBD7	-0.42043	0.074008	0.29558	None
5423	CDKN1C	-0.45362	0.074064	0.29575	None
5424	FTSJ3	-0.22482	0.074096	0.295798	None
5425	CTB-25B13	-0.1445	0.074103	0.295798	None
5426	C6orf89	-0.35921	0.074119	0.295808	None
5427	FAM199X	-0.32754	0.074142	0.295843	None
5428	OK/SW-CL	0.004066	0.074167	0.295889	None
5429	PROSC	-0.33007	0.074378	0.296676	None
5430	TRIM38	0.240398	0.074406	0.296735	None
5431	RP11-1191	0.265631	0.074554	0.297269	None
5432	PRKG2	0.547354	0.07459	0.297311	None
5433	LOC15117	-0.25776	0.074592	0.297311	None
5434	CSNK1E	0.190123	0.07463	0.297402	None
5435	ITGA2	-0.33243	0.074642	0.297402	None
5436	OTUD1	-0.49941	0.074694	0.297514	None
5437	GUSBP5	0.336391	0.074698	0.297514	None
5438	PIK3AP1	0.307837	0.074721	0.297553	None
5439	RP13-39P1	-0.09006	0.074857	0.298037	None
5440	RINT1	0.262312	0.074901	0.298158	None
5441	FAM174B	-0.33207	0.075015	0.298484	None
5442	BCL9	-0.12559	0.075027	0.298484	None
5443	AC009120.	-0.30131	0.075028	0.298484	None
5444	RPL37A	-0.51443	0.075044	0.298484	None
5445	IRAK2	0.061263	0.075052	0.298484	None
5446	PSD3	0.59383	0.075122	0.29871	None
5447	MYCBP2	0.208781	0.075238	0.299066	None
5448	ETHE1	-0.34286	0.07524	0.299066	None
5449	CCDC112	0.32306	0.075265	0.299112	None
5450	CAPNS2	0.079355	0.075347	0.299383	None

5451	EP300	-0.30886	0.075425	0.299637	None
5452	MANF	0.248492	0.075441	0.299647	None
5453	ASGR1	-0.16615	0.075519	0.299903	None
5454	FGF18	0.030095	0.075539	0.299927	None
5455	SLC2A4RG	-0.17133	0.07561	0.300152	None
5456	PSKH1	-0.13473	0.075646	0.300186	None
5457	ZNF354C	-0.13296	0.075646	0.300186	None
5458	LOC10028	-0.17426	0.075674	0.300241	None
5459	GID4	-0.16722	0.075699	0.300248	None
5460	SCPEP1	0.283931	0.075703	0.300248	None
5461	ZFHX2	0.048396	0.075829	0.300563	None
5462	AQP2	0.051104	0.075861	0.300563	None
5463	GCNT1	-0.35095	0.075877	0.300563	None
5464	C6orf163	-0.17999	0.075879	0.300563	None
5465	PRDM12	0.09094	0.075881	0.300563	None
5466	SNCA	-0.36445	0.075887	0.300563	None
5467	LOC10272	0.709799	0.075887	0.300563	None
5468	C17orf104	-0.15182	0.075898	0.300563	None
5469	SLC2A6	-0.33104	0.075908	0.300563	None
5470	KCNQ4	-0.15384	0.076036	0.301017	None
5471	FEZ1	-0.56003	0.076099	0.301212	None
5472	KIF1B	-0.43611	0.076125	0.301259	None
5473	SPIRE1	-0.39926	0.076212	0.301546	None
5474	RASL10B	-0.10016	0.076225	0.301546	None
5475	ZNF100	0.241815	0.076249	0.301585	None
5476	DMTN	-0.39293	0.076283	0.301635	None
5477	FENDRR	0.065654	0.076304	0.301635	None
5478	LINS	-0.20459	0.076304	0.301635	None
5479	HFM1	0.16338	0.07637	0.301842	None
5480	XYLB	0.061838	0.076399	0.301903	None
5481	ITIH4	-0.15893	0.076439	0.301956	None
5482	TMEM87A	0.165657	0.076441	0.301956	None
5483	FLJ30679	0.003563	0.076514	0.30219	None
5484	SLC31A1	-0.25879	0.076555	0.302285	None
5485	RETSAT	-0.30839	0.076566	0.302285	None
5486	LOC28391	0.076376	0.076698	0.302751	None
5487	C8orf48	-0.14055	0.076758	0.302935	None
5488	COPB1	0.161148	0.07678	0.302966	None
5489	LY86	0.816536	0.076847	0.303157	None
5490	ATP6V0D2	-0.76078	0.076857	0.303157	None
5491	B4GALT7	-0.19773	0.076889	0.303195	None
5492	OTOGL	0.009994	0.076894	0.303195	None
5493	NGRN	0.229615	0.07691	0.303201	None
5494	IMP4	-0.27871	0.076948	0.303295	None
5495	FUT2	0.033672	0.076973	0.303314	None
5496	RP4-798A1	-0.31965	0.07698	0.303314	None
5497	TSC22D2	0.151173	0.077032	0.303461	None
5498	PDSS2	-0.24615	0.077068	0.303548	None
5499	DNAJC5	-0.31879	0.077087	0.303569	None
5500	OR2H2	0.058049	0.077101	0.303569	None
5501	FLCN	0.137736	0.077174	0.303802	None
5502	WDR82	-0.14663	0.077235	0.303984	None
5503	LOC10192	0.011365	0.077281	0.30411	None
5504	HOXC11	0.085394	0.077417	0.30459	None
5505	NUDCD3	0.112347	0.077495	0.304754	None
5506	AXDND1	0.020361	0.077517	0.304754	None
5507	CDH4	-0.15602	0.077519	0.304754	None
5508	MYOD1	0.077749	0.077523	0.304754	None

5509	GADD45G	0.337987	0.07754	0.304754	None
5510	ADI1	-0.38766	0.077543	0.304754	None
5511	ALG2	0.156859	0.077596	0.304905	None
5512	NKX3-1	0.043242	0.077674	0.305148	None
5513	PGS1	-0.10459	0.077686	0.305148	None
5514	TTC39C	-0.30463	0.077707	0.305178	None
5515	TBCD	0.332018	0.077765	0.305329	None
5516	CDC42	0.315513	0.077774	0.305329	None
5517	NSUN7	-0.26988	0.07779	0.305336	None
5518	NKX2-3	-0.73096	0.077824	0.305414	None
5519	FAM201A	-0.31204	0.07785	0.305461	None
5520	ACN9	0.349853	0.07788	0.305471	None
5521	RAD54L2	0.193276	0.077881	0.305471	None
5522	LOC10272	-0.23414	0.07794	0.305647	None
5523	CUL5	-0.21685	0.078054	0.306041	None
5524	MNT	-0.0846	0.078095	0.306147	None
5525	WDR3	-0.1804	0.078145	0.30624	None
5526	CITED2	-0.54199	0.078147	0.30624	None
5527	RP11-999E	-0.42913	0.078246	0.30656	None
5528	NOTCH3	-0.07541	0.078257	0.30656	None
5529	STYXL1	-0.31742	0.078276	0.306578	None
5530	LOC28581	0.394097	0.078295	0.306596	None
5531	LOC10050	-0.31004	0.078343	0.306731	None
5532	RAB39B	-0.22708	0.078415	0.306955	None
5533	CLK3	-0.2172	0.078473	0.307127	None
5534	FKBP1B	-0.70174	0.07854	0.307287	None
5535	FCGR2C	-0.42418	0.078542	0.307287	None
5536	MYH1	0.012479	0.078558	0.307293	None
5537	RP5-991G	-0.23858	0.078642	0.307566	None
5538	MX1	-0.75078	0.078659	0.307576	None
5539	C16orf91	0.269383	0.078707	0.30771	None
5540	LOC10193	-0.47069	0.078739	0.307779	None
5541	CASP8AP2	-0.1751	0.078822	0.308048	None
5542	FAM181B	-0.00993	0.078837	0.308049	None
5543	TFB2M	0.052313	0.078854	0.308049	None
5544	EOGT	0.445234	0.078865	0.308049	None
5545	C11orf58	0.201021	0.078899	0.308126	None
5546	DNAJC6	-0.32448	0.078924	0.308168	None
5547	GPC6	-0.41233	0.079042	0.308476	None
5548	PCOLCE2	-0.65824	0.079055	0.308476	None
5549	HECW1	0.036207	0.079056	0.308476	None
5550	TMEM129	-0.2298	0.07906	0.308476	None
5551	SRR	-0.31718	0.079098	0.308569	None
5552	RARA	-0.18749	0.079141	0.308668	None
5553	CKS2	-0.25416	0.079152	0.308668	None
5554	TBC1D12	-0.35108	0.079186	0.30873	None
5555	DNAJC2	-0.12805	0.079196	0.30873	None
5556	LRRC38	0.071139	0.079247	0.308801	None
5557	TMCO6	-0.36404	0.079255	0.308801	None
5558	MYZAP	-0.56337	0.079257	0.308801	None
5559	ENPP3	0.171719	0.079273	0.308807	None
5560	CISH	-0.29667	0.079314	0.308888	None
5561	CTA-29F1	0.394608	0.079322	0.308888	None
5562	ZNF594	-0.16377	0.079343	0.308898	None
5563	PML	-0.13163	0.079354	0.308898	None
5564	LRRC2	-0.26718	0.079452	0.309225	None
5565	ZC3H7B	-0.10704	0.079528	0.309466	None
5566	GLTSCR1L	-0.2153	0.079575	0.309504	None

5567	PIM3	0.396951	0.07958	0.309504	None
5568	SPRY1	0.355939	0.079581	0.309504	None
5569	CCDC37-A	0.022451	0.079621	0.309606	None
5570	TRA2A	0.328708	0.079674	0.309714	None
5571	TJP3	-0.04814	0.079685	0.309714	None
5572	FGFR2	-0.01465	0.079692	0.309714	None
5573	CCT6B	0.297667	0.079763	0.309935	None
5574	MRPL16	-0.22482	0.079821	0.310103	None
5575	PLCB3	-0.14909	0.079849	0.310127	None
5576	RNF145	-0.59575	0.079855	0.310127	None
5577	GPR75	0.087644	0.079894	0.310183	None
5578	HEXA	-0.26974	0.079898	0.310183	None
5579	GNG12	-0.67593	0.079936	0.310197	None
5580	TBC1D17	-0.12293	0.079943	0.310197	None
5581	INSRR	0.007595	0.079945	0.310197	None
5582	POLM	-0.22252	0.079974	0.310233	None
5583	CSF1R	-0.67133	0.079983	0.310233	None
5584	FHL2	-0.78335	0.080047	0.310425	None
5585	POLR2F	0.160717	0.080067	0.310446	None
5586	GPHN	-0.29695	0.080115	0.310576	None
5587	ZDHHC23	-0.44895	0.080144	0.310634	None
5588	PLXNB3	0.047783	0.080247	0.310977	None
5589	FOXRED2	-0.19867	0.080302	0.311138	None
5590	NUB1	0.297729	0.08032	0.311149	None
5591	C7orf49	-0.30064	0.080371	0.311293	None
5592	PRKRA	0.245151	0.080389	0.311305	None
5593	ZBTB11	-0.31711	0.080403	0.311305	None
5594	GALNT5	-0.11481	0.080429	0.311305	None
5595	GPRC5D	0.019308	0.080432	0.311305	None
5596	PKP4	-0.48258	0.080493	0.311443	None
5597	QPRT	-0.43034	0.080503	0.311443	None
5598	ASPA	-0.29427	0.080518	0.311443	None
5599	CAMTA1	-0.22428	0.080525	0.311443	None
5600	STAG3L4	0.19601	0.080539	0.311443	None
5601	EGF	-0.51422	0.080555	0.311446	None
5602	FAF1	-0.16126	0.080616	0.3116	None
5603	KCNH2	-0.60092	0.080624	0.3116	None
5604	MCM9	-0.26918	0.080638	0.3116	None
5605	TMUB1	-0.26127	0.08068	0.311622	None
5606	TMEM42	-0.2455	0.080685	0.311622	None
5607	ATN1	0.014031	0.080686	0.311622	None
5608	FLT1	0.247138	0.080708	0.311651	None
5609	TMEM80	-0.21689	0.080729	0.311664	None
5610	BCL2L11	0.436819	0.080741	0.311664	None
5611	RHOH	0.444556	0.080819	0.311872	None
5612	DZIP1	-0.40133	0.080836	0.311872	None
5613	UBE4A	-0.14921	0.080838	0.311872	None
5614	AKR1A1	-0.20744	0.080854	0.311881	None
5615	LEMD3	0.209963	0.080874	0.3119	None
5616	CTBP1	-0.26695	0.080896	0.311931	None
5617	C8orf58	-0.11645	0.08094	0.312046	None
5618	CNKSR1	-0.18735	0.080995	0.312201	None
5619	SNX15	-0.16903	0.081079	0.312469	None
5620	TAB2	-0.29759	0.081106	0.31252	None
5621	AHI1	0.143186	0.081145	0.312614	None
5622	GJD2	0.105504	0.081174	0.312627	None
5623	C14orf1	-0.57483	0.081178	0.312627	None
5624	LOC44102!	0.063179	0.081247	0.312838	None

5625	TMEM255A	-0.62392	0.081283	0.312901	None
5626	MRPL27	-0.31433	0.081293	0.312901	None
5627	GEMIN2	-0.39036	0.081325	0.312901	None
5628	C5orf63	-0.22848	0.081329	0.312901	None
5629	PDZD2	-0.40911	0.081357	0.312901	None
5630	KDM4C	0.276939	0.081358	0.312901	None
5631	CPO	0.037823	0.081364	0.312901	None
5632	CYR61	0.232569	0.081419	0.313057	None
5633	DENND5B	-0.32261	0.08145	0.313121	None
5634	ATF6B	0.161331	0.081473	0.313151	None
5635	FTO	-0.16859	0.081528	0.313259	None
5636	RIPK2	-0.27199	0.08153	0.313259	None
5637	LEPREL1	-0.293	0.081579	0.313336	None
5638	CDK7	-0.22841	0.081579	0.313336	None
5639	CCDC78	-0.09649	0.081648	0.313444	None
5640	GAFA3	0.026223	0.08165	0.313444	None
5641	GUCY1B3	-0.42744	0.08165	0.313444	None
5642	ABCD4	0.093838	0.081762	0.313804	None
5643	ELF5	-0.27711	0.081773	0.313804	None
5644	ITK	0.19159	0.081797	0.313839	None
5645	PRSS36	0.077547	0.081852	0.313937	None
5646	TRMT10B	-0.16188	0.081855	0.313937	None
5647	CCND1	-0.53212	0.081884	0.313937	None
5648	ZNF573	-0.1799	0.081891	0.313937	None
5649	RNF149	-0.56979	0.081895	0.313937	None
5650	POLR2I	-0.30274	0.081919	0.313973	None
5651	AK021977	0.549294	0.082018	0.314257	None
5652	RNPC3	-0.38904	0.082022	0.314257	None
5653	OAS2	-0.49989	0.082075	0.314388	None
5654	MGRN1	-0.17932	0.082101	0.314388	None
5655	GDF5	0.079064	0.082104	0.314388	None
5656	CCDC163P	0.150572	0.082128	0.314388	None
5657	MIATNB	-0.19694	0.082129	0.314388	None
5658	CPS1-IT1	0.054417	0.082145	0.314394	None
5659	CCHCR1	-0.17226	0.082209	0.314583	None
5660	LOC10050	0.036845	0.08225	0.314686	None
5661	NINJ2	-0.37301	0.082337	0.314927	None
5662	IK	0.273818	0.082342	0.314927	None
5663	SLC40A1	-0.37127	0.082364	0.314957	None
5664	GPR115	0.017372	0.082438	0.315131	None
5665	KDM6A	0.36288	0.082439	0.315131	None
5666	THAP2	-0.23648	0.082551	0.31541	None
5667	C9orf64	-0.24222	0.082552	0.31541	None
5668	ORMDL1	-0.32623	0.082557	0.31541	None
5669	ACTB	-0.23914	0.08257	0.31541	None
5670	LL22NC03-	-0.6732	0.082597	0.315411	None
5671	AMIGO1	-0.14245	0.08262	0.315411	None
5672	CENPF	0.443361	0.082623	0.315411	None
5673	RP11-49K2	0.072374	0.082629	0.315411	None
5674	CCNL2	-0.27897	0.082651	0.315425	None
5675	ELSPBP1	0.010647	0.082671	0.315425	None
5676	B3GAT2	0.234288	0.082676	0.315425	None
5677	ZNF26	0.272193	0.082695	0.315442	None
5678	HSPE1	-0.27437	0.082721	0.315442	None
5679	PHACTR3	-0.1505	0.08273	0.315442	None
5680	PPP5C	-0.08138	0.082739	0.315442	None
5681	CRKL	-0.09437	0.082794	0.315587	None
5682	HLA-DOB	-0.26127	0.082806	0.315587	None

5683	CDC42SE1	-0.20982	0.082862	0.315743	None
5684	SIRT7	-0.20783	0.08289	0.315781	None
5685	RRN3P1	-0.29427	0.082901	0.315781	None
5686	VARS	-0.20428	0.082956	0.315892	None
5687	LOC10027	-0.08814	0.082959	0.315892	None
5688	TSHB	0.012281	0.082996	0.31593	None
5689	CRIP1	0.710201	0.083009	0.31593	None
5690	ZNF200	-0.31347	0.083013	0.31593	None
5691	PYROXD2	-0.13326	0.083027	0.31593	None
5692	NUDT9	-0.25304	0.083062	0.316007	None
5693	RCN3	-0.15074	0.083097	0.316084	None
5694	FBXO15	0.321207	0.083122	0.316124	None
5695	RP11-271(	0.41545	0.083156	0.316149	None
5696	GTF2E2	-0.21248	0.083158	0.316149	None
5697	DUS2	-0.19282	0.08319	0.316197	None
5698	R3HCC1L	-0.10362	0.0832	0.316197	None
5699	RP11-1012	0.029844	0.083214	0.316197	None
5700	LPAR1	-0.44001	0.083232	0.316207	None
5701	AF001548.!	-0.26062	0.083255	0.316242	None
5702	F3	0.473888	0.083273	0.316255	None
5703	FDXACB1	-0.21953	0.083339	0.316406	None
5704	LAMTOR2	-0.39708	0.083342	0.316406	None
5705	CDKN2B	-0.26654	0.083514	0.317002	None
5706	C19orf83	-0.20296	0.083561	0.317097	None
5707	CXCR3	0.075209	0.083568	0.317097	None
5708	C7orf60	0.241718	0.083597	0.317151	None
5709	RP5-894A1	-0.35042	0.083632	0.317229	None
5710	LOC72881!	-0.38603	0.083676	0.31734	None
5711	OSBPL9	-0.1507	0.083707	0.3174	None
5712	RPTOR	-0.24968	0.083763	0.317558	None
5713	SLC18B1	-0.5767	0.083782	0.317574	None
5714	ABHD17C	0.52313	0.083867	0.317841	None
5715	CRYL1	-0.42471	0.083907	0.317938	None
5716	CD244	-0.1848	0.08394	0.318005	None
5717	NFIX	-0.16528	0.083969	0.31806	None
5718	SOX21	0.022224	0.084046	0.318296	None
5719	RDH11	-0.15349	0.084086	0.318392	None
5720	CFL1	-0.3193	0.084127	0.318485	None
5721	BC043356	-0.23686	0.084143	0.318485	None
5722	RAB35	-0.17068	0.084155	0.318485	None
5723	CCDC9	0.14594	0.084199	0.318598	None
5724	PPFIA3	-0.15259	0.084216	0.318606	None
5725	MRGBP	-0.20888	0.084274	0.318769	None
5726	TLR5	-0.38364	0.084377	0.319105	None
5727	CRBN	-0.18041	0.084402	0.319122	None
5728	LINC00515	-0.16617	0.084411	0.319122	None
5729	LAMB2	-0.45612	0.084502	0.319407	None
5730	AFF1	0.191358	0.084674	0.320002	None
5731	GADD45A	0.280698	0.084802	0.320431	None
5732	VN1R2	0.005901	0.084819	0.320441	None
5733	FAM120C	0.178287	0.084855	0.320472	None
5734	TTC13	-0.24905	0.084857	0.320472	None
5735	LOC10192	0.031141	0.084873	0.320475	None
5736	TADA2A	-0.24357	0.084919	0.320595	None
5737	LOC10050(	0.508084	0.084952	0.320607	None
5738	WARS	-0.40576	0.084952	0.320607	None
5739	RFX5	-0.16066	0.085005	0.320749	None
5740	DDX58	-0.38751	0.085042	0.32079	None

5741	LOC10013:	-0.0955	0.085045	0.32079	None
5742	C9orf85	-0.21501	0.085091	0.320812	None
5743	ZEB2	0.412196	0.085112	0.320812	None
5744	LOC10050:	-0.18188	0.085123	0.320812	None
5745	SLC38A5	-0.37769	0.085124	0.320812	None
5746	LFNG	0.107397	0.085125	0.320812	None
5747	BOC	-0.06299	0.085173	0.320912	None
5748	STOM	-0.31347	0.085181	0.320912	None
5749	GIT1	0.236594	0.085198	0.320919	None
5750	EPHX2	-0.40981	0.08524	0.321021	None
5751	TAF1	-0.34494	0.085287	0.32106	None
5752	RNF139	0.17813	0.085287	0.32106	None
5753	GNGT2	-0.41024	0.085295	0.32106	None
5754	SHISA5	-0.22155	0.085325	0.321095	None
5755	NEURL1B	-0.47217	0.085334	0.321095	None
5756	TRPV5	0.030401	0.085359	0.321135	None
5757	CNGA4	0.061977	0.085381	0.321162	None
5758	METTL2B	-0.16904	0.085409	0.321209	None
5759	P2RY13	-0.71201	0.085424	0.32121	None
5760	VSIG10L	-0.23544	0.08545	0.321254	None
5761	SLC39A14	0.095001	0.085483	0.321322	None
5762	LINC00158	0.052354	0.085512	0.321374	None
5763	DTX1	0.223838	0.085581	0.321577	None
5764	ZNF835	-0.12312	0.085618	0.321628	None
5765	LOC72968:	-0.09384	0.085624	0.321628	None
5766	BATF3	-0.30245	0.085682	0.321792	None
5767	POU6F2-A	0.007654	0.085748	0.321983	None
5768	DNMBP	-0.2895	0.085861	0.322333	None
5769	ZNF396	0.009452	0.085871	0.322333	None
5770	FOXP1-IT1	0.351758	0.085898	0.322378	None
5771	EPT1	0.295136	0.085932	0.322428	None
5772	TCHH	-0.6157	0.085953	0.322428	None
5773	SRBD1	-0.2291	0.085963	0.322428	None
5774	RNF168	0.145603	0.085982	0.322428	None
5775	ARL15	-0.22266	0.085986	0.322428	None
5776	SPIB	0.392206	0.086036	0.322562	None
5777	WNT7B	-0.08843	0.086096	0.322729	None
5778	CYP2E1	-0.30736	0.086122	0.322771	None
5779	CRELD1	-0.32357	0.086146	0.322805	None
5780	STRN4	-0.24228	0.086203	0.322964	None
5781	CD83	0.561245	0.086277	0.323141	None
5782	XKR6	-0.09196	0.08628	0.323141	None
5783	PFKP	-0.15076	0.086329	0.323267	None
5784	C4orf27	-0.1176	0.086409	0.323511	None
5785	DOCK10	0.317455	0.086488	0.323751	None
5786	LOC10050!	0.021074	0.086506	0.323761	None
5787	LSG1	-0.22842	0.086522	0.323765	None
5788	FAM210A	-0.23344	0.086539	0.323774	None
5789	STX8	-0.09993	0.086646	0.324119	None
5790	RP11-363E	-0.33445	0.086704	0.324231	None
5791	STX16	-0.18805	0.086706	0.324231	None
5792	GP9	-0.38633	0.086727	0.324249	None
5793	CSN1S1	-0.51683	0.086743	0.324249	None
5794	TNK2	0.142148	0.08676	0.324249	None
5795	NSMCE4A	0.396069	0.086771	0.324249	None
5796	BSDC1	-0.20505	0.086845	0.324424	None
5797	SLC24A1	-0.05792	0.086848	0.324424	None
5798	VEZF1	0.188771	0.086899	0.324505	None

5799	SIMC1	-0.30509	0.086899	0.324505	None
5800	UBAC1	-0.33545	0.086925	0.324515	None
5801	SLC24A3	-0.67896	0.086938	0.324515	None
5802	ANKZF1	-0.22378	0.086961	0.324515	None
5803	DGCR2	-0.10567	0.086962	0.324515	None
5804	LOC10012!	0.016292	0.087013	0.324605	None
5805	TMEM37	-0.3778	0.087024	0.324605	None
5806	SDC3	-0.46646	0.087031	0.324605	None
5807	MMADHC	-0.15735	0.08706	0.324658	None
5808	TYROBP	-0.68168	0.087165	0.324994	None
5809	ITGA7	0.107728	0.087185	0.324997	None
5810	VPS33B	-0.19048	0.087196	0.324997	None
5811	CIC	-0.29997	0.087239	0.3251	None
5812	GDPD3	-0.2073	0.087272	0.32517	None
5813	VPS36	-0.09486	0.087333	0.325339	None
5814	TMEM120E	-0.13309	0.087394	0.325402	None
5815	BANF1	-0.3168	0.087395	0.325402	None
5816	MAOA	-0.25283	0.087395	0.325402	None
5817	MGAT1	-0.27419	0.087418	0.325426	None
5818	EPB41L5	-0.16281	0.087432	0.325426	None
5819	PSTPIP1	-0.275	0.087483	0.325531	None
5820	CMKLR1	-0.42499	0.08749	0.325531	None
5821	PDZD3	0.027183	0.087506	0.325535	None
5822	CTBS	-0.30372	0.087566	0.325703	None
5823	EDC3	-0.22965	0.087606	0.325795	None
5824	TTF2	0.195952	0.087633	0.325838	None
5825	TNFRSF17	-0.50955	0.087656	0.325869	None
5826	UQCC1	-0.1942	0.087679	0.3259	None
5827	GIT2	-0.28205	0.087858	0.326507	None
5828	TMEM68	-0.25013	0.087913	0.326656	None
5829	IKBKE	-0.12111	0.087928	0.326656	None
5830	LOC44011	0.030262	0.088067	0.327118	None
5831	PRDX5	0.250706	0.088096	0.327167	None
5832	TRIM16	-0.26893	0.088132	0.327247	None
5833	DYNC1I2	-0.39502	0.088154	0.327272	None
5834	USF1	-0.26588	0.088254	0.327497	None
5835	SMIM10	-0.45143	0.088263	0.327497	None
5836	SMOC2	0.064725	0.088281	0.327497	None
5837	CYP7B1	-0.41988	0.088286	0.327497	None
5838	PORCN	-0.20454	0.08829	0.327497	None
5839	MRPS25	-0.19126	0.08833	0.327589	None
5840	GPR126	-0.39482	0.088375	0.327697	None
5841	E2F3	0.182958	0.088496	0.328092	None
5842	LRIG2	-0.13107	0.088528	0.328155	None
5843	FDPS	0.209546	0.088615	0.328379	None
5844	GSG2	0.282064	0.088647	0.328379	None
5845	RFXAP	-0.2056	0.088663	0.328379	None
5846	NBL1	-0.34009	0.088663	0.328379	None
5847	PPP1R3C	-0.21433	0.088665	0.328379	None
5848	RNASE3	0.824602	0.088695	0.328435	None
5849	LINC0069C	0.003427	0.088726	0.328494	None
5850	ARHGAP44	-0.18571	0.088741	0.328494	None
5851	PCSK9	0.030741	0.088926	0.329122	None
5852	ANKMY1	-0.17392	0.088948	0.329146	None
5853	IRF2BP1	0.131215	0.088965	0.329153	None
5854	SFR1	-0.23556	0.089067	0.329435	None
5855	MB21D2	0.358116	0.089083	0.329435	None
5856	PLEC	0.054813	0.089087	0.329435	None

5857	CLMN	-0.16414	0.089137	0.329553	None
5858	ILF2	0.199648	0.089149	0.329553	None
5859	PAK6	-0.23088	0.089232	0.329791	None
5860	IGSF21	-0.44405	0.089244	0.329791	None
5861	TSPY26P	-0.14594	0.089294	0.32992	None
5862	ITPKB	-0.28035	0.089329	0.329994	None
5863	PDZD4	0.033015	0.089384	0.330142	None
5864	RP11-158(	0.565784	0.089408	0.330172	None
5865	ME1	-0.9767	0.089423	0.330172	None
5866	KCNK17	-0.61412	0.089532	0.330504	None
5867	FOXA3	-0.20702	0.089551	0.330504	None
5868	SYTL1	-0.50832	0.089559	0.330504	None
5869	DUSP13	0.084443	0.089604	0.330613	None
5870	SUGCT	-0.20249	0.089673	0.330812	None
5871	ST6GALNA	-0.18643	0.08981	0.33124	None
5872	OR51E1	0.02884	0.08982	0.33124	None
5873	DCBLD1	-0.28073	0.089835	0.33124	None
5874	LOC10192	0.040231	0.089851	0.331243	None
5875	LMF2	-0.17782	0.089906	0.33132	None
5876	DLAT	-0.33969	0.089914	0.33132	None
5877	IQGAP3	0.057519	0.089918	0.33132	None
5878	UBXN11	0.1504	0.089941	0.331348	None
5879	HLA-DRB4	0.912086	0.08999	0.331457	None
5880	SPTY2D1	0.252419	0.090001	0.331457	None
5881	TRMT44	-0.10051	0.090046	0.331566	None
5882	QKI	-0.31887	0.090099	0.331708	None
5883	RP11-120k	0.092741	0.090146	0.331821	None
5884	LOC10050(	0.143806	0.090212	0.332009	None
5885	LOC10192!	0.131074	0.09023	0.332019	None
5886	RABEPK	-0.12113	0.090302	0.332227	None
5887	HM13	-0.26504	0.090342	0.332296	None
5888	LOC10192!	-0.16771	0.090351	0.332296	None
5889	BCL2L2	-0.0946	0.09037	0.33231	None
5890	LBR	0.181941	0.09041	0.33233	None
5891	GALNT11	-0.23784	0.090413	0.33233	None
5892	LOC10050	0.159349	0.090432	0.33233	None
5893	AX747507	0.365567	0.090437	0.33233	None
5894	BC062753	-0.17831	0.090468	0.332331	None
5895	BFSP1	-0.22536	0.090471	0.332331	None
5896	NOL11	-0.16927	0.090489	0.332331	None
5897	PRELID2	-0.22465	0.090504	0.332331	None
5898	FLJ25917	-0.08106	0.09052	0.332331	None
5899	CTBP1-AS	0.054296	0.090536	0.332331	None
5900	KPNA5	0.394975	0.090553	0.332331	None
5901	PRICKLE1	-0.35576	0.090568	0.332331	None
5902	UBR7	0.194897	0.090583	0.332331	None
5903	TGM2	-0.21801	0.090591	0.332331	None
5904	C5	0.381767	0.090649	0.332482	None
5905	KIAA0895L	-0.20102	0.090663	0.332482	None
5906	RANBP17	-0.2418	0.090716	0.332619	None
5907	PLEKHH1	-0.17483	0.090768	0.332756	None
5908	SLC26A8	-0.21627	0.090854	0.332901	None
5909	P4HA3	0.0501	0.090857	0.332901	None
5910	HDAC4	-0.40421	0.090861	0.332901	None
5911	MMAB	-0.2383	0.09087	0.332901	None
5912	LOC33946	0.02441	0.09091	0.332985	None
5913	PTMA	0.124623	0.090936	0.332985	None
5914	SNX19	-0.09549	0.090939	0.332985	None

5915	GIN1	-0.30004	0.091028	0.333256	None
5916	WNT10A	0.017006	0.091074	0.33337	None
5917	DNAJC11	-0.24795	0.091139	0.333552	None
5918	XRCC2	0.294788	0.091156	0.333555	None
5919	NUDCD1	-0.26038	0.091329	0.33412	None
5920	RP11-422F	-0.20044	0.091341	0.33412	None
5921	PRPS1L1	0.048887	0.091474	0.334549	None
5922	ZSWIM7	-0.27486	0.091568	0.334839	None
5923	WSB2	-0.28326	0.091648	0.335074	None
5924	IL12A	-0.36873	0.091689	0.335167	None
5925	DNAJC3	0.367444	0.091729	0.33517	None
5926	OSBPL11	-0.37248	0.091729	0.33517	None
5927	RAB2A	0.156075	0.091736	0.33517	None
5928	ZCRB1	-0.21644	0.091785	0.335292	None
5929	ESYT2	0.280817	0.091832	0.335405	None
5930	LOC64621	0.228011	0.091954	0.335796	None
5931	ANO9	-0.15913	0.091979	0.335829	None
5932	TCEA3	-0.14399	0.092039	0.335938	None
5933	PIR	-0.60161	0.092053	0.335938	None
5934	PROM2	0.087652	0.092055	0.335938	None
5935	AATK	-0.17602	0.092167	0.336288	None
5936	AP1M1	-0.24567	0.092201	0.336356	None
5937	NINL	-0.40347	0.09222	0.336358	None
5938	RAB3A	-0.08652	0.092232	0.336358	None
5939	RPL7	0.058959	0.092278	0.336442	None
5940	YPEL1	0.207763	0.092287	0.336442	None
5941	RP11-930F	0.079316	0.092302	0.336442	None
5942	FABP1	0.014148	0.092321	0.336454	None
5943	CDK20	-0.16927	0.092444	0.336836	None
5944	LOC65227	0.045758	0.092457	0.336836	None
5945	DYRK3	0.138634	0.092481	0.336838	None
5946	TRRAP	-0.20172	0.092494	0.336838	None
5947	PRLHR	0.012502	0.092509	0.336838	None
5948	FXYD2	0.117243	0.09252	0.336838	None
5949	TBX4	0.043825	0.092542	0.336863	None
5950	C16orf13	-0.29444	0.092631	0.337097	None
5951	DEFB126	0.006294	0.092638	0.337097	None
5952	CEBPB	-0.46094	0.092672	0.337165	None
5953	RABGGTA	-0.26222	0.092747	0.337382	None
5954	RP11-473I	-0.29613	0.092791	0.337487	None
5955	ZNRF4	0.102138	0.09284	0.337608	None
5956	MBOAT7	-0.27401	0.092935	0.337806	None
5957	PRDX3	-0.12597	0.092936	0.337806	None
5958	MRC2	-0.24888	0.092958	0.337806	None
5959	ZNF490	-0.15276	0.092979	0.337806	None
5960	ISOC2	-0.30488	0.092988	0.337806	None
5961	LINC00293	0.002521	0.092988	0.337806	None
5962	LOC10028	0.024669	0.093032	0.337909	None
5963	SLC25A45	-0.1927	0.093084	0.337992	None
5964	BIN1	-0.41739	0.093086	0.337992	None
5965	LINC00550	0.066461	0.093178	0.338267	None
5966	CITED4	-0.12662	0.093319	0.338627	None
5967	KIFAP3	-0.32109	0.093321	0.338627	None
5968	PUF60	-0.22806	0.093338	0.338627	None
5969	THAP1	-0.1867	0.093339	0.338627	None
5970	PRRG1	-0.29812	0.093395	0.338771	None
5971	LOC10192	0.015988	0.093545	0.339207	None
5972	CAB39	-0.19958	0.093546	0.339207	None

5973	LINC00266	-0.18838	0.0936	0.339313	None
5974	C1orf159	-0.08507	0.093607	0.339313	None
5975	PPIL4	0.377279	0.09363	0.33934	None
5976	INCENP	0.094002	0.093667	0.339419	None
5977	SH2D4A	-0.14425	0.093708	0.339493	None
5978	NOL6	-0.06967	0.093737	0.339493	None
5979	PROCA1	-0.1252	0.093748	0.339493	None
5980	RP11-174C	0.206768	0.09375	0.339493	None
5981	ZCCHC6	-0.31749	0.09377	0.339505	None
5982	TTC28-AS1	-0.14772	0.093804	0.339547	None
5983	TBC1D31	-0.15986	0.093814	0.339547	None
5984	UNG	0.322905	0.09384	0.339547	None
5985	RNFT2	0.595048	0.093859	0.339547	None
5986	BRD7	0.221068	0.09386	0.339547	None
5987	UBR1	0.227179	0.093917	0.339698	None
5988	TIGD6	-0.06136	0.09398	0.33987	None
5989	AP3B1	0.19194	0.093996	0.339871	None
5990	ACSL1	0.461183	0.094026	0.339921	None
5991	KLF3-AS1	0.381075	0.094081	0.340064	None
5992	AMN1	-0.31575	0.094133	0.340196	None
5993	SNX7	-0.66753	0.094165	0.340254	None
5994	MRPS10	-0.41058	0.094215	0.340376	None
5995	NAGA	-0.29473	0.094401	0.340977	None
5996	SMAD2	-0.15563	0.094412	0.340977	None
5997	MRAS	-0.31955	0.094431	0.340988	None
5998	MICU2	0.254668	0.094454	0.341015	None
5999	DGCR11	0.136764	0.094479	0.341046	None
6000	FRMPD3	0.086773	0.094532	0.341158	None
6001	MGMT	-0.26533	0.094553	0.341158	None
6002	MMP24-AS	-0.14722	0.094557	0.341158	None
6003	SSR1	-0.15767	0.094575	0.341165	None
6004	LINC00685	-0.13981	0.0946	0.341192	None
6005	NLRP1	0.070965	0.094627	0.341192	None
6006	TLN2	-0.15507	0.094629	0.341192	None
6007	FAM184A	-0.29107	0.094746	0.341557	None
6008	LOC10013	-0.29053	0.094773	0.341598	None
6009	LOC10013	-0.35562	0.094868	0.341883	None
6010	CXCL10	-0.86161	0.094893	0.341914	None
6011	SH3BP1	-0.11483	0.095038	0.342378	None
6012	ARHGAP32	-0.31227	0.095053	0.342378	None
6013	NLK	-0.29419	0.095084	0.342431	None
6014	CD84	-0.27619	0.095105	0.342449	None
6015	CEP95	0.292402	0.095195	0.34272	None
6016	CD5L	-1.16887	0.095225	0.342767	None
6017	ABCA13	0.314686	0.095356	0.343183	None
6018	CAT	0.38216	0.095385	0.343198	None
6019	COA1	0.225365	0.095392	0.343198	None
6020	SIGMAR1	-0.34948	0.095458	0.343378	None
6021	CRYBB2P1	-0.09777	0.095526	0.343543	None
6022	RP11-61L1	0.275466	0.095535	0.343543	None
6023	THRA	-0.13165	0.095618	0.343784	None
6024	ZNF620	0.072736	0.095673	0.343925	None
6025	LOC101928	0.068248	0.095725	0.343934	None
6026	AP5M1	-0.31149	0.095735	0.343934	None
6027	SHF	-0.15563	0.095742	0.343934	None
6028	CCDC19	0.15052	0.095746	0.343934	None
6029	RP11-457I	0.069598	0.095755	0.343934	None
6030	AX746823	-0.58202	0.095791	0.343981	None

6031	PCSK5	0.336343	0.0958	0.343981	None
6032	PYCRL	-0.15091	0.095924	0.34437	None
6033	CCDC174	-0.27312	0.095955	0.344403	None
6034	FARSA	-0.28681	0.095971	0.344403	None
6035	DNM2	-0.20348	0.095981	0.344403	None
6036	FBXO38	0.221652	0.09603	0.344469	None
6037	CARS	-0.21104	0.096044	0.344469	None
6038	ABI2	0.181655	0.096047	0.344469	None
6039	N4BP2	0.307379	0.096115	0.344592	None
6040	ATG101	0.295106	0.096122	0.344592	None
6041	C4orf19	0.015569	0.096132	0.344592	None
6042	GALNT7	0.329735	0.096145	0.344592	None
6043	KCNE1L	0.556947	0.096226	0.344772	None
6044	BAIAP3	0.046586	0.096237	0.344772	None
6045	GALNT14	-0.49936	0.096243	0.344772	None
6046	TMEM242	-0.17322	0.096354	0.345075	None
6047	C16orf74	-0.31579	0.096368	0.345075	None
6048	CYSTM1	-0.41937	0.096376	0.345075	None
6049	CDH2	0.378869	0.096451	0.345286	None
6050	UBAC2	-0.20593	0.09656	0.345621	None
6051	LOC10192!	0.023017	0.096637	0.345755	None
6052	DRAP1	-0.31638	0.096649	0.345755	None
6053	SNRPA1	0.177	0.096653	0.345755	None
6054	CRNDE	0.508589	0.096661	0.345755	None
6055	LINC00226	0.027075	0.096687	0.345788	None
6056	MIR670HG	0.088074	0.096721	0.345803	None
6057	COX20	-0.22304	0.096727	0.345803	None
6058	HDAC5	-0.15529	0.09674	0.345803	None
6059	CTBP2	-0.19061	0.096755	0.345803	None
6060	MARK2	-0.07894	0.096809	0.345939	None
6061	CMTM5	-0.4087	0.096861	0.34602	None
6062	MRPL51	-0.17499	0.096873	0.34602	None
6063	ACOT7	-0.23877	0.096879	0.34602	None
6064	TLK2	-0.20185	0.09695	0.346179	None
6065	TSNARE1	-0.13749	0.096977	0.346179	None
6066	MXD1	0.497475	0.096982	0.346179	None
6067	NOC2L	-0.13014	0.096998	0.346179	None
6068	SETBP1	-0.61158	0.097006	0.346179	None
6069	HIST1H2AI	-0.56866	0.097027	0.346179	None
6070	LOC10192!	-0.31362	0.097036	0.346179	None
6071	CWC15	-0.28023	0.097053	0.346185	None
6072	PA2G4	-0.39832	0.097072	0.346194	None
6073	LAP3	-0.26486	0.097112	0.346282	None
6074	RIPK1	0.170338	0.097178	0.346371	None
6075	KIF19	0.014776	0.097179	0.346371	None
6076	KLRG2	-0.15728	0.097185	0.346371	None
6077	RNF187	0.006138	0.097216	0.346403	None
6078	ABCA17P	-0.36156	0.097226	0.346403	None
6079	BPTF	-0.18635	0.097294	0.346551	None
6080	ZNF599	-0.16644	0.0973	0.346551	None
6081	CCNI	-0.19727	0.097376	0.346738	None
6082	OCLN	-0.38381	0.097388	0.346738	None
6083	LINC00282	-0.34214	0.0974	0.346738	None
6084	JMJD4	-0.13059	0.097465	0.346841	None
6085	SEMA4F	-0.22944	0.097475	0.346841	None
6086	RP11-295(	-0.15919	0.097493	0.346841	None
6087	LYAR	-0.28703	0.097493	0.346841	None
6088	KANSL1L	0.384211	0.097537	0.346901	None

6089	CRTAC1	0.036818	0.097542	0.346901	None
6090	SIGLEC1	-0.62705	0.097568	0.346935	None
6091	RP11-873E	0.104962	0.097601	0.346996	None
6092	AK093205	0.018318	0.097706	0.347221	None
6093	LINC00675	0.062611	0.097709	0.347221	None
6094	DBF4B	-0.11622	0.097713	0.347221	None
6095	KIF22	0.31663	0.097859	0.347684	None
6096	RP11-403N	0.079724	0.097944	0.34793	None
6097	HBQ1	-0.54209	0.097985	0.347979	None
6098	KIAA0391	-0.09195	0.098017	0.347979	None
6099	BNC2	0.005141	0.098019	0.347979	None
6100	CDKN1A	0.648481	0.098022	0.347979	None
6101	COBLL1	-0.23997	0.098054	0.347981	None
6102	CBFA2T2	-0.10234	0.098073	0.347981	None
6103	WDFY2	0.29718	0.098089	0.347981	None
6104	TMEM254	-0.19474	0.09809	0.347981	None
6105	NOP14	-0.24386	0.098103	0.347981	None
6106	PPP2R3A	-0.26531	0.098157	0.348115	None
6107	ARNT	-0.20259	0.098182	0.348146	None
6108	MTCP1	0.205479	0.098267	0.34839	None
6109	SLC25A43	-0.40262	0.098395	0.348786	None
6110	TMEM72	-0.18716	0.098468	0.34899	None
6111	ZFYVE27	-0.23869	0.098555	0.349213	None
6112	AC087501.	0.034537	0.098563	0.349213	None
6113	CCL16	0.036524	0.098582	0.349222	None
6114	FLJ13773	-0.21659	0.098602	0.349235	None
6115	VAT1	-0.32644	0.098646	0.349333	None
6116	MRPS7	-0.28622	0.098696	0.349454	None
6117	FTHL17	0.034953	0.098736	0.34954	None
6118	DENND6B	-0.20561	0.098759	0.349563	None
6119	AP4S1	-0.17596	0.098817	0.349711	None
6120	PHF1	-0.18618	0.098847	0.349761	None
6121	SIPA1L2	-0.40014	0.098998	0.350229	None
6122	RALY-AS1	-0.14848	0.099012	0.350229	None
6123	SWSAP1	-0.24759	0.099145	0.350643	None
6124	IFIH1	-0.42152	0.099245	0.350864	None
6125	RP11-16P6	-0.20196	0.099256	0.350864	None
6126	LOC10192	0.080448	0.099263	0.350864	None
6127	NOD2	-0.31329	0.099273	0.350864	None
6128	HIST1H2BK	-0.46272	0.099332	0.351018	None
6129	C19orf60	-0.26698	0.099436	0.351327	None
6130	KIAA1109	0.046213	0.099521	0.351572	None
6131	VPS4B	0.228225	0.099539	0.351577	None
6132	GPR22	-0.10805	0.099609	0.351735	None
6133	C2orf42	-0.3019	0.09963	0.351735	None
6134	RPS9	0.235366	0.099635	0.351735	None
6135	TSPAN6	-0.5018	0.099657	0.351735	None
6136	GYG1	-0.17706	0.099665	0.351735	None
6137	ZNRF3	-0.36871	0.099691	0.351768	None
6138	HS6ST3	0.00764	0.099718	0.351807	None
6139	FAM200A	-0.19755	0.099749	0.351846	None
6140	RNPS1	-0.16034	0.099777	0.351846	None
6141	SMPD1	0.04935	0.099778	0.351846	None
6142	ILF3-AS1	-0.49499	0.099797	0.351855	None
6143	LOC10099	-0.12547	0.099832	0.351922	None
6144	TMEM9	-0.23142	0.099887	0.352059	None
6145	LINC01366	0.088356	0.09996	0.35223	None
6146	PPP2R5B	-0.11204	0.099968	0.35223	None

6147	SLC35B3	-0.15338	0.099991	0.35223	None
6148	CCNF	-0.20689	0.100005	0.35223	None
6149	TMEM95	0.085458	0.100017	0.35223	None
6150	P2RY8	-0.40125	0.10006	0.352326	None
6151	LAMP3	-0.39941	0.100189	0.352721	None
6152	RANGAP1	-0.24597	0.100234	0.352822	None
6153	CCDC114	-0.21729	0.100262	0.352864	None
6154	ARPC2	-0.14798	0.100279	0.352867	None
6155	MTPN	-0.47612	0.100344	0.353036	None
6156	SPHK2	-0.11618	0.10036	0.353036	None
6157	LINC00112	0.005806	0.100401	0.353125	None
6158	FAM114A2	-0.2641	0.10044	0.353191	None
6159	ZNF542P	-0.27671	0.100465	0.353191	None
6160	IFIT5	-0.3717	0.100472	0.353191	None
6161	CNN2	-0.21638	0.100485	0.353191	None
6162	GNA13	0.321195	0.10055	0.353361	None
6163	WDR63	0.004918	0.100604	0.353493	None
6164	PRR34-AS1	-0.21527	0.100688	0.353732	None
6165	CHMP7	-0.21473	0.100737	0.353845	None
6166	ECE2	0.045698	0.100776	0.353854	None
6167	SSUH2	0.005031	0.100798	0.353854	None
6168	TTLL11	-0.15205	0.100806	0.353854	None
6169	LRCH4	-0.22759	0.100816	0.353854	None
6170	SLC12A6	-0.38038	0.100835	0.353854	None
6171	S100A1	0.007001	0.100837	0.353854	None
6172	PURA	-0.33682	0.100885	0.353914	None
6173	MTHFD1L	0.047098	0.100887	0.353914	None
6174	ITGA4	-0.2832	0.10099	0.354216	None
6175	MRPS17	-0.34418	0.101016	0.354253	None
6176	LOC15774(	0.068106	0.101124	0.354571	None
6177	SYDE1	-0.04634	0.101174	0.354689	None
6178	IPO9-AS1	0.060492	0.101236	0.354851	None
6179	ABLIM3	-0.36178	0.101282	0.354946	None
6180	CHMP1A	-0.19524	0.101312	0.354946	None
6181	BRK1	-0.10638	0.101313	0.354946	None
6182	QRSL1	0.28438	0.101329	0.354946	None
6183	SCARF2	0.004312	0.10139	0.355095	None
6184	RHOA	-0.48505	0.101404	0.355095	None
6185	NINJ1	-0.61	0.101495	0.355356	None
6186	LRRFIP1	0.415846	0.101557	0.355516	None
6187	ABCB10	-0.32825	0.101603	0.35562	None
6188	CRIPT	0.163147	0.101637	0.355681	None
6189	DDX42	0.309663	0.101703	0.355761	None
6190	CTNNB1	0.283673	0.101713	0.355761	None
6191	LPIN1	0.257923	0.101718	0.355761	None
6192	LOC10192	0.038036	0.101733	0.355761	None
6193	LINC00858	0.020734	0.101761	0.355761	None
6194	RP11-796E	0.159818	0.101802	0.355761	None
6195	DET1	-0.20698	0.101821	0.355761	None
6196	ATP6V1G1	-0.17278	0.101825	0.355761	None
6197	TMEM176A	-0.58475	0.101847	0.355761	None
6198	DIXDC1	-0.12706	0.101847	0.355761	None
6199	LY96	-0.87679	0.101858	0.355761	None
6200	TRAT1	-0.47195	0.101859	0.355761	None
6201	OGDH	-0.16061	0.101874	0.355761	None
6202	NR5A1	0.079907	0.10189	0.355761	None
6203	APOA1BP	-0.25318	0.102003	0.356071	None
6204	LOC10050!	0.007527	0.102012	0.356071	None

6205	USP16	0.166925	0.102056	0.356167	None
6206	MAP2K4	-0.16448	0.10209	0.356228	None
6207	SLC7A5	0.481332	0.102109	0.356237	None
6208	UQCRB	-0.15776	0.102193	0.356474	None
6209	GNPDA2	-0.26786	0.102214	0.356491	None
6210	ENPP4	0.29373	0.102365	0.35696	None
6211	IL13RA1	-0.3522	0.102444	0.357176	None
6212	ZRANB2	-0.15216	0.102483	0.357181	None
6213	PAPD4	0.343831	0.102489	0.357181	None
6214	SERPINB12	0.046846	0.102495	0.357181	None
6215	ZNF702P	-0.12717	0.102534	0.357182	None
6216	GABPB1	0.225466	0.102536	0.357182	None
6217	PRG3	0.322077	0.102544	0.357182	None
6218	PAPOLA	0.19975	0.10258	0.35725	None
6219	MST4	0.325931	0.102633	0.357377	None
6220	GRAMD3	-0.23591	0.102694	0.357521	None
6221	PLA2G12A	-0.2992	0.102708	0.357521	None
6222	PPP2R2D	-0.18173	0.102738	0.35757	None
6223	CUL9	-0.10287	0.102768	0.357615	None
6224	PLK1	-0.40071	0.102823	0.357701	None
6225	CTHRC1	-0.77095	0.102826	0.357701	None
6226	KLF16	-0.13473	0.102863	0.357774	None
6227	TBL1XR1	0.230523	0.102888	0.357784	None
6228	ROGDI	-0.26412	0.102913	0.357784	None
6229	MYH10	-0.61169	0.102915	0.357784	None
6230	LOC10272	-0.28894	0.102962	0.357888	None
6231	CUEDC1	-0.25049	0.103042	0.358107	None
6232	TBCEL	-0.20114	0.103095	0.358234	None
6233	PALM	-0.11006	0.103134	0.358314	None
6234	REEP6	-0.13689	0.103152	0.358317	None
6235	LOC10192	-0.23233	0.103299	0.358771	None
6236	GALM	-0.43514	0.103343	0.358866	None
6237	COL6A5	-0.37444	0.103363	0.35888	None
6238	CTD-2076I	0.009717	0.103381	0.358882	None
6239	TAZ	-0.21092	0.103411	0.35893	None
6240	LOC44166	-0.12097	0.103478	0.359106	None
6241	LPCAT3	-0.31577	0.103548	0.359291	None
6242	PLK4	0.39361	0.103569	0.359305	None
6243	SPAG5-AS	0.094616	0.103601	0.35936	None
6244	RP11-426(	-0.11963	0.103637	0.359427	None
6245	CD99	0.394376	0.103678	0.359511	None
6246	TAS2R38	0.047621	0.103706	0.359544	None
6247	CLUH	-0.29314	0.103743	0.359544	None
6248	CEP72	-0.29273	0.103767	0.359544	None
6249	LOC64479	-0.0929	0.10378	0.359544	None
6250	RP11-220I	0.027161	0.103795	0.359544	None
6251	PGM2L1	-0.18792	0.1038	0.359544	None
6252	CYP24A1	0.010433	0.103804	0.359544	None
6253	UBA1	-0.15732	0.103847	0.359637	None
6254	THOC1	-0.20216	0.103891	0.35973	None
6255	ACTL6A	0.397426	0.10392	0.359776	None
6256	LOC10013	-0.07078	0.103972	0.359897	None
6257	TPBG	-0.42636	0.103993	0.359912	None
6258	MANEA	0.157345	0.104062	0.360094	None
6259	CBLN2	0.163614	0.104121	0.360238	None
6260	TRAF4	0.142486	0.104148	0.360275	None
6261	PRDM1	0.251209	0.104285	0.360694	None
6262	NAT8L	0.102291	0.104317	0.360707	None

6263	PCTP	-0.31061	0.104341	0.360707	None
6264	SYNGR3	-0.32735	0.104344	0.360707	None
6265	DIS3L2	0.227687	0.104356	0.360707	None
6266	SCCPDH	-0.16433	0.104386	0.360745	None
6267	IDH1-AS1	-0.12718	0.104413	0.360745	None
6268	OR8G2	0.029988	0.104417	0.360745	None
6269	SPON1	-0.63885	0.104483	0.360915	None
6270	PTPDC1	-0.17718	0.104562	0.36113	None
6271	CCL18	-1.13681	0.104771	0.361693	None
6272	ECSIT	-0.29388	0.10478	0.361693	None
6273	YJEFN3	-0.0976	0.104815	0.361693	None
6274	EME2	-0.14277	0.104821	0.361693	None
6275	SURF2	-0.14152	0.104821	0.361693	None
6276	LOC15005	0.095581	0.104825	0.361693	None
6277	LOC10050	-0.2441	0.104852	0.361712	None
6278	YY1	-0.15915	0.104864	0.361712	None
6279	TNFRSF12A	0.205289	0.104944	0.36193	None
6280	NOL4L	-0.188	0.104989	0.361974	None
6281	TEX40	0.072359	0.105002	0.361974	None
6282	ZNF8	0.286799	0.105011	0.361974	None
6283	KLK2	0.016732	0.105023	0.361974	None
6284	COG5	-0.25117	0.105048	0.362001	None
6285	ARL1	-0.18057	0.105142	0.362268	None
6286	BMS1P20	0.226854	0.105172	0.362294	None
6287	LINC01234	-0.02993	0.105198	0.362294	None
6288	NFIB	-0.34981	0.1052	0.362294	None
6289	TFAP2A	0.005273	0.105229	0.362336	None
6290	CDK5RAP3	-0.23053	0.105248	0.362343	None
6291	SCN1B	-0.47657	0.105267	0.362353	None
6292	RPS6KL1	-0.18258	0.105292	0.362379	None
6293	AGAP1	-0.14894	0.105356	0.362507	None
6294	CTA-250D	0.16253	0.105391	0.362507	None
6295	PICALM	-0.27503	0.1054	0.362507	None
6296	TMEM147	-0.20273	0.105401	0.362507	None
6297	EFNA4	-0.14656	0.10542	0.362507	None
6298	RASGEF1B	0.397762	0.105429	0.362507	None
6299	ADAP2	-0.81567	0.105449	0.362517	None
6300	CERS5	-0.11016	0.105611	0.362949	None
6301	CDH5	-0.40405	0.105618	0.362949	None
6302	DGUOK	-0.25218	0.105625	0.362949	None
6303	LOC55310	0.06055	0.105655	0.362996	None
6304	INPP1	-0.3079	0.105694	0.363064	None
6305	SNAP23	-0.29927	0.105708	0.363064	None
6306	CD86	-0.6164	0.105731	0.363084	None
6307	RPS7	-0.25311	0.105819	0.363258	None
6308	ANKRD36	0.211813	0.105822	0.363258	None
6309	ZBTB2	0.262262	0.105832	0.363258	None
6310	LOC10012	0.080305	0.105914	0.363481	None
6311	RP11-66N	-0.11338	0.105951	0.363541	None
6312	STX18	0.223973	0.105965	0.363541	None
6313	CXXC1	-0.18881	0.106044	0.363711	None
6314	TRIM59	0.32359	0.106048	0.363711	None
6315	BBIP1	-0.1687	0.106068	0.363715	None
6316	EPHA4	0.002391	0.106098	0.363715	None
6317	OGFOD3	-0.20889	0.1061	0.363715	None
6318	ODF3L1	0.017806	0.106118	0.36372	None
6319	TUBG1	-0.35642	0.106135	0.363723	None
6320	CKB	-0.36084	0.106189	0.363848	None

6321	PCED1B	-0.32925	0.106214	0.363875	None
6322	JRK	-0.14037	0.106323	0.364192	None
6323	LINC00824	0.055713	0.106345	0.364209	None
6324	CTC-523E2	-0.30894	0.106405	0.364358	None
6325	PQLC1	-0.11251	0.106434	0.364399	None
6326	PPM1G	0.292264	0.106564	0.364709	None
6327	RP11-560C	0.043791	0.106567	0.364709	None
6328	MAP1A	0.137798	0.106575	0.364709	None
6329	PLA2G4C	-0.18313	0.106607	0.364724	None
6330	NRARP	0.343225	0.106613	0.364724	None
6331	RP11-112J	-0.41869	0.106649	0.364762	None
6332	INTS7	-0.28772	0.106658	0.364762	None
6333	DGKG	-0.16736	0.106703	0.364817	None
6334	FNBP1L	0.346793	0.106707	0.364817	None
6335	RTKN	-0.1435	0.106731	0.36484	None
6336	VPS8	-0.38381	0.106781	0.364954	None
6337	GGACT	-0.09356	0.106807	0.364984	None
6338	AHSA2	0.364483	0.106844	0.365055	None
6339	CABYR	0.038625	0.106904	0.3652	None
6340	SDHAF1	-0.19276	0.106924	0.365213	None
6341	DIP2A	-0.14817	0.106954	0.365257	None
6342	TSPAN4	-0.37111	0.10698	0.365274	None
6343	MCM3AP-	-0.22764	0.106994	0.365274	None
6344	PDIK1L	0.223904	0.10701	0.365274	None
6345	ZNF711	0.431696	0.107159	0.365727	None
6346	BAHD1	-0.22279	0.107186	0.365759	None
6347	MRPL52	-0.17114	0.107575	0.367031	None
6348	MTFMT	-0.35279	0.107629	0.367156	None
6349	ESD	-0.16988	0.10775	0.367493	None
6350	KIAA0040	0.198901	0.107762	0.367493	None
6351	THAP5	0.19353	0.107807	0.367591	None
6352	SH3PXD2B	-0.03623	0.107857	0.367661	None
6353	LOC10012	0.40193	0.107862	0.367661	None
6354	TFRC	-0.33572	0.107883	0.367677	None
6355	ERVFRD-1	-0.37451	0.107945	0.367777	None
6356	TIE1	-0.16907	0.107947	0.367777	None
6357	PARP14	-0.14648	0.107998	0.367894	None
6358	ZNF226	-0.14421	0.108023	0.367922	None
6359	PYCARD	-0.26965	0.108202	0.368473	None
6360	PHLDA2	0.060662	0.108239	0.368541	None
6361	M6PR	-0.29182	0.108292	0.368633	None
6362	TSPAN31	-0.23565	0.1083	0.368633	None
6363	MORN4	-0.11289	0.108346	0.368692	None
6364	KRBA1	-0.1628	0.108352	0.368692	None
6365	RBM17	-0.1591	0.108407	0.368821	None
6366	STARD7	-0.18318	0.108511	0.369109	None
6367	UNC93B1	-0.18982	0.108536	0.369109	None
6368	FKBPL	-0.30911	0.108551	0.369109	None
6369	GBP2	-0.55654	0.108559	0.369109	None
6370	DNAJC3-A	-0.22188	0.108625	0.369257	None
6371	CCDC81	0.306352	0.108646	0.369257	None
6372	MYOF	-0.32156	0.108654	0.369257	None
6373	HELQ	-0.19971	0.108674	0.369268	None
6374	MAPKAPK2	0.319263	0.108704	0.369312	None
6375	CDC26	-0.18321	0.108778	0.369504	None
6376	KMT2D	-0.16476	0.108831	0.369566	None
6377	MEAF6	-0.26266	0.108843	0.369566	None
6378	SPAG6	-0.59516	0.108866	0.369566	None

6379	KLHL5	0.253145	0.108867	0.369566	None
6380	COQ3	-0.27354	0.108889	0.369566	None
6381	GPM6B	0.438714	0.108899	0.369566	None
6382	YIF1B	-0.19945	0.109029	0.369917	None
6383	PPARG	-0.70688	0.109036	0.369917	None
6384	RP11-722E	-0.29725	0.109187	0.370316	None
6385	AAGAB	-0.25894	0.109188	0.370316	None
6386	COPZ2	-0.19504	0.109253	0.370399	None
6387	CTDSPL	-0.30948	0.109255	0.370399	None
6388	TBC1D8	-0.15543	0.109264	0.370399	None
6389	ASUN	-0.17326	0.109309	0.370494	None
6390	ASAP3	0.041758	0.109389	0.370706	None
6391	LRRC8B	-0.34304	0.109565	0.371236	None
6392	PLA2G15	-0.46089	0.109579	0.371236	None
6393	MRPL1	-0.25187	0.109696	0.371573	None
6394	HYAL3	-0.33397	0.109714	0.371577	None
6395	MSRB2	-0.25546	0.109746	0.371584	None
6396	EXOSC8	-0.17968	0.109755	0.371584	None
6397	BST1	0.29069	0.109769	0.371584	None
6398	GIMAP4	-0.8406	0.109785	0.371584	None
6399	PCK2	-0.27072	0.109852	0.371746	None
6400	CABP4	0.210439	0.109883	0.371746	None
6401	KRTAP17-1	0.033053	0.109884	0.371746	None
6402	GOLGA2	-0.15203	0.109954	0.371789	None
6403	PSMA7	-0.2294	0.109972	0.371789	None
6404	ZNF320	0.229393	0.109973	0.371789	None
6405	TUBAL3	-0.3991	0.109975	0.371789	None
6406	ARIH2OS	-0.20242	0.109992	0.371789	None
6407	PTPRA	-0.28848	0.110024	0.371789	None
6408	CTD-2528I	-0.36235	0.110026	0.371789	None
6409	SRP54	0.22436	0.110034	0.371789	None
6410	CENPK	0.477184	0.110124	0.372012	None
6411	RNA45S5	-0.44807	0.110135	0.372012	None
6412	HIP1	-0.25513	0.110178	0.372043	None
6413	TLN1	-0.2471	0.110178	0.372043	None
6414	WDFY3	-0.30132	0.110274	0.37226	None
6415	LOC10272	0.218784	0.110277	0.37226	None
6416	MSTO1	-0.18282	0.110304	0.372294	None
6417	IFT172	-0.2372	0.11044	0.372676	None
6418	STAP1	0.541869	0.110452	0.372676	None
6419	SLC25A11	-0.27993	0.110501	0.372783	None
6420	MOCOS	-0.0786	0.110528	0.372815	None
6421	CLDN5	-0.5123	0.110553	0.372843	None
6422	ROCK1	0.12859	0.110621	0.372964	None
6423	DNAJC28	-0.13971	0.110623	0.372964	None
6424	LPCAT1	-0.28071	0.110688	0.373087	None
6425	SIT1	-0.08329	0.110694	0.373087	None
6426	ZNF655	-0.32957	0.110885	0.373614	None
6427	MATK	-0.359	0.110897	0.373614	None
6428	SESN3	-0.24554	0.110902	0.373614	None
6429	NBEA	0.448404	0.110937	0.37367	None
6430	TOLLIP	-0.11051	0.110953	0.37367	None
6431	EIF5B	-0.22117	0.110995	0.37373	None
6432	RP11-79P5	0.069491	0.111006	0.37373	None
6433	TIPARP-AS	0.113114	0.111081	0.373924	None
6434	CASC10	-0.21817	0.111149	0.374095	None
6435	INVS	0.192978	0.111229	0.374286	None
6436	PKLR	-0.20345	0.111256	0.374286	None

6437	AP2S1	-0.29576	0.11127	0.374286	None
6438	TAF1B	-0.22473	0.111275	0.374286	None
6439	DHX57	-0.14643	0.111351	0.374438	None
6440	CES3	-0.06719	0.111359	0.374438	None
6441	LOC100128	0.052447	0.111372	0.374438	None
6442	LETM2	0.123737	0.111421	0.374544	None
6443	RBPMS2	-0.77358	0.111468	0.374646	None
6444	COL7A1	0.07125	0.111523	0.37477	None
6445	LIN7A	-0.26112	0.111563	0.374781	None
6446	ZBTB16	-0.48111	0.111574	0.374781	None
6447	RNF144A	-0.10837	0.111595	0.374781	None
6448	NELL2	0.307305	0.111596	0.374781	None
6449	ABTB2	-0.15568	0.111623	0.374781	None
6450	MLX	-0.1766	0.111631	0.374781	None
6451	INPP4B	-0.37608	0.111647	0.374781	None
6452	LINC01133	-0.1997	0.111717	0.374929	None
6453	RP11-524C	0.11822	0.111725	0.374929	None
6454	TMEM185E	-0.18886	0.111754	0.374948	None
6455	UBTD2	0.246007	0.111791	0.374948	None
6456	YIPF1	-0.17299	0.111797	0.374948	None
6457	C11orf54	-0.26331	0.111801	0.374948	None
6458	CRHR1	0.010063	0.111828	0.374983	None
6459	RP11-483I	-0.1933	0.111862	0.374986	None
6460	LOC10272	0.085295	0.111864	0.374986	None
6461	SLC36A1	-0.16283	0.111898	0.375043	None
6462	TKT	-0.35083	0.111934	0.375103	None
6463	LOC10192	0.034794	0.111951	0.375103	None
6464	LOC64465	-0.34718	0.112048	0.375368	None
6465	PIWIL4	0.433972	0.11207	0.375368	None
6466	ZFR2	0.045677	0.112097	0.375368	None
6467	ZCCHC9	-0.21064	0.112105	0.375368	None
6468	UFM1	0.276972	0.112116	0.375368	None
6469	VPS54	0.188871	0.112187	0.375545	None
6470	NUP35	-0.18237	0.112258	0.375726	None
6471	BPGM	0.453498	0.112282	0.375749	None
6472	OR7E2P	0.006147	0.112363	0.375963	None
6473	PPARGC1A	-0.2471	0.112413	0.376071	None
6474	SCML4	-0.13943	0.112514	0.376294	None
6475	HHLA3	-0.15861	0.112515	0.376294	None
6476	SLC5A3	-0.16968	0.112554	0.376364	None
6477	JUND	-0.38783	0.11257	0.376364	None
6478	PTTG1	0.36576	0.112632	0.376514	None
6479	RCOR1	-0.1017	0.112685	0.376563	None
6480	C19orf33	-0.37183	0.112687	0.376563	None
6481	AGAP2	-0.0949	0.112699	0.376563	None
6482	DCXR	-0.29168	0.112758	0.376594	None
6483	C7orf34	0.032157	0.112765	0.376594	None
6484	LINC01007	0.025147	0.112777	0.376594	None
6485	BTC	-0.14266	0.112778	0.376594	None
6486	BIN3	0.146404	0.112796	0.376596	None
6487	TSEN2	-0.16176	0.112866	0.376738	None
6488	HCFC2	-0.17954	0.112874	0.376738	None
6489	HUWE1	0.214124	0.1129	0.376745	None
6490	SEL1L	-0.16527	0.11291	0.376745	None
6491	ZG16	0.0462	0.112931	0.376755	None
6492	PLEKHM1	-0.11615	0.113052	0.377103	None
6493	NCOA5	-0.17623	0.113173	0.377325	None
6494	CCNT2	0.18006	0.11318	0.377325	None

6495	TMEM59L	0.061227	0.113181	0.377325	None
6496	MRPS2	-0.30063	0.113189	0.377325	None
6497	SYNGR2	-0.14774	0.113237	0.377388	None
6498	ACTR5	-0.23077	0.113256	0.377388	None
6499	GALNTL6	0.011611	0.11326	0.377388	None
6500	LOC64532	0.028786	0.113303	0.377422	None
6501	RASSF4	-0.17502	0.113323	0.377422	None
6502	TACC1	-0.32341	0.113325	0.377422	None
6503	REG3A	0.028271	0.11334	0.377422	None
6504	CFI	-0.48329	0.113427	0.377597	None
6505	FAM109B	-0.24465	0.113438	0.377597	None
6506	PTPN14	-0.30804	0.113445	0.377597	None
6507	CTDSP2	-0.1701	0.113519	0.377725	None
6508	IQCH-AS1	-0.24108	0.113529	0.377725	None
6509	TCIRG1	-0.30081	0.113536	0.377725	None
6510	RNF150	-0.3047	0.113565	0.377766	None
6511	AGER	-0.03828	0.113648	0.377983	None
6512	HSD17B14	-0.32292	0.113712	0.378077	None
6513	DCAF4	-0.1859	0.113726	0.378077	None
6514	KLRB1	0.336872	0.113774	0.378077	None
6515	NME8	-0.43192	0.113777	0.378077	None
6516	SEPP1	-0.53621	0.113779	0.378077	None
6517	SMARCA5	0.170082	0.113781	0.378077	None
6518	GPC5	-0.35721	0.113833	0.378192	None
6519	MPP6	-0.28396	0.113879	0.378285	None
6520	FADS2	-0.0977	0.113921	0.378359	None
6521	APOL6	-0.2372	0.113936	0.378359	None
6522	FAM189A1	0.047647	0.114048	0.378672	None
6523	ZNF770	0.151597	0.114167	0.379011	None
6524	EIF1B	-0.09991	0.114216	0.379114	None
6525	TM9SF3	0.187563	0.114343	0.379377	None
6526	CHMP1B	0.313166	0.114344	0.379377	None
6527	NAPEPLD	-0.26075	0.114348	0.379377	None
6528	PRPF40B	-0.06114	0.114402	0.3795	None
6529	NR1I2	-0.18972	0.114524	0.379845	None
6530	NDUFAF4	-0.2198	0.114551	0.379849	None
6531	SLITRK2	0.052105	0.11457	0.379849	None
6532	DZIP3	0.315617	0.114577	0.379849	None
6533	SERINC5	0.350404	0.114616	0.379879	None
6534	TRPC6	-0.27169	0.114622	0.379879	None
6535	AASDHPPT	-0.18833	0.114676	0.379967	None
6536	CHMP4C	-0.53787	0.114694	0.379967	None
6537	ZFAS1	-0.38348	0.114701	0.379967	None
6538	PAQR5	-0.17721	0.114728	0.38	None
6539	RP11-140I	-0.3955	0.114787	0.380106	None
6540	EPB41L4A-	0.303479	0.114809	0.380106	None
6541	RAB9A	0.220677	0.114813	0.380106	None
6542	VEGFC	-0.3321	0.114871	0.380239	None
6543	LOC10050	-0.27654	0.114925	0.380361	None
6544	PRO2964	-0.34452	0.114968	0.380393	None
6545	UBE2F	-0.25457	0.11497	0.380393	None
6546	FAIM	0.272134	0.115011	0.380471	None
6547	GSS	-0.22664	0.115137	0.380829	None
6548	RP11-248J	0.295137	0.115229	0.380994	None
6549	MIA2	0.014851	0.115234	0.380994	None
6550	LOC10272	-0.08018	0.11524	0.380994	None
6551	LRRC8A	-0.18755	0.115323	0.381213	None
6552	LOC10272	-0.27654	0.115401	0.381378	None

6553	TMEM178A	-0.28821	0.115408	0.381378	None
6554	FAAH2	-0.15956	0.115436	0.381411	None
6555	SPINT1	-0.11231	0.115483	0.381509	None
6556	APLP1	0.044737	0.115525	0.381586	None
6557	MAP6D1	-0.1646	0.115552	0.381586	None
6558	CDKN2D	0.159533	0.115559	0.381586	None
6559	FOXO6	0.407101	0.115598	0.381615	None
6560	CLCN2	-0.08932	0.115604	0.381615	None
6561	ATF7	-0.13648	0.115632	0.381652	None
6562	TNFRSF11A	-0.25194	0.11566	0.381685	None
6563	GLUD2	-0.14248	0.115716	0.381811	None
6564	KIT	-0.39056	0.115743	0.381844	None
6565	USP38	0.19959	0.115773	0.381884	None
6566	SIRPB2	-0.39691	0.115828	0.382006	None
6567	IL1RL1	0.111668	0.115883	0.38208	None
6568	ZNF417	0.075783	0.115886	0.38208	None
6569	PPP2R1B	0.203141	0.115921	0.382139	None
6570	TRIM62	0.126254	0.115954	0.38219	None
6571	NUDCD2	-0.19862	0.116034	0.382394	None
6572	LCLAT1	-0.19889	0.116074	0.382468	None
6573	DCX	0.010681	0.116122	0.382569	None
6574	KBTBD3	-0.18377	0.116158	0.38263	None
6575	TAOK3	-0.13333	0.116179	0.382639	None
6576	ALB	0.002563	0.116211	0.382688	None
6577	HTR1E	0.033872	0.116257	0.382779	None
6578	PICK1	-0.13461	0.116593	0.383827	None
6579	ENSA	-0.34783	0.116699	0.384109	None
6580	ZMYND12	-0.25771	0.116725	0.384109	None
6581	MRPL21	-0.32706	0.116731	0.384109	None
6582	CACNG5	0.045797	0.116849	0.384438	None
6583	COL26A1	0.102535	0.116909	0.384577	None
6584	SZRD1	-0.18087	0.116987	0.384775	None
6585	AQP4-AS1	0.005325	0.117073	0.384914	None
6586	TLCD1	-0.12862	0.11709	0.384914	None
6587	PCNXL3	-0.27282	0.117097	0.384914	None
6588	ANO10	-0.19635	0.117101	0.384914	None
6589	PRR12	-0.09062	0.117128	0.384929	None
6590	LOC10192	0.014706	0.117158	0.384929	None
6591	ZNF630	-0.14854	0.117158	0.384929	None
6592	PRKACA	-0.05213	0.117219	0.385022	None
6593	KCTD10	0.253806	0.117234	0.385022	None
6594	IRX1	0.602605	0.11724	0.385022	None
6595	ZCCHC17	-0.17308	0.117299	0.385155	None
6596	LINC01314	0.019525	0.117418	0.385448	None
6597	VILL	-0.28121	0.117432	0.385448	None
6598	HERC4	-0.20799	0.117457	0.385448	None
6599	C16orf87	0.25878	0.117459	0.385448	None
6600	SIX5	-0.07262	0.117487	0.385462	None
6601	COG8	-0.16236	0.117499	0.385462	None
6602	KBTBD6	-0.31986	0.117539	0.385481	None
6603	CREB3L4	-0.29939	0.11754	0.385481	None
6604	CD46	-0.19138	0.117584	0.385565	None
6605	EDRF1	0.2119	0.117604	0.385575	None
6606	RPS16	0.096542	0.117726	0.385914	None
6607	C5orf34	-0.29943	0.117779	0.386029	None
6608	MVB12B	-0.10292	0.117845	0.386187	None
6609	LOC10050	0.035448	0.117893	0.386286	None
6610	SYCE1L	0.098234	0.118017	0.386634	None

6611	UBR4	-0.09336	0.118052	0.386691	None
6612	DRAM1	-0.32863	0.118118	0.386839	None
6613	PPIEL	-0.09045	0.118149	0.386839	None
6614	SLC51B	0.036018	0.118151	0.386839	None
6615	WDR53	0.202949	0.118181	0.386879	None
6616	NDEL1	-0.0977	0.118232	0.386967	None
6617	TEX13B	0.034717	0.118244	0.386967	None
6618	THBD	0.522068	0.118261	0.386967	None
6619	HIVEP3	-0.24264	0.118316	0.387088	None
6620	CBFA2T3	-0.32382	0.118389	0.387269	None
6621	GSTK1	-0.24323	0.118514	0.38762	None
6622	SCAMP5	-0.10435	0.118573	0.387718	None
6623	TDRKH	-0.27767	0.11858	0.387718	None
6624	CCDC34	-0.1433	0.118628	0.387815	None
6625	TTC18	-0.18817	0.118799	0.388315	None
6626	RASSF2	0.278054	0.118842	0.388342	None
6627	NSRP1	0.17808	0.118843	0.388342	None
6628	SDCCAG8	-0.19849	0.118878	0.388349	None
6629	KIAA0087	-0.52557	0.118881	0.388349	None
6630	GOLGA3	-0.17492	0.118952	0.388523	None
6631	LOC10192!	0.003973	0.119025	0.388664	None
6632	NAP1L5	0.355568	0.119037	0.388664	None
6633	AMFR	-0.45637	0.119055	0.388664	None
6634	SLC35F6	-0.07459	0.119067	0.388664	None
6635	LOC10192	-0.09564	0.1191	0.388713	None
6636	TRMT10A	0.317758	0.119128	0.388747	None
6637	FAM175B	-0.16906	0.11915	0.388759	None
6638	EMC6	0.12821	0.119221	0.38889	None
6639	RIN3	-0.30956	0.119226	0.38889	None
6640	HBM	-1.02786	0.119274	0.388988	None
6641	PRMT3	-0.22604	0.119319	0.389075	None
6642	LINC00493	-0.18093	0.119381	0.389127	None
6643	UVRAG	-0.24057	0.119386	0.389127	None
6644	PKD1P1	-0.32542	0.119389	0.389127	None
6645	HLA-DMB	0.299392	0.119453	0.389278	None
6646	FYN	0.367357	0.11957	0.3896	None
6647	C20orf181	0.043286	0.119633	0.389747	None
6648	URB1	-0.19954	0.119686	0.389841	None
6649	PPP1R36	-0.10731	0.119698	0.389841	None
6650	PGM2	-0.16732	0.119735	0.389873	None
6651	CLASRP	-0.25399	0.119774	0.389873	None
6652	SPRR4	0.090288	0.119776	0.389873	None
6653	MECP2	-0.24904	0.11978	0.389873	None
6654	EEF2	-0.18361	0.119804	0.389895	None
6655	BRCA1	-0.26481	0.119876	0.390068	None
6656	ZBTB12	0.004884	0.119951	0.390256	None
6657	RPL36	-0.37294	0.120078	0.390609	None
6658	SEC16A	-0.15205	0.120178	0.390876	None
6659	RAVER1	-0.2954	0.120255	0.391067	None
6660	MYLIP	-0.31554	0.120273	0.391067	None
6661	KDELC2	0.261222	0.120325	0.391177	None
6662	IRF2BP2	-0.2134	0.12037	0.391264	None
6663	PTCHD1	0.003075	0.120403	0.391266	None
6664	ZSCAN31	-0.33529	0.120408	0.391266	None
6665	AACS	-0.22321	0.120424	0.391266	None
6666	DPH7	-0.14756	0.120486	0.391321	None
6667	VOPP1	-0.25898	0.120487	0.391321	None
6668	HDAC2	-0.12166	0.120495	0.391321	None

6669	TGFBR2	-0.16336	0.120514	0.391322	None
6670	PRDM11	-0.02343	0.120537	0.391339	None
6671	SOSTDC1	-0.30791	0.120683	0.391756	None
6672	PCDHB2	-0.49057	0.120788	0.392035	None
6673	PTTG3P	0.222329	0.120881	0.392241	None
6674	HSH2D	-0.37117	0.120887	0.392241	None
6675	LOC10050	-0.13017	0.120911	0.392258	None
6676	CCDC71L	-0.14541	0.120986	0.39242	None
6677	RIPPLY2	0.004939	0.120997	0.39242	None
6678	PAK1	-0.23615	0.121096	0.392652	None
6679	TSSC4	-0.20955	0.121133	0.392652	None
6680	MTIF2	-0.15907	0.121148	0.392652	None
6681	FOXP1	0.209469	0.121154	0.392652	None
6682	TRIM51	0.037045	0.121159	0.392652	None
6683	LOC72929	0.035372	0.121325	0.39313	None
6684	POM121C	-0.35665	0.121627	0.394049	None
6685	BBS10	-0.31773	0.121676	0.39414	None
6686	PDE4DIP	-0.2799	0.121691	0.39414	None
6687	LOC10193	-0.11109	0.12176	0.394214	None
6688	MFGE8	-0.06914	0.121771	0.394214	None
6689	AGL	-0.27715	0.121776	0.394214	None
6690	ERF	-0.09608	0.121811	0.394214	None
6691	RALGDS	-0.32349	0.121819	0.394214	None
6692	ATG16L1	0.240075	0.121823	0.394214	None
6693	MRPL55	-0.15545	0.121895	0.394354	None
6694	MSMO1	-0.32296	0.121903	0.394354	None
6695	ATRAID	-0.12335	0.121924	0.394361	None
6696	HNRNPDL	0.224903	0.121941	0.394361	None
6697	DMXL1	0.277696	0.122039	0.394617	None
6698	LOC10272	0.003065	0.122073	0.394669	None
6699	LPIN2	-0.22175	0.122135	0.394798	None
6700	CELSR3	-0.29045	0.122164	0.394798	None
6701	GUF1	-0.2441	0.122168	0.394798	None
6702	RP11-690I	0.279213	0.122241	0.394977	None
6703	GPATCH3	-0.26838	0.122357	0.395292	None
6704	CAPN10	-0.06027	0.122379	0.395305	None
6705	MPPED2	0.510185	0.122449	0.395472	None
6706	LCT	-0.37812	0.12249	0.395543	None
6707	SYNJ2	-0.14464	0.122545	0.395597	None
6708	SQRDL	-0.28577	0.122565	0.395597	None
6709	FOXN2	-0.11345	0.122579	0.395597	None
6710	ELK2AP	-0.13641	0.122583	0.395597	None
6711	CXCR6	0.027672	0.122598	0.395597	None
6712	C18orf54	-0.3049	0.122825	0.396271	None
6713	GNB1	-0.16119	0.122868	0.396316	None
6714	METTL23	-0.13576	0.122875	0.396316	None
6715	ZNF627	0.129279	0.122954	0.396429	None
6716	NME9	0.011703	0.122961	0.396429	None
6717	TFPI	0.460164	0.122965	0.396429	None
6718	PWAR5	-0.11808	0.123053	0.396654	None
6719	STK39	0.224456	0.123138	0.396869	None
6720	CCDC58	-0.43724	0.12317	0.396912	None
6721	SLC38A1	0.509404	0.123254	0.397124	None
6722	RPL6	0.056044	0.123303	0.397223	None
6723	GLB1L2	-0.19788	0.123394	0.3974	None
6724	LOC40094	0.002267	0.123395	0.3974	None
6725	C10orf128	-0.32072	0.123497	0.397669	None
6726	C5orf28	0.317899	0.123732	0.398368	None

6727	ANKDD1A	0.209896	0.123782	0.398432	None
6728	CNNM1	-0.09613	0.123789	0.398432	None
6729	AC018755.	-0.11431	0.12382	0.398474	None
6730	LINC01094	-0.31428	0.123915	0.398719	None
6731	LOC10192!	0.058158	0.123968	0.398805	None
6732	TRIM23	-0.14758	0.123979	0.398805	None
6733	ATG9B	0.048557	0.124098	0.399131	None
6734	KIF16B	-0.22796	0.124129	0.399151	None
6735	SYTL4	-0.15427	0.124155	0.399151	None
6736	TOMM20	-0.18313	0.124171	0.399151	None
6737	IRG1	0.434032	0.124199	0.399151	None
6738	LOC44093	0.26093	0.124211	0.399151	None
6739	LOC10192	0.012896	0.124215	0.399151	None
6740	PPARD	0.129573	0.124248	0.399196	None
6741	TIAM2	-0.23163	0.124325	0.399362	None
6742	KIAA0020	0.125927	0.124336	0.399362	None
6743	THAP7-AS	-0.04479	0.124379	0.399381	None
6744	CENPH	0.276892	0.124395	0.399381	None
6745	OFD1	0.212016	0.124397	0.399381	None
6746	CCNJ	-0.24045	0.124431	0.399429	None
6747	RP11-123K	-0.05997	0.124465	0.39948	None
6748	TMEM40	-0.32102	0.124502	0.39954	None
6749	FJX1	0.228364	0.12457	0.3997	None
6750	LONRF2	-0.38454	0.124646	0.399884	None
6751	TPTE2P6	-0.56298	0.124778	0.400246	None
6752	REEP1	-0.42443	0.124805	0.400275	None
6753	RP5-930J4	-0.36434	0.124869	0.40042	None
6754	ANAPC16	-0.12759	0.124894	0.400442	None
6755	OPLAH	-0.21041	0.124941	0.400532	None
6756	GNAI1	0.504891	0.125004	0.400676	None
6757	ATPAF1	-0.16706	0.125046	0.400752	None
6758	C9orf41	-0.15755	0.125191	0.400989	None
6759	MCF2L-AS	-0.0756	0.125194	0.400989	None
6760	KIAA1210	0.007751	0.125201	0.400989	None
6761	COPS6	0.258993	0.125216	0.400989	None
6762	ATP6V1D	-0.24689	0.125218	0.400989	None
6763	KIAA2026	-0.20396	0.125232	0.400989	None
6764	TRIM66	-0.32785	0.125334	0.401211	None
6765	LOC38878	0.091982	0.125362	0.401211	None
6766	NEU1	-0.44125	0.125385	0.401211	None
6767	PLAG1	-0.63778	0.125393	0.401211	None
6768	MAGT1	0.274121	0.125394	0.401211	None
6769	LOC10028	-0.14596	0.12554	0.401564	None
6770	NFKBIB	-0.13985	0.125541	0.401564	None
6771	CBX3P2	0.143062	0.12572	0.402064	None
6772	SLU7	0.214739	0.125734	0.402064	None
6773	LRRN3	0.03973	0.125861	0.402409	None
6774	GZMM	0.043434	0.126021	0.402863	None
6775	LOC10099	-0.13259	0.126095	0.403038	None
6776	ZNF667-A	-0.36974	0.126118	0.403051	None
6777	TCP10	0.020101	0.126192	0.40323	None
6778	LOC10050	0.026497	0.126278	0.403339	None
6779	AR	-0.40454	0.126305	0.403339	None
6780	LRRC41	-0.19892	0.126307	0.403339	None
6781	GCHFR	-0.25582	0.126308	0.403339	None
6782	HTR7P1	-0.14372	0.126323	0.403339	None
6783	LOC10192!	0.093307	0.126338	0.403339	None
6784	MIEF2	-0.14128	0.126358	0.403344	None

6785	PARD6A	0.224061	0.126493	0.403715	None
6786	PPP1R2	0.200238	0.126559	0.403866	None
6787	ZNF117	0.259683	0.126597	0.403893	None
6788	RAB11B	-0.02786	0.126606	0.403893	None
6789	OSBP2	-0.22228	0.126638	0.403893	None
6790	CMC2	-0.16319	0.126642	0.403893	None
6791	CTA-246H	-0.20259	0.126697	0.404008	None
6792	ATP6V0E2	0.264166	0.126764	0.40412	None
6793	CNTFR	-0.08543	0.126769	0.40412	None
6794	HIC2	-0.24496	0.126848	0.404313	None
6795	NIPA1	-0.13611	0.126868	0.404317	None
6796	AC145343.	-0.04862	0.126946	0.404504	None
6797	IRS4	0.096278	0.127046	0.404764	None
6798	ZNF672	0.010252	0.127076	0.4048	None
6799	CCL23	-0.30084	0.127256	0.405221	None
6800	RP11-686D	-0.20854	0.127259	0.405221	None
6801	NARS	-0.1294	0.127271	0.405221	None
6802	GIMAP8	-0.377	0.127298	0.405221	None
6803	LARGE	-0.0686	0.127331	0.405221	None
6804	BAHCC1	0.056634	0.127337	0.405221	None
6805	TRAF6	-0.18984	0.127339	0.405221	None
6806	U2SURP	-0.20084	0.12737	0.405261	None
6807	RP1-179N1	-0.14562	0.127438	0.405374	None
6808	TOMM22	-0.21185	0.127443	0.405374	None
6809	CCDC106	-0.10466	0.127478	0.405425	None
6810	PUS1	-0.29067	0.127529	0.405507	None
6811	CCDC158	0.005576	0.127541	0.405507	None
6812	TBC1D22B	0.22656	0.127616	0.405669	None
6813	ENTPD4	0.160471	0.127641	0.405669	None
6814	ZFP36L2	-0.48156	0.127649	0.405669	None
6815	LINC00032	0.021297	0.127699	0.40577	None
6816	RP11-352E	0.023808	0.127732	0.405816	None
6817	FZD1	-0.07966	0.127754	0.405825	None
6818	CASQ1	0.00551	0.127941	0.406359	None
6819	IGLL5	-0.73574	0.128025	0.406566	None
6820	TERF1	-0.17087	0.128089	0.40671	None
6821	RP11-432M	0.068387	0.128154	0.406859	None
6822	TDRD3	-0.22752	0.128223	0.406993	None
6823	CTA-445C9	-0.30306	0.128234	0.406993	None
6824	DPY19L2P4	-0.05218	0.12831	0.407175	None
6825	TK1	0.341663	0.128373	0.407313	None
6826	XPNPEP3	-0.21568	0.128459	0.407385	None
6827	AMELY	0.037219	0.128472	0.407385	None
6828	LOC100500	-0.13256	0.128472	0.407385	None
6829	CD99P1	-0.27751	0.128477	0.407385	None
6830	SV2A	-0.35667	0.128505	0.407385	None
6831	TMEM128	-0.24188	0.128521	0.407385	None
6832	LOC284889	-0.16177	0.128537	0.407385	None
6833	LRRC59	0.010879	0.128546	0.407385	None
6834	ENO3	-0.11547	0.128605	0.407512	None
6835	SLC11A1	0.126049	0.12863	0.407534	None
6836	TYMS	0.441265	0.128676	0.407617	None
6837	PRLR	0.006347	0.128727	0.407684	None
6838	PGBD5	-0.37946	0.128742	0.407684	None
6839	FOXN3-AS	0.019662	0.128753	0.407684	None
6840	LOH12CR2	-0.18086	0.128792	0.407746	None
6841	TET3	0.17603	0.128865	0.407917	None
6842	SCAMP3	-0.2078	0.129027	0.408373	None

6843	C22orf34	0.168026	0.129059	0.408411	None
6844	ORC6	0.260751	0.129077	0.408411	None
6845	ITPR1-AS1	-0.17588	0.129099	0.408421	None
6846	CD97	-0.40203	0.129145	0.408507	None
6847	PEAK1	-0.14128	0.129188	0.408582	None
6848	NRM	0.271201	0.129215	0.408586	None
6849	GK5	0.197905	0.129227	0.408586	None
6850	GUSB	0.117242	0.129323	0.408814	None
6851	IGF1	-0.50532	0.129346	0.408814	None
6852	S1PR4	0.204923	0.129355	0.408814	None
6853	CLSTN1	-0.25026	0.129419	0.408955	None
6854	TRIP10	-0.15424	0.129472	0.409064	None
6855	AHNAK	0.097735	0.129506	0.409112	None
6856	PRRT3	-0.16766	0.129548	0.409184	None
6857	SLC22A11	0.031073	0.129569	0.40919	None
6858	CYHR1	-0.10955	0.12959	0.409197	None
6859	SLC25A29	-0.32596	0.129652	0.409334	None
6860	FLJ33360	0.069679	0.12968	0.409362	None
6861	CBFB	0.157101	0.129742	0.409497	None
6862	CTB-92J24	0.231969	0.129804	0.409568	None
6863	DNTTIP1	-0.20792	0.129819	0.409568	None
6864	POC1B	0.297635	0.129821	0.409568	None
6865	AK025288	0.395168	0.129861	0.409635	None
6866	MTOR	-0.05851	0.129895	0.409682	None
6867	SLC47A1	-0.44772	0.129962	0.409835	None
6868	ANXA2P1	0.10583	0.129983	0.40984	None
6869	LDOC1	-0.36118	0.130005	0.409851	None
6870	LOC10192	-0.23285	0.130024	0.409851	None
6871	ZSCAN18	-0.3361	0.130068	0.409921	None
6872	RABGEF1	0.297928	0.130084	0.409921	None
6873	SEH1L	0.1759	0.130127	0.409944	None
6874	EMID1	-0.13374	0.13013	0.409944	None
6875	PGGT1B	0.320138	0.130183	0.410006	None
6876	RP11-495F	0.073441	0.130187	0.410006	None
6877	MTX3	0.235018	0.130242	0.410117	None
6878	GMDS	-0.21464	0.13026	0.410117	None
6879	CNIH4	-0.24229	0.130308	0.410202	None
6880	ZNF232	-0.38251	0.130325	0.410202	None
6881	PANX2	-0.0222	0.130402	0.410383	None
6882	XIST	1.631074	0.130464	0.410519	None
6883	RRAS2	-0.34658	0.13052	0.410536	None
6884	FAM218A	0.037151	0.130557	0.410536	None
6885	PHKB	-0.16178	0.130565	0.410536	None
6886	RASGEF1A	-0.25025	0.130574	0.410536	None
6887	SLC25A51	-0.22231	0.130581	0.410536	None
6888	POLD3	0.140378	0.130583	0.410536	None
6889	NUPR1	-0.41248	0.130629	0.410621	None
6890	PAPLN	-0.12844	0.130699	0.410779	None
6891	LAMTOR5-	-0.15214	0.130745	0.410779	None
6892	IDH3B	0.241923	0.130765	0.410779	None
6893	EIF3J	0.117011	0.130768	0.410779	None
6894	MTSS1	-0.12861	0.130774	0.410779	None
6895	TES	0.319679	0.13083	0.410834	None
6896	PNPLA4	-0.18864	0.130832	0.410834	None
6897	IGSF9B	0.024938	0.130849	0.410834	None
6898	PSMD7	-0.12651	0.130918	0.410974	None
6899	LOC10192	-0.34771	0.130931	0.410974	None
6900	ALG6	-0.24786	0.131031	0.411177	None

6901	IQGAP2	0.213309	0.131033	0.411177	None
6902	PLCD3	-0.04781	0.131081	0.411267	None
6903	FBXO5	0.282759	0.131257	0.411758	None
6904	ZNF554	-0.07934	0.131308	0.411854	None
6905	STEAP1	-0.30676	0.131359	0.411854	None
6906	CHD3	-0.22824	0.13136	0.411854	None
6907	SCN10A	0.05129	0.131363	0.411854	None
6908	RTP1	0.034241	0.131385	0.411863	None
6909	LOC10013(	0.05113	0.131465	0.412052	None
6910	LAPTM4A	0.16038	0.131602	0.412334	None
6911	RAB5A	-0.23629	0.131635	0.412334	None
6912	RP5-882O	0.040021	0.131637	0.412334	None
6913	PPP1R13L	-0.09889	0.131638	0.412334	None
6914	MAGEC3	0.019251	0.13165	0.412334	None
6915	JPH1	-0.17321	0.131729	0.412483	None
6916	RBAK	0.285105	0.131736	0.412483	None
6917	RNF130	0.250599	0.131906	0.412956	None
6918	TIMD4	-1.24957	0.131948	0.41303	None
6919	LINC00993	0.040969	0.131993	0.413112	None
6920	ZRSR2	-0.25885	0.132031	0.413118	None
6921	RNF217	-0.28197	0.132034	0.413118	None
6922	PAWR	-0.33296	0.132057	0.413133	None
6923	SERTAD1	0.393047	0.13212	0.413268	None
6924	ZCWPW1	-0.12128	0.132186	0.41336	None
6925	BIRC6	-0.19332	0.132227	0.41336	None
6926	CELF2	0.270636	0.132229	0.41336	None
6927	REC8	-0.18205	0.132245	0.41336	None
6928	HHLA1	-0.04775	0.132245	0.41336	None
6929	LOC10192	-0.33777	0.132269	0.413376	None
6930	CD109	0.364599	0.132291	0.413385	None
6931	L3MBTL3	-0.28912	0.132372	0.41358	None
6932	ZMYM5	0.309162	0.132423	0.413629	None
6933	DNAJA2	-0.16936	0.132426	0.413629	None
6934	MYO1C	-0.11559	0.132511	0.413737	None
6935	LINC01341	-0.3133	0.132517	0.413737	None
6936	G6PC3	-0.22273	0.13252	0.413737	None
6937	AK130486	0.056471	0.132537	0.413737	None
6938	RNF19B	-0.39591	0.132589	0.413803	None
6939	TIMM22	-0.24892	0.132605	0.413803	None
6940	C14orf142	-0.32891	0.132616	0.413803	None
6941	PPP1R7	-0.19668	0.132689	0.413971	None
6942	LOXL1	-0.19587	0.132734	0.414052	None
6943	PHAX	0.317529	0.132876	0.414437	None
6944	TNRC6C	-0.04969	0.13297	0.414669	None
6945	HTRA1	-0.35322	0.133042	0.414815	None
6946	ZBTB14	-0.1051	0.133055	0.414815	None
6947	SP3	0.285648	0.133088	0.414817	None
6948	IFT74	-0.25862	0.133094	0.414817	None
6949	NEDD1	0.217215	0.133175	0.41501	None
6950	LINC00851	0.069794	0.13321	0.415059	None
6951	CTTNBP2N	-0.28748	0.13326	0.415154	None
6952	TNNI3	0.069689	0.133308	0.415246	None
6953	TCAP	0.134334	0.133537	0.415898	None
6954	CAMK2G	-0.19028	0.133583	0.415914	None
6955	SPTB	-0.13642	0.133593	0.415914	None
6956	LINC01252	0.251484	0.1336	0.415914	None
6957	GPRC6A	0.020485	0.133643	0.415935	None
6958	GNL1	0.134899	0.133651	0.415935	None

6959	MTMR9LP	-0.04657	0.13369	0.415935	None
6960	BUD31	-0.10825	0.133701	0.415935	None
6961	ZBED3-AS:	-0.12053	0.133702	0.415935	None
6962	ERN1	0.242286	0.133774	0.416099	None
6963	NME3	-0.30017	0.133865	0.416322	None
6964	ZNHIT6	-0.1355	0.133931	0.416468	None
6965	PRPF38B	0.237065	0.133982	0.416565	None
6966	CHRDL1	0.7737	0.134032	0.416614	None
6967	LOC10260(	-0.2517	0.134047	0.416614	None
6968	MSL2	-0.35551	0.134055	0.416614	None
6969	P2RX4	-0.27677	0.134121	0.416745	None
6970	FAM131A	0.053076	0.134136	0.416745	None
6971	PKD2L1	-0.29291	0.13428	0.417074	None
6972	TACO1	-0.13663	0.13428	0.417074	None
6973	ZNF286A	0.147503	0.134371	0.417296	None
6974	MAPK7	-0.15684	0.134431	0.417421	None
6975	CENPC	0.221734	0.134454	0.417432	None
6976	KRT12	0.016229	0.134483	0.417465	None
6977	GIPC2	-0.50202	0.13455	0.41755	None
6978	PEMT	-0.26513	0.134569	0.41755	None
6979	MR1	-0.20014	0.134569	0.41755	None
6980	TUBA1C	0.148012	0.134718	0.417954	None
6981	SUMO1	0.148602	0.134749	0.41799	None
6982	PIGO	0.116617	0.134832	0.418187	None
6983	TBX21	0.048202	0.134858	0.418209	None
6984	CEP63	-0.34228	0.134892	0.418248	None
6985	EPX	-0.4353	0.13491	0.418248	None
6986	LINC00998	0.287843	0.134957	0.418311	None
6987	LINC00934	0.008976	0.134968	0.418311	None
6988	DUXAP10	-0.32257	0.135015	0.418396	None
6989	C19orf26	0.052896	0.13504	0.418414	None
6990	NEK6	0.195153	0.135097	0.418529	None
6991	SH2D3A	-0.04199	0.13521	0.418811	None
6992	SMIM17	-0.21978	0.135226	0.418811	None
6993	IL6ST	-0.3897	0.135247	0.418816	None
6994	DCBLD2	-0.16418	0.135299	0.418917	None
6995	PDK2	-0.12832	0.135323	0.418932	None
6996	TBC1D15	-0.1593	0.13536	0.418968	None
6997	TCEB3-AS:	-0.28598	0.135374	0.418968	None
6998	IGFBP4	-0.21823	0.135424	0.419009	None
6999	RRS1	-0.22482	0.135466	0.419009	None
7000	TRPT1	-0.1903	0.135481	0.419009	None
7001	KDM5D	-1.22718	0.13549	0.419009	None
7002	PIF1	-0.0895	0.135512	0.419009	None
7003	NPHP4	-0.09498	0.135518	0.419009	None
7004	VAC14	0.101978	0.135522	0.419009	None
7005	CEP57L1	-0.09337	0.135607	0.419209	None
7006	USP18	-0.48782	0.135626	0.419211	None
7007	SEC22A	-0.1723	0.13566	0.419255	None
7008	SLC26A1	-0.02864	0.13587	0.419817	None
7009	LOC72986(	0.039259	0.135881	0.419817	None
7010	SIRT2	-0.15041	0.135942	0.419948	None
7011	DTL	0.31337	0.135986	0.420022	None
7012	EDAR	0.081669	0.13603	0.42006	None
7013	RP11-506E	0.010838	0.136037	0.42006	None
7014	LRRC46	0.051638	0.136081	0.420072	None
7015	HIST1H4D	-0.12954	0.136092	0.420072	None
7016	AMPD3	-0.23669	0.136099	0.420072	None

7017	SLC2A5	0.311534	0.136193	0.420277	None
7018	KANK2	0.085581	0.136204	0.420277	None
7019	IGLON5	0.018439	0.136244	0.420339	None
7020	LRRC75B	-0.04282	0.13635	0.420547	None
7021	RP11-210M	-0.05303	0.136367	0.420547	None
7022	TMED6	0.306538	0.136387	0.420547	None
7023	YIPF6	-0.162	0.136389	0.420547	None
7024	CADPS2	-0.50751	0.136424	0.420595	None
7025	ZFPM2	-0.44148	0.136502	0.420776	None
7026	VAMP4	-0.34837	0.136566	0.420864	None
7027	SFRP5	-0.20713	0.136569	0.420864	None
7028	EPS8	-0.74025	0.136647	0.420992	None
7029	VEGFA	0.159803	0.13665	0.420992	None
7030	POMGNT1	0.026589	0.136817	0.421448	None
7031	RGS13	0.144176	0.136939	0.421763	None
7032	POLE	-0.28342	0.136983	0.42184	None
7033	LOC28386	-0.24335	0.137043	0.421965	None
7034	SLC24A4	-0.25364	0.137192	0.422361	None
7035	FAM172A	-0.22873	0.137211	0.422361	None
7036	RABGAP1L	-0.19109	0.137279	0.422509	None
7037	TAL2	-0.04603	0.137323	0.422539	None
7038	KLF12	0.146458	0.137328	0.422539	None
7039	UPF3A	0.137465	0.137347	0.422539	None
7040	EDNRB	-0.65223	0.137382	0.422585	None
7041	WDR89	-0.23538	0.137448	0.422729	None
7042	GLUL	-0.5355	0.137585	0.423044	None
7043	FGFRL1	-0.04983	0.137594	0.423044	None
7044	SPESP1	-0.65192	0.137609	0.423044	None
7045	TMTC2	-0.24153	0.137649	0.423107	None
7046	ALPK3	-0.23513	0.137728	0.423289	None
7047	LRP6	0.136192	0.137756	0.423315	None
7048	CDK8	0.257338	0.137803	0.423401	None
7049	CA8	-0.11217	0.137865	0.42353	None
7050	OAT	-0.23945	0.137935	0.423627	None
7051	NIPSNAP3	0.327621	0.137952	0.423627	None
7052	KRT35	0.002136	0.137985	0.423627	None
7053	FAM9B	0.006658	0.137991	0.423627	None
7054	NDUFS2	-0.17892	0.13801	0.423627	None
7055	SIRT1	0.247131	0.138014	0.423627	None
7056	TBP	0.166948	0.138047	0.42367	None
7057	ZNF18	-0.12471	0.138086	0.423682	None
7058	RECQL5	-0.08644	0.138091	0.423682	None
7059	ABT1	0.215196	0.138116	0.4237	None
7060	MMAA	-0.1797	0.138146	0.423732	None
7061	MAP2K2	-0.20794	0.138219	0.423895	None
7062	SIK2	0.106464	0.138263	0.423897	None
7063	SEC24B-AS	-0.19331	0.138278	0.423897	None
7064	ULK1	-0.21503	0.138286	0.423897	None
7065	VSIG4	-0.99323	0.138297	0.423897	None
7066	SETD6	-0.21619	0.138358	0.424023	None
7067	AHCYL1	-0.22396	0.138428	0.424121	None
7068	ZNF557	-0.08792	0.138429	0.424121	None
7069	JPX	-0.52924	0.138643	0.424716	None
7070	CENPBD1F	-0.21678	0.138747	0.424975	None
7071	MTM1	0.164633	0.138799	0.425019	None
7072	CLIC6	-0.27517	0.13881	0.425019	None
7073	GRHL1	-0.14954	0.138821	0.425019	None
7074	DIO3OS	0.016033	0.138909	0.425207	None

7075	ARMCX6	-0.11403	0.138935	0.425207	None
7076	SIX3	0.010102	0.138955	0.425207	None
7077	PECR	-0.10984	0.138961	0.425207	None
7078	STAT5B	-0.12687	0.138993	0.425246	None
7079	DROSHA	-0.17844	0.139171	0.4257	None
7080	SPHK1	0.212394	0.139181	0.4257	None
7081	NIM1K	0.034867	0.139295	0.425989	None
7082	FOXJ2	0.077831	0.139357	0.42612	None
7083	RDX	-0.24499	0.139428	0.426226	None
7084	LOC15786(	-0.11053	0.139437	0.426226	None
7085	IRF4	0.01685	0.139451	0.426226	None
7086	C4orf48	-0.25657	0.13949	0.426286	None
7087	NDUFAF7	-0.16426	0.139554	0.42642	None
7088	HCG27	-0.17483	0.139591	0.426473	None
7089	IL4I1	0.143091	0.139668	0.426649	None
7090	ZNF382	-0.12878	0.139745	0.426825	None
7091	ZNF271	-0.21182	0.139767	0.426829	None
7092	ETV6	-0.23591	0.139931	0.427243	None
7093	ENDOG	-0.3298	0.139942	0.427243	None
7094	SLC22A17	-0.09908	0.139997	0.427352	None
7095	TBC1D1	0.144166	0.140037	0.427415	None
7096	ALOX12B	-0.07734	0.140159	0.427726	None
7097	LOC14870!	0.022817	0.140224	0.427864	None
7098	BMPR1B	-0.27722	0.140254	0.427896	None
7099	PPP1R32	0.051679	0.140359	0.428028	None
7100	APRT	-0.21947	0.140364	0.428028	None
7101	AP001171.	-0.43617	0.140364	0.428028	None
7102	CDC42-IT1	0.390504	0.140377	0.428028	None
7103	ITGB1	-0.0903	0.140403	0.428049	None
7104	WNT11	-0.17157	0.140469	0.428191	None
7105	TM7SF2	-0.23804	0.140509	0.42825	None
7106	MAS1L	0.023075	0.140557	0.42829	None
7107	SLC26A6	0.388862	0.140561	0.42829	None
7108	SKIV2L2	-0.16927	0.140691	0.428623	None
7109	RBL2	0.166653	0.140795	0.428881	None
7110	LRRN1	-0.50675	0.140846	0.428975	None
7111	DCSTAMP	0.078047	0.140899	0.429076	None
7112	INPPL1	-0.13208	0.140925	0.429097	None
7113	WAC-AS1	-0.20824	0.14111	0.4296	None
7114	RABL3	-0.17799	0.141264	0.430007	None
7115	TRIM32	-0.21872	0.141339	0.430176	None
7116	DNHD1	-0.07332	0.141489	0.430509	None
7117	NMNAT3	-0.19462	0.141496	0.430509	None
7118	ALPK1	-0.22681	0.141525	0.430509	None
7119	NRD1	-0.16972	0.141528	0.430509	None
7120	RP1-305G2	0.029533	0.141697	0.430962	None
7121	UPK2	0.021251	0.141848	0.431313	None
7122	PPM1M	-0.3222	0.141868	0.431313	None
7123	KIF6	-0.01208	0.141872	0.431313	None
7124	MAPRE2	-0.26508	0.141909	0.431328	None
7125	RPAIN	-0.11675	0.141917	0.431328	None
7126	CUL7	-0.08832	0.141964	0.431361	None
7127	LOC10192	-0.25551	0.141968	0.431361	None
7128	PRRG4	-0.29041	0.142033	0.431498	None
7129	UNC5CL	-0.22058	0.14206	0.431522	None
7130	AGFG1	0.277611	0.142093	0.431559	None
7131	SPATA18	-0.07421	0.142189	0.431773	None
7132	LINC00844	0.002991	0.142203	0.431773	None

7133	ACSM2B	0.007561	0.142251	0.431842	None
7134	KIF26A	-0.21201	0.142266	0.431842	None
7135	UMPS	-0.17856	0.14231	0.431859	None
7136	TOP3B	-0.07845	0.142311	0.431859	None
7137	GLYR1	-0.08718	0.142429	0.432137	None
7138	TMED2	-0.57204	0.142442	0.432137	None
7139	SNHG4	-0.08506	0.142499	0.432249	None
7140	ACTRT3	-0.11836	0.14261	0.432524	None
7141	DOPEY1	0.147313	0.14266	0.432575	None
7142	LINC00996	-0.33846	0.142673	0.432575	None
7143	KLHL23	-0.32093	0.142687	0.432575	None
7144	GYPB	-0.47815	0.142849	0.432903	None
7145	MYOG	-0.08282	0.142862	0.432903	None
7146	POLR2G	-0.13179	0.14287	0.432903	None
7147	PUM1	-0.18933	0.142875	0.432903	None
7148	TBC1D21	0.020633	0.142902	0.432926	None
7149	PNPLA3	-0.16635	0.14294	0.432979	None
7150	GPR124	-0.19139	0.142997	0.433092	None
7151	ANKRD10-	0.263366	0.143048	0.433123	None
7152	CKMT2	0.098313	0.143048	0.433123	None
7153	GAMT	-0.3093	0.143072	0.433135	None
7154	NT5C2	0.19577	0.143232	0.43356	None
7155	CDK9	0.257449	0.143274	0.433626	None
7156	NUMA1	-0.1769	0.14348	0.434139	None
7157	SNRPD3	0.181293	0.143483	0.434139	None
7158	AP1S1	-0.26711	0.143664	0.434585	None
7159	UTP11L	-0.08673	0.143672	0.434585	None
7160	RPL30	-0.05462	0.143703	0.434585	None
7161	RSPH1	-0.37898	0.143711	0.434585	None
7162	TESC	-0.25986	0.143752	0.434649	None
7163	RBM39	0.239545	0.143838	0.434847	None
7164	CPNE7	-0.13444	0.143938	0.435089	None
7165	ANP32A-IT	-0.33236	0.144	0.435141	None
7166	CSF1	0.046032	0.14401	0.435141	None
7167	GSPT1	0.193194	0.144015	0.435141	None
7168	RCHY1	-0.15518	0.144036	0.435141	None
7169	CC2D1A	-0.08932	0.144083	0.435225	None
7170	SEC24D	-0.26	0.144153	0.435342	None
7171	ZC3HAV1	0.168412	0.144162	0.435342	None
7172	ZCCHC2	-0.27334	0.14421	0.435426	None
7173	PLEKHG1	-0.37566	0.144253	0.435493	None
7174	GP1BA	-0.45634	0.144285	0.43553	None
7175	ROM1	-0.12017	0.144316	0.435562	None
7176	AEN	-0.2333	0.144403	0.435729	None
7177	AIF1L	-0.27968	0.144411	0.435729	None
7178	GNAS-AS1	-0.1322	0.1445	0.435936	None
7179	LOC10192(	0.008587	0.144542	0.436003	None
7180	OGFRL1	0.224812	0.144617	0.436168	None
7181	FAM207A	-0.15476	0.144687	0.436273	None
7182	KCNQ1-AS	0.077199	0.144692	0.436273	None
7183	GSTT1	-0.31687	0.144744	0.436368	None
7184	FNIP1	0.22916	0.14479	0.436436	None
7185	STAM-AS1	0.061724	0.144807	0.436436	None
7186	HPS3	-0.15712	0.144874	0.436515	None
7187	KCNMB3	0.184876	0.144888	0.436515	None
7188	NDFIP2	-0.30593	0.144893	0.436515	None
7189	EFCAB7	-0.23365	0.144932	0.436539	None
7190	LY6G6E	0.027228	0.144958	0.436539	None

7191	COPG2IT1	-0.40229	0.144962	0.436539	None
7192	LOC10192!	0.024437	0.145076	0.436823	None
7193	CENPJ	-0.33003	0.145139	0.436855	None
7194	ZNF610	-0.27704	0.14515	0.436855	None
7195	CENPT	-0.05067	0.145182	0.436855	None
7196	PSMC1	0.185707	0.145189	0.436855	None
7197	HIPK3	-0.13774	0.145204	0.436855	None
7198	SLC37A2	-0.30246	0.145208	0.436855	None
7199	ZXDB	0.123007	0.14527	0.436981	None
7200	UBN1	-0.15037	0.145303	0.43702	None
7201	UBR2	-0.12439	0.145449	0.437397	None
7202	ZNF217	0.32999	0.145564	0.437681	None
7203	SRRM2	-0.18943	0.145595	0.437685	None
7204	MIP	-0.0581	0.145605	0.437685	None
7205	RP1-193H1	0.16231	0.145683	0.437858	None
7206	FBXO8	-0.25586	0.145705	0.437864	None
7207	RP11-135A	0.058708	0.145774	0.43801	None
7208	C1orf27	-0.14197	0.145807	0.438028	None
7209	LOC10065	0.011864	0.145821	0.438028	None
7210	CAMK2D	-0.29068	0.145876	0.438118	None
7211	CYTH3	0.034853	0.145898	0.438118	None
7212	ATP6	-0.13438	0.145941	0.438118	None
7213	PITPNA-AS	-0.21224	0.145949	0.438118	None
7214	DNPEP	-0.26774	0.145952	0.438118	None
7215	BABAM1	-0.18777	0.146004	0.438214	None
7216	C1orf233	-0.11657	0.146115	0.438368	None
7217	TSPAN18	-0.1852	0.146125	0.438368	None
7218	BCAP29	-0.19154	0.146127	0.438368	None
7219	SLA2	-0.1971	0.146136	0.438368	None
7220	SLC1A5	-0.23584	0.146297	0.438755	None
7221	RP11-672L	-0.31576	0.146306	0.438755	None
7222	ERMARD	-0.19795	0.146364	0.438832	None
7223	CD300LB	0.051847	0.146399	0.438832	None
7224	C10orf25	0.491979	0.146404	0.438832	None
7225	RP11-90P1	0.066236	0.146412	0.438832	None
7226	SLC35B1	0.208216	0.14645	0.438885	None
7227	ADAMTS3	-0.52517	0.146661	0.439392	None
7228	MAN1A2	-0.14804	0.146664	0.439392	None
7229	IP6K1	-0.20126	0.14668	0.439392	None
7230	TREML5P	0.008233	0.146803	0.439698	None
7231	MRPL3	-0.14289	0.146961	0.440111	None
7232	NAPA-AS1	-0.05243	0.147059	0.440344	None
7233	KIAA0408	0.004809	0.147099	0.440401	None
7234	ALMS1-IT1	0.418813	0.147146	0.440445	None
7235	RP2	0.189126	0.147158	0.440445	None
7236	MS4A14	-0.28222	0.147174	0.440445	None
7237	RP1-39G2	-0.2155	0.147207	0.440482	None
7238	EIF2B5	-0.08406	0.14726	0.440581	None
7239	TRIM71	-0.52561	0.147396	0.440926	None
7240	NDRG3	-0.28593	0.147578	0.441409	None
7241	TMCC1	0.233025	0.147659	0.441497	None
7242	RP11-845C	0.043644	0.147665	0.441497	None
7243	GLTSCR1	-0.13822	0.147669	0.441497	None
7244	GTF2IRD2	0.15578	0.147783	0.441779	None
7245	AK094644	-0.02323	0.147827	0.441848	None
7246	FRMD6	0.5438	0.14788	0.441945	None
7247	SMAD3	0.092935	0.147967	0.4421	None
7248	ZNF34	-0.20754	0.148003	0.4421	None

7249	VAMP2	-0.18753	0.148006	0.4421	None
7250	AGPAT9	-0.56482	0.148013	0.4421	None
7251	CA5BP1	-0.19447	0.148084	0.442198	None
7252	CTD-2325/	-0.32612	0.148087	0.442198	None
7253	LGALS3	-0.42524	0.148135	0.442282	None
7254	LINC00661	0.025782	0.14817	0.442326	None
7255	LIMK2	-0.22232	0.148199	0.442349	None
7256	ITGB1BP2	-0.1155	0.148317	0.442619	None
7257	DLST	-0.22499	0.14833	0.442619	None
7258	HBD	-0.67611	0.14836	0.442649	None
7259	TNKS1BP1	-0.07965	0.148445	0.442813	None
7260	SETX	-0.25931	0.148462	0.442813	None
7261	LILRB5	-0.58849	0.148511	0.442813	None
7262	CDC25C	0.030471	0.148519	0.442813	None
7263	CR936796	-0.46691	0.148537	0.442813	None
7264	MAGEL2	-0.27382	0.148538	0.442813	None
7265	KRTAP13-1	0.029572	0.148572	0.442852	None
7266	PRKXP1	-0.13515	0.148602	0.442861	None
7267	C11orf65	0.116153	0.148616	0.442861	None
7268	LOC10012	0.007357	0.148656	0.442921	None
7269	TKTL1	0.094901	0.148736	0.443097	None
7270	SBF1	-0.16927	0.148768	0.443131	None
7271	YARS2	-0.13887	0.148811	0.443198	None
7272	SEC11C	0.149437	0.148857	0.443277	None
7273	10-Sep	-0.45483	0.14891	0.443336	None
7274	RECQL4	-0.28934	0.148918	0.443336	None
7275	FBXL7	0.178476	0.148944	0.443353	None
7276	SMEK3P	0.002132	0.149042	0.443581	None
7277	STX6	-0.15848	0.149108	0.443717	None
7278	SRSF12	-0.11156	0.149226	0.443921	None
7279	PAIP2B	-0.25854	0.149237	0.443921	None
7280	GABARAPL	0.355802	0.149238	0.443921	None
7281	SH3RF1	0.226179	0.149263	0.443936	None
7282	BRIP1	0.291627	0.149325	0.44403	None
7283	MGAT3	-0.16826	0.149355	0.44403	None
7284	YES1	-0.37041	0.149363	0.44403	None
7285	BC043540	-0.03327	0.149377	0.44403	None
7286	AREL1	0.104468	0.149453	0.444196	None
7287	HNRNPU-/	0.348015	0.149527	0.444355	None
7288	CELF1	0.20537	0.149592	0.444432	None
7289	VAMP5	0.205581	0.149594	0.444432	None
7290	SRPX	-0.5254	0.14964	0.444507	None
7291	RP11-1007	-0.09549	0.149662	0.444511	None
7292	NMNAT1	-0.1372	0.1497	0.444562	None
7293	ARAP3	-0.08362	0.149766	0.444697	None
7294	DPH6	-0.27339	0.149891	0.445008	None
7295	MYBPC2	0.025004	0.149927	0.445035	None
7296	RTTN	0.340041	0.149941	0.445035	None
7297	KRT18	-0.66653	0.150028	0.445209	None
7298	ARHGAP3(	-0.18705	0.150041	0.445209	None
7299	DSC3	0.002029	0.150066	0.445223	None
7300	KSR1	-0.03363	0.150143	0.445389	None
7301	NAA38	-0.27052	0.150218	0.445551	None
7302	PAF1	0.19497	0.150258	0.445569	None
7303	ADAM10	-0.26605	0.150265	0.445569	None
7304	CD226	-0.47581	0.150396	0.445826	None
7305	SETD3	-0.09945	0.15042	0.445826	None
7306	GDF11	-0.24532	0.150431	0.445826	None

7307	LTC4S	-0.26208	0.150434	0.445826	None
7308	ACRC	0.307896	0.150487	0.445922	None
7309	SSRP1	-0.20064	0.15061	0.446163	None
7310	GPR162	-0.16749	0.150614	0.446163	None
7311	RBPMS	-0.36449	0.150641	0.446163	None
7312	ITPRIPL1	-0.27166	0.150651	0.446163	None
7313	SMIM13	-0.19645	0.150694	0.446229	None
7314	S100B	-0.08851	0.1508	0.446429	None
7315	TEX37	0.051137	0.150803	0.446429	None
7316	MMP1	-0.26753	0.150851	0.446513	None
7317	HAT1	0.152623	0.150931	0.446673	None
7318	GSTM2	-0.40344	0.150947	0.446673	None
7319	KMT2E-AS	-0.10242	0.150971	0.446685	None
7320	KCTD11	-0.1566	0.151015	0.446751	None
7321	KCNJ10	-0.18119	0.151087	0.446904	None
7322	FNIP2	-0.21475	0.151124	0.446936	None
7323	CST3	-0.1421	0.151163	0.446936	None
7324	N4BP1	-0.10754	0.151168	0.446936	None
7325	LCORL	0.252985	0.15118	0.446936	None
7326	GLCCI1	-0.19563	0.151271	0.447144	None
7327	IL1RN	0.093038	0.151345	0.4473	None
7328	SLC25A27	-0.22097	0.151372	0.44732	None
7329	POP4	-0.123	0.151451	0.447384	None
7330	AKAP13	0.221937	0.151451	0.447384	None
7331	PRDX6	-0.08801	0.151456	0.447384	None
7332	OLIG2	0.020793	0.151486	0.447414	None
7333	LOC90246	-0.06592	0.15154	0.447458	None
7334	CDK2AP1	-0.15197	0.151543	0.447458	None
7335	EU250746	0.14487	0.151566	0.447466	None
7336	CD58	0.244814	0.151631	0.447555	None
7337	SYCE2	-0.14775	0.151638	0.447555	None
7338	WDR1	-0.3245	0.152003	0.448573	None
7339	OR51I2	0.057651	0.152033	0.4486	None
7340	KCNS2	0.003933	0.152072	0.448644	None
7341	SH3BGRL	-0.13791	0.152089	0.448644	None
7342	KCNJ5	-0.12929	0.152175	0.448836	None
7343	CCDC82	0.09925	0.15228	0.449044	None
7344	WDFY4	-0.11437	0.152306	0.449044	None
7345	TPRG1L	-0.20877	0.152308	0.449044	None
7346	CUL3	0.170777	0.152367	0.449107	None
7347	HSD3B1	0.002133	0.152378	0.449107	None
7348	ZNF35	0.274107	0.152418	0.449107	None
7349	CHEK2	-0.28981	0.152422	0.449107	None
7350	SAMD3	-0.12728	0.152433	0.449107	None
7351	RPS3	0.060496	0.152488	0.449186	None
7352	FAM65C	-0.23552	0.152501	0.449186	None
7353	KIF5A	0.045698	0.152583	0.449365	None
7354	ALDOA	-0.15821	0.152671	0.449562	None
7355	MGC16142	-0.04492	0.152753	0.449743	None
7356	DYX1C1	-0.22502	0.15281	0.449851	None
7357	NDUFAF1	-0.24715	0.152989	0.450316	None
7358	FRMD4A	-0.1734	0.153257	0.451044	None
7359	KDELR2	0.225041	0.153304	0.45112	None
7360	DAAM1	-0.36645	0.153325	0.451121	None
7361	LOC22112	0.032646	0.153426	0.451358	None
7362	KIAA0226	0.089141	0.153465	0.451364	None
7363	MAP4K3	0.396494	0.15347	0.451364	None
7364	DCAF15	-0.07341	0.153602	0.451692	None

7365	LOC20077	-0.4941	0.153715	0.451929	None
7366	CDC42EP1	0.04457	0.153725	0.451929	None
7367	RP5-1180E	-0.09381	0.153858	0.452197	None
7368	XPOT	-0.12704	0.153858	0.452197	None
7369	LINC00965	-0.33991	0.154008	0.452494	None
7370	ILKAP	-0.2164	0.154022	0.452494	None
7371	DOK3	-0.15738	0.15404	0.452494	None
7372	ARMC8	0.15362	0.154042	0.452494	None
7373	USP27X-A	-0.07541	0.154242	0.453021	None
7374	KCNJ9	0.018656	0.154263	0.453021	None
7375	TRAPPC3L	0.19961	0.154301	0.453068	None
7376	SLFN12	-0.26818	0.154415	0.453284	None
7377	MPHOSPH	-0.16561	0.154416	0.453284	None
7378	SAMD1	-0.09225	0.154464	0.453364	None
7379	HIST1H3E	0.098292	0.154517	0.453456	None
7380	WDR7	-0.10496	0.154592	0.453617	None
7381	KHSRP	-0.0821	0.15474	0.453988	None
7382	RP11-155	0.127052	0.154761	0.453991	None
7383	TNKS	-0.04092	0.154806	0.45406	None
7384	POLR3GL	-0.15386	0.154955	0.454435	None
7385	RP4-539M	0.029847	0.155054	0.454616	None
7386	TRAFD1	-0.13735	0.155075	0.454616	None
7387	MSH6	-0.46537	0.155087	0.454616	None
7388	LOC10099	0.148047	0.155105	0.454616	None
7389	PLCH2	0.041391	0.155122	0.454616	None
7390	NRP1	-0.2333	0.155162	0.454623	None
7391	LOC72873	-0.10446	0.155171	0.454623	None
7392	CTD-2616	0.031505	0.155187	0.454623	None
7393	LAMP1	-0.20443	0.155354	0.45505	None
7394	GLT8D1	-0.16328	0.15544	0.455241	None
7395	NAP1L3	0.600637	0.155466	0.455255	None
7396	PRMT7	-0.28338	0.155703	0.455828	None
7397	AMZ2P1	-0.22075	0.155703	0.455828	None
7398	TMEM252	0.055027	0.155763	0.455932	None
7399	SMC1A	0.109878	0.155791	0.455932	None
7400	ZNF292	0.340307	0.155822	0.455932	None
7401	LOC10050	0.002272	0.155823	0.455932	None
7402	FAM188A	-0.15189	0.155851	0.455952	None
7403	GSAP	0.305353	0.155891	0.456008	None
7404	IRGQ	0.121554	0.156028	0.456347	None
7405	SMPD3	0.063243	0.156074	0.456402	None
7406	TTBK2	-0.14688	0.156108	0.456402	None
7407	ANGPT1	-0.46016	0.156117	0.456402	None
7408	LOC10028	-0.15278	0.156137	0.456402	None
7409	UNK	0.295615	0.156158	0.456402	None
7410	LINC00526	-0.27883	0.156177	0.456402	None
7411	ZBTB46	-0.09804	0.156195	0.456402	None
7412	HIBADH	-0.33254	0.156268	0.45655	None
7413	NUS1P3	-0.13808	0.156306	0.45655	None
7414	AIRE	0.094106	0.156309	0.45655	None
7415	DDX49	-0.20694	0.156349	0.456551	None
7416	RP11-366L	-0.08933	0.156351	0.456551	None
7417	LINC00982	-0.17072	0.156411	0.456666	None
7418	RP11-209A	0.202445	0.156467	0.456746	None
7419	CYP20A1	-0.19586	0.156481	0.456746	None
7420	LOC28627	-0.42649	0.156502	0.456746	None
7421	ZMYM3	-0.34306	0.156578	0.456907	None
7422	IGIP	-0.20924	0.156665	0.457099	None

7423	CENPQ	0.260289	0.156761	0.457317	None
7424	TPM1	-0.29029	0.156891	0.457579	None
7425	SERINC3	-0.16469	0.156893	0.457579	None
7426	RAD51	0.212544	0.157177	0.458282	None
7427	ZNF876P	0.041891	0.157191	0.458282	None
7428	BIN3-IT1	-0.18173	0.157198	0.458282	None
7429	LINC00544	0.036158	0.157367	0.458675	None
7430	SHC2	-0.09163	0.157375	0.458675	None
7431	IFNAR1	-0.20052	0.157441	0.458764	None
7432	TP53BP1	-0.15323	0.157448	0.458764	None
7433	PHF5A	-0.3136	0.157506	0.458772	None
7434	YME1L1	0.170177	0.15751	0.458772	None
7435	KLK4	0.008655	0.157519	0.458772	None
7436	CCNA1	-0.59066	0.157535	0.458772	None
7437	RPS17	0.0759	0.157655	0.459058	None
7438	BC041025	-0.41579	0.157691	0.459094	None
7439	GPR146	-0.34734	0.157737	0.459094	None
7440	NCBP2	-0.14449	0.157746	0.459094	None
7441	PSME1	0.156544	0.157752	0.459094	None
7442	WDR26	-0.21333	0.157882	0.459295	None
7443	FBRSL1	-0.08404	0.157895	0.459295	None
7444	RAB11FIP3	0.161017	0.157901	0.459295	None
7445	TNIK	-0.30961	0.157906	0.459295	None
7446	CNOT8	0.194707	0.157981	0.459453	None
7447	INPP5B	-0.16065	0.158004	0.459457	None
7448	UBQLN2	-0.20261	0.158085	0.459604	None
7449	SNX32	-0.05763	0.158097	0.459604	None
7450	CAMK1	-0.16008	0.158136	0.459608	None
7451	SREK1	0.203789	0.158165	0.459608	None
7452	BIRC5	-0.47239	0.158168	0.459608	None
7453	APBA1	-0.12168	0.158191	0.459608	None
7454	KLHL18	-0.16422	0.158204	0.459608	None
7455	KPNA4	-0.16675	0.158379	0.460053	None
7456	LINC00958	0.061893	0.158416	0.460098	None
7457	STOML2	0.199697	0.158455	0.460139	None
7458	ZNF781	-0.09896	0.158517	0.460139	None
7459	LINC01362	0.017348	0.158522	0.460139	None
7460	TSC1	-0.13303	0.158527	0.460139	None
7461	NUFIP2	-0.15552	0.158544	0.460139	None
7462	ACYP1	-0.22271	0.158576	0.460139	None
7463	NT5DC3	-0.10652	0.158579	0.460139	None
7464	CHRNB3	0.003034	0.158613	0.460176	None
7465	RP11-5N1:	0.045632	0.158723	0.460413	None
7466	LRRC66	-0.04346	0.158737	0.460413	None
7467	KY	-0.01511	0.158782	0.460484	None
7468	KCNF1	0.107176	0.158835	0.460557	None
7469	HOOK2	-0.0995	0.15885	0.460557	None
7470	RUFY3	0.25812	0.159042	0.461052	None
7471	SLC44A4	0.035131	0.159134	0.461129	None
7472	WWP1	-0.36765	0.159155	0.461129	None
7473	WEE2-AS1	-0.03221	0.159157	0.461129	None
7474	PPM1L	0.138428	0.159183	0.461129	None
7475	LINC01144	0.014037	0.1592	0.461129	None
7476	LSM4	-0.38246	0.159216	0.461129	None
7477	C19orf70	-0.3122	0.159218	0.461129	None
7478	DDX11	-0.19306	0.15927	0.46122	None
7479	TTC32	-0.17212	0.159326	0.461319	None
7480	USP11	-0.24606	0.159406	0.461456	None

7481	ZNF341	-0.07908	0.159428	0.461456 None
7482	NIPA2	-0.15069	0.159472	0.461456 None
7483	LYZL4	-0.02359	0.159486	0.461456 None
7484	LOC10012{	0.045987	0.159498	0.461456 None
7485	FAM91A1	0.10109	0.159501	0.461456 None
7486	RPS14	0.06343	0.15963	0.461766 None
7487	SNRNP48	-0.17234	0.159667	0.461811 None
7488	SERPINB6	-0.21406	0.159696	0.461833 None
7489	CRISP2	-0.28091	0.159722	0.461847 None
7490	NOVA1	-0.12161	0.159776	0.461942 None
7491	CLCN1	-0.04309	0.159818	0.462003 None
7492	TUBA4A	0.359084	0.159952	0.462329 None
7493	KLF3	0.288543	0.160112	0.462575 None
7494	BLOC1S1	-0.26437	0.160116	0.462575 None
7495	PRKAA1	0.28299	0.160121	0.462575 None
7496	FRAT1	-0.20845	0.160123	0.462575 None
7497	RP4-730D4	0.013564	0.160277	0.462958 None
7498	C11orf73	-0.21512	0.160423	0.463259 None
7499	TUBA1B	0.158295	0.160453	0.463259 None
7500	ERI2	-0.08925	0.160461	0.463259 None
7501	EIF2D	-0.17228	0.160467	0.463259 None
7502	MEF2BNB	-0.16329	0.160501	0.463259 None
7503	USP24	-0.19198	0.160509	0.463259 None
7504	KIAA0101	0.22866	0.160618	0.463511 None
7505	TTC26	0.137605	0.16071	0.463709 None
7506	FLJ36848	-0.16667	0.160741	0.463709 None
7507	CA2	-0.70248	0.160751	0.463709 None
7508	PAK4	-0.14465	0.160889	0.463937 None
7509	EIF1AX	0.199556	0.160892	0.463937 None
7510	UTP15	-0.10869	0.160894	0.463937 None
7511	LRP1	-0.12669	0.160962	0.46405 None
7512	TSFM	-0.23564	0.160976	0.46405 None
7513	TNFSF9	-0.16136	0.160999	0.464055 None
7514	CTA-390C1	-0.26882	0.16103	0.464081 None
7515	LOC28563{	0.030643	0.161069	0.464133 None
7516	ANKRD53	0.045477	0.161125	0.464163 None
7517	STX11	0.314756	0.161133	0.464163 None
7518	CCDC107	0.250976	0.161144	0.464163 None
7519	APBB1IP	0.293347	0.161168	0.46417 None
7520	RABIF	-0.14199	0.161201	0.464202 None
7521	LOC28378{	-0.20767	0.161251	0.464286 None
7522	CCDC157	-0.05746	0.161319	0.464419 None
7523	TMEM75	0.018529	0.161368	0.464499 None
7524	PABPC1	-0.06978	0.1614	0.46453 None
7525	GHITM	0.136811	0.161447	0.464603 None
7526	ARPC3	0.190058	0.161615	0.465025 None
7527	HOXA6	-0.24269	0.161678	0.465143 None
7528	IFT20	-0.13295	0.161768	0.465311 None
7529	ZNF266	0.207368	0.161799	0.465311 None
7530	USP53	0.201079	0.161801	0.465311 None
7531	SPATA33	-0.12189	0.161831	0.465338 None
7532	LOC38976!	-0.24541	0.161896	0.465462 None
7533	KCNH3	-0.05441	0.161931	0.465499 None
7534	NOTCH1	-0.13412	0.161977	0.465536 None
7535	PIGT	0.127936	0.162006	0.465536 None
7536	TLCD2	-0.08942	0.162008	0.465536 None
7537	CRB1	-0.11023	0.162065	0.465638 None
7538	TRMT11	-0.233	0.162112	0.465713 None

7539	LINC00092	0.081073	0.162145	0.465729	None
7540	AGTR1	0.074033	0.162176	0.465729	None
7541	BCL2L10	-0.09186	0.162194	0.465729	None
7542	UHRF2	0.154731	0.162204	0.465729	None
7543	GFPT2	0.036277	0.16231	0.465971	None
7544	LIPA	-0.34852	0.162406	0.466167	None
7545	MEF2A	0.272771	0.162421	0.466167	None
7546	ZRANB1	-0.18864	0.162452	0.466193	None
7547	HLA-DRA	0.293754	0.162479	0.466211	None
7548	LEKR1	-0.09428	0.162624	0.466562	None
7549	TSHZ3	-0.26563	0.162669	0.466628	None
7550	C3orf17	-0.08582	0.162713	0.466628	None
7551	PTTG2	0.054927	0.162713	0.466628	None
7552	PLRG1	-0.14608	0.162735	0.466628	None
7553	IPO4	-0.24979	0.162754	0.466628	None
7554	LOC10272	0.068168	0.162791	0.466671	None
7555	RHOBTB2	-0.0657	0.162849	0.466731	None
7556	UFD1L	-0.16996	0.162855	0.466731	None
7557	DNAJC1	-0.20792	0.162922	0.466862	None
7558	TRIM22	-0.24282	0.163006	0.467016	None
7559	F2RL1	-0.20679	0.163019	0.467016	None
7560	CIDEC	-0.11329	0.163159	0.467355	None
7561	FAM3B	-0.21555	0.163256	0.467573	None
7562	JADE1	-0.15776	0.163406	0.467939	None
7563	GPNMB	-0.75502	0.163456	0.468022	None
7564	CYP2A7	-0.03557	0.16357	0.468231	None
7565	DMBT1	0.053687	0.163573	0.468231	None
7566	LINC01118	-0.07828	0.163753	0.468686	None
7567	RUVBL2	-0.26686	0.163791	0.468733	None
7568	CD38	0.38699	0.163815	0.468738	None
7569	FBXO43	0.333922	0.163851	0.46878	None
7570	MRPL38	-0.14486	0.163895	0.468842	None
7571	FGD5-AS1	0.084814	0.163953	0.468937	None
7572	PIGQ	-0.07134	0.163971	0.468937	None
7573	ZNF865	-0.05757	0.164012	0.468955	None
7574	HERPUD2	-0.30822	0.164023	0.468955	None
7575	SNRPB	0.219139	0.164056	0.468955	None
7576	HDDC2	-0.1885	0.164111	0.468955	None
7577	C16orf54	0.370132	0.164133	0.468955	None
7578	PDLIM7	-0.01306	0.164138	0.468955	None
7579	LINC01111	0.011061	0.164158	0.468955	None
7580	LOC10019	0.271829	0.164162	0.468955	None
7581	LOC10050	0.017324	0.164173	0.468955	None
7582	CCDC94	-0.24572	0.164194	0.468955	None
7583	ZNF44	-0.109	0.16432	0.469216	None
7584	CTD-2561	-0.19382	0.164329	0.469216	None
7585	ZNF587	0.141822	0.164395	0.469345	None
7586	NPC2	-0.26863	0.164467	0.469488	None
7587	NEO1	-0.17512	0.164539	0.469631	None
7588	ZNF184	0.257933	0.164605	0.469758	None
7589	LINC00536	0.010634	0.164688	0.469934	None
7590	ZNF337	0.221127	0.164722	0.469969	None
7591	SELM	-0.23569	0.164762	0.470021	None
7592	NT5C3B	-0.24559	0.164814	0.470106	None
7593	PSMD3	-0.20947	0.164888	0.470257	None
7594	SOWAHC	-0.35875	0.164925	0.470275	None
7595	MRFAP1L1	-0.21044	0.164938	0.470275	None
7596	SYNDIG1	-0.25287	0.164969	0.470301	None

7597	SMIM22	0.019167	0.165101	0.470615	None
7598	TMEM200/	0.691717	0.165178	0.470639	None
7599	FAM178A	-0.14628	0.165192	0.470639	None
7600	TUSC1	0.373698	0.165199	0.470639	None
7601	E2F2	0.160297	0.165205	0.470639	None
7602	GALK1	0.049794	0.165218	0.470639	None
7603	PLCD1	0.185808	0.165255	0.470683	None
7604	MFSD4	-0.04045	0.165293	0.470727	None
7605	PTAFR	-0.2973	0.165339	0.470758	None
7606	TDRD6	-0.25972	0.165347	0.470758	None
7607	RBM10	-0.17548	0.165465	0.471033	None
7608	CEACAM2:	-0.14705	0.165501	0.471072	None
7609	ZNF205-A:	0.00702	0.165532	0.471099	None
7610	PPP1R9A	-0.35676	0.165837	0.471884	None
7611	NFIA	-0.4512	0.165851	0.471884	None
7612	LINC00665	-0.06368	0.165918	0.472012	None
7613	TRDMT1	0.176145	0.166051	0.472321	None
7614	KIAA0247	-0.17565	0.16607	0.472321	None
7615	SHKBP1	-0.14745	0.166126	0.472401	None
7616	GLRA2	0.024805	0.166142	0.472401	None
7617	SUV39H2	0.147369	0.166167	0.472411	None
7618	ZSCAN25	0.109115	0.166229	0.472524	None
7619	MRPS12	-0.14065	0.166521	0.473291	None
7620	SPDEF	0.031479	0.166614	0.473495	None
7621	RP11-263k	0.083149	0.166704	0.473682	None
7622	GRM3	0.016627	0.166724	0.473682	None
7623	ZBTB24	0.171544	0.166763	0.473732	None
7624	LOC10192	-0.32135	0.166817	0.473805	None
7625	METRN	-0.08764	0.166847	0.473805	None
7626	CERCAM	-0.04131	0.166855	0.473805	None
7627	CKMT2-AS	-0.19856	0.166922	0.473926	None
7628	TCF7L1	-0.19307	0.166947	0.473926	None
7629	RP4-680D!	-0.03981	0.166963	0.473926	None
7630	TPT1-AS1	-0.209	0.166996	0.473959	None
7631	DEF6	-0.1718	0.167031	0.473978	None
7632	ARHGAP4	-0.2926	0.167068	0.473978	None
7633	FAM111A	0.103187	0.167098	0.473978	None
7634	ZNF804A	-0.37489	0.167101	0.473978	None
7635	NSUN6	-0.26682	0.167113	0.473978	None
7636	SPRY4	0.018843	0.167194	0.474088	None
7637	SLC4A1	-0.49828	0.167195	0.474088	None
7638	HORMAD2	0.014102	0.167231	0.474127	None
7639	INTU	-0.36202	0.167347	0.474374	None
7640	CTDP1	-0.25651	0.167377	0.474374	None
7641	PCSK7	-0.15904	0.167384	0.474374	None
7642	RP11-288H	0.077531	0.167447	0.47449	None
7643	LOC44014!	-0.13845	0.167508	0.474547	None
7644	RP11-305K	-0.23068	0.167526	0.474547	None
7645	FAT2	0.033411	0.167548	0.474547	None
7646	ITGA2B	-0.53515	0.167554	0.474547	None
7647	SLC30A6	0.213587	0.167597	0.474573	None
7648	PITPNA	-0.19266	0.167616	0.474573	None
7649	C12orf74	0.015777	0.167647	0.474573	None
7650	KIF13A	-0.19121	0.16767	0.474573	None
7651	DTNB	-0.03734	0.167687	0.474573	None
7652	LOC28401	0.020211	0.167725	0.474573	None
7653	NAA15	-0.17039	0.167735	0.474573	None
7654	TBC1D2B	-0.32087	0.167739	0.474573	None

7655	RNASE6	0.578699	0.167789	0.474654	None
7656	HMGN5	0.201984	0.16789	0.474821	None
7657	CCDC59	-0.16712	0.167892	0.474821	None
7658	H1F0	-0.3518	0.167958	0.474945	None
7659	PLA2G6	-0.15375	0.167993	0.474983	None
7660	ME3	0.155852	0.168117	0.475153	None
7661	CTD-2269I	0.034207	0.168125	0.475153	None
7662	JAM3	0.04106	0.168132	0.475153	None
7663	NANP	-0.26322	0.168141	0.475153	None
7664	RP11-182L	0.303427	0.168222	0.475319	None
7665	LYRM7	-0.22917	0.168311	0.475476	None
7666	ATF7IP	-0.15247	0.168337	0.475476	None
7667	PSMB4	-0.1941	0.168343	0.475476	None
7668	TMPRSS11	0.017245	0.168376	0.475506	None
7669	DUSP28	-0.15356	0.168399	0.475511	None
7670	NQO1	-0.35194	0.168476	0.475665	None
7671	TM9SF4	-0.18605	0.168764	0.476417	None
7672	PAFAH1B1	-0.19291	0.168842	0.476575	None
7673	CYB561D2	-0.12474	0.168941	0.47679	None
7674	WNT16	0.007246	0.169016	0.476941	None
7675	ANKFY1	-0.06512	0.169089	0.477083	None
7676	RP3-522P1	0.110054	0.169201	0.477337	None
7677	ZBTB33	-0.0781	0.169231	0.477361	None
7678	TSNAX	-0.16341	0.16933	0.477578	None
7679	ZNF444	-0.09926	0.169542	0.478113	None
7680	UPK3B	0.031482	0.169734	0.478593	None
7681	LOC10192	-0.27601	0.169801	0.478688	None
7682	SRRT	-0.18355	0.169823	0.478688	None
7683	CLGN	0.439863	0.169834	0.478688	None
7684	CTD-3025I	-0.2425	0.169922	0.478874	None
7685	GAS6-AS1	-0.3484	0.169964	0.478928	None
7686	CEP350	0.196452	0.170022	0.479032	None
7687	TEP1	-0.21654	0.170052	0.479054	None
7688	NGFR	0.029778	0.170078	0.479065	None
7689	EML3	-0.13538	0.170142	0.479183	None
7690	CARS2	-0.0736	0.170183	0.479235	None
7691	FANCE	-0.20319	0.170272	0.479424	None
7692	RP11-644F	-0.09329	0.170399	0.479717	None
7693	ACADVL	-0.1899	0.170477	0.479782	None
7694	CACNA1H	0.038469	0.17048	0.479782	None
7695	POMP	0.223589	0.170488	0.479782	None
7696	TICRR	-0.16702	0.170521	0.479812	None
7697	OBP2B	0.01588	0.170568	0.47984	None
7698	HSDL1	-0.23076	0.170575	0.47984	None
7699	DDX24	-0.15948	0.170771	0.480328	None
7700	MYOM1	-0.20774	0.170829	0.480428	None
7701	ZNF559	-0.24024	0.170894	0.48054	None
7702	ZNF460	0.030633	0.170913	0.48054	None
7703	HOXB9	-0.02379	0.170964	0.480598	None
7704	MRPL35	-0.2832	0.170978	0.480598	None
7705	GFER	-0.10002	0.171009	0.480622	None
7706	ZFP37	-0.09712	0.171075	0.480746	None
7707	RPS6KA3	0.161388	0.171206	0.481049	None
7708	TSPAN12	-0.049	0.171227	0.481049	None
7709	QTRT1	-0.29145	0.17125	0.481049	None
7710	MESP2	0.030145	0.17131	0.481157	None
7711	RAB33B	0.263531	0.171341	0.481182	None
7712	ADIRF-AS1	0.028244	0.171372	0.481207	None

7713	ZNF529	-0.2378	0.171443	0.481343	None
7714	LOC44112	-0.19393	0.171506	0.481454	None
7715	LINC0026C	-0.15356	0.171527	0.481454	None
7716	LINC00476	0.237824	0.171617	0.481638	None
7717	STT3B	0.276379	0.171637	0.481638	None
7718	XKRX	0.010906	0.171688	0.48172	None
7719	KCNIP1	0.00249	0.171777	0.481869	None
7720	SPAG1	0.481502	0.171799	0.481869	None
7721	XRCC5	0.151862	0.171808	0.481869	None
7722	SLC25A14	0.16691	0.171936	0.482164	None
7723	LMLN	-0.13592	0.172117	0.482521	None
7724	TMEM192	-0.24644	0.172124	0.482521	None
7725	LUZP4	0.001996	0.17213	0.482521	None
7726	TMEM33	0.168147	0.172154	0.482526	None
7727	TNNT1	-0.38162	0.172248	0.482726	None
7728	GABRP	0.006766	0.172361	0.48296	None
7729	TXNL4A	0.207668	0.172378	0.48296	None
7730	CNBD1	0.037252	0.172418	0.48296	None
7731	XPNPEP1	-0.19993	0.17242	0.48296	None
7732	HP	0.579582	0.172449	0.482977	None
7733	TYSND1	-0.11393	0.172653	0.483479	None
7734	LYNX1	0.036083	0.172673	0.483479	None
7735	NTRK1	-0.1907	0.172724	0.48356	None
7736	IPCEF1	-0.37186	0.172802	0.483716	None
7737	ANKRD65	-0.06743	0.172939	0.483883	None
7738	DAGLB	0.326353	0.172955	0.483883	None
7739	LOC10050	-0.23158	0.172958	0.483883	None
7740	PHYHD1	-0.13115	0.172979	0.483883	None
7741	CYP2W1	0.064913	0.172989	0.483883	None
7742	PID1	0.077821	0.173017	0.483883	None
7743	NDUFAB1	-0.19273	0.173025	0.483883	None
7744	MYO7A	-0.21157	0.17304	0.483883	None
7745	CRISP1	0.006743	0.173097	0.483979	None
7746	LOC10192	0.023602	0.173194	0.484174	None
7747	DUSP5	-0.40586	0.173212	0.484174	None
7748	SEC61A2	0.173429	0.173269	0.484225	None
7749	HYPK	-0.08072	0.173274	0.484225	None
7750	HSF2	-0.26159	0.173358	0.484378	None
7751	HMGXB3	-0.19068	0.173377	0.484378	None
7752	RP11-533E	-0.22194	0.173396	0.484378	None
7753	DEPDC1B	0.340945	0.173575	0.484815	None
7754	AC005162.	0.060727	0.173674	0.485028	None
7755	PPIP5K2	0.271223	0.173721	0.485097	None
7756	FBXO22	-0.208	0.173816	0.485271	None
7757	CCDC97	-0.11462	0.17383	0.485271	None
7758	FOCAD	-0.14066	0.173861	0.485271	None
7759	PIEZO1	0.103588	0.173873	0.485271	None
7760	UBE2H	-0.18505	0.173921	0.485278	None
7761	GSTO1	-0.14568	0.173929	0.485278	None
7762	PRKD2	0.270741	0.173953	0.485278	None
7763	RAB15	0.213801	0.173965	0.485278	None
7764	DHRS3	-0.46634	0.174031	0.485399	None
7765	SH3PXD2A	0.013811	0.174082	0.485452	None
7766	MAML1	-0.13617	0.174095	0.485452	None
7767	LOC10192	0.072404	0.174271	0.485882	None
7768	ASAP1	-0.25324	0.174415	0.486145	None
7769	SRI	-0.14842	0.174423	0.486145	None
7770	DOT1L	-0.24393	0.174433	0.486145	None

7771	BC036209	0.021539	0.174504	0.48628	None
7772	LHX6	-0.10536	0.174534	0.486303	None
7773	SLC9A1	-0.15292	0.17456	0.486312	None
7774	HIST1H4A	-0.21352	0.174634	0.486456	None
7775	PTPN12	-0.31593	0.174729	0.486574	None
7776	IGKC	0.182083	0.174747	0.486574	None
7777	FBRS	-0.14066	0.174763	0.486574	None
7778	RER1	-0.18229	0.174767	0.486574	None
7779	UBTF	-0.26481	0.174824	0.486664	None
7780	CNGB3	0.060417	0.174844	0.486664	None
7781	U2AF1	-0.29778	0.17489	0.48673	None
7782	FAM110B	-0.12522	0.174934	0.48679	None
7783	ATP2B1	0.429904	0.175141	0.487302	None
7784	USP4	-0.12735	0.175199	0.487344	None
7785	TMEM52B	0.25225	0.17522	0.487344	None
7786	LINC00324	0.157393	0.175223	0.487344	None
7787	TAF10	0.078226	0.175259	0.487347	None
7788	ZNF777	-0.04237	0.175269	0.487347	None
7789	RPL38	-0.19148	0.175321	0.487386	None
7790	PTRHD1	-0.1958	0.175328	0.487386	None
7791	PKP3	0.026057	0.175361	0.487387	None
7792	SMKR1	-0.39343	0.175374	0.487387	None
7793	MYO5C	0.462808	0.175411	0.487428	None
7794	BAD	0.105371	0.175496	0.487561	None
7795	CCDC62	0.182754	0.175555	0.487561	None
7796	MKRN2	-0.28976	0.175564	0.487561	None
7797	COQ10A	0.225162	0.175566	0.487561	None
7798	LOC10050!	0.05396	0.175582	0.487561	None
7799	DLGAP5	0.439799	0.175594	0.487561	None
7800	SCRT1	0.025391	0.175622	0.487575	None
7801	CDK6	0.227952	0.175796	0.487932	None
7802	PLAC8	-0.26146	0.1758	0.487932	None
7803	EIF4B	-0.18978	0.17582	0.487932	None
7804	ID1	0.637482	0.17584	0.487932	None
7805	RHPN2	-0.26603	0.175892	0.487982	None
7806	SNORA68	-0.12112	0.175903	0.487982	None
7807	RPL39L	0.369574	0.175932	0.487991	None
7808	TSPAN13	0.248047	0.175999	0.487991	None
7809	PIAS2	-0.20834	0.176001	0.487991	None
7810	BD495725	0.377294	0.176015	0.487991	None
7811	GPKOW	-0.23426	0.176019	0.487991	None
7812	AMMECR1	-0.35073	0.176056	0.488031	None
7813	PPIL1	-0.26916	0.176192	0.488346	None
7814	AHDC1	0.039133	0.176264	0.488481	None
7815	MRPS18A	-0.20203	0.176337	0.488566	None
7816	CHSY3	-0.19514	0.176339	0.488566	None
7817	EAPP	0.180481	0.17642	0.488691	None
7818	SLC2A9	-0.09163	0.17643	0.488691	None
7819	AIP	-0.17953	0.176511	0.488853	None
7820	ZNF211	-0.20352	0.176562	0.488933	None
7821	LOC10192	0.1841	0.176693	0.489232	None
7822	TYW3	-0.18619	0.176733	0.489263	None
7823	ACAP2	0.229898	0.176749	0.489263	None
7824	NUDT18	-0.23881	0.176831	0.48939	None
7825	PSMD11	0.168113	0.17684	0.48939	None
7826	SLC35B4	-0.2094	0.176875	0.489424	None
7827	UBE2I	0.25356	0.176926	0.489502	None
7828	LOC49415	-0.19038	0.177156	0.490076	None

7829	TMEM62	0.106842	0.177209	0.490103	None
7830	ADSSL1	-0.10339	0.177211	0.490103	None
7831	RFTN2	0.027468	0.177265	0.490189	None
7832	HIVEP2	0.287647	0.177309	0.490225	None
7833	SLC22A16	0.248584	0.177323	0.490225	None
7834	USP10	-0.20132	0.177379	0.490316	None
7835	CTB-50L17	-0.12653	0.177437	0.490415	None
7836	INTS9	-0.13437	0.177482	0.490476	None
7837	LOC10050	0.326863	0.177655	0.490822	None
7838	C18orf25	0.1749	0.177658	0.490822	None
7839	FAM175A	-0.2511	0.177675	0.490822	None
7840	FKBP15	-0.08971	0.177707	0.490848	None
7841	LOC10050	0.003994	0.177854	0.491133	None
7842	HSD17B6	-0.12959	0.177856	0.491133	None
7843	CAMK2N2	0.020879	0.177944	0.491298	None
7844	POLD1	-0.26489	0.177961	0.491298	None
7845	VHL	-0.16931	0.178064	0.49152	None
7846	GFRA2	-0.33278	0.178141	0.491671	None
7847	CYB5B	0.158545	0.178386	0.492283	None
7848	TRNAU1AF	-0.14981	0.178628	0.492889	None
7849	OAZ1	-0.13764	0.178664	0.492894	None
7850	TMEM170A	-0.19888	0.178675	0.492894	None
7851	DOPEY2	-0.13648	0.178742	0.493014	None
7852	PEX14	-0.07519	0.178805	0.493082	None
7853	CYBA	0.181329	0.178814	0.493082	None
7854	CD2BP2	0.180475	0.178835	0.493082	None
7855	NCF2	-0.62738	0.178942	0.493315	None
7856	FAM220A	-0.17827	0.179028	0.493445	None
7857	RALB	-0.17032	0.179035	0.493445	None
7858	CMAS	-0.11845	0.179108	0.493585	None
7859	PHF19	-0.26312	0.179164	0.493675	None
7860	CIB2	-0.09401	0.179241	0.493806	None
7861	HNRNPA3I	0.042797	0.179257	0.493806	None
7862	ADAM32	-0.26069	0.179289	0.493824	None
7863	GPATCH8	-0.16467	0.179309	0.493824	None
7864	CEACAM6	0.618738	0.179357	0.493869	None
7865	COX17	-0.14265	0.179371	0.493869	None
7866	POLR1D	-0.17173	0.179408	0.493908	None
7867	BATF2	-0.15881	0.179531	0.4941	None
7868	DNAJC30	-0.16915	0.179553	0.4941	None
7869	ASPH	-0.2329	0.179564	0.4941	None
7870	CLPB	-0.14368	0.179569	0.4941	None
7871	TMTC1	-0.09182	0.17973	0.494439	None
7872	TMEM69	-0.22989	0.179759	0.494439	None
7873	KHDC3L	0.01368	0.179761	0.494439	None
7874	KCNA3	0.254379	0.179785	0.494442	None
7875	DIP2C	-0.22023	0.179834	0.494514	None
7876	MEX3A	-0.12577	0.179942	0.494749	None
7877	RP11-420K	-0.73735	0.180161	0.495176	None
7878	SMARCA1	-0.29401	0.180183	0.495176	None
7879	CLN6	0.076703	0.180187	0.495176	None
7880	B4GALT3	-0.09374	0.180189	0.495176	None
7881	FAM53B	0.085798	0.18035	0.495556	None
7882	DDHD1	0.205717	0.180374	0.495559	None
7883	SPAM1	0.002944	0.180426	0.495639	None
7884	TUBB3	0.249365	0.180514	0.495817	None
7885	SLC39A9	-0.03713	0.180576	0.495866	None
7886	LPAR6	-0.28251	0.180577	0.495866	None

7887	C16orf80	0.181617	0.180686	0.496074	None
7888	SDK2	-0.10082	0.180699	0.496074	None
7889	ARPC5	-0.204	0.180736	0.496115	None
7890	PEX10	-0.1206	0.180893	0.496482	None
7891	GAN	0.113247	0.180973	0.496639	None
7892	FAM169A	0.306984	0.181003	0.496658	None
7893	MTERF4	-0.11198	0.181056	0.496741	None
7894	NDUFAF2	-0.20885	0.181111	0.496746	None
7895	MCFD2	0.241738	0.181135	0.496746	None
7896	LOC10192[illegible]	0.032939	0.181139	0.496746	None
7897	TDRG1	0.118578	0.18115	0.496746	None
7898	ZBTB8OS	-0.18549	0.181187	0.496749	None
7899	IGSF8	-0.02825	0.181197	0.496749	None
7900	ETS1	0.26162	0.181315	0.497004	None
7901	BCL2L13	-0.16702	0.181336	0.497004	None
7902	GLRX	0.283654	0.181432	0.497173	None
7903	CNNM2	-0.09216	0.181497	0.497173	None
7904	LOC10192[illegible]	0.018098	0.181499	0.497173	None
7905	ANAPC2	-0.15532	0.181503	0.497173	None
7906	CHRM3-A[illegible]	-0.2753	0.181527	0.497173	None
7907	PCDHB14	-0.22461	0.181542	0.497173	None
7908	ARL6IP4	-0.19719	0.181558	0.497173	None
7909	SLC25A42	0.029276	0.181667	0.497408	None
7910	SPATC1	0.049508	0.181699	0.497431	None
7911	C14orf2	0.134039	0.181825	0.497714	None
7912	CACNB2	-0.2473	0.181976	0.498065	None
7913	FAM134A	-0.11704	0.182027	0.498076	None
7914	HERPUD1	0.206138	0.182073	0.498076	None
7915	KCNN1	0.050585	0.182089	0.498076	None
7916	SVIL	-0.20629	0.182113	0.498076	None
7917	IFT140	-0.12726	0.182113	0.498076	None
7918	PCSK1N	-0.0819	0.182128	0.498076	None
7919	GORASP1	-0.09375	0.182141	0.498076	None
7920	LOC40109[illegible]	0.017903	0.182189	0.498144	None
7921	HEMK1	-0.12526	0.182406	0.498675	None
7922	GPS1	-0.19521	0.182446	0.498699	None
7923	ATXN1	-0.23787	0.182485	0.498699	None
7924	PLAU	0.30919	0.182488	0.498699	None
7925	CCDC22	-0.18527	0.182519	0.498699	None
7926	P4HA1	-0.10123	0.182544	0.498699	None
7927	TRIM52	-0.271	0.182586	0.498699	None
7928	TMEM53	-0.13633	0.182602	0.498699	None
7929	GOLGA2P[illegible]	0.229632	0.182614	0.498699	None
7930	MSH4	0.00313	0.182622	0.498699	None
7931	ANKRD7	-0.02525	0.18265	0.498712	None
7932	RALGAPA[illegible]	-0.28311	0.182736	0.498884	None
7933	ST6GALNA[illegible]	-0.19069	0.182801	0.498999	None
7934	POLR2L	-0.24597	0.182882	0.499142	None
7935	LINC0095[illegible]	0.3234	0.1829	0.499142	None
7936	FOXP3	0.031792	0.183065	0.499474	None
7937	MAP3K5	-0.15981	0.183067	0.499474	None
7938	CLVS2	0.023993	0.183116	0.499544	None
7939	BCCIP	-0.15529	0.183191	0.499626	None
7940	CASP6	-0.14558	0.183192	0.499626	None
7941	SYBU	-0.26063	0.183306	0.499861	None
7942	ITGA6	-0.33256	0.183346	0.499861	None
7943	RBM11	-0.25601	0.183354	0.499861	None
7944	PPP2R2B	0.112092	0.183371	0.499861	None

7945	ATAD2	0.237054	0.183455	0.500026	None
7946	CENPN	0.231829	0.183514	0.500125	None
7947	RP11-769(	0.291432	0.18357	0.500216	None
7948	ALG1	-0.12483	0.183662	0.500375	None
7949	ARRDC4	-0.37177	0.183683	0.500375	None
7950	MAL2	0.018221	0.183706	0.500375	None
7951	NCOA3	-0.27304	0.183725	0.500375	None
7952	ADCY9	-0.07488	0.183765	0.500375	None
7953	EGLN1	-0.21005	0.183767	0.500375	None
7954	LOC10192!	0.019417	0.183809	0.500375	None
7955	WBP2NL	-0.1134	0.183814	0.500375	None
7956	FLJ16779	0.014709	0.183893	0.500529	None
7957	PRKX	0.24147	0.18392	0.500538	None
7958	TRPM7	-0.14847	0.184007	0.500594	None
7959	HNMT	-0.28605	0.184009	0.500594	None
7960	ANKRD16	-0.18842	0.18401	0.500594	None
7961	RHOXF1	-0.13443	0.184123	0.500768	None
7962	C2	-0.3063	0.184151	0.500768	None
7963	PP13	-0.06993	0.184158	0.500768	None
7964	TAL1	-0.26682	0.184166	0.500768	None
7965	NOA1	-0.2669	0.184243	0.500785	None
7966	STEAP2	-0.281	0.18426	0.500785	None
7967	RP11-1275	-0.23507	0.184284	0.500785	None
7968	NEUROG3	-0.0227	0.184304	0.500785	None
7969	RP11-862L	-0.03821	0.184308	0.500785	None
7970	MOSPD3	-0.14655	0.184311	0.500785	None
7971	RECK	0.171602	0.184369	0.50088	None
7972	NMI	-0.13803	0.184451	0.501029	None
7973	FAM131B	-0.13848	0.18447	0.501029	None
7974	AGTR2	0.00215	0.184581	0.501267	None
7975	MTCL1	-0.45129	0.184647	0.501346	None
7976	CREBL2	-0.32985	0.184657	0.501346	None
7977	TTC16	-0.02907	0.184687	0.501367	None
7978	RFFL	-0.07502	0.184765	0.501475	None
7979	NECAP1	0.185873	0.184773	0.501475	None
7980	ELMO3	-0.04155	0.184899	0.501696	None
7981	TFAM	-0.34082	0.184901	0.501696	None
7982	WDR76	0.226549	0.184976	0.501837	None
7983	FAM167B	-0.19604	0.185058	0.501995	None
7984	TMEM132I	0.016874	0.185093	0.502029	None
7985	FLG2	0.04257	0.185141	0.502095	None
7986	IFFO2	-0.23679	0.185234	0.502285	None
7987	TTC37	0.335477	0.185276	0.502329	None
7988	PIBF1	-0.22591	0.185343	0.502329	None
7989	ANKRD13(	0.225703	0.185343	0.502329	None
7990	TECR	-0.10851	0.185343	0.502329	None
7991	SLC16A10	-0.35301	0.185369	0.502336	None
7992	LOC10192!	-0.14253	0.185491	0.502542	None
7993	IGLV1-44	-0.3369	0.185491	0.502542	None
7994	USP3	0.177915	0.185536	0.502554	None
7995	TAF12	-0.32192	0.185542	0.502554	None
7996	SSMEM1	0.008465	0.185608	0.50262	None
7997	ALKBH1	-0.12731	0.185614	0.50262	None
7998	OSTCP1	0.024305	0.185636	0.50262	None
7999	LIPC	-0.12964	0.185666	0.502637	None
8000	SLC35F1	0.003264	0.18571	0.50266	None
8001	LOC10050	0.080601	0.185724	0.50266	None
8002	AC006538.	-0.11762	0.185744	0.50266	None

8003	TREML1	-0.59827	0.185788	0.502717	None
8004	KLHL13	0.425589	0.185815	0.502726	None
8005	SDCBP	-0.21322	0.185845	0.502745	None
8006	NCAPG	0.335454	0.185961	0.502963	None
8007	SEC14L1	-0.3004	0.185972	0.502963	None
8008	SCGB3A1	-0.14604	0.18601	0.503002	None
8009	LY9	0.161859	0.186174	0.503275	None
8010	LOC10192!	-0.17329	0.186188	0.503275	None
8011	LCMT2	-0.16339	0.1862	0.503275	None
8012	DYRK1B	-0.04422	0.186203	0.503275	None
8013	LOC10192	0.021784	0.186261	0.503316	None
8014	RNF186	0.013045	0.186265	0.503316	None
8015	GAREML	-0.10302	0.186365	0.503521	None
8016	RRP9	-0.2381	0.186508	0.503846	None
8017	RALGAPA1	0.158181	0.186543	0.503877	None
8018	EIF2B3	-0.24834	0.186633	0.504026	None
8019	SH3YL1	-0.10331	0.186644	0.504026	None
8020	PCDH18	0.147726	0.186697	0.504105	None
8021	LRPAP1	-0.2107	0.18681	0.504303	None
8022	TM2D2	-0.19077	0.186832	0.504303	None
8023	MSLN	0.041376	0.18684	0.504303	None
8024	WDR59	-0.12399	0.186977	0.504558	None
8025	SLC16A4	-0.15083	0.187006	0.504558	None
8026	RXRB	-0.06887	0.187008	0.504558	None
8027	EMD	-0.20238	0.187045	0.504558	None
8028	ZNF778	-0.06562	0.187065	0.504558	None
8029	TTLL9	0.014775	0.187074	0.504558	None
8030	C5orf24	0.27829	0.187188	0.504802	None
8031	DNAJC12	-0.41785	0.187219	0.504822	None
8032	RPS10P7	-0.31372	0.187327	0.505051	None
8033	COX14	-0.17832	0.187393	0.505167	None
8034	GALNT16	-0.02521	0.187488	0.505359	None
8035	ARPC1B	-0.26076	0.187574	0.505483	None
8036	VPS33A	-0.11201	0.187588	0.505483	None
8037	RNF14	-0.30443	0.187604	0.505483	None
8038	ZNF267	0.312065	0.187727	0.505751	None
8039	SYNRG	-0.15184	0.187806	0.505901	None
8040	CATSPERB	0.011608	0.187925	0.506159	None
8041	MFN1	-0.168	0.188149	0.5067	None
8042	AP000462.	0.017039	0.188209	0.506799	None
8043	GTPBP6	-0.16541	0.188302	0.506984	None
8044	RBP1	-0.12145	0.1884	0.507184	None
8045	BSG	-0.30439	0.188423	0.507184	None
8046	LRRC52	0.006969	0.188495	0.507315	None
8047	LRRC40	0.158623	0.18856	0.507407	None
8048	MXRA5	0.213107	0.18859	0.507407	None
8049	PDZRN4	-0.50145	0.188618	0.507407	None
8050	SHE	-0.32096	0.188623	0.507407	None
8051	BTBD7	-0.2362	0.188681	0.507484	None
8052	EFCC1	-0.13191	0.188719	0.507484	None
8053	ZNF791	0.232675	0.188722	0.507484	None
8054	LOC10050	0.03567	0.188855	0.507756	None
8055	ZBTB46-AS	0.015762	0.188871	0.507756	None
8056	RNF126P1	-0.10441	0.188893	0.507756	None
8057	LPAR4	-0.36332	0.189014	0.508018	None
8058	CPEB1	-0.09593	0.189136	0.508139	None
8059	CHRM1	0.010715	0.189141	0.508139	None
8060	EDEM3	-0.2961	0.189156	0.508139	None

8061	PLVAP	-0.07725	0.189181	0.508139	None
8062	UXS1	0.152826	0.189226	0.508139	None
8063	ABCA5	-0.19772	0.189255	0.508139	None
8064	PGLYRP2	-0.06565	0.189256	0.508139	None
8065	RTCA	-0.10043	0.18926	0.508139	None
8066	PIGS	-0.15533	0.189291	0.508139	None
8067	LOC28616:	0.2019	0.189298	0.508139	None
8068	DDO	-0.19308	0.189367	0.508139	None
8069	STOX1	-0.15109	0.189369	0.508139	None
8070	METTL15	0.013946	0.189376	0.508139	None
8071	LOC37489(	0.045417	0.189388	0.508139	None
8072	TNNI2	-0.24099	0.189468	0.508232	None
8073	TANGO6	-0.15004	0.189485	0.508232	None
8074	MTG1	-0.23086	0.189493	0.508232	None
8075	RAPGEF1	-0.10923	0.189648	0.508575	None
8076	C11orf16	0.101162	0.189667	0.508575	None
8077	SYPL1	0.184993	0.189748	0.508714	None
8078	DDX11-AS	-0.047	0.189775	0.508714	None
8079	PON2	-0.27724	0.18979	0.508714	None
8080	LMOD1	0.032218	0.18988	0.508894	None
8081	ZNF576	-0.10623	0.189981	0.509099	None
8082	RP11-97C:	-0.26405	0.190032	0.509168	None
8083	NEDD4L	-0.30393	0.190053	0.509168	None
8084	SYS1	-0.1685	0.190185	0.509377	None
8085	RP1-199J3	-0.06851	0.19019	0.509377	None
8086	MDFI	-0.04214	0.190202	0.509377	None
8087	IGJ	0.80354	0.190277	0.509475	None
8088	GRIK5	0.052625	0.190285	0.509475	None
8089	CR1	-0.31503	0.190323	0.509512	None
8090	LCAT	-0.03012	0.190432	0.509654	None
8091	PXDC1	-0.27766	0.190445	0.509654	None
8092	ZSCAN29	-0.17665	0.19048	0.509654	None
8093	HSPC081	0.060138	0.190515	0.509654	None
8094	EPRS	-0.17287	0.190515	0.509654	None
8095	AHSP	-0.82796	0.190533	0.509654	None
8096	MIEN1	-0.18395	0.190541	0.509654	None
8097	ICA1	-0.08378	0.190594	0.509722	None
8098	RAB40B	0.143427	0.190639	0.509722	None
8099	SLFNL1	-0.08319	0.190647	0.509722	None
8100	RP5-1085F	0.134289	0.19066	0.509722	None
8101	LOC10050'	-0.06711	0.190794	0.509993	None
8102	C1orf226	-0.15038	0.190809	0.509993	None
8103	RPL32	-0.08547	0.190854	0.510004	None
8104	RP3-507I1!	-0.01414	0.19086	0.510004	None
8105	TRDV3	-0.12475	0.190888	0.510015	None
8106	GPATCH2L	-0.16434	0.190983	0.510105	None
8107	FAM49B	-0.20636	0.190986	0.510105	None
8108	ADAMTS1	0.2402	0.190992	0.510105	None
8109	CLK1	0.271238	0.191086	0.510254	None
8110	CHD6	0.123992	0.191109	0.510254	None
8111	CCL4	0.844025	0.191129	0.510254	None
8112	HIST1H1B	0.441787	0.191142	0.510254	None
8113	GRIPAP1	-0.1911	0.191202	0.510317	None
8114	ACAA2	-0.20533	0.191213	0.510317	None
8115	DEPDC5	-0.27819	0.191262	0.510384	None
8116	CHRNB1	-0.11847	0.191316	0.510467	None
8117	WIPF3	-0.12369	0.191391	0.510467	None
8118	LPPR2	-0.05488	0.191398	0.510467	None

8119	LNX1	-0.27701	0.191417	0.510467	None
8120	RP11-333(	0.013735	0.19143	0.510467	None
8121	CDSN	0.0146	0.191434	0.510467	None
8122	ZDHHC17	0.098949	0.191476	0.510515	None
8123	KLF4	0.405826	0.191634	0.510875	None
8124	SLC7A11	-0.18247	0.191669	0.510892	None
8125	TUBB	0.246151	0.191688	0.510892	None
8126	KLF9	-0.11384	0.191721	0.510919	None
8127	RAB31	-0.57486	0.191783	0.511021	None
8128	LGI4	0.040508	0.191874	0.511199	None
8129	TMEM31	0.025608	0.192026	0.511541	None
8130	MPRIP	0.110826	0.192155	0.511796	None
8131	ARL2	-0.2357	0.192169	0.511796	None
8132	IGLL3P	0.41547	0.192324	0.512084	None
8133	NOP2	-0.16818	0.192324	0.512084	None
8134	RP11-138/	0.440079	0.192354	0.512101	None
8135	SENP3	-0.11794	0.192413	0.512196	None
8136	IL36RN	0.058165	0.192497	0.512354	None
8137	NBEAL1	-0.1076	0.192623	0.512627	None
8138	LINC01093	0.090547	0.192723	0.512814	None
8139	ZNF195	-0.19057	0.192741	0.512814	None
8140	DDX26B	-0.20247	0.192789	0.51288	None
8141	PROSER3	-0.0559	0.192837	0.512902	None
8142	BC032415	-0.10587	0.192845	0.512902	None
8143	BEX5	0.617981	0.192878	0.512916	None
8144	MUC8	0.055858	0.192897	0.512916	None
8145	PSMC6	-0.12521	0.192944	0.512963	None
8146	FBXW12	0.09354	0.192995	0.512963	None
8147	MCTS1	-0.31679	0.193013	0.512963	None
8148	CANX	0.127953	0.193027	0.512963	None
8149	LCOR	0.190634	0.193033	0.512963	None
8150	LINC00638	-0.09263	0.193148	0.513206	None
8151	AMDHD1	-0.15952	0.193188	0.513249	None
8152	TSPYL4	0.205197	0.193421	0.513683	None
8153	SMARCAD	-0.14965	0.193425	0.513683	None
8154	FAM228B	0.152843	0.193448	0.513683	None
8155	ACSM3	0.403447	0.193462	0.513683	None
8156	MNS1	0.266786	0.19347	0.513683	None
8157	MAPK8IP2	-0.02912	0.193574	0.51382	None
8158	FLG	0.013621	0.193575	0.51382	None
8159	GBF1	-0.20718	0.193593	0.51382	None
8160	ZNF233	-0.0672	0.193619	0.513826	None
8161	ETV4	-0.04033	0.193661	0.513874	None
8162	MPZL1	0.130074	0.193774	0.514068	None
8163	GPR55	-0.028	0.193781	0.514068	None
8164	ZNF234	-0.10046	0.193809	0.514079	None
8165	PRR16	-0.31041	0.19385	0.514125	None
8166	MAK	-0.13536	0.193936	0.514289	None
8167	SLC39A5	0.037562	0.193971	0.51432	None
8168	SCN2B	0.009944	0.194064	0.514484	None
8169	FAR1	-0.20977	0.194081	0.514484	None
8170	ARHGEF7	0.238131	0.194198	0.514733	None
8171	KCTD20	-0.283	0.19427	0.514783	None
8172	RP11-342L	-0.22026	0.19428	0.514783	None
8173	ATM	-0.21611	0.194291	0.514783	None
8174	PLCL1	-0.16988	0.194312	0.514783	None
8175	LOC10192!	0.001816	0.194403	0.514959	None
8176	KLK15	0.030838	0.194655	0.515551	None

8177	ARGLU1	0.215684	0.194674	0.515551	None
8178	MINOS1P1	-0.37532	0.194713	0.515591	None
8179	ELMOD3	-0.09771	0.194783	0.515715	None
8180	ZNF182	-0.15855	0.194822	0.515723	None
8181	RAD51C	-0.20107	0.194834	0.515723	None
8182	ANKRD35	-0.35405	0.194882	0.515786	None
8183	LOC10192ǂ	-0.00825	0.194942	0.515882	None
8184	CAPN10-A	0.062985	0.194992	0.515953	None
8185	TBC1D20	-0.07992	0.195086	0.516139	None
8186	RP11-846E	-0.32068	0.195133	0.516199	None
8187	FLJ20021	-0.17471	0.195165	0.51622	None
8188	WTIP	-0.04799	0.19529	0.516483	None
8189	KIF9	-0.12097	0.195312	0.516483	None
8190	OXT	-0.06605	0.195387	0.516619	None
8191	CLDN10	0.568063	0.195523	0.516915	None
8192	ZSWIM6	0.220294	0.195583	0.517011	None
8193	TGM3	0.032504	0.195803	0.51753	None
8194	TMEM255E	-0.09439	0.19587	0.517643	None
8195	KIF23	0.2397	0.195902	0.517665	None
8196	LUZP1	-0.19315	0.196011	0.517889	None
8197	CLASP1	0.204433	0.196068	0.517975	None
8198	LOC10050	-0.33993	0.196102	0.518004	None
8199	SALL3	0.014069	0.196147	0.518058	None
8200	RRP1B	-0.11419	0.196221	0.518172	None
8201	WT1-AS	-0.25875	0.196238	0.518172	None
8202	LOC10050(	0.03131	0.196311	0.518302	None
8203	RP11-554J	-0.23052	0.196402	0.518478	None
8204	IPO11	0.035659	0.196513	0.518697	None
8205	LYPD5	-0.03102	0.196532	0.518697	None
8206	USP33	0.179009	0.196622	0.51887	None
8207	ZPR1	-0.17359	0.19665	0.518878	None
8208	FIGN	-0.59467	0.196689	0.518878	None
8209	RAB11FIP1	-0.21021	0.196697	0.518878	None
8210	PKM	-0.18798	0.196744	0.518921	None
8211	WNT5B	-0.05032	0.196783	0.518921	None
8212	ADO	0.110908	0.196797	0.518921	None
8213	LDLRAD4-.	0.00868	0.196809	0.518921	None
8214	THEMIS2	0.352801	0.196865	0.518926	None
8215	LOC10050(	-0.11923	0.196884	0.518926	None
8216	EXO5	-0.12561	0.196886	0.518926	None
8217	PRPF6	-0.19428	0.196907	0.518926	None
8218	LYPLA1	0.197745	0.196967	0.519021	None
8219	RHD	-0.49522	0.197006	0.51906	None
8220	9-Sep	0.233514	0.197169	0.519426	None
8221	INPP5A	-0.09784	0.1972	0.519426	None
8222	SLC39A11	-0.22796	0.197216	0.519426	None
8223	AHSA1	-0.20187	0.197431	0.519927	None
8224	EFCAB4B	0.109667	0.197534	0.520114	None
8225	ERICH6	0.001797	0.197563	0.520114	None
8226	NAT8B	-0.1593	0.197574	0.520114	None
8227	LINC00326	0.035912	0.197642	0.520231	None
8228	KLC2	-0.11099	0.197769	0.520501	None
8229	BPNT1	-0.18001	0.19799	0.521021	None
8230	PALD1	0.343257	0.198046	0.521103	None
8231	COG6	-0.09068	0.198141	0.52129	None
8232	SH3GL2	-0.32191	0.198285	0.521606	None
8233	SPTLC1	0.185646	0.19837	0.521727	None
8234	LOC22127:	-0.10292	0.198379	0.521727	None

8235	TANK	-0.23296	0.198448	0.521845	None
8236	FCGBP	-0.38909	0.198478	0.52186	None
8237	RP11-846E	-0.27977	0.198519	0.521905	None
8238	GET4	-0.14028	0.198552	0.521929	None
8239	ZNF546	-0.12539	0.19861	0.522018	None
8240	CCDC77	-0.22831	0.19881	0.522479	None
8241	TRA2B	-0.22236	0.199074	0.52311	None
8242	NPHP3-AS	0.002997	0.199241	0.523392	None
8243	C11orf45	-0.28489	0.199247	0.523392	None
8244	IL33	-0.51636	0.199254	0.523392	None
8245	FAM65A	-0.23594	0.199363	0.523586	None
8246	P2RY6	-0.1602	0.199376	0.523586	None
8247	COQ10B	-0.11587	0.199459	0.52368	None
8248	OSBPL3	-0.19426	0.19946	0.52368	None
8249	CD37	-0.22485	0.199573	0.523829	None
8250	EMP2	-0.1967	0.199585	0.523829	None
8251	ST6GALNA	-0.1341	0.199591	0.523829	None
8252	MTAP	-0.22194	0.199614	0.523829	None
8253	LOC72782(	-0.16902	0.199707	0.52401	None
8254	AX748267	-0.17516	0.199782	0.524142	None
8255	C9orf66	-0.12357	0.199806	0.524142	None
8256	TMEM245	-0.17178	0.199834	0.524153	None
8257	PPIA	0.07262	0.199896	0.524251	None
8258	COL8A2	-0.22297	0.19996	0.524355	None
8259	RNH1	-0.12376	0.200006	0.524414	None
8260	LOC10050(	-0.10586	0.200063	0.524477	None
8261	NLE1	-0.25837	0.200084	0.524477	None
8262	MSANTD3	-0.19811	0.200103	0.524477	None
8263	SHMT1	0.04166	0.200163	0.524496	None
8264	SYT1	-0.3084	0.200172	0.524496	None
8265	ATF5	-0.08742	0.200183	0.524496	None
8266	ZFP36L1	-0.13179	0.200266	0.524651	None
8267	SHD	-0.26084	0.200327	0.524746	None
8268	RP11-250E	-0.07623	0.200467	0.525049	None
8269	RP11-60A2	0.058357	0.200535	0.525164	None
8270	VWA3A	0.067107	0.20059	0.525246	None
8271	INSR	0.220951	0.200677	0.525371	None
8272	CXCR2	-0.45397	0.200694	0.525371	None
8273	RP11-464F	-0.07882	0.200711	0.525371	None
8274	DDX28	0.105367	0.200784	0.5255	None
8275	LINC00869	-0.11368	0.200912	0.525771	None
8276	FGD3	-0.11468	0.200936	0.525771	None
8277	PLK2	0.448803	0.201013	0.525908	None
8278	RP11-456H	-0.03774	0.201107	0.525969	None
8279	FKBP5	-0.26843	0.201133	0.525969	None
8280	EEF1A2	0.060665	0.201179	0.525969	None
8281	OMA1	-0.29789	0.201196	0.525969	None
8282	DUSP10	-0.3647	0.201224	0.525969	None
8283	LIMK1	0.023979	0.201228	0.525969	None
8284	ARL10	0.053632	0.201239	0.525969	None
8285	LNP1	-0.19441	0.201254	0.525969	None
8286	GABRR2	0.066974	0.201255	0.525969	None
8287	XCR1	0.006888	0.201329	0.526098	None
8288	DAG1	-0.18121	0.201415	0.526225	None
8289	LRRC23	-0.19693	0.201435	0.526225	None
8290	SDK1	-0.07596	0.201468	0.526225	None
8291	AL133493.1	-0.70263	0.201475	0.526225	None
8292	PPP1R18	-0.3327	0.201591	0.526305	None

8293	SLC38A10	-0.09381	0.201601	0.526305	None
8294	LINC00664	0.062442	0.20161	0.526305	None
8295	SIGLEC5	-0.20299	0.201619	0.526305	None
8296	RHOBTB3	-0.19677	0.201627	0.526305	None
8297	CCDC142	-0.12934	0.201694	0.526323	None
8298	LOC10063(	-0.23243	0.201697	0.526323	None
8299	LOC10192	-0.25406	0.201744	0.526323	None
8300	PTGER1	0.019562	0.201754	0.526323	None
8301	EDF1	-0.14718	0.201775	0.526323	None
8302	GPR1-AS	-0.19044	0.20178	0.526323	None
8303	LOC10192	-0.21794	0.201871	0.526499	None
8304	FAM155B	-0.0896	0.201993	0.526753	None
8305	ADAT2	-0.17372	0.202025	0.526773	None
8306	SMAD9	-0.17205	0.202078	0.52681	None
8307	PLA2G7	-0.83117	0.202131	0.52681	None
8308	CDK2	-0.18466	0.202136	0.52681	None
8309	KCNJ8	-0.26791	0.202137	0.52681	None
8310	RAPGEF4	-0.26984	0.202178	0.52683	None
8311	CLPX	-0.11829	0.202213	0.52683	None
8312	BRD9	0.165912	0.202217	0.52683	None
8313	ADIRF	0.062413	0.202268	0.526858	None
8314	GABBR1	-0.2104	0.20229	0.526858	None
8315	BAALC	0.49569	0.202301	0.526858	None
8316	BRF2	-0.13027	0.202377	0.526992	None
8317	DAB2	-0.13488	0.202405	0.527003	None
8318	ARL6	-0.13433	0.202468	0.527102	None
8319	MCU	-0.17152	0.202517	0.527127	None
8320	KHDRBS1	-0.19428	0.202526	0.527127	None
8321	SLC28A2	0.00317	0.202607	0.527224	None
8322	FBXL16	-0.05992	0.202612	0.527224	None
8323	PNISR	0.216898	0.202699	0.527387	None
8324	LINC00308	0.013844	0.202729	0.527401	None
8325	SAAL1	0.218631	0.202771	0.527449	None
8326	LOC10192	-0.11128	0.202839	0.527563	None
8327	SLC30A1	-0.81358	0.202914	0.527631	None
8328	NDUFB1	0.075689	0.202914	0.527631	None
8329	SIGLEC16	-0.26816	0.202958	0.527659	None
8330	RP11-173E	0.148791	0.202974	0.527659	None
8331	ATP1A1-A	-0.09784	0.203151	0.528006	None
8332	CCDC148	-0.13208	0.203163	0.528006	None
8333	LOC10192	-0.05055	0.203211	0.528006	None
8334	LINC00922	0.041226	0.20322	0.528006	None
8335	KCNAB3	0.033149	0.203247	0.528006	None
8336	HIST2H2BE	-0.47798	0.203254	0.528006	None
8337	NCAPH2	-0.12412	0.203284	0.528021	None
8338	LOC10050(	-0.057	0.20332	0.528051	None
8339	IFT27	-0.17441	0.203499	0.528452	None
8340	LOC10192	-0.02865	0.203712	0.528942	None
8341	FBXO7	-0.2534	0.203737	0.528944	None
8342	PLEKHA6	-0.13864	0.203859	0.529185	None
8343	FAM217A	-0.03099	0.203878	0.529185	None
8344	RSAD2	-0.40535	0.204036	0.52953	None
8345	AXIN2	0.003249	0.204111	0.529656	None
8346	PRRX2	0.071855	0.204133	0.529656	None
8347	MLXIP	0.145404	0.20424	0.52987	None
8348	U2AF2	-0.09778	0.204268	0.529878	None
8349	PPT1	-0.13994	0.20442	0.53018	None
8350	NECAB1	-0.25164	0.204481	0.53018	None

8351	PTK6	0.016758	0.2045	0.53018	None
8352	RBKS	0.317003	0.204512	0.53018	None
8353	SNX33	-0.08795	0.20453	0.53018	None
8354	LOC28493(	0.002832	0.204531	0.53018	None
8355	ARHGEF28	0.011683	0.204572	0.530222	None
8356	TMEM117	-0.16598	0.204632	0.530315	None
8357	ZNF333	-0.10144	0.204735	0.530471	None
8358	PRPF18	0.060661	0.204741	0.530471	None
8359	ZFP28	-0.05091	0.204782	0.530512	None
8360	CCSAP	-0.06067	0.204822	0.530523	None
8361	LIMS2	0.052149	0.204835	0.530523	None
8362	LRRK2	-0.29654	0.204877	0.530569	None
8363	GGH	0.227504	0.205015	0.530861	None
8364	SH2B2	0.147871	0.205043	0.530871	None
8365	DECR2	-0.18528	0.205093	0.530937	None
8366	PSENEN	-0.21915	0.205182	0.531105	None
8367	DKFZp434I	0.011561	0.205292	0.531265	None
8368	THAP3	-0.0632	0.205314	0.531265	None
8369	RIT1	-0.32109	0.205318	0.531265	None
8370	UROD	-0.26382	0.205418	0.531462	None
8371	MAF	-0.18211	0.205504	0.531612	None
8372	SLC46A1	-0.17505	0.205525	0.531612	None
8373	OAZ2	-0.23545	0.205618	0.531622	None
8374	ATP8B4	-0.51821	0.20563	0.531622	None
8375	ACAD8	-0.17923	0.205635	0.531622	None
8376	TP53RK	-0.25258	0.205645	0.531622	None
8377	KRT222	0.022372	0.205652	0.531622	None
8378	ZC3HC1	-0.17231	0.205735	0.531772	None
8379	FAM168A	-0.14279	0.205795	0.531846	None
8380	PCNT	-0.19177	0.205813	0.531846	None
8381	POU6F1	-0.04271	0.205861	0.531907	None
8382	KDM5C	-0.1312	0.205934	0.532032	None
8383	IL37	0.051737	0.20604	0.532245	None
8384	SET	-0.37443	0.206095	0.532322	None
8385	TLE2	-0.07562	0.206174	0.532461	None
8386	STK3	-0.08242	0.206383	0.532939	None
8387	RPL37	-0.04336	0.206506	0.533145	None
8388	NGFRAP1	-0.14967	0.206541	0.533145	None
8389	IL27	0.034718	0.206547	0.533145	None
8390	SPANXA2-	-0.01756	0.206561	0.533145	None
8391	PKIB	-0.15261	0.206599	0.533179	None
8392	FBXO18	-0.07096	0.206647	0.533238	None
8393	FNDC3B	0.095505	0.206673	0.533242	None
8394	TXLNGY	-1.01386	0.206725	0.533312	None
8395	UBL5	0.136426	0.206818	0.533491	None
8396	GAST	-0.05481	0.206911	0.533619	None
8397	RPLP1	-0.03316	0.206918	0.533619	None
8398	HHAT	0.169611	0.206973	0.533684	None
8399	CSN2	0.060913	0.207015	0.533684	None
8400	FANCL	-0.2132	0.207017	0.533684	None
8401	LOC38983	-0.16819	0.207231	0.534172	None
8402	TNP2	0.010417	0.207417	0.534529	None
8403	KLF5	0.264716	0.207419	0.534529	None
8404	RAB22A	0.128241	0.207527	0.534698	None
8405	RP11-445L	0.020946	0.207534	0.534698	None
8406	EMC4	0.139211	0.207771	0.535246	None
8407	PLAA	0.195557	0.20787	0.535436	None
8408	SMAGP	-0.29849	0.207981	0.535661	None

8409	TAPT1-AS1	0.404777	0.208024	0.535707	None
8410	MIR205	0.023746	0.208129	0.535875	None
8411	PITPNM3	-0.14636	0.208139	0.535875	None
8412	AOX1	0.20041	0.208597	0.536992	None
8413	LINC01119	-0.03808	0.208817	0.537492	None
8414	TMEM198	-0.04483	0.208927	0.537713	None
8415	PI4K2A	-0.22266	0.209173	0.53822	None
8416	MRPS22	-0.12419	0.209178	0.53822	None
8417	MEX3B	-0.39165	0.209199	0.53822	None
8418	AXL	-0.39123	0.209301	0.538385	None
8419	MIER1	-0.1713	0.209364	0.538385	None
8420	RP11-568N	-0.37711	0.209365	0.538385	None
8421	CACNA1C	-0.0217	0.209373	0.538385	None
8422	LOC10192	0.034827	0.2094	0.538385	None
8423	HIST1H2AJ	-0.30872	0.209412	0.538385	None
8424	HIVEP1	0.198036	0.209537	0.538591	None
8425	LPPR1	0.025646	0.209566	0.538591	None
8426	ZFP69	-0.19577	0.209567	0.538591	None
8427	ANAPC15	0.221113	0.209647	0.538734	None
8428	C1QTNF7	0.006543	0.209774	0.538995	None
8429	EXOSC5	-0.2259	0.209824	0.539059	None
8430	PPP1R17	0.028275	0.209881	0.539069	None
8431	APOBEC3B	0.401065	0.209907	0.539069	None
8432	DSE	-0.01583	0.209916	0.539069	None
8433	OBSL1	-0.11128	0.209939	0.539069	None
8434	CLK4	0.247519	0.209953	0.539069	None
8435	ZGLP1	-0.08614	0.209977	0.539069	None
8436	HSD11B1L	-0.03029	0.210009	0.539088	None
8437	CDPF1	-0.10281	0.210034	0.539088	None
8438	GTF3C2-AS	0.010717	0.210116	0.539234	None
8439	LGALS12	0.314355	0.210173	0.539316	None
8440	ENKD1	-0.17499	0.21021	0.539341	None
8441	FAM122A	-0.19596	0.210232	0.539341	None
8442	CMTM1	0.056823	0.210348	0.53953	None
8443	CIB3	0.055832	0.210356	0.53953	None
8444	BRD7P3	-0.03516	0.210441	0.539686	None
8445	MDM1	-0.18965	0.21049	0.539705	None
8446	KCNQ1	-0.11055	0.210499	0.539705	None
8447	FRS3	-0.03834	0.210551	0.539732	None
8448	LOC73115	-0.19109	0.210559	0.539732	None
8449	INSIG1	0.274221	0.210692	0.539989	None
8450	NIT2	0.200716	0.210739	0.539989	None
8451	TEX12	0.031225	0.21074	0.539989	None
8452	OLFML2A	-0.32449	0.210759	0.539989	None
8453	LINC00421	0.009163	0.210955	0.540373	None
8454	EPHA1	-0.02109	0.210959	0.540373	None
8455	LOC10192	0.034816	0.211033	0.540499	None
8456	ATG2A	-0.19603	0.211067	0.540522	None
8457	CNTD2	-0.05834	0.211136	0.540636	None
8458	MUC19	-0.30766	0.211214	0.540679	None
8459	TMEM206	-0.17427	0.211219	0.540679	None
8460	CFHR4	0.108895	0.211228	0.540679	None
8461	LRRN2	-0.07509	0.211371	0.540935	None
8462	C6orf48	-0.17262	0.211393	0.540935	None
8463	TTC36	0.072923	0.211403	0.540935	None
8464	RP11-252E	-0.12235	0.211488	0.541056	None
8465	ZNF667	-0.24174	0.211553	0.541056	None
8466	HSPA1L	-0.09076	0.211563	0.541056	None

8467	TMEM71	0.445885	0.211569	0.541056	None
8468	ELAVL4	-0.09798	0.211575	0.541056	None
8469	NEK5	-0.19803	0.211662	0.541173	None
8470	YTHDF2	-0.11259	0.211681	0.541173	None
8471	GRIN1	0.012221	0.211696	0.541173	None
8472	SPG20OS	0.167504	0.211786	0.541326	None
8473	HACE1	0.224598	0.211806	0.541326	None
8474	ATP6V0E2-	-0.0719	0.211953	0.541639	None
8475	SESTD1	0.296986	0.212042	0.541732	None
8476	MGST2	-0.17659	0.212055	0.541732	None
8477	SRCAP	-0.02186	0.212089	0.541732	None
8478	DNAJC8	-0.15552	0.21209	0.541732	None
8479	LINC01114	-0.17977	0.21224	0.541921	None
8480	DGCR9	-0.0299	0.212243	0.541921	None
8481	THSD7A	-0.48244	0.212246	0.541921	None
8482	HMGN3-A	0.04954	0.212271	0.541921	None
8483	CYP1A2	0.07494	0.212308	0.541921	None
8484	HESX1	-0.22874	0.212324	0.541921	None
8485	PPP6R3	0.171882	0.212358	0.541921	None
8486	ILK	-0.24289	0.212364	0.541921	None
8487	SLMAP	0.108812	0.212464	0.542112	None
8488	HERC6	-0.14905	0.212507	0.542158	None
8489	PTPRH	0.052786	0.212541	0.542182	None
8490	KRTAP5-9	0.04903	0.212593	0.54222	None
8491	STAU2	-0.30918	0.212606	0.54222	None
8492	FANCB	-0.11877	0.212796	0.542577	None
8493	RP11-384F	-0.13609	0.212796	0.542577	None
8494	CSF2RA	-0.15532	0.212877	0.54272	None
8495	RNF38	0.104537	0.21295	0.54284	None
8496	MYLK	0.092192	0.212997	0.542897	None
8497	CD48	0.28249	0.213126	0.542932	None
8498	AP2A1	-0.06078	0.213129	0.542932	None
8499	UBE2Z	-0.19237	0.213154	0.542932	None
8500	C1orf112	-0.29338	0.213163	0.542932	None
8501	EVC	0.038059	0.213164	0.542932	None
8502	RP11-567I.	-0.06659	0.213182	0.542932	None
8503	COL13A1	0.049012	0.213224	0.542932	None
8504	LA16c-358	-0.11126	0.213232	0.542932	None
8505	AGMAT	-0.14897	0.213236	0.542932	None
8506	CHKA	-0.17918	0.213265	0.542941	None
8507	POMT1	-0.25393	0.21336	0.543072	None
8508	FAM102B	0.175148	0.213367	0.543072	None
8509	GRB2	-0.2268	0.21343	0.54317	None
8510	ZNF683	-0.09268	0.213511	0.54331	None
8511	CXorf36	-0.00493	0.213615	0.543438	None
8512	KCNJ6	-0.01909	0.213616	0.543438	None
8513	SGSM2	0.25141	0.213636	0.543438	None
8514	DYNLL1	-0.21186	0.213664	0.543446	None
8515	PCNXL2	-0.12916	0.213726	0.543455	None
8516	LOC10050(	-0.16407	0.213738	0.543455	None
8517	LOC10192!	0.028281	0.213743	0.543455	None
8518	SUGT1P1	-0.10381	0.213785	0.543498	None
8519	CLUHP3	-0.14437	0.213966	0.543824	None
8520	POMC	-0.09103	0.21397	0.543824	None
8521	WDR13	-0.10292	0.213988	0.543824	None
8522	PGM5	-0.08874	0.214066	0.543958	None
8523	DGCR6L	0.025285	0.214178	0.544064	None
8524	TIMP4	0.13042	0.214225	0.544064	None

8525	C17orf89	-0.07776	0.214228	0.544064	None
8526	SRRM1	-0.20616	0.214238	0.544064	None
8527	LSS	-0.15863	0.214266	0.544064	None
8528	CERK	0.219265	0.214279	0.544064	None
8529	CYB5A	-0.22534	0.214284	0.544064	None
8530	FLJ35934	-0.135	0.214367	0.54421	None
8531	LOC10272	0.011162	0.214426	0.544238	None
8532	PTPRK	-0.20104	0.214428	0.544238	None
8533	SUPT3H	-0.19752	0.214453	0.544238	None
8534	LOC10099	0.004322	0.214523	0.544352	None
8535	AGO2	-0.24414	0.214602	0.5444	None
8536	EFNB2	0.348717	0.214617	0.5444	None
8537	MFAP2	0.208268	0.214617	0.5444	None
8538	C14orf79	-0.06876	0.214652	0.544424	None
8539	ETAA1	0.133346	0.214741	0.54451	None
8540	CLEC4A	0.319233	0.214743	0.54451	None
8541	EWSR1	0.168848	0.214786	0.54451	None
8542	MBD3L2	0.005965	0.214787	0.54451	None
8543	NEK2	0.251231	0.214893	0.544715	None
8544	BCL7A	0.137552	0.215067	0.545027	None
8545	AUP1	-0.21013	0.215083	0.545027	None
8546	PCGF6	0.1762	0.215091	0.545027	None
8547	SMAP1	0.154717	0.215146	0.545102	None
8548	MED17	0.201911	0.215239	0.545144	None
8549	LOC10012	-0.10288	0.21524	0.545144	None
8550	EVPL	-0.10149	0.215251	0.545144	None
8551	NEIL2	-0.12847	0.215271	0.545144	None
8552	FXYD5	-0.3124	0.215291	0.545144	None
8553	SUSD5	-0.21475	0.215314	0.545144	None
8554	FAM86C1	-0.03979	0.215422	0.545354	None
8555	GRK6	-0.13549	0.215514	0.545522	None
8556	MKNK2	-0.23098	0.215557	0.545522	None
8557	NLGN1	-0.20138	0.215579	0.545522	None
8558	LOC72806	-0.28122	0.215614	0.545522	None
8559	PTCD3	-0.17804	0.215614	0.545522	None
8560	ARMC4	-0.07817	0.215645	0.545537	None
8561	CALN1	-0.40712	0.215732	0.545645	None
8562	CLTC-IT1	0.218918	0.215738	0.545645	None
8563	HDC	0.553777	0.215808	0.545757	None
8564	AP001462.	-0.14243	0.215851	0.545764	None
8565	RNF139-A	-0.1252	0.215861	0.545764	None
8566	GVINP1	0.136788	0.215922	0.545848	None
8567	LOC44002	0.011814	0.215944	0.545848	None
8568	DQ576994	-0.08856	0.215984	0.545883	None
8569	RPS4X	0.039948	0.216166	0.546235	None
8570	ANKRA2	-0.20606	0.216175	0.546235	None
8571	KIAA1598	-0.49488	0.216239	0.546235	None
8572	LOC10028	-0.07084	0.216241	0.546235	None
8573	EPN1	-0.13732	0.216249	0.546235	None
8574	TRIM65	-0.04597	0.216286	0.546263	None
8575	ECH1	-0.15431	0.216311	0.546263	None
8576	GALNT1	-0.09982	0.21636	0.546324	None
8577	LRRC43	0.021869	0.216404	0.546371	None
8578	LOC10013	0.044264	0.216445	0.546411	None
8579	TBKBP1	-0.03139	0.21657	0.546664	None
8580	DLG1	-0.28737	0.21672	0.546974	None
8581	C19orf18	-0.20783	0.216765	0.546974	None
8582	RPUSD2	-0.20561	0.216769	0.546974	None

8583	LEF1	0.202935	0.216806	0.547003	None
8584	SLC43A2	0.166225	0.216855	0.547013	None
8585	CUX1	-0.21271	0.216882	0.547013	None
8586	LINC00862	0.027273	0.216885	0.547013	None
8587	TEAD3	0.040351	0.216949	0.54711	None
8588	USP43	0.170673	0.216982	0.54713	None
8589	LOC15368	-0.29885	0.217046	0.547226	None
8590	SLC25A53	-0.03506	0.217091	0.547275	None
8591	MYBPH	0.012089	0.217162	0.547319	None
8592	MCM7	0.242851	0.21718	0.547319	None
8593	KPNA3	-0.14784	0.21719	0.547319	None
8594	GDF10	-0.22031	0.217209	0.547319	None
8595	CASP2	-0.23316	0.217287	0.547355	None
8596	LOC44124	-0.06268	0.217299	0.547355	None
8597	TMEM167	-0.10891	0.217306	0.547355	None
8598	PTGR2	0.234826	0.217324	0.547355	None
8599	FRS2	-0.09708	0.217385	0.547411	None
8600	NCAPH	0.324421	0.217397	0.547411	None
8601	LOC10272	0.061087	0.217453	0.547464	None
8602	ZC2HC1C	-0.06338	0.217469	0.547464	None
8603	CD6	0.010724	0.217715	0.547983	None
8604	C22orf46	-0.09112	0.217726	0.547983	None
8605	LOC10050	0.050756	0.217777	0.548049	None
8606	ATRN	-0.1401	0.217905	0.548307	None
8607	RYR3	0.606148	0.217966	0.548396	None
8608	COL8A1	0.004878	0.217995	0.548407	None
8609	ZNF718	0.198627	0.218081	0.548482	None
8610	CCNDBP1	-0.18109	0.218091	0.548482	None
8611	BBS5	-0.05467	0.218101	0.548482	None
8612	ATXN2L	-0.12346	0.218162	0.548508	None
8613	RAP2C	0.278043	0.218162	0.548508	None
8614	LENG1	-0.16897	0.218228	0.54861	None
8615	TMTC4	-0.21469	0.218301	0.548727	None
8616	NANOS1	-0.24269	0.218329	0.548727	None
8617	CYP2C19	0.015059	0.21835	0.548727	None
8618	MZT2B	0.176214	0.218531	0.549063	None
8619	HCG26	0.248494	0.218535	0.549063	None
8620	VCPKMT	0.160764	0.218595	0.549067	None
8621	ERVH-1	0.112848	0.218616	0.549067	None
8622	ZCCHC24	-0.36018	0.21862	0.549067	None
8623	TMCO3	-0.12579	0.218638	0.549067	None
8624	WNT7A	0.025857	0.218733	0.549242	None
8625	RPL27	-0.04715	0.218811	0.549374	None
8626	LOC10050	-0.26757	0.218899	0.549531	None
8627	BCL2L12	-0.22939	0.218942	0.549575	None
8628	ZNF850	0.356693	0.219058	0.549728	None
8629	RUFY1	-0.18274	0.219075	0.549728	None
8630	RP11-436D	0.018437	0.219079	0.549728	None
8631	RP11-792	-0.09738	0.219132	0.549799	None
8632	NSMCE2	-0.13906	0.219198	0.549901	None
8633	CPA3	0.481267	0.219326	0.550131	None
8634	C14orf166	-0.07256	0.219341	0.550131	None
8635	H3F3A	-0.18656	0.21941	0.550195	None
8636	KIRREL	-0.01006	0.219423	0.550195	None
8637	RAB38	-0.3571	0.219443	0.550195	None
8638	ATP5F1	-0.11742	0.219622	0.55058	None
8639	LOC10192	0.212303	0.219675	0.550651	None
8640	PATL2	-0.15257	0.21974	0.550714	None

8641	RNLS	-0.11381	0.219751	0.550714	None
8642	DCAF7	-0.21109	0.219782	0.550727	None
8643	RP11-111J	-0.06771	0.219857	0.550851	None
8644	FANCC	0.045	0.219894	0.55088	None
8645	KB-431C1.	-0.05482	0.219945	0.55091	None
8646	CERS4	-0.10626	0.219957	0.55091	None
8647	ARPIN	-0.12808	0.220008	0.550975	None
8648	ATP5H	-0.17355	0.220137	0.551233	None
8649	FKBP3	-0.07457	0.220334	0.551515	None
8650	POMZP3	-0.3338	0.220343	0.551515	None
8651	MALAT1	0.221906	0.220351	0.551515	None
8652	FAR2	-0.21458	0.220368	0.551515	None
8653	COPZ1	-0.15102	0.220399	0.551515	None
8654	MORF4L2-	-0.097	0.220417	0.551515	None
8655	LINC00939	0.013145	0.220443	0.551515	None
8656	NLRP12	-0.12541	0.220453	0.551515	None
8657	CLTC	-0.08689	0.220523	0.551626	None
8658	HS3ST4	-0.15559	0.220596	0.551702	None
8659	KCTD1	-0.09061	0.220604	0.551702	None
8660	PDK4	-0.23335	0.220673	0.551764	None
8661	MUC6	-0.03587	0.220693	0.551764	None
8662	RP11-58O:	0.041719	0.220706	0.551764	None
8663	GYG2	-0.08577	0.220764	0.551769	None
8664	FAM161B	0.103243	0.22078	0.551769	None
8665	IER5L	-0.08025	0.220784	0.551769	None
8666	ANKRD20A	0.029528	0.220861	0.551899	None
8667	HOXB4	-0.16404	0.220956	0.552071	None
8668	GPX3	-0.5537	0.221034	0.552202	None
8669	PIK3CG	0.211274	0.221103	0.55227	None
8670	C1QBP	0.182851	0.221112	0.55227	None
8671	POLD4	-0.19815	0.221166	0.55234	None
8672	FAM183CP	-0.02234	0.22127	0.552443	None
8673	SDR39U1	0.136673	0.221298	0.552443	None
8674	MEPCE	-0.19376	0.221321	0.552443	None
8675	CLPS	-0.03263	0.221322	0.552443	None
8676	C18orf12	0.050635	0.221342	0.552443	None
8677	OLFM3	-0.25643	0.22136	0.552443	None
8678	WFDC3	-0.04953	0.221392	0.552444	None
8679	ARMCX1	0.268386	0.221411	0.552444	None
8680	CDK1	0.38163	0.221502	0.552583	None
8681	BIK	-0.22417	0.221551	0.552583	None
8682	TFAP2E	-0.16504	0.221579	0.552583	None
8683	WDR4	-0.14091	0.221641	0.552583	None
8684	PTGER4	0.149208	0.221647	0.552583	None
8685	DOCK6	-0.021	0.221654	0.552583	None
8686	C20orf141	-0.03606	0.221658	0.552583	None
8687	LOC10192	0.037327	0.221697	0.552583	None
8688	ZNF223	-0.13898	0.221717	0.552583	None
8689	SALL4	-0.41263	0.221722	0.552583	None
8690	CXorf56	-0.20628	0.221844	0.552795	None
8691	RP11-27I1.	-0.15913	0.221858	0.552795	None
8692	IQCG	-0.12928	0.221998	0.55308	None
8693	OTUD6B	0.279182	0.222067	0.553094	None
8694	GAS6	-0.16421	0.222069	0.553094	None
8695	MPND	-0.23839	0.22208	0.553094	None
8696	CAMSAP3	0.014776	0.222261	0.553479	None
8697	NAPSB	0.503856	0.222529	0.554078	None
8698	PSMB9	0.220121	0.222552	0.554078	None

8699	TNFRSF21	0.293378	0.222943	0.554985	None
8700	ATG4D	0.173406	0.222968	0.554985	None
8701	LOC10012{	-0.27103	0.223069	0.555173	None
8702	KCNAB1	-0.16599	0.223129	0.555258	None
8703	MLLT10	-0.13477	0.223237	0.555367	None
8704	C3orf27	-0.0494	0.22324	0.555367	None
8705	MGAT2	-0.18081	0.223294	0.555367	None
8706	FAM227B	-0.05698	0.223298	0.555367	None
8707	NCR2	0.054267	0.223301	0.555367	None
8708	FOXN3	0.110749	0.223342	0.555372	None
8709	MAGI2	-0.10283	0.223371	0.555372	None
8710	ENTPD1	-0.35546	0.223419	0.555372	None
8711	SMARCA4	-0.24434	0.223436	0.555372	None
8712	DCTN6	-0.13731	0.223448	0.555372	None
8713	METTL25	-0.13481	0.223457	0.555372	None
8714	CPB2	0.0031	0.223559	0.555562	None
8715	CCDC105	0.035559	0.223788	0.55602	None
8716	DLEC1	0.013798	0.223794	0.55602	None
8717	CYB5RL	-0.11895	0.224039	0.556446	None
8718	CRYGS	-0.11711	0.22405	0.556446	None
8719	LRP2	0.046402	0.224052	0.556446	None
8720	AX746968	0.063523	0.224069	0.556446	None
8721	BTRC	0.060689	0.224148	0.55658	None
8722	GDAP1L1	-0.0618	0.224219	0.556624	None
8723	C12orf57	0.233377	0.224293	0.556624	None
8724	MYO1B	0.117268	0.224304	0.556624	None
8725	C1orf74	-0.14106	0.224323	0.556624	None
8726	TEX15	-0.07941	0.224337	0.556624	None
8727	CYP2B7P	-0.04137	0.224341	0.556624	None
8728	MIR302B	-0.20604	0.224366	0.556624	None
8729	CDC5L	-0.20428	0.224372	0.556624	None
8730	LOC10013:	0.033794	0.224489	0.556802	None
8731	LOC10099(	-0.20239	0.224495	0.556802	None
8732	CCDC117	-0.12343	0.22453	0.556826	None
8733	ZNF274	-0.19304	0.224639	0.557032	None
8734	KTN1	0.216518	0.22479	0.557341	None
8735	LOC10004!	-0.15178	0.224833	0.557372	None
8736	KIAA1429	0.24562	0.224865	0.557372	None
8737	TMEM51	-0.21516	0.224879	0.557372	None
8738	C12orf42	0.022288	0.22491	0.557384	None
8739	SHANK2-A	0.034839	0.224935	0.557384	None
8740	GAB4	0.010532	0.224965	0.557393	None
8741	COX16	-0.25288	0.22512	0.557682	None
8742	TP53INP2	0.278964	0.225151	0.557682	None
8743	REC114	0.044106	0.225159	0.557682	None
8744	ZNF484	0.015373	0.22521	0.557744	None
8745	SRSF8	0.119522	0.22524	0.557756	None
8746	POLH	-0.14046	0.225306	0.557855	None
8747	SLC9A6	-0.16787	0.225463	0.55811	None
8748	SNRPF	-0.2286	0.225468	0.55811	None
8749	HIAT1	0.138839	0.225486	0.55811	None
8750	SFMBT1	0.167806	0.225532	0.558159	None
8751	SAT1	-0.32007	0.225648	0.558383	None
8752	AGPS	0.167799	0.225761	0.5586	None
8753	TSPYL2	0.252897	0.225817	0.558674	None
8754	MAFG-AS1	-0.04346	0.225876	0.558756	None
8755	SF1	0.361825	0.225941	0.558853	None
8756	RCC2	0.119243	0.225968	0.558855	None

8757	LOC10192!	0.084504	0.226068	0.559038	None
8758	FAM32A	-0.13023	0.226167	0.559156	None
8759	TUBGCP5	0.143191	0.226167	0.559156	None
8760	TTC7A	0.277639	0.226203	0.559162	None
8761	MIER2	-0.0272	0.226221	0.559162	None
8762	KRT8	-0.19149	0.226279	0.559205	None
8763	LINC00930	0.036552	0.226309	0.559205	None
8764	LINC01057	0.005331	0.226345	0.559205	None
8765	HPYR1	0.028331	0.226351	0.559205	None
8766	LRTOMT	-0.01696	0.226368	0.559205	None
8767	MORC3	0.170033	0.226452	0.559351	None
8768	SLC6A6	0.033437	0.226524	0.559465	None
8769	C6orf164	-0.1218	0.226843	0.560181	None
8770	YBEY	-0.25918	0.226866	0.560181	None
8771	NDRG2	0.012784	0.226922	0.56025	None
8772	UBLCP1	-0.2001	0.226946	0.56025	None
8773	GPR182	-0.3257	0.227043	0.560407	None
8774	FKBP14	-0.31784	0.227061	0.560407	None
8775	PKDREJ	0.025509	0.227108	0.560457	None
8776	MPP3	-0.13123	0.227191	0.5606	None
8777	TCL6	0.098088	0.227241	0.560625	None
8778	DCLK2	-0.03704	0.227279	0.560625	None
8779	TCL1A	-0.36819	0.2273	0.560625	None
8780	FAM126A	-0.07064	0.227328	0.560625	None
8781	NCALD	-0.06934	0.227331	0.560625	None
8782	HRH1	-0.18834	0.227443	0.560804	None
8783	CALM1	0.247456	0.227455	0.560804	None
8784	MAN1C1	-0.38034	0.22752	0.560877	None
8785	INPP5J	-0.113	0.227547	0.560877	None
8786	MAPK12	-0.08225	0.227563	0.560877	None
8787	SNRPB2	0.086466	0.227622	0.560959	None
8788	LOC10192!	0.017108	0.227699	0.561085	None
8789	LOC10272	0.002919	0.227857	0.561412	None
8790	IRX3	-0.41058	0.227901	0.561436	None
8791	LOC10192!	0.015449	0.227919	0.561436	None
8792	OAZ3	-0.08081	0.228052	0.561599	None
8793	FAM179A	-0.14085	0.228076	0.561599	None
8794	ZNF22	0.127765	0.228091	0.561599	None
8795	VLDLR	-0.23673	0.228103	0.561599	None
8796	LOC10013	-0.23822	0.228117	0.561599	None
8797	PLEKHG4B	-0.17851	0.228141	0.561599	None
8798	LOC10050	-0.02911	0.228167	0.561599	None
8799	YTHDC2	0.194888	0.228192	0.561599	None
8800	CMTR2	0.303484	0.228304	0.56168	None
8801	CLCN4	-0.12034	0.228316	0.56168	None
8802	C5orf51	-0.08233	0.228347	0.56168	None
8803	KRCC1	-0.23139	0.22835	0.56168	None
8804	CD52	-0.3445	0.228355	0.56168	None
8805	LOC10050	0.059779	0.228463	0.5618	None
8806	AC100830.	-0.06035	0.228469	0.5618	None
8807	HAVCR1P1	0.092131	0.228482	0.5618	None
8808	ZNF408	-0.08891	0.22853	0.561854	None
8809	RP3-334F4	0.128709	0.228572	0.561893	None
8810	STL	-0.07018	0.228618	0.561944	None
8811	LOC10050!	-0.06216	0.22865	0.561958	None
8812	RFPL1S	-0.26662	0.228813	0.562296	None
8813	DDIAS	-0.28457	0.228887	0.562357	None
8814	FLJ45513	-0.16797	0.22889	0.562357	None

8815	LOC64898	0.150822	0.228931	0.562388	None
8816	LINC00475	0.048412	0.229001	0.562388	None
8817	RNASE4	0.195007	0.229005	0.562388	None
8818	STT3A	-0.24816	0.229007	0.562388	None
8819	ZNF287	-0.01713	0.229042	0.562411	None
8820	NUDT5	-0.11587	0.229143	0.562572	None
8821	PCP2	-0.10758	0.229194	0.562572	None
8822	MMP7	-0.32213	0.229234	0.562572	None
8823	NARFL	-0.14333	0.229234	0.562572	None
8824	SLCO1B3	0.04907	0.229252	0.562572	None
8825	RFT1	-0.13929	0.229263	0.562572	None
8826	NR2E3	0.020425	0.229351	0.562711	None
8827	CHMP5	-0.16784	0.229372	0.562711	None
8828	AP4M1	-0.13084	0.229434	0.5628	None
8829	PTPRO	-0.3913	0.229615	0.563179	None
8830	PIGM	-0.22862	0.229688	0.563295	None
8831	PP12719	0.152838	0.229718	0.563304	None
8832	ARID3A	-0.12368	0.229745	0.563308	None
8833	DDX54	-0.15423	0.229804	0.563355	None
8834	LOC72997	-0.11625	0.229817	0.563355	None
8835	TRPV4	-0.05935	0.229883	0.563444	None
8836	PDGFRB	-0.13451	0.229905	0.563444	None
8837	AMPD1	-0.21843	0.22999	0.563511	None
8838	TFAP2B	0.001652	0.229998	0.563511	None
8839	CLTB	-0.20774	0.23001	0.563511	None
8840	CD74	-0.22765	0.230138	0.563591	None
8841	HOMER3	-0.23869	0.230162	0.563591	None
8842	GSTM1	-0.30354	0.230175	0.563591	None
8843	ATP6V1G2	-0.12924	0.230183	0.563591	None
8844	PLTP	-0.3887	0.230207	0.563591	None
8845	COPRS	-0.16426	0.230225	0.563591	None
8846	RP1-20C7.	0.057972	0.230225	0.563591	None
8847	GPR125	0.226983	0.230601	0.56437	None
8848	CPED1	0.225457	0.23062	0.56437	None
8849	LOC28550	-0.08853	0.230622	0.56437	None
8850	C17orf99	-0.4436	0.230739	0.564593	None
8851	OTUD3	-0.16309	0.230777	0.564623	None
8852	CBX8	0.046519	0.230851	0.56474	None
8853	ZKSCAN2	-0.18413	0.230983	0.564952	None
8854	SPIN3	-0.13169	0.23099	0.564952	None
8855	RNF34	0.224932	0.231025	0.564973	None
8856	GRPEL1	-0.21981	0.231097	0.565086	None
8857	APLN	-0.07862	0.231204	0.565237	None
8858	TUSC8	0.097555	0.231211	0.565237	None
8859	PJA2	0.159292	0.231284	0.565352	None
8860	KIAA1211	-0.35852	0.231358	0.565437	None
8861	LOC28605	0.013495	0.231371	0.565437	None
8862	SLC4A5	-0.05337	0.231432	0.565518	None
8863	C9orf37	-0.13919	0.231456	0.565518	None
8864	ARFGEF1	-0.25009	0.231496	0.56555	None
8865	KCNC2	-0.03508	0.231524	0.565557	None
8866	MEOX1	0.053716	0.23163	0.565751	None
8867	CCDC91	0.218923	0.231677	0.565803	None
8868	CTD-2035I	0.381519	0.231791	0.565959	None
8869	IL11RA	-0.11235	0.231794	0.565959	None
8870	FBLN2	-0.19419	0.231834	0.565995	None
8871	POM121	0.117652	0.231878	0.565999	None
8872	EPHB6	-0.18023	0.231889	0.565999	None

8873	SLC25A3	0.069433	0.231933	0.566043	None
8874	HERC1	-0.13468	0.231981	0.566098	None
8875	DUSP15	0.055907	0.232022	0.566131	None
8876	ITM2B	-0.18881	0.232047	0.566131	None
8877	ADD3	0.178955	0.232157	0.566336	None
8878	LOC10013	0.21688	0.232277	0.566564	None
8879	C22orf39	-0.11283	0.232329	0.566627	None
8880	TUBB4B	0.230216	0.232404	0.566745	None
8881	DNAJB8	-0.02552	0.232438	0.566745	None
8882	CREB3L3	-0.0259	0.232475	0.566745	None
8883	KIAA1652	-0.10013	0.232482	0.566745	None
8884	PLA2G2A	0.047698	0.232846	0.56757	None
8885	EFHC1	0.263951	0.232929	0.567707	None
8886	AIFM3	-0.05808	0.232976	0.567713	None
8887	ZNF530	-0.08852	0.232984	0.567713	None
8888	ADHFE1	-0.25895	0.233083	0.56789	None
8889	CDKN2C	0.317443	0.233159	0.567968	None
8890	LOC10192	0.015781	0.233167	0.567968	None
8891	NR2E1	-0.01051	0.233353	0.568357	None
8892	PITX3	-0.07544	0.233409	0.568429	None
8893	KCNH7	0.009285	0.233466	0.568505	None
8894	RP3-508D	0.014303	0.233554	0.568606	None
8895	RP11-180	-0.44135	0.233606	0.568606	None
8896	MELK	0.283398	0.233634	0.568606	None
8897	NFASC	0.012617	0.233635	0.568606	None
8898	SAPCD2	-0.28822	0.23365	0.568606	None
8899	TMEM52	0.056274	0.233665	0.568606	None
8900	OLFM4	0.441776	0.233726	0.56869	None
8901	MTG2	-0.12388	0.23384	0.568903	None
8902	RP9	-0.11306	0.233944	0.569093	None
8903	MAMDC4	-0.02288	0.234128	0.569472	None
8904	SELL	0.309579	0.234153	0.569472	None
8905	LOC10012	-0.20439	0.234296	0.569719	None
8906	PTPLAD1	-0.19356	0.234312	0.569719	None
8907	CCDC152	0.247439	0.234374	0.569719	None
8908	MSR1	-0.12243	0.234398	0.569719	None
8909	SLC9A3R2	0.020784	0.234426	0.569719	None
8910	GATA6	0.041234	0.23446	0.569719	None
8911	ANKRD54	-0.08046	0.234478	0.569719	None
8912	LOC10013	-0.14699	0.23448	0.569719	None
8913	C14orf28	-0.1804	0.234498	0.569719	None
8914	ATL1	0.289916	0.234522	0.569719	None
8915	FKBP8	-0.07794	0.2346	0.569719	None
8916	MAPKBP1	0.107042	0.234601	0.569719	None
8917	NMUR1	0.030276	0.234616	0.569719	None
8918	SIRT6	-0.06682	0.23464	0.569719	None
8919	AC092667.	0.024039	0.234691	0.569719	None
8920	KIF14	0.32418	0.2347	0.569719	None
8921	MED13L	0.222924	0.234701	0.569719	None
8922	SKI	0.322024	0.234868	0.570059	None
8923	VPS41	-0.29596	0.235003	0.570304	None
8924	SERPINB1	0.373046	0.235022	0.570304	None
8925	RPL13AP1	0.02591	0.23516	0.570527	None
8926	OR52K3P	-0.13787	0.235166	0.570527	None
8927	TRIM2	0.084474	0.235271	0.570717	None
8928	CCDC11	0.032422	0.235354	0.570855	None
8929	PGM5-AS1	-0.30843	0.235433	0.570966	None
8930	LDHB	0.09137	0.235453	0.570966	None

8931	BICC1	-0.16362	0.235535	0.571102	None
8932	RP4-675G8	0.023452	0.235571	0.571126	None
8933	LINC00328	-0.11419	0.235787	0.571417	None
8934	GYPA	-0.56215	0.235788	0.571417	None
8935	OGDHL	-0.12982	0.235802	0.571417	None
8936	GOLPH3	0.177705	0.23581	0.571417	None
8937	SLC10A2	0.042778	0.235823	0.571417	None
8938	NCAPD3	0.164739	0.235942	0.571641	None
8939	FOXE1	-0.01265	0.236004	0.571727	None
8940	MICALL2	-0.12268	0.236067	0.571815	None
8941	ENTHD2	0.057849	0.236108	0.571843	None
8942	PMS2L2	-0.25687	0.236131	0.571843	None
8943	TPM2	-0.05022	0.236159	0.571846	None
8944	C6orf132	-0.03956	0.236495	0.572545	None
8945	SYNE2	-0.00864	0.2365	0.572545	None
8946	MYH6	-0.01331	0.236587	0.57269	None
8947	SLC35F2	-0.1982	0.236617	0.572698	None
8948	NADSYN1	-0.08326	0.236769	0.572967	None
8949	PSMB2	0.106576	0.23679	0.572967	None
8950	ZDHHC7	-0.14417	0.236807	0.572967	None
8951	KRT33B	0.034167	0.236895	0.57307	None
8952	ZNF767P	-0.25206	0.236903	0.57307	None
8953	MYEOV2	-0.07936	0.236933	0.573079	None
8954	CCNB1IP1	-0.19207	0.237026	0.573241	None
8955	HTR3A	0.016627	0.237175	0.573536	None
8956	COX7A1	-0.1206	0.237241	0.57361	None
8957	PCGEM1	0.009373	0.237258	0.57361	None
8958	GTSE1-AS1	-0.01221	0.237311	0.573674	None
8959	FAM161A	-0.16952	0.237496	0.573993	None
8960	LOC10192	-0.02623	0.237507	0.573993	None
8961	AX747652	0.213839	0.237523	0.573993	None
8962	LRRC8D	-0.13404	0.237621	0.574165	None
8963	USP34	0.153447	0.237647	0.574165	None
8964	APC	-0.13955	0.237708	0.574248	None
8965	DLL1	0.169022	0.237779	0.574356	None
8966	GNL2	0.15197	0.237847	0.574456	None
8967	CDX1	0.032217	0.237883	0.574479	None
8968	RPA4	-0.26166	0.23791	0.574481	None
8969	AQP10	-0.23147	0.238017	0.574675	None
8970	LGALS1	0.48503	0.238094	0.574734	None
8971	DDX50	-0.21481	0.238094	0.574734	None
8972	PCAT18	-0.27404	0.23818	0.574877	None
8973	HAPLN3	-0.06748	0.238222	0.574885	None
8974	COL16A1	-0.10502	0.238237	0.574885	None
8975	TSNAXIP1	-0.02607	0.23827	0.574901	None
8976	TSPYL5	0.435062	0.238507	0.575351	None
8977	LINC01010	0.020583	0.23851	0.575351	None
8978	TPSAB1	-0.39186	0.238593	0.575399	None
8979	DIO3	-0.0514	0.238603	0.575399	None
8980	KIF3A	0.135453	0.238609	0.575399	None
8981	LOC10013	-0.34689	0.238684	0.575514	None
8982	TAF6	-0.20069	0.238717	0.57553	None
8983	PFDN2	-0.13501	0.238764	0.57558	None
8984	PTGFRN	-0.08975	0.238867	0.575714	None
8985	MAP3K2	0.240362	0.238873	0.575714	None
8986	TMSB4X	-0.19559	0.238956	0.575833	None
8987	OR7E47P	-0.03556	0.238988	0.575833	None
8988	NHLRC4	0.073914	0.239002	0.575833	None

8989	PPP1R14B	0.208284	0.239099	0.575993	None
8990	RHOC	-0.14385	0.239122	0.575993	None
8991	CCL8	0.627467	0.23917	0.57604	None
8992	U91328.20	-0.04275	0.239247	0.57604	None
8993	TMEM67	-0.15273	0.239272	0.57604	None
8994	C3orf49	0.062519	0.239273	0.57604	None
8995	KRAS	0.280211	0.239298	0.57604	None
8996	LTV1	-0.14564	0.239316	0.57604	None
8997	NDUFB4	-0.10843	0.239351	0.57604	None
8998	CASP3	-0.24415	0.239354	0.57604	None
8999	ADH5	-0.1164	0.239535	0.576302	None
9000	NPB	-0.06002	0.23954	0.576302	None
9001	LRP5	-0.05671	0.239565	0.576302	None
9002	ANKRD18E	-0.03637	0.239573	0.576302	None
9003	ZNF17	-0.17684	0.239596	0.576302	None
9004	PFN1	-0.42674	0.239641	0.576347	None
9005	LOC10050	0.014135	0.239754	0.576476	None
9006	DIS3	-0.18793	0.239755	0.576476	None
9007	RHPN1	-0.06771	0.239789	0.576476	None
9008	COMMD1(	-0.25361	0.239801	0.576476	None
9009	NLGN3	-0.03612	0.239888	0.576607	None
9010	CCDC71	-0.11696	0.239915	0.576607	None
9011	SPI1	-0.12882	0.239936	0.576607	None
9012	PRKCE	0.2101	0.239999	0.576696	None
9013	CRYZ	-0.21787	0.240063	0.576786	None
9014	CER1	0.044539	0.240094	0.576797	None
9015	ARHGDIB	0.264019	0.240155	0.576811	None
9016	C2orf49	-0.2138	0.240183	0.576811	None
9017	DPEP3	0.573824	0.240186	0.576811	None
9018	SYT5	-0.01636	0.240207	0.576811	None
9019	DNAJC18	0.070883	0.240304	0.57698	None
9020	C10orf62	0.033183	0.240333	0.576987	None
9021	SENCR	-0.10717	0.24047	0.577251	None
9022	MUM1	-0.17023	0.240521	0.577282	None
9023	HNRNPF	0.167742	0.240563	0.577282	None
9024	RTN4R	-0.16813	0.240563	0.577282	None
9025	MLH3	-0.22652	0.240727	0.577613	None
9026	LOC10192	0.025345	0.240889	0.577935	None
9027	HS2ST1	0.162975	0.240978	0.578087	None
9028	PTHLH	0.002081	0.241082	0.57827	None
9029	PTP4A2	-0.12882	0.241143	0.578336	None
9030	PDIA6	0.175935	0.241209	0.578336	None
9031	PC	-0.09613	0.241214	0.578336	None
9032	ABHD15	0.324057	0.241216	0.578336	None
9033	BCHE	-0.28942	0.241341	0.578572	None
9034	SLC35G2	-0.22158	0.241377	0.578593	None
9035	EPC1	0.153538	0.241494	0.578749	None
9036	RP11-108F	0.047696	0.241495	0.578749	None
9037	BMP8B	-0.0293	0.241575	0.578877	None
9038	STXBP3	0.219307	0.241692	0.579093	None
9039	TMEM234	-0.09515	0.241769	0.579214	None
9040	CTC-462L7	0.049876	0.241815	0.579245	None
9041	MDH1B	-0.03063	0.241847	0.579245	None
9042	ALDH6A1	-0.17669	0.241887	0.579245	None
9043	VPS28	-0.15864	0.241889	0.579245	None
9044	LOC10028	-0.22718	0.241923	0.579262	None
9045	C20orf196	-0.15469	0.24206	0.579432	None
9046	BRI3	-0.20509	0.242066	0.579432	None

9047	SEMA3C	0.259196	0.2421	0.579432	None
9048	NPTN	-0.19026	0.242101	0.579432	None
9049	EMX2OS	-0.02157	0.24214	0.579437	None
9050	NCKAP5L	-0.05138	0.242157	0.579437	None
9051	BHMT	0.031034	0.242245	0.579585	None
9052	LOC10049!	0.075116	0.242298	0.579647	None
9053	UBQLNL	-0.08621	0.242357	0.57971	None
9054	DCN	0.160526	0.242378	0.57971	None
9055	KIAA1656	-0.06177	0.242449	0.579817	None
9056	SUSD4	0.035503	0.242491	0.579853	None
9057	ZBTB20-AS	0.012023	0.242544	0.579916	None
9058	HSPA4	-0.21479	0.242632	0.58006	None
9059	NLRC5	0.220265	0.242671	0.580091	None
9060	NUMB	0.229546	0.24277	0.580264	None
9061	LOC10106(	-0.0326	0.242914	0.580544	None
9062	MSX1	0.069291	0.242986	0.580651	None
9063	WFDC2	0.012795	0.243022	0.580653	None
9064	RP1-68D1{	0.046097	0.24306	0.580653	None
9065	PRSS30P	-0.03837	0.243067	0.580653	None
9066	CTSK	0.266486	0.243105	0.58068	None
9067	BC027448	0.076869	0.243205	0.580855	None
9068	LOC72916	0.074046	0.243307	0.580998	None
9069	SOS2	0.090757	0.243319	0.580998	None
9070	TMED5	0.158922	0.243518	0.58141	None
9071	DHRS2	-0.02298	0.243661	0.581679	None
9072	SLC16A1	-0.24946	0.243685	0.581679	None
9073	ANKRD33	-0.02406	0.243725	0.581711	None
9074	MGST1	-0.52398	0.243815	0.581861	None
9075	RGL1	-0.71488	0.24395	0.582074	None
9076	CDC45	0.282181	0.243958	0.582074	None
9077	ADAL	-0.12182	0.244133	0.582417	None
9078	RNF170	-0.19161	0.244172	0.582417	None
9079	SEC61A1	-0.1586	0.244182	0.582417	None
9080	DEGS1	0.134201	0.244291	0.582612	None
9081	KIN	0.193641	0.244342	0.582671	None
9082	PXN-AS1	-0.17851	0.244447	0.582856	None
9083	LOC10192	0.010179	0.244578	0.583037	None
9084	GLDN	-0.1983	0.244592	0.583037	None
9085	TMEM229E	0.027571	0.244614	0.583037	None
9086	BHLHE41	-0.05213	0.244631	0.583037	None
9087	HPS6	-0.06401	0.244682	0.583095	None
9088	MYO15B	-0.27718	0.244746	0.583171	None
9089	PLIN5	0.015645	0.244862	0.583171	None
9090	LRRC20	-0.13573	0.244878	0.583171	None
9091	TRABD2A	0.033547	0.244931	0.583171	None
9092	CENPM	0.262158	0.244935	0.583171	None
9093	CDK18	-0.05287	0.245018	0.583171	None
9094	KIAA1211L	-0.10722	0.245031	0.583171	None
9095	ZNF488	-0.0427	0.24507	0.583171	None
9096	DKFZP434I	-0.01799	0.245085	0.583171	None
9097	H2AFY	-0.30051	0.245105	0.583171	None
9098	PLEKHN1	0.052454	0.245114	0.583171	None
9099	RP11-166F	0.07893	0.245117	0.583171	None
9100	PHF3	0.154117	0.24516	0.583171	None
9101	PTGDR2	-0.0778	0.245161	0.583171	None
9102	PTGS1	-0.39871	0.245188	0.583171	None
9103	CHST2	-0.19377	0.245225	0.583171	None
9104	MOB1B	0.207121	0.24525	0.583171	None

9105	GAPT	-0.34417	0.245261	0.583171	None
9106	LINC00337	0.036139	0.245284	0.583171	None
9107	CTB-12A17	-0.17414	0.245294	0.583171	None
9108	LOC728690	0.020496	0.245303	0.583171	None
9109	AVP	0.131081	0.245334	0.583171	None
9110	CAMKV	-0.02274	0.245353	0.583171	None
9111	UNC45A	-0.09269	0.24536	0.583171	None
9112	VEZT	0.150961	0.245431	0.583236	None
9113	GNB4	0.205621	0.245441	0.583236	None
9114	C6orf141	-0.23432	0.245527	0.583376	None
9115	HNRNPL	-0.29194	0.245557	0.583382	None
9116	DEPDC7	-0.35771	0.245687	0.583629	None
9117	KMT2A	-0.25548	0.245762	0.583742	None
9118	GPR34	-0.61905	0.245937	0.584093	None
9119	POLA2	0.175156	0.246023	0.584235	None
9120	KIAA0100	-0.1632	0.246058	0.584252	None
9121	RP11-215E	0.048004	0.246198	0.584485	None
9122	DSPP	0.098583	0.24621	0.584485	None
9123	PADI3	0.017649	0.246242	0.584496	None
9124	GFPT1	-0.18123	0.246286	0.584537	None
9125	EIF4E	0.234727	0.24635	0.584537	None
9126	FAM19A2	0.449388	0.246413	0.584537	None
9127	TRY2P	0.009748	0.246415	0.584537	None
9128	THEM4	-0.2541	0.246445	0.584537	None
9129	LOC100505	-0.194	0.246446	0.584537	None
9130	ABCA2	-0.01808	0.246495	0.584537	None
9131	ZNF524	-0.02568	0.246498	0.584537	None
9132	DEPDC1-A	-0.0375	0.246502	0.584537	None
9133	SLC25A25	-0.16283	0.246537	0.584557	None
9134	KIAA0556	-0.14364	0.246651	0.584764	None
9135	HECTD4	0.092567	0.246872	0.585188	None
9136	ARSG	-0.09585	0.246884	0.585188	None
9137	LRGUK	0.025492	0.246923	0.585215	None
9138	HOTAIRM1	-0.15325	0.247001	0.585336	None
9139	TRIM33	-0.12043	0.247044	0.585374	None
9140	ZNF592	-0.16959	0.247111	0.585469	None
9141	KATNB1	-0.20908	0.247145	0.585487	None
9142	ARHGEF11	-0.01773	0.247356	0.585921	None
9143	CRABP1	-0.04808	0.247421	0.586012	None
9144	TMEM63C	-0.03572	0.247616	0.586407	None
9145	FLVCR1	0.175537	0.247642	0.586407	None
9146	CD40LG	-0.11016	0.247727	0.586543	None
9147	LTBR	-0.03386	0.247756	0.586549	None
9148	CCNO	0.014011	0.24791	0.586828	None
9149	ZNF420	-0.1923	0.247958	0.586828	None
9150	PNMAL2	-0.10617	0.247982	0.586828	None
9151	UGGT1	-0.23209	0.248004	0.586828	None
9152	C9orf135	-0.00498	0.24801	0.586828	None
9153	NUP54	0.305342	0.248114	0.586956	None
9154	C5orf66	0.10233	0.248118	0.586956	None
9155	PRAME	-0.30003	0.248172	0.586968	None
9156	LOC100240	0.021036	0.248177	0.586968	None
9157	NUDT21	0.181997	0.248225	0.587016	None
9158	CDO1	-0.19504	0.248268	0.587055	None
9159	MRPL17	-0.23854	0.248334	0.587147	None
9160	EI24	-0.12164	0.248502	0.587479	None
9161	ADM5	-0.13513	0.248591	0.587626	None
9162	PEX16	-0.20226	0.248667	0.58774	None

9163	KIAA1024	-0.07931	0.248711	0.587779	None
9164	PAFAH2	-0.0716	0.248737	0.587779	None
9165	LCN12	0.04805	0.24882	0.587885	None
9166	CNOT6L	-0.11294	0.24888	0.587885	None
9167	SULT1C2	-0.17998	0.248928	0.587885	None
9168	PABPC1L	-0.22782	0.248949	0.587885	None
9169	C6orf47	-0.13372	0.248949	0.587885	None
9170	FRA10AC1	-0.15114	0.248958	0.587885	None
9171	ZNF75D	-0.12739	0.248972	0.587885	None
9172	LOC10050	-0.04996	0.249141	0.588169	None
9173	SLCO3A1	-0.18645	0.249161	0.588169	None
9174	SSR2	0.135705	0.249174	0.588169	None
9175	TUBB1	-0.5486	0.249252	0.588288	None
9176	RPIA	-0.12604	0.249323	0.588392	None
9177	PAGE5	-0.0299	0.249363	0.588422	None
9178	TTC8	-0.22254	0.249453	0.588571	None
9179	RP11-295F	0.072185	0.249491	0.588597	None
9180	NPHS1	-0.01388	0.249626	0.588851	None
9181	NUP107	-0.16439	0.249725	0.58902	None
9182	TUBA4B	0.127724	0.249759	0.58903	None
9183	DDC	0.021205	0.249783	0.58903	None
9184	RP11-63D	0.035607	0.24988	0.589095	None
9185	INPP5F	-0.11137	0.249881	0.589095	None
9186	LTK	-0.09466	0.249907	0.589095	None
9187	ZMYND11	-0.16376	0.249925	0.589095	None
9188	A4GALT	-0.06702	0.249947	0.589095	None
9189	PRDM10	-0.07236	0.249999	0.589115	None
9190	SLAMF7	-0.23563	0.25001	0.589115	None
9191	WDFY3-AS	-0.1643	0.250082	0.58917	None
9192	SLC13A1	0.003579	0.250093	0.58917	None
9193	RP11-348N	-0.06503	0.250125	0.58917	None
9194	SMYD3	0.194111	0.250142	0.58917	None
9195	RRN3P3	0.02816	0.250347	0.589588	None
9196	EML4	0.112745	0.250426	0.58965	None
9197	SNCB	0.034443	0.250428	0.58965	None
9198	RP1-39J2.1	0.006103	0.250531	0.589829	None
9199	CNFN	-0.06856	0.250638	0.590016	None
9200	FBXL20	-0.17663	0.25082	0.590353	None
9201	C2orf73	0.029276	0.250843	0.590353	None
9202	ARHGEF16	0.031911	0.250863	0.590353	None
9203	RP11-732A	-0.09624	0.250949	0.590473	None
9204	TMEM105	-0.03374	0.250968	0.590473	None
9205	ACER1	-0.01895	0.251113	0.590682	None
9206	DHX15	0.106434	0.251135	0.590682	None
9207	RP11-700H	-0.01913	0.251139	0.590682	None
9208	ZNF260	-0.17451	0.251244	0.590864	None
9209	CDC42BPA	-0.28612	0.251299	0.590931	None
9210	KIF4A	0.318244	0.251359	0.591009	None
9211	DST	-0.05625	0.251427	0.591104	None
9212	RPS4Y1	-1.52691	0.251562	0.591305	None
9213	PNCK	0.051591	0.251567	0.591305	None
9214	PRR29	0.067306	0.251717	0.591592	None
9215	RPL19	-0.12479	0.25181	0.591747	None
9216	SMTNL1	0.027393	0.25194	0.591988	None
9217	SMIM24	0.291848	0.252016	0.592103	None
9218	YKT6	-0.20772	0.252071	0.592166	None
9219	EMC2	0.231683	0.252234	0.592486	None
9220	CKAP2L	0.380177	0.252284	0.592539	None

9221	SH3RF3-A!	0.010733	0.25247	0.592793	None
9222	SNTG2	-0.01329	0.252473	0.592793	None
9223	SNAPC2	-0.06353	0.252477	0.592793	None
9224	CD163	-0.90007	0.252501	0.592793	None
9225	SLC16A1-/	-0.0496	0.252647	0.593007	None
9226	TUSC2	-0.15872	0.252647	0.593007	None
9227	ZBTB7A	-0.04887	0.252775	0.593189	None
9228	CAMKK2	-0.14725	0.252807	0.593189	None
9229	RP3-507I1!	-0.1213	0.252807	0.593189	None
9230	SRPK3	-0.0331	0.252902	0.593346	None
9231	LOC28636	0.208175	0.252949	0.593393	None
9232	FGFR1	0.136582	0.253019	0.593465	None
9233	BCL10	0.325485	0.253035	0.593465	None
9234	SLC2A13	-0.03415	0.253196	0.59378	None
9235	RFPL3	0.036187	0.253279	0.593878	None
9236	PPP1R8	-0.12854	0.253312	0.593878	None
9237	PRR11	0.145187	0.25332	0.593878	None
9238	LOC38983	-0.18879	0.253351	0.593887	None
9239	FABP5	-0.20167	0.253446	0.594044	None
9240	CXCL14	0.041969	0.253482	0.594064	None
9241	RCOR3	-0.15403	0.253535	0.594125	None
9242	SIGLEC9	-0.21298	0.253664	0.594355	None
9243	AQR	-0.17815	0.253688	0.594355	None
9244	PLEKHA2	0.385154	0.25378	0.594487	None
9245	RP11-1277	-0.10545	0.2538	0.594487	None
9246	KIR2DL5A	0.070548	0.253972	0.594815	None
9247	SLC25A24	0.09323	0.253995	0.594815	None
9248	GLIS3	-0.08852	0.254061	0.594907	None
9249	ATXN7L3B	-0.19628	0.254102	0.594937	None
9250	RBM3	0.165383	0.254141	0.594964	None
9251	C17orf70	0.064035	0.254444	0.59561	None
9252	RAD51-AS	-0.15828	0.254509	0.595639	None
9253	FAM222A	-0.02686	0.254514	0.595639	None
9254	BARX2	0.080353	0.254539	0.595639	None
9255	KIAA0930	-0.18103	0.254581	0.595671	None
9256	SART3	-0.1407	0.254608	0.595671	None
9257	MRPL30	-0.1285	0.254659	0.595697	None
9258	CPNE5	-0.20475	0.25469	0.595697	None
9259	SIDT2	-0.17133	0.254701	0.595697	None
9260	DARS	-0.17264	0.254778	0.595813	None
9261	RP4-539M	-0.00633	0.254913	0.596063	None
9262	PNRC2	-0.11158	0.255044	0.596305	None
9263	MACROD2	0.080262	0.255177	0.596505	None
9264	P2RY12	-0.36061	0.2552	0.596505	None
9265	CHI3L1	-0.28815	0.255212	0.596505	None
9266	CIART	-0.07622	0.255311	0.59664	None
9267	TMEM221	-0.03803	0.255325	0.59664	None
9268	NMRAL1	-0.14508	0.255358	0.59664	None
9269	MACF1	-0.19719	0.255462	0.59664	None
9270	TNFRSF13I	-0.08695	0.255473	0.59664	None
9271	KCNA2	-0.01437	0.255486	0.59664	None
9272	RPL10A	0.059703	0.255507	0.59664	None
9273	SIRT3	-0.05762	0.255521	0.59664	None
9274	TRAPPC2	-0.07279	0.255533	0.59664	None
9275	N4BP2L2-I	-0.12936	0.255579	0.59664	None
9276	TMEM186	-0.21109	0.255591	0.59664	None
9277	CYC1	-0.19413	0.255625	0.59664	None
9278	TM4SF1	-0.47052	0.255657	0.59664	None

9279	ARHGAP2(	-0.04911	0.255678	0.59664	None
9280	LINGO2	-0.25929	0.255683	0.59664	None
9281	RP1-149C7	0.007351	0.255769	0.596742	None
9282	ABCC6P1	-0.05267	0.255782	0.596742	None
9283	WSB1	0.342166	0.255923	0.596913	None
9284	FHL1	0.227759	0.255932	0.596913	None
9285	CHMP2B	-0.11604	0.255938	0.596913	None
9286	UBAP1	-0.14244	0.256188	0.597432	None
9287	ZNF277	0.170228	0.256271	0.597561	None
9288	SLC4A8	-0.11623	0.256336	0.597649	None
9289	RP1-151F1	-0.13777	0.256403	0.597697	None
9290	PRDM16	-0.28516	0.256412	0.597697	None
9291	MS4A4A	-0.55519	0.256511	0.597807	None
9292	GPRC5C	-0.07365	0.256514	0.597807	None
9293	SKIV2L	0.154661	0.256555	0.597837	None
9294	DNTTIP2	0.115751	0.256736	0.598187	None
9295	FAF2	-0.12251	0.256764	0.598187	None
9296	TPSB2	-0.38085	0.256788	0.598187	None
9297	LOC10192	0.032848	0.256853	0.598273	None
9298	LRRC6	-0.15343	0.256891	0.598299	None
9299	ZNF37BP	0.21438	0.256953	0.598378	None
9300	ARL9	0.313472	0.257104	0.598666	None
9301	RP11-402D	-0.03038	0.257426	0.599351	None
9302	KLHDC7A	-0.01526	0.257502	0.599463	None
9303	LMAN2	-0.08294	0.257572	0.599562	None
9304	C1orf115	-0.1071	0.257897	0.600154	None
9305	SRSF1	0.144253	0.257907	0.600154	None
9306	BRICD5	-0.03549	0.25791	0.600154	None
9307	LOC10050	-0.05567	0.257949	0.600181	None
9308	ZBTB44	-0.21489	0.258042	0.600296	None
9309	SLC35F3	-0.06069	0.258054	0.600296	None
9310	LOC10050(	0.004239	0.258223	0.600624	None
9311	LPP-AS2	-0.01066	0.258278	0.600688	None
9312	LINC01355	-0.12214	0.258331	0.600748	None
9313	RAD51B	0.072726	0.258372	0.600778	None
9314	RPL3	0.071586	0.258498	0.601008	None
9315	DCAF11	-0.1301	0.258649	0.601235	None
9316	B3GALNT1	-0.27502	0.258656	0.601235	None
9317	RP11-164F	0.015428	0.25868	0.601235	None
9318	PRKCDBP	-0.1001	0.258726	0.601252	None
9319	NDC80	0.26223	0.258742	0.601252	None
9320	LINC01449	-0.01739	0.258804	0.601331	None
9321	ALDH1A3	-0.1563	0.258884	0.6014	None
9322	PCDHB8	0.007964	0.258889	0.6014	None
9323	DNER	0.006882	0.258989	0.601521	None
9324	CPAMD8	-0.10656	0.259029	0.601521	None
9325	SDPR	-0.33721	0.259068	0.601521	None
9326	LOC10192	0.005794	0.259072	0.601521	None
9327	C19orf12	-0.19584	0.259081	0.601521	None
9328	TPTE2P5	-0.06597	0.259141	0.601598	None
9329	LLGL2	-0.05879	0.259307	0.601919	None
9330	CTB-102L5	-0.04332	0.259613	0.602563	None
9331	SENP6	0.175451	0.259673	0.602597	None
9332	LEPRE1	-0.14169	0.259692	0.602597	None
9333	LOC40068	-0.04189	0.259711	0.602597	None
9334	SMIM14	-0.30263	0.259831	0.602812	None
9335	FBXL13	-0.07439	0.259875	0.602849	None
9336	LOC10099(	0.032585	0.259932	0.602879	None

9337	SRRM2-AS	0.034306	0.259964	0.602879	None
9338	CEP78	0.177569	0.259971	0.602879	None
9339	NSFL1C	-0.12177	0.260029	0.602897	None
9340	SULT1B1	-0.20905	0.260035	0.602897	None
9341	CCDC124	-0.07673	0.260164	0.603047	None
9342	ATP11A	-0.02713	0.26018	0.603047	None
9343	LINC01271	0.025897	0.260197	0.603047	None
9344	HP09025	-0.02932	0.260226	0.603047	None
9345	SCAMP4	-0.0641	0.260239	0.603047	None
9346	PON1	0.00942	0.260298	0.60312	None
9347	TMEM104	-0.2271	0.260357	0.603136	None
9348	UPRT	-0.15467	0.260361	0.603136	None
9349	FAXC	-0.09789	0.260488	0.603366	None
9350	FAM216A	-0.16207	0.26055	0.603445	None
9351	DNAJC27-	0.029003	0.260744	0.60383	None
9352	STON1	-0.15234	0.260825	0.603952	None
9353	TMEM259	-0.04049	0.261091	0.604503	None
9354	ZNF710	-0.09943	0.26113	0.60453	None
9355	NFE2L3	-0.23084	0.261246	0.604732	None
9356	ZMAT5	-0.22145	0.261353	0.604868	None
9357	C11orf53	0.068661	0.261387	0.604868	None
9358	SCRN1	-0.29335	0.261388	0.604868	None
9359	USP44	-0.12579	0.26145	0.604906	None
9360	XRCC6BP1	-0.10805	0.26146	0.604906	None
9361	MYLK4	-0.18932	0.261604	0.605174	None
9362	U91328.2	-0.14578	0.261633	0.605176	None
9363	RAB13	-0.17353	0.261698	0.605262	None
9364	MMP16	-0.02419	0.261791	0.605414	None
9365	NPAT	-0.20653	0.261836	0.605452	None
9366	GPR18	0.304841	0.261891	0.605515	None
9367	CDK13	0.195261	0.261956	0.6056	None
9368	PTPMT1	-0.12808	0.262014	0.60567	None
9369	PRDM5	0.022474	0.262086	0.60576	None
9370	RBP4	-0.06542	0.262109	0.60576	None
9371	ACP5	-0.36318	0.262195	0.605865	None
9372	H6PD	0.10064	0.262211	0.605865	None
9373	NDUFV2-A	0.154709	0.262267	0.605932	None
9374	ITGB5	-0.25974	0.26237	0.606105	None
9375	HOXA3	-0.17471	0.262444	0.60621	None
9376	PCDHB7	-0.0214	0.262483	0.606236	None
9377	HK2	-0.15119	0.262559	0.606332	None
9378	GRB10	-0.2185	0.26258	0.606332	None
9379	CASZ1	-0.1004	0.262665	0.606448	None
9380	N4BP2L1	-0.17218	0.262701	0.606448	None
9381	BC039487	-0.03868	0.262737	0.606448	None
9382	DYNC1LI1	-0.07293	0.262743	0.606448	None
9383	LINC00668	0.013952	0.262798	0.606511	None
9384	MGC45800	-0.03082	0.262922	0.606733	None
9385	DDIT4L	-0.16863	0.262994	0.606823	None
9386	RBBP8	0.172943	0.263024	0.606823	None
9387	HOXC13	0.004534	0.26306	0.606823	None
9388	LOC73009	0.044228	0.26308	0.606823	None
9389	LOC64598	-0.13619	0.263102	0.606823	None
9390	MAP2K6	0.092721	0.263137	0.606839	None
9391	GNG8	-0.10897	0.263321	0.607201	None
9392	HOXC4	0.109682	0.263386	0.607286	None
9393	FBLN5	-0.23557	0.263463	0.607398	None
9394	INTS1	-0.08718	0.263592	0.607619	None

9395	PRSS21	0.809994	0.263615	0.607619	None
9396	AC083949.	0.013823	0.263714	0.607782	None
9397	CTAGE1	0.011093	0.26388	0.608003	None
9398	ZNRF2	0.100733	0.263905	0.608003	None
9399	HDGFRP2	-0.11121	0.263918	0.608003	None
9400	GCC2	0.072685	0.263922	0.608003	None
9401	SNORA37	0.022851	0.263981	0.608063	None
9402	FLT3	0.420892	0.26401	0.608063	None
9403	DOLPP1	-0.14094	0.264032	0.608063	None
9404	THRAP3	-0.16762	0.264066	0.608076	None
9405	FSTL3	-0.04787	0.264288	0.608496	None
9406	ZIK1	0.187165	0.26434	0.608496	None
9407	RP11-650K	0.016713	0.26434	0.608496	None
9408	COL9A3	-0.15194	0.264371	0.608496	None
9409	PTPN20B	-0.52545	0.264389	0.608496	None
9410	CEP120	0.125763	0.264493	0.608655	None
9411	TTLL7	-0.18174	0.264514	0.608655	None
9412	TSSK1B	0.009424	0.264684	0.608982	None
9413	UBB	0.054983	0.264739	0.60901	None
9414	RAP1GDS1	0.207537	0.264753	0.60901	None
9415	MVB12A	-0.14539	0.26489	0.609235	None
9416	MBNL2	-0.11785	0.264907	0.609235	None
9417	CDCA7	0.231441	0.264987	0.609341	None
9418	TBRG1	-0.17027	0.265009	0.609341	None
9419	NBPF4	-0.13196	0.265112	0.609513	None
9420	CERS6	0.16645	0.265183	0.609611	None
9421	SCARA3	-0.05794	0.265234	0.60963	None
9422	A4GNT	0.003651	0.265247	0.60963	None
9423	IL20RB	0.087566	0.265326	0.609684	None
9424	ABHD17B	0.216192	0.265327	0.609684	None
9425	PTS	0.183559	0.265402	0.60979	None
9426	PRPF4B	0.192531	0.265458	0.609855	None
9427	LOC10192	-0.02252	0.265501	0.609888	None
9428	GUCA2A	-0.07431	0.265675	0.610145	None
9429	FGL1	0.02929	0.265676	0.610145	None
9430	TNFSF13B	-0.26039	0.265697	0.610145	None
9431	PLEK2	-0.33271	0.265726	0.610148	None
9432	AQP9	0.474142	0.265833	0.610328	None
9433	ZNF146	-0.15586	0.265881	0.610332	None
9434	GPR85	-0.08691	0.265908	0.610332	None
9435	CTD-3028I	-0.05543	0.265919	0.610332	None
9436	FMNL2	-0.25916	0.266076	0.610574	None
9437	CCL5	-0.67829	0.266081	0.610574	None
9438	KCNJ2	-0.41531	0.266131	0.610587	None
9439	KIAA1919	-0.09874	0.266143	0.610587	None
9440	HCCS	-0.16361	0.266207	0.610615	None
9441	SLMO2	0.210737	0.266212	0.610615	None
9442	ENPP6	-0.14435	0.266327	0.610804	None
9443	SERHL2	0.113783	0.266351	0.610804	None
9444	CDK12	-0.11208	0.266446	0.610958	None
9445	LOC37444	-0.19926	0.266515	0.611042	None
9446	LOC10192	-0.06564	0.266558	0.611042	None
9447	TEPP	-0.0371	0.266592	0.611042	None
9448	DRD5	0.008604	0.266596	0.611042	None
9449	CCR1	-0.55586	0.266651	0.611074	None
9450	TMEM127	-0.14442	0.266666	0.611074	None
9451	ZNF446	-0.04486	0.266833	0.611392	None
9452	ABCB4	-0.10855	0.266912	0.611508	None

9453	HAO2	0.014512	0.266969	0.61154	None
9454	RNF7	0.127899	0.266982	0.61154	None
9455	GS1-304P7	0.00841	0.267038	0.611603	None
9456	PPP1R35	-0.15824	0.267139	0.61177	None
9457	PSD	-0.04278	0.26726	0.611982	None
9458	SPICE1	-0.08391	0.267346	0.612114	None
9459	C6orf165	-0.04623	0.267386	0.612142	None
9460	MYO9A	-0.15091	0.26743	0.612167	None
9461	TGM5	-0.06233	0.267454	0.612167	None
9462	ZNF257	0.25017	0.267493	0.612192	None
9463	TELO2	-0.13656	0.267584	0.612335	None
9464	P2RX5	-0.42042	0.267657	0.612391	None
9465	HBP1	0.350026	0.267665	0.612391	None
9466	LOC10028	-0.15378	0.267721	0.612399	None
9467	ALKBH2	-0.21052	0.267725	0.612399	None
9468	AKT2	-0.09327	0.267792	0.612425	None
9469	TVP23A	-0.14775	0.267793	0.612425	None
9470	MIR10A	-0.29912	0.267861	0.612485	None
9471	DHRS7B	-0.18487	0.267876	0.612485	None
9472	DNAH11	-0.00465	0.26812	0.612979	None
9473	SNX10	-0.26521	0.268194	0.613056	None
9474	GJB6	0.176156	0.26821	0.613056	None
9475	LOC10106	-0.10316	0.268253	0.613089	None
9476	ATAD5	0.211931	0.268304	0.613096	None
9477	SCGB2B2	0.083099	0.268335	0.613096	None
9478	C19orf73	-0.01824	0.268341	0.613096	None
9479	SLC39A8	-0.06676	0.268452	0.613285	None
9480	LOC10050	0.014613	0.268504	0.61334	None
9481	PDE7A	-0.19802	0.268637	0.613573	None
9482	LOC20202	0.23314	0.268663	0.613573	None
9483	NSA2	-0.11192	0.268737	0.613622	None
9484	DPH2	-0.15769	0.268791	0.613622	None
9485	MFSD6L	0.03057	0.268814	0.613622	None
9486	KIF5B	0.11482	0.268834	0.613622	None
9487	TLK1	-0.14021	0.268834	0.613622	None
9488	MRPL47	0.078042	0.268855	0.613622	None
9489	LOC10272	0.024429	0.268951	0.613704	None
9490	HOXA5	-0.33578	0.268981	0.613704	None
9491	RP11-28F1	0.35633	0.268997	0.613704	None
9492	LOC10192	-0.02853	0.269004	0.613704	None
9493	UGT2B28	0.090264	0.269125	0.613916	None
9494	EPS15	-0.10745	0.269184	0.613972	None
9495	AUNIP	-0.22767	0.269234	0.613972	None
9496	SELENBP1	-0.64707	0.269235	0.613972	None
9497	SNRPG	0.122908	0.269376	0.614178	None
9498	DKFZP761	-0.02332	0.269382	0.614178	None
9499	EVI2A	-0.22246	0.269417	0.614193	None
9500	RFPL3S	-0.13645	0.269507	0.614275	None
9501	DDB2	-0.18224	0.269509	0.614275	None
9502	MAFB	-0.65615	0.269784	0.614691	None
9503	VPS13C	0.22793	0.269817	0.614691	None
9504	TMEM167E	-0.12242	0.269823	0.614691	None
9505	HS3ST1	0.100775	0.269853	0.614691	None
9506	DANCR	-0.26767	0.269853	0.614691	None
9507	TBXA2R	-0.1763	0.269862	0.614691	None
9508	IFNW1	-0.02991	0.270169	0.615229	None
9509	PSRC1	-0.2641	0.270187	0.615229	None
9510	SUV420H1	-0.14503	0.270198	0.615229	None

9511	LOH12CR1	0.166034	0.270212	0.615229	None
9512	ACVR2A	-0.14575	0.270297	0.615358	None
9513	GRK5	0.036693	0.270335	0.61538	None
9514	MESDC1	0.21889	0.270427	0.615513	None
9515	VPS39	-0.13428	0.270451	0.615513	None
9516	TUSC3	-0.09384	0.270543	0.615604	None
9517	RHOD	-0.00858	0.270606	0.615604	None
9518	CTD-3126I	0.037785	0.270629	0.615604	None
9519	TUBB2B	-0.0228	0.27063	0.615604	None
9520	FCRL5	-0.10897	0.270644	0.615604	None
9521	RIIAD1	-0.02749	0.270661	0.615604	None
9522	ITPKA	-0.13914	0.270704	0.615636	None
9523	GDPD2	0.034311	0.270734	0.61564	None
9524	ASXL2	-0.22484	0.270788	0.615698	None
9525	CHGB	-0.07632	0.270984	0.61608	None
9526	SCUBE1	-0.08744	0.271033	0.616127	None
9527	MZT1	-0.08958	0.271093	0.616197	None
9528	PCDH8	0.203222	0.271145	0.616253	None
9529	MRVI1	-0.24919	0.271212	0.616338	None
9530	BLMH	0.229023	0.271381	0.616546	None
9531	CDK14	0.08512	0.271384	0.616546	None
9532	TNFRSF10I	-0.06892	0.271415	0.616546	None
9533	ATG4B	0.126218	0.271419	0.616546	None
9534	DHFR	0.203691	0.271445	0.616546	None
9535	LOC14584!	0.029162	0.271568	0.616761	None
9536	PHEX	-0.04931	0.271675	0.616849	None
9537	RP11-498I	-0.15529	0.271684	0.616849	None
9538	C19orf44	-0.04675	0.271693	0.616849	None
9539	SPTBN4	-0.04287	0.271738	0.616856	None
9540	LARP6	-0.09789	0.271753	0.616856	None
9541	ADAMTS6	-0.10322	0.271836	0.616921	None
9542	OR7A10	0.06236	0.271889	0.616921	None
9543	LUC7L3	0.155859	0.27192	0.616921	None
9544	LOC10050(	0.053077	0.27192	0.616921	None
9545	RPS12	0.032017	0.271967	0.616921	None
9546	SUV39H1	-0.18309	0.271979	0.616921	None
9547	NELFCD	-0.18728	0.271981	0.616921	None
9548	AX748273	-0.08741	0.272016	0.616936	None
9549	GMFB	0.158076	0.27214	0.617153	None
9550	ZNF527	0.060954	0.272278	0.617359	None
9551	DDX56	-0.12526	0.272288	0.617359	None
9552	AIFM2	-0.0837	0.27241	0.617572	None
9553	ARF5	-0.2736	0.27246	0.61762	None
9554	OR7E12P	-0.10228	0.272544	0.61768	None
9555	SLCO2A1	0.085955	0.272547	0.61768	None
9556	RBAKDN	-0.08276	0.272572	0.61768	None
9557	ATG14	-0.14677	0.272753	0.618024	None
9558	TRMU	-0.13045	0.272804	0.618067	None
9559	PDAP1	-0.12009	0.272853	0.618067	None
9560	LOC10272‹	-0.27177	0.272857	0.618067	None
9561	SUB1	0.123992	0.272901	0.618101	None
9562	FAM83C	-0.02633	0.272959	0.618169	None
9563	LOC10013:	-0.22059	0.273131	0.618484	None
9564	CHEK1	0.243048	0.273214	0.618484	None
9565	GAS2L3	-0.18348	0.273235	0.618484	None
9566	GADL1	0.007884	0.273239	0.618484	None
9567	PHF14	0.115235	0.273241	0.618484	None
9568	AGAP9	-0.16111	0.273306	0.618565	None

9569	PCA3	0.015645	0.273372	0.618652	None
9570	PEG3-AS1	-0.17707	0.273442	0.618744	None
9571	COQ4	-0.14476	0.273564	0.618957	None
9572	HAGH	-0.26049	0.273701	0.6191	None
9573	CDH15	-0.02569	0.27375	0.6191	None
9574	IGSF1	0.024558	0.273763	0.6191	None
9575	IFT80	-0.10945	0.273789	0.6191	None
9576	HECW2	0.147705	0.27384	0.6191	None
9577	SELK	0.146898	0.273849	0.6191	None
9578	UBL7-AS1	-0.12725	0.273856	0.6191	None
9579	RP11-297L	0.059785	0.273856	0.6191	None
9580	ZDHHC20	0.097654	0.273921	0.619181	None
9581	SPAG11A	-0.04387	0.274024	0.619349	None
9582	RBPJL	-0.01532	0.274138	0.619542	None
9583	OR2C3	0.021632	0.274198	0.619578	None
9584	ZNF503-AS	-0.06206	0.274234	0.619578	None
9585	MAPRE3	0.020996	0.27424	0.619578	None
9586	PPP1R15B	0.17402	0.274342	0.619745	None
9587	COQ2	-0.11568	0.274396	0.619776	None
9588	GRTP1	-0.314	0.274421	0.619776	None
9589	C1RL-AS1	-0.06209	0.274442	0.619776	None
9590	SERGEF	-0.18227	0.27459	0.620046	None
9591	LINC00320	0.007845	0.274698	0.620226	None
9592	LINC01018	0.013876	0.274727	0.620226	None
9593	ZNF252P-A	0.008011	0.275017	0.620816	None
9594	KIAA1217	-0.01074	0.275264	0.621309	None
9595	LOC54944	-0.03676	0.2753	0.621327	None
9596	CDADC1	0.157832	0.27543	0.621504	None
9597	LOC64707(	0.086588	0.275436	0.621504	None
9598	KIRREL3-A	0.032129	0.275479	0.621535	None
9599	SEL1L3	-0.23445	0.27553	0.621586	None
9600	SERPINE2	0.204261	0.27559	0.621656	None
9601	FAM46A	-0.22683	0.275632	0.621687	None
9602	GCA	-0.21206	0.275682	0.621734	None
9603	C19orf84	0.069017	0.275753	0.62183	None
9604	ZNF227	-0.16159	0.275837	0.62193	None
9605	VCX2	-0.04503	0.275855	0.62193	None
9606	EIF6	0.15833	0.275889	0.621932	None
9607	MAEL	-0.08585	0.275913	0.621932	None
9608	MEF2D	-0.13011	0.275982	0.622023	None
9609	CDKN1B	0.2036	0.276028	0.622062	None
9610	BUD13	-0.16311	0.276182	0.622343	None
9611	LURAP1L	-0.13737	0.276575	0.622907	None
9612	SLC25A22	-0.11283	0.276602	0.622907	None
9613	MTUS2	-0.08683	0.276606	0.622907	None
9614	KLC3	-0.06948	0.276616	0.622907	None
9615	AK097453	0.11358	0.276648	0.622907	None
9616	PELI2	0.100113	0.276656	0.622907	None
9617	DPYS	-0.08712	0.276661	0.622907	None
9618	SSR4P1	-0.01547	0.276679	0.622907	None
9619	HSD3B2	0.005586	0.276691	0.622907	None
9620	ARHGAP33	-0.0478	0.276738	0.622908	None
9621	TLX2	-0.01553	0.276749	0.622908	None
9622	GYPC	-0.3592	0.276794	0.622944	None
9623	DNAH14	-0.02857	0.276861	0.623031	None
9624	HIST1H2AI	-0.37048	0.276927	0.623101	None
9625	CRNKL1	-0.15268	0.276987	0.623101	None
9626	TMEM106A	-0.10138	0.276989	0.623101	None

9627	RP5-1092A	-0.38098	0.277007	0.623101	None
9628	FW339973	-0.0873	0.277103	0.623251	None
9629	CYP2A7P1	-0.0112	0.27714	0.623271	None
9630	NACAD	0.013517	0.277318	0.623569	None
9631	FAM86B3P	-0.03932	0.27733	0.623569	None
9632	MOB3A	-0.14001	0.27739	0.623588	None
9633	RP4-581F1	0.131366	0.27741	0.623588	None
9634	LOC10192	0.054159	0.27744	0.623588	None
9635	SAMM50	-0.08354	0.277454	0.623588	None
9636	ZNF461	-0.01788	0.277534	0.623702	None
9637	TMEM65	-0.14013	0.277643	0.623883	None
9638	LINC00632	0.003065	0.277732	0.624019	None
9639	NEK3	-0.12475	0.27777	0.62404	None
9640	LEPREL2	-0.04596	0.277846	0.624144	None
9641	ACKR2	-0.00742	0.277909	0.624207	None
9642	HTR2A	-0.0634	0.277931	0.624207	None
9643	EVI5	-0.17323	0.277985	0.624263	None
9644	CLEC2L	-0.05581	0.278027	0.624292	None
9645	SERTAD3	-0.18133	0.278083	0.624328	None
9646	FPR3	-0.36031	0.2781	0.624328	None
9647	LINC01315	-0.12648	0.27838	0.62489	None
9648	PRKAA2	-0.06973	0.278488	0.625069	None
9649	PPEF1	0.005648	0.278576	0.6252	None
9650	KLHL25	-0.04018	0.278621	0.625238	None
9651	CEP70	-0.21549	0.278665	0.625271	None
9652	AJUBA	-0.0593	0.278727	0.625346	None
9653	FBP1	-0.355	0.278796	0.62536	None
9654	EIF1AY	-0.97038	0.278815	0.62536	None
9655	BMP1	-0.01867	0.27882	0.62536	None
9656	RRH	0.009109	0.278954	0.625543	None
9657	SPOCK3	-0.27044	0.27896	0.625543	None
9658	HPRT1	-0.05661	0.278998	0.625564	None
9659	PAIP1	0.153166	0.279164	0.625872	None
9660	TTI2	-0.1937	0.279259	0.62602	None
9661	LINC00478	-0.181	0.279292	0.626029	None
9662	ZNF563	-0.10252	0.279358	0.626113	None
9663	HIST1H4E	-0.3036	0.279531	0.626435	None
9664	LOC10050	-0.03098	0.279595	0.626514	None
9665	MIPEPP3	-0.12023	0.27967	0.626618	None
9666	CELA1	-0.04728	0.279878	0.627017	None
9667	KDM5A	-0.12392	0.279988	0.627137	None
9668	RP11-477N	-0.17854	0.280007	0.627137	None
9669	ZNF395	-0.12377	0.280018	0.627137	None
9670	PRKCI	-0.14728	0.280156	0.627382	None
9671	CROCC	-0.06375	0.280389	0.627839	None
9672	TUG1	0.153257	0.280458	0.627894	None
9673	GAS5-AS1	-0.03058	0.280472	0.627894	None
9674	OLFM2	-0.01469	0.280537	0.627963	None
9675	METTL20	-0.0199	0.280582	0.627963	None
9676	AOC4P	0.056985	0.28059	0.627963	None
9677	VPS13B	-0.09425	0.280718	0.628075	None
9678	IRAK4	-0.12126	0.280719	0.628075	None
9679	CDKL1	-0.14922	0.280727	0.628075	None
9680	DQ570096	-0.12579	0.280784	0.628114	None
9681	FAM26E	-0.09577	0.280802	0.628114	None
9682	BTG2	0.207938	0.280896	0.628149	None
9683	C19orf52	-0.12117	0.280911	0.628149	None
9684	ITM2A	-0.2493	0.28092	0.628149	None

9685	HIST1H2BJ	-0.17407	0.280934	0.628149	None
9686	RP4-781K5	-0.07717	0.281048	0.628184	None
9687	KIF21A	-0.34155	0.28105	0.628184	None
9688	RP11-981C	-0.15416	0.281073	0.628184	None
9689	LRR1	0.113465	0.28109	0.628184	None
9690	HIST1H2AI	-0.18012	0.281094	0.628184	None
9691	EIF4A2	-0.01061	0.28123	0.628383	None
9692	WDR27	-0.11937	0.281252	0.628383	None
9693	ATP12A	-0.0109	0.281297	0.628383	None
9694	TNP1	-0.05	0.281322	0.628383	None
9695	BAIAP2L1	-0.04301	0.281341	0.628383	None
9696	CAPN5	0.013625	0.281358	0.628383	None
9697	KIAA0196	0.227163	0.281464	0.628556	None
9698	MCMDC2	-0.0129	0.281603	0.628768	None
9699	CAMSAP2	-0.20384	0.281625	0.628768	None
9700	SNORD114	-0.14817	0.281646	0.628768	None
9701	RP11-227D	-0.01639	0.281687	0.62878	None
9702	USP36	0.154635	0.281709	0.62878	None
9703	RBFOX2	-0.12722	0.281882	0.629099	None
9704	4-Sep	-0.00826	0.281911	0.6291	None
9705	ZNF703	-0.09036	0.282032	0.629305	None
9706	BOD1	-0.14354	0.282143	0.629489	None
9707	PRKCH	0.16051	0.282214	0.629582	None
9708	DMKN	-0.13948	0.282298	0.629661	None
9709	SLC35E1	-0.11894	0.282308	0.629661	None
9710	ST5	-0.11922	0.282389	0.629724	None
9711	STAG3L3	-0.37325	0.282394	0.629724	None
9712	FRMD8P1	-0.03197	0.28247	0.629828	None
9713	LOC10013	-0.18426	0.282537	0.629876	None
9714	NTNG1	-0.01641	0.28255	0.629876	None
9715	IL6R	-0.29322	0.282613	0.629951	None
9716	ARHGEF40	-0.184	0.282736	0.630162	None
9717	PEX11A	-0.10001	0.282796	0.630231	None
9718	CPM	-0.28695	0.282884	0.630338	None
9719	LOC28424	-0.03101	0.282902	0.630338	None
9720	HINT3	-0.22882	0.283029	0.630554	None
9721	TMEM9B	-0.15175	0.283097	0.63064	None
9722	PNOC	-0.21639	0.283137	0.630666	None
9723	LOC10192	-0.01397	0.283196	0.63068	None
9724	PRNP	0.142738	0.283202	0.63068	None
9725	SNRPE	-0.24769	0.283261	0.630748	None
9726	LOC10192	0.005497	0.283362	0.630903	None
9727	SFT2D3	-0.08781	0.283395	0.630903	None
9728	USP35	-0.03593	0.283419	0.630903	None
9729	ANO6	0.092822	0.283472	0.630926	None
9730	ASPSCR1	-0.16508	0.283487	0.630926	None
9731	LIN52	-0.11002	0.283596	0.631104	None
9732	OXA1L	0.146674	0.28365	0.631159	None
9733	SP110	-0.24326	0.283702	0.631185	None
9734	KRT13	-0.20289	0.283739	0.631185	None
9735	TMEM160	-0.28671	0.283749	0.631185	None
9736	LOC64308	-0.04717	0.283901	0.631457	None
9737	PIK3R2	-0.03165	0.283963	0.631458	None
9738	SEPT7P2	0.130294	0.283975	0.631458	None
9739	NAP1L4	-0.09982	0.284032	0.631458	None
9740	HSDL2	-0.14742	0.28404	0.631458	None
9741	HSPA14	0.184139	0.284049	0.631458	None
9742	CDC27	-0.14106	0.284076	0.631458	None

9743	FBXL8	-0.05563	0.284176	0.631615	None
9744	POPDC2	-0.05923	0.284378	0.631849	None
9745	PTBP2	0.145456	0.284387	0.631849	None
9746	NFE2L2	-0.18107	0.284393	0.631849	None
9747	ARIH1	0.195804	0.284398	0.631849	None
9748	CXCL9	-0.44413	0.284486	0.631981	None
9749	FUBP1	-0.16114	0.284616	0.632205	None
9750	RAPGEF2	0.256239	0.284812	0.632576	None
9751	LOC10050!	0.036873	0.28506	0.63306	None
9752	MPI	-0.10794	0.28509	0.633062	None
9753	MUTYH	-0.13701	0.285307	0.633478	None
9754	DMD	-0.02118	0.285346	0.633501	None
9755	NOL7	0.106393	0.285414	0.633542	None
9756	MPC2	-0.15328	0.285444	0.633542	None
9757	PDZRN3	-0.12796	0.28546	0.633542	None
9758	NCKAP5	-0.17691	0.285482	0.633542	None
9759	NAV2-AS2	-0.10723	0.285539	0.633604	None
9760	IFIT2	-0.33673	0.285743	0.633928	None
9761	MMD2	0.02632	0.285749	0.633928	None
9762	RP11-360/	0.029032	0.285803	0.633928	None
9763	SLC6A13	0.078081	0.285834	0.633928	None
9764	KRT20	0.030213	0.285847	0.633928	None
9765	DPY19L2P3	-0.07125	0.28586	0.633928	None
9766	SPRED1	-0.2116	0.286003	0.634107	None
9767	ARAF	0.147382	0.286049	0.634107	None
9768	LOC64518l	0.001566	0.28605	0.634107	None
9769	LGALS8-A!	-0.0211	0.286058	0.634107	None
9770	HIST1H3I	-0.09073	0.286117	0.634173	None
9771	EIF3M	0.207948	0.286184	0.634255	None
9772	RP4-561L2	0.003042	0.286224	0.63428	None
9773	AC083843.	-0.20703	0.286297	0.634284	None
9774	USH1C	0.057213	0.286309	0.634284	None
9775	DOCK5	-0.1858	0.286338	0.634284	None
9776	FUNDC1	-0.1263	0.286343	0.634284	None
9777	PRO1483	-0.15256	0.286392	0.634327	None
9778	CELA2B	-0.01598	0.286474	0.634444	None
9779	RNF20	-0.11431	0.286512	0.634464	None
9780	LOC10192!	0.065682	0.286594	0.634482	None
9781	LINC00626	0.006371	0.286607	0.634482	None
9782	TMEM44	-0.22582	0.286611	0.634482	None
9783	AX747405	0.06139	0.286643	0.634482	None
9784	C12orf76	-0.13626	0.286696	0.634482	None
9785	FGD1	0.014469	0.286712	0.634482	None
9786	TRIM44	-0.12182	0.286725	0.634482	None
9787	SGSH	-0.20412	0.286785	0.634542	None
9788	GUCA1A	0.009012	0.28688	0.634542	None
9789	USP3-AS1	-0.11313	0.286919	0.634542	None
9790	DHX29	0.098374	0.286924	0.634542	None
9791	INSL6	0.002184	0.287101	0.634542	None
9792	SEZ6L	-0.00657	0.287123	0.634542	None
9793	CNOT7	0.121324	0.287146	0.634542	None
9794	EXOC8	0.144045	0.287154	0.634542	None
9795	CBR3-AS1	-0.0173	0.287207	0.634542	None
9796	RARG	-0.02175	0.287208	0.634542	None
9797	HSPB8	-0.03853	0.287223	0.634542	None
9798	RP11-669N	0.005864	0.287266	0.634542	None
9799	ISM2	-0.04371	0.287274	0.634542	None
9800	MRPS16	-0.13046	0.287322	0.634542	None

9801	PFKM	0.284062	0.287348	0.634542	None
9802	DMC1	0.051385	0.287388	0.634542	None
9803	RP13-1032	0.028569	0.287415	0.634542	None
9804	DLEU2	0.241961	0.287439	0.634542	None
9805	ACO2	-0.09633	0.287449	0.634542	None
9806	TRIP6	-0.24509	0.287455	0.634542	None
9807	RCAN2	-0.12002	0.287465	0.634542	None
9808	FCGR2B	-0.30212	0.28747	0.634542	None
9809	NFKBIL1	-0.05141	0.287474	0.634542	None
9810	NOTUM	-0.04529	0.287475	0.634542	None
9811	MALRD1	-0.45439	0.287507	0.634542	None
9812	EHHADH	-0.26884	0.287515	0.634542	None
9813	RAP1GAP2	0.178235	0.287582	0.634627	None
9814	IGDCC3	-0.02351	0.287632	0.63464	None
9815	ARFGAP2	-0.07799	0.287665	0.63464	None
9816	C1orf228	0.074454	0.287676	0.63464	None
9817	MAP1LC3A	-0.02871	0.287823	0.6349	None
9818	DNAJC27	-0.13194	0.287943	0.635016	None
9819	PARM1	-0.10216	0.287961	0.635016	None
9820	U2AF1L4	-0.22029	0.287964	0.635016	None
9821	DMTF1	0.185192	0.288042	0.635091	None
9822	ALDOC	-0.23591	0.288056	0.635091	None
9823	HIST1H2AI	-0.10235	0.28809	0.635101	None
9824	WDR24	-0.08202	0.288159	0.635102	None
9825	LOC10028	-0.19876	0.288171	0.635102	None
9826	TNPO1	-0.11454	0.288179	0.635102	None
9827	RP11-95D1	-0.1232	0.288249	0.635192	None
9828	IMMP2L	0.17293	0.288279	0.635194	None
9829	TP53BP2	-0.18012	0.288344	0.635271	None
9830	PPM1H	-0.13485	0.288406	0.635338	None
9831	CD59	-0.1838	0.288433	0.635338	None
9832	KLLN	0.042171	0.288582	0.635545	None
9833	RP11-676J	-0.02342	0.288585	0.635545	None
9834	RBX1	-0.15728	0.288789	0.635929	None
9835	MEX3C	-0.29099	0.288837	0.63597	None
9836	ZNF677	-0.13	0.288875	0.63599	None
9837	FRMD1	0.037051	0.288911	0.636003	None
9838	RP11-400N	-0.0384	0.289092	0.636338	None
9839	TCEAL7	-0.30326	0.289131	0.636358	None
9840	UBE4B	-0.11727	0.289187	0.636418	None
9841	MAPRE1	0.122866	0.289239	0.636468	None
9842	HMBS	-0.32223	0.289345	0.636636	None
9843	TOB1	0.278448	0.289392	0.636675	None
9844	PMAIP1	0.253019	0.28945	0.636737	None
9845	CMTM4	-0.2354	0.289607	0.637019	None
9846	PADI4	-0.12771	0.289665	0.637067	None
9847	WDR77	-0.16918	0.289714	0.637067	None
9848	LRCH2	0.321181	0.289753	0.637067	None
9849	PAPSS1	-0.11841	0.289756	0.637067	None
9850	ZNF664	0.079275	0.289776	0.637067	None
9851	OSTC	0.110085	0.289929	0.637275	None
9852	RPS6	0.087352	0.28993	0.637275	None
9853	FAM103A1	-0.1336	0.290062	0.6375	None
9854	NAMPT	0.246373	0.290221	0.637786	None
9855	CCRN4L	-0.12726	0.290317	0.637932	None
9856	C2orf83	-0.04517	0.290414	0.638081	None
9857	LOXL1-AS1	-0.30823	0.290473	0.638145	None
9858	STAT2	-0.07936	0.290586	0.638289	None

9859	MGC40069	-0.06871	0.290608	0.638289	None
9860	CYP2B6	0.028619	0.290655	0.638289	None
9861	CD3EAP	-0.19848	0.290678	0.638289	None
9862	CXADR	-0.3357	0.290686	0.638289	None
9863	INTS2	-0.16472	0.290765	0.638336	None
9864	PRSS12	-0.00644	0.29077	0.638336	None
9865	PWAR6	0.338145	0.290809	0.638336	None
9866	SPPL3	-0.11691	0.290839	0.638336	None
9867	TTC23	-0.02285	0.290855	0.638336	None
9868	ZEB1-AS1	-0.25058	0.290905	0.638373	None
9869	FMO3	0.173014	0.290931	0.638373	None
9870	LOC10050	-0.07176	0.291005	0.638434	None
9871	CTTN	-0.21857	0.291017	0.638434	None
9872	GALNT18	-0.05755	0.291236	0.638803	None
9873	NHP2	-0.12185	0.291245	0.638803	None
9874	LACE1	0.02886	0.29137	0.638949	None
9875	BRSK1	-0.02726	0.291381	0.638949	None
9876	RNASEH1-	-0.17902	0.29143	0.638949	None
9877	KIAA1586	0.152414	0.291467	0.638949	None
9878	TATDN3	-0.07274	0.291473	0.638949	None
9879	KLRG1	-0.12853	0.291513	0.638949	None
9880	CREBBP	0.086219	0.291518	0.638949	None
9881	TRIM28	-0.21637	0.291697	0.639277	None
9882	LAGE3	-0.14975	0.291848	0.639544	None
9883	LOC10050	0.084255	0.291932	0.639573	None
9884	ZFP92	0.149206	0.29195	0.639573	None
9885	LINC00652	-0.03525	0.29195	0.639573	None
9886	PRR22	-0.02469	0.292165	0.639978	None
9887	WRB	-0.13703	0.292224	0.640002	None
9888	AK000798	-0.11081	0.292235	0.640002	None
9889	TOP1MT	-0.11709	0.292338	0.640163	None
9890	SCARB2	0.155712	0.29237	0.640169	None
9891	PSMB10	0.201433	0.292483	0.640287	None
9892	MAD2L1BP	-0.16089	0.292489	0.640287	None
9893	BEND3	-0.11891	0.292534	0.640287	None
9894	CPLX1	-0.13261	0.292542	0.640287	None
9895	LOC15354	-0.24798	0.292606	0.640352	None
9896	FASTKD5	-0.11763	0.292631	0.640352	None
9897	LOC79999	-0.02689	0.292805	0.640667	None
9898	HEPH	-0.03118	0.292876	0.640759	None
9899	C19orf57	-0.04389	0.293095	0.641172	None
9900	EED	-0.16561	0.293144	0.641172	None
9901	C17orf58	-0.24895	0.293156	0.641172	None
9902	FAM135A	-0.13894	0.293183	0.641172	None
9903	TATDN2	-0.05414	0.293306	0.641267	None
9904	ZNF552	-0.04509	0.293326	0.641267	None
9905	METTL21A	-0.19351	0.293332	0.641267	None
9906	AK5	-0.23496	0.293345	0.641267	None
9907	CAPN12	-0.07898	0.293386	0.641276	None
9908	PPP1R13B	-0.11045	0.293412	0.641276	None
9909	TAOK1	0.10125	0.293438	0.641276	None
9910	ITPR1	0.194209	0.293478	0.641296	None
9911	ZNF264	-0.33568	0.293507	0.641296	None
9912	AHCY	-0.18136	0.293812	0.641899	None
9913	LOC38989	-0.07068	0.293972	0.642122	None
9914	C14orf159	-0.08872	0.293974	0.642122	None
9915	TIMM13	-0.08378	0.294083	0.642244	None
9916	CCDC57	0.08409	0.294089	0.642244	None

9917	POLN	0.034438	0.294224	0.642413	None
9918	NBPF8	0.023207	0.294255	0.642413	None
9919	UTP6	-0.06338	0.294258	0.642413	None
9920	CTD-3064I	-0.02333	0.294298	0.642413	None
9921	MAPK13	-0.11291	0.294326	0.642413	None
9922	DDX52	-0.10667	0.294368	0.642413	None
9923	LOC10065:	-0.05602	0.294404	0.642413	None
9924	COL2A1	-0.18434	0.29442	0.642413	None
9925	RSPH6A	-0.02248	0.294433	0.642413	None
9926	CPS1	-0.1326	0.294496	0.642485	None
9927	CTD-2619:	0.023464	0.29455	0.642539	None
9928	FAM45A	-0.00995	0.294647	0.642684	None
9929	FAM92A1	-0.27916	0.294765	0.642878	None
9930	PDIA4	-0.18798	0.29483	0.642919	None
9931	MAX	-0.24486	0.294843	0.642919	None
9932	C21orf59	-0.08582	0.295019	0.643215	None
9933	CUEDC2	0.133033	0.295058	0.643215	None
9934	TLDC2	-0.01673	0.295082	0.643215	None
9935	CD180	-0.32628	0.295108	0.643215	None
9936	BC017209	0.0144	0.295144	0.643215	None
9937	DSCR9	-0.03719	0.295157	0.643215	None
9938	MORN5	-0.0162	0.295301	0.64346	None
9939	LATS2	-0.22923	0.295329	0.64346	None
9940	NPPA	0.077514	0.295417	0.643496	None
9941	LOC44152ᵢ	0.133511	0.295419	0.643496	None
9942	ADPRM	-0.18718	0.295448	0.643496	None
9943	CLCNKB	0.00465	0.295483	0.643496	None
9944	SLC41A2	-0.12357	0.295494	0.643496	None
9945	MRPS11	-0.09195	0.295588	0.643637	None
9946	NF2	-0.03756	0.295652	0.643675	None
9947	RP11-305(	-0.38693	0.295665	0.643675	None
9948	ATP5E	-0.11935	0.295981	0.644203	None
9949	DPY19L1	-0.07443	0.296005	0.644203	None
9950	LAMTOR3	-0.12763	0.296027	0.644203	None
9951	GRIN3A	-0.05798	0.296027	0.644203	None
9952	LIF	0.125818	0.296075	0.644244	None
9953	CTSB	-0.37457	0.296181	0.644335	None
9954	CTC-510F1	-0.16002	0.296199	0.644335	None
9955	CSNK2A1	-0.12122	0.296256	0.644335	None
9956	SLC12A7	-0.14089	0.296282	0.644335	None
9957	HOXA7	-0.18431	0.296329	0.644335	None
9958	NLRP7	-0.07796	0.296333	0.644335	None
9959	PHF2P1	-0.01501	0.296353	0.644335	None
9960	LPPR3	-0.11238	0.296355	0.644335	None
9961	ARID3B	-0.16932	0.296413	0.644396	None
9962	HGD	-0.26874	0.296532	0.644534	None
9963	TRIP11	-0.13609	0.296536	0.644534	None
9964	UFSP1	-0.04606	0.296584	0.644572	None
9965	ZNF813	-0.12265	0.296679	0.644715	None
9966	CASP9	0.085567	0.296724	0.644748	None
9967	ZNF641	-0.10208	0.29685	0.644958	None
9968	TPRG1	0.041133	0.296958	0.645127	None
9969	HIST1H1C	0.322068	0.297003	0.645159	None
9970	SPSB3	-0.10401	0.29706	0.645219	None
9971	RP11-953E	0.034221	0.297133	0.645314	None
9972	LOC10012!	-0.03084	0.297322	0.645604	None
9973	ZNF174	-0.09039	0.297336	0.645604	None
9974	C1GALT1	-0.12061	0.29738	0.645604	None

9975	LOC10099(	-0.3222	0.297386	0.645604	None
9976	MINK1	-0.0512	0.297494	0.645774	None
9977	GBP1P1	-0.07569	0.297768	0.646295	None
9978	CDHR1	0.008328	0.297794	0.646295	None
9979	STOX2	-0.24431	0.297876	0.646345	None
9980	LOC15222!	0.032787	0.297877	0.646345	None
9981	CCDC33	-0.02904	0.297982	0.646448	None
9982	CREB3	-0.19527	0.298028	0.646448	None
9983	PET117	0.102039	0.29803	0.646448	None
9984	SLC6A20	0.01938	0.298044	0.646448	None
9985	SNAI3	-0.04945	0.29811	0.646527	None
9986	BEX2	0.33735	0.298206	0.646671	None
9987	CGRRF1	-0.11374	0.298255	0.646689	None
9988	DNAAF2	-0.21539	0.2983	0.646689	None
9989	SHC3	0.026848	0.298304	0.646689	None
9990	EEFSEC	-0.12554	0.29844	0.646918	None
9991	TMEM151/	-0.0219	0.298481	0.646942	None
9992	mir-223	-0.33923	0.298564	0.647057	None
9993	SEZ6L2	-0.06425	0.298615	0.647103	None
9994	ZNF510	0.154292	0.298698	0.64722	None
9995	LOC10192	0.021844	0.298768	0.647307	None
9996	P2RX7	-0.17608	0.29888	0.647485	None
9997	GULP1	-0.12074	0.299007	0.647693	None
9998	BRWD1	0.143338	0.299121	0.647728	None
9999	RP11-356E	-0.04295	0.299123	0.647728	None
10000	ADAMTS14	-0.05931	0.299128	0.647728	None
10001	LOC10192	0.020851	0.299142	0.647728	None
10002	RAB11FIP4	-0.07096	0.299319	0.647952	None
10003	PSPC1	0.156754	0.299322	0.647952	None
10004	DPY19L2P2	-0.25051	0.299361	0.647952	None
10005	HIST1H3F	-0.14845	0.299366	0.647952	None
10006	RTN4RL1	0.045222	0.299455	0.648081	None
10007	SMO	-0.11627	0.299488	0.648082	None
10008	DUSP6	-0.3403	0.299515	0.648082	None
10009	LDHD	-0.01771	0.299579	0.648155	None
10010	DEFA4	0.589309	0.299731	0.648418	None
10011	TMX2	-0.11149	0.299862	0.648638	None
10012	LMBR1	-0.10733	0.299973	0.648813	None
10013	LOC10050(	0.009194	0.300025	0.648861	None
10014	HIST1H2BE	-0.20783	0.300104	0.64891	None
10015	MYO7B	-0.02658	0.300179	0.64891	None
10016	CXorf24	0.189884	0.300196	0.64891	None
10017	TSPO2	-0.35666	0.300198	0.64891	None
10018	HIPK2	-0.14237	0.300222	0.64891	None
10019	PKDCC	0.031986	0.300236	0.64891	None
10020	MPP7	0.115455	0.300258	0.64891	None
10021	GLOD5	-0.06929	0.300347	0.649039	None
10022	MACROD1	-0.10703	0.30045	0.649196	None
10023	C2orf15	0.033981	0.300507	0.649254	None
10024	ASB14	0.039772	0.300656	0.649512	None
10025	LOC10013:	-0.265	0.300722	0.649528	None
10026	CCDC125	-0.14825	0.300723	0.649528	None
10027	IL12B	0.046004	0.300755	0.649531	None
10028	LOC10050(	0.033497	0.300881	0.649739	None
10029	LPA	-0.01573	0.300968	0.649757	None
10030	ANGPTL4	0.053069	0.301016	0.649757	None
10031	RHOQ	-0.2229	0.301054	0.649757	None
10032	SLC15A3	-0.35308	0.301105	0.649757	None

10033	WDR60	-0.08467	0.301129	0.649757	None
10034	RP11-38L1	0.045489	0.30114	0.649757	None
10035	UBE2W	0.164364	0.301148	0.649757	None
10036	BC044614	0.020427	0.301158	0.649757	None
10037	LOC10012!	-0.07578	0.301179	0.649757	None
10038	GHRLOS	0.075168	0.301222	0.649757	None
10039	CTD-2537I	-0.02092	0.301249	0.649757	None
10040	PIGB	-0.12727	0.301249	0.649757	None
10041	ZNF622	-0.13787	0.30131	0.649823	None
10042	LOC44043	-0.12732	0.301413	0.649859	None
10043	GPR157	0.086342	0.301424	0.649859	None
10044	RP11-783K	-0.0796	0.301447	0.649859	None
10045	SRD5A3-A	-0.05161	0.301475	0.649859	None
10046	TOMM40	-0.13273	0.301477	0.649859	None
10047	TMEM139	-0.01349	0.301682	0.650236	None
10048	MGC16275	-0.08088	0.301796	0.650418	None
10049	RASGEF1C	0.017957	0.301837	0.650441	None
10050	E2F4	-0.03127	0.302031	0.650794	None
10051	VRTN	-0.05644	0.302064	0.6508	None
10052	LOC10013(	0.03424	0.302325	0.651235	None
10053	LUC7L	-0.14592	0.302326	0.651235	None
10054	KNSTRN	-0.25831	0.302615	0.651732	None
10055	ARMC2	-0.04079	0.302659	0.651732	None
10056	VSTM2L	-0.04255	0.302662	0.651732	None
10057	DGCR12	0.022993	0.302677	0.651732	None
10058	PTRF	-0.17479	0.302795	0.651806	None
10059	ABI3	-0.202	0.302807	0.651806	None
10060	CACNA2D	-0.10491	0.302869	0.651806	None
10061	CCDC181	-0.1697	0.302898	0.651806	None
10062	CHST10	-0.1257	0.302925	0.651806	None
10063	GATA5	-0.00654	0.302927	0.651806	None
10064	NACA	0.0703	0.302966	0.651806	None
10065	TPH1	0.019458	0.30302	0.651806	None
10066	DUOX1	-0.02564	0.30303	0.651806	None
10067	CRYBB1	-0.13736	0.303032	0.651806	None
10068	GZMH	-0.26439	0.303042	0.651806	None
10069	RPPH1	-0.11844	0.303101	0.651811	None
10070	TPM3	-0.19588	0.303133	0.651811	None
10071	BOD1L1	-0.25481	0.303154	0.651811	None
10072	MS4A6A	-0.56841	0.303169	0.651811	None
10073	LY6K	-0.05696	0.303201	0.651811	None
10074	LOC14583	0.014996	0.303231	0.651811	None
10075	MCRS1	-0.19095	0.303265	0.651811	None
10076	OTUD4	0.109596	0.303286	0.651811	None
10077	DUSP5P1	-0.08901	0.303343	0.651828	None
10078	GPR25	-0.04349	0.3034	0.651828	None
10079	TXNDC16	0.190864	0.303403	0.651828	None
10080	C2orf16	-0.03172	0.303414	0.651828	None
10081	RP11-182J	0.005467	0.303471	0.651886	None
10082	NPY6R	-0.0124	0.303552	0.651995	None
10083	PATZ1	-0.08308	0.303585	0.652002	None
10084	RYBP	0.144279	0.303673	0.652088	None
10085	MRPL2	-0.15758	0.303693	0.652088	None
10086	MROH1	-0.10028	0.30373	0.652088	None
10087	ZNF445	0.078174	0.303768	0.652088	None
10088	MTFP1	-0.24878	0.303776	0.652088	None
10089	C1orf54	-0.34999	0.30383	0.652139	None
10090	EXOC3	-0.12325	0.303889	0.652172	None

10091	FLJ20712	0.034297	0.303905	0.652172	None
10092	BTN2A3P	-0.03385	0.304056	0.652419	None
10093	CYP26A1	0.032407	0.304081	0.652419	None
10094	SDHB	-0.09042	0.304126	0.652427	None
10095	GAPVD1	-0.18002	0.304145	0.652427	None
10096	FZD9	0.037357	0.304181	0.652441	None
10097	RP11-354I	0.001831	0.304242	0.652508	None
10098	MED28	-0.12359	0.304326	0.652623	None
10099	RP11-513M	-0.07012	0.304468	0.652862	None
10100	CLEC11A	-0.45508	0.304613	0.652941	None
10101	ATP5G1	-0.15188	0.30464	0.652941	None
10102	LOC10192	-0.09526	0.304675	0.652941	None
10103	LINC00836	-0.02084	0.304695	0.652941	None
10104	MIR146A	-0.03203	0.30471	0.652941	None
10105	DBN1	0.179953	0.304719	0.652941	None
10106	HMGA2	-0.01088	0.304768	0.652941	None
10107	SFXN5	-0.04584	0.304788	0.652941	None
10108	RAB43	0.072681	0.304805	0.652941	None
10109	RP11-247L	-0.21089	0.304818	0.652941	None
10110	CLDN1	0.018753	0.304866	0.652941	None
10111	ZNF474	0.032127	0.304881	0.652941	None
10112	DDR2	0.075989	0.304937	0.652941	None
10113	IGSF6	-0.51297	0.304941	0.652941	None
10114	NOM1	0.147207	0.304958	0.652941	None
10115	APOA1	-0.02752	0.304987	0.652941	None
10116	GNAZ	-0.3071	0.305166	0.653222	None
10117	UBE2K	0.106578	0.305192	0.653222	None
10118	B2M	-0.13726	0.305209	0.653222	None
10119	INHBA	0.36893	0.305323	0.653402	None
10120	KCNT2	-0.09423	0.305379	0.65345	None
10121	STEAP1B	-0.07994	0.305414	0.65345	None
10122	ZNF29P	-0.00711	0.305436	0.65345	None
10123	AVIL	0.086863	0.305496	0.65346	None
10124	PHC1	-0.13023	0.305524	0.65346	None
10125	PPP3CB	0.103646	0.305531	0.65346	None
10126	MTX1	-0.12597	0.305595	0.65353	None
10127	HARBI1	-0.07788	0.305624	0.65353	None
10128	LOC10050	-0.03954	0.305831	0.653819	None
10129	HYKK	-0.03617	0.305841	0.653819	None
10130	SPATA8	-0.07416	0.305862	0.653819	None
10131	ZNF358	-0.02475	0.305884	0.653819	None
10132	RP3-327A1	-0.21138	0.305919	0.653819	None
10133	FAM71E1	-0.03985	0.30597	0.653819	None
10134	WASL	-0.14028	0.305971	0.653819	None
10135	MMEL1	-0.18157	0.306095	0.654019	None
10136	RAMP2-AS	0.050448	0.306202	0.654184	None
10137	MSS51	-0.11538	0.306239	0.654198	None
10138	ISG15	-0.357	0.306386	0.654448	None
10139	CA10	0.007311	0.306439	0.654495	None
10140	CDY1	0.010496	0.306482	0.654523	None
10141	MRPL34	-0.20173	0.306543	0.654545	None
10142	PMPCA	-0.13337	0.306553	0.654545	None
10143	USP12	0.105779	0.306641	0.654573	None
10144	RPA1	-0.15508	0.30665	0.654573	None
10145	CETP	-0.46564	0.306656	0.654573	None
10146	ZNF566	-0.19024	0.306726	0.654658	None
10147	STARD7-A	-0.08963	0.30691	0.654936	None
10148	LOC10050	-0.01113	0.306961	0.654936	None

10149	LINC00537	-0.03173	0.306977	0.654936	None
10150	WDHD1	0.207786	0.306988	0.654936	None
10151	ODF2	-0.14622	0.307008	0.654936	None
10152	UBE2S	-0.35737	0.307086	0.654997	None
10153	ONECUT3	0.015855	0.307097	0.654997	None
10154	AMZ2	0.116544	0.307222	0.655112	None
10155	LOC10192	-0.04557	0.307232	0.655112	None
10156	ADAMTSL5	-0.03187	0.307242	0.655112	None
10157	LOC10193	-0.03206	0.30737	0.655321	None
10158	CLUAP1	-0.1594	0.307422	0.655367	None
10159	AMBRA1	0.049797	0.307502	0.655473	None
10160	WDR37	-0.18714	0.30759	0.655553	None
10161	TMED9	0.182471	0.3076	0.655553	None
10162	TC2N	-0.24924	0.307679	0.655629	None
10163	ABCC5	-0.18117	0.307696	0.655629	None
10164	ABTB1	-0.00913	0.307754	0.655688	None
10165	PACSIN2	0.134717	0.307813	0.655728	None
10166	CDS1	-0.10375	0.307846	0.655728	None
10167	CEP57	0.162387	0.307864	0.655728	None
10168	CXCL11	-0.19843	0.307923	0.655766	None
10169	TIAM1	0.155359	0.307942	0.655766	None
10170	PRAMEF11	-0.02836	0.308167	0.6561	None
10171	MIRLET7BH	-0.06696	0.308186	0.6561	None
10172	DEFB1	-0.07506	0.308214	0.6561	None
10173	TEK	-0.17346	0.308227	0.6561	None
10174	CETN2	-0.12928	0.308293	0.6561	None
10175	JAZF1	-0.30769	0.308302	0.6561	None
10176	TMEM114	0.009915	0.308311	0.6561	None
10177	BAG3	-0.35209	0.308411	0.6562	None
10178	UNC5A	0.029949	0.308419	0.6562	None
10179	VPS51	-0.17417	0.308458	0.65622	None
10180	NAALADL2	0.022505	0.308506	0.656257	None
10181	DNAJB2	-0.21495	0.308576	0.656341	None
10182	GAS2	-0.15101	0.308623	0.656378	None
10183	ANGPT2	-0.1414	0.308739	0.656519	None
10184	PPP1R14C	-0.20853	0.308755	0.656519	None
10185	YIPF2	-0.06382	0.308791	0.656519	None
10186	KIAA1551	-0.19762	0.308832	0.656519	None
10187	C21orf88	0.003039	0.30887	0.656519	None
10188	DNM3	-0.28778	0.308876	0.656519	None
10189	UTY	-0.27407	0.308985	0.656519	None
10190	PBK	0.441481	0.309001	0.656519	None
10191	AFTPH	-0.08854	0.309015	0.656519	None
10192	GLRX5	-0.16137	0.309017	0.656519	None
10193	PHTF2	0.07982	0.309041	0.656519	None
10194	STAB2	-0.13742	0.309053	0.656519	None
10195	LOC10050	-0.08929	0.309121	0.656597	None
10196	CUTA	-0.10639	0.309157	0.656609	None
10197	ZNF814	-0.1737	0.309237	0.656716	None
10198	CTD-2542I	0.02325	0.3093	0.656729	None
10199	IL13	0.030002	0.309315	0.656729	None
10200	RP11-397A	-0.01312	0.309335	0.656729	None
10201	ZNF674-AS	0.200578	0.30945	0.656911	None
10202	ST3GAL5	0.258525	0.309495	0.656933	None
10203	FAM195B	-0.08751	0.309522	0.656933	None
10204	NFKB1	0.181093	0.309577	0.656987	None
10205	TCHP	-0.09078	0.309633	0.657028	None
10206	HIST1H3B	-0.25316	0.309703	0.657028	None

10207	GOT2	-0.16268	0.309725	0.657028	None
10208	LOC40062:	-0.02529	0.309727	0.657028	None
10209	LOC10192!	0.004102	0.309748	0.657028	None
10210	TNFAIP6	0.438139	0.309779	0.65703	None
10211	TOX	0.208067	0.309816	0.657044	None
10212	PGRMC1	-0.18698	0.309855	0.657061	None
10213	MIR142	-0.20817	0.309914	0.657122	None
10214	ASF1B	-0.2728	0.309965	0.657166	None
10215	PCDH12	0.172744	0.310038	0.657194	None
10216	NAB2	-0.12101	0.310039	0.657194	None
10217	LOC10272	-0.30181	0.310075	0.657205	None
10218	FCER1A	-0.2061	0.310162	0.657325	None
10219	SLC50A1	-0.17276	0.310631	0.658195	None
10220	CXCL17	0.028781	0.310633	0.658195	None
10221	MAPK1	-0.1544	0.310705	0.658284	None
10222	CIITA	0.018531	0.310756	0.658297	None
10223	LARP4	-0.11944	0.310772	0.658297	None
10224	HIST1H2B(	-0.36721	0.310803	0.658297	None
10225	7-Mar	0.242647	0.310835	0.658302	None
10226	APOD	0.147796	0.310922	0.658421	None
10227	NFE4	-0.03165	0.310966	0.65845	None
10228	ZDHHC19	0.014758	0.311013	0.658484	None
10229	PDXK	-0.14489	0.311266	0.658957	None
10230	TOE1	0.115411	0.311356	0.659083	None
10231	RRP36	-0.11831	0.311387	0.659084	None
10232	BEAN1	0.005004	0.311498	0.659253	None
10233	TNFRSF4	0.026832	0.311662	0.659537	None
10234	SORBS3	0.053354	0.311727	0.659555	None
10235	NCBP1	-0.10724	0.311752	0.659555	None
10236	ALDH18A1	-0.14039	0.311784	0.659555	None
10237	LOC10272	-0.0677	0.3118	0.659555	None
10238	SLURP1	0.002557	0.311823	0.659555	None
10239	IP6K2	-0.13789	0.31188	0.659559	None
10240	CHRDL2	0.023509	0.311906	0.659559	None
10241	SLC7A7	-0.53274	0.311916	0.659559	None
10242	UBXN1	0.148784	0.311967	0.65959	None
10243	TAGAP	0.184345	0.311992	0.65959	None
10244	BCL9L	-0.05022	0.312175	0.659874	None
10245	ICAM5	-0.02205	0.312215	0.659874	None
10246	EYA3	0.094464	0.312218	0.659874	None
10247	MORF4L1	0.086198	0.312268	0.659916	None
10248	TIGD4	0.016337	0.312348	0.660022	None
10249	LOC65478(	0.069149	0.312402	0.660071	None
10250	TMED1	-0.14489	0.312441	0.660089	None
10251	ANLN	0.262648	0.312473	0.660091	None
10252	CABS1	0.001683	0.312693	0.660493	None
10253	ACY3	-0.14348	0.312883	0.660829	None
10254	MYO1E	-0.08924	0.312929	0.660862	None
10255	CDK5	-0.1896	0.312973	0.660891	None
10256	LINC00592	0.014335	0.313065	0.660938	None
10257	AK021933	-0.03393	0.313066	0.660938	None
10258	SAG	0.033369	0.313087	0.660938	None
10259	PHF11	0.115792	0.31318	0.661027	None
10260	RNF207	-0.03755	0.31319	0.661027	None
10261	RPL34	-0.11348	0.313255	0.6611	None
10262	LOC28607:	-0.08749	0.313398	0.661337	None
10263	SRGAP2C	0.229509	0.313471	0.661426	None
10264	RP5-1039K	-0.07977	0.313509	0.661442	None

10265	DDOST	0.131936	0.313556	0.661467	None
10266	AVEN	0.151489	0.313582	0.661467	None
10267	VPS13D	-0.14703	0.313624	0.66149	None
10268	DCAF4L1	-0.03843	0.313674	0.661533	None
10269	ANG	-0.13255	0.313789	0.66171	None
10270	JOSD2	-0.02265	0.313868	0.661778	None
10271	MTERF1	-0.14512	0.313882	0.661778	None
10272	SH3PXD2A	-0.09502	0.314046	0.662059	None
10273	SLC25A36	-0.16995	0.31414	0.662146	None
10274	RARRES1	-0.14633	0.314149	0.662146	None
10275	YIPF4	0.130972	0.31422	0.662232	None
10276	AWAT1	-0.01589	0.314446	0.662618	None
10277	CPSF1	0.044576	0.314488	0.662618	None
10278	PSG7	0.002465	0.314495	0.662618	None
10279	MARCKS	-0.42279	0.314557	0.662684	None
10280	PSKH2	0.01897	0.314644	0.662775	None
10281	VAV3	0.153967	0.314661	0.662775	None
10282	MBIP	0.202662	0.314755	0.66283	None
10283	SLC47A2	0.012386	0.314779	0.66283	None
10284	GABRB3	-0.12658	0.314782	0.66283	None
10285	ADH4	-0.01339	0.314811	0.66283	None
10286	ABCC13	-0.11692	0.31484	0.66283	None
10287	CIB1	0.142338	0.315153	0.663424	None
10288	TSTD2	0.020812	0.315236	0.663533	None
10289	LRRC39	-0.12246	0.315396	0.663758	None
10290	C3	-0.19051	0.315424	0.663758	None
10291	PLCXD2	0.157432	0.315434	0.663758	None
10292	EPS8L1	-0.04874	0.31551	0.663852	None
10293	ATOH7	-0.00505	0.315563	0.6639	None
10294	ZNF805	-0.14468	0.315666	0.663998	None
10295	ZNF572	-0.08088	0.315671	0.663998	None
10296	FLJ21408	-0.00998	0.315747	0.664037	None
10297	AGR3	-0.00929	0.315772	0.664037	None
10298	POT1	-0.13053	0.315799	0.664037	None
10299	EIF5A2	-0.03968	0.315822	0.664037	None
10300	HPR	-0.01316	0.315843	0.664037	None
10301	DOC2B	-0.04782	0.315999	0.664259	None
10302	TAP2	-0.14165	0.31601	0.664259	None
10303	CST4	-0.04027	0.316116	0.664319	None
10304	ZNF852	0.031241	0.316126	0.664319	None
10305	ZFP91	0.117138	0.316131	0.664319	None
10306	LCE1B	-0.01488	0.316175	0.664348	None
10307	C1QB	-0.79821	0.316289	0.664523	None
10308	YPEL5	0.177273	0.316363	0.664614	None
10309	TMEM258	-0.12993	0.316608	0.665063	None
10310	LOC10192	-0.01431	0.316645	0.665076	None
10311	GTPBP3	-0.06273	0.31668	0.665086	None
10312	ZNF407	0.019409	0.316723	0.665112	None
10313	TNIP1	-0.15987	0.316802	0.665214	None
10314	DLGAP3	-0.01914	0.316835	0.665218	None
10315	SLC44A2	0.257896	0.316999	0.665413	None
10316	PHB	0.148792	0.317014	0.665413	None
10317	TINAGL1	-0.04118	0.317023	0.665413	None
10318	FBXO24	-0.05621	0.317056	0.665413	None
10319	LGALSL	-0.36022	0.317081	0.665413	None
10320	JAK1	0.189554	0.317218	0.665635	None
10321	MTF2	0.128623	0.317264	0.66565	None
10322	PAXBP1-A	-0.04784	0.31735	0.66565	None

10323	CFB	-0.03522	0.317388	0.66565	None
10324	PHLDB2	0.14899	0.317396	0.66565	None
10325	CD44	-0.29206	0.317403	0.66565	None
10326	LINC00839	-0.12932	0.317409	0.66565	None
10327	TTC12	-0.10082	0.317505	0.665722	None
10328	LINC00566	0.032273	0.317515	0.665722	None
10329	TMEM64	0.196639	0.317544	0.665722	None
10330	ANGPT4	0.02104	0.317567	0.665722	None
10331	GPX7	0.198441	0.317603	0.665733	None
10332	MAGOHB	0.117756	0.31776	0.665997	None
10333	HEATR3	-0.17289	0.31788	0.666177	None
10334	SLC38A2	0.135936	0.317907	0.666177	None
10335	CTD-2540I	-0.07348	0.31803	0.666272	None
10336	LINC00938	0.262271	0.318131	0.666272	None
10337	USP14	-0.09816	0.318195	0.666272	None
10338	LMNB2	-0.21482	0.318203	0.666272	None
10339	BTBD8	-0.00897	0.31823	0.666272	None
10340	LBX1-AS1	-0.01855	0.318243	0.666272	None
10341	BAG2	-0.19191	0.318307	0.666272	None
10342	SF3B5	-0.14761	0.318308	0.666272	None
10343	GSN-AS1	-0.01365	0.318311	0.666272	None
10344	OR51B4	-0.00688	0.318377	0.666272	None
10345	ELAC1	-0.1355	0.318425	0.666272	None
10346	RPH3A	-0.05916	0.31844	0.666272	None
10347	TIMM17A	0.118493	0.318447	0.666272	None
10348	LYRM1	-0.13084	0.318478	0.666272	None
10349	OPTN	-0.2413	0.318484	0.666272	None
10350	CLYBL	-0.11086	0.318501	0.666272	None
10351	GIPC3	-0.03666	0.318554	0.666272	None
10352	AZU1	0.588828	0.318564	0.666272	None
10353	PSG11	0.017105	0.318601	0.666272	None
10354	USP1	0.130475	0.318609	0.666272	None
10355	SEPT7P9	-0.08976	0.318619	0.666272	None
10356	SPN	0.026328	0.318629	0.666272	None
10357	TUBBP5	-0.02441	0.318723	0.666346	None
10358	GLP1R	-0.02167	0.318726	0.666346	None
10359	DDX5	0.140469	0.318862	0.666556	None
10360	KIZ	-0.15409	0.318888	0.666556	None
10361	LENG9	-0.05355	0.318934	0.666588	None
10362	C10orf99	0.036214	0.319045	0.666756	None
10363	RP11-708J	0.103988	0.319135	0.666879	None
10364	GXYLT2	-0.2349	0.319198	0.666929	None
10365	AC006026.	-0.20076	0.31922	0.666929	None
10366	APMAP	-0.15305	0.319269	0.666951	None
10367	LOC10099(	0.023501	0.319293	0.666951	None
10368	LOC10050	0.05517	0.319413	0.667139	None
10369	AY940074	0.208941	0.319625	0.6675	None
10370	FBXL12	-0.05941	0.319681	0.6675	None
10371	ACKR1	-0.23594	0.319704	0.6675	None
10372	ZNF663P	-0.03357	0.319709	0.6675	None
10373	RSL24D1	0.119459	0.319748	0.667515	None
10374	CYTH4	-0.15003	0.319795	0.667549	None
10375	WNK1	-0.25617	0.319839	0.667577	None
10376	OTOR	-0.02141	0.319917	0.667676	None
10377	CEBPD	-0.34953	0.319977	0.667737	None
10378	LOC10192	-0.01154	0.320074	0.667815	None
10379	NCK2	-0.14643	0.320076	0.667815	None
10380	LOC10050(	0.01251	0.320132	0.667866	None

10381	S100A2	-0.01795	0.320215	0.667976	None
10382	CLEC12B	-0.12723	0.320499	0.668504	None
10383	LINC00924	0.010022	0.320718	0.668895	None
10384	NAIP	-0.28491	0.320754	0.668907	None
10385	ATG10	-0.14845	0.320792	0.668922	None
10386	ERH	-0.17687	0.320839	0.668956	None
10387	NDUFB11	-0.1683	0.320933	0.669086	None
10388	NKX3-2	-0.01221	0.321114	0.669364	None
10389	UBIAD1	-0.13586	0.321128	0.669364	None
10390	S100A12	0.667758	0.321216	0.669483	None
10391	RORB	-0.05306	0.321391	0.669766	None
10392	LRRC17	-0.05463	0.321413	0.669766	None
10393	EXOC4	-0.218	0.321728	0.670336	None
10394	SPINK2	0.315146	0.321749	0.670336	None
10395	CECR7	-0.03044	0.321798	0.670336	None
10396	NLGN4Y	-0.0322	0.321817	0.670336	None
10397	MED27	0.141368	0.321842	0.670336	None
10398	GPRIN2	0.016096	0.321998	0.670548	None
10399	TERT	0.092373	0.322006	0.670548	None
10400	VPS37B	-0.21508	0.322175	0.67079	None
10401	NEGR1	0.341804	0.322184	0.67079	None
10402	CEACAM1	-0.09714	0.322263	0.670891	None
10403	TTLL1	-0.15404	0.322336	0.670978	None
10404	KLHL1	-0.01086	0.322419	0.671057	None
10405	PEX7	0.146808	0.322436	0.671057	None
10406	SLC39A13	-0.05441	0.322543	0.671197	None
10407	GOT1L1	-0.02918	0.322565	0.671197	None
10408	PLXDC2	-0.24741	0.322633	0.671274	None
10409	RP11-138I	-0.02004	0.322685	0.671275	None
10410	GABRA3	-0.00664	0.322696	0.671275	None
10411	LOC40074	0.046484	0.322779	0.671384	None
10412	SLC12A4	0.033226	0.322937	0.671649	None
10413	KBTBD11	0.33955	0.323011	0.671737	None
10414	ECRP	0.439729	0.323121	0.671843	None
10415	NHS	-0.03373	0.323148	0.671843	None
10416	SLC4A3	-0.05072	0.323155	0.671843	None
10417	PCBD2	0.204697	0.323233	0.671895	None
10418	LOC10192	0.044291	0.323247	0.671895	None
10419	RP11-619L	-0.02267	0.323273	0.671895	None
10420	CXorf31	-0.01575	0.323319	0.671926	None
10421	PTGER3	-0.07629	0.323403	0.671991	None
10422	ABCE1	-0.0835	0.323412	0.671991	None
10423	LOC10272	0.200153	0.323444	0.671994	None
10424	DOK6	-0.18592	0.323524	0.672095	None
10425	MYT1	-0.04353	0.323592	0.672158	None
10426	OR2L13	-0.11667	0.323617	0.672158	None
10427	EML2	-0.05781	0.323729	0.672327	None
10428	CTD-3193	0.010916	0.323771	0.67235	None
10429	NUP50-AS	-0.13714	0.323844	0.672436	None
10430	SMLR1	-0.01429	0.324308	0.673222	None
10431	ONECUT2	0.002539	0.324338	0.673222	None
10432	FBXO36	-0.01932	0.324371	0.673222	None
10433	TEX10	-0.09455	0.324371	0.673222	None
10434	NMRK2	-0.02507	0.324378	0.673222	None
10435	LOC64551	-0.13394	0.324435	0.673277	None
10436	LOC28345	0.024925	0.324469	0.673282	None
10437	RPP30	-0.13073	0.324582	0.673452	None
10438	UGT8	-0.15551	0.324636	0.673501	None

10439	DENND1B	0.205256	0.324672	0.673509	None
10440	POLB	0.144897	0.324894	0.673906	None
10441	ANKRD29	-0.15486	0.325009	0.674056	None
10442	PMS2P3	-0.13609	0.325028	0.674056	None
10443	OXSR1	-0.19535	0.325097	0.674133	None
10444	GTPBP1	-0.03431	0.32517	0.674221	None
10445	CTD-2021I	0.040467	0.325291	0.67433	None
10446	RP11-134(	-0.08508	0.325315	0.67433	None
10447	BRD8	0.054495	0.32533	0.67433	None
10448	MAN2C1	-0.13563	0.325348	0.67433	None
10449	CASC5	0.182	0.325463	0.674506	None
10450	ARMC5	-0.12196	0.325507	0.674531	None
10451	CNR1	0.001383	0.325652	0.674611	None
10452	ZNF169	-0.07965	0.325658	0.674611	None
10453	RPF1	-0.19278	0.325679	0.674611	None
10454	MRPS18C	-0.08069	0.325702	0.674611	None
10455	TMEM218	-0.09705	0.325713	0.674611	None
10456	HAS3	-0.0249	0.32576	0.674611	None
10457	RP11-471(	-0.02382	0.325764	0.674611	None
10458	MAPK1IP1I	0.097598	0.325844	0.674713	None
10459	SV2B	-0.0252	0.325899	0.674742	None
10460	TAOK2	-0.05648	0.32592	0.674742	None
10461	LSM5	0.154403	0.325998	0.674838	None
10462	PARG	0.106213	0.326099	0.674942	None
10463	FOXRED1	-0.11754	0.326189	0.674942	None
10464	DCDC2	-0.05636	0.32621	0.674942	None
10465	CTD-2297I	-0.03739	0.326244	0.674942	None
10466	HMGCS1	0.103237	0.32625	0.674942	None
10467	UBE2Q1	-0.15499	0.326267	0.674942	None
10468	MYRF	-0.01923	0.326271	0.674942	None
10469	SPOPL	0.130386	0.326343	0.674942	None
10470	XDH	0.037642	0.326343	0.674942	None
10471	LOC64298(	-0.03113	0.32636	0.674942	None
10472	UNC45B	-0.01183	0.326404	0.67497	None
10473	9-Mar	-0.05046	0.326534	0.675126	None
10474	SLC3A2	0.143979	0.326571	0.675126	None
10475	PIDD1	-0.08647	0.326573	0.675126	None
10476	SYNDIG1L	-0.01797	0.326633	0.675185	None
10477	RP3-368A4	0.30027	0.326672	0.6752	None
10478	SEMG2	-0.01182	0.326732	0.67526	None
10479	UQCR11	-0.10877	0.326784	0.675305	None
10480	SNAPC3	-0.12104	0.326937	0.675541	None
10481	SLC25A10	-0.08151	0.326961	0.675541	None
10482	RP13-30A9	-0.01666	0.327109	0.675721	None
10483	CCNE1	-0.14537	0.327111	0.675721	None
10484	WDR66	-0.00725	0.327281	0.675984	None
10485	GUCD1	0.13598	0.327324	0.675984	None
10486	CCDC144C	0.009606	0.327333	0.675984	None
10487	PEBP4	-0.02085	0.327363	0.675984	None
10488	PON3	-0.01136	0.327436	0.67607	None
10489	BZRAP1	-0.36427	0.327491	0.676079	None
10490	EBLN2	0.199704	0.327503	0.676079	None
10491	TMIE	-0.06804	0.327617	0.676133	None
10492	EXOSC4	-0.17402	0.327674	0.676133	None
10493	HES5	-0.02772	0.327678	0.676133	None
10494	ANXA11	-0.11505	0.32768	0.676133	None
10495	KGFLP2	-0.07382	0.327711	0.676133	None
10496	FLAD1	-0.11947	0.327716	0.676133	None

10497	UTP3	-0.1133	0.327851	0.676315	None
10498	MAML2	0.158788	0.327875	0.676315	None
10499	PPM1D	-0.12523	0.327898	0.676315	None
10500	MCCC1	0.098702	0.327969	0.676317	None
10501	KCNH8	-0.06944	0.327977	0.676317	None
10502	SNORA43	0.021188	0.327993	0.676317	None
10503	FAM171B	-0.06605	0.328065	0.676353	None
10504	IL10RB-AS	-0.14396	0.328073	0.676353	None
10505	EEA1	0.233334	0.328142	0.676384	None
10506	RP11-539I!	-0.07821	0.32815	0.676384	None
10507	C20orf166-	-0.03015	0.328204	0.67643	None
10508	PAX9	-0.0074	0.32836	0.676677	None
10509	ADCYAP1	-0.14171	0.328386	0.676677	None
10510	PTPRR	-0.00438	0.328537	0.676837	None
10511	POLDIP2	-0.12919	0.32855	0.676837	None
10512	LOC10013:	0.016926	0.328578	0.676837	None
10513	SRPX2	-0.10413	0.328588	0.676837	None
10514	CCND3	0.22833	0.328622	0.67684	None
10515	CCDC85A	-0.02143	0.328657	0.676849	None
10516	SULT4A1	-0.05094	0.328757	0.676987	None
10517	C1D	-0.15375	0.328787	0.676987	None
10518	POLR3K	-0.13524	0.328921	0.677154	None
10519	B9D1	-0.09403	0.32893	0.677154	None
10520	SPCS3	-0.15965	0.328976	0.677184	None
10521	OR13C4	-0.01749	0.329095	0.677289	None
10522	AMPH	-0.01889	0.329101	0.677289	None
10523	MYH16	0.018531	0.329121	0.677289	None
10524	PQBP1	-0.19062	0.329227	0.677443	None
10525	SNX29	0.076367	0.32929	0.677508	None
10526	HRCT1	0.029239	0.329371	0.677611	None
10527	NPR3	0.295764	0.329417	0.677641	None
10528	FAM122C	0.080355	0.329493	0.677734	None
10529	ARHGDIG	-0.03606	0.329538	0.677735	None
10530	C19orf47	-0.01033	0.329556	0.677735	None
10531	IQCB1	0.111903	0.329617	0.67776	None
10532	HOXB6	-0.15219	0.329631	0.67776	None
10533	PRRT3-AS:	-0.2012	0.329911	0.678202	None
10534	APH1A	-0.14075	0.329962	0.678202	None
10535	RP11-362K	-0.10215	0.329969	0.678202	None
10536	ZNF827	-0.22157	0.329972	0.678202	None
10537	DLD	0.112603	0.330137	0.678477	None
10538	NOL10	-0.08647	0.330311	0.678771	None
10539	ZUFSP	-0.12199	0.330344	0.678774	None
10540	BC069776	-0.04553	0.330419	0.678864	None
10541	MEPE	0.007591	0.330484	0.678933	None
10542	C15orf37	0.119869	0.330552	0.679007	None
10543	NAPA	-0.24824	0.33064	0.679084	None
10544	TOP1	-0.12851	0.330653	0.679084	None
10545	CREBZF	0.176206	0.330703	0.679084	None
10546	EPM2AIP1	-0.18779	0.330714	0.679084	None
10547	COL6A6	-0.21932	0.330752	0.679096	None
10548	PLCL2	-0.26549	0.330823	0.679179	None
10549	OR1I1	-0.02486	0.330893	0.67924	None
10550	TSEN34	-0.10488	0.330934	0.67924	None
10551	LAMTOR5	-0.06988	0.330947	0.67924	None
10552	ZFYVE28	-0.01676	0.331039	0.6793	None
10553	SSC5D	-0.05672	0.331039	0.6793	None
10554	ADRB3	0.015332	0.331141	0.679444	None

10555	PHF8	0.057056	0.3313	0.679617	None
10556	IQCA1	0.012686	0.331321	0.679617	None
10557	CLEC2B	-0.25389	0.331361	0.679617	None
10558	LINC00957	-0.03847	0.331365	0.679617	None
10559	FAM81A	0.01788	0.331382	0.679617	None
10560	USP8	0.128535	0.331434	0.679659	None
10561	NUDT15	0.137987	0.331706	0.680153	None
10562	SLC25A4	0.068377	0.331802	0.680286	None
10563	TTLL5	-0.07015	0.331866	0.680308	None
10564	C9orf47	-0.03696	0.331881	0.680308	None
10565	ITGAV	0.212133	0.33195	0.680308	None
10566	EFCAB14	-0.11078	0.331955	0.680308	None
10567	SERPINA2	0.002233	0.33197	0.680308	None
10568	C12orf4	-0.13756	0.332012	0.680329	None
10569	LOC10272:	0.008932	0.33208	0.680404	None
10570	VPS13A	0.201706	0.332137	0.680457	None
10571	MEFV	-0.07196	0.332292	0.680628	None
10572	COPS4	-0.04294	0.332316	0.680628	None
10573	SCNN1A	-0.02368	0.332346	0.680628	None
10574	GDA	0.008227	0.332347	0.680628	None
10575	XCL1	0.025964	0.332474	0.680824	None
10576	SMIM2-AS	-0.02409	0.332524	0.680864	None
10577	NAPSA	-0.03652	0.332606	0.680967	None
10578	FUS	-0.19704	0.332824	0.681302	None
10579	LOC10028	-0.04726	0.332833	0.681302	None
10580	ZNF613	0.147982	0.332976	0.681532	None
10581	PCDHB15	-0.08795	0.333144	0.681761	None
10582	IL1B	0.283796	0.333154	0.681761	None
10583	TRDN	-0.12149	0.333183	0.681761	None
10584	SKP1	-0.10463	0.333245	0.681824	None
10585	TAF7L	-0.00451	0.333526	0.682334	None
10586	ZBTB43	0.138843	0.333601	0.682374	None
10587	SCN2A	-0.12711	0.333613	0.682374	None
10588	LRFN3	-0.0229	0.33367	0.682374	None
10589	CCNG2	-0.19211	0.333672	0.682374	None
10590	CCDC60	0.045354	0.333734	0.682374	None
10591	PRND	0.007571	0.33376	0.682374	None
10592	TAF1A-AS:	-0.08182	0.333766	0.682374	None
10593	ACKR4	-0.02963	0.333929	0.682643	None
10594	KIF7	-0.13467	0.333982	0.682677	None
10595	PRO0471	-0.10119	0.334018	0.682677	None
10596	PIK3CD	0.10013	0.33404	0.682677	None
10597	RP1-170O:	-0.06555	0.334266	0.68305	None
10598	MDH1	-0.09844	0.334286	0.68305	None
10599	MGARP	-0.06486	0.334364	0.683138	None
10600	FN3K	0.001296	0.334392	0.683138	None
10601	COX7A2L	0.091767	0.334435	0.683162	None
10602	NUDT14	-0.09351	0.33447	0.683168	None
10603	SAMD10	-0.03547	0.334541	0.683248	None
10604	DEFB132	-0.01153	0.334624	0.683319	None
10605	RAB40C	-0.02075	0.334667	0.683319	None
10606	CRTAM	-0.19836	0.33467	0.683319	None
10607	LOC72922	-0.02797	0.33472	0.683351	None
10608	UBAP1L	-0.03455	0.334749	0.683351	None
10609	CRADD	0.152687	0.33491	0.683595	None
10610	POLRMT	0.031039	0.334932	0.683595	None
10611	C18orf8	0.101916	0.334996	0.683661	None
10612	PCAT19	-0.08487	0.335027	0.683661	None

10613	LINC01056	0.0013	0.335174	0.683896	None
10614	DBR1	0.167879	0.335272	0.684031	None
10615	TTC39B	-0.20765	0.33536	0.684139	None
10616	TAAR5	-0.02326	0.335455	0.684139	None
10617	TRIP4	-0.10904	0.335467	0.684139	None
10618	SFXN4	-0.13232	0.335479	0.684139	None
10619	CCDC155	-0.02048	0.335484	0.684139	None
10620	KCTD21	-0.15027	0.335555	0.684139	None
10621	TMX3	0.158924	0.335557	0.684139	None
10622	LMTK2	-0.01722	0.335577	0.684139	None
10623	LOC10192	-0.01154	0.335656	0.684236	None
10624	SLC35D3	-0.37693	0.335729	0.684319	None
10625	LINC00494	-0.02633	0.335772	0.684343	None
10626	HDAC6	-0.05433	0.335838	0.684382	None
10627	BROX	-0.17824	0.335854	0.684382	None
10628	ATP1B3	-0.04585	0.336122	0.684762	None
10629	INSM2	-0.01389	0.336134	0.684762	None
10630	TRIT1	0.112788	0.336158	0.684762	None
10631	SRSF6	-0.20284	0.33619	0.684762	None
10632	KLRAP1	-0.01968	0.336227	0.684762	None
10633	CCDC8	-0.14455	0.336247	0.684762	None
10634	TUFT1	-0.25594	0.336262	0.684762	None
10635	LOC10192	-0.20174	0.33637	0.684917	None
10636	LOC10192	-0.01297	0.336485	0.685025	None
10637	FCGR3B	-0.51321	0.336486	0.685025	None
10638	CCKAR	-0.03971	0.336593	0.685178	None
10639	PIP4K2A	-0.25016	0.336796	0.685469	None
10640	CLEC3A	0.017171	0.336799	0.685469	None
10641	LRG1	-0.22708	0.336931	0.685672	None
10642	BUB1B	0.213274	0.337024	0.685756	None
10643	TSPAN11	0.041008	0.337035	0.685756	None
10644	LOC10028	0.147288	0.337083	0.685767	None
10645	VKORC1L1	-0.09559	0.337119	0.685767	None
10646	ANXA2P2	-0.19481	0.337162	0.685767	None
10647	UBAC2-AS	-0.06918	0.337168	0.685767	None
10648	PHPT1	-0.14701	0.337282	0.685892	None
10649	RNF112	-0.02372	0.337323	0.685892	None
10650	AFAP1L2	-0.17695	0.337324	0.685892	None
10651	BCKDHB	-0.09683	0.33739	0.685961	None
10652	SGSM1	-0.01366	0.337483	0.686066	None
10653	LOC10028	-0.10263	0.337528	0.686066	None
10654	SRP9	-0.11359	0.337536	0.686066	None
10655	HIST3H3	0.021769	0.33761	0.686151	None
10656	ATP6V1G3	-0.01653	0.337659	0.686162	None
10657	CACNG2	0.002498	0.337679	0.686162	None
10658	ATP6V1B1	0.015309	0.337729	0.6862	None
10659	PRO1804	-0.06586	0.337852	0.686385	None
10660	WNT5A	-0.06528	0.337886	0.68639	None
10661	SAMD4A	-0.06425	0.337933	0.686412	None
10662	RP1-28C2(	-0.00359	0.33796	0.686412	None
10663	C14orf180	-0.01989	0.338032	0.686494	None
10664	LOC10027	0.026609	0.338079	0.686499	None
10665	SUMF2	-0.16647	0.338098	0.686499	None
10666	LOC10192	-0.05559	0.338181	0.686603	None
10667	TAX1BP1	-0.11259	0.338218	0.686615	None
10668	FMN2	-0.01639	0.338261	0.686622	None
10669	RAB7A	0.05475	0.338285	0.686622	None
10670	PSMG1	-0.14302	0.338334	0.686657	None

10671	MIR34A	-0.08012	0.338386	0.686698	None
10672	CSNK1A1	0.209374	0.338497	0.686858	None
10673	AMER1	-0.02976	0.338588	0.686979	None
10674	TRIM61	0.090181	0.338795	0.687335	None
10675	MUC7	-0.00675	0.338908	0.6875	None
10676	PCOLCE-A	-0.01616	0.338945	0.687511	None
10677	PDE2A	-0.11067	0.339035	0.687613	None
10678	PDCL	0.087407	0.339059	0.687613	None
10679	MT1M	-0.1324	0.339126	0.687684	None
10680	LINC00348	-0.01883	0.339185	0.687709	None
10681	TCEB3	0.09855	0.339202	0.687709	None
10682	SPIDR	-0.18257	0.339239	0.687719	None
10683	POLE3	0.128104	0.339359	0.687883	None
10684	HEATR9	0.031239	0.339383	0.687883	None
10685	MPEG1	0.542547	0.339539	0.688135	None
10686	LOC28542	-0.01503	0.339645	0.688285	None
10687	SSTR5	0.051786	0.339689	0.68831	None
10688	EXD3	-0.03285	0.339828	0.688527	None
10689	ELMOD2	0.078513	0.34002	0.688762	None
10690	TTC9C	-0.11434	0.340078	0.688762	None
10691	LDHC	-0.19192	0.340085	0.688762	None
10692	SHANK2	-0.12074	0.340112	0.688762	None
10693	KANK4	-0.06666	0.340134	0.688762	None
10694	NEUROD4	-0.02098	0.340135	0.688762	None
10695	HAND1	0.020063	0.340286	0.688977	None
10696	NAF1	0.219574	0.340305	0.688977	None
10697	GIPR	0.021745	0.340363	0.688977	None
10698	LTB4R2	-0.06068	0.340368	0.688977	None
10699	SOX6	-0.10233	0.340486	0.689151	None
10700	NOP58	0.096192	0.340557	0.689212	None
10701	VDAC1	0.285169	0.34058	0.689212	None
10702	IP6K3	-0.04496	0.340631	0.689251	None
10703	IGK	-0.22073	0.34073	0.689342	None
10704	OSER1-AS	0.061707	0.340739	0.689342	None
10705	LOC10050	-0.03017	0.340795	0.689354	None
10706	MRPL12	-0.13109	0.340809	0.689354	None
10707	SEC14L2	-0.02635	0.340955	0.689547	None
10708	CACTIN	-0.07777	0.340968	0.689547	None
10709	TFAP4	0.060038	0.341162	0.689875	None
10710	AKAP11	-0.16456	0.341248	0.689984	None
10711	AK098263	0.023837	0.341418	0.690264	None
10712	TMEM262	-0.02944	0.341487	0.690338	None
10713	PDCD1LG2	-0.11481	0.34155	0.69035	None
10714	CYP2A13	-0.05645	0.341557	0.69035	None
10715	CCDC88A	0.121269	0.341595	0.690363	None
10716	SNORA78	-0.02957	0.341697	0.690506	None
10717	GPM6A	-0.25156	0.341841	0.690677	None
10718	SEPHS1	0.119834	0.341846	0.690677	None
10719	PAAF1	-0.12119	0.34191	0.690742	None
10720	HMX1	0.064807	0.342021	0.690894	None
10721	WDR93	-0.02863	0.342049	0.690894	None
10722	NAE1	0.144924	0.342152	0.690988	None
10723	LOC10192	0.056508	0.34216	0.690988	None
10724	LOC10192	0.00559	0.342318	0.69109	None
10725	LINC00174	0.013969	0.342319	0.69109	None
10726	FAM110D	-0.04121	0.34236	0.69109	None
10727	TDRP	-0.26133	0.342363	0.69109	None
10728	PARP1	0.124617	0.342392	0.69109	None

10729	FLJ46875	0.145279	0.342431	0.69109	None
10730	HS3ST3A1	-0.06766	0.342433	0.69109	None
10731	ARHGAP22	-0.00743	0.342466	0.691092	None
10732	CYP4X1	-0.25906	0.342534	0.691154	None
10733	PTMS	-0.05667	0.342561	0.691154	None
10734	CPLX2	0.019534	0.34279	0.691538	None
10735	TAC3	-0.02415	0.342824	0.691538	None
10736	NUP85	-0.14879	0.342856	0.691538	None
10737	CNKSR3	-0.05181	0.342962	0.691538	None
10738	DCUN1D2	-0.1914	0.342987	0.691538	None
10739	TTTY10	-0.15622	0.342988	0.691538	None
10740	KIR2DL3	0.029413	0.343034	0.691538	None
10741	RUNX2	-0.09936	0.343045	0.691538	None
10742	MPZ	-0.03151	0.343046	0.691538	None
10743	LOC10050	0.038471	0.343096	0.691538	None
10744	APP	-0.24228	0.343102	0.691538	None
10745	KRBOX1	0.168263	0.343148	0.691547	None
10746	CAMP	0.408404	0.343202	0.691547	None
10747	CACFD1	0.045768	0.343203	0.691547	None
10748	ALX3	-0.02152	0.343291	0.691661	None
10749	CD4	-0.11172	0.343562	0.692142	None
10750	ZNF862	-0.17398	0.343624	0.692179	None
10751	LOC10192	0.042453	0.343644	0.692179	None
10752	KMT2C	-0.22398	0.343823	0.692475	None
10753	CUTC	-0.09066	0.343912	0.692588	None
10754	ALG8	-0.14434	0.344063	0.69283	None
10755	LOC44079	-0.0176	0.344129	0.692893	None
10756	KCTD4	0.007476	0.344183	0.692893	None
10757	CCDC182	-0.01695	0.344191	0.692893	None
10758	PTCRA	-0.14446	0.344307	0.693026	None
10759	BRINP1	0.008797	0.344321	0.693026	None
10760	ZFP69B	-0.15282	0.344405	0.693132	None
10761	SUZ12	0.269438	0.344552	0.693326	None
10762	ZAP70	0.108516	0.344596	0.693326	None
10763	ARHGEF6	-0.10162	0.344598	0.693326	None
10764	SPG20	-0.08821	0.34475	0.693527	None
10765	APPL2	-0.19747	0.344794	0.693527	None
10766	8-Mar	-0.17542	0.344826	0.693527	None
10767	ZCCHC3	0.063324	0.344826	0.693527	None
10768	PSMB1	0.096772	0.345068	0.693949	None
10769	ARHGEF15	0.035286	0.345126	0.694001	None
10770	TMSB10	0.142675	0.345166	0.694017	None
10771	RP11-403F	-0.23816	0.345524	0.694523	None
10772	STX18-AS1	-0.06674	0.345527	0.694523	None
10773	USH1G	0.026579	0.345528	0.694523	None
10774	USP29	0.00128	0.345587	0.694523	None
10775	ACSS1	0.023078	0.345671	0.694523	None
10776	MORN2	-0.16063	0.345684	0.694523	None
10777	GLMN	-0.11326	0.345699	0.694523	None
10778	HECA	-0.21108	0.345754	0.694523	None
10779	HCN4	0.034156	0.345757	0.694523	None
10780	ANXA2	-0.24678	0.34577	0.694523	None
10781	SGMS2	-0.13235	0.34577	0.694523	None
10782	USP13	-0.12069	0.345896	0.694711	None
10783	FAM192A	-0.09996	0.345955	0.694766	None
10784	FLRT1	-0.02271	0.346022	0.69479	None
10785	GLI1	-0.0158	0.346031	0.69479	None
10786	LOC10050	-0.08444	0.346086	0.694802	None

10787	C20orf96	-0.00935	0.346122	0.694802	None
10788	OR4D1	-0.01769	0.346159	0.694802	None
10789	TRIM69	0.038404	0.34619	0.694802	None
10790	RP4-773N1	-0.14515	0.346223	0.694802	None
10791	VPRBP	0.133709	0.34623	0.694802	None
10792	SNX18	-0.09504	0.34627	0.694819	None
10793	EXOC3L4	-0.01683	0.3464	0.695014	None
10794	LOC10050(	-0.00594	0.346435	0.69502	None
10795	GOLGA6A	-0.01278	0.346544	0.695117	None
10796	UGGT2	-0.13032	0.346548	0.695117	None
10797	FAM13A-A	-0.17631	0.346624	0.695159	None
10798	FCRL2	-0.00852	0.346632	0.695159	None
10799	NOV	-0.19255	0.346681	0.695191	None
10800	RCN1	-0.12782	0.346729	0.695223	None
10801	TMEM92	-0.04041	0.346812	0.695253	None
10802	GOLGA8N	-0.15242	0.34683	0.695253	None
10803	LRRC69	-0.18007	0.346865	0.695253	None
10804	ALKBH3	-0.12336	0.346872	0.695253	None
10805	CCL24	-0.0121	0.34697	0.695362	None
10806	AFAP1	-0.0829	0.346991	0.695362	None
10807	LOC33986:	-0.22837	0.347027	0.69537	None
10808	NKTR	0.244212	0.34709	0.695433	None
10809	NR6A1	0.020352	0.347173	0.695531	None
10810	KLHDC3	-0.19834	0.347213	0.695531	None
10811	LOC10192	-0.024	0.347235	0.695531	None
10812	NEDD4	-0.13626	0.347324	0.695553	None
10813	ZNF319	-0.06186	0.347332	0.695553	None
10814	KRT32	-0.02239	0.347377	0.695553	None
10815	C2orf40	-0.20513	0.347382	0.695553	None
10816	GNGT1	-0.03413	0.347407	0.695553	None
10817	OOEP	-0.08791	0.347602	0.69586	None
10818	SMCP	0.01019	0.347625	0.69586	None
10819	PADI2	0.019378	0.347687	0.695908	None
10820	KDELC1	0.247477	0.347717	0.695908	None
10821	FLJ32955	0.028865	0.347745	0.695908	None
10822	RP1-190J2	-0.01339	0.347931	0.696216	None
10823	RAPGEF5	-0.02016	0.34797	0.69623	None
10824	CAAP1	0.125227	0.348113	0.696442	None
10825	C15orf26	-0.17362	0.34814	0.696442	None
10826	LOC10050!	0.01671	0.348233	0.696563	None
10827	GALNT8	-0.01264	0.348389	0.696811	None
10828	TMEM132(	-0.07138	0.348654	0.697275	None
10829	CHD5	-0.02921	0.348748	0.697399	None
10830	LINC00463	0.036713	0.348815	0.69747	None
10831	LINC00967	-0.02207	0.348918	0.69761	None
10832	EPYC	0.012658	0.348953	0.697617	None
10833	GDAP1	0.162807	0.349134	0.697837	None
10834	CD80	-0.09471	0.349185	0.697837	None
10835	GEMIN7	-0.17431	0.349211	0.697837	None
10836	FAHD2A	-0.0894	0.349221	0.697837	None
10837	IL9R	-0.03393	0.349225	0.697837	None
10838	PRSS3	0.098912	0.349313	0.697945	None
10839	GPT	-0.03593	0.349343	0.697945	None
10840	TRIL	0.011378	0.349419	0.698033	None
10841	C4orf36	-0.01202	0.349482	0.69804	None
10842	TPSD1	-0.03913	0.349547	0.69804	None
10843	FBN2	-0.07514	0.349548	0.69804	None
10844	EGR4	-0.11296	0.349552	0.69804	None

10845	MAP3K10	-0.04269	0.349633	0.698137	None
10846	CLIP3	-0.05447	0.34968	0.698159	None
10847	MICAL2	-0.18048	0.349708	0.698159	None
10848	PIGN	0.148902	0.349894	0.698465	None
10849	C7orf13	0.157074	0.35	0.698555	None
10850	CYB5R4	-0.12817	0.350003	0.698555	None
10851	RPS6KB1	0.083722	0.350075	0.698634	None
10852	ARSD	-0.17469	0.350117	0.698653	None
10853	ZNF507	-0.10375	0.350163	0.698681	None
10854	ZNF439	-0.235	0.350248	0.69875	None
10855	LOC73020:	-0.0505	0.350262	0.69875	None
10856	SPX	-0.21602	0.350335	0.698773	None
10857	PABPC3	-0.07203	0.350338	0.698773	None
10858	BC012193	-0.0643	0.350394	0.69882	None
10859	SLC2A12	0.022587	0.350427	0.69882	None
10860	PINK1-AS	0.121724	0.350514	0.69893	None
10861	TMEM237	0.137043	0.350577	0.698991	None
10862	CTD-2286I	-0.14658	0.350797	0.699298	None
10863	ABCF2	0.102026	0.35082	0.699298	None
10864	C1orf127	-0.0215	0.350846	0.699298	None
10865	NR2F1-AS:	0.031339	0.350875	0.699298	None
10866	MAGED1	0.126982	0.350892	0.699298	None
10867	C5orf22	-0.07962	0.351062	0.699501	None
10868	SNPH	-0.0753	0.35111	0.699501	None
10869	BC047615	0.003936	0.351141	0.699501	None
10870	GAFA2	0.008093	0.351149	0.699501	None
10871	PRORY	-0.02064	0.351155	0.699501	None
10872	UCN3	0.068365	0.351244	0.699613	None
10873	TLR4	-0.31207	0.351295	0.699649	None
10874	HNRNPAB	0.109441	0.351381	0.699698	None
10875	EPC2	-0.13179	0.351384	0.699698	None
10876	ASH1L	0.114428	0.35144	0.699746	None
10877	CCT2	0.106803	0.35161	0.699986	None
10878	FAM104A	-0.12081	0.351625	0.699986	None
10879	KATNAL2	-0.07183	0.351694	0.699993	None
10880	CHCHD1	-0.1173	0.351724	0.699993	None
10881	THAP9	-0.10165	0.351726	0.699993	None
10882	RASSF8-AS	-0.06836	0.351786	0.700049	None
10883	ACCS	-0.15335	0.352013	0.700436	None
10884	RP5-1136(	0.191065	0.35209	0.700514	None
10885	PDLIM4	-0.0104	0.352117	0.700514	None
10886	RP11-454F	-0.17155	0.35218	0.700574	None
10887	TEX35	0.033229	0.352242	0.700617	None
10888	CD5	-0.05857	0.352274	0.700617	None
10889	ZNF221	-0.01552	0.352352	0.700617	None
10890	CLUL1	-0.01471	0.352376	0.700617	None
10891	LOC10192	-0.0195	0.352391	0.700617	None
10892	MRC1	-0.50953	0.352424	0.700617	None
10893	SON	-0.16029	0.352427	0.700617	None
10894	DNM1P46	-0.01967	0.352691	0.701025	None
10895	GOLT1A	-0.02134	0.352743	0.701025	None
10896	SLC17A2	-0.01507	0.352843	0.701025	None
10897	HCN3	-0.05363	0.352855	0.701025	None
10898	SRRM5	-0.04005	0.352866	0.701025	None
10899	ZSCAN32	-0.0664	0.352907	0.701025	None
10900	RBM41	-0.19722	0.352916	0.701025	None
10901	TAF5L	0.092516	0.352945	0.701025	None
10902	RP11-184E	-0.01941	0.352966	0.701025	None

10903	TM4SF18	-0.30978	0.352994	0.701025	None
10904	CCDC50	0.284553	0.353013	0.701025	None
10905	LOC10192	-0.0143	0.353021	0.701025	None
10906	DNAJB12	0.074233	0.353174	0.701265	None
10907	ZNF619	-0.03705	0.353224	0.701299	None
10908	AMMECR1	0.122932	0.353774	0.702325	None
10909	DUSP9	-0.03507	0.353822	0.702342	None
10910	PODN	-0.00972	0.353909	0.702342	None
10911	CACNA2D	-0.09875	0.35391	0.702342	None
10912	DSTN	-0.21614	0.353912	0.702342	None
10913	TSPYL6	-0.01535	0.354028	0.702508	None
10914	C3orf62	-0.03157	0.354101	0.70259	None
10915	TAS2R10	0.067445	0.354305	0.702909	None
10916	SLC22A25	-0.01228	0.354327	0.702909	None
10917	RASSF10	-0.00422	0.354384	0.702958	None
10918	TUBA8	-0.03654	0.354507	0.703083	None
10919	ISLR2	-0.01369	0.354553	0.703083	None
10920	MREG	0.184357	0.354599	0.703083	None
10921	ZCCHC11	-0.14976	0.354601	0.703083	None
10922	RP11-403F	0.12737	0.35461	0.703083	None
10923	VDAC3	-0.1168	0.354758	0.703152	None
10924	TET2	-0.17725	0.354758	0.703152	None
10925	LINC00944	-0.00622	0.354766	0.703152	None
10926	CDC6	0.293438	0.354808	0.703152	None
10927	SMIM2	0.015151	0.354833	0.703152	None
10928	ITGB7	-0.23924	0.354839	0.703152	None
10929	DYRK1A	0.008676	0.354901	0.703191	None
10930	QSOX1	-0.08753	0.354938	0.703191	None
10931	SLC39A6	0.112363	0.354958	0.703191	None
10932	LOC10042	0.012966	0.354989	0.703191	None
10933	LOC10028	0.013948	0.355029	0.703207	None
10934	SCN9A	-0.23854	0.355128	0.70321	None
10935	FN1	0.182087	0.355179	0.70321	None
10936	LOC10050	-0.12191	0.355241	0.70321	None
10937	TARDBP	0.105783	0.355253	0.70321	None
10938	ZNF653	-0.02419	0.355254	0.70321	None
10939	NTSR1	-0.01721	0.355276	0.70321	None
10940	DNAJC9	0.152805	0.355287	0.70321	None
10941	DNASE2B	-0.07826	0.355321	0.70321	None
10942	ZNF230	-0.20256	0.355344	0.70321	None
10943	GDF15	-0.03612	0.355356	0.70321	None
10944	OSCAR	-0.15018	0.355482	0.703365	None
10945	SMU1	-0.11968	0.355499	0.703365	None
10946	CLTCL1	-0.03547	0.3556	0.703365	None
10947	PSD2	0.051549	0.355603	0.703365	None
10948	R3HDM4	-0.16871	0.35561	0.703365	None
10949	LINC00347	-0.01793	0.355629	0.703365	None
10950	MUC12	0.00574	0.355713	0.703467	None
10951	COX6C	-0.12908	0.355756	0.703488	None
10952	LOC28585	-0.0276	0.355855	0.70362	None
10953	AHCTF1	0.134788	0.355968	0.703778	None
10954	KLC4	-0.03727	0.356094	0.703964	None
10955	NDUFS3	0.119598	0.356162	0.704033	None
10956	SLC39A1	-0.10679	0.356245	0.704111	None
10957	MRE11A	0.180432	0.356266	0.704111	None
10958	PPP2R1A	0.206971	0.356322	0.704111	None
10959	STRAP	-0.11537	0.356331	0.704111	None
10960	SETD4	-0.15005	0.356396	0.704123	None

10961	LOC10192!	-0.00601	0.356402	0.704123	None
10962	LOC10192!	-0.01247	0.356516	0.704283	None
10963	RPS18	-0.03458	0.356642	0.704469	None
10964	LOC10050!	-0.05516	0.356713	0.704545	None
10965	G3BP2	0.124404	0.356754	0.704562	None
10966	DBIL5P	-0.05659	0.356854	0.704694	None
10967	YWHAEP7	0.004857	0.357026	0.704968	None
10968	RBM20	-0.01094	0.357101	0.705053	None
10969	TMEM107	-0.16704	0.357422	0.705518	None
10970	ANKLE2	-0.12456	0.357456	0.705518	None
10971	ZNF571-A!	0.123197	0.357459	0.705518	None
10972	PKMYT1	-0.1433	0.357498	0.705518	None
10973	RP13-270F	-0.13637	0.357529	0.705518	None
10974	C19orf24	-0.12756	0.357532	0.705518	None
10975	LOC10099(	-0.01137	0.357627	0.705629	None
10976	LOC10050(	-0.2445	0.357653	0.705629	None
10977	C4BPB	-0.03495	0.357756	0.705768	None
10978	ITM2C	0.191404	0.357819	0.705828	None
10979	CASC1	-0.05075	0.357952	0.706025	None
10980	POLR1C	-0.13679	0.357986	0.706028	None
10981	RAB11B-A!	-0.06798	0.358154	0.706295	None
10982	ADPRH	-0.04245	0.358232	0.706384	None
10983	RP1-286D(	-0.02456	0.358274	0.706403	None
10984	SGPP2	-0.01793	0.35841	0.706607	None
10985	S1PR2	0.050975	0.358508	0.706735	None
10986	MTA3	-0.06684	0.358572	0.706797	None
10987	ARR3	-0.03367	0.358706	0.706998	None
10988	PI16	-0.07263	0.358753	0.707025	None
10989	KIAA1755	-0.00445	0.358832	0.707117	None
10990	CD164	-0.20801	0.358883	0.70713	None
10991	MTBP	-0.01317	0.358916	0.70713	None
10992	GFI1	0.28266	0.358937	0.70713	None
10993	FMO5	-0.1547	0.358987	0.707165	None
10994	ZNF497	-0.01192	0.359035	0.707173	None
10995	IER5	0.195506	0.359056	0.707173	None
10996	SPTY2D1-/	0.032793	0.359128	0.707193	None
10997	PSMD5-AS	-0.13642	0.359132	0.707193	None
10998	KRT15	0.022582	0.359198	0.70726	None
10999	MFN2	0.05258	0.359263	0.707323	None
11000	SLC1A3	-0.21419	0.359389	0.707506	None
11001	KLHL8	-0.15959	0.359433	0.707528	None
11002	ZNF738	0.136191	0.359504	0.707584	None
11003	UST	-0.09251	0.359526	0.707584	None
11004	ACKR3	0.168435	0.359583	0.707631	None
11005	KMO	-0.39476	0.359701	0.70769	None
11006	PTPRN2	-0.10711	0.359706	0.70769	None
11007	BTNL2	-0.00936	0.359711	0.70769	None
11008	WAC	-0.11142	0.359827	0.707793	None
11009	CXXC4	-0.0145	0.359833	0.707793	None
11010	NLGN2	-0.00877	0.359861	0.707793	None
11011	TROVE2	-0.06607	0.360076	0.70815	None
11012	LOC64320	-0.14255	0.360111	0.708154	None
11013	ASPHD2	-0.14864	0.36023	0.708325	None
11014	RP11-793H	-0.10497	0.360366	0.708528	None
11015	CDC14B	-0.08831	0.360457	0.708642	None
11016	ABCB7	0.218531	0.360554	0.708769	None
11017	RPS11	0.197342	0.360631	0.708777	None
11018	PLXNA2	-0.0334	0.360657	0.708777	None

11019	LOC65358:	-0.01567	0.360657	0.708777	None
11020	PACRG	-0.02244	0.360722	0.708794	None
11021	ADAMTS9-	0.001259	0.36073	0.708794	None
11022	ZNF462	-0.19664	0.360806	0.708879	None
11023	LOC10050(	-0.16235	0.360844	0.708888	None
11024	TRIM4	-0.1323	0.360898	0.708913	None
11025	PARP11	-0.20565	0.360922	0.708913	None
11026	SF3A3	-0.12004	0.36107	0.70914	None
11027	LOC65299:	-0.0262	0.361181	0.709265	None
11028	ABCG2	0.304926	0.3612	0.709265	None
11029	GAL3ST3	-0.0163	0.361254	0.709307	None
11030	PMS1	-0.1009	0.361328	0.709359	None
11031	BICD1	-0.1311	0.36136	0.709359	None
11032	MURC	-0.05562	0.361452	0.709359	None
11033	C1orf200	0.045761	0.361456	0.709359	None
11034	TYMP	-0.1347	0.36146	0.709359	None
11035	CPE	0.061681	0.361503	0.709359	None
11036	LINC00610	-0.05203	0.361532	0.709359	None
11037	NRG3	0.013459	0.361542	0.709359	None
11038	LRCH3	-0.23869	0.361599	0.709407	None
11039	GTF2IRD1	0.097769	0.361697	0.709534	None
11040	ADRB1	-0.01744	0.361768	0.709541	None
11041	PI4KA	-0.13614	0.36177	0.709541	None
11042	RP3-384D2	0.015332	0.361802	0.709541	None
11043	OR51I1	-0.01063	0.361832	0.709541	None
11044	IDUA	-0.02528	0.36208	0.709957	None
11045	KANK1	0.066293	0.362136	0.709957	None
11046	CPXM1	-0.19794	0.362165	0.709957	None
11047	EML5	-0.01706	0.362189	0.709957	None
11048	TMEM125	-0.09794	0.362208	0.709957	None
11049	GALNT9	-0.01506	0.362324	0.710121	None
11050	LOC10192ε	-0.02209	0.362498	0.710397	None
11051	SLC6A15	-0.07673	0.362673	0.710545	None
11052	MAP3K14-	-0.01857	0.362689	0.710545	None
11053	KIRREL3	-0.01492	0.362704	0.710545	None
11054	CAPNS1	-0.21377	0.362705	0.710545	None
11055	LOC10272	-0.06403	0.362739	0.710549	None
11056	ACY1	-0.0154	0.362837	0.710677	None
11057	LINC00698	-0.02814	0.362963	0.710859	None
11058	LINC01101	-0.01354	0.363056	0.710976	None
11059	COLQ	-0.12044	0.363281	0.711352	None
11060	CTAGE5	0.198773	0.363396	0.711515	None
11061	FOXS1	-0.00817	0.363552	0.711756	None
11062	C17orf80	-0.11485	0.363716	0.712011	None
11063	RP11-480I	0.015087	0.363861	0.712044	None
11064	LOC10065	0.044591	0.363879	0.712044	None
11065	SEC63	0.132642	0.363894	0.712044	None
11066	ODF3L2	-0.01566	0.363908	0.712044	None
11067	PXYLP1	-0.13145	0.363919	0.712044	None
11068	RAB26	0.008585	0.363956	0.712044	None
11069	HLA-B	-0.17928	0.363963	0.712044	None
11070	C17orf51	-0.04691	0.364008	0.712068	None
11071	ZNF273	-0.17378	0.364084	0.712141	None
11072	SLC44A5	-0.06352	0.364111	0.712141	None
11073	VENTXP1	-0.00667	0.36426	0.712367	None
11074	SEC61B	0.126166	0.364428	0.712631	None
11075	LOC10192	0.217344	0.364495	0.712699	None
11076	LOC15756:	-0.12158	0.364699	0.713032	None

11077	KIR2DL2	-0.03214	0.364973	0.713505	None
11078	LOC10050	-0.00705	0.365183	0.71385	None
11079	POLR2J	-0.20468	0.365375	0.714063	None
11080	GJA1	-0.58412	0.365378	0.714063	None
11081	ATP1B2	-0.0571	0.365414	0.714063	None
11082	GUSBP2	-0.15241	0.365457	0.714063	None
11083	AF198444	-0.0293	0.365474	0.714063	None
11084	C1QA	-0.68031	0.365489	0.714063	None
11085	ZNF33B	-0.13422	0.365596	0.714207	None
11086	POLQ	0.25274	0.365636	0.71422	None
11087	KIAA1161	0.015411	0.365746	0.71436	None
11088	ERBB3	-0.00882	0.365794	0.71436	None
11089	ELF1	0.092547	0.365896	0.71436	None
11090	SNTA1	-0.06934	0.365921	0.71436	None
11091	DCTN4	-0.19361	0.36594	0.71436	None
11092	PCID2	-0.10409	0.36595	0.71436	None
11093	HPN	-0.01906	0.365968	0.71436	None
11094	ALPPL2	-0.02541	0.366033	0.71436	None
11095	SOCS3	0.097278	0.36607	0.71436	None
11096	MCC	-0.14446	0.36608	0.71436	None
11097	MYL12B	0.104798	0.366097	0.71436	None
11098	ANKRD19F	-0.04571	0.366123	0.71436	None
11099	PHLDA3	-0.02547	0.366159	0.71436	None
11100	RP5-1065J	0.087216	0.366169	0.71436	None
11101	PARD6G	-0.03568	0.366227	0.714372	None
11102	ATXN7L2	-0.01465	0.36626	0.714372	None
11103	HMOX1	-0.72729	0.36631	0.714372	None
11104	LINC00905	-0.01089	0.366311	0.714372	None
11105	FCGR1B	-0.32941	0.366352	0.714372	None
11106	RNF5	0.034498	0.366413	0.714372	None
11107	LRP8	-0.17634	0.366438	0.714372	None
11108	LOC10099	-0.15505	0.36648	0.714372	None
11109	COL6A2	-0.0686	0.366487	0.714372	None
11110	LINC00880	-0.0154	0.366533	0.714372	None
11111	COX7B	-0.14494	0.366587	0.714372	None
11112	CTH	-0.1238	0.366599	0.714372	None
11113	CEP250	-0.03458	0.366604	0.714372	None
11114	FANK1	-0.11378	0.366725	0.714543	None
11115	RP4-657D1	0.059239	0.366827	0.714678	None
11116	RP11-495K	-0.06637	0.366895	0.714745	None
11117	F10	-0.01357	0.366941	0.71477	None
11118	DUT	-0.14172	0.367017	0.714854	None
11119	JAG1	-0.10945	0.36709	0.714878	None
11120	LDHAL6A	-0.10415	0.367111	0.714878	None
11121	TFAP2D	-0.01713	0.367128	0.714878	None
11122	BC030152	0.202725	0.367168	0.714886	None
11123	LOC10192	-0.02368	0.367198	0.714886	None
11124	TRIO	0.079157	0.367318	0.715055	None
11125	FGFR4	0.011317	0.367528	0.71535	None
11126	NEDD9	0.041582	0.367536	0.71535	None
11127	ANKRD36C	-0.17552	0.367646	0.71541	None
11128	EMC7	0.054197	0.367649	0.71541	None
11129	LONRF1	0.230927	0.367666	0.71541	None
11130	KRTAP4-5	0.027217	0.367719	0.71545	None
11131	GPBAR1	-0.11414	0.367878	0.715633	None
11132	APOM	0.127074	0.367879	0.715633	None
11133	TRG-AS1	0.407711	0.367934	0.715676	None
11134	SLCO1B1	-0.01038	0.368088	0.715911	None

11135	UBC	0.088236	0.368173	0.71594	None
11136	KRT4	-0.00539	0.368215	0.71594	None
11137	ANXA6	-0.09527	0.36824	0.71594	None
11138	FBLIM1	0.037465	0.368254	0.71594	None
11139	PLD5	0.006903	0.368269	0.71594	None
11140	ADAM6	-0.00744	0.368454	0.716195	None
11141	SPA17	0.211579	0.368466	0.716195	None
11142	DNAI2	0.018273	0.368531	0.716247	None
11143	SIGLEC11	-0.14554	0.368566	0.716247	None
11144	ERGIC1	0.14615	0.368592	0.716247	None
11145	KRT31	0.020472	0.368723	0.71638	None
11146	LOC40106‹	-0.02636	0.368726	0.71638	None
11147	NXF5	-0.01556	0.368766	0.716392	None
11148	POFUT2	0.019476	0.368803	0.716401	None
11149	CYP11B2	-0.07656	0.368898	0.716521	None
11150	SULT1C4	0.13255	0.369049	0.716733	None
11151	MS4A5	-0.01236	0.369074	0.716733	None
11152	ERCC6L2	0.111347	0.369167	0.716851	None
11153	SLC1A1	-0.02503	0.369216	0.716864	None
11154	TNFAIP1	-0.09237	0.36924	0.716864	None
11155	CTC-428G	-0.04526	0.369333	0.716965	None
11156	INHBC	0.025441	0.369373	0.716965	None
11157	SRGAP2	0.249145	0.369392	0.716965	None
11158	MRPL36	-0.13181	0.369572	0.71725	None
11159	TEX13A	-0.0055	0.369678	0.717365	None
11160	STAT4	0.40851	0.369762	0.717365	None
11161	FAM83C-A	-0.0192	0.369778	0.717365	None
11162	MRPS15	-0.10356	0.369817	0.717365	None
11163	KIAA1751	0.008742	0.369829	0.717365	None
11164	ITGA1	-0.01113	0.36983	0.717365	None
11165	TMA7	0.077372	0.369906	0.717449	None
11166	BDH1	-0.08566	0.370142	0.717841	None
11167	ERP27	0.222585	0.370267	0.71802	None
11168	LOC28517‹	0.192799	0.370399	0.718212	None
11169	AACSP1	0.007704	0.3705	0.718342	None
11170	SIN3A	0.119011	0.370569	0.718395	None
11171	NXNL1	-0.02364	0.370601	0.718395	None
11172	SPATA6	0.088822	0.370626	0.718395	None
11173	C11orf80	0.114637	0.370667	0.7184	None
11174	LOC10050(	-0.04974	0.370732	0.7184	None
11175	HADH	-0.12912	0.370745	0.7184	None
11176	DKFZp667I	0.119757	0.370799	0.7184	None
11177	METTL18	-0.20828	0.370825	0.7184	None
11178	ACTL6B	-0.00907	0.370828	0.7184	None
11179	RP11-44F1	-0.03952	0.370876	0.718428	None
11180	MEF2C-AS	-0.06406	0.371026	0.718656	None
11181	LINC00882	0.012167	0.371072	0.718681	None
11182	SYCP2L	-0.13259	0.37116	0.718786	None
11183	LOC10050(	-0.00952	0.371221	0.718818	None
11184	SHROOM4	-0.05347	0.371243	0.718818	None
11185	ZNF830	0.102315	0.37129	0.718844	None
11186	COX5B	-0.12517	0.371406	0.718997	None
11187	RGS8	0.013451	0.371435	0.718997	None
11188	GAREM	-0.06649	0.371473	0.719006	None
11189	LA16c-380	-0.0392	0.371521	0.71901	None
11190	ZSWIM5	-0.07747	0.371567	0.71901	None
11191	ERCC6	-0.07471	0.371589	0.71901	None
11192	LOC10192	-0.03277	0.371637	0.71901	None

11193	SNX12	-0.0617	0.371641	0.71901	None
11194	NPM3	0.266255	0.371799	0.719253	None
11195	RAF1	-0.11748	0.371911	0.719384	None
11196	MSH2	-0.12584	0.371934	0.719384	None
11197	KCNMB1	-0.05858	0.372002	0.719452	None
11198	CWC22	0.151333	0.372083	0.719537	None
11199	AC005523.	0.045569	0.372113	0.719537	None
11200	TMEM181	-0.12413	0.372145	0.719537	None
11201	FRMD4B	0.031076	0.372218	0.719612	None
11202	DNAJB14	0.153494	0.372385	0.719756	None
11203	ALDH1L2	-0.01092	0.372387	0.719756	None
11204	ITGB3	-0.21121	0.372392	0.719756	None
11205	PTRH1	-0.08569	0.372558	0.719975	None
11206	DSG2	-0.22552	0.372572	0.719975	None
11207	KIAA2018	-0.04108	0.372663	0.720044	None
11208	ACSM2A	-0.011	0.372674	0.720044	None
11209	LOC15148	-0.01266	0.372839	0.720299	None
11210	LRRN4	-0.04736	0.372906	0.720315	None
11211	C11orf84	-0.04783	0.372914	0.720315	None
11212	RP1-170O	-0.02506	0.373092	0.720583	None
11213	FOLH1B	-0.0754	0.373129	0.720583	None
11214	VWA9	0.073479	0.373153	0.720583	None
11215	RP11-217E	-0.04033	0.373375	0.720915	None
11216	LRFN1	-0.1169	0.373396	0.720915	None
11217	S100A14	0.022953	0.373432	0.720915	None
11218	ACTL8	-0.04861	0.373458	0.720915	None
11219	TIMM17B	-0.07249	0.373559	0.721046	None
11220	WDR90	0.068086	0.373732	0.721233	None
11221	NFIL3	0.272388	0.373735	0.721233	None
11222	GPR135	-0.01898	0.373756	0.721233	None
11223	RP11-65J3	0.057811	0.373829	0.721311	None
11224	KIAA1468	-0.17855	0.373948	0.721476	None
11225	KNDC1	0.001969	0.374055	0.721559	None
11226	NFYA	0.099313	0.374058	0.721559	None
11227	LINC01395	-0.00706	0.374145	0.721584	None
11228	LOC10192	-0.15374	0.374151	0.721584	None
11229	INMT	-0.07852	0.374228	0.721584	None
11230	LOC10050	-0.01913	0.374251	0.721584	None
11231	TTC17	-0.11324	0.374264	0.721584	None
11232	GPS2	-0.15028	0.374294	0.721584	None
11233	DPM3	0.128256	0.374304	0.721584	None
11234	SIRPD	0.030984	0.374353	0.721596	None
11235	GPR39	-0.00308	0.374377	0.721596	None
11236	LOC28066	0.004874	0.374429	0.721633	None
11237	RPS5	0.113907	0.374551	0.721803	None
11238	RASAL1	0.018226	0.374758	0.722138	None
11239	AQP5	-0.04366	0.374914	0.722178	None
11240	ADCK5	-0.09861	0.374944	0.722178	None
11241	RPL12	-0.06718	0.374958	0.722178	None
11242	CCDC101	-0.12363	0.374974	0.722178	None
11243	LOC40004	-0.09672	0.374994	0.722178	None
11244	PRKD3	0.164982	0.375011	0.722178	None
11245	NPBWR1	-0.02806	0.37503	0.722178	None
11246	MOGAT2	-0.00794	0.375046	0.722178	None
11247	MMP11	-0.00379	0.375228	0.722409	None
11248	ZNF24	0.147584	0.375232	0.722409	None
11249	BEST4	0.028879	0.375296	0.722467	None
11250	PCK1	-0.04464	0.375505	0.722476	None

11251	ISL2	-0.00776	0.375526	0.722476	None
11252	CTNNA3	-0.06681	0.375562	0.722476	None
11253	PRKAB1	-0.13491	0.375569	0.722476	None
11254	PLXNB1	-0.0358	0.375598	0.722476	None
11255	SIGLEC17P	-0.21464	0.375604	0.722476	None
11256	EXOC5	-0.19648	0.375606	0.722476	None
11257	PLAGL1	-0.19853	0.375625	0.722476	None
11258	EIF2B2	-0.11606	0.375664	0.722476	None
11259	IQCF2	-0.01091	0.37567	0.722476	None
11260	METAP2	0.116152	0.375698	0.722476	None
11261	C5orf47	-0.00295	0.375701	0.722476	None
11262	SEC11A	-0.08901	0.375734	0.722476	None
11263	CHRNA2	0.070795	0.37583	0.722552	None
11264	ITGAX	-0.0909	0.375841	0.722552	None
11265	C16orf45	-0.06191	0.375949	0.722677	None
11266	ZNF654	0.151657	0.375972	0.722677	None
11267	C3orf22	0.017435	0.376038	0.72274	None
11268	BCMO1	-0.03141	0.376083	0.722761	None
11269	SENP7	0.12088	0.37618	0.722845	None
11270	MPPE1	-0.10209	0.376251	0.722845	None
11271	LOC10050	-0.07767	0.376263	0.722845	None
11272	AMIGO2	0.33061	0.37627	0.722845	None
11273	CD3D	0.228901	0.376293	0.722845	None
11274	FLJ31715	-0.04041	0.376481	0.723142	None
11275	SLC23A1	0.195987	0.376821	0.723675	None
11276	LINC00894	-0.03534	0.376956	0.723675	None
11277	AGTPBP1	0.141298	0.37696	0.723675	None
11278	SMC5	-0.19858	0.37697	0.723675	None
11279	PEX1	0.104604	0.376989	0.723675	None
11280	ZFYVE16	0.242232	0.376992	0.723675	None
11281	TDRD9	0.348172	0.377003	0.723675	None
11282	OVOL1-AS	-0.04549	0.377026	0.723675	None
11283	PLD4	-0.13771	0.377176	0.723899	None
11284	C8orf88	0.235597	0.377265	0.724005	None
11285	C1orf111	-0.01728	0.377584	0.724554	None
11286	CEACAM19	-0.03667	0.377676	0.724593	None
11287	SCARF1	-0.1168	0.377715	0.724593	None
11288	LDB2	-0.17289	0.377715	0.724593	None
11289	DLG3-AS1	-0.01966	0.377773	0.724593	None
11290	NCAPD2	0.195599	0.377782	0.724593	None
11291	ENO1	-0.19145	0.377806	0.724593	None
11292	ZNF121	0.121343	0.377909	0.724658	None
11293	LOC10192	0.007114	0.377922	0.724658	None
11294	PANK4	-0.13969	0.37794	0.724658	None
11295	PSG4	-0.01263	0.377988	0.724687	None
11296	PPP1CC	0.084203	0.378023	0.724689	None
11297	CFC1	-0.01046	0.378091	0.724711	None
11298	CLEC10A	-0.22678	0.378184	0.724711	None
11299	EFCAB12	-0.02153	0.378188	0.724711	None
11300	OXCT1	0.150569	0.378222	0.724711	None
11301	PIGL	0.089783	0.37823	0.724711	None
11302	GPR128	-0.24775	0.378237	0.724711	None
11303	MUT	0.118033	0.378269	0.724711	None
11304	APIP	0.062965	0.378397	0.724893	None
11305	RP11-843E	0.126422	0.378529	0.72501	None
11306	GTF2F2	-0.11126	0.37856	0.72501	None
11307	SLC14A1	-0.22135	0.378562	0.72501	None
11308	RP11-334C	-0.04275	0.378592	0.72501	None

11309	VPS16	-0.05612	0.378661	0.725077	None
11310	DACT3	-0.0123	0.378711	0.72511	None
11311	LELP1	0.023091	0.378847	0.725306	None
11312	PSTK	-0.07084	0.378912	0.725362	None
11313	ARSA	-0.03744	0.378943	0.725362	None
11314	RPS8	-0.02213	0.378993	0.725393	None
11315	EPHA5-AS	-0.04766	0.379206	0.725736	None
11316	ABHD8	-0.0523	0.379254	0.725764	None
11317	STK4	0.172021	0.379384	0.725948	None
11318	BFSP2-AS1	-0.00772	0.379492	0.72609	None
11319	ASB9	0.210716	0.379574	0.726185	None
11320	C10orf71-/	-0.01863	0.379677	0.726316	None
11321	BLACAT1	-0.01819	0.379755	0.726363	None
11322	RP11-375I:	-0.00238	0.379768	0.726363	None
11323	KCNK3	-0.00689	0.379897	0.726544	None
11324	COL27A1	-0.03787	0.379973	0.726627	None
11325	SLC6A3	-0.01487	0.380102	0.726702	None
11326	MUC5B	-0.01307	0.380113	0.726702	None
11327	RSRC1	0.148283	0.380151	0.726702	None
11328	LOC64253:	0.066747	0.38016	0.726702	None
11329	PEF1	-0.11439	0.380181	0.726702	None
11330	RCOR2	0.046611	0.380603	0.727445	None
11331	TMEM163	0.274034	0.380649	0.727458	None
11332	SMEK1	0.166624	0.380677	0.727458	None
11333	CLEC5A	0.233679	0.380773	0.727554	None
11334	MIB1	0.118767	0.380794	0.727554	None
11335	MINPP1	-0.24536	0.38084	0.727577	None
11336	CTD-2587I	-0.01125	0.380939	0.727598	None
11337	RP11-210k	0.025103	0.38096	0.727598	None
11338	GTSF1	0.580889	0.380968	0.727598	None
11339	FGF4	-0.01144	0.381002	0.727598	None
11340	LRRC16B	0.07513	0.38103	0.727598	None
11341	CREG2	-0.01369	0.381053	0.727598	None
11342	MAP3K13	-0.03139	0.381176	0.72777	None
11343	NACC1	-0.05259	0.381242	0.727827	None
11344	OTULIN	0.110055	0.381283	0.727827	None
11345	LOC10192!	0.170595	0.381334	0.727827	None
11346	FAM132A	0.056105	0.381341	0.727827	None
11347	NPFF	-0.0756	0.381406	0.727858	None
11348	SH2B3	0.190273	0.381424	0.727858	None
11349	ERICH4	0.036343	0.381501	0.727941	None
11350	HAUS7	0.133952	0.381591	0.728049	None
11351	HSPB2	-0.02912	0.381708	0.72814	None
11352	IKBIP	-0.15763	0.381749	0.72814	None
11353	NCOA2	-0.11708	0.381767	0.72814	None
11354	CMTM6	0.077163	0.381825	0.72814	None
11355	CES4A	-0.04602	0.381833	0.72814	None
11356	FAM208B	0.134171	0.381865	0.72814	None
11357	LOC10050!	-0.00632	0.381882	0.72814	None
11358	LOC64223(	-0.21116	0.381925	0.72814	None
11359	SSX3	0.011085	0.381942	0.72814	None
11360	BC062763	-0.03234	0.382039	0.728262	None
11361	STYX	-0.12746	0.382143	0.728396	None
11362	RAD54L	0.167086	0.382183	0.728407	None
11363	AC091633.	-0.02538	0.382243	0.728415	None
11364	RP4-758J2	0.146831	0.382262	0.728415	None
11365	TTC9	-0.09015	0.382288	0.728415	None
11366	OR3A2	-0.00764	0.382349	0.728466	None

11367	FOXD2-AS	-0.01808	0.382454	0.728466	None
11368	EXD1	-0.00891	0.382459	0.728466	None
11369	CTD-2033(	-0.00566	0.382493	0.728466	None
11370	TCEA2	0.091202	0.382502	0.728466	None
11371	ELK4	0.108298	0.382516	0.728466	None
11372	LOC10192!	0.039416	0.382575	0.728513	None
11373	RP11-468E	0.231142	0.382612	0.728513	None
11374	APOA2	-0.01253	0.382644	0.728513	None
11375	CEP44	-0.11378	0.382731	0.728513	None
11376	LOC10192	-0.00705	0.38274	0.728513	None
11377	GRM2	0.030833	0.382743	0.728513	None
11378	DLL3	-0.00935	0.382814	0.728585	None
11379	BAZ1B	-0.09853	0.382911	0.728651	None
11380	ITGB2	-0.22588	0.382916	0.728651	None
11381	C5orf15	0.070273	0.383024	0.728792	None
11382	DQ592442	-0.24462	0.383074	0.728823	None
11383	IDH2	0.122333	0.383224	0.728967	None
11384	TFEB	-0.12495	0.383237	0.728967	None
11385	LOC25304	-0.01835	0.38325	0.728967	None
11386	ADAMTSL	0.038233	0.38332	0.729035	None
11387	CACNB3	-0.13205	0.383498	0.72929	None
11388	SLC25A26	-0.12643	0.383538	0.72929	None
11389	DNAJB1	-0.15251	0.383555	0.72929	None
11390	GIMAP7	-0.35453	0.383589	0.72929	None
11391	LOC28421	0.10605	0.383642	0.729326	None
11392	OR51B2	0.023115	0.383693	0.72936	None
11393	SENP1	0.065501	0.383817	0.729525	None
11394	TUBE1	-0.10544	0.383893	0.729525	None
11395	MAP3K4	-0.08206	0.383901	0.729525	None
11396	FRY-AS1	-0.03501	0.383989	0.729525	None
11397	KRT19	-0.01388	0.383994	0.729525	None
11398	DNAJB7	-0.04316	0.384024	0.729525	None
11399	RP11-298H	-0.01086	0.384026	0.729525	None
11400	TSPAN16	-0.01944	0.384053	0.729525	None
11401	RREB1	-0.10802	0.384083	0.729525	None
11402	ATP6V0D1	-0.17917	0.384204	0.729669	None
11403	LOC10012!	0.019785	0.384254	0.729669	None
11404	CUL1	0.123133	0.384323	0.729669	None
11405	FZD5	-0.11476	0.384337	0.729669	None
11406	CDC42EP5	-0.01612	0.38436	0.729669	None
11407	LOC10012!	-0.01216	0.384489	0.729669	None
11408	LOC28304!	0.005184	0.384532	0.729669	None
11409	FAM200B	-0.13646	0.384572	0.729669	None
11410	IL10RA	-0.39718	0.384583	0.729669	None
11411	ARHGAP12	-0.2037	0.384587	0.729669	None
11412	ASNSD1	-0.07977	0.384596	0.729669	None
11413	ATP6V1E2	-0.13788	0.384597	0.729669	None
11414	SNX17	0.12826	0.384625	0.729669	None
11415	MYPN	-0.02353	0.384706	0.729669	None
11416	IL22RA1	0.017146	0.384712	0.729669	None
11417	BCL2L1	-0.08115	0.384734	0.729669	None
11418	7-Sep	0.133479	0.384744	0.729669	None
11419	PMVK	-0.16317	0.384765	0.729669	None
11420	CSDC2	0.025343	0.384991	0.730033	None
11421	SMAD5	-0.21052	0.385049	0.73008	None
11422	TRPM8	-0.01831	0.385199	0.730299	None
11423	SNORA65	0.043024	0.385265	0.730362	None
11424	NCS1	-0.01779	0.385305	0.730373	None

11425	RPS15	0.031087	0.385634	0.730885	None
11426	RCE1	-0.03651	0.385669	0.730885	None
11427	PDE8B	-0.06334	0.385685	0.730885	None
11428	KCNK12	-0.07648	0.38571	0.730885	None
11429	ADRA2B	-0.02956	0.385752	0.7309	None
11430	LILRA5	-0.09062	0.3858	0.730928	None
11431	PIGR	-0.00799	0.385924	0.73106	None
11432	BC048420	-0.02372	0.385942	0.73106	None
11433	SLC18A1	-0.00908	0.385972	0.73106	None
11434	DAZL	0.011933	0.386141	0.731317	None
11435	FOS	-0.25904	0.386177	0.731323	None
11436	LOC25405	0.224548	0.386301	0.731361	None
11437	TCP11L1	0.06383	0.38631	0.731361	None
11438	CHI3L2	-0.12377	0.386328	0.731361	None
11439	IGLC1	-0.18393	0.386333	0.731361	None
11440	LOC10099	-0.01473	0.386387	0.731399	None
11441	KCTD18	0.093962	0.386505	0.731559	None
11442	LOC10013	-0.00573	0.386735	0.731912	None
11443	PDPK1	-0.01718	0.386759	0.731912	None
11444	C14orf37	-0.07043	0.386816	0.731956	None
11445	LEMD1	0.020828	0.387012	0.732177	None
11446	BTF3	-0.06999	0.387031	0.732177	None
11447	RP11-63K6	-0.02662	0.387034	0.732177	None
11448	DCLK3	0.025541	0.387136	0.732257	None
11449	DPH6-AS1	-0.0032	0.387152	0.732257	None
11450	RP11-245J	0.090853	0.387178	0.732257	None
11451	PDE12	0.083044	0.387229	0.732289	None
11452	SORL1	0.237245	0.387307	0.732312	None
11453	RP11-231C	0.021022	0.387349	0.732312	None
11454	KCNV1	-0.00713	0.387353	0.732312	None
11455	PNLIPRP3	-0.00721	0.387397	0.732312	None
11456	KCTD19	-0.00847	0.38741	0.732312	None
11457	LOC39990	-0.09272	0.387504	0.732376	None
11458	RP11-190A	-0.22711	0.387511	0.732376	None
11459	CPT2	-0.11696	0.38758	0.732441	None
11460	C3orf33	0.083326	0.387741	0.732682	None
11461	LRRC37A4I	-0.20351	0.387836	0.732684	None
11462	ALOX12P2	-0.0416	0.387867	0.732684	None
11463	SDCCAG3	-0.18544	0.387873	0.732684	None
11464	TBL1X	-0.05867	0.387878	0.732684	None
11465	OTP	-0.01575	0.387956	0.732697	None
11466	CD1E	-0.06281	0.387985	0.732697	None
11467	MYH14	-0.00201	0.388013	0.732697	None
11468	C10orf91	-0.01227	0.38802	0.732697	None
11469	FAM47C	-0.02176	0.388057	0.732697	None
11470	RNF25	-0.07863	0.388149	0.732697	None
11471	ENDOV	-0.03364	0.38824	0.732697	None
11472	COL4A2	-0.1474	0.388241	0.732697	None
11473	CHST1	0.006126	0.38826	0.732697	None
11474	GRIN2D	0.003356	0.38826	0.732697	None
11475	SAYSD1	-0.13795	0.388295	0.732697	None
11476	ITSN1	-0.03692	0.388318	0.732697	None
11477	LINC00577	0.044112	0.388335	0.732697	None
11478	ERCC8	0.072701	0.388358	0.732697	None
11479	DYM	-0.13282	0.388397	0.732706	None
11480	SPECC1	-0.09155	0.388498	0.73283	None
11481	RP11-373D	-0.33265	0.38853	0.73283	None
11482	DOCK4	-0.18979	0.388591	0.732881	None

11483	LYSMD4	-0.09237	0.388728	0.733075	None
11484	RP11-44N1	-0.02329	0.388839	0.733221	None
11485	REPS1	0.21249	0.388962	0.733358	None
11486	KLHDC1	0.110601	0.388979	0.733358	None
11487	IL36A	-0.01575	0.389035	0.733398	None
11488	PROS1	-0.38165	0.389123	0.733471	None
11489	ACTR3C	-0.05892	0.389141	0.733471	None
11490	HMMR-AS	-0.03817	0.389248	0.733608	None
11491	OSBPL7	-0.02295	0.389326	0.733693	None
11492	ESR2	-0.02217	0.38941	0.733786	None
11493	CCR6	-0.03095	0.389516	0.733923	None
11494	MAU2	-0.0286	0.389721	0.734245	None
11495	NR0B1	-0.03962	0.389894	0.734506	None
11496	SIGLEC6	-0.03327	0.390074	0.73474	None
11497	PDIA3	0.111866	0.390114	0.73474	None
11498	YIPF5	-0.18174	0.39015	0.73474	None
11499	MATN4	-0.07315	0.390188	0.73474	None
11500	ADARB1	0.044601	0.390217	0.73474	None
11501	GRB14	-0.21477	0.390221	0.73474	None
11502	PDE11A	0.002255	0.390262	0.734753	None
11503	FITM1	0.038702	0.390315	0.734785	None
11504	ADAM9	-0.17427	0.390389	0.734785	None
11505	ANK3	-0.1577	0.390411	0.734785	None
11506	PP13439	-0.12292	0.390415	0.734785	None
11507	SV2C	-0.02912	0.390554	0.734868	None
11508	TRPV6	-0.02456	0.39056	0.734868	None
11509	DCLK1	-0.07099	0.390561	0.734868	None
11510	ZNF792	-0.20935	0.39076	0.735108	None
11511	PCBP2	-0.11091	0.390813	0.735108	None
11512	TTLL7-IT1	-0.04062	0.390846	0.735108	None
11513	C1orf213	-0.12238	0.390855	0.735108	None
11514	C16orf86	-0.07973	0.390858	0.735108	None
11515	BMP4	-0.05216	0.390959	0.735183	None
11516	LINC01121	-0.03438	0.390966	0.735183	None
11517	TTTY5	0.038873	0.391003	0.735189	None
11518	MMP14	0.010223	0.391064	0.73519	None
11519	WI2-89031	-0.02073	0.391071	0.73519	None
11520	GTDC1	0.061879	0.391204	0.735316	None
11521	NEK11	-0.00418	0.391235	0.735316	None
11522	SMCR8	-0.09006	0.39124	0.735316	None
11523	ZNF611	0.063451	0.39135	0.735458	None
11524	LOC10013	-0.08461	0.391418	0.73548	None
11525	HES4	0.070024	0.39148	0.73548	None
11526	G2E3	0.100952	0.391485	0.73548	None
11527	BEND6	-0.16224	0.391508	0.73548	None
11528	HMGN4	0.107048	0.391552	0.73548	None
11529	PP12708	0.213187	0.391583	0.73548	None
11530	SP140L	0.10525	0.391599	0.73548	None
11531	SRPK1	0.062575	0.391862	0.7358	None
11532	AIDA	-0.12026	0.391874	0.7358	None
11533	HDAC1	-0.06543	0.391876	0.7358	None
11534	RASAL2	-0.01875	0.391905	0.7358	None
11535	LINC01159	-0.02654	0.391979	0.735873	None
11536	BTBD9	0.0222	0.392031	0.735873	None
11537	LINC00708	-0.01581	0.392046	0.735873	None
11538	VCL	-0.09098	0.392158	0.735981	None
11539	TSC22D4	-0.11696	0.392172	0.735981	None
11540	ETV7	-0.04779	0.392475	0.736426	None

11541	SLC29A4	0.009435	0.392483	0.736426	None
11542	SULT1E1	-0.00693	0.392541	0.736426	None
11543	SLC6A11	-0.03269	0.392605	0.736426	None
11544	KLHL30-AS	-0.01785	0.392706	0.736426	None
11545	ZNF259P1	-0.0122	0.392713	0.736426	None
11546	CNGB1	0.019419	0.392809	0.736426	None
11547	RP11-395I	0.215735	0.392875	0.736426	None
11548	HEPACAM	-0.13175	0.392894	0.736426	None
11549	LOC28460	-0.07286	0.392902	0.736426	None
11550	FAM83B	-0.01095	0.392946	0.736426	None
11551	CCDC186	0.182099	0.392955	0.736426	None
11552	SPSB4	-0.09148	0.392978	0.736426	None
11553	AC068039.	0.12377	0.392993	0.736426	None
11554	UFC1	0.076361	0.393007	0.736426	None
11555	MTDH	0.097796	0.393057	0.736426	None
11556	LRRC28	0.072328	0.393079	0.736426	None
11557	ELOVL7	-0.29663	0.393137	0.736426	None
11558	POF1B	-0.00404	0.39314	0.736426	None
11559	CCDC28B	-0.09582	0.39315	0.736426	None
11560	GARNL3	-0.01735	0.393198	0.736426	None
11561	F11	-0.00416	0.393243	0.736426	None
11562	RP11-1024	0.008679	0.393273	0.736426	None
11563	NFKBID	0.223656	0.39332	0.736426	None
11564	ATP2A3	-0.06253	0.393389	0.736426	None
11565	FIG4	-0.19756	0.393422	0.736426	None
11566	RP11-196C	0.205479	0.393511	0.736426	None
11567	AASS	0.233053	0.393556	0.736426	None
11568	ZSCAN20	-0.00458	0.393558	0.736426	None
11569	TP53TG5	-0.0256	0.393603	0.736426	None
11570	C1orf204	-0.01571	0.393613	0.736426	None
11571	RMDN2	-0.06559	0.393615	0.736426	None
11572	SPRNP1	-0.0159	0.393618	0.736426	None
11573	ACTR6	0.058367	0.393623	0.736426	None
11574	LINC00960	-0.22231	0.393644	0.736426	None
11575	ANAPC5	0.109787	0.393669	0.736426	None
11576	RPP38	-0.10471	0.393689	0.736426	None
11577	NRAS	-0.08179	0.393701	0.736426	None
11578	CLNS1A	-0.12182	0.393754	0.73644	None
11579	ZNF235	-0.03619	0.393777	0.73644	None
11580	CCDC40	0.008616	0.393967	0.736663	None
11581	MEIG1	-0.03957	0.393982	0.736663	None
11582	MOSPD1	-0.14087	0.393998	0.736663	None
11583	CD200R1	-0.07227	0.394123	0.73679	None
11584	ARG2	-0.24757	0.394134	0.73679	None
11585	SERPINB9F	0.184519	0.394218	0.736799	None
11586	LINC00302	0.00702	0.394259	0.736799	None
11587	NIPBL	-0.11624	0.394264	0.736799	None
11588	MAP2	-0.02944	0.394275	0.736799	None
11589	TIRAP	-0.02099	0.394323	0.736824	None
11590	LAIR1	0.173814	0.394524	0.737122	None
11591	ANP32B	0.049538	0.39455	0.737122	None
11592	DYNLRB2	-0.0413	0.394661	0.737216	None
11593	QSER1	0.120344	0.394683	0.737216	None
11594	FLJ38717	0.175737	0.394702	0.737216	None
11595	ERVFH21-1	0.028265	0.394757	0.737254	None
11596	CCDC88B	-0.05066	0.394873	0.737355	None
11597	LOC28437	-0.03382	0.394879	0.737355	None
11598	RLF	-0.12495	0.39497	0.737461	None

11599	SNED1	-0.04432	0.395069	0.737506	None
11600	TRIM8	-0.10647	0.395081	0.737506	None
11601	GNB3	-0.06127	0.39511	0.737506	None
11602	RP11-250E	-0.17389	0.39513	0.737506	None
11603	ASAP2	-0.30951	0.395264	0.737644	None
11604	LOC10192ɛ	-0.00915	0.395272	0.737644	None
11605	KPTN	-0.10888	0.395338	0.737703	None
11606	PTPRD	0.192284	0.395508	0.737957	None
11607	KLHL31	-0.0353	0.395568	0.737959	None
11608	LOC10246'	-0.01429	0.395577	0.737959	None
11609	ATP2A1	-0.0138	0.395641	0.738014	None
11610	TRIM46	0.039926	0.395701	0.738042	None
11611	AIMP1	0.10086	0.395749	0.738042	None
11612	DENND2C	-0.09281	0.395758	0.738042	None
11613	CCDC61	0.02575	0.395798	0.738052	None
11614	MGC2889	-0.01197	0.395855	0.738059	None
11615	C19orf45	-0.01922	0.395877	0.738059	None
11616	ODAM	0.00827	0.395919	0.738059	None
11617	PCDHB3	-0.03784	0.395938	0.738059	None
11618	DHH	-0.00485	0.396	0.7381	None
11619	BEX4	0.128321	0.396028	0.7381	None
11620	NNAT	0.070648	0.396124	0.73814	None
11621	COG2	-0.06013	0.396158	0.73814	None
11622	C11orf31	0.068421	0.39618	0.73814	None
11623	HSPB7	-0.01129	0.39621	0.73814	None
11624	PRKAR1A	-0.12459	0.39622	0.73814	None
11625	MCM2	0.168942	0.396283	0.738174	None
11626	COLCA1	-0.06988	0.39631	0.738174	None
11627	KLF2	-0.2235	0.396368	0.738174	None
11628	HCFC1	-0.04253	0.396375	0.738174	None
11629	REXO1	-0.05897	0.396442	0.738213	None
11630	IMPG2	-0.01698	0.396464	0.738213	None
11631	RP3-359N1	0.032087	0.396539	0.738222	None
11632	C12orf54	-0.00677	0.39657	0.738222	None
11633	ACBD5	0.119534	0.396571	0.738222	None
11634	GABRB2	0.030815	0.396743	0.738388	None
11635	KB-1836B5	-0.03328	0.396791	0.738388	None
11636	FAM53B-A	-0.00916	0.396799	0.738388	None
11637	MTRF1L	-0.06793	0.396803	0.738388	None
11638	SLC13A4	-0.00987	0.39683	0.738388	None
11639	C10orf126	-0.02434	0.396937	0.738523	None
11640	CIRH1A	-0.12449	0.396999	0.738575	None
11641	RYR1	-0.03699	0.397087	0.738676	None
11642	RHPN1-AS	-0.06188	0.397127	0.738685	None
11643	LOC10192!	-0.01054	0.397432	0.73919	None
11644	SLC38A3	-0.02367	0.397482	0.739219	None
11645	NNMT	-0.01419	0.397611	0.739397	None
11646	RP11-214K	0.011456	0.397726	0.739446	None
11647	TNFRSF10I	-0.10483	0.397776	0.739446	None
11648	SORT1	-0.17882	0.397807	0.739446	None
11649	C9orf38	0.35643	0.397846	0.739446	None
11650	CTU2	0.064107	0.397857	0.739446	None
11651	HECW1-IT	-0.01308	0.397924	0.739446	None
11652	AGRN	0.038823	0.397953	0.739446	None
11653	RP11-61L1	0.115849	0.398085	0.739446	None
11654	NACAP1	0.02243	0.398094	0.739446	None
11655	RAMP1	0.207548	0.398097	0.739446	None
11656	LYZL6	-0.03526	0.398097	0.739446	None

11657	POLD2	-0.15708	0.398112	0.739446	None
11658	XAGE2	0.060969	0.398139	0.739446	None
11659	GSTP1	-0.19095	0.398168	0.739446	None
11660	CENPB	-0.08019	0.398234	0.739446	None
11661	LOC10192	-0.00826	0.398239	0.739446	None
11662	FLJ13224	-0.02412	0.398242	0.739446	None
11663	FER	-0.1149	0.398259	0.739446	None
11664	IL17A	0.004623	0.398295	0.739446	None
11665	SAMD8	0.132507	0.398321	0.739446	None
11666	ASB1	-0.09556	0.398462	0.739639	None
11667	RNMTL1	0.128718	0.398493	0.739639	None
11668	NAT10	-0.12135	0.398564	0.739675	None
11669	FAM90A1	-0.05566	0.398581	0.739675	None
11670	HIRIP3	-0.10486	0.398676	0.739789	None
11671	TTN-AS1	-0.12364	0.398766	0.739832	None
11672	HIBCH	-0.12399	0.398768	0.739832	None
11673	CEACAM4	-0.08165	0.398834	0.739891	None
11674	LINC01096	0.005006	0.398914	0.739977	None
11675	ZSCAN30	-0.07206	0.399085	0.740231	None
11676	TMEM41B	0.118938	0.399151	0.740289	None
11677	ADCY10	-0.0105	0.399203	0.740321	None
11678	TMEM119	-0.09202	0.39924	0.740327	None
11679	GLIS1	-0.01699	0.399326	0.740394	None
11680	LPXN	0.158563	0.399344	0.740394	None
11681	RP11-664D	0.030337	0.399464	0.740424	None
11682	NEMF	-0.10696	0.399484	0.740424	None
11683	NKX6-1	-0.01965	0.399492	0.740424	None
11684	C11orf96	0.341507	0.399497	0.740424	None
11685	FBLN1	-0.05414	0.399604	0.740558	None
11686	KHNYN	-0.10921	0.399661	0.7406	None
11687	LOC10106	-0.06946	0.399772	0.740653	None
11688	ZBP1	-0.038	0.399777	0.740653	None
11689	AL832163	-0.03412	0.399795	0.740653	None
11690	LINC0054C	0.080984	0.39984	0.740653	None
11691	RP11-429E	0.005719	0.39986	0.740653	None
11692	PITRM1-AS	-0.02273	0.399954	0.740716	None
11693	ACOT9	0.128201	0.399963	0.740716	None
11694	RADIL	0.135586	0.400076	0.740862	None
11695	FCN1	0.487999	0.400142	0.740921	None
11696	ADCK2	0.138314	0.400212	0.740988	None
11697	HOMER1	0.131644	0.400288	0.741003	None
11698	IGFN1	0.018075	0.400289	0.741003	None
11699	HIST1H1A	-0.05712	0.400525	0.741309	None
11700	TRIP12	-0.11837	0.400548	0.741309	None
11701	LINC00445	-0.01682	0.400557	0.741309	None
11702	FBXO17	-0.12061	0.400625	0.741365	None
11703	MYF6	-0.0745	0.400655	0.741365	None
11704	RP11-348F	0.13472	0.400753	0.741483	None
11705	FARS2	0.133363	0.400837	0.741575	None
11706	LINC01088	-0.00397	0.400938	0.741698	None
11707	SOGA1	0.040138	0.401019	0.741769	None
11708	PSMA4	0.07054	0.401045	0.741769	None
11709	CRYGN	-0.00791	0.401114	0.741834	None
11710	KIAA1456	-0.007	0.401218	0.741918	None
11711	CPNE2	-0.15741	0.401244	0.741918	None
11712	SNCAIP	-0.07261	0.401263	0.741918	None
11713	TRO	0.184917	0.401326	0.741934	None
11714	MGC39584	0.021247	0.40134	0.741934	None

11715	RAB23	-0.21991	0.401386	0.741956	None
11716	SPOCD1	-0.05937	0.401425	0.741964	None
11717	HOMEZ	-0.03605	0.401463	0.741972	None
11718	LMF1	-0.0854	0.401564	0.742095	None
11719	LHX1	-0.02178	0.401783	0.742148	None
11720	CTSO	0.196036	0.401795	0.742148	None
11721	ARHGAP5	0.189602	0.401797	0.742148	None
11722	CAMSAP1	0.140542	0.401818	0.742148	None
11723	SCOC	-0.18318	0.401885	0.742148	None
11724	GLYATL2	-0.09862	0.40191	0.742148	None
11725	CNOT6	0.071579	0.401914	0.742148	None
11726	RP11-82L1	0.024254	0.401919	0.742148	None
11727	SGTB	0.088007	0.401927	0.742148	None
11728	C14orf39	-0.05171	0.401961	0.742148	None
11729	KCNK1	-0.10814	0.402018	0.742148	None
11730	SPO11	-0.01572	0.402022	0.742148	None
11731	ANXA9	0.018432	0.40204	0.742148	None
11732	VMP1	0.374509	0.402093	0.742148	None
11733	KIAA1661	-0.00629	0.40214	0.742148	None
11734	TIMM23B	0.144077	0.402152	0.742148	None
11735	DNAH12	-0.0493	0.40222	0.742148	None
11736	SSX2IP	-0.14582	0.402228	0.742148	None
11737	LINC01342	-0.01645	0.402244	0.742148	None
11738	LOC10192!	-0.02722	0.402343	0.742224	None
11739	SLC33A1	-0.11863	0.402366	0.742224	None
11740	CTC-471C:	-0.00306	0.40243	0.742224	None
11741	MOB3B	0.123386	0.40243	0.742224	None
11742	ANTXR2	0.123167	0.402465	0.742224	None
11743	ZNF431	-0.20782	0.402499	0.742224	None
11744	KXD1	-0.02693	0.402554	0.742224	None
11745	LOC10192ł	-0.00909	0.402583	0.742224	None
11746	CREB5	-0.25265	0.402663	0.742224	None
11747	LINC01356	0.078926	0.402688	0.742224	None
11748	CCDC66	-0.08078	0.402749	0.742224	None
11749	ZNF518A	0.171854	0.402756	0.742224	None
11750	EFHB	-0.04966	0.402763	0.742224	None
11751	ADAMTS1(	-0.06471	0.402765	0.742224	None
11752	WSCD2	-0.01245	0.402837	0.742243	None
11753	VNN1	-0.25119	0.402844	0.742243	None
11754	CHCHD5	-0.03208	0.402986	0.74239	None
11755	FRK	-0.03464	0.402992	0.74239	None
11756	PLB1	0.037183	0.403032	0.7424	None
11757	CAP2	-0.00395	0.403093	0.742449	None
11758	LOC10013(	0.013961	0.403218	0.742617	None
11759	IDH3G	-0.08488	0.403343	0.742785	None
11760	LOC73096:	0.030895	0.40339	0.742806	None
11761	ZFP36	-0.2666	0.403483	0.742889	None
11762	GPX4	-0.1754	0.403503	0.742889	None
11763	SNRNP27	-0.08612	0.403552	0.742915	None
11764	LOC10028'	-0.12849	0.403621	0.742979	None
11765	RP11-452L	-0.17526	0.403667	0.742996	None
11766	SLC52A3	-0.05729	0.403729	0.742996	None
11767	IBA57	0.138124	0.403733	0.742996	None
11768	CWF19L2	-0.06746	0.403797	0.743052	None
11769	CCNB2	-0.0614	0.403867	0.743117	None
11770	MAPK6	-0.05957	0.403906	0.743126	None
11771	RP11-480/	0.153403	0.404073	0.743369	None
11772	RABGGTB	0.103847	0.404216	0.74357	None

11773	B9D2	-0.09874	0.404531	0.744085	None
11774	PSMC3	-0.14325	0.404639	0.744221	None
11775	SPRR2B	-0.03979	0.404746	0.744324	None
11776	PSMB7	0.111237	0.404783	0.744324	None
11777	C6orf223	0.031235	0.40483	0.744324	None
11778	SLC22A8	0.029789	0.404832	0.744324	None
11779	LOC10049!	-0.00581	0.404871	0.744331	None
11780	DPYSL3	-0.02385	0.404915	0.744349	None
11781	ADH7	0.027057	0.405046	0.744396	None
11782	LOC10013(	-0.0646	0.4051	0.744396	None
11783	FOXP4	-0.04307	0.405117	0.744396	None
11784	EMX1	-0.01602	0.405135	0.744396	None
11785	RP11-1017	-0.04	0.405136	0.744396	None
11786	USP9Y	-0.48109	0.405154	0.744396	None
11787	CLIP1-AS1	-0.03095	0.405206	0.744396	None
11788	LOC10192(	-0.03698	0.405215	0.744396	None
11789	CDC123	0.095124	0.405436	0.74465	None
11790	B3GALT2	-0.00395	0.405443	0.74465	None
11791	UBA5	-0.08883	0.405541	0.74465	None
11792	HSPB9	0.033688	0.405555	0.74465	None
11793	FAM115A	-0.00696	0.405589	0.74465	None
11794	ADH6	0.013089	0.405625	0.74465	None
11795	KLHL7	0.200302	0.405666	0.74465	None
11796	CDIPT-AS1	0.022262	0.405685	0.74465	None
11797	CLIC5	0.011058	0.40572	0.74465	None
11798	PNMT	-0.21929	0.405744	0.74465	None
11799	PITPNC1	0.07234	0.405756	0.74465	None
11800	MFI2	-0.06239	0.405766	0.74465	None
11801	RP11-65L1	-0.03052	0.40593	0.744886	None
11802	CTNNBL1	0.159501	0.406003	0.744958	None
11803	PARPBP	0.147852	0.406077	0.745031	None
11804	NCOA6	-0.06071	0.406271	0.745323	None
11805	CDK5R2	0.026983	0.406357	0.745418	None
11806	NID1	-0.07054	0.40648	0.74545	None
11807	THG1L	0.200816	0.406484	0.74545	None
11808	RP11-1007	-0.01108	0.406513	0.74545	None
11809	METTL6	-0.04253	0.406532	0.74545	None
11810	PIP5K1A	-0.03432	0.406546	0.74545	None
11811	RP11-539(	0.001938	0.406636	0.745551	None
11812	EGR1	-0.2677	0.406691	0.745589	None
11813	NUAK1	-0.19585	0.406922	0.745889	None
11814	CDYL2	0.081986	0.406924	0.745889	None
11815	LOC10050(	0.036352	0.407121	0.745971	None
11816	CELF3	-0.03397	0.407134	0.745971	None
11817	HUNK	0.03152	0.407175	0.745971	None
11818	AP006547.	0.033167	0.407227	0.745971	None
11819	CEPT1	0.160008	0.40723	0.745971	None
11820	LINC00663	-0.03425	0.407235	0.745971	None
11821	LOC72807:	0.066869	0.407242	0.745971	None
11822	RPAP3	0.107163	0.407244	0.745971	None
11823	PCDHB5	-0.23136	0.407322	0.746051	None
11824	TBC1D22A	-0.10067	0.407371	0.746077	None
11825	LOC10192	0.011708	0.407647	0.746446	None
11826	LOC10106(	-0.01853	0.407676	0.746446	None
11827	LINC00605	0.009138	0.407688	0.746446	None
11828	TCF15	-0.15252	0.407714	0.746446	None
11829	HSPA12A	-0.00776	0.407745	0.746446	None
11830	NAP1L2	-0.3073	0.407908	0.746651	None

11831	ZNF880	0.144564	0.407926	0.746651	None
11832	TXNDC15	-0.11206	0.408049	0.746815	None
11833	FTH1P5	0.111247	0.4081	0.746843	None
11834	PALLD	-0.18812	0.408172	0.746912	None
11835	HSD17B3	0.030232	0.408279	0.746992	None
11836	KIRREL2	0.019519	0.408284	0.746992	None
11837	TCTE3	0.001755	0.408345	0.74704	None
11838	RP11-533E	-0.0458	0.408515	0.747196	None
11839	PROCR	-0.14705	0.408531	0.747196	None
11840	SCARNA17	0.266765	0.408534	0.747196	None
11841	LOC72965	-0.00993	0.408595	0.747244	None
11842	LOC10050	-0.03632	0.408649	0.74728	None
11843	LOC28541	-0.06228	0.408728	0.747311	None
11844	KLHL3	-0.11079	0.408739	0.747311	None
11845	AX748292	-0.01272	0.408769	0.747311	None
11846	TRIB2	-0.35939	0.40883	0.747359	None
11847	ARNTL2-A	-0.1636	0.408918	0.747457	None
11848	LINC00624	-0.00509	0.408989	0.747522	None
11849	ANKFN1	0.009033	0.409181	0.747811	None
11850	SYT12	-0.03817	0.409369	0.748074	None
11851	LOC10013	0.083992	0.409431	0.748074	None
11852	LOC10192	0.028417	0.409448	0.748074	None
11853	LOC10192	-0.07571	0.40951	0.748074	None
11854	MECOM	-0.12271	0.409523	0.748074	None
11855	OVGP1	-0.12225	0.409532	0.748074	None
11856	PCDHA10	-0.03011	0.409576	0.748092	None
11857	XAGE3	-0.01824	0.409621	0.748111	None
11858	PNPLA8	0.143788	0.409702	0.74818	None
11859	ARL4A	-0.14081	0.409729	0.74818	None
11860	CD8A	0.168812	0.409855	0.748328	None
11861	DNAJA1	-0.07273	0.409921	0.748328	None
11862	GLIS2	-0.02894	0.409947	0.748328	None
11863	LOC10193	-0.13181	0.409948	0.748328	None
11864	PCED1B-A	-0.02269	0.410084	0.74849	None
11865	ABHD1	-0.01979	0.410109	0.74849	None
11866	LOC10013	-0.11998	0.41014	0.74849	None
11867	LRRC29	-0.01941	0.410244	0.748566	None
11868	LOC72965	-0.00809	0.410251	0.748566	None
11869	RP11-102M	-0.00579	0.410327	0.748642	None
11870	TMCC3	0.180155	0.410479	0.748856	None
11871	CCDC28A	-0.07825	0.410536	0.748882	None
11872	OGG1	-0.13196	0.410562	0.748882	None
11873	ANKH	-0.07065	0.410623	0.74893	None
11874	RP11-515C	-0.0186	0.410806	0.7492	None
11875	ASIC1	0.012519	0.410863	0.74924	None
11876	CROCCP2	-0.08816	0.411101	0.749613	None
11877	FOXN4	-0.00733	0.411229	0.749783	None
11878	ADAMTS5	-0.10875	0.411298	0.749789	None
11879	LOC10012	0.040778	0.411302	0.749789	None
11880	FAM89B	-0.10762	0.411435	0.749825	None
11881	TMEM2	-0.23291	0.411473	0.749825	None
11882	LAMA2	-0.10853	0.411481	0.749825	None
11883	DLG4	-0.0542	0.411482	0.749825	None
11884	MON2	-0.11789	0.411495	0.749825	None
11885	LRP1B	-0.0518	0.41157	0.749899	None
11886	SPATA22	-0.21892	0.411639	0.749961	None
11887	LOC10192	0.018044	0.411776	0.750147	None
11888	ASIC2	-0.01598	0.411896	0.75028	None

11889	ZNF764	-0.09884	0.411958	0.75028	None
11890	RP11-177N	-0.01949	0.412004	0.75028	None
11891	GINS4	-0.0706	0.41202	0.75028	None
11892	SHROOM1	0.054601	0.412064	0.75028	None
11893	IGSF10	-0.32061	0.412066	0.75028	None
11894	SLC6A8	-0.16285	0.412125	0.75028	None
11895	SLC44A3	-0.10624	0.412167	0.75028	None
11896	RP11-798M	-0.01554	0.412167	0.75028	None
11897	RP11-320N	-0.00922	0.412195	0.75028	None
11898	TRPV3	0.018343	0.412276	0.75032	None
11899	BSND	0.008751	0.41229	0.75032	None
11900	NCDN	-0.01896	0.412392	0.75032	None
11901	CWF19L1	0.113156	0.412393	0.75032	None
11902	TRPS1	-0.18142	0.412394	0.75032	None
11903	CCDC120	-0.01794	0.412425	0.75032	None
11904	ACSS3	-0.12529	0.412525	0.750439	None
11905	OIP5	-0.20414	0.412617	0.750501	None
11906	TPX2	0.216922	0.412628	0.750501	None
11907	GOSR1	-0.09926	0.412686	0.750544	None
11908	ZNRD1-AS	-0.13841	0.412771	0.750635	None
11909	LACC1	-0.14131	0.412887	0.750771	None
11910	ARSB	-0.0675	0.412915	0.750771	None
11911	FAM223B	0.136343	0.413033	0.750921	None
11912	SULT2B1	0.031029	0.413112	0.750941	None
11913	TPGS2	0.114875	0.413124	0.750941	None
11914	ATP6AP2	-0.1583	0.413147	0.750941	None
11915	FLJ44087	-0.01293	0.413253	0.751069	None
11916	NKG7	-0.28381	0.413403	0.751107	None
11917	RERG	0.018976	0.413407	0.751107	None
11918	LINC00355	-0.06917	0.413464	0.751107	None
11919	RPS24	-0.12947	0.413485	0.751107	None
11920	TRIQK	-0.142	0.413504	0.751107	None
11921	SP6	-0.01548	0.41355	0.751107	None
11922	EIF3D	0.085687	0.413553	0.751107	None
11923	LOC10013(	0.004552	0.413572	0.751107	None
11924	EIF3A	-0.10864	0.413669	0.751107	None
11925	LOC10192	-0.01924	0.413681	0.751107	None
11926	ATP13A4	-0.00952	0.413707	0.751107	None
11927	UHRF1BP1	0.025055	0.413713	0.751107	None
11928	MYOZ1	-0.01179	0.413725	0.751107	None
11929	RPL11	-0.13003	0.413811	0.751202	None
11930	ZDBF2	0.127295	0.413894	0.751229	None
11931	OR1A1	-0.01461	0.413896	0.751229	None
11932	HEXIM2	-0.13625	0.413936	0.751239	None
11933	SPAST	-0.10276	0.414051	0.751386	None
11934	ZHX1-C8o	-0.14867	0.414191	0.751576	None
11935	FAM117B	0.115605	0.414285	0.751684	None
11936	ALDOAP2	-0.02611	0.414428	0.751817	None
11937	SCGB1D2	-0.00251	0.414428	0.751817	None
11938	LINC01270	-0.00506	0.414509	0.75184	None
11939	IL1RAPL1	-0.00682	0.41451	0.75184	None
11940	NR2F6	-0.0143	0.414707	0.752134	None
11941	SLC17A9	0.083753	0.414772	0.752189	None
11942	ELOVL2-AS	-0.00407	0.414864	0.752262	None
11943	SSSCA1-AS	-0.01283	0.414882	0.752262	None
11944	LL0XNC01-	0.020844	0.414949	0.752321	None
11945	T	0.054802	0.415085	0.752504	None
11946	LOC10272	0.161135	0.41517	0.752595	None

11947	KCNRG	0.082165	0.415225	0.752613	None
11948	TRPM5	-0.02219	0.415279	0.752613	None
11949	DNAJC5G	0.005526	0.415354	0.752613	None
11950	LINC01126	-0.16243	0.415402	0.752613	None
11951	C12orf10	-0.11368	0.415408	0.752613	None
11952	OR7E156P	-0.04023	0.415412	0.752613	None
11953	ELL	-0.07449	0.415432	0.752613	None
11954	LOC10192!	0.042776	0.415551	0.752613	None
11955	TCF21	-0.00338	0.415592	0.752613	None
11956	RP4-621B1	-0.01942	0.415623	0.752613	None
11957	NEK9	-0.01517	0.415639	0.752613	None
11958	FARP2	-0.03705	0.41565	0.752613	None
11959	DMWD	-0.02824	0.415657	0.752613	None
11960	CXCL6	0.159179	0.415696	0.752613	None
11961	TBX19	-0.0908	0.415701	0.752613	None
11962	LOC10192!	0.031111	0.415739	0.752619	None
11963	AMHR2	-0.06474	0.415845	0.752747	None
11964	NDST3	-0.08259	0.415944	0.752818	None
11965	GPR174	-0.05555	0.415964	0.752818	None
11966	IGHV3-54	-0.01783	0.415988	0.752818	None
11967	TUBB4A	0.03476	0.416193	0.753074	None
11968	WDR70	0.12188	0.416224	0.753074	None
11969	PPP5D1	-0.0946	0.416234	0.753074	None
11970	RTN4IP1	-0.16311	0.416286	0.753106	None
11971	PRR7	-0.06534	0.41633	0.753123	None
11972	ARHGEF7-	0.003588	0.416395	0.753149	None
11973	LOC10192{	-0.00577	0.416477	0.753149	None
11974	TRPM2	-0.09797	0.41649	0.753149	None
11975	RP11-324J	-0.01335	0.416524	0.753149	None
11976	FAM219A	-0.06512	0.416532	0.753149	None
11977	PPP4R1L	-0.0031	0.416554	0.753149	None
11978	UBXN2B	-0.0788	0.41663	0.753189	None
11979	CAV1	0.27197	0.416669	0.753189	None
11980	SRPK2	-0.12687	0.41668	0.753189	None
11981	AQP3	0.053356	0.416719	0.753197	None
11982	MBLAC2	-0.10974	0.41679	0.753263	None
11983	PMP22	0.062467	0.416826	0.753264	None
11984	CPA6	-0.01183	0.41688	0.753295	None
11985	C21orf49	-0.01738	0.416912	0.753295	None
11986	LOC10013:	0.121339	0.416993	0.753351	None
11987	KLF1	-0.40561	0.417043	0.753351	None
11988	ST18	-0.0124	0.417048	0.753351	None
11989	CPSF3L	-0.05698	0.417138	0.753395	None
11990	DNAH2	-0.05806	0.417171	0.753395	None
11991	DZIP1L	-0.10031	0.417177	0.753395	None
11992	RSPRY1	-0.09753	0.417241	0.753449	None
11993	CELSR2	-0.0177	0.417306	0.753503	None
11994	SERPINA1(	-0.03566	0.417399	0.753514	None
11995	CT55	0.009403	0.417468	0.753514	None
11996	TCEAL3	-0.13189	0.41749	0.753514	None
11997	PPIB	0.15129	0.417553	0.753514	None
11998	DDB1	-0.079	0.417599	0.753514	None
11999	ITPA	-0.1157	0.417603	0.753514	None
12000	LOC10192!	0.002929	0.417611	0.753514	None
12001	ANKRD30E	0.012372	0.41763	0.753514	None
12002	ZBTB7C	-0.04568	0.41766	0.753514	None
12003	LOC10192{	-0.01949	0.41766	0.753514	None
12004	RP5-1061H	-0.02136	0.417757	0.753526	None

12005	BBS7	-0.1485	0.417779	0.753526	None
12006	BEND4	-0.12242	0.41779	0.753526	None
12007	RPRML	-0.06848	0.417806	0.753526	None
12008	BTD	0.093894	0.41787	0.753579	None
12009	TAS1R1	0.005281	0.417983	0.753719	None
12010	PIK3IP1	0.101311	0.418035	0.753751	None
12011	CBX1	-0.11104	0.418079	0.753768	None
12012	AK055458	-0.03252	0.418206	0.75391	None
12013	NDUFA11	0.19889	0.418253	0.75391	None
12014	PARP8	0.375558	0.418291	0.75391	None
12015	GPR21	-0.09932	0.418297	0.75391	None
12016	EXOC3L1	-0.009	0.418343	0.753929	None
12017	SYCP3	0.026717	0.418498	0.754146	None
12018	LOC10272:	-0.0669	0.418637	0.754334	None
12019	SMYD1	-0.02764	0.4188	0.754548	None
12020	LOC10192{	-0.00409	0.418825	0.754548	None
12021	MMP24	-0.00812	0.419023	0.754693	None
12022	PITPNM2	0.010006	0.419034	0.754693	None
12023	JAKMIP2	-0.14484	0.41904	0.754693	None
12024	SYNJ1	-0.01607	0.419049	0.754693	None
12025	CTC-471J1	-0.11114	0.41908	0.754693	None
12026	HEXIM1	-0.13062	0.419154	0.754747	None
12027	EPB41L4A	-0.02168	0.419202	0.754747	None
12028	CLDN6	-0.01072	0.419215	0.754747	None
12029	AHR	-0.20772	0.419255	0.754756	None
12030	RRP1	0.069524	0.419359	0.754816	None
12031	CSF3	0.040048	0.419363	0.754816	None
12032	LOC10192]	-0.01215	0.419393	0.754816	None
12033	PGM1	-0.10627	0.419445	0.754847	None
12034	NALCN-AS	-0.0141	0.419528	0.754873	None
12035	FGF2	-0.05283	0.419529	0.754873	None
12036	HAMP	0.087813	0.419587	0.754883	None
12037	TNNT3	-0.05844	0.419604	0.754883	None
12038	TMEM246	-0.30109	0.4197	0.754964	None
12039	CD81	0.154668	0.419719	0.754964	None
12040	FUT6	-0.01814	0.419779	0.75501	None
12041	LOC10192]	0.017157	0.420052	0.755402	None
12042	SH2D3C	0.138519	0.420067	0.755402	None
12043	NDUFA2	-0.01699	0.420105	0.755408	None
12044	C14orf80	0.11083	0.420212	0.755475	None
12045	ARHGAP1!	0.138057	0.420212	0.755475	None
12046	LOC10050(	0.03076	0.420287	0.755547	None
12047	MKS1	-0.06286	0.420356	0.755607	None
12048	QRICH2	-0.05242	0.420394	0.755614	None
12049	BLVRA	-0.22867	0.420488	0.755719	None
12050	TNNC2	-0.05973	0.420593	0.755795	None
12051	C11orf63	-0.05143	0.420634	0.755795	None
12052	HIST1H2B(	-0.09568	0.420635	0.755795	None
12053	VDR	-0.08389	0.420811	0.75605	None
12054	FZR1	-0.04476	0.420909	0.756162	None
12055	SOCS4	0.076628	0.421053	0.756358	None
12056	RP11-359E	0.004904	0.421143	0.756458	None
12057	EXT1	0.106678	0.421291	0.756587	None
12058	RP11-472k	-0.01006	0.421303	0.756587	None
12059	PTPRCAP	-0.20337	0.42132	0.756587	None
12060	RP11-5C23	-0.08424	0.4214	0.756668	None
12061	TG	-0.01539	0.421454	0.756702	None
12062	TACSTD2	-0.00398	0.421539	0.756712	None

12063	ERICH5	-0.03156	0.421553	0.756712	None
12064	TTC22	-0.0027	0.421564	0.756712	None
12065	COPG2	0.063011	0.421779	0.756974	None
12066	NSUN2	0.085676	0.42182	0.756974	None
12067	PRDX2	0.079264	0.421832	0.756974	None
12068	SUN5	-0.01398	0.42185	0.756974	None
12069	HAGLROS	-0.00871	0.42195	0.757091	None
12070	ARL8B	-0.07871	0.422106	0.757152	None
12071	CWC25	-0.11165	0.422131	0.757152	None
12072	RP11-552N	-0.04536	0.42215	0.757152	None
12073	LOC10028	-0.19009	0.42215	0.757152	None
12074	IRGM	-0.06692	0.422159	0.757152	None
12075	GSTO2	-0.1531	0.4222	0.757163	None
12076	NDUFA6	-0.11067	0.422291	0.757229	None
12077	LCP1	-0.13823	0.422309	0.757229	None
12078	FLJ41455	0.105027	0.422408	0.757229	None
12079	ZNF16	-0.11924	0.422434	0.757229	None
12080	YBX1	0.141061	0.4225	0.757229	None
12081	FMOD	-0.05771	0.422511	0.757229	None
12082	FAM69A	-0.12133	0.422535	0.757229	None
12083	CGN	-0.01423	0.422541	0.757229	None
12084	ACAT1	-0.02954	0.422551	0.757229	None
12085	FAM47B	-0.0134	0.422607	0.757266	None
12086	ADAM15	-0.04908	0.422714	0.757292	None
12087	SMIM12	0.062505	0.422739	0.757292	None
12088	GKAP1	-0.15651	0.422776	0.757292	None
12089	PTGS2	0.379111	0.422848	0.757292	None
12090	LGALS3BP	0.351131	0.422862	0.757292	None
12091	SLPI	-0.16618	0.422869	0.757292	None
12092	SHFM1	0.081176	0.422883	0.757292	None
12093	FGR	-0.30039	0.422951	0.757292	None
12094	SLC26A2	0.160105	0.422999	0.757292	None
12095	LINC00857	-0.00392	0.423003	0.757292	None
12096	SLC26A3	-0.00326	0.423007	0.757292	None
12097	SEMA6C	-0.04592	0.423253	0.757563	None
12098	DTD2	-0.11243	0.42326	0.757563	None
12099	LOC10050	-0.14267	0.423326	0.757563	None
12100	DMGDH	-0.00884	0.423344	0.757563	None
12101	LRP11	-0.2648	0.423372	0.757563	None
12102	GFRA4	-0.0072	0.423377	0.757563	None
12103	DKK1	-0.11113	0.423403	0.757563	None
12104	CHD7	-0.08341	0.423469	0.757583	None
12105	APOC1	-0.31786	0.423493	0.757583	None
12106	SLC5A11	-0.04384	0.423519	0.757583	None
12107	PVRL1	-0.02636	0.423597	0.75766	None
12108	DHRS7	-0.12827	0.423737	0.757847	None
12109	TFIP11	0.091489	0.423771	0.757847	None
12110	RP11-1114	-0.12906	0.423819	0.75787	None
12111	UBXN7	-0.12432	0.423894	0.757942	None
12112	CSTF3	0.109624	0.424067	0.758113	None
12113	NANOS3	-0.03395	0.424071	0.758113	None
12114	NEFL	-0.01325	0.424112	0.758113	None
12115	LSM8	-0.11336	0.424158	0.758113	None
12116	MIR4296	-0.05163	0.424203	0.758113	None
12117	RBFADN	0.02929	0.424228	0.758113	None
12118	OTUB2	-0.00873	0.424235	0.758113	None
12119	THUMPD2	-0.09604	0.424295	0.758125	None
12120	A2M	-0.24328	0.424332	0.758125	None

12121	EHMT2	-0.0812	0.424347	0.758125	None
12122	TAF3	0.080159	0.424486	0.758267	None
12123	ST6GAL1	-0.02412	0.424592	0.758267	None
12124	PGD	-0.00509	0.424625	0.758267	None
12125	C8orf74	-0.01129	0.424635	0.758267	None
12126	RP11-6I2.3	-0.06831	0.424697	0.758267	None
12127	WNK3	-0.02388	0.424752	0.758267	None
12128	CMA1	-0.00246	0.424758	0.758267	None
12129	SPAG17	-0.14498	0.42478	0.758267	None
12130	FLJ11710	-0.1224	0.424838	0.758267	None
12131	LOC10106(	-0.01786	0.424847	0.758267	None
12132	CEBPG	-0.09837	0.42492	0.758267	None
12133	KLRD1	0.023834	0.424934	0.758267	None
12134	MPDZ	0.291237	0.425015	0.758267	None
12135	AGPAT4-IT	-0.18236	0.425058	0.758267	None
12136	HCRT	-0.00431	0.425087	0.758267	None
12137	MMP26	-0.0121	0.425146	0.758267	None
12138	COL4A1	-0.15593	0.425196	0.758267	None
12139	RTP3	-0.01083	0.425197	0.758267	None
12140	PRDM2	0.130146	0.425221	0.758267	None
12141	KLHL6	-0.11956	0.425225	0.758267	None
12142	UQCRC1	-0.14698	0.425231	0.758267	None
12143	HOXA-AS2	-0.00432	0.425245	0.758267	None
12144	SPATA3	-0.01526	0.425258	0.758267	None
12145	LOC10272	-0.00927	0.425267	0.758267	None
12146	RP1-202O8	-0.01593	0.425323	0.758305	None
12147	FAM170B	-0.00191	0.425367	0.758321	None
12148	POLR2D	0.097643	0.425475	0.758361	None
12149	LOC101928	0.014498	0.425496	0.758361	None
12150	MOCS1	-0.05203	0.425511	0.758361	None
12151	SUPV3L1	-0.10826	0.42553	0.758361	None
12152	TAS2R45	-0.01189	0.425629	0.758476	None
12153	CDRT15L2	-0.01811	0.425748	0.758517	None
12154	LOC10192	-0.01072	0.425763	0.758517	None
12155	LINC00167	-0.01135	0.425804	0.758517	None
12156	FFAR4	-0.01139	0.425826	0.758517	None
12157	REG1P	-0.00619	0.42583	0.758517	None
12158	ACOT8	-0.10408	0.425908	0.758517	None
12159	BSPRY	0.132745	0.425919	0.758517	None
12160	SUGP2	-0.10883	0.425933	0.758517	None
12161	MED14OS	-0.03983	0.426106	0.758542	None
12162	THUMPD3	-0.05855	0.426112	0.758542	None
12163	OSTM1-AS	-0.00467	0.426169	0.758542	None
12164	ZBTB39	0.061342	0.42617	0.758542	None
12165	BC028670	-0.01826	0.426197	0.758542	None
12166	CAND1	0.108213	0.426335	0.758542	None
12167	GPR84	0.285067	0.426337	0.758542	None
12168	RP11-121C	-0.1309	0.42635	0.758542	None
12169	ZNF808	0.106795	0.42636	0.758542	None
12170	SALL2	-0.17685	0.426365	0.758542	None
12171	NCL	0.036296	0.426366	0.758542	None
12172	ZNF99	-0.09569	0.426367	0.758542	None
12173	S100A4	-0.24167	0.426437	0.758592	None
12174	USPL1	0.100151	0.426465	0.758592	None
12175	LOC10050	0.024294	0.42653	0.758646	None
12176	LECT1	-0.05831	0.426642	0.758783	None
12177	AP006216.	-0.02778	0.426686	0.758798	None
12178	POLR2J2	-0.07755	0.426759	0.758866	None

12179	TMEM251	-0.11698	0.426962	0.759109	None
12180	CRIP3	-0.03586	0.426966	0.759109	None
12181	LOX	-0.13574	0.427021	0.759121	None
12182	ZNF414	0.01384	0.427043	0.759121	None
12183	IL21R-AS1	-0.00998	0.427156	0.759259	None
12184	PPP4R1	0.07742	0.427341	0.75951	None
12185	CRYGB	-0.01875	0.427367	0.75951	None
12186	PSG9	-0.00591	0.427531	0.759717	None
12187	FLJ38668	-0.02001	0.427601	0.759717	None
12188	POU2F3	-0.00862	0.427616	0.759717	None
12189	C14orf132	-0.04904	0.427624	0.759717	None
12190	KRT72	-0.00641	0.427675	0.759746	None
12191	FAM99B	-0.0554	0.427748	0.759786	None
12192	TRAP1	-0.08284	0.427776	0.759786	None
12193	TNFRSF8	-0.0291	0.427803	0.759786	None
12194	LOC10272	0.149404	0.42785	0.759808	None
12195	RFC2	0.117231	0.427899	0.759833	None
12196	OVCH1-AS	-0.02878	0.428058	0.760051	None
12197	CCDC13	0.005394	0.428282	0.760387	None
12198	HLA-DPB2	-0.06244	0.428323	0.760397	None
12199	CBX4	-0.10784	0.428367	0.760414	None
12200	PTAR1	-0.1442	0.428463	0.760522	None
12201	SEC61G	-0.13118	0.428571	0.760642	None
12202	RP11-847H	-0.01628	0.428601	0.760642	None
12203	PLLP	-0.01117	0.428843	0.761009	None
12204	BPIFC	-0.01327	0.428892	0.761034	None
12205	MANBAL	-0.12916	0.429024	0.761181	None
12206	SNX4	0.07568	0.429046	0.761181	None
12207	IL2RA	-0.12483	0.429143	0.761291	None
12208	TNFRSF10	0.054876	0.429219	0.761364	None
12209	NEDD8	-0.14068	0.429297	0.761406	None
12210	ZYG11B	-0.09832	0.429313	0.761406	None
12211	LOC10050	0.036918	0.429487	0.761538	None
12212	VPS26B	-0.10181	0.429487	0.761538	None
12213	ARPC5L	0.116337	0.429526	0.761538	None
12214	LOC10028	0.047893	0.429534	0.761538	None
12215	SLC52A1	-0.04946	0.429578	0.761538	None
12216	LOC10272	0.026136	0.429614	0.761538	None
12217	TRHDE	-0.13904	0.429633	0.761538	None
12218	MIR5188	-0.02996	0.429698	0.761591	None
12219	LBP	-0.11451	0.429775	0.761664	None
12220	RIC3	-0.00441	0.429882	0.761787	None
12221	RP11-341N	-0.01613	0.429939	0.761787	None
12222	SGOL2	-0.2002	0.42995	0.761787	None
12223	GRAP2	-0.13021	0.430027	0.761862	None
12224	FNDC8	-0.02241	0.430068	0.761871	None
12225	FMO1	-0.03299	0.430244	0.762027	None
12226	DSCR8	-0.01735	0.43032	0.762027	None
12227	NHSL1	-0.02647	0.43035	0.762027	None
12228	TPD52L2	-0.10728	0.43046	0.762027	None
12229	TRPC1	-0.26443	0.430461	0.762027	None
12230	PRPF39	0.18407	0.430469	0.762027	None
12231	HYMAI	0.173278	0.430481	0.762027	None
12232	TLR8	-0.20512	0.430516	0.762027	None
12233	HK3	-0.2643	0.430531	0.762027	None
12234	RP3-329A5	0.062047	0.430542	0.762027	None
12235	LAMA1	-0.0081	0.430543	0.762027	None
12236	GAS1	-0.1159	0.430751	0.762333	None

12237	C8orf34	-0.00933	0.430912	0.762553	None
12238	REXO2	-0.10868	0.431	0.762553	None
12239	GABRG3	0.00407	0.431087	0.762553	None
12240	CTR9	-0.12495	0.431108	0.762553	None
12241	NMRK1	-0.10632	0.431113	0.762553	None
12242	RGS7BP	-0.00513	0.431116	0.762553	None
12243	GINS2	0.031764	0.431121	0.762553	None
12244	RBM15	0.126994	0.431306	0.762746	None
12245	MPZL2	0.170909	0.431354	0.762746	None
12246	LOC40146	-0.00977	0.431385	0.762746	None
12247	LOC10272	-0.08061	0.43144	0.762746	None
12248	RP11-298D	0.006398	0.431441	0.762746	None
12249	IRF6	-0.01928	0.431442	0.762746	None
12250	SNORA72	-0.17191	0.431486	0.762761	None
12251	RP11-25I1	0.027731	0.431556	0.762823	None
12252	RP11-2E11	-0.18384	0.431597	0.762834	None
12253	RPL36A	-0.16073	0.431638	0.762843	None
12254	UBE2E2	-0.20622	0.431745	0.76297	None
12255	NRP2	-0.00932	0.431819	0.763039	None
12256	CTNNAP1	-0.00968	0.431874	0.763074	None
12257	B3GNT3	-0.02901	0.431989	0.763135	None
12258	SRMS	-0.00244	0.432049	0.763135	None
12259	SYNPO2L	-0.00746	0.432092	0.763135	None
12260	LOC72929	-0.01867	0.432151	0.763135	None
12261	LOC10192	-0.00523	0.432162	0.763135	None
12262	SETD5	-0.11637	0.432186	0.763135	None
12263	ZC3H12B	0.048871	0.432284	0.763135	None
12264	CCDC85B	0.025831	0.432417	0.763135	None
12265	NRSN1	0.026339	0.432423	0.763135	None
12266	NUTM2B	-0.01784	0.432426	0.763135	None
12267	LOC44060	0.030175	0.432453	0.763135	None
12268	SLC5A2	-0.01402	0.432459	0.763135	None
12269	ZDHHC1	0.024603	0.432474	0.763135	None
12270	TNNT2	-0.02076	0.432475	0.763135	None
12271	CCT4	-0.05188	0.432491	0.763135	None
12272	CITED1	-0.02192	0.432498	0.763135	None
12273	AGAP4	-0.16547	0.432555	0.763135	None
12274	EFNA1	0.170478	0.432587	0.763135	None
12275	LAMC2	-0.00735	0.432591	0.763135	None
12276	LOC81691	0.129042	0.432614	0.763135	None
12277	HSPA6	-0.25279	0.432745	0.763305	None
12278	MIR210HG	-0.00563	0.432841	0.763387	None
12279	OTX2	-0.00458	0.432893	0.763387	None
12280	GART	-0.11837	0.432897	0.763387	None
12281	KCNA4	0.016303	0.433047	0.763589	None
12282	SUSD3	0.079519	0.433203	0.763801	None
12283	MGP	-0.06932	0.433265	0.763849	None
12284	KLHDC8A	-0.03689	0.433357	0.763892	None
12285	PTPRG	-0.00306	0.433378	0.763892	None
12286	SPIN1	0.104862	0.433395	0.763892	None
12287	ZNF503	0.02244	0.433469	0.76396	None
12288	TNF	0.260658	0.433624	0.764171	None
12289	SPATA25	-0.02471	0.433675	0.764199	None
12290	VCAM1	-0.59361	0.433763	0.764291	None
12291	ZNF548	0.144777	0.433866	0.764411	None
12292	PDCD4-AS	-0.14408	0.433962	0.764462	None
12293	PCDHGA9	-0.01516	0.433966	0.764462	None
12294	ZBBX	-0.01182	0.43401	0.764478	None

12295	LOC10065:	0.014985	0.434263	0.764819	None
12296	REPIN1	-0.05523	0.434274	0.764819	None
12297	TMEM204	-0.02646	0.434314	0.764826	None
12298	KANSL3	-0.03775	0.434371	0.764864	None
12299	TAS2R9	-0.01331	0.434439	0.764922	None
12300	SYNPO2	0.007464	0.434574	0.765042	None
12301	RBFOX3	-0.02392	0.434578	0.765042	None
12302	HORMAD1	-0.02958	0.434683	0.765142	None
12303	LOC44108:	-0.10628	0.434727	0.765142	None
12304	COL5A2	-0.12896	0.43474	0.765142	None
12305	PSPN	0.028223	0.434824	0.765142	None
12306	SNORA74/	-0.02348	0.434834	0.765142	None
12307	PLEKHA4	-0.01625	0.434847	0.765142	None
12308	PARP15	-0.0448	0.435085	0.765335	None
12309	RAB11A	0.131363	0.435143	0.765335	None
12310	POU3F4	-0.0057	0.435193	0.765335	None
12311	RP11-355F	-0.00676	0.435199	0.765335	None
12312	BC010186	0.104224	0.435217	0.765335	None
12313	CACYBP	-0.1031	0.435264	0.765335	None
12314	AC079767.	-0.06276	0.435295	0.765335	None
12315	SLAMF9	-0.01504	0.435302	0.765335	None
12316	KDM1A	0.094469	0.435305	0.765335	None
12317	LOC10050(	-0.01436	0.43531	0.765335	None
12318	C3orf56	-0.02424	0.435413	0.765455	None
12319	RP11-52A:	-0.00347	0.435721	0.765819	None
12320	TJP1	-0.14109	0.435775	0.765819	None
12321	SCD5	0.056514	0.435782	0.765819	None
12322	TM2D1	0.13751	0.435801	0.765819	None
12323	SSTR3	-0.00751	0.435865	0.765819	None
12324	RP11-320H	-0.13689	0.435868	0.765819	None
12325	EFCAB11	-0.07696	0.435875	0.765819	None
12326	C1orf94	-0.01472	0.435903	0.765819	None
12327	UBXN10-A	-0.06997	0.435941	0.765823	None
12328	C9orf131	0.028155	0.435977	0.765824	None
12329	CSNK2B	-0.11081	0.436148	0.765982	None
12330	RFPL1	0.038542	0.43616	0.765982	None
12331	PNLIPRP2	-0.00313	0.436173	0.765982	None
12332	GPR171	0.386784	0.436242	0.765993	None
12333	LAMA5-AS	-0.01806	0.43625	0.765993	None
12334	CHN1	-0.10044	0.43631	0.766036	None
12335	SERPINI1	0.262309	0.436409	0.766057	None
12336	SEMA6D	-0.00407	0.436458	0.766057	None
12337	PLSCR4	-0.22263	0.436463	0.766057	None
12338	SUFU	-0.01402	0.436618	0.766057	None
12339	PMEL	0.016777	0.436635	0.766057	None
12340	PCDHGA1(	-0.01065	0.436737	0.766057	None
12341	ZFP30	0.108756	0.436737	0.766057	None
12342	ATG12	0.115949	0.436744	0.766057	None
12343	LATS1	0.038603	0.436826	0.766057	None
12344	GNG3	-0.0222	0.436837	0.766057	None
12345	CHADL	-0.04504	0.436838	0.766057	None
12346	CYP2S1	-0.00922	0.436908	0.766057	None
12347	UNC5B-AS	-0.01835	0.436947	0.766057	None
12348	PTOV1-AS	-0.01171	0.436975	0.766057	None
12349	ADAM2	-0.00521	0.436987	0.766057	None
12350	LOC10013(	-0.01508	0.437	0.766057	None
12351	LINC00948	0.004487	0.437008	0.766057	None
12352	PALM3	-0.00708	0.437056	0.766057	None

12353	BEST1	-0.03027	0.43707	0.766057	None
12354	LOC10050	-0.02329	0.437082	0.766057	None
12355	GLS	-0.10551	0.437096	0.766057	None
12356	FGF14-IT1	-0.00512	0.437102	0.766057	None
12357	LINC01012	-0.00815	0.437168	0.766057	None
12358	MAGI1	-0.00539	0.437171	0.766057	None
12359	PDGFRL	-0.02965	0.437306	0.766218	None
12360	PRDM13	-0.00625	0.437369	0.766218	None
12361	ABCA11P	-0.13211	0.437388	0.766218	None
12362	LINC00487	-0.05845	0.437404	0.766218	None
12363	MAP3K14	-0.06752	0.437582	0.766468	None
12364	CEP162	-0.10459	0.437625	0.76647	None
12365	CTD-2534I	0.01536	0.437654	0.76647	None
12366	ZNF836	-0.06755	0.437713	0.76651	None
12367	TCFL5	-0.12234	0.437768	0.766517	None
12368	CENPL	0.136196	0.437804	0.766517	None
12369	LTBP4	-0.07041	0.437823	0.766517	None
12370	BANP	-0.11774	0.437871	0.76654	None
12371	DHCR7	0.162924	0.43796	0.766634	None
12372	RP11-433A	-0.00642	0.43804	0.766678	None
12373	DHDH	-0.04299	0.438063	0.766678	None
12374	PAPOLB	-0.00524	0.4381	0.766678	None
12375	SEC14L4	-0.09592	0.438153	0.766678	None
12376	GLT8D2	-0.04246	0.438162	0.766678	None
12377	RAB40AL	-0.0066	0.438324	0.766773	None
12378	LYPD3	-0.03198	0.438324	0.766773	None
12379	PLEKHA5	-0.11023	0.438325	0.766773	None
12380	PDS5A	0.096683	0.438358	0.766773	None
12381	SLC25A32	0.098468	0.438439	0.766851	None
12382	IQCJ-SCHI	-0.01406	0.438762	0.767355	None
12383	RP11-73M	-0.01509	0.438851	0.767449	None
12384	CASK	-0.04066	0.439031	0.767648	None
12385	AL928742.1	-0.01235	0.439036	0.767648	None
12386	DACH1	0.188009	0.439321	0.768045	None
12387	ZDHHC8P1	-0.02365	0.439335	0.768045	None
12388	PDPR	-0.14394	0.43937	0.768045	None
12389	C1orf109	-0.14382	0.439417	0.768045	None
12390	LOC22007	-0.00626	0.439447	0.768045	None
12391	RP11-278J	-0.01327	0.439488	0.768045	None
12392	FANCF	-0.10951	0.439511	0.768045	None
12393	UNC5B	0.05803	0.439581	0.768104	None
12394	TRAPPC11	-0.10333	0.439841	0.768399	None
12395	APOL1	-0.13637	0.439848	0.768399	None
12396	IFT52	0.074502	0.439856	0.768399	None
12397	REV3L	0.120968	0.439951	0.768504	None
12398	TRIM17	-0.0185	0.439991	0.768512	None
12399	JRKL	-0.09446	0.440074	0.768557	None
12400	CALCOCO1	0.126931	0.440117	0.768557	None
12401	SPATA9	-0.01743	0.440154	0.768557	None
12402	NANS	-0.05485	0.440194	0.768557	None
12403	THSD7B	-0.01875	0.440216	0.768557	None
12404	GRASPOS	0.005644	0.440261	0.768557	None
12405	LGSN	-0.09131	0.440273	0.768557	None
12406	EPHX4	-0.00778	0.440337	0.768557	None
12407	LOC33869	-0.00588	0.440362	0.768557	None
12408	TEX28	0.003807	0.440429	0.768557	None
12409	TMIGD2	0.102809	0.440433	0.768557	None
12410	SPATA5	-0.17433	0.440495	0.768557	None

12411	HOXC10	-0.01259	0.44053	0.768557	None
12412	ASAH2B	-0.0301	0.440566	0.768557	None
12413	LSMEM2	-0.0182	0.440596	0.768557	None
12414	SLC25A41	-0.01757	0.440621	0.768557	None
12415	CNKSR2	-0.01663	0.440632	0.768557	None
12416	LOC10012ε	-0.04116	0.440688	0.768557	None
12417	RNASET2	-0.17379	0.440691	0.768557	None
12418	SLC37A3	0.14439	0.440749	0.768595	None
12419	HSP90AA1	-0.05745	0.440822	0.768625	None
12420	LINC01069	0.025967	0.440853	0.768625	None
12421	IL22	-0.00485	0.440872	0.768625	None
12422	LRRC58	0.054282	0.440939	0.768664	None
12423	BCR	0.179629	0.440966	0.768664	None
12424	MAP2K1	-0.10767	0.441061	0.768767	None
12425	B4GALT6	-0.11848	0.441111	0.768793	None
12426	LAYN	-0.01964	0.441276	0.768896	None
12427	SPCS2	0.097631	0.441314	0.768896	None
12428	USP32	-0.09664	0.441324	0.768896	None
12429	FOLR1	-0.02487	0.441329	0.768896	None
12430	ATP5J	0.072571	0.441373	0.768896	None
12431	LINC00173	-0.16526	0.441383	0.768896	None
12432	WFDC10B	-0.01757	0.441495	0.769029	None
12433	LOC10031(	-0.13011	0.441541	0.769047	None
12434	SCRN2	-0.08539	0.44162	0.769123	None
12435	TIGD7	-0.18482	0.441676	0.769155	None
12436	EMC3-AS1	-0.12439	0.441709	0.769155	None
12437	FLJ22763	-0.00435	0.441772	0.769155	None
12438	MKRN7P	-0.01506	0.441819	0.769155	None
12439	SYCP1	-0.05727	0.441872	0.769155	None
12440	LINC01115	0.040894	0.441953	0.769155	None
12441	AL590762.1	0.061186	0.441971	0.769155	None
12442	AGO1	-0.0735	0.442021	0.769155	None
12443	OSGIN1	-0.09419	0.442022	0.769155	None
12444	PRRG3	-0.0046	0.442026	0.769155	None
12445	FAM124A	-0.01465	0.442086	0.769155	None
12446	WDR78	-0.01596	0.442105	0.769155	None
12447	COX6A1	-0.07269	0.442125	0.769155	None
12448	KRT23	-0.06335	0.442135	0.769155	None
12449	PIAS1	-0.10065	0.442269	0.769295	None
12450	PLXNB2	-0.08254	0.442287	0.769295	None
12451	NR0B2	-0.00701	0.442338	0.769322	None
12452	LOC40069	-0.02322	0.44239	0.769351	None
12453	APOL2	-0.07321	0.442435	0.769367	None
12454	F7	-0.02662	0.442584	0.769387	None
12455	RPL23AP53	0.021367	0.442631	0.769387	None
12456	NLRP14	-0.01155	0.442682	0.769387	None
12457	SHANK3	-0.12905	0.442701	0.769387	None
12458	NEU3	0.013983	0.442707	0.769387	None
12459	ZNFX1	-0.13732	0.442717	0.769387	None
12460	DIP2B	0.172783	0.442736	0.769387	None
12461	RP11-416N	-0.12746	0.442744	0.769387	None
12462	SPINK1	0.135961	0.442806	0.769387	None
12463	PDZD9	-0.00486	0.442812	0.769387	None
12464	CTD-2130(	-0.00793	0.442857	0.769387	None
12465	ADH1C	0.032531	0.442873	0.769387	None
12466	INTS4L1	0.012969	0.443092	0.769707	None
12467	LOC10192	-0.01342	0.443381	0.770023	None
12468	IL16	-0.19082	0.443385	0.770023	None

12469	ASCC3	0.109804	0.443394	0.770023	None
12470	TDO2	-0.05504	0.443417	0.770023	None
12471	CLIP2	-0.14709	0.443497	0.770062	None
12472	MSX2	-0.00189	0.44353	0.770062	None
12473	ACOX3	-0.10654	0.44355	0.770062	None
12474	SPDYE2	-0.18187	0.443581	0.770062	None
12475	SFT2D2	0.079821	0.443628	0.770081	None
12476	LINC01082	-0.00925	0.443807	0.770304	None
12477	DERL2	-0.08691	0.443847	0.770304	None
12478	STK17A	0.136683	0.443874	0.770304	None
12479	PER3	0.036765	0.443898	0.770304	None
12480	RP11-791M	0.006668	0.443969	0.770329	None
12481	TSIX	0.206419	0.444057	0.770329	None
12482	PIGC	-0.12569	0.444135	0.770329	None
12483	DSTYK	-0.05953	0.444142	0.770329	None
12484	RP5-1031D	0.024075	0.444214	0.770329	None
12485	LOC10192	-0.01115	0.444234	0.770329	None
12486	RP11-195M	-0.00829	0.444237	0.770329	None
12487	CABP5	-0.08725	0.444242	0.770329	None
12488	MYL12A	0.136208	0.444253	0.770329	None
12489	LINC00911	0.012901	0.444289	0.770329	None
12490	CA7	-0.01657	0.444353	0.770329	None
12491	CEBPA	-0.19631	0.444372	0.770329	None
12492	CTD-2366I	-0.03009	0.444375	0.770329	None
12493	PDCL2	-0.01239	0.444449	0.770353	None
12494	LINC00313	-0.00736	0.44446	0.770353	None
12495	APOE	-0.40515	0.444699	0.770659	None
12496	IL15	-0.19515	0.444708	0.770659	None
12497	LINC01405	-0.02102	0.444844	0.770829	None
12498	TMEM30A	0.140859	0.444914	0.770829	None
12499	CTF1	0.035172	0.444934	0.770829	None
12500	LINC01023	-0.10936	0.444949	0.770829	None
12501	ECSCR	-0.11385	0.444987	0.770834	None
12502	GRIA1	-0.01755	0.445091	0.770953	None
12503	WDR44	-0.04431	0.445173	0.771032	None
12504	CYP39A1	-0.08774	0.445287	0.771066	None
12505	CSNK1G2	-0.10099	0.445309	0.771066	None
12506	CRIPAK	-0.09263	0.445327	0.771066	None
12507	MAPKAPK5	-0.10765	0.445338	0.771066	None
12508	RP4-593C1	-0.0042	0.44537	0.771066	None
12509	RNF182	-0.31253	0.445482	0.771198	None
12510	SLC35F5	0.159971	0.445528	0.771217	None
12511	KHK	-0.05418	0.445607	0.771264	None
12512	COMT	-0.09434	0.445644	0.771264	None
12513	VCP	-0.08066	0.445663	0.771264	None
12514	ACPP	-0.02046	0.44587	0.771556	None
12515	LINC00687	0.013187	0.445903	0.771556	None
12516	CAMK1G	0.015012	0.446024	0.771705	None
12517	USP30-AS1	-0.08312	0.446089	0.771734	None
12518	TMEM194B	0.114946	0.446112	0.771734	None
12519	DIRAS1	-0.03174	0.446285	0.771948	None
12520	TBX1	-0.01308	0.446307	0.771948	None
12521	LOC10192	0.02566	0.446373	0.772	None
12522	CDH26	0.099287	0.446426	0.77201	None
12523	ISM1	-0.01075	0.44646	0.77201	None
12524	C6orf226	0.084385	0.446486	0.77201	None
12525	SP5	-0.01232	0.446559	0.772019	None
12526	PRSS37	0.020851	0.446567	0.772019	None

12527	SLC6A2	-0.00765	0.446598	0.772019	None
12528	LIN9	-0.02197	0.446692	0.772049	None
12529	MSANTD1	-0.00551	0.446698	0.772049	None
12530	CELSR1	-0.00258	0.446722	0.772049	None
12531	GLYATL1	-0.07197	0.446797	0.772103	None
12532	GHET1	-0.00456	0.446825	0.772103	None
12533	PRG4	-0.121	0.446876	0.772129	None
12534	TATDN1	-0.09992	0.447003	0.772288	None
12535	MEP1A	-0.02112	0.447072	0.772346	None
12536	GPX5	-0.00714	0.447289	0.772658	None
12537	RBM26-AS	-0.09397	0.447473	0.772914	None
12538	RRP8	-0.03789	0.447553	0.772921	None
12539	ETNK1	0.11621	0.447577	0.772921	None
12540	LINC01425	0.002223	0.447584	0.772921	None
12541	MRPL33	-0.11902	0.447718	0.773091	None
12542	HMGCS2	0.012786	0.447834	0.773184	None
12543	CTD-2619	-0.04839	0.447849	0.773184	None
12544	CCBL2	0.072726	0.447879	0.773184	None
12545	HIST1H3A	-0.04812	0.447957	0.773257	None
12546	CFD	-0.33034	0.448111	0.773397	None
12547	RNF19A	-0.10769	0.448205	0.773397	None
12548	HPCAL4	0.021373	0.448211	0.773397	None
12549	LINC00427	0.007654	0.448216	0.773397	None
12550	TTC19	0.102292	0.448278	0.773397	None
12551	LRBA	-0.11727	0.448294	0.773397	None
12552	DDC-AS1	-0.00683	0.44834	0.773397	None
12553	KB-431C1.	-0.19777	0.448359	0.773397	None
12554	RPS10	-0.06789	0.448394	0.773397	None
12555	TIMM10	-0.13601	0.448395	0.773397	None
12556	ECM1	-0.06727	0.448433	0.7734	None
12557	ZFAND2A	-0.12705	0.448579	0.773475	None
12558	TVP23B	0.164673	0.448586	0.773475	None
12559	RP3-336K2	0.016895	0.448588	0.773475	None
12560	LOC10192	-0.0079	0.44865	0.773475	None
12561	PPM1J	-0.11494	0.448655	0.773475	None
12562	DYNC1LI2	0.107569	0.4488	0.773664	None
12563	RGS7	-0.00735	0.448847	0.773684	None
12564	LOC33956	0.015902	0.448894	0.773702	None
12565	FGD2	-0.04487	0.448938	0.773717	None
12566	ABCC3	-0.06392	0.449002	0.773765	None
12567	LTBP2	-0.11244	0.449153	0.773965	None
12568	CRIP2	0.019305	0.449321	0.774137	None
12569	RIBC2	-0.12143	0.449325	0.774137	None
12570	GOLGA4	0.105054	0.449397	0.7742	None
12571	LOC10027	-0.21431	0.449472	0.774268	None
12572	LINC00900	-0.02649	0.449608	0.774441	None
12573	MRPL39	0.102197	0.449687	0.774494	None
12574	RASGRP3	-0.11036	0.449742	0.774494	None
12575	RP11-400N	-0.01096	0.449747	0.774494	None
12576	TFCP2L1	-0.0297	0.44985	0.774599	None
12577	FGF19	-0.00526	0.449879	0.774599	None
12578	HIST1H4L	-0.03045	0.449924	0.774599	None
12579	TMEM51-A	-0.0078	0.449951	0.774599	None
12580	IHH	0.035992	0.450124	0.774835	None
12581	C16orf78	0.015194	0.450217	0.774934	None
12582	CHERP	-0.04411	0.450496	0.775295	None
12583	GOLGA6L2	-0.01223	0.450506	0.775295	None
12584	OSCP1	-0.02397	0.450534	0.775295	None

12585	MGC10814	-0.02315	0.450621	0.77535	None
12586	NGEF	-0.00575	0.450638	0.77535	None
12587	ZCCHC12	-0.0084	0.450754	0.775406	None
12588	BMPER	-0.01848	0.450777	0.775406	None
12589	ZNF385C	-0.06795	0.450864	0.775406	None
12590	TMEM191A	-0.04026	0.45088	0.775406	None
12591	ARHGEF7-	0.007181	0.450886	0.775406	None
12592	HSD17B8	0.136473	0.45089	0.775406	None
12593	RNF219	0.072264	0.450942	0.775406	None
12594	ENO1-AS1	-0.08322	0.450976	0.775406	None
12595	ABCF1	-0.09476	0.450992	0.775406	None
12596	OSGEP	-0.10502	0.451233	0.77571	None
12597	LOC10028	-0.08968	0.451241	0.77571	None
12598	PMP2	-0.15901	0.451322	0.775788	None
12599	LOC10050	-0.00514	0.451565	0.776024	None
12600	C6orf58	-0.00629	0.451612	0.776024	None
12601	TOLLIP-AS	-0.09623	0.451614	0.776024	None
12602	PRKACG	-0.02695	0.451676	0.776024	None
12603	LOC10192	-0.01686	0.451684	0.776024	None
12604	C1orf192	-0.05141	0.451709	0.776024	None
12605	DQ599616	0.015768	0.451727	0.776024	None
12606	HNRNPK	0.063135	0.451756	0.776024	None
12607	SCARNA15	0.096139	0.451812	0.776024	None
12608	TLE6	-0.00923	0.451833	0.776024	None
12609	A2MP1	-0.02159	0.451853	0.776024	None
12610	MC1R	-0.04985	0.451971	0.776164	None
12611	COL23A1	-0.19253	0.452068	0.776178	None
12612	LOC10192	0.044785	0.452085	0.776178	None
12613	GNAQ	0.111779	0.452106	0.776178	None
12614	CHST11	0.108848	0.452122	0.776178	None
12615	ATP5I	-0.12151	0.452313	0.776444	None
12616	SMIM8	0.065189	0.452382	0.7765	None
12617	MAGEB6	0.008036	0.452428	0.776519	None
12618	FST	0.154622	0.452488	0.776533	None
12619	RP11-274H	-0.02364	0.452508	0.776533	None
12620	AK9	-0.09983	0.452558	0.776557	None
12621	TBC1D25	0.045717	0.452795	0.776856	None
12622	ARHGEF26	-0.07138	0.452841	0.776856	None
12623	SCNN1B	-0.02181	0.452871	0.776856	None
12624	HPGDS	0.33135	0.452876	0.776856	None
12625	RP11-301C	-0.07778	0.452916	0.776864	None
12626	UBE2V2	-0.13779	0.452991	0.776864	None
12627	ALX1	-0.01724	0.453026	0.776864	None
12628	ASF1A	-0.09054	0.453029	0.776864	None
12629	NOLC1	0.13496	0.45306	0.776864	None
12630	SURF1	-0.083	0.453139	0.776892	None
12631	SLIT2	-0.02442	0.453148	0.776892	None
12632	RP11-787E	-0.01069	0.45322	0.776893	None
12633	BTBD11	-0.15684	0.453258	0.776893	None
12634	BC022892	0.155129	0.453269	0.776893	None
12635	C17orf97	-0.08731	0.453292	0.776893	None
12636	DPYSL5	0.009649	0.453391	0.777	None
12637	MTMR9	0.121659	0.453579	0.777193	None
12638	LOC10192	-0.11302	0.453585	0.777193	None
12639	CAGE1	0.009778	0.453611	0.777193	None
12640	PAQR9	-0.08154	0.453684	0.777256	None
12641	ZC3H18	-0.09705	0.453759	0.777324	None
12642	LOC15487	-0.01344	0.453895	0.777479	None

12643	PMS2P1	-0.08085	0.453921	0.777479	None
12644	STGC3	-0.00364	0.453959	0.777482	None
12645	ETNPPL	-0.00617	0.454038	0.777527	None
12646	SFTA2	-0.01726	0.454057	0.777527	None
12647	RP1-149A1	-0.02219	0.454256	0.777784	None
12648	PSMA5	-0.08755	0.454306	0.777784	None
12649	MAGEH1	0.117428	0.454382	0.777784	None
12650	PCP4L1	0.029904	0.454383	0.777784	None
12651	LOC28456	-0.06145	0.454387	0.777784	None
12652	VPS9D1-A	-0.03106	0.454479	0.77788	None
12653	TPSG1	-0.02115	0.454696	0.77814	None
12654	LOC10192	-0.01587	0.454702	0.77814	None
12655	GLB1L3	-0.00552	0.454765	0.778185	None
12656	LRRC55	0.013985	0.454841	0.778253	None
12657	PKD1L2	0.009217	0.454933	0.778349	None
12658	MYH7	-0.0016	0.455027	0.778373	None
12659	FAM76B	0.092185	0.455051	0.778373	None
12660	CDX2	-0.02582	0.455054	0.778373	None
12661	SAP130	-0.09206	0.455148	0.778433	None
12662	RPL3L	-0.02813	0.455183	0.778433	None
12663	RHOV	-0.03039	0.455197	0.778433	None
12664	SPG7	-0.01213	0.455259	0.778449	None
12665	LOC10013	-0.03382	0.455279	0.778449	None
12666	FBXO40	-0.01184	0.455387	0.778573	None
12667	PLA2G3	0.042694	0.455467	0.77858	None
12668	RFC1	-0.07884	0.455508	0.77858	None
12669	TREML3P	-0.03707	0.455531	0.77858	None
12670	GRIP2	-0.02177	0.455574	0.77858	None
12671	ACAD9	-0.11335	0.455578	0.77858	None
12672	DENND3	-0.08514	0.455634	0.77858	None
12673	NPSR1-AS	-0.00583	0.455643	0.77858	None
12674	RAPGEFL1	-0.01006	0.455707	0.778629	None
12675	GUCY2D	-0.0304	0.455773	0.77868	None
12676	GBP6	0.006578	0.455862	0.778705	None
12677	APLNR	0.061786	0.455935	0.778705	None
12678	RRS1-AS1	-0.00162	0.455958	0.778705	None
12679	ND6	0.213323	0.456005	0.778705	None
12680	DPPA2	0.047407	0.456034	0.778705	None
12681	LOC10192	-0.00316	0.456058	0.778705	None
12682	DFNB31	-0.00552	0.456096	0.778705	None
12683	PAX4	-0.00354	0.456109	0.778705	None
12684	RASA3	-0.01835	0.456111	0.778705	None
12685	C10orf85	-0.02214	0.456292	0.778952	None
12686	IKBKAP	-0.05537	0.456368	0.778963	None
12687	DLGAP1-A	-0.03057	0.456433	0.778963	None
12688	PPARA	-0.06388	0.456437	0.778963	None
12689	MRGPRX4	0.041338	0.456443	0.778963	None
12690	OLIG1	-0.20407	0.456608	0.779184	None
12691	CHRNA10	0.002032	0.456674	0.779235	None
12692	GNA14	0.020897	0.456767	0.779314	None
12693	FBXW11	0.039889	0.456862	0.779314	None
12694	ABCG4	-0.02192	0.456864	0.779314	None
12695	PITPNB	-0.06609	0.456864	0.779314	None
12696	RP1-100J1	-0.0376	0.45692	0.779348	None
12697	SLC6A4	-0.09794	0.457002	0.779393	None
12698	RPGRIP1	-0.01691	0.457018	0.779393	None
12699	SRFBP1	0.151231	0.457185	0.779471	None
12700	PLA2G12B	-0.01205	0.457215	0.779471	None

12701	IGLV6-57	-0.02744	0.45723	0.779471	None
12702	PUS7L	-0.10108	0.457235	0.779471	None
12703	MIR7-3HG	-0.00793	0.457246	0.779471	None
12704	CPNE4	-0.02957	0.45728	0.779471	None
12705	SNAI1	0.027997	0.457574	0.779892	None
12706	TNAP	-0.02796	0.457616	0.779892	None
12707	RP11-44F2	-0.12856	0.457635	0.779892	None
12708	KCNK10	-0.00551	0.457717	0.77997	None
12709	STARD3NL	-0.08009	0.457844	0.779986	None
12710	MRAP2	-0.11663	0.457866	0.779986	None
12711	SEMA3G	0.104305	0.457873	0.779986	None
12712	FARP1	-0.06568	0.457889	0.779986	None
12713	CD1A	-0.01893	0.457937	0.779986	None
12714	LOC72880!	-0.00263	0.457993	0.779986	None
12715	RAD50	-0.14786	0.457999	0.779986	None
12716	ST7-AS2	-0.00343	0.458036	0.779986	None
12717	RP1-142L7	0.002598	0.458051	0.779986	None
12718	SEC1P	-0.01751	0.458204	0.780129	None
12719	DDX3Y	-0.29104	0.458227	0.780129	None
12720	LILRB4	-0.0683	0.458242	0.780129	None
12721	DLG5-AS1	-0.0209	0.458398	0.780333	None
12722	ADRBK2	-0.11985	0.458442	0.780345	None
12723	RASGRP4	-0.0238	0.458537	0.780446	None
12724	CDNF	-0.00302	0.458856	0.780927	None
12725	SNRNP35	0.1561	0.458947	0.780987	None
12726	PAPSS2	-0.16852	0.458963	0.780987	None
12727	GS1-279B7	-0.01765	0.459059	0.781089	None
12728	GTF2I	0.08212	0.459181	0.781154	None
12729	MDC1	-0.07872	0.459186	0.781154	None
12730	LINC01220	-0.01931	0.459205	0.781154	None
12731	PIGA	0.125357	0.459252	0.781171	None
12732	SGSM3	-0.06775	0.45935	0.781278	None
12733	ZXDC	0.10331	0.459468	0.781416	None
12734	KIAA1143	0.058507	0.459541	0.78148	None
12735	FAM159A	-0.00416	0.459682	0.781606	None
12736	BCL6	0.158268	0.459688	0.781606	None
12737	UAP1	-0.09311	0.459734	0.781624	None
12738	MTO1	0.083621	0.459794	0.781663	None
12739	PRRG2	-0.0102	0.45997	0.781818	None
12740	NUDT16L1	-0.10867	0.459978	0.781818	None
12741	LOC10192!	-0.01773	0.460005	0.781818	None
12742	ULBP3	-0.01105	0.460029	0.781818	None
12743	LINC01289	-0.02437	0.460083	0.781848	None
12744	RP11-467D	0.039425	0.460302	0.781912	None
12745	LAMP5	-0.13451	0.460395	0.781912	None
12746	CKM	-0.07519	0.460497	0.781912	None
12747	KLK1	-0.0257	0.460502	0.781912	None
12748	CSPG4	0.012283	0.460506	0.781912	None
12749	ZNF740	-0.05535	0.460525	0.781912	None
12750	GTF2A1L	0.004163	0.460556	0.781912	None
12751	PRDX1	0.118994	0.460579	0.781912	None
12752	FANCD2OS	0.012297	0.460579	0.781912	None
12753	PCDHGA8	-0.0463	0.460634	0.781912	None
12754	USP37	-0.10247	0.460656	0.781912	None
12755	TRH	0.214788	0.460686	0.781912	None
12756	ARL13B	-0.1063	0.460701	0.781912	None
12757	GPC1	-0.02078	0.460719	0.781912	None
12758	ENAH	-0.15146	0.460745	0.781912	None

12759	LOC10192	-0.02224	0.460752	0.781912	None
12760	SND1	-0.07481	0.460797	0.781912	None
12761	FURIN	-0.10687	0.460797	0.781912	None
12762	ZNF449	-0.07582	0.46085	0.781912	None
12763	TM4SF5	-0.00744	0.460919	0.781912	None
12764	THYN1	-0.07748	0.460924	0.781912	None
12765	AX746830	-0.00576	0.460983	0.781912	None
12766	STAMBP	-0.09583	0.461015	0.781912	None
12767	UQCRFS1	-0.08525	0.461037	0.781912	None
12768	MOV10L1	-0.00348	0.461064	0.781912	None
12769	PSMD10	0.093006	0.46108	0.781912	None
12770	FADD	-0.16597	0.461095	0.781912	None
12771	PLEKHM3	-0.08665	0.461208	0.782042	None
12772	NFATC4	-0.00842	0.461333	0.782145	None
12773	LINC00929	-0.00762	0.461368	0.782145	None
12774	RP1-265C2	-0.0214	0.461377	0.782145	None
12775	LOC10192	0.006409	0.461442	0.782177	None
12776	ENAM	-0.0044	0.461489	0.782177	None
12777	BRIX1	-0.07679	0.461532	0.782177	None
12778	WDR5	-0.08787	0.46154	0.782177	None
12779	CD55	-0.13391	0.461585	0.782192	None
12780	ALDH1L1-/	-0.01867	0.461669	0.782272	None
12781	FAM134C	0.11736	0.461721	0.782282	None
12782	AF067845.:	-0.00331	0.461759	0.782282	None
12783	ADM2	-0.03776	0.461783	0.782282	None
12784	LMBRD1	-0.07492	0.461856	0.782345	None
12785	SPP2	-0.01864	0.461938	0.782391	None
12786	TMEM30B	-0.03274	0.461956	0.782391	None
12787	PDYN	0.001138	0.462027	0.782405	None
12788	CLDN23	0.014516	0.462036	0.782405	None
12789	CCAR2	-0.13559	0.462077	0.782413	None
12790	LOC10192	-0.0072	0.462124	0.782431	None
12791	SCARNA2	-0.10186	0.462226	0.782539	None
12792	SLC9A8	-0.07389	0.462268	0.782539	None
12793	C9orf117	-0.02459	0.462296	0.782539	None
12794	DUX1	-0.03207	0.462438	0.782579	None
12795	PLIN1	0.001019	0.462449	0.782579	None
12796	ERVH-4	-0.04675	0.462458	0.782579	None
12797	RBM7	0.073323	0.462464	0.782579	None
12798	AF086184	0.020437	0.462618	0.782686	None
12799	AX747064	-0.0073	0.462619	0.782686	None
12800	SKA1	-0.16611	0.462639	0.782686	None
12801	CYSLTR1	0.163882	0.462672	0.782686	None
12802	RP11-1069	0.031857	0.462791	0.782827	None
12803	SOX1	-0.00489	0.462948	0.783031	None
12804	RASSF6	-0.16241	0.463075	0.783063	None
12805	COMTD1	-0.11178	0.463105	0.783063	None
12806	LINC01293	-0.01957	0.463105	0.783063	None
12807	LINC00636	0.027628	0.463129	0.783063	None
12808	HLA-DQA:	0.359532	0.463148	0.783063	None
12809	SGK1	0.3291	0.463283	0.783198	None
12810	ZNF112	0.173504	0.4633	0.783198	None
12811	RP11-96D:	0.007331	0.463368	0.783251	None
12812	TSR3	-0.06177	0.463491	0.783347	None
12813	ELOVL2	0.039687	0.463497	0.783347	None
12814	CAV3	-0.00299	0.463548	0.783373	None
12815	AY927499	-0.06698	0.463752	0.783656	None
12816	TSC22D1-/	-0.08117	0.463878	0.783793	None

12817	FBF1	-0.05293	0.463922	0.783793	None
12818	LOC10012!	-0.03634	0.463942	0.783793	None
12819	MDFIC	0.200405	0.46402	0.783861	None
12820	IRF1	-0.1857	0.464054	0.783861	None
12821	SLC25A21	-0.25805	0.464152	0.783965	None
12822	RPLP2	-0.06941	0.464288	0.784133	None
12823	AVPI1	0.140791	0.464349	0.784146	None
12824	BTBD17	-0.00635	0.464408	0.784146	None
12825	PYY2	-0.08204	0.464424	0.784146	None
12826	TTC29	-0.0606	0.46444	0.784146	None
12827	LSAMP-AS	-0.03293	0.464634	0.784412	None
12828	PCYT2	-0.06659	0.464718	0.784493	None
12829	CUX2	-0.06092	0.464943	0.784811	None
12830	DAB1-AS1	-0.00264	0.465006	0.784846	None
12831	WDR74	-0.10211	0.465089	0.784846	None
12832	NTF3	0.02823	0.465122	0.784846	None
12833	WNT8B	-0.03795	0.465129	0.784846	None
12834	GPRIN1	-0.00841	0.465145	0.784846	None
12835	FTCDNL1	-0.12257	0.465231	0.784902	None
12836	ABLIM1	0.137794	0.46534	0.784902	None
12837	RP11-209D	-0.04817	0.465392	0.784902	None
12838	ANXA7	0.061854	0.465415	0.784902	None
12839	PPP1R15A	-0.22125	0.465522	0.784902	None
12840	WFDC11	0.005169	0.465526	0.784902	None
12841	IFNL1	-0.01169	0.46554	0.784902	None
12842	TPK1	-0.15603	0.465554	0.784902	None
12843	TMPO-AS1	0.056109	0.465566	0.784902	None
12844	ABI3BP	0.009763	0.465569	0.784902	None
12845	ENTPD7	0.085219	0.465605	0.784902	None
12846	EDN3	-0.00461	0.465617	0.784902	None
12847	GSN	-0.02824	0.465732	0.784902	None
12848	ITGA8	-0.02221	0.465737	0.784902	None
12849	NLGN4X	0.036798	0.465801	0.784902	None
12850	ASB15	-0.00634	0.465834	0.784902	None
12851	NUTM1	0.022764	0.465873	0.784902	None
12852	CCNA2	0.188519	0.46588	0.784902	None
12853	CCNY	-0.10458	0.46591	0.784902	None
12854	LOC10272:	-0.01352	0.46594	0.784902	None
12855	TPGS1	-0.05643	0.465968	0.784902	None
12856	DOK5	-0.05568	0.465993	0.784902	None
12857	YARS	-0.10208	0.466026	0.784902	None
12858	NLRP11	-0.0576	0.466048	0.784902	None
12859	TNFSF4	0.23166	0.466216	0.785124	None
12860	SRSF5	0.097893	0.466332	0.78525	None
12861	USMG5	-0.07445	0.4664	0.78525	None
12862	UBXN4	-0.08856	0.466408	0.78525	None
12863	DHX33	-0.10949	0.466476	0.78525	None
12864	WHSC1	0.114218	0.466492	0.78525	None
12865	DARS2	-0.10468	0.466508	0.78525	None
12866	CCL22	-0.01996	0.466585	0.785318	None
12867	LOC44034(	-0.00798	0.466646	0.785359	None
12868	WNT2	-0.02928	0.46683	0.785474	None
12869	IFNB1	-0.0156	0.466834	0.785474	None
12870	RP11-109N	0.032508	0.466859	0.785474	None
12871	ALK	-0.02597	0.466859	0.785474	None
12872	LOC10192'	-0.00728	0.46699	0.785518	None
12873	CTC-471F3	-0.03934	0.467006	0.785518	None
12874	KIAA1462	-0.04322	0.467021	0.785518	None

12875	TEX38	-0.01687	0.467031	0.785518	None
12876	OSGIN2	-0.06548	0.467144	0.785626	None
12877	GID8	-0.07296	0.467189	0.785626	None
12878	CHRNA6	-0.00524	0.46722	0.785626	None
12879	ATP5L	-0.08464	0.46724	0.785626	None
12880	PBOV1	-0.00835	0.467277	0.785627	None
12881	TUBGCP3	-0.11462	0.467419	0.785775	None
12882	INHBE	-0.00671	0.467437	0.785775	None
12883	KIDINS220	-0.15061	0.467549	0.785808	None
12884	TFG	-0.1143	0.467583	0.785808	None
12885	STRA13	-0.11832	0.467601	0.785808	None
12886	PNPT1	-0.1007	0.467602	0.785808	None
12887	TRIB1	-0.00304	0.467694	0.785893	None
12888	RAB1A	0.083696	0.467793	0.785893	None
12889	ANKRD28	-0.16243	0.467797	0.785893	None
12890	MPPED1	-0.01452	0.467798	0.785893	None
12891	KIAA1377	-0.16561	0.467889	0.785921	None
12892	MFSD11	-0.06714	0.467901	0.785921	None
12893	PPIC	-0.14734	0.467936	0.785921	None
12894	FAS-AS1	-0.00675	0.467968	0.785921	None
12895	SEC14L5	-0.15927	0.467996	0.785921	None
12896	NLRP13	-0.01044	0.468105	0.786043	None
12897	INS	-0.00822	0.468391	0.78643	None
12898	SELP	-0.32337	0.468448	0.78643	None
12899	BANCR	-0.00625	0.468451	0.78643	None
12900	UCKL1	-0.08506	0.468514	0.78643	None
12901	GPR88	-0.13748	0.468551	0.78643	None
12902	MYL6B	0.104819	0.468595	0.78643	None
12903	CCDC26	0.254709	0.468612	0.78643	None
12904	BPIFA4P	0.005738	0.468626	0.78643	None
12905	MMP17	-0.01703	0.468666	0.786436	None
12906	LOC10192[illegible]	-0.0238	0.468721	0.786467	None
12907	FUT5	0.049308	0.468967	0.786726	None
12908	LOC10159[illegible]	-0.04253	0.468989	0.786726	None
12909	ZNF471	-0.04676	0.469017	0.786726	None
12910	PRPF19	-0.12796	0.46902	0.786726	None
12911	CSNK1D	-0.14104	0.469211	0.786984	None
12912	FERMT1	-0.18303	0.469265	0.786995	None
12913	UBE3A	0.087045	0.469289	0.786995	None
12914	LOC64673[illegible]	-0.0039	0.469427	0.787075	None
12915	LTF	0.503155	0.469432	0.787075	None
12916	METTL17	-0.10203	0.469521	0.787075	None
12917	FBXW8	0.049068	0.469522	0.787075	None
12918	RAD23B	-0.09836	0.469546	0.787075	None
12919	SENP2	-0.1069	0.469556	0.787075	None
12920	C1orf95	-0.03642	0.469767	0.787232	None
12921	CHAF1B	0.123652	0.469792	0.787232	None
12922	BC036311	-0.01585	0.469825	0.787232	None
12923	TBCE	-0.08171	0.469842	0.787232	None
12924	A1BG	0.045543	0.469896	0.787232	None
12925	LINC01136	-0.01065	0.469899	0.787232	None
12926	GPN1	0.075737	0.469948	0.787232	None
12927	CILP2	-0.01785	0.469978	0.787232	None
12928	LOC40131[illegible]	-0.00249	0.470051	0.787232	None
12929	ICA1L	0.038203	0.470091	0.787232	None
12930	LOC10050[illegible]	-0.11068	0.470119	0.787232	None
12931	SYDE2	-0.07003	0.470122	0.787232	None
12932	AC104667.[illegible]	-0.01295	0.470187	0.787232	None

12933	RIN1	-0.06113	0.470264	0.787232	None
12934	SHC4	-0.00937	0.470294	0.787232	None
12935	KCNC1	-0.01636	0.470322	0.787232	None
12936	AAED1	-0.12965	0.47033	0.787232	None
12937	LOC72917:	-0.01228	0.470352	0.787232	None
12938	LRRC4	-0.04703	0.470362	0.787232	None
12939	EFCAB5	-0.00438	0.470401	0.787232	None
12940	FBXO30	-0.08689	0.470412	0.787232	None
12941	MEP1B	-0.0054	0.470573	0.787439	None
12942	ATP9A	0.149271	0.470637	0.787485	None
12943	LOC10028!	-0.09761	0.470685	0.787485	None
12944	FAM131C	-0.02532	0.470845	0.787485	None
12945	MIR31HG	-0.00267	0.470856	0.787485	None
12946	KISS1	-0.00251	0.470859	0.787485	None
12947	RP1-140C:	-0.01173	0.470867	0.787485	None
12948	ERC2	-0.05038	0.470889	0.787485	None
12949	OOSP2	0.007529	0.470921	0.787485	None
12950	PRICKLE2	-0.04806	0.47097	0.787485	None
12951	UCHL3	-0.09158	0.470984	0.787485	None
12952	GLS2	-0.01185	0.471	0.787485	None
12953	ASH2L	-0.10369	0.471171	0.787565	None
12954	MYCL	0.019318	0.471251	0.787565	None
12955	TMEM161E	-0.08528	0.471282	0.787565	None
12956	LOC10192	0.038705	0.471292	0.787565	None
12957	KRT81	-0.00899	0.471324	0.787565	None
12958	LEMD1-AS	-0.00491	0.471334	0.787565	None
12959	RFX2	-0.09396	0.471349	0.787565	None
12960	PCDHA9	0.026066	0.471361	0.787565	None
12961	CTC-360P!	-0.02085	0.471375	0.787565	None
12962	CYP1B1-A:	-0.00659	0.471436	0.78757	None
12963	SCAMP1	-0.08926	0.471451	0.78757	None
12964	ZNF503-A:	-0.00703	0.471556	0.787631	None
12965	TRMT13	-0.16292	0.47156	0.787631	None
12966	KANSL1	0.103977	0.471653	0.787725	None
12967	RP1-58B11	0.044583	0.47181	0.787772	None
12968	LOC10024(	-0.00236	0.47182	0.787772	None
12969	LRCH1	-0.10342	0.471877	0.787772	None
12970	DCUN1D1	0.087916	0.471882	0.787772	None
12971	FCN3	-0.0537	0.471898	0.787772	None
12972	C1orf64	-0.03207	0.471959	0.787772	None
12973	CECR9	0.011029	0.471987	0.787772	None
12974	RAB3C	-0.03098	0.471994	0.787772	None
12975	H1FOO	0.02052	0.472059	0.787772	None
12976	SCGN	-0.01834	0.472099	0.787772	None
12977	PLEKHG3	-0.00819	0.472115	0.787772	None
12978	LOC10192{	-0.01195	0.472136	0.787772	None
12979	ZNF90	0.025152	0.472154	0.787772	None
12980	XPR1	-0.0981	0.472211	0.787806	None
12981	WDR65	-0.01448	0.472273	0.787826	None
12982	HMMR	0.210688	0.472295	0.787826	None
12983	PSMB8	-0.10749	0.472344	0.787846	None
12984	LOC10013:	-0.19511	0.472475	0.787988	None
12985	ZNF491	-0.01352	0.472502	0.787988	None
12986	EXOC3L2	-0.07325	0.472558	0.788002	None
12987	GGN	-0.00745	0.472605	0.788002	None
12988	MGC32805	-0.00847	0.472689	0.788002	None
12989	TYRO3P	-0.00551	0.472738	0.788002	None
12990	SLAIN2	-0.11774	0.472741	0.788002	None

12991	LMOD2	-0.01244	0.472746	0.788002	None
12992	LOC10012	0.008907	0.472765	0.788002	None
12993	PRTG	-0.14008	0.472875	0.788073	None
12994	KLF14	-0.01465	0.472905	0.788073	None
12995	IMPAD1	0.100736	0.47295	0.788073	None
12996	LOC10028	0.025298	0.472953	0.788073	None
12997	SERPINB5	-0.0053	0.47301	0.788092	None
12998	TAAR8	-0.01021	0.473037	0.788092	None
12999	TTR	-0.02088	0.473185	0.788277	None
13000	NEXN-AS1	-0.00453	0.473389	0.788508	None
13001	CPA1	-0.00169	0.473396	0.788508	None
13002	ANKRD40	0.068589	0.473452	0.78854	None
13003	RP11-329E	-0.02845	0.473516	0.788586	None
13004	CTB-43E15	-0.01355	0.473642	0.788649	None
13005	EBPL	0.099956	0.47365	0.788649	None
13006	FZD4	-0.06765	0.473666	0.788649	None
13007	COLEC11	-0.06906	0.473699	0.788649	None
13008	MLH1	0.074669	0.473756	0.788683	None
13009	KCNJ13	-0.00624	0.473867	0.788735	None
13010	SLC5A8	-0.00596	0.473915	0.788735	None
13011	RP11-635N	-0.02105	0.473919	0.788735	None
13012	TSN	0.075371	0.474009	0.788735	None
13013	PDZK1	-0.02384	0.47401	0.788735	None
13014	GNAL	-0.01373	0.474011	0.788735	None
13015	NOBOX	0.009194	0.474042	0.788735	None
13016	KIF25-AS1	-0.01597	0.474106	0.78878	None
13017	DACH2	-0.00522	0.47425	0.788935	None
13018	LOC28358	-0.13022	0.474289	0.788935	None
13019	LOC10192	-0.01857	0.474308	0.788935	None
13020	EGOT	0.033595	0.474419	0.789058	None
13021	OR7A17	-0.00567	0.474715	0.78949	None
13022	AOC2	0.007726	0.474822	0.789586	None
13023	P2RX6	0.00772	0.474846	0.789586	None
13024	BGN	-0.04079	0.474928	0.789645	None
13025	CAPG	0.189409	0.474954	0.789645	None
13026	MEGF9	-0.10468	0.475136	0.789742	None
13027	RP11-332H	-0.00597	0.47517	0.789742	None
13028	LOC72874	-0.00865	0.475172	0.789742	None
13029	MYO16	-0.08646	0.475188	0.789742	None
13030	CBLN3	0.050978	0.475195	0.789742	None
13031	C7orf63	-0.03221	0.475384	0.789957	None
13032	CGREF1	-0.12023	0.475423	0.789957	None
13033	PLCZ1	-0.00356	0.475462	0.789957	None
13034	ABHD12	-0.13327	0.475474	0.789957	None
13035	TSKS	-0.03209	0.475506	0.789957	None
13036	LOC10192	-0.006	0.475559	0.789963	None
13037	SMYD2	-0.08229	0.475613	0.789963	None
13038	ZNF891	-0.07535	0.475619	0.789963	None
13039	TBC1D30	-0.04053	0.475822	0.790239	None
13040	HNRNPLL	-0.17969	0.475914	0.790331	None
13041	GNAT1	-0.00563	0.47599	0.790397	None
13042	METTL3	0.154566	0.476114	0.790543	None
13043	IL17C	-0.0509	0.476169	0.790572	None
13044	UPF3B	0.059762	0.476513	0.791027	None
13045	RRP12	-0.10696	0.476526	0.791027	None
13046	OR5H1	-0.01067	0.476575	0.791027	None
13047	ENDOD1	-0.1513	0.476619	0.791027	None
13048	TIGD1	-0.15683	0.476626	0.791027	None

13049	LINC01165	0.017652	0.476662	0.791027	None
13050	RPL18	-0.0571	0.476706	0.79104	None
13051	RGPD4-AS	-0.0029	0.476746	0.791045	None
13052	MAGI3	-0.03294	0.476794	0.791064	None
13053	FNBP4	-0.11201	0.476906	0.79119	None
13054	RP11-408I	-0.01164	0.476979	0.791203	None
13055	CDC14A	-0.12915	0.476998	0.791203	None
13056	CNTNAP3F	-0.01565	0.477041	0.791203	None
13057	CTD-2541	-0.00293	0.47706	0.791203	None
13058	LOC10192	-0.05144	0.477108	0.791222	None
13059	S100PBP	-0.10636	0.477255	0.791399	None
13060	LINC00527	0.008171	0.477317	0.791399	None
13061	OR7C1	-0.02368	0.477324	0.791399	None
13062	ZSWIM4	-0.01725	0.477379	0.791429	None
13063	HELB	-0.08949	0.477642	0.791749	None
13064	LINC01187	-0.01397	0.477645	0.791749	None
13065	PAK3	-0.01125	0.477707	0.79179	None
13066	ALLC	0.01762	0.47775	0.791802	None
13067	NPR1	-0.0461	0.477866	0.791933	None
13068	ZBED9	-0.04891	0.47806	0.792189	None
13069	LINC01241	-0.00492	0.478094	0.792189	None
13070	MRPS14	-0.08864	0.478138	0.79219	None
13071	PPP1R10	0.154166	0.478167	0.79219	None
13072	LOC10192	-0.106	0.478207	0.792195	None
13073	MFSD6	-0.12088	0.478278	0.7922	None
13074	LOC33862	-0.09738	0.478283	0.7922	None
13075	C1QL1	-0.01196	0.478348	0.792233	None
13076	FKTN	-0.12608	0.478376	0.792233	None
13077	HAUS5	-0.10021	0.478476	0.792252	None
13078	LOC40096	-0.00425	0.47857	0.792252	None
13079	CLCA3P	-0.01001	0.478587	0.792252	None
13080	NPTN-IT1	-0.1528	0.478655	0.792252	None
13081	SYT11	0.129504	0.478747	0.792252	None
13082	WNT2B	-0.00728	0.478767	0.792252	None
13083	WNK2	0.015221	0.478774	0.792252	None
13084	FKBP11	-0.20796	0.478832	0.792252	None
13085	RPS25	0.062596	0.478839	0.792252	None
13086	C21orf15	-0.01623	0.478921	0.792252	None
13087	CDR1	-0.00758	0.47893	0.792252	None
13088	SLC6A5	-0.01158	0.478988	0.792252	None
13089	GTF3C5	-0.07778	0.479035	0.792252	None
13090	PPM1K	-0.05868	0.479059	0.792252	None
13091	SEMA3D	-0.01446	0.479073	0.792252	None
13092	LOC10192	0.010893	0.479115	0.792252	None
13093	APOB	-0.02496	0.479127	0.792252	None
13094	PTPRG-AS	-0.04265	0.47914	0.792252	None
13095	BRSK2	-0.004	0.479147	0.792252	None
13096	LOC28608	-0.01383	0.479149	0.792252	None
13097	SOX9	-0.0062	0.479156	0.792252	None
13098	SERP2	-0.00178	0.479284	0.792277	None
13099	LOC10192	-0.00261	0.479382	0.792277	None
13100	SLC35A5	-0.0979	0.479418	0.792277	None
13101	DNAJC15	-0.1355	0.479473	0.792277	None
13102	MAP1B	-0.05623	0.479491	0.792277	None
13103	MS4A6E	-0.01207	0.479555	0.792277	None
13104	CLDN9	-0.00622	0.479563	0.792277	None
13105	LINC00616	0.00577	0.479566	0.792277	None
13106	YPEL4	-0.2189	0.479612	0.792277	None

13107	MAGEB3	-0.00762	0.479618	0.792277	None
13108	MVK	-0.04066	0.479704	0.792277	None
13109	TTC14	0.175526	0.479735	0.792277	None
13110	PCBP3	-0.05179	0.479746	0.792277	None
13111	LDB3	-0.00846	0.479799	0.792277	None
13112	LINC00304	-0.01411	0.479838	0.792277	None
13113	LOC10013:	-0.01161	0.479852	0.792277	None
13114	ZNF429	-0.00763	0.479856	0.792277	None
13115	HCG22	0.025873	0.479949	0.792277	None
13116	DICER1	0.13374	0.479984	0.792277	None
13117	ARHGAP6	-0.17354	0.480004	0.792277	None
13118	RP11-37C7	0.011376	0.480006	0.792277	None
13119	ICAM1	0.232199	0.480021	0.792277	None
13120	PAXBP1	0.105814	0.480043	0.792277	None
13121	LY6D	-0.01736	0.480071	0.792277	None
13122	HDAC3	0.100844	0.480225	0.792277	None
13123	KIF27	-0.07612	0.480232	0.792277	None
13124	CCNT1	-0.10871	0.480237	0.792277	None
13125	PNP	-0.15008	0.480244	0.792277	None
13126	C3orf35	-0.01433	0.480271	0.792277	None
13127	CDH6	-0.01513	0.48035	0.792277	None
13128	STX3	-0.15936	0.480375	0.792277	None
13129	NAV2-IT1	-0.00175	0.480409	0.792277	None
13130	MAT2B	-0.04312	0.480434	0.792277	None
13131	DLGAP1-A	-0.0108	0.480437	0.792277	None
13132	DHFRL1	-0.1083	0.480452	0.792277	None
13133	ANKRD36E	-0.24377	0.480597	0.792288	None
13134	BAALCOS	0.057107	0.480623	0.792288	None
13135	SIK3	0.113085	0.480738	0.792288	None
13136	AC124997.	-0.00955	0.480764	0.792288	None
13137	RP6-91H8.	-0.00397	0.480771	0.792288	None
13138	HLA-F	-0.12081	0.480774	0.792288	None
13139	PYGL	-0.13019	0.480813	0.792288	None
13140	RP11-235E	-0.10437	0.480823	0.792288	None
13141	LOC20060!	0.051363	0.480833	0.792288	None
13142	HYDIN2	-0.00239	0.480878	0.792288	None
13143	KIF25	-0.00969	0.480898	0.792288	None
13144	OIT3	-0.00346	0.480901	0.792288	None
13145	PALMD	-0.03129	0.480934	0.792288	None
13146	LOC10192	0.008846	0.481105	0.792439	None
13147	RPL24	0.053277	0.481159	0.792439	None
13148	KCNA10	-0.00443	0.481248	0.792439	None
13149	CCDC84	0.075153	0.481305	0.792439	None
13150	C1orf234	0.009002	0.48131	0.792439	None
13151	MAT1A	-0.01256	0.481316	0.792439	None
13152	VWA1	0.001879	0.481336	0.792439	None
13153	BSN	-0.00423	0.481336	0.792439	None
13154	TF	0.111881	0.481355	0.792439	None
13155	TMPRSS4	-0.03587	0.481512	0.792542	None
13156	ST8SIA1	0.039487	0.48153	0.792542	None
13157	MRVI1-AS.	-0.01506	0.481561	0.792542	None
13158	MED14	-0.09576	0.481571	0.792542	None
13159	DCUN1D3	0.12674	0.481614	0.792542	None
13160	RP11-21G:	-0.00824	0.481639	0.792542	None
13161	EMILIN3	0.006136	0.481743	0.792542	None
13162	CAPSL	0.101309	0.481792	0.792542	None
13163	ZBED5	0.062507	0.481797	0.792542	None
13164	FAM216B	-0.00314	0.481811	0.792542	None

13165	CDA	-0.11132	0.481885	0.792542	None
13166	NPM2	-0.10371	0.481901	0.792542	None
13167	SMTNL2	-0.00602	0.481904	0.792542	None
13168	CXorf30	-0.00368	0.481969	0.792542	None
13169	WBSCR16	-0.08704	0.481989	0.792542	None
13170	SLC16A7	0.143837	0.482003	0.792542	None
13171	TWF1	-0.01203	0.482326	0.792987	None
13172	RARRES3	0.140981	0.482358	0.792987	None
13173	AMACR	0.012235	0.482388	0.792987	None
13174	PFDN4	0.079107	0.482499	0.792987	None
13175	ENKUR	-0.07922	0.482514	0.792987	None
13176	C11orf83	0.10744	0.482527	0.792987	None
13177	SPDYA	-0.00255	0.48253	0.792987	None
13178	VPS53	-0.07945	0.482633	0.793054	None
13179	UBE2D2	0.100077	0.482644	0.793054	None
13180	CCDC171	-0.09457	0.482737	0.793147	None
13181	LINC00467	-0.11088	0.482839	0.793244	None
13182	COPA	-0.07775	0.48287	0.793244	None
13183	SKA2	-0.08408	0.483032	0.79333	None
13184	FMR1NB	-0.00722	0.483041	0.79333	None
13185	AMOT	-0.019	0.483099	0.79333	None
13186	AKAP6	0.01193	0.4831	0.79333	None
13187	CNTN4	-0.04411	0.483129	0.79333	None
13188	HIRA	-0.05136	0.483142	0.79333	None
13189	ZNF800	0.060501	0.483184	0.793338	None
13190	RSPO2	-0.00269	0.48329	0.793452	None
13191	LOC38845(	-0.00164	0.483346	0.793485	None
13192	HS3ST5	-0.03269	0.483427	0.793508	None
13193	IQCC	0.080089	0.483448	0.793508	None
13194	PRSS35	-0.03278	0.48348	0.793508	None
13195	DSG4	-0.00527	0.483507	0.793508	None
13196	NOXO1	0.011661	0.483572	0.793533	None
13197	SLC22A15	-0.22143	0.483595	0.793533	None
13198	RP11-329E	-0.00619	0.483652	0.793565	None
13199	SYP	-0.04999	0.483706	0.793578	None
13200	LMO4	-0.10399	0.483733	0.793578	None
13201	USE1	-0.11225	0.483785	0.793603	None
13202	PRSS3P2	0.158574	0.483991	0.793823	None
13203	RP4-594L9	0.012631	0.484016	0.793823	None
13204	CLEC7A	-0.1638	0.484029	0.793823	None
13205	AGPAT6	-0.04895	0.484084	0.793853	None
13206	LYZL1	-0.01401	0.48412	0.793853	None
13207	MEIS3	0.004983	0.484163	0.793863	None
13208	LOC10050!	-0.01099	0.484223	0.79389	None
13209	DCHS2	-0.00568	0.484253	0.79389	None
13210	DNAJB6	0.144035	0.484289	0.79389	None
13211	LOC10192	-0.02018	0.484352	0.793933	None
13212	WBSCR22	0.058753	0.48446	0.79405	None
13213	RASSF8	0.012303	0.484635	0.794123	None
13214	FLJ20518	-0.0023	0.48465	0.794123	None
13215	LINC01105	0.000893	0.48466	0.794123	None
13216	TIMM50	-0.10876	0.484714	0.794123	None
13217	MID2	-0.03082	0.484741	0.794123	None
13218	KLC1	-0.09976	0.484743	0.794123	None
13219	AK7	-0.00229	0.484772	0.794123	None
13220	SLC22A2	-0.00409	0.484849	0.794123	None
13221	B3GAT1	0.001903	0.484853	0.794123	None
13222	BC079832	-0.10521	0.484872	0.794123	None

13223	GPR123	-0.00406	0.485028	0.794165	None
13224	ZNF451	0.189514	0.485036	0.794165	None
13225	GPR83	-0.01806	0.485074	0.794165	None
13226	VAC14-AS	-0.01457	0.485154	0.794165	None
13227	LOC10192!	-0.0109	0.485182	0.794165	None
13228	CDKAL1	-0.10976	0.485222	0.794165	None
13229	C7orf55	0.10729	0.485233	0.794165	None
13230	ATRNL1	-0.00762	0.485315	0.794165	None
13231	CYB561A3	-0.11746	0.485422	0.794165	None
13232	CLSTN2	-0.07841	0.485484	0.794165	None
13233	LINC01087	-0.00995	0.48551	0.794165	None
13234	DPYD	-0.07271	0.485604	0.794165	None
13235	VWF	-0.14274	0.485631	0.794165	None
13236	SNORA71E	-0.0209	0.485674	0.794165	None
13237	PCP4	-0.00671	0.485703	0.794165	None
13238	GABRD	-0.00236	0.485824	0.794165	None
13239	VNN3	-0.01704	0.485865	0.794165	None
13240	PPM1F	-0.07414	0.485874	0.794165	None
13241	EIF4G2	-0.05515	0.485875	0.794165	None
13242	THBS2	-0.01742	0.485875	0.794165	None
13243	B3GNT9	-0.03973	0.485964	0.794165	None
13244	SATB2	-0.02411	0.48597	0.794165	None
13245	KLHL11	-0.01132	0.485986	0.794165	None
13246	LOC10028!	-0.01253	0.486074	0.794165	None
13247	HTR3B	-0.00445	0.486087	0.794165	None
13248	PAIP2	0.098935	0.486109	0.794165	None
13249	DHRS1	-0.11771	0.486117	0.794165	None
13250	GABARAPL	-0.08026	0.486155	0.794165	None
13251	SPATA31E:	-0.01014	0.486176	0.794165	None
13252	MARK1	-0.05813	0.48621	0.794165	None
13253	HCP5B	-0.01504	0.486222	0.794165	None
13254	AGPAT5	0.111869	0.486242	0.794165	None
13255	MAPK11	-0.01816	0.486243	0.794165	None
13256	FRG1B	0.06464	0.486252	0.794165	None
13257	SLC7A4	-0.00412	0.486334	0.794165	None
13258	LRRFIP2	-0.1291	0.486374	0.794165	None
13259	CUBN	0.01804	0.486468	0.794165	None
13260	STARD13-,	-0.00418	0.486475	0.794165	None
13261	PRORSD1F	-0.19763	0.48649	0.794165	None
13262	LOC55420(	0.035862	0.486499	0.794165	None
13263	BC045791	-0.00864	0.486523	0.794165	None
13264	HLA-E	-0.0683	0.486594	0.794165	None
13265	LY75	0.207117	0.486635	0.794165	None
13266	FUT8-AS1	0.069917	0.486757	0.794165	None
13267	ZCWPW2	-0.01276	0.48677	0.794165	None
13268	SULT2A1	-0.00317	0.486794	0.794165	None
13269	TRIM10	-0.05041	0.486797	0.794165	None
13270	MYO6	-0.03958	0.486803	0.794165	None
13271	DPT	-0.01152	0.486854	0.794165	None
13272	RP11-108F	0.015946	0.486879	0.794165	None
13273	NYAP1	-0.00612	0.486879	0.794165	None
13274	B4GALT4	-0.06244	0.486882	0.794165	None
13275	ZNF254	0.126123	0.486886	0.794165	None
13276	SLC17A8	-0.03031	0.486922	0.794165	None
13277	C3orf58	-0.00727	0.48693	0.794165	None
13278	LOC10192	-0.00185	0.486951	0.794165	None
13279	NOXA1	-0.04909	0.48706	0.794225	None
13280	CLIC4	-0.11047	0.487077	0.794225	None

13281	LOC10099(	-0.01171	0.487098	0.794225	None
13282	PRMT5-AS	-0.0039	0.487234	0.794254	None
13283	MAPK8IP1	-0.01203	0.487277	0.794254	None
13284	UMOD	-0.00659	0.487288	0.794254	None
13285	KCNE2	-0.01109	0.487323	0.794254	None
13286	LOC10013	-0.0041	0.487328	0.794254	None
13287	PACSIN3	-0.03552	0.487335	0.794254	None
13288	RGMB	0.003587	0.487379	0.794264	None
13289	SLC22A9	-0.00777	0.487482	0.794327	None
13290	KIAA1654	-0.00947	0.487491	0.794327	None
13291	ELTD1	-0.36258	0.487587	0.794369	None
13292	PSMA8	-0.14302	0.487589	0.794369	None
13293	TNFAIP8	0.119034	0.487679	0.794454	None
13294	LILRA6	-0.06433	0.487798	0.794565	None
13295	RASGRP1	0.253549	0.48782	0.794565	None
13296	TGM1	-0.01819	0.48803	0.794785	None
13297	NCAN	-0.00678	0.488066	0.794785	None
13298	OTX1	-0.01216	0.488078	0.794785	None
13299	HTR1B	-0.00837	0.488102	0.794785	None
13300	SLC25A44	-0.05846	0.488152	0.794807	None
13301	SNW1	-0.09085	0.488328	0.795034	None
13302	PRR35	-0.02709	0.488474	0.795038	None
13303	KISS1R	-0.05414	0.488492	0.795038	None
13304	ZBTB32	-0.00393	0.488512	0.795038	None
13305	LOC10192!	-0.01521	0.488566	0.795038	None
13306	RP11-288L	-0.01975	0.488575	0.795038	None
13307	MRPL11	-0.11495	0.488615	0.795038	None
13308	CTXN1	-0.01306	0.488616	0.795038	None
13309	LRRC45	-0.05704	0.488625	0.795038	None
13310	B4GALT1	-0.03126	0.488677	0.795064	None
13311	ACVR2B-A	-0.01336	0.48877	0.795116	None
13312	NTM	-0.00414	0.488788	0.795116	None
13313	GPLD1	0.008366	0.488848	0.795116	None
13314	ALOX15B	-0.03577	0.488875	0.795116	None
13315	MAN1A1	-0.155	0.488893	0.795116	None
13316	LOC10027(	0.171743	0.488961	0.795167	None
13317	LINC01360	0.001331	0.489103	0.795339	None
13318	DUSP16	0.053245	0.489146	0.795348	None
13319	LOC10192	0.075271	0.489349	0.795619	None
13320	TCP11	-0.00724	0.489424	0.795653	None
13321	ABHD11	-0.02837	0.489443	0.795653	None
13322	MAMLD1	-0.04328	0.489515	0.795709	None
13323	ST6GALNA	-0.01583	0.489588	0.795759	None
13324	RND1	-0.0562	0.489619	0.795759	None
13325	INADL	-0.02106	0.489722	0.795867	None
13326	LOC10192!	0.023586	0.489764	0.795876	None
13327	RP11-549J	-0.09521	0.489867	0.795982	None
13328	RPL27A	0.137785	0.489903	0.795982	None
13329	ID4	0.007352	0.490016	0.796096	None
13330	RP11-303E	-0.00786	0.490055	0.796096	None
13331	PRAF2	-0.12434	0.49017	0.796096	None
13332	ITLN1	-0.0945	0.490272	0.796096	None
13333	TPPP2	0.002918	0.490287	0.796096	None
13334	ABRACL	0.094682	0.490305	0.796096	None
13335	LINC00102	0.015038	0.490306	0.796096	None
13336	LOC10192	-0.02466	0.490337	0.796096	None
13337	GMPR2	0.114816	0.490338	0.796096	None
13338	FAM186A	-0.00596	0.490371	0.796096	None

13339	AURKB	0.143087	0.490386	0.796096	None
13340	CHAT	-0.00285	0.490414	0.796096	None
13341	IGFBPL1	-0.00727	0.4905	0.796135	None
13342	DQ586822	-0.00901	0.490533	0.796135	None
13343	FCN2	-0.08938	0.490554	0.796135	None
13344	LOC10192[illegible]	0.010911	0.490585	0.796135	None
13345	SNHG17	-0.12979	0.490702	0.796265	None
13346	GPD1	-0.00916	0.49074	0.796266	None
13347	LOC10050[illegible]	-0.00298	0.490779	0.79627	None
13348	KRTAP4-4	-0.01089	0.490853	0.79633	None
13349	SATB2-AS[illegible]	-0.00702	0.490945	0.79642	None
13350	CSAD	-0.06168	0.491055	0.796486	None
13351	PCDHB16	-0.04184	0.491059	0.796486	None
13352	QRFPR	-0.00327	0.491111	0.79651	None
13353	LOC44145[illegible]	-0.01066	0.491268	0.796705	None
13354	AEBP1	0.168395	0.491384	0.79675	None
13355	S100A7A	0.016596	0.491399	0.79675	None
13356	PDE6C	-0.0027	0.491406	0.79675	None
13357	HGFAC	-0.00537	0.491503	0.796838	None
13358	TRIM78P	-0.01159	0.491539	0.796838	None
13359	LGALS4	0.021108	0.491572	0.796838	None
13360	LINC00408	-0.00492	0.491676	0.796838	None
13361	ZNF583	-0.01533	0.491691	0.796838	None
13362	TNRC18	-0.03305	0.491704	0.796838	None
13363	DMP1	-0.00667	0.491718	0.796838	None
13364	USP25	0.16463	0.491994	0.797073	None
13365	KDM2A	-0.09395	0.492074	0.797073	None
13366	CTD-3080[illegible]	-0.00584	0.492154	0.797073	None
13367	KCNMA1	-0.07851	0.4922	0.797073	None
13368	PLEKHO1	-0.17605	0.492265	0.797073	None
13369	DIRC3	0.020917	0.492293	0.797073	None
13370	GPR20	-0.00853	0.492324	0.797073	None
13371	METTL4	-0.09546	0.492372	0.797073	None
13372	LOC10192[illegible]	-0.00698	0.492406	0.797073	None
13373	LINC01116	-0.02755	0.492456	0.797073	None
13374	TACR3	-0.01373	0.492506	0.797073	None
13375	NFU1	-0.06142	0.492576	0.797073	None
13376	C10orf82	-0.06208	0.492602	0.797073	None
13377	THRB	-0.27762	0.492609	0.797073	None
13378	OR10D1P	-0.00409	0.492612	0.797073	None
13379	IFNE	0.003757	0.492624	0.797073	None
13380	CXCL5	-0.09249	0.492626	0.797073	None
13381	SLC18A2	-0.17544	0.492631	0.797073	None
13382	BRWD3	0.078675	0.492649	0.797073	None
13383	CCDC140	-0.01134	0.492668	0.797073	None
13384	RP11-214[illegible]	0.054227	0.492671	0.797073	None
13385	BAZ1A	0.113945	0.492673	0.797073	None
13386	MCM10	0.15685	0.492734	0.797114	None
13387	CNTNAP1	-0.01418	0.492987	0.797344	None
13388	OR2J2	0.004249	0.493065	0.797344	None
13389	FAM162B	-0.10164	0.493129	0.797344	None
13390	C3orf36	-0.01586	0.493162	0.797344	None
13391	LSMEM1	-0.11328	0.493164	0.797344	None
13392	KIR2DS1	0.009248	0.493173	0.797344	None
13393	EFEMP1	0.023748	0.49318	0.797344	None
13394	HN1L	-0.07621	0.493211	0.797344	None
13395	ZNF75A	-0.08305	0.493248	0.797344	None
13396	DKFZP434[illegible]	0.007648	0.49328	0.797344	None

13397	SRPR	-0.08358	0.493282	0.797344	None
13398	DNAJB13	-0.02321	0.493363	0.797415	None
13399	BDP1	0.138581	0.493718	0.79793	None
13400	CCDC159	-0.12332	0.493784	0.79797	None
13401	ORMDL3	-0.04853	0.493816	0.79797	None
13402	LINC01393	-0.00759	0.493934	0.798101	None
13403	RP11-1008	-0.03089	0.494029	0.798194	None
13404	PRSS54	-0.00503	0.494146	0.798199	None
13405	TEX264	-0.1282	0.494174	0.798199	None
13406	MIA3	-0.09764	0.494245	0.798199	None
13407	LOC10192	-0.00187	0.494245	0.798199	None
13408	TROAP	-0.10149	0.494254	0.798199	None
13409	CIB4	-0.00958	0.494287	0.798199	None
13410	IFT122	0.032824	0.494339	0.798199	None
13411	LOC10192	-0.00557	0.494351	0.798199	None
13412	MLLT4-AS	-0.02833	0.494364	0.798199	None
13413	GSKIP	0.10211	0.494573	0.798457	None
13414	RP11-669	-0.00481	0.494597	0.798457	None
13415	ZNF577	0.059316	0.494678	0.798475	None
13416	LOC10192	-0.00448	0.494769	0.798475	None
13417	HSD17B4	-0.04634	0.494782	0.798475	None
13418	RP11-402	-0.00252	0.494788	0.798475	None
13419	SEC24A	-0.17186	0.494792	0.798475	None
13420	MAT2A	-0.11431	0.495155	0.798903	None
13421	RP11-489	-0.0101	0.495161	0.798903	None
13422	SIAH3	-0.03632	0.495171	0.798903	None
13423	CAPN9	-0.00554	0.495206	0.798903	None
13424	KIAA1257	0.029141	0.495273	0.798925	None
13425	MAP4K4	0.10131	0.495355	0.798925	None
13426	PYGO1	-0.02282	0.495364	0.798925	None
13427	PLCG1	0.049821	0.495366	0.798925	None
13428	CASP16	-0.00249	0.495418	0.798948	None
13429	EXOSC7	-0.08754	0.495552	0.799106	None
13430	TACR2	-0.01328	0.495646	0.799189	None
13431	ATP13A4-	-0.00422	0.495678	0.799189	None
13432	CLDND2	-0.00574	0.495716	0.799191	None
13433	ACACB	0.087854	0.495806	0.799229	None
13434	EPAS1	-0.19393	0.495813	0.799229	None
13435	SCARA5	0.016671	0.495851	0.79923	None
13436	C2orf80	-0.01101	0.495976	0.799372	None
13437	RP11-10K1	0.154433	0.496131	0.799495	None
13438	BCORL1	0.019616	0.496151	0.799495	None
13439	LSM7	-0.15563	0.49617	0.799495	None
13440	DOC2A	-0.02439	0.496205	0.799495	None
13441	LINC00474	-0.01098	0.496237	0.799495	None
13442	BVES	-0.00932	0.496335	0.799593	None
13443	COX5A	-0.08169	0.496382	0.799609	None
13444	SMG7	-0.09994	0.496426	0.799615	None
13445	BMP5	0.055257	0.496478	0.799615	None
13446	RPGRIP1L	0.063565	0.49653	0.799615	None
13447	FER1L6-AS	0.000897	0.496553	0.799615	None
13448	RPL14	0.069859	0.496603	0.799615	None
13449	TRIM29	0.007419	0.496607	0.799615	None
13450	LOC10013	-0.00665	0.496792	0.799686	None
13451	SAGE1	-0.10463	0.496793	0.799686	None
13452	NHP2L1	0.113546	0.496795	0.799686	None
13453	STAC	-0.20679	0.496818	0.799686	None
13454	C15orf65	-0.07399	0.496836	0.799686	None

13455	CDCA2	0.145523	0.496873	0.799687 None
13456	TUBB6	0.24504	0.496934	0.799704 None
13457	NDUFB9	-0.09607	0.496958	0.799704 None
13458	PLAC1	-0.04048	0.497166	0.79994 None
13459	RBM12B-A	-0.01735	0.497224	0.79994 None
13460	LOC10013	-0.00355	0.497232	0.79994 None
13461	BARHL1	0.006601	0.497252	0.79994 None
13462	CTD-2124I	-0.12256	0.497295	0.799949 None
13463	DDX25	-0.10829	0.49734	0.799963 None
13464	FAM221A	0.155696	0.497446	0.800003 None
13465	LOC10192	-0.05467	0.497449	0.800003 None
13466	SORCS1	-0.02814	0.497476	0.800003 None
13467	IBTK	0.099785	0.497602	0.800124 None
13468	RSPH4A	-0.05891	0.497733	0.800124 None
13469	KRT85	-0.00633	0.497758	0.800124 None
13470	SERPINF2	-0.01729	0.497833	0.800124 None
13471	SLC38A4	0.001162	0.497841	0.800124 None
13472	CPEB2	-0.12202	0.497898	0.800124 None
13473	NOL4	-0.00209	0.497909	0.800124 None
13474	HTR5A	-0.02127	0.497916	0.800124 None
13475	ARHGAP27	-0.00294	0.497953	0.800124 None
13476	LOC10050	-0.00297	0.497969	0.800124 None
13477	PDE3A	-0.05554	0.49799	0.800124 None
13478	INHA	-0.00163	0.498086	0.800124 None
13479	LOC10192	-0.01132	0.498089	0.800124 None
13480	ZNF700	-0.11758	0.498096	0.800124 None
13481	SYN3	-0.00822	0.49817	0.800124 None
13482	LOC10272	-0.10364	0.498175	0.800124 None
13483	TOX3	-0.00223	0.498201	0.800124 None
13484	ATP6AP1L	-0.04378	0.498318	0.800124 None
13485	PGR	-0.03087	0.498321	0.800124 None
13486	LOC10192	-0.0066	0.498329	0.800124 None
13487	DAGLA	-0.01042	0.498389	0.800124 None
13488	LINC00964	-0.00479	0.498392	0.800124 None
13489	TSSC1	0.102623	0.498446	0.800124 None
13490	LIPF	-0.01133	0.498469	0.800124 None
13491	LIPG	-0.0214	0.498475	0.800124 None
13492	MALSU1	-0.12968	0.498526	0.800133 None
13493	ITGB1BP1	-0.20364	0.498591	0.800133 None
13494	LINC00692	0.006545	0.498591	0.800133 None
13495	LOC10192	-0.00147	0.498658	0.800135 None
13496	SNAPC5	-0.08631	0.498666	0.800135 None
13497	LINC00284	-0.00383	0.498706	0.80014 None
13498	EDEM2	-0.12451	0.49887	0.800343 None
13499	LANCL2	0.107016	0.498954	0.800418 None
13500	NRG2	-0.00667	0.499052	0.800439 None
13501	THUMPD3	0.161486	0.499079	0.800439 None
13502	LINC00853	-0.08642	0.499219	0.800439 None
13503	MKRN1	-0.09692	0.499225	0.800439 None
13504	LOC10193	0.021534	0.499309	0.800439 None
13505	ACTR3	-0.11722	0.49931	0.800439 None
13506	RNF212B	-0.1009	0.49934	0.800439 None
13507	SYT3	-0.01258	0.499399	0.800439 None
13508	LINC00870	-0.00295	0.499399	0.800439 None
13509	ZNHIT2	-0.00954	0.499428	0.800439 None
13510	LOC28369	0.018516	0.49949	0.800439 None
13511	RP11-110	-0.01131	0.499497	0.800439 None
13512	PRC1	0.154504	0.499534	0.800439 None

13513	SPATA31C	-0.02863	0.499556	0.800439	None
13514	PES1	-0.05338	0.499579	0.800439	None
13515	M1AP	-0.00468	0.499658	0.800439	None
13516	C14orf182	-0.01428	0.499664	0.800439	None
13517	ABL2	-0.12015	0.499725	0.800439	None
13518	LOC10192!	0.058687	0.499732	0.800439	None
13519	LOC10272-	-0.00535	0.499756	0.800439	None
13520	SUV420H2	-0.03048	0.499843	0.800439	None
13521	LHFPL4	-0.03394	0.499859	0.800439	None
13522	CATSPER1	-0.0134	0.499939	0.800439	None
13523	RP11-109E	-0.00761	0.499971	0.800439	None
13524	PRSS53	-0.03036	0.499986	0.800439	None
13525	GTF2H3	0.105734	0.50003	0.800439	None
13526	B3GALT6	-0.11052	0.50003	0.800439	None
13527	LINC00927	0.008186	0.500038	0.800439	None
13528	AGTRAP	-0.15619	0.500038	0.800439	None
13529	GALP	-0.02359	0.500094	0.800469	None
13530	LINC01209	-0.0078	0.500209	0.800543	None
13531	RP11-1072	-0.02546	0.500244	0.800543	None
13532	CDH10	-0.1909	0.500292	0.800543	None
13533	FLJ30064	-0.00658	0.5003	0.800543	None
13534	LOC10192!	-0.00348	0.500326	0.800543	None
13535	OR10J1	-0.00293	0.500527	0.800806	None
13536	ASXL3	-0.02264	0.50066	0.800944	None
13537	AK2	0.098046	0.500721	0.800944	None
13538	RP4-633H:	-0.00384	0.500813	0.800944	None
13539	TMSB4Y	-0.07826	0.500837	0.800944	None
13540	DNMT3L	0.004666	0.500852	0.800944	None
13541	LOC10192ℓ	-0.02967	0.500897	0.800944	None
13542	CPNE6	-0.00937	0.500898	0.800944	None
13543	NKAIN1	-0.00977	0.500938	0.800944	None
13544	FANCA	0.1161	0.500959	0.800944	None
13545	TMEM50A	0.083787	0.501056	0.800944	None
13546	LOC28495(	-0.00412	0.50106	0.800944	None
13547	LOC28457ℓ	-0.01901	0.501119	0.800944	None
13548	LINC00423	0.02556	0.501122	0.800944	None
13549	ECHDC1	-0.08921	0.501159	0.800944	None
13550	TRABD	0.049731	0.501168	0.800944	None
13551	GS1-259H:	-0.05451	0.501312	0.801115	None
13552	LOC10192!	0.014895	0.501471	0.801247	None
13553	CYSRT1	0.010084	0.501489	0.801247	None
13554	RASA1	-0.10974	0.501561	0.801247	None
13555	RP11-17A-	-0.00933	0.501562	0.801247	None
13556	PLCE1	0.007527	0.501636	0.801247	None
13557	NTN3	0.021632	0.50164	0.801247	None
13558	FAM101B	-0.11888	0.501664	0.801247	None
13559	PRO1596	-0.03211	0.501693	0.801247	None
13560	FLJ40288	-0.00751	0.501727	0.801247	None
13561	SPPL2B	0.078759	0.501767	0.801249	None
13562	MBP	-0.03182	0.501805	0.801249	None
13563	C12orf61	-0.01969	0.501856	0.801249	None
13564	SERPINB1	0.098212	0.501905	0.801249	None
13565	RIMKLA	-0.01761	0.501914	0.801249	None
13566	BC045779	-0.01735	0.501955	0.801256	None
13567	HIST1H2AI	-0.08702	0.502153	0.801392	None
13568	AADAC	0.013998	0.502192	0.801392	None
13569	CASR	-0.00364	0.502196	0.801392	None
13570	ETV2	0.038512	0.50227	0.801392	None

13571	CORO2B	0.097232	0.502277	0.801392	None
13572	TRAC	0.065758	0.502301	0.801392	None
13573	TAS2R19	-0.0123	0.502325	0.801392	None
13574	UPK1B	-0.00895	0.502357	0.801392	None
13575	GNAI2	-0.12642	0.502373	0.801392	None
13576	GALR1	-0.01705	0.502451	0.801444	None
13577	PRO2949	-0.0028	0.502508	0.801444	None
13578	ATF2	-0.10439	0.502517	0.801444	None
13579	PRR26	0.001933	0.502646	0.801573	None
13580	ZNF765	-0.12289	0.502672	0.801573	None
13581	DYNC1H1	-0.03771	0.502742	0.801626	None
13582	CYYR1	-0.22935	0.502828	0.801664	None
13583	LOC40105	-0.02357	0.502848	0.801664	None
13584	THOC7	-0.09541	0.502887	0.801664	None
13585	LOC10012	0.024108	0.502914	0.801664	None
13586	HMCN2	-0.00742	0.502956	0.801672	None
13587	CDH3	-0.01078	0.50307	0.801794	None
13588	UBE2L6	-0.10144	0.503155	0.801871	None
13589	NRG4	-0.07135	0.503217	0.80191	None
13590	RALGAPB	-0.08035	0.50326	0.801921	None
13591	SH3GL3	-0.03906	0.503389	0.802047	None
13592	ODC1	0.128508	0.503413	0.802047	None
13593	PPP1R3E	-0.14418	0.503523	0.802162	None
13594	EPHX1	-0.0778	0.503561	0.802164	None
13595	BAI3	-0.07249	0.503654	0.802252	None
13596	LIPE	0.023793	0.503751	0.802348	None
13597	SALL1	-0.00217	0.503872	0.802427	None
13598	PXDNL	-0.00746	0.503875	0.802427	None
13599	ANKRD23	-0.10294	0.503947	0.802484	None
13600	MCAT	0.113451	0.50409	0.802583	None
13601	TCP11L2	-0.02222	0.504094	0.802583	None
13602	ZBTB47	-0.03951	0.504133	0.802583	None
13603	ANO1	0.01529	0.504196	0.802583	None
13604	FCGRT	-0.24115	0.504203	0.802583	None
13605	CHD2	0.093688	0.504307	0.802583	None
13606	AMD1	-0.05761	0.504357	0.802583	None
13607	USP49	-0.02526	0.504364	0.802583	None
13608	SART1	-0.09324	0.504375	0.802583	None
13609	JARID2-AS	-0.01384	0.50438	0.802583	None
13610	LOC64526	-0.00587	0.504497	0.80271	None
13611	CFHR2	-0.05654	0.504706	0.802906	None
13612	CD207	-0.00209	0.50474	0.802906	None
13613	ANO7	-0.00907	0.504745	0.802906	None
13614	BC048132	-0.03527	0.504775	0.802906	None
13615	LOC10012	-0.00257	0.504806	0.802906	None
13616	LOC10192	-0.00368	0.504919	0.803028	None
13617	ZNF483	0.007046	0.504969	0.803048	None
13618	RP11-255C	0.075472	0.505018	0.803067	None
13619	ZNF644	0.09445	0.505176	0.803238	None
13620	ASZ1	-0.00835	0.505209	0.803238	None
13621	SGIP1	-0.12961	0.505267	0.803238	None
13622	SLC30A4	0.024978	0.50531	0.803238	None
13623	CRYZL1	-0.13267	0.505325	0.803238	None
13624	TRIOBP	0.069357	0.505379	0.803238	None
13625	SGCG	-0.119	0.505431	0.803238	None
13626	ROPN1L	-0.019	0.505432	0.803238	None
13627	ADCY4	-0.09514	0.50546	0.803238	None
13628	PM20D1	-0.01889	0.505596	0.803396	None

13629	IL20	-0.00861	0.505753	0.803474	None
13630	HP08942	-0.01515	0.505754	0.803474	None
13631	MIRLET7DI	-0.10757	0.505756	0.803474	None
13632	SMNDC1	0.084734	0.505858	0.803576	None
13633	AS3MT	0.06924	0.505923	0.803577	None
13634	AK055981	0.138639	0.505933	0.803577	None
13635	DIRC2	0.078151	0.506011	0.803601	None
13636	UNC79	-0.00406	0.506022	0.803601	None
13637	ZNF681	0.044527	0.506124	0.803646	None
13638	TBC1D16	-0.09107	0.506124	0.803646	None
13639	RP11-45M	-0.00297	0.506182	0.803679	None
13640	LRP4	-0.11522	0.506265	0.803751	None
13641	OR2A4	-0.00762	0.506423	0.803943	None
13642	RHO	0.021539	0.506527	0.804049	None
13643	C3P1	-0.00405	0.506589	0.804054	None
13644	SCLY	-0.0483	0.506604	0.804054	None
13645	C1orf168	-0.00502	0.506724	0.804108	None
13646	LOC10050!	-0.1096	0.506735	0.804108	None
13647	SPATA3-A	-0.00403	0.506751	0.804108	None
13648	WDR18	-0.11733	0.506787	0.804108	None
13649	AK056982	-0.01053	0.506855	0.804157	None
13650	SEPT5-GP1	-0.04359	0.506997	0.804269	None
13651	TMEM158	-0.21974	0.507045	0.804269	None
13652	NR5A2	-0.09041	0.507057	0.804269	None
13653	MRPL22	0.134584	0.507093	0.804269	None
13654	RP5-1098D	0.000972	0.507187	0.804269	None
13655	LPIN3	-0.03237	0.507187	0.804269	None
13656	SHISA7	0.023081	0.507239	0.804269	None
13657	PRKG1	-0.04364	0.507252	0.804269	None
13658	C17orf78	-0.00339	0.507287	0.804269	None
13659	AKR1C3	-0.1129	0.507297	0.804269	None
13660	TMEM102	-0.02402	0.507394	0.804364	None
13661	HTATSF1P2	-0.0711	0.507516	0.804406	None
13662	INSL3	-0.01089	0.507539	0.804406	None
13663	ALDH2	-0.12488	0.507608	0.804406	None
13664	LOC10192!	-0.00677	0.507608	0.804406	None
13665	ATAD1	0.101081	0.50763	0.804406	None
13666	SGCZ	-0.01108	0.507643	0.804406	None
13667	HABP2	-0.00884	0.507748	0.804497	None
13668	LOC72835:	0.010201	0.507804	0.804497	None
13669	SLC6A9	0.003363	0.507812	0.804497	None
13670	FAM198B	0.128949	0.508005	0.804659	None
13671	CHAC2	-0.17868	0.50801	0.804659	None
13672	NHLH1	-0.02068	0.508026	0.804659	None
13673	APCDD1	-0.01921	0.508137	0.804776	None
13674	LRIG1	-0.00227	0.50821	0.804834	None
13675	ARMCX4	-0.01963	0.508287	0.804886	None
13676	RIN2	-0.11677	0.508318	0.804886	None
13677	SLC6A1	-0.02736	0.508377	0.804921	None
13678	CXCR5	0.017804	0.508562	0.805155	None
13679	ULK4	0.04748	0.508745	0.805347	None
13680	CTXN3	-0.00139	0.508757	0.805347	None
13681	C9orf84	-0.00215	0.508923	0.805538	None
13682	POM121L9	-0.01446	0.50896	0.805538	None
13683	LOC10192!	-0.00686	0.50899	0.805538	None
13684	ZRANB3	0.048768	0.509027	0.805539	None
13685	PHLPP2	0.050488	0.509122	0.805603	None
13686	MRPL44	-0.07404	0.509143	0.805603	None

13687	RP11-998D	-0.0148	0.50918	0.805603	None
13688	RP11-517C	-0.01757	0.509337	0.805793	None
13689	SMR3B	0.015894	0.509454	0.805918	None
13690	RP11-50E1	-0.10891	0.509505	0.805941	None
13691	ZNF614	-0.05968	0.509599	0.806031	None
13692	RS1	-0.00637	0.509707	0.806063	None
13693	GP6	-0.14922	0.509707	0.806063	None
13694	FAM106A	0.040106	0.509795	0.806063	None
13695	IMPDH2	0.087207	0.509813	0.806063	None
13696	RP4-612B1	-0.08244	0.509833	0.806063	None
13697	AC017002.	-0.19483	0.509939	0.806063	None
13698	PRDM8	-0.01728	0.50997	0.806063	None
13699	LOC28369:	0.015633	0.510035	0.806063	None
13700	ANPEP	-0.00145	0.510047	0.806063	None
13701	11-Sep	-0.14659	0.51005	0.806063	None
13702	SIPA1L1	0.123634	0.51011	0.806063	None
13703	PDSS1	-0.08132	0.510211	0.806063	None
13704	LOC10192!	0.003193	0.510243	0.806063	None
13705	FAM46B	-0.00498	0.510256	0.806063	None
13706	RP11-258C	-0.14646	0.510265	0.806063	None
13707	PDIA2	-0.00375	0.510274	0.806063	None
13708	RELL2	-0.0345	0.510274	0.806063	None
13709	PDE1C	-0.00787	0.51029	0.806063	None
13710	TXNDC11	0.093962	0.510356	0.80611	None
13711	C4BPA	-0.08453	0.510409	0.806134	None
13712	SLN	-0.00288	0.510512	0.806166	None
13713	GPR137C	-0.04306	0.510541	0.806166	None
13714	C17orf100	-0.0914	0.510558	0.806166	None
13715	FRMD7	-0.02166	0.510615	0.806166	None
13716	YIF1A	-0.10153	0.510615	0.806166	None
13717	LA16c-380	0.077453	0.510711	0.806259	None
13718	ATP6V0B	0.108358	0.510769	0.806291	None
13719	ARSJ	-0.00346	0.510938	0.8065	None
13720	BC045560	-0.05105	0.511001	0.8065	None
13721	ZFYVE20	-0.05199	0.511042	0.8065	None
13722	CADM4	-0.00716	0.51106	0.8065	None
13723	VN1R1	-0.0246	0.511148	0.8065	None
13724	RP11-456F	-0.1006	0.511149	0.8065	None
13725	RBFA	0.082287	0.511162	0.8065	None
13726	SFTPD	-0.0313	0.511327	0.806702	None
13727	LOC10192	0.006989	0.511416	0.806751	None
13728	ASPHD1	-0.01706	0.511456	0.806751	None
13729	CTA-384D	-0.01319	0.51147	0.806751	None
13730	LOC33980	-0.00868	0.51156	0.806835	None
13731	ANKRD9	-0.11523	0.511624	0.806876	None
13732	TMEM132/	0.016379	0.511669	0.806888	None
13733	LOC28437:	-0.06642	0.511829	0.807082	None
13734	FAM118B	-0.11833	0.511871	0.80709	None
13735	TRPM4	-0.01534	0.511929	0.807122	None
13736	MTCH1	-0.06937	0.511982	0.807129	None
13737	FLJ12120	-0.06267	0.512008	0.807129	None
13738	ECI2	-0.0967	0.512052	0.80714	None
13739	GOLGA6L6	-0.00674	0.512131	0.807204	None
13740	MBTPS2	0.017738	0.512167	0.807204	None
13741	C19orf53	-0.09854	0.512336	0.807412	None
13742	LOC10272	0.020166	0.512435	0.807458	None
13743	GS1-164F2	0.045555	0.51244	0.807458	None
13744	COPS3	-0.07584	0.512537	0.807552	None

13745	TMC7	-0.01404	0.51258	0.80756	None
13746	ZNF362	-0.04636	0.512638	0.807565	None
13747	AC114752.	-0.06796	0.512657	0.807565	None
13748	MCM8	-0.09454	0.512805	0.807674	None
13749	SLC3A1	-0.02099	0.512838	0.807674	None
13750	LOC64652	-0.00368	0.512838	0.807674	None
13751	LOC28638	0.012003	0.512884	0.807686	None
13752	GRB7	-0.00953	0.512925	0.807694	None
13753	LOC10050	-0.00678	0.513105	0.807896	None
13754	LOC10192	-0.00283	0.513155	0.807896	None
13755	INGX	-0.02294	0.51317	0.807896	None
13756	RP11-315F	0.018245	0.513239	0.807896	None
13757	RD3	-0.00244	0.51324	0.807896	None
13758	LINC00462	-0.00241	0.51342	0.808031	None
13759	LOC10192	-0.00634	0.513453	0.808031	None
13760	EMP1	0.141925	0.513507	0.808031	None
13761	KCMF1	-0.07634	0.513512	0.808031	None
13762	EDN2	-0.00561	0.513513	0.808031	None
13763	FAM57A	0.157792	0.513791	0.80826	None
13764	AFF4	0.073314	0.513798	0.80826	None
13765	POU5F1P3	-0.01035	0.513823	0.80826	None
13766	MTHFD2L	-0.08664	0.513844	0.80826	None
13767	ALPL	0.028322	0.513877	0.80826	None
13768	DESI2	0.063798	0.513883	0.80826	None
13769	TENM4	-0.04401	0.514005	0.808395	None
13770	TMEM61	-0.00982	0.514049	0.808401	None
13771	PTPRT	-0.00991	0.514099	0.808401	None
13772	TAS2R14	-0.05385	0.514121	0.808401	None
13773	GRAMD2	-0.01471	0.514208	0.808478	None
13774	ADRA1D	-0.00701	0.514247	0.808481	None
13775	DDX60L	-0.09357	0.514361	0.808601	None
13776	HMGN2P4	0.013624	0.514491	0.808666	None
13777	RWDD2A	-0.04868	0.514495	0.808666	None
13778	COL1A1	0.051652	0.514516	0.808666	None
13779	C2orf72	-0.00257	0.514555	0.808666	None
13780	POTEM	-0.00312	0.514589	0.808666	None
13781	RP11-50B3	0.135869	0.514663	0.808724	None
13782	AC092620.	-0.14071	0.51473	0.808756	None
13783	RP6-201G	-0.05231	0.514822	0.808756	None
13784	AIMP2	-0.09183	0.514849	0.808756	None
13785	TSPEAR	0.007273	0.514859	0.808756	None
13786	LOC28464	-0.00506	0.51487	0.808756	None
13787	THEG5	-0.00918	0.514929	0.80879	None
13788	TBX2	0.00728	0.51502	0.808843	None
13789	RP1-86D1.	-0.0371	0.51507	0.808843	None
13790	CALCA	-0.00178	0.515127	0.808843	None
13791	SLC27A2	-0.1637	0.515143	0.808843	None
13792	KRTAP4-3	-0.00265	0.515149	0.808843	None
13793	RNF128	-0.00613	0.51523	0.808895	None
13794	BC070490	-0.0027	0.515286	0.808895	None
13795	PGC	-0.00373	0.515315	0.808895	None
13796	ANAPC10	-0.07887	0.515332	0.808895	None
13797	EIF2S3	-0.07158	0.515441	0.808994	None
13798	OSBP	-0.10958	0.515474	0.808994	None
13799	DPY19L1P	-0.04827	0.515507	0.808994	None
13800	RBM24	-0.00233	0.515552	0.809006	None
13801	SARDH	-0.0085	0.515646	0.809095	None
13802	PROX1-AS	-0.00638	0.515731	0.809136	None

13803	BRI3BP	-0.10582	0.515747	0.809136	None
13804	FAM178B	-0.06611	0.515835	0.809214	None
13805	BMPR1A	-0.03285	0.515939	0.80926	None
13806	CTD-2165I	-0.06731	0.51597	0.80926	None
13807	CASC18	0.014674	0.515975	0.80926	None
13808	C1orf21	-0.11409	0.516028	0.809267	None
13809	ELAVL1	-0.07822	0.516055	0.809267	None
13810	ZNF782	-0.08687	0.516197	0.809431	None
13811	ROR2	-0.01715	0.516351	0.80956	None
13812	LOC93444	0.023139	0.516371	0.80956	None
13813	DACT2	-0.00809	0.516391	0.80956	None
13814	GRK1	-0.01534	0.516457	0.809603	None
13815	ARHGEF2	-0.10327	0.516521	0.809603	None
13816	OR12D2	-0.00812	0.516531	0.809603	None
13817	ZNF132	-0.01687	0.516693	0.809799	None
13818	LOC44105:	-0.05429	0.516775	0.809813	None
13819	EFHC2	0.099668	0.516777	0.809813	None
13820	ZNF540	-0.03078	0.516831	0.809839	None
13821	KRBOX1-A	-0.01747	0.516905	0.809896	None
13822	RASGRP2	-0.08279	0.516975	0.809947	None
13823	ZNF138	0.069084	0.517117	0.810079	None
13824	MAGI2-AS	-0.14195	0.517141	0.810079	None
13825	H2BFM	-0.02236	0.517171	0.810079	None
13826	SLC25A6	0.082282	0.517361	0.810318	None
13827	LOC10028	-0.00226	0.51746	0.810354	None
13828	ZNF213-A:	0.059634	0.517475	0.810354	None
13829	C5orf66-A	-0.00269	0.5175	0.810354	None
13830	TACC2	-0.04258	0.517534	0.810354	None
13831	SHH	-0.00778	0.51764	0.810428	None
13832	ZNF345	-0.06942	0.517656	0.810428	None
13833	C3orf43	-0.01091	0.517757	0.810469	None
13834	LOC28617	-0.01377	0.517757	0.810469	None
13835	RABEP1	-0.07037	0.517933	0.810639	None
13836	TMEM207	-0.01162	0.51794	0.810639	None
13837	CCDC138	-0.08182	0.518046	0.810745	None
13838	CCNL1	-0.1721	0.518165	0.810814	None
13839	GNAI3	-0.09516	0.518206	0.810814	None
13840	CAMKK1	0.045518	0.518239	0.810814	None
13841	KRT73	-0.00249	0.51824	0.810814	None
13842	JAKMIP1	-0.00729	0.518281	0.810821	None
13843	CTD-2196I	-0.07148	0.518335	0.810847	None
13844	C11orf57	-0.07569	0.518465	0.81086	None
13845	RP11-425I	-0.127	0.518485	0.81086	None
13846	RP11-399E	-0.0056	0.518504	0.81086	None
13847	RAB11FIP2	0.102904	0.518513	0.81086	None
13848	C7orf69	-0.01436	0.518565	0.81086	None
13849	STXBP5	-0.14453	0.518582	0.81086	None
13850	HMG20B	-0.12173	0.518607	0.81086	None
13851	LOC10192	-0.00518	0.518659	0.81086	None
13852	RP11-634E	0.008737	0.51868	0.81086	None
13853	NOP9	-0.06058	0.51878	0.810957	None
13854	SLC31A2	0.171408	0.518843	0.810981	None
13855	CRISPLD2	0.084322	0.518871	0.810981	None
13856	SIDT1	0.172762	0.518939	0.81103	None
13857	SERPINB11	-0.00508	0.519029	0.811111	None
13858	RP11-448A	-0.02705	0.51911	0.81112	None
13859	C15orf49	-0.00646	0.51913	0.81112	None
13860	RP11-540C	-0.03338	0.519147	0.81112	None

13861	LOC101928	-0.00641	0.519197	0.81114	None
13862	SLC7A1	-0.04138	0.519373	0.81126	None
13863	C9orf172	-0.02063	0.519442	0.81126	None
13864	RP11-131L	-0.00618	0.519491	0.81126	None
13865	ZNF425	-0.00246	0.519524	0.81126	None
13866	LINC00968	-0.04518	0.519527	0.81126	None
13867	LPHN3	-0.09958	0.519544	0.81126	None
13868	CTD-2194I	-0.00345	0.519565	0.81126	None
13869	WEE1	-0.14563	0.519573	0.81126	None
13870	LINC00528	-0.13672	0.519735	0.811454	None
13871	CYP17A1	-0.00584	0.519803	0.811501	None
13872	LOC152578	-0.00264	0.519979	0.81165	None
13873	BC044596	0.21498	0.520007	0.81165	None
13874	CDH18	-0.00628	0.520058	0.81165	None
13875	CES1P1	-0.02489	0.520078	0.81165	None
13876	ZAN	-0.01752	0.520085	0.81165	None
13877	TMEM171	-0.00639	0.520135	0.811668	None
13878	HERC2P7	-0.00801	0.520178	0.811677	None
13879	MAGI2-IT1	-0.00709	0.520311	0.811775	None
13880	PBXIP1	0.153338	0.520352	0.811775	None
13881	RPUSD4	-0.1196	0.520373	0.811775	None
13882	SSTR5-AS1	-0.00526	0.520417	0.811775	None
13883	FAM181A-	-0.00251	0.520434	0.811775	None
13884	RNF181	-0.11281	0.520465	0.811775	None
13885	MLST8	-0.1181	0.520566	0.811839	None
13886	PJA1	0.093277	0.520592	0.811839	None
13887	PARD3-AS	-0.01025	0.520619	0.811839	None
13888	LINC01004	-0.02014	0.520935	0.812253	None
13889	FAM8A1	-0.09934	0.52096	0.812253	None
13890	FUT4	0.108358	0.521021	0.812291	None
13891	C1orf105	-0.00476	0.521206	0.812447	None
13892	POM121L2	0.007552	0.521265	0.812447	None
13893	CEP192	0.106365	0.52129	0.812447	None
13894	RBPMS-AS	-0.04477	0.521337	0.812447	None
13895	UNC93A	-0.00586	0.521367	0.812447	None
13896	NDUFS1	0.118972	0.521386	0.812447	None
13897	HIPK1	-0.10879	0.521387	0.812447	None
13898	TAGLN	-0.02614	0.521422	0.812447	None
13899	OPN3	-0.13047	0.521524	0.812509	None
13900	SCN7A	-0.0237	0.521537	0.812509	None
13901	ZC3H6	0.077483	0.521592	0.812536	None
13902	PCDH19	-0.00713	0.521711	0.812664	None
13903	GATS	0.040998	0.52184	0.812703	None
13904	VSNL1	0.004559	0.521962	0.812703	None
13905	MTHFSD	-0.05467	0.521997	0.812703	None
13906	RP11-753A	-0.0148	0.522004	0.812703	None
13907	RP11-266A	-0.03363	0.522041	0.812703	None
13908	YAF2	-0.06257	0.522074	0.812703	None
13909	PBDC1	-0.09371	0.522085	0.812703	None
13910	RPA3	0.142813	0.522111	0.812703	None
13911	C8orf31	-0.00738	0.522129	0.812703	None
13912	CCER1	-0.00905	0.522136	0.812703	None
13913	GNB5	-0.10418	0.522162	0.812703	None
13914	RP11-109D	-0.00532	0.522238	0.812703	None
13915	C5orf30	-0.11119	0.522267	0.812703	None
13916	PHKG1	0.010863	0.522298	0.812703	None
13917	PVRL3-AS1	-0.0072	0.522299	0.812703	None
13918	LRFN5	-0.00243	0.522384	0.812718	None

13919	SCN8A	-0.00508	0.522438	0.812718	None
13920	NETO1	-0.00147	0.522453	0.812718	None
13921	CD93	-0.16809	0.522459	0.812718	None
13922	INTS10	-0.1173	0.522532	0.812773	None
13923	FLJ26850	-0.01254	0.522636	0.812844	None
13924	IVL	0.018939	0.522652	0.812844	None
13925	ZNF585A	-0.09005	0.522716	0.812884	None
13926	RP11-806L	-0.00699	0.522784	0.812932	None
13927	SYNGR4	-0.00511	0.522947	0.812969	None
13928	PRSS2	0.279147	0.522947	0.812969	None
13929	LOC10050	-0.04448	0.522951	0.812969	None
13930	LLNLR-246	-0.01465	0.522969	0.812969	None
13931	FAT3	-0.03288	0.523029	0.812969	None
13932	LOC101928	-0.00556	0.523033	0.812969	None
13933	LOC10192	-0.00891	0.523194	0.813155	None
13934	FLJ37201	-0.00944	0.523241	0.813155	None
13935	RP11-218C	0.005044	0.523279	0.813155	None
13936	LOC100288	0.017308	0.523303	0.813155	None
13937	ENTHD1	-0.00794	0.523371	0.813202	None
13938	RPS21	0.087414	0.523548	0.813391	None
13939	CYP46A1	-0.0283	0.523567	0.813391	None
13940	NEUROD6	-0.0024	0.523641	0.813446	None
13941	FLVCR2	-0.06434	0.523905	0.81378	None
13942	CAMLG	-0.10418	0.523931	0.81378	None
13943	LINC00240	-0.02327	0.523971	0.813784	None
13944	SPIN2A	-0.01263	0.524028	0.813814	None
13945	TOX2	-0.06451	0.524289	0.814162	None
13946	NRG1-IT1	-0.00588	0.524365	0.814221	None
13947	NEK7	0.11606	0.524497	0.814348	None
13948	HIST1H3C	-0.05725	0.524531	0.814348	None
13949	OMD	-0.02152	0.524589	0.814348	None
13950	CCNB3	-0.02801	0.524616	0.814348	None
13951	LINC01282	-0.00187	0.524635	0.814348	None
13952	HLA-J	-0.10954	0.524753	0.814383	None
13953	TMEM179	-0.01051	0.524772	0.814383	None
13954	SEPSECS	0.100869	0.524829	0.814383	None
13955	IMP3	-0.11484	0.524875	0.814383	None
13956	MINOS1	-0.05578	0.524878	0.814383	None
13957	GATA3	-0.11484	0.524942	0.814383	None
13958	NANOS2	-0.01047	0.524957	0.814383	None
13959	C5orf27	-0.01526	0.524979	0.814383	None
13960	PPP3CB-AS	-0.04391	0.525029	0.814383	None
13961	GPR144	-0.07004	0.525066	0.814383	None
13962	HBS1L	0.126278	0.525131	0.814383	None
13963	NTN5	-0.01049	0.525136	0.814383	None
13964	PNLIPRP1	-0.00244	0.525157	0.814383	None
13965	DDX20	0.070204	0.525184	0.814383	None
13966	PHOX2B	-0.00288	0.525276	0.814416	None
13967	CFH	-0.25403	0.525283	0.814416	None
13968	THBS4	0.135712	0.525318	0.814416	None
13969	SLC9A2	-0.01076	0.525639	0.814772	None
13970	MORC2-AS	-0.13461	0.525662	0.814772	None
13971	SNAP25	0.008739	0.525735	0.814772	None
13972	CNTNAP2	-0.02094	0.525738	0.814772	None
13973	SLFNL1-AS	0.008568	0.525767	0.814772	None
13974	PDLIM1	-0.18383	0.525826	0.814772	None
13975	LOC10192	-0.00245	0.525827	0.814772	None
13976	CCDC13-A	-0.00721	0.525848	0.814772	None

13977	PRSS16	-0.0106	0.525895	0.814773	None
13978	SLC6A18	-0.01617	0.525925	0.814773	None
13979	MAGI2-AS	0.001349	0.52605	0.814794	None
13980	COX10-AS	0.084264	0.526078	0.814794	None
13981	HEATR4	0.023816	0.526129	0.814794	None
13982	SVOP	-0.0115	0.526188	0.814794	None
13983	SPATA2L	0.113995	0.526196	0.814794	None
13984	FBXW2	0.0839	0.526209	0.814794	None
13985	ALOX12	-0.2431	0.526213	0.814794	None
13986	IGFALS	-0.05461	0.526239	0.814794	None
13987	LOC10192(	-0.0892	0.526388	0.814966	None
13988	WWC2-AS	-0.00967	0.526477	0.815012	None
13989	C15orf45	-0.00535	0.526496	0.815012	None
13990	SLCO4A1	-0.07361	0.52653	0.815012	None
13991	PPP1R3A	0.002746	0.526718	0.81511	None
13992	LOC10192ᵢ	-0.00414	0.526726	0.81511	None
13993	LRRTM1	-0.00289	0.526731	0.81511	None
13994	LOC43995	-0.00415	0.52679	0.81511	None
13995	PCDH15	-0.00279	0.526822	0.81511	None
13996	LOC10192	0.006053	0.526854	0.81511	None
13997	ACOT12	-0.01589	0.526921	0.81511	None
13998	LOC80154	-0.05001	0.526992	0.81511	None
13999	FCRL3	-0.07403	0.527038	0.81511	None
14000	EPB41L1	-0.03178	0.527056	0.81511	None
14001	MSTN	-0.00872	0.527058	0.81511	None
14002	REG4	0.003997	0.527081	0.81511	None
14003	DMPK	-0.01294	0.527171	0.81511	None
14004	LOC10192	-0.00132	0.52718	0.81511	None
14005	IMPACT	0.085248	0.527247	0.81511	None
14006	RP11-1152	-0.00379	0.527335	0.81511	None
14007	WDR31	-0.01391	0.527341	0.81511	None
14008	AKAP4	-0.0121	0.52735	0.81511	None
14009	IL10RB	-0.09705	0.527366	0.81511	None
14010	LIFR	-0.07585	0.527386	0.81511	None
14011	SFRP2	-0.00207	0.527419	0.81511	None
14012	NOTCH4	0.046306	0.52745	0.81511	None
14013	CWH43	-0.02067	0.527461	0.81511	None
14014	ALG10	0.072851	0.527549	0.81511	None
14015	FGFBP3	-0.06294	0.527594	0.81511	None
14016	PLEKHF1	-0.04856	0.527679	0.81511	None
14017	CSPG4P1Y	-0.01678	0.527688	0.81511	None
14018	NEFM	-0.00234	0.527692	0.81511	None
14019	RP11-465I	-0.00741	0.527717	0.81511	None
14020	ZMYND15	-0.033	0.527725	0.81511	None
14021	LOC10029	-0.05048	0.527761	0.81511	None
14022	FIGNL1	-0.11555	0.527857	0.8152	None
14023	OSER1	0.091405	0.52793	0.815255	None
14024	MYRFL	-0.04557	0.528026	0.815346	None
14025	UNC80	-0.00203	0.528119	0.81538	None
14026	KCNG1	-0.01224	0.528124	0.81538	None
14027	LOC10272	-0.00428	0.528182	0.815411	None
14028	RRM1	0.098702	0.528325	0.815514	None
14029	ENHO	0.028756	0.528355	0.815514	None
14030	ADCY3	-0.0963	0.528439	0.815514	None
14031	MED15P9	-0.01394	0.528444	0.815514	None
14032	TIMM8A	-0.05201	0.528517	0.815514	None
14033	ZFAND6	0.076781	0.528524	0.815514	None
14034	LINC01272	-0.02514	0.528529	0.815514	None

14035	SPTLC3	0.087455	0.52855	0.815514	None
14036	C15orf52	-0.13983	0.528627	0.815523	None
14037	S1PR3	0.075492	0.528631	0.815523	None
14038	HGC6.3	0.054816	0.528682	0.815545	None
14039	RP11-445H	-0.01284	0.528747	0.815587	None
14040	CARD11	-0.05247	0.528824	0.815647	None
14041	NBR2	-0.08849	0.52895	0.815783	None
14042	KRT38	-0.01068	0.529036	0.815838	None
14043	LOC10192	-0.00342	0.529061	0.815838	None
14044	TRIB3	-0.0788	0.529103	0.815845	None
14045	LIPE-AS1	-0.02486	0.529256	0.816023	None
14046	FBXO16	-0.06491	0.529363	0.81613	None
14047	AC003989.	-0.00735	0.529511	0.816299	None
14048	CRHR2	-0.00609	0.529645	0.816448	None
14049	ALS2CR11	-0.00231	0.529722	0.81646	None
14050	LOC10192	-0.00649	0.529745	0.81646	None
14051	SERP1	0.076472	0.529797	0.81646	None
14052	PRKAR1B	-0.02643	0.529803	0.81646	None
14053	AK3	0.088016	0.529844	0.816464	None
14054	CENPI	0.038881	0.530043	0.816713	None
14055	LINC00314	-0.01615	0.530103	0.816747	None
14056	TRPC5	-0.00529	0.530177	0.816753	None
14057	LOC15727	-0.00319	0.530182	0.816753	None
14058	PPTC7	0.095789	0.530259	0.816813	None
14059	RP11-524D	-0.00136	0.530362	0.816913	None
14060	OPN5	0.01375	0.530577	0.817136	None
14061	CETN3	-0.13218	0.530616	0.817136	None
14062	RP1-155D2	-0.01128	0.53062	0.817136	None
14063	GPR62	-0.0053	0.530658	0.817137	None
14064	CD40	-0.03226	0.530735	0.817197	None
14065	CLMP	-0.0272	0.530808	0.817202	None
14066	LINC00852	0.040115	0.530824	0.817202	None
14067	AK090844	0.063884	0.530851	0.817202	None
14068	LOC10012	-0.07844	0.530934	0.817272	None
14069	DHRS7C	0.013885	0.531008	0.817278	None
14070	LINC01138	-0.02148	0.531014	0.817278	None
14071	FIRRE	-0.11958	0.531084	0.817328	None
14072	ERP29	0.094732	0.531125	0.817333	None
14073	EHMT1	-0.143	0.531214	0.817386	None
14074	RP11-108L	-0.07971	0.531276	0.817386	None
14075	SPRR2G	-0.00805	0.531279	0.817386	None
14076	CENPV	-0.19391	0.531339	0.817386	None
14077	MIOX	-0.00636	0.531377	0.817386	None
14078	DUSP22	-0.10248	0.531395	0.817386	None
14079	INIP	-0.07504	0.531424	0.817386	None
14080	CLEC4G	-0.27506	0.531517	0.817472	None
14081	ENTPD3-A	-0.05217	0.531612	0.817537	None
14082	LOC10013	-0.00188	0.531635	0.817537	None
14083	RSPO4	-0.01034	0.531759	0.817669	None
14084	LIN28A	-0.0194	0.531817	0.817701	None
14085	C7	-0.14569	0.531911	0.817743	None
14086	RPL22L1	-0.09906	0.531936	0.817743	None
14087	SNHG18	-0.01533	0.531958	0.817743	None
14088	RAB27A	0.114612	0.532082	0.817875	None
14089	FAM212B-	-0.13783	0.532172	0.817914	None
14090	CYAT1	-0.02743	0.532216	0.817914	None
14091	MIA	-0.04412	0.532243	0.817914	None
14092	LL0XNC01-	-0.07475	0.532258	0.817914	None

14093	ZNF85	-0.09407	0.532323	0.817956	None
14094	CARD10	-0.04101	0.532421	0.818049	None
14095	CDH17	-0.00569	0.532525	0.818105	None
14096	BC040311	-0.00568	0.532575	0.818105	None
14097	KCTD16	0.007044	0.532592	0.818105	None
14098	MYL10	-0.02852	0.532617	0.818105	None
14099	RHEBL1	-0.08138	0.532672	0.818105	None
14100	GJA3	-0.02382	0.532685	0.818105	None
14101	LOC10272	0.128762	0.532752	0.81815	None
14102	CCDC135	0.012384	0.532883	0.818289	None
14103	IDO2	-0.00238	0.532945	0.818289	None
14104	LINC01227	0.009331	0.533023	0.818289	None
14105	TAS2R3	-0.00216	0.533045	0.818289	None
14106	MSL3	0.095244	0.533046	0.818289	None
14107	LHX8	-0.00164	0.533069	0.818289	None
14108	HMCN1	-0.05247	0.533248	0.818476	None
14109	CCM2L	0.039399	0.533305	0.818476	None
14110	LOC10192	-0.01613	0.533347	0.818476	None
14111	MOB1A	0.088469	0.53335	0.818476	None
14112	NPSA	-0.00829	0.533419	0.818476	None
14113	RP11-1017	-0.00523	0.533422	0.818476	None
14114	FAM184B	-0.24019	0.533495	0.818476	None
14115	SOX13	-0.00907	0.533576	0.818476	None
14116	NAB1	-0.11225	0.533653	0.818476	None
14117	UNKL	-0.06141	0.533657	0.818476	None
14118	ARL3	-0.04105	0.533667	0.818476	None
14119	DUSP1	-0.17834	0.533679	0.818476	None
14120	FAM149A	-0.04814	0.533682	0.818476	None
14121	SLC41A3	0.095499	0.533776	0.818525	None
14122	LINC00867	-0.00271	0.533821	0.818525	None
14123	FBN3	0.025977	0.533827	0.818525	None
14124	DPEP1	-0.01418	0.533926	0.818618	None
14125	DNAH8	-0.0083	0.534088	0.818808	None
14126	EAF2	-0.03591	0.534175	0.818841	None
14127	LOC10192	-0.00763	0.534185	0.818841	None
14128	SMAD1-AS	0.006905	0.534401	0.818954	None
14129	MTHFD2	0.06449	0.53441	0.818954	None
14130	SYNE4	-0.00312	0.534423	0.818954	None
14131	SH2D1A	-0.0961	0.534438	0.818954	None
14132	COMMD2	0.063549	0.534447	0.818954	None
14133	SQLE	0.103272	0.534492	0.818965	None
14134	RPL26L1	-0.06854	0.534618	0.819099	None
14135	TMC2	-0.00762	0.53472	0.819198	None
14136	NOP14-AS	0.009166	0.534855	0.819346	None
14137	LOC34058	-0.00182	0.534937	0.819415	None
14138	ZNF250	0.039394	0.535016	0.819477	None
14139	LOC10013	-0.01846	0.535142	0.819563	None
14140	ZNF606	0.110196	0.535165	0.819563	None
14141	LOC14779	-0.00695	0.5352	0.819563	None
14142	SOX7	0.07394	0.535223	0.819563	None
14143	SURF4	0.091143	0.535265	0.819569	None
14144	SGK494	-0.1213	0.535427	0.819731	None
14145	DQ588163	-0.0052	0.535447	0.819731	None
14146	CASS4	-0.05604	0.535548	0.819772	None
14147	ASMT	0.009772	0.535549	0.819772	None
14148	LOC10192	-0.00855	0.53561	0.819807	None
14149	GSTT2	-0.02886	0.535703	0.819892	None
14150	BAI2	-0.00375	0.535746	0.819899	None

14151	VN1R4	-0.00176	0.535804	0.81993	None
14152	FEM1C	-0.13376	0.535953	0.820101	None
14153	GGT6	-0.01009	0.536054	0.820197	None
14154	MIDN	-0.00944	0.536144	0.820276	None
14155	RHBG	-0.00498	0.536257	0.820334	None
14156	HOXB-AS1	-0.06995	0.536282	0.820334	None
14157	SPATA45	-0.01706	0.536295	0.820334	None
14158	ATP4A	0.019743	0.536339	0.820343	None
14159	TMEM257	-0.00606	0.536403	0.820384	None
14160	TMEM150(	-0.11503	0.536492	0.820462	None
14161	ASB6	-0.05772	0.536594	0.820536	None
14162	AP3M1	-0.06218	0.536639	0.820536	None
14163	C1QTNF1-	-0.01978	0.536655	0.820536	None
14164	LALBA	-0.00198	0.536984	0.820771	None
14165	RPS6KA2-/	-0.02021	0.536991	0.820771	None
14166	ZNF586	-0.04804	0.537082	0.820771	None
14167	FAM166B	-0.00491	0.537094	0.820771	None
14168	AC018766.	-0.00124	0.5371	0.820771	None
14169	11-Mar	0.020141	0.537148	0.820771	None
14170	TRERF1	-0.05476	0.537166	0.820771	None
14171	LOC10029	0.015108	0.537174	0.820771	None
14172	LOC10192	-0.0237	0.537246	0.820771	None
14173	BCDIN3D	-0.09102	0.537314	0.820771	None
14174	LINC01305	-0.00663	0.537338	0.820771	None
14175	PHOSPHO	-0.02609	0.537342	0.820771	None
14176	ARHGAP9	0.093182	0.537442	0.820771	None
14177	ANKRD49	-0.07031	0.537463	0.820771	None
14178	FAT1	-0.03738	0.537465	0.820771	None
14179	ATP6V1H	-0.10652	0.537477	0.820771	None
14180	LOC10013(	-0.0051	0.537507	0.820771	None
14181	MND1	0.216354	0.537533	0.820771	None
14182	ADARB2	-0.02	0.537541	0.820771	None
14183	AOC1	-0.0452	0.537566	0.820771	None
14184	COL4A3BP	-0.08937	0.537648	0.820839	None
14185	LOC72908:	-0.01575	0.537694	0.82085	None
14186	CTSL	-0.31494	0.537936	0.821162	None
14187	POLG	-0.07706	0.538079	0.821323	None
14188	LOC10192	0.051819	0.538326	0.821628	None
14189	ANO8	-0.01441	0.538355	0.821628	None
14190	RGS9	0.008041	0.538468	0.821648	None
14191	KLHL22	-0.01321	0.538505	0.821648	None
14192	BZRAP1-A	0.151155	0.538512	0.821648	None
14193	CDH8	-0.00201	0.53854	0.821648	None
14194	CRCP	0.055723	0.538558	0.821648	None
14195	SH3KBP1	-0.06742	0.538637	0.82171	None
14196	FOXE3	-0.00301	0.538715	0.821717	None
14197	DQ582785	-0.00309	0.538717	0.821717	None
14198	AGAP11	-0.00224	0.538814	0.821731	None
14199	PPIH	0.095961	0.538881	0.821731	None
14200	VTCN1	0.012326	0.538991	0.821731	None
14201	GZMA	-0.19583	0.538997	0.821731	None
14202	AC008088.	-0.06252	0.539015	0.821731	None
14203	PBLD	0.048556	0.539123	0.821731	None
14204	PDE1B	-0.00903	0.539143	0.821731	None
14205	HIST1H1T	-0.02644	0.539261	0.821731	None
14206	SRD5A3	0.098517	0.539303	0.821731	None
14207	GRAPL	0.003372	0.539314	0.821731	None
14208	EPHB3	0.013152	0.539373	0.821731	None

14209	DND1	-0.09974	0.539397	0.821731	None
14210	TTC27	-0.0966	0.539438	0.821731	None
14211	JARID2	-0.08561	0.539452	0.821731	None
14212	MS4A1	-0.09963	0.53947	0.821731	None
14213	GBX1	0.027491	0.539482	0.821731	None
14214	RIPK4	-0.0176	0.539488	0.821731	None
14215	LOC10192!	0.000874	0.539491	0.821731	None
14216	DNAAF3	-0.14761	0.539509	0.821731	None
14217	LOC28483'	-0.08472	0.539516	0.821731	None
14218	ZNF385B	-0.00086	0.539544	0.821731	None
14219	ANKRD31	0.03756	0.539606	0.821731	None
14220	RP11-749H	-0.01514	0.53961	0.821731	None
14221	SDR16C5	-0.01082	0.539637	0.821731	None
14222	PREX2	-0.00405	0.53971	0.821755	None
14223	VWC2	0.007512	0.539729	0.821755	None
14224	CLDN8	-0.00611	0.540101	0.822237	None
14225	LOC10192!	-0.0098	0.540149	0.822237	None
14226	PTH	-0.00148	0.540201	0.822237	None
14227	MICA	-0.0769	0.540238	0.822237	None
14228	CNN1	-0.09718	0.540271	0.822237	None
14229	SEC22B	0.101109	0.540273	0.822237	None
14230	LOC10192!	-0.09688	0.540329	0.822264	None
14231	GCGR	-0.01006	0.540379	0.822283	None
14232	AKTIP	0.071241	0.540449	0.822324	None
14233	ADRBK1	-0.01644	0.540527	0.822324	None
14234	METAP1D	-0.00907	0.540577	0.822324	None
14235	TMEM136	-0.11305	0.540582	0.822324	None
14236	FMNL3	-0.03438	0.540596	0.822324	None
14237	RNF183	-0.0159	0.540712	0.8224	None
14238	LOC10192!	-0.01917	0.540753	0.8224	None
14239	C11orf44	-0.00325	0.540798	0.8224	None
14240	SLC39A2	-0.00464	0.540814	0.8224	None
14241	ST6GALNA	-0.08692	0.540835	0.8224	None
14242	ERVMER34	-0.02903	0.540914	0.822452	None
14243	EME1	0.083937	0.540963	0.822452	None
14244	LOC65434:	0.013265	0.540984	0.822452	None
14245	DTX3	-0.00325	0.541222	0.822756	None
14246	CTD-2373:	-0.01333	0.541412	0.822939	None
14247	C1R	-0.10627	0.541418	0.822939	None
14248	NUS1	-0.07712	0.541563	0.823102	None
14249	RPS27A	-0.12227	0.541628	0.823113	None
14250	ATXN3L	-0.00528	0.541647	0.823113	None
14251	ZBED4	0.067555	0.541737	0.823173	None
14252	KCTD12	-0.26356	0.541772	0.823173	None
14253	NR1I3	-0.00251	0.541808	0.823173	None
14254	ZNF41	-0.01733	0.541844	0.823173	None
14255	CDS2	0.100957	0.541891	0.823173	None
14256	LOC72811	0.015249	0.54197	0.823173	None
14257	EFS	-0.05638	0.541994	0.823173	None
14258	HDX	-0.01186	0.542009	0.823173	None
14259	MAPK15	-0.01539	0.542028	0.823173	None
14260	COQ7	-0.0828	0.542225	0.823398	None
14261	RPUSD3	-0.15742	0.542322	0.823398	None
14262	AC005498.	0.027285	0.542343	0.823398	None
14263	GPR65	0.195979	0.542373	0.823398	None
14264	CCDC87	-0.0086	0.542386	0.823398	None
14265	ANKS4B	-0.00501	0.542405	0.823398	None
14266	DAZAP1	0.072739	0.542461	0.823426	None

14267	BC113958	-0.10824	0.54254	0.823489	None
14268	PCDHB6	0.013857	0.542606	0.823515	None
14269	LOC10050‹	-0.00246	0.54265	0.823515	None
14270	AATF	-0.08179	0.542672	0.823515	None
14271	RP13-20L1	-0.00188	0.542848	0.823724	None
14272	ZAR1	-0.00579	0.543038	0.823858	None
14273	CHRND	-0.02753	0.543042	0.823858	None
14274	FSHR	0.001198	0.54305	0.823858	None
14275	LOC10192‹	-0.00662	0.543117	0.823902	None
14276	RASL10A	0.059911	0.543269	0.823963	None
14277	APELA	-0.00745	0.543281	0.823963	None
14278	C2orf68	0.102758	0.543321	0.823963	None
14279	TMED10	-0.07974	0.543329	0.823963	None
14280	PPAP2B	-0.25405	0.543406	0.823963	None
14281	ADRA1B	-0.00305	0.543409	0.823963	None
14282	TJP2	0.058057	0.543423	0.823963	None
14283	OTUD7B	-0.0173	0.543512	0.824039	None
14284	AC144652.	-0.11714	0.543554	0.824046	None
14285	LHX4	0.004615	0.543782	0.824332	None
14286	DR1	-0.05965	0.543876	0.82439	None
14287	RNF11B	-0.0046	0.543928	0.82439	None
14288	LOC10192‹	-0.07017	0.543974	0.82439	None
14289	LHX2	-0.00345	0.544	0.82439	None
14290	PCNXL4	0.041151	0.544075	0.82439	None
14291	APOA5	0.013238	0.544086	0.82439	None
14292	PCDHGB7	-0.04121	0.544086	0.82439	None
14293	DNM1P41	-0.02552	0.544138	0.824411	None
14294	CBX5	0.100864	0.544208	0.824459	None
14295	CBX2	-0.05634	0.544286	0.824513	None
14296	RP11-567(	0.015458	0.544399	0.824513	None
14297	REM1	0.045174	0.544401	0.824513	None
14298	LOC10028‹	-0.00349	0.544431	0.824513	None
14299	GPA33	-0.15494	0.544505	0.824513	None
14300	APAF1	-0.02792	0.544522	0.824513	None
14301	PRELID1	-0.10983	0.544541	0.824513	None
14302	F2RL3	0.009812	0.544548	0.824513	None
14303	STARD10	-0.13258	0.544619	0.824563	None
14304	C1QTNF9E	-0.02928	0.544831	0.824826	None
14305	LOC10192(	-0.00733	0.544883	0.824847	None
14306	PLA2G2E	-0.00277	0.544942	0.824856	None
14307	HCN2	-0.01194	0.544965	0.824856	None
14308	GLIS3-AS1	-0.00249	0.545137	0.824965	None
14309	ALS2CL	-0.03936	0.545152	0.824965	None
14310	UTS2	-0.05856	0.545173	0.824965	None
14311	KRTCAP2	0.037311	0.545189	0.824965	None
14312	TREM2	-0.02653	0.545256	0.825008	None
14313	SYN1	0.009653	0.545507	0.825193	None
14314	LOC10192‹	-0.00328	0.545557	0.825193	None
14315	TUBB7P	-0.01197	0.545586	0.825193	None
14316	ASMTL	0.081001	0.545601	0.825193	None
14317	RP11-338I:	-0.0105	0.545667	0.825193	None
14318	TAPBPL	-0.1112	0.545696	0.825193	None
14319	STARD6	-0.02091	0.545711	0.825193	None
14320	ZFC3H1	-0.08213	0.545712	0.825193	None
14321	CD276	0.018279	0.545721	0.825193	None
14322	CHP2	0.001785	0.545812	0.825273	None
14323	MAGEB4	0.008835	0.545891	0.825334	None
14324	P4HB	0.128535	0.54594	0.825352	None

14325	TXNDC9	-0.08341	0.546002	0.825387	None
14326	LOC10192	0.011675	0.54608	0.825448	None
14327	TMEM88	0.097705	0.546175	0.825461	None
14328	GNPTAB	-0.10216	0.546195	0.825461	None
14329	USP5	-0.07058	0.546228	0.825461	None
14330	ANO2	-0.01059	0.546316	0.825461	None
14331	PEX3	-0.0862	0.546355	0.825461	None
14332	C9orf116	-0.01417	0.546395	0.825461	None
14333	STK31	0.074549	0.546442	0.825461	None
14334	RP3-334F4	0.00549	0.546464	0.825461	None
14335	STAC2	-0.01448	0.54647	0.825461	None
14336	LOC10013	-0.00574	0.54647	0.825461	None
14337	HIC1	-0.02042	0.546586	0.825538	None
14338	C7orf62	-0.00468	0.546598	0.825538	None
14339	MTSS1L	-0.01894	0.546707	0.825633	None
14340	SRGAP1	-0.00686	0.546736	0.825633	None
14341	LOC72968	0.200763	0.546893	0.825787	None
14342	DUSP27	-0.1218	0.546915	0.825787	None
14343	LOC10272	-0.00822	0.547121	0.826041	None
14344	12-Sep	-0.01088	0.54722	0.826133	None
14345	EDIL3	-0.00124	0.547462	0.82644	None
14346	NXPE3	-0.08279	0.547622	0.826569	None
14347	CNTD1	-0.00307	0.547623	0.826569	None
14348	SDR9C7	-0.00986	0.547694	0.826613	None
14349	WDR49	0.123673	0.547758	0.826613	None
14350	KANK3	-0.07847	0.547858	0.826613	None
14351	MYH3	-0.12169	0.547921	0.826613	None
14352	LOC10013	-0.00327	0.547924	0.826613	None
14353	NPC1	0.047169	0.547926	0.826613	None
14354	CCSER2	0.052144	0.547952	0.826613	None
14355	LOC10099	-0.01721	0.547958	0.826613	None
14356	ZMPSTE24	-0.0563	0.548012	0.826637	None
14357	OR1E1	-0.01421	0.548075	0.826674	None
14358	ASRGL1	0.141612	0.548174	0.826744	None
14359	LOC10063	-0.03957	0.548198	0.826744	None
14360	NADK	-0.07061	0.548383	0.826966	None
14361	LINC00856	-0.00932	0.548506	0.827079	None
14362	OLA1	0.056736	0.548534	0.827079	None
14363	LINC00280	0.020005	0.548632	0.827127	None
14364	MATN1	-0.03953	0.548643	0.827127	None
14365	ACHE	0.00857	0.548714	0.827153	None
14366	LOC10192	-0.00943	0.548736	0.827153	None
14367	EFTUD2	-0.0422	0.548806	0.827177	None
14368	LOC10192	-0.01664	0.548872	0.827177	None
14369	CYP27C1	0.012508	0.548882	0.827177	None
14370	LOC10265	-0.01015	0.548905	0.827177	None
14371	GZMK	-0.11668	0.549214	0.827525	None
14372	DEFB119	-0.00135	0.549229	0.827525	None
14373	SLC25A13	-0.10549	0.54925	0.827525	None
14374	C16orf82	-0.00198	0.549295	0.827535	None
14375	LOC10272	-0.00499	0.549434	0.827676	None
14376	ZDHHC15	-0.13803	0.549473	0.827676	None
14377	GRHL3	-0.00178	0.549539	0.827676	None
14378	SCMH1	0.114131	0.549563	0.827676	None
14379	CUZD1	-0.06234	0.54958	0.827676	None
14380	UBOX5	-0.05218	0.549688	0.827731	None
14381	OPRL1	-0.01588	0.549699	0.827731	None
14382	CLSTN3	-0.06229	0.549767	0.827731	None

14383	ERVH-6	-0.01689	0.549804	0.827731 None
14384	ACTN2	0.004397	0.549809	0.827731 None
14385	NONO	-0.06243	0.549846	0.827731 None
14386	HIST3H2A	0.103384	0.549914	0.827777 None
14387	LOC10192!	-0.00313	0.550021	0.827879 None
14388	RP11-171I:	-0.00219	0.550165	0.827898 None
14389	TSHR	-0.00883	0.550167	0.827898 None
14390	BRCA2	0.104032	0.55023	0.827898 None
14391	LOC10272‹	-0.01608	0.550289	0.827898 None
14392	LRRC73	-0.0173	0.550311	0.827898 None
14393	PTEN	-0.06359	0.550311	0.827898 None
14394	CEACAM8	0.311072	0.550323	0.827898 None
14395	TNNC1	-0.00475	0.550382	0.827898 None
14396	ELMO2	-0.0657	0.550408	0.827898 None
14397	LMX1B	-0.013	0.550416	0.827898 None
14398	PDE6A	-0.02051	0.550624	0.827969 None
14399	PDCD1	0.010529	0.550657	0.827969 None
14400	CD2	-0.16667	0.550663	0.827969 None
14401	COX6B1	-0.08531	0.550669	0.827969 None
14402	C7orf71	-0.00463	0.550741	0.827969 None
14403	ITIH6	-0.00568	0.550745	0.827969 None
14404	CAPN14	-0.07647	0.550746	0.827969 None
14405	LOC15476:	-0.13402	0.550831	0.827969 None
14406	LIMD1-AS:	-0.04293	0.550839	0.827969 None
14407	FAM66D	-0.00811	0.550845	0.827969 None
14408	RP3-476K8	-0.00605	0.550922	0.827999 None
14409	RAI14	-0.12642	0.550942	0.827999 None
14410	KIAA0509	-0.00362	0.551107	0.82815 None
14411	H2AFJ	0.084691	0.551118	0.82815 None
14412	MROH2A	-0.00682	0.551361	0.828457 None
14413	TAF15	-0.10815	0.551412	0.828476 None
14414	WNT6	-0.00364	0.551606	0.828705 None
14415	C3orf38	0.107239	0.551682	0.828705 None
14416	ASB2	-0.01519	0.551697	0.828705 None
14417	JUN	-0.22555	0.551717	0.828705 None
14418	ANKRD32	0.060739	0.551845	0.828776 None
14419	SHANK1	-0.00476	0.551855	0.828776 None
14420	LINC-PINT	-0.09368	0.551914	0.828776 None
14421	SP7	0.007214	0.551968	0.828776 None
14422	APPL1	0.087579	0.552018	0.828776 None
14423	PRIMA1	0.026193	0.552033	0.828776 None
14424	COMMD9	-0.0771	0.552038	0.828776 None
14425	LINC00317	-0.00749	0.552079	0.828776 None
14426	SLC22A1	0.029429	0.552109	0.828776 None
14427	RP1-102H:	0.112036	0.55224	0.828807 None
14428	BANK1	0.158284	0.55241	0.828807 None
14429	RPH3AL	-0.01352	0.552427	0.828807 None
14430	LOC10192ŧ	-0.01607	0.552462	0.828807 None
14431	KIAA1731	-0.06912	0.55254	0.828807 None
14432	ADAM3A	-0.02071	0.552588	0.828807 None
14433	RP11-466/	-0.00744	0.552595	0.828807 None
14434	LOC10050(	-0.01635	0.552662	0.828807 None
14435	KIAA0125	0.141009	0.552665	0.828807 None
14436	LOC10192ŧ	-0.00376	0.552666	0.828807 None
14437	RP11-503(	0.02434	0.552669	0.828807 None
14438	NCBP2-AS	0.070716	0.552718	0.828807 None
14439	WDR64	-0.01351	0.55274	0.828807 None
14440	SHISA8	-0.00791	0.552765	0.828807 None

14441	NNT	0.069119	0.55277	0.828807	None
14442	IQCF5	-0.00981	0.552772	0.828807	None
14443	IFNAR2	0.065277	0.55283	0.828807	None
14444	ACR	-0.01283	0.552846	0.828807	None
14445	DAP	-0.10577	0.552897	0.828807	None
14446	SLC22A18/	0.029491	0.552953	0.828807	None
14447	IGHG1	-0.00206	0.553013	0.828807	None
14448	CNOT10	-0.0774	0.553026	0.828807	None
14449	WRNIP1	-0.07503	0.553075	0.828807	None
14450	PYHIN1	-0.06195	0.553107	0.828807	None
14451	RP11-753D	-0.00422	0.553218	0.828807	None
14452	FAM221B	-0.00584	0.55323	0.828807	None
14453	GLI2	-0.02434	0.55323	0.828807	None
14454	SPINK13	-0.00231	0.553244	0.828807	None
14455	CDH7	-0.06184	0.553293	0.828807	None
14456	RASL12	-0.00937	0.553356	0.828807	None
14457	TSPAN7	-0.21274	0.553393	0.828807	None
14458	LOC10050(	0.013675	0.553421	0.828807	None
14459	ZNF155	-0.05198	0.55343	0.828807	None
14460	RQCD1	-0.08386	0.553494	0.828807	None
14461	ZBTB38	-0.0712	0.553501	0.828807	None
14462	C1orf145	-0.00834	0.553508	0.828807	None
14463	BHMT2	-0.0066	0.553559	0.828826	None
14464	NAG18	0.065736	0.553601	0.828833	None
14465	TMOD2	0.094693	0.553752	0.829002	None
14466	ESPL1	0.149048	0.553793	0.829006	None
14467	LOC10192	-0.00675	0.553886	0.829087	None
14468	LRP12	-0.12563	0.554013	0.829162	None
14469	FKBP2	-0.12948	0.554042	0.829162	None
14470	3-Sep	-0.0269	0.554055	0.829162	None
14471	MCTP1	0.147425	0.55409	0.829162	None
14472	MICAL3	0.030434	0.554138	0.829162	None
14473	ST8SIA5	-0.01938	0.554202	0.829162	None
14474	C21orf2	0.05103	0.554366	0.829162	None
14475	LINC01267	-0.00697	0.554388	0.829162	None
14476	TSGA10IP	-0.01252	0.554397	0.829162	None
14477	FGF23	-0.0108	0.554427	0.829162	None
14478	BC028044	-0.01115	0.554444	0.829162	None
14479	AP001063.	-0.0043	0.554524	0.829162	None
14480	FOXQ1	-0.0024	0.554531	0.829162	None
14481	SCN4B	-0.0105	0.554535	0.829162	None
14482	SPRED2	0.01237	0.554659	0.829162	None
14483	BAGE	-0.00476	0.554696	0.829162	None
14484	SYT7	-0.00179	0.55479	0.829162	None
14485	LHFPL5	-0.00192	0.554826	0.829162	None
14486	AK021804	-0.12757	0.554892	0.829162	None
14487	ST8SIA6-A	-0.03593	0.554932	0.829162	None
14488	COL24A1	-0.2401	0.554939	0.829162	None
14489	TCEB2	-0.08324	0.554977	0.829162	None
14490	ELOVL4	-0.01371	0.555051	0.829162	None
14491	RWDD3	-0.11266	0.555083	0.829162	None
14492	RP11-318/	-0.05539	0.555144	0.829162	None
14493	LRRIQ3	-0.03814	0.555163	0.829162	None
14494	LOC40117(	-0.01042	0.555168	0.829162	None
14495	LOC10254(	0.007649	0.555181	0.829162	None
14496	PCCB	-0.06584	0.555211	0.829162	None
14497	OR1F2P	-0.00986	0.555247	0.829162	None
14498	LOC10192:	-0.00308	0.555298	0.829162	None

14499	DKFZp779I	0.017947	0.555406	0.829162	None
14500	LOC10192	-0.00328	0.555446	0.829162	None
14501	RHBDF2	-0.07182	0.555488	0.829162	None
14502	CCDC154	-0.0311	0.555527	0.829162	None
14503	HEMGN	-0.18552	0.555582	0.829162	None
14504	NME1	0.105112	0.555584	0.829162	None
14505	PHF21B	-0.00171	0.55559	0.829162	None
14506	RNF115	-0.0508	0.555592	0.829162	None
14507	DUSP19	-0.0417	0.555594	0.829162	None
14508	CCDC24	-0.01134	0.555612	0.829162	None
14509	ARHGDIA	-0.07819	0.555664	0.829162	None
14510	MGAT5B	-0.01647	0.555717	0.829162	None
14511	ARMCX2	-0.18743	0.555756	0.829162	None
14512	KLK10	-0.00275	0.555761	0.829162	None
14513	IFNG	-0.00112	0.555769	0.829162	None
14514	RGS9BP	-0.01193	0.555808	0.829162	None
14515	KCNE1	-0.04195	0.555816	0.829162	None
14516	DYNLT3	0.087084	0.555881	0.829162	None
14517	BID	-0.08657	0.555953	0.829162	None
14518	PVALB	-0.10045	0.555989	0.829162	None
14519	LOC10012	-0.00796	0.556001	0.829162	None
14520	NET1	-0.11755	0.556016	0.829162	None
14521	EGFL6	-0.11446	0.556047	0.829162	None
14522	KCNA6	-0.00378	0.556051	0.829162	None
14523	LOC15000	-0.01418	0.55608	0.829162	None
14524	PROX2	-0.00608	0.55612	0.829164	None
14525	HPGD	-0.21528	0.556222	0.829259	None
14526	CNTN5	-0.00397	0.556295	0.82931	None
14527	C9orf152	-0.01495	0.556366	0.829359	None
14528	SAE1	0.126567	0.556437	0.829407	None
14529	GMNN	0.108233	0.556524	0.829407	None
14530	LOC10192	-0.00122	0.556542	0.829407	None
14531	ZNF790-A	0.085449	0.556551	0.829407	None
14532	CTC-436K	-0.00272	0.556618	0.82945	None
14533	RAET1E	-0.00206	0.55668	0.82946	None
14534	FMO6P	-0.00426	0.556715	0.82946	None
14535	IQCF6	-0.00745	0.55674	0.82946	None
14536	DGCR10	-0.00963	0.556783	0.829468	None
14537	RYR2	-0.03747	0.556904	0.829531	None
14538	DUS4L	0.083837	0.556916	0.829531	None
14539	GGT1	0.013208	0.556941	0.829531	None
14540	UCA1	0.187723	0.55702	0.829545	None
14541	RBMS3-AS	-0.00139	0.557044	0.829545	None
14542	STK24-AS1	-0.01934	0.557065	0.829545	None
14543	LINC01352	-0.00363	0.557124	0.829575	None
14544	AVPR1A	-0.02028	0.557188	0.829597	None
14545	ADAMTS2	-0.00284	0.557215	0.829597	None
14546	APBB2	-0.02268	0.557295	0.829659	None
14547	RGS6	-0.02681	0.557538	0.829963	None
14548	GLE1	-0.06524	0.557591	0.829986	None
14549	MXRA8	-0.04608	0.557722	0.83008	None
14550	ROBO4	-0.08688	0.557731	0.83008	None
14551	TEAD2	0.014394	0.557781	0.830098	None
14552	C6orf120	-0.06201	0.557896	0.830212	None
14553	GRIK1-AS2	-0.00453	0.558048	0.830333	None
14554	TMEM108	-0.03552	0.558054	0.830333	None
14555	GPRC5B	0.012933	0.55815	0.830335	None
14556	LOC10192	-0.06676	0.558163	0.830335	None

14557	RSU1	-0.04934	0.558171	0.830335	None
14558	LOC10050!	-0.0171	0.558236	0.830375	None
14559	IGFBP6	-0.08339	0.558317	0.830439	None
14560	AFG3L1P	-0.07147	0.558367	0.830456	None
14561	HNRNPU	-0.09405	0.558449	0.830457	None
14562	IL23R	0.004302	0.558452	0.830457	None
14563	NUDT1	0.1146	0.558483	0.830457	None
14564	LINC01146	-0.0026	0.558573	0.830534	None
14565	ABCC2	-0.02148	0.558673	0.83062	None
14566	VIL1	-0.03163	0.558724	0.83062	None
14567	LOC10193(	0.028522	0.558746	0.83062	None
14568	SPTBN2	-0.01622	0.558808	0.830656	None
14569	RP11-138I.	-0.0082	0.558886	0.830694	None
14570	LOC10192!	-0.00176	0.558911	0.830694	None
14571	DBNDD1	0.010959	0.559044	0.830835	None
14572	C12orf73	0.085004	0.559255	0.831092	None
14573	FREM3	-0.00661	0.559346	0.831094	None
14574	HTR7	0.012255	0.559404	0.831094	None
14575	GBA3	-0.01886	0.559407	0.831094	None
14576	C17orf85	0.080761	0.55941	0.831094	None
14577	DAW1	-0.00125	0.559552	0.831224	None
14578	MGAT4C	-0.00541	0.559574	0.831224	None
14579	C19orf81	-0.02311	0.559703	0.831225	None
14580	LOC10192!	-0.00139	0.559757	0.831225	None
14581	IZUMO1	-0.01607	0.559803	0.831225	None
14582	ATF1	0.08741	0.559834	0.831225	None
14583	AIG1	-0.09894	0.559834	0.831225	None
14584	ADAM11	0.021618	0.559866	0.831225	None
14585	MIR205HG	-0.00312	0.559869	0.831225	None
14586	PTPN11	0.043831	0.559882	0.831225	None
14587	CXorf67	-0.00621	0.55999	0.831329	None
14588	SSSCA1	-0.11005	0.560152	0.831428	None
14589	ARHGEF25	0.028847	0.560167	0.831428	None
14590	DAZAP2	-0.05526	0.56018	0.831428	None
14591	ADAMTS4	0.009481	0.560322	0.831428	None
14592	SOX21-AS	-0.01198	0.560328	0.831428	None
14593	RP11-498(	-0.00442	0.560417	0.831428	None
14594	FAM185A	-0.067	0.560418	0.831428	None
14595	E2F1	0.06056	0.560428	0.831428	None
14596	ALDH1A1	0.259803	0.560437	0.831428	None
14597	ZWILCH	0.085341	0.560451	0.831428	None
14598	ATP5S	-0.06891	0.560496	0.831428	None
14599	C8orf46	-0.00117	0.560518	0.831428	None
14600	AC000111.	-0.00564	0.560629	0.831472	None
14601	SNRNP70	-0.05005	0.56065	0.831472	None
14602	LOC10192!	-0.01223	0.560701	0.831472	None
14603	TNFRSF9	-0.00676	0.560701	0.831472	None
14604	C14orf64	0.01331	0.560787	0.831542	None
14605	TMEM8B	-0.02395	0.560893	0.831601	None
14606	SPINK6	0.001131	0.560958	0.831601	None
14607	HYAL1	-0.00119	0.560969	0.831601	None
14608	CNTN1	-0.02147	0.56098	0.831601	None
14609	CALY	-0.00652	0.561053	0.831615	None
14610	FCRLA	0.054714	0.561066	0.831615	None
14611	TPO	-0.00853	0.561479	0.832141	None
14612	LOC10192!	-0.00415	0.561498	0.832141	None
14613	ZNF148	0.066215	0.56161	0.832178	None
14614	TEAD1	-0.00586	0.56163	0.832178	None

14615	SAP30	-0.06214	0.561638	0.832178	None
14616	RP4-647C1	-0.01341	0.561698	0.832209	None
14617	RAI1	0.033938	0.561738	0.832212	None
14618	CEMP1	0.03396	0.561856	0.832329	None
14619	EIF2AK2	-0.07477	0.562073	0.832549	None
14620	CNOT4	-0.08179	0.562129	0.832549	None
14621	VWDE	-0.20133	0.562139	0.832549	None
14622	TTI1	0.105465	0.562158	0.832549	None
14623	BCL2A1	0.23351	0.562339	0.832761	None
14624	LOC10192	-0.02335	0.5625	0.832942	None
14625	CDC37	-0.10447	0.56267	0.833136	None
14626	GYPE	0.08357	0.562769	0.833175	None
14627	RIOK3	0.093718	0.562802	0.833175	None
14628	FOXB1	-0.0026	0.562812	0.833175	None
14629	GSG1L	-0.02066	0.562876	0.833176	None
14630	BBOX1	-0.01132	0.562889	0.833176	None
14631	PODXL	-0.13432	0.56293	0.83318	None
14632	LOC10050	-0.00462	0.563038	0.833245	None
14633	WNK4	-0.00355	0.563073	0.833245	None
14634	GPR133	-0.04035	0.563179	0.833245	None
14635	LINC00507	-0.00292	0.563208	0.833245	None
14636	PTPN9	-0.03366	0.563234	0.833245	None
14637	DNASE2	-0.04812	0.563243	0.833245	None
14638	TMC5	-0.01569	0.563244	0.833245	None
14639	HINT2	-0.08551	0.563393	0.833355	None
14640	LOC10192	-0.01449	0.563395	0.833355	None
14641	CCDC141	-0.06874	0.563447	0.833375	None
14642	EDNRB-AS	-0.00651	0.563607	0.833421	None
14643	ASL	-0.11744	0.563613	0.833421	None
14644	LOC72802	-0.07661	0.563699	0.833421	None
14645	CNTLN	-0.07037	0.56372	0.833421	None
14646	FBXL14	-0.03301	0.56373	0.833421	None
14647	BCL7B	0.094438	0.563734	0.833421	None
14648	RP3-452M	0.000834	0.563781	0.833421	None
14649	FBXO32	-0.02967	0.563843	0.833421	None
14650	IRX4	-0.00564	0.56385	0.833421	None
14651	FLJ46026	-0.00621	0.563906	0.833421	None
14652	LINC00582	-0.01947	0.563929	0.833421	None
14653	SLC28A3	0.039128	0.56394	0.833421	None
14654	PDE6H	-0.00464	0.564108	0.833577	None
14655	IRX5	-0.04583	0.564142	0.833577	None
14656	CAMK2A	-0.0025	0.564221	0.833577	None
14657	CD3E	-0.00424	0.564227	0.833577	None
14658	SUGT1P3	-0.05818	0.564248	0.833577	None
14659	GTF2A2	-0.08195	0.564363	0.833577	None
14660	DNAH7	0.004456	0.564371	0.833577	None
14661	HSP90AB1	-0.10808	0.564383	0.833577	None
14662	ADAMTS7	-0.00601	0.564392	0.833577	None
14663	EMR3	-0.06089	0.564497	0.833669	None
14664	RP11-231E	0.012089	0.564538	0.833669	None
14665	ZNF2	-0.08583	0.56457	0.833669	None
14666	TNRC6B	0.079542	0.564619	0.833685	None
14667	LOC10192	-0.00488	0.564693	0.833723	None
14668	PHLDB3	-0.00189	0.564729	0.833723	None
14669	RANGRF	0.06982	0.56484	0.833723	None
14670	RP11-559N	-0.04639	0.564856	0.833723	None
14671	C21orf37	0.011973	0.564912	0.833723	None
14672	RTN4	0.05557	0.564927	0.833723	None

14673	IL1RAP	0.145845	0.564965	0.833723	None
14674	WDR83	0.093854	0.565021	0.833723	None
14675	ATG3	-0.07677	0.565102	0.833723	None
14676	PTH1R	-0.03011	0.565106	0.833723	None
14677	WDR16	-0.00745	0.565116	0.833723	None
14678	FAM155A-	-0.00226	0.565141	0.833723	None
14679	ACSM1	-0.01183	0.565145	0.833723	None
14680	CTD-2350	0.002797	0.565192	0.833736	None
14681	CASD1	-0.00894	0.565242	0.833752	None
14682	FAM53C	-0.10428	0.565293	0.83377	None
14683	RBMXL2	-0.0017	0.565349	0.833797	None
14684	KPNA1	-0.07862	0.56553	0.833836	None
14685	SGOL1	-0.0693	0.565548	0.833836	None
14686	TRPM2-AS	0.020058	0.565558	0.833836	None
14687	COL11A1	-0.06258	0.565595	0.833836	None
14688	RAPGEF4-/	0.005679	0.5656	0.833836	None
14689	NRXN3	-0.01879	0.565656	0.833836	None
14690	ADAMTS2	-0.01515	0.565663	0.833836	None
14691	RP11-79P5	-0.00585	0.565684	0.833836	None
14692	C19orf40	-0.04242	0.565725	0.83384	None
14693	BC043291	-0.00756	0.565764	0.83384	None
14694	C14orf183	0.012984	0.56596	0.834054	None
14695	ZNF697	-0.06201	0.565987	0.834054	None
14696	F2	-0.01134	0.566035	0.834054	None
14697	CD36	0.283443	0.566063	0.834054	None
14698	LOC64485	-0.03834	0.566182	0.834126	None
14699	RP13-638	-0.01069	0.566241	0.834126	None
14700	SLC22A13	-0.00321	0.566269	0.834126	None
14701	IAPP	-0.00428	0.566352	0.834126	None
14702	CCL1	-0.0059	0.566359	0.834126	None
14703	SSR3	-0.06438	0.566363	0.834126	None
14704	IL32	-0.00766	0.566381	0.834126	None
14705	LOC10192	-0.0104	0.56659	0.834377	None
14706	IL26	-0.01513	0.566668	0.834434	None
14707	LOC64413	-0.00277	0.566846	0.834434	None
14708	PAX8-AS1	0.276727	0.566849	0.834434	None
14709	ZFP62	-0.07818	0.56685	0.834434	None
14710	APOC3	-0.0114	0.566855	0.834434	None
14711	NKX2-2	-0.00116	0.56694	0.834434	None
14712	LOC10192	0.018203	0.566965	0.834434	None
14713	TFPI2	-0.093	0.566992	0.834434	None
14714	LOC10192	-0.00156	0.567071	0.834434	None
14715	GUCY1B2	-0.00737	0.567158	0.834434	None
14716	TMEM208	0.106196	0.567179	0.834434	None
14717	INSL4	-0.00196	0.567195	0.834434	None
14718	ZNF354B	-0.12131	0.567214	0.834434	None
14719	CKAP5	-0.08149	0.567227	0.834434	None
14720	ZNF207	0.105528	0.567237	0.834434	None
14721	LOC10028	-0.0011	0.567265	0.834434	None
14722	TRAPPC5	-0.12317	0.567295	0.834434	None
14723	AK6	-0.07756	0.567323	0.834434	None
14724	KLHL32	-0.00302	0.567378	0.834437	None
14725	GBX2	-0.00291	0.567412	0.834437	None
14726	PHF7	0.049444	0.567476	0.834437	None
14727	SLCO4C1	0.068296	0.56753	0.834437	None
14728	FEV	-0.01472	0.56755	0.834437	None
14729	RP11-353N	0.018933	0.567556	0.834437	None
14730	LOC10192	-0.00309	0.567628	0.834486	None

14731	MT4	-0.00178	0.567735	0.834501	None
14732	ANKRD50	-0.11466	0.567783	0.834501	None
14733	EPB41L4A-	-0.01412	0.567856	0.834501	None
14734	ZCCHC13	-0.00257	0.567886	0.834501	None
14735	HSPD1	-0.20506	0.567905	0.834501	None
14736	TMEM25	0.023669	0.567932	0.834501	None
14737	CDC25B	0.118811	0.567934	0.834501	None
14738	DNAH17	0.006037	0.567946	0.834501	None
14739	ASMTL-AS	-0.01323	0.568033	0.834572	None
14740	TRPC5OS	-0.03501	0.568237	0.834815	None
14741	GRM4	0.014712	0.568343	0.83485	None
14742	LOC10050(	0.019516	0.568347	0.83485	None
14743	TBL2	0.040185	0.568377	0.83485	None
14744	RP11-352(	-0.00312	0.568455	0.83485	None
14745	CYP4F22	-0.01118	0.568494	0.83485	None
14746	EGFEM1P	-0.19185	0.568513	0.83485	None
14747	GPX8	-0.03523	0.568531	0.83485	None
14748	LOC10099(	-0.01692	0.568708	0.835028	None
14749	PCDHGC4	0.003498	0.568729	0.835028	None
14750	SERINC1	0.107947	0.568798	0.835045	None
14751	LOC10192!	-0.06626	0.568838	0.835045	None
14752	MAP3K7CL	-0.20447	0.568878	0.835045	None
14753	ZNF300P1	-0.02815	0.568967	0.835045	None
14754	FAM19A5	-0.0593	0.568986	0.835045	None
14755	RAMP3	-0.05238	0.568993	0.835045	None
14756	SEZ6	0.015136	0.56901	0.835045	None
14757	DIAPH3-A!	-0.00083	0.569173	0.835227	None
14758	PAN3-AS1	0.179328	0.569498	0.835647	None
14759	DMRT1	-0.02393	0.56955	0.835661	None
14760	CALCR	0.005272	0.569601	0.835661	None
14761	FILIP1L	-0.05967	0.569663	0.835661	None
14762	CRYAA	-0.00157	0.569705	0.835661	None
14763	CYB5R2	-0.02267	0.569708	0.835661	None
14764	LCE3D	-0.02359	0.569794	0.835661	None
14765	PDE1A	-0.01749	0.569886	0.835661	None
14766	CD96	0.16762	0.569886	0.835661	None
14767	ZC3H7A	-0.08585	0.56989	0.835661	None
14768	KRTAP3-1	-0.00763	0.569893	0.835661	None
14769	DDX60	-0.16436	0.569988	0.835743	None
14770	ZNF821	0.001517	0.570031	0.83575	None
14771	UNC5D	-0.02539	0.570169	0.835848	None
14772	FBP2	-0.01217	0.570209	0.835848	None
14773	HYAL4	-0.057	0.570349	0.835848	None
14774	LAMC3	-0.01017	0.570377	0.835848	None
14775	XRRA1	0.021741	0.570386	0.835848	None
14776	C15orf56	-0.02207	0.57039	0.835848	None
14777	SETD7	0.090984	0.570406	0.835848	None
14778	C10orf90	-0.00789	0.570438	0.835848	None
14779	NME7	-0.10783	0.570483	0.835848	None
14780	CYB5D2	-0.07994	0.570552	0.835848	None
14781	SIRPG	0.018463	0.570575	0.835848	None
14782	ZNF334	-0.12912	0.570613	0.835848	None
14783	MMRN1	0.20992	0.570614	0.835848	None
14784	LRRC32	-0.03384	0.570638	0.835848	None
14785	HIST1H4I	-0.04565	0.57068	0.835852	None
14786	GPT2	0.117706	0.570755	0.835905	None
14787	KITLG	-0.00977	0.570832	0.835962	None
14788	PDE6G	-0.09248	0.571067	0.836239	None

14789	KIR2DL1	0.027454	0.571098	0.836239	None
14790	LOC28606[illegible]	-0.00277	0.571209	0.836319	None
14791	LOC10050[illegible]	-0.0056	0.57124	0.836319	None
14792	GLYCTK	0.029148	0.571296	0.836319	None
14793	HCG4B	-0.0164	0.571365	0.836319	None
14794	TMEM231	-0.12164	0.571385	0.836319	None
14795	RNASE7	-0.00339	0.571389	0.836319	None
14796	LOC10192[illegible]	0.015111	0.571424	0.836319	None
14797	PSMC5	0.069317	0.571524	0.836371	None
14798	WBSCR27	-0.01411	0.571536	0.836371	None
14799	SULF2	0.096603	0.571613	0.836409	None
14800	GPR113	0.023594	0.571649	0.836409	None
14801	GCM2	-0.00111	0.571678	0.836409	None
14802	PSORS1C3	-0.00377	0.571751	0.836459	None
14803	FAM71F2	-0.00314	0.571969	0.836683	None
14804	LOC40095[illegible]	-0.00466	0.571981	0.836683	None
14805	RFX6	-0.00233	0.572133	0.836849	None
14806	BPIFA2	-0.0075	0.572282	0.836918	None
14807	UBASH3B	0.104548	0.572313	0.836918	None
14808	CALCB	-0.00481	0.572337	0.836918	None
14809	LINC01426	0.004547	0.572359	0.836918	None
14810	BCL11B	0.075554	0.572377	0.836918	None
14811	SOX8	-0.02172	0.572461	0.836918	None
14812	LOC14869[illegible]	-0.00736	0.572495	0.836918	None
14813	SEMA4A	0.068951	0.572558	0.836918	None
14814	SIL1	-0.10981	0.572607	0.836918	None
14815	TEX29	-0.00236	0.572688	0.836918	None
14816	CTB-174D[illegible]	-0.00141	0.572771	0.836918	None
14817	YWHAB	0.094852	0.572822	0.836918	None
14818	LOC28574[illegible]	-0.00308	0.57285	0.836918	None
14819	TIFAB	-0.15157	0.572869	0.836918	None
14820	ACTL10	0.034892	0.572924	0.836918	None
14821	BAIAP2L2	-0.02036	0.572951	0.836918	None
14822	LOC10012[illegible]	0.008385	0.572956	0.836918	None
14823	MAP1LC3C	0.076788	0.572984	0.836918	None
14824	RP11-13K1	-0.00775	0.572992	0.836918	None
14825	CTC-444N	0.123439	0.573001	0.836918	None
14826	CAPN8	-0.01063	0.573035	0.836918	None
14827	RFX3	-0.08664	0.573066	0.836918	None
14828	DHX34	0.027686	0.57307	0.836918	None
14829	IMPA2	0.092102	0.573115	0.836928	None
14830	HHATL	-0.00223	0.573218	0.837021	None
14831	C18orf42	-0.01483	0.573444	0.83725	None
14832	DAO	-0.01441	0.573477	0.83725	None
14833	ADSS	0.05094	0.57349	0.83725	None
14834	RBPJ	0.101286	0.573657	0.837435	None
14835	WBSCR17	-0.00941	0.573695	0.837435	None
14836	CSNK1A1P	-0.00402	0.573736	0.837435	None
14837	C16orf3	-0.00337	0.573771	0.837435	None
14838	NDOR1	-0.01905	0.57388	0.837508	None
14839	LLPH	0.145714	0.573973	0.837508	None
14840	BDH2	-0.04212	0.574063	0.837508	None
14841	LOC10192[illegible]	-0.0824	0.57413	0.837508	None
14842	RP11-413[illegible]	-0.00262	0.574227	0.837508	None
14843	ROBO2	-0.00206	0.574247	0.837508	None
14844	CST11	0.004125	0.574249	0.837508	None
14845	SRP19	0.075227	0.574271	0.837508	None
14846	SMIM6	-0.05235	0.574284	0.837508	None

14847	PIWIL2	-0.01093	0.574285	0.837508	None
14848	EFCAB6	-0.00288	0.57429	0.837508	None
14849	ZNF669	0.076731	0.574303	0.837508	None
14850	SMIM11	0.036584	0.574347	0.837508	None
14851	IGFBP1	-0.0041	0.574497	0.837508	None
14852	LOC339622	-0.00248	0.574527	0.837508	None
14853	TBC1D10C	-0.12162	0.57459	0.837508	None
14854	MROH8	0.027473	0.574687	0.837508	None
14855	LOC10192	-0.00487	0.574694	0.837508	None
14856	RP11-29P2	-0.01917	0.574723	0.837508	None
14857	CRLF1	0.026895	0.574739	0.837508	None
14858	DENND5A	-0.15176	0.574762	0.837508	None
14859	CDKL5	-0.00536	0.574789	0.837508	None
14860	ZNF684	-0.04739	0.574844	0.837508	None
14861	HAGLR	-0.01244	0.574853	0.837508	None
14862	CCZ1B	-0.0814	0.574859	0.837508	None
14863	C16orf71	-0.01184	0.574867	0.837508	None
14864	PMCH	-0.02856	0.574918	0.837508	None
14865	SLC7A2	-0.00179	0.574936	0.837508	None
14866	ALDH3B2	-0.00242	0.574943	0.837508	None
14867	CHST3	-0.00835	0.57499	0.83752	None
14868	LOC10192	-0.00122	0.575031	0.837524	None
14869	FAM65B	-0.09002	0.575212	0.837625	None
14870	PEX11G	-0.08756	0.575307	0.837625	None
14871	OR6W1P	-0.00811	0.575313	0.837625	None
14872	POGZ	-0.07171	0.57536	0.837625	None
14873	SIRPA	-0.07852	0.575379	0.837625	None
14874	LOC10192	-0.00331	0.575416	0.837625	None
14875	LOC10050	-0.01364	0.575468	0.837625	None
14876	LINC00879	0.00407	0.575469	0.837625	None
14877	FPR1	-0.15419	0.575469	0.837625	None
14878	UPK3A	-0.01532	0.575581	0.837625	None
14879	RP11-217E	-0.00621	0.575616	0.837625	None
14880	RP11-744D	0.008894	0.575632	0.837625	None
14881	MAF1	0.09297	0.575658	0.837625	None
14882	PSMB5	-0.11117	0.575665	0.837625	None
14883	LOC10192	0.01595	0.575718	0.837625	None
14884	CNIH1	-0.06245	0.57572	0.837625	None
14885	SYCE3	0.062941	0.575768	0.837639	None
14886	SBK1	-0.04793	0.575856	0.837711	None
14887	IGFLR1	-0.10423	0.575912	0.837736	None
14888	ROMO1	-0.15269	0.576016	0.837831	None
14889	BDKRB2	0.012318	0.576192	0.837939	None
14890	SIVA1	0.101498	0.576206	0.837939	None
14891	ORF1	-0.02016	0.576273	0.837939	None
14892	PTPRU	-0.03411	0.576281	0.837939	None
14893	LOC10050	0.089859	0.576284	0.837939	None
14894	AF090939	0.103921	0.576344	0.837941	None
14895	ACTL7A	-0.0092	0.576387	0.837941	None
14896	RP1-28O1	0.006906	0.576402	0.837941	None
14897	IGHM	0.109084	0.576532	0.838031	None
14898	PKP1	-0.01008	0.57656	0.838031	None
14899	RP11-63A	-0.00122	0.576579	0.838031	None
14900	ADAMTS8	-0.00507	0.576716	0.838173	None
14901	PPP4R4	-0.00163	0.57679	0.838225	None
14902	CYP4F11	-0.00231	0.576844	0.838248	None
14903	PIGG	-0.02601	0.576967	0.83837	None
14904	DNAJC21	0.043151	0.577072	0.838422	None

14905	CATX-1	-0.00225	0.577081	0.838422	None
14906	NEGR1-IT1	-0.00683	0.577148	0.838464	None
14907	EFCAB4A	-0.03351	0.577258	0.838494	None
14908	RP6-91H8.	-0.03416	0.577283	0.838494	None
14909	LINC00970	-0.005	0.577323	0.838494	None
14910	C8orf76	0.066762	0.577359	0.838494	None
14911	FAM49A	-0.16638	0.577462	0.838494	None
14912	GPR173	-0.00315	0.577463	0.838494	None
14913	OR3A1	-0.01508	0.577465	0.838494	None
14914	PLEKHG6	0.023219	0.577512	0.838494	None
14915	LPGAT1	0.076915	0.577569	0.838494	None
14916	RDM1	0.112989	0.57758	0.838494	None
14917	CDKN2B-A	-0.00178	0.577648	0.838494	None
14918	TCN1	-0.24544	0.57767	0.838494	None
14919	FUCA2	-0.11014	0.577672	0.838494	None
14920	KRT1	-0.12694	0.577728	0.838518	None
14921	TENC1	-0.02632	0.577781	0.83854	None
14922	GNB1L	-0.03708	0.577845	0.838556	None
14923	BC024169	-0.00756	0.57787	0.838556	None
14924	RENBP	-0.05776	0.578028	0.838638	None
14925	PERP	-0.01061	0.578056	0.838638	None
14926	MDGA1	-0.0298	0.578087	0.838638	None
14927	D21S2088E	0.002799	0.578107	0.838638	None
14928	DDX21	0.062184	0.578123	0.838638	None
14929	STRN	0.088291	0.578164	0.838638	None
14930	URB2	-0.09856	0.578212	0.838638	None
14931	LINGO3	-0.02653	0.578236	0.838638	None
14932	RLTPR	-0.04346	0.578294	0.838665	None
14933	MRPL28	-0.09104	0.578373	0.838725	None
14934	RWDD4	0.060241	0.578482	0.838807	None
14935	KLF8	0.075821	0.578526	0.838807	None
14936	Igk	-0.00578	0.578546	0.838807	None
14937	PRPS1	-0.08236	0.578613	0.838847	None
14938	IVNS1ABP	0.09759	0.578768	0.839016	None
14939	LOC10192	-0.05509	0.578842	0.839068	None
14940	C9orf62	-0.01343	0.578954	0.8391	None
14941	CCDC96	-0.02183	0.578956	0.8391	None
14942	LOC10192	-0.00641	0.578981	0.8391	None
14943	LOC38964	-0.04143	0.579058	0.839106	None
14944	PSMD12	-0.06349	0.579063	0.839106	None
14945	SLC46A2	-0.03515	0.579175	0.839213	None
14946	TSPO	0.131324	0.57935	0.83925	None
14947	TBC1D27	-0.01218	0.579393	0.83925	None
14948	CTD-2302E	-0.01015	0.579423	0.83925	None
14949	NAV2-AS5	-0.00176	0.579443	0.83925	None
14950	APOL5	-0.00447	0.579451	0.83925	None
14951	GAS8	0.063724	0.579464	0.83925	None
14952	DOCK8	0.103885	0.579472	0.83925	None
14953	DDX51	-0.02191	0.579594	0.83937	None
14954	TMEM126A	-0.07023	0.579705	0.839461	None
14955	RP11-157E	0.001236	0.579734	0.839461	None
14956	LOC10050	-0.00304	0.579786	0.83948	None
14957	CLCN5	-0.03951	0.579926	0.839627	None
14958	FAM83E	-0.00156	0.580231	0.839977	None
14959	RP11-134L	0.027573	0.580245	0.839977	None
14960	RP11-728F	-0.01842	0.580303	0.840003	None
14961	ANGPTL3	-0.00108	0.580342	0.840003	None
14962	CDX4	-0.00123	0.58046	0.840003	None

14963	GSTA4	0.102812	0.580481	0.840003	None
14964	LOC10272:	0.036557	0.580528	0.840003	None
14965	MOK	0.15894	0.580559	0.840003	None
14966	LOC10192	-0.00217	0.580591	0.840003	None
14967	EML1	-0.01043	0.580594	0.840003	None
14968	TGFBR3	-0.24172	0.580662	0.840003	None
14969	RP11-310J	0.050397	0.580666	0.840003	None
14970	ZNF608	0.091704	0.58069	0.840003	None
14971	ACBD3	0.057594	0.580807	0.840017	None
14972	LOC10192	-0.00133	0.580831	0.840017	None
14973	LOC15227	-0.00183	0.580861	0.840017	None
14974	MLEC	-0.08003	0.5809	0.840017	None
14975	WDR52	-0.09512	0.580911	0.840017	None
14976	GUSBP1	-0.00593	0.580954	0.840017	None
14977	LINC01432	-0.00266	0.581009	0.840017	None
14978	RP4-614O	-0.11654	0.581011	0.840017	None
14979	HDGFRP3	0.141611	0.581053	0.840017	None
14980	DHX38	-0.09556	0.581091	0.840017	None
14981	ALX4	-0.00468	0.581147	0.840017	None
14982	ROBO1	0.294672	0.581201	0.840017	None
14983	MAP3K15	-0.01145	0.581211	0.840017	None
14984	XG	-0.0101	0.581243	0.840017	None
14985	PRADC1	0.116853	0.581328	0.840084	None
14986	MTTP	-0.00706	0.581387	0.840114	None
14987	DSEL	-0.00553	0.581464	0.840132	None
14988	DCP1A	-0.07265	0.581537	0.840132	None
14989	PAQR4	0.090612	0.58154	0.840132	None
14990	KCNK9	0.007151	0.581556	0.840132	None
14991	PDX1	0.001391	0.581603	0.840145	None
14992	SLC18A3	-0.00677	0.581831	0.840325	None
14993	LOC10028	-0.00108	0.581841	0.840325	None
14994	MRPL41	-0.02459	0.581916	0.840325	None
14995	ART3	-0.00345	0.581968	0.840325	None
14996	ATP5J2	0.028452	0.58197	0.840325	None
14997	BEST2	-0.00775	0.58203	0.840325	None
14998	MIS18A	-0.07561	0.582048	0.840325	None
14999	LOC10028	-0.14845	0.582091	0.840325	None
15000	ADAMTSL2	-0.01805	0.582124	0.840325	None
15001	LOC10192	-0.00763	0.582139	0.840325	None
15002	CTB-176F2	-0.00683	0.582154	0.840325	None
15003	KAT6B	-0.08599	0.582288	0.840351	None
15004	PAXIP1	0.078629	0.582347	0.840351	None
15005	HCG9	-0.00334	0.582356	0.840351	None
15006	IFNGR2	-0.09502	0.582382	0.840351	None
15007	FAM166A	-0.0171	0.582385	0.840351	None
15008	TTTY13	-0.00314	0.582472	0.840351	None
15009	KAT2B	0.125521	0.582475	0.840351	None
15010	RP11-388N	-0.00156	0.582509	0.840351	None
15011	HUS1	-0.0647	0.582522	0.840351	None
15012	FOXN1	-0.00284	0.582602	0.840364	None
15013	LOC10192	-0.01842	0.58264	0.840364	None
15014	IFNA1	-0.00217	0.582647	0.840364	None
15015	GPR32	-0.00249	0.582702	0.840387	None
15016	CA5B	-0.04948	0.582763	0.840419	None
15017	LMO1	0.033065	0.582806	0.840425	None
15018	CCR2	-0.15219	0.582904	0.840445	None
15019	KBTBD8	0.134798	0.582926	0.840445	None
15020	OLIG3	-0.00267	0.582945	0.840445	None

15021	S100A5	-0.00991	0.582975	0.840445	None
15022	VAMP1	-0.09998	0.583151	0.840511	None
15023	DIO2-AS1	-0.01456	0.583245	0.840511	None
15024	JDP2	-0.05979	0.583257	0.840511	None
15025	EDARADD	-0.00572	0.583289	0.840511	None
15026	CCDC150	0.091748	0.583329	0.840511	None
15027	NAALADL2	-0.00093	0.583389	0.840511	None
15028	LOC10013	-0.00663	0.583397	0.840511	None
15029	MRGPRG-/	-0.01228	0.583409	0.840511	None
15030	LEFTY1	-0.04696	0.583447	0.840511	None
15031	PAX3	-0.00199	0.583457	0.840511	None
15032	GJB3	-0.01077	0.583476	0.840511	None
15033	LINC01104	-0.00279	0.583494	0.840511	None
15034	C8orf22	-0.00353	0.583545	0.840511	None
15035	LOC10272	-0.00215	0.583564	0.840511	None
15036	ATPIF1	0.081788	0.583702	0.840619	None
15037	LOC40132	-0.00229	0.583716	0.840619	None
15038	ELF3	-0.00325	0.583772	0.840643	None
15039	RPRD1A	-0.06064	0.583823	0.840659	None
15040	KCNG4	-0.00197	0.583914	0.840686	None
15041	DDX4	0.0008	0.583942	0.840686	None
15042	COL3A1	0.074387	0.583958	0.840686	None
15043	SSU72	-0.07534	0.584022	0.840724	None
15044	BAAT	-0.01863	0.584157	0.840831	None
15045	HOXD-AS2	-0.00156	0.584201	0.840831	None
15046	LOC10272	0.014706	0.584213	0.840831	None
15047	ARHGAP21	0.105388	0.584276	0.840856	None
15048	JHDM1D-/	0.072158	0.584309	0.840856	None
15049	GBAS	0.086964	0.584485	0.840988	None
15050	WHAMM	-0.07559	0.584523	0.840988	None
15051	SRF	-0.03258	0.584562	0.840988	None
15052	PTK2	0.103273	0.58466	0.840988	None
15053	LOC10012	-0.11624	0.584671	0.840988	None
15054	NEFH	0.250052	0.584684	0.840988	None
15055	COX8A	-0.0931	0.584704	0.840988	None
15056	RP11-496I	-0.0017	0.584728	0.840988	None
15057	AC013733.	-0.00154	0.58475	0.840988	None
15058	B4GALT5	-0.09332	0.584857	0.841046	None
15059	SAR1A	-0.12901	0.584868	0.841046	None
15060	CNTN3	-0.04315	0.584943	0.841052	None
15061	NKD1	0.00748	0.58495	0.841052	None
15062	GTF3C3	-0.07219	0.585102	0.841098	None
15063	MACROD2	-0.00145	0.58511	0.841098	None
15064	SPEM1	0.01576	0.585126	0.841098	None
15065	AIM1L	0.019162	0.585205	0.841098	None
15066	LOC10192	-0.00495	0.585234	0.841098	None
15067	MYO1H	-0.00467	0.58527	0.841098	None
15068	BCAR3	-0.07652	0.58532	0.841098	None
15069	GSTTP1	-0.00337	0.585364	0.841098	None
15070	CYP26B1	-0.04962	0.585391	0.841098	None
15071	MS4A2	0.153296	0.585431	0.841098	None
15072	LINC00525	-0.00454	0.585447	0.841098	None
15073	DPPA5	-0.00447	0.585447	0.841098	None
15074	RNF157	-0.01963	0.585598	0.841145	None
15075	BMP3	-0.00376	0.58563	0.841145	None
15076	TMEM74B	-0.00371	0.585685	0.841145	None
15077	TDP1	0.080099	0.585708	0.841145	None
15078	BC047626	-0.00195	0.585711	0.841145	None

15079	ARHGAP31	-0.02851	0.585713	0.841145	None
15080	DOCK9-AS	-0.00498	0.58579	0.841171	None
15081	EPHA1-AS	0.081388	0.585809	0.841171	None
15082	YWHAG	-0.04154	0.585892	0.841234	None
15083	LAS1L	-0.06597	0.585943	0.841251	None
15084	LSM14A	-0.05301	0.586168	0.841392	None
15085	EMILIN2	-0.07344	0.586176	0.841392	None
15086	LOC10192	-0.0049	0.58619	0.841392	None
15087	UBL4B	0.007689	0.5862	0.841392	None
15088	RBBP8NL	-0.02521	0.586355	0.841392	None
15089	TDRD1	-0.01627	0.586359	0.841392	None
15090	KRTAP4-1	-0.00132	0.586401	0.841392	None
15091	GGCX	-0.10684	0.586444	0.841392	None
15092	SLC41A1	-0.0561	0.58645	0.841392	None
15093	LOC73013	0.033948	0.586454	0.841392	None
15094	ALCAM	-0.11824	0.586474	0.841392	None
15095	HOTAIR	-0.00641	0.586507	0.841392	None
15096	NTHL1	-0.07703	0.586731	0.841657	None
15097	CCR8	-0.01055	0.586807	0.84171	None
15098	MFSD2A	0.05661	0.58685	0.841717	None
15099	SUDS3	-0.06953	0.586929	0.841774	None
15100	SCD	0.053765	0.587157	0.841938	None
15101	SS18L1	0.093346	0.587167	0.841938	None
15102	SYN2	-0.00303	0.587182	0.841938	None
15103	TCEANC2	-0.04088	0.587208	0.841938	None
15104	KIAA1045	0.010832	0.587238	0.841938	None
15105	AC079807.	0.002279	0.587312	0.841987	None
15106	PVRL4	-0.0234	0.587417	0.841987	None
15107	LCTL	-0.00178	0.58743	0.841987	None
15108	AC010145.	-0.00719	0.587456	0.841987	None
15109	LHX3	0.023107	0.587466	0.841987	None
15110	ST7L	-0.06899	0.587508	0.841992	None
15111	LOC72930	-0.00123	0.587668	0.842165	None
15112	TMEM145	-0.00305	0.587842	0.842321	None
15113	RP5-892K4	-0.0163	0.587855	0.842321	None
15114	GALNT15	0.050433	0.587927	0.842369	None
15115	TEKT3	-0.00377	0.588017	0.842442	None
15116	LOC10192	0.000693	0.58819	0.842496	None
15117	EIF4E2	0.077508	0.588255	0.842496	None
15118	ASPRV1	0.100009	0.588368	0.842496	None
15119	GJA10	-0.00391	0.588386	0.842496	None
15120	DYNC2H1	-0.04818	0.588389	0.842496	None
15121	LOC10192	0.018598	0.588391	0.842496	None
15122	LOC10013	-0.00324	0.588438	0.842496	None
15123	RP11-118C	-0.00303	0.588448	0.842496	None
15124	GHR	-0.08257	0.588454	0.842496	None
15125	RP11-490C	-0.00124	0.588493	0.842496	None
15126	ANP32D	-0.00753	0.58851	0.842496	None
15127	RP1-263J7	-0.00707	0.588595	0.842496	None
15128	PRR30	-0.00398	0.58861	0.842496	None
15129	ACVRL1	-0.0737	0.588644	0.842496	None
15130	BEX1	0.272693	0.588647	0.842496	None
15131	FBXO34	0.088212	0.588677	0.842496	None
15132	PAMR1	-0.00985	0.588735	0.842523	None
15133	SLC6A7	-0.00746	0.588846	0.842588	None
15134	MTMR10	-0.04627	0.588859	0.842588	None
15135	AP000473.	-0.04265	0.589061	0.842804	None
15136	RBMXL1	-0.09272	0.589124	0.842804	None

15137	CA5A	-0.04659	0.589149	0.842804	None
15138	RP11-727F	-0.01064	0.589165	0.842804	None
15139	RBM26	-0.10759	0.589231	0.842842	None
15140	TLDC1	-0.05393	0.589281	0.842857	None
15141	AC007349.	-0.00276	0.589319	0.842857	None
15142	LINC00052	-0.00128	0.589417	0.842926	None
15143	RAC1	-0.09613	0.589445	0.842926	None
15144	LOC15793	-0.00814	0.589493	0.842939	None
15145	CYP4F2	-0.12187	0.589675	0.843094	None
15146	SMC5-AS1	-0.01497	0.589679	0.843094	None
15147	RP1-135L2	-0.02496	0.589817	0.843184	None
15148	BC039319	0.011503	0.58982	0.843184	None
15149	ASPG	-0.00534	0.589985	0.843193	None
15150	TAF5	-0.08742	0.590039	0.843193	None
15151	RP11-96H1	-0.00335	0.590059	0.843193	None
15152	ING5	0.065638	0.590075	0.843193	None
15153	MIR622	-0.00367	0.590085	0.843193	None
15154	GPCPD1	0.087764	0.590102	0.843193	None
15155	LOC10012	-0.00737	0.590137	0.843193	None
15156	LINC01424	0.015885	0.590138	0.843193	None
15157	RAMP2	0.01732	0.590184	0.843203	None
15158	AC016999.	-0.01404	0.590471	0.843511	None
15159	SLC34A2	-0.01006	0.590477	0.843511	None
15160	TRIM45	-0.0901	0.590559	0.843572	None
15161	LOC10192	-0.002	0.590657	0.843627	None
15162	CST1	0.009349	0.590675	0.843627	None
15163	GCM1	0.005586	0.590842	0.843792	None
15164	LOC25357	-0.00183	0.590868	0.843792	None
15165	ADAM22	-0.00663	0.590937	0.843801	None
15166	GAP43	-0.00111	0.590973	0.843801	None
15167	ZNF84	-0.07613	0.590992	0.843801	None
15168	HTR1A	-0.01027	0.591109	0.843904	None
15169	U47924.27	-0.00781	0.591142	0.843904	None
15170	FAM163A	-0.00351	0.591229	0.843973	None
15171	OR11A1	-0.004	0.591313	0.844012	None
15172	C18orf61	-0.01669	0.591334	0.844012	None
15173	C2orf27A	-0.0476	0.59141	0.844036	None
15174	FOLR2	-0.15287	0.591472	0.844036	None
15175	LOC64262	-0.0055	0.591493	0.844036	None
15176	LOC10192	0.005954	0.591507	0.844036	None
15177	C15orf41	-0.03283	0.591612	0.84413	None
15178	FGF13-AS	-0.00152	0.591797	0.844339	None
15179	KLRC3	-0.03361	0.591889	0.844414	None
15180	NEUROG2	-0.00258	0.592036	0.844531	None
15181	DERL1	0.056874	0.592074	0.844531	None
15182	TPPP	-0.00526	0.592088	0.844531	None
15183	DSN1	0.10257	0.592227	0.844674	None
15184	BC038205	-0.01991	0.592332	0.844697	None
15185	LA16c-381	-0.00856	0.592357	0.844697	None
15186	CALHM3	-0.01101	0.592361	0.844697	None
15187	ZPLD1	-0.00105	0.592532	0.844885	None
15188	GABPB2	-0.0991	0.59257	0.844885	None
15189	LOC10192	-0.00242	0.592729	0.845055	None
15190	CASC22	-0.01233	0.592785	0.845079	None
15191	GTF3C4	-0.06277	0.592852	0.84512	None
15192	CFC1B	0.005132	0.592914	0.845152	None
15193	FGF20	-0.02302	0.593081	0.845334	None
15194	MID1	0.131201	0.593223	0.845363	None

15195	LOC28311:	-0.00786	0.593231	0.845363	None
15196	ART5	0.020689	0.593273	0.845363	None
15197	CHAC1	0.044567	0.593357	0.845363	None
15198	LOC254028	-0.00422	0.593363	0.845363	None
15199	FGGY	-0.06	0.593465	0.845363	None
15200	GGT7	-0.01225	0.593506	0.845363	None
15201	GNRH1	0.029751	0.593511	0.845363	None
15202	ITGB4	-0.00892	0.593513	0.845363	None
15203	KRT5	0.007165	0.593594	0.845363	None
15204	BCL2L15	-0.03445	0.593633	0.845363	None
15205	TP73	-0.00404	0.593647	0.845363	None
15206	TFDP1	-0.1557	0.593673	0.845363	None
15207	ERBB2	-0.00662	0.593827	0.845363	None
15208	CYP1A1	0.048986	0.593903	0.845363	None
15209	FXYD1	-0.00253	0.593924	0.845363	None
15210	ZNF136	-0.10345	0.593965	0.845363	None
15211	LOC101928	-0.18175	0.59402	0.845363	None
15212	STXBP5L	-0.00104	0.594053	0.845363	None
15213	LOC10050!	-0.08325	0.59407	0.845363	None
15214	IFNK	-0.00727	0.594104	0.845363	None
15215	VBP1	-0.04481	0.594104	0.845363	None
15216	EXOC2	-0.07165	0.594279	0.845363	None
15217	CISD2	0.136857	0.594403	0.845363	None
15218	CREB3L1	-0.0386	0.594435	0.845363	None
15219	SORCS2	0.004059	0.594442	0.845363	None
15220	F13B	-0.0048	0.594469	0.845363	None
15221	ZNF521	-0.15818	0.594504	0.845363	None
15222	DTWD1	-0.07322	0.594526	0.845363	None
15223	BTNL9	-0.06191	0.594527	0.845363	None
15224	RP4-714D!	-0.03787	0.594587	0.845363	None
15225	CHPT1	-0.10926	0.594597	0.845363	None
15226	COL6A1	-0.00099	0.5946	0.845363	None
15227	TBX18	-0.00416	0.594614	0.845363	None
15228	LOC10272:	-0.00364	0.594647	0.845363	None
15229	LINC00838	-0.00612	0.594667	0.845363	None
15230	LOC28619(	-0.00611	0.594667	0.845363	None
15231	STS	0.028246	0.594672	0.845363	None
15232	LOC10192!	-0.0032	0.594726	0.845363	None
15233	ANXA13	-0.00589	0.594727	0.845363	None
15234	KRT14	-0.01726	0.594734	0.845363	None
15235	RXFP2	-0.00149	0.594748	0.845363	None
15236	LOC10192!	-0.01231	0.594784	0.845363	None
15237	D21S2090E	-0.00523	0.594828	0.845363	None
15238	C2orf70	0.001268	0.594878	0.845363	None
15239	MGC27382	-0.00582	0.594905	0.845363	None
15240	LOC10012!	-0.03374	0.594936	0.845363	None
15241	C1QTNF3	0.054144	0.594979	0.84537	None
15242	POU5F1P4	-0.0037	0.595275	0.845734	None
15243	TCF24	0.0007	0.595509	0.84583	None
15244	LOC15176(	-0.0015	0.595513	0.84583	None
15245	1-Sep	0.080382	0.595553	0.84583	None
15246	AP1S2	-0.0953	0.595564	0.84583	None
15247	PAX6	-0.00738	0.595566	0.84583	None
15248	BCAN	0.002997	0.595622	0.84583	None
15249	GRIK2	0.000717	0.595714	0.84583	None
15250	RP11-272D	0.038904	0.595747	0.84583	None
15251	POLE4	-0.1137	0.595764	0.84583	None
15252	ICAM3	-0.11967	0.595792	0.84583	None

15253	SFTA1P	-0.0079	0.595802	0.84583	None
15254	NENF	-0.02656	0.595909	0.84583	None
15255	NPY5R	-0.0032	0.595995	0.84583	None
15256	BC047644	-0.00524	0.596017	0.84583	None
15257	SCN4A	-0.01309	0.596047	0.84583	None
15258	FOXJ3	0.031596	0.596074	0.84583	None
15259	SMC1B	0.009156	0.59609	0.84583	None
15260	COL4A6	-0.00828	0.596205	0.84583	None
15261	RP1-178F1	-0.00676	0.596208	0.84583	None
15262	FLJ30403	-0.00669	0.596208	0.84583	None
15263	PKHD1L1	-0.09476	0.596238	0.84583	None
15264	DQ576800	-0.00714	0.596306	0.84583	None
15265	CLEC4GP1	0.017232	0.596447	0.84583	None
15266	R3HDM1	-0.04384	0.596505	0.84583	None
15267	RP11-116D	-0.0022	0.596553	0.84583	None
15268	SLC22A6	-0.00686	0.596604	0.84583	None
15269	ZNF281	-0.0499	0.596612	0.84583	None
15270	MICAL1	0.139705	0.596697	0.84583	None
15271	KRMP1	-0.03671	0.596714	0.84583	None
15272	EFNB1	-0.01355	0.596742	0.84583	None
15273	LOC10192	-0.0012	0.596787	0.84583	None
15274	GABARAPL	-0.01632	0.59685	0.84583	None
15275	RP11-161D	-0.00156	0.596893	0.84583	None
15276	KRT84	-0.00398	0.59691	0.84583	None
15277	LOC10192	-0.00409	0.596933	0.84583	None
15278	KCNIP4	0.007172	0.59695	0.84583	None
15279	TUSC7	-0.00776	0.596981	0.84583	None
15280	KIF2B	-0.00699	0.596998	0.84583	None
15281	VSTM4	-0.01447	0.597094	0.84583	None
15282	LOC100128	-0.00281	0.597174	0.84583	None
15283	KIF20B	-0.07625	0.597188	0.84583	None
15284	STK16	-0.03958	0.597221	0.84583	None
15285	GREB1	-0.00315	0.597236	0.84583	None
15286	ATAD3A	-0.07381	0.597296	0.84583	None
15287	WFIKKN1	-0.01137	0.597331	0.84583	None
15288	KARS	-0.04233	0.597408	0.84583	None
15289	ABCA12	-0.00602	0.597485	0.84583	None
15290	C2CD4C	0.010085	0.597517	0.84583	None
15291	LOC10192	-0.00266	0.597561	0.84583	None
15292	MRGPRF	0.016058	0.597564	0.84583	None
15293	NDUFS6	0.074206	0.597572	0.84583	None
15294	ZNF750	-0.00285	0.597578	0.84583	None
15295	PPIL6	0.022391	0.597583	0.84583	None
15296	TMEM205	-0.08903	0.597603	0.84583	None
15297	ARHGAP42	-0.00539	0.597603	0.84583	None
15298	PANK3	0.04444	0.597671	0.84583	None
15299	HEBP2	-0.04057	0.597678	0.84583	None
15300	FRMPD1	-0.00725	0.59768	0.84583	None
15301	LOC28343	-0.00171	0.597689	0.84583	None
15302	SRGAP3	-0.02488	0.597704	0.84583	None
15303	CKLF	-0.0578	0.597735	0.84583	None
15304	LOC10192	-0.00391	0.597764	0.84583	None
15305	CHODL	-0.01083	0.597845	0.845883	None
15306	FCHO1	-0.04388	0.597934	0.845883	None
15307	TRIM54	-0.00593	0.597944	0.845883	None
15308	NDUFA10	-0.01543	0.597958	0.845883	None
15309	LINC00271	0.02809	0.598058	0.845903	None
15310	LOC10050	-0.00384	0.598092	0.845903	None

15311	RP11-521I	-0.02593	0.598096	0.845903	None
15312	CTD-2587I	-0.00954	0.598129	0.845903	None
15313	SFPQ	-0.07716	0.598352	0.845996	None
15314	DNALI1	-0.00432	0.598369	0.845996	None
15315	EVA1A	-0.00132	0.598401	0.845996	None
15316	LOC28618	-0.0029	0.598431	0.845996	None
15317	PLA1A	-0.00685	0.598477	0.845996	None
15318	TMEM132	0.023127	0.598489	0.845996	None
15319	FLJ31945	-0.00347	0.598491	0.845996	None
15320	AK056098	-0.00551	0.598515	0.845996	None
15321	MTFR2	0.125294	0.598545	0.845996	None
15322	EFCAB13	-0.00598	0.598696	0.846109	None
15323	HKDC1	0.00476	0.598704	0.846109	None
15324	LOC44070	-0.00307	0.598764	0.846139	None
15325	DRD1	-0.00316	0.598846	0.8462	None
15326	GUK1	-0.04535	0.598921	0.846232	None
15327	TEF	-0.01058	0.598952	0.846232	None
15328	OGN	0.013938	0.598986	0.846232	None
15329	KCNQ5	0.066465	0.599185	0.846369	None
15330	MYH15	-0.00189	0.599194	0.846369	None
15331	ZNF224	-0.10394	0.5992	0.846369	None
15332	L1TD1	-0.02199	0.599276	0.846421	None
15333	NOP16	-0.04688	0.59936	0.846452	None
15334	C12orf75	0.151722	0.599376	0.846452	None
15335	PCDHGB6	-0.00687	0.599442	0.84649	None
15336	OPHN1	-0.07995	0.599587	0.846639	None
15337	LINC00877	0.014938	0.599668	0.846653	None
15338	RPS6KA5	0.104719	0.599734	0.846653	None
15339	LINC00514	-0.00663	0.599741	0.846653	None
15340	PRSS27	-0.00301	0.599753	0.846653	None
15341	DCTN1-AS	-0.00481	0.599815	0.846685	None
15342	LMX1A	-0.0072	0.599862	0.846696	None
15343	LOC10192	-0.00197	0.599961	0.846754	None
15344	MIER3	-0.11958	0.600014	0.846754	None
15345	NDST4	-0.04459	0.600047	0.846754	None
15346	EMB	0.078849	0.600059	0.846754	None
15347	AK024936	-0.05134	0.6002	0.846772	None
15348	ITGA9-AS1	-0.02217	0.600235	0.846772	None
15349	XKR7	-0.0022	0.600302	0.846772	None
15350	PTGES3L	-0.01449	0.600314	0.846772	None
15351	SLC45A1	-0.0196	0.600353	0.846772	None
15352	SDF2L1	-0.12736	0.600356	0.846772	None
15353	ALG13	-0.10804	0.600365	0.846772	None
15354	KB-1410C	-0.00321	0.600414	0.846772	None
15355	LSM2	-0.13638	0.600468	0.846772	None
15356	CTD-2537I	-0.01722	0.600469	0.846772	None
15357	PHIP	0.095422	0.600537	0.846772	None
15358	MC4R	-0.00277	0.600611	0.846772	None
15359	DSP	-0.01347	0.600614	0.846772	None
15360	CYP3A4	0.004472	0.600619	0.846772	None
15361	LOC37519	0.017318	0.600782	0.846946	None
15362	KRTAP11-	-0.00119	0.600918	0.847083	None
15363	SLC39A12	-0.01956	0.601061	0.847228	None
15364	MGC12488	0.11541	0.601116	0.847228	None
15365	LOC10192	-0.00985	0.601139	0.847228	None
15366	C17orf82	-0.00771	0.601233	0.847254	None
15367	ABCA9	-0.0116	0.601316	0.847254	None
15368	PLG	0.001428	0.601356	0.847254	None

15369	KLHL41	0.005157	0.601375	0.847254	None
15370	BC040734	-0.00966	0.601393	0.847254	None
15371	RP11-506N	-0.00221	0.601473	0.847254	None
15372	CACNA2D1	-0.03769	0.601479	0.847254	None
15373	TRPC4AP	-0.06951	0.601507	0.847254	None
15374	AA06	0.02809	0.601573	0.847254	None
15375	KIAA1644	-0.00283	0.601618	0.847254	None
15376	COX6A2	-0.00287	0.60162	0.847254	None
15377	AK096159	-0.01459	0.601701	0.847254	None
15378	NDUFS7	-0.05331	0.601704	0.847254	None
15379	RDH10	-0.04333	0.601757	0.847254	None
15380	PRCP	-0.03212	0.601791	0.847254	None
15381	SLC26A11	-0.1312	0.601805	0.847254	None
15382	OR5P3	-0.00684	0.601899	0.847254	None
15383	BCAS1	-0.03844	0.601986	0.847254	None
15384	EAF1	0.078444	0.602021	0.847254	None
15385	ZNF295-AS	-0.01807	0.602031	0.847254	None
15386	SEL1L2	-0.00203	0.602041	0.847254	None
15387	LOC728445	-0.00814	0.602048	0.847254	None
15388	G0S2	0.314092	0.602089	0.847254	None
15389	LOC10050	-0.00114	0.602117	0.847254	None
15390	CPNE3	-0.07364	0.602172	0.847254	None
15391	OAS3	-0.09792	0.602191	0.847254	None
15392	CHIT1	0.048048	0.602223	0.847254	None
15393	C22orf24	-0.00569	0.602252	0.847254	None
15394	GNG13	-0.00132	0.60248	0.847473	None
15395	OR5K1	-0.00258	0.60251	0.847473	None
15396	NKD2	-0.03265	0.602526	0.847473	None
15397	LOC10192	0.016319	0.60269	0.847649	None
15398	NEIL3	0.152526	0.602795	0.847714	None
15399	PARD6B	-0.02028	0.602814	0.847714	None
15400	PFDN5	-0.09282	0.603056	0.847963	None
15401	OPN1SW	-0.00428	0.60311	0.847963	None
15402	CPEB3	-0.03447	0.603154	0.847963	None
15403	CST13P	-0.00526	0.603166	0.847963	None
15404	BST2	0.154546	0.603187	0.847963	None
15405	GRM1	-0.00366	0.603319	0.848055	None
15406	PAPOLG	-0.07758	0.60351	0.848055	None
15407	RPRM	-0.00377	0.603554	0.848055	None
15408	CTCF	-0.05814	0.603623	0.848055	None
15409	LOC10192	0.048398	0.603643	0.848055	None
15410	LOC10192	-0.00249	0.603688	0.848055	None
15411	C21orf119	-0.06592	0.603702	0.848055	None
15412	TMEM256	-0.12176	0.603711	0.848055	None
15413	C7orf61	-0.0466	0.603788	0.848055	None
15414	PCDHGC3	-0.00547	0.603816	0.848055	None
15415	MAPT	-0.00936	0.603847	0.848055	None
15416	FBXL3	0.039506	0.603887	0.848055	None
15417	SLITRK6	-0.0866	0.6039	0.848055	None
15418	GDNF	0.006437	0.603902	0.848055	None
15419	KDF1	-0.00438	0.603943	0.848055	None
15420	USHBP1	-0.02515	0.603948	0.848055	None
15421	PF4V1	-0.29732	0.603955	0.848055	None
15422	KRT79	0.015962	0.604038	0.848055	None
15423	HHIPL1	-0.00242	0.604048	0.848055	None
15424	BRDT	-0.00136	0.604079	0.848055	None
15425	LINC00323	-0.00324	0.604091	0.848055	None
15426	CCDC42B	-0.02345	0.604114	0.848055	None

15427	FGB	-0.00867	0.604286	0.848168	None
15428	CDCA4	0.078662	0.604311	0.848168	None
15429	RP11-414H	0.002282	0.604312	0.848168	None
15430	RP11-214N	-0.01286	0.604372	0.848196	None
15431	DCDC2B	0.019841	0.604466	0.848274	None
15432	CPEB2-AS1	-0.00744	0.604593	0.848277	None
15433	HELZ2	-0.00715	0.604614	0.848277	None
15434	CLEC4E	0.070145	0.604639	0.848277	None
15435	KCNK7	-0.00519	0.604666	0.848277	None
15436	NUP210L	-0.01738	0.60468	0.848277	None
15437	LINC00926	0.091222	0.604763	0.848277	None
15438	PI3	-0.04721	0.604798	0.848277	None
15439	LEPROTL1	0.065052	0.604805	0.848277	None
15440	LOC100507	-0.00245	0.604823	0.848277	None
15441	RP11-255A	-0.00153	0.604917	0.848277	None
15442	MAP4	-0.05389	0.604977	0.848277	None
15443	KLK14	-0.01952	0.604984	0.848277	None
15444	CYP7A1	-0.00294	0.605008	0.848277	None
15445	RP11-194N	-0.04177	0.605017	0.848277	None
15446	SUMO4	0.102227	0.605096	0.848333	None
15447	OR10A5	-0.0043	0.605198	0.848358	None
15448	RAX	-0.00774	0.605237	0.848358	None
15449	CREBRF	-0.10937	0.605252	0.848358	None
15450	ARPP19	0.073594	0.605271	0.848358	None
15451	CLDND1	0.046735	0.605482	0.848553	None
15452	NKAPL	0.013251	0.605488	0.848553	None
15453	FAM19A1	-0.00486	0.605703	0.848725	None
15454	GPBP1	-0.04054	0.605718	0.848725	None
15455	SRRD	0.098389	0.605728	0.848725	None
15456	NRN1	0.070815	0.605784	0.848748	None
15457	SSR4	-0.06865	0.605824	0.848749	None
15458	TRPM6	-0.00646	0.605977	0.84884	None
15459	C5orf49	-0.00732	0.606014	0.84884	None
15460	LOC100500	-0.0034	0.606043	0.84884	None
15461	CPB1	-0.03925	0.606045	0.84884	None
15462	GABRR3	-0.00809	0.606089	0.848847	None
15463	RP11-439E	-0.00368	0.606213	0.848964	None
15464	ACOX2	-0.09347	0.606361	0.849117	None
15465	MEX3D	-0.00834	0.606544	0.849285	None
15466	DYDC2	-0.00348	0.60656	0.849285	None
15467	GLA	0.072807	0.606683	0.849404	None
15468	LOC101928	0.005495	0.606861	0.849447	None
15469	SNAI3-AS1	-0.01098	0.606867	0.849447	None
15470	BC045805	-0.00366	0.606882	0.849447	None
15471	CASC14	-0.04369	0.606924	0.849447	None
15472	LOC101927	0.002596	0.606997	0.849447	None
15473	LA16c-395	-0.00205	0.607017	0.849447	None
15474	CTD-2553C	0.021495	0.607019	0.849447	None
15475	AQP6	0.012307	0.607028	0.849447	None
15476	SEMA3E	0.011903	0.607081	0.849448	None
15477	NUDT12	0.067143	0.607107	0.849448	None
15478	SPTSSA	-0.06992	0.607268	0.849617	None
15479	KIAA0754	-0.07925	0.607364	0.849617	None
15480	PSMC4	-0.08782	0.607366	0.849617	None
15481	FIBCD1	0.033542	0.607447	0.849617	None
15482	GNG4	-0.00431	0.607528	0.849617	None
15483	PGLS	0.05392	0.60757	0.849617	None
15484	ZBED5-AS1	-0.10442	0.607628	0.849617	None

15485	DRG2	-0.07793	0.607634	0.849617	None
15486	POR	-0.11631	0.607637	0.849617	None
15487	WDR86	-0.01426	0.60767	0.849617	None
15488	SCNN1D	-0.01167	0.607692	0.849617	None
15489	PCSK1	-0.00753	0.607722	0.849617	None
15490	STK32C	0.076723	0.607757	0.849617	None
15491	TP73-AS1	-0.02432	0.607777	0.849617	None
15492	KDM3B	-0.09408	0.607871	0.849687	None
15493	PARN	-0.05503	0.607906	0.849687	None
15494	RP11-874J	-0.08081	0.607975	0.849729	None
15495	AZGP1	-0.02112	0.608098	0.849846	None
15496	KCNJ4	-0.00959	0.608218	0.849958	None
15497	DNM1P35	-0.00654	0.608369	0.85004	None
15498	S100P	-0.2381	0.608415	0.85004	None
15499	TMEM101	-0.08446	0.608433	0.85004	None
15500	CDR2L	-0.02963	0.608435	0.85004	None
15501	LOC10192	-0.00914	0.60851	0.85004	None
15502	LOC10050	-0.00319	0.608583	0.85004	None
15503	NCF1	-0.1761	0.608636	0.85004	None
15504	C17orf74	-0.00168	0.608666	0.85004	None
15505	P2RX2	0.000989	0.608719	0.85004	None
15506	CASC11	-0.0014	0.608725	0.85004	None
15507	LINC00028	-0.01975	0.608729	0.85004	None
15508	MICALCL	-0.04573	0.608747	0.85004	None
15509	ZNF25	-0.07898	0.60879	0.850044	None
15510	LOC72987	-0.03943	0.608861	0.85009	None
15511	CLDN3	-0.00596	0.608924	0.850122	None
15512	GRIK3	-0.00526	0.609079	0.850276	None
15513	C8orf44	-0.11191	0.609148	0.850276	None
15514	PTPN2	-0.03398	0.609181	0.850276	None
15515	ETV5	-0.07279	0.609191	0.850276	None
15516	BCAM	0.012774	0.609245	0.850297	None
15517	ASB13	-0.07492	0.609384	0.850436	None
15518	LINC00705	-0.00237	0.609427	0.850441	None
15519	ADSL	-0.05957	0.609594	0.850511	None
15520	RP11-804H	-0.08936	0.609651	0.850511	None
15521	PASD1	-0.00453	0.609734	0.850511	None
15522	ZNF43	0.094642	0.609752	0.850511	None
15523	SHQ1	-0.09275	0.609771	0.850511	None
15524	ENTPD2	-0.00777	0.609779	0.850511	None
15525	BEND5	-0.05901	0.609781	0.850511	None
15526	LOC10192	-0.00434	0.609793	0.850511	None
15527	LOC10192	0.034713	0.609831	0.850511	None
15528	DMRTB1	-0.00996	0.609954	0.850539	None
15529	XPO1	0.067027	0.610001	0.850539	None
15530	LOC10050	-0.01124	0.610008	0.850539	None
15531	CCNT2-AS	-0.02324	0.610056	0.850539	None
15532	HID1	0.014846	0.610079	0.850539	None
15533	RP13-238F	-0.00175	0.610086	0.850539	None
15534	NPNT	-0.00268	0.610206	0.850558	None
15535	MPHOSPH	-0.07338	0.610213	0.850558	None
15536	EPPK1	-0.00351	0.610236	0.850558	None
15537	PLIN4	0.042635	0.610257	0.850558	None
15538	ISLR	-0.01462	0.61033	0.850605	None
15539	EMX2	-0.00197	0.610475	0.850706	None
15540	LOC10050	0.009691	0.610481	0.850706	None
15541	PROSER2-,	-0.0064	0.610562	0.85072	None
15542	LOC10192	-0.00637	0.610597	0.85072	None

15543	TCTN1	0.042474	0.610609	0.85072	None
15544	AQP1	-0.10812	0.610782	0.850907	None
15545	TGFA	-0.01294	0.611101	0.851113	None
15546	GPIHBP1	-0.01076	0.611112	0.851113	None
15547	CTNNA2	-0.01251	0.61112	0.851113	None
15548	OR51B5	-0.00271	0.611121	0.851113	None
15549	LINC00648	-0.05062	0.611143	0.851113	None
15550	PLEKHF2	-0.07638	0.61119	0.851113	None
15551	EEPD1	0.018872	0.611206	0.851113	None
15552	GPR143	-0.00392	0.611408	0.851194	None
15553	OR7E14P	-0.0073	0.611431	0.851194	None
15554	DNAH3	-0.00131	0.611455	0.851194	None
15555	MAPK9	0.042923	0.611501	0.851194	None
15556	RING1	-0.05764	0.611518	0.851194	None
15557	NLRP8	-0.00304	0.611529	0.851194	None
15558	MB	0.035109	0.611539	0.851194	None
15559	DLG1-AS1	-0.00285	0.611682	0.851311	None
15560	MBD4	-0.04173	0.611702	0.851311	None
15561	LIM2	-0.00763	0.611872	0.851424	None
15562	ZBTB20	-0.11188	0.611883	0.851424	None
15563	DENND4A	0.087491	0.611901	0.851424	None
15564	WFDC9	0.014263	0.612135	0.851696	None
15565	FOXL2	-0.01296	0.612244	0.851792	None
15566	PLCH1	-0.0079	0.612343	0.851813	None
15567	HIST1H2BN	-0.01044	0.612362	0.851813	None
15568	RGS4	-0.00119	0.612383	0.851813	None
15569	LIPH	-0.03928	0.612432	0.851813	None
15570	RP11-124L	0.11353	0.612528	0.851813	None
15571	RP3-388M	-0.00209	0.612533	0.851813	None
15572	TWSG1	-0.0644	0.612535	0.851813	None
15573	APC2	-0.00814	0.612591	0.851837	None
15574	EMG1	-0.0757	0.612665	0.851885	None
15575	BHLHE23	-0.00497	0.612792	0.851924	None
15576	CDKL3	-0.0382	0.612799	0.851924	None
15577	ITGBL1	-0.05698	0.612811	0.851924	None
15578	GJC1	0.000806	0.612861	0.851939	None
15579	FAM83F	0.037934	0.613007	0.852054	None
15580	ZNF541	0.019605	0.613048	0.852054	None
15581	RUNDC3A	-0.07259	0.6131	0.852054	None
15582	RAP1GAP	-0.0299	0.613101	0.852054	None
15583	ECE1	-0.05723	0.613328	0.852223	None
15584	ZNF551	0.030066	0.613339	0.852223	None
15585	MYL6	0.051733	0.613341	0.852223	None
15586	SNORA71A	-0.00522	0.613465	0.852308	None
15587	RP11-809F	-0.00256	0.613587	0.852308	None
15588	LOC283387	-0.00611	0.613607	0.852308	None
15589	SNAP91	-0.00116	0.613626	0.852308	None
15590	AGA	0.094447	0.613626	0.852308	None
15591	KIF5C	0.030158	0.613642	0.852308	None
15592	NIPAL4	0.059	0.613683	0.852308	None
15593	RGL3	0.006249	0.613748	0.852308	None
15594	RP11-161I	-0.02629	0.613756	0.852308	None
15595	SYF2	0.043142	0.613834	0.852362	None
15596	DKFZp434J	-0.00392	0.613892	0.852387	None
15597	BNIPL	-0.00282	0.613983	0.852437	None
15598	AX746710	-0.00267	0.614007	0.852437	None
15599	BC042374	-0.00423	0.614192	0.85264	None
15600	NUTM2A-A	-0.10936	0.614273	0.852654	None

15601	SLC9A7	0.085929	0.614281	0.852654	None
15602	MTHFD1	-0.06861	0.614327	0.852663	None
15603	HFE2	0.017676	0.614389	0.852695	None
15604	IL17RE	-0.014	0.614624	0.852966	None
15605	MUM1L1	-0.00598	0.61471	0.85303	None
15606	GNN	-0.00111	0.614797	0.853097	None
15607	PSMC2	-0.06794	0.614867	0.853139	None
15608	RBBP4	0.05863	0.614906	0.853139	None
15609	LOC10050!	-0.00121	0.614964	0.853164	None
15610	KLRC4	-0.00454	0.615042	0.853168	None
15611	GADD45GI	-0.00178	0.615081	0.853168	None
15612	CTC-550B1	0.013398	0.615095	0.853168	None
15613	LOC10192!	-0.00377	0.615128	0.853168	None
15614	PTPRC	-0.1232	0.615222	0.853168	None
15615	LOC64373:	-0.03476	0.615275	0.853168	None
15616	LINC00615	-0.00632	0.615339	0.853168	None
15617	POTEKP	-0.09318	0.615379	0.853168	None
15618	LOC10272	0.054787	0.615388	0.853168	None
15619	LOC40076	0.008831	0.615393	0.853168	None
15620	LOC34001	-0.00091	0.6154	0.853168	None
15621	AMBP	-0.00374	0.615462	0.853175	None
15622	LOC33929	-0.00628	0.615548	0.853175	None
15623	KCND3	-0.00505	0.615631	0.853175	None
15624	CNOT2	-0.0474	0.615632	0.853175	None
15625	MMP21	-0.01143	0.615634	0.853175	None
15626	NHEG1	-0.00807	0.615663	0.853175	None
15627	TAS2R4	-0.00348	0.615681	0.853175	None
15628	C5AR2	-0.02451	0.615746	0.85319	None
15629	RP11-1L12	-0.00498	0.615777	0.85319	None
15630	LOC33997	-0.00216	0.61581	0.85319	None
15631	PACRG-AS	-0.00452	0.615852	0.853193	None
15632	ODF1	0.016569	0.615927	0.853243	None
15633	PALM2	-0.03749	0.616006	0.853298	None
15634	C22orf15	-0.01774	0.616067	0.853328	None
15635	FBLN7	-0.02962	0.616162	0.85335	None
15636	DRD3	-0.0014	0.616219	0.85335	None
15637	TUSC5	-0.02852	0.616238	0.85335	None
15638	IFI35	-0.13357	0.61624	0.85335	None
15639	ACD	-0.08367	0.616284	0.853356	None
15640	TOPBP1	0.062228	0.616393	0.853452	None
15641	PDLIM3	-0.00109	0.616475	0.853476	None
15642	CFHR5	-0.00523	0.616489	0.853476	None
15643	MGC4859	-0.00127	0.616569	0.853524	None
15644	LOC10050	-0.00203	0.616602	0.853524	None
15645	MAP7D2	0.169196	0.616711	0.853563	None
15646	RP3-497J2	-0.0071	0.616727	0.853563	None
15647	MAGEA10	-0.00239	0.616749	0.853563	None
15648	RP1-228H1	0.048694	0.61687	0.853675	None
15649	MCTP2	0.120665	0.616936	0.853713	None
15650	MGAT4A	-0.07765	0.617019	0.853773	None
15651	GABRR1	-0.00127	0.617176	0.853936	None
15652	EIF2B4	-0.06706	0.617258	0.853995	None
15653	INSM1	-0.02	0.617476	0.854241	None
15654	RP11-736K	-0.00449	0.617523	0.854251	None
15655	LOC10012!	-0.14473	0.617643	0.854363	None
15656	IKZF3	-0.0284	0.617683	0.854364	None
15657	GYS2	-0.00275	0.617749	0.854386	None
15658	TGM4	-0.00678	0.617797	0.854386	None

15659	RP5-1184F	0.013728	0.617817	0.854386	None
15660	FOXG1	-0.00487	0.618095	0.854715	None
15661	VEPH1	-0.03201	0.618138	0.854721	None
15662	SPOCK2	0.057006	0.618203	0.854756	None
15663	EPG5	-0.06554	0.618301	0.854821	None
15664	GK	-0.05504	0.61839	0.854821	None
15665	RFX4	-0.00256	0.618399	0.854821	None
15666	DCAF4L2	-0.00708	0.618408	0.854821	None
15667	EIF3C	0.120558	0.618479	0.854865	None
15668	POLA1	0.074163	0.618586	0.854895	None
15669	LINC00162	-0.00563	0.618627	0.854895	None
15670	RP11-339A	-0.00295	0.618642	0.854895	None
15671	AP1S3	-0.00952	0.618677	0.854895	None
15672	GPX1	-0.08288	0.618698	0.854895	None
15673	SCRG1	0.001628	0.618785	0.854959	None
15674	S100A10	0.153132	0.618862	0.854959	None
15675	DBI	-0.05703	0.618863	0.854959	None
15676	GM2A	-0.06239	0.618954	0.854989	None
15677	LRRD1	0.006334	0.618964	0.854989	None
15678	AMELX	0.00331	0.619065	0.854994	None
15679	PIK3R4	-0.02187	0.619097	0.854994	None
15680	OR1G1	-0.001	0.619138	0.854994	None
15681	LOC142937	0.126446	0.619266	0.854994	None
15682	CHRNA1	-0.02165	0.619286	0.854994	None
15683	BC021061	-0.00099	0.619292	0.854994	None
15684	GJA8	0.010084	0.619315	0.854994	None
15685	CFTR	-0.00175	0.619339	0.854994	None
15686	CAPN13	-0.00236	0.61939	0.854994	None
15687	OR2C1	-0.00339	0.619398	0.854994	None
15688	ALKBH7	-0.06771	0.619402	0.854994	None
15689	INHBA-AS	0.007875	0.619511	0.855071	None
15690	KAAG1	-0.0017	0.619549	0.855071	None
15691	ROPN1B	-0.01279	0.619576	0.855071	None
15692	N4BP2L2	-0.06653	0.619616	0.855072	None
15693	AC008753.	0.042358	0.619856	0.855349	None
15694	GPR110	-0.02741	0.61997	0.855406	None
15695	DOCK2	0.034202	0.62	0.855406	None
15696	BTN2A2	-0.05103	0.620033	0.855406	None
15697	LOC10106(	-0.00949	0.620056	0.855406	None
15698	DNM3OS	-0.00942	0.6201	0.855412	None
15699	TMEM43	-0.06616	0.620275	0.855552	None
15700	FAM35A	-0.04967	0.62028	0.855552	None
15701	OXER1	-0.02151	0.620349	0.855568	None
15702	SAMD4B	-0.0343	0.620405	0.855568	None
15703	LPPR5	-0.00653	0.620411	0.855568	None
15704	LOC10192ε	-0.00151	0.62048	0.855609	None
15705	UBE2Q2	0.094119	0.620596	0.855716	None
15706	OR1D2	-0.00976	0.620696	0.855763	None
15707	CHRNA4	-0.00087	0.620742	0.855763	None
15708	BTK	-0.07798	0.620768	0.855763	None
15709	ANHX	0.013081	0.620894	0.855763	None
15710	LOC10192T	-0.00597	0.620911	0.855763	None
15711	SDR42E1	-0.06997	0.620946	0.855763	None
15712	TMCC2	-0.0708	0.620979	0.855763	None
15713	DSCAM	-0.01047	0.621005	0.855763	None
15714	SELT	0.073159	0.621019	0.855763	None
15715	KIAA0319	-0.00295	0.621098	0.855763	None
15716	SVOPL	-0.10038	0.621132	0.855763	None

15717	ZNF7	-0.01447	0.621144	0.855763	None
15718	ZNF837	-0.01201	0.621187	0.855763	None
15719	GPER1	-0.02136	0.621195	0.855763	None
15720	MKX	-0.00149	0.621223	0.855763	None
15721	MOXD1	0.020299	0.621263	0.855764	None
15722	NUDT13	-0.07123	0.621351	0.855791	None
15723	LGR5	-0.01525	0.621379	0.855791	None
15724	DSCR10	-0.00611	0.621426	0.855791	None
15725	RPL34-AS1	-0.00761	0.621458	0.855791	None
15726	OLFML1	-0.00187	0.621519	0.855791	None
15727	RP13-379(	0.006177	0.621521	0.855791	None
15728	WIPI2	-0.07014	0.621677	0.855801	None
15729	PMFBP1	-0.03836	0.621683	0.855801	None
15730	RP11-330N	-0.00212	0.621757	0.855801	None
15731	AC005606.	-0.00382	0.621864	0.855801	None
15732	LOC10192!	-0.00746	0.622016	0.855801	None
15733	RP11-893F	0.002393	0.622079	0.855801	None
15734	LRTM2	-0.00762	0.622107	0.855801	None
15735	TINCR	-0.00826	0.62211	0.855801	None
15736	FSD2	-0.00127	0.622171	0.855801	None
15737	LOC28463:	-0.0058	0.622181	0.855801	None
15738	PARP6	0.053578	0.622193	0.855801	None
15739	AP1M2	-0.00084	0.622198	0.855801	None
15740	LRRC8E	-0.02071	0.622225	0.855801	None
15741	PODNL1	0.024388	0.622253	0.855801	None
15742	ZNF560	-0.02362	0.622284	0.855801	None
15743	FLJ32790	-0.00356	0.622328	0.855801	None
15744	NLRP2	0.145723	0.622333	0.855801	None
15745	TNXB	-0.00156	0.622338	0.855801	None
15746	HAUS1	0.072753	0.622449	0.855801	None
15747	LOC40054:	0.004472	0.62247	0.855801	None
15748	SH3BP5	0.184127	0.622472	0.855801	None
15749	LOC10272!	-0.0015	0.622499	0.855801	None
15750	EPS8L2	-0.00517	0.622571	0.855801	None
15751	MAOB	-0.00093	0.622609	0.855801	None
15752	XXbac-B47	-0.01103	0.622698	0.855801	None
15753	RERE	-0.00744	0.622703	0.855801	None
15754	MFAP1	-0.07355	0.622769	0.855801	None
15755	LOC10192	-0.00113	0.622844	0.855801	None
15756	ZNF528	-0.04953	0.622868	0.855801	None
15757	REXO1L1P	-0.00203	0.622949	0.855801	None
15758	GSG1	-0.00087	0.622955	0.855801	None
15759	ASB5	-0.00115	0.622969	0.855801	None
15760	SBSPON	-0.00097	0.623032	0.855801	None
15761	LOC10192!	-0.00275	0.623038	0.855801	None
15762	CCDC90B	0.086804	0.623047	0.855801	None
15763	F9	-0.00125	0.623051	0.855801	None
15764	RRBP1	-0.02108	0.623082	0.855801	None
15765	LINC00889	-0.02026	0.623099	0.855801	None
15766	HLX	-0.17523	0.623135	0.855801	None
15767	CBY3	-0.00252	0.623157	0.855801	None
15768	NT5DC4	-0.0011	0.623161	0.855801	None
15769	IPO8	-0.03175	0.623252	0.855801	None
15770	IFT43	-0.06427	0.623261	0.855801	None
15771	RICTOR	-0.09004	0.623266	0.855801	None
15772	SETD1A	-0.05166	0.623424	0.855963	None
15773	PIK3CB	-0.07844	0.623464	0.855963	None
15774	AAMP	0.085946	0.623648	0.856145	None

15775	ZNF571	-0.10009	0.623675	0.856145	None
15776	BTBD1	-0.03791	0.62382	0.856282	None
15777	TEX9	0.034614	0.623872	0.856282	None
15778	RAB3IP	-0.04378	0.623893	0.856282	None
15779	CCR5	0.100144	0.623976	0.85634	None
15780	SPAG16	-0.00962	0.624081	0.85643	None
15781	SH3D21	0.091855	0.62416	0.856455	None
15782	PXK	-0.08417	0.624178	0.856455	None
15783	ETS2	0.091295	0.624292	0.85646	None
15784	EBF3	-0.04268	0.624314	0.85646	None
15785	ZNF568	-0.02196	0.624368	0.85646	None
15786	CENPO	0.074956	0.624382	0.85646	None
15787	PSMB8-AS	0.043906	0.624396	0.85646	None
15788	MSANTD4	0.019247	0.624419	0.85646	None
15789	C3orf80	-0.17601	0.624473	0.85648	None
15790	C6orf25	-0.00405	0.624513	0.85648	None
15791	DYNC1I1	-0.02417	0.62459	0.85651	None
15792	PRDM9	0.009737	0.624687	0.85651	None
15793	SDE2	0.09913	0.624714	0.85651	None
15794	VENTX	-0.04918	0.624718	0.85651	None
15795	SCGB2A2	-0.0089	0.62483	0.85651	None
15796	SEC23B	0.05999	0.624847	0.85651	None
15797	TCTN2	-0.02626	0.62486	0.85651	None
15798	BTF3L4	0.045121	0.62491	0.85651	None
15799	ERV9-1	0.015695	0.624924	0.85651	None
15800	LOC65484	-0.002	0.62493	0.85651	None
15801	OR2F1	0.013842	0.625118	0.856638	None
15802	LOXL2	-0.00092	0.625156	0.856638	None
15803	IL20RA	-0.0023	0.625217	0.856638	None
15804	AADACP1	-0.00726	0.625218	0.856638	None
15805	IGH	0.025796	0.625221	0.856638	None
15806	DPP8	0.045603	0.625261	0.856639	None
15807	PPP1R1B	-0.00414	0.625345	0.856687	None
15808	IDI2	-0.00237	0.625375	0.856687	None
15809	EIF1AD	-0.06157	0.625425	0.8567	None
15810	LOC10272	-0.00092	0.625472	0.856711	None
15811	MOS	-0.00315	0.625571	0.856792	None
15812	JPH3	-0.00151	0.625722	0.856878	None
15813	WDR38	-0.00092	0.625727	0.856878	None
15814	LINC00545	-0.00207	0.625752	0.856878	None
15815	CXCL8	0.279106	0.625928	0.857024	None
15816	SYT17	-0.01132	0.625938	0.857024	None
15817	FZD3	-0.16742	0.626129	0.857191	None
15818	UBE2DNL	-0.00299	0.62614	0.857191	None
15819	FAM155A	-0.00205	0.626229	0.857259	None
15820	ADM	0.196858	0.626302	0.857305	None
15821	LINC01097	-0.00654	0.626411	0.857326	None
15822	TBXAS1	-0.07982	0.626422	0.857326	None
15823	LRRC3	-0.00476	0.626436	0.857326	None
15824	SLX4	-0.01876	0.626594	0.857421	None
15825	LRRC42	-0.05924	0.626608	0.857421	None
15826	OR10H2	-0.00099	0.626624	0.857421	None
15827	RP11-304L	-0.00127	0.626724	0.857469	None
15828	ITGA3	-0.01644	0.626755	0.857469	None
15829	CCDC153	-0.01813	0.62686	0.857469	None
15830	LINC0102C	0.00238	0.62686	0.857469	None
15831	GKN1	-0.00273	0.626862	0.857469	None
15832	LOC28317	0.010226	0.626897	0.857469	None

15833	RP11-401F	-0.01333	0.62701	0.857529	None
15834	FAM151A	-0.00115	0.627034	0.857529	None
15835	KRTAP19-1	-0.00178	0.627059	0.857529	None
15836	AGXT	-0.00174	0.627121	0.857555	None
15837	MRPL54	-0.11082	0.627195	0.857555	None
15838	EBI3	-0.11827	0.627197	0.857555	None
15839	HES1	0.162695	0.627318	0.857572	None
15840	FAM83G	0.045965	0.627335	0.857572	None
15841	USP19	-0.02644	0.627361	0.857572	None
15842	B3GNT1	-0.03748	0.627387	0.857572	None
15843	LINC00460	0.007327	0.627446	0.857572	None
15844	CHUK	0.078676	0.6275	0.857572	None
15845	FER1L4	0.011579	0.627518	0.857572	None
15846	KAL1	-0.09246	0.627527	0.857572	None
15847	CEP89	-0.03198	0.627711	0.857623	None
15848	THEMIS	-0.02938	0.627765	0.857623	None
15849	SIGLECL1	0.005097	0.627766	0.857623	None
15850	KRTAP4-9	0.003535	0.627768	0.857623	None
15851	SLC25A33	0.077928	0.627794	0.857623	None
15852	LOC10099(	-0.00602	0.627801	0.857623	None
15853	C17orf53	-0.02102	0.627949	0.857727	None
15854	SYNPR	-0.01411	0.627988	0.857727	None
15855	PLEKHA1	-0.0825	0.627996	0.857727	None
15856	C12orf80	0.011405	0.628122	0.857825	None
15857	PRO2012	-0.00129	0.628148	0.857825	None
15858	C2orf50	-0.00292	0.628217	0.857865	None
15859	ZNF32	-0.05757	0.628326	0.857914	None
15860	KCNN3	-0.00123	0.628332	0.857914	None
15861	TUBA3FP	0.019134	0.62843	0.857951	None
15862	HOXD12	-0.00281	0.628482	0.857951	None
15863	CHKB	0.082922	0.628486	0.857951	None
15864	ASCC1	-0.05106	0.628533	0.857951	None
15865	EXOG	0.021494	0.628556	0.857951	None
15866	CENPE	0.161361	0.628695	0.858016	None
15867	FLJ31306	-0.11196	0.628761	0.858016	None
15868	RP11-440I	-0.00525	0.628779	0.858016	None
15869	TPD52L3	0.019253	0.628801	0.858016	None
15870	CNGA3	-0.00202	0.628802	0.858016	None
15871	SPIN4	0.135667	0.628977	0.858089	None
15872	KRTAP3-3	-0.00196	0.628989	0.858089	None
15873	STOML3	-0.00893	0.629014	0.858089	None
15874	SOX10	-0.0066	0.629114	0.858089	None
15875	ZNF214	-0.00686	0.629174	0.858089	None
15876	PGBD2	-0.05296	0.629174	0.858089	None
15877	SHOX	-0.01169	0.62919	0.858089	None
15878	CATSPER3	-0.03641	0.629196	0.858089	None
15879	C1orf43	0.092738	0.629234	0.858089	None
15880	PRO2852	-0.13395	0.629252	0.858089	None
15881	LRRC4B	-0.00174	0.629322	0.858119	None
15882	TMTC3	-0.08066	0.629353	0.858119	None
15883	C1QTNF4	0.198765	0.629502	0.85823	None
15884	KAT5	-0.05585	0.629536	0.85823	None
15885	LOC10192	-0.00138	0.629602	0.85823	None
15886	CELA3A	0.005564	0.629626	0.85823	None
15887	SLC19A3	-0.00213	0.629674	0.85823	None
15888	RP11-307(	-0.00205	0.629699	0.85823	None
15889	UHRF1BP1	-0.05479	0.629744	0.85823	None
15890	FCGR1A	-0.00159	0.629752	0.85823	None

15891	RP11-259(	-0.00207	0.629833	0.858287	None
15892	GPC4	-0.03215	0.629963	0.85841	None
15893	LOC34035	-0.00148	0.630225	0.858712	None
15894	NPTXR	-0.01116	0.630289	0.858712	None
15895	BLM	-0.08336	0.630304	0.858712	None
15896	DIO2	-0.00326	0.630403	0.85873	None
15897	STEAP4	0.0794	0.630427	0.85873	None
15898	C1QTNF6	-0.00786	0.630436	0.85873	None
15899	MAGEE1	-0.06946	0.630609	0.858857	None
15900	NTRK3	-0.00531	0.630619	0.858857	None
15901	COX19	-0.07997	0.630648	0.858857	None
15902	RNF133	-0.00714	0.630801	0.859011	None
15903	ASIC4	-0.01498	0.631023	0.859152	None
15904	CCDC70	0.01096	0.631098	0.859152	None
15905	LRRC48	0.02406	0.631127	0.859152	None
15906	GRK4	-0.03987	0.631131	0.859152	None
15907	NAALAD2	-0.00895	0.63117	0.859152	None
15908	GUSBP11	0.024448	0.631172	0.859152	None
15909	ISYNA1	0.029639	0.631182	0.859152	None
15910	BC033241	-0.01038	0.63127	0.859218	None
15911	JMY	0.087148	0.631333	0.859232	None
15912	LINC01128	-0.02656	0.63136	0.859232	None
15913	LOC10272	0.025706	0.63146	0.859264	None
15914	ZNF519	-0.09064	0.631463	0.859264	None
15915	EXOSC6	0.062958	0.631628	0.85927	None
15916	LOC10099	-0.0065	0.631716	0.85927	None
15917	RP11-279(	-0.00487	0.631724	0.85927	None
15918	DYNLRB1	0.019693	0.631743	0.85927	None
15919	KDM8	-0.02199	0.631745	0.85927	None
15920	CSMD3	-0.06575	0.631773	0.85927	None
15921	RBP5	-0.00564	0.631846	0.85927	None
15922	LOC10013	0.029655	0.631876	0.85927	None
15923	LOC10254	-0.00543	0.631896	0.85927	None
15924	SLC22A23	-0.01991	0.631989	0.85927	None
15925	C11orf86	0.026806	0.631994	0.85927	None
15926	MGAM	-0.1674	0.63202	0.85927	None
15927	NFKB2	-0.03143	0.632021	0.85927	None
15928	SCN1A	-0.00285	0.632023	0.85927	None
15929	EXTL3	-0.0203	0.632355	0.859666	None
15930	RP3-406P2	0.064445	0.632471	0.859666	None
15931	LOC10192	-0.00684	0.632571	0.859666	None
15932	IDI2-AS1	-0.02566	0.632612	0.859666	None
15933	PCCA	-0.03313	0.63266	0.859666	None
15934	DERL3	0.073315	0.632678	0.859666	None
15935	LOC28608	0.000647	0.632693	0.859666	None
15936	SERTM1	-0.01365	0.632758	0.859666	None
15937	LOC10272	0.02804	0.63277	0.859666	None
15938	FLJ25758	-0.008	0.632777	0.859666	None
15939	GSTA1	-0.03671	0.632785	0.859666	None
15940	BAG5	-0.13788	0.63279	0.859666	None
15941	C11orf88	-0.00438	0.632866	0.859669	None
15942	R3HDML	-0.00137	0.632872	0.859669	None
15943	SYT2	-0.00199	0.633132	0.859952	None
15944	MORC1	-0.04607	0.63316	0.859952	None
15945	APLP2	-0.10251	0.633208	0.859963	None
15946	TACR1	-0.00309	0.633448	0.859995	None
15947	LAX1	0.101324	0.633467	0.859995	None
15948	PRPSAP2	-0.04562	0.633468	0.859995	None

15949	SEMA3B	-0.01703	0.633509	0.859995	None
15950	KLHL29	-0.00235	0.633522	0.859995	None
15951	ANKRD26F	-0.03642	0.633553	0.859995	None
15952	RP11-110S	-0.00134	0.633565	0.859995	None
15953	GUCA2B	-0.00144	0.63357	0.859995	None
15954	TRPA1	-0.00084	0.633589	0.859995	None
15955	RUVBL1	-0.08165	0.633763	0.860099	None
15956	YTHDC1	0.075604	0.633804	0.860099	None
15957	LOC10192	-0.00507	0.633904	0.860099	None
15958	IFNA7	0.003019	0.633933	0.860099	None
15959	ASGR2	-0.05521	0.633937	0.860099	None
15960	RP11-141M	-0.00131	0.63394	0.860099	None
15961	DEDD	-0.06371	0.633943	0.860099	None
15962	OR2L1P	-0.00387	0.634211	0.860375	None
15963	IRX2	0.008205	0.634226	0.860375	None
15964	MYL3	0.014666	0.634393	0.860548	None
15965	LOC10028	-0.05661	0.634579	0.86071	None
15966	AC005838.	-0.00128	0.634631	0.86071	None
15967	LOC10192	-0.00089	0.634632	0.86071	None
15968	LINC01091	-0.00905	0.634827	0.860905	None
15969	TNFRSF19	-0.00442	0.634877	0.860905	None
15970	ARSF	0.009926	0.634953	0.860905	None
15971	TMEM115	-0.07955	0.635013	0.860905	None
15972	FBN1	-0.11739	0.635056	0.860905	None
15973	RHEB	-0.05972	0.635073	0.860905	None
15974	LINC01226	0.032077	0.635135	0.860905	None
15975	BMPR2	-0.1029	0.635138	0.860905	None
15976	CCDC93	0.042878	0.63516	0.860905	None
15977	KIFC3	0.023654	0.635173	0.860905	None
15978	TTYH1	-0.00657	0.635313	0.861001	None
15979	RNASEH1	0.075527	0.635345	0.861001	None
15980	ASIP	-0.00172	0.63538	0.861001	None
15981	LOC10013	0.01733	0.635404	0.861001	None
15982	ZCCHC4	-0.01412	0.635443	0.861001	None
15983	NOL12	0.053719	0.635625	0.861065	None
15984	GPR26	0.007439	0.635649	0.861065	None
15985	NR2F2	-0.04815	0.635656	0.861065	None
15986	NDST1	0.051796	0.635719	0.861065	None
15987	NCKAP1	-0.07258	0.635815	0.861065	None
15988	CNDP1	0.000618	0.635821	0.861065	None
15989	ATP1A2	-0.00945	0.635827	0.861065	None
15990	RETNLB	-0.00163	0.635895	0.861065	None
15991	TLX3	-0.00663	0.63591	0.861065	None
15992	SLCO5A1	-0.131	0.635932	0.861065	None
15993	LINC01134	0.013997	0.636058	0.861065	None
15994	ARSE	-0.02933	0.63614	0.861065	None
15995	IFI27L2	-0.06936	0.63615	0.861065	None
15996	WISP1	-0.02323	0.636153	0.861065	None
15997	TEKT1	-0.00297	0.636159	0.861065	None
15998	TMCO5B	-0.01032	0.636187	0.861065	None
15999	HIST1H4H	-0.20093	0.636232	0.861065	None
16000	SIRPB1	-0.02439	0.636251	0.861065	None
16001	CCT8L2	-0.00317	0.636342	0.861065	None
16002	SRP68	0.059819	0.636393	0.861065	None
16003	LMNTD1	-0.00624	0.636398	0.861065	None
16004	RP11-534L	-0.05229	0.636411	0.861065	None
16005	LOC10272	-0.01995	0.636424	0.861065	None
16006	AGBL3	-0.01369	0.636455	0.861065	None

16007	ELK1	0.021318	0.636502	0.861065	None
16008	MTMR1	0.029624	0.636533	0.861065	None
16009	PKNOX1	-0.04564	0.636639	0.861065	None
16010	POU5F1B	0.018053	0.636672	0.861065	None
16011	VWA5B1	-0.0072	0.636679	0.861065	None
16012	GCFC2	-0.04966	0.636708	0.861065	None
16013	RPL8	0.047394	0.636789	0.861065	None
16014	LOC10192	-0.00152	0.636801	0.861065	None
16015	DNAJC17	-0.08286	0.636802	0.861065	None
16016	DQ594366	-0.01591	0.636954	0.861216	None
16017	SPATA42	-0.00908	0.637001	0.861225	None
16018	ABCC11	-0.00416	0.637103	0.86131	None
16019	ATRX	0.06568	0.63716	0.861333	None
16020	TAB1	-0.04261	0.637303	0.861386	None
16021	PPM1E	-0.02814	0.637329	0.861386	None
16022	C3orf20	-0.00887	0.637375	0.861386	None
16023	ADORA2A	-0.002	0.637451	0.861386	None
16024	ARHGAP3	0.026622	0.637458	0.861386	None
16025	MYEOV	-0.01946	0.637467	0.861386	None
16026	LOC10192	-0.00153	0.637477	0.861386	None
16027	OBP2A	-0.0008	0.637616	0.861519	None
16028	C1orf177	-0.00152	0.637777	0.86153	None
16029	CYP1B1	0.260722	0.63779	0.86153	None
16030	MIR3916	0.112593	0.637825	0.86153	None
16031	RP11-257I	-0.00144	0.637836	0.86153	None
16032	LOC10272	-0.00111	0.63786	0.86153	None
16033	LINC0033C	0.010704	0.637862	0.86153	None
16034	STK32A	-0.00099	0.637948	0.861562	None
16035	RP11-36B6	-0.00741	0.638015	0.861562	None
16036	MEGF10	-0.01506	0.638055	0.861562	None
16037	RPL35	-0.05933	0.638065	0.861562	None
16038	RGS11	-0.00162	0.638222	0.861562	None
16039	DEFB129	-0.00183	0.638241	0.861562	None
16040	TIGIT	-0.01704	0.638298	0.861562	None
16041	PRPF3	0.055716	0.638354	0.861562	None
16042	CEBPA-AS	0.047776	0.638384	0.861562	None
16043	LINC00238	-0.00643	0.6384	0.861562	None
16044	TVP23C	-0.02332	0.638466	0.861562	None
16045	LOC10192	-0.00232	0.638467	0.861562	None
16046	SLC8B1	0.040316	0.638565	0.861562	None
16047	AF186192.	-0.00087	0.638621	0.861562	None
16048	FARSB	0.125421	0.638623	0.861562	None
16049	LOC10192	-0.00626	0.638632	0.861562	None
16050	FAM63A	-0.05505	0.638634	0.861562	None
16051	RGN	0.014479	0.638677	0.861562	None
16052	SH3RF3	-0.11984	0.638715	0.861562	None
16053	LOC10192	-0.01072	0.638731	0.861562	None
16054	CLYBL-AS	0.01211	0.638744	0.861562	None
16055	GRID1-AS	-0.00159	0.638761	0.861562	None
16056	SP3P	-0.0017	0.638877	0.861664	None
16057	CTC-527H	-0.01121	0.63903	0.861752	None
16058	YPEL3	-0.09618	0.639048	0.861752	None
16059	SRRM3	-0.00323	0.639085	0.861752	None
16060	LOC10272	0.004548	0.639102	0.861752	None
16061	MAGED2	-0.0626	0.63918	0.861805	None
16062	KANSL1-A	0.089439	0.63927	0.861873	None
16063	CLDN12	-0.094	0.639394	0.861942	None
16064	FAM24B	0.169674	0.639401	0.861942	None

16065	HFE	-0.01928	0.639457	0.861963	None
16066	LOC101928	-0.00137	0.639547	0.862018	None
16067	HRH4	-0.00666	0.639623	0.862018	None
16068	MECR	-0.09479	0.639659	0.862018	None
16069	CA12	-0.00094	0.639735	0.862018	None
16070	PRL	-0.01142	0.639759	0.862018	None
16071	FOXK1	-0.00855	0.639768	0.862018	None
16072	DKFZP434/	-0.01118	0.639776	0.862018	None
16073	EHD4	-0.04929	0.639849	0.862063	None
16074	PPP1R3F	0.015066	0.639947	0.86214	None
16075	LINC00973	-0.00321	0.640059	0.862201	None
16076	EPS8L3	-0.01934	0.640126	0.862201	None
16077	SVIP	-0.06717	0.640167	0.862201	None
16078	MIR4313	-0.00578	0.640175	0.862201	None
16079	IL17F	-0.00136	0.640264	0.862201	None
16080	RP11-504/	-0.0019	0.640267	0.862201	None
16081	TMEM155	0.013793	0.64027	0.862201	None
16082	LINC00358	-0.00558	0.640382	0.862295	None
16083	FMNL1	0.066912	0.64042	0.862295	None
16084	LOC10050	-0.00409	0.640478	0.86232	None
16085	LOC10192	-0.00162	0.640569	0.862373	None
16086	POPDC3	-0.03292	0.640612	0.862373	None
16087	SLC25A3P1	-0.001	0.640653	0.862373	None
16088	COL21A1	0.053118	0.640713	0.862373	None
16089	TBX20	-0.00203	0.640724	0.862373	None
16090	NOMO3	0.082388	0.640795	0.862373	None
16091	AC112198.	-0.03291	0.640841	0.862373	None
16092	RP11-340/	-0.00112	0.640875	0.862373	None
16093	LOC10192	-0.00259	0.640876	0.862373	None
16094	RNF144B	-0.07449	0.640933	0.862397	None
16095	CEBPZ	-0.05008	0.64103	0.862433	None
16096	MED12L	-0.03692	0.64104	0.862433	None
16097	ACTC1	-0.02171	0.64114	0.862456	None
16098	OR5J2	-0.00131	0.641158	0.862456	None
16099	FXR1	-0.05703	0.641188	0.862456	None
16100	SCIN	0.010935	0.641253	0.862456	None
16101	OR2B6	0.01669	0.641256	0.862456	None
16102	FLRT3	-0.06835	0.641343	0.862519	None
16103	CNTNAP5	-0.0132	0.641397	0.862519	None
16104	LPAL2	-0.00349	0.641423	0.862519	None
16105	WNT9B	-0.00239	0.641582	0.86268	None
16106	TCTE1	-0.00785	0.641675	0.862751	None
16107	NMD3	0.049314	0.641746	0.862793	None
16108	APPBP2	-0.05909	0.641788	0.862796	None
16109	NDUFA9	-0.06779	0.641865	0.862846	None
16110	LOC10192	-0.0024	0.641946	0.862901	None
16111	LOC10272	-0.01039	0.642076	0.862973	None
16112	LOC10192	-0.00182	0.642079	0.862973	None
16113	HMP19	0.007104	0.642167	0.863034	None
16114	AKNAD1	0.000595	0.642253	0.863034	None
16115	LOC28611	-0.00857	0.642277	0.863034	None
16116	FAM215A	-0.01107	0.642321	0.863034	None
16117	MYCBPAP	-0.01396	0.642363	0.863034	None
16118	TCRBV12S	-0.00335	0.642364	0.863034	None
16119	CT62	-0.01395	0.642485	0.863144	None
16120	CLIC3	-0.01007	0.642596	0.86319	None
16121	ATP8B1	0.084938	0.642599	0.86319	None
16122	LHX5	-0.00253	0.642735	0.863241	None

16123	GATA3-AS	-0.00203	0.642807	0.863241	None
16124	CEP76	-0.06803	0.642847	0.863241	None
16125	AK097370	-0.0131	0.642851	0.863241	None
16126	C2CD4A	-0.00139	0.642908	0.863241	None
16127	MMP8	-0.20603	0.64291	0.863241	None
16128	COL6A3	-0.15716	0.642916	0.863241	None
16129	LOC34007	-0.00361	0.642964	0.863252	None
16130	MAGI1-IT1	-0.0027	0.64319	0.863404	None
16131	CBLN1	-0.00298	0.643262	0.863404	None
16132	GPR116	-0.10738	0.643269	0.863404	None
16133	LOC10192	-0.00627	0.643274	0.863404	None
16134	AKR1E2	-0.07772	0.643285	0.863404	None
16135	SEC23A	0.06383	0.64337	0.863404	None
16136	TENM2	-0.05146	0.643421	0.863404	None
16137	LINC00887	-0.00347	0.643434	0.863404	None
16138	RP11-677C	-0.00345	0.643437	0.863404	None
16139	NES	0.0126	0.643604	0.863575	None
16140	ADIPOR2	0.068378	0.643759	0.863716	None
16141	CARD18	-0.00285	0.64384	0.863716	None
16142	CUL4B	0.07226	0.643886	0.863716	None
16143	RFPL2	0.022366	0.643906	0.863716	None
16144	CLNK	-0.0317	0.643953	0.863716	None
16145	SGCE	0.056171	0.643984	0.863716	None
16146	OR4D2	-0.01205	0.644032	0.863716	None
16147	AKAP7	-0.03809	0.644079	0.863716	None
16148	MTNR1B	-0.00091	0.644091	0.863716	None
16149	FREM2	-0.01977	0.644168	0.863716	None
16150	LSM3	-0.03811	0.644173	0.863716	None
16151	SPATA41	-0.0012	0.644187	0.863716	None
16152	BC042029	-0.04728	0.644228	0.863717	None
16153	RPL36AL	0.063627	0.644306	0.863768	None
16154	PCDHB13	0.010255	0.64446	0.86392	None
16155	HIPK4	-0.01033	0.644541	0.863976	None
16156	MYOM2	-0.1072	0.644617	0.864025	None
16157	UEVLD	-0.03819	0.6447	0.864026	None
16158	LINC00086	-0.0038	0.644721	0.864026	None
16159	GCAT	-0.0855	0.644767	0.864026	None
16160	ERICH3-AS	0.009736	0.64479	0.864026	None
16161	LOC10192	-0.02094	0.644818	0.864026	None
16162	LRP2BP	-0.00362	0.644951	0.864151	None
16163	OGFRP1	-0.00428	0.645132	0.864307	None
16164	LOC10192	0.032206	0.64519	0.864307	None
16165	FZD6	-0.15483	0.645245	0.864307	None
16166	CYP4Z2P	-0.00203	0.645267	0.864307	None
16167	MATR3	-0.11901	0.645279	0.864307	None
16168	LOC10272	-0.0257	0.645307	0.864307	None
16169	USP48	-0.06662	0.64537	0.864339	None
16170	SEMA7A	-0.03067	0.645636	0.864523	None
16171	HS1BP3	0.010337	0.645734	0.864523	None
16172	C5orf58	-0.00077	0.645746	0.864523	None
16173	PEX2	-0.06445	0.645761	0.864523	None
16174	DBF4	0.070802	0.645772	0.864523	None
16175	SLC30A10	-0.00308	0.645825	0.864523	None
16176	C16orf70	-0.03005	0.64592	0.864523	None
16177	FAM173B	-0.05503	0.645963	0.864523	None
16178	JUNB	-0.14584	0.646003	0.864523	None
16179	CLSPN	-0.02648	0.646005	0.864523	None
16180	LOC10192	0.02082	0.646022	0.864523	None

16181	ST6GALNA	-0.17628	0.646026	0.864523	None
16182	DDI1	-0.0011	0.646027	0.864523	None
16183	LOC10192	-0.01202	0.646311	0.864807	None
16184	RP11-505K	-0.00232	0.646333	0.864807	None
16185	HES7	0.017074	0.646359	0.864807	None
16186	BFSP2	-0.01853	0.646399	0.864808	None
16187	ABO	0.017867	0.646659	0.86506	None
16188	ZP2	0.003446	0.646683	0.86506	None
16189	C14orf23	-0.00796	0.646722	0.86506	None
16190	TESK2	-0.05241	0.646749	0.86506	None
16191	ZSCAN22	-0.03807	0.646788	0.86506	None
16192	PPP3R2	0.000608	0.646857	0.865061	None
16193	TCRBV15S	-0.0011	0.646885	0.865061	None
16194	RP11-692F	-0.02415	0.646962	0.865061	None
16195	LRRC49	-0.0755	0.646984	0.865061	None
16196	ZMAT2	0.056513	0.646988	0.865061	None
16197	NCR1	-0.00756	0.647063	0.8651	None
16198	LINC00479	-0.00086	0.647097	0.8651	None
16199	GSTM5	-0.20232	0.647186	0.865165	None
16200	VWA7	-0.00157	0.647364	0.865273	None
16201	FBXL19-AS	-0.0062	0.647366	0.865273	None
16202	MGC45922	-0.02956	0.647448	0.865273	None
16203	PRDM7	-0.01254	0.647484	0.865273	None
16204	PCSK6	0.004301	0.647508	0.865273	None
16205	ERICH3	-0.01126	0.647525	0.865273	None
16206	LOC10050	-0.004	0.647621	0.865273	None
16207	LOC55420	-0.00162	0.647694	0.865273	None
16208	C9orf173	0.018799	0.647728	0.865273	None
16209	CD63	-0.07526	0.647776	0.865273	None
16210	MVD	-0.03364	0.647799	0.865273	None
16211	LOC78052	0.01485	0.647807	0.865273	None
16212	CYP2C9	-0.04584	0.647807	0.865273	None
16213	ZIC3	0.002767	0.647831	0.865273	None
16214	TEX43	-0.00077	0.647896	0.865273	None
16215	GPR50	-0.01534	0.647942	0.865273	None
16216	MAN1B1-A	-0.05504	0.647979	0.865273	None
16217	C20orf26	-0.00393	0.647986	0.865273	None
16218	ATP5O	0.02747	0.648041	0.86528	None
16219	AFP	-0.01063	0.648071	0.86528	None
16220	ZNF160	-0.06479	0.64816	0.865345	None
16221	RNF6	-0.01398	0.6483	0.865441	None
16222	TM9SF2	0.041415	0.648326	0.865441	None
16223	KCND2	-0.01566	0.648352	0.865441	None
16224	CLIP4	0.074291	0.648415	0.865444	None
16225	AP000253.	0.021259	0.648433	0.865444	None
16226	PLCD4	0.020255	0.648766	0.865694	None
16227	LOC10192	-0.00319	0.648827	0.865694	None
16228	TLL1	-0.00082	0.648866	0.865694	None
16229	POLR2B	0.043427	0.648868	0.865694	None
16230	RXRG	-0.00282	0.64887	0.865694	None
16231	RP11-740C	-0.00251	0.648881	0.865694	None
16232	FAM224A	-0.01514	0.648901	0.865694	None
16233	MCL1	0.119076	0.648962	0.865722	None
16234	KLHL35	-0.01584	0.649015	0.865726	None
16235	CEBPE	-0.10133	0.649047	0.865726	None
16236	RP11-360K	-0.00233	0.649094	0.865726	None
16237	CTD-2611C	-0.004	0.649231	0.865726	None
16238	ETV1	-0.00109	0.649259	0.865726	None

16239	SYNM	-0.12038	0.649299	0.865726	None
16240	HIST1H2A(	-0.17224	0.649336	0.865726	None
16241	ZNF793	0.081932	0.64936	0.865726	None
16242	LCN1	-0.00677	0.649415	0.865726	None
16243	GJD4	-0.00682	0.649421	0.865726	None
16244	MCF2L2	-0.00826	0.649429	0.865726	None
16245	DPYSL4	-0.00534	0.649472	0.865726	None
16246	FAM109A	-0.04595	0.64957	0.865726	None
16247	GNA15	-0.08574	0.649602	0.865726	None
16248	UIMC1	0.05045	0.64961	0.865726	None
16249	BANF2	-0.00213	0.649624	0.865726	None
16250	CCDC116	-0.00173	0.649644	0.865726	None
16251	ADCY8	0.016705	0.649688	0.865731	None
16252	SCN3B	-0.00098	0.649733	0.865738	None
16253	TECTA	0.017131	0.649853	0.865768	None
16254	ZC3H12C	0.053508	0.649857	0.865768	None
16255	ELOF1	-0.06952	0.649876	0.865768	None
16256	FAM168B	0.024728	0.649973	0.865811	None
16257	PSAPL1	-0.02817	0.649988	0.865811	None
16258	ZNF300	0.18651	0.650396	0.86625	None
16259	FAM205A	-0.00178	0.650438	0.86625	None
16260	TRAK1	-0.06957	0.650463	0.86625	None
16261	SNX24	-0.01409	0.650571	0.86625	None
16262	QRICH1	-0.0531	0.650593	0.86625	None
16263	RP5-1189k	-0.00107	0.650695	0.86625	None
16264	SPINK5	-0.00318	0.650697	0.86625	None
16265	AICDA	-0.00277	0.650707	0.86625	None
16266	ESCO1	-0.08831	0.65071	0.86625	None
16267	MDM2	0.038292	0.650718	0.86625	None
16268	TMEFF2	-0.05067	0.650814	0.866325	None
16269	LRTM1	-0.02239	0.650969	0.866478	None
16270	LINC01158	0.013465	0.651049	0.866493	None
16271	RP4-794H1	-0.00588	0.651076	0.866493	None
16272	MIPEP	-0.10092	0.651105	0.866493	None
16273	RPN1	-0.0752	0.651176	0.866493	None
16274	PPP1R12C	0.01341	0.65118	0.866493	None
16275	EPHB4	-0.08338	0.651373	0.866675	None
16276	LINC01085	-0.0097	0.651425	0.866675	None
16277	LINC00277	-0.00577	0.651437	0.866675	None
16278	CYP4F30P	-0.00122	0.651517	0.86669	None
16279	ZW10	0.063062	0.651607	0.86669	None
16280	USP22	-0.01574	0.65166	0.86669	None
16281	SHISA3	-0.02184	0.651707	0.86669	None
16282	SPOCK1	-0.04527	0.651715	0.86669	None
16283	NLRP5	-0.00216	0.651731	0.86669	None
16284	RP1-212P9	-0.00163	0.651758	0.86669	None
16285	C20orf144	-0.00455	0.651806	0.86669	None
16286	SOBP	-0.00105	0.651808	0.86669	None
16287	HSPA12B	0.022847	0.651878	0.866729	None
16288	L3MBTL4	-0.07198	0.652053	0.866796	None
16289	FAM43B	0.004784	0.652076	0.866796	None
16290	DLG2	-0.00875	0.652083	0.866796	None
16291	MASP2	-0.00515	0.652114	0.866796	None
16292	TRIM9	-0.07644	0.652128	0.866796	None
16293	POFUT1	0.030505	0.652248	0.866901	None
16294	LOC10012!	0.046949	0.652344	0.866963	None
16295	ENTPD5	-0.04566	0.652411	0.866963	None
16296	RIMS1	-0.01719	0.652448	0.866963	None

16297	YWHAZ	0.059795	0.652502	0.866963	None
16298	PGBD4	-0.06575	0.652522	0.866963	None
16299	AKR7A2	-0.04971	0.652586	0.866963	None
16300	IL22RA2	-0.00923	0.652589	0.866963	None
16301	RUNX1-IT1	0.108861	0.652614	0.866963	None
16302	CTSV	-0.09866	0.652682	0.867	None
16303	SCNN1G	0.005002	0.652752	0.86704	None
16304	GSK3A	-0.02179	0.652936	0.867113	None
16305	CECR1	-0.17078	0.652972	0.867113	None
16306	AQPEP	-0.00169	0.653039	0.867113	None
16307	CLECL1	-0.08253	0.653064	0.867113	None
16308	GLI3	-0.04904	0.653076	0.867113	None
16309	BASP1	-0.1008	0.653117	0.867113	None
16310	ST8SIA2	-0.00271	0.653126	0.867113	None
16311	CTB-119C2	-0.02334	0.65317	0.867113	None
16312	LOC219690	-0.00234	0.653173	0.867113	None
16313	IL23A	-0.02079	0.653208	0.867113	None
16314	IL12A-AS1	-0.01376	0.653279	0.867131	None
16315	AHNAK2	-0.00742	0.653369	0.867131	None
16316	LOC100990	-0.00219	0.65341	0.867131	None
16317	SLC12A8	-0.00724	0.653423	0.867131	None
16318	MPZL3	-0.02634	0.653472	0.867131	None
16319	LINC00659	-0.00113	0.653497	0.867131	None
16320	DSG1	-0.00213	0.653502	0.867131	None
16321	FAM69C	-0.01216	0.653565	0.867163	None
16322	SLC25A30	-0.031	0.653632	0.867172	None
16323	UPP1	0.140787	0.653665	0.867172	None
16324	10-Mar	-0.00325	0.653777	0.867172	None
16325	GS1-124K5	-0.03538	0.653789	0.867172	None
16326	CYS1	-0.00387	0.653816	0.867172	None
16327	RP11-1109	-0.03162	0.653833	0.867172	None
16328	PCYT1A	-0.06225	0.653853	0.867172	None
16329	PHYH	-0.07317	0.653956	0.867256	None
16330	CCDC68	-0.04775	0.654152	0.867315	None
16331	COA3	-0.03622	0.654193	0.867315	None
16332	STRN3	0.063715	0.654261	0.867315	None
16333	C6orf15	0.02672	0.654271	0.867315	None
16334	TTLL13	0.00477	0.654279	0.867315	None
16335	CXCL16	-0.21052	0.654287	0.867315	None
16336	TRIM7	-0.03687	0.654329	0.867315	None
16337	FZD10-AS1	-0.00126	0.654413	0.867315	None
16338	IQUB	0.005956	0.654425	0.867315	None
16339	PROSER1	-0.04571	0.654429	0.867315	None
16340	CALML4	-0.0743	0.654441	0.867315	None
16341	LYSMD2	-0.06564	0.654632	0.86743	None
16342	DNPH1	-0.11272	0.654652	0.86743	None
16343	NXPE4	-0.00434	0.654653	0.86743	None
16344	ACMSD	-0.00255	0.654688	0.86743	None
16345	SOX9-AS1	-0.00364	0.654813	0.867542	None
16346	CDH20	-0.00386	0.654955	0.867677	None
16347	TRAPPC6A	0.076896	0.655197	0.867794	None
16348	LOC10013:	0.121716	0.655237	0.867794	None
16349	LOC152580	-0.00119	0.655254	0.867794	None
16350	PROZ	-0.05193	0.655297	0.867794	None
16351	LRRC56	-0.04016	0.655325	0.867794	None
16352	LCNL1	-0.00208	0.655335	0.867794	None
16353	RASGRF2	-0.01805	0.65535	0.867794	None
16354	FAM47E	0.006973	0.655395	0.867794	None

16355	CBS	-0.01585	0.655404	0.867794	None
16356	TDRD12	0.038798	0.655455	0.867808	None
16357	PPA2	0.104929	0.655624	0.867896	None
16358	LOC10192!	-0.0062	0.655667	0.867896	None
16359	SBNO2	-0.0232	0.655687	0.867896	None
16360	SERPIND1	-0.00746	0.655717	0.867896	None
16361	MIR133A1I	0.012182	0.65575	0.867896	None
16362	MIR100HG	-0.07788	0.65579	0.867896	None
16363	FOXO4	-0.04386	0.655827	0.867896	None
16364	ZC3H8	0.052361	0.655868	0.867896	None
16365	LOC10272	-0.00231	0.655922	0.867896	None
16366	LOC10050	-0.00109	0.655937	0.867896	None
16367	CYP2J2	-0.03787	0.655962	0.867896	None
16368	UBA3	0.047794	0.656032	0.867935	None
16369	RBM8A	-0.05221	0.656097	0.867969	None
16370	NHLRC3	0.086991	0.656219	0.867975	None
16371	REEP4	-0.04303	0.65622	0.867975	None
16372	TNK1	0.024955	0.656266	0.867975	None
16373	RP11-59H	-0.01764	0.656268	0.867975	None
16374	LOC64690	-0.00383	0.656302	0.867975	None
16375	PCOLCE	-0.03639	0.656441	0.867983	None
16376	LOC28478	-0.00152	0.656488	0.867983	None
16377	ERVK3-2	0.012677	0.6565	0.867983	None
16378	C8G	-0.00397	0.656525	0.867983	None
16379	PRM2	-0.00152	0.656556	0.867983	None
16380	CDH1	-0.15025	0.656621	0.867983	None
16381	MOGAT3	-0.00705	0.656621	0.867983	None
16382	PRR14L	-0.03898	0.656629	0.867983	None
16383	MMP19	0.061756	0.656711	0.868039	None
16384	LOC10013	-0.00091	0.656816	0.868124	None
16385	HOXD13	-0.00177	0.656876	0.86815	None
16386	SCIMP	-0.00235	0.657066	0.86833	None
16387	IQCF5-AS1	0.001169	0.657127	0.86833	None
16388	C1orf116	-0.01105	0.657148	0.86833	None
16389	OPRD1	-0.01671	0.657185	0.86833	None
16390	LOC28542	-0.00204	0.657223	0.86833	None
16391	RP11-102C	-0.00959	0.657252	0.86833	None
16392	CSRP3	-0.00428	0.657314	0.868359	None
16393	PAK7	-0.00169	0.657399	0.868418	None
16394	FOLH1	-0.01041	0.65745	0.868432	None
16395	SRP72	0.045009	0.657537	0.868448	None
16396	ROR1	-0.00084	0.657617	0.868448	None
16397	BLID	-0.00084	0.657626	0.868448	None
16398	UGCG	0.149527	0.657629	0.868448	None
16399	LINC00482	-0.03199	0.657707	0.868448	None
16400	SLFN13	0.114444	0.657751	0.868448	None
16401	PTGDR	-0.0194	0.657766	0.868448	None
16402	HSPB11	-0.07907	0.657829	0.868448	None
16403	MYH11	0.001491	0.657848	0.868448	None
16404	RP11-102L	-0.01813	0.657907	0.868448	None
16405	GPC3	-0.00778	0.65802	0.868448	None
16406	AP000696.	-0.0054	0.658021	0.868448	None
16407	FBXO39	0.011526	0.658023	0.868448	None
16408	RNF2	-0.01921	0.658061	0.868448	None
16409	FKBP6	-0.00951	0.658072	0.868448	None
16410	PIK3R5	-0.02827	0.658206	0.868448	None
16411	CDC25A	0.128112	0.658424	0.868448	None
16412	ARVCF	0.02449	0.658447	0.868448	None

16413	SHPK	-0.04027	0.65851	0.868448	None
16414	RDH16	-0.02233	0.658551	0.868448	None
16415	WIBG	-0.04071	0.658609	0.868448	None
16416	AC141928.	-0.00738	0.658639	0.868448	None
16417	LOC10192	-0.0026	0.658666	0.868448	None
16418	FABP2	-0.02973	0.658694	0.868448	None
16419	LOC10027	-0.01826	0.658726	0.868448	None
16420	Ndufaf4	-0.00092	0.65874	0.868448	None
16421	GPR37	-0.007	0.658814	0.868448	None
16422	LOC10192	0.014563	0.658828	0.868448	None
16423	LOC28577	-0.00114	0.658828	0.868448	None
16424	LOC10192	-0.00199	0.658861	0.868448	None
16425	USP46	-0.06133	0.658876	0.868448	None
16426	G6PC	-0.00322	0.658909	0.868448	None
16427	CNDP2	0.060025	0.658922	0.868448	None
16428	RP11-680	-0.00509	0.658939	0.868448	None
16429	LINC00477	-0.00291	0.658961	0.868448	None
16430	LOC40059	-0.00323	0.658971	0.868448	None
16431	LOC10192	-0.03038	0.658988	0.868448	None
16432	MRPL9	0.04133	0.659004	0.868448	None
16433	ATP5G3	-0.07532	0.659049	0.868448	None
16434	SEPSECS-A	-0.05861	0.659066	0.868448	None
16435	KRT2	-0.00156	0.659154	0.868512	None
16436	MAP3K9	-0.02071	0.659262	0.868601	None
16437	ANKRD2	-0.02838	0.659367	0.868639	None
16438	LOC33997	-0.01887	0.659377	0.868639	None
16439	LINC00483	-0.00793	0.659411	0.868639	None
16440	KRT82	-0.01877	0.659605	0.868756	None
16441	APLF	-0.03305	0.659612	0.868756	None
16442	SLAMF6	-0.04565	0.659647	0.868756	None
16443	STPG2	-0.00518	0.65966	0.868756	None
16444	LOC10013	-0.0066	0.659739	0.868806	None
16445	CALR3	-0.00356	0.65982	0.868861	None
16446	ARHGAP26	-0.05965	0.659887	0.868879	None
16447	LOC10192	-0.00082	0.65995	0.868879	None
16448	DDIT3	-0.13019	0.659967	0.868879	None
16449	ZSCAN4	0.022737	0.659995	0.868879	None
16450	RP11-642D	0.060744	0.660081	0.868887	None
16451	TLR2	-0.13685	0.660081	0.868887	None
16452	VARS2	0.064896	0.660267	0.869079	None
16453	ZNF678	-0.07204	0.660353	0.869096	None
16454	KCNE4	-0.00122	0.660361	0.869096	None
16455	EIF3K	0.060376	0.660559	0.869267	None
16456	LOC64658	-0.01106	0.66067	0.869267	None
16457	WFDC13	-0.00367	0.660681	0.869267	None
16458	C14orf93	-0.05651	0.660718	0.869267	None
16459	RP11-7F17	-0.00181	0.660725	0.869267	None
16460	RP11-63A1	-0.00356	0.660756	0.869267	None
16461	LOC10050	-0.00117	0.660807	0.869267	None
16462	DNASE1L2	-0.01549	0.660812	0.869267	None
16463	DIAPH1	-0.04365	0.660963	0.869296	None
16464	NDUFAF5	0.068137	0.660986	0.869296	None
16465	AAMDC	0.057072	0.661078	0.869296	None
16466	KRT9	-0.00175	0.661112	0.869296	None
16467	MYH4	-0.01881	0.66113	0.869296	None
16468	SCG5	0.042359	0.661148	0.869296	None
16469	FAM154A	-0.00702	0.661149	0.869296	None
16470	CCNYL2	-0.00099	0.661155	0.869296	None

16471	MSI2	-0.10167	0.66134	0.869476	None
16472	FAM160B1	-0.03405	0.661372	0.869476	None
16473	CCDC173	-0.10993	0.661531	0.869632	None
16474	MUCL1	-0.00709	0.661608	0.86968	None
16475	KIRREL3-A	-0.00309	0.661708	0.869713	None
16476	GFRA1	-0.00793	0.661713	0.869713	None
16477	ZC2HC1B	-0.00077	0.661796	0.869725	None
16478	RNF122	0.023047	0.661822	0.869725	None
16479	TNFSF14	0.037553	0.661843	0.869725	None
16480	IRS1	-0.03761	0.661955	0.869821	None
16481	GNAO1	-0.02235	0.66205	0.869837	None
16482	PLEKHH2	-0.002	0.662073	0.869837	None
16483	PTP4A1	0.074892	0.662088	0.869837	None
16484	TNR	-0.00072	0.66215	0.869865	None
16485	DOCK9	0.008054	0.662196	0.869873	None
16486	DLEU7	-0.00101	0.662365	0.870009	None
16487	ZNF92	-0.02386	0.66238	0.870009	None
16488	STRIP1	-0.0376	0.66248	0.870087	None
16489	TXLNG	0.076897	0.662526	0.870095	None
16490	RP11-1C8.	-0.00185	0.662608	0.87015	None
16491	TAF6L	0.012007	0.662653	0.870157	None
16492	LOC10192ɛ	-0.00114	0.662726	0.870194	None
16493	LINC00311	0.009069	0.662762	0.870194	None
16494	ARPC1A	-0.08193	0.662866	0.870255	None
16495	CYP11B1	-0.00455	0.662908	0.870255	None
16496	SOWAHA	-0.0008	0.662929	0.870255	None
16497	EIF2AK4	0.035277	0.663103	0.870406	None
16498	DCAF12L1	-0.03616	0.663197	0.870406	None
16499	FAM60A	-0.06462	0.663229	0.870406	None
16500	WHSC1L1	-0.04984	0.66335	0.870406	None
16501	TAF1A	-0.08805	0.663384	0.870406	None
16502	VWA8-AS1	-0.00601	0.663409	0.870406	None
16503	DGKI	-0.03199	0.663418	0.870406	None
16504	LINC00925	0.033	0.663433	0.870406	None
16505	LOC10192	-0.00123	0.663466	0.870406	None
16506	KLF15	0.015053	0.663571	0.870406	None
16507	POC1A	-0.07509	0.663671	0.870406	None
16508	TBRG4	-0.05136	0.663687	0.870406	None
16509	ADAMDEC	-0.0419	0.663766	0.870406	None
16510	LOC10050(	-0.00156	0.663776	0.870406	None
16511	C9orf3	-0.06025	0.663791	0.870406	None
16512	PPP1R2P9	-0.00633	0.663799	0.870406	None
16513	COLEC10	-0.00373	0.663847	0.870406	None
16514	FGF7	-0.00205	0.663926	0.870406	None
16515	FAM53A	-0.04192	0.66395	0.870406	None
16516	PVR	0.015945	0.664001	0.870406	None
16517	PLEKHG2	-0.03157	0.664006	0.870406	None
16518	KNG1	-0.00403	0.664042	0.870406	None
16519	EGLN2	-0.00163	0.664048	0.870406	None
16520	AGBL1	-0.00206	0.664064	0.870406	None
16521	LOC10192ɛ	-0.0028	0.664072	0.870406	None
16522	IGSF22	-0.01802	0.664089	0.870406	None
16523	GCK	-0.00616	0.664384	0.870541	None
16524	ERVK13-1	-0.04676	0.664414	0.870541	None
16525	NFYB	-0.0642	0.664437	0.870541	None
16526	ZNF180	0.045743	0.66445	0.870541	None
16527	RHCG	-0.01122	0.664467	0.870541	None
16528	C9orf43	-0.12983	0.664471	0.870541	None

16529	C22orf42	-0.00086	0.664552	0.870541	None
16530	LYPD1	-0.04861	0.664589	0.870541	None
16531	EEF1E1	-0.04531	0.664708	0.870541	None
16532	HDLBP	-0.04121	0.664718	0.870541	None
16533	TEX36-AS1	-0.00094	0.66473	0.870541	None
16534	PLXNA3	-0.00132	0.664734	0.870541	None
16535	RP5-1039K	-0.00086	0.664748	0.870541	None
16536	DRG1	-0.05262	0.664779	0.870541	None
16537	KIAA1549L	-0.0011	0.664801	0.870541	None
16538	RNF32	-0.00122	0.664835	0.870541	None
16539	PPAP2C	-0.01654	0.664966	0.87057	None
16540	LINC00689	-0.00084	0.664967	0.87057	None
16541	GPALPP1	-0.05752	0.664978	0.87057	None
16542	SEMA5A	-0.00398	0.665153	0.870747	None
16543	KIAA0825	0.010738	0.665269	0.870837	None
16544	LHPP	-0.05196	0.665352	0.870837	None
16545	ZNF157	0.010922	0.665374	0.870837	None
16546	LINC00454	-0.0022	0.665438	0.870837	None
16547	KLHL34	-0.01164	0.665442	0.870837	None
16548	GAS7	-0.07805	0.665463	0.870837	None
16549	SERBP1	0.046229	0.665563	0.870862	None
16550	LOC28335	-0.00466	0.665578	0.870862	None
16551	LOC10028	-0.00079	0.665603	0.870862	None
16552	ST3GAL3	-0.00349	0.665687	0.870891	None
16553	SRRM4	-0.00525	0.665763	0.870891	None
16554	MYRIP	-0.06232	0.665775	0.870891	None
16555	SLC7A10	-0.0037	0.665786	0.870891	None
16556	ROR1-AS1	-0.01342	0.665863	0.870934	None
16557	IDH3A	0.066379	0.6659	0.870934	None
16558	CYLD	-0.09092	0.666065	0.87099	None
16559	BMP10	-0.01221	0.666089	0.87099	None
16560	LOC10050	-0.00068	0.666126	0.87099	None
16561	CDC40	-0.03236	0.666126	0.87099	None
16562	Dbpht2	0.005967	0.66621	0.87099	None
16563	SLC35F4	-0.00641	0.66627	0.87099	None
16564	PP2D1	0.006915	0.66627	0.87099	None
16565	UTS2B	-0.00138	0.666299	0.87099	None
16566	TRAPPC10	0.093201	0.666305	0.87099	None
16567	TFF1	0.021868	0.666393	0.871052	None
16568	ATG13	0.054827	0.666432	0.871052	None
16569	CRYGD	-0.13823	0.666561	0.871168	None
16570	SPACA1	-0.02298	0.666629	0.871204	None
16571	BPESC1	-0.00093	0.666679	0.871217	None
16572	PPP2R2A	0.083643	0.666769	0.871234	None
16573	COL14A1	-0.00721	0.666773	0.871234	None
16574	LINC01354	-0.00639	0.666888	0.871332	None
16575	LOC10012	-0.0009	0.667116	0.871577	None
16576	FAM189A2	-0.00071	0.667192	0.871605	None
16577	HIST1H4J	0.091596	0.667239	0.871605	None
16578	RNF165	-0.02123	0.667267	0.871605	None
16579	IGFBP7	-0.08986	0.667331	0.871605	None
16580	GRID2	-0.00181	0.667338	0.871605	None
16581	TMED4	0.08819	0.667412	0.871613	None
16582	DIABLO	0.046392	0.667458	0.871613	None
16583	TSPY1	-0.00983	0.667482	0.871613	None
16584	LOC10192	-0.00111	0.667536	0.871613	None
16585	TLX1	-0.00452	0.667547	0.871613	None
16586	TRAV8-3	-0.00466	0.667586	0.871613	None

16587	SHOX2	-0.00337	0.667643	0.871635	None
16588	NSL1	-0.04077	0.667764	0.87174	None
16589	TM9SF1	-0.04006	0.667896	0.871817	None
16590	SPOP	-0.06975	0.66795	0.871817	None
16591	LOC10192!	-0.00079	0.667982	0.871817	None
16592	BMP15	-0.00197	0.668001	0.871817	None
16593	RIOK2	0.065683	0.668089	0.871817	None
16594	RAB24	-0.07086	0.668119	0.871817	None
16595	LOC10192!	-0.01653	0.668177	0.871817	None
16596	HYPM	-0.00229	0.668206	0.871817	None
16597	C9orf89	-0.04343	0.668212	0.871817	None
16598	KIAA0355	-0.10176	0.668225	0.871817	None
16599	LMO3	-0.00119	0.668344	0.871829	None
16600	SPACA6P	-0.01436	0.668357	0.871829	None
16601	LOC10012	0.003649	0.668382	0.871829	None
16602	LOC10012!	-0.04258	0.668435	0.871829	None
16603	FGA	-0.0025	0.668488	0.871829	None
16604	USP47	-0.05925	0.668515	0.871829	None
16605	MRPS5	-0.06244	0.668516	0.871829	None
16606	CECR3	-0.0018	0.668574	0.871848	None
16607	LOC10013	-0.01272	0.668646	0.871848	None
16608	GDI2	-0.04924	0.668652	0.871848	None
16609	TMEM235	-0.00292	0.668706	0.871848	None
16610	LOC10192	0.001617	0.668732	0.871848	None
16611	BRINP2	-0.00978	0.668811	0.871863	None
16612	SPDL1	0.069148	0.668864	0.871863	None
16613	ZNF384	-0.02578	0.668932	0.871863	None
16614	LAPTM4B	-0.10832	0.668947	0.871863	None
16615	RP4-584D	-0.00113	0.668984	0.871863	None
16616	LINC00639	-0.00228	0.66902	0.871863	None
16617	BC069739	-0.01689	0.669026	0.871863	None
16618	NDN	0.158595	0.669188	0.87199	None
16619	SLC8A2	-0.0012	0.669203	0.87199	None
16620	GTF2B	0.057485	0.669279	0.872036	None
16621	TBCC	0.05151	0.669324	0.872041	None
16622	MGC39545	-0.00348	0.66937	0.87205	None
16623	PELI1	-0.11885	0.66948	0.872069	None
16624	LOC64365!	-0.00104	0.669506	0.872069	None
16625	COL9A1	-0.002	0.669563	0.872069	None
16626	LOC10050	-0.00206	0.669587	0.872069	None
16627	ELOVL3	-0.00113	0.66965	0.872069	None
16628	LOC28509!	-0.00384	0.669681	0.872069	None
16629	ACBD6	-0.04694	0.6697	0.872069	None
16630	LOC64275	-0.00526	0.669793	0.872069	None
16631	TSPAN1	-0.01609	0.669805	0.872069	None
16632	RP11-215H	-0.00159	0.669824	0.872069	None
16633	AC073321.	-0.00697	0.669842	0.872069	None
16634	ZNF296	-0.0458	0.669869	0.872069	None
16635	KCNV2	-0.00186	0.669958	0.872134	None
16636	TRPM1	-0.0009	0.670173	0.872305	None
16637	C21orf62	0.007398	0.670265	0.872305	None
16638	IL13RA2	-0.10537	0.670267	0.872305	None
16639	PMCHL2	-0.00395	0.67029	0.872305	None
16640	SBSN	-0.00118	0.670294	0.872305	None
16641	LINC00643	-0.00141	0.670331	0.872305	None
16642	SACS	0.061651	0.670372	0.872305	None
16643	BC045784	-0.01945	0.670437	0.872338	None
16644	LOC14679!	0.003521	0.670519	0.872392	None

16645	RP11-284F	-0.00146	0.670602	0.872448	None
16646	LOC10050(	-0.00181	0.670658	0.872468	None
16647	LINC0114C	0.028921	0.670756	0.872476	None
16648	PRX	-0.02356	0.670764	0.872476	None
16649	CPNE1	0.087956	0.670785	0.872476	None
16650	LOC10192!	0.01892	0.670914	0.872591	None
16651	RELB	0.099899	0.670967	0.872597	None
16652	ERCC1	-0.05307	0.670999	0.872597	None
16653	BC069756	-0.0125	0.671221	0.872631	None
16654	AK074476	0.015114	0.671232	0.872631	None
16655	DNAJB8-A	-0.00273	0.671301	0.872631	None
16656	GCSAML-/	0.174238	0.67135	0.872631	None
16657	ASB11	-0.00129	0.671389	0.872631	None
16658	ARL14	-0.00354	0.671442	0.872631	None
16659	RP5-1027C	-0.01298	0.671494	0.872631	None
16660	NMUR2	0.006986	0.671515	0.872631	None
16661	GGTLC2	0.024784	0.671551	0.872631	None
16662	LOC10192!	-0.012	0.671579	0.872631	None
16663	ZNF205	-0.06502	0.671608	0.872631	None
16664	C9	-0.01004	0.671639	0.872631	None
16665	TMEM134	0.007071	0.671642	0.872631	None
16666	BTBD18	-0.0417	0.671649	0.872631	None
16667	CHAF1A	-0.09831	0.671657	0.872631	None
16668	CDC14C	-0.00101	0.671706	0.872631	None
16669	SLFN11	-0.06892	0.671714	0.872631	None
16670	LOC10050(	-0.0032	0.67175	0.872631	None
16671	RP3-337H4	0.020885	0.67185	0.872682	None
16672	STARD3	-0.06824	0.671914	0.872682	None
16673	LOC10050(	-0.00095	0.672021	0.872682	None
16674	HMHA1	-0.03299	0.672023	0.872682	None
16675	RP11-399C	-0.00205	0.672025	0.872682	None
16676	LINC00559	-0.00586	0.672099	0.872682	None
16677	LINC00305	-0.00313	0.672101	0.872682	None
16678	LINC00319	-0.00342	0.67215	0.872682	None
16679	SP4	0.044771	0.672174	0.872682	None
16680	RP11-486/	0.003643	0.672226	0.872682	None
16681	LOC40132(	-0.04503	0.672245	0.872682	None
16682	CLCA4	-0.00082	0.672301	0.872682	None
16683	FERD3L	-0.00435	0.672374	0.872682	None
16684	SYNC	-0.11619	0.672382	0.872682	None
16685	RP11-753/	-0.00411	0.672404	0.872682	None
16686	ZNF282	-0.06605	0.672434	0.872682	None
16687	IFNA2	-0.00147	0.672514	0.872732	None
16688	STX5	-0.06706	0.672662	0.872744	None
16689	SFRP1	-0.00156	0.672731	0.872744	None
16690	APOBEC3F	-0.02935	0.672769	0.872744	None
16691	RP11-186F	-0.00769	0.672793	0.872744	None
16692	LINC0133C	-0.00264	0.67281	0.872744	None
16693	GPR156	0.010339	0.672921	0.872744	None
16694	RNF169	0.038329	0.672929	0.872744	None
16695	SMAD6	-0.01347	0.672946	0.872744	None
16696	RP11-285J	-0.05628	0.672977	0.872744	None
16697	OR5I1	-0.00354	0.673029	0.872744	None
16698	PPY2	-0.00151	0.673037	0.872744	None
16699	KDSR	0.095157	0.673124	0.872744	None
16700	CCDC102B	-0.037	0.673151	0.872744	None
16701	4-Mar	-0.01093	0.673177	0.872744	None
16702	LINC00883	0.053137	0.673223	0.872744	None

16703	ITCH	0.058497	0.67325	0.872744	None
16704	LOC10192‹	0.026663	0.67327	0.872744	None
16705	PCDHB9	-0.00362	0.673306	0.872744	None
16706	SOX3	-0.0043	0.673365	0.872744	None
16707	CELF5	-0.01124	0.673378	0.872744	None
16708	PLCXD3	-0.0119	0.673379	0.872744	None
16709	NEBL	-0.00084	0.673409	0.872744	None
16710	NFKBIZ	-0.08833	0.673453	0.872749	None
16711	ITGA11	-0.00058	0.67355	0.872822	None
16712	DCTN3	0.045748	0.673686	0.872914	None
16713	IYD	-0.0055	0.673702	0.872914	None
16714	FAM160B2	-0.02266	0.673778	0.872952	None
16715	TCTEX1D1	-0.16529	0.673812	0.872952	None
16716	AADACL2	-0.00205	0.674044	0.873097	None
16717	DFNB59	-0.07159	0.674046	0.873097	None
16718	CCR10	-0.03015	0.67407	0.873097	None
16719	FAM73A	-0.05899	0.674085	0.873097	None
16720	NDST2	0.040513	0.674226	0.873228	None
16721	SNRK-AS1	-0.06536	0.674347	0.873332	None
16722	GBE1	0.041715	0.674394	0.87334	None
16723	ASB17	-0.00125	0.674525	0.873373	None
16724	STXBP5-A‹	-0.00123	0.674625	0.873373	None
16725	DVL3	0.030446	0.674633	0.873373	None
16726	COX8C	-0.00171	0.674657	0.873373	None
16727	LOC44108‹	-0.00426	0.674674	0.873373	None
16728	SLITRK1	-0.00247	0.674688	0.873373	None
16729	CYP3A43	-0.00428	0.674701	0.873373	None
16730	C17orf67	-0.03901	0.675005	0.873713	None
16731	LOC10028‹	-0.01658	0.67505	0.873719	None
16732	SPINK4	-0.01662	0.675101	0.873734	None
16733	VAX2	-0.0076	0.675315	0.873771	None
16734	TMEM254-	-0.02887	0.675347	0.873771	None
16735	SLC20A2	0.028531	0.675348	0.873771	None
16736	FGD6	-0.0237	0.675383	0.873771	None
16737	CTSE	0.091574	0.675417	0.873771	None
16738	RP1-35C2‹	-0.00388	0.675436	0.873771	None
16739	RP4-676L2	-0.0018	0.675483	0.873771	None
16740	CDKN3	0.122869	0.675506	0.873771	None
16741	LINC0092C	0.025646	0.675512	0.873771	None
16742	PRMT6	0.08403	0.675582	0.873771	None
16743	ZSCAN23	0.015145	0.675593	0.873771	None
16744	RACGAP1F	-0.00272	0.675633	0.873771	None
16745	CNTN6	-0.00696	0.675683	0.873771	None
16746	RP1-192P‹	0.001447	0.675694	0.873771	None
16747	NTRK3-AS	-0.00163	0.675784	0.873827	None
16748	AKR1D1	-0.00073	0.675819	0.873827	None
16749	MUC4	-0.00565	0.675964	0.873915	None
16750	OR8D1	-0.00691	0.676005	0.873915	None
16751	LOC28351‹	-0.00493	0.676008	0.873915	None
16752	TACC3	-0.05847	0.676109	0.873966	None
16753	SPATA16	-0.01178	0.676165	0.873966	None
16754	LINC01281	-0.00248	0.676168	0.873966	None
16755	HNRNPM	-0.09034	0.676241	0.874003	None
16756	GABRA1	-0.01481	0.676407	0.874003	None
16757	AF131215.‹	-0.05538	0.676426	0.874003	None
16758	SMDT1	-0.06849	0.676461	0.874003	None
16759	COL5A3	0.004307	0.676492	0.874003	None
16760	DRP2	-0.00333	0.676496	0.874003	None

16761	RNASE1	-0.10872	0.676509	0.874003	None
16762	GPR61	-0.00153	0.676543	0.874003	None
16763	FAM222A-	-0.00145	0.676665	0.874003	None
16764	LOC10012[illegible]	-0.01524	0.676722	0.874003	None
16765	TPBGL	-0.01084	0.676732	0.874003	None
16766	CABLES2	-0.05944	0.67675	0.874003	None
16767	SERPINF1	0.122531	0.676752	0.874003	None
16768	CH25H	-0.04775	0.676829	0.874003	None
16769	CMSS1	-0.06059	0.676839	0.874003	None
16770	ISX	-0.00475	0.676847	0.874003	None
16771	BMS1P6	0.095792	0.676883	0.874003	None
16772	RAPGEF6	0.089021	0.676935	0.874018	None
16773	TRAV12-2	-0.0123	0.676996	0.874039	None
16774	AX747261	-0.01351	0.677032	0.874039	None
16775	EVPLL	0.01047	0.677072	0.874039	None
16776	LOC28504[illegible]	-0.0014	0.677221	0.874146	None
16777	RP11-324L	-0.00496	0.677236	0.874146	None
16778	LPL	0.168895	0.677345	0.874234	None
16779	RP11-203E	-0.01094	0.677388	0.874238	None
16780	FAM9C	0.006441	0.677475	0.874279	None
16781	LOC38924[illegible]	0.007922	0.6775	0.874279	None
16782	HYDIN	-0.00424	0.677683	0.87445	None
16783	C19orf68	0.056756	0.677753	0.87445	None
16784	MB21D1	-0.07984	0.677819	0.87445	None
16785	SNRPN	-0.00759	0.677834	0.87445	None
16786	IGFBP7-AS	-0.06165	0.677835	0.87445	None
16787	C20orf197	0.141949	0.678038	0.874576	None
16788	PPP2R3C	0.098544	0.678052	0.874576	None
16789	DCLRE1C	-0.0651	0.678054	0.874576	None
16790	PCMTD2	-0.07015	0.678275	0.874804	None
16791	TSC22D3	0.062714	0.678311	0.874804	None
16792	RP5-1103[illegible]	-0.00206	0.678367	0.874814	None
16793	ARHGEF33	-0.00711	0.6784	0.874814	None
16794	MC5R	-0.00133	0.678455	0.874832	None
16795	LOC10192[illegible]	-0.0026	0.678527	0.874873	None
16796	TRAIP	-0.07622	0.678621	0.874902	None
16797	RNF185-A[illegible]	-0.00272	0.678733	0.874902	None
16798	HHLA2	-0.00625	0.678811	0.874902	None
16799	YTHDF3-A	0.032426	0.678828	0.874902	None
16800	FGD5	0.072715	0.678894	0.874902	None
16801	LTN1	-0.08147	0.678905	0.874902	None
16802	SIRT5	-0.05266	0.678949	0.874902	None
16803	LOC10050[illegible]	-0.00163	0.678975	0.874902	None
16804	LOC44057[illegible]	-0.00973	0.678978	0.874902	None
16805	P2RY10	0.043408	0.67898	0.874902	None
16806	RP6-149D[illegible]	0.00349	0.678995	0.874902	None
16807	TMEM178[illegible]	0.008431	0.679034	0.874902	None
16808	NT5E	0.031081	0.67911	0.874942	None
16809	RP11-443E	0.040142	0.679146	0.874942	None
16810	ABRA	-0.00404	0.679275	0.875056	None
16811	BRPF1	-0.05179	0.679359	0.875113	None
16812	GRP	-0.0018	0.679504	0.87518	None
16813	LOC10192[illegible]	-0.00253	0.679505	0.87518	None
16814	LOC10192[illegible]	-0.00561	0.679561	0.87518	None
16815	CXCL3	0.184079	0.679575	0.87518	None
16816	C7orf73	-0.06029	0.679634	0.87518	None
16817	CBLB	0.065553	0.679663	0.87518	None
16818	CCDC30	-0.01068	0.679725	0.87518	None

16819	LINC00529	0.000764	0.679734	0.87518	None
16820	UPK1A	-0.01683	0.679811	0.875188	None
16821	FAM107A	-0.01788	0.679822	0.875188	None
16822	UGDH	-0.07691	0.679904	0.875242	None
16823	HSPA9	-0.06625	0.68002	0.875268	None
16824	EPN2-AS1	0.009197	0.680044	0.875268	None
16825	OR2J3	-0.00082	0.680147	0.875268	None
16826	FGFBP1	0.007052	0.680189	0.875268	None
16827	PP14571	-0.00718	0.680208	0.875268	None
16828	SPATS1	0.012853	0.680227	0.875268	None
16829	OTOF	-0.00219	0.680317	0.875268	None
16830	NIP7	0.057622	0.680354	0.875268	None
16831	MCF2	-0.00073	0.680387	0.875268	None
16832	LOC10192	0.003008	0.680518	0.875268	None
16833	LOC10192	0.003927	0.680533	0.875268	None
16834	PASK	0.065039	0.680569	0.875268	None
16835	LOC10050	-0.00541	0.680615	0.875268	None
16836	HCRTR2	-0.00346	0.680621	0.875268	None
16837	CD28	-0.00147	0.680658	0.875268	None
16838	LOC10192	0.101383	0.680715	0.875268	None
16839	LINC00242	-0.00456	0.680717	0.875268	None
16840	TMCC1-AS	0.001811	0.680788	0.875268	None
16841	NDUFA4L2	-0.0073	0.680789	0.875268	None
16842	SOHLH2	-0.00299	0.680817	0.875268	None
16843	LOC28348	-0.0041	0.680834	0.875268	None
16844	PRUNE2	0.044825	0.680872	0.875268	None
16845	PSMB6	0.060408	0.680873	0.875268	None
16846	SNAI2	0.072986	0.680894	0.875268	None
16847	CETN1	-0.0097	0.680962	0.87528	None
16848	AKAP9	0.10375	0.681025	0.87528	None
16849	IGDCC4	-0.00274	0.681034	0.87528	None
16850	GS1-111G	-0.07523	0.681065	0.87528	None
16851	SLC7A13	-0.00132	0.681148	0.875335	None
16852	NXPH4	-0.0111	0.681193	0.875341	None
16853	EDN1	0.019855	0.681265	0.875344	None
16854	PTPN21	-0.0013	0.681277	0.875344	None
16855	AGFG2	0.001768	0.681458	0.875525	None
16856	GOT1	-0.06997	0.681662	0.875691	None
16857	WBP5	0.083973	0.681747	0.875691	None
16858	SLC35D2	-0.05545	0.681772	0.875691	None
16859	LOC10192	-0.00089	0.68178	0.875691	None
16860	ATP13A3	0.06078	0.681789	0.875691	None
16861	LOC10192	-0.00269	0.682013	0.875923	None
16862	LOC10192	0.004227	0.682051	0.875923	None
16863	GLOD4	0.045535	0.682341	0.876133	None
16864	RP11-680	0.080367	0.68236	0.876133	None
16865	MYO3B	-0.00376	0.682381	0.876133	None
16866	PILRA	-0.06503	0.682403	0.876133	None
16867	TM4SF20	0.013162	0.682423	0.876133	None
16868	GALR2	-0.0022	0.682459	0.876133	None
16869	VMA21	-0.01691	0.682521	0.876133	None
16870	RSL1D1	0.0557	0.682538	0.876133	None
16871	AQP8	-0.00183	0.682719	0.876313	None
16872	PDHA1	-0.05053	0.682858	0.876406	None
16873	LINC00470	-0.01237	0.682899	0.876406	None
16874	AC004941.	0.060375	0.682913	0.876406	None
16875	GPR27	-0.0883	0.682961	0.876406	None
16876	PCDHB4	-0.01592	0.682993	0.876406	None

16877	RDH12	-0.00653	0.683135	0.876474	None
16878	GSC2	-0.00155	0.683177	0.876474	None
16879	BREA2	-0.0062	0.683206	0.876474	None
16880	VAPA	0.067043	0.683208	0.876474	None
16881	RP1-142L7	0.012477	0.683251	0.876477	None
16882	KRTAP4-7	-0.00072	0.683438	0.876614	None
16883	LOC10192[illegible]	-0.00192	0.683441	0.876614	None
16884	HIST1H1E	0.097393	0.68348	0.876614	None
16885	RP11-474F	-0.01353	0.68359	0.876658	None
16886	PNPLA1	-0.00953	0.683595	0.876658	None
16887	SHB	-0.03099	0.683639	0.876663	None
16888	LKAAEAR1	-0.01723	0.683835	0.876707	None
16889	DUOXA1	-0.00705	0.683859	0.876707	None
16890	P2RY4	-0.00399	0.683865	0.876707	None
16891	LA16c-395	-0.00265	0.683903	0.876707	None
16892	LOC10192[illegible]	-0.00103	0.683917	0.876707	None
16893	LYRM9	-0.04254	0.683971	0.876707	None
16894	MCHR2-A[illegible]	-0.00866	0.683975	0.876707	None
16895	PNLIP	-0.00095	0.683997	0.876707	None
16896	LINC01139	-0.00611	0.684219	0.876939	None
16897	PROK2	-0.26783	0.684645	0.877329	None
16898	RP11-15P1	0.005576	0.684665	0.877329	None
16899	UROC1	0.00295	0.684687	0.877329	None
16900	DUSP12	-0.0339	0.684723	0.877329	None
16901	LOC40079[illegible]	0.008538	0.684726	0.877329	None
16902	LOC10192[illegible]	0.10864	0.684946	0.877521	None
16903	LOC10192[illegible]	-0.00075	0.684957	0.877521	None
16904	FOXH1	0.009366	0.685148	0.877529	None
16905	AZIN2	-0.00712	0.685189	0.877529	None
16906	LOC10050[illegible]	-0.00704	0.685196	0.877529	None
16907	LINC01312	0.016138	0.685219	0.877529	None
16908	LOC10192[illegible]	-0.05215	0.685261	0.877529	None
16909	RHNO1	-0.05371	0.685267	0.877529	None
16910	PLA2G1B	-0.02936	0.685352	0.877529	None
16911	LOC10192[illegible]	-0.00383	0.685409	0.877529	None
16912	TEX11	-0.00205	0.685414	0.877529	None
16913	VCAN	0.203155	0.68543	0.877529	None
16914	ANK2	-0.00068	0.685493	0.877529	None
16915	IGLJ3	-0.12456	0.685504	0.877529	None
16916	MDGA2	-0.02074	0.685556	0.877529	None
16917	NUDT19	-0.082	0.685598	0.877529	None
16918	GMEB2	-0.08271	0.685612	0.877529	None
16919	TUFM	-0.05533	0.685653	0.877529	None
16920	RP11-410D	-0.00581	0.685677	0.877529	None
16921	FAM228A	-0.01206	0.685699	0.877529	None
16922	BACH1	-0.00771	0.685733	0.877529	None
16923	GABRA6	-0.0012	0.685852	0.877578	None
16924	C10orf71	-0.01845	0.685853	0.877578	None
16925	SLC39A7	0.07724	0.68598	0.877636	None
16926	JUP	0.125458	0.686021	0.877636	None
16927	PTGIS	-0.00248	0.686054	0.877636	None
16928	TMEM106[illegible]	0.06934	0.68606	0.877636	None
16929	SECISBP2	-0.03462	0.686119	0.87766	None
16930	KIR2DS2	0.004919	0.686166	0.877668	None
16931	CHRNA3	-0.00081	0.686248	0.877721	None
16932	DNAI1	0.006603	0.686402	0.877783	None
16933	ALPK2	-0.00322	0.686419	0.877783	None
16934	LINC0021[illegible]	-0.00248	0.686447	0.877783	None

16935	SPPL2C	-0.00711	0.686458	0.877783 None
16936	KRT17P5	-0.00188	0.686655	0.877983 None
16937	LOC10192	-0.0029	0.686779	0.878089 None
16938	LUM	0.103095	0.686969	0.878237 None
16939	ZBTB49	0.038266	0.687074	0.878237 None
16940	FSCN2	-0.00103	0.687079	0.878237 None
16941	TLR10	-0.01946	0.687079	0.878237 None
16942	RP11-960E	0.00172	0.687097	0.878237 None
16943	ABCG8	0.004298	0.687275	0.878413 None
16944	RP11-532F	0.040861	0.687318	0.878416 None
16945	FRRS1	-0.00092	0.687397	0.878433 None
16946	TIMM23	-0.00176	0.687451	0.878433 None
16947	EGFLAM	-0.03486	0.687453	0.878433 None
16948	PAK1IP1	-0.06727	0.68761	0.878533 None
16949	TGM7	-0.00748	0.687631	0.878533 None
16950	SLC9A5	0.009622	0.687669	0.878533 None
16951	TECRL	-0.00421	0.687737	0.878533 None
16952	ATPAF2	0.042384	0.687803	0.878533 None
16953	C1orf158	-0.00377	0.687825	0.878533 None
16954	ITGAD	-0.10705	0.687844	0.878533 None
16955	LOC10012	-0.00101	0.68789	0.878533 None
16956	SEC13	-0.05285	0.687949	0.878533 None
16957	S100A8	0.180536	0.687953	0.878533 None
16958	RP1-93H1	-0.1038	0.687978	0.878533 None
16959	NEBL-AS1	-0.00687	0.688022	0.878537 None
16960	CXCL13	-0.00246	0.688084	0.878564 None
16961	GON4L	-0.04176	0.688205	0.878667 None
16962	LINC0091C	-0.00387	0.688419	0.878889 None
16963	TOR1A	-0.05271	0.6885	0.878937 None
16964	RASSF9	0.040967	0.688538	0.878937 None
16965	MAATS1	0.00055	0.688725	0.879074 None
16966	RPTN	-0.00126	0.688755	0.879074 None
16967	CMPK1	0.045899	0.688821	0.879074 None
16968	WBSCR28	-0.00062	0.688896	0.879074 None
16969	RP11-513N	0.011121	0.688919	0.879074 None
16970	YTHDF3	0.060605	0.688931	0.879074 None
16971	LOC28388	0.020139	0.688966	0.879074 None
16972	C6orf99	-0.00486	0.68897	0.879074 None
16973	MYO19	0.072286	0.689058	0.879134 None
16974	STATH	-0.00094	0.689226	0.879258 None
16975	ASIC5	-0.00101	0.689267	0.879258 None
16976	RP4-665N	0.005624	0.689277	0.879258 None
16977	C9orf163	-0.01052	0.689402	0.879367 None
16978	FGF14-AS	0.064733	0.689541	0.879452 None
16979	CTSS	-0.09182	0.689575	0.879452 None
16980	PCDHB11	-0.0023	0.689591	0.879452 None
16981	HOGA1	-0.00158	0.689687	0.879522 None
16982	BOK	-0.02275	0.689804	0.879553 None
16983	CACNG3	0.008239	0.689812	0.879553 None
16984	STKLD1	-0.0038	0.689833	0.879553 None
16985	BMF	0.072926	0.689885	0.879568 None
16986	FAM198A	0.001568	0.69021	0.879774 None
16987	FXYD4	-0.00112	0.690234	0.879774 None
16988	CLEC1B	-0.21918	0.690272	0.879774 None
16989	EML6	-0.04689	0.690274	0.879774 None
16990	MARCO	0.120532	0.690311	0.879774 None
16991	DLX6-AS1	-0.00819	0.690383	0.879774 None
16992	DAOA-AS	-0.00303	0.690457	0.879774 None

16993	BZW2	-0.04057	0.690505	0.879774	None
16994	AC136289.	0.012174	0.690514	0.879774	None
16995	ADAMTS18	-0.01774	0.690602	0.879774	None
16996	LOC10050	-0.00071	0.690612	0.879774	None
16997	SUCLG2-A	0.006929	0.690695	0.879774	None
16998	EYA1	-0.03964	0.690724	0.879774	None
16999	SLC4A10	-0.00997	0.690745	0.879774	None
17000	OR8D2	-0.01135	0.69076	0.879774	None
17001	PRF1	-0.07253	0.690768	0.879774	None
17002	CORO7	-0.0446	0.690832	0.879774	None
17003	KIR3DL3	-0.00828	0.690838	0.879774	None
17004	OR2W1	-0.00084	0.690891	0.879774	None
17005	CC2D1B	-0.0227	0.690921	0.879774	None
17006	IL2	-0.00199	0.690958	0.879774	None
17007	CCDC79	-0.00122	0.691005	0.879774	None
17008	PRSS22	0.0107	0.691055	0.879774	None
17009	KCNJ11	-0.00371	0.691065	0.879774	None
17010	LOC72817	-0.00168	0.691162	0.879774	None
17011	DEFB122	-0.00275	0.691173	0.879774	None
17012	CACNA1D	-0.03779	0.691183	0.879774	None
17013	ATP4B	-0.00149	0.691185	0.879774	None
17014	FAM98B	-0.10334	0.69127	0.879783	None
17015	LOC10192	0.070276	0.691273	0.879783	None
17016	ESRRG	-0.01417	0.691375	0.879807	None
17017	LINC00841	-0.00113	0.691398	0.879807	None
17018	SASH3	0.078079	0.691413	0.879807	None
17019	EPHB2	0.008545	0.691481	0.879813	None
17020	DSCC1	0.041787	0.691562	0.879813	None
17021	C1orf167	0.010042	0.691565	0.879813	None
17022	LOC10013	-0.00819	0.691643	0.879813	None
17023	PDE5A	-0.03108	0.691704	0.879813	None
17024	UPP2	0.002238	0.691754	0.879813	None
17025	RASSF1	-0.06308	0.691779	0.879813	None
17026	TOM1	-0.04521	0.691782	0.879813	None
17027	TBC1D32	-0.0693	0.691784	0.879813	None
17028	HYALP1	-0.00196	0.691885	0.879881	None
17029	CDC42BPG	-0.00442	0.691918	0.879881	None
17030	INHBB	-0.00404	0.692035	0.879936	None
17031	RP11-353N	-0.00402	0.692079	0.879936	None
17032	GNA11	-0.04587	0.692111	0.879936	None
17033	LINC00933	-0.00071	0.692124	0.879936	None
17034	NDUFB6	-0.03964	0.692197	0.879972	None
17035	ITFG2	0.041308	0.692254	0.879972	None
17036	TM2D3	-0.06003	0.692275	0.879972	None
17037	CTA-331P3	0.08585	0.692346	0.880011	None
17038	HIF3A	-0.00906	0.692474	0.880012	None
17039	ZSCAN2	0.044429	0.692475	0.880012	None
17040	LOC10192	-0.00104	0.692513	0.880012	None
17041	GLRX3	-0.0397	0.692515	0.880012	None
17042	SLIT2-IT1	-0.00077	0.69255	0.880012	None
17043	PPEF2	-0.00316	0.692608	0.880035	None
17044	SORCS3	-0.00101	0.6927	0.880035	None
17045	LOC10192	0.018562	0.692725	0.880035	None
17046	ITGA10	-0.00793	0.692731	0.880035	None
17047	KDM7A	-0.10151	0.692778	0.880044	None
17048	PRTFDC1	-0.10982	0.692825	0.880052	None
17049	LOC10106	0.06307	0.693244	0.880533	None
17050	OR2F2	-0.00275	0.693331	0.880592	None

17051	SLC8A1	-0.00454	0.693523	0.88078	None
17052	CRYBA4	-0.00212	0.693565	0.88078	None
17053	PIEZO2	0.002553	0.693644	0.88078	None
17054	AURKC	-0.03641	0.693655	0.88078	None
17055	NPFFR2	-0.001	0.693683	0.88078	None
17056	DCP2	-0.07476	0.693787	0.88086	None
17057	AGPAT2	0.072996	0.693831	0.880864	None
17058	ERVMER61	-0.0006	0.693973	0.880994	None
17059	NTRK2	-0.0006	0.694064	0.881057	None
17060	PTPRB	-0.01181	0.694131	0.881091	None
17061	BEGAIN	-0.01418	0.694199	0.881125	None
17062	ENTPD3	-0.00432	0.694263	0.881155	None
17063	GRIK1-AS1	0.005174	0.694607	0.88154	None
17064	RP11-715J	0.027662	0.694714	0.881583	None
17065	LOC10050!	-0.0105	0.694722	0.881583	None
17066	IFNA8	-0.00122	0.694856	0.881635	None
17067	SPAG7	-0.04174	0.694864	0.881635	None
17068	FHDC1	-0.11341	0.694886	0.881635	None
17069	CDCA5	0.096843	0.694978	0.881701	None
17070	ST7-OT4	-0.00141	0.695155	0.881823	None
17071	PABPC5	-0.00088	0.695163	0.881823	None
17072	RP11-347D	-0.00082	0.695196	0.881823	None
17073	LOC10192!	-0.01851	0.695249	0.881837	None
17074	GDAP2	-0.04158	0.695327	0.88185	None
17075	LOC10192	-0.00082	0.69534	0.88185	None
17076	SPRR2C	-0.00565	0.695604	0.882133	None
17077	LOC10028	-0.00879	0.695668	0.882154	None
17078	SLC22A14	-0.01808	0.695702	0.882154	None
17079	LZTS1-AS1	-0.00569	0.695829	0.882263	None
17080	JAGN1	-0.05106	0.69588	0.882276	None
17081	ALDH1A2	-0.01137	0.696091	0.882376	None
17082	CAPZA3	-0.00139	0.696101	0.882376	None
17083	DDX41	-0.05142	0.696168	0.882376	None
17084	LINC00703	-0.00079	0.696199	0.882376	None
17085	HAVCR2	-0.03772	0.696226	0.882376	None
17086	HSPA5	0.066775	0.69623	0.882376	None
17087	FNDC5	-0.01409	0.696266	0.882376	None
17088	BTF3P11	-0.00671	0.696284	0.882376	None
17089	RALY	-0.06084	0.69637	0.882419	None
17090	GATSL2	0.051894	0.696416	0.882419	None
17091	DAB1	-0.02171	0.696467	0.882419	None
17092	RP11-151E	-0.00238	0.696497	0.882419	None
17093	EID2B	-0.07478	0.696562	0.882419	None
17094	HOXB1	0.005708	0.696563	0.882419	None
17095	RP11-255F	-0.004	0.696712	0.882556	None
17096	YRDC	-0.05409	0.69681	0.882628	None
17097	WDR62	-0.03391	0.696977	0.88266	None
17098	RAB12	-0.06199	0.697024	0.88266	None
17099	LMOD3	-0.00225	0.69703	0.88266	None
17100	IQCF4	-0.005	0.697076	0.88266	None
17101	COL18A1-	-0.00188	0.6971	0.88266	None
17102	PPP1R27	-0.03097	0.697125	0.88266	None
17103	ZNF154	0.028774	0.697134	0.88266	None
17104	RAP1A	-0.03992	0.697161	0.88266	None
17105	SHROOM3	-0.00064	0.697292	0.882748	None
17106	TSEN15	-0.08552	0.697312	0.882748	None
17107	LOC10013	-0.00202	0.697374	0.882749	None
17108	CLP1	0.024143	0.697394	0.882749	None

17109	LOC10050	-0.00411	0.697465	0.882787	None
17110	RP11-489E	-0.07181	0.697581	0.882861	None
17111	RHOB	-0.00085	0.697633	0.882861	None
17112	IARS2	-0.04638	0.697645	0.882861	None
17113	WNT10B	-0.03329	0.697756	0.882949	None
17114	POLR2E	-0.08522	0.697901	0.883	None
17115	RBMS2	-0.04386	0.697919	0.883	None
17116	SLC26A10	-0.00361	0.697966	0.883	None
17117	MCM3AP	-0.02106	0.69802	0.883	None
17118	LOC10013	0.000493	0.698029	0.883	None
17119	KB-1000E4	0.006839	0.698141	0.883	None
17120	CCT6A	-0.03869	0.698164	0.883	None
17121	LOC64409	0.006074	0.698305	0.883	None
17122	HSD11B1	-0.01364	0.69835	0.883	None
17123	PCDH17	0.113864	0.698361	0.883	None
17124	ZIM3	-0.00073	0.698426	0.883	None
17125	KCNG3	-0.0014	0.698432	0.883	None
17126	MSANTD2	0.07659	0.698451	0.883	None
17127	GNA14-AS	-0.00964	0.698458	0.883	None
17128	PDHB	-0.04538	0.698474	0.883	None
17129	LZTFL1	0.039482	0.698484	0.883	None
17130	RP11-510	-0.00069	0.698504	0.883	None
17131	AP001605.	-0.00245	0.698534	0.883	None
17132	ZNF28	-0.00832	0.698571	0.883	None
17133	DUSP21	-0.01884	0.698656	0.883021	None
17134	DKFZP434I	0.008583	0.698709	0.883021	None
17135	ANGPTL2	0.017347	0.698814	0.883021	None
17136	C11orf42	-0.00489	0.698821	0.883021	None
17137	SERPINA1	-0.09239	0.698823	0.883021	None
17138	MUC15	-0.00286	0.698866	0.883021	None
17139	ESYT3	-0.00202	0.698873	0.883021	None
17140	EPN3	-0.01096	0.698948	0.883027	None
17141	ARMC9	0.024157	0.69896	0.883027	None
17142	PARP10	-0.01398	0.699019	0.883042	None
17143	CECR5-AS	-0.00538	0.699057	0.883042	None
17144	SYT15	0.009375	0.699174	0.883042	None
17145	FIBP	-0.06119	0.699258	0.883042	None
17146	HP1BP3	0.041241	0.699285	0.883042	None
17147	FBXO44	0.017444	0.699303	0.883042	None
17148	FAM122B	0.073166	0.699366	0.883042	None
17149	TMPRSS13	0.017644	0.699371	0.883042	None
17150	MYH2	-0.01122	0.699552	0.883042	None
17151	LOC10192	0.013293	0.699608	0.883042	None
17152	MCM5	-0.06946	0.699654	0.883042	None
17153	CDC7	0.07519	0.699668	0.883042	None
17154	LINC01448	-0.00299	0.6997	0.883042	None
17155	RNF151	0.020548	0.699743	0.883042	None
17156	DYDC1	-0.00088	0.699788	0.883042	None
17157	RNF17	-0.00207	0.699794	0.883042	None
17158	BC035400	-0.0014	0.699823	0.883042	None
17159	ADARB2-A	-0.00191	0.699877	0.883042	None
17160	SLC38A9	0.063529	0.699884	0.883042	None
17161	PCDHGB5	0.010305	0.699923	0.883042	None
17162	GSX2	-0.00202	0.69996	0.883042	None
17163	CFHR3	-0.01493	0.700003	0.883042	None
17164	ANKRD13A	0.04585	0.700034	0.883042	None
17165	PRDX4	0.079359	0.700048	0.883042	None
17166	LINC00658	-0.00283	0.700062	0.883042	None

17167	RP11-115/	-0.00935	0.700097	0.883042	None
17168	DKFZp434I	-0.00101	0.700099	0.883042	None
17169	POLK	-0.0437	0.700113	0.883042	None
17170	C2orf69	0.059736	0.700223	0.883081	None
17171	KLHDC10	-0.0658	0.700226	0.883081	None
17172	FAM133A	0.098534	0.700352	0.883189	None
17173	TIGD2	-0.05701	0.700418	0.883214	None
17174	OTC	-0.00895	0.700453	0.883214	None
17175	PTPRJ	-0.0018	0.70055	0.883273	None
17176	COL17A1	-0.00177	0.700675	0.883273	None
17177	SHCBP1L	-0.01036	0.700686	0.883273	None
17178	C16orf93	0.055265	0.700696	0.883273	None
17179	SGCA	-0.00309	0.700704	0.883273	None
17180	SCN5A	-0.01217	0.700786	0.883325	None
17181	C21orf91-(	0.007756	0.700958	0.883453	None
17182	TTC39A	-0.0028	0.700969	0.883453	None
17183	C2CD4B	-0.01233	0.701025	0.883472	None
17184	BC070118	-0.00373	0.701157	0.883586	None
17185	C4orf6	0.001994	0.70139	0.88379	None
17186	GALNT2	0.04502	0.7014	0.88379	None
17187	SPACA7	-0.00265	0.701459	0.883814	None
17188	NAT2	-0.00497	0.701557	0.883885	None
17189	C15orf43	-0.00236	0.701754	0.883928	None
17190	LMTK3	0.024007	0.701785	0.883928	None
17191	RP11-153K	-0.00225	0.701812	0.883928	None
17192	H2BFXP	-0.04668	0.701838	0.883928	None
17193	EXTL1	0.017032	0.701888	0.883928	None
17194	PDE7B	0.005004	0.70194	0.883928	None
17195	ANKRD30/	-0.00287	0.701948	0.883928	None
17196	GLIPR1L1	0.003529	0.701967	0.883928	None
17197	LRRC3B	-0.02169	0.701989	0.883928	None
17198	TBCK	-0.05322	0.702004	0.883928	None
17199	DLX1	-0.01611	0.702074	0.883928	None
17200	LINC0070C	-0.00377	0.702081	0.883928	None
17201	OR7C2	-0.00069	0.702143	0.883955	None
17202	TEKT2	-0.02619	0.702224	0.884005	None
17203	LINC00935	-0.00238	0.702276	0.884013	None
17204	CNPY1	-0.00428	0.702355	0.884013	None
17205	MMP12	-0.00165	0.70238	0.884013	None
17206	NDNL2	-0.05271	0.702393	0.884013	None
17207	CACNA1F	-0.00252	0.702466	0.884019	None
17208	CLIP1	0.056892	0.702479	0.884019	None
17209	ATL3	-0.08018	0.70255	0.884056	None
17210	MBTD1	0.072408	0.702612	0.884059	None
17211	C4orf17	0.012817	0.702634	0.884059	None
17212	FBL	0.039624	0.702744	0.884089	None
17213	DTD1	0.038	0.702753	0.884089	None
17214	LOC10013(	-0.00178	0.702799	0.884089	None
17215	LOC10192(	-0.00339	0.702821	0.884089	None
17216	UBE2C	-0.10487	0.702914	0.884148	None
17217	LHFPL3	-0.00237	0.702949	0.884148	None
17218	SCAF4	0.073032	0.703147	0.884212	None
17219	TLR3	-0.06924	0.70319	0.884212	None
17220	FAIM2	-0.00797	0.703258	0.884212	None
17221	ZBTB11-AS	0.01699	0.703272	0.884212	None
17222	RP11-502N	-0.00137	0.703282	0.884212	None
17223	LOC10050!	-0.00205	0.703283	0.884212	None
17224	FZD10	-0.00088	0.70331	0.884212	None

17225	FLJ35816	-0.00158	0.703327	0.884212	None
17226	NUBP1	0.055367	0.70353	0.884387	None
17227	LOC64662(	0.028979	0.703548	0.884387	None
17228	TFR2	-0.06095	0.703708	0.884494	None
17229	NXPH1	-0.00396	0.703715	0.884494	None
17230	FAM26D	-0.00163	0.703961	0.884707	None
17231	LINC00919	-0.00301	0.703978	0.884707	None
17232	DIMT1	-0.04452	0.704007	0.884707	None
17233	RP3-406A7	-0.06058	0.70416	0.884848	None
17234	UG0898H0	-0.0091	0.704236	0.884851	None
17235	TMEM238	-0.00714	0.704244	0.884851	None
17236	RALYL	-0.00628	0.704326	0.884853	None
17237	IKZF1	0.104167	0.704338	0.884853	None
17238	ELMSAN1	0.046683	0.704431	0.884853	None
17239	BPIFB4	-0.005	0.704477	0.884853	None
17240	LOC10192	-0.00313	0.704483	0.884853	None
17241	GPR64	-0.00329	0.704502	0.884853	None
17242	ZYG11A	-0.00898	0.704572	0.884853	None
17243	HTR6	-0.00687	0.704573	0.884853	None
17244	DEFB114	-0.00187	0.704666	0.884892	None
17245	NEXN	-0.16747	0.704685	0.884892	None
17246	MIR663AH	-0.00102	0.704801	0.884934	None
17247	LINC01255	-0.01469	0.704841	0.884934	None
17248	KRTAP9-9	-0.00132	0.704842	0.884934	None
17249	XPO7	0.090265	0.704988	0.884956	None
17250	FN3KRP	-0.04986	0.705019	0.884956	None
17251	PCSK4	-0.0186	0.705038	0.884956	None
17252	MAGEA12	-0.00307	0.705103	0.884956	None
17253	CTB-78F1.1	-0.00158	0.705128	0.884956	None
17254	POLR2K	0.047321	0.705133	0.884956	None
17255	MGC70870	-0.10958	0.705185	0.884956	None
17256	ZCCHC10	0.068757	0.705195	0.884956	None
17257	OR9A1P	-0.0064	0.705284	0.884956	None
17258	INTS6-AS1	-0.05924	0.705324	0.884956	None
17259	KBTBD4	0.02968	0.705325	0.884956	None
17260	PSORS1C1	0.002608	0.705383	0.884956	None
17261	TRPC4	-0.00139	0.70539	0.884956	None
17262	TNPO3	0.048926	0.705535	0.885008	None
17263	PCDHB18	0.006508	0.705638	0.885008	None
17264	LOC10192	0.004145	0.70575	0.885008	None
17265	RP11-654A	-0.00506	0.705846	0.885008	None
17266	LRRC74	-0.01253	0.705899	0.885008	None
17267	GK3P	0.03766	0.705962	0.885008	None
17268	RIOK1	-0.03853	0.706024	0.885008	None
17269	RP11-661A	-0.00128	0.706042	0.885008	None
17270	LOC10192	-0.0006	0.706053	0.885008	None
17271	RP11-111M	-0.07951	0.706109	0.885008	None
17272	KRT33A	-0.00129	0.70611	0.885008	None
17273	NYAP2	-0.00234	0.706113	0.885008	None
17274	MCPH1	-0.04688	0.706152	0.885008	None
17275	MYO5B	-0.01232	0.706165	0.885008	None
17276	LINC00221	-0.00085	0.706179	0.885008	None
17277	PLA2G4F	-0.01642	0.706186	0.885008	None
17278	RTP5	-0.00321	0.706204	0.885008	None
17279	KLHL28	0.103134	0.706227	0.885008	None
17280	THOC2	-0.03612	0.706243	0.885008	None
17281	HOXB8	-0.01537	0.706249	0.885008	None
17282	C3orf79	-0.0026	0.706373	0.885088	None

17283	COMMD1	-0.06305	0.706476	0.885088	None
17284	IFNA17	-0.0016	0.706492	0.885088	None
17285	PRKG1-AS	-0.00143	0.706508	0.885088	None
17286	S100A3	-0.00233	0.706518	0.885088	None
17287	GH2	0.004999	0.70677	0.885154	None
17288	CADM3-A:	-0.00081	0.706788	0.885154	None
17289	LINC0135C	-0.00792	0.706877	0.885154	None
17290	C1orf210	-0.01061	0.706881	0.885154	None
17291	LOC55417.	0.011826	0.706919	0.885154	None
17292	TAS2R8	-0.00174	0.706947	0.885154	None
17293	ANKRD45	-0.02573	0.706968	0.885154	None
17294	RP11-295N	0.062263	0.706977	0.885154	None
17295	GNAT3	-0.00372	0.707003	0.885154	None
17296	LOC64151!	-0.00528	0.707005	0.885154	None
17297	UXT	-0.04955	0.707019	0.885154	None
17298	PNKP	-0.05929	0.707075	0.885173	None
17299	CSMD2	-0.00122	0.707193	0.885269	None
17300	C4orf47	-0.00407	0.707293	0.885342	None
17301	LRRC1	-0.03545	0.707337	0.885347	None
17302	PROL1	0.019793	0.707435	0.885407	None
17303	SOX14	-0.00068	0.707523	0.885407	None
17304	LINC0095C	0.000952	0.707604	0.885407	None
17305	UBXN7-AS	0.04179	0.707623	0.885407	None
17306	DNAJC7	0.054196	0.707629	0.885407	None
17307	INTS6	0.040675	0.707666	0.885407	None
17308	RP11-945A	-0.0008	0.707671	0.885407	None
17309	RP11-476D	-0.0007	0.707818	0.885464	None
17310	IL1R1	-0.08161	0.707826	0.885464	None
17311	CAMK2N1	-0.0017	0.70784	0.885464	None
17312	FANCD2	0.02536	0.707908	0.885499	None
17313	NCR3	-0.00277	0.708071	0.885569	None
17314	NPHP1	-0.0091	0.708182	0.885569	None
17315	H1FNT	-0.00075	0.70819	0.885569	None
17316	AC091133.	0.053751	0.708207	0.885569	None
17317	AC084219.	-0.00233	0.708225	0.885569	None
17318	MAP1LC3E	0.004387	0.708238	0.885569	None
17319	ARHGEF4	-0.00274	0.70825	0.885569	None
17320	ZFP57	-0.1613	0.708403	0.88565	None
17321	FA2H	-0.00644	0.708421	0.88565	None
17322	ZNF687	-0.01994	0.708439	0.88565	None
17323	KDM3A	0.022768	0.708572	0.88576	None
17324	PPY	-0.00171	0.708617	0.88576	None
17325	FAM196A	0.004682	0.708649	0.88576	None
17326	NOS2	-0.00377	0.708703	0.885777	None
17327	BCAS2	0.044784	0.70877	0.885799	None
17328	REG1B	-0.00095	0.708813	0.885799	None
17329	CPT1C	-0.01772	0.708941	0.885799	None
17330	LOC33959:	-0.00134	0.708949	0.885799	None
17331	NIN	-0.06168	0.708956	0.885799	None
17332	CRABP2	-0.01438	0.709006	0.885799	None
17333	SIM1	-0.01165	0.709008	0.885799	None
17334	LOC10099(	-0.0021	0.709048	0.885799	None
17335	PHOSPHO	-0.07572	0.709103	0.885816	None
17336	IPPK	0.016746	0.709383	0.886101	None
17337	LINC00403	-0.0008	0.709438	0.886101	None
17338	ELAVL3	-0.00256	0.709466	0.886101	None
17339	SYTL5	-0.00066	0.709495	0.886101	None
17340	LOC10192	-0.01011	0.709579	0.886156	None

17341	PCDHGC5	-0.00087	0.709739	0.886204	None
17342	LOC10028!	-0.00625	0.709762	0.886204	None
17343	DERA	-0.04464	0.709852	0.886204	None
17344	SP100	-0.05987	0.709885	0.886204	None
17345	IL3	0.001677	0.709891	0.886204	None
17346	LOC10050	-0.00412	0.709893	0.886204	None
17347	TMEM168	-0.04666	0.710043	0.886204	None
17348	LOC40065!	-0.01195	0.710094	0.886204	None
17349	SLC6A19	0.042219	0.710116	0.886204	None
17350	CDCP1	-0.01795	0.710123	0.886204	None
17351	LINC00113	-0.00084	0.710136	0.886204	None
17352	ZCCHC18	0.086648	0.710183	0.886204	None
17353	LOC10192!	-0.00614	0.710198	0.886204	None
17354	AP4E1	-0.00412	0.710237	0.886204	None
17355	GRTP1-AS	-0.0019	0.710287	0.886204	None
17356	HAP1	-0.00664	0.71033	0.886204	None
17357	FABP4	0.160297	0.710357	0.886204	None
17358	BC047364	0.011571	0.710391	0.886204	None
17359	RORA	-0.03489	0.710395	0.886204	None
17360	ZNF544	-0.03575	0.710472	0.886219	None
17361	MIR4500H	-0.00109	0.710535	0.886219	None
17362	HAUS6	0.08611	0.710542	0.886219	None
17363	ALG14	0.037193	0.710597	0.886219	None
17364	AQP11	-0.01096	0.710612	0.886219	None
17365	ESRP1	-0.01008	0.710681	0.886235	None
17366	MXRA7	-0.03308	0.710747	0.886235	None
17367	LOC40113	-0.04407	0.710749	0.886235	None
17368	NFX1	0.049074	0.71088	0.886235	None
17369	RP11-39H	0.003875	0.710901	0.886235	None
17370	CPT1A	-0.05802	0.710911	0.886235	None
17371	TMEM156	-0.07001	0.710953	0.886235	None
17372	RASSF1-A!	-0.02405	0.710969	0.886235	None
17373	MYT1L	-0.00062	0.710993	0.886235	None
17374	LOC14935	-0.00091	0.711038	0.88624	None
17375	NAGS	0.006963	0.711177	0.886362	None
17376	ISCA1	-0.05809	0.711261	0.886415	None
17377	CCDC89	-0.00653	0.711372	0.886503	None
17378	SH3TC2	-0.00331	0.711554	0.886679	None
17379	LOC41430	-0.00088	0.711639	0.886734	None
17380	LOC64242	0.003663	0.711757	0.886766	None
17381	ESF1	-0.05856	0.711779	0.886766	None
17382	MAML3	-0.01144	0.711788	0.886766	None
17383	LOC10192	-0.00163	0.712007	0.886948	None
17384	MAP10	0.008532	0.712016	0.886948	None
17385	OR8G1	-0.00263	0.712196	0.88699	None
17386	CSPG4P5	-0.00063	0.712208	0.88699	None
17387	STBD1	-0.0125	0.712245	0.88699	None
17388	SNX14	-0.04489	0.712285	0.88699	None
17389	RDH5	-0.08176	0.712319	0.88699	None
17390	WNT4	-0.00243	0.712328	0.88699	None
17391	C3orf14	0.096108	0.712336	0.88699	None
17392	LOR	-0.00087	0.712427	0.887013	None
17393	DHX36	0.035848	0.712437	0.887013	None
17394	ATP9B	0.011841	0.712538	0.887021	None
17395	KIR2DS4	-0.00197	0.71258	0.887021	None
17396	SLC17A3	-0.00063	0.712583	0.887021	None
17397	BRMS1	-0.05923	0.712619	0.887021	None
17398	AKR1B1	0.065617	0.712672	0.887021	None

17399	RNF31	-0.01466	0.712704	0.887021	None
17400	COLGALT2	-0.04467	0.71273	0.887021	None
17401	AX746699	-0.00832	0.712882	0.887159	None
17402	SLC25A17	-0.04677	0.712943	0.887178	None
17403	LOC10192	-0.00083	0.712979	0.887178	None
17404	ZC2HC1A	-0.09044	0.71304	0.887183	None
17405	KCNIP2	0.001815	0.713065	0.887183	None
17406	C16orf89	-0.00766	0.713216	0.88732	None
17407	CTAG2	-0.04756	0.713272	0.887339	None
17408	SLC4A11	0.010821	0.713336	0.887367	None
17409	ORC1	0.095513	0.713448	0.887414	None
17410	TNFRSF11I	0.004148	0.713455	0.887414	None
17411	PTGER2	0.073543	0.71353	0.887456	None
17412	AC092192.	-0.00421	0.713608	0.887502	None
17413	NUDT9P1	-0.03528	0.713746	0.887613	None
17414	MYBPC1	-0.00598	0.713792	0.887613	None
17415	LOC15112	-0.00114	0.713837	0.887613	None
17416	LINC01095	-0.00105	0.713949	0.887613	None
17417	PAGE1	0.009364	0.713976	0.887613	None
17418	RBBP9	-0.01	0.713982	0.887613	None
17419	POLR1B	-0.04771	0.713984	0.887613	None
17420	LOC10192	-0.00059	0.714219	0.887696	None
17421	TBX6	0.011463	0.714225	0.887696	None
17422	BOLL	0.001677	0.714287	0.887696	None
17423	MRPL48	-0.04819	0.714287	0.887696	None
17424	RP4-545K1	0.030868	0.714296	0.887696	None
17425	LINC00908	0.005755	0.714336	0.887696	None
17426	LINC01102	-0.00278	0.714392	0.887696	None
17427	HCCAT5	-0.00226	0.714396	0.887696	None
17428	C19orf82	-0.05138	0.71442	0.887696	None
17429	LDLR	-0.01925	0.714479	0.887707	None
17430	ANKRD12	0.044835	0.714511	0.887707	None
17431	RBBP6	-0.04961	0.714592	0.887757	None
17432	RP11-442(	-0.00053	0.714668	0.887771	None
17433	LOC10192	-0.00112	0.714685	0.887771	None
17434	WDR48	0.048238	0.714772	0.887824	None
17435	ODF2L	-0.03754	0.714856	0.887824	None
17436	CSF2RB	-0.13793	0.715013	0.887824	None
17437	ZMIZ1	-0.09529	0.715025	0.887824	None
17438	CMTM2	0.105175	0.715073	0.887824	None
17439	FOXD3	-0.00194	0.715099	0.887824	None
17440	NKX6-2	0.005775	0.715151	0.887824	None
17441	CD200	-0.11201	0.715152	0.887824	None
17442	LOC10050!	-0.02945	0.715197	0.887824	None
17443	RALA	0.049573	0.715209	0.887824	None
17444	SERPINB7	-0.00058	0.715216	0.887824	None
17445	SCGB3A2	-0.01262	0.71522	0.887824	None
17446	C1orf87	-0.00189	0.715342	0.887859	None
17447	TNFSF18	-0.00336	0.715439	0.887859	None
17448	RPE65	-0.00477	0.715465	0.887859	None
17449	SS18	-0.03666	0.715487	0.887859	None
17450	REEP5	0.078246	0.715492	0.887859	None
17451	RP11-1151	0.004031	0.715494	0.887859	None
17452	PIH2	-0.0022	0.715544	0.88787	None
17453	KCNC4	-0.01192	0.715678	0.887935	None
17454	NPAS4	-0.0015	0.715678	0.887935	None
17455	NUP188	-0.02946	0.715814	0.888052	None
17456	GTF2H5	-0.0322	0.715984	0.888204	None

17457	FILIP1	-0.00532	0.716018	0.888204	None
17458	RP11-573N	-0.00091	0.716081	0.88823	None
17459	PTBP1	-0.01541	0.716122	0.888231	None
17460	RP1-187B2	-0.00097	0.71626	0.888315	None
17461	LOC10192	-0.00227	0.716272	0.888315	None
17462	POP7	0.076012	0.716396	0.888418	None
17463	LINC00310	-0.00653	0.716502	0.888457	None
17464	FKSG29	-0.00057	0.716515	0.888457	None
17465	CRYBG3	-0.09597	0.716551	0.888457	None
17466	QTRTD1	-0.02499	0.716633	0.888474	None
17467	NPAP1	-0.00597	0.716646	0.888474	None
17468	LOC10192	-0.00336	0.716748	0.888549	None
17469	F2R	0.075585	0.716808	0.888572	None
17470	MARVELD3	-0.00374	0.716848	0.888572	None
17471	B3GALT4	-0.02906	0.717056	0.888674	None
17472	RAD21L1	-0.00735	0.717097	0.888674	None
17473	NUDT4	-0.00914	0.717107	0.888674	None
17474	DQ581328	-0.0114	0.717141	0.888674	None
17475	CCDC69	0.082577	0.717161	0.888674	None
17476	HOPX	0.010223	0.717177	0.888674	None
17477	PPP4C	-0.05753	0.717275	0.888745	None
17478	MAGEB18	-0.00234	0.717375	0.888818	None
17479	KIAA1715	-0.05777	0.717582	0.888979	None
17480	DCST1	-0.00098	0.717588	0.888979	None
17481	TMEM98	0.166096	0.717681	0.889045	None
17482	TLL2	-0.00123	0.717772	0.889106	None
17483	LRRC4C	-0.02665	0.717898	0.889206	None
17484	MSRB3	-0.09454	0.71797	0.889206	None
17485	FLJ45825	0.0074	0.717975	0.889206	None
17486	LGI2	0.027948	0.718174	0.889234	None
17487	FHOD3	-0.02507	0.718186	0.889234	None
17488	TAS2R7	-0.00084	0.718189	0.889234	None
17489	KHDC1	-0.04376	0.71819	0.889234	None
17490	LOC10272	-0.00426	0.718204	0.889234	None
17491	LMO7DN	-0.01056	0.718261	0.889254	None
17492	LINC00301	-0.00087	0.71849	0.889446	None
17493	BBS9	-0.04698	0.718518	0.889446	None
17494	MBD3L1	0.005552	0.718539	0.889446	None
17495	IRS2	0.069244	0.718753	0.889659	None
17496	LINC01153	-0.00073	0.719161	0.890072	None
17497	NARF	-0.0326	0.719169	0.890072	None
17498	ESPN	-0.01105	0.719265	0.890109	None
17499	SPRR1B	-0.0013	0.71928	0.890109	None
17500	HNRNPUL	-0.02978	0.719435	0.890199	None
17501	HOXB7	-0.05173	0.719435	0.890199	None
17502	ATAD2B	-0.05778	0.719528	0.890262	None
17503	ADRB2	-0.15897	0.719617	0.890322	None
17504	RP5-856G	-0.00136	0.719686	0.890357	None
17505	RP11-359K	-0.00092	0.719784	0.890427	None
17506	ALOX15	-0.01418	0.719872	0.890484	None
17507	LOC10050	-0.00063	0.720001	0.890593	None
17508	UBE2U	-0.00429	0.720053	0.890607	None
17509	DIAPH3	-0.03263	0.720165	0.890694	None
17510	FLNC	-0.01883	0.720384	0.890915	None
17511	MED31	-0.08359	0.720467	0.89093	None
17512	NEUROD2	-0.01078	0.720499	0.89093	None
17513	SYPL2	-0.01462	0.72052	0.89093	None
17514	LOC10192	-0.00852	0.72063	0.890932	None

17515	TMEM184/	0.012256	0.720665	0.890932	None
17516	ZNF83	-0.06205	0.720672	0.890932	None
17517	LOC10192:	0.012446	0.720718	0.890932	None
17518	LOC44117:	0.004489	0.720763	0.890932	None
17519	DDRGK1	-0.04187	0.720769	0.890932	None
17520	ZNF404	-0.11233	0.720843	0.890937	None
17521	RP11-552F	-0.00076	0.720857	0.890937	None
17522	C11orf85	-0.00538	0.720896	0.890937	None
17523	NOB1	-0.05875	0.721027	0.890951	None
17524	C10orf12	-0.01127	0.721051	0.890951	None
17525	FAM170B-	-0.00055	0.721062	0.890951	None
17526	MCF2L	-0.00161	0.721072	0.890951	None
17527	AC005785.	0.010149	0.72113	0.890972	None
17528	TTC33	-0.02856	0.721216	0.891028	None
17529	SLC2A1	-0.06775	0.721273	0.891048	None
17530	SLC12A5	0.024007	0.721388	0.891139	None
17531	LINC00671	-0.00288	0.721494	0.891218	None
17532	LOC51145	-0.00317	0.721563	0.891253	None
17533	LOC10192	0.016665	0.721612	0.891262	None
17534	SNRNP25	-0.06982	0.721757	0.891362	None
17535	HTR1D	-0.00066	0.721774	0.891362	None
17536	EYA2	-0.00063	0.722035	0.891583	None
17537	SRSF4	-0.09788	0.72204	0.891583	None
17538	MARCKSL1	-0.0931	0.722078	0.891583	None
17539	FAM71C	-0.0009	0.72212	0.891585	None
17540	ITGB3BP	0.051175	0.72218	0.891608	None
17541	LOC28555	-0.00316	0.722275	0.891618	None
17542	ART1	-0.00602	0.722305	0.891618	None
17543	GPATCH4	-0.00943	0.722312	0.891618	None
17544	CT83	-0.00559	0.722376	0.891647	None
17545	GAL3ST2	0.014116	0.722458	0.891697	None
17546	EXPH5	-0.0476	0.722507	0.891703	None
17547	MYL7	-0.00392	0.722584	0.891703	None
17548	EEF1D	-0.05461	0.722587	0.891703	None
17549	NUPL2	0.064519	0.722655	0.891709	None
17550	ATP8A1	-0.03503	0.722712	0.891709	None
17551	LILRB3	0.047987	0.722715	0.891709	None
17552	LYSMD1	-0.01453	0.722792	0.891754	None
17553	ARC	-0.00296	0.722916	0.891764	None
17554	RP3-412A:	0.010437	0.722974	0.891764	None
17555	LOC10192	-0.00558	0.722989	0.891764	None
17556	RP11-167H	-0.00667	0.723014	0.891764	None
17557	ZFR	0.057802	0.723027	0.891764	None
17558	TM6SF1	-0.12202	0.723048	0.891764	None
17559	CCDC184	-0.00232	0.723136	0.891823	None
17560	LINC00472	-0.01141	0.723199	0.891849	None
17561	ZMYM6	-0.06584	0.7233	0.891923	None
17562	SNTG1	-0.00056	0.723428	0.89193	None
17563	SMAD5-AS	-0.00067	0.723455	0.89193	None
17564	PSMD13	0.045684	0.72347	0.89193	None
17565	SLITRK3	-0.00137	0.723471	0.89193	None
17566	FABP7	-0.00106	0.723543	0.891936	None
17567	FAM150B	0.103336	0.723558	0.891936	None
17568	SERPINA3	-0.02387	0.72365	0.891999	None
17569	TRAPPC6B	-0.05732	0.723746	0.892067	None
17570	OBSCN	-0.01776	0.723813	0.892098	None
17571	LOC93432	-0.00122	0.723862	0.892108	None
17572	RP11-585F	0.042802	0.723943	0.892157	None

17573	IRGC	-0.00371	0.724024	0.892166	None
17574	DSERG1	0.089984	0.724032	0.892166	None
17575	GREB1L	-0.00525	0.724157	0.892269	None
17576	MMRN2	-0.04615	0.72421	0.892271	None
17577	LOC28367	-0.00089	0.724241	0.892271	None
17578	NEK8	0.035274	0.724461	0.892491	None
17579	CD320	-0.08869	0.724516	0.892499	None
17580	DLGAP2	-0.01177	0.72455	0.892499	None
17581	FAM230B	-0.0022	0.724692	0.892532	None
17582	MYLK2	0.005087	0.724716	0.892532	None
17583	WDR86-AS	-0.01243	0.724748	0.892532	None
17584	LOC10192	0.005406	0.724759	0.892532	None
17585	GSC	-0.00276	0.724807	0.892532	None
17586	ITGA5	0.068966	0.72483	0.892532	None
17587	RP11-388M	-0.07001	0.724865	0.892532	None
17588	EIF3I	-0.03871	0.725027	0.892661	None
17589	MLANA	-0.00117	0.725082	0.892661	None
17590	SDHAF2	0.055557	0.725094	0.892661	None
17591	SPIRE2	0.003641	0.725308	0.892809	None
17592	ANKRD46	-0.07832	0.725345	0.892809	None
17593	SEC14L1P1	-0.07059	0.72535	0.892809	None
17594	PHF6	-0.03273	0.725379	0.892809	None
17595	LOC10272	0.006722	0.725506	0.892914	None
17596	LOC10192	-0.00277	0.725717	0.8931	None
17597	C11orf72	0.008312	0.725765	0.8931	None
17598	AC005523.	-0.08286	0.725781	0.8931	None
17599	RP11-319C	0.08396	0.725848	0.893133	None
17600	HLA-DQB2	0.021329	0.725934	0.893175	None
17601	FGF17	-0.00957	0.726039	0.893175	None
17602	BCKDHA	0.050338	0.726042	0.893175	None
17603	GRM8	0.005102	0.726058	0.893175	None
17604	MUC2	0.009861	0.726089	0.893175	None
17605	COPS2	0.041106	0.726187	0.893245	None
17606	MAGIX	-0.00152	0.726323	0.893362	None
17607	GABRA4	-0.00748	0.726407	0.893375	None
17608	PIRT	-0.00113	0.726416	0.893375	None
17609	CLRN1-AS	-0.00852	0.726481	0.893404	None
17610	GUCY2F	-0.00053	0.726564	0.893455	None
17611	SPATA6L	-0.00077	0.726901	0.893819	None
17612	FLJ41170	-0.00376	0.727028	0.893875	None
17613	DHX40	0.031039	0.727029	0.893875	None
17614	RHOJ	-0.00638	0.727118	0.893933	None
17615	KRTAP9-3	-0.0022	0.72717	0.893946	None
17616	XRCC6	0.039629	0.727241	0.893959	None
17617	FKSG49	0.030377	0.727308	0.893959	None
17618	NKX2-8	-0.01249	0.727319	0.893959	None
17619	LIMS1	-0.05011	0.727345	0.893959	None
17620	OPRM1	-0.00124	0.727448	0.893968	None
17621	LOC25565	-0.00061	0.727455	0.893968	None
17622	PRM3	-0.00363	0.727476	0.893968	None
17623	CXorf58	-0.00401	0.727525	0.893976	None
17624	ADAM23	-0.0057	0.727616	0.89399	None
17625	NUP155	0.066029	0.727648	0.89399	None
17626	MAP3K3	-0.04669	0.727693	0.89399	None
17627	SNTN	0.001217	0.727805	0.89399	None
17628	TBC1D28	-0.00198	0.727809	0.89399	None
17629	FAM169B	0.006075	0.72782	0.89399	None
17630	PAFAH1B3	-0.05825	0.727825	0.89399	None

17631	GNE	0.037668	0.7279	0.894032	None
17632	VN1R5	-0.0005	0.727945	0.894037	None
17633	LOC10050(	-0.00966	0.728014	0.89407	None
17634	RAB30-AS:	-0.03968	0.728073	0.894093	None
17635	PRPF38A	0.030612	0.728166	0.894149	None
17636	LIPI	-0.00245	0.728202	0.894149	None
17637	LINC01279	-0.02514	0.728334	0.89426	None
17638	RP11-95O:	-0.00223	0.72839	0.894278	None
17639	LOC10272	0.011626	0.728601	0.894395	None
17640	HAGHL	0.051828	0.728609	0.894395	None
17641	SLC22A31	-0.01107	0.728629	0.894395	None
17642	LOC10013(	-0.04712	0.72865	0.894395	None
17643	LOC10192	-0.00162	0.728847	0.894586	None
17644	PITX2	-0.05645	0.728944	0.894615	None
17645	CSNK1G3	-0.05268	0.728953	0.894615	None
17646	CDKN2A	-0.03416	0.729051	0.894654	None
17647	OR10C1	-0.00053	0.729068	0.894654	None
17648	PLCH1-AS	-0.00084	0.729247	0.894686	None
17649	LINC00189	-0.01018	0.729281	0.894686	None
17650	OR1J4	0.01141	0.729285	0.894686	None
17651	CFLAR-AS:	-0.00586	0.729299	0.894686	None
17652	LINC00269	-0.00437	0.729332	0.894686	None
17653	RP11-701I:	-0.00631	0.729379	0.894686	None
17654	BCL2	0.034611	0.729412	0.894686	None
17655	CYP11A1	-0.00131	0.729424	0.894686	None
17656	PAX2	-0.00227	0.72954	0.894754	None
17657	TBX10	0.00874	0.729562	0.894754	None
17658	LOC10192(	-0.00503	0.72965	0.894811	None
17659	ASPDH	-0.03341	0.729814	0.894958	None
17660	GUCY2C	0.000438	0.729902	0.894958	None
17661	ZSCAN1	-0.00094	0.729932	0.894958	None
17662	LINC00842	-0.00233	0.729955	0.894958	None
17663	SCAF1	-0.02035	0.729977	0.894958	None
17664	AMER3	-0.00057	0.730032	0.894975	None
17665	SSTR1	-0.00551	0.730145	0.895049	None
17666	SLC38A6	-0.08123	0.730179	0.895049	None
17667	LOC10192	-0.00142	0.730216	0.895049	None
17668	SLC10A7	0.022778	0.730266	0.895059	None
17669	AC009133.	-0.02417	0.730317	0.895072	None
17670	CECR6	0.017149	0.730501	0.895197	None
17671	RP11-389(	-0.00506	0.730536	0.895197	None
17672	VSIG10	0.018154	0.730544	0.895197	None
17673	RPS27	-0.05322	0.730625	0.895222	None
17674	ZNF442	0.033652	0.730655	0.895222	None
17675	RUNX1T1	-0.03192	0.730688	0.895222	None
17676	LOC10050!	-0.00188	0.730749	0.895225	None
17677	LINC00523	-0.00933	0.730773	0.895225	None
17678	LRRC7	-0.02839	0.730975	0.895288	None
17679	SERPINB3	-0.00086	0.731064	0.895288	None
17680	RP5-1170D	-0.00828	0.7311	0.895288	None
17681	PARVG	-0.08034	0.731183	0.895288	None
17682	MAS1	-0.00161	0.731188	0.895288	None
17683	BOD1L2	0.013694	0.731264	0.895288	None
17684	TMEM74	0.003183	0.731364	0.895288	None
17685	KAT6A	-0.04289	0.731421	0.895288	None
17686	LIG1	0.067045	0.731458	0.895288	None
17687	LOC10013:	-0.00236	0.731488	0.895288	None
17688	BPIFB1	-0.016	0.731492	0.895288	None

17689	LOC14970:	-0.00304	0.731499	0.895288	None
17690	ADRA1A	-0.00051	0.731631	0.895288	None
17691	TMC4	-0.00966	0.731657	0.895288	None
17692	MIOS	-0.04137	0.731685	0.895288	None
17693	ALOX5	-0.05276	0.731699	0.895288	None
17694	CA6	-0.00508	0.731736	0.895288	None
17695	CST5	-0.01404	0.731759	0.895288	None
17696	MED7	-0.05702	0.731789	0.895288	None
17697	PSCA	-0.0112	0.731793	0.895288	None
17698	CTD-2313:	-0.0009	0.731802	0.895288	None
17699	SPATA21	-0.01221	0.731832	0.895288	None
17700	CECR5	-0.04763	0.731907	0.895288	None
17701	SPARC	0.081208	0.732039	0.895288	None
17702	LINC00486	-0.00131	0.732096	0.895288	None
17703	IL4R	-0.06332	0.732176	0.895288	None
17704	PDE10A	-0.00905	0.732207	0.895288	None
17705	XRN2	-0.05506	0.732217	0.895288	None
17706	KRT16	0.001959	0.732235	0.895288	None
17707	LOC10192:	0.007648	0.732239	0.895288	None
17708	LOC10192:	-0.0019	0.732239	0.895288	None
17709	SDHD	-0.02436	0.732299	0.895288	None
17710	BRS3	0.00395	0.73232	0.895288	None
17711	TUBGCP2	0.032987	0.732338	0.895288	None
17712	LOC10050(	-0.06245	0.732587	0.895288	None
17713	CLCA2	-0.00051	0.732694	0.895288	None
17714	ALPP	-0.00597	0.732706	0.895288	None
17715	SLC5A9	-0.00397	0.732717	0.895288	None
17716	BC047651	0.016047	0.732783	0.895288	None
17717	HCN1	-0.01443	0.73279	0.895288	None
17718	FRMD6-AS	-0.02372	0.732879	0.895288	None
17719	RSPH9	0.013433	0.732891	0.895288	None
17720	LOC10192!	-0.00112	0.732948	0.895288	None
17721	PCBP4	-0.02348	0.733036	0.895288	None
17722	EFTUD1	0.045825	0.733067	0.895288	None
17723	DLEU7-AS:	-0.00256	0.733104	0.895288	None
17724	XRCC3	0.024555	0.73311	0.895288	None
17725	ITPK1	-0.01304	0.73313	0.895288	None
17726	LINC01364	-0.0061	0.733136	0.895288	None
17727	P2RY1	-0.0863	0.733229	0.895288	None
17728	PCDHB1	-0.00053	0.73324	0.895288	None
17729	ATL2	0.027466	0.733251	0.895288	None
17730	RPL35A	-0.05016	0.733279	0.895288	None
17731	LOC28482!	-0.00064	0.733288	0.895288	None
17732	KCP	0.011391	0.733302	0.895288	None
17733	SLC30A2	-0.01181	0.733331	0.895288	None
17734	RBSG3	-0.00167	0.733375	0.895288	None
17735	SLC30A9	0.040758	0.733397	0.895288	None
17736	LINC01214	0.005827	0.733405	0.895288	None
17737	ZNF335	0.016245	0.733472	0.895288	None
17738	C17orf50	-0.01051	0.733494	0.895288	None
17739	GATM	-0.00478	0.733542	0.895288	None
17740	CAMK1D	0.040606	0.733624	0.895288	None
17741	FLI1	-0.11881	0.733627	0.895288	None
17742	RP11-1277	0.007208	0.733698	0.895288	None
17743	DECR1	-0.04043	0.733704	0.895288	None
17744	ACTA2-AS	-0.00371	0.733707	0.895288	None
17745	CFDP1	0.056872	0.733759	0.895288	None
17746	DHX8	-0.04575	0.733785	0.895288	None

17747	GPI	-0.06499	0.733827	0.895288	None
17748	PMF1	0.048763	0.733893	0.895288	None
17749	TAGLN3	-0.00736	0.733902	0.895288	None
17750	PPL	0.008826	0.733902	0.895288	None
17751	SCRN3	0.021618	0.733949	0.895288	None
17752	TYR	-0.00061	0.733961	0.895288	None
17753	KIAA0485	-0.06885	0.734069	0.895288	None
17754	CTAGE11P	-0.00795	0.734111	0.895288	None
17755	SCFD1	-0.05562	0.734133	0.895288	None
17756	LOC10192	-0.00068	0.734143	0.895288	None
17757	ADAMTS1	-0.00059	0.734201	0.895288	None
17758	STK17B	0.106457	0.734203	0.895288	None
17759	LINC00645	-0.00051	0.734215	0.895288	None
17760	LOC10272	-0.01193	0.734312	0.895346	None
17761	BC033164	0.01605	0.734359	0.895346	None
17762	TSGA13	-0.0183	0.734424	0.895346	None
17763	MYLK3	-0.00085	0.734487	0.895346	None
17764	DNAJC5B	-0.02849	0.734493	0.895346	None
17765	CHST6	-0.00192	0.734518	0.895346	None
17766	OR5P2	-0.00096	0.734552	0.895346	None
17767	RP11-674F	-0.00124	0.73467	0.895432	None
17768	LYG1	-0.04562	0.734764	0.895432	None
17769	LOC10050	0.024248	0.734781	0.895432	None
17770	PHYKPL	-0.05815	0.734788	0.895432	None
17771	KLB	-0.00205	0.734915	0.895499	None
17772	LINC01242	0.038155	0.734933	0.895499	None
17773	AFM	-0.00131	0.734967	0.895499	None
17774	PFKFB1	-0.00123	0.735124	0.895617	None
17775	ZNF275	0.047257	0.735186	0.895617	None
17776	CABP7	-0.00813	0.735228	0.895617	None
17777	PFKFB2	-0.01917	0.735229	0.895617	None
17778	CRYBA1	0.005271	0.735336	0.895697	None
17779	RP11-799D	-0.00067	0.735445	0.895779	None
17780	GAL3ST1	-0.01082	0.735543	0.895817	None
17781	CHST5	0.002383	0.735558	0.895817	None
17782	GRIA2	-0.00126	0.735641	0.895853	None
17783	ZMYND10	-0.00649	0.735671	0.895853	None
17784	SOD3	-0.02238	0.735824	0.895967	None
17785	LOC10192	0.007198	0.735875	0.895967	None
17786	RANBP9	0.065683	0.735889	0.895967	None
17787	TBX15	0.01429	0.736044	0.896036	None
17788	ALS2CR12	0.013667	0.736104	0.896036	None
17789	VIP	-0.0745	0.736191	0.896036	None
17790	S100A7	-0.00056	0.736199	0.896036	None
17791	EPHA5	-0.04057	0.736251	0.896036	None
17792	PLCB4	0.114155	0.736266	0.896036	None
17793	ZNF536	-0.00062	0.736301	0.896036	None
17794	LOC28319	0.029507	0.736454	0.896036	None
17795	C1orf110	-0.00096	0.736462	0.896036	None
17796	TEX36	-0.00063	0.736481	0.896036	None
17797	RLN1	-0.01818	0.736491	0.896036	None
17798	LILRA2	0.097703	0.736494	0.896036	None
17799	MYADML2	0.005912	0.736513	0.896036	None
17800	VPS9D1	0.032838	0.736525	0.896036	None
17801	EP400NL	-0.03814	0.736613	0.896057	None
17802	FLJ27354	-0.01649	0.736624	0.896057	None
17803	XKR4	-0.00172	0.736668	0.89606	None
17804	SDHA	-0.03665	0.736769	0.896132	None

17805	C11orf87	-0.00449	0.736846	0.896176	None
17806	SNX16	0.062208	0.736922	0.896189	None
17807	NPAS2	-0.00117	0.73694	0.896189	None
17808	FER1L5	-0.00386	0.73709	0.89628	None
17809	MEGF11	-0.00063	0.73713	0.89628	None
17810	LOC10272!	0.005316	0.737142	0.89628	None
17811	RP1-272E8	-0.00462	0.737181	0.89628	None
17812	NUBPL	-0.04295	0.737286	0.896322	None
17813	TDRD10	0.03569	0.737298	0.896322	None
17814	HAS2	0.016825	0.737403	0.8964	None
17815	DLGAP1-A	-0.03563	0.737508	0.896468	None
17816	CACNA1B	-0.0154	0.737567	0.896468	None
17817	AC007680.	0.002093	0.737584	0.896468	None
17818	ZNF14	0.058855	0.737726	0.896524	None
17819	DIO1	-0.00459	0.737811	0.896524	None
17820	MPV17L	-0.03379	0.737826	0.896524	None
17821	GJB4	-0.00713	0.737844	0.896524	None
17822	CDH12	-0.01814	0.737892	0.896524	None
17823	RPS28	-0.05048	0.737895	0.896524	None
17824	LOC10192	-0.01094	0.737922	0.896524	None
17825	CD1B	-0.01759	0.737961	0.896524	None
17826	TMEM18	0.049222	0.738007	0.896524	None
17827	CLHC1	0.081792	0.738048	0.896524	None
17828	XRCC4	0.051543	0.738095	0.896524	None
17829	LILRB1	-0.06982	0.738127	0.896524	None
17830	LOC10192	-0.0009	0.738169	0.896526	None
17831	LOC10192	-0.00053	0.738317	0.896622	None
17832	CELA3B	0.006786	0.738331	0.896622	None
17833	SLC1A2	-0.00074	0.738423	0.896626	None
17834	ROPN1	-0.00187	0.738447	0.896626	None
17835	LINC00458	-0.00349	0.738459	0.896626	None
17836	BPIFB6	-0.00171	0.73855	0.89664	None
17837	CAPN6	-0.00632	0.738553	0.89664	None
17838	CYP8B1	-0.00062	0.738899	0.897	None
17839	MLNR	-0.00094	0.738955	0.897	None
17840	LINC01098	-0.00342	0.739037	0.897	None
17841	LOC10013	-0.00321	0.739073	0.897	None
17842	FAM118A	0.086009	0.739092	0.897	None
17843	MRPL24	-0.06138	0.739214	0.897	None
17844	LOC10192	-0.00839	0.739216	0.897	None
17845	LINC00682	-0.00122	0.739217	0.897	None
17846	TMLHE	-0.0561	0.739259	0.897	None
17847	LOC28373	-0.00372	0.739264	0.897	None
17848	FSCN3	-0.00194	0.739446	0.897171	None
17849	LOC10192	-0.00243	0.739593	0.897298	None
17850	BRMS1L	0.023993	0.739762	0.897426	None
17851	CTNND2	-0.0006	0.739818	0.897426	None
17852	C14orf105	-0.0021	0.739881	0.897426	None
17853	TDRD5	-0.00697	0.739917	0.897426	None
17854	GPR82	-0.03968	0.739966	0.897426	None
17855	WDR43	-0.04222	0.74005	0.897426	None
17856	LOC10192	-0.00052	0.740076	0.897426	None
17857	PRO2958	-0.00076	0.740157	0.897426	None
17858	ZNF665	0.039492	0.740161	0.897426	None
17859	BCL2L14	-0.0015	0.740179	0.897426	None
17860	NKAPP1	0.041089	0.740241	0.897426	None
17861	ANKRD39	-0.0584	0.740278	0.897426	None
17862	ART4	-0.0295	0.740335	0.897426	None

17863	FAM135B	-0.00074	0.740343	0.897426	None
17864	FOXI1	0.002913	0.740353	0.897426	None
17865	UNC13C	-0.01974	0.740361	0.897426	None
17866	RP11-680F	-0.0005	0.740443	0.897459	None
17867	DQ580846	-0.0133	0.740471	0.897459	None
17868	ZNF175	0.039581	0.740513	0.897459	None
17869	ATP8B5P	-0.00193	0.74061	0.897467	None
17870	RFK	-0.04535	0.740634	0.897467	None
17871	C9orf91	-0.05992	0.740725	0.897467	None
17872	C6orf57	-0.06542	0.740751	0.897467	None
17873	SNF8	0.05198	0.740793	0.897467	None
17874	TEX101	0.016427	0.740796	0.897467	None
17875	CSTL1	-0.00067	0.740825	0.897467	None
17876	NPY1R	-0.02116	0.740851	0.897467	None
17877	INSC	-0.00269	0.740929	0.897511	None
17878	SRY	-0.00159	0.740988	0.897533	None
17879	ZFAND1	0.051323	0.74112	0.8976	None
17880	C4orf3	-0.05087	0.741137	0.8976	None
17881	RAB11FIP5	0.022014	0.741168	0.8976	None
17882	FOXC2	-0.0072	0.741209	0.8976	None
17883	TRIM55	-0.0017	0.741301	0.897661	None
17884	LOC10192(	-0.00271	0.741569	0.897896	None
17885	LOC72973:	-0.02181	0.741592	0.897896	None
17886	RP11-378J	0.005902	0.741659	0.897896	None
17887	IFRD1	-0.06837	0.741695	0.897896	None
17888	CTCFL	0.007357	0.741702	0.897896	None
17889	COL25A1	-0.00514	0.74178	0.897939	None
17890	DNAJC10	0.051682	0.741873	0.897955	None
17891	HOXA10	-0.06596	0.741884	0.897955	None
17892	TMEM177	-0.04535	0.74192	0.897955	None
17893	MAP3K19	0.008951	0.741976	0.897955	None
17894	LOXHD1	-0.00162	0.742013	0.897955	None
17895	POU3F3	-0.00063	0.742049	0.897955	None
17896	FLJ37035	-0.00528	0.742083	0.897955	None
17897	EDA	0.004961	0.742127	0.897959	None
17898	TMEM190	-0.00425	0.742195	0.89799	None
17899	PMCHL1	-0.00076	0.742253	0.898011	None
17900	PHF10	0.039746	0.742359	0.898047	None
17901	CLCN7	-0.03586	0.74249	0.898047	None
17902	HSF4	-0.0267	0.742506	0.898047	None
17903	NS3BP	-0.00116	0.742525	0.898047	None
17904	C18orf21	0.026006	0.742588	0.898047	None
17905	LOC72861:	0.071167	0.742588	0.898047	None
17906	CHRNB2	0.016331	0.74261	0.898047	None
17907	TTC40	-0.00433	0.742615	0.898047	None
17908	RP11-510(	-0.00058	0.74272	0.898054	None
17909	HPX	0.002297	0.742766	0.898054	None
17910	GAGE3	-0.00059	0.742807	0.898054	None
17911	SMCO4	0.078887	0.742858	0.898054	None
17912	CATSPERG	-0.00158	0.742868	0.898054	None
17913	KCNT1	-0.00069	0.742869	0.898054	None
17914	KRT77	-0.00137	0.742936	0.898084	None
17915	NALCN	-0.00372	0.743148	0.898183	None
17916	RP11-90C4	0.005227	0.743168	0.898183	None
17917	WWC1	0.00427	0.743169	0.898183	None
17918	WASF1	-0.08764	0.743184	0.898183	None
17919	IFNGR1	0.068762	0.743331	0.89823	None
17920	SLFN5	0.040884	0.743344	0.89823	None

17921	LOC10272	-0.00288	0.743347	0.89823	None
17922	LOC44160	-0.01069	0.743395	0.898237	None
17923	F2RL2	-0.02741	0.743494	0.898289	None
17924	TBL1Y	-0.01509	0.743535	0.898289	None
17925	PP12613	-0.00421	0.74359	0.898289	None
17926	GPR87	-0.00087	0.743603	0.898289	None
17927	LOC10192	-0.00145	0.74388	0.898544	None
17928	SAMD12	-0.04716	0.743997	0.898544	None
17929	AHRR	-0.08263	0.744015	0.898544	None
17930	TEKT5	-0.00188	0.74402	0.898544	None
17931	SMIM21	-0.00259	0.744022	0.898544	None
17932	DKFZp547	-0.00163	0.744145	0.898638	None
17933	CD14	-0.20013	0.744183	0.898638	None
17934	TMEM225	-0.00394	0.744314	0.898652	None
17935	MED1	-0.03163	0.744318	0.898652	None
17936	TMPRSS12	-0.00101	0.744319	0.898652	None
17937	MAGEA1	-0.01616	0.744477	0.898787	None
17938	RNF166	-0.06288	0.744611	0.898787	None
17939	LOC10192	-0.00572	0.744616	0.898787	None
17940	RP11-152L	-0.00098	0.744631	0.898787	None
17941	RP11-546C	-0.00105	0.744715	0.898787	None
17942	MYOT	-0.00202	0.744754	0.898787	None
17943	CTD-2083I	0.011073	0.744819	0.898787	None
17944	RP11-300A	-0.00427	0.74482	0.898787	None
17945	TFF3	0.040514	0.744837	0.898787	None
17946	SPATA12	-0.00188	0.744845	0.898787	None
17947	CES5A	-0.0046	0.745051	0.898961	None
17948	A1CF	0.011062	0.745138	0.898961	None
17949	THPO	-0.00451	0.745238	0.898961	None
17950	GRIN3B	-0.00367	0.745242	0.898961	None
17951	USP30	-0.03451	0.745257	0.898961	None
17952	THADA	-0.01943	0.745289	0.898961	None
17953	DLGAP2-A	-0.00493	0.745289	0.898961	None
17954	CCDC18	-0.07893	0.745321	0.898961	None
17955	NT5DC2	0.016205	0.745371	0.89897	None
17956	LOC10192	-0.0018	0.745474	0.899044	None
17957	PUM2	-0.03465	0.745542	0.899051	None
17958	RTN4RL2	-0.00098	0.745607	0.899051	None
17959	FHL5	-0.00101	0.74561	0.899051	None
17960	KIF1A	-0.00153	0.745717	0.899051	None
17961	MACC1	-0.0712	0.745758	0.899051	None
17962	6-Mar	-0.03305	0.745783	0.899051	None
17963	DLX5	-0.00196	0.745789	0.899051	None
17964	RP11-544D	-0.00236	0.745881	0.899051	None
17965	CCDC65	0.003753	0.745911	0.899051	None
17966	RAPGEF3	-0.0237	0.745938	0.899051	None
17967	ANP32C	0.000544	0.746005	0.899051	None
17968	MRPL57	-0.03784	0.746009	0.899051	None
17969	ATAD3C	0.020687	0.746027	0.899051	None
17970	ST7-AS1	0.057544	0.746116	0.899051	None
17971	LOC10192	-0.01196	0.746179	0.899051	None
17972	MMP15	0.008252	0.746252	0.899051	None
17973	TRIM52-AS	-0.0317	0.746274	0.899051	None
17974	SHISA9	-0.00321	0.746366	0.899051	None
17975	TMEM63B	-0.04798	0.746387	0.899051	None
17976	LOC28530	-0.00247	0.746389	0.899051	None
17977	COL19A1	-0.00132	0.746409	0.899051	None
17978	LOC28569	-0.00728	0.746416	0.899051	None

17979	WWC2	-0.00709	0.746434	0.899051	None
17980	KLHDC2	0.03111	0.746499	0.899078	None
17981	RBM46	0.002046	0.746557	0.899099	None
17982	RGS22	0.011317	0.746601	0.899102	None
17983	ZNF584	-0.04828	0.746704	0.899129	None
17984	IL9	-0.00099	0.746707	0.899129	None
17985	ATR	-0.04375	0.746816	0.899165	None
17986	TRNP1	0.005484	0.746847	0.899165	None
17987	HBZ	0.038909	0.74693	0.899165	None
17988	CXCL2	0.076663	0.74694	0.899165	None
17989	MCM3	-0.0692	0.746945	0.899165	None
17990	RP11-389C	0.024385	0.747011	0.899196	None
17991	NCOA4	-0.03621	0.747107	0.899233	None
17992	CTA-254O	-0.00053	0.747126	0.899233	None
17993	LOC101928	-0.00205	0.747257	0.899326	None
17994	CRCT1	-0.00066	0.747285	0.899326	None
17995	ANKRD24	-0.00255	0.747361	0.899367	None
17996	DLX2	-0.01089	0.747577	0.899527	None
17997	LOC101928	0.001517	0.747577	0.899527	None
17998	UBR5	-0.04375	0.747676	0.899596	None
17999	MYH13	-0.0051	0.747763	0.899596	None
18000	HPSE2	-0.02718	0.747786	0.899596	None
18001	RGP1	0.038471	0.747866	0.899596	None
18002	YBX2	-0.01782	0.747874	0.899596	None
18003	DAD1	-0.05148	0.747884	0.899596	None
18004	SAMD5	-0.00197	0.747966	0.899602	None
18005	SLC12A3	-0.00101	0.748013	0.899602	None
18006	SMIM4	-0.01386	0.748037	0.899602	None
18007	RC3H2	-0.04407	0.74806	0.899602	None
18008	ZNF302	0.075801	0.748125	0.899602	None
18009	KRTAP1-3	-0.00072	0.748183	0.899602	None
18010	LRIG3	-0.00956	0.748315	0.899602	None
18011	SLC2A4	-0.00137	0.748324	0.899602	None
18012	LINC00901	-0.00153	0.748353	0.899602	None
18013	TXNDC2	0.009937	0.7485	0.899602	None
18014	GUCY1A2	-0.00074	0.748535	0.899602	None
18015	HAR1A	-0.02951	0.748539	0.899602	None
18016	NAIF1	-0.01894	0.748554	0.899602	None
18017	GSX1	-0.00238	0.74864	0.899602	None
18018	SERPINC1	-0.0008	0.748693	0.899602	None
18019	FSTL4	-0.00184	0.748693	0.899602	None
18020	MKLN1	0.047684	0.748717	0.899602	None
18021	AC013463.	-0.00957	0.748725	0.899602	None
18022	MGC15885	-0.00051	0.748726	0.899602	None
18023	YPEL2	0.043216	0.748785	0.899602	None
18024	UMODL1	0.130494	0.748877	0.899602	None
18025	FNDC7	-0.00317	0.748893	0.899602	None
18026	C20orf78	-0.00173	0.748996	0.899602	None
18027	RP11-474D	0.006601	0.74904	0.899602	None
18028	SFTA3	-0.00177	0.74906	0.899602	None
18029	RP11-646J	-0.00089	0.749081	0.899602	None
18030	CASC16	0.00584	0.749091	0.899602	None
18031	AL022341.3	0.009818	0.749098	0.899602	None
18032	TM4SF4	-0.00203	0.749112	0.899602	None
18033	KRT19P2	-0.00066	0.749135	0.899602	None
18034	SLC39A10	-0.04603	0.74926	0.899657	None
18035	TARP	0.010258	0.74933	0.899657	None
18036	ESR1	-0.00363	0.749346	0.899657	None

18037	LOC10050!	-0.00063	0.749366	0.899657	None
18038	KIF18B	0.07845	0.749389	0.899657	None
18039	MAPK10	-0.01028	0.749457	0.899685	None
18040	C15orf32	-0.00071	0.74953	0.899685	None
18041	SNRPA	0.06667	0.749537	0.899685	None
18042	MBD2	0.004655	0.749642	0.899761	None
18043	DCST2	-0.00531	0.749785	0.89979	None
18044	BC048997	-0.00048	0.749786	0.89979	None
18045	RP11-266L	-0.00105	0.749809	0.89979	None
18046	REG1A	-0.0079	0.749832	0.89979	None
18047	PEG3	0.063033	0.750014	0.899959	None
18048	UGP2	-0.05097	0.750125	0.900042	None
18049	FOXD1	0.005577	0.750267	0.900162	None
18050	ERCC6L	-0.09418	0.750527	0.900424	None
18051	HSD17B7	0.028479	0.750749	0.900641	None
18052	LRRC37BP1	0.003427	0.750872	0.900738	None
18053	FGF10	-0.00092	0.750989	0.900753	None
18054	NUDT2	-0.05923	0.751041	0.900753	None
18055	TCEAL8	0.05068	0.751083	0.900753	None
18056	PNO1	-0.04533	0.751096	0.900753	None
18057	TGIF1	-0.06346	0.751115	0.900753	None
18058	LOC28486!	-0.01	0.751192	0.900753	None
18059	AMER2	-0.00068	0.75121	0.900753	None
18060	EXO1	0.083477	0.751254	0.900753	None
18061	LINC00051	-0.00134	0.751263	0.900753	None
18062	EBF4	0.012462	0.751324	0.900753	None
18063	VCPIP1	-0.03996	0.751417	0.900753	None
18064	PSORS1C2	-0.00178	0.75142	0.900753	None
18065	KLK13	-0.00384	0.751451	0.900753	None
18066	MYO10	-0.03958	0.751468	0.900753	None
18067	PLS1	-0.09921	0.751508	0.900753	None
18068	ANKRD55	0.048041	0.751588	0.90076	None
18069	LOC10192!	0.016927	0.751614	0.90076	None
18070	LAMA3	-0.00056	0.751678	0.90076	None
18071	NXT1	0.037163	0.75168	0.90076	None
18072	TAF11	-0.06538	0.751821	0.900829	None
18073	RP4-555D2	-0.00068	0.75186	0.900829	None
18074	RPL23A	-0.01632	0.751921	0.900829	None
18075	LHCGR	-0.0007	0.751972	0.900829	None
18076	DDX53	-0.00861	0.751976	0.900829	None
18077	ALDH8A1	-0.08638	0.752023	0.900829	None
18078	DIAPH2	0.05239	0.752065	0.900829	None
18079	GABRA5	-0.00099	0.752148	0.900829	None
18080	NPAS3	-0.00148	0.752213	0.900829	None
18081	IL36B	-0.00193	0.752224	0.900829	None
18082	HTR2C	-0.00111	0.752226	0.900829	None
18083	CTB-31O2	-0.00114	0.752237	0.900829	None
18084	RP11-587D	-0.00117	0.752299	0.900837	None
18085	GCNT2	0.03661	0.752327	0.900837	None
18086	NHLRC2	-0.01708	0.75241	0.900886	None
18087	SSB	-0.05146	0.752637	0.901046	None
18088	HINT1	-0.03802	0.752664	0.901046	None
18089	CRAMP1L	0.02044	0.752667	0.901046	None
18090	IER3IP1	-0.02947	0.752754	0.901073	None
18091	VIPR2	-0.00553	0.752874	0.901073	None
18092	CDCA8	0.080839	0.752879	0.901073	None
18093	RNF141	-0.05187	0.752938	0.901073	None
18094	ADAM20	-0.0073	0.752939	0.901073	None

18095	ZFP42	-0.00058	0.752951	0.901073	None
18096	SGCB	-0.04169	0.752982	0.901073	None
18097	BC042590	-0.03964	0.75304	0.901093	None
18098	SPAG8	-0.01342	0.753229	0.901269	None
18099	PPAP2A	-0.07088	0.75338	0.90134	None
18100	PLAT	0.094054	0.75341	0.90134	None
18101	LINC00702	-0.00343	0.753481	0.90134	None
18102	LEP	0.013199	0.75349	0.90134	None
18103	CCDC168	-0.00061	0.753496	0.90134	None
18104	RCBTB2	-0.03611	0.753554	0.901359	None
18105	CHID1	-0.05899	0.753701	0.901431	None
18106	SSBP3-AS1	-0.06296	0.753706	0.901431	None
18107	MROH9	-0.00295	0.753749	0.901431	None
18108	SYNE1	-0.00859	0.75378	0.901431	None
18109	RAB9BP1	-0.00079	0.753914	0.901512	None
18110	POU1F1	-0.00512	0.753996	0.901512	None
18111	ZNF280A	-0.00064	0.754052	0.901512	None
18112	CXCL12	-0.23429	0.7541	0.901512	None
18113	FAM71E2	-0.00369	0.75411	0.901512	None
18114	AC106801.	-0.00968	0.754226	0.901512	None
18115	KRT6A	0.000595	0.754269	0.901512	None
18116	SLC19A1	-0.00377	0.754344	0.901512	None
18117	APOA4	0.014042	0.754346	0.901512	None
18118	CHRNE	-0.00778	0.754374	0.901512	None
18119	RFC3	-0.07946	0.754394	0.901512	None
18120	SLC12A9	0.027374	0.754487	0.901512	None
18121	MRPL50	0.086731	0.754523	0.901512	None
18122	HOXA11-A	-0.00334	0.754566	0.901512	None
18123	IGFL2	0.009286	0.75459	0.901512	None
18124	RALGPS1	-0.00913	0.754625	0.901512	None
18125	RP3-496C2	0.010947	0.754642	0.901512	None
18126	SDC1	-0.05439	0.754864	0.901512	None
18127	ZNF304	0.047347	0.754892	0.901512	None
18128	CTD-2547I	-0.01438	0.754901	0.901512	None
18129	MIR3663H	-0.00067	0.754952	0.901512	None
18130	GABRG1	-0.04664	0.755053	0.901512	None
18131	FBXW9	-0.04395	0.755069	0.901512	None
18132	MNX1-AS1	-0.00113	0.755086	0.901512	None
18133	LOC10192	-0.00052	0.755102	0.901512	None
18134	SP8	-0.02283	0.755149	0.901512	None
18135	C12orf5	0.09357	0.755157	0.901512	None
18136	GRK7	-0.00473	0.755172	0.901512	None
18137	DQ570835	-0.1101	0.755203	0.901512	None
18138	CNRIP1	0.15954	0.75523	0.901512	None
18139	PSMA1	-0.02225	0.755247	0.901512	None
18140	BC039686	-0.01366	0.75525	0.901512	None
18141	TGS1	-0.03791	0.755289	0.901512	None
18142	LOC10192	-0.00096	0.75533	0.901512	None
18143	ZNF826P	0.052836	0.755331	0.901512	None
18144	RERGL	-0.00046	0.755347	0.901512	None
18145	VIT	0.004631	0.755516	0.901664	None
18146	C7orf50	-0.03803	0.755618	0.901665	None
18147	EBP	0.059974	0.755633	0.901665	None
18148	LOC10192	-0.00078	0.755642	0.901665	None
18149	RBM34	0.0262	0.755846	0.901792	None
18150	HDAC8	0.044661	0.755871	0.901792	None
18151	RP11-222K	-0.00107	0.755872	0.901792	None
18152	ARHGEF12	-0.00103	0.755998	0.901839	None

18153	LINC01122	0.047484	0.75602	0.901839	None
18154	NOS1	-0.00086	0.756072	0.901839	None
18155	HLA-DMA	-0.04779	0.756079	0.901839	None
18156	C11orf70	0.001992	0.756147	0.90186	None
18157	LOC10192	-0.00127	0.75618	0.90186	None
18158	C10orf107	-0.00096	0.756256	0.901896	None
18159	PRRC1	-0.04203	0.756293	0.901896	None
18160	STMN3	-0.0264	0.756446	0.902029	None
18161	ASH1L-AS	-0.05446	0.756616	0.90218	None
18162	SPAG5	-0.07449	0.756657	0.90218	None
18163	ORMDL2	-0.05815	0.756721	0.902207	None
18164	VN1R10P	-0.00046	0.75679	0.902233	None
18165	ZNF419	-0.03484	0.756842	0.902233	None
18166	PWP1	-0.03676	0.756915	0.902233	None
18167	COPS8	-0.02614	0.756921	0.902233	None
18168	LOC10192	-0.00083	0.756951	0.902233	None
18169	MST1L	-0.00562	0.757054	0.902264	None
18170	PTPRS	-0.00693	0.75706	0.902264	None
18171	LINC00619	-0.02342	0.757127	0.902294	None
18172	GPR137	-0.00687	0.757172	0.902299	None
18173	LOC10012	0.000398	0.757272	0.902318	None
18174	AGO4	-0.04204	0.75729	0.902318	None
18175	LOC10028	-0.00067	0.757314	0.902318	None
18176	NGDN	0.029163	0.757396	0.902324	None
18177	C1QTNF1	0.009891	0.757435	0.902324	None
18178	COL20A1	-0.01933	0.757446	0.902324	None
18179	ZNF549	-0.001	0.757485	0.902324	None
18180	FAM46C	0.08941	0.757574	0.902379	None
18181	LOC40065	-0.00052	0.757627	0.902393	None
18182	GPR153	-0.01822	0.757705	0.902411	None
18183	CCDC80	-0.02131	0.757725	0.902411	None
18184	RAP2A	-0.01373	0.757844	0.902467	None
18185	PDLIM5	0.030283	0.757856	0.902467	None
18186	PPIL3	0.044683	0.758058	0.902613	None
18187	IL1RL2	-0.00062	0.758062	0.902613	None
18188	FBXL5	-0.03225	0.758161	0.902682	None
18189	ANKUB1	-0.00082	0.758208	0.902688	None
18190	ATXN8OS	-0.0009	0.758279	0.902723	None
18191	MGC15705	-0.00524	0.758366	0.902777	None
18192	RP11-629	-0.03574	0.758509	0.90279	None
18193	IFNA21	-0.00116	0.758514	0.90279	None
18194	CCR3	0.011019	0.758553	0.90279	None
18195	C7orf57	0.006637	0.758598	0.90279	None
18196	OGT	0.04942	0.758648	0.90279	None
18197	CASC6	-0.00077	0.758664	0.90279	None
18198	MUL1	0.050076	0.758668	0.90279	None
18199	DNAJC14	-0.03022	0.758798	0.90285	None
18200	CCND2	0.071995	0.758813	0.90285	None
18201	NUP205	0.031189	0.758844	0.90285	None
18202	TPPP3	-0.01176	0.758947	0.902873	None
18203	LINC00896	-0.01065	0.758947	0.902873	None
18204	DISP2	-0.00584	0.759017	0.902906	None
18205	CDK4	-0.05508	0.759067	0.902916	None
18206	PYGB	0.017986	0.759162	0.902979	None
18207	JTB	-0.01758	0.75922	0.902999	None
18208	IGHMBP2	-0.01554	0.75943	0.9032	None
18209	MGEA5	0.04697	0.759602	0.90333	None
18210	APBB1	-0.02033	0.759623	0.90333	None

18211	TLR7	0.046118	0.759699	0.903371	None
18212	LOC10192!	-0.00058	0.759855	0.903499	None
18213	SERAC1	-0.04926	0.759921	0.903499	None
18214	ERLIN1	-0.05155	0.759957	0.903499	None
18215	NEURL1	-0.00662	0.759974	0.903499	None
18216	RBM47	0.014418	0.760066	0.903559	None
18217	ABCA6	-0.04814	0.760226	0.903684	None
18218	SLC13A2	0.007479	0.760255	0.903684	None
18219	CATSPERD	-0.00046	0.760355	0.903752	None
18220	SLBP	-0.04137	0.760395	0.903752	None
18221	SGPL1	0.066214	0.760442	0.903758	None
18222	ATP2B3	-0.00053	0.760511	0.90379	None
18223	LOC28588!	-0.00167	0.760637	0.903825	None
18224	USP39	-0.0403	0.76064	0.903825	None
18225	LOC10192{	0.021312	0.760674	0.903825	None
18226	PNLDC1	-0.02533	0.760729	0.903825	None
18227	BUB1	-0.00537	0.760749	0.903825	None
18228	PLOD2	-0.10615	0.760981	0.904052	None
18229	SOX4	-0.06425	0.761095	0.904096	None
18230	HRASLS2	0.007352	0.76117	0.904096	None
18231	GAD1	-0.09097	0.761182	0.904096	None
18232	KCNK16	-0.00105	0.761186	0.904096	None
18233	PRSS3P3	0.004491	0.761294	0.9041	None
18234	CALML6	0.016371	0.761311	0.9041	None
18235	GPR68	0.010263	0.76138	0.9041	None
18236	AC017104.	-0.01159	0.761402	0.9041	None
18237	SNN	0.06137	0.761449	0.9041	None
18238	RBFOX1	-0.00109	0.761493	0.9041	None
18239	AC007787.	-0.00104	0.761521	0.9041	None
18240	TRAM1L1	-0.03032	0.761624	0.9041	None
18241	SLC7A6	0.067066	0.761632	0.9041	None
18242	GABRA2	-0.03841	0.761692	0.9041	None
18243	CEACAM3	0.016871	0.761697	0.9041	None
18244	CNBP	0.0224	0.76173	0.9041	None
18245	LINC00471	-0.01598	0.761732	0.9041	None
18246	ADCY10P1	-0.03889	0.761808	0.904141	None
18247	CHM	0.04051	0.762088	0.9043	None
18248	LOC10029⁄	-0.01654	0.762094	0.9043	None
18249	LINC00299	-0.00242	0.762101	0.9043	None
18250	PCDHA5	-0.01304	0.762125	0.9043	None
18251	CNIH2	-0.00563	0.762164	0.9043	None
18252	NGLY1	-0.0524	0.762193	0.9043	None
18253	GVQW1	-0.01871	0.762327	0.90441	None
18254	NOXRED1	-0.00674	0.762386	0.90443	None
18255	GTPBP8	0.046662	0.762521	0.904444	None
18256	CD160	-0.02099	0.762525	0.904444	None
18257	TRMT2B	-0.05164	0.762535	0.904444	None
18258	C12orf56	-0.00053	0.762565	0.904444	None
18259	ATP10D	-0.07107	0.762659	0.904487	None
18260	CTNNBIP1	-0.04241	0.762685	0.904487	None
18261	TBX5	-0.00108	0.762745	0.904509	None
18262	CRYAB	0.00211	0.762813	0.90454	None
18263	DIDO1	-0.03492	0.762884	0.904575	None
18264	FOXD3-AS	-0.00849	0.763045	0.904704	None
18265	COX6B2	-0.00048	0.763102	0.904704	None
18266	LCMT1	-0.0431	0.763118	0.904704	None
18267	TMEM54	-0.00197	0.76323	0.904787	None
18268	SYCN	-0.0005	0.763312	0.904835	None

18269	PRPF40A	-0.03064	0.76339	0.904853	None
18270	SNX1	-0.05265	0.76345	0.904853	None
18271	KLHDC9	-0.02081	0.763463	0.904853	None
18272	DNASE1L3	0.093335	0.763495	0.904853	None
18273	LOC57453	-0.00091	0.763568	0.904891	None
18274	RAPSN	-0.00697	0.763614	0.904895	None
18275	RSPO3	-0.01418	0.763656	0.904896	None
18276	BAI1	0.015454	0.764141	0.905338	None
18277	RP11-285E	0.005503	0.764177	0.905338	None
18278	PNMAL1	-0.15833	0.764249	0.905338	None
18279	GFAP	-0.00049	0.76425	0.905338	None
18280	CNGA1	-0.01702	0.764275	0.905338	None
18281	ZNF416	-0.04256	0.76428	0.905338	None
18282	LOC28373	-0.00054	0.764399	0.905348	None
18283	IZUMO2	0.004565	0.764506	0.905348	None
18284	PRKACB	-0.04878	0.764556	0.905348	None
18285	KIR3DS1	-0.00095	0.764567	0.905348	None
18286	MAPK8IP3	0.018307	0.764598	0.905348	None
18287	PYGM	0.004512	0.76463	0.905348	None
18288	CCL19	0.002844	0.764689	0.905348	None
18289	FAM171A2	-0.00048	0.764772	0.905348	None
18290	ZNF426	-0.04928	0.764804	0.905348	None
18291	LINC00936	-0.09379	0.764819	0.905348	None
18292	CTD-2118I	0.002823	0.764823	0.905348	None
18293	LOC28466	-0.00328	0.764873	0.905348	None
18294	SFRP4	0.000415	0.764899	0.905348	None
18295	PAGE4	0.002116	0.764911	0.905348	None
18296	C3orf55	-0.01586	0.764915	0.905348	None
18297	KRT37	-0.00045	0.765066	0.905427	None
18298	HS6ST2	-0.03164	0.765066	0.905427	None
18299	CYP3A7-C	-0.00041	0.76514	0.905465	None
18300	STK19	0.030871	0.765229	0.905521	None
18301	MSC	-0.0293	0.765389	0.905661	None
18302	LBX1	-0.00057	0.765506	0.90575	None
18303	C9orf57	-0.00194	0.7656	0.905811	None
18304	DKKL1	0.012954	0.765687	0.905834	None
18305	RP11-143I	-0.0034	0.765711	0.905834	None
18306	VN1R3	-0.00044	0.765786	0.905834	None
18307	GPR19	0.018049	0.765786	0.905834	None
18308	MYL4	-0.08397	0.765892	0.905888	None
18309	ARF1	-0.07019	0.765916	0.905888	None
18310	TOP1P2	-0.00076	0.766014	0.905948	None
18311	BC023201	-0.0008	0.76605	0.905948	None
18312	B3GNT5	-0.05669	0.766211	0.906051	None
18313	HSD17B12	0.04018	0.766221	0.906051	None
18314	ZFP41	-0.037	0.766342	0.906145	None
18315	PDILT	-0.00181	0.766425	0.906193	None
18316	GPHB5	-0.00245	0.766639	0.906361	None
18317	POU5F2	0.002406	0.76665	0.906361	None
18318	SENP5	-0.04389	0.76678	0.906378	None
18319	CD247	0.035523	0.76679	0.906378	None
18320	RP11-85A	0.007345	0.766819	0.906378	None
18321	PCDH10	-0.00042	0.766832	0.906378	None
18322	HIST1H2BL	-0.00061	0.766936	0.906424	None
18323	LOC49725	-0.00058	0.766955	0.906424	None
18324	CCDC36	0.002499	0.767045	0.906481	None
18325	B4GALNT2	0.013734	0.767294	0.906583	None
18326	AX747630	-0.00245	0.767361	0.906583	None

18327	LOC10192[illegible]	-0.00075	0.767444	0.906583	None
18328	LOC10192[illegible]	-0.00248	0.767481	0.906583	None
18329	ENPP2	-0.13555	0.767494	0.906583	None
18330	RPL29	-0.03817	0.767496	0.906583	None
18331	PHF2	0.017858	0.767549	0.906583	None
18332	CXXC1P1	-0.00083	0.76757	0.906583	None
18333	TDH	0.003152	0.76759	0.906583	None
18334	LMAN1L	0.003402	0.767651	0.906583	None
18335	CDC20	-0.09749	0.767671	0.906583	None
18336	MPL	0.00903	0.767686	0.906583	None
18337	PMM1	0.027617	0.767734	0.906583	None
18338	FGF12	-0.0005	0.767906	0.906583	None
18339	FRY	-0.04684	0.767929	0.906583	None
18340	FAM229A	-0.0114	0.767933	0.906583	None
18341	TMEM38B	-0.04911	0.768008	0.906583	None
18342	HAO1	-0.00128	0.76801	0.906583	None
18343	DUS3L	0.030903	0.768061	0.906583	None
18344	SLC5A1	-0.00323	0.768075	0.906583	None
18345	LOC69224[illegible]	-0.00603	0.768098	0.906583	None
18346	UBE2D3	0.039927	0.768109	0.906583	None
18347	PCDHGA1	-0.00061	0.768111	0.906583	None
18348	LOC10012[illegible]	-0.01793	0.768136	0.906583	None
18349	ZNF713	0.02546	0.76825	0.906632	None
18350	PSG1	-0.0005	0.768334	0.906632	None
18351	RP11-75C[illegible]	-0.03326	0.768334	0.906632	None
18352	NPBWR2	-0.00221	0.768345	0.906632	None
18353	CERS3	0.00171	0.768461	0.906694	None
18354	GLRA1	0.000373	0.768495	0.906694	None
18355	MIR17HG	-0.01623	0.768523	0.906694	None
18356	ACE	-0.00709	0.768576	0.906707	None
18357	ZER1	-0.02225	0.768629	0.90672	None
18358	RGCC	-0.07916	0.7689	0.90695	None
18359	ANKRD34[illegible]	0.000442	0.768907	0.90695	None
18360	LOC10050[illegible]	0.01043	0.769156	0.907161	None
18361	CTRB2	0.010162	0.769181	0.907161	None
18362	LOC28576[illegible]	-0.00048	0.769223	0.907161	None
18363	KRT74	-0.00297	0.769347	0.907161	None
18364	LINC00622	-0.03927	0.769412	0.907161	None
18365	KRT36	-0.00072	0.769419	0.907161	None
18366	GS1-24F4.[illegible]	0.017249	0.769439	0.907161	None
18367	CCDC37	0.006801	0.769453	0.907161	None
18368	ELOVL6	0.087352	0.76949	0.907161	None
18369	RBBP7	-0.02536	0.769506	0.907161	None
18370	KRTAP4-1[illegible]	0.002893	0.769557	0.907172	None
18371	PSD4	0.022755	0.769635	0.907215	None
18372	F5	-0.04901	0.769879	0.907445	None
18373	ADRA2C	-0.02634	0.769914	0.907445	None
18374	ANTXR1	-0.00041	0.769957	0.907446	None
18375	FAM13C	-0.01083	0.770021	0.907473	None
18376	ZFP64	-0.00757	0.770112	0.90753	None
18377	FRMD5	0.022465	0.770158	0.907535	None
18378	HVCN1	-0.05528	0.770223	0.907563	None
18379	LOC28321[illegible]	-0.00165	0.770351	0.907663	None
18380	MUC13	0.009273	0.770538	0.907834	None
18381	LOC10192[illegible]	-0.00045	0.770664	0.907933	None
18382	GDF9	-0.00585	0.770871	0.908097	None
18383	CACNG1	0.009215	0.770887	0.908097	None
18384	PKIG	0.078923	0.770968	0.908133	None

18385	LOC10050(	-0.00311	0.771001	0.908133	None
18386	EN1	-0.00678	0.771174	0.908288	None
18387	ZNF48	0.032668	0.771302	0.908389	None
18388	LINC00943	-0.00408	0.771388	0.908441	None
18389	PTGFR	-0.00171	0.771738	0.908779	None
18390	STAU1	-0.0367	0.771801	0.908779	None
18391	NOX3	0.002309	0.771801	0.908779	None
18392	LOC10272	0.003018	0.771884	0.908827	None
18393	TEDDM1	0.004378	0.772042	0.908932	None
18394	C4orf26	0.000572	0.772068	0.908932	None
18395	NFKBIA	0.051234	0.772099	0.908932	None
18396	TMEM222	-0.04038	0.772145	0.908937	None
18397	RP5-1068E	0.012922	0.772239	0.908949	None
18398	TAT	0.000694	0.772241	0.908949	None
18399	C1orf56	-0.01871	0.772281	0.908949	None
18400	NDP	-0.00368	0.772777	0.909386	None
18401	ASTN1	-0.00053	0.77278	0.909386	None
18402	RHBDD2	-0.04003	0.77283	0.909386	None
18403	PLSCR1	-0.05075	0.772832	0.909386	None
18404	DNAJB11	0.047133	0.77287	0.909386	None
18405	LINC00561	-0.0005	0.772905	0.909386	None
18406	COL12A1	0.000399	0.773002	0.909451	None
18407	MED26	-0.02419	0.773148	0.909573	None
18408	ENDOU	0.003248	0.773257	0.909642	None
18409	COL15A1	-0.02701	0.77329	0.909642	None
18410	LOC10193(	0.020578	0.773512	0.909843	None
18411	SLC8A3	0.045017	0.773545	0.909843	None
18412	HLA-F-AS:	-0.03307	0.773646	0.909845	None
18413	GSTZ1	0.051547	0.773672	0.909845	None
18414	ZBTB5	-0.04733	0.77369	0.909845	None
18415	LOC10050(	-0.00072	0.773715	0.909845	None
18416	TP53AIP1	0.001056	0.773899	0.909966	None
18417	ATP8B3	-0.00932	0.774057	0.909966	None
18418	S100A16	0.075778	0.774063	0.909966	None
18419	FAM24A	-0.00046	0.774092	0.909966	None
18420	TST	-0.09721	0.774137	0.909966	None
18421	ASAP1-IT1	0.049762	0.774198	0.909966	None
18422	TIAF1	-0.02322	0.774198	0.909966	None
18423	FSIP2	-0.00213	0.774199	0.909966	None
18424	CPNE9	-0.01634	0.77427	0.909966	None
18425	DSC1	-0.00378	0.774305	0.909966	None
18426	TMCO1	-0.02056	0.774377	0.909966	None
18427	FTSJ1	0.036442	0.774386	0.909966	None
18428	AVL9	0.026419	0.774395	0.909966	None
18429	GOLT1B	-0.03036	0.774457	0.909966	None
18430	NAA25	0.044863	0.774508	0.909966	None
18431	PFKL	-0.03669	0.774541	0.909966	None
18432	SPATA13	0.034115	0.774611	0.909966	None
18433	C3AR1	0.117098	0.774626	0.909966	None
18434	LOC10192	-0.00142	0.774647	0.909966	None
18435	TYMSOS	-0.01715	0.774658	0.909966	None
18436	UBL7	-0.04499	0.774786	0.910024	None
18437	ANKRD30E	-0.00703	0.774792	0.910024	None
18438	SORBS2	-0.00075	0.774924	0.91013	None
18439	AP000230.	-0.00828	0.775021	0.910152	None
18440	ZDHHC21	0.038527	0.775027	0.910152	None
18441	RP1-155D2	0.001198	0.775167	0.91023	None
18442	CORIN	-0.00074	0.77518	0.91023	None

18443	GS1-124K5	-0.00073	0.775256	0.91023	None
18444	NLRP6	-0.00045	0.775274	0.91023	None
18445	CPD	-0.05299	0.775303	0.91023	None
18446	CTC-384G	-0.0082	0.775508	0.910421	None
18447	SLC5A10	-0.02132	0.775616	0.910498	None
18448	COMMD4	-0.06027	0.775832	0.910498	None
18449	SPPL2A	0.066954	0.775835	0.910498	None
18450	AP5B1	-0.03833	0.775847	0.910498	None
18451	FAM26F	-0.05555	0.775866	0.910498	None
18452	RPL39	-0.00868	0.775873	0.910498	None
18453	LOC10028	-0.00784	0.775916	0.910498	None
18454	TMEM187	-0.0532	0.775926	0.910498	None
18455	VKORC1	-0.04524	0.776007	0.910498	None
18456	LINC00176	0.007093	0.776028	0.910498	None
18457	RP11-673E	0.004042	0.776036	0.910498	None
18458	CCT7	0.034154	0.776093	0.910515	None
18459	LOC64732	0.00071	0.776193	0.910583	None
18460	SPINT3	-0.00042	0.776334	0.91065	None
18461	LOC10192	-0.00073	0.776369	0.91065	None
18462	FLNB	-0.05589	0.776376	0.91065	None
18463	NAALADL1	-0.06602	0.776454	0.910692	None
18464	AP3S1	0.030888	0.776602	0.91077	None
18465	ADCY2	-0.00274	0.776662	0.91077	None
18466	SLK	0.032199	0.776706	0.91077	None
18467	LOC10050	-0.00049	0.776708	0.91077	None
18468	OTUD7A	0.000359	0.77673	0.91077	None
18469	TRIML1	-0.00072	0.776976	0.911008	None
18470	LOC28519	-0.01018	0.777083	0.911065	None
18471	CA11	0.030301	0.777108	0.911065	None
18472	AF289551	0.041348	0.777213	0.911139	None
18473	HABP4	-0.02499	0.777348	0.911247	None
18474	ABHD16A	-0.02582	0.777404	0.911264	None
18475	IQCF3	-0.00151	0.777492	0.91129	None
18476	AK1	-0.05277	0.777545	0.91129	None
18477	LOC10050	0.001394	0.777553	0.91129	None
18478	OCM2	0.002489	0.777596	0.911292	None
18479	CP	-0.06419	0.777686	0.911347	None
18480	ADAM19	-0.0274	0.777752	0.911376	None
18481	RETN	0.060746	0.777845	0.911415	None
18482	IER2	-0.06601	0.77787	0.911415	None
18483	ZNF440	-0.03088	0.777951	0.911461	None
18484	PRM1	-0.00253	0.778026	0.911496	None
18485	SCEL	-0.00038	0.778065	0.911496	None
18486	SETD1B	-0.02848	0.778155	0.911522	None
18487	TYRO3	0.01287	0.778171	0.911522	None
18488	KCNK6	-0.04544	0.778219	0.911529	None
18489	CASKIN1	0.003394	0.778353	0.911635	None
18490	LOC10050	-0.02866	0.778428	0.911674	None
18491	KCNA7	-0.0004	0.778637	0.91187	None
18492	RP11-379F	0.018342	0.778737	0.911937	None
18493	LINC00589	-0.00255	0.778805	0.911968	None
18494	LINC00923	-0.01738	0.779192	0.912331	None
18495	KLK11	-0.00357	0.779199	0.912331	None
18496	TRBC1	0.096279	0.779438	0.912561	None
18497	TMEM35	-0.00041	0.779485	0.912567	None
18498	KCNK4	-0.00069	0.779544	0.912587	None
18499	C2orf57	-0.0044	0.779698	0.912644	None
18500	TTLL2	-0.00107	0.779746	0.912644	None

18501	NHSL2	-0.00303	0.779759	0.912644	None
18502	RP3-400B1	-0.00095	0.779787	0.912644	None
18503	SLC4A4	0.004976	0.779827	0.912644	None
18504	NTAN1	-0.04867	0.779884	0.912644	None
18505	CSE1L	0.048823	0.779888	0.912644	None
18506	GLIDR	-0.03936	0.780034	0.91271	None
18507	FOLR3	0.064496	0.780039	0.91271	None
18508	LINC00518	-0.00041	0.780071	0.91271	None
18509	TRMT10C	-0.02839	0.780127	0.912721	None
18510	TTPA	-0.00052	0.780232	0.912721	None
18511	LOC10192	-0.00046	0.78026	0.912721	None
18512	PLD2	-0.02382	0.78027	0.912721	None
18513	PARL	-0.01558	0.780324	0.912721	None
18514	LOC10192	-0.0581	0.780333	0.912721	None
18515	CCDC167	-0.05447	0.780637	0.912984	None
18516	MROH2B	-0.00067	0.780643	0.912984	None
18517	RNF121	-0.03851	0.780815	0.913017	None
18518	UBE2D1	-0.06428	0.780833	0.913017	None
18519	ZNF91	-0.02551	0.780837	0.913017	None
18520	PRIM2	0.051938	0.780839	0.913017	None
18521	ASPN	-0.00287	0.781151	0.913308	None
18522	PDRG1	-0.05055	0.781172	0.913308	None
18523	C2orf61	-0.00044	0.781297	0.91337	None
18524	PDCD7	0.018939	0.781312	0.91337	None
18525	ACVR2B	0.04517	0.781352	0.91337	None
18526	RP11-309C	-0.03954	0.781488	0.913476	None
18527	CCL26	-0.00583	0.781527	0.913476	None
18528	GSDMB	-0.0313	0.781613	0.913521	None
18529	LOC10192	-0.00294	0.781659	0.913521	None
18530	ZSCAN12P	0.004911	0.781693	0.913521	None
18531	PPP1R14A	-0.11963	0.781735	0.913522	None
18532	LOC10192	0.00378	0.782193	0.914007	None
18533	LARS	-0.03631	0.782326	0.914114	None
18534	UBE2N	-0.03283	0.782449	0.914163	None
18535	KCNU1	-0.00056	0.782452	0.914163	None
18536	SLC7A14	0.004209	0.782572	0.914253	None
18537	ADAT3	0.003639	0.782645	0.914289	None
18538	C8orf4	-0.06768	0.782693	0.914296	None
18539	ZIC5	-0.00941	0.782816	0.914368	None
18540	NOX1	-0.00243	0.78287	0.914368	None
18541	UGT2B17	-0.00859	0.782935	0.914368	None
18542	LIN28B	-0.02658	0.783	0.914368	None
18543	CTC1	-0.02895	0.783018	0.914368	None
18544	S100A9	-0.16169	0.783023	0.914368	None
18545	PTGER4P2	-0.00045	0.78305	0.914368	None
18546	CDC73	0.038664	0.783119	0.914393	None
18547	LOC10192	-0.00823	0.783178	0.914393	None
18548	ITIH1	-0.00301	0.783199	0.914393	None
18549	SFXN2	0.036588	0.783279	0.914413	None
18550	COMMD8	0.04863	0.783318	0.914413	None
18551	C9orf24	-0.01496	0.783382	0.914413	None
18552	CBLL1	-0.0636	0.783384	0.914413	None
18553	PAPPA	-0.0079	0.783541	0.914458	None
18554	LOC10192	0.00323	0.783584	0.914458	None
18555	LIN7B	-0.02412	0.783611	0.914458	None
18556	KLF6	0.078509	0.783692	0.914458	None
18557	ZNF383	-0.04108	0.783729	0.914458	None
18558	LINC01207	-0.00058	0.783771	0.914458	None

18559	BLCAP	0.043685	0.783783	0.914458	None
18560	NOVA2	-0.00594	0.783804	0.914458	None
18561	ZNF430	0.044515	0.783881	0.914458	None
18562	LOC28429	-0.00058	0.783904	0.914458	None
18563	ZSWIM2	-0.00042	0.78395	0.914458	None
18564	CAPS2	-0.02234	0.783956	0.914458	None
18565	FGF3	-0.00043	0.783972	0.914458	None
18566	CTB-12O2	-0.00879	0.784098	0.914547	None
18567	ITPK1-AS1	0.030256	0.784175	0.914547	None
18568	CENPW	0.05526	0.784245	0.914547	None
18569	LRRC34	0.060927	0.784253	0.914547	None
18570	POLR2J4	0.04229	0.784279	0.914547	None
18571	TTTY15	-0.07198	0.784301	0.914547	None
18572	AX747250	-0.0006	0.784449	0.91467	None
18573	PECAM1	0.054528	0.784587	0.914777	None
18574	CASP8	-0.03937	0.784625	0.914777	None
18575	MAGEC1	-0.00043	0.784673	0.914783	None
18576	FLJ31104	-0.0074	0.785031	0.91515	None
18577	RP11-381F	-0.00725	0.785145	0.915234	None
18578	KDR	0.09307	0.785259	0.915272	None
18579	LOC20083	0.007893	0.785302	0.915272	None
18580	RHBDL3	-0.00156	0.785304	0.915272	None
18581	RP11-313C	-0.0154	0.785358	0.915286	None
18582	DDHD2	0.034017	0.78547	0.915367	None
18583	UGT2A3	-0.00039	0.785528	0.915385	None
18584	PDHX	-0.02427	0.785576	0.915392	None
18585	MLLT11	0.051467	0.785828	0.915626	None
18586	DSCR4	-0.00043	0.785898	0.915626	None
18587	TKTL2	-0.00051	0.785903	0.915626	None
18588	GORAB	0.051595	0.786137	0.915809	None
18589	C21orf128	-0.00039	0.786161	0.915809	None
18590	EOMES	-0.01619	0.786216	0.915809	None
18591	LOC10192	-0.00059	0.78624	0.915809	None
18592	SMC3	-0.05484	0.786272	0.915809	None
18593	HLA-A	-0.02735	0.786394	0.915881	None
18594	RGAG1	-0.00853	0.786418	0.915881	None
18595	PELI3	0.031522	0.786539	0.915972	None
18596	RALGPS2	-0.05004	0.786725	0.916134	None
18597	RP5-935K1	-0.02715	0.786763	0.916134	None
18598	LRRC72	-0.00551	0.786908	0.916254	None
18599	CCDC108	-0.0005	0.786958	0.916263	None
18600	NLRP4	-0.00225	0.787035	0.916303	None
18601	CCDC151	-0.0293	0.787175	0.916417	None
18602	LGR4	0.012807	0.787475	0.916717	None
18603	RP5-991O	-0.00121	0.787637	0.916842	None
18604	R3HCC1	-0.03943	0.787667	0.916842	None
18605	PRELP	-0.00413	0.787751	0.916852	None
18606	LOC10192	0.000993	0.787771	0.916852	None
18607	KIAA1683	-0.02544	0.787828	0.916852	None
18608	NECAB2	0.012616	0.787845	0.916852	None
18609	CPN1	-0.00232	0.787915	0.916885	None
18610	FOSL1	-0.06029	0.787985	0.916916	None
18611	HLA-DPA1	-0.06222	0.788088	0.91696	None
18612	RP11-319E	-0.02058	0.788124	0.91696	None
18613	PPP1R1C	-0.00669	0.788243	0.91696	None
18614	KRT76	-0.00735	0.788277	0.91696	None
18615	LOC10192	-0.00797	0.788281	0.91696	None
18616	A2ML1	-0.00527	0.788292	0.91696	None

18617	TENM1	-0.01052	0.788319	0.91696	None
18618	PRB4	-0.01085	0.788491	0.917077	None
18619	LAMB1	0.041391	0.788553	0.917077	None
18620	ZNF93	-0.02502	0.788562	0.917077	None
18621	HECTD1	-0.05031	0.788589	0.917077	None
18622	ZNF624	-0.04081	0.788717	0.917176	None
18623	PACSIN1	0.036786	0.788763	0.917182	None
18624	NOTCH2N	-0.05255	0.78885	0.917232	None
18625	DEPTOR	0.02736	0.789057	0.917344	None
18626	LOC33866	-0.00059	0.789068	0.917344	None
18627	ZNF775	-0.00422	0.789072	0.917344	None
18628	LOC10192	-0.00759	0.789412	0.917687	None
18629	OPA3	-0.02392	0.789452	0.917687	None
18630	TADA2B	-0.04384	0.789552	0.917707	None
18631	SLC34A1	0.007234	0.789554	0.917707	None
18632	BRAF	-0.0389	0.789714	0.917844	None
18633	TBC1D19	0.043459	0.789794	0.917849	None
18634	PSMD1	-0.0271	0.789841	0.917849	None
18635	RP11-171N	-0.00051	0.789875	0.917849	None
18636	PAEP	-0.0004	0.789888	0.917849	None
18637	PIK3C3	-0.03803	0.789965	0.917889	None
18638	RNF208	-0.00058	0.790093	0.917988	None
18639	DUSP2	-0.10069	0.790177	0.918026	None
18640	ACTR8	-0.02949	0.790211	0.918026	None
18641	NR2C1	0.026012	0.790285	0.918033	None
18642	CACNA2D3	-0.10696	0.790301	0.918033	None
18643	UBE2E2-AS	-0.00119	0.790377	0.918063	None
18644	MGC34800	0.008874	0.790412	0.918063	None
18645	OR7E19P	-0.00136	0.790624	0.91826	None
18646	RP11-461A	-0.00889	0.79103	0.918683	None
18647	CCDC63	-0.00461	0.791099	0.918713	None
18648	RAD18	-0.0473	0.791142	0.918714	None
18649	CPSF4	-0.04234	0.79153	0.919116	None
18650	LOC10192	-0.02364	0.791661	0.919218	None
18651	FAM71F1	-0.00814	0.791805	0.919336	None
18652	CCDC146	-0.03904	0.792004	0.919518	None
18653	PKP2	0.050074	0.792196	0.919692	None
18654	INO80D	-0.03078	0.792263	0.91972	None
18655	EYS	-0.00128	0.792415	0.919773	None
18656	SPNS3	-0.09248	0.792444	0.919773	None
18657	DRICH1	0.007292	0.79251	0.919773	None
18658	RP4-635E1	0.052284	0.792527	0.919773	None
18659	SMCR5	0.003013	0.792571	0.919773	None
18660	HGSNAT	0.045766	0.792593	0.919773	None
18661	GSPT2	0.052453	0.792606	0.919773	None
18662	SLC35C2	-0.02702	0.79293	0.920035	None
18663	C8orf12	-0.00056	0.792934	0.920035	None
18664	MRPL45P2	0.007105	0.792991	0.920035	None
18665	C2CD5	0.033681	0.793014	0.920035	None
18666	RP11-466F	-0.00407	0.793044	0.920035	None
18667	LINC00630	-0.00748	0.793125	0.920079	None
18668	APOH	0.003582	0.793197	0.920113	None
18669	VSTM2A	-0.00055	0.793295	0.920178	None
18670	C1orf106	-0.03451	0.793686	0.920565	None
18671	ATF3	-0.04262	0.793714	0.920565	None
18672	AGBL5	-0.03274	0.793815	0.920634	None
18673	LCN15	0.01353	0.793887	0.920667	None
18674	TRIM36	-0.00042	0.794068	0.920742	None

18675	UTP23	0.026347	0.794078	0.920742	None
18676	EXOSC1	-0.02972	0.794092	0.920742	None
18677	NKPD1	0.008762	0.794121	0.920742	None
18678	FAM9A	-0.00807	0.794335	0.920881	None
18679	DPY19L2	-0.07219	0.794381	0.920881	None
18680	TNFSF11	-0.00756	0.794425	0.920881	None
18681	GNRH2	-0.00927	0.794428	0.920881	None
18682	MMP20	-0.00043	0.794454	0.920881	None
18683	PERM1	0.007944	0.79451	0.920896	None
18684	LOC10192	-0.00049	0.794703	0.921071	None
18685	UNC13D	-0.01675	0.794913	0.921221	None
18686	RP11-91I8.	-0.00039	0.794952	0.921221	None
18687	DEFB127	-0.00043	0.79496	0.921221	None
18688	MYNN	-0.0372	0.795156	0.921399	None
18689	TNFRSF18	0.008223	0.795244	0.921408	None
18690	RP11-714L	-0.00428	0.795249	0.921408	None
18691	EVI5L	-0.00243	0.795372	0.921484	None
18692	GJA5	-0.01825	0.795436	0.921484	None
18693	CD163L1	-0.04499	0.795442	0.921484	None
18694	MYL5	0.043274	0.795526	0.921532	None
18695	GPR31	-0.00044	0.795576	0.92154	None
18696	MLN	-0.00585	0.795625	0.921546	None
18697	LOC10192	-0.00047	0.795666	0.921546	None
18698	SLC5A7	-0.00334	0.795799	0.921651	None
18699	VPS26A	0.024636	0.795854	0.921657	None
18700	TRAF1	0.008185	0.79589	0.921657	None
18701	GABRQ	-0.00243	0.795962	0.921692	None
18702	FOXF2	0.002621	0.796062	0.921758	None
18703	LOC10192	-0.00537	0.796118	0.921774	None
18704	USP26	-0.00046	0.796225	0.921818	None
18705	PABPN1	-0.05028	0.796282	0.921818	None
18706	ZNF391	-0.00325	0.796308	0.921818	None
18707	SAMD11	0.005462	0.796328	0.921818	None
18708	HLA-DOA	0.013478	0.796369	0.921818	None
18709	PRO2214	0.002678	0.796501	0.921921	None
18710	CCDC7	0.020542	0.796657	0.921973	None
18711	AJAP1	-0.00053	0.79673	0.921973	None
18712	PCDP1	-0.0004	0.79673	0.921973	None
18713	ZNF701	0.016546	0.796731	0.921973	None
18714	ZFY	-0.07947	0.796758	0.921973	None
18715	ZNF32-AS	-0.00055	0.79687	0.922052	None
18716	LOC10012	0.024867	0.796931	0.922074	None
18717	NOL3	0.012015	0.797045	0.922157	None
18718	BEND7	-0.00332	0.797089	0.922158	None
18719	MAGEB1	-0.00235	0.797218	0.922258	None
18720	SPTAN1	-0.02734	0.797597	0.922647	None
18721	RP11-548H	-0.01603	0.797865	0.922908	None
18722	CARD14	-0.00303	0.798005	0.923021	None
18723	BCAR1	0.008369	0.798077	0.923029	None
18724	ZNF493	-0.03945	0.798118	0.923029	None
18725	RPS16P5	-0.06872	0.798173	0.923029	None
18726	PDGFRA	-0.01114	0.798183	0.923029	None
18727	LINC00309	-0.00035	0.798325	0.92307	None
18728	FAM110C	-0.00298	0.798334	0.92307	None
18729	DAB2IP	-0.00539	0.798346	0.92307	None
18730	SEMA3F	-0.02781	0.798437	0.923126	None
18731	MIXL1	-0.00899	0.798547	0.923204	None
18732	LOC10012	-0.00801	0.798609	0.923226	None

18733	IL21	-0.00046	0.798717	0.923302	None
18734	LINC00672	-0.03056	0.798933	0.923473	None
18735	SMOX	0.018373	0.79895	0.923473	None
18736	MOG	-0.00047	0.799097	0.923485	None
18737	WDR88	-0.00048	0.799117	0.923485	None
18738	OPALIN	-0.00043	0.799128	0.923485	None
18739	SMC6	-0.03408	0.799132	0.923485	None
18740	GNAT2	-0.00543	0.799265	0.92359	None
18741	SCAI	-0.04716	0.799426	0.923727	None
18742	SSNA1	0.034056	0.799515	0.923739	None
18743	ZNF253	-0.03041	0.799525	0.923739	None
18744	COMP	0.003859	0.799673	0.923739	None
18745	ZFHX3	-0.01151	0.799714	0.923739	None
18746	CYP2C18	-0.02266	0.799733	0.923739	None
18747	IL1R2	0.074048	0.799742	0.923739	None
18748	PPIF	0.06068	0.799802	0.923739	None
18749	GCKR	0.011833	0.799814	0.923739	None
18750	SNRPD2	-0.04141	0.799903	0.923739	None
18751	RP11-748H	-0.00491	0.799915	0.923739	None
18752	LOC10254(	0.000354	0.799938	0.923739	None
18753	PROK1	0.002219	0.799974	0.923739	None
18754	EGLN3	-0.056	0.799991	0.923739	None
18755	IPO5	-0.03403	0.800163	0.923889	None
18756	SPINK7	-0.0005	0.800477	0.924202	None
18757	KRT8P12	-0.01572	0.800828	0.924558	None
18758	APOBEC2	0.015326	0.801073	0.924779	None
18759	ACTA1	0.00307	0.801105	0.924779	None
18760	CAPZA2	0.053611	0.801289	0.924942	None
18761	ATF4	-0.02263	0.801436	0.925024	None
18762	PLIN2	0.031422	0.801445	0.925024	None
18763	GPR15	-0.00042	0.801505	0.925035	None
18764	CBL	-0.08442	0.801574	0.925035	None
18765	SH3GLB2	-0.01731	0.801583	0.925035	None
18766	ACADM	0.049299	0.801825	0.925203	None
18767	PANK2	-0.01745	0.801858	0.925203	None
18768	CD70	-0.01277	0.801865	0.925203	None
18769	SEC22C	-0.02941	0.8019	0.925203	None
18770	LOC101928	-0.00043	0.801972	0.925238	None
18771	PAX7	0.003728	0.802206	0.925425	None
18772	PKD2	0.050369	0.802256	0.925425	None
18773	ATP10A	-0.00593	0.802263	0.925425	None
18774	DRAM2	-0.02979	0.802327	0.925449	None
18775	MAB21L2	-0.00475	0.802423	0.925511	None
18776	CCDC110	-0.00197	0.802716	0.925767	None
18777	PIH1D1	-0.03287	0.802761	0.925767	None
18778	OR14J1	-0.00049	0.802798	0.925767	None
18779	TSPAN10	-0.00846	0.802816	0.925767	None
18780	ATP5G2	0.024853	0.80286	0.925768	None
18781	LINC00662	-0.02568	0.803126	0.925985	None
18782	TAPBP	-0.02921	0.803134	0.925985	None
18783	C2orf71	-0.00049	0.803228	0.926045	None
18784	MYCT1	-0.06959	0.803369	0.926158	None
18785	PGAM2	-0.00307	0.803424	0.926173	None
18786	C12orf55	-0.00127	0.803544	0.926261	None
18787	HMGCLL1	-0.00043	0.803754	0.92641	None
18788	ICE2	-0.02814	0.803758	0.92641	None
18789	SLC25A5-/	-0.0109	0.803831	0.926445	None
18790	APOO	0.043874	0.804015	0.926561	None

18791	GLI4	-0.00131	0.804043	0.926561	None
18792	SYT8	0.004301	0.804061	0.926561	None
18793	EVA1C	0.06519	0.804259	0.926671	None
18794	CCDC122	-0.00808	0.804263	0.926671	None
18795	TRIM3	0.003933	0.804284	0.926671	None
18796	LYST	-0.04442	0.804446	0.926755	None
18797	SF3B1	-0.04138	0.804466	0.926755	None
18798	KIF1C	0.020992	0.804486	0.926755	None
18799	RPS19	-0.013	0.804563	0.926795	None
18800	ZNHIT1	-0.03987	0.804641	0.926836	None
18801	FLJ16734	-0.01736	0.804779	0.926875	None
18802	RNF212	-0.0073	0.804795	0.926875	None
18803	GREM1	0.001334	0.80481	0.926875	None
18804	RP5-968J1	-0.01451	0.804954	0.926875	None
18805	LINC00606	-0.0004	0.804969	0.926875	None
18806	TAS2R13	-0.00038	0.804983	0.926875	None
18807	ANKRD60	-0.00054	0.804993	0.926875	None
18808	LOC10272:	-0.00342	0.805042	0.926875	None
18809	C9orf9	-0.0147	0.805061	0.926875	None
18810	MAST1	-0.0011	0.805251	0.926966	None
18811	CRLS1	0.043906	0.805268	0.926966	None
18812	ZBTB10	0.043899	0.805278	0.926966	None
18813	ADAM28	-0.0751	0.80533	0.926966	None
18814	LGALS2	-0.07314	0.805354	0.926966	None
18815	CLDN14	-0.01009	0.805474	0.927055	None
18816	ERV3-2	0.069018	0.805632	0.927107	None
18817	LOC10106(	-0.00036	0.805649	0.927107	None
18818	LOC40122(	-0.00047	0.805691	0.927107	None
18819	PLCB1	0.01784	0.805695	0.927107	None
18820	KCNJ16	-0.00368	0.805787	0.927107	None
18821	SLC5A12	-0.00125	0.805799	0.927107	None
18822	LOC10192	-0.00044	0.805825	0.927107	None
18823	DTNA	-0.00036	0.805888	0.927107	None
18824	MFSD7	0.009425	0.805904	0.927107	None
18825	FBXL22	-0.00069	0.806001	0.927169	None
18826	TMEM196	-0.00147	0.806178	0.927324	None
18827	RN7SKP15(	-0.00047	0.806232	0.927336	None
18828	PRKAR2A	-0.02821	0.806305	0.927371	None
18829	GPR12	0.007017	0.80637	0.927373	None
18830	DEPDC1	-0.06677	0.806409	0.927373	None
18831	CHAD	-0.01038	0.806436	0.927373	None
18832	SLC25A2	-0.0036	0.806568	0.927434	None
18833	CTC-459F4	-0.04772	0.806574	0.927434	None
18834	VGLL3	0.027522	0.806733	0.92756	None
18835	C1orf86	0.00342	0.806808	0.92756	None
18836	LOC10192	-0.01888	0.806932	0.92756	None
18837	VGLL2	-0.01126	0.806949	0.92756	None
18838	ZBED2	-0.00046	0.806951	0.92756	None
18839	ACTN3	0.001654	0.806982	0.92756	None
18840	LINC01029	0.001768	0.807012	0.92756	None
18841	BC043227	0.00205	0.807043	0.92756	None
18842	PDZD8	0.056114	0.807069	0.92756	None
18843	CHDC2	-0.00458	0.80715	0.927579	None
18844	KCNK2	0.001226	0.807172	0.927579	None
18845	PRAP1	0.001055	0.807441	0.927839	None
18846	LOC28581!	-0.00274	0.807539	0.927903	None
18847	SNX9	0.072612	0.807695	0.928027	None
18848	RTF1	0.03926	0.807752	0.928027	None

18849	CDH9	0.107781	0.807776	0.928027	None
18850	AC068831.	-0.011	0.807898	0.928119	None
18851	KRT40	-0.00119	0.807989	0.928126	None
18852	PIKFYVE	0.036714	0.80799	0.928126	None
18853	SNRK	0.063561	0.808109	0.928213	None
18854	LRRC57	-0.01753	0.808219	0.92829	None
18855	DOHH	-0.03131	0.808293	0.928326	None
18856	ARHGAP36	-0.00044	0.808433	0.928438	None
18857	LOC10192	-0.00486	0.808514	0.92844	None
18858	UBE2B	0.040822	0.808531	0.92844	None
18859	CTD-2251I	-0.0029	0.808579	0.92844	None
18860	NXF1	0.032363	0.808607	0.92844	None
18861	CHD9	0.050309	0.808826	0.928558	None
18862	TMEM141	0.043429	0.808835	0.928558	None
18863	UGT1A6	-0.00042	0.808859	0.928558	None
18864	DYNLL2	-0.03676	0.808922	0.928558	None
18865	RP11-543C	-0.00363	0.808931	0.928558	None
18866	C6orf10	-0.00052	0.809008	0.928558	None
18867	RP11-461L	-0.00552	0.80901	0.928558	None
18868	NSMF	0.015096	0.809334	0.928881	None
18869	FLOT1	-0.02551	0.809455	0.92897	None
18870	RP11-747H	0.038889	0.809501	0.928975	None
18871	TTLL4	0.012771	0.809606	0.929045	None
18872	LOC10192	0.04735	0.809723	0.929131	None
18873	FGF21	0.007605	0.809915	0.929279	None
18874	TCF19	0.048155	0.80998	0.929279	None
18875	TRIM67	-0.00594	0.809981	0.929279	None
18876	PSMG4	-0.02428	0.810092	0.929344	None
18877	SH2D5	0.002884	0.810142	0.929344	None
18878	PPBP	-0.15199	0.810167	0.929344	None
18879	SLCO2B1	-0.0569	0.810309	0.929387	None
18880	ZFHX4	-0.00045	0.81031	0.929387	None
18881	SLIT3	-0.00462	0.810332	0.929387	None
18882	LRRC19	-0.00076	0.810416	0.929433	None
18883	ATP2A2	0.025741	0.810498	0.929441	None
18884	FSHB	0.005351	0.810525	0.929441	None
18885	IPO5P1	0.004723	0.81059	0.929441	None
18886	PATE2	0.003011	0.810594	0.929441	None
18887	HSD11B2	-0.00035	0.810752	0.929573	None
18888	LOC10013	-0.00044	0.810853	0.929583	None
18889	SUCNR1	0.099082	0.810874	0.929583	None
18890	TTK	-0.06181	0.810934	0.929583	None
18891	ZNF732	0.000935	0.810935	0.929583	None
18892	ENPEP	-0.02999	0.810976	0.929583	None
18893	LY6H	-0.01076	0.811024	0.929589	None
18894	TCF25	0.031859	0.811258	0.929808	None
18895	LIN7C	-0.02933	0.81135	0.929834	None
18896	KIAA0895	0.043763	0.811366	0.929834	None
18897	LINC01300	0.007643	0.811447	0.929877	None
18898	TOX4	0.034328	0.811767	0.930082	None
18899	TRMT5	0.026494	0.811826	0.930082	None
18900	BX648501	-0.00036	0.81184	0.930082	None
18901	SPRY3	-0.01169	0.81186	0.930082	None
18902	CLRN3	-0.00042	0.811936	0.930082	None
18903	SERPINI2	-0.03129	0.812034	0.930082	None
18904	CTA-280A	-0.00798	0.812071	0.930082	None
18905	RTFDC1	-0.0265	0.812109	0.930082	None
18906	GPR1	0.008826	0.812138	0.930082	None

18907	PDC	-0.00037	0.812146	0.930082	None
18908	SNX20	-0.02266	0.812166	0.930082	None
18909	SLC16A8	0.016294	0.812181	0.930082	None
18910	IFI16	0.035675	0.812185	0.930082	None
18911	LOC10192	0.008018	0.812293	0.930158	None
18912	TTC30B	0.029397	0.812414	0.930246	None
18913	ING2	-0.01397	0.812496	0.930292	None
18914	PLEKHG5	-0.01662	0.812789	0.930551	None
18915	LRPPRC	0.025985	0.812808	0.930551	None
18916	RP11-700J	-0.06442	0.812884	0.930582	None
18917	HAPLN1	-0.00032	0.81297	0.930582	None
18918	KLF13	-0.07111	0.812989	0.930582	None
18919	ZNF780B	-0.01102	0.813008	0.930582	None
18920	LOC10050	0.00522	0.813096	0.930598	None
18921	ABCG5	-0.00044	0.813124	0.930598	None
18922	RP11-10N	-0.00282	0.813151	0.930598	None
18923	POLDIP3	-0.01568	0.813201	0.930606	None
18924	MED24	-0.00043	0.813295	0.930649	None
18925	ZNF385A	-0.02791	0.813356	0.930649	None
18926	LOC10063	-0.00106	0.813367	0.930649	None
18927	RP1	-0.00559	0.813448	0.930693	None
18928	WFDC21P	-0.00504	0.81353	0.93072	None
18929	CDT1	-0.05074	0.813641	0.93072	None
18930	LINC00342	0.044972	0.813676	0.93072	None
18931	B3GALT1	0.00332	0.813714	0.93072	None
18932	PRKRIR	0.021023	0.81373	0.93072	None
18933	LOC10050	-0.00035	0.813749	0.93072	None
18934	APOBEC3C	-0.01789	0.813773	0.93072	None
18935	ACVR1B	0.007359	0.813825	0.93073	None
18936	SERPINA7	-0.00042	0.813983	0.930828	None
18937	TAAR2	-0.00043	0.814104	0.930828	None
18938	LOC10192	0.000314	0.814185	0.930828	None
18939	MIR124-2H	-0.00035	0.814199	0.930828	None
18940	LOC10192	-0.00235	0.814206	0.930828	None
18941	KLHL4	-0.00032	0.814207	0.930828	None
18942	SRSF7	0.029471	0.814211	0.930828	None
18943	FAM96A	0.030039	0.814382	0.930905	None
18944	ZFP14	-0.02956	0.814396	0.930905	None
18945	IGSF3	0.001614	0.814408	0.930905	None
18946	APOBEC1	-0.00623	0.814523	0.930968	None
18947	LOC28562	-0.00034	0.814566	0.930968	None
18948	OR1C1	-0.0045	0.814591	0.930968	None
18949	LOC1720	-0.00349	0.814715	0.93106	None
18950	AC007401.	0.029994	0.814874	0.931192	None
18951	LOC44041	-0.00034	0.814938	0.931215	None
18952	LOC10272	-0.00102	0.815	0.931215	None
18953	MCHR1	-0.00042	0.815022	0.931215	None
18954	AKR7A3	0.028632	0.815459	0.931597	None
18955	RAB28	0.025562	0.815485	0.931597	None
18956	PANX3	-0.00179	0.815487	0.931597	None
18957	ACADSB	-0.02681	0.815566	0.93164	None
18958	LINC00202	-0.01783	0.815677	0.931717	None
18959	FIS1	-0.04576	0.815779	0.931784	None
18960	PCSK2	-0.04508	0.815849	0.931789	None
18961	CCDC121	0.025936	0.81587	0.931789	None
18962	CLDN7	-0.01081	0.816121	0.931988	None
18963	ECEL1	-0.00042	0.81613	0.931988	None
18964	ACOT11	-0.0159	0.816224	0.932018	None

18965	ZNF215	-0.02528	0.816279	0.932018	None
18966	RBM25	0.028519	0.816312	0.932018	None
18967	WWTR1	0.018205	0.816328	0.932018	None
18968	SCAF11	0.027412	0.816533	0.932203	None
18969	LINC01019	-0.00037	0.816676	0.932316	None
18970	TAS2R50	-0.00173	0.816785	0.932392	None
18971	ADCK4	-0.01369	0.817021	0.932582	None
18972	KRTAP9-8	-0.00033	0.817071	0.932582	None
18973	HNRNPA1	-0.04656	0.817163	0.932582	None
18974	FTH1	0.047327	0.817294	0.932582	None
18975	CRYBB3	-0.01293	0.817308	0.932582	None
18976	KLHL42	-0.03978	0.817315	0.932582	None
18977	RRAGA	-0.02493	0.817318	0.932582	None
18978	TNNI3K	-0.03175	0.817407	0.932582	None
18979	KNTC1	-0.0413	0.81742	0.932582	None
18980	CHCHD2	-0.03388	0.817422	0.932582	None
18981	PRCC	-0.04359	0.817425	0.932582	None
18982	LOC10050(	-0.01262	0.817524	0.932646	None
18983	C1orf220	-0.01202	0.817571	0.932651	None
18984	LRRIQ1	0.002285	0.817843	0.932847	None
18985	PIGX	0.039	0.817872	0.932847	None
18986	LOC10012!	-0.0004	0.817882	0.932847	None
18987	TLR1	0.072502	0.817953	0.932847	None
18988	IAH1	-0.02932	0.817959	0.932847	None
18989	DGCR5	0.000956	0.818044	0.932895	None
18990	RARS2	-0.01188	0.81864	0.933436	None
18991	PNPLA2	0.012145	0.81864	0.933436	None
18992	SAMD14	-0.00491	0.818648	0.933436	None
18993	CD300C	-0.02658	0.818696	0.933442	None
18994	C1QTNF2	-0.0004	0.818808	0.93352	None
18995	CXorf21	-0.04714	0.818875	0.933548	None
18996	RIMBP2	-0.00573	0.819059	0.933671	None
18997	NFXL1	-0.02926	0.81907	0.933671	None
18998	SETD2	0.033701	0.819142	0.933705	None
18999	ENPP1	-0.00032	0.819346	0.93386	None
19000	RIMS4	0.008687	0.819364	0.93386	None
19001	VSTM1	-0.06941	0.819597	0.933867	None
19002	LOC10050(	0.001247	0.819623	0.933867	None
19003	STX1B	-0.00184	0.819675	0.933867	None
19004	PRSS50	-0.00799	0.819677	0.933867	None
19005	CD8B	-0.00078	0.819684	0.933867	None
19006	FAM20C	0.023642	0.819731	0.933867	None
19007	DCD	-0.00232	0.819757	0.933867	None
19008	ADD3-AS1	-0.01218	0.819785	0.933867	None
19009	ALKBH3-A	-0.00039	0.81982	0.933867	None
19010	CRISP3	0.128559	0.819826	0.933867	None
19011	RP5-1118D	0.008286	0.819851	0.933867	None
19012	DAP3	0.017304	0.819888	0.933867	None
19013	TTC9B	0.007578	0.819953	0.933891	None
19014	MOV10	0.035317	0.820273	0.934166	None
19015	C1orf123	0.025953	0.82028	0.934166	None
19016	B4GALNT3	-0.0046	0.820376	0.934195	None
19017	AGRP	-0.00051	0.820391	0.934195	None
19018	AC007365.	-0.00863	0.820481	0.934248	None
19019	KAT8	-0.02531	0.820663	0.93429	None
19020	AC012499.	-0.00574	0.820669	0.93429	None
19021	LOC10192:	-0.00292	0.82068	0.93429	None
19022	MTMR7	-0.00033	0.820838	0.93429	None

19023	IQCE	-0.01566	0.82086	0.93429	None
19024	TRIM14	-0.02671	0.820876	0.93429	None
19025	GDPGP1	0.01557	0.820914	0.93429	None
19026	RP11-38C1	-0.00975	0.820915	0.93429	None
19027	MBTPS1	-0.02903	0.820949	0.93429	None
19028	PLIN3	-0.02811	0.82098	0.93429	None
19029	LOC10014	-0.03135	0.820993	0.93429	None
19030	OSBPL8	0.037665	0.821204	0.934481	None
19031	LOC10192	-0.00061	0.821335	0.934514	None
19032	CDON	0.000285	0.821342	0.934514	None
19033	IRAK3	0.041449	0.821362	0.934514	None
19034	CEP83-AS1	0.005939	0.82152	0.934547	None
19035	MIPOL1	0.002316	0.821542	0.934547	None
19036	HNRNPC	-0.03117	0.821583	0.934547	None
19037	TBCCD1	-0.04218	0.821595	0.934547	None
19038	NAAA	0.031632	0.821661	0.934547	None
19039	DTX2P1-U	-0.01407	0.821719	0.934547	None
19040	UGT1A1	-0.00033	0.821794	0.934547	None
19041	ATP11A-A	-0.00034	0.8218	0.934547	None
19042	LOC33896	-0.00844	0.821802	0.934547	None
19043	FIGLA	-0.00034	0.821823	0.934547	None
19044	MRPL14	0.037763	0.822195	0.934905	None
19045	PPIG	0.036868	0.822274	0.934905	None
19046	AMOTL2	-0.03639	0.822294	0.934905	None
19047	MLIP	-0.00034	0.82236	0.934905	None
19048	TCEB3B	0.010826	0.822379	0.934905	None
19049	RGR	-0.0006	0.822397	0.934905	None
19050	LOC28643	-0.0585	0.822509	0.934983	None
19051	SLCO4A1-	0.004557	0.822737	0.935193	None
19052	CACNA1G	0.004313	0.823023	0.935469	None
19053	CD1C	-0.06856	0.823073	0.935477	None
19054	IGF2BP1	-0.00031	0.823326	0.935645	None
19055	RP11-327J	-0.00038	0.823329	0.935645	None
19056	ANKRD13D	-0.03639	0.82335	0.935645	None
19057	SPATA17	0.00374	0.823408	0.935661	None
19058	LOC10192	-0.00066	0.823602	0.93579	None
19059	LOC10192	0.009034	0.823608	0.93579	None
19060	RRP7A	0.057502	0.823706	0.935853	None
19061	LINC00161	-0.00406	0.823885	0.935966	None
19062	UBE2A	0.02128	0.823927	0.935966	None
19063	LOC10065	0.002778	0.823961	0.935966	None
19064	SMG7-AS1	0.004636	0.823978	0.935966	None
19065	HIST1H3G	-0.02036	0.82407	0.935979	None
19066	FGF1	-0.00035	0.824077	0.935979	None
19067	LINC00587	0.005226	0.824233	0.936104	None
19068	GMEB1	0.0271	0.82429	0.936104	None
19069	RFWD3	-0.05319	0.824316	0.936104	None
19070	FLT4	0.010755	0.824479	0.936196	None
19071	ALG9	0.028677	0.824484	0.936196	None
19072	MKNK1-AS	-0.00032	0.824646	0.936331	None
19073	AC074212.	-0.00499	0.824711	0.936334	None
19074	DPF1	-0.00851	0.824735	0.936334	None
19075	SFT2D1	-0.03885	0.824798	0.936357	None
19076	KDELR3	0.013077	0.82492	0.936437	None
19077	RRAGB	0.039821	0.824955	0.936437	None
19078	LDHAL6B	-0.0003	0.825056	0.936503	None
19079	MLK7-AS1	0.002249	0.82516	0.936571	None
19080	CDH16	-0.00038	0.825274	0.936618	None

19081	CYP4B1	-0.00285	0.825288	0.936618	None
19082	FLJ31356	-0.00613	0.825387	0.936681	None
19083	TSC22D1	0.033735	0.825523	0.936787	None
19084	RP11-108E	-0.00139	0.825593	0.936803	None
19085	NCK1	-0.01906	0.825624	0.936803	None
19086	LOC10050	-0.01466	0.825675	0.936812	None
19087	WFDC5	0.002008	0.825955	0.936917	None
19088	AC139100.	-0.00136	0.825966	0.936917	None
19089	FBXL2	-0.02133	0.825971	0.936917	None
19090	NEK4	0.023572	0.825975	0.936917	None
19091	LOC10050	-0.01646	0.825984	0.936917	None
19092	RBM15B	0.02588	0.82615	0.937046	None
19093	KIF2C	0.05769	0.826184	0.937046	None
19094	TMEM143	0.015248	0.826425	0.937246	None
19095	TMEM59	-0.02603	0.826447	0.937246	None
19096	THBS1	-0.05376	0.826613	0.937345	None
19097	DCT	-0.00115	0.826621	0.937345	None
19098	MAGEC2	-0.00379	0.826667	0.937348	None
19099	ACTR2	0.039664	0.826726	0.937365	None
19100	LOC28307	-0.04753	0.826908	0.937523	None
19101	ALDH3A1	-0.0156	0.826963	0.937536	None
19102	VSIG2	0.010351	0.82702	0.937552	None
19103	IL18RAP	-0.07371	0.827202	0.937709	None
19104	SACM1L	0.033512	0.827319	0.937728	None
19105	RHOF	0.025563	0.827347	0.937728	None
19106	TBC1D29	0.006758	0.827349	0.937728	None
19107	IMMP1L	-0.03524	0.827427	0.937767	None
19108	KCNN2	0.002231	0.827658	0.937981	None
19109	HTR4	0.002987	0.827945	0.938256	None
19110	AC007362.	-0.00035	0.828124	0.938363	None
19111	CNTFR-AS	-0.00351	0.828145	0.938363	None
19112	SLC17A1	-0.00036	0.828169	0.938363	None
19113	ARHGEF26	0.029979	0.828227	0.93838	None
19114	CTD-2287	-0.01538	0.828296	0.938409	None
19115	ZFYVE9	-0.00612	0.828429	0.938511	None
19116	TMEM223	-0.00229	0.828559	0.938597	None
19117	CTSLP8	0.054992	0.828645	0.938597	None
19118	SOHLH1	-0.00031	0.828668	0.938597	None
19119	GNLY	-0.05013	0.828678	0.938597	None
19120	GRHPR	-0.03217	0.828785	0.938655	None
19121	GCNT3	-0.00726	0.82884	0.938655	None
19122	CACNA1G	0.008765	0.82886	0.938655	None
19123	MEIS2	0.048433	0.829004	0.938769	None
19124	EID2	-0.0292	0.829094	0.938822	None
19125	NEU2	0.006752	0.82914	0.938824	None
19126	TRAM1	-0.03116	0.829267	0.9389	None
19127	PURB	-0.00279	0.829313	0.9389	None
19128	CGGBP1	-0.02068	0.829405	0.9389	None
19129	TIPIN	-0.03872	0.829414	0.9389	None
19130	CXorf22	-0.00929	0.829492	0.9389	None
19131	LOC10050	0.001602	0.829507	0.9389	None
19132	DCAF12L2	-0.00415	0.82951	0.9389	None
19133	AX748339	-0.03003	0.829589	0.93894	None
19134	SLC25A18	-0.00031	0.829684	0.938999	None
19135	PDE8A	-0.0201	0.82974	0.939014	None
19136	ATP5B	-0.02267	0.830035	0.939298	None
19137	LINC00895	0.001793	0.830111	0.939319	None
19138	CDKN2AIP	-0.03841	0.83014	0.939319	None

19139	HIST1H2BN	-0.0239	0.830288	0.939437	None
19140	PGP	-0.02973	0.830387	0.939448	None
19141	MYCNOS	-0.00965	0.830447	0.939448	None
19142	ZNF567	0.042264	0.830447	0.939448	None
19143	UQCRQ	-0.03555	0.830471	0.939448	None
19144	IGF2-AS	0.003567	0.830667	0.939602	None
19145	VWA5B2	-0.00268	0.830744	0.939602	None
19146	ISM1-AS1	-0.00032	0.83081	0.939602	None
19147	LOC10050(	-0.00489	0.830826	0.939602	None
19148	PRDM14	0.005665	0.830846	0.939602	None
19149	RBL1	0.01886	0.830876	0.939602	None
19150	GIMAP2	-0.04691	0.83094	0.939602	None
19151	TAS2R39	-0.00032	0.831024	0.939602	None
19152	TMSB15B	-0.01932	0.831027	0.939602	None
19153	LINC01398	-0.00032	0.831042	0.939602	None
19154	CABP1	-0.00401	0.831151	0.939652	None
19155	MCM4	-0.04538	0.831172	0.939652	None
19156	IFNA4	0.00027	0.831222	0.939659	None
19157	LOC28500(	-0.00226	0.831282	0.939678	None
19158	ATP5A1	-0.01	0.831374	0.939733	None
19159	BDNF-AS	-0.00575	0.831444	0.939763	None
19160	C5orf17	-0.01203	0.831603	0.939774	None
19161	LOC25517	0.000921	0.831619	0.939774	None
19162	ZNF705G	-0.00034	0.831622	0.939774	None
19163	C1orf68	-0.00237	0.831678	0.939774	None
19164	CENPA	-0.03332	0.831763	0.939774	None
19165	ST8SIA3	-0.00031	0.831766	0.939774	None
19166	CAPN3	0.032245	0.831773	0.939774	None
19167	LOC10155(	0.011666	0.831825	0.939774	None
19168	TCF7L2	-0.05237	0.831844	0.939774	None
19169	TCF7	0.00558	0.832095	0.93996	None
19170	CCBL1	0.012836	0.83212	0.93996	None
19171	GNG2	-0.01668	0.832193	0.93996	None
19172	AC008746.	-0.02534	0.832209	0.93996	None
19173	LOC10192	-0.00497	0.832226	0.93996	None
19174	LOC10192(	-0.00374	0.832344	0.940045	None
19175	KLRF1	0.085215	0.832439	0.940103	None
19176	PCF11	-0.02299	0.832582	0.940215	None
19177	SSH2	-0.0127	0.832727	0.940294	None
19178	ABCG1	0.05251	0.832751	0.940294	None
19179	GS1-600G8	-0.00153	0.832783	0.940294	None
19180	SUCLA2	-0.0362	0.833079	0.94058	None
19181	RP11-171I	-0.02554	0.833185	0.940609	None
19182	NBEAP1	-0.00079	0.833192	0.940609	None
19183	IGHD	0.034752	0.83324	0.940614	None
19184	EBLN3	-0.03975	0.83333	0.940668	None
19185	ZNF251	0.020162	0.833472	0.940778	None
19186	CNOT1	0.037816	0.833585	0.940857	None
19187	CCM2	-0.02824	0.833703	0.940941	None
19188	LOC10028	-0.02082	0.833863	0.941035	None
19189	RP11-443(	0.007344	0.833886	0.941035	None
19190	CXCL1	0.097688	0.833943	0.941035	None
19191	GFM1	-0.02557	0.834042	0.941035	None
19192	CA1	0.067772	0.834043	0.941035	None
19193	HTR1F	-0.04637	0.834047	0.941035	None
19194	LOC28358	-0.00032	0.834095	0.94104	None
19195	ERN2	0.010269	0.834163	0.941067	None
19196	MIR143HG	-0.00501	0.834224	0.941088	None

19197	NAA50	-0.0269	0.834294	0.941117	None
19198	LINC01333	-0.00116	0.834485	0.941284	None
19199	LRRC31	-0.00356	0.834594	0.941358	None
19200	NOG	-0.08008	0.834711	0.941364	None
19201	CYSLTR2	0.015136	0.834724	0.941364	None
19202	DOK4	-0.00819	0.834767	0.941364	None
19203	TOB1-AS1	-0.01609	0.834879	0.941364	None
19204	GPRIN3	0.024504	0.834945	0.941364	None
19205	KIR2DS5	0.008496	0.834959	0.941364	None
19206	ERICH1-AS	0.002567	0.834978	0.941364	None
19207	TENM3	-0.00033	0.835045	0.941364	None
19208	LOC91450	-0.01136	0.835053	0.941364	None
19209	LINC01213	0.001887	0.835053	0.941364	None
19210	LHFPL1	0.001031	0.835078	0.941364	None
19211	GGT5	-0.01394	0.835138	0.941384	None
19212	AKAP8L	-0.02647	0.835426	0.941659	None
19213	ZNF776	0.033131	0.835486	0.941677	None
19214	LOC10192	-0.00135	0.835556	0.941707	None
19215	FAM187B	-0.0003	0.835814	0.941949	None
19216	LOC10028	-0.00028	0.83594	0.942042	None
19217	FGF22	-0.00028	0.836021	0.942084	None
19218	ORAOV1	0.017924	0.836087	0.942096	None
19219	SCART1	-0.00808	0.836118	0.942096	None
19220	MATN3	-0.00252	0.836233	0.94215	None
19221	KIFC2	0.008142	0.836284	0.94215	None
19222	SPACA3	0.00179	0.836312	0.94215	None
19223	PHKA2	0.021051	0.836382	0.94215	None
19224	MAP6	0.001269	0.836512	0.94215	None
19225	C2CD3	-0.01722	0.836648	0.94215	None
19226	ITGA9	-0.02572	0.83666	0.94215	None
19227	CPOX	-0.04625	0.836679	0.94215	None
19228	ADAMTS9	-0.00093	0.836748	0.94215	None
19229	C15orf54	-0.0303	0.836771	0.94215	None
19230	C6orf201	-0.00338	0.836859	0.94215	None
19231	LOC10192	0.015073	0.836866	0.94215	None
19232	LOC10050	0.05834	0.836874	0.94215	None
19233	RP11-4M2	0.005472	0.836875	0.94215	None
19234	CNN3	-0.0754	0.836899	0.94215	None
19235	TCEAL1	-0.0234	0.836906	0.94215	None
19236	LINC00564	-0.00028	0.83696	0.94215	None
19237	FXYD7	-0.00364	0.837041	0.94215	None
19238	UCN2	-0.00322	0.837056	0.94215	None
19239	RPS23	0.03137	0.837082	0.94215	None
19240	POU6F2	0.003207	0.837107	0.94215	None
19241	PYDC1	-0.00136	0.837123	0.94215	None
19242	MGC4294	-0.0025	0.837299	0.942298	None
19243	ERICH1	-0.03542	0.837462	0.942387	None
19244	TRIM63	-0.01238	0.837544	0.942387	None
19245	PART1	-0.00374	0.83762	0.942387	None
19246	RP11-663N	0.003461	0.837622	0.942387	None
19247	SUCLG2	-0.08788	0.837627	0.942387	None
19248	SLC22A7	-0.00138	0.837639	0.942387	None
19249	MRAP	-0.00068	0.837728	0.942397	None
19250	AX748157	-0.00669	0.837735	0.942397	None
19251	EIF4G3	0.040084	0.837779	0.942398	None
19252	LINC01343	-0.00468	0.837872	0.942452	None
19253	SCT	-0.01318	0.837939	0.942452	None
19254	FMR1	0.044335	0.837958	0.942452	None

19255	LA16c-83F	-0.00135	0.838089	0.942551	None
19256	GRIA3	-0.00031	0.838288	0.942726	None
19257	C1S	-0.02857	0.838637	0.943001	None
19258	LOC10192	-0.00426	0.838688	0.943001	None
19259	TRAPPC8	-0.02776	0.838695	0.943001	None
19260	SND1-IT1	-0.03659	0.838707	0.943001	None
19261	LOC10192	-0.0003	0.838783	0.943037	None
19262	SLC35G3	-0.00068	0.838855	0.94307	None
19263	TBCA	0.020078	0.838904	0.943076	None
19264	POM121L1	-0.00336	0.83896	0.94309	None
19265	TRIM15	-0.00421	0.83901	0.943097	None
19266	KRT24	0.00927	0.839053	0.943097	None
19267	ABCB1	0.066237	0.839223	0.943116	None
19268	ITIH2	0.002516	0.839276	0.943116	None
19269	PITX1	0.000259	0.839278	0.943116	None
19270	PLS3	-0.08302	0.839313	0.943116	None
19271	TCEANC	0.001498	0.839425	0.943116	None
19272	FAM83D	-0.07972	0.839434	0.943116	None
19273	SNRPC	-0.02777	0.839465	0.943116	None
19274	YOD1	0.026143	0.839476	0.943116	None
19275	LOC10192	-0.00277	0.839506	0.943116	None
19276	RIMS2	-0.00339	0.839506	0.943116	None
19277	RP11-1081	-0.00027	0.839621	0.943152	None
19278	DPCD	-0.04603	0.839625	0.943152	None
19279	CNIH3	-0.00607	0.839829	0.943332	None
19280	LY86-AS1	0.00509	0.839873	0.943332	None
19281	DRAXIN	-0.00247	0.839997	0.943378	None
19282	NYX	-0.01079	0.84	0.943378	None
19283	EFNA2	-0.00027	0.840145	0.943458	None
19284	ZSCAN5A	-0.02176	0.840194	0.943458	None
19285	PEG10	0.011167	0.840213	0.943458	None
19286	RP11-932C	0.046176	0.840267	0.943458	None
19287	MMP25	-0.01331	0.84029	0.943458	None
19288	H1FX	0.041882	0.840423	0.943559	None
19289	DDX1	0.023633	0.840551	0.943653	None
19290	FOXC1	-0.0741	0.840621	0.943683	None
19291	BSN-AS2	0.006511	0.840767	0.943759	None
19292	HAPLN4	-0.00032	0.840833	0.943759	None
19293	NGF	-0.01065	0.840852	0.943759	None
19294	LOC10192	0.002346	0.840896	0.943759	None
19295	LOC22181	-0.0078	0.840907	0.943759	None
19296	LAIR2	0.025163	0.841025	0.943843	None
19297	GALC	0.034962	0.841157	0.943932	None
19298	PTN	0.010674	0.841191	0.943932	None
19299	MED9	0.027181	0.841395	0.944112	None
19300	HOXA2	-0.01091	0.841489	0.944113	None
19301	LOC10192	-0.00807	0.841564	0.944113	None
19302	PRR15L	-0.00386	0.841569	0.944113	None
19303	RP11-218I	0.002249	0.841585	0.944113	None
19304	TFAP2C	-0.0003	0.841614	0.944113	None
19305	HLA-DPB1	0.03003	0.841729	0.944193	None
19306	ZNF480	-0.01946	0.841804	0.944228	None
19307	TMEM200C	0.001411	0.841946	0.944331	None
19308	MSRA	0.011398	0.841983	0.944331	None
19309	USH2A	-0.00026	0.842116	0.944382	None
19310	C16orf92	-0.00683	0.842165	0.944382	None
19311	NADK2	0.042742	0.842188	0.944382	None
19312	DMXL2	-0.03541	0.842203	0.944382	None

19313	LOC10013:	-0.02523	0.842454	0.944615	None
19314	BC016361	-0.06582	0.842677	0.944815	None
19315	FAM153A	-0.00415	0.842827	0.944881	None
19316	CALHM2	0.041888	0.842833	0.944881	None
19317	FLJ32154	-0.00031	0.842866	0.944881	None
19318	FNDC9	-0.00576	0.84296	0.944922	None
19319	WHAMMP	0.047396	0.843054	0.944922	None
19320	EDDM3B	-0.00026	0.843064	0.944922	None
19321	PIFO	-0.00561	0.843112	0.944922	None
19322	CST7	0.103665	0.843121	0.944922	None
19323	IL24	-0.00788	0.843236	0.944926	None
19324	FAU	0.017481	0.843244	0.944926	None
19325	IFI27L1	0.036041	0.843278	0.944926	None
19326	SHISA4	-0.01277	0.843299	0.944926	None
19327	MRPL43	-0.04106	0.843447	0.945043	None
19328	CXXC5	-0.02859	0.843633	0.945202	None
19329	C22orf23	0.001656	0.843752	0.945236	None
19330	LINC01346	-0.0003	0.843785	0.945236	None
19331	DMRTA2	0.002949	0.843799	0.945236	None
19332	ARHGEF17	-0.03226	0.843862	0.945236	None
19333	AURKAIP1	0.009203	0.843881	0.945236	None
19334	RGL4	-0.06381	0.844053	0.94538	None
19335	CC2D2A	0.002158	0.844128	0.945415	None
19336	INSL5	0.001653	0.844219	0.945468	None
19337	SCTR	0.001286	0.844267	0.945472	None
19338	LIMCH1	-0.04531	0.844333	0.94549	None
19339	GOLGB1	-0.02361	0.844405	0.94549	None
19340	MTMR12	0.019659	0.844413	0.94549	None
19341	LINC01348	-0.00453	0.844505	0.945543	None
19342	IL34	-0.01628	0.844774	0.945795	None
19343	EPHX3	-0.00979	0.844976	0.945795	None
19344	PGLYRP3	-0.00027	0.845014	0.945795	None
19345	LOC10099(	-0.00031	0.845016	0.945795	None
19346	MNX1	0.004604	0.84511	0.945795	None
19347	LOC10192{	0.007657	0.845132	0.945795	None
19348	DLC1	0.012029	0.845158	0.945795	None
19349	EIF3G	0.026061	0.845206	0.945795	None
19350	PET100	0.032922	0.845239	0.945795	None
19351	PRAC1	-0.01333	0.84525	0.945795	None
19352	NME5	0.015259	0.845263	0.945795	None
19353	PKD1L1	-0.00101	0.845282	0.945795	None
19354	RP11-116(	-0.0003	0.845298	0.945795	None
19355	CTB-167B!	-0.01677	0.845541	0.946019	None
19356	DNAH17-/	-0.0003	0.845624	0.946024	None
19357	LOC64354!	-0.00028	0.845633	0.946024	None
19358	FABP6	-0.00027	0.845739	0.946035	None
19359	OPA1	-0.02074	0.845762	0.946035	None
19360	CDK5RAP1	0.023374	0.845779	0.946035	None
19361	CPSF6	-0.03917	0.845841	0.946035	None
19362	UTF1	0.00311	0.845862	0.946035	None
19363	FAM71A	0.01143	0.846071	0.946181	None
19364	SPTBN5	-0.00241	0.84608	0.946181	None
19365	RLN3	-0.0003	0.846146	0.946207	None
19366	SLCO1A2	-0.00028	0.84627	0.946296	None
19367	TMEM27	-0.01751	0.846511	0.946517	None
19368	LRRC25	0.021415	0.846556	0.946518	None
19369	ECI1	-0.03265	0.846777	0.946685	None
19370	MAG	0.004899	0.846793	0.946685	None

19371	AF213884.1	0.01766	0.846875	0.946729	None
19372	LOC10050	-0.00266	0.846953	0.946767	None
19373	SCGB2A1	-0.0003	0.84707	0.946823	None
19374	MTMR11	-0.01868	0.84709	0.946823	None
19375	PRPS2	0.026293	0.847235	0.946831	None
19376	PCMT1	0.023618	0.847241	0.946831	None
19377	TBR1	-0.00029	0.847255	0.946831	None
19378	RBM22	0.030792	0.847273	0.946831	None
19379	PROX1	-0.00077	0.847507	0.947044	None
19380	PBX1	0.075544	0.847733	0.94719	None
19381	DKK3	-0.00029	0.84781	0.94719	None
19382	EPO	0.006698	0.847845	0.94719	None
19383	EIF4E3	0.051274	0.847853	0.94719	None
19384	MUC5AC	0.001534	0.847937	0.94719	None
19385	GIGYF1	0.014977	0.847956	0.94719	None
19386	RP4-593H1	-0.00744	0.847981	0.94719	None
19387	SLC5A4	-0.00028	0.847988	0.94719	None
19388	DDX3X	0.036744	0.848065	0.947226	None
19389	AP1AR	-0.00976	0.848107	0.947226	None
19390	NUDT10	-0.01335	0.848159	0.947235	None
19391	LINC01222	0.008483	0.848236	0.947272	None
19392	AK091729	0.011397	0.848435	0.947428	None
19393	FFAR2	-0.02288	0.848463	0.947428	None
19394	SPINT2	-0.04029	0.848537	0.947462	None
19395	RBM45	0.013066	0.848624	0.94751	None
19396	SGPP1	0.060068	0.848737	0.947587	None
19397	FLYWCH2	0.028074	0.848836	0.947649	None
19398	LRRC36	0.012177	0.849076	0.947868	None
19399	LOC10192	-0.00368	0.84921	0.947968	None
19400	NEUROD1	-0.00027	0.84926	0.947976	None
19401	ELP6	0.034692	0.849309	0.947982	None
19402	RBM28	-0.02105	0.84936	0.947989	None
19403	PRKCA	-0.00861	0.849449	0.947992	None
19404	PSMD8	0.031817	0.849465	0.947992	None
19405	DYNLT1	-0.02702	0.849494	0.947992	None
19406	QSOX2	0.019769	0.849577	0.948036	None
19407	LOC10192	-0.00202	0.849779	0.948213	None
19408	USP45	-0.03559	0.849839	0.94823	None
19409	DIRAS2	-0.00027	0.849949	0.948277	None
19410	GABPB1-A	-0.06407	0.849968	0.948277	None
19411	ATOH1	-0.00214	0.850269	0.948519	None
19412	AC114730.	-0.002	0.850272	0.948519	None
19413	PIH1D2	0.004745	0.850351	0.948558	None
19414	SOX11	0.000381	0.850447	0.948593	None
19415	SCUBE2	-0.00464	0.850471	0.948593	None
19416	NPPB	-0.00485	0.850556	0.948639	None
19417	KIAA1324	-0.00026	0.85072	0.948774	None
19418	FPGS	0.029081	0.850825	0.948842	None
19419	KANSL2	-0.0275	0.850876	0.94885	None
19420	SNHG19	0.042014	0.851232	0.949175	None
19421	IFNLR1	0.013525	0.851255	0.949175	None
19422	SHMT2	0.033999	0.851353	0.949235	None
19423	HNF1A	0.00327	0.851608	0.949403	None
19424	DPP6	-0.00026	0.851637	0.949403	None
19425	HNF4A-AS	0.005405	0.851676	0.949403	None
19426	PARD6G-A	-0.00215	0.851714	0.949403	None
19427	BC041998	-0.00323	0.851725	0.949403	None
19428	LINC01428	-0.00026	0.851766	0.949403	None

19429	LOC10013(	-0.00369	0.851885	0.949486	None
19430	MYO1G	0.036128	0.852012	0.949547	None
19431	HOXB13	-0.00303	0.852034	0.949547	None
19432	DGCR14	-0.00927	0.852133	0.949547	None
19433	FETUB	0.000526	0.852151	0.949547	None
19434	PTGES3	-0.01233	0.852274	0.949547	None
19435	RAB4B	-0.00872	0.852277	0.949547	None
19436	MAB21L3	-0.00025	0.852316	0.949547	None
19437	SSTR4	-0.00028	0.852317	0.949547	None
19438	VSX1	-0.00039	0.852334	0.949547	None
19439	COL4A4	-0.00025	0.852414	0.949587	None
19440	CYP4Z1	-0.00025	0.852493	0.949626	None
19441	FSTL5	-0.00024	0.852799	0.949885	None
19442	CAB39L	0.034772	0.852813	0.949885	None
19443	RRP15	-0.02461	0.852967	0.950007	None
19444	FXYD3	0.009598	0.853095	0.950047	None
19445	ADH1A	0.010274	0.853102	0.950047	None
19446	ZSCAN12	-0.00605	0.853212	0.950047	None
19447	KIAA1804	0.053956	0.853214	0.950047	None
19448	MMP9	0.08055	0.853222	0.950047	None
19449	FBXL18	0.004264	0.853287	0.950071	None
19450	ATP13A5	0.003859	0.85341	0.950147	None
19451	ZIC2	0.002049	0.853523	0.950147	None
19452	AP001189.	0.028995	0.853573	0.950147	None
19453	HAUS8	0.035912	0.85359	0.950147	None
19454	MUC20	0.014185	0.853596	0.950147	None
19455	ACSL6	0.00074	0.853618	0.950147	None
19456	ICE1	-0.01671	0.853955	0.950422	None
19457	NOS3	-0.00182	0.853976	0.950422	None
19458	EMCN	0.053028	0.854011	0.950422	None
19459	GCC1	-0.00548	0.854041	0.950422	None
19460	VWA3B	-0.00024	0.854089	0.950426	None
19461	STX19	0.003378	0.854338	0.950654	None
19462	FKBP10	0.003954	0.85451	0.950665	None
19463	RAB18	0.019333	0.854532	0.950665	None
19464	PTPN1	-0.00271	0.854537	0.950665	None
19465	PUS10	-0.02624	0.85459	0.950665	None
19466	WDR73	0.022501	0.854605	0.950665	None
19467	FRYL	-0.01928	0.85462	0.950665	None
19468	P2RY2	0.046028	0.854654	0.950665	None
19469	CAMK2B	-0.00045	0.85482	0.950707	None
19470	PROM1	0.069256	0.854844	0.950707	None
19471	GOLIM4	0.046088	0.854858	0.950707	None
19472	GRPR	-0.00982	0.854877	0.950707	None
19473	PIN1	0.025717	0.854912	0.950707	None
19474	CTA-268H	0.006167	0.855121	0.950891	None
19475	RP11-665(	-0.00025	0.855568	0.951339	None
19476	PDGFA	0.034897	0.855682	0.951417	None
19477	CATIP-AS1	0.021392	0.855802	0.95143	None
19478	ZCCHC8	0.016322	0.855825	0.95143	None
19479	RAB3GAP1	-0.01704	0.855826	0.95143	None
19480	POMK	0.004531	0.856	0.951575	None
19481	RP11-179E	-0.00084	0.85613	0.95167	None
19482	SLC25A47	0.003679	0.856195	0.95168	None
19483	GPR4	-0.00784	0.85623	0.95168	None
19484	LOC10192'	-0.00026	0.856359	0.95168	None
19485	CRNN	0.00025	0.856362	0.95168	None
19486	FTX	-0.07229	0.856367	0.95168	None

19487	AKR1C4	-0.00708	0.856402	0.95168	None
19488	ARID2	0.026678	0.856526	0.951769	None
19489	C20orf85	0.000332	0.856637	0.951769	None
19490	TMEM11	-0.02311	0.856731	0.951769	None
19491	ACAT2	0.023532	0.856768	0.951769	None
19492	TAS2R40	-0.00025	0.856801	0.951769	None
19493	KLK12	-0.00636	0.856819	0.951769	None
19494	PCDHGA3	-0.00024	0.856826	0.951769	None
19495	RP11-876N	0.019337	0.856834	0.951769	None
19496	CCDC148-	-0.00024	0.856914	0.951776	None
19497	CKS1B	-0.02941	0.856928	0.951776	None
19498	LOC10050(	0.002383	0.856992	0.951798	None
19499	LYZ	0.081524	0.857087	0.951832	None
19500	SLC9C2	0.001206	0.857212	0.951832	None
19501	CTD-2377I	0.002024	0.857255	0.951832	None
19502	LAMA4	0.000233	0.857341	0.951832	None
19503	ELN	-0.00581	0.857366	0.951832	None
19504	RBP2	-0.01355	0.857369	0.951832	None
19505	MLXIPL	0.012964	0.857405	0.951832	None
19506	BARX1-AS	-0.00213	0.857411	0.951832	None
19507	RAB8B	0.040424	0.857418	0.951832	None
19508	C21orf90	-0.00451	0.857482	0.951854	None
19509	GLO1	-0.01829	0.857584	0.951919	None
19510	LOC28479	-0.00025	0.857685	0.951949	None
19511	LINC00608	0.006335	0.857699	0.951949	None
19512	LPPR4	-0.00024	0.857905	0.952089	None
19513	ULBP2	0.006661	0.858029	0.952089	None
19514	LINC01431	0.000766	0.858053	0.952089	None
19515	KIR3DL1	0.004087	0.858103	0.952089	None
19516	CTD-2520I	-0.00392	0.858116	0.952089	None
19517	POLE2	0.047722	0.858193	0.952089	None
19518	CTBP1-AS	-0.01132	0.858267	0.952089	None
19519	ILF3	0.023291	0.858277	0.952089	None
19520	CCL11	0.004625	0.85829	0.952089	None
19521	TRIM5	-0.01379	0.858393	0.952089	None
19522	LPO	0.001931	0.858432	0.952089	None
19523	EDDM3A	0.001055	0.858436	0.952089	None
19524	SCUBE3	-0.00615	0.858486	0.952089	None
19525	ZNF708	0.031455	0.858506	0.952089	None
19526	PHLPP1	0.019069	0.858601	0.952089	None
19527	LOC10105	-0.00026	0.858605	0.952089	None
19528	STK38L	0.019121	0.858624	0.952089	None
19529	ANKRD11	0.023127	0.858629	0.952089	None
19530	PLXDC1	0.003769	0.85872	0.952089	None
19531	MIR194-2	-0.00259	0.85877	0.952089	None
19532	SMTN	-0.0046	0.858777	0.952089	None
19533	RP11-333I	-0.00056	0.858816	0.952089	None
19534	RANBP3L	-0.00028	0.858836	0.952089	None
19535	FAM229B	-0.02683	0.858941	0.952156	None
19536	C12orf40	0.002747	0.859026	0.952185	None
19537	COX7B2	-0.00069	0.859076	0.952185	None
19538	LOC64624	-0.00028	0.859102	0.952185	None
19539	RPS6KA6	0.002556	0.859143	0.952185	None
19540	GFM2	0.025991	0.859231	0.952199	None
19541	PITHD1	-0.01502	0.859243	0.952199	None
19542	CRYM	-0.05177	0.859327	0.952242	None
19543	NRXN1	-0.00023	0.859448	0.952328	None
19544	RP3-406C	-0.00198	0.859669	0.952524	None

19545	CRX	0.000232	0.859818	0.95264	None
19546	TAAR1	0.000376	0.859992	0.952752	None
19547	SLC26A4	0.003589	0.86004	0.952752	None
19548	GP2	-0.00376	0.860051	0.952752	None
19549	TANC1	-0.01301	0.860135	0.952762	None
19550	LOC10272:	-0.00502	0.860188	0.952762	None
19551	MPP1	-0.02954	0.860192	0.952762	None
19552	RP11-421F	0.001501	0.86027	0.9528	None
19553	SLC6A14	-0.00024	0.860355	0.952845	None
19554	REEP2	-0.00851	0.860455	0.952861	None
19555	CCNB1	-0.04285	0.860457	0.952861	None
19556	MMP10	-0.00236	0.86056	0.952926	None
19557	USP46-AS:	-0.01644	0.860697	0.953	None
19558	PTPRZ1	-0.00026	0.860715	0.953	None
19559	MSH3	-0.01571	0.860817	0.953064	None
19560	LINC00565	-0.00332	0.860966	0.95317	None
19561	ARMC12	0.006247	0.861005	0.95317	None
19562	DCAF13	0.02109	0.861067	0.95317	None
19563	GABRB1	0.004003	0.861088	0.95317	None
19564	LINC00290	-0.00243	0.861149	0.953188	None
19565	KCNQ1DN	-0.00942	0.861288	0.953293	None
19566	STC2	-0.00444	0.861402	0.953324	None
19567	SERPINE1	-0.02744	0.861403	0.953324	None
19568	LOC10192!	0.010109	0.861497	0.953332	None
19569	KCTD5	0.017731	0.861568	0.953332	None
19570	RNF41	0.017824	0.861591	0.953332	None
19571	HTR2B	-0.00144	0.861624	0.953332	None
19572	BGLT3	-0.01196	0.861631	0.953332	None
19573	PSG2	0.000241	0.86172	0.953382	None
19574	ZNF283	0.02704	0.861811	0.953434	None
19575	TARBP1	0.027827	0.861875	0.953456	None
19576	AGR2	-0.01603	0.861963	0.953505	None
19577	C8A	-0.00025	0.862017	0.953515	None
19578	IBSP	-0.01804	0.86211	0.95357	None
19579	IL1F10	-0.00971	0.862154	0.95357	None
19580	ACOXL	-0.00346	0.862368	0.95373	None
19581	TBCB	-0.02312	0.862387	0.95373	None
19582	LOC10028	0.002118	0.862586	0.953832	None
19583	TBC1D7	-0.01551	0.862587	0.953832	None
19584	HOXC5	-0.00494	0.862611	0.953832	None
19585	VPS29	0.011566	0.862755	0.953935	None
19586	GINS1	0.037753	0.862799	0.953935	None
19587	ARID4B	-0.02068	0.862837	0.953935	None
19588	IL10	-0.02776	0.862936	0.953996	None
19589	ADAM12	-0.00023	0.862981	0.953997	None
19590	NKAIN2	-0.04252	0.863037	0.954011	None
19591	ORAI2	-0.03392	0.863145	0.954081	None
19592	TECTB	-0.00025	0.863326	0.954108	None
19593	RP11-457K	-0.00128	0.863357	0.954108	None
19594	SLC35A2	0.00929	0.863389	0.954108	None
19595	RP11-631N	-0.01588	0.863424	0.954108	None
19596	PTPN5	-0.00025	0.863445	0.954108	None
19597	TMPRSS15	0.005139	0.863514	0.954108	None
19598	CCSER1	-0.00531	0.863548	0.954108	None
19599	SLC25A21-	0.026837	0.863563	0.954108	None
19600	HAUS2	0.023298	0.863581	0.954108	None
19601	ENY2	-0.02421	0.863646	0.954108	None
19602	LOC10012!	0.007362	0.863654	0.954108	None

19603	LINC01210	-0.00026	0.863831	0.954255	None
19604	HOTS	-0.00565	0.863924	0.954309	None
19605	ARMCX3	-0.02356	0.86408	0.954419	None
19606	UNC119B	-0.01066	0.864112	0.954419	None
19607	METTL7A	0.021614	0.864207	0.954436	None
19608	HEXA-AS1	-0.00475	0.864216	0.954436	None
19609	OSMR	-0.00877	0.864385	0.954575	None
19610	DNAJC4	0.008351	0.864461	0.954597	None
19611	TRIP13	0.042812	0.864737	0.954597	None
19612	CEP83	-0.02858	0.864748	0.954597	None
19613	CTLA4	0.000437	0.864814	0.954597	None
19614	PTPN13	0.023379	0.864831	0.954597	None
19615	FAM204A	-0.01561	0.86484	0.954597	None
19616	LOC10192	-0.01068	0.864884	0.954597	None
19617	RP11-508N	-0.00381	0.864937	0.954597	None
19618	TSPAN8	-0.00646	0.86495	0.954597	None
19619	SNX31	-0.00189	0.864958	0.954597	None
19620	IL36G	-0.00023	0.864995	0.954597	None
19621	MYB	0.021856	0.865013	0.954597	None
19622	MYEF2	0.045559	0.865071	0.954597	None
19623	LOC10192	-0.00278	0.865117	0.954597	None
19624	SST	0.001397	0.865171	0.954597	None
19625	PRSS8	-0.00025	0.865227	0.954597	None
19626	OCIAD2	-0.03304	0.865237	0.954597	None
19627	CEP152	-0.02313	0.865245	0.954597	None
19628	DUOX2	-0.00408	0.865273	0.954597	None
19629	PTCHD2	0.003129	0.865287	0.954597	None
19630	GAGE1	0.000682	0.865384	0.954634	None
19631	PBX4	0.019394	0.865444	0.954634	None
19632	AGK	-0.02107	0.865488	0.954634	None
19633	CDH11	-0.01209	0.865575	0.954634	None
19634	LOC28366	0.005199	0.865595	0.954634	None
19635	AMT	0.037105	0.865605	0.954634	None
19636	LOC10192	-0.00054	0.865628	0.954634	None
19637	HNRNPD	0.020735	0.865731	0.954698	None
19638	ZNF280C	-0.02547	0.865885	0.954819	None
19639	KCNK15	-0.00025	0.865929	0.954819	None
19640	PIGU	0.033776	0.865974	0.954821	None
19641	SHISA6	-0.00023	0.866064	0.954871	None
19642	GRAMD4	0.016238	0.866216	0.95499	None
19643	SULF1	-0.00314	0.866363	0.955069	None
19644	PCDH11X	0.001778	0.866388	0.955069	None
19645	HOXA11	-0.00035	0.86642	0.955069	None
19646	CBLC	-0.00265	0.866507	0.955116	None
19647	PDE6D	-0.02125	0.866615	0.955186	None
19648	USP32P2	-0.04849	0.866746	0.955278	None
19649	ZNF660	0.030563	0.866786	0.955278	None
19650	LOC10192	-0.00549	0.86689	0.955312	None
19651	KHDC1L	-0.00025	0.866937	0.955312	None
19652	DAPL1	0.0121	0.866949	0.955312	None
19653	PRR34	0.00391	0.867157	0.955459	None
19654	PTPRF	0.003443	0.867171	0.955459	None
19655	LINC00621	0.015818	0.867251	0.955498	None
19656	GPR97	-0.01999	0.867335	0.955504	None
19657	ACSL4	-0.02656	0.867345	0.955504	None
19658	SEC14L3	0.0003	0.867458	0.955581	None
19659	RP11-391M	0.02817	0.867663	0.955701	None
19660	LOC10192	-0.00023	0.867744	0.955701	None

19661	YAP1	0.007418	0.867799	0.955701	None
19662	CADM1	-0.00163	0.86787	0.955701	None
19663	TEX33	-0.00254	0.867944	0.955701	None
19664	TAS2R41	-0.00024	0.867961	0.955701	None
19665	RP11-385F	0.014233	0.868027	0.955701	None
19666	EPHB1	-0.00634	0.868051	0.955701	None
19667	LOC39988	-0.00362	0.868086	0.955701	None
19668	KIF26B	0.006465	0.868158	0.955701	None
19669	ZPBP2	-0.00196	0.868206	0.955701	None
19670	LINC01021	-0.02314	0.868214	0.955701	None
19671	LIG3	-0.02762	0.868236	0.955701	None
19672	B3GALTL	0.021877	0.868266	0.955701	None
19673	CHGA	-0.00384	0.868292	0.955701	None
19674	SUSD2	0.001987	0.868295	0.955701	None
19675	TMEM39A	0.017015	0.868319	0.955701	None
19676	RNF4	-0.01744	0.868362	0.955701	None
19677	KCND1	-0.02002	0.868567	0.955837	None
19678	FAM209B	0.015055	0.86865	0.955837	None
19679	EIF3B	0.026439	0.868712	0.955837	None
19680	HLA-G	-0.02828	0.868716	0.955837	None
19681	UBE2E1	-0.01495	0.868719	0.955837	None
19682	SDC2	0.038352	0.86875	0.955837	None
19683	SLC22A12	0.003886	0.86891	0.95594	None
19684	DSCAML1	0.001076	0.868954	0.95594	None
19685	LOC20218	0.040391	0.868976	0.95594	None
19686	KLHL9	0.010173	0.869104	0.956031	None
19687	LOC10192	-0.00277	0.869147	0.956031	None
19688	ANKRD36E	-0.03714	0.869215	0.956031	None
19689	SPAG4	-0.0257	0.869335	0.956031	None
19690	HS3ST3B1	0.027374	0.869508	0.956031	None
19691	ANKRD34C	-0.00063	0.869509	0.956031	None
19692	IGBP1	0.024313	0.869535	0.956031	None
19693	SHROOM2	0.012439	0.86955	0.956031	None
19694	MTMR14	0.023511	0.869554	0.956031	None
19695	LOC10099	-0.00022	0.86959	0.956031	None
19696	HNF4A	0.000409	0.869596	0.956031	None
19697	LOC10192	0.004262	0.869634	0.956031	None
19698	IREB2	-0.03033	0.869654	0.956031	None
19699	GJC2	0.001501	0.869677	0.956031	None
19700	C1orf101	0.006884	0.869742	0.956054	None
19701	COL9A2	-0.0053	0.869831	0.956103	None
19702	OTX2-AS1	0.00228	0.870111	0.956362	None
19703	LOC10192	-0.0053	0.870405	0.956606	None
19704	SLC25A34	-0.01321	0.870421	0.956606	None
19705	SNX21	-0.00535	0.870643	0.956792	None
19706	LSAMP	-0.00022	0.870679	0.956792	None
19707	TCEA1	0.021469	0.870761	0.956794	None
19708	LAMTOR4	-0.03027	0.870841	0.956794	None
19709	ARHGAP1	-0.00946	0.870845	0.956794	None
19710	ZSCAN10	-0.0003	0.870906	0.956794	None
19711	LOC33987	0.001538	0.870921	0.956794	None
19712	IRAK1BP1	-0.02688	0.870988	0.956794	None
19713	SPATA4	-0.00023	0.871008	0.956794	None
19714	NKX2-5	-0.00371	0.871034	0.956794	None
19715	TXNL1	0.038131	0.871102	0.956821	None
19716	CHST7	-0.02479	0.871156	0.956823	None
19717	DTX4	0.025555	0.871218	0.956823	None
19718	MS4A8	-0.00024	0.871237	0.956823	None

19719	SLC16A2	-0.02968	0.871282	0.956824	None
19720	AF070581	-0.0007	0.871459	0.956937	None
19721	NPAS1	-0.00708	0.87149	0.956937	None
19722	POLR2A	-0.00479	0.871547	0.956937	None
19723	EGFR	-0.00247	0.871562	0.956937	None
19724	NOSTRIN	0.026831	0.871626	0.956959	None
19725	WIPF1	-0.02293	0.871671	0.95696	None
19726	FBXO27	0.003324	0.871871	0.957131	None
19727	C5orf64	-0.00616	0.872091	0.957265	None
19728	TTYH2	0.015192	0.87219	0.957265	None
19729	EIF2S1	-0.02211	0.872197	0.957265	None
19730	HGF	0.050535	0.872244	0.957265	None
19731	HHIPL2	-0.0024	0.872259	0.957265	None
19732	TLR9	0.00788	0.872296	0.957265	None
19733	AGBL2	-0.01655	0.872342	0.957265	None
19734	HCAR1	-0.00023	0.872389	0.957265	None
19735	KIAA1407	0.027399	0.872429	0.957265	None
19736	RP11-38P2	-0.03346	0.872435	0.957265	None
19737	TPRKB	0.021426	0.872557	0.957272	None
19738	BPHL	-0.0207	0.872557	0.957272	None
19739	AIPL1	-0.00188	0.872574	0.957272	None
19740	PTCH2	-0.00023	0.872623	0.957277	None
19741	CPSF3	-0.01637	0.872806	0.957332	None
19742	LINC01181	-0.01946	0.872824	0.957332	None
19743	LOC10192	-0.00361	0.872828	0.957332	None
19744	MED13	0.020432	0.872918	0.957332	None
19745	SCP2	-0.01734	0.872972	0.957332	None
19746	IGFL1	-0.00023	0.873009	0.957332	None
19747	PRO1082	-0.01611	0.873041	0.957332	None
19748	RBM23	-0.0177	0.873045	0.957332	None
19749	ZNF76	0.016878	0.873071	0.957332	None
19750	LOC10050	-0.00588	0.873141	0.957361	None
19751	SLC13A3	0.000214	0.873205	0.957368	None
19752	LOC10192	-0.00497	0.873236	0.957368	None
19753	RCC1	-0.02868	0.873419	0.957469	None
19754	RAP1B	0.015085	0.873431	0.957469	None
19755	TRIM72	0.007069	0.873495	0.957469	None
19756	CRYGA	-0.00022	0.873515	0.957469	None
19757	C3orf67	-0.01272	0.873549	0.957469	None
19758	TTLL6	-0.00086	0.873737	0.957578	None
19759	RSRC2	-0.01788	0.873737	0.957578	None
19760	LOC10050	0.003405	0.873821	0.957581	None
19761	LEPR	0.007635	0.873829	0.957581	None
19762	SPEF1	0.009098	0.874048	0.957773	None
19763	STAM	0.023009	0.874188	0.957795	None
19764	KCNJ2-AS	0.002629	0.874189	0.957795	None
19765	NDUFA12	0.018137	0.874212	0.957795	None
19766	TSR2	0.024088	0.874245	0.957795	None
19767	SLC7A11-A	0.000201	0.874499	0.958024	None
19768	LINC00641	-0.00307	0.874621	0.958066	None
19769	STMN4	0.00312	0.874625	0.958066	None
19770	CHL1-AS1	-0.00116	0.874718	0.958094	None
19771	UBXN2A	0.014622	0.874757	0.958094	None
19772	DGKA	0.019361	0.874783	0.958094	None
19773	C2CD2L	0.007219	0.874951	0.958229	None
19774	LOC10192	-0.02256	0.87522	0.958475	None
19775	CUL2	-0.01419	0.875306	0.958492	None
19776	FAT4	0.030031	0.875338	0.958492	None

19777	NKAIN4	0.000733	0.875394	0.958492	None
19778	CCKBR	0.004288	0.875413	0.958492	None
19779	CEP41	-0.01098	0.875468	0.958504	None
19780	LINC00917	0.00094	0.875576	0.958574	None
19781	ATP2B2	-0.00021	0.875667	0.958606	None
19782	CALML5	-0.00644	0.875769	0.958606	None
19783	MPHOSPH	-0.022	0.875809	0.958606	None
19784	GIF	0.003603	0.875823	0.958606	None
19785	CXorf23	0.023918	0.875868	0.958606	None
19786	PAQR6	-0.02046	0.875871	0.958606	None
19787	LOC10050	0.003785	0.87601	0.95871	None
19788	SLC15A1	-0.0026	0.876085	0.958744	None
19789	CYP27B1	0.022367	0.876146	0.958762	None
19790	CYP2F1	-0.00022	0.876312	0.958879	None
19791	DNMT1	0.019403	0.876343	0.958879	None
19792	ELP5	0.034083	0.876396	0.958879	None
19793	RP11-394I	0.005156	0.87643	0.958879	None
19794	EHF	-0.00021	0.876574	0.958926	None
19795	T-18	0.001685	0.876644	0.958926	None
19796	DRD4	-0.00021	0.87665	0.958926	None
19797	CAND1.11	0.002654	0.876671	0.958926	None
19798	BCORP1	0.003608	0.876695	0.958926	None
19799	ZNF629	-0.02656	0.87689	0.959092	None
19800	NF1	-0.01076	0.877028	0.959194	None
19801	MYOZ3	0.001652	0.877126	0.959208	None
19802	LMCD1	-0.00346	0.87713	0.959208	None
19803	LOC10192	0.020864	0.877262	0.959305	None
19804	PP2672	-0.00022	0.877362	0.95936	None
19805	RP11-1E4.	-0.0002	0.87745	0.95936	None
19806	THOC5	-0.01154	0.877488	0.95936	None
19807	FAM47A	-0.0002	0.87749	0.95936	None
19808	SIGLEC8	0.001462	0.877711	0.959491	None
19809	FAM126B	0.023337	0.877715	0.959491	None
19810	RP11-687F	-0.00921	0.877774	0.959491	None
19811	PIN4	0.026667	0.877786	0.959491	None
19812	BTG4	0.000509	0.878044	0.959612	None
19813	C19orf10	0.02979	0.878062	0.959612	None
19814	AZI2	0.019378	0.878098	0.959612	None
19815	EPB41L3	-0.07747	0.878111	0.959612	None
19816	YLPM1	0.029418	0.878155	0.959612	None
19817	DNAJA4	-0.03817	0.878163	0.959612	None
19818	PLA2G16	-0.02672	0.878314	0.959661	None
19819	LINC01419	-0.0002	0.878332	0.959661	None
19820	LOC28605	-0.00384	0.878341	0.959661	None
19821	LOC10192	-0.00022	0.878621	0.959919	None
19822	HRH2	0.0045	0.878714	0.959971	None
19823	COG1	0.005937	0.878807	0.960025	None
19824	COX4I1	0.022003	0.878872	0.960047	None
19825	KCTD17	-0.00468	0.878951	0.960085	None
19826	SYNGR1	0.026121	0.879328	0.96037	None
19827	ZNF81	0.002541	0.879342	0.96037	None
19828	RFC5	0.025338	0.879345	0.96037	None
19829	DUSP11	0.015636	0.879457	0.960428	None
19830	CCL21	-0.00275	0.879491	0.960428	None
19831	PCDHB17	-0.00334	0.879587	0.960428	None
19832	FGF10-AS	-0.0003	0.879606	0.960428	None
19833	TP53INP1	0.008733	0.879637	0.960428	None
19834	OLFML3	0.025747	0.879665	0.960428	None

19835	SS18L2	-0.0138	0.879743	0.960466	None
19836	FNDC4	-0.00587	0.879839	0.960522	None
19837	CLEC4D	-0.02961	0.879981	0.960613	None
19838	AX746627	-0.00428	0.880011	0.960613	None
19839	ARHGAP23	0.009425	0.880093	0.960653	None
19840	CATR1	-0.0002	0.880143	0.96066	None
19841	TBX5-AS1	-0.0002	0.880212	0.960687	None
19842	FREM1	-0.06489	0.880369	0.96081	None
19843	TRAF3IP1	-0.01147	0.880414	0.960811	None
19844	TYRP1	-0.00021	0.880458	0.960811	None
19845	GRM5	0.004761	0.880525	0.960835	None
19846	ZNF229	-0.01081	0.880598	0.960867	None
19847	DSCAM-AS	0.005353	0.880704	0.960928	None
19848	RP11-440L	0.005509	0.880801	0.960928	None
19849	UTP14A	-0.01261	0.880862	0.960928	None
19850	TSSK4	0.004391	0.880885	0.960928	None
19851	PRICKLE3	0.003459	0.880926	0.960928	None
19852	UTRN	-0.01713	0.880981	0.960928	None
19853	CD27	-0.02556	0.881017	0.960928	None
19854	ITFG1	-0.02105	0.881018	0.960928	None
19855	C6orf62	-0.03261	0.881054	0.960928	None
19856	PRRT2	0.005426	0.881185	0.960976	None
19857	ZMYND8	-0.02442	0.881187	0.960976	None
19858	LMNA	0.032707	0.881242	0.960987	None
19859	ACAD10	0.007478	0.881368	0.961077	None
19860	FAM3D	0.007325	0.881476	0.961114	None
19861	KIAA1430	-0.01002	0.881498	0.961114	None
19862	KIAA1244	-0.00103	0.881617	0.961114	None
19863	APCS	-0.00541	0.88165	0.961114	None
19864	ZZEF1	0.019928	0.881657	0.961114	None
19865	BECN1	-0.01682	0.881669	0.961114	None
19866	LRRC15	0.004301	0.881804	0.961214	None
19867	IGSF5	-0.00021	0.88185	0.961215	None
19868	SAA3P	0.00635	0.882019	0.961351	None
19869	FLJ37453	-0.00577	0.882599	0.961934	None
19870	EVX1	0.005844	0.882687	0.961982	None
19871	RELN	0.010252	0.882863	0.962078	None
19872	RGMB-AS1	-0.00052	0.88294	0.962078	None
19873	C6orf1	-0.01353	0.882977	0.962078	None
19874	SDF2	-0.01913	0.88298	0.962078	None
19875	NSUN3	0.014608	0.882997	0.962078	None
19876	SLC27A5	-0.01684	0.883165	0.962213	None
19877	MIF	0.036963	0.883252	0.962216	None
19878	KRT80	0.002866	0.88331	0.962216	None
19879	TMBIM6	0.01545	0.88343	0.962216	None
19880	ETFDH	0.018657	0.883499	0.962216	None
19881	TSEN54	0.01419	0.883561	0.962216	None
19882	ATP5C1	0.011691	0.883564	0.962216	None
19883	IGSF9	0.008471	0.883597	0.962216	None
19884	TCP10L	-0.00671	0.883632	0.962216	None
19885	HMGB4	-0.00607	0.883656	0.962216	None
19886	LINC00491	-0.00019	0.883664	0.962216	None
19887	OPRK1	-0.00089	0.883761	0.962216	None
19888	GSTA3	-0.00347	0.883808	0.962216	None
19889	LOC28623	-0.00362	0.88385	0.962216	None
19890	CALU	-0.02757	0.883874	0.962216	None
19891	NR3C2	0.019245	0.883888	0.962216	None
19892	UBA52	-0.01429	0.883949	0.962216	None

19893	NRG1	-0.00552	0.883963	0.962216	None
19894	INTS12	-0.01589	0.883968	0.962216	None
19895	GLTSCR2	0.013474	0.884069	0.962255	None
19896	LOC10065:	-0.00021	0.884138	0.962255	None
19897	IFNA10	0.001055	0.884179	0.962255	None
19898	LOC34010	-0.00467	0.884182	0.962255	None
19899	LOC43993	-0.01227	0.884411	0.962456	None
19900	RASEF	0.006136	0.884486	0.962489	None
19901	LOC28408	-0.00062	0.884599	0.962493	None
19902	C12orf77	-0.0002	0.884623	0.962493	None
19903	AKR1C1	0.013378	0.884671	0.962493	None
19904	HAL	-0.04727	0.884694	0.962493	None
19905	CCDC172	-0.00512	0.884711	0.962493	None
19906	MED10	-0.0181	0.884788	0.962529	None
19907	LYPD6	-0.00019	0.885012	0.962723	None
19908	KDM2B	0.015656	0.885196	0.962836	None
19909	C12orf50	0.000182	0.885265	0.962836	None
19910	PRMT5	0.01995	0.88527	0.962836	None
19911	RP11-343H	-0.00797	0.885293	0.962836	None
19912	BC045789	-0.01706	0.8854	0.962882	None
19913	FDFT1	0.023216	0.885424	0.962882	None
19914	PRIM1	0.025018	0.885494	0.962909	None
19915	RP11-285A	0.003896	0.885604	0.962917	None
19916	CASP12	0.002085	0.885607	0.962917	None
19917	INSIG2	0.020246	0.885635	0.962917	None
19918	IKZF2	0.004891	0.88569	0.962929	None
19919	LOC10013	-0.02393	0.885761	0.962958	None
19920	DPPA4	0.049502	0.885887	0.963047	None
19921	LINC00654	-0.0002	0.885964	0.963076	None
19922	NPR2	0.011219	0.886003	0.963076	None
19923	ZRANB2-A	-0.00291	0.886089	0.96312	None
19924	AC064852.	-0.0002	0.886277	0.963173	None
19925	ETFB	-0.02157	0.886454	0.963173	None
19926	BC048141	-0.00094	0.886517	0.963173	None
19927	LOC10012	-0.00019	0.886592	0.963173	None
19928	LOC10099	-0.00019	0.886613	0.963173	None
19929	SCAF8	-0.02911	0.88662	0.963173	None
19930	PLSCR2	0.002029	0.88662	0.963173	None
19931	KRT7	0.000264	0.886687	0.963173	None
19932	DNAH1	-0.00019	0.886747	0.963173	None
19933	ARF4	0.016213	0.886763	0.963173	None
19934	DLL4	0.012561	0.886788	0.963173	None
19935	MRPS27	-0.00887	0.886789	0.963173	None
19936	TOMM34	0.017542	0.886799	0.963173	None
19937	GLP2R	-0.00019	0.886802	0.963173	None
19938	CTB-31O2	-0.02333	0.886838	0.963173	None
19939	ZFP1	-0.00182	0.886866	0.963173	None
19940	CCDC176	0.017509	0.886893	0.963173	None
19941	PRKCB	-0.02168	0.887197	0.963417	None
19942	SIX6	-0.00019	0.887207	0.963417	None
19943	DNASE1	0.020482	0.887454	0.963541	None
19944	KIAA1524	0.026952	0.887519	0.963541	None
19945	YBX3	0.015691	0.887538	0.963541	None
19946	PRSS23	0.014955	0.887624	0.963541	None
19947	PGLYRP4	0.00465	0.887626	0.963541	None
19948	CIR1	0.018434	0.887635	0.963541	None
19949	LOC10106	-0.02143	0.887695	0.963541	None
19950	LINC00593	-0.00052	0.887698	0.963541	None

19951	TSPAN2	0.055022	0.887722	0.963541	None
19952	TNN	-0.00787	0.887925	0.963682	None
19953	SNIP1	0.014703	0.88794	0.963682	None
19954	LOC40332:	0.020992	0.888083	0.963777	None
19955	LRRC10B	-0.00474	0.888117	0.963777	None
19956	LOC101928	-0.00393	0.888237	0.963843	None
19957	PLXNA1	0.029321	0.888267	0.963843	None
19958	HIST1H4G	0.002531	0.888386	0.963924	None
19959	THAP6	0.019011	0.888504	0.964002	None
19960	DNAH5	0.01078	0.88856	0.964002	None
19961	TTC25	0.024139	0.888592	0.964002	None
19962	TSSK2	-0.00146	0.888743	0.96409	None
19963	SFXN1	-0.02443	0.888803	0.96409	None
19964	NOSIP	-0.01845	0.888806	0.96409	None
19965	C8orf49	-0.00182	0.888956	0.964205	None
19966	SLC9A9	-0.03233	0.889007	0.964211	None
19967	PTRH2	0.020289	0.889072	0.964233	None
19968	ZNF621	0.023095	0.88915	0.964264	None
19969	CDKL2	0.002877	0.889189	0.964264	None
19970	HMGCR	0.020395	0.889307	0.964344	None
19971	UCP3	-0.00719	0.889358	0.96435	None
19972	SCN11A	-0.00344	0.889531	0.964444	None
19973	KRT27	-0.00018	0.889533	0.964444	None
19974	RP11-55K1	0.001755	0.889668	0.964542	None
19975	MDK	-0.0404	0.889741	0.964563	None
19976	GPATCH2	0.023344	0.889777	0.964563	None
19977	ILDR1	-0.00441	0.88989	0.964563	None
19978	DMAP1	-0.01565	0.889896	0.964563	None
19979	CLIC1	0.014627	0.889946	0.964563	None
19980	TMEM110	-0.01311	0.890028	0.964563	None
19981	MAGEA4	-0.00018	0.890139	0.964563	None
19982	LOC10050(	-0.00019	0.890143	0.964563	None
19983	NKX2-1-A	-0.00097	0.890147	0.964563	None
19984	SLC26A4-/	-0.00078	0.890155	0.964563	None
19985	LINC00298	-0.00019	0.890177	0.964563	None
19986	CHD1L	0.019258	0.890488	0.964851	None
19987	NHLH2	-0.00221	0.890684	0.96499	None
19988	PSMA3	-0.02713	0.890705	0.96499	None
19989	FLJ36840	-0.00018	0.890829	0.965076	None
19990	PXMP2	-0.02451	0.890897	0.965084	None
19991	WDR36	0.016092	0.890926	0.965084	None
19992	RP11-112L	0.001934	0.891006	0.965112	None
19993	CELP	0.001454	0.89104	0.965112	None
19994	RP4-740C4	0.025669	0.891212	0.965232	None
19995	USP2	0.004244	0.89124	0.965232	None
19996	C17orf64	0.001804	0.891371	0.965325	None
19997	SPRY2	0.04153	0.891422	0.965332	None
19998	LINC00963	-0.01615	0.89147	0.965336	None
19999	DNAH9	-0.00018	0.89165	0.965482	None
20000	LOC10050!	-0.00443	0.891936	0.965707	None
20001	RASGRF1	-0.0003	0.892005	0.965707	None
20002	LOC10099(	-0.00018	0.892024	0.965707	None
20003	LOC14567!	-0.00234	0.892036	0.965707	None
20004	CNTNAP4	-0.00043	0.892113	0.965724	None
20005	ZNF443	-0.00285	0.892141	0.965724	None
20006	RP11-286E	0.000468	0.892339	0.96586	None
20007	TMEM130	-0.00018	0.892381	0.96586	None
20008	RP11-465E	-0.00188	0.8924	0.96586	None

20009	SCP2D1	-0.00018	0.892465	0.965882	None
20010	TRMT6	-0.01531	0.892798	0.966194	None
20011	SLAMF1	-0.00093	0.892895	0.966211	None
20012	LOC38869:	-0.01544	0.892903	0.966211	None
20013	CCK	-0.00051	0.893095	0.966371	None
20014	NDRG1	-0.027	0.893178	0.966412	None
20015	LOC10050(	-0.01385	0.893344	0.96648	None
20016	SYT14	-0.00018	0.893406	0.96648	None
20017	GOLGA7B	0.00925	0.893443	0.96648	None
20018	ADAMTS1!	0.001118	0.893478	0.96648	None
20019	RP11-16N:	-0.01472	0.893487	0.96648	None
20020	NR2C2	0.01646	0.893508	0.96648	None
20021	RTCB	0.016188	0.893566	0.966494	None
20022	NUMBL	-0.0089	0.893681	0.96657	None
20023	CELSR3-AS	0.001181	0.89379	0.966573	None
20024	LOC38888:	-0.00294	0.89383	0.966573	None
20025	NSD1	-0.01201	0.893856	0.966573	None
20026	MTCH2	-0.01647	0.893863	0.966573	None
20027	SAMSN1	0.03235	0.894116	0.96676	None
20028	LCN2	-0.0629	0.894134	0.96676	None
20029	TMEM217	0.026005	0.894179	0.96676	None
20030	KIAA0368	-0.015	0.89424	0.96676	None
20031	VAMP3	0.019588	0.894261	0.96676	None
20032	AC006129.	0.00463	0.894303	0.96676	None
20033	CASC4	-0.03201	0.894623	0.967058	None
20034	CTD-2256I	0.006877	0.894677	0.967067	None
20035	C6orf211	0.030448	0.894809	0.967162	None
20036	MBL2	-0.00017	0.894901	0.967196	None
20037	RP1-118J2	0.01006	0.894993	0.967196	None
20038	BCO2	0.00754	0.895055	0.967196	None
20039	NTMT1	-0.02659	0.895078	0.967196	None
20040	TXLNB	0.022558	0.895082	0.967196	None
20041	SPZ1	-0.0002	0.895109	0.967196	None
20042	LOC10192:	-0.00018	0.895271	0.967276	None
20043	C5orf46	-0.00851	0.895315	0.967276	None
20044	LOC64371:	-0.00018	0.895319	0.967276	None
20045	LOC10192!	-0.01082	0.895391	0.967276	None
20046	BDKRB1	0.002962	0.895406	0.967276	None
20047	KIAA1958	-0.02256	0.895634	0.967474	None
20048	LOC10050!	0.019998	0.895682	0.967477	None
20049	SOX15	-0.01038	0.895843	0.967603	None
20050	FLJ35700	-0.00134	0.895915	0.967633	None
20051	UBA6	0.033391	0.896157	0.967846	None
20052	ABCB11	0.002819	0.896357	0.967917	None
20053	RP11-285F	-0.02375	0.896393	0.967917	None
20054	DLK2	0.005987	0.896395	0.967917	None
20055	ADPGK	0.018277	0.896402	0.967917	None
20056	MAGEE2	-0.00441	0.896541	0.967996	None
20057	ERVH-3	-0.02019	0.896586	0.967996	None
20058	LOC64277(	0.001938	0.896642	0.967996	None
20059	SGK2	0.002572	0.896653	0.967996	None
20060	BC045559	-0.0032	0.896906	0.96819	None
20061	TPCN2	0.015524	0.896922	0.96819	None
20062	PHF20L1	-0.0178	0.897084	0.968298	None
20063	STYK1	0.03889	0.897112	0.968298	None
20064	AIFM1	-0.02273	0.897208	0.968341	None
20065	RP11-53A:	-0.00017	0.897339	0.968341	None
20066	VGLL1	-0.00017	0.897393	0.968341	None

20067	KIR3DX1	0.004668	0.897409	0.968341	None
20068	ELL2	-0.03619	0.897422	0.968341	None
20069	GTSE1	-0.03329	0.897464	0.968341	None
20070	DEFB118	0.00577	0.897534	0.968341	None
20071	RP5-855D2	-0.00286	0.897553	0.968341	None
20072	DOCK1	-0.01027	0.897554	0.968341	None
20073	ANXA5	0.044224	0.897733	0.968474	None
20074	RP11-536C	-0.00761	0.897767	0.968474	None
20075	ZNF423	-0.02456	0.89782	0.968483	None
20076	TTTY11	-0.00307	0.897927	0.968507	None
20077	LOC10050!	0.000576	0.897996	0.968507	None
20078	CLPTM1L	0.014438	0.898001	0.968507	None
20079	SMIM5	0.045442	0.898057	0.968507	None
20080	DMBX1	0.000953	0.898066	0.968507	None
20081	SPACA4	-0.00251	0.898197	0.968577	None
20082	STAP2	-0.0059	0.89822	0.968577	None
20083	LOC10050	-0.00017	0.898368	0.968688	None
20084	NRIP3	-0.03006	0.89855	0.968836	None
20085	LOC10192	-0.00017	0.89874	0.968954	None
20086	SCARB1	0.021467	0.898749	0.968954	None
20087	TMEM174	-0.00291	0.898868	0.969034	None
20088	SOCS5	0.023214	0.899021	0.969151	None
20089	VAV2	0.009166	0.89917	0.969229	None
20090	LOC73010	0.032731	0.899207	0.969229	None
20091	SLC2A2	-0.00017	0.899228	0.969229	None
20092	AF007147	-0.02746	0.899357	0.96929	None
20093	KIR2DL4	-0.00017	0.899374	0.96929	None
20094	LOC25380!	-0.00245	0.899435	0.969295	None
20095	FLT3LG	-0.00736	0.899498	0.969295	None
20096	LOC10192	-0.02323	0.899513	0.969295	None
20097	LINC00892	-0.00016	0.899696	0.969404	None
20098	LOC54147	0.001283	0.899703	0.969404	None
20099	IL12RB1	0.006752	0.899868	0.969533	None
20100	GRID1	-0.00017	0.900111	0.969704	None
20101	GSTCD	-0.01783	0.900158	0.969704	None
20102	OCLM	-0.00233	0.900161	0.969704	None
20103	SERPINB4	0.000177	0.900268	0.969756	None
20104	RP11-6F2.!	0.001263	0.900301	0.969756	None
20105	SMURF1	-0.00377	0.900343	0.969756	None
20106	CORO1A	-0.0299	0.900467	0.96984	None
20107	DFNA5	-0.03326	0.90063	0.969968	None
20108	RP11-678C	-0.00226	0.900771	0.970072	None
20109	EFR3B	0.00466	0.900974	0.970212	None
20110	PDE6B	0.008632	0.900994	0.970212	None
20111	LOC14651	0.003635	0.901148	0.970212	None
20112	LINC00937	0.006514	0.901207	0.970212	None
20113	ZFP3	-0.02861	0.901268	0.970212	None
20114	DTHD1	0.000231	0.901298	0.970212	None
20115	EIF1	-0.01378	0.901481	0.970212	None
20116	RAB3GAP2	-0.00619	0.901503	0.970212	None
20117	LOC40191	0.009688	0.901518	0.970212	None
20118	PGAM1	-0.01413	0.90156	0.970212	None
20119	DLEU2L	-0.00478	0.901608	0.970212	None
20120	SLC6A16	0.01447	0.901613	0.970212	None
20121	ELOVL5	-0.02079	0.901616	0.970212	None
20122	SGMS1	-0.03469	0.901651	0.970212	None
20123	ACTG2	0.005155	0.901662	0.970212	None
20124	WISP2	0.003473	0.901704	0.970212	None

20125	RNF180	-0.00392	0.901707	0.970212	None
20126	NDUFA13	0.023173	0.901708	0.970212	None
20127	SLC25A31	-0.0002	0.901806	0.970269	None
20128	PGLYRP1	-0.03163	0.90189	0.970295	None
20129	PRMT8	-0.00261	0.90192	0.970295	None
20130	SLC6A12	-0.00017	0.902162	0.970507	None
20131	XBP1	-0.01523	0.902275	0.970581	None
20132	PDE4A	-0.00469	0.902321	0.970582	None
20133	KNCN	0.002563	0.902485	0.970672	None
20134	CRYBB2	-0.00408	0.902494	0.970672	None
20135	CDRT15	-0.0067	0.902609	0.970748	None
20136	CSHL1	0.000746	0.90266	0.970754	None
20137	AKAP8	-0.0137	0.902745	0.970787	None
20138	ODF3B	0.012027	0.902801	0.970787	None
20139	ACADL	-0.00423	0.902825	0.970787	None
20140	RP11-255(	0.003384	0.90288	0.970798	None
20141	PTPN4	0.011515	0.903004	0.970883	None
20142	BTN2A1	-0.01636	0.903066	0.970902	None
20143	ABCD2	-0.00016	0.903222	0.971021	None
20144	HOXA13	-0.00016	0.903293	0.97103	None
20145	OR6B1	-0.00017	0.90332	0.97103	None
20146	PWP2	-0.00277	0.903527	0.971204	None
20147	ANGPTL7	-0.00127	0.903605	0.97124	None
20148	KCNMB4	0.026399	0.903657	0.971248	None
20149	OR1F1	0.002141	0.903703	0.971249	None
20150	FBXW7	0.019061	0.903774	0.971264	None
20151	CILP	0.007619	0.903829	0.971264	None
20152	GML	-0.00016	0.904127	0.971264	None
20153	SLC7A9	0.006874	0.904134	0.971264	None
20154	RP11-560/	0.001856	0.904135	0.971264	None
20155	ARHGAP24	0.009457	0.904167	0.971264	None
20156	LINC00837	-0.00016	0.904236	0.971264	None
20157	ANKRD30E	0.001501	0.904286	0.971264	None
20158	TRMT112	0.015905	0.904291	0.971264	None
20159	LOC10193(	0.003716	0.904345	0.971264	None
20160	RAD9B	0.008764	0.904373	0.971264	None
20161	CPXCR1	0.002054	0.904397	0.971264	None
20162	HAX1	-0.01087	0.904414	0.971264	None
20163	LINC00315	-0.00044	0.904452	0.971264	None
20164	HRAS	0.022298	0.904464	0.971264	None
20165	NFKBIE	0.029353	0.904499	0.971264	None
20166	RP11-1217	-0.00016	0.904502	0.971264	None
20167	C15orf59	0.004259	0.904524	0.971264	None
20168	KCNA1	-0.00066	0.904626	0.971325	None
20169	CEP68	-0.02047	0.904705	0.971361	None
20170	DNAJC13	-0.02222	0.904886	0.971459	None
20171	LOC28368:	-0.00303	0.904886	0.971459	None
20172	DPP4	-0.00897	0.905027	0.971562	None
20173	RP11-475/	-0.00025	0.905305	0.971783	None
20174	ITIH3	0.001398	0.905322	0.971783	None
20175	AC005224.	0.00386	0.90542	0.97184	None
20176	ADAMTS1(	-0.00016	0.905579	0.971963	None
20177	CLSTN2-A:	-0.00015	0.905643	0.971983	None
20178	MIR646HG	0.033553	0.905759	0.972059	None
20179	HNRNPR	0.021643	0.90602	0.972291	None
20180	PANK1	-0.03274	0.906172	0.972406	None
20181	AX747444	0.001764	0.90627	0.972463	None
20182	LMO7	-0.00236	0.90641	0.972565	None

20183	MAP1LC3E	-0.02254	0.906483	0.972595	None
20184	THRSP	0.001736	0.906705	0.97276	None
20185	C11orf97	-0.00016	0.906766	0.97276	None
20186	EFR3A	0.019065	0.906771	0.97276	None
20187	LOC10013:	-0.00646	0.906974	0.972885	None
20188	CCDC67	-0.00015	0.906978	0.972885	None
20189	FH	-0.01571	0.907024	0.972886	None
20190	CCDC83	-0.00016	0.907069	0.972886	None
20191	PINX1	-0.0175	0.907184	0.972962	None
20192	CLRN1	-0.00015	0.907267	0.973003	None
20193	MICU3	-0.03201	0.907446	0.973003	None
20194	KIF17	0.009832	0.907465	0.973003	None
20195	SLC26A9	0.004048	0.907472	0.973003	None
20196	BAZ2B	0.019483	0.907472	0.973003	None
20197	FDCSP	-0.00063	0.907492	0.973003	None
20198	CTD-2541I	-0.01882	0.907639	0.973096	None
20199	LOC34009(	-0.00026	0.907668	0.973096	None
20200	POM121L1	0.002045	0.907976	0.973377	None
20201	FAM179B	0.019587	0.908158	0.973524	None
20202	TMPRSS11	-0.00015	0.908288	0.97358	None
20203	ANO4	-0.00015	0.9083	0.97358	None
20204	DBIL5P2	0.007023	0.908408	0.973587	None
20205	ZNF45	0.011307	0.908425	0.973587	None
20206	LOC10192ℓ	-0.00015	0.908482	0.973587	None
20207	STMN2	-0.00321	0.908486	0.973587	None
20208	GPAT2	-0.067	0.908629	0.973593	None
20209	GPR158	-0.00058	0.908658	0.973593	None
20210	STXBP4	0.007255	0.908667	0.973593	None
20211	POGK	-0.02308	0.908709	0.973593	None
20212	CRTAP	0.015296	0.908731	0.973593	None
20213	INPP5K	-0.01662	0.908762	0.973593	None
20214	TGFBI	0.068529	0.908888	0.973681	None
20215	HIGD1A	-0.01948	0.908957	0.973705	None
20216	SPRN	0.003324	0.909164	0.973715	None
20217	SCYL2	-0.0178	0.909164	0.973715	None
20218	3-Mar	0.016145	0.9092	0.973715	None
20219	CD300LG	-0.00263	0.909208	0.973715	None
20220	C21orf67	-0.00019	0.909255	0.973715	None
20221	LOC21968ℓ	-0.00433	0.90927	0.973715	None
20222	ZNF652	0.026073	0.90928	0.973715	None
20223	RP11-574ŀ	-0.00015	0.90935	0.973742	None
20224	LINC00558	-0.00015	0.909424	0.973773	None
20225	CLYBL-AS1	-0.00049	0.909563	0.973852	None
20226	C4orf22	-0.00015	0.909636	0.973852	None
20227	KERA	-0.00015	0.909643	0.973852	None
20228	ZNF639	-0.01535	0.909678	0.973852	None
20229	FBXL4	-0.01846	0.909899	0.973982	None
20230	NBPF20	0.012079	0.909961	0.973982	None
20231	GRIK1	-0.00746	0.910024	0.973982	None
20232	TRIM37	-0.01309	0.910026	0.973982	None
20233	BHLHE22	0.006209	0.910062	0.973982	None
20234	AC007292.	-0.00363	0.910148	0.973982	None
20235	LOC10192!	-0.00015	0.910194	0.973982	None
20236	MIF4GD	0.02017	0.910203	0.973982	None
20237	LANCL1	0.009378	0.910204	0.973982	None
20238	IPO7	-0.01158	0.910489	0.974231	None
20239	ZBTB41	0.026056	0.910527	0.974231	None
20240	ANO3	-0.00015	0.910651	0.974292	None

20241	HPD	-0.00014	0.910684	0.974292	None
20242	LOC28518:	-0.00687	0.910742	0.974292	None
20243	ZDHHC24	0.017281	0.910768	0.974292	None
20244	GPR98	-0.00015	0.910848	0.974292	None
20245	LOC10192!	0.0028	0.910854	0.974292	None
20246	CLVS1	0.00016	0.910914	0.974308	None
20247	LOC10192!	-0.00842	0.91111	0.97447	None
20248	IGFBP3	-0.01195	0.911158	0.974472	None
20249	FAP	-0.00014	0.911617	0.974892	None
20250	MLLT4	-0.00015	0.91164	0.974892	None
20251	CNTN2	-0.00072	0.911898	0.97512	None
20252	ACVR1C	0.001372	0.912009	0.975125	None
20253	MEST	0.024361	0.912045	0.975125	None
20254	VPS18	0.008755	0.912068	0.975125	None
20255	APCDD1L	-0.00572	0.912118	0.975125	None
20256	PSIP1	0.014323	0.912166	0.975125	None
20257	C5orf55	0.010957	0.912186	0.975125	None
20258	LOC10028!	-0.00429	0.912317	0.975125	None
20259	ACTR1A	0.014789	0.912359	0.975125	None
20260	MBNL1-AS	0.014157	0.912389	0.975125	None
20261	JMJD1C-A:	-0.00585	0.912391	0.975125	None
20262	PRTN3	0.056591	0.912398	0.975125	None
20263	YEATS2	0.009727	0.912492	0.975167	None
20264	CD177	0.020653	0.912565	0.975167	None
20265	EIF1B-AS1	0.009086	0.912573	0.975167	None
20266	ITGAM	0.041335	0.912629	0.975179	None
20267	REM2	0.010304	0.912762	0.975274	None
20268	FIZ1	-0.00466	0.912853	0.975322	None
20269	LIMD1	0.008736	0.912906	0.975331	None
20270	RAD21	0.013754	0.912962	0.975342	None
20271	AC108056.	0.000139	0.913019	0.975355	None
20272	ATP2C2	-0.00188	0.913109	0.975366	None
20273	FASLG	0.001542	0.913151	0.975366	None
20274	KIF2A	0.015008	0.913165	0.975366	None
20275	LOC10012	-0.00014	0.913236	0.975395	None
20276	FAM71B	-0.00014	0.913409	0.975395	None
20277	AC090627.	-0.00175	0.913423	0.975395	None
20278	SLC10A4	0.060817	0.913442	0.975395	None
20279	WDPCP	0.010546	0.913478	0.975395	None
20280	MAD2L1	-0.03709	0.913509	0.975395	None
20281	ATP1A4	-0.00014	0.91354	0.975395	None
20282	BLZF1	0.019883	0.913552	0.975395	None
20283	LBX2-AS1	0.009621	0.913627	0.975403	None
20284	KALRN	-0.00151	0.913649	0.975403	None
20285	RP11-362k	-0.01164	0.91373	0.975441	None
20286	OR2B2	-0.00014	0.913791	0.975442	None
20287	MAP2K7	0.012299	0.91382	0.975442	None
20288	GABRG2	0.00301	0.913914	0.975481	None
20289	XYLT1	0.011671	0.913947	0.975481	None
20290	LOC15843	0.000471	0.914	0.975487	None
20291	LYPD6B	-0.00967	0.914043	0.975487	None
20292	RAPH1	-0.0135	0.914144	0.975546	None
20293	CA9	-0.00486	0.914201	0.975559	None
20294	BNIP1	-0.0177	0.91425	0.975561	None
20295	CCNYL1	0.018556	0.914293	0.975561	None
20296	NEK10	0.003467	0.914447	0.975678	None
20297	LOC38933:	0.003272	0.914527	0.975715	None
20298	DEFA6	0.001683	0.914823	0.975931	None

20299	LINC01141	-0.00014	0.914855	0.975931	None
20300	LOC10050!	0.002633	0.914879	0.975931	None
20301	DAPK2	-0.00447	0.91491	0.975931	None
20302	FMO9P	-0.00102	0.914962	0.975939	None
20303	PSPH	-0.03552	0.915046	0.97598	None
20304	MAP7D3	0.016362	0.915419	0.97633	None
20305	FGF5	-0.00014	0.915547	0.976353	None
20306	FLJ34521	0.002511	0.915552	0.976353	None
20307	FLJ39080	-0.00375	0.915576	0.976353	None
20308	NEUROG1	-0.00079	0.915711	0.976429	None
20309	PCDHAC2	0.000518	0.915765	0.976429	None
20310	PRKAR2B	-0.03307	0.915783	0.976429	None
20311	RAP2C-AS	-0.00603	0.915844	0.976447	None
20312	CRLF3	-0.00937	0.916164	0.976714	None
20313	GPR78	0.004345	0.916185	0.976714	None
20314	CACUL1	-0.007	0.916279	0.976734	None
20315	LARGE-AS	-0.00013	0.916295	0.976734	None
20316	DEFB123	-0.00014	0.916448	0.97685	None
20317	ISL1	-0.00014	0.916593	0.976956	None
20318	GPRASP1	-0.03947	0.916705	0.977028	None
20319	NDUFA4	0.010705	0.91677	0.977048	None
20320	WT1	0.027719	0.916899	0.977076	None
20321	CEMIP	-0.01576	0.916907	0.977076	None
20322	PYROXD1	-0.02083	0.916954	0.977076	None
20323	HTN3	-0.00014	0.916977	0.977076	None
20324	FUT3	0.000766	0.917034	0.977076	None
20325	PLAC9	-0.00179	0.917066	0.977076	None
20326	FAM149B1	-0.0164	0.917276	0.977224	None
20327	CYP4V2	0.021117	0.917302	0.977224	None
20328	LOC10192	-0.00181	0.917341	0.977224	None
20329	HNRNPH1	0.02587	0.917472	0.977285	None
20330	LGMN	0.064345	0.917488	0.977285	None
20331	SCAND2P	0.00203	0.917713	0.977476	None
20332	LOC10012	-0.00014	0.917793	0.977514	None
20333	NPFFR1	-0.00485	0.917924	0.97759	None
20334	Y16709	-0.02144	0.918035	0.97759	None
20335	DSG3	-0.00058	0.91804	0.97759	None
20336	APEX1	0.011133	0.918046	0.97759	None
20337	PBRM1	-0.01301	0.918284	0.977679	None
20338	AP4B1	-0.01006	0.918314	0.977679	None
20339	MMP13	0.004074	0.918318	0.977679	None
20340	LINC01233	-0.00088	0.918323	0.977679	None
20341	UCP1	-0.00014	0.918388	0.977679	None
20342	HSD17B10	0.015449	0.918454	0.977679	None
20343	CDK19	-0.01559	0.918523	0.977679	None
20344	TNFRSF1B	0.040302	0.91853	0.977679	None
20345	PARVA	0.00117	0.918586	0.977679	None
20346	PHLDB1	-0.00013	0.918615	0.977679	None
20347	STAR	0.032416	0.918626	0.977679	None
20348	SSPO	-0.0038	0.919045	0.978021	None
20349	WDR20	0.017525	0.919079	0.978021	None
20350	KLHL40	-0.00013	0.919082	0.978021	None
20351	LOC10192	-0.0043	0.919168	0.978047	None
20352	CCDC64B	0.001491	0.919197	0.978047	None
20353	LINC00556	-0.00013	0.919297	0.978105	None
20354	HECTD2	0.006636	0.919344	0.978107	None
20355	H2AFV	0.013388	0.91952	0.978204	None
20356	RP11-219E	-0.00179	0.919557	0.978204	None

20357	AC002064.	-0.00013	0.919587	0.978204	None
20358	BC053951	0.001715	0.919662	0.978204	None
20359	RP11-285(	0.000483	0.919686	0.978204	None
20360	BEND2	0.025042	0.919706	0.978204	None
20361	WFIKKN2	0.000939	0.919756	0.978209	None
20362	ZNF784	-0.00901	0.91999	0.978281	None
20363	DNLZ	-0.02309	0.920037	0.978281	None
20364	KIAA1614-	-0.00226	0.920067	0.978281	None
20365	LOC10192	0.001326	0.920185	0.978281	None
20366	MYLPF	-0.0041	0.920187	0.978281	None
20367	TEX22	0.005225	0.920334	0.978281	None
20368	ARFGAP3	0.019108	0.920351	0.978281	None
20369	LOC10013	-0.00013	0.920379	0.978281	None
20370	HCP5	0.027532	0.920387	0.978281	None
20371	DENND2A	-0.00013	0.9204	0.978281	None
20372	WDR87	-0.00317	0.920419	0.978281	None
20373	DPF3	-0.00342	0.920449	0.978281	None
20374	PPP1R1A	0.001324	0.920459	0.978281	None
20375	CIAPIN1	-0.01426	0.920509	0.978281	None
20376	RAB25	0.000127	0.92051	0.978281	None
20377	AGAP2-AS	-0.00013	0.920551	0.978281	None
20378	ARID5B	0.001447	0.920596	0.978281	None
20379	RLN2	0.023047	0.920637	0.978281	None
20380	TMEM45B	-0.00164	0.920691	0.978291	None
20381	ZNF12	-0.0177	0.920932	0.978444	None
20382	LOC28595	-0.02467	0.920968	0.978444	None
20383	ILDR2	0.014555	0.920971	0.978444	None
20384	BC042366	0.006211	0.92102	0.978448	None
20385	MAP4K5	-0.01526	0.921238	0.978621	None
20386	TSACC	0.004924	0.921302	0.978621	None
20387	TSSK6	-0.00067	0.921318	0.978621	None
20388	TMX4	0.013791	0.921379	0.978637	None
20389	FGL2	-0.04498	0.921586	0.97878	None
20390	LINC00555	-0.00013	0.921615	0.97878	None
20391	SLTM	0.010043	0.921649	0.97878	None
20392	RP11-199F	-0.02537	0.922129	0.979218	None
20393	PFN2	0.032114	0.922151	0.979218	None
20394	CSPP1	0.018053	0.9225	0.97954	None
20395	TNFRSF14	-0.01776	0.922631	0.979598	None
20396	SNRNP40	0.029427	0.922646	0.979598	None
20397	RP11-517E	0.009626	0.922776	0.979689	None
20398	LOC10192	-0.00575	0.922877	0.979744	None
20399	NOX5	0.002497	0.922972	0.979744	None
20400	ZNF547	0.007572	0.923065	0.979744	None
20401	KRT83	-0.00276	0.923115	0.979744	None
20402	NCOR2	-0.00564	0.923208	0.979744	None
20403	NLRC3	-0.01935	0.923232	0.979744	None
20404	MST1R	0.001116	0.923238	0.979744	None
20405	CHRNA5	-0.00187	0.923268	0.979744	None
20406	RWDD1	-0.01064	0.923308	0.979744	None
20407	NSG1	-0.00418	0.923339	0.979744	None
20408	F13A1	-0.04418	0.923379	0.979744	None
20409	TUNAR	-0.00108	0.923428	0.979744	None
20410	LOC10192	-0.00013	0.923461	0.979744	None
20411	LOC10013	-0.00229	0.923514	0.979744	None
20412	PKIA	0.027082	0.923518	0.979744	None
20413	CD7	0.007208	0.923552	0.979744	None
20414	ZC3H13	0.015411	0.923634	0.979774	None

20415	ADAM30	0.000648	0.923721	0.979774	None
20416	TRIM50	-0.00013	0.923734	0.979774	None
20417	FAM213A	-0.02361	0.923761	0.979774	None
20418	CHURC1	0.017069	0.923933	0.979908	None
20419	LOC10099(	-0.00044	0.924047	0.979931	None
20420	ERBB4	-0.00038	0.924114	0.979931	None
20421	LBH	0.023288	0.924121	0.979931	None
20422	HMGN3	-0.01244	0.924176	0.979931	None
20423	PTH2	-0.00331	0.924181	0.979931	None
20424	C10orf53	-0.00077	0.924284	0.979993	None
20425	RND3	-0.0452	0.924354	0.98001	None
20426	DPP7	-0.01796	0.924401	0.98001	None
20427	ZNF428	-0.0028	0.924475	0.98001	None
20428	TTC6	-0.00014	0.924482	0.98001	None
20429	MTPAP	-0.01221	0.924541	0.980025	None
20430	HSP90B1	-0.01755	0.924692	0.980138	None
20431	ERBB2IP	0.014186	0.924797	0.980168	None
20432	LOC10192	-0.00012	0.924811	0.980168	None
20433	RP11-314M	-0.01442	0.924888	0.980201	None
20434	AP000347.	-0.01448	0.924991	0.980262	None
20435	LOC10192	0.001381	0.925302	0.980494	None
20436	CRYBA2	-0.00015	0.92534	0.980494	None
20437	RMND5A	-0.01231	0.925412	0.980494	None
20438	LOC34488	-0.00384	0.925421	0.980494	None
20439	RBM48	0.013285	0.925436	0.980494	None
20440	ASB16	-0.00036	0.925488	0.980501	None
20441	TNMD	-0.00218	0.925562	0.980531	None
20442	VIPR1	-0.00355	0.92567	0.980598	None
20443	KRTAP2-1	-0.00031	0.925767	0.980628	None
20444	CNTNAP3	-0.00012	0.925789	0.980628	None
20445	RP11-676J	0.000393	0.925905	0.980688	None
20446	CADM2-A	0.000785	0.925939	0.980688	None
20447	MORF4L2	0.021708	0.925982	0.980688	None
20448	STX10	-0.01068	0.92604	0.98069	None
20449	RP11-305(	-0.00108	0.926082	0.98069	None
20450	CASQ2	-0.00167	0.926169	0.98069	None
20451	AC012360.	-0.00012	0.926185	0.98069	None
20452	LINC00969	-0.01812	0.926209	0.98069	None
20453	LOC10192	0.001367	0.926389	0.980818	None
20454	TMEM241	0.010002	0.926443	0.980818	None
20455	LOC44033(	-0.00215	0.926467	0.980818	None
20456	LINC00629	-0.00323	0.926528	0.980835	None
20457	LINC00551	-0.00043	0.92669	0.980928	None
20458	MMP2	0.002245	0.926706	0.980928	None
20459	ADCYAP1F	0.001469	0.92683	0.980964	None
20460	NFYC-AS1	-0.01414	0.926831	0.980964	None
20461	MYADM	0.016574	0.926925	0.980977	None
20462	HOXC12	-0.00317	0.926964	0.980977	None
20463	GAS2L2	-0.00012	0.926996	0.980977	None
20464	PCBP1-AS	-0.0053	0.927024	0.980977	None
20465	FOXF1	0.001131	0.927097	0.981006	None
20466	FGF11	-0.00208	0.927154	0.981018	None
20467	TWIST1	-0.02344	0.927523	0.981361	None
20468	TTC30A	-0.00849	0.927608	0.981396	None
20469	TULP1	0.001159	0.927647	0.981396	None
20470	SSFA2	-0.01695	0.927697	0.981401	None
20471	TTTY12	-0.00012	0.927784	0.981408	None
20472	NEK1	0.016034	0.927794	0.981408	None

20473	RP13-122E	0.001814	0.927867	0.981437	None
20474	LOC10192	-0.00039	0.928087	0.981612	None
20475	LILRB2	-0.03216	0.928132	0.981612	None
20476	ALDH1L1	-0.00288	0.928169	0.981612	None
20477	CCL25	-0.00183	0.928238	0.981638	None
20478	FBXO2	-0.00076	0.928516	0.981733	None
20479	RP11-225H	-0.00012	0.928516	0.981733	None
20480	PCGF3	-0.00723	0.928588	0.981733	None
20481	HEXB	0.012334	0.928597	0.981733	None
20482	RNASEH2A	-0.01971	0.928616	0.981733	None
20483	ELP2	-0.01371	0.928678	0.981733	None
20484	SPEF2	0.007261	0.928698	0.981733	None
20485	ZNF550	0.007418	0.928725	0.981733	None
20486	SASS6	0.010302	0.928736	0.981733	None
20487	CAMKMT	0.006826	0.928797	0.981749	None
20488	SMPD2	-0.01055	0.928909	0.981819	None
20489	KIAA1324L	-0.02038	0.929118	0.981993	None
20490	AP000265.	0.000727	0.929259	0.98201	None
20491	FLJ12825	-0.00035	0.929267	0.98201	None
20492	ZNF208	0.004657	0.929288	0.98201	None
20493	GDF2	-0.00279	0.929315	0.98201	None
20494	LINC00669	-0.01075	0.929366	0.982015	None
20495	CD47	0.022797	0.929519	0.982129	None
20496	ASCL3	0.000114	0.929643	0.982129	None
20497	KCNJ15	0.004122	0.929649	0.982129	None
20498	NPM1	0.011096	0.929655	0.982129	None
20499	TMEM57	-0.01411	0.929727	0.982138	None
20500	DOCK11	-0.01325	0.929765	0.982138	None
20501	ATCAY	-0.00438	0.9298	0.982138	None
20502	TTLL10	-0.00011	0.929969	0.982245	None
20503	AC005537.	-0.00011	0.930014	0.982245	None
20504	LOC38894	0.000965	0.930094	0.982245	None
20505	POM121L8	0.004194	0.930116	0.982245	None
20506	RP11-378A	0.014711	0.930129	0.982245	None
20507	CCL28	0.014491	0.930184	0.982245	None
20508	LOC10050	-0.00037	0.930234	0.982245	None
20509	CRISPLD1	-0.0159	0.930264	0.982245	None
20510	SNRPD1	0.009347	0.930397	0.982337	None
20511	C7orf33	0.00138	0.930487	0.982385	None
20512	C9orf50	-0.00344	0.930693	0.982554	None
20513	WISP3	-0.00029	0.93076	0.982577	None
20514	CHST8	-0.00363	0.93089	0.982647	None
20515	OR1A2	-0.00028	0.930916	0.982647	None
20516	TUBB2A	-0.04647	0.931115	0.982782	None
20517	LOC28333	0.001732	0.931135	0.982782	None
20518	CRH	0.002172	0.931243	0.982848	None
20519	RPE	0.011016	0.931522	0.982965	None
20520	VNN2	0.03647	0.931536	0.982965	None
20521	UQCC2	-0.00282	0.93162	0.982965	None
20522	NR2F2-AS	-0.00087	0.931622	0.982965	None
20523	DLGAP1	-0.00073	0.93163	0.982965	None
20524	SMAP2	-0.0138	0.931655	0.982965	None
20525	CTB-181H	0.020276	0.931672	0.982965	None
20526	ZNF649	-0.00423	0.931724	0.982972	None
20527	TMEM220	-0.01267	0.931791	0.982995	None
20528	GNG10	0.014951	0.931913	0.983075	None
20529	PGF	0.005973	0.932096	0.983213	None
20530	ANKAR	-0.00156	0.932134	0.983213	None

20531	SNORD8	-0.00438	0.932204	0.983238	None
20532	CSRNP2	-0.00778	0.932362	0.983313	None
20533	AFG3L2	-0.01152	0.932365	0.983313	None
20534	PDGFD	0.017918	0.932601	0.983514	None
20535	PLAC4	0.002106	0.932824	0.983701	None
20536	PRKCG	-0.00115	0.933011	0.98379	None
20537	VDAC2	-0.00794	0.933035	0.98379	None
20538	PLCG1-AS.	-0.0017	0.933086	0.98379	None
20539	LOC10192!	0.002613	0.933162	0.98379	None
20540	CHIA	0.000921	0.933165	0.98379	None
20541	TBATA	0.004891	0.933181	0.98379	None
20542	C6orf118	0.00069	0.933293	0.983842	None
20543	SNORA21	0.020629	0.933321	0.983842	None
20544	IDO1	0.008227	0.933383	0.98386	None
20545	AC079305.	0.017176	0.933505	0.9839	None
20546	LMBR1L	-0.01302	0.933525	0.9839	None
20547	TRABD2B	-0.00011	0.933587	0.9839	None
20548	JSRP1	-0.00645	0.933604	0.9839	None
20549	KLHDC4	0.007832	0.933755	0.984013	None
20550	CHST9	-0.00011	0.933871	0.984087	None
20551	TCERG1L	-0.00011	0.933925	0.984095	None
20552	PHYHIP	-0.00011	0.934187	0.984324	None
20553	LOC10192!	0.001059	0.934322	0.984394	None
20554	OR10D3	0.000668	0.934345	0.984394	None
20555	ST8SIA4	-0.02249	0.934409	0.984397	None
20556	FLJ38576	-0.00011	0.934438	0.984397	None
20557	RHBDL1	-0.00048	0.934789	0.984655	None
20558	RMST	-0.0001	0.934795	0.984655	None
20559	SLC28A1	-0.00095	0.93491	0.984655	None
20560	HOXA-AS3	0.001914	0.934985	0.984655	None
20561	PPP6C	-0.0079	0.935008	0.984655	None
20562	SPRY4-IT1	-0.0001	0.935022	0.984655	None
20563	EFEMP2	-0.00993	0.935042	0.984655	None
20564	ZFP2	0.007725	0.935072	0.984655	None
20565	LOC10192!	0.014998	0.935093	0.984655	None
20566	LOC10192!	-0.0001	0.935207	0.984717	None
20567	C2orf82	-0.00376	0.935242	0.984717	None
20568	U47924.29	-0.0001	0.935329	0.984748	None
20569	AKIRIN1	-0.01298	0.935422	0.984748	None
20570	SRCIN1	-0.0001	0.935439	0.984748	None
20571	BAZ2A	0.00676	0.935454	0.984748	None
20572	AF131215..	0.006194	0.935618	0.984839	None
20573	PSMD4	-0.00933	0.935631	0.984839	None
20574	FIP1L1	0.012207	0.935781	0.984929	None
20575	FGFR1OP	0.010226	0.93585	0.984929	None
20576	ZNF470	-0.00571	0.935877	0.984929	None
20577	ZDHHC2	-0.01129	0.935899	0.984929	None
20578	LINC00620	-0.0001	0.93606	0.984946	None
20579	GHRL	0.003978	0.936069	0.984946	None
20580	KREMEN2	0.001752	0.936112	0.984946	None
20581	ZNF140	-0.01828	0.93612	0.984946	None
20582	ZNF578	-0.01729	0.936142	0.984946	None
20583	HTATSF1	0.010738	0.936282	0.985046	None
20584	RGS17	-0.00595	0.936382	0.985103	None
20585	SFMBT2	0.011377	0.936496	0.985174	None
20586	LOC10192!	0.002152	0.936709	0.985351	None
20587	IQSEC1	0.016652	0.936794	0.985367	None
20588	TCEAL4	0.008289	0.936855	0.985367	None

20589	LACRT	0.001516	0.936861	0.985367	None
20590	LOC28451	-0.01565	0.936949	0.985412	None
20591	DMRTA1	-0.00018	0.937021	0.985439	None
20592	SCG3	-0.0001	0.937143	0.985442	None
20593	LOC33953	-0.00109	0.937265	0.985442	None
20594	CDH22	0.001462	0.937373	0.985442	None
20595	FOXM1	0.02274	0.937503	0.985442	None
20596	C17orf105	0.001821	0.937585	0.985442	None
20597	GCNT4	0.003232	0.937589	0.985442	None
20598	LOC10192	-0.0001	0.937623	0.985442	None
20599	CHST4	-0.00387	0.937684	0.985442	None
20600	IPO9	0.009094	0.937708	0.985442	None
20601	EPHA7	-9.95E-05	0.93771	0.985442	None
20602	HAND2-A	-0.00088	0.937723	0.985442	None
20603	PKD2L2	0.002747	0.937739	0.985442	None
20604	MEI1	-0.01091	0.93776	0.985442	None
20605	APCDD1L-	-9.98E-05	0.93776	0.985442	None
20606	ZDHHC18	0.003253	0.937782	0.985442	None
20607	DTNBP1	0.013675	0.937791	0.985442	None
20608	RORC	0.000801	0.937796	0.985442	None
20609	LOC40089	-0.00234	0.937943	0.985548	None
20610	SLC1A6	0.020392	0.938048	0.985581	None
20611	SLC25A46	-0.01041	0.938066	0.985581	None
20612	PARD3B	-0.00056	0.938189	0.98559	None
20613	RP11-416I	-0.0001	0.938195	0.98559	None
20614	GNG11	0.024938	0.938225	0.98559	None
20615	FEM1B	-0.012	0.938374	0.98559	None
20616	VMO1	0.010141	0.93842	0.98559	None
20617	PIANP	-0.00191	0.938447	0.98559	None
20618	IMPA1	-0.01216	0.93846	0.98559	None
20619	LOC33998	0.00975	0.938473	0.98559	None
20620	MLF1	0.025501	0.938564	0.98559	None
20621	TMEM26	-0.00225	0.938576	0.98559	None
20622	LINC00165	-9.96E-05	0.938751	0.98559	None
20623	FGF6	-0.00407	0.938857	0.98559	None
20624	SSTR2	0.00092	0.938874	0.98559	None
20625	LINC0071C	0.0002	0.938897	0.98559	None
20626	NTN4	-0.01932	0.938951	0.98559	None
20627	IBA57-AS1	-0.0028	0.938954	0.98559	None
20628	ARG1	0.001367	0.938961	0.98559	None
20629	USP42	0.002465	0.939007	0.98559	None
20630	OR1D5	-9.88E-05	0.939052	0.98559	None
20631	LIPJ	-9.73E-05	0.939094	0.98559	None
20632	TMEM150E	-0.00134	0.939102	0.98559	None
20633	WRAP53	-0.00939	0.939107	0.98559	None
20634	LOC72988	0.003528	0.939121	0.98559	None
20635	ABCA8	-9.77E-05	0.939288	0.985677	None
20636	CHD4	0.011122	0.939295	0.985677	None
20637	ATP6V0A2	-0.01322	0.939375	0.985713	None
20638	HYLS1	0.018681	0.939506	0.98574	None
20639	DGCR7	0.000721	0.939588	0.98574	None
20640	RBM27	0.009271	0.93963	0.98574	None
20641	RP11-307F	-0.00187	0.939728	0.98574	None
20642	LOC10099	0.000534	0.939805	0.98574	None
20643	CLEC4M	0.010151	0.939857	0.98574	None
20644	SRL	0.003887	0.939918	0.98574	None
20645	CYP2D6	0.002659	0.94001	0.98574	None
20646	PSG3	-9.58E-05	0.94004	0.98574	None

20647	CEP128	0.011955	0.94005	0.98574	None
20648	PRSS33	0.002285	0.940101	0.98574	None
20649	CEP135	0.013156	0.940109	0.98574	None
20650	SLC19A2	0.016097	0.940114	0.98574	None
20651	ZDHHC13	0.008092	0.940154	0.98574	None
20652	ERV3-1	-0.01331	0.940182	0.98574	None
20653	RP11-305E	-0.0193	0.940208	0.98574	None
20654	PDGFB	-0.00249	0.940289	0.98574	None
20655	PLP2	-0.01778	0.940362	0.98574	None
20656	ARHGEF5	0.004012	0.940406	0.98574	None
20657	LOC64677	0.011221	0.940437	0.98574	None
20658	PLA2G5	-9.67E-05	0.940469	0.98574	None
20659	SIAE	-0.00222	0.940479	0.98574	None
20660	UBQLN3	-9.59E-05	0.940516	0.98574	None
20661	ZNF570	-0.00466	0.940548	0.98574	None
20662	HNF4G	-0.00132	0.940588	0.98574	None
20663	OVOL3	-0.00036	0.940619	0.98574	None
20664	LINC00485	0.002045	0.9407	0.98574	None
20665	LOC10192	-0.00148	0.940714	0.98574	None
20666	SERPINA5	-0.00513	0.940721	0.98574	None
20667	SPG11	0.003534	0.940801	0.985777	None
20668	MAFK	-0.00878	0.940974	0.985846	None
20669	CMTM3	-0.01395	0.940974	0.985846	None
20670	TPCN1	-0.00561	0.941039	0.985846	None
20671	HEY2	-0.00181	0.941049	0.985846	None
20672	TRIM40	0.00141	0.9411	0.985851	None
20673	TIFA	0.015451	0.941181	0.985861	None
20674	FCER2	-0.00149	0.9412	0.985861	None
20675	CELF2-AS1	0.001573	0.941305	0.985923	None
20676	PF4	-0.046	0.941434	0.98601	None
20677	IL31RA	-9.48E-05	0.941525	0.986058	None
20678	BAIAP2	0.003853	0.94165	0.986141	None
20679	SH3BP5-A	-0.01254	0.941751	0.986199	None
20680	LOC10192	0.003691	0.941956	0.986301	None
20681	SAMD13	0.011257	0.942022	0.986301	None
20682	GGTA1P	-0.02763	0.942025	0.986301	None
20683	PAX8	0.001254	0.942052	0.986301	None
20684	PSMD6-AS	0.011103	0.942076	0.986301	None
20685	KCNIP3	-9.37E-05	0.942186	0.986351	None
20686	NADK2-AS	0.000705	0.942214	0.986351	None
20687	FAM205B	0.001779	0.942369	0.986433	None
20688	TMCO5A	-9.34E-05	0.942395	0.986433	None
20689	TEX30	-0.01004	0.942482	0.986433	None
20690	1-Mar	-0.02447	0.942508	0.986433	None
20691	TNIP3	0.001083	0.94252	0.986433	None
20692	RP13-436F	0.001276	0.942638	0.986464	None
20693	TAS1R2	-9.28E-05	0.942693	0.986464	None
20694	ABCB5	-9.25E-05	0.942708	0.986464	None
20695	PELO	-0.00178	0.942732	0.986464	None
20696	CAPN11	-0.00862	0.942818	0.986504	None
20697	NAA20	0.00828	0.942935	0.986504	None
20698	CSMD1	-9.24E-05	0.94296	0.986504	None
20699	CDK5R1	0.007165	0.943042	0.986504	None
20700	NDC1	0.01125	0.943081	0.986504	None
20701	LRRC2-AS	-9.09E-05	0.943102	0.986504	None
20702	LOC28559	0.000888	0.943112	0.986504	None
20703	C19orf43	0.008495	0.943135	0.986504	None
20704	PADI1	-0.00258	0.943205	0.986524	None

20705	RAC3	-0.00624	0.943276	0.986524	None
20706	IFT88	0.008121	0.943291	0.986524	None
20707	RP11-109E	-0.00097	0.943452	0.986574	None
20708	KIF24	0.000824	0.943679	0.986574	None
20709	IL4	-0.00135	0.943706	0.986574	None
20710	CHRM2	-0.00018	0.943759	0.986574	None
20711	AGPAT4	-0.01137	0.943816	0.986574	None
20712	CHPF	0.002595	0.943858	0.986574	None
20713	ZFAND4	0.006333	0.943867	0.986574	None
20714	TOPORS	-0.01288	0.943874	0.986574	None
20715	PHF20	-0.01312	0.943877	0.986574	None
20716	DQ583756	-0.00199	0.943878	0.986574	None
20717	JPH2	-9.08E-05	0.943915	0.986574	None
20718	AKAP14	-8.94E-05	0.943915	0.986574	None
20719	DCTPP1	0.010059	0.943931	0.986574	None
20720	TEX26-AS1	-9.06E-05	0.944043	0.986588	None
20721	LOC10013(	-0.01105	0.944046	0.986588	None
20722	GDF3	0.003014	0.944099	0.986588	None
20723	C3orf65	0.000125	0.944143	0.986588	None
20724	PIP5KL1	0.003991	0.944224	0.986588	None
20725	IDNK	0.012746	0.944298	0.986588	None
20726	WDR11-AS	-0.00457	0.94436	0.986588	None
20727	ZNF556	-0.00278	0.944366	0.986588	None
20728	LEMD2	-0.00686	0.944438	0.986588	None
20729	FDPSP2	0.002255	0.944454	0.986588	None
20730	LOC10192	-8.85E-05	0.944483	0.986588	None
20731	WNT8A	-0.00148	0.944522	0.986588	None
20732	AOC3	-0.02371	0.944634	0.986588	None
20733	DMRT3	0.000149	0.944654	0.986588	None
20734	COL10A1	-0.01005	0.944704	0.986588	None
20735	FOXD2	0.001011	0.944736	0.986588	None
20736	IGSF11	-8.87E-05	0.944737	0.986588	None
20737	RFX7	0.0084	0.944765	0.986588	None
20738	CCDC12	-0.00871	0.94508	0.986827	None
20739	MBNL1	0.015305	0.945201	0.986827	None
20740	RP11-305L	-8.86E-05	0.945218	0.986827	None
20741	RP11-395N	0.000573	0.94524	0.986827	None
20742	SMPX	-8.74E-05	0.945307	0.986827	None
20743	PNPLA5	-0.00014	0.945358	0.986827	None
20744	LZTS1	-8.83E-05	0.94537	0.986827	None
20745	N6AMT1	0.003438	0.945476	0.986827	None
20746	LMNTD2	0.005579	0.945498	0.986827	None
20747	RPS10L	-0.0115	0.945541	0.986827	None
20748	ZKSCAN5	0.00693	0.945569	0.986827	None
20749	PRLH	0.005092	0.945616	0.986827	None
20750	ECT2	0.003783	0.945655	0.986827	None
20751	GJB5	-8.73E-05	0.945677	0.986827	None
20752	HMOX2	-0.00817	0.945677	0.986827	None
20753	RP1-78O1	-8.70E-05	0.945818	0.986901	None
20754	WIPI1	-0.0223	0.945872	0.986901	None
20755	STX17	-0.00932	0.945912	0.986901	None
20756	ZNF623	0.013591	0.94593	0.986901	None
20757	LOC10013(	0.008598	0.946179	0.987087	None
20758	PPBPP2	-8.64E-05	0.946199	0.987087	None
20759	CD81-AS1	0.000888	0.946272	0.987107	None
20760	MIS18BP1	0.020142	0.946378	0.987107	None
20761	CROCCP3	0.000682	0.946417	0.987107	None
20762	UBE2T	-0.02012	0.946431	0.987107	None

20763	HENMT1	0.008015	0.946456	0.987107	None
20764	FUT7	-0.00521	0.946492	0.987107	None
20765	BC040833	-0.00117	0.946552	0.987122	None
20766	RP11-727A	0.012804	0.946757	0.987247	None
20767	GBP1	0.022277	0.946763	0.987247	None
20768	PAGR1	-0.0075	0.946873	0.987287	None
20769	ARMC3	-8.57E-05	0.94693	0.987287	None
20770	PDCD10	-0.00905	0.946939	0.987287	None
20771	KCTD6	-0.0093	0.94704	0.987346	None
20772	LIMD2	-0.00337	0.947128	0.987355	None
20773	ABCA4	0.000505	0.947141	0.987355	None
20774	MYCN	0.000451	0.947243	0.987414	None
20775	PRPH2	-8.41E-05	0.947362	0.98749	None
20776	SRC	8.47E-05	0.947698	0.987793	None
20777	GPR107	0.006247	0.947802	0.987855	None
20778	RNF11	0.018477	0.947995	0.987957	None
20779	PGK2	-0.002	0.948015	0.987957	None
20780	EREG	0.0282	0.948082	0.987957	None
20781	NUP93	0.004647	0.948088	0.987957	None
20782	ANAPC11	0.003413	0.948128	0.987957	None
20783	PHC3	-0.01187	0.948226	0.988007	None
20784	IGFBP5	-0.00152	0.948267	0.988007	None
20785	HCAR3	0.032268	0.948325	0.988019	None
20786	TMEM86A	-0.00791	0.948412	0.988062	None
20787	PIH1D3	-0.00133	0.948587	0.988197	None
20788	NDUFA1	-0.0068	0.948705	0.988272	None
20789	LTBP1	-0.02559	0.948846	0.988371	None
20790	FOSB	0.020821	0.949438	0.98891	None
20791	LOC10037!	-0.00349	0.949472	0.98891	None
20792	RP11-24D1	-8.05E-05	0.949538	0.98891	None
20793	CYP4F8	0.001726	0.94955	0.98891	None
20794	AC012065.	0.003571	0.949667	0.98891	None
20795	FICD	0.006929	0.949699	0.98891	None
20796	NAP1L1	-0.0094	0.949806	0.98891	None
20797	SIGLEC15	0.00707	0.949856	0.98891	None
20798	SPERT	-0.00084	0.949908	0.98891	None
20799	TMX1	0.008842	0.949916	0.98891	None
20800	LOC10192!	-0.00121	0.949929	0.98891	None
20801	IL17RA	0.013223	0.949929	0.98891	None
20802	RAE1	-0.01021	0.950001	0.98891	None
20803	BTG3	0.007655	0.95001	0.98891	None
20804	BCRP3	-0.00143	0.950048	0.98891	None
20805	TTN	-0.00256	0.950147	0.988966	None
20806	DOCK4-AS	0.001308	0.950194	0.988967	None
20807	CYP27A1	-0.00508	0.950442	0.989178	None
20808	LOC10106	-7.91E-05	0.950577	0.989271	None
20809	PCDHA2	-7.93E-05	0.950734	0.989383	None
20810	SOCS7	0.007158	0.950792	0.989383	None
20811	CYBB	-0.01266	0.950822	0.989383	None
20812	CSN3	-7.89E-05	0.950971	0.9894	None
20813	LOC64335!	-0.00036	0.951002	0.9894	None
20814	ASAH1	-0.01011	0.951045	0.9894	None
20815	GLRA3	-9.56E-05	0.951086	0.9894	None
20816	ROS1	-0.00066	0.951131	0.9894	None
20817	LINC00354	0.000381	0.951131	0.9894	None
20818	RGAG4	-0.00721	0.951158	0.9894	None
20819	LOC10192!	-0.00038	0.951218	0.989415	None
20820	RFC4	-0.00898	0.951335	0.989455	None

20821	PAPD5	0.008485	0.951375	0.989455	None
20822	SHPRH	-0.01398	0.951394	0.989455	None
20823	MTA2	-0.00279	0.951496	0.989494	None
20824	PSMA2	0.005503	0.951523	0.989494	None
20825	MS4A15	-0.00024	0.95158	0.989506	None
20826	CALCRL	-0.01273	0.951748	0.989591	None
20827	LOC28335	0.013359	0.951753	0.989591	None
20828	ELAVL2	-0.0006	0.951803	0.989596	None
20829	MOP-1	0.00943	0.952068	0.989824	None
20830	LRRC63	0.002048	0.952233	0.989914	None
20831	CALCOCO	-0.00976	0.952246	0.989914	None
20832	KIAA1614	-0.0006	0.952292	0.989914	None
20833	CST6	0.001897	0.952477	0.989985	None
20834	LOC10192	-7.57E-05	0.952497	0.989985	None
20835	LOC10272	0.003582	0.952497	0.989985	None
20836	FGF8	0.000504	0.952626	0.990011	None
20837	GRPEL2	0.006535	0.952662	0.990011	None
20838	LOC10192	-7.54E-05	0.952681	0.990011	None
20839	HADHA	-0.00863	0.952705	0.990011	None
20840	MET	-0.00032	0.952973	0.990174	None
20841	NBAS	0.010939	0.95301	0.990174	None
20842	TRHDE-AS	-0.00727	0.953036	0.990174	None
20843	NAA10	0.007366	0.953045	0.990174	None
20844	HTN1	-7.47E-05	0.953094	0.990176	None
20845	KLHL17	-0.00146	0.95318	0.990176	None
20846	RMI2	0.012998	0.95327	0.990176	None
20847	PCDH1	-0.00238	0.953291	0.990176	None
20848	HMGXB4	0.007081	0.95331	0.990176	None
20849	MPC1	0.004099	0.953322	0.990176	None
20850	RP11-742E	-0.0003	0.953453	0.990265	None
20851	RP11-619L	-7.44E-05	0.953684	0.990456	None
20852	PIWIL1	0.004443	0.953757	0.990456	None
20853	ZNF418	-0.01204	0.953775	0.990456	None
20854	HUS1B	0.00326	0.954132	0.990767	None
20855	PSME4	-0.00667	0.954197	0.990767	None
20856	LRRC37A5I	7.23E-05	0.954216	0.990767	None
20857	EMR2	0.009494	0.954257	0.990767	None
20858	BCAR4	-7.25E-05	0.95439	0.990858	None
20859	ATP1A1	-0.00857	0.95446	0.990884	None
20860	LILRA3	0.015089	0.954521	0.990899	None
20861	VTI1A	-0.00879	0.954839	0.991159	None
20862	ZADH2	0.00758	0.954936	0.991159	None
20863	PRR32	-0.00174	0.954941	0.991159	None
20864	KLK8	-0.00134	0.954955	0.991159	None
20865	UBR3	-0.00482	0.955162	0.991231	None
20866	LOC10192	0.001593	0.955166	0.991231	None
20867	LRP10	-0.00748	0.955194	0.991231	None
20868	POU2F1	-0.00495	0.955207	0.991231	None
20869	ARHGAP11	0.012349	0.955405	0.991372	None
20870	GFRA3	-0.00066	0.955434	0.991372	None
20871	LOC40054	-7.13E-05	0.955515	0.991408	None
20872	SMS	0.010482	0.955674	0.991485	None
20873	DLX3	0.00117	0.955681	0.991485	None
20874	GLYAT	-0.00015	0.955988	0.991756	None
20875	PAOX	-0.00836	0.9561	0.991825	None
20876	PLK5	-6.96E-05	0.956166	0.991846	None
20877	FGFBP2	0.007923	0.956214	0.991848	None
20878	RLBP1	-6.98E-05	0.956441	0.992036	None

20879	LOC10012!	-6.92E-05	0.956537	0.992083	None
20880	ZNF143	-0.00566	0.956634	0.992083	None
20881	SNHG10	0.003777	0.956639	0.992083	None
20882	CTD-2293I	-0.00321	0.95667	0.992083	None
20883	CYP4F3	-0.0161	0.956767	0.992137	None
20884	C20orf166	-0.00271	0.957021	0.992352	None
20885	AK131021	0.000682	0.957156	0.992434	None
20886	ALG5	0.000997	0.957193	0.992434	None
20887	TMEM70	0.006868	0.957237	0.992434	None
20888	MNDA	0.025103	0.957365	0.992519	None
20889	CXorf65	-0.00962	0.957494	0.992552	None
20890	FBXL17	0.010797	0.957543	0.992552	None
20891	TMPRSS2	-0.00139	0.957554	0.992552	None
20892	BMP8A	0.000798	0.957584	0.992552	None
20893	NCAM1	-0.00011	0.957626	0.992552	None
20894	CDCA3	-0.01491	0.957802	0.992668	None
20895	EMC9	0.012057	0.957878	0.992668	None
20896	IL17D	-0.00744	0.957916	0.992668	None
20897	G6PC2	-0.00071	0.957948	0.992668	None
20898	UBTD1	0.002898	0.957967	0.992668	None
20899	SFTPB	0.002822	0.958109	0.992727	None
20900	MPP4	-0.00096	0.958115	0.992727	None
20901	ALYREF	0.014522	0.958186	0.992752	None
20902	UHMK1	0.012378	0.958281	0.992775	None
20903	RP11-83N!	0.001923	0.9583	0.992775	None
20904	COLCA2	-0.00053	0.95836	0.99279	None
20905	LOC10192!	-6.64E-05	0.95852	0.992893	None
20906	LOC10192	-0.00163	0.958551	0.992893	None
20907	SEMA3A	0.001411	0.958643	0.992941	None
20908	DPY19L3	0.006621	0.958831	0.993051	None
20909	CALD1	0.001705	0.958876	0.993051	None
20910	INTS4	-0.00271	0.958887	0.993051	None
20911	FAM105A	0.014058	0.958962	0.993065	None
20912	RNFT1	-0.00672	0.958993	0.993065	None
20913	CDHR5	-6.54E-05	0.959112	0.993068	None
20914	ZNF506	0.008016	0.959116	0.993068	None
20915	METTL9	-0.00688	0.959153	0.993068	None
20916	LOC10050(	0.002564	0.959179	0.993068	None
20917	BARX1	-0.00092	0.959265	0.993104	None
20918	RP11-114I	-0.00081	0.959317	0.993104	None
20919	MED29	0.006538	0.959351	0.993104	None
20920	SPATS2L	-0.00458	0.959575	0.993282	None
20921	PER4	0.00019	0.959615	0.993282	None
20922	DONSON	0.008235	0.959755	0.99338	None
20923	DUOXA2	0.006802	0.959867	0.993449	None
20924	SOX2-OT	-6.37E-05	0.95992	0.993456	None
20925	ATP6V1C2	-0.00034	0.960472	0.993934	None
20926	WDR19	0.00333	0.960474	0.993934	None
20927	RP11-157E	0.000795	0.960633	0.994051	None
20928	RNASEH2C	-0.00582	0.960724	0.994098	None
20929	ANKRD22	0.007217	0.960933	0.994267	None
20930	PEX5L	-6.16E-05	0.961233	0.994529	None
20931	LINC01015	-0.00212	0.961302	0.994546	None
20932	SORCS3-A	0.000285	0.961347	0.994546	None
20933	RHOBTB1	-0.0138	0.961413	0.994546	None
20934	LOC28307!	-0.00027	0.961432	0.994546	None
20935	LOC10050(	0.000259	0.961564	0.994635	None
20936	A1BG-AS1	0.00342	0.961891	0.994873	None

20937	ARL4D	-0.00153	0.961907	0.994873	None
20938	TMF1	-0.00654	0.961933	0.994873	None
20939	LOC10192	0.000169	0.962095	0.994994	None
20940	LOC10065	-0.00074	0.962212	0.995067	None
20941	LOC40144	-5.99E-05	0.962425	0.99524	None
20942	PANX1	0.005358	0.962512	0.995282	None
20943	NXN	0.012391	0.962579	0.995304	None
20944	LINC00208	-0.00143	0.962695	0.995377	None
20945	LINC00907	-5.97E-05	0.962744	0.995379	None
20946	LOC10192	-5.92E-05	0.96291	0.995399	None
20947	PKI55	0.008548	0.962917	0.995399	None
20948	KREMEN1	-0.00136	0.962997	0.995399	None
20949	HAPLN2	0.000878	0.96301	0.995399	None
20950	BPY2	-0.00118	0.963026	0.995399	None
20951	RARRES2	-0.0103	0.963108	0.995399	None
20952	EPHA6	9.55E-05	0.963178	0.995399	None
20953	ECM2	0.003457	0.963197	0.995399	None
20954	C6orf52	-0.00104	0.963199	0.995399	None
20955	CREB1	-0.00692	0.963264	0.995399	None
20956	LARP1B	0.005196	0.963269	0.995399	None
20957	DCAF8	-0.00525	0.963365	0.995411	None
20958	ERAP2	0.020357	0.963372	0.995411	None
20959	FOXK2	0.005641	0.963536	0.995532	None
20960	KIAA1841	0.005073	0.963621	0.995573	None
20961	MYO5A	0.007315	0.963723	0.995603	None
20962	C5AR1	0.020521	0.963742	0.995603	None
20963	ZNF135	-0.00453	0.963801	0.995616	None
20964	BRINP3	0.000464	0.964077	0.995854	None
20965	LOC10192	0.001606	0.964157	0.99589	None
20966	SNTB2	-0.0004	0.964227	0.995914	None
20967	USP2-AS1	0.002492	0.964306	0.995948	None
20968	FLJ37786	-0.0006	0.964388	0.995976	None
20969	STK33	-0.00738	0.964425	0.995976	None
20970	LOC10192	-5.62E-05	0.964661	0.996113	None
20971	RP11-110	0.000742	0.964715	0.996113	None
20972	MGC5734	-0.00411	0.964772	0.996113	None
20973	HSD17B7P	-0.00194	0.964788	0.996113	None
20974	RAB3D	-0.00393	0.964885	0.996113	None
20975	TNFAIP8L3	-0.00052	0.964903	0.996113	None
20976	XRN1	0.008546	0.964923	0.996113	None
20977	ABCA1	0.010068	0.964926	0.996113	None
20978	ISOC1	0.012218	0.965071	0.996216	None
20979	GPR112	-0.00031	0.965143	0.996243	None
20980	KRT75	-5.50E-05	0.96533	0.996356	None
20981	RGS20	-0.00068	0.965353	0.996356	None
20982	LOC38990	-0.00865	0.965391	0.996356	None
20983	ANXA1	0.015327	0.965525	0.996408	None
20984	ACE2	0.001931	0.965533	0.996408	None
20985	SLC16A11	-0.00082	0.965725	0.996558	None
20986	GAR1	0.007024	0.96581	0.996599	None
20987	TMEM194	-0.0047	0.965924	0.996646	None
20988	LURAP1	-0.00236	0.966032	0.996646	None
20989	LOC61326	-0.00034	0.966061	0.996646	None
20990	STOML1	0.005061	0.966087	0.996646	None
20991	TPP2	-0.00824	0.966113	0.996646	None
20992	RP11-119F	-0.01083	0.966148	0.996646	None
20993	CKAP4	-0.01318	0.966178	0.996646	None
20994	CAMK4	-0.00036	0.966393	0.99682	None

20995	FOXA2	-5.32E-05	0.966461	0.996843	None
20996	BMX	0.009964	0.966548	0.996885	None
20997	KIAA2022	0.000573	0.96664	0.996933	None
20998	P2RX1	-0.00897	0.966695	0.996942	None
20999	KRTAP1-5	-5.27E-05	0.966766	0.996968	None
21000	CSRNP3	-5.24E-05	0.966887	0.996987	None
21001	SIX2	0.000403	0.966889	0.996987	None
21002	CLCA1	-0.00111	0.966923	0.996987	None
21003	EDA2R	0.002359	0.967036	0.997034	None
21004	LRRCC1	-0.00678	0.967061	0.997034	None
21005	CHORDC1	-0.0035	0.967125	0.997052	None
21006	FOXA1	-0.00054	0.967198	0.99708	None
21007	SEMG1	-5.20E-05	0.96725	0.997082	None
21008	OR2K2	0.000741	0.967334	0.997082	None
21009	GMFG	0.005242	0.967338	0.997082	None
21010	PRR19	0.002599	0.967405	0.997104	None
21011	CSTF1	-0.00479	0.967456	0.997109	None
21012	LOC64548!	-5.13E-05	0.967636	0.997247	None
21013	ARHGAP2{	-5.11E-05	0.967864	0.997394	None
21014	IDE	0.007396	0.96787	0.997394	None
21015	RP6-24A2:	-5.04E-05	0.968155	0.997574	None
21016	ZNF638	-0.00533	0.968161	0.997574	None
21017	LINC00906	0.000562	0.968184	0.997574	None
21018	SLC9A3	4.98E-05	0.968467	0.997772	None
21019	CACHD1	-0.00603	0.968487	0.997772	None
21020	LOC10272:	-0.00067	0.968568	0.997772	None
21021	FLJ22184	-4.99E-05	0.968642	0.997772	None
21022	KLK6	-4.98E-05	0.968652	0.997772	None
21023	FERMT2	0.004107	0.968653	0.997772	None
21024	TBX3	-4.97E-05	0.968731	0.997772	None
21025	TULP2	-0.00091	0.968744	0.997772	None
21026	PCLO	0.001214	0.968803	0.997785	None
21027	PLXNC1	0.004287	0.968903	0.99784	None
21028	LILRP2	-0.00133	0.96912	0.998017	None
21029	TMEM116	-0.00252	0.969185	0.998036	None
21030	LOC10192!	0.001056	0.969232	0.998037	None
21031	IQCH	-4.87E-05	0.969372	0.998064	None
21032	SKA3	0.009003	0.969385	0.998064	None
21033	TMEM100	0.001323	0.969397	0.998064	None
21034	FAM84A	-4.84E-05	0.969511	0.998104	None
21035	LOC10192{	-4.84E-05	0.969531	0.998104	None
21036	PNMA1	-0.00745	0.969582	0.998104	None
21037	ZNF365	0.000396	0.969619	0.998104	None
21038	LOC10099(	0.002832	0.969776	0.998182	None
21039	ZC3HAV1L	0.004727	0.969812	0.998182	None
21040	WLS	0.010708	0.969834	0.998182	None
21041	FAM230C	-4.76E-05	0.970035	0.998245	None
21042	NPPC	-4.76E-05	0.970048	0.998245	None
21043	CYP51A1-/	-4.75E-05	0.970108	0.998245	None
21044	MED6	-0.00556	0.970142	0.998245	None
21045	LGALS14	0.000553	0.970156	0.998245	None
21046	ZNF596	0.002405	0.970263	0.998245	None
21047	ATF7IP2	-0.00819	0.970266	0.998245	None
21048	WWOX	0.004493	0.970304	0.998245	None
21049	TNNI1	0.000479	0.97031	0.998245	None
21050	PDE9A	0.003816	0.970364	0.998253	None
21051	PLBD1	0.022238	0.970437	0.998264	None
21052	TREML2	0.001568	0.970466	0.998264	None

21053	ZBTB8A	-0.00825	0.970553	0.998287	None
21054	KRT6B	-0.00097	0.970648	0.998287	None
21055	RP6-99M1	0.011433	0.970713	0.998287	None
21056	GRIK4	0.000571	0.970717	0.998287	None
21057	RPN2	-0.00379	0.970748	0.998287	None
21058	FSD1	-0.00238	0.970782	0.998287	None
21059	ZFHX4-AS	-0.00144	0.970811	0.998287	None
21060	PFDN1	0.004421	0.970909	0.998339	None
21061	C9orf135-/	0.000924	0.97107	0.998455	None
21062	MMP27	0.000948	0.971125	0.998455	None
21063	KIF9-AS1	-0.00143	0.971159	0.998455	None
21064	EPHA2	-0.00092	0.971248	0.998499	None
21065	EPM2A	0.004919	0.971322	0.998528	None
21066	IQCF1	-4.55E-05	0.971391	0.99855	None
21067	LRIT1	-4.49E-05	0.971625	0.998737	None
21068	LOC72791(	0.001119	0.971707	0.998737	None
21069	LOC39070!	-0.00163	0.971711	0.998737	None
21070	ZP1	-4.47E-05	0.971854	0.998819	None
21071	MYL1	9.53E-05	0.971882	0.998819	None
21072	SLC25A16	-0.00383	0.971935	0.998826	None
21073	GAFA1	-8.83E-05	0.971983	0.998828	None
21074	LOC10192	-4.40E-05	0.972312	0.999012	None
21075	GLT1D1	0.007805	0.972324	0.999012	None
21076	ABCC8	0.000283	0.972341	0.999012	None
21077	LOC72809!	-0.00132	0.972347	0.999012	None
21078	DENND4C	-0.0063	0.972744	0.99936	None
21079	TTL	0.00288	0.972778	0.99936	None
21080	CCDC160	0.000491	0.972988	0.999529	None
21081	GYLTL1B	0.002355	0.973127	0.999594	None
21082	TMEM14A	0.006092	0.973147	0.999594	None
21083	ZNF71	-0.00068	0.97319	0.999594	None
21084	RPS29	-0.00331	0.973342	0.99961	None
21085	LINC01003	-0.00663	0.973387	0.99961	None
21086	OASL	0.008494	0.973555	0.99961	None
21087	PLA2G2D	0.00129	0.973589	0.99961	None
21088	MMGT1	0.003749	0.973625	0.99961	None
21089	BVES-AS1	-4.18E-05	0.973639	0.99961	None
21090	SLIT1	0.001108	0.97368	0.99961	None
21091	HJURP	0.00802	0.973682	0.99961	None
21092	LOC10272	-4.17E-05	0.973717	0.99961	None
21093	PTPRM	0.012451	0.973724	0.99961	None
21094	AP1B1	0.004888	0.973794	0.99961	None
21095	LINC00163	-0.0004	0.973813	0.99961	None
21096	SIX3-AS1	0.000769	0.97389	0.99961	None
21097	LINC00845	-4.14E-05	0.973895	0.99961	None
21098	HOTTIP	-0.00081	0.97399	0.99961	None
21099	LOC10013	-0.00545	0.973998	0.99961	None
21100	SLC51A	-0.00038	0.974041	0.99961	None
21101	LINC01127	-4.12E-05	0.974041	0.99961	None
21102	STARD13	-0.0022	0.974124	0.99961	None
21103	MPP2	-0.0009	0.974129	0.99961	None
21104	ARHGAP2	-0.00036	0.97425	0.999686	None
21105	RANBP1	0.003275	0.9744	0.999794	None
21106	SLC1A7	-0.00042	0.974531	0.999853	None
21107	PPP1R14D	-0.00083	0.974586	0.999853	None
21108	LOC10192	-4.03E-05	0.974597	0.999853	None
21109	TMEM106(	0.002825	0.974733	0.999943	None
21110	RIPPLY1	0.000837	0.974811	0.999943	None

21111	LOC10192	-3.99E-05	0.974823	0.999943	None
21112	IL17RB	-3.97E-05	0.974987	0.999968	None
21113	BCAP31	0.003793	0.974994	0.999968	None
21114	GPR45	-0.00052	0.975015	0.999968	None
21115	ZNF324	0.005585	0.975041	0.999968	None
21116	SIX4	-0.00038	0.975078	0.999968	None
21117	MAPK4	-3.94E-05	0.975138	0.999982	None
21118	POLR3E	-3.93E-05	0.975198	0.999996	None
21119	ORM1	0.004553	0.975315	1	None
21120	QPCT	0.011844	0.975325	1	None
21121	ARIH2	-0.00449	0.975521	1	None
21122	KDM6B	0.00622	0.97554	1	None
21123	RRAD	-0.00427	0.975682	1	None
21124	PTPN22	-0.00483	0.97579	1	None
21125	HERC5	0.006705	0.975797	1	None
21126	DHX35	0.001732	0.975806	1	None
21127	CCR9	-0.00245	0.975873	1	None
21128	STPG1	-0.00141	0.975989	1	None
21129	C1GALT1C	0.002907	0.976045	1	None
21130	EHD1	0.003235	0.97618	1	None
21131	LOC10028	-3.77E-05	0.976233	1	None
21132	SGK223	0.010961	0.976314	1	None
21133	ACRV1	-3.74E-05	0.976441	1	None
21134	ADPRHL2	-0.00547	0.976506	1	None
21135	FNBP1	0.005575	0.976531	1	None
21136	RP11-77H	0.000685	0.976578	1	None
21137	RDH8	0.000648	0.976599	1	None
21138	DIRC1	-0.00032	0.976861	1	None
21139	UGT2B4	-3.62E-05	0.977106	1	None
21140	CST9L	-3.62E-05	0.977146	1	None
21141	AC010524.	-0.00075	0.977168	1	None
21142	NWD2	-3.62E-05	0.977186	1	None
21143	PAICS	0.004499	0.977204	1	None
21144	OR2B3	-3.59E-05	0.977342	1	None
21145	GREM2	-3.58E-05	0.977424	1	None
21146	PYY	0.00017	0.977451	1	None
21147	PLEKHH3	0.000478	0.97747	1	None
21148	AURKA	0.008291	0.977536	1	None
21149	SOX18	-3.56E-05	0.977557	1	None
21150	LOC10050	-3.55E-05	0.977579	1	None
21151	PSMD9	0.0028	0.977673	1	None
21152	NRCAM	-0.00025	0.977679	1	None
21153	TINAG	-3.53E-05	0.977731	1	None
21154	EPB41L4B	-3.51E-05	0.977821	1	None
21155	SOX2	-3.50E-05	0.977891	1	None
21156	RNASEH2E	0.004121	0.977899	1	None
21157	FSTL1	0.005469	0.977923	1	None
21158	CCDC17	-0.00072	0.977981	1	None
21159	ZNF597	-0.00228	0.978029	1	None
21160	RUFY2	-0.00137	0.978057	1	None
21161	LOC10013	-3.47E-05	0.978082	1	None
21162	ACTL7B	-0.00058	0.978172	1	None
21163	TGFB2	-3.43E-05	0.978315	1	None
21164	FOXL1	0.000571	0.978315	1	None
21165	FUZ	-0.00145	0.97833	1	None
21166	RP11-471E	0.001492	0.978394	1	None
21167	LINC00488	0.000281	0.978429	1	None
21168	GGNBP2	-0.00362	0.978497	1	None

21169	LOC10026‹	-3.40E-05	0.97855	1	None
21170	METAP1	-0.00216	0.978583	1	None
21171	CARS-AS1	-3.38E-05	0.978639	1	None
21172	LOC10012‹	-0.00478	0.978664	1	None
21173	TMEM14B	-0.00379	0.97873	1	None
21174	STAC3	0.003839	0.97874	1	None
21175	GJD3	-0.00029	0.978766	1	None
21176	EDNRA	-3.36E-05	0.978809	1	None
21177	NCAM2	-0.00015	0.978816	1	None
21178	C2orf81	0.002295	0.978946	1	None
21179	GPR150	-0.00047	0.97923	1	None
21180	ATP8A2	-0.00129	0.979257	1	None
21181	BC045788	0.000834	0.979269	1	None
21182	HES2	-0.00042	0.979288	1	None
21183	HRG	-0.00042	0.979559	1	None
21184	PVRL3	-0.00049	0.979608	1	None
21185	DNAJC9-A	-3.22E-05	0.979634	1	None
21186	DENR	-0.00345	0.979655	1	None
21187	RP11-201‹	0.000446	0.979805	1	None
21188	LOC10192‹	8.99E-05	0.97988	1	None
21189	C6	0.00079	0.979896	1	None
21190	SNRNP200	-0.0047	0.980016	1	None
21191	CYP2U1	-0.00263	0.980083	1	None
21192	NKX2-1	0.000197	0.980089	1	None
21193	TPTEP1	-0.00409	0.980289	1	None
21194	LCA5	0.004311	0.980323	1	None
21195	RNF113A	0.003341	0.980382	1	None
21196	FCAR	0.002207	0.980798	1	None
21197	SYCE1	0.001169	0.980849	1	None
21198	PNN	-0.00239	0.980886	1	None
21199	PTX3	0.011782	0.980974	1	None
21200	ZNF329	0.003041	0.981108	1	None
21201	LOC10192‹	-2.95E-05	0.981346	1	None
21202	LOC14688‹	0.000871	0.981363	1	None
21203	HRC	-2.94E-05	0.981445	1	None
21204	PSMD2	-0.00252	0.981486	1	None
21205	CALB1	0.001389	0.981548	1	None
21206	ACTRT2	0.000395	0.981896	1	None
21207	ASB4	-2.85E-05	0.981968	1	None
21208	PHB2	0.003361	0.981985	1	None
21209	SMC4	0.00403	0.982001	1	None
21210	CNTRL	0.003663	0.982067	1	None
21211	GNL3	-0.00344	0.982086	1	None
21212	CSRP2	0.00557	0.982108	1	None
21213	MASP1	7.21E-05	0.982243	1	None
21214	ADAMTSL‹	0.000395	0.982358	1	None
21215	GTF2IRD2E	0.000767	0.982385	1	None
21216	MUSK	-2.78E-05	0.982415	1	None
21217	SPHKAP	-2.77E-05	0.982488	1	None
21218	TGIF2	-0.00336	0.982529	1	None
21219	LIX1	-3.10E-05	0.982534	1	None
21220	TPD52	0.004086	0.982539	1	None
21221	TMEM50B	0.00235	0.982603	1	None
21222	RP11-118‹	0.000569	0.982754	1	None
21223	LOC10192‹	-2.72E-05	0.982767	1	None
21224	CDHR2	-0.00081	0.982781	1	None
21225	LOC40056‹	0.000385	0.982867	1	None
21226	RP5-1154L	0.000258	0.982884	1	None

21227	MAP3K1	0.003943	0.982902	1	None
21228	HLA-C	-0.00279	0.982961	1	None
21229	IFT57	0.002447	0.983202	1	None
21230	ZBTB26	-0.00298	0.983207	1	None
21231	LOC10050!	-0.00491	0.983213	1	None
21232	GP5	-0.00386	0.98323	1	None
21233	MUC16	-2.65E-05	0.983254	1	None
21234	ANKRD44-	0.000656	0.983345	1	None
21235	PLXNA4	-0.00088	0.98336	1	None
21236	CBLN4	0.000184	0.983383	1	None
21237	LYVE1	-0.00846	0.983419	1	None
21238	SRSF10	-0.00316	0.983542	1	None
21239	NXT2	0.002588	0.983545	1	None
21240	RIBC1	0.000517	0.98358	1	None
21241	EXOSC9	0.002418	0.983595	1	None
21242	SNX22	-0.00175	0.983607	1	None
21243	LOC72794	-0.00074	0.98366	1	None
21244	PRNT	-2.58E-05	0.983663	1	None
21245	ACOT6	-2.58E-05	0.983701	1	None
21246	MYOZ2	-2.55E-05	0.983864	1	None
21247	ANKRD33E	0.004687	0.983866	1	None
21248	TTC38	0.000792	0.984104	1	None
21249	RAB30	-0.00304	0.984129	1	None
21250	VPS25	-0.00268	0.984265	1	None
21251	EFNA5	0.000239	0.984353	1	None
21252	ZNF469	-0.00305	0.984698	1	None
21253	OR5E1P	-2.41E-05	0.984744	1	None
21254	SAP30BP	-0.00196	0.984871	1	None
21255	ITPRIP	-0.00226	0.984903	1	None
21256	LTA	-0.00069	0.984913	1	None
21257	ABCC12	-2.38E-05	0.984929	1	None
21258	LOC10013	-2.38E-05	0.984934	1	None
21259	BPIFB2	-0.00051	0.985017	1	None
21260	LOC10192	-2.35E-05	0.985128	1	None
21261	FAM20A	-0.00254	0.985184	1	None
21262	MGC5072	0.000717	0.985198	1	None
21263	EBF2	-2.34E-05	0.985213	1	None
21264	TPH2	-2.34E-05	0.985225	1	None
21265	SERPINA6	-2.31E-05	0.985375	1	None
21266	GCG	-2.30E-05	0.985434	1	None
21267	TSR1	-0.0029	0.98547	1	None
21268	ERI3	0.003412	0.985686	1	None
21269	UBE2D4	0.001765	0.98599	1	None
21270	TMOD3	0.002453	0.986016	1	None
21271	OR10A3	-0.00017	0.986033	1	None
21272	ATP7A	0.002595	0.98611	1	None
21273	LOC10192	-0.00064	0.986164	1	None
21274	KB-1568E2	-2.19E-05	0.986164	1	None
21275	RP11-483(	-2.17E-05	0.986259	1	None
21276	DNAL4	0.001636	0.986292	1	None
21277	GNMT	-0.0016	0.986335	1	None
21278	GRIN2A	-2.14E-05	0.986468	1	None
21279	TAS2R5	-0.00088	0.986511	1	None
21280	PINLYP	-0.00359	0.986569	1	None
21281	RP11-274E	7.67E-05	0.986681	1	None
21282	TREH	6.74E-05	0.986692	1	None
21283	AGBL4	-0.00019	0.986747	1	None
21284	RP11-157F	-0.00041	0.986859	1	None

21285	GNRHR	-2.07E-05	0.986937	1	None
21286	CACNA1S	0.000617	0.986991	1	None
21287	RP11-410L	0.002186	0.987054	1	None
21288	KIF20A	-0.00484	0.987063	1	None
21289	CYLC2	-2.03E-05	0.987154	1	None
21290	IFNL2	0.00014	0.987159	1	None
21291	TIMM21	-0.00227	0.987277	1	None
21292	NDUFB2	0.002609	0.987295	1	None
21293	LOC10050!	-0.00368	0.987297	1	None
21294	OR8B2	-2.01E-05	0.987306	1	None
21295	RP11-586K	-7.24E-05	0.987346	1	None
21296	LOC10012!	0.000314	0.987368	1	None
21297	RP5-1102E	0.000406	0.987519	1	None
21298	MRFAP1	-0.00114	0.987592	1	None
21299	ARF6	-0.00176	0.987607	1	None
21300	FASTKD2	0.003067	0.987644	1	None
21301	MOBP	-1.94E-05	0.987699	1	None
21302	LOC10192!	0.000216	0.987777	1	None
21303	ASB10	-1.93E-05	0.987781	1	None
21304	PCDH7	-1.93E-05	0.987807	1	None
21305	HKR1	-1.93E-05	0.987815	1	None
21306	CLOCK	-0.00148	0.987917	1	None
21307	SWT1	0.002277	0.988021	1	None
21308	ADCY1	-0.00017	0.988037	1	None
21309	NMBR	-1.88E-05	0.988102	1	None
21310	LOC28426:	-1.87E-05	0.988182	1	None
21311	FUT9	-1.86E-05	0.988231	1	None
21312	BTLA	-0.00316	0.988283	1	None
21313	SH3GL1P2	-1.85E-05	0.988292	1	None
21314	ATP2C1	0.001662	0.988335	1	None
21315	TSPYL1	0.001578	0.988336	1	None
21316	IGF2R	-0.00453	0.988341	1	None
21317	LOC14594!	-0.00056	0.988396	1	None
21318	ASCL1	-1.81E-05	0.988535	1	None
21319	LOC10192!	-1.85E-05	0.988552	1	None
21320	RAX2	0.000398	0.988622	1	None
21321	LOC10099(	-1.79E-05	0.98865	1	None
21322	FOXR1	-1.79E-05	0.988698	1	None
21323	NRK	0.000171	0.988926	1	None
21324	RUNDC3B	-0.00231	0.989046	1	None
21325	COPE	0.001776	0.989172	1	None
21326	GC	-1.70E-05	0.989239	1	None
21327	ZNF853	0.000757	0.989271	1	None
21328	KIF18A	0.003088	0.989294	1	None
21329	SOST	-1.68E-05	0.989355	1	None
21330	SYT16	0.000134	0.989364	1	None
21331	AVPR1B	-0.00031	0.989416	1	None
21332	HS6ST1	-0.00027	0.989429	1	None
21333	IL18R1	-0.00375	0.98946	1	None
21334	IL2RG	0.003471	0.989588	1	None
21335	DKFZp451I	-1.64E-05	0.989594	1	None
21336	COL1A2	0.002727	0.989595	1	None
21337	KRTAP5-2	-1.64E-05	0.989595	1	None
21338	DEFA5	-1.63E-05	0.989713	1	None
21339	OXTR	-0.00212	0.989761	1	None
21340	SDC4	0.003532	0.98987	1	None
21341	RP11-435(	-0.00061	0.990029	1	None
21342	CRYGC	-0.00015	0.990164	1	None

21343	PRB1	0.001169	0.990185	1	None
21344	GLIPR1	-0.0036	0.990215	1	None
21345	C8orf60	-0.00391	0.990242	1	None
21346	HSD17B2	-1.54E-05	0.990266	1	None
21347	AFAP1-AS	0.000365	0.990348	1	None
21348	RNF148	-1.50E-05	0.990509	1	None
21349	BCL11A	0.002505	0.990549	1	None
21350	NPC1L1	-1.44E-05	0.990866	1	None
21351	ASCL5	-1.43E-05	0.990953	1	None
21352	GHSR	-1.42E-05	0.991089	1	None
21353	THEM5	-2.61E-05	0.991098	1	None
21354	EFCAB1	-1.40E-05	0.991125	1	None
21355	MAPT-AS1	0.000438	0.991195	1	None
21356	RP11-470I	-1.38E-05	0.991296	1	None
21357	LINC00909	0.001383	0.991357	1	None
21358	PNRC1	-0.00163	0.991418	1	None
21359	ADAM7	-1.35E-05	0.991435	1	None
21360	PRKY	0.003388	0.991496	1	None
21361	ZNF771	-1.34E-05	0.991511	1	None
21362	FRZB	0.000932	0.991523	1	None
21363	GOLGA8A	-0.00252	0.991539	1	None
21364	YWHAH	-0.00231	0.991623	1	None
21365	SRA1	0.002036	0.991674	1	None
21366	SPC24	0.001401	0.991737	1	None
21367	SLC2A1-A	0.000237	0.991768	1	None
21368	CEP290	0.00156	0.991781	1	None
21369	RTBDN	9.08E-05	0.991889	1	None
21370	WDR72	-1.28E-05	0.99189	1	None
21371	OLFM1	0.000427	0.991908	1	None
21372	RUNX3	-0.0027	0.992039	1	None
21373	CLEC1A	0.000924	0.992106	1	None
21374	PWWP2B	-0.00072	0.992106	1	None
21375	LINC01204	6.41E-05	0.992178	1	None
21376	FBXL21	-1.23E-05	0.992237	1	None
21377	PMS2P8	0.001501	0.992407	1	None
21378	CAPRIN1	0.000942	0.992453	1	None
21379	EVI2B	-0.00175	0.992486	1	None
21380	EPCAM	-0.00653	0.992502	1	None
21381	LOC65316	-0.00013	0.99252	1	None
21382	ENOX1	0.000809	0.992547	1	None
21383	COL28A1	0.000253	0.99258	1	None
21384	SOX30	0.000168	0.992749	1	None
21385	LSM14B	-0.00034	0.992797	1	None
21386	RP11-710	-1.13E-05	0.992816	1	None
21387	CPSF2	-0.00116	0.992843	1	None
21388	S100G	1.14E-05	0.992847	1	None
21389	SIX1	-1.12E-05	0.992887	1	None
21390	CACNA1E	-1.12E-05	0.992922	1	None
21391	DCC	-1.11E-05	0.992995	1	None
21392	KLHL2	-0.00127	0.993106	1	None
21393	OR4C1P	-1.09E-05	0.993115	1	None
21394	LINC00261	-1.08E-05	0.993135	1	None
21395	RPS15A	0.001888	0.993171	1	None
21396	CADPS	-1.08E-05	0.993184	1	None
21397	GMCL1	-0.00141	0.993218	1	None
21398	EYA4	-1.06E-05	0.993306	1	None
21399	C11orf40	-1.04E-05	0.993405	1	None
21400	SUN3	-0.00045	0.993444	1	None

21401	NR2F1	0.003512	0.993518	1	None
21402	CNPY4	0.000685	0.993552	1	None
21403	CEACAM7	-1.01E-05	0.993589	1	None
21404	RP11-521(	-1.01E-05	0.993597	1	None
21405	AANAT	7.27E-05	0.993617	1	None
21406	H2AFX	0.000559	0.993703	1	None
21407	ITPKB-IT1	-0.0004	0.993752	1	None
21408	KLKB1	0.000443	0.993826	1	None
21409	SEMA6B	-0.00028	0.993836	1	None
21410	WWTR1-A	-9.72E-06	0.993844	1	None
21411	USP27X	-0.00052	0.993925	1	None
21412	RGS12	-0.00014	0.993946	1	None
21413	TMPRSS6	0.000244	0.994177	1	None
21414	RAB36	0.000103	0.994208	1	None
21415	LOC10192!	-0.00035	0.994223	1	None
21416	ZIC1	-9.07E-06	0.994259	1	None
21417	LPAR3	0.000241	0.994361	1	None
21418	CRYGEP	0.000685	0.994381	1	None
21419	CMPK2	-0.00156	0.994437	1	None
21420	FBXO31	0.000959	0.994469	1	None
21421	VSIG1	-2.15E-05	0.994495	1	None
21422	SLC35E3	-0.0005	0.994516	1	None
21423	SAMHD1	-0.00303	0.994535	1	None
21424	ITGAE	-0.0009	0.994535	1	None
21425	EPHA3	-8.59E-06	0.994563	1	None
21426	RP11-31K2	5.68E-05	0.994589	1	None
21427	PLEKHB1	0.000136	0.994701	1	None
21428	PNMA2	0.000217	0.994717	1	None
21429	FOXN3-AS	-0.00035	0.994761	1	None
21430	ZHX3	0.000382	0.995013	1	None
21431	TPT1P8	-0.00046	0.995098	1	None
21432	NUDC	-0.00092	0.995104	1	None
21433	CARNS1	5.83E-05	0.995113	1	None
21434	RP11-214N	7.67E-06	0.995151	1	None
21435	KCNH4	9.04E-05	0.995241	1	None
21436	AF279780	-5.88E-05	0.995298	1	None
21437	PLGLB2	5.41E-05	0.995529	1	None
21438	LOC10192	8.95E-05	0.995536	1	None
21439	LOC10192!	0.000183	0.995608	1	None
21440	UGT2B15	-9.46E-05	0.995614	1	None
21441	SLC23A2	-0.00061	0.995645	1	None
21442	POLR3G	0.000404	0.995673	1	None
21443	FAM208A	0.000571	0.995717	1	None
21444	FAM170A	-3.55E-05	0.995853	1	None
21445	RP4-595K1	0.000934	0.995856	1	None
21446	HIST1H2B/	-6.28E-06	0.996021	1	None
21447	LYPD8	-0.00013	0.996141	1	None
21448	ERO1L	-0.00068	0.996382	1	None
21449	IMPG1	-5.69E-06	0.996394	1	None
21450	LINC00657	-0.00067	0.996424	1	None
21451	ZNF625	0.000226	0.9966	1	None
21452	RCBTB1	0.000389	0.996631	1	None
21453	PSMB3	-0.0006	0.996635	1	None
21454	RP11-330(	0.000261	0.996635	1	None
21455	NXPH2	-5.20E-06	0.996706	1	None
21456	LINC00673	3.13E-05	0.996762	1	None
21457	LOC10334	0.000332	0.996852	1	None
21458	BDNF	0.000742	0.996931	1	None

21459	GMNC	-4.67E-06	0.997041	1	None
21460	ILVBL	0.000667	0.997045	1	None
21461	SSX2B	2.40E-05	0.997055	1	None
21462	TM6SF2	-8.36E-05	0.997059	1	None
21463	CCDC144A	-5.13E-05	0.997148	1	None
21464	OR10A4	3.24E-05	0.997271	1	None
21465	KCNQ2	-4.25E-06	0.997306	1	None
21466	SVEP1	-4.24E-06	0.997316	1	None
21467	LOC10272	-4.18E-06	0.997354	1	None
21468	PCDHGB8I	-3.79E-06	0.997598	1	None
21469	CPVL	-0.00095	0.997769	1	None
21470	BBX	0.000306	0.997855	1	None
21471	AC005592.	-3.33E-06	0.997893	1	None
21472	LINC0118C	3.21E-05	0.997893	1	None
21473	FAM154B	-0.00047	0.997917	1	None
21474	GRIA4	-3.28E-06	0.997921	1	None
21475	LOC10192	-3.27E-06	0.997929	1	None
21476	CHRM3	0.000662	0.997934	1	None
21477	MLPH	-8.08E-05	0.997971	1	None
21478	CSN1S2AP	-4.57E-05	0.997979	1	None
21479	LOC10192	-3.15E-06	0.99801	1	None
21480	MC3R	1.41E-05	0.998245	1	None
21481	ANKRD26	0.000256	0.998321	1	None
21482	CHRNA9	-6.32E-05	0.998359	1	None
21483	MRPL23	-0.00022	0.998416	1	None
21484	OR2L2	-2.44E-06	0.998454	1	None
21485	LOC72804	2.49E-05	0.998466	1	None
21486	LHFP	0.000385	0.998505	1	None
21487	ANP32E	-0.00034	0.998512	1	None
21488	CLDN11	-2.34E-06	0.998518	1	None
21489	CHTF18	-0.00029	0.998544	1	None
21490	LINC00607	-7.01E-06	0.998598	1	None
21491	PPP1R3B	-0.00014	0.998601	1	None
21492	LOC10192	-2.02E-06	0.998718	1	None
21493	RP11-111k	2.08E-05	0.998747	1	None
21494	SERPINB13	-1.94E-06	0.99877	1	None
21495	S1PR5	-1.91E-06	0.998791	1	None
21496	CNTF	-1.91E-06	0.998792	1	None
21497	MYO18B	3.57E-05	0.998802	1	None
21498	BC034444	-1.89E-06	0.998806	1	None
21499	ZNF804B	-1.56E-06	0.999014	1	None
21500	ERLEC1	-0.00012	0.999121	1	None
21501	HOXC-AS3	-1.31E-06	0.999172	1	None
21502	CCDC177	-1.27E-06	0.999198	1	None
21503	RP11-109D	-1.26E-06	0.999204	1	None
21504	PHGR1	1.52E-05	0.999207	1	None
21505	ABCC9	-1.22E-06	0.999229	1	None
21506	TMEM120A	-0.00015	0.99924	1	None
21507	APOC2	2.69E-05	0.999251	1	None
21508	LQFBS-1	-1.31E-05	0.999342	1	None
21509	TPTE	2.41E-06	0.999349	1	None
21510	ATP1B4	-9.92E-06	0.999363	1	None
21511	LINC01125	-7.14E-05	0.9994	1	None
21512	CHRAC1	-9.14E-05	0.999463	1	None
21513	TSPAN15	-5.84E-05	0.999488	1	None
21514	NEB	3.48E-05	0.999489	1	None
21515	KCNMB2	1.70E-05	0.999493	1	None
21516	LOC10050	4.48E-05	0.999498	1	None

21517	AK054988	1.28E-05	0.999524	1	None
21518	KRTAP9-4	-6.76E-07	0.999572	1	None
21519	MIR1244-3	-5.00E-05	0.999603	1	None
21520	RASIP1	-1.68E-05	0.999764	1	None
21521	NDRG4	-4.17E-06	0.999782	1	None
21522	LOC72886	-3.00E-07	0.99981	1	None
21523	LOC10013	-1.05E-05	0.999841	1	None
21524	DEFB125	-2.29E-07	0.999855	1	None
21525	KRTDAP	-2.17E-07	0.999862	1	None
21526	JAZF1-AS1	-1.72E-07	0.999891	1	None
21527	OR7D2	-1.97E-05	0.999897	1	None
21528	LINC00307	-1.20E-07	0.999924	1	None
21529	FLJ34503	-1.19E-07	0.999924	1	None
21530	OFCC1	-4.41E-08	0.999972	1	None
21531	SMG8	-3.78E-06	0.99998	1	None
21532	BC034416	-1.62E-08	0.99999	1	None
21533	FAM46D	6.30E-16	1	1	None
21534	CRB2	6.30E-16	1	1	None
21535	BTBD16	6.30E-16	1	1	None
21536	PXT1	6.30E-16	1	1	None
21537	HTR3C	6.30E-16	1	1	None
21538	TAAR9	6.30E-16	1	1	None
21539	UTS2R	6.30E-16	1	1	None
21540	OXGR1	6.30E-16	1	1	None
21541	FLJ13744	6.30E-16	1	1	None
21542	CSNK1G2-	6.30E-16	1	1	None
21543	MRGPRX2	6.30E-16	1	1	None
21544	PLA2G4D	6.30E-16	1	1	None
21545	LOC28303	6.30E-16	1	1	None
21546	LOC10050	6.30E-16	1	1	None
21547	AC067956.	6.30E-16	1	1	None
21548	EHMT1-IT	6.30E-16	1	1	None
21549	BC039122	6.30E-16	1	1	None
21550	LOC72808	6.30E-16	1	1	None
21551	RP11-495K	6.30E-16	1	1	None
21552	JAKMIP2-A	6.30E-16	1	1	None
21553	LOC10192	6.30E-16	1	1	None
21554	RP11-95H	6.30E-16	1	1	None
21555	LOC10192	6.30E-16	1	1	None
21556	LOC10192	6.30E-16	1	1	None
21557	LOC33968	6.30E-16	1	1	None
21558	LOC10192	6.30E-16	1	1	None
21559	LOC10192	6.30E-16	1	1	None
21560	LOC10192	6.30E-16	1	1	None
21561	NKX1-1	6.30E-16	1	1	None
21562	LOC10192	6.30E-16	1	1	None
21563	LOC10272	6.30E-16	1	1	None
21564	LOC10042	6.30E-16	1	1	None
21565	LOC10192	6.30E-16	1	1	None
21566	SCGB1A1	6.30E-16	1	1	None
21567	LGI1	6.30E-16	1	1	None
21568	HSPB3	6.30E-16	1	1	None
21569	NTSR2	6.30E-16	1	1	None
21570	HAVCR1	6.30E-16	1	1	None
21571	BTN1A1	6.30E-16	1	1	None
21572	SCGB1D1	6.30E-16	1	1	None
21573	IFNA14	6.30E-16	1	1	None
21574	C8orf17	6.30E-16	1	1	None

21575	IFNA16	6.30E-16	1	1	None
21576	KCNG2	6.30E-16	1	1	None
21577	MYL2	6.30E-16	1	1	None
21578	IFNA5	6.30E-16	1	1	None
21579	LOC10192	6.30E-16	1	1	None
21580	C19orf80	6.30E-16	1	1	None
21581	CST8	6.30E-16	1	1	None
21582	THEG	6.30E-16	1	1	None
21583	RXFP3	6.30E-16	1	1	None
21584	LOC10050	6.30E-16	1	1	None
21585	OMP	6.30E-16	1	1	None
21586	TAS2R16	6.30E-16	1	1	None
21587	RP11-245F	6.30E-16	1	1	None
21588	DAPK3	6.30E-16	1	1	None
21589	GLTPD2	6.30E-16	1	1	None
21590	TMCO2	6.30E-16	1	1	None
21591	KRT71	6.30E-16	1	1	None
21592	RNASE11	6.30E-16	1	1	None
21593	CASP14	6.30E-16	1	1	None
21594	HOXC9	6.30E-16	1	1	None
21595	KIAA1875	6.30E-16	1	1	None
21596	RP11-495F	6.30E-16	1	1	None
21597	ABHD16B	6.30E-16	1	1	None
21598	OPN4	6.30E-16	1	1	None
21599	OR51M1	6.30E-16	1	1	None
21600	LOC10192	6.30E-16	1	1	None
21601	LOC10272	6.30E-16	1	1	None
21602	AP001630.	6.30E-16	1	1	None
21603	CTD-2281	6.30E-16	1	1	None
21604	LOC10013	6.30E-16	1	1	None
21605	CCDC54	6.30E-16	1	1	None
21606	ADAMTSL	6.30E-16	1	1	None
21607	ALDOB	6.30E-16	1	1	None
21608	ANKS1B	6.30E-16	1	1	None
21609	AQP4	6.30E-16	1	1	None
21610	CASC2	6.30E-16	1	1	None
21611	CDC20B	6.30E-16	1	1	None
21612	CELF4	6.30E-16	1	1	None
21613	CLDN18	6.30E-16	1	1	None
21614	COL4A3	6.30E-16	1	1	None
21615	CYP19A1	6.30E-16	1	1	None
21616	DLX6	6.30E-16	1	1	None
21617	DNAH6	6.30E-16	1	1	None
21618	EQTN	6.30E-16	1	1	None
21619	ESRRB	6.30E-16	1	1	None
21620	FEZF2	6.30E-16	1	1	None
21621	FGF14	6.30E-16	1	1	None
21622	FOXP2	6.30E-16	1	1	None
21623	FRAS1	6.30E-16	1	1	None
21624	GAD2	6.30E-16	1	1	None
21625	GATA4	6.30E-16	1	1	None
21626	GHRHR	6.30E-16	1	1	None
21627	HOXD10	6.30E-16	1	1	None
21628	LAMB4	6.30E-16	1	1	None
21629	LHX9	6.30E-16	1	1	None
21630	LINC01191	6.30E-16	1	1	None
21631	LOC10050	6.30E-16	1	1	None
21632	LOC10192	6.30E-16	1	1	None

21633	NAV3	6.30E-16	1	1	None
21634	NFIA-AS2	6.30E-16	1	1	None
21635	NMNAT2	6.30E-16	1	1	None
21636	NPY2R	6.30E-16	1	1	None
21637	NR1H4	6.30E-16	1	1	None
21638	OR2H1	6.30E-16	1	1	None
21639	OR51E2	6.30E-16	1	1	None
21640	PARK2	6.30E-16	1	1	None
21641	PLN	6.30E-16	1	1	None
21642	PPFIA2	6.30E-16	1	1	None
21643	RAB3B	6.30E-16	1	1	None
21644	RARB	6.30E-16	1	1	None
21645	RNF144A-,	6.30E-16	1	1	None
21646	SFTPC	6.30E-16	1	1	None
21647	SOX5	6.30E-16	1	1	None
21648	THSD4	6.30E-16	1	1	None
21649	TMEM232	6.30E-16	1	1	None
21650	TP63	6.30E-16	1	1	None
21651	TREML4	6.30E-16	1	1	None
21652	TRIM31	6.30E-16	1	1	None
21653	TRIM42	6.30E-16	1	1	None
21654	TRPM3	6.30E-16	1	1	None
21655	UNC5C	6.30E-16	1	1	None

Table S2 The differentially expressed NRGs between MDS and health in GSE58831 dataset.

	x
1	TNFRSF1A
2	PPID
3	PLA2G4A
4	MLKL
5	IL1A
6	TNFSF10
7	FAS
8	JAK2
9	STAT1
10	STAT3
11	IRF9
12	USP21
13	BAX

Table S3 The DEGs between two clusters in GSE58831 dataset.

gene	log2FC	pvalue	adjusted_pvalue
IRF9	1.989874	5.46E-33	1.18E-28
IFI6	2.061466	2.46E-20	2.66E-16
STAT1	1.211882	1.29E-17	9.29E-14
OAS1	2.042707	3.02E-17	1.64E-13
RAB8A	0.910772	8.18E-17	3.54E-13
AP2A2	0.968797	4.08E-16	1.37E-12
C19orf66	0.921978	4.44E-16	1.37E-12
TNFSF10	1.328944	1.08E-15	2.93E-12
ISG15	1.651383	1.44E-15	3.47E-12
CHMP2A	0.714799	9.04E-15	1.96E-11
FAM50A	1.016034	1.39E-14	2.73E-11
RAB5C	1.31148	1.76E-14	3.17E-11
EIF2AK2	0.578596	1.98E-14	3.29E-11
BLOC1S2	0.677689	2.50E-14	3.87E-11
CEBPB	1.214422	6.18E-14	8.93E-11
FBXO6	1.153917	6.71E-14	9.08E-11
IFIT3	1.534844	1.07E-13	1.36E-10
ATP6V0C	1.020441	1.14E-13	1.37E-10
STAT3	0.888535	1.63E-13	1.86E-10
HIP1	0.728203	1.84E-13	1.99E-10
ARPC4	0.80503	2.04E-13	2.10E-10
LGALS9	0.850861	2.43E-13	2.39E-10
S100A11	1.874631	2.64E-13	2.42E-10
PFN1	1.628012	2.68E-13	2.42E-10
OAS3	0.795986	2.83E-13	2.45E-10
UBE2M	1.740582	3.11E-13	2.59E-10
COPG1	1.303609	4.16E-13	3.34E-10
ALKBH5	1.250027	4.52E-13	3.50E-10
MESDC1	0.866909	5.78E-13	4.31E-10
GLUD1	0.624871	5.97E-13	4.31E-10
NFYC	0.86421	6.75E-13	4.71E-10
CORO1B	0.905236	7.38E-13	4.92E-10
TSPAN4	1.015017	7.49E-13	4.92E-10
IFI27	2.576917	8.06E-13	5.01E-10
EPB41L2	1.527955	8.10E-13	5.01E-10
OSTF1	1.001406	8.61E-13	5.18E-10
UAP1L1	0.835346	9.35E-13	5.34E-10
RAD23A	0.862092	9.37E-13	5.34E-10
GLTP	0.686509	9.87E-13	5.44E-10
ZNF106	0.58855	1.00E-12	5.44E-10
C1QC	2.762207	1.08E-12	5.58E-10
WDFY1	0.566671	1.08E-12	5.58E-10
RNF145	1.484673	1.91E-12	9.64E-10
RPL22	-0.28568	2.21E-12	1.08E-09
AES	1.003768	2.24E-12	1.08E-09
RHOA	1.264268	2.28E-12	1.08E-09
MX1	1.795564	2.62E-12	1.21E-09
SMIM14	1.162772	2.75E-12	1.22E-09
WSB2	0.713987	2.76E-12	1.22E-09
VEGFB	0.715728	2.90E-12	1.25E-09
TMED2	1.677333	3.32E-12	1.41E-09
ARF5	1.055428	3.67E-12	1.53E-09
NFIC	0.630027	3.95E-12	1.61E-09
HSPB1	1.354747	4.34E-12	1.74E-09
PARP9	0.799524	5.00E-12	1.96E-09
BLOC1S1	0.807748	5.08E-12	1.96E-09

SH3BP2	0.868244	6.53E-12	2.48E-09
CSTB	0.698143	6.87E-12	2.57E-09
IFITM3	0.914778	7.38E-12	2.68E-09
IFIT1	2.26312	7.44E-12	2.68E-09
PSMA7	0.599111	8.41E-12	2.98E-09
XAF1	1.05141	9.46E-12	3.31E-09
MLLT6	0.965506	1.02E-11	3.48E-09
RNF149	1.345319	1.03E-11	3.48E-09
OASL	1.042261	1.10E-11	3.61E-09
ZFAND3	0.538678	1.11E-11	3.61E-09
ABCF3	0.659038	1.12E-11	3.61E-09
GDE1	0.747818	1.25E-11	3.98E-09
ARL2BP	0.672757	1.38E-11	4.33E-09
ADRM1	1.154814	1.79E-11	5.53E-09
ERH	0.731685	1.90E-11	5.79E-09
CYSTM1	1.041556	2.39E-11	7.20E-09
LAP3	0.644067	2.67E-11	7.91E-09
CHIC2	1.16453	2.77E-11	8.12E-09
IFNGR2	0.697609	2.87E-11	8.30E-09
C14orf1	1.348308	3.37E-11	9.59E-09
NUP62	0.956846	3.43E-11	9.66E-09
COX16	0.862514	4.81E-11	1.33E-08
JAK1	0.753185	4.88E-11	1.34E-08
PICALM	0.675226	5.45E-11	1.48E-08
FCER1G	1.919069	5.60E-11	1.50E-08
VASP	0.955479	5.73E-11	1.51E-08
CAPZB	0.568383	7.05E-11	1.84E-08
EXOC6B	0.430733	7.65E-11	1.97E-08
SIPA1L2	0.983412	8.56E-11	2.18E-08
SH3GLB1	0.51773	8.97E-11	2.26E-08
RBMS1	0.594014	9.97E-11	2.48E-08
ATP6V0D1	0.815002	1.07E-10	2.64E-08
MAPK7	0.425578	1.10E-10	2.68E-08
RNF10	0.621762	1.11E-10	2.68E-08
FAM103A1	0.503634	1.23E-10	2.92E-08
SERTAD3	0.670345	1.37E-10	3.24E-08
IFI44L	2.193766	1.40E-10	3.25E-08
PPP1R18	1.0182	1.54E-10	3.54E-08
ATP6V1D	0.639379	1.57E-10	3.54E-08
RPLP0	-0.15022	1.58E-10	3.54E-08
GUK1	0.333172	1.60E-10	3.54E-08
YKT6	0.715972	1.60E-10	3.54E-08
ATP6V1A	0.593428	1.63E-10	3.57E-08
MYD88	0.438474	1.68E-10	3.63E-08
SKAP2	0.961622	1.73E-10	3.71E-08
STT3A	0.805397	1.76E-10	3.73E-08
ARPC5	0.605794	1.80E-10	3.78E-08
LOC10192	-0.42247	1.96E-10	4.07E-08
CARM1	0.93105	2.07E-10	4.26E-08
POR	0.878576	2.11E-10	4.30E-08
CXCL10	2.032342	2.38E-10	4.83E-08
NPC2	0.746496	2.50E-10	5.01E-08
RP4-781L3	0.662159	2.57E-10	5.10E-08
EIF5A	1.015115	2.65E-10	5.20E-08
HCFC1R1	0.411925	2.66E-10	5.20E-08
DYSF	1.4941	2.71E-10	5.25E-08
WDFY3	0.75176	2.76E-10	5.29E-08
PIAS2	0.607397	2.85E-10	5.39E-08

SP1	0.641104	2.86E-10	5.39E-08
HN1	0.664043	3.16E-10	5.90E-08
ZNFX1	0.696099	3.29E-10	6.08E-08
DDA1	0.481013	3.37E-10	6.19E-08
PPP3R1	1.028243	3.52E-10	6.41E-08
EHBP1L1	0.674761	3.74E-10	6.74E-08
CHMP4B	0.639617	3.77E-10	6.75E-08
ASCC2	0.706844	3.81E-10	6.77E-08
RBBP5	0.815167	4.32E-10	7.60E-08
TOR4A	0.653905	4.37E-10	7.63E-08
TMEM2	1.100259	4.43E-10	7.63E-08
AMFR	1.132273	4.44E-10	7.63E-08
RAB29	0.792133	4.58E-10	7.81E-08
SRSF11	-0.74196	4.64E-10	7.85E-08
ELMO1	1.119356	4.93E-10	8.27E-08
CIAO1	0.608553	5.20E-10	8.66E-08
PHF5A	0.851945	5.53E-10	9.15E-08
PLEKHO2	0.922509	5.79E-10	9.49E-08
MAP7D1	0.784243	6.90E-10	1.12E-07
OAS2	1.063687	6.95E-10	1.12E-07
RHOQ	0.832526	7.21E-10	1.15E-07
GAA	1.007394	7.21E-10	1.15E-07
LACTB	1.001354	7.45E-10	1.18E-07
SMUG1	0.339447	7.90E-10	1.24E-07
TYMP	0.572979	8.00E-10	1.25E-07
DUSP3	0.734034	8.36E-10	1.29E-07
COTL1	1.183742	8.84E-10	1.35E-07
AP2S1	0.715347	8.86E-10	1.35E-07
ATOX1	1.011521	8.91E-10	1.35E-07
PPDPF	0.743428	9.06E-10	1.36E-07
C1orf162	0.861242	9.16E-10	1.37E-07
GYPC	1.268354	9.24E-10	1.37E-07
SIGLEC1	1.460064	1.02E-09	1.49E-07
TRIM21	0.590381	1.02E-09	1.49E-07
ATP6V1B2	0.588673	1.03E-09	1.49E-07
RASSF4	0.428525	1.08E-09	1.55E-07
HPS1	0.464435	1.08E-09	1.55E-07
THEMIS2	1.008447	1.09E-09	1.55E-07
RALGAPB	0.45478	1.14E-09	1.61E-07
DPP9	0.489721	1.14E-09	1.61E-07
TIGD2	-0.55907	1.19E-09	1.65E-07
PIK3C2A	0.757952	1.19E-09	1.65E-07
VHL	0.483957	1.22E-09	1.68E-07
TXLNA	0.329538	1.22E-09	1.68E-07
VDAC1	1.137434	1.26E-09	1.71E-07
NAA60	0.535796	1.28E-09	1.73E-07
IGFBP2	1.38893	1.33E-09	1.79E-07
KDELR1	0.651032	1.35E-09	1.80E-07
THOC6	0.948317	1.38E-09	1.83E-07
SMARCC2	0.813479	1.42E-09	1.87E-07
GRIPAP1	0.533436	1.43E-09	1.87E-07
SDC3	1.042007	1.45E-09	1.87E-07
LINC00339	0.950002	1.45E-09	1.87E-07
DESI1	0.550908	1.45E-09	1.87E-07
IL15RA	0.748881	1.47E-09	1.89E-07
CRK	0.700882	1.48E-09	1.89E-07
NINJ1	1.384805	1.53E-09	1.94E-07
TMEM8A	0.389666	1.54E-09	1.94E-07

SNRNP40	1.132355	1.56E-09	1.95E-07
USF1	0.59969	1.58E-09	1.96E-07
CTSD	1.103808	1.61E-09	2.00E-07
DEK	-0.41619	1.64E-09	2.01E-07
ZNF655	0.769201	1.68E-09	2.05E-07
CIZ1	0.749679	1.70E-09	2.05E-07
HNRNPL	0.962272	1.71E-09	2.05E-07
DTX3L	0.516447	1.71E-09	2.05E-07
SLC2A4RG	0.368169	1.72E-09	2.05E-07
TXN2	0.679753	1.72E-09	2.05E-07
COX8A	0.650953	1.82E-09	2.15E-07
ATP5H	0.536073	1.91E-09	2.24E-07
LAMTOR1	0.679234	1.97E-09	2.29E-07
ISCU	0.595177	1.98E-09	2.29E-07
CHCHD10	0.97693	1.99E-09	2.29E-07
KLHL28	-1.02823	1.99E-09	2.29E-07
STIP1	0.798368	2.01E-09	2.30E-07
SRRM1	0.624546	2.08E-09	2.37E-07
KDM3B	0.678669	2.13E-09	2.42E-07
DLST	0.570473	2.15E-09	2.42E-07
MAGOH2	-0.36168	2.23E-09	2.50E-07
PEA15	0.528254	2.34E-09	2.61E-07
ZBED1	0.67747	2.54E-09	2.81E-07
NACC2	0.548929	2.55E-09	2.81E-07
RSAD2	1.163593	2.57E-09	2.82E-07
NDUFV2	0.826683	2.66E-09	2.91E-07
GAPVD1	0.649293	2.68E-09	2.91E-07
LTC4S	0.690859	2.69E-09	2.91E-07
HPCAL1	0.609354	2.74E-09	2.94E-07
SPR	0.609997	2.75E-09	2.94E-07
PDE6D	0.476003	2.75E-09	2.94E-07
LOC10050(	1.202672	2.84E-09	3.01E-07
CNIH1	-0.41948	2.88E-09	3.04E-07
RAB42	1.624949	2.92E-09	3.07E-07
GLUL	1.339327	2.94E-09	3.08E-07
NDUFA3	0.703884	2.99E-09	3.11E-07
BRMS1	0.591463	3.09E-09	3.21E-07
IFIT2	1.156904	3.15E-09	3.25E-07
STAM2	0.489692	3.17E-09	3.25E-07
CDC37	0.653682	3.26E-09	3.33E-07
RABL6	0.784728	3.43E-09	3.49E-07
OTUD1	1.049515	3.54E-09	3.58E-07
C16orf13	0.658956	3.57E-09	3.58E-07
TRIM41	0.811724	3.57E-09	3.58E-07
FABP3	1.834098	3.80E-09	3.78E-07
PTGR1	0.829935	3.80E-09	3.78E-07
GPSM3	0.781763	4.00E-09	3.96E-07
RP11-389(	1.146077	4.05E-09	3.99E-07
NT5C	0.567702	4.07E-09	3.99E-07
BRI3	0.643396	4.13E-09	4.03E-07
MFHAS1	0.86672	4.25E-09	4.12E-07
UBE2S	1.303688	4.26E-09	4.12E-07
RFWD2	0.423994	4.29E-09	4.13E-07
POM121C	0.856515	4.38E-09	4.20E-07
ZNF184	-0.67398	4.57E-09	4.36E-07
LOC34008!	-0.50523	5.01E-09	4.76E-07
WASF2	0.543926	5.05E-09	4.77E-07
PTPN6	0.630388	5.19E-09	4.89E-07

TRAF3IP1	-0.27043	5.29E-09	4.96E-07
NEU1	1.018889	5.35E-09	5.00E-07
GGA2	0.558634	5.54E-09	5.15E-07
NRROS	1.028936	5.89E-09	5.45E-07
RPS4X	-0.11834	5.92E-09	5.46E-07
SDHC	0.665193	6.09E-09	5.59E-07
ARHGEF1	-0.52344	6.14E-09	5.59E-07
MPV17L2	0.255861	6.14E-09	5.59E-07
CLPTM1	0.408005	6.19E-09	5.60E-07
CCDC163P	-0.31047	6.79E-09	6.13E-07
MTPN	1.071156	7.23E-09	6.49E-07
UQCR10	0.714471	7.42E-09	6.64E-07
TSEN15	0.807214	7.55E-09	6.73E-07
DDX19A	0.827497	7.70E-09	6.83E-07
CDKN2B	0.568704	8.01E-09	7.08E-07
LOC10272	-0.50017	8.14E-09	7.16E-07
GTF2H4	0.938459	8.24E-09	7.22E-07
GLUD2	0.311289	8.29E-09	7.24E-07
LITAF	0.873053	8.33E-09	7.25E-07
C21orf33	0.600961	8.57E-09	7.42E-07
MS4A7	2.442333	8.62E-09	7.44E-07
NDRG3	0.713239	9.25E-09	7.92E-07
CASP2	0.692262	9.25E-09	7.92E-07
AHCYL1	0.545742	9.42E-09	8.04E-07
SLK	-0.41582	9.56E-09	8.12E-07
ARL8A	0.373806	1.01E-08	8.55E-07
MKNK2	0.653498	1.03E-08	8.68E-07
NCF1	1.242628	1.06E-08	8.89E-07
CNOT11	0.441869	1.07E-08	8.91E-07
PKD2L1	0.712885	1.08E-08	8.94E-07
TMEM256	0.847733	1.08E-08	8.94E-07
GNG5	0.395623	1.22E-08	1.01E-06
SUPT4H1	0.485324	1.23E-08	1.01E-06
LILRB5	1.472475	1.23E-08	1.01E-06
SLFN5	0.40762	1.24E-08	1.01E-06
AP3D1	0.849412	1.25E-08	1.02E-06
NOL11	-0.35546	1.27E-08	1.03E-06
C11orf53	-0.2182	1.33E-08	1.07E-06
C20orf27	0.625232	1.33E-08	1.07E-06
SH3BGRL3	1.126083	1.35E-08	1.09E-06
NME6	0.787372	1.37E-08	1.09E-06
ZNF804A	-0.96932	1.37E-08	1.09E-06
HLA-G	0.608735	1.39E-08	1.10E-06
CTSC	0.69718	1.42E-08	1.12E-06
HLA-B	0.695497	1.42E-08	1.12E-06
OLFML2B	0.821647	1.50E-08	1.18E-06
MCOLN1	1.373753	1.53E-08	1.20E-06
PCNXL3	0.62999	1.58E-08	1.23E-06
SMPDL3A	0.960589	1.61E-08	1.25E-06
SLAMF8	1.199666	1.70E-08	1.32E-06
ZNF793	-0.62262	1.71E-08	1.32E-06
RBM6	-1.20553	1.72E-08	1.32E-06
RPN1	0.587002	1.77E-08	1.35E-06
CBR1	0.728272	1.84E-08	1.40E-06
PDE6G	0.562644	1.89E-08	1.43E-06
PCBD1	0.815992	1.91E-08	1.45E-06
CD86	1.340896	1.92E-08	1.45E-06
GABARAP	0.401556	1.97E-08	1.48E-06

TTYH3	0.734219	1.98E-08	1.48E-06
POLR1B	-0.44565	1.99E-08	1.48E-06
LSM4	0.972802	1.99E-08	1.48E-06
RP11-432M	-0.1565	2.03E-08	1.50E-06
SLC25A39	1.111136	2.04E-08	1.50E-06
MAP1LC3B	0.662986	2.04E-08	1.50E-06
LSM11	0.389357	2.05E-08	1.50E-06
BAG1	0.502748	2.08E-08	1.52E-06
LAMTOR2	0.819915	2.15E-08	1.57E-06
APOL6	0.498946	2.17E-08	1.58E-06
SMARCA2	0.866941	2.19E-08	1.58E-06
PRMT1	0.658022	2.21E-08	1.60E-06
TDG	-0.46752	2.22E-08	1.60E-06
CCL2	1.175359	2.23E-08	1.60E-06
SNW1	0.449553	2.26E-08	1.61E-06
POLR2L	0.651425	2.26E-08	1.61E-06
RARA	0.360928	2.29E-08	1.63E-06
TMEM176B	0.70996	2.36E-08	1.66E-06
RAB32	0.49127	2.36E-08	1.66E-06
ZNF486	-0.37124	2.44E-08	1.71E-06
ACSM5	1.326494	2.47E-08	1.73E-06
MPDU1	0.565778	2.59E-08	1.81E-06
AP1B1	0.507921	2.63E-08	1.83E-06
HYOU1	1.039661	2.67E-08	1.85E-06
CTBP1	0.53371	2.68E-08	1.85E-06
CDK2AP2	0.906899	2.72E-08	1.87E-06
C1orf43	0.666235	2.74E-08	1.89E-06
RPL7AL2	-0.54203	2.82E-08	1.94E-06
JDP2	0.38214	2.87E-08	1.96E-06
ZDHHC3	0.464588	2.94E-08	2.00E-06
MRPS5	0.51652	2.96E-08	2.01E-06
XAB2	0.490168	2.99E-08	2.02E-06
PDE8A	0.317981	3.00E-08	2.02E-06
CALR	1.227622	3.02E-08	2.03E-06
DAAM2	1.1055	3.16E-08	2.12E-06
TYROBP	1.374635	3.20E-08	2.14E-06
PDAP1	0.38286	3.22E-08	2.14E-06
DOPEY2	0.35922	3.22E-08	2.14E-06
NIT1	0.541676	3.43E-08	2.27E-06
SSBP3	0.568688	3.44E-08	2.27E-06
SEC61G	0.583815	3.60E-08	2.37E-06
BNIP3L	0.61396	3.66E-08	2.40E-06
CSDE1	0.345635	3.66E-08	2.40E-06
SRSF4	0.954599	3.69E-08	2.41E-06
PLD3	1.198792	3.75E-08	2.44E-06
POLD4	0.567273	3.83E-08	2.48E-06
BAG3	1.199892	3.90E-08	2.52E-06
VAMP8	0.419749	3.93E-08	2.53E-06
GOT1	0.59165	3.94E-08	2.53E-06
IRF7	0.895933	3.96E-08	2.53E-06
RP11-121C	-0.56345	4.02E-08	2.56E-06
LINC01094	0.721387	4.14E-08	2.64E-06
LACC1	0.59618	4.18E-08	2.65E-06
MARK4	0.538738	4.19E-08	2.65E-06
ZNF808	-0.44547	4.27E-08	2.70E-06
TLR4	1.169101	4.31E-08	2.71E-06
CD14	2.040011	4.42E-08	2.77E-06
SIN3B	0.434479	4.44E-08	2.78E-06

NUPR1	0.95875	4.46E-08	2.78E-06
KCTD6	0.482922	4.54E-08	2.83E-06
SASH1	1.352207	4.62E-08	2.87E-06
MTPAP	-0.43876	4.64E-08	2.87E-06
BAX	0.724756	4.74E-08	2.92E-06
MICALL1	0.538316	4.76E-08	2.93E-06
ATP6AP2	0.668964	4.77E-08	2.93E-06
FCGRT	1.241316	4.82E-08	2.94E-06
HLA-F	0.589514	4.83E-08	2.94E-06
LRPPRC	-0.38283	4.83E-08	2.94E-06
SDHB	0.30422	4.85E-08	2.94E-06
MRPS10	0.854717	4.93E-08	2.98E-06
AP2B1	0.56624	4.97E-08	2.99E-06
CCND1	1.064686	4.99E-08	2.99E-06
TNFRSF1B	1.315333	5.00E-08	2.99E-06
MYH9	0.54614	5.01E-08	2.99E-06
PA2G4	0.83678	5.01E-08	2.99E-06
COLEC12	1.542894	5.11E-08	3.04E-06
TSHZ3	0.666073	5.13E-08	3.04E-06
FTL	0.648062	5.13E-08	3.04E-06
GRN	0.8141	5.39E-08	3.18E-06
PRPF6	0.524794	5.47E-08	3.22E-06
EFHD2	0.549057	5.53E-08	3.24E-06
HSPA6	1.111665	5.54E-08	3.24E-06
TUSC2	0.476466	5.64E-08	3.29E-06
NOP10	0.818692	5.67E-08	3.30E-06
MTAP	0.594351	5.72E-08	3.32E-06
IMPDH1	0.540831	5.82E-08	3.37E-06
SHC1	0.287159	5.84E-08	3.37E-06
MXI1	0.766671	5.98E-08	3.45E-06
CTD-2325/	0.786311	6.09E-08	3.50E-06
PRRC2A	0.747058	6.12E-08	3.51E-06
IGF2R	1.059986	6.18E-08	3.53E-06
MCC	0.54833	6.26E-08	3.57E-06
AIF1	0.642039	6.85E-08	3.89E-06
THRAP3	0.520912	6.89E-08	3.91E-06
VPS26B	0.420573	6.98E-08	3.95E-06
PRR3	0.771765	7.11E-08	4.01E-06
QKI	0.644785	7.38E-08	4.15E-06
HIBADH	0.768929	7.48E-08	4.19E-06
ZSWIM8	0.343818	7.49E-08	4.19E-06
APH1A	0.493088	7.65E-08	4.25E-06
KLHL42	-0.57989	7.65E-08	4.25E-06
NEK8	-0.34343	7.65E-08	4.25E-06
ADAM33	-0.22486	7.76E-08	4.29E-06
GRB2	0.626793	7.77E-08	4.29E-06
ROMO1	0.939397	7.80E-08	4.30E-06
TGFB1	0.723817	7.90E-08	4.34E-06
IFI35	0.889862	7.96E-08	4.35E-06
LOC10193(	0.546476	7.96E-08	4.35E-06
ACP2	0.916424	8.09E-08	4.41E-06
CDK16	0.440674	8.29E-08	4.51E-06
E2F3	-0.36654	8.47E-08	4.59E-06
COMMD1(	0.72809	8.49E-08	4.59E-06
PRKDC	1.323684	8.51E-08	4.59E-06
BAHD1	0.475791	8.51E-08	4.59E-06
AP1S1	0.631294	8.59E-08	4.61E-06
TESK1	0.491635	8.66E-08	4.64E-06

ARHGAP17	0.542132	8.71E-08	4.66E-06
ENG	0.438287	8.84E-08	4.70E-06
LINC00116	0.858918	8.85E-08	4.70E-06
ACLY	0.47605	8.86E-08	4.70E-06
MSH6	1.093626	9.07E-08	4.80E-06
GOSR2	0.426226	9.37E-08	4.95E-06
RP11-173M	0.628673	9.60E-08	5.06E-06
ARPC2	0.306406	9.75E-08	5.12E-06
KHDRBS1	0.518737	1.02E-07	5.34E-06
CCDC23	0.538056	1.02E-07	5.34E-06
CLTA	0.311644	1.06E-07	5.51E-06
TMEM127	0.427471	1.06E-07	5.52E-06
B3GNT8	0.513095	1.08E-07	5.62E-06
PSMB4	0.472411	1.09E-07	5.62E-06
LSM12	0.437682	1.10E-07	5.70E-06
FKRP	0.286381	1.13E-07	5.79E-06
ATXN7L3	0.382074	1.13E-07	5.79E-06
TRIM47	0.795134	1.13E-07	5.79E-06
BLVRA	0.964082	1.13E-07	5.80E-06
C19orf24	0.470155	1.15E-07	5.87E-06
LOC10192	-0.45846	1.15E-07	5.87E-06
TJAP1	0.290188	1.16E-07	5.90E-06
POLR2E	0.744048	1.16E-07	5.90E-06
MARCKS	1.414093	1.18E-07	5.99E-06
CEP350	-0.47313	1.20E-07	6.04E-06
GPS2	0.557995	1.22E-07	6.16E-06
CMIP	0.650494	1.23E-07	6.17E-06
PLA2G16	0.58854	1.24E-07	6.22E-06
ALMS1	-0.51801	1.24E-07	6.22E-06
PSMC4	0.566343	1.25E-07	6.25E-06
NUDCD1	-0.45463	1.27E-07	6.33E-06
QPRT	0.82496	1.31E-07	6.51E-06
TRNT1	0.524868	1.33E-07	6.58E-06
FEZ2	0.505938	1.34E-07	6.61E-06
AKAP17A	0.778627	1.34E-07	6.61E-06
GPX4	0.698351	1.36E-07	6.71E-06
FAM49A	0.977011	1.37E-07	6.72E-06
FNTA	0.495519	1.38E-07	6.75E-06
CTSS	0.759768	1.38E-07	6.76E-06
WAC	0.38609	1.40E-07	6.83E-06
HCLS1	0.46743	1.42E-07	6.90E-06
MRPS12	0.344233	1.43E-07	6.93E-06
NDUFB7	0.368572	1.46E-07	7.06E-06
HCK	1.438219	1.47E-07	7.10E-06
MSL2	0.793973	1.47E-07	7.11E-06
SPNS1	0.634573	1.49E-07	7.18E-06
GEMIN2	0.745849	1.50E-07	7.19E-06
S100PBP	-0.46291	1.52E-07	7.26E-06
ZNF236	-0.42218	1.52E-07	7.27E-06
PARP12	0.669399	1.55E-07	7.41E-06
MIR1244-3	0.338347	1.56E-07	7.41E-06
Y16709	-0.70309	1.58E-07	7.50E-06
ZMAT5	0.654167	1.61E-07	7.65E-06
NDUFA7	0.35169	1.63E-07	7.70E-06
UBC	0.322558	1.63E-07	7.70E-06
DSTNP2	0.370755	1.64E-07	7.72E-06
SAMSN1	0.80044	1.66E-07	7.80E-06
FNDC3A	0.358585	1.70E-07	7.98E-06

WWC3	0.597158	1.71E-07	7.98E-06
FCHO2	0.786424	1.71E-07	7.98E-06
PHC2	0.231885	1.72E-07	7.99E-06
UPP1	0.984565	1.75E-07	8.12E-06
CLASRP	0.540577	1.76E-07	8.15E-06
CTSZ	0.561638	1.82E-07	8.40E-06
C9orf69	0.517571	1.84E-07	8.50E-06
MRPS28	0.760627	1.85E-07	8.50E-06
TMEM230	0.432938	1.85E-07	8.51E-06
RNF19B	0.887429	1.86E-07	8.52E-06
TMSB4X	0.558254	1.86E-07	8.52E-06
WDR53	-0.43046	1.87E-07	8.52E-06
B3GAT2	-0.45049	1.88E-07	8.56E-06
LYVE1	1.318056	1.89E-07	8.60E-06
TOR1AIP1	0.605264	1.91E-07	8.69E-06
THRA	0.258938	1.92E-07	8.69E-06
GNS	0.928677	1.92E-07	8.69E-06
TANGO2	0.352595	1.94E-07	8.77E-06
AXL	1.010874	1.95E-07	8.77E-06
CD59	0.573589	1.96E-07	8.79E-06
OGFR	0.338686	1.96E-07	8.79E-06
FUT8	0.55611	1.99E-07	8.89E-06
FSCN1	0.583219	1.99E-07	8.90E-06
DAZAP1	-0.39823	2.01E-07	8.97E-06
TNS1	0.672538	2.04E-07	9.09E-06
STX6	0.357148	2.05E-07	9.11E-06
CEBPD	1.172982	2.07E-07	9.15E-06
HNRNPH1	0.834548	2.08E-07	9.18E-06
TMEM5	-0.39221	2.08E-07	9.18E-06
ATP13A2	0.673901	2.08E-07	9.18E-06
GBP1	1.085831	2.12E-07	9.30E-06
GSTP1	0.751123	2.13E-07	9.32E-06
OSBPL1A	0.868318	2.13E-07	9.34E-06
PRR13	0.353665	2.15E-07	9.37E-06
UCP2	0.729077	2.18E-07	9.49E-06
PRKCD	0.993784	2.24E-07	9.75E-06
EPAS1	0.91722	2.25E-07	9.77E-06
PPARG	1.45852	2.26E-07	9.79E-06
SNX9	0.97209	2.34E-07	1.01E-05
ZNF865	0.138349	2.39E-07	1.03E-05
RMND1	0.593056	2.41E-07	1.04E-05
SET	0.980728	2.43E-07	1.04E-05
BAG6	0.503153	2.46E-07	1.05E-05
ACTN4	0.62889	2.52E-07	1.08E-05
VCPKMT	-0.42965	2.54E-07	1.08E-05
NFE2L1	0.371717	2.54E-07	1.08E-05
PLA2G15	0.936328	2.60E-07	1.11E-05
PKP4	0.908422	2.61E-07	1.11E-05
ARRB2	0.625797	2.65E-07	1.12E-05
RFNG	0.525442	2.67E-07	1.13E-05
FPR3	1.087101	2.67E-07	1.13E-05
USF2	0.42649	2.69E-07	1.13E-05
ARHGAP4	0.690144	2.73E-07	1.15E-05
CHMP1B	0.634699	2.76E-07	1.16E-05
HERC6	0.378498	2.78E-07	1.16E-05
CHD8	0.527832	2.80E-07	1.17E-05
CTD-3092/	-0.61554	2.82E-07	1.18E-05
BATF3	0.58122	2.82E-07	1.18E-05

CREBL2	0.813995	2.84E-07	1.18E-05
NBR1	0.253454	2.84E-07	1.18E-05
NAPA	0.816083	2.86E-07	1.18E-05
PBDC1	0.475482	2.94E-07	1.22E-05
SRA1	0.638704	2.97E-07	1.23E-05
METAP2	-0.42098	2.98E-07	1.23E-05
ZFP62	-0.42638	3.04E-07	1.25E-05
ARAP1	0.526382	3.07E-07	1.26E-05
GNGT2	0.789783	3.08E-07	1.26E-05
PPFIBP2	1.016426	3.08E-07	1.26E-05
NCOA5	0.35965	3.11E-07	1.27E-05
LOC10192	-0.56204	3.14E-07	1.28E-05
CMTR1	0.400101	3.19E-07	1.30E-05
CTDNEP1	0.460504	3.21E-07	1.30E-05
METAP1	-0.25632	3.25E-07	1.32E-05
PSAP	0.801473	3.29E-07	1.33E-05
C4orf3	0.500269	3.35E-07	1.35E-05
ACP1	0.44388	3.40E-07	1.37E-05
CEP89	0.214884	3.43E-07	1.38E-05
2-Mar	1.044736	3.45E-07	1.38E-05
RANBP9	0.620071	3.46E-07	1.38E-05
RP11-199F	-0.85144	3.47E-07	1.39E-05
ASNA1	0.579599	3.47E-07	1.39E-05
RPS11	-0.71001	3.51E-07	1.40E-05
TBC1D9	0.951106	3.52E-07	1.40E-05
ZNF33B	-0.47316	3.54E-07	1.40E-05
MRPL54	0.740627	3.54E-07	1.40E-05
SUPT5H	0.358017	3.65E-07	1.44E-05
ISY1	0.468222	3.66E-07	1.44E-05
DTWD1	-0.44186	3.67E-07	1.44E-05
DPY30	0.424971	3.72E-07	1.46E-05
RNF123	0.456003	3.75E-07	1.47E-05
RNF34	-0.61295	3.86E-07	1.51E-05
PRCC	0.613889	3.87E-07	1.51E-05
GINM1	0.429994	3.91E-07	1.52E-05
MFNG	0.697092	3.91E-07	1.52E-05
GBF1	0.519631	3.96E-07	1.54E-05
GALM	0.871602	3.97E-07	1.54E-05
ZC3H8	-0.37821	3.98E-07	1.54E-05
RAB35	0.324159	4.11E-07	1.59E-05
COA6	0.351048	4.18E-07	1.62E-05
RBM17	0.317228	4.25E-07	1.64E-05
IKZF4	0.385683	4.35E-07	1.67E-05
ND6	-0.93407	4.48E-07	1.72E-05
GRINA	0.610372	4.54E-07	1.74E-05
FAM65A	0.566745	4.55E-07	1.74E-05
CHP1	0.336107	4.65E-07	1.78E-05
ZMYM2	-0.60604	4.69E-07	1.79E-05
FUCA1	0.668629	4.72E-07	1.80E-05
SRPX2	0.345282	4.74E-07	1.80E-05
SH2B1	0.507602	4.76E-07	1.81E-05
DNASE1L1	0.496732	4.80E-07	1.82E-05
NR1H3	0.902391	4.81E-07	1.82E-05
ZBTB24	-0.38744	4.88E-07	1.84E-05
NEDD8	0.576939	4.89E-07	1.84E-05
OSTM1	0.637648	4.90E-07	1.84E-05
ZNF550	-0.26782	4.95E-07	1.86E-05
GNB2	0.689785	5.04E-07	1.89E-05

FPR1	0.867059	5.06E-07	1.89E-05
RPL3	-0.20542	5.06E-07	1.89E-05
CALU	0.607529	5.11E-07	1.90E-05
SIRPA	0.444863	5.16E-07	1.92E-05
SLC38A7	0.426071	5.17E-07	1.92E-05
ZCRB1	0.415108	5.37E-07	1.99E-05
SDC2	0.711488	5.49E-07	2.03E-05
PEBP1	0.433137	5.51E-07	2.04E-05
ZBTB8OS	0.446101	5.55E-07	2.05E-05
ACTR1A	0.414413	5.64E-07	2.08E-05
COG8	-0.31635	5.65E-07	2.08E-05
KPNA3	0.37116	5.66E-07	2.08E-05
FLII	0.294749	5.68E-07	2.08E-05
SBF1	0.370681	5.70E-07	2.08E-05
EEF1A1	-0.08781	5.70E-07	2.08E-05
OAZ1	0.327635	5.73E-07	2.09E-05
TAGLN2	0.863905	5.77E-07	2.10E-05
RNF213	0.443115	5.81E-07	2.11E-05
ENO1	0.692628	5.93E-07	2.15E-05
ATP6V0B	0.508979	5.94E-07	2.15E-05
GNAI2	0.595851	6.03E-07	2.18E-05
APBA1	0.279171	6.03E-07	2.18E-05
APOL1	0.556716	6.07E-07	2.19E-05
PI4K2A	0.572156	6.09E-07	2.19E-05
ERC1	0.408486	6.21E-07	2.23E-05
ST6GALNA	0.456155	6.25E-07	2.24E-05
C7orf43	0.332484	6.26E-07	2.24E-05
MAPK14	0.557364	6.26E-07	2.24E-05
GSTM4	0.459164	6.28E-07	2.24E-05
LBR	-0.33732	6.30E-07	2.24E-05
MS4A4A	1.565765	6.30E-07	2.24E-05
CEBPA	0.813588	6.41E-07	2.27E-05
PLTP	1.023638	6.57E-07	2.33E-05
APOE	1.657242	6.57E-07	2.33E-05
RAB20	0.599625	6.63E-07	2.34E-05
ACOT13	0.722271	6.72E-07	2.37E-05
TULP4	0.303188	6.75E-07	2.38E-05
BATF2	0.381097	6.79E-07	2.39E-05
AF090939	-0.59077	6.80E-07	2.39E-05
CEP97	-0.59464	6.89E-07	2.42E-05
PLCB3	0.273077	6.93E-07	2.42E-05
NDUFAF3	0.575302	6.94E-07	2.42E-05
MRO	1.246331	6.96E-07	2.43E-05
RAC1	0.56893	6.99E-07	2.43E-05
YWHAE	0.504288	7.00E-07	2.43E-05
RAB9A	0.42861	7.04E-07	2.44E-05
PAIP2	0.440051	7.05E-07	2.44E-05
MAP3K2	0.631852	7.13E-07	2.47E-05
C1orf174	0.51511	7.15E-07	2.47E-05
SLC22A18	0.576401	7.19E-07	2.48E-05
EGFL7	0.802544	7.20E-07	2.48E-05
CDC34	0.328019	7.20E-07	2.48E-05
LMF2	0.335037	7.22E-07	2.48E-05
CYB5R1	0.358851	7.23E-07	2.48E-05
SOWAHC	0.829671	7.24E-07	2.48E-05
PLEKHB2	0.555306	7.26E-07	2.48E-05
MRPS7	0.545249	7.41E-07	2.53E-05
SEC22B	-0.51862	7.44E-07	2.53E-05

TK2	0.486755	7.56E-07	2.57E-05
YWHAB	0.533245	7.58E-07	2.57E-05
FES	0.534265	7.60E-07	2.58E-05
OSBPL11	0.70155	7.67E-07	2.59E-05
HNMT	0.686057	7.73E-07	2.61E-05
UBE2H	0.430938	7.88E-07	2.66E-05
BMPR2	0.690012	7.93E-07	2.67E-05
MIS12	-0.357	8.15E-07	2.74E-05
PPM1M	0.697022	8.21E-07	2.76E-05
NOC2L	0.246497	8.37E-07	2.80E-05
GTF2F1	0.562756	8.37E-07	2.80E-05
TFEB	0.444236	8.55E-07	2.85E-05
SULT1A2	0.597619	8.55E-07	2.85E-05
ST6GALNA	0.328186	8.55E-07	2.85E-05
ING3	-0.54078	8.58E-07	2.85E-05
MYO9B	0.661555	8.65E-07	2.87E-05
ALDH1B1	0.376317	8.81E-07	2.92E-05
GNG12	1.223577	8.81E-07	2.92E-05
NCEH1	1.004345	8.82E-07	2.92E-05
PAM16	0.567651	8.88E-07	2.93E-05
TPI1	0.509601	9.00E-07	2.97E-05
HEBP2	0.24199	9.02E-07	2.97E-05
RANBP2	-0.38147	9.16E-07	3.01E-05
TRAPPC10	0.664875	9.20E-07	3.02E-05
ZRANB1	0.412015	9.25E-07	3.03E-05
ZNF513	0.512832	9.28E-07	3.04E-05
HYMAI	-0.69409	9.30E-07	3.04E-05
HIGD2A	0.352075	9.45E-07	3.08E-05
PBX2	0.386423	9.57E-07	3.12E-05
CUEDC1	0.48823	9.63E-07	3.13E-05
GPBP1L1	0.505007	9.70E-07	3.15E-05
SF3A2	0.753611	9.70E-07	3.15E-05
NACAP1	-0.08383	9.73E-07	3.15E-05
STARD3	0.507892	9.88E-07	3.19E-05
DDX20	-0.33852	9.90E-07	3.20E-05
PALLD	0.717024	9.98E-07	3.21E-05
PPP1R9B	0.288038	1.01E-06	3.25E-05
GPX3	1.400498	1.01E-06	3.26E-05
SPIDR	0.60166	1.03E-06	3.30E-05
POLR2I	0.547103	1.03E-06	3.30E-05
CASKIN2	0.350315	1.03E-06	3.30E-05
MYBBP1A	0.337371	1.03E-06	3.30E-05
VAMP2	0.403654	1.04E-06	3.31E-05
LOC10012	-0.38241	1.04E-06	3.31E-05
MRGBP	0.373307	1.05E-06	3.34E-05
MKS1	0.2457	1.07E-06	3.39E-05
BCAR3	0.434832	1.07E-06	3.39E-05
COX6C	0.438153	1.07E-06	3.40E-05
PGBD5	0.788845	1.08E-06	3.43E-05
PCNP	0.518479	1.09E-06	3.43E-05
TAF11	0.650781	1.09E-06	3.43E-05
FASN	0.331902	1.10E-06	3.46E-05
GBP5	0.864915	1.10E-06	3.46E-05
BIRC2	-0.34109	1.13E-06	3.54E-05
ZNHIT1	0.507785	1.13E-06	3.55E-05
SLTM	-0.31815	1.13E-06	3.55E-05
ZMYM3	-0.74079	1.16E-06	3.63E-05
ABHD12	0.57195	1.16E-06	3.63E-05

FAM193B	0.393318	1.17E-06	3.63E-05
PPIC	0.631266	1.17E-06	3.63E-05
ZFP36L2	0.990954	1.17E-06	3.63E-05
RNF13	0.406355	1.17E-06	3.63E-05
TSTA3	0.645421	1.18E-06	3.65E-05
SCARB2	0.445376	1.19E-06	3.67E-05
MRPL4	0.688148	1.19E-06	3.68E-05
HNRNPUL1	0.480588	1.19E-06	3.69E-05
CAMK2G	0.400462	1.20E-06	3.70E-05
ITCH	0.418262	1.20E-06	3.70E-05
SIRT2	0.31001	1.21E-06	3.70E-05
LIMK2	0.466411	1.22E-06	3.75E-05
FDX1L	0.582058	1.23E-06	3.76E-05
CTSL	1.512253	1.23E-06	3.76E-05
TAF1	0.62955	1.23E-06	3.76E-05
RAP1GDS1	-0.5768	1.23E-06	3.76E-05
FEM1A	0.21883	1.25E-06	3.80E-05
ATF7	0.273173	1.25E-06	3.82E-05
TMUB1	0.460869	1.26E-06	3.83E-05
ASB16-AS1	0.554545	1.27E-06	3.84E-05
TRIM24	-0.39068	1.28E-06	3.86E-05
1-Mar	1.149922	1.29E-06	3.89E-05
LSM2	0.79656	1.31E-06	3.96E-05
SRM	0.694887	1.32E-06	3.97E-05
ZNF587B	-0.48012	1.32E-06	3.99E-05
RNF141	0.507675	1.33E-06	4.01E-05
SLCO4A1	0.343919	1.34E-06	4.04E-05
SAMD9	0.495087	1.35E-06	4.04E-05
DNAJC17	0.541675	1.36E-06	4.08E-05
POLE4	0.663868	1.36E-06	4.08E-05
PCBP1	0.360889	1.37E-06	4.08E-05
CCDC115	0.347809	1.37E-06	4.09E-05
CCR1	1.523622	1.40E-06	4.18E-05
POTEKP	0.574466	1.41E-06	4.21E-05
IRF2	0.507145	1.42E-06	4.21E-05
CPSF3	-0.31479	1.43E-06	4.23E-05
C16orf87	-0.5105	1.43E-06	4.24E-05
SUMO1	0.294021	1.44E-06	4.25E-05
RAB14	0.563287	1.44E-06	4.25E-05
VPS4B	-0.43012	1.45E-06	4.27E-05
SNF8	0.477084	1.47E-06	4.32E-05
ZNF85	-0.44943	1.47E-06	4.32E-05
ZNF689	0.43021	1.48E-06	4.36E-05
PIGH	0.475751	1.49E-06	4.38E-05
AKT1	0.552129	1.50E-06	4.38E-05
DAXX	0.322711	1.50E-06	4.38E-05
AKIRIN2	0.492667	1.50E-06	4.39E-05
MRPL43	0.61521	1.51E-06	4.41E-05
IGSF6	1.537921	1.52E-06	4.43E-05
CXCL9	1.277529	1.53E-06	4.44E-05
CFL1	0.562897	1.53E-06	4.44E-05
FGL2	1.391696	1.53E-06	4.44E-05
NAAA	0.430405	1.53E-06	4.45E-05
CD84	0.509903	1.54E-06	4.45E-05
PROSC	0.536914	1.54E-06	4.45E-05
RSL1D1	-0.41849	1.55E-06	4.46E-05
IGFBP4	0.456835	1.56E-06	4.50E-05
IQCB1	-0.3516	1.56E-06	4.50E-05

DDX3X	0.587851	1.57E-06	4.51E-05
LINC00674	-0.2532	1.58E-06	4.53E-05
NCOA7	0.299231	1.58E-06	4.54E-05
APPL2	0.621568	1.60E-06	4.59E-05
RP1-30M3	0.51117	1.61E-06	4.62E-05
MRPL55	0.306574	1.62E-06	4.62E-05
RBP7	1.51531	1.63E-06	4.65E-05
BC047484	-0.41669	1.64E-06	4.67E-05
TFE3	0.284079	1.67E-06	4.74E-05
KCNJ2	1.128269	1.67E-06	4.75E-05
C19orf60	0.492166	1.68E-06	4.76E-05
SWI5	0.492213	1.69E-06	4.78E-05
FAM223B	-0.50833	1.69E-06	4.78E-05
ETV5	0.442791	1.70E-06	4.79E-05
MRPL40	0.466348	1.70E-06	4.79E-05
ZNF350	-0.57094	1.74E-06	4.92E-05
MOCS2	0.440633	1.75E-06	4.94E-05
ZNF571	-0.60363	1.77E-06	4.98E-05
UFD1L	0.358705	1.77E-06	4.98E-05
FABP5	0.543004	1.79E-06	5.02E-05
C6orf223	-0.11435	1.79E-06	5.02E-05
TMEM160	0.819875	1.83E-06	5.11E-05
IGSF21	0.807962	1.84E-06	5.14E-05
LILRB2	1.091355	1.88E-06	5.25E-05
CWC15	0.515804	1.89E-06	5.27E-05
ARF3	0.578333	1.90E-06	5.29E-05
IFITM2	0.575385	1.90E-06	5.29E-05
POLR2J	0.693315	1.93E-06	5.36E-05
ARHGDIA	0.407024	1.94E-06	5.39E-05
RNPEP	0.493348	1.95E-06	5.39E-05
RGS19	0.522905	1.97E-06	5.46E-05
PRPS2	-0.41453	2.00E-06	5.53E-05
DGKZ	0.322708	2.00E-06	5.53E-05
RP11-843E	-0.42111	2.01E-06	5.55E-05
LILRB4	0.283102	2.02E-06	5.57E-05
ME2	0.534599	2.03E-06	5.58E-05
CTSV	0.653349	2.05E-06	5.64E-05
SMEK2	-0.50024	2.06E-06	5.66E-05
ADAM10	0.558564	2.08E-06	5.69E-05
XRCC2	-0.51529	2.10E-06	5.73E-05
DRG2	0.454668	2.12E-06	5.79E-05
POLM	0.38889	2.13E-06	5.79E-05
ADAD2	-0.24067	2.13E-06	5.79E-05
RPL27A	-0.61341	2.14E-06	5.82E-05
CCDC71	-0.29181	2.15E-06	5.85E-05
CTD-2035I	-0.96619	2.16E-06	5.86E-05
ELF5	0.490881	2.16E-06	5.86E-05
RNF181	0.538258	2.16E-06	5.86E-05
MTO1	-0.33934	2.20E-06	5.95E-05
ZFYVE26	0.345823	2.23E-06	6.03E-05
LOC10012	-0.13136	2.27E-06	6.12E-05
SH3GL1	0.420256	2.28E-06	6.13E-05
ZNF593	0.71574	2.29E-06	6.16E-05
NAG18	-0.32169	2.30E-06	6.16E-05
DEXI	0.594752	2.30E-06	6.16E-05
C19orf25	0.335476	2.31E-06	6.18E-05
IFITM1	1.032346	2.34E-06	6.26E-05
MTFMT	0.65409	2.36E-06	6.31E-05

RALGDS	0.625499	2.36E-06	6.31E-05
CAPN7	0.339646	2.37E-06	6.32E-05
APOPT1	0.236523	2.40E-06	6.38E-05
ERGIC1	0.485066	2.41E-06	6.42E-05
TSN	-0.31309	2.44E-06	6.48E-05
ATG7	0.441751	2.46E-06	6.53E-05
TMEM68	-0.44085	2.46E-06	6.53E-05
SARS	0.414194	2.49E-06	6.60E-05
NUDT16	0.263798	2.51E-06	6.63E-05
TMSB10	0.459927	2.54E-06	6.72E-05
GLYR1	0.182158	2.57E-06	6.79E-05
PLBD2	0.296406	2.58E-06	6.79E-05
KIAA1279	0.612658	2.59E-06	6.81E-05
NR1H2	0.500612	2.59E-06	6.81E-05
HSPB11	0.544927	2.64E-06	6.92E-05
CDC5L	0.508291	2.64E-06	6.92E-05
ADAP2	1.516024	2.64E-06	6.92E-05
SCN1B	0.894283	2.69E-06	7.03E-05
FCGR1B	1.09564	2.73E-06	7.14E-05
S100A9	1.723367	2.74E-06	7.14E-05
CETP	1.348132	2.75E-06	7.16E-05
COMMD2	-0.30901	2.76E-06	7.19E-05
ADIG	-0.14899	2.84E-06	7.39E-05
CCNYL1	0.519957	2.85E-06	7.39E-05
MYL5	0.502238	2.89E-06	7.49E-05
FAR1	0.485384	2.90E-06	7.51E-05
ENSA	0.661198	2.90E-06	7.52E-05
FTH1	0.594907	2.91E-06	7.52E-05
PTOV1	0.486826	2.92E-06	7.55E-05
EIF4EBP1	0.777358	2.97E-06	7.65E-05
PPP1R3C	0.383324	2.98E-06	7.67E-05
TIMP3	0.725013	2.99E-06	7.68E-05
KLF13	0.890735	3.03E-06	7.78E-05
IGF1	1.000491	3.06E-06	7.85E-05
GIMAP4	1.561913	3.09E-06	7.92E-05
TMEM120A	0.45691	3.12E-06	7.97E-05
MYPOP	0.424363	3.13E-06	8.00E-05
CYP4F12	-0.63815	3.17E-06	8.10E-05
LINC01208	-0.30016	3.19E-06	8.12E-05
MILR1	0.758324	3.19E-06	8.12E-05
FIBP	0.466358	3.19E-06	8.13E-05
LOC10050(	0.661644	3.24E-06	8.23E-05
MAFB	1.735662	3.24E-06	8.23E-05
AMMECR1	-0.39794	3.26E-06	8.27E-05
PFDN4	-0.33817	3.26E-06	8.27E-05
NIPSNAP1	0.48852	3.29E-06	8.32E-05
IFI44	0.817241	3.29E-06	8.32E-05
IQSEC1	0.61677	3.33E-06	8.41E-05
LOC10192	0.601961	3.35E-06	8.44E-05
TMEM170A	0.422381	3.35E-06	8.44E-05
EIF4G1	0.514018	3.38E-06	8.49E-05
CCDC137	0.462193	3.38E-06	8.50E-05
TNFRSF21	0.713741	3.40E-06	8.53E-05
FAM98C	0.500888	3.41E-06	8.55E-05
CLTB	0.506784	3.43E-06	8.60E-05
TPD52L1	0.903195	3.46E-06	8.65E-05
C9orf38	-1.24468	3.47E-06	8.66E-05
ATP5D	0.555443	3.47E-06	8.66E-05

WARS	0.693965	3.49E-06	8.69E-05
PDZD11	0.65421	3.52E-06	8.77E-05
C1QB	2.333059	3.53E-06	8.77E-05
CPEB4	0.892048	3.53E-06	8.77E-05
RP11-173E	-0.3441	3.53E-06	8.77E-05
NDUFB11	0.504535	3.55E-06	8.80E-05
SLC4A1	1.055983	3.58E-06	8.86E-05
FCGR2A	0.611452	3.60E-06	8.89E-05
CACNB3	-0.45296	3.63E-06	8.96E-05
RPL35A	-0.44009	3.64E-06	8.99E-05
RNF114	0.386323	3.67E-06	9.05E-05
SSBP4	0.43448	3.69E-06	9.08E-05
ZNF346	0.492532	3.70E-06	9.08E-05
MVP	0.529985	3.70E-06	9.08E-05
CFP	0.882511	3.71E-06	9.11E-05
TSPAN31	0.424076	3.76E-06	9.22E-05
MRPL34	0.567882	3.77E-06	9.22E-05
DUS1L	0.442357	3.79E-06	9.25E-05
C1orf85	0.659727	3.80E-06	9.27E-05
CSNK1G2	0.395548	3.81E-06	9.29E-05
RDX	0.496724	3.83E-06	9.31E-05
ADORA3	1.422446	3.83E-06	9.31E-05
SIGLEC9	0.561638	3.87E-06	9.41E-05
SGK1	1.288226	3.88E-06	9.43E-05
SKA1	0.676455	3.89E-06	9.43E-05
ZNF721	-0.43149	3.89E-06	9.43E-05
PTPN18	0.530228	3.90E-06	9.45E-05
RHBDD2	0.399795	3.95E-06	9.54E-05
RPS6KA4	0.230891	3.96E-06	9.55E-05
NLRC4	0.48058	3.96E-06	9.55E-05
SLC7A8	0.809307	3.97E-06	9.56E-05
PDK4	0.57392	3.98E-06	9.57E-05
GRAMD1A	0.543664	4.02E-06	9.67E-05
COPB2	0.227925	4.04E-06	9.69E-05
SLC25A37	0.932674	4.04E-06	9.69E-05
TMEM176A	1.048255	4.05E-06	9.69E-05
VAMP5	0.423412	4.07E-06	9.73E-05
TRIM11	0.386625	4.08E-06	9.75E-05
CRYL1	0.711699	4.09E-06	9.77E-05
JUNB	0.897192	4.11E-06	9.80E-05
PDLIM2	0.318675	4.12E-06	9.81E-05
MAD2L1	1.007476	4.15E-06	9.88E-05
RNASE1	0.744511	4.21E-06	1.00E-04
YWHAQ	-0.27872	4.21E-06	1.00E-04
KHSRP	0.172392	4.24E-06	1.01E-04
TAP1	0.447133	4.25E-06	1.01E-04
MVB12B	0.195287	4.27E-06	1.01E-04
CD180	0.919995	4.28E-06	1.01E-04
SNX14	-0.35446	4.28E-06	1.01E-04
SNX15	0.287147	4.31E-06	1.02E-04
FKBP15	0.198816	4.36E-06	1.03E-04
NMRK1	0.399595	4.37E-06	1.03E-04
EVI5	0.47664	4.44E-06	1.04E-04
GALNS	0.200437	4.47E-06	1.05E-04
CLEC7A	0.681104	4.47E-06	1.05E-04
DUSP23	0.429498	4.49E-06	1.05E-04
KCNQ5	-0.37642	4.52E-06	1.06E-04
AKIP1	0.314618	4.52E-06	1.06E-04

STUB1	0.438657	4.53E-06	1.06E-04
TRAPPC5	0.628637	4.54E-06	1.06E-04
C2	0.680718	4.55E-06	1.06E-04
KIAA2026	0.39499	4.56E-06	1.06E-04
ATP1A1	0.434031	4.62E-06	1.07E-04
LENG8	0.884858	4.67E-06	1.08E-04
METTL7A	-0.36443	4.74E-06	1.10E-04
SLC26A11	0.721396	4.77E-06	1.11E-04
CEP63	0.65297	4.78E-06	1.11E-04
RHOC	0.358296	4.82E-06	1.11E-04
GYPA	1.377346	4.82E-06	1.11E-04
CNOT3	0.499834	4.83E-06	1.12E-04
MAP1LC3C	0.380739	4.84E-06	1.12E-04
LRRC42	0.354839	4.85E-06	1.12E-04
HLA-J	0.494219	4.85E-06	1.12E-04
CLASP1	-0.46622	4.87E-06	1.12E-04
S100A6	0.451108	4.88E-06	1.12E-04
WDR61	-0.30687	4.90E-06	1.13E-04
AGAP4	-0.60868	4.92E-06	1.13E-04
MED29	0.376095	4.93E-06	1.13E-04
GLIPR2	0.574263	4.94E-06	1.13E-04
WDFY3-AS	0.426669	4.96E-06	1.13E-04
PTPRO	0.964999	4.97E-06	1.13E-04
GOLGA7	0.292463	5.04E-06	1.15E-04
DEAF1	0.353341	5.12E-06	1.17E-04
ATP6V0E1	1.064283	5.14E-06	1.17E-04
CLIC2	0.917323	5.15E-06	1.17E-04
VPS54	-0.34969	5.21E-06	1.18E-04
GNA11	0.338992	5.28E-06	1.20E-04
GPNMB	1.569087	5.33E-06	1.21E-04
ARFGEF1	0.594122	5.34E-06	1.21E-04
MTM1	-0.3243	5.36E-06	1.21E-04
UPF3B	-0.24492	5.37E-06	1.21E-04
TRIM22	0.493561	5.42E-06	1.22E-04
TMEM255A	1.062034	5.43E-06	1.22E-04
USB1	0.480379	5.45E-06	1.23E-04
RHOU	0.804217	5.46E-06	1.23E-04
PRR14	0.488936	5.49E-06	1.23E-04
FBXW12	-0.20932	5.51E-06	1.24E-04
EIF3L	-0.17017	5.53E-06	1.24E-04
CCL13	0.553287	5.54E-06	1.24E-04
C12orf5	0.875205	5.55E-06	1.24E-04
ABCA1	0.639195	5.55E-06	1.24E-04
SNRNP35	-0.62196	5.57E-06	1.24E-04
PAK2	0.34584	5.59E-06	1.25E-04
IL10RA	1.310795	5.61E-06	1.25E-04
ZNF790-A	-0.42202	5.62E-06	1.25E-04
SNRPF	0.557794	5.64E-06	1.25E-04
PARVG	0.686659	5.70E-06	1.27E-04
PSENEN	0.505948	5.77E-06	1.28E-04
TMEM86B	0.314042	5.78E-06	1.28E-04
MRPS6	0.363914	5.80E-06	1.28E-04
OGDH	0.287106	5.81E-06	1.28E-04
CEACAM2	0.31389	5.82E-06	1.29E-04
CLUAP1	0.456541	5.85E-06	1.29E-04
DFFA	0.356332	5.87E-06	1.29E-04
SLC25A20	0.419414	5.87E-06	1.29E-04
RND3	1.377138	5.88E-06	1.29E-04

CHTF8	0.356586	5.89E-06	1.29E-04
TBC1D20	0.177564	5.95E-06	1.31E-04
HMOX1	2.315234	6.02E-06	1.32E-04
SMARCC1	0.583609	6.04E-06	1.32E-04
CCL4	1.764222	6.11E-06	1.34E-04
FAM134C	-0.46034	6.12E-06	1.34E-04
ADAP1	0.367088	6.12E-06	1.34E-04
SPIC	1.608807	6.18E-06	1.35E-04
PUM1	0.371754	6.19E-06	1.35E-04
C5AR1	1.270899	6.20E-06	1.35E-04
ULK1	0.379153	6.21E-06	1.35E-04
CPM	0.783089	6.26E-06	1.36E-04
CTC-425F1	-0.62703	6.29E-06	1.37E-04
SLIRP	0.513921	6.31E-06	1.37E-04
RANGAP1	0.436937	6.32E-06	1.37E-04
TMIGD2	-0.38901	6.35E-06	1.38E-04
CD72	1.263481	6.36E-06	1.38E-04
VAT1	0.574034	6.46E-06	1.40E-04
CAMSAP2	0.555438	6.48E-06	1.40E-04
AIM2	0.985749	6.52E-06	1.41E-04
NUP54	-0.76015	6.56E-06	1.41E-04
LGALS3	0.822867	6.59E-06	1.42E-04
NRP1	0.479671	6.59E-06	1.42E-04
PTPN23	0.18331	6.61E-06	1.42E-04
MECP2	0.459081	6.71E-06	1.44E-04
OPA3	0.25626	6.79E-06	1.46E-04
CCDC117	0.291969	6.83E-06	1.46E-04
ASPA	0.489784	6.87E-06	1.47E-04
S100A8	1.279761	7.00E-06	1.50E-04
CMKLR1	0.729769	7.07E-06	1.51E-04
ADAR	0.274103	7.11E-06	1.52E-04
MDK	0.82701	7.20E-06	1.53E-04
VPS13A	-0.59398	7.23E-06	1.54E-04
CR1L	1.136022	7.29E-06	1.55E-04
NPL	0.982439	7.32E-06	1.56E-04
SEC14L1	0.646713	7.36E-06	1.56E-04
SFPQ	0.415194	7.46E-06	1.58E-04
HPS5	0.321516	7.51E-06	1.59E-04
CD47	-0.70737	7.52E-06	1.59E-04
PPIB	0.539088	7.67E-06	1.62E-04
C6orf106	0.556505	7.73E-06	1.63E-04
ZNF84	-0.3923	7.79E-06	1.64E-04
PREX1	0.4681	7.84E-06	0.000165
EIF1	0.311677	7.86E-06	1.66E-04
TMPO	-0.67988	7.92E-06	1.67E-04
RTN2	0.421102	7.92E-06	1.67E-04
PPP2CB	0.183346	7.98E-06	1.68E-04
PLEKHH1	0.301168	8.03E-06	1.68E-04
CTD-2012I	-0.25397	8.04E-06	1.68E-04
DNAJB1	0.499648	8.06E-06	1.69E-04
RPL36	0.687374	8.20E-06	1.72E-04
SLC25A1	0.539698	8.21E-06	1.72E-04
TMCC2	0.408369	8.26E-06	1.73E-04
RARRES3	0.579588	8.27E-06	1.73E-04
CD4	0.337588	8.30E-06	1.73E-04
C1QA	2.112454	8.35E-06	1.74E-04
VPS9D1	0.252081	8.42E-06	1.75E-04
PRADC1	0.601217	8.45E-06	1.75E-04

AKT1S1	0.178987	8.45E-06	1.75E-04
NDUFA5	-0.50047	8.47E-06	1.76E-04
AKAP13	0.430026	8.54E-06	1.77E-04
SULT1A1	0.626978	8.65E-06	1.79E-04
HMGXB3	0.397632	8.66E-06	1.79E-04
ABHD17A	0.557159	8.67E-06	1.79E-04
TNFRSF1A	0.537279	8.67E-06	1.79E-04
RPTOR	0.409524	8.69E-06	1.79E-04
PDE6B	0.196138	8.86E-06	1.83E-04
LZTFL1	-0.2682	8.91E-06	1.83E-04
RNF219	-0.27095	8.92E-06	1.83E-04
HRAS	0.52596	9.09E-06	1.87E-04
CCL23	0.571637	9.12E-06	1.87E-04
RP11-155(	-0.24987	9.16E-06	1.88E-04
ACIN1	0.517197	9.23E-06	1.89E-04
LRRC48	-0.1402	9.24E-06	1.89E-04
TAPBP	0.336083	9.45E-06	1.93E-04
CNOT6	-0.24045	9.50E-06	1.94E-04
AY940074	-0.60002	9.64E-06	1.97E-04
UACA	0.30976	9.66E-06	1.97E-04
ATXN7	0.508904	9.75E-06	1.99E-04
FAM89B	0.362259	9.77E-06	1.99E-04
SAP30	0.302997	9.92E-06	2.02E-04
TSR3	0.233315	9.95E-06	2.02E-04
PNPLA6	0.515304	9.97E-06	2.02E-04
PMPCB	-0.23775	9.99E-06	2.02E-04
NAGK	0.851955	9.99E-06	2.02E-04
GFPT1	0.432145	1.00E-05	2.03E-04
CUL9	0.181316	1.01E-05	2.04E-04
LILRB1	0.597449	1.01E-05	2.05E-04
TOM1	0.311124	1.02E-05	2.06E-04
MYB	-0.36674	1.02E-05	2.06E-04
ZMYND11	0.405985	1.02E-05	2.06E-04
DLG1	0.658527	1.02E-05	2.06E-04
VAMP3	0.412154	1.03E-05	2.07E-04
GALNT6	0.619022	1.03E-05	2.07E-04
SRSF3	-0.3806	1.03E-05	2.07E-04
CXCL16	1.307033	1.04E-05	2.08E-04
TNIP2	0.379653	1.04E-05	2.08E-04
TMEM9	0.385376	1.05E-05	2.10E-04
BCL7C	0.568997	1.05E-05	2.10E-04
SLCO2B1	0.671908	1.05E-05	2.10E-04
RNF138	-0.41328	1.05E-05	2.10E-04
MCM3	0.603451	1.06E-05	2.11E-04
CAND1	-0.36926	1.06E-05	2.11E-04
TMEM219	0.301386	1.06E-05	2.11E-04
MYEOV2	0.188993	1.06E-05	2.11E-04
MGAT1	0.440132	1.07E-05	2.12E-04
C19orf70	0.625675	1.07E-05	2.12E-04
DROSHA	-0.32895	1.08E-05	2.15E-04
HERC5	0.622825	1.09E-05	2.15E-04
MAN1C1	0.892279	1.09E-05	2.16E-04
CYP1B1	1.524142	1.10E-05	2.17E-04
IDH1	0.580528	1.10E-05	2.18E-04
ADAM15	0.175091	1.11E-05	2.18E-04
SRSF8	-0.27553	1.11E-05	2.18E-04
GYG1	0.287734	1.11E-05	2.20E-04
CTSB	1.015713	1.12E-05	2.20E-04

TMEM37	0.623911	1.12E-05	2.21E-04
DHX58	0.390631	1.12E-05	2.21E-04
CTB-31O2(	-0.46144	1.13E-05	2.21E-04
RPP14	0.351951	1.13E-05	2.21E-04
PITPNA	0.38879	1.13E-05	2.21E-04
LRCH1	0.409833	1.13E-05	2.21E-04
ZNF511	0.394296	1.13E-05	2.21E-04
PPAP2A	0.627957	1.15E-05	2.25E-04
COPZ2	0.346831	1.15E-05	2.25E-04
PHF12	0.256774	1.16E-05	2.26E-04
NADK	0.334279	1.16E-05	2.27E-04
ZDHHC17	-0.21485	1.17E-05	2.27E-04
FXYD6	1.223557	1.17E-05	2.28E-04
SECTM1	0.597376	1.18E-05	2.28E-04
RP5-882O7	-0.07495	1.18E-05	2.28E-04
NFXL1	-0.35284	1.19E-05	2.31E-04
CD55	0.486164	1.19E-05	2.31E-04
FAM110B	0.264799	1.20E-05	2.33E-04
SLC48A1	0.76065	1.22E-05	2.35E-04
SELT	0.381251	1.22E-05	2.36E-04
MRPL57	-0.3108	1.22E-05	2.36E-04
ENPP2	1.277983	1.23E-05	2.37E-04
CEBPA-AS	-0.28178	1.23E-05	2.37E-04
MEAF6	-0.45541	1.23E-05	2.38E-04
STOM	0.513871	1.24E-05	2.39E-04
ZFP36L1	0.294624	1.25E-05	2.39E-04
LOC65478(	-0.18664	1.25E-05	2.39E-04
PEF1	0.345213	1.25E-05	2.39E-04
P2RX4	0.505328	1.25E-05	2.40E-04
DDX24	0.327	1.25E-05	2.40E-04
ORAI1	0.547086	1.27E-05	2.43E-04
DBP	0.32004	1.28E-05	2.45E-04
RP5-930J4	0.676739	1.28E-05	2.45E-04
C14orf169	0.413107	1.28E-05	2.45E-04
FRMD4A	0.344889	1.28E-05	2.45E-04
ADIPOR1	0.610381	1.29E-05	2.46E-04
ZNF490	0.256922	1.31E-05	2.49E-04
KCNJ10	0.35969	1.32E-05	2.51E-04
FIBCD1	-0.18072	1.32E-05	2.51E-04
TGFBI	1.683015	1.32E-05	2.51E-04
LOC10192	-0.69564	1.32E-05	2.51E-04
LRP5	0.13761	1.33E-05	2.52E-04
ANKRD9	0.49279	1.34E-05	2.53E-04
DRAP1	0.529415	1.34E-05	2.54E-04
SLC1A3	0.655495	1.35E-05	2.55E-04
ACAD8	0.396347	1.35E-05	2.55E-04
USO1	-0.31794	1.35E-05	2.55E-04
MAP2K2	0.397911	1.37E-05	2.58E-04
GCDH	0.543857	1.38E-05	2.59E-04
LOC10272!	-0.13672	1.39E-05	2.62E-04
PAQR9	0.306625	1.40E-05	2.63E-04
RPL37A	0.815725	1.42E-05	2.66E-04
NUFIP1	0.460769	1.43E-05	2.68E-04
LAMTOR4	0.514496	1.43E-05	2.68E-04
RP11-613M	-0.47591	1.43E-05	2.68E-04
GM2A	0.348117	1.46E-05	2.73E-04
MPP5	0.372654	1.46E-05	2.74E-04
UBN2	0.659466	1.47E-05	2.75E-04

RNF125	-0.58489	1.47E-05	2.75E-04
ZCCHC2	0.510337	1.48E-05	2.75E-04
SKI	0.717654	1.48E-05	2.76E-04
SEPN1	0.646	1.51E-05	2.81E-04
DIP2B	-0.61881	1.52E-05	2.83E-04
NUP214	0.397851	1.52E-05	2.84E-04
RXRA	0.5977	1.53E-05	2.84E-04
FNIP2	0.416841	1.53E-05	2.84E-04
NR5A1	-0.13031	1.53E-05	2.84E-04
RPL10L	-0.38571	1.53E-05	2.84E-04
BLVRB	1.001769	1.54E-05	2.85E-04
NTAN1	0.484511	1.54E-05	2.85E-04
LY96	1.479252	1.55E-05	2.87E-04
COX7B	0.453367	1.57E-05	2.90E-04
HIATL1	-0.24778	1.57E-05	2.90E-04
PRR29	-0.16515	1.59E-05	2.94E-04
RP11-792A	0.224125	1.60E-05	2.94E-04
TIMP2	0.709039	1.60E-05	2.95E-04
CDYL	0.436774	1.60E-05	2.95E-04
IRAK1	0.166349	1.61E-05	2.95E-04
MRPL21	0.580397	1.61E-05	2.96E-04
IPMK	-0.24924	1.62E-05	2.98E-04
PHF14	-0.27248	1.63E-05	2.99E-04
MAPK3	0.220616	1.64E-05	3.01E-04
SLC50A1	0.474849	1.65E-05	0.000302
JAZF1	0.841337	1.66E-05	3.03E-04
A2M	0.825834	1.68E-05	3.08E-04
EIF3M	-0.54441	1.71E-05	3.11E-04
NSF	-0.25605	1.71E-05	3.11E-04
GAMT	0.569954	1.71E-05	3.11E-04
P2RY13	1.155224	1.72E-05	3.13E-04
RAMP2-AS	-0.13234	1.72E-05	3.13E-04
ASPSCR1	0.41308	1.72E-05	3.13E-04
NMT1	0.444699	1.73E-05	3.13E-04
GFRA2	0.693631	1.73E-05	3.14E-04
CDCA7L	-0.39315	1.74E-05	3.16E-04
MORF4L1	0.228925	1.76E-05	3.20E-04
C4orf27	-0.16829	1.77E-05	3.21E-04
FAM71A	-0.16285	1.80E-05	3.25E-04
WDR81	0.311688	1.80E-05	3.25E-04
LIN7B	0.244968	1.80E-05	3.26E-04
PDE4DIP	0.504623	1.81E-05	3.27E-04
RMDN3	0.356939	1.82E-05	3.27E-04
CTU2	-0.20721	1.83E-05	3.30E-04
SNX2	0.487961	1.85E-05	3.33E-04
COX6A1	0.263532	1.85E-05	3.34E-04
CCL8	1.421139	1.86E-05	3.35E-04
RIN1	-0.23588	1.87E-05	3.36E-04
BRD4	0.375092	1.87E-05	3.36E-04
XPO4	0.381263	1.88E-05	3.37E-04
SIPA1	0.605789	1.88E-05	3.37E-04
B2M	0.363294	1.89E-05	3.37E-04
NDFIP1	0.739173	1.89E-05	3.37E-04
TCF7L2	0.682599	1.89E-05	3.37E-04
RPGR	-0.4652	1.92E-05	3.44E-04
NUP133	-0.18045	1.94E-05	3.45E-04
CTDSP1	0.304389	1.94E-05	3.46E-04
GPR137B	1.149247	1.96E-05	3.48E-04

MAX	0.639925	1.98E-05	3.53E-04
P2RY6	0.349649	1.99E-05	3.53E-04
SAE1	0.587131	1.99E-05	3.53E-04
LCP1	0.454863	1.99E-05	3.54E-04
CDKAL1	0.42072	1.99E-05	3.54E-04
NCF4	0.742068	2.00E-05	3.54E-04
GNPTG	0.379629	2.00E-05	3.54E-04
SERPINA1	0.643002	2.00E-05	3.54E-04
TGFBR2	0.283875	2.02E-05	3.57E-04
TMEM200I	0.45605	2.03E-05	3.59E-04
FKBP4	0.381796	2.07E-05	3.64E-04
MOB1A	0.382118	2.07E-05	3.64E-04
SAMD4A	0.186703	2.10E-05	3.71E-04
PPP1R14B	0.473589	2.11E-05	3.72E-04
KCNAB1	0.380443	2.12E-05	3.73E-04
BNIP3	0.40029	2.12E-05	3.73E-04
UNC13B	0.599402	2.12E-05	3.73E-04
FANCG	-0.40177	2.13E-05	3.73E-04
MRPS36	0.473299	2.13E-05	3.74E-04
HLA-DQA1	1.309084	2.14E-05	3.75E-04
AK1	0.509752	2.14E-05	3.75E-04
SF3B6	0.28101	2.15E-05	3.76E-04
SNX1	0.469564	2.17E-05	3.79E-04
MID1IP1	0.566493	2.18E-05	3.81E-04
CD274	0.56391	2.21E-05	3.85E-04
PSMD5-AS	0.410464	2.21E-05	3.85E-04
MRPL12	0.375342	2.21E-05	3.85E-04
DNAJC16	0.592031	2.22E-05	3.86E-04
JUND	0.678983	2.22E-05	3.87E-04
HEATR2	0.43716	2.23E-05	3.88E-04
RP11-1191	-0.40431	2.24E-05	3.88E-04
TBC1D17	0.194042	2.24E-05	3.89E-04
ZSWIM6	0.454881	2.24E-05	3.89E-04
YPEL3	0.560006	2.27E-05	3.93E-04
TMEM144	0.29038	2.30E-05	3.97E-04
CHRNB1	0.252085	2.30E-05	3.98E-04
FHOD1	0.520757	2.34E-05	4.05E-04
APOA1BP	0.414412	2.34E-05	4.05E-04
GABPB1	0.35593	2.35E-05	4.06E-04
IL13RA1	0.593791	2.36E-05	4.07E-04
ZNF682	-0.34732	2.37E-05	4.08E-04
ZNF264	-0.86506	2.37E-05	4.08E-04
RNF168	-0.22681	2.44E-05	4.19E-04
TMCO4	0.241059	2.48E-05	4.26E-04
SUZ12	-0.7803	2.48E-05	4.26E-04
LILRA6	0.255991	2.48E-05	4.26E-04
BCL2L2	0.147454	2.50E-05	4.29E-04
PCGF5	0.61107	2.51E-05	4.30E-04
TIMM21	-0.38545	2.52E-05	4.32E-04
GPR146	0.654651	2.53E-05	4.33E-04
ACTN1	0.585194	2.54E-05	4.34E-04
CDC42BPB	0.306177	2.54E-05	4.34E-04
ZMYM1	-0.45098	2.56E-05	4.37E-04
KMO	1.170674	2.56E-05	4.37E-04
KLHDC7B	0.489139	2.56E-05	4.37E-04
NSRP1	-0.30811	2.58E-05	4.39E-04
GSTO1	0.284414	2.60E-05	4.43E-04
P4HB	0.574761	2.63E-05	4.47E-04

ZFAND2B	0.336164	2.68E-05	4.54E-04
TSPAN14	0.513331	2.68E-05	4.54E-04
SLC9A9	0.633556	2.68E-05	4.54E-04
MAPKAPK!	0.380534	2.68E-05	4.54E-04
CPSF7	0.239369	2.68E-05	4.54E-04
NEURL4	0.455939	2.69E-05	4.54E-04
SUCO	-0.3039	2.69E-05	4.54E-04
VSIG4	1.773356	2.70E-05	4.56E-04
BCL2L12	0.501136	2.71E-05	4.58E-04
TOP1	0.347805	2.71E-05	4.58E-04
HLA-DRA	0.562378	2.74E-05	4.62E-04
DPM2	0.421078	2.77E-05	4.67E-04
INIP	0.321305	2.80E-05	4.71E-04
ANXA5	0.942409	2.81E-05	4.73E-04
PCYOX1	0.473713	2.82E-05	4.73E-04
DCXR	0.490744	2.82E-05	4.73E-04
PHKA2	-0.27569	2.84E-05	4.76E-04
PPP6R1	0.422852	2.84E-05	4.76E-04
EBP	-0.52605	2.85E-05	4.77E-04
PXN	0.244437	2.86E-05	4.78E-04
AGO2	0.511185	2.86E-05	4.78E-04
SELENBP1	1.581997	2.86E-05	4.78E-04
KATNAL1	-0.33821	2.86E-05	4.78E-04
BATF	0.408959	2.88E-05	4.81E-04
FAS	0.549505	2.89E-05	4.82E-04
ALYREF	0.756761	2.89E-05	4.82E-04
ACTB	0.363627	2.90E-05	4.83E-04
ERRFI1	0.616896	2.90E-05	4.83E-04
PILRA	0.433462	2.91E-05	4.83E-04
AC091133.	-0.38197	2.95E-05	4.89E-04
RNASEH2E	-0.39628	2.95E-05	4.89E-04
MZT2B	-0.38484	2.95E-05	4.89E-04
GLRX	0.578872	2.95E-05	4.89E-04
MMGT1	-0.29273	2.99E-05	4.95E-04
CAPZA2	0.56139	3.00E-05	4.96E-04
UBE2A	0.248337	3.03E-05	5.01E-04
RAP2B	0.641962	3.03E-05	5.01E-04
RSBN1	-0.35748	3.04E-05	5.01E-04
ARIH2	0.395079	3.04E-05	5.01E-04
STXBP3	-0.51048	3.04E-05	5.01E-04
10-Sep	0.843258	3.05E-05	5.02E-04
TBC1D13	0.350094	3.07E-05	5.06E-04
MOB3A	0.345313	3.08E-05	5.06E-04
CRIM1	-0.65214	3.09E-05	5.07E-04
RPS7	0.422812	3.10E-05	5.09E-04
SRXN1	0.713223	3.20E-05	5.25E-04
CYBA	0.365426	3.20E-05	5.25E-04
UBA2	-0.30886	3.21E-05	5.26E-04
YPEL4	0.825726	3.21E-05	5.26E-04
MTA3	0.196561	3.23E-05	5.28E-04
C14orf119	0.458302	3.23E-05	5.29E-04
GTF2H3	-0.42168	3.24E-05	5.29E-04
UBA3	-0.28087	3.25E-05	5.31E-04
SMCHD1	-0.37115	3.28E-05	5.35E-04
CHD1L	-0.37058	3.31E-05	5.40E-04
ACOT4	0.654825	3.33E-05	5.43E-04
SLMO2	-0.49837	3.34E-05	5.43E-04
OTUD6B-A	-0.34726	3.34E-05	5.43E-04

EIF3F	-0.58172	3.35E-05	5.45E-04
RNPEPL1	0.175689	3.39E-05	5.50E-04
FIS1	0.523875	3.39E-05	5.50E-04
NDUFB10	0.454034	3.41E-05	5.53E-04
SSU72	0.343799	3.42E-05	5.54E-04
HHLA3	0.270508	3.42E-05	5.54E-04
NPR3	-0.80975	3.43E-05	5.54E-04
ELL2	0.74578	3.49E-05	5.64E-04
C6orf89	0.530279	3.51E-05	5.66E-04
C12orf65	0.479536	3.53E-05	5.69E-04
MOV10	0.409496	3.55E-05	5.72E-04
PECAM1	0.520858	3.56E-05	5.73E-04
STX11	0.58764	3.59E-05	5.78E-04
DPY19L2P2	-0.62346	3.60E-05	5.80E-04
GYPB	0.87815	3.61E-05	5.80E-04
SIGLEC7	0.630893	3.62E-05	5.81E-04
LINC01128	0.145534	3.63E-05	5.83E-04
GSTK1	0.414971	3.64E-05	5.83E-04
UBE2O	0.302309	3.67E-05	5.88E-04
C9orf16	0.700645	3.67E-05	5.89E-04
BC030152	-0.60366	3.74E-05	5.99E-04
RPUSD4	-0.49404	3.76E-05	6.02E-04
RPS27A	-0.5414	3.78E-05	6.04E-04
EPS8	1.315713	3.79E-05	6.06E-04
HSD17B14	0.549527	3.81E-05	6.08E-04
SON	0.454189	3.82E-05	6.09E-04
CYBB	0.539129	3.82E-05	6.09E-04
RAPH1	0.333906	3.83E-05	6.10E-04
CCDC159	0.469897	3.84E-05	6.12E-04
TSPO2	0.918837	3.87E-05	6.16E-04
YBX3	0.290229	3.88E-05	6.16E-04
DDR1-AS1	-0.08218	3.88E-05	6.16E-04
FARSB	-0.70635	3.89E-05	6.17E-04
CHST7	0.392511	3.89E-05	6.17E-04
MSR1	0.278484	3.93E-05	6.23E-04
MIR302B	0.455294	3.98E-05	6.29E-04
RRP12	-0.39744	4.00E-05	6.32E-04
FYB	1.007364	4.00E-05	6.32E-04
AKNA	0.287065	4.04E-05	6.38E-04
CASP1	0.657563	4.05E-05	6.40E-04
ZDHHC4	0.264181	4.06E-05	6.40E-04
C19orf53	0.402127	4.08E-05	6.43E-04
SRGAP2C	0.606165	4.09E-05	6.45E-04
PSMB3	0.370061	4.12E-05	6.48E-04
C3AR1	1.048189	4.13E-05	6.49E-04
BECN1	0.297194	4.13E-05	6.49E-04
ARPC1A	0.484462	4.19E-05	6.57E-04
SERPINF1	0.780831	4.19E-05	6.57E-04
CD5L	1.818185	4.19E-05	6.57E-04
RP3-486D2	-0.28533	4.22E-05	6.61E-04
PHF1	0.291344	4.22E-05	6.61E-04
SIGLEC5	0.428321	4.23E-05	6.61E-04
DHDDS	0.44646	4.23E-05	6.61E-04
CLPP	0.402759	4.24E-05	6.63E-04
TMEM143	-0.18355	4.25E-05	6.64E-04
GPR65	0.822893	4.26E-05	6.65E-04
ZDHHC15	-0.60876	4.28E-05	6.67E-04
LOC72916	-0.165	4.28E-05	6.67E-04

MARCKSL1	0.688648	4.30E-05	6.69E-04
SAFB2	0.275101	4.33E-05	6.73E-04
KIF21B	0.436923	4.36E-05	6.78E-04
FABP4	1.10981	4.39E-05	6.81E-04
PLAUR	0.917847	4.39E-05	6.82E-04
EBI3	0.640543	4.39E-05	6.82E-04
ELAVL1	0.306658	4.40E-05	6.82E-04
PTPRA	0.478601	4.41E-05	6.84E-04
MGRN1	0.27522	4.44E-05	6.87E-04
PBRM1	0.332184	4.44E-05	6.87E-04
BASP1	0.596938	4.46E-05	6.89E-04
C10orf25	-0.87176	4.51E-05	6.97E-04
RPS6KA1	0.383424	4.53E-05	6.99E-04
HECTD3	0.40852	4.53E-05	6.99E-04
CDK6	-0.44369	4.65E-05	7.17E-04
IL10RB	0.402257	4.66E-05	7.17E-04
CYB561	0.337138	4.69E-05	7.22E-04
KIAA1143	-0.20137	4.70E-05	7.23E-04
SLC38A9	-0.42674	4.73E-05	7.26E-04
CBX7	0.480559	4.75E-05	7.29E-04
ZNF639	-0.34262	4.75E-05	7.29E-04
LINC00535	-0.11262	4.76E-05	7.29E-04
MFSD12	0.286391	4.76E-05	7.30E-04
KIAA0141	0.421833	4.77E-05	7.30E-04
TMEM19	0.271626	4.77E-05	7.30E-04
DOK2	0.922379	4.77E-05	7.30E-04
COPS7A	0.405515	4.78E-05	7.31E-04
WRNIP1	-0.33185	4.79E-05	7.32E-04
CPVL	0.897736	4.81E-05	7.33E-04
ERAL1	0.504058	4.81E-05	7.34E-04
SLAIN2	0.429347	4.84E-05	7.37E-04
BACE2	0.955714	4.84E-05	7.37E-04
ZNF76	0.273616	4.85E-05	7.37E-04
GIMAP8	0.641906	4.85E-05	7.37E-04
PLBD1	1.544245	4.85E-05	7.37E-04
BANP	-0.38456	4.86E-05	7.38E-04
DPH3	0.338065	4.86E-05	7.38E-04
CCNB1IP1	-0.42898	4.87E-05	7.39E-04
TMEM55B	0.393713	4.88E-05	7.39E-04
WDR45	0.36966	4.90E-05	7.42E-04
CNN3	0.957573	4.90E-05	7.42E-04
MAPK13	0.287517	4.93E-05	7.45E-04
SIGLEC16	0.562828	4.93E-05	7.45E-04
CARS	0.328899	4.94E-05	7.45E-04
SIPA1L3	0.098546	4.94E-05	7.45E-04
LZTS2	0.214428	4.94E-05	7.45E-04
MRAS	0.504729	4.95E-05	7.45E-04
ZNF320	-0.37045	4.95E-05	7.45E-04
MAN2B1	0.497317	4.95E-05	7.45E-04
NDUFS7	0.268506	4.96E-05	7.45E-04
UBTF	0.511766	5.02E-05	7.55E-04
IFNAR1	0.369249	5.04E-05	7.56E-04
IFIT5	0.55044	5.04E-05	7.56E-04
SYNC	-0.72207	5.04E-05	7.56E-04
PRKAG2	0.365571	5.05E-05	7.56E-04
LOC10028	0.627928	5.07E-05	7.60E-04
OGFOD1	0.375603	5.09E-05	7.61E-04
ANP32E	-0.47859	5.13E-05	7.67E-04

TMEM44	-0.54407	5.16E-05	7.71E-04
TCHH	0.951169	5.17E-05	7.72E-04
AC004941.	0.389792	5.21E-05	7.77E-04
MFSD6	0.444155	5.21E-05	7.77E-04
PGRMC2	-0.32998	5.22E-05	7.77E-04
VPS13C	-0.52766	5.22E-05	7.77E-04
KIAA0895L	0.315491	5.22E-05	7.78E-04
NDUFA4	0.27102	5.24E-05	7.79E-04
M6PR	0.468583	5.24E-05	7.79E-04
PRKAR1A	0.371533	5.25E-05	7.80E-04
SLC4A7	-0.30604	5.28E-05	7.83E-04
GTPBP4	-0.36274	5.37E-05	7.96E-04
CCND2	-0.621	5.38E-05	7.97E-04
DENND6B	0.329922	5.39E-05	7.99E-04
CCDC124	0.17772	5.44E-05	8.06E-04
TBC1D4	0.7873	5.45E-05	8.06E-04
EMP2	0.405489	5.49E-05	8.11E-04
ZNF551	-0.15166	5.52E-05	8.16E-04
ATP6AP1	0.408146	5.59E-05	8.25E-04
NME4	0.521469	5.60E-05	8.26E-04
GCLM	0.55721	5.68E-05	8.38E-04
FAM167B	0.389821	5.74E-05	8.45E-04
CA2	1.280796	5.74E-05	8.45E-04
PARP14	0.228095	5.75E-05	8.45E-04
SLC15A3	0.87539	5.75E-05	8.45E-04
BAK1	0.316669	5.75E-05	8.45E-04
SIGLEC11	0.426763	5.75E-05	8.45E-04
LINC01023	-0.37074	5.79E-05	8.49E-04
FUOM	0.406612	5.79E-05	8.49E-04
PLGRKT	0.565554	5.91E-05	8.66E-04
FAXC	0.230252	5.91E-05	8.66E-04
CAPNS1	0.593888	5.95E-05	8.71E-04
UFM1	-0.45239	5.96E-05	8.71E-04
ARF4	0.290676	5.96E-05	8.71E-04
PATL1	0.256603	5.96E-05	8.71E-04
ZNF432	-0.38644	5.98E-05	8.73E-04
EXOSC2	0.654681	6.09E-05	8.88E-04
BRD1	-0.32836	6.13E-05	8.93E-04
SNAPIN	0.338389	6.17E-05	8.98E-04
FEZ1	0.814979	6.20E-05	9.01E-04
PCF11	-0.27916	6.20E-05	9.01E-04
GUSBP2	0.441498	6.25E-05	9.08E-04
SELK	0.337676	6.25E-05	9.08E-04
NPAT	0.482333	6.26E-05	9.08E-04
ZBTB21	-0.58089	6.26E-05	9.08E-04
SBNO1	0.424756	6.26E-05	9.08E-04
ERGIC2	0.370993	6.33E-05	9.17E-04
FAM102A	0.205582	6.36E-05	9.21E-04
RPS19BP1	0.378541	6.36E-05	9.21E-04
HSBP1L1	0.441614	6.39E-05	9.24E-04
FOLR2	0.728559	6.40E-05	9.24E-04
ZNF267	-0.60861	6.42E-05	9.27E-04
RNF216	0.290375	6.43E-05	9.28E-04
ANKRD11	0.32716	6.46E-05	9.31E-04
KLHDC1	-0.31474	6.47E-05	9.32E-04
ISOC2	0.472924	6.48E-05	9.34E-04
ECHDC3	0.446846	6.49E-05	9.34E-04
SKP1	0.279377	6.49E-05	9.34E-04

PNKD	0.529009	6.54E-05	9.40E-04
AP5M1	-0.47097	6.55E-05	9.40E-04
AF520793	-0.53914	6.55E-05	9.40E-04
KIAA1191	0.286052	6.59E-05	9.45E-04
VEGFA	0.259112	6.59E-05	9.45E-04
EVA1B	0.522462	6.62E-05	9.49E-04
CKMT2-AS	-0.35909	6.64E-05	9.51E-04
PATZ1	0.207874	6.69E-05	9.57E-04
RSAD1	0.394231	6.71E-05	9.59E-04
UNC93B1	0.309147	6.72E-05	9.60E-04
PCM1	-0.32332	6.75E-05	9.63E-04
PDE9A	0.261042	6.76E-05	9.65E-04
SMAD2	-0.22853	6.81E-05	9.71E-04
SEPT7P2	-0.3108	6.88E-05	9.80E-04
SMU1	0.322611	6.89E-05	9.80E-04
ST8SIA4	0.690157	6.89E-05	9.80E-04
UHRF2	-0.28571	6.90E-05	9.81E-04
TMEM110	0.238237	6.98E-05	9.92E-04
ACSF3	0.382454	7.00E-05	9.93E-04
PODNL1	-0.12579	7.00E-05	9.93E-04
CCDC53	0.329427	7.04E-05	9.98E-04
NMB	0.330855	7.04E-05	9.98E-04
MRPL23	0.285451	7.07E-05	0.001001
ASCC3	-0.357	7.11E-05	0.001006
AAED1	0.459789	7.12E-05	0.001008
TRAM1	0.36394	7.14E-05	0.001009
GSR	0.447635	7.14E-05	0.001009
TCN2	0.421378	7.15E-05	0.00101
PDIK1L	-0.35712	7.21E-05	0.001017
NDUFA11	0.630073	7.31E-05	0.00103
RPS10P7	-0.60177	7.32E-05	0.001031
MPEG1	1.470588	7.35E-05	0.001034
NFE2L2	0.442061	7.35E-05	0.001034
MRPS18B	0.404759	7.36E-05	0.001034
RAC2	0.487288	7.38E-05	0.001037
SPI1	0.286476	7.39E-05	0.001037
LOC10031(	-0.43573	7.39E-05	0.001037
CD63	0.419407	7.42E-05	0.00104
FCGR3B	1.362214	7.42E-05	0.00104
MLXIP	0.283891	7.52E-05	0.001054
CCDC77	-0.45368	7.58E-05	0.00106
TNRC6A	0.354546	7.58E-05	0.00106
COX10-AS	-0.33142	7.60E-05	0.001063
MAPK8	-0.39243	7.61E-05	0.001063
DENND1A	0.4348	7.63E-05	0.001065
FAHD1	0.341819	7.69E-05	0.001072
GPR34	1.359204	7.69E-05	0.001072
SNRPE	0.589211	7.71E-05	0.001075
PLOD1	0.426608	7.73E-05	0.001076
ZC3H18	0.32728	7.74E-05	0.001077
COMMD4	0.545379	7.76E-05	0.001079
TLN1	0.40199	7.77E-05	0.001079
UBIAD1	0.348562	7.77E-05	0.001079
ZNF37BP	-0.47623	7.78E-05	0.001079
PQLC2	0.323295	7.83E-05	0.001086
MANEA	-0.22923	7.85E-05	0.001089
CNNM1	0.162221	7.86E-05	0.001089
LOC10050	0.545123	7.88E-05	0.001091

KCTD12	1.103194	7.89E-05	0.001091
MAVS	0.327937	7.90E-05	0.001092
POLR2C	0.366428	7.90E-05	0.001092
MEGF9	0.377278	7.92E-05	0.001094
GPX1	0.420093	7.93E-05	0.001095
TRDN	0.326918	7.94E-05	0.001095
CHCHD2	0.375803	7.95E-05	0.001095
TCF7L1	0.363251	7.95E-05	0.001095
UBE2L6	0.374688	7.96E-05	0.001096
GANAB	0.448245	8.05E-05	0.001107
SF3B5	0.37822	8.07E-05	0.001109
SC5D	-0.20428	8.09E-05	0.001112
RBMS2	0.288465	8.11E-05	0.001113
KLC2	0.223752	8.11E-05	0.001114
GSN	0.097533	8.15E-05	0.001118
CRISPLD1	-0.46527	8.18E-05	0.001121
CDC7	-0.49252	8.20E-05	0.001122
MORC2	0.446614	8.20E-05	0.001122
METTL7B	0.77629	8.21E-05	0.001123
CCAR2	-0.45831	8.22E-05	0.001123
TIMD4	2.071132	8.24E-05	0.001126
MORN2	0.433109	8.25E-05	0.001127
LOC15896(	0.466736	8.29E-05	0.001131
APBA3	0.147085	8.31E-05	0.001133
FBXO46	0.340259	8.32E-05	0.001133
LOC10013	-0.46414	8.37E-05	0.00114
TUBB3	0.467779	8.39E-05	0.001142
MYO1D	0.305386	8.39E-05	0.001142
GSTA1	-0.19497	8.41E-05	0.001142
FAM20B	0.297548	8.41E-05	0.001142
PYCARD	0.421853	8.41E-05	0.001142
LPPR3	0.277833	8.43E-05	0.001144
SCYL1	0.149947	8.44E-05	0.001144
C4orf48	0.443669	8.54E-05	0.001157
CCDC132	0.380078	8.55E-05	0.001157
ARL4D	-0.08225	8.55E-05	0.001157
COQ10A	-0.41516	8.61E-05	0.001164
RNF126	0.316985	8.64E-05	0.001168
RALY	0.391781	8.66E-05	0.00117
CD163	1.948512	8.67E-05	0.001171
SAMHD1	1.133923	8.73E-05	0.001178
HMG20B	0.477	8.77E-05	0.001182
NMD3	-0.27268	8.81E-05	0.001187
HIST1H4I	-0.20769	8.82E-05	0.001187
HCAR3	1.240041	8.88E-05	0.001195
NUDT3	0.449384	8.91E-05	0.001199
SCRIB	0.355775	8.92E-05	0.001199
GLCCI1	0.350583	8.92E-05	0.001199
LOH12CR1	0.374861	8.94E-05	0.0012
ZNF829	-0.19409	9.01E-05	0.001208
ZNF280C	-0.3807	9.03E-05	0.001211
TCEB2	0.36058	9.04E-05	0.001212
ZNF438	0.279552	9.06E-05	0.001213
SYNGR2	0.232805	9.07E-05	0.001213
BC069776	0.120843	9.11E-05	0.001219
ACACB	-0.32552	9.14E-05	0.001222
NF2	0.091683	9.17E-05	0.001225
FKBP11	0.75252	9.21E-05	0.001229

GAPT	-0.72261	9.25E-05	0.001234
SLC7A7	1.325108	9.25E-05	0.001234
ERLEC1	0.269212	9.31E-05	0.001241
EDF1	0.292001	9.35E-05	0.001245
LDHB	-0.19687	9.36E-05	0.001245
AAGAB	0.411642	9.47E-05	0.00126
RANBP6	-0.37745	9.54E-05	0.001268
LPIN2	0.353797	9.56E-05	0.00127
STRN4	0.359937	9.57E-05	0.00127
PIGT	-0.23175	9.58E-05	0.001271
PINK1	0.390597	9.62E-05	0.001275
CPNE2	0.474993	9.63E-05	0.001276
RAB5A	0.392598	9.64E-05	0.001277
DDX52	-0.25281	9.72E-05	0.001287
RELA	0.333914	9.76E-05	0.001291
HIF1AN	0.267556	9.81E-05	0.001297
LIN37	0.197144	9.89E-05	0.001307
ABR	0.285652	9.95E-05	0.001314
SCAMP4	0.146242	9.97E-05	0.001316
TRAPPC12	0.366508	9.99E-05	0.001317
LINC00847	0.251885	1.00E-04	0.001319
SLC46A1	0.354684	1.01E-04	0.001324
RAVER1	0.480797	1.01E-04	0.00133
PHF23	0.314796	1.01E-04	0.00133
RPS15	-0.0897	1.02E-04	0.001335
FAM20C	0.249786	1.02E-04	0.001336
CYB5A	0.448462	1.03E-04	0.001354
ABHD6	0.28025	1.04E-04	0.00136
PKN1	0.497374	1.04E-04	0.001367
LOC10050	-0.42283	1.05E-04	0.001374
TEX264	0.460889	1.05E-04	0.001377
INTS3	0.204979	1.05E-04	0.001377
IL15	0.64214	1.05E-04	0.001379
COPS2	-0.28835	1.06E-04	0.001388
CELF1	0.342193	1.06E-04	0.00139
FAM13B	-0.34272	1.06E-04	0.00139
NCKAP5	0.423005	1.07E-04	0.001401
TNK1	-0.13573	1.08E-04	0.001404
ISG20L2	0.283857	1.08E-04	0.001404
RENBP	0.264115	1.08E-04	0.001407
IL10RB-AS	0.374154	1.09E-04	0.001416
PARG	-0.24984	1.09E-04	0.001417
ADI1	0.553104	1.09E-04	0.001417
CFDP1	-0.40366	1.09E-04	0.001417
CD37	0.425619	1.10E-04	0.001427
RBP1	0.235556	1.10E-04	0.001429
SHARPIN	0.2906	0.000111	0.001436
ZNF180	-0.25366	1.12E-04	0.001451
UBQLN1	0.360837	1.12E-04	0.001457
RAB5B	0.398243	1.13E-04	0.00146
PPFIA1	0.275422	1.13E-04	0.00146
NR2C1	-0.24129	1.14E-04	0.001471
CADPS2	0.862389	1.14E-04	0.001476
PGAP3	0.287737	1.15E-04	0.00148
SLC2A8	0.250941	1.15E-04	0.00149
CHCHD6	0.435562	1.16E-04	0.0015
MZT1	0.198645	1.17E-04	0.001514
ABHD5	0.525539	1.19E-04	0.001529

FAM13A	0.44102	1.19E-04	0.00153
JADE3	-0.32667	1.19E-04	0.001531
FMO4	0.453802	1.19E-04	0.001537
TXNDC12	0.249341	1.20E-04	0.001544
HSPH1	0.637424	1.20E-04	0.001545
VPS53	0.284302	1.21E-04	0.001549
DNMT3A	0.44363	1.21E-04	0.001551
HVCN1	0.473396	1.21E-04	0.001554
GABARAPL	0.284753	1.21E-04	0.001554
RPS15A	-0.55521	1.22E-04	0.001562
CSRP1	0.342826	1.22E-04	0.001567
HINT2	0.370786	1.23E-04	0.001569
HMGCR	-0.36066	1.23E-04	0.001572
PRKACA	0.083867	1.23E-04	0.001573
PLXND1	0.452684	1.23E-04	0.001574
PRIMPOL	-0.26504	1.23E-04	0.001574
TRAPPC3	0.314114	1.23E-04	0.001574
THUMPD1	-0.38636	1.24E-04	0.001574
PLA2G7	1.607874	1.24E-04	0.001574
PRDM4	0.210028	1.24E-04	0.001582
RAD9A	0.316053	1.24E-04	0.001583
EHD1	0.262811	1.24E-04	0.001583
SMARCAD	-0.28398	1.25E-04	0.001591
TMEM57	0.386403	1.25E-04	0.001594
HSPA14	-0.42623	1.25E-04	0.001594
FAM210B	0.47709	1.26E-04	0.001598
SURF1	0.268819	1.26E-04	0.0016
NDUFV3	0.369247	1.28E-04	0.001619
TBC1D8	0.24104	1.28E-04	0.001622
SLC37A2	0.519262	1.29E-04	0.001635
ATP5O	-0.1465	1.30E-04	0.00164
RP11-119F	-0.6185	1.30E-04	0.001646
CCNG2	0.494772	1.32E-04	0.001667
6-Mar	-0.25303	1.32E-04	0.001674
LPCAT1	0.432346	1.34E-04	0.001689
RPP25	0.375474	1.34E-04	0.001689
PAPOLA	-0.30543	1.34E-04	0.001691
FAM120AC	0.327733	1.34E-04	0.001691
MYO1C	0.189524	1.36E-04	0.001708
GAS2L3	0.417322	1.36E-04	0.001716
ZNF680	-0.40296	1.38E-04	0.001732
ENTPD1	0.728375	1.38E-04	0.001732
LOC10050(	-0.10821	1.38E-04	0.001735
YIPF3	0.520454	1.38E-04	0.00174
GPANK1	0.224963	1.39E-04	0.00174
TDRKH	0.446453	1.39E-04	0.00175
ANKRA2	-0.41142	1.40E-04	0.001756
ACVRL1	0.339694	1.40E-04	0.001757
SMARCB1	0.259239	1.41E-04	0.001761
PMP2	0.526845	1.41E-04	0.001761
IFI27L1	-0.45707	1.41E-04	0.001761
SMIM7	0.253803	1.42E-04	0.001776
COMMD1	0.41541	1.42E-04	0.001779
COMMD9	0.317972	1.42E-04	0.001779
NAA38	0.467562	1.43E-04	0.001779
HOXC8	-0.19522	1.44E-04	0.001791
PPM1N	0.259997	1.44E-04	0.001792
PGGT1B	-0.52738	1.44E-04	0.001796

BAG5	0.713634	1.45E-04	0.0018
ZDHHC12	0.285264	1.45E-04	0.0018
IFT81	-0.20682	1.45E-04	0.001803
AGTRAP	0.569924	1.45E-04	0.001803
PDCD1LG2	0.303188	1.45E-04	0.001804
RP4-612B1	-0.3089	1.46E-04	0.001813
RINT1	-0.36351	1.46E-04	0.001817
ARMCX6	-0.1913	1.46E-04	0.001817
CDK2	0.358724	0.000147	0.001818
CENPB	0.231048	1.47E-04	0.001819
FCGR2B	0.682224	1.47E-04	0.001825
NDUFC1	0.251243	1.48E-04	0.00183
CXCL12	1.792914	1.48E-04	0.001834
SEC24C	0.308549	1.48E-04	0.001834
ALKBH7	0.334855	1.49E-04	0.001839
FHDC1	0.70827	1.49E-04	0.001839
FAM160A2	0.341986	1.49E-04	0.001843
QSER1	-0.34617	1.49E-04	0.001843
PPT1	0.265513	1.50E-04	0.001846
RNGTT	-0.34079	1.50E-04	0.001849
SGSH	0.469514	1.50E-04	0.001849
POT1	-0.30922	1.51E-04	0.001855
CTSH	1.046712	1.51E-04	0.001857
MYOF	0.500888	1.51E-04	0.001862
HTATSF1	0.322717	1.52E-04	0.001867
GTF3C1	0.276189	1.52E-04	0.001867
UNC119	0.256542	1.53E-04	0.001873
IQCE	0.170525	1.53E-04	0.001875
NFYA	0.269269	1.53E-04	0.001876
DNAJC8	-0.30168	1.53E-04	0.001877
BPNT1	-0.3235	1.53E-04	0.001877
PAPOLG	0.356386	1.53E-04	0.001877
IFT27	0.336273	1.55E-04	0.001895
RP11-124L	-0.54868	1.56E-04	0.001907
GATAD1	0.444444	1.56E-04	0.001908
UBE2G2	0.287878	1.56E-04	0.001909
CTC-444N	-0.53367	1.57E-04	0.001921
XAGE2	-0.17152	1.59E-04	0.001938
HEG1	0.369578	1.59E-04	0.001938
NUMBL	-0.16284	1.59E-04	0.001938
ZBTB4	0.245097	1.59E-04	0.001938
SAMD1	0.16089	1.60E-04	0.00195
PROSER2	-0.36887	1.61E-04	0.001952
TFAM	0.633129	1.61E-04	0.001952
LGMN	1.514198	1.61E-04	0.001956
GALNT10	0.481659	1.61E-04	0.001957
PPWD1	-0.30216	1.61E-04	0.001958
TNFSF4	-0.76811	1.62E-04	0.001961
MRPS11	0.214108	1.62E-04	0.001964
UBE2K	-0.25045	1.63E-04	0.001971
MED4	-0.31755	1.63E-04	0.001976
ZNF787	0.211197	1.63E-04	0.001976
PARK7	-0.21322	1.64E-04	0.001984
PPP1R15A	0.716268	1.65E-04	0.001995
TP53TG1	0.388386	1.65E-04	0.001998
CTNNA1	0.402253	1.66E-04	0.002002
CUL4B	0.383831	1.66E-04	0.002003
L3HYPDH	0.482495	1.66E-04	0.002003

RETSAT	-0.42545	1.68E-04	0.00203
PIM2	0.412595	1.70E-04	0.002042
CSGALNAC	-0.59578	1.70E-04	0.002044
MCM2	-0.48748	1.70E-04	0.002045
ACKR1	0.577127	1.71E-04	0.002051
KPNA6	0.257875	1.71E-04	0.002051
LASP1	0.274405	1.71E-04	0.002054
LA16c-380	-0.28921	1.71E-04	0.002058
FCN1	1.362509	1.72E-04	0.002059
MORC2-AS	-0.51076	1.72E-04	0.002059
PRPF4B	-0.42451	1.73E-04	0.00207
RTTN	-0.56309	1.73E-04	0.00207
TNFAIP2	0.424129	1.74E-04	0.002079
PUSL1	0.464973	1.75E-04	0.00209
KEAP1	0.388467	1.75E-04	0.002091
NRBF2	0.364222	1.75E-04	0.002097
TOPBP1	-0.30304	1.76E-04	0.002106
MS4A6A	1.345185	1.77E-04	0.002108
CLCN5	0.172569	1.78E-04	0.00212
RHPN2	0.486665	1.78E-04	0.002125
BRD2	0.391793	1.78E-04	0.002126
CD300A	0.569982	1.78E-04	0.002126
CCP110	-0.28049	1.82E-04	0.002161
RP11-109C	-0.16358	1.82E-04	0.002161
SHQ1	-0.43113	1.82E-04	0.002162
CCDC142	0.249241	1.82E-04	0.002162
OTUD6B	-0.56037	1.82E-04	0.002166
CTBP1-AS2	0.15513	1.83E-04	0.002169
TNFRSF14	0.441291	1.83E-04	0.00217
EPB42	1.209493	1.83E-04	0.00217
ARL1	0.265116	1.83E-04	0.002171
MAP7D3	-0.36725	1.86E-04	0.002199
NPFFR1	-0.10893	1.86E-04	0.002203
EPB41L3	1.233613	1.88E-04	0.002222
RPL7	-0.0853	1.88E-04	0.002222
DOPEY1	-0.24332	1.88E-04	0.002222
CFLAR	0.376473	1.88E-04	0.002224
LOC10012	-0.14383	1.89E-04	0.002229
KB-431C1.	-0.62002	1.89E-04	0.002232
CAPN3	0.364676	1.91E-04	0.002255
LST1	0.6923	1.92E-04	0.002263
TYK2	0.302016	1.93E-04	0.002268
GOLGA7B	-0.16239	1.93E-04	0.00227
SCAMP2	0.258516	1.93E-04	0.002272
FAM127A	0.40359	1.96E-04	0.002308
RCN3	0.21319	1.97E-04	0.00231
FBXO7	0.476277	1.97E-04	0.00231
INPP4A	0.235524	1.97E-04	0.002317
BRAT1	0.34946	1.98E-04	0.002319
DENND4B	0.364312	1.98E-04	0.002325
ORAI2	0.469039	1.99E-04	0.002334
AP1M1	0.338732	1.99E-04	0.002334
NQO1	0.620841	2.00E-04	0.002339
FLOT1	0.256326	2.00E-04	0.002344
NKTR	-0.62949	2.00E-04	0.002345
DEDD2	0.315031	2.01E-04	0.002351
GNPDA1	0.780887	2.02E-04	0.002355
FUS	0.49201	2.03E-04	0.002366

LY86	1.125061	2.03E-04	0.002366
CIC	0.422739	2.04E-04	0.002382
IWS1	0.313839	2.04E-04	0.002382
RGS14	0.26483	2.04E-04	0.002382
PRORSD1F	-0.67652	2.05E-04	0.002382
DGUOK	0.364485	2.06E-04	0.002394
GPR171	-1.19494	2.06E-04	0.002398
CCDC69	0.556568	2.07E-04	0.00241
TOMM40L	0.372722	2.08E-04	0.002413
NBEAL1	0.195219	2.08E-04	0.00242
RP11-28F1	-0.77557	2.09E-04	0.00242
ZNF345	0.260559	2.09E-04	0.00242
IFNGR1	0.492136	2.09E-04	0.002428
RYK	0.35008	2.10E-04	0.002439
RP3-327A1	0.503514	2.11E-04	0.002442
SELM	0.414996	2.11E-04	0.002448
RAPGEF1	0.203709	2.12E-04	0.002455
C9orf142	0.284494	2.12E-04	0.002456
S100P	1.093638	2.13E-04	0.002463
MSRB1	0.482894	2.14E-04	0.002469
TRAF3	0.277971	2.14E-04	0.002469
IGLL5	1.175761	2.14E-04	0.002471
MPP1	0.403631	2.14E-04	0.002474
FCGBP	0.739802	2.15E-04	0.00248
PRO2964	-0.52919	2.15E-04	0.00248
ZXDB	-0.20297	2.16E-04	0.002492
GNL3	-0.37094	2.18E-04	0.002508
LOC10192	-0.48295	2.19E-04	0.002519
RAD54L2	-0.25805	0.000219	0.002522
WDR11	-0.36861	2.20E-04	0.002525
IDNK	-0.43465	2.20E-04	0.002527
RP11-295C	0.23328	2.21E-04	0.002535
RNF14	0.555684	2.21E-04	0.002537
CENPU	-0.55016	2.21E-04	0.002537
AK130486	-0.08303	2.21E-04	0.002537
CCDC107	0.424127	2.21E-04	0.002537
UTP20	0.421877	2.22E-04	0.002537
WIPF2	0.253293	2.22E-04	0.002542
LOC64598	0.296706	2.22E-04	0.002544
LARP4B	0.271885	2.22E-04	0.002544
ADAM19	0.2323	2.23E-04	0.002544
CRYBB1	0.32582	2.23E-04	0.002547
TLR8	0.614042	2.23E-04	0.002548
HEBP1	0.398915	2.23E-04	0.002548
TDRD7	0.52744	2.24E-04	0.002548
PLXNB2	0.252462	2.24E-04	0.002548
AQP1	0.516149	2.24E-04	0.002553
PRTN3	1.236647	2.25E-04	0.00256
GSE1	0.444779	2.26E-04	0.002575
EDEM2	0.431409	2.29E-04	0.002603
SERPINB1	-0.34817	2.29E-04	0.002606
SEPP1	0.807655	2.30E-04	0.00261
CCDC33	0.067594	2.30E-04	0.00261
TBP	-0.26839	2.30E-04	0.002613
YIPF4	-0.3128	2.31E-04	0.002616
DNAAF2	0.491342	2.31E-04	0.002621
HLA-A	0.236345	2.32E-04	0.002632
ZCCHC24	0.706322	2.32E-04	0.002632

NAA40	0.34635	2.33E-04	0.002635
FKSG49	0.206589	2.34E-04	0.002643
FBXO43	-0.56596	2.35E-04	0.002654
AKR1B1	0.423662	2.36E-04	0.002668
KIAA0020	-0.19009	2.38E-04	0.002686
HOXB7	0.33956	2.38E-04	0.002686
HUWE1	-0.32254	2.38E-04	0.002686
MORC4	0.36625	2.40E-04	0.002703
MGMT	0.367033	2.40E-04	0.002703
ARL6IP4	0.349977	2.40E-04	0.002703
MAP1S	0.486888	2.42E-04	0.00272
LOC10029:	-0.35042	2.42E-04	0.00272
YWHAG	0.181192	2.42E-04	0.00272
FAM63A	-0.28156	2.43E-04	0.002733
PCED1B	0.493791	2.45E-04	0.002749
ARNT	-0.29141	2.46E-04	0.002759
TLCD2	0.153509	2.48E-04	0.002779
RQCD1	0.334232	2.50E-04	0.002805
METTL3	-0.51537	2.52E-04	0.002824
COL24A1	-0.96884	2.52E-04	0.002824
RP11-271C	-0.56932	2.53E-04	0.002832
TRIM56	0.475024	2.53E-04	0.002838
TUBG1	0.529065	2.55E-04	0.002853
PSMD10	0.299403	2.55E-04	0.002854
DAP	0.421734	2.56E-04	0.002859
STK36	0.191035	2.56E-04	0.002859
LYPLA2	0.249943	2.56E-04	0.002859
SAT2	0.381416	2.57E-04	0.002867
TINAGL1	0.099427	2.58E-04	0.002873
PFKFB2	0.135455	2.58E-04	0.002882
CCL18	1.636721	2.62E-04	0.002918
ATP5A1	-0.1118	2.63E-04	0.002925
JAG2	-0.12418	2.63E-04	0.002927
ICAM1	0.754539	2.63E-04	0.002929
GUCA2A	-0.16006	2.63E-04	0.002929
MTIF2	-0.23961	2.65E-04	0.002946
ABCC13	0.280859	2.66E-04	0.002955
USP33	-0.32903	2.67E-04	0.002965
DIP2C	0.395072	2.67E-04	0.002965
TOR2A	0.140761	2.67E-04	0.002965
LOC10050!	0.193439	2.67E-04	0.002965
ALAS2	0.882664	2.68E-04	0.002965
PLEKHA8	-0.1595	2.69E-04	0.002978
GIMAP6	0.813283	2.69E-04	0.002983
CTB-50L17	0.225878	2.70E-04	0.002985
PITPNM3	0.277301	2.70E-04	0.002985
CABP4	-0.29529	2.70E-04	0.002987
ASPH	-0.40587	2.71E-04	0.002989
GORASP2	-0.22667	2.71E-04	0.002992
TOLLIP	0.163394	2.74E-04	0.003022
PCED1A	0.251208	2.74E-04	0.003022
CST7	1.23606	2.74E-04	0.003022
C18orf54	-0.44926	2.74E-04	0.003024
T	-0.14936	2.75E-04	0.003024
CATIP-AS1	-0.27676	2.76E-04	0.003032
YBX1	0.417862	2.76E-04	0.003032
ORC2	-0.26585	2.76E-04	0.003032
ART4	0.212469	2.76E-04	0.003037

RP11-687F	0.13934	0.000277	0.003041
ZBTB26	-0.32335	2.77E-04	0.003043
HSPG2	0.315644	2.77E-04	0.003043
CD40	0.122497	2.77E-04	0.003043
NPLOC4	0.304618	2.78E-04	0.003047
TMPRSS15	-0.07085	2.78E-04	0.003047
ZNF420	-0.37523	2.79E-04	0.003053
ARIH1	0.423189	2.79E-04	0.003053
IBTK	-0.34256	2.79E-04	0.003055
CTSLP8	0.579134	2.80E-04	0.003066
MRPL11	0.388863	2.82E-04	0.003086
ZFAND1	-0.36867	2.83E-04	0.003087
TMEM209	-0.26103	2.83E-04	0.003087
OS9	0.337277	2.83E-04	0.003088
VPS4A	0.230971	2.84E-04	0.003102
ITPRIPL2	0.586465	2.85E-04	0.003102
EXOC8	-0.31397	2.85E-04	0.003102
FXYD5	0.587797	2.85E-04	0.003103
HK2	0.311032	2.85E-04	0.003103
LILRB3	0.31831	2.86E-04	0.003111
PRR11	-0.29835	2.88E-04	0.003129
ZSCAN25	-0.18227	2.89E-04	0.003141
KMT2B	0.264849	2.89E-04	0.003143
SMIM19	0.332524	2.92E-04	0.003167
FMNL2	0.553451	2.92E-04	0.003175
CACNA1I	-0.03485	2.93E-04	0.003182
HAGH	0.558953	2.94E-04	0.00319
TRIM52-AS	0.232064	2.95E-04	0.003193
LAPTM5	0.317374	2.95E-04	0.003193
DDX10	-0.1448	2.95E-04	0.003193
SLC35A1	-0.26595	2.96E-04	0.003199
ARID1A	0.586241	2.96E-04	0.003201
WBP4	0.22986	2.97E-04	0.003208
EIF3E	-0.29519	2.97E-04	0.003214
ME1	1.336887	2.98E-04	0.003214
IL27RA	0.484993	2.98E-04	0.003217
C3	0.452703	2.99E-04	0.003225
SCARNA15	-0.30265	2.99E-04	0.003229
SVIL	0.368054	3.00E-04	0.003239
TMEM119	0.261214	3.03E-04	0.003261
ZNF248	-0.29823	3.03E-04	0.003267
MARCO	0.6888	3.05E-04	0.003279
RAC3	0.207141	3.05E-04	0.003281
SRRT	0.314821	3.06E-04	0.003295
EEF1A1P42	-0.12605	3.07E-04	0.003298
ATP6V1C1	0.408623	3.08E-04	0.003309
9-Sep	0.414022	3.08E-04	0.003309
MTRR	-0.23503	3.10E-04	0.003323
RRAS	0.460527	3.10E-04	0.003327
CHRAC1	-0.31589	3.10E-04	0.003329
DOCK4	0.503319	3.12E-04	0.003347
COX7A2L	-0.21857	3.13E-04	0.003348
AFAP1L1	0.354449	3.14E-04	0.003357
MRPL33	0.369266	3.14E-04	0.003359
NDST2	0.215376	3.17E-04	0.003393
RP11-309C	0.331872	3.18E-04	0.003403
MAPKAPK2	0.448248	3.19E-04	0.003404
MAF	0.342275	3.20E-04	0.003412

USPL1	-0.28995	3.20E-04	0.003412
GPR1	-0.08558	3.21E-04	0.00342
PLCL1	0.310508	3.21E-04	0.003422
TLE3	0.265137	3.21E-04	0.003422
ACP5	0.751112	3.21E-04	0.003422
DKFZP586I	-0.36054	3.23E-04	0.003437
PDIA4	0.415143	3.23E-04	0.003439
VTI1B	0.317317	3.24E-04	0.003448
RBCK1	0.239094	3.24E-04	0.003449
SENP2	0.323086	3.29E-04	0.003492
TNS3	0.234397	3.31E-04	0.003512
ZNF532	0.730576	3.31E-04	0.003512
ZFYVE21	0.438407	3.31E-04	0.003512
TFPT	0.41	3.33E-04	0.003528
HK3	0.782282	3.35E-04	0.003549
CASZ1	0.212399	3.36E-04	0.003556
MOCS3	0.285805	3.37E-04	0.003572
HOMER3	0.47216	3.38E-04	0.003575
DTNB	0.064179	3.39E-04	0.003581
PGPEP1	0.255666	3.40E-04	0.003594
DUSP5	0.682687	3.40E-04	0.003598
TPP1	0.336311	3.41E-04	0.003606
PLEKHA2	-0.79268	3.42E-04	0.003609
WAS	0.147033	3.42E-04	0.003609
SRSF6	0.490797	3.44E-04	0.003625
ATP5G3	0.402552	3.44E-04	0.003625
BC069739	0.091526	3.44E-04	0.003625
EVL	0.346285	3.44E-04	0.003628
RTN4R	0.337686	3.45E-04	0.003637
DENND1B	-0.4818	3.47E-04	0.00365
ANXA1	0.823749	3.48E-04	0.00366
NAB1	0.419365	3.49E-04	0.003671
SLC22A3	-0.19867	3.51E-04	0.003687
ANXA6	0.244362	3.53E-04	0.003702
U2AF2	0.179084	3.53E-04	0.003703
HRH1	0.369117	3.54E-04	0.00371
SLC31A2	0.623098	3.54E-04	0.003712
COMT	0.275252	3.55E-04	0.003717
PLEK	0.375473	3.58E-04	0.003755
CCDC22	0.319325	3.61E-04	0.003777
TNFAIP1	0.2288	3.61E-04	0.003777
CKAP4	0.703564	3.62E-04	0.003792
KIF26A	0.338771	3.63E-04	0.003794
PRRC2B	0.376458	3.63E-04	0.003794
FAM122B	-0.44037	3.66E-04	0.003826
GPR85	0.184387	3.67E-04	0.003833
G0S2	1.325765	3.67E-04	0.003833
NATD1	0.411675	3.69E-04	0.003851
ZNF138	-0.24496	3.69E-04	0.003852
C14orf142	0.501868	3.70E-04	0.003856
DCAF12	0.384995	3.71E-04	0.00386
TRAM2-AS	0.425524	3.71E-04	0.003861
ATPIF1	-0.34969	3.71E-04	0.003861
TGFBR1	-0.71657	3.73E-04	0.00388
TBL1X	0.156479	3.73E-04	0.003881
FAM161B	-0.19439	3.74E-04	0.003881
EMD	0.344974	3.74E-04	0.003881
CD58	0.390266	3.74E-04	0.003883

CHAF1A	0.539334	3.74E-04	0.003886
ZFP41	-0.28553	3.76E-04	0.003902
MRPS34	0.483065	3.76E-04	0.003902
ZNF548	-0.42405	3.77E-04	0.003911
SAMD9L	0.549127	3.78E-04	0.003912
HCST	0.543261	3.80E-04	0.00393
RP3-522P1	0.175699	3.81E-04	0.003946
SQRDL	0.429945	3.83E-04	0.003959
CD81	0.440711	3.84E-04	0.003973
TUBB	0.429953	3.85E-04	0.003973
PNO1	0.32913	3.85E-04	0.003978
ZNF587	-0.23347	3.89E-04	0.004016
DCTN5	0.426997	3.90E-04	0.004021
PPCS	0.277583	3.91E-04	0.004034
PPAP2B	0.935692	3.92E-04	0.004038
UTP3	0.258967	3.92E-04	0.004038
ACYP1	-0.34121	3.93E-04	0.004049
GRAMD3	0.333454	3.96E-04	0.004079
EXOSC3	0.228549	3.98E-04	0.00409
KCNMA1	0.267045	4.00E-04	0.00411
ATP2C1	-0.2566	4.00E-04	0.00411
THAP8	0.227263	4.01E-04	0.004122
NYNRIN	0.56283	4.02E-04	0.004126
AOAH	0.654688	4.03E-04	0.004132
CCNL2	0.36788	4.03E-04	0.004132
BLMH	-0.48185	4.03E-04	0.004132
RP4-710M	-0.30505	4.04E-04	0.004142
ABHD13	-0.29129	4.04E-04	0.004142
NCOA3	0.451578	4.06E-04	0.004162
SNAPC4	0.280914	4.07E-04	0.004166
PRPF4	0.335096	4.09E-04	0.004181
LENG9	0.12576	4.09E-04	0.004187
ANKFY1	0.110207	4.12E-04	0.004212
TBCB	0.302566	4.12E-04	0.004212
HAVCR2	0.22437	4.13E-04	0.004216
HBQ1	0.76521	4.13E-04	0.004218
PCYT1A	0.321189	4.14E-04	0.004218
PIGG	-0.10545	4.14E-04	0.004218
UCK1	0.165943	4.16E-04	0.004241
PXMP4	0.329347	4.17E-04	0.004241
R3HCC1	0.333768	4.17E-04	0.004241
ARHGEF9	0.31272	4.18E-04	0.004254
MOSPD1	0.381974	4.19E-04	0.004258
MALSU1	0.43423	4.19E-04	0.004263
OTUD4	-0.24111	4.23E-04	0.004299
MED12	0.476725	4.23E-04	0.004299
SF3A3	0.2949	4.24E-04	0.004305
HDGF	0.290914	4.26E-04	0.004323
RAB31	1.013466	4.26E-04	0.004323
SPRED3	-0.21846	4.27E-04	0.004331
ZC3H3	0.166815	4.27E-04	0.004331
MBD6	0.581878	4.28E-04	0.004333
PM20D2	-0.62984	4.29E-04	0.004342
ESYT2	-0.38468	4.30E-04	0.004349
UCN3	-0.16027	4.31E-04	0.004359
MIR17HG	0.124196	4.31E-04	0.004359
SQSTM1	0.464271	4.33E-04	0.004371
PRRX2	-0.11703	4.33E-04	0.004371

WHAMMP	-0.55036	4.33E-04	0.004371
PPP1CA	0.356735	4.34E-04	0.004382
THAP7	0.278072	4.34E-04	0.004382
DEF8	0.332221	4.35E-04	0.004388
CREB3	0.425463	4.35E-04	0.004388
LEPREL1	0.38729	4.37E-04	0.004397
NDFIP2	0.483668	4.37E-04	0.004397
HLA-E	0.220565	4.37E-04	0.004397
SSH3	0.242316	4.39E-04	0.004411
LENG1	0.309249	4.39E-04	0.004417
RP11-182L	-0.50099	4.41E-04	0.004433
MTIF3	0.217698	4.44E-04	0.004459
ZNF283	-0.34909	4.45E-04	0.004471
FCHSD1	0.123909	4.47E-04	0.004489
ARRDC1	0.238306	4.48E-04	0.004492
OSBPL9	-0.1992	4.51E-04	0.004516
RBM7	-0.23071	4.51E-04	0.004516
ACP6	-0.4223	4.51E-04	0.004518
NEK5	-0.36056	4.53E-04	0.004537
TMTC4	0.401956	4.56E-04	0.004565
PLXNA1	0.482626	4.60E-04	0.004599
POLR2B	-0.20439	4.60E-04	0.004601
RP11-38P2	-0.47139	4.62E-04	0.004618
SLITRK2	-0.07237	4.65E-04	0.004643
HSP90B1	0.420639	4.67E-04	0.004664
UBA6	0.578438	4.67E-04	0.004665
ZNF670	-0.48375	4.68E-04	0.004667
SP110	0.505705	4.68E-04	0.004671
PIK3R3	0.424441	4.69E-04	0.004674
URB2	-0.39588	4.70E-04	0.004677
EGR2	0.776321	4.71E-04	0.004692
UQCRQ	0.382262	4.72E-04	0.004692
GMIP	0.177004	4.73E-04	0.004707
PCSK2	0.435695	4.75E-04	0.004721
IL1R1	0.472663	4.80E-04	0.004768
ARL11	0.332553	4.82E-04	0.004785
BC044596	-0.75086	4.84E-04	0.004804
SPICE1	-0.16656	4.85E-04	0.00481
PPIF	0.537275	4.85E-04	0.004811
S1PR2	-0.12374	4.87E-04	0.004825
CBX3	-0.29364	4.89E-04	0.004843
CDC26	0.256194	4.89E-04	0.004843
SMG5	0.233392	4.90E-04	0.004847
ANKLE1	0.690017	4.90E-04	0.004848
CCDC138	-0.28184	4.90E-04	0.004848
POC5	-0.35931	4.90E-04	0.004848
LRRCC1	-0.34213	4.91E-04	0.004848
ERAP2	0.977439	4.91E-04	0.004849
RPS3	-0.09696	4.92E-04	0.004858
RPGRIP1L	-0.19629	4.92E-04	0.004858
EMC4	0.250218	4.93E-04	0.004861
POLR1A	0.301121	4.97E-04	0.004901
ALDOA	0.252123	4.98E-04	0.00491
EPC1	-0.29093	4.98E-04	0.00491
FCGR2C	0.553747	4.99E-04	0.00491
ZNF615	-0.31033	4.99E-04	0.004914
RPS28	-0.34191	5.00E-04	0.004914
KBTBD6	0.464445	5.00E-04	0.004914

CLTC-IT1	-0.40255	5.00E-04	0.004917
TMEM64	-0.44856	5.00E-04	0.004917
DCLRE1C	-0.34067	5.01E-04	0.004919
INO80B	0.203273	5.01E-04	0.00492
ARID3A	0.231239	5.01E-04	0.004921
COQ10B	0.203098	5.04E-04	0.004946
HYAL2	0.414993	5.06E-04	0.004964
KRBA2	-0.65824	5.07E-04	0.004969
TMEM126I	0.150935	5.09E-04	0.004984
METTL25	-0.23703	5.09E-04	0.004986
EPB41	0.264894	5.10E-04	0.004989
MRPL52	0.240954	5.12E-04	0.00501
BICD1	-0.31742	5.13E-04	0.005015
PEX11A	-0.2084	5.15E-04	0.005034
DHRS3	0.774458	5.15E-04	0.005034
ZNF687	0.119587	5.17E-04	0.005046
TLR5	0.502518	5.18E-04	0.005053
ZNF629	-0.38324	5.18E-04	0.005053
ZNF100	-0.29826	5.18E-04	0.005053
SLC31A1	0.326578	5.19E-04	0.005061
ITLN1	0.314285	5.23E-04	0.00509
EXOC6	0.425228	5.24E-04	0.005099
CCDC9	-0.18026	5.24E-04	0.005103
MRPS26	0.215059	5.27E-04	0.005122
PARVB	0.483844	5.27E-04	0.005122
CWC22	-0.38085	5.27E-04	0.005126
TRIQK	-0.38611	5.28E-04	0.00513
PEX19	0.216499	5.31E-04	0.005153
LRRC4	0.149139	5.31E-04	0.005153
RRAS2	0.505388	5.31E-04	0.005155
PELP1	0.300647	5.34E-04	0.005177
CIB1	0.308169	5.35E-04	0.005187
CAB39	0.262975	5.37E-04	0.005199
CBFA2T3	-0.43957	5.38E-04	0.005206
CSF2RA	0.285498	5.38E-04	0.005206
PI4KB	0.518102	5.38E-04	0.005206
MMP19	0.311336	5.41E-04	0.005234
CTPS2	-0.21103	5.42E-04	0.005236
NIFK	-0.23547	5.43E-04	0.005243
CPTP	0.228953	5.45E-04	0.005264
CD163L1	0.384824	5.46E-04	0.005269
PJA1	-0.32602	5.46E-04	0.005274
SMS	0.419986	5.47E-04	0.005277
SPSB4	0.245179	5.52E-04	0.005318
VMO1	0.284206	5.53E-04	0.005329
SMIM8	-0.18483	5.54E-04	0.005338
CCDC106	0.156832	5.55E-04	0.005346
TBC1D10A	0.141129	5.57E-04	0.005362
GK	0.243101	5.60E-04	0.005388
PIR	0.802925	5.61E-04	0.005397
KIF6	0.018757	5.72E-04	0.005498
HIATL2	0.346129	5.75E-04	0.005521
BCKDK	0.365421	5.76E-04	0.005528
FERMT2	0.228416	5.76E-04	0.005533
LRRK2	0.527465	5.78E-04	0.005541
RPAP2	-0.26971	5.79E-04	0.005558
XPC	0.256823	5.81E-04	0.005567
WNT10B	-0.19507	5.85E-04	0.005603

TM6SF1	0.761033	5.85E-04	0.005604
TOX2	0.229665	5.87E-04	0.005614
SPATA5L1	0.264995	5.87E-04	0.005614
U2AF1L4	0.461649	5.87E-04	0.005614
GPD2	0.361062	5.87E-04	0.005614
MOAP1	-0.25737	5.89E-04	0.005623
SCO2	0.385998	5.89E-04	0.005623
UBXN2A	-0.20302	5.90E-04	0.005632
EP300	0.385072	5.90E-04	0.005635
NFKBIB	0.203561	5.92E-04	0.005643
WBP2	0.503871	5.94E-04	0.005665
HELLS	-0.46696	5.95E-04	0.005667
TAF1D	-0.45358	5.98E-04	0.005698
SATB1	-0.53543	5.99E-04	0.005708
TP53I13	0.36136	6.01E-04	0.005717
POLR1E	-0.53181	6.01E-04	0.00572
RP4-581F1	0.266439	6.02E-04	0.005727
C16orf46	-0.14744	6.02E-04	0.005727
LIPA	0.55064	0.000603	0.005733
MYOM1	-0.3426	6.04E-04	0.005733
CNOT6L	0.2224	6.04E-04	0.005738
C6orf1	0.206024	6.07E-04	0.005756
ARHGEF6	-0.23214	6.10E-04	0.005786
NUCB1	0.374075	6.10E-04	0.005787
CD74	0.427649	6.13E-04	0.005812
MMP9	0.94657	6.14E-04	0.005816
PPP6R2	0.212093	6.15E-04	0.005821
MIF	0.546977	6.15E-04	0.005821
FLT3	-0.84415	6.15E-04	0.005821
IFT20	0.208933	6.17E-04	0.005831
CLN5	0.344467	6.17E-04	0.005835
SAR1A	0.527372	6.18E-04	0.005835
ANGPT1	-0.7051	6.18E-04	0.005839
DOK3	0.245323	6.20E-04	0.005854
INPP1	-0.42569	6.21E-04	0.005858
SLC39A10	-0.30931	6.25E-04	0.005895
SCD	0.218316	6.27E-04	0.00591
FAM35A	-0.21829	6.28E-04	0.005914
SLC35G2	-0.42502	6.28E-04	0.005914
EIF2B4	-0.29656	6.28E-04	0.005915
TRAPPC1	0.519661	6.34E-04	0.005964
COIL	0.232736	6.35E-04	0.005978
GAB3	-0.45677	6.36E-04	0.005978
UBE4A	-0.1834	6.36E-04	0.005978
TMEM123	-0.45427	6.38E-04	0.005998
BC032415	0.183979	6.39E-04	0.006001
RPL10A	-0.11802	6.41E-04	0.006014
GAS6	0.304263	6.41E-04	0.006014
DCAF10	0.269049	6.41E-04	0.006014
DYRK4	0.375492	6.42E-04	0.006016
LUC7L3	-0.31111	6.45E-04	0.006047
RREB1	-0.27826	6.48E-04	0.00607
RBM41	0.462261	6.49E-04	0.006078
EFCAB14	0.252064	6.50E-04	0.006084
STOX2	0.523281	6.54E-04	0.006114
SRPRB	0.373904	6.55E-04	0.00612
S100A12	1.455778	6.55E-04	0.00612
FOXN2	-0.1523	6.55E-04	0.00612

GP2	-0.04753	6.55E-04	0.00612
ALDH3B1	0.488555	6.56E-04	0.006126
FMNL3	0.125554	6.59E-04	0.006146
AUP1	0.371535	6.60E-04	0.006152
C20orf196	0.293065	6.63E-04	0.006182
TVP23B	-0.48881	6.66E-04	0.006206
USP11	0.360235	6.71E-04	0.006248
NAP1L1	-0.33155	6.71E-04	0.006248
CITED2	0.669028	6.73E-04	0.006263
AC004692.	-0.31587	6.76E-04	0.006288
ADSL	-0.25689	6.77E-04	0.006291
NOC4L	0.420975	6.80E-04	0.006324
AC025442.	-0.07228	6.84E-04	0.006352
ZNF611	-0.16297	6.84E-04	0.006354
XRCC5	-0.23843	6.86E-04	0.006364
PLEKHM2	0.277804	6.90E-04	0.006398
CTA-390C:	-0.42207	6.92E-04	0.006416
PIK3AP1	0.374204	6.92E-04	0.006416
RBM38	0.487712	6.93E-04	0.00642
ELK3	-0.28216	6.94E-04	0.006426
MTMR2	-0.26652	6.95E-04	0.006432
SAFB	0.459744	6.96E-04	0.006441
SMIM1	0.578654	6.97E-04	0.006447
ZNF785	0.622649	6.98E-04	0.006452
HBM	1.437004	6.99E-04	0.006464
USP20	0.402772	7.01E-04	0.006475
CHFR	0.223352	7.03E-04	0.006494
XXyac-YX1	-0.29679	7.10E-04	0.006558
RNF182	0.905994	7.12E-04	0.006567
TMEM212	-0.13441	7.14E-04	0.006579
SAC3D1	0.44654	7.14E-04	0.006579
UBE2R2	0.202871	7.15E-04	0.006592
NLRC3	-0.44595	7.17E-04	0.0066
SOX6	0.241216	7.18E-04	0.006612
RGL1	1.344348	7.25E-04	0.006673
TYMS	0.634123	7.27E-04	0.006689
LRRC8D	-0.24672	7.29E-04	0.006704
ZNF652	-0.49382	7.31E-04	0.006718
MYOD1	-0.08641	7.34E-04	0.00674
BMP7	-0.06636	7.34E-04	0.006744
CKAP2	-0.47525	7.37E-04	0.006762
MYO10	0.275225	7.37E-04	0.006765
HIST1H1B	-0.72856	7.39E-04	0.006776
NARS2	-0.25977	7.39E-04	0.006776
C4BPA	0.28773	7.42E-04	0.006802
AGL	-0.38838	7.48E-04	0.006848
LPAR1	0.566002	7.48E-04	0.006848
KLF6	0.634534	7.48E-04	0.00685
MAP4K2	0.488209	7.53E-04	0.006888
LILRA5	0.233781	7.55E-04	0.006901
HAUS3	-0.28488	7.57E-04	0.006917
FAM102B	0.294926	7.59E-04	0.006935
KMT2A	0.481344	7.64E-04	0.006981
RHD	0.822978	7.66E-04	0.006995
UNC13A	-0.05408	7.69E-04	0.007017
KPNA1	0.299963	7.69E-04	0.007018
RP3-337H4	-0.1071	7.71E-04	0.007032
CCBL2	-0.20823	7.74E-04	0.007051

C10orf11	0.437565	7.77E-04	0.007082
TMEM184E	0.289058	7.87E-04	0.007163
TWISTNB	0.269801	7.87E-04	0.007168
TP53	0.331007	7.88E-04	0.007168
KDM7A	0.549238	7.88E-04	0.007168
LINC01341	-0.45603	7.89E-04	0.007175
SENP8	0.274961	7.90E-04	0.007183
PEX16	0.379052	7.94E-04	0.007209
FGD6	0.125812	7.94E-04	0.007209
BCAP29	0.289993	7.96E-04	0.007227
RP11-111M	-0.55713	7.97E-04	0.007227
MORC3	-0.30718	8.04E-04	0.007287
SPIN4	-0.62129	8.04E-04	0.00729
SNX10	0.528549	8.05E-04	0.007295
EHMT1	-0.50353	8.08E-04	0.007322
PLIN3	0.265547	8.09E-04	0.007322
CHAC1	0.170808	8.10E-04	0.007327
CCDC91	-0.3983	8.12E-04	0.007341
WLS	0.601622	8.13E-04	0.007348
THAP9-AS	-0.29081	8.14E-04	0.00736
TADA3	0.227507	8.16E-04	0.007369
DTNBP1	0.352049	8.16E-04	0.00737
MCU	0.296541	8.23E-04	0.007431
GTPBP8	-0.33797	8.25E-04	0.007444
GBE1	-0.21534	8.26E-04	0.007454
IGLL3P	0.701391	8.27E-04	0.00746
CREM	0.480018	8.33E-04	0.007505
TRMT10A	-0.43533	8.35E-04	0.007522
PRO0471	0.232344	8.36E-04	0.007529
COX5B	0.304821	8.37E-04	0.007531
LRP1	0.199736	8.38E-04	0.007537
SMARCA4	0.425918	8.38E-04	0.007537
DIS3L	0.323699	8.39E-04	0.007537
PNPLA3	0.250291	8.39E-04	0.007537
RBM25	0.270043	8.40E-04	0.007541
SRSF5	-0.29118	8.42E-04	0.007557
PRKAA1	-0.4412	8.44E-04	0.007578
RIPK3	0.291582	8.46E-04	0.007587
ITPRIP	0.24593	8.46E-04	0.007589
NHS	0.075699	8.47E-04	0.007589
TTLL3	-0.13975	8.47E-04	0.007591
FAM208A	-0.21616	8.50E-04	0.007609
ORAOV1	-0.18751	8.53E-04	0.007636
ZNF143	-0.21916	8.54E-04	0.007639
ZNF749	-0.15436	8.55E-04	0.007649
KIF2A	-0.29846	8.57E-04	0.007664
PLEKHA8P.	-0.38879	8.58E-04	0.007672
TICAM1	0.308942	8.65E-04	0.007731
RP5-1136(	-0.43842	8.69E-04	0.007757
HS3ST2	1.211075	8.69E-04	0.007761
DOCK5	-0.38191	8.70E-04	0.007762
C12orf45	0.419466	8.73E-04	0.007787
ITPR2	0.361773	8.75E-04	0.007803
IFT22	-0.21942	8.76E-04	0.007805
PDXK	0.309115	8.77E-04	0.007816
SNAP23	0.401237	8.78E-04	0.007817
NPEPPS	0.2841	8.79E-04	0.007821
KCMF1	0.252455	8.79E-04	0.007821

ORMDL2	0.41296	8.80E-04	0.007825
FAM20A	0.290686	8.81E-04	0.007828
PES1	0.171377	8.87E-04	0.007881
DOHH	-0.28336	8.88E-04	0.007886
PIGL	-0.21451	8.96E-04	0.007952
RHOT2	0.267308	8.97E-04	0.007957
MTG2	0.227359	8.97E-04	0.007957
CCDC62	-0.28935	8.97E-04	0.007958
EXOSC6	0.283908	9.00E-04	0.007977
SPATA2L	0.375985	9.00E-04	0.007977
FKBP7	-0.27883	9.02E-04	0.007986
SYMPK	0.164592	9.03E-04	0.00799
C5	-0.48539	9.03E-04	0.00799
SUN2	-0.40119	9.03E-04	0.00799
LOC10192	0.023962	9.04E-04	0.007994
TPMT	0.270838	9.05E-04	0.007998
CEBPG	0.265659	9.05E-04	0.007998
5-Mar	-0.19796	9.07E-04	0.008008
TMEM9B	0.299382	9.08E-04	0.008012
CSNK1G1	0.223762	9.08E-04	0.008012
UBE2Q1	0.337579	9.11E-04	0.008038
PAQR3	-0.20839	9.13E-04	0.00805
XPR1	-0.2901	9.15E-04	0.008062
ODF3	-0.12553	9.16E-04	0.008074
VPRBP	0.292335	9.19E-04	0.008093
TMEM129	0.278472	9.19E-04	0.008093
ZNF606	-0.37821	9.22E-04	0.008116
PRRC2C	0.419487	9.23E-04	0.008119
OSBPL6	0.60997	9.24E-04	0.00812
POLG2	-0.30354	9.24E-04	0.008124
AQP9	0.863498	9.26E-04	0.008132
KLF4	0.663188	9.26E-04	0.008135
FIP1L1	0.326913	9.29E-04	0.008154
NUS1P3	0.212074	9.30E-04	0.008159
APRT	0.316011	9.33E-04	0.008179
GPER1	0.093976	9.34E-04	0.008187
S100A4	0.663744	9.38E-04	0.008222
TNS4	-0.07969	9.41E-04	0.00824
BRF2	0.214545	9.44E-04	0.008263
MAFG	0.363654	9.45E-04	0.008271
PRKD3	-0.39677	9.48E-04	0.008297
TASP1	-0.21536	9.52E-04	0.008324
CCDC58	0.610997	9.54E-04	0.008337
SESN3	0.336177	9.55E-04	0.008339
SRP68	-0.27339	9.55E-04	0.008339
LOC72802	-0.28696	9.55E-04	0.008339
REST	-0.21968	9.56E-04	0.008342
WDR82	-0.17596	9.56E-04	0.008342
EXOSC9	0.247017	9.57E-04	0.008344
PLCL2	0.549499	9.57E-04	0.008344
RNF169	-0.19466	9.60E-04	0.008368
SYK	-0.35455	9.62E-04	0.008383
MTMR14	0.304525	9.64E-04	0.008391
TIMM8B	0.306449	9.64E-04	0.008391
SYNDIG1	0.397202	9.65E-04	0.008399
ZNF354A	0.325621	9.69E-04	0.008425
RP3-334F4	-0.22413	9.72E-04	0.008446
ARL4C	0.823246	9.77E-04	0.008494

CUL3	-0.25576	9.81E-04	0.008519
PPP1R11	0.23485	9.83E-04	0.008532
CYB561D1	0.167093	9.83E-04	0.008532
LOC10050(	-0.17454	9.85E-04	0.008542
SAP18	-0.38009	9.88E-04	0.008566
NDUFA1	0.229566	9.92E-04	0.008599
MLST8	0.394142	9.93E-04	0.008606
ZNF746	0.219762	9.96E-04	0.008624
GMPR	0.77438	0.001	0.00866
SOCS2	0.946356	0.001001	0.00866
P4HA1	0.16179	0.001001	0.008663
DNAJC24	-0.16883	0.001002	0.008666
ERCC5	-0.20568	0.001004	0.008675
C11orf96	0.829421	0.001004	0.008675
COPG2IT1	-0.58471	0.001007	0.0087
SAMD12	-0.30668	0.001011	0.008728
STAT6	0.196284	0.001011	0.008728
ZSCAN30	-0.18338	0.001018	0.008786
SERTAD1	0.524766	0.00102	0.008799
CAMK1	0.247317	0.001022	0.008807
NELFCD	0.364432	0.001023	0.008814
DNTTIP1	0.281437	0.001024	0.008825
APLP2	0.46151	0.001027	0.008839
AKAP7	-0.17556	0.00103	0.008864
ZBTB40	0.386956	0.001032	0.008882
IMPDH2	-0.28508	0.001034	0.008891
MYO7A	0.33771	0.001036	0.008903
ZC3HAV1L	-0.25325	0.001041	0.008943
ASF1B	0.57763	0.001042	0.00895
LYRM2	0.301518	0.001045	0.00897
CREG1	0.312523	0.001045	0.00897
LILRA2	0.620522	0.001045	0.00897
LOC10193(	-0.14114	0.001046	0.008975
CDK1	0.674588	0.001048	0.008981
LOC10028!	-0.32644	0.001048	0.008981
TMEM140	0.739633	0.001053	0.009024
CASC5	0.39696	0.001065	0.009121
C1orf122	0.261447	0.001066	0.009121
VWA9	-0.1748	0.001071	0.009165
TMEM117	0.282347	0.001072	0.00917
ACO1	0.177301	0.001077	0.009203
ATF6	0.177324	0.001081	0.009236
LRRK1	0.270009	0.001082	0.009244
G6PD	0.484693	0.001087	0.00928
CABIN1	0.348893	0.00109	0.009307
ABHD11	0.08896	0.001092	0.009317
RILPL1	0.488336	0.001094	0.009333
FAM127B	0.223664	0.001103	0.009401
STXBP2	0.193991	0.001103	0.009401
RTN3	0.234547	0.001104	0.009401
RP11-348N	0.1226	0.001106	0.009415
PLEKHM1	0.154583	0.001106	0.009415
CIPC	0.43794	0.001107	0.009415
BRCC3	0.3072	0.001107	0.009416
C2orf81	-0.18023	0.001108	0.009417
SUPT6H	0.310822	0.001111	0.009439
CCDC174	0.348563	0.001115	0.009473
SHC2	0.13992	0.001119	0.009499

CRIPT	-0.21122	0.001129	0.00958
TMUB2	0.231228	0.001129	0.009582
CMA1	0.006628	0.001132	0.009599
RRP15	-0.28115	0.001133	0.009604
ZNF526	0.289996	0.001133	0.009606
RAP2C-AS	0.122193	0.001135	0.009617
EWSR1	0.288268	0.001138	0.009635
FOXJ3	-0.1241	0.001141	0.009663
BCL2A1	0.822004	0.001143	0.009671
MAZ	0.223259	0.001146	0.00969
AEBP2	-0.19179	0.001156	0.009776
AFMID	0.28672	0.001157	0.009776
RP11-196(	-0.51318	0.001165	0.009838
NUS1	0.252272	0.001165	0.009838
TRAFD1	0.197937	0.001169	0.00987
LCLAT1	-0.26093	0.00117	0.009875
PPIL6	-0.09094	0.001173	0.009897
GOLGA1	0.360734	0.001174	0.009897
DBI	0.24212	0.001175	0.009908
LSM8	-0.29595	0.001176	0.009912
RSF1	-0.19964	0.001177	0.009912
LBH	0.512316	0.001177	0.009912
SLC25A17	-0.26787	0.001178	0.009912
NSMAF	-0.46372	0.001183	0.009951
PSMC3	0.358034	0.001184	0.009959
UFSP2	0.307641	0.001189	0.009996
SPATC1L	0.555721	0.00119	0.010003
ODF2	0.296773	0.001194	0.010033
LOC10013(	-0.3307	0.001201	0.010087
WEE1	0.476234	0.001209	0.01015
GPD1L	0.415183	0.001212	0.010165
APOC1	0.840493	0.001216	0.010193
MRPL13	0.388284	0.001216	0.010193
CAST	0.489578	0.001217	0.010198
INO80E	0.367939	0.001222	0.010236
ABI3	0.416829	0.001224	0.010252
NOTCH2	0.209837	0.001225	0.010252
CDO1	0.358848	0.001226	0.010261
PIGX	0.354793	0.001228	0.010275
PIGQ	0.10927	0.001231	0.010289
CIRBP-AS1	0.068575	0.001232	0.010298
LIAS	0.535124	0.001233	0.010298
NUDT14	0.203066	0.001234	0.010305
DNA2	-0.40287	0.00124	0.010348
RDH10	0.176707	0.00124	0.010348
CDK9	0.365745	0.001251	0.010432
TMEM71	-0.75252	0.001251	0.010435
NACC1	0.126682	0.001254	0.010451
ADPRHL2	-0.39113	0.001255	0.010453
DCHS1	0.35138	0.001255	0.010455
PDS5B	-0.21331	0.001258	0.010473
SULT1C2	0.333509	0.001258	0.010473
LOC10050(	-0.21785	0.00126	0.01048
PPP2R5B	0.145708	0.001262	0.010491
MIIP	0.263243	0.001262	0.010493
ZBTB7A	0.090934	0.001263	0.010497
POLR3K	0.294462	0.001265	0.010504
SLC22A5	0.200966	0.001266	0.01051

MYOM2	-0.47879	0.001268	0.010523
SART3	-0.2523	0.001269	0.010531
FAM96B	0.240642	0.001276	0.010586
UCKL1	0.249243	0.001277	0.010588
ANKRD44	-0.34254	0.001278	0.010588
LTB4R	0.457455	0.001284	0.010638
C17orf70	-0.11567	0.001285	0.010638
TMEM69	-0.35749	0.001288	0.010662
RHOG	0.380072	0.001291	0.010681
C19orf68	-0.29111	0.001291	0.010681
C8orf33	0.237532	0.001294	0.0107
SCFD1	0.344894	0.001295	0.010707
RPP30	-0.28002	0.001298	0.010725
DNAJB11	0.339523	0.001302	0.010748
DYNLL1	0.342366	0.001302	0.010748
USP44	0.239529	0.001303	0.010752
RNF157	0.076156	0.001303	0.010752
SP100	0.324717	0.001306	0.010764
LRP1B	0.134994	0.001306	0.010764
MRPL27	0.381174	0.001306	0.010764
ALOX5AP	0.731001	0.001307	0.010766
PELI2	-0.19023	0.001309	0.010781
POLDIP3	0.138787	0.001313	0.010809
AHCTF1	-0.30338	0.001319	0.01085
MOB4	-0.2219	0.001319	0.010852
CTSA	0.54836	0.001321	0.010858
AC068831.	-0.09509	0.001332	0.010941
GPT2	-0.4406	0.001332	0.010941
PSMD8	0.347712	0.001332	0.010942
JARID2	-0.28734	0.001333	0.010946
MIB2	0.140729	0.001334	0.010948
ZNF17	-0.30193	0.001336	0.010959
MDM1	-0.31083	0.001339	0.010975
PIGB	-0.25657	0.001339	0.010975
PIP4K2A	-0.5517	0.001341	0.010986
DAAM1	-0.53219	0.001343	0.011001
UTP6	-0.11946	0.001348	0.011036
TIMM17B	0.170062	0.001351	0.011056
CSF1R	0.809626	0.001354	0.01108
HERPUD2	0.469022	0.001355	0.011084
NR1D2	-0.41192	0.001363	0.011143
WDR27	-0.23187	0.001364	0.011144
WDTC1	0.23175	0.001367	0.011162
ZFYVE19	0.191137	0.001367	0.011162
QDPR	0.159804	0.001368	0.011164
NUAK1	0.486428	0.001371	0.011178
TKT	0.458404	0.001371	0.011178
TRMT10C	-0.2126	0.001371	0.011178
PDGFC	0.445754	0.001372	0.011182
AMZ2P1	0.316628	0.001375	0.011205
COPS4	-0.09129	0.001377	0.011212
HSF1	0.106544	0.001379	0.011227
SPIRE1	0.473242	0.00138	0.01123
LTF	1.393423	0.001381	0.011231
VPS8	-0.48655	0.001383	0.011244
PRELID1	0.374667	0.001383	0.011244
CC2D1B	0.117384	0.001387	0.011269
MREG	-0.41767	0.00139	0.011294

HMGN2	0.159772	0.001392	0.011305
CACYBP	0.273641	0.001398	0.011344
ZNF296	0.226032	0.001405	0.011401
SMAD5	0.493749	0.001414	0.011463
RPRD2	0.304031	0.001414	0.011463
ITPK1-AS1	-0.22746	0.001416	0.011473
RING1	0.226066	0.001428	0.011572
DDX23	-0.29905	0.001429	0.011573
ZNF418	-0.42658	0.00143	0.011573
LINC00526	-0.40542	0.001431	0.011578
MDGA1	-0.11313	0.001434	0.011603
RAD18	-0.35441	0.001436	0.011613
BTK	-0.32105	0.001437	0.011613
INO80D	-0.22848	0.001438	0.011616
MBNL2	0.21999	0.001444	0.011666
ANKRD22	0.307719	0.001447	0.011679
SLC2A1	0.397173	0.001448	0.011686
TIMP1	0.596718	0.001453	0.011726
CR1	0.503068	0.001454	0.011729
HNRNPR	-0.36822	0.001456	0.011742
ATP6V1E1	0.223117	0.00146	0.011762
ZNF384	-0.12195	0.00146	0.011762
APEX1	-0.22476	0.001461	0.011762
ELL3	-0.3082	0.001467	0.011806
LARP1	0.592311	0.001469	0.011819
ASUN	-0.22038	0.001474	0.01186
SETD2	0.297758	0.001475	0.01186
PPRC1	0.398284	0.001475	0.01186
RTP4	0.546476	0.001478	0.011877
RAB43	0.133536	0.00148	0.01189
IGF1R	0.378411	0.001486	0.011934
ZNF574	0.292731	0.001487	0.011934
MGLL	0.473172	0.001492	0.011967
SYT11	-0.37009	0.001492	0.011967
SLA	0.415735	0.001495	0.01199
RANBP10	0.201294	0.001497	0.011998
UBAP2L	0.267935	0.001506	0.012066
CISD1	0.389811	0.001509	0.012081
RPL6	-0.07563	0.001509	0.012082
RPL15	-0.2251	0.00151	0.012082
PCSK7	0.23499	0.001512	0.012092
COL11A2	-0.10345	0.001521	0.012161
DPH5	-0.31905	0.001527	0.012204
UBE2C	0.575722	0.001527	0.012204
LTBP2	0.309265	0.001531	0.012231
XPO6	0.163951	0.001534	0.012247
ZNF529	-0.34433	0.001535	0.012253
UTP14A	-0.1749	0.001544	0.012322
BRIX1	-0.2147	0.001546	0.01233
ZNF585B	0.172749	0.001547	0.01233
FAM173B	-0.2299	0.001549	0.012342
OST4	0.428861	0.001551	0.012359
STK10	0.277136	0.001555	0.012382
ZNF75D	0.228613	0.001556	0.012386
AUTS2	-0.35013	0.001558	0.012398
CMC4	0.255861	0.001564	0.012445
LOC15368	0.336132	0.001576	0.012529
NOP14	-0.30671	0.001576	0.012529

RPA3	0.467114	0.001577	0.012532
CCRL2	0.605552	0.001583	0.012569
ZNF324	0.359461	0.001583	0.012569
MAD2L2	0.399392	0.001584	0.012569
DCAF7	0.351916	0.001585	0.012569
OR7E37P	0.511293	0.001585	0.012569
MOB3C	0.261115	0.001587	0.012585
GIT2	0.324998	0.001591	0.012613
AK097453	-0.20615	0.001594	0.012633
TROAP	0.309351	0.001595	0.012637
CSDC2	-0.05645	0.001601	0.012674
RHOBTB2	0.098544	0.001609	0.012738
LRRC1	-0.18652	0.00161	0.012738
C11orf86	-0.11446	0.001611	0.012744
EZR	0.551571	0.001613	0.012755
RELL1	-0.24392	0.001614	0.012755
TCIRG1	0.385996	0.001615	0.012758
SEC24B	-0.25902	0.001615	0.012758
IGF2BP2	0.225504	0.001619	0.012783
C16orf72	0.450483	0.001621	0.012791
TMBIM4	0.164562	0.001621	0.012791
IL10	0.331897	0.001623	0.012796
FBXO24	-0.11452	0.001623	0.012796
LOC10192	-0.13452	0.001628	0.012824
IFT80	-0.19322	0.001628	0.012824
FTSJ1	0.254888	0.00163	0.012837
GLRX5	0.325663	0.001636	0.012881
ZNF485	-0.30055	0.001641	0.012906
KIAA1147	0.400307	0.001641	0.012906
ZMAT1	-0.31071	0.001641	0.012906
ACOX1	0.272003	0.001642	0.012906
MANBAL	0.336052	0.001645	0.012928
LINC00463	-0.07664	0.001647	0.012933
AP1AR	0.103922	0.001647	0.012933
NUP88	-0.20465	0.00165	0.012948
TBCD	-0.38858	0.001655	0.012985
RIOK1	-0.21028	0.001656	0.012985
EXOC7	0.358586	0.001657	0.012989
USP3	-0.27124	0.001662	0.013026
SLC35A4	0.413719	0.001663	0.01303
YEATS2	-0.18193	0.001669	0.013075
AHSP	1.288777	0.001671	0.013081
ITFG2	-0.21284	0.001679	0.013134
SCOC	0.447438	0.00168	0.013134
INTS7	-0.34492	0.00168	0.013134
DPP3	0.298457	0.001681	0.013134
MTCH1	0.213306	0.001681	0.013134
HSD17B11	-0.31053	0.001683	0.013146
SIRPB2	0.525452	0.001685	0.013155
CHAMP1	0.529192	0.001686	0.013162
TMEM186	-0.37792	0.00169	0.013186
PAFAH1B1	0.260978	0.001693	0.013204
SUMO3	0.154303	0.001696	0.013222
PTK7	0.077306	0.001699	0.013243
BD495725	-0.56608	0.0017	0.013247
FBP1	0.667512	0.001705	0.013284
TUBB4B	0.394639	0.001707	0.013295
PHKA1	0.318866	0.001716	0.013356

NANS	0.145567	0.001717	0.013358
NAGLU	0.203113	0.001718	0.013358
PLEK2	0.617449	0.001718	0.013358
COBLL1	0.301867	0.001722	0.013387
PML	0.154139	0.001724	0.013395
PLCD1	-0.27138	0.001725	0.013401
EPB41L4A	0.054503	0.001736	0.013475
GAN	-0.17005	0.00174	0.013499
VPS33A	0.175952	0.00174	0.013499
RPS6KA3	-0.24105	0.001741	0.013499
DCUN1D5	-0.28099	0.001742	0.013499
COPE	0.258033	0.001742	0.013499
SLC25A14	-0.24721	0.001752	0.013576
PLCB2	0.171514	0.001753	0.013578
LOC10027(	-0.50134	0.001757	0.013603
NXF3	0.387755	0.001759	0.013614
CAMP	0.840013	0.00176	0.013618
ANKRD49	-0.22134	0.001764	0.013643
ITGAD	0.530689	0.001765	0.013643
TMBIM6	0.211858	0.001767	0.013655
GPI	0.381507	0.00177	0.013669
CDS1	0.21179	0.00177	0.013669
TMEM167/	0.180554	0.001776	0.013712
ZFX	-0.20199	0.001784	0.013766
RAB11FIP3	-0.23181	0.001786	0.013781
ZNF329	-0.26252	0.001792	0.013817
IQCC	-0.22943	0.001797	0.013854
MEFV	0.154317	0.001798	0.013856
NOD2	0.38797	0.001799	0.013861
SS18	0.207066	0.001806	0.013905
ZMAT3	0.464325	0.001815	0.013973
STIL	-0.44784	0.00182	0.014
UBE2D1	0.461383	0.00182	0.014
TAF12	0.494382	0.001823	0.01402
TRIM8	-0.25537	0.001824	0.01402
RP11-350F	-0.24817	0.001825	0.014026
TATDN1	-0.26709	0.001833	0.014084
S1PR3	0.240639	0.001839	0.014118
IQGAP2	-0.28264	0.001839	0.014119
RAB37	-0.41417	0.001841	0.014123
TMEM258	0.266446	0.001841	0.014124
MAGEH1	-0.31761	0.001849	0.014179
SAT1	0.529561	0.001851	0.014191
EIF2S3	-0.22167	0.001853	0.014196
RDH11	0.181723	0.001855	0.014207
NDNL2	0.281106	0.001857	0.014217
DUSP13	-0.08963	0.001866	0.014284
C6orf136	0.307428	0.001867	0.014284
EXOC1	-0.20368	0.00187	0.014303
SLC27A4	0.234833	0.001875	0.014337
DSPP	-0.17218	0.00188	0.014368
FANCL	-0.33213	0.00188	0.014368
ZNF678	-0.32674	0.001882	0.014377
USP18	0.637274	0.001886	0.014403
ZNF776	-0.32217	0.001893	0.014449
C22orf34	-0.22401	0.001895	0.014458
KRR1	-0.23682	0.001902	0.014507
SIGMAR1	0.418864	0.001904	0.014516

ZNF112	-0.48003	0.001906	0.014525
SZRD1	0.228718	0.001906	0.014525
RPAP1	0.266062	0.001911	0.014555
DNASE2B	0.174983	0.001912	0.014555
IK	-0.32339	0.001912	0.014555
LRP3	0.167962	0.001914	0.014562
DUSP16	0.147301	0.001916	0.014571
ZNF135	-0.19137	0.001917	0.014575
MAN2B2	0.313623	0.001921	0.014602
ARL6IP6	-0.30914	0.001925	0.014627
ACBD3	0.206046	0.001926	0.014627
TMLHE	-0.34067	0.001932	0.014665
PDCD6	-0.52821	0.001932	0.014665
TBC1D25	-0.12118	0.001933	0.014668
TOPORS	0.371896	0.001934	0.01467
NDUFAF5	0.299339	0.001935	0.01467
UNC50	-0.17564	0.001937	0.01468
CLPX	-0.17934	0.00194	0.014697
TAF8	0.251651	0.001941	0.0147
UNG	-0.3788	0.001943	0.014709
IPO9-AS1	-0.06496	0.00195	0.014761
UTRN	0.234536	0.001955	0.014792
C6orf15	-0.11814	0.001958	0.014807
DIAPH1	0.199456	0.00196	0.01482
FLI1	0.689261	0.001962	0.014833
TRIM23	0.197184	0.001966	0.014853
NAT10	-0.29013	0.001967	0.014858
FTCDNL1	0.333142	0.001969	0.014865
BSG	0.471258	0.001971	0.01488
COLGALT1	0.222457	0.001976	0.014909
MPC1	0.142234	0.001977	0.014912
CD2BP2	-0.27134	0.001984	0.014957
TESC	0.360754	0.001992	0.015011
SLC15A4	-0.26442	0.001993	0.015011
IPO5	-0.26386	0.001993	0.015011
SRSF10	0.302523	0.001994	0.015011
CARF	0.206783	0.001997	0.015028
NPR2	-0.15869	0.002001	0.015053
BC022047	-0.32663	0.00201	0.015116
RPS29	-0.19704	0.002011	0.015119
NOMO3	-0.35922	0.002015	0.015141
MVB12A	0.254278	0.002015	0.015141
HLA-C	0.263953	0.002016	0.015143
PIP4K2C	0.273488	0.002017	0.015145
B4GALT1	-0.09156	0.002018	0.015145
TSTD1	0.29642	0.002023	0.015179
DCAF6	0.259132	0.002032	0.015244
MAP3K3	0.252453	0.002045	0.015334
HEATR6	-0.3015	0.002048	0.015348
GNLY	0.460059	0.002049	0.015351
GUCY1A3	-0.48903	0.002053	0.015372
NAMPT	0.469309	0.002053	0.015372
KIAA1377	-0.45673	0.002057	0.0154
KAZN	0.244175	0.002059	0.015406
TSC22D4	0.27622	0.00206	0.015411
RASSF3	0.333287	0.002061	0.015414
PIF1	0.122136	0.002067	0.015454
TCEA1	-0.26433	0.002068	0.015456

PARP10	0.073207	0.002071	0.015466
BUD31	0.142647	0.002074	0.015485
SH3TC1	0.37003	0.002084	0.015548
RSU1	-0.16998	0.002084	0.015548
DYNLT1	0.282672	0.002085	0.015548
NDUFB8	0.286374	0.002085	0.015548
WWOX	0.241508	0.002086	0.015548
IMMP2L	-0.32051	0.002089	0.015566
ZNF266	-0.29282	0.0021	0.015641
TMEM86A	0.24824	0.002107	0.015689
AC000111.	0.019714	0.002108	0.015695
SLC8B1	0.164091	0.002113	0.015725
NUDT22	0.152924	0.002114	0.015725
ARHGAP23	-0.12368	0.002135	0.015876
BTAF1	-0.27496	0.00214	0.015908
GDPD1	0.289334	0.002143	0.015927
XPO1	-0.25773	0.002145	0.015937
BIVM	0.31183	0.002146	0.015939
DNAJA3	-0.2952	0.002154	0.015984
PIP5K1C	0.284169	0.002154	0.015984
KCNIP2-AS	-0.17648	0.00216	0.016022
ADAM17	-0.23697	0.002161	0.016026
XRCC6	-0.22153	0.002164	0.016045
MBLAC2	-0.26387	0.002166	0.016053
MED7	0.334063	0.002171	0.016086
POC1B	-0.39237	0.002175	0.01611
PKIB	0.246335	0.002179	0.016132
SSSCA1	0.380826	0.002185	0.016169
LAMP3	0.487292	0.002192	0.016214
PAWR	-0.44063	0.0022	0.016271
SUMF2	0.348245	0.002203	0.016286
BNIP2	-0.21252	0.002211	0.016338
RNF146	0.290129	0.002211	0.016338
R3HDM2	0.219873	0.002213	0.016345
NRGN	0.828179	0.002223	0.016414
ZSCAN31	-0.41856	0.002224	0.016414
ZFP69B	0.323078	0.002225	0.016414
SLC52A2	0.301626	0.002231	0.016452
HADHA	0.284471	0.002232	0.016454
PRDX3	0.146686	0.002235	0.016473
AKT3	-0.36299	0.002242	0.016515
C6orf62	0.424689	0.002242	0.016515
GPRIN2	-0.03206	0.002243	0.016515
C19orf82	-0.28349	0.002244	0.016515
TMEM138	0.23383	0.002245	0.016518
INPP5B	0.22775	0.002248	0.016533
N4BP2	-0.36707	0.002252	0.016556
NQO2	0.282237	0.002253	0.016556
NDUFB9	0.284107	0.002253	0.016556
MAP3K12	0.209522	0.002263	0.016623
PDIA6	0.294801	0.002266	0.016637
DCTN4	0.420066	0.002281	0.016745
SLC6A8	0.398099	0.002286	0.016775
DNAJA4	0.492294	0.002288	0.016781
IL6	0.552378	0.002288	0.016781
MRC1	1.074429	0.00229	0.016786
CARD16	0.306786	0.002305	0.016889
PUS1	-0.37788	0.002305	0.016889

PIN1	0.28107	0.002307	0.016897
ECSCR	0.29915	0.002309	0.016897
GPR155	0.385042	0.00231	0.016897
OSGIN1	0.241477	0.00231	0.016897
TMED8	0.332648	0.00231	0.016897
PTP4A1	0.333525	0.00232	0.016963
PAPD5	0.274671	0.002322	0.016966
FOXRED2	0.229746	0.002322	0.016966
WRN	-0.30776	0.002325	0.016984
SPRR2B	-0.09513	0.002331	0.017014
ZC4H2	-0.21557	0.002331	0.017014
KIAA1429	-0.40449	0.002334	0.01703
SCRN1	-0.52561	0.002343	0.017088
KANK2	-0.10691	0.002348	0.017117
MYADM	0.361319	0.002349	0.017118
ANO6	-0.1749	0.002356	0.017166
NFIX	0.193191	0.002359	0.017183
DNAJA1	0.16905	0.002364	0.017212
FAM208B	0.299451	0.002376	0.017292
VCAM1	1.486167	0.00238	0.017317
NBR2	-0.2782	0.002385	0.017348
DNAJA2	0.221906	0.002386	0.017348
LOC55420(	-0.09682	0.002387	0.017354
IER3IP1	-0.18297	0.002395	0.017407
CSNK2A1	0.227857	0.002398	0.017417
SLC41A3	-0.28756	0.002402	0.017442
SERPING1	0.455528	0.002408	0.017479
OGFOD3	0.254831	0.00241	0.017486
GNL2	-0.24914	0.002415	0.01752
PCDHGB5	-0.05039	0.002422	0.017562
HM13	0.311582	0.002431	0.017624
TIPRL	0.21039	0.002433	0.017631
RP11-517E	-0.19447	0.00244	0.017678
ANXA2	0.518265	0.002455	0.017777
POLK	0.221714	0.002456	0.017781
LGALS1	0.824714	0.002457	0.017782
RP11-90P1	-0.08416	0.002458	0.017782
FAM98B	0.499971	0.002459	0.017782
BCDIN3D	0.289646	0.002462	0.017803
RPLP2	-0.18627	0.002467	0.01783
HDDC2	0.267241	0.002476	0.017878
REV3L	-0.31375	0.002476	0.017878
TACC1	0.398208	0.002476	0.017878
OXT	0.102773	0.002482	0.017918
LSM10	0.326146	0.002485	0.017932
TUBA1B	0.215841	0.002496	0.018004
MIR10A	0.532411	0.002499	0.018024
ANXA2P2	0.400139	0.00251	0.01809
KARS	-0.15895	0.00251	0.01809
CLEC16A	0.14437	0.002512	0.018096
CHSY1	-0.23545	0.002522	0.018163
CDS2	0.315173	0.002524	0.018173
LOC28402:	-0.325	0.002531	0.018217
FAM213A	0.495065	0.002534	0.018233
CMTM3	0.357273	0.002537	0.018244
CNPY3	0.256375	0.002541	0.018265
AC005523.	-0.46798	0.002543	0.01828
GCLC	0.322303	0.002544	0.018281

RAD51-AS	0.274404	0.002549	0.018311
KLHL18	0.232543	0.002551	0.018314
ANKRD40	0.183807	0.002552	0.018314
PRB3	-0.21591	0.002552	0.018314
LOC10099(	-0.5249	0.002555	0.018329
ZDHHC18	-0.0829	0.002557	0.018335
TMEM92	0.085996	0.002558	0.018339
YRDC	0.260882	0.002563	0.018361
CTD-2528I	-0.42317	0.002564	0.018361
SLC17A7	-0.06976	0.002564	0.018361
HMGN3	-0.25252	0.002566	0.018366
CALHM1	-0.09493	0.002568	0.018381
EPB41L4A-	-0.38228	0.002569	0.018382
PABPC1	0.095151	0.002573	0.018401
SESN2	0.304592	0.002579	0.01844
SCARNA17	-0.63858	0.002586	0.018483
TRAF7	0.176871	0.00259	0.018502
API5	-0.33476	0.002592	0.018511
SH3BP5	0.73659	0.002595	0.018526
LOC10012!	0.06253	0.002596	0.018526
SNX11	0.306775	0.002599	0.018545
SEMA4B	0.289307	0.002601	0.018553
RBM39	-0.32273	0.002609	0.018604
DYNC1H1	0.11119	0.00261	0.018604
RP11-357(	-0.18101	0.002613	0.018618
TMEM33	-0.24225	0.002614	0.018618
CXCL1	0.866008	0.002615	0.018624
GAS7	0.356202	0.002631	0.01873
ID1	0.89132	0.002632	0.018731
GRIN3A	0.110557	0.002638	0.018766
PRPS1	0.28709	0.00264	0.018774
C7	0.452809	0.002642	0.018782
SAP30BP	0.194232	0.002643	0.018783
NLRX1	0.176564	0.002645	0.018788
ZNF668	0.132841	0.002654	0.018852
RP11-379F	-0.12633	0.002656	0.018856
STX12	0.319564	0.00267	0.018948
CCDC151	0.215611	0.002675	0.018976
ANKRD52	0.202065	0.002675	0.018976
TRMT2B	-0.3304	0.002678	0.018991
LOC10192	0.337853	0.002687	0.019048
TOP2B	-0.29965	0.002694	0.019083
CAAP1	-0.26137	0.002694	0.019083
AURKB	0.409647	0.002697	0.019099
RHEB	-0.24001	0.00271	0.019182
ZNF669	-0.26117	0.002713	0.019203
PPP4R2	-0.37192	0.002724	0.019272
OMD	0.065925	0.002727	0.019288
CD69	-0.68208	0.002732	0.019317
FRS3	0.0611	0.002739	0.019358
LYL1	0.323074	0.002741	0.019369
GATA2	0.367779	0.002754	0.019454
KIF13A	0.268026	0.002756	0.019457
BLOC1S3	0.165714	0.002764	0.01951
C11orf73	0.28894	0.002773	0.019564
NFE2	0.457433	0.002774	0.019569
ENPP7	-0.23452	0.002783	0.019621
ZNF101	-0.24153	0.00279	0.019668

LOC73013!	-0.12018	0.002798	0.01972
FUK	0.244583	0.002803	0.019746
CEACAM1!	0.081846	0.002808	0.019772
PRDX1	0.316132	0.002814	0.019807
WWP1	0.514745	0.00282	0.019845
ZNF544	-0.18625	0.002822	0.019856
CHPT1	0.39026	0.002825	0.019867
ATXN1	0.345189	0.002831	0.019902
ZBTB5	-0.31874	0.002832	0.019904
IVNS1ABP	0.345754	0.002834	0.019909
HCG18	-0.19254	0.002836	0.019919
ELP5	0.423686	0.002842	0.01995
TSPO	0.453776	0.002842	0.01995
CD300C	0.225092	0.002851	0.020006
PACRGL	0.247961	0.002856	0.020036
WDR54	-0.35163	0.002857	0.020037
STAT2	0.148733	0.00286	0.020049
PCBP4	-0.13243	0.002863	0.020061
PRR22	0.046581	0.002866	0.020076
RANBP17	-0.27765	0.002873	0.020123
MYZAP	0.627817	0.002878	0.02015
MAP4K5	0.294023	0.002888	0.020213
TANK	0.354766	0.00289	0.020222
CEP131	0.213157	0.002892	0.020228
FOXP1-IT1	-0.39231	0.002893	0.020228
PITPNB	-0.17009	0.002895	0.020239
RUNDC3A	0.284531	0.002903	0.020283
TMEM11	0.245829	0.002904	0.020283
ID2	0.829719	0.002918	0.020378
NCF2	0.889644	0.00292	0.020385
SPEN	0.363967	0.002924	0.020403
CREBZF	-0.35063	0.002925	0.020408
LOC10050!	0.013497	0.00293	0.020432
DNAJC28	0.172973	0.002939	0.020493
TIAM2	-0.29568	0.00294	0.020493
NKG7	0.67289	0.002942	0.020497
REXO2	0.269591	0.002949	0.02054
CDR1	0.021212	0.002953	0.02056
USP46-AS:	0.180713	0.002956	0.020577
MAP4K4	-0.28725	0.002957	0.020578
OPLAH	0.270633	0.002964	0.020616
MERTK	0.407335	0.002965	0.020622
SARAF	-0.23471	0.002967	0.020624
ANP32A	-0.1707	0.002975	0.020678
DAPK1	0.20404	0.00298	0.020702
KIAA0247	0.236567	0.002982	0.02071
HINFP	-0.32328	0.002985	0.020726
SLC35B3	-0.14986	0.002997	0.020804
QPCT	0.727519	0.002998	0.020804
SULF2	0.326101	0.003002	0.020808
ZBTB8A	-0.43368	0.003003	0.020808
PCK2	0.326755	0.003003	0.020808
TLN2	0.182782	0.003004	0.020808
MAPRE3	-0.03683	0.003005	0.020808
MGAT4B	0.354926	0.003005	0.020808
DNAJB4	0.378129	0.003007	0.02082
MRPS31	0.400463	0.003015	0.020868
AGK	-0.24011	0.003018	0.020881

SEC13	0.254267	0.003024	0.020913
VNN2	0.824802	0.003027	0.020928
DNPH1	0.491167	0.003035	0.020977
MRPL37	0.29594	0.003041	0.021012
MED16	0.285396	0.00305	0.021066
SLC16A4	-0.22371	0.003062	0.021145
ZKSCAN7	-0.24778	0.003063	0.021147
YAF2	0.183871	0.003065	0.02115
CTD-2541I	-0.31792	0.003067	0.021151
VTI1A	-0.29998	0.003067	0.021151
S100A13	0.218795	0.003073	0.021187
RP4-612B1	-0.02632	0.003074	0.021187
LGALS3BP	0.856025	0.003082	0.021235
CTD-2292I	-0.50166	0.003088	0.021266
RHOBTB3	0.29875	0.003092	0.021292
EDNRB	0.862893	0.003097	0.02132
MIEF2	0.180453	0.003099	0.021327
DFNA5	0.511905	0.003101	0.021332
UQCR11	0.214989	0.003107	0.021368
RP1-93H18	0.495014	0.003112	0.021395
DGCR2	-0.11692	0.003116	0.021413
CALML4	0.314288	0.003121	0.02144
RPL9	-0.06319	0.003124	0.021454
WDR62	-0.16241	0.003136	0.021527
CD80	0.199033	0.003136	0.021527
PSTK	-0.1538	0.003143	0.021567
ZBED5	-0.1693	0.003152	0.02162
LOC100505	-0.4681	0.003159	0.02166
THOC5	-0.14471	0.003165	0.021697
IPCEF1	0.530528	0.003171	0.021728
KIAA0319L	0.189154	0.003178	0.021772
ITGAL	0.491569	0.003185	0.02181
ARHGEF37	0.294972	0.003186	0.02181
THAP5	-0.23124	0.003187	0.02181
RP11-477N	-0.31596	0.003188	0.02181
LOXL3	0.306455	0.00319	0.021821
OTUD5	0.235404	0.003192	0.021825
CORO2A	0.294225	0.003202	0.021888
RNF31	0.076449	0.00321	0.021934
CHSY3	0.283355	0.003211	0.021936
LINC01315	0.228592	0.003219	0.021981
PET100	0.328665	0.00322	0.021981
GNB1	0.194923	0.003222	0.02199
PLSCR1	0.336529	0.003224	0.021995
WIPI1	0.630878	0.00323	0.02203
MNDA	0.911434	0.003232	0.02203
LOC153546	-0.44121	0.003232	0.02203
MRPL39	-0.25993	0.003234	0.022035
RUSC2	0.140501	0.003236	0.022046
LNP1	-0.29618	0.003241	0.022073
RORA	0.182048	0.00325	0.022124
RAD51AP1	-0.4682	0.003265	0.022223
ARMC10	0.248704	0.003281	0.022322
MCFD2	0.345856	0.003283	0.022331
EFCAB7	-0.28909	0.003287	0.022346
DGKH	-0.18006	0.003288	0.022346
TEX9	-0.13283	0.003291	0.022358
SNRNP27	0.196303	0.003291	0.022358

LOC10050	-0.16953	0.003293	0.022363
UBR7	-0.21835	0.003303	0.022424
MLLT10	0.210529	0.00331	0.022463
RPL36AL	0.2621	0.003317	0.022501
RPL36A	-0.39875	0.003321	0.022525
CSTF1	-0.22777	0.003322	0.022525
RP5-894A1	0.395536	0.003329	0.022561
NONO	0.200146	0.00333	0.022561
TCF7	-0.04754	0.003331	0.022561
LINC00836	0.039753	0.003333	0.022561
ADHFE1	-0.41392	0.003334	0.022561
PPARGC1B	0.171411	0.003334	0.022561
AC006026.	-0.38837	0.003336	0.022565
CD93	0.506978	0.003337	0.022565
PAK1	0.294596	0.003339	0.022578
SLC47A1	0.577312	0.003342	0.022587
MS4A14	0.380331	0.003343	0.022587
ITSN1	0.083299	0.003351	0.022633
LOC40113	-0.22919	0.003356	0.022662
INSL3	0.031446	0.003367	0.022725
SRFBP1	-0.38759	0.003387	0.022854
ARSA	0.082323	0.003393	0.022891
ZNF813	-0.22564	0.003396	0.022897
RP11-349E	-0.20585	0.003397	0.022897
CHCHD1	0.242968	0.003397	0.022897
CXCR2	0.686224	0.003401	0.022907
SMIM10	0.509254	0.003401	0.022907
TTPAL	0.198105	0.003402	0.022908
NFRKB	0.207361	0.003406	0.022922
WDR24	0.148808	0.003406	0.022922
GLIDR	-0.27141	0.003417	0.022983
CENPT	0.067821	0.003418	0.022983
LOC10272	0.257939	0.003428	0.023049
FBXL12	0.108675	0.003431	0.023052
CHMP1A	0.222822	0.003431	0.023052
LEMD3	-0.22674	0.003432	0.023052
FLVCR1	-0.29264	0.003435	0.023056
MRPL17	0.399228	0.003436	0.023056
ALDH5A1	0.250305	0.003436	0.023056
CLN8	0.28388	0.003438	0.023063
ZER1	0.142314	0.003445	0.023104
PHACTR4	0.262478	0.003452	0.023141
NAB2	0.225332	0.003454	0.023142
COX14	0.254276	0.003454	0.023142
ATP6V1E2	-0.29673	0.003456	0.023148
POLR2H	-0.25931	0.003458	0.023156
PABPN1	0.377763	0.003466	0.023199
CHRNB2	-0.09474	0.003472	0.023234
UST	0.194805	0.003473	0.023234
NRBP1	0.319139	0.003479	0.023266
F2RL2	-0.16112	0.003481	0.02327
PEX10	0.169981	0.003482	0.02327
SLC38A1	-0.63	0.003484	0.023277
CCBL1	-0.11238	0.003489	0.023307
ZCCHC10	0.347015	0.003493	0.023324
KDELR2	0.290891	0.003496	0.02334
AC092192.	0.022105	0.003506	0.023398
NOL12	0.216566	0.003512	0.023433

PMEPA1	0.429988	0.003518	0.023464
D2HGDH	-0.43981	0.003522	0.02348
ATP5G2	0.190298	0.003528	0.023514
STARD5	0.300079	0.003532	0.023537
OPTN	0.465849	0.003538	0.023565
EML4	0.175975	0.003539	0.023565
MEIS3P1	0.595312	0.00354	0.023565
FAM171B	0.131402	0.003545	0.023591
INE1	-0.17056	0.003548	0.023603
SMARCA1	0.42436	0.003552	0.023616
ZC3HAV1	0.216553	0.003552	0.023616
NPPA	-0.13823	0.003557	0.023645
OSBP2	0.281342	0.003566	0.023693
ZNF608	0.304286	0.003569	0.023705
MLLT11	-0.36467	0.003572	0.023716
SPTB	0.17466	0.003573	0.023716
RALB	0.240171	0.003577	0.023732
ATXN7L3B	0.330383	0.003578	0.023732
C12orf73	-0.27595	0.003578	0.023732
LUC7L2	-0.24493	0.003581	0.023746
STK40	0.197028	0.003594	0.02382
BTG2	0.369296	0.003595	0.023821
ZBTB7C	0.109535	0.003598	0.023834
GGTA1P	0.727687	0.0036	0.023838
C1orf131	-0.29568	0.003601	0.023838
GJD2	-0.10931	0.003602	0.023839
DDOST	-0.24709	0.003605	0.023854
KBTBD4	-0.14242	0.00362	0.023947
RP11-29H2	-0.32262	0.003622	0.023951
C3orf62	0.065976	0.003627	0.023971
KIF5B	0.193562	0.003627	0.023971
KREMEN1	0.054078	0.003631	0.023987
UPF2	0.235759	0.003632	0.023987
TSPYL4	-0.30147	0.003638	0.024016
PEX2	-0.26687	0.003652	0.024102
OMA1	-0.40324	0.003654	0.024107
VPS28	0.254344	0.003658	0.024127
CDK4	0.339202	0.003689	0.024323
G6PC3	0.27608	0.00369	0.024327
GTSE1	0.492039	0.003706	0.02442
B3GNT2	0.431006	0.003728	0.024557
LOC10050	-0.17856	0.00373	0.024563
KDM6B	0.377636	0.003737	0.024607
RP11-819C	-0.34288	0.003739	0.02461
DDX49	0.274319	0.003746	0.024648
JMJD4	0.151713	0.003759	0.024726
TWSG1	-0.24279	0.003764	0.02475
AC009133.	0.132041	0.003781	0.024855
ZFP36	0.603928	0.003784	0.024861
ZZEF1	0.229878	0.003784	0.024861
F8	0.431559	0.003792	0.024907
PADI1	-0.069	0.003797	0.024931
RAP1GAP	0.111892	0.003804	0.024971
ZBTB41	-0.4228	0.003806	0.024971
RP11-488L	-0.48854	0.003806	0.024971
RP11-357C	-0.03563	0.003813	0.025001
ATP6V1H	0.325987	0.003815	0.025001
ATG5	0.285668	0.003815	0.025001

NUP50	0.295535	0.003816	0.025001
RASGRP2	0.242586	0.003822	0.025035
SYS1	0.234598	0.00383	0.025078
ZNF467	0.208411	0.003836	0.025113
R3HDM1	-0.14937	0.003841	0.025124
MYLK-AS1	-0.3776	0.003842	0.025124
NRP2	0.022881	0.003842	0.025124
SLC18B1	0.629894	0.003843	0.025124
MTMR1	-0.11232	0.003845	0.025131
ERCC3	-0.22237	0.003851	0.025163
SLC25A25	0.260566	0.003857	0.025192
PSMC5	-0.23192	0.003859	0.025192
IL17C	-0.13464	0.003861	0.025192
RP11-334C	0.093643	0.003861	0.025192
LOC15357	0.417715	0.003861	0.025192
LPL	0.74563	0.003863	0.025196
CCDC152	-0.39351	0.003868	0.025222
PVRL2	0.227058	0.00387	0.025226
CHRNA1	0.084021	0.003873	0.025242
TET1	-0.41955	0.003885	0.025312
ZC3H7B	0.11724	0.003897	0.025383
CBLB	0.298221	0.003901	0.025398
CD151	0.302766	0.003903	0.025404
EBPL	-0.25971	0.003907	0.025424
CDC42-IT1	-0.49694	0.003911	0.025438
ISG20	0.720832	0.003953	0.025698
CEP95	-0.32506	0.003953	0.025698
SLCO1B1	0.022168	0.003956	0.025707
DUT	0.299216	0.003957	0.02571
SP3	-0.35631	0.003961	0.025725
KIAA1598	0.759537	0.003962	0.025725
SNRPA1	-0.1954	0.00398	0.025833
NR6A1	-0.03938	0.003981	0.025833
LINC00909	-0.23855	0.003983	0.025839
GABARAPL	0.444012	0.003986	0.025853
BMP2K	0.416611	0.004007	0.025971
COPRS	0.25455	0.004007	0.025971
SLC7A1	0.121277	0.00401	0.025983
APOBR	0.307199	0.00402	0.026038
TTC19	-0.2515	0.004022	0.026047
TIGD7	-0.43238	0.004033	0.026108
SNX30	0.301401	0.004043	0.026168
STARD9	-0.51014	0.004046	0.026176
SHE	0.456393	0.004052	0.026208
POMGNT2	0.412648	0.004053	0.026208
SLC25A11	0.326964	0.00406	0.026245
ALG9	-0.24005	0.004065	0.026272
SOD1	0.195545	0.004081	0.026363
SPRYD3	0.252575	0.004083	0.026373
CENPF	-0.47571	0.004087	0.026378
EPSTI1	0.541755	0.004088	0.026378
NAGA	0.327475	0.004088	0.026378
DUSP22	-0.31223	0.004097	0.026429
STAB1	0.343151	0.004101	0.026444
DNAJC4	-0.09209	0.004119	0.026557
CAP1	0.18079	0.004127	0.026594
LOC10012	-0.06646	0.004128	0.026594
GPR160	0.541403	0.004138	0.026651

BCL9L	-0.09124	0.004139	0.026651
LOC81691	-0.31022	0.00416	0.026781
LOC10050	-0.12738	0.004166	0.026813
KLRAP1	0.039117	0.004172	0.026839
DPY19L4	-0.32654	0.004173	0.026839
MTDH	-0.21155	0.004187	0.026924
XYLT1	-0.19868	0.004189	0.026924
CYP2A6	-0.01821	0.00419	0.026924
CDADC1	-0.263	0.004195	0.026948
NFIA	0.611316	0.004201	0.026981
ANAPC2	0.21954	0.004203	0.026983
YIPF5	0.393976	0.004208	0.027
AMPD3	-0.2944	0.004208	0.027
GPR137C	0.124722	0.00421	0.027
CRKL	0.098466	0.004211	0.027
EIF2A	-0.23466	0.004217	0.027036
FLNA	0.414536	0.004226	0.027084
ADAT2	-0.24618	0.00423	0.027103
PSMA5	-0.21443	0.004233	0.027112
ECSIT	0.329224	0.00424	0.027146
ZNF557	0.112779	0.004242	0.027156
TPK1	0.396887	0.004252	0.027207
SLC26A6	-0.49204	0.004264	0.027276
CXorf56	-0.3114	0.004276	0.027348
LOC10013	-0.07925	0.004286	0.027405
FASTKD1	-0.24364	0.004289	0.02741
HSPE1	0.291399	0.00429	0.02741
ZNF766	-0.20464	0.004291	0.02741
HNRNPC	0.256737	0.004294	0.02742
MITF	0.547094	0.004297	0.027435
CDKN3	0.554796	0.004308	0.027496
G2E3	-0.21836	0.004323	0.02758
BTG3	0.228183	0.004334	0.027645
ZNF276	0.166778	0.00434	0.027676
STAB2	0.249923	0.004342	0.027678
C7orf60	-0.24977	0.004348	0.027708
KLHDC10	0.315263	0.004354	0.027738
LOC10013	-0.31295	0.004361	0.027775
H2BFXP	-0.22504	0.004362	0.027775
TMEM106	0.158877	0.004366	0.027792
ALKBH8	-0.23889	0.004368	0.027793
ZNF638	-0.24983	0.004373	0.027815
ZNF559	-0.30609	0.004374	0.027815
RP11-112J	-0.46554	0.004381	0.027852
BTN3A3	0.298274	0.004383	0.02786
PPM1B	-0.27445	0.004386	0.02787
RP11-214K	0.024237	0.004391	0.027896
LOC10013	-0.18715	0.004402	0.027952
CTD-2540	0.139907	0.004406	0.027971
DLL4	-0.16469	0.004412	0.027992
C16orf70	-0.12145	0.004413	0.027992
PPIG	0.307203	0.004414	0.027992
ACAP1	-0.3603	0.004414	0.027992
WDR55	0.181698	0.004418	0.028009
SLC37A1	0.151959	0.004425	0.028046
TRIM52	0.370109	0.004434	0.028094
TCEAL1	-0.21264	0.004437	0.028104
FRY	-0.29125	0.004444	0.028138

ADO	-0.15888	0.004449	0.028164
CSNK2B	0.258114	0.004458	0.02821
ANKS6	0.173719	0.00446	0.02821
SPATA5	-0.41019	0.00446	0.02821
EML2	-0.11054	0.004472	0.028275
FBXO25	0.351582	0.004476	0.028295
CYP4X1	0.513775	0.004483	0.028327
SLC43A1	0.312584	0.004493	0.028368
EAPP	0.238737	0.004493	0.028368
CRAT	0.185335	0.004494	0.028368
NUPL1	-0.26427	0.004495	0.028368
TWF2	0.219574	0.004504	0.028422
HNRNPF	-0.26166	0.004506	0.028425
FBXL4	-0.30119	0.004511	0.028449
RSPH1	-0.48775	0.004514	0.028456
LINC00588	0.010082	0.004516	0.028459
C11orf24	0.171718	0.004518	0.028467
HDDC3	0.264598	0.004528	0.028524
WNK1	0.476507	0.004531	0.028532
PWAR6	-0.58894	0.004533	0.028538
GLT8D1	-0.20116	0.004537	0.028551
LOC15368:	-0.44447	0.004538	0.028551
ZRANB2	-0.16542	0.004541	0.028563
ZNF641	0.180618	0.004548	0.028594
FERMT3	0.297568	0.004553	0.028619
CNOT7	-0.20878	0.004557	0.028636
KPNA5	-0.43826	0.004559	0.028639
BHLHE41	0.084682	0.004563	0.028654
RP9	0.177219	0.004564	0.028654
TF	0.281092	0.00458	0.028748
LECT1	0.138911	0.004582	0.02875
PMS2P3	0.255368	0.004606	0.028887
NPDC1	0.514417	0.004606	0.028887
LSR	0.185936	0.004608	0.028892
IL6ST	0.478106	0.004619	0.028951
ADAM11	-0.06637	0.004621	0.028957
MCMBP	-0.30624	0.004631	0.029004
IQGAP3	-0.0566	0.004632	0.029004
FAM114A1	0.281069	0.004633	0.029004
AKR1A1	0.216576	0.004636	0.029014
CCDC176	-0.22865	0.004656	0.02913
SDCBP2-A	-0.16284	0.004658	0.029139
FBXO28	-0.2221	0.004671	0.029207
ZC3H13	-0.29072	0.00468	0.029259
TCEB3	0.187842	0.004688	0.029296
CHD4	0.269803	0.004689	0.029296
LRRTM2	-0.16441	0.004691	0.029296
MCHR2-AS	-0.03916	0.004692	0.029296
LETMD1	-0.28101	0.004699	0.029335
ZNF566	0.341785	0.004703	0.029341
ZNF480	0.181038	0.004703	0.029341
8-Mar	0.343416	0.004708	0.029367
FTH1P5	0.238236	0.004712	0.02938
CEACAM8	0.92263	0.004726	0.029454
LOC10192	-0.06473	0.004726	0.029454
PTMA	0.137006	0.004733	0.029486
TRIM10	0.136702	0.004738	0.029507
PIN4	-0.3193	0.004741	0.029518

LINC00515	-0.18008	0.004745	0.029532
SAYSD1	-0.28989	0.004746	0.029534
CTC1	0.195798	0.004753	0.029566
SYAP1	0.353564	0.00476	0.029598
PSTPIP2	0.31723	0.004761	0.029598
COA4	0.179159	0.004762	0.029598
RP11-308D	-0.07051	0.004764	0.029605
LRRC37A2	-0.24499	0.004776	0.029667
CNTRL	-0.28819	0.004794	0.029769
CARD10	0.119643	0.004806	0.029837
MGA	0.355661	0.004809	0.02985
ARHGDIG	-0.0693	0.004821	0.029911
CRY2	0.13613	0.004823	0.029916
SMG9	0.233178	0.004839	0.030008
FAM174A	-0.34249	0.004845	0.030035
SACM1L	-0.28289	0.004848	0.030046
BHLHB9	0.302015	0.004855	0.030076
PXDC1	0.388986	0.004855	0.030076
STK16	0.137452	0.004867	0.030137
ZSWIM7	0.281527	0.00487	0.030151
ZSCAN26	0.321241	0.004878	0.030187
SMARCD2	-0.26687	0.004881	0.030198
FGD2	0.11142	0.004894	0.030263
LOC10013(	-0.04828	0.004894	0.030263
ENC1	0.303561	0.004897	0.030263
CELSR2	0.040993	0.004897	0.030263
LONP1	0.297825	0.004905	0.030302
SPRY2	0.570447	0.004906	0.030302
LOC28369:	-0.04206	0.00491	0.030317
ST3GAL5	0.465914	0.004918	0.03036
NUDT16P1	0.119018	0.00492	0.03036
SCAMP3	-0.25269	0.004921	0.03036
SHKBP1	0.197302	0.004925	0.030375
WDR45B	-0.22215	0.004941	0.03046
TAB2	0.310049	0.004942	0.03046
LOC10050(	0.250029	0.004943	0.03046
TNPO3	0.237654	0.004951	0.030499
TMEM191/	0.099183	0.004959	0.030536
ANKRD39	0.324249	0.004959	0.030536
UGDH	-0.32959	0.00497	0.030595
NCK1-AS1	0.428933	0.004975	0.030609
ATF4	0.161955	0.004975	0.030609
C10orf128	0.381422	0.004984	0.030655
BCAS2	-0.21499	0.004987	0.030665
CCNK	0.094328	0.004991	0.030678
SMEK1	0.347046	0.004992	0.030678
DBT	0.231799	0.005013	0.030794
STK38L	-0.19706	0.005031	0.030895
KYNU	0.69918	0.005047	0.030986
LRRC46	-0.0622	0.005058	0.031048
TADA2B	0.305331	0.005072	0.031124
ZNF570	-0.11381	0.005095	0.031253
ZNF367	-0.39592	0.0051	0.031279
GDI2	0.199923	0.00511	0.031326
SLC25A12	-0.20807	0.005112	0.031326
RP11-680(	-0.35174	0.005112	0.031326
USP40	0.209235	0.005116	0.031341
MICU3	-0.50703	0.005121	0.031365

TARSL2	-0.12591	0.005125	0.031377
KCNJ5	0.165594	0.005131	0.031406
DEPTOR	-0.18592	0.005137	0.031425
PLEKHO1	0.466469	0.005138	0.031425
UBXN4	0.220435	0.005139	0.031425
ZBTB38	0.216242	0.005146	0.031459
ASB13	-0.26545	0.005148	0.031459
PKD1P1	0.380991	0.005149	0.031459
FBRS	0.191869	0.00515	0.031459
CNIH4	-0.29274	0.005163	0.031532
TBC1D30	0.106244	0.00517	0.031564
LRRC74	-0.05922	0.005175	0.031586
LINC01339	-0.13315	0.005193	0.031685
ZNF30	-0.37039	0.005209	0.031774
ZSCAN16	-0.25908	0.005219	0.031829
NPIPA1	0.371699	0.005222	0.031838
TSC22D1-/	-0.20116	0.005227	0.03186
TRIM26	0.294872	0.005239	0.031921
GCNT7	-0.14324	0.005251	0.031986
HTR7P1	0.17319	0.005254	0.031993
ERCC1	0.227418	0.005263	0.032043
RP9P	0.186877	0.005265	0.032045
MFSD5	0.316204	0.005267	0.032045
LYG1	-0.23919	0.005271	0.032065
DUSP14	-0.28018	0.005278	0.032098
APBB1IP	-0.36754	0.005304	0.032238
PRKRIR	-0.1614	0.005304	0.032238
PFDN5	0.329788	0.005306	0.032238
PRX	-0.10097	0.005307	0.032239
NUSAP1	0.474345	0.005312	0.032259
LOC72873(	0.135978	0.005321	0.032303
LSM7	0.418178	0.005348	0.032455
C2orf68	-0.30917	0.005351	0.032459
GNB4	-0.32665	0.005353	0.032459
CEP250	0.070361	0.005354	0.032459
USP19	-0.09805	0.005354	0.032459
KIAA0825	-0.04436	0.005356	0.032459
PEX11G	0.286967	0.005365	0.032507
ARHGAP35	0.22193	0.005376	0.032565
WDPCP	-0.17318	0.005378	0.032565
SENP1	-0.13348	0.00538	0.032569
ZNF207	-0.33676	0.005384	0.03258
ZHX1-C8o	-0.33082	0.005385	0.03258
LOC10192	-0.33767	0.005389	0.032596
CCT6A	-0.18006	0.005397	0.032638
DLD	-0.2108	0.005403	0.032662
PCDHGA8	-0.11365	0.005412	0.03271
KLHL22	0.039725	0.00542	0.032748
EPT1	-0.29999	0.005424	0.032761
CENPA	0.285785	0.005425	0.032761
SPECC1L	-0.23925	0.005428	0.032768
GLE1	0.202431	0.005431	0.032777
GPR153	0.109186	0.005432	0.032777
DENND2D	0.28268	0.005435	0.032787
IKBKB	0.36397	0.005442	0.032817
RBX1	0.274079	0.005453	0.032868
ZMAT2	0.226435	0.005453	0.032868
TTYH2	0.171582	0.005458	0.03288

FBXO42	-0.15996	0.005458	0.03288
ERO1L	-0.26967	0.005461	0.032888
ZCCHC8	-0.16286	0.005476	0.032956
C4orf46	-0.30998	0.005477	0.032956
PRPF31	0.369422	0.005477	0.032956
C11orf57	-0.20817	0.005487	0.033008
ZDBF2	0.278334	0.00552	0.033198
BAIAP2-AS	0.270574	0.005529	0.033241
TBC1D5	0.227507	0.005537	0.03328
ZW10	-0.25025	0.005539	0.033282
ABL2	0.314489	0.005555	0.03337
LINC00869	0.163798	0.005562	0.033398
RWDD2A	0.134882	0.005563	0.033401
RGS20	0.027889	0.005571	0.033438
CMBL	0.312991	0.005574	0.033445
SUSD5	0.32067	0.005579	0.033469
RAD21	0.231016	0.005582	0.033475
DCTN1	0.224349	0.005622	0.033706
ZMIZ1	-0.45674	0.005624	0.033708
GAS2	0.274254	0.005646	0.033829
TJP1	0.322607	0.005648	0.033835
ZNF473	-0.25536	0.005653	0.033843
ASH1L	-0.22379	0.005653	0.033843
SLC39A1	0.209547	0.005655	0.033843
TMEM99	-0.46042	0.005656	0.033843
MYNN	-0.25964	0.005659	0.033851
BEX4	0.264649	0.005661	0.033853
TUBB2A	0.968513	0.005663	0.033858
CHST2	0.30497	0.005684	0.033973
CYGB	-0.07307	0.005692	0.034006
CENPK	-0.54395	0.005692	0.034006
ANKS1A	0.251818	0.005699	0.034038
SPRED1	0.361813	0.005712	0.034104
LOC72848	-0.30307	0.005724	0.034163
ARMCX1	-0.40061	0.005738	0.03424
AC005256.	-0.0385	0.005769	0.034415
TMEM106	0.168461	0.005771	0.034417
ASH1L-AS	-0.30875	0.005778	0.034452
FH	0.237359	0.00578	0.034452
RIN2	0.316882	0.005797	0.034542
MRM1	0.178331	0.005802	0.034564
LRRC41	0.228995	0.005808	0.03459
TMX2	0.190646	0.005813	0.034612
ZNF609	0.299705	0.005826	0.034679
MTRF1	-0.19214	0.00583	0.034695
SLC44A2	0.462914	0.00584	0.034745
NIF3L1	-0.33327	0.005849	0.034785
C11orf30	-0.13846	0.005854	0.034807
GAREM	0.137014	0.005863	0.034843
EXOC3L2	-0.18246	0.005864	0.034843
PYY2	-0.20181	0.005865	0.034843
MPV17	0.262993	0.005881	0.034931
FUT11	0.199201	0.005883	0.034931
CYP20A1	0.246809	0.005885	0.034931
SIRT5	-0.22356	0.005898	0.035005
RBM26	0.352139	0.005904	0.035027
GLG1	0.180713	0.00591	0.035055
TXN	0.242969	0.005922	0.035118

MAP3K11	0.144939	0.00593	0.035154
NDUFA13	0.334648	0.005932	0.035157
UPF1	0.200941	0.005935	0.035164
RABGGTA	0.279597	0.005947	0.035223
SH2B3	0.377156	0.005966	0.035327
BPGM	0.510413	0.005977	0.035385
HOXA6	0.312826	0.00599	0.035449
HHAT	-0.23766	0.00602	0.035621
TRRAP	0.212233	0.00603	0.03567
ZC3H12C	-0.21047	0.006036	0.035692
SLC29A3	0.294069	0.006041	0.035712
PNRC2	0.175336	0.006046	0.035724
HAUS7	0.267777	0.006046	0.035724
CISH	0.296151	0.006048	0.035724
CCDC82	-0.12344	0.006053	0.035743
CEP19	0.176423	0.006061	0.035784
CENPBD1F	-0.25964	0.006065	0.035794
DHX15	-0.16386	0.006066	0.035794
BCL10	-0.51493	0.006072	0.035816
C5orf66	-0.14041	0.006078	0.035844
DDX56	0.206395	0.006107	0.036008
KCNQ1	0.15696	0.00612	0.03607
SS18L2	-0.16394	0.006129	0.036109
PFKFB4	0.18146	0.006131	0.036109
ITPKB-IT1	-0.08914	0.006131	0.036109
CHD9	-0.36031	0.006136	0.03613
TNFRSF11/	0.2924	0.00615	0.036197
RPA3OS	-0.31057	0.006159	0.036244
ERMAP	0.374816	0.006178	0.036344
LOC64707(	-0.13672	0.006182	0.03636
UBE2Z	0.273696	0.006187	0.036378
AX746968	-0.09022	0.00619	0.036385
AAMP	0.306293	0.006196	0.036407
BMS1	-0.25691	0.006197	0.036407
ALDH3A2	0.322375	0.006199	0.036407
LRBA	-0.27235	0.006208	0.036452
IFRD2	0.267229	0.00622	0.036513
NARS	-0.15067	0.006222	0.036515
DMXL1	-0.32321	0.006224	0.036515
MAPK1	0.264413	0.006226	0.036516
LOC10192(	-0.27251	0.006235	0.03656
TRAK2	0.363986	0.00625	0.03664
TMEM14C	0.296071	0.006274	0.036761
LOC10192{	-0.05689	0.006274	0.036761
SYCE1L	-0.11095	0.006284	0.036797
LOC40004:	0.198895	0.006284	0.036797
TRAPPC13	-0.43298	0.00629	0.036821
IGLL1	0.793941	0.006294	0.036831
ADAM28	-0.54865	0.006295	0.036831
LOC10192	0.061328	0.006302	0.036865
HLA-DPB1	0.269424	0.006305	0.036872
SYNGAP1	0.081927	0.006308	0.036877
SRCRB4D	-0.19253	0.006316	0.036917
FAM155A	0.007618	0.006318	0.036918
CISD3	0.171172	0.006343	0.037044
CPXM1	-0.39004	0.006343	0.037044
KPNB1	0.176109	0.006345	0.037044
NEDD1	-0.25967	0.006348	0.037051

ABHD8	0.107821	0.006349	0.037051
PHACTR2	0.338722	0.006354	0.037066
LOC10272	0.121985	0.006362	0.037105
OARD1	-0.19658	0.006384	0.03722
GGH	-0.32516	0.006412	0.037374
RBM15B	0.201067	0.006419	0.037407
MLLT1	0.099846	0.006421	0.037407
PIGK	0.271001	0.006425	0.03742
ADCK4	-0.10451	0.006432	0.037451
C1orf115	0.169353	0.006478	0.037712
FAM26F	0.338584	0.006481	0.037715
SLC25A23	0.136502	0.006485	0.037722
LOC10192	-0.0976	0.006485	0.037722
AURKC	0.167741	0.006495	0.037768
EPS15	-0.17417	0.006515	0.037877
HTRA1	0.425907	0.006525	0.037922
SLC25A13	0.314763	0.006531	0.037947
DCAF5	0.210499	0.006544	0.03801
HIPK1	-0.30496	0.006551	0.038042
SIN3A	-0.22722	0.006554	0.038052
VAMP1	-0.30933	0.006577	0.038172
OXSR1	0.330989	0.00658	0.038176
GNE	-0.18911	0.006581	0.038176
DIRC2	0.207003	0.006584	0.038176
ZNRF2	0.158922	0.006585	0.038176
C1orf35	0.219811	0.006604	0.038278
SCAND1	0.257732	0.006607	0.03828
ZNF738	-0.26398	0.006608	0.03828
MFF	0.153742	0.006617	0.038322
CSGALNA(	0.719649	0.006624	0.038349
SPIRE2	-0.01837	0.006625	0.038349
SPIN3	0.193701	0.006627	0.038349
COG6	-0.11689	0.006633	0.038375
CX3CR1	0.399835	0.006638	0.038394
TADA1	-0.14892	0.006644	0.038418
GORAB	-0.33713	0.006667	0.038542
LOC28643	-0.45487	0.006676	0.038585
CCDC102A	0.273302	0.006684	0.038621
CSRNP2	0.143268	0.006699	0.038666
HOXA1	0.578047	0.006699	0.038666
MAN1B1-/	-0.21033	0.006699	0.038666
COQ2	0.189893	0.006699	0.038666
GTF2IRD2E	0.061841	0.00671	0.038714
TGFBR3	0.760431	0.006718	0.03875
RNASEH2A	0.386394	0.006732	0.038825
NAT16	-0.03717	0.006792	0.039158
ZNF212	0.182604	0.006794	0.03916
NUP153	-0.24595	0.0068	0.039182
TNFAIP8L2	0.442119	0.006809	0.039228
PRUNE	0.244943	0.006818	0.039255
MR1	0.238378	0.006819	0.039255
IL4R	0.320299	0.00682	0.039255
ZFP90	-0.24162	0.006832	0.039319
PET117	-0.17492	0.006846	0.039389
SPATS2L	0.159513	0.00685	0.0394
CHMP2B	0.178779	0.006854	0.039409
TMEM161E	-0.23722	0.006866	0.039467
TEPP	0.060319	0.006868	0.039472

PSMD1	0.179902	0.006873	0.039488
C20orf197	-0.6095	0.006877	0.0395
RALGPS2	-0.3217	0.006896	0.039603
AP006547.	-0.07083	0.006905	0.03964
PPIH	-0.27992	0.006908	0.039645
S1PR4	-0.23171	0.006916	0.039681
PGAM1	-0.20441	0.006922	0.039706
SH3PXD2B	0.040571	0.006926	0.039719
TCTEX1D1	-0.6952	0.006928	0.039719
GATAD2B	0.241442	0.006944	0.039793
TRIP12	0.249674	0.006944	0.039793
DOCK11	-0.24995	0.006948	0.039803
TDP1	-0.25361	0.006954	0.039826
MED15	0.258342	0.006956	0.039826
BMF	0.318007	0.006961	0.039849
TMEM192	0.317571	0.006974	0.03991
BC070490	0.007446	0.006976	0.03991
HOXB-AS3	0.302349	0.00698	0.039926
PSMG2	0.167652	0.006986	0.039948
GNAQ	-0.26069	0.006995	0.039989
SLC52A1	0.112738	0.007003	0.040022
GLB1L2	0.230779	0.007006	0.040032
SNAP29	0.271057	0.007012	0.040052
UBE2F	0.283478	0.007021	0.040097
MRPL28	0.288668	0.007028	0.040125
LOC10192	-0.0575	0.007034	0.040134
MYO7B	-0.04621	0.007035	0.040134
GIMAP7	0.71656	0.007037	0.040134
CAPRIN2	0.259123	0.00704	0.040134
TSC22D2	0.142101	0.00704	0.040134
WNT5A	0.12272	0.007041	0.040134
ZBTB44	0.331673	0.007045	0.040149
CDV3	0.256571	0.007061	0.04023
TXNIP	0.46123	0.007066	0.040245
GORASP1	0.120284	0.007073	0.040269
SOX15	-0.13878	0.007074	0.040269
RP11-97C1	-0.34551	0.007079	0.040285
UBE2E2	0.468903	0.007114	0.040475
MCRS1	0.324575	0.007118	0.040486
SOCS3	0.184355	0.00712	0.040488
ZNF142	0.197583	0.007127	0.040521
OPHN1	0.273045	0.007158	0.040683
ALG8	-0.2524	0.007161	0.040691
BAZ1B	0.196678	0.007163	0.040692
RABL3	0.210201	0.00718	0.040775
AKIRIN1	0.280002	0.007182	0.040777
ZNF763	0.201122	0.007189	0.040807
ALPK1	-0.26536	0.007194	0.040824
UFSP1	0.079255	0.00721	0.040907
MED8	0.264664	0.007255	0.04115
CRIP1	0.712649	0.007268	0.04121
LOC10272	0.014232	0.007276	0.041248
RNF2	-0.07793	0.00728	0.041259
RNU2-22P	-0.33438	0.007282	0.041259
CBX2	-0.161	0.007304	0.041373
PDP1	0.280047	0.007325	0.04147
ANKRD29	0.280747	0.007326	0.04147
COL26A1	-0.11222	0.007327	0.04147

NID1	0.152093	0.007331	0.041485
NIFK-AS1	-0.21844	0.007354	0.041603
GSG1L	-0.06234	0.007367	0.041662
TTBK1	-0.0595	0.007369	0.041662
SSTR5	-0.0932	0.007376	0.041693
NEK3	0.202	0.007378	0.041693
GIN1	-0.287	0.007388	0.041739
RP11-158(	-0.5856	0.007392	0.041743
TMEM80	-0.20404	0.007392	0.041743
FUT10	-0.10974	0.007394	0.041743
C1QTNF3	-0.1695	0.007413	0.041836
TRMT10B	-0.16006	0.007425	0.041892
ZNF663P	-0.06028	0.007429	0.041908
GNG2	0.138295	0.007448	0.042002
DPY19L2	-0.47836	0.007468	0.042106
PJA2	0.234405	0.007479	0.042152
TTF2	-0.19473	0.007491	0.042212
RUSC1	0.245976	0.007493	0.042212
C5orf28	-0.36398	0.007496	0.042217
BID	0.255334	0.007498	0.042217
SIX3-AS1	-0.04064	0.007509	0.042266
MTUS1	0.087487	0.007511	0.042266
RP11-559M	-0.14318	0.007517	0.042294
UBALD2	0.212539	0.007521	0.042306
SIGIRR	0.305797	0.007537	0.042384
RP11-348F	-0.27317	0.007541	0.042387
NCKAP1L	0.244632	0.007542	0.042387
SAMD13	-0.26108	0.007562	0.042486
LZTR1	0.168611	0.007564	0.042486
SUN1	0.156045	0.007565	0.042486
AK021977	-0.54982	0.00757	0.042499
DDX19B	-0.32283	0.007574	0.042511
USP3-AS1	0.186739	0.007577	0.042521
DPEP2	0.407286	0.00758	0.042524
ACTR6	-0.11646	0.007585	0.042542
AFTPH	-0.15303	0.007598	0.042606
ZKSCAN3	-0.12359	0.007604	0.042617
AIFM2	0.135739	0.007606	0.042617
NKIRAS2	0.201193	0.007606	0.042617
LOC10272:	-0.10819	0.007611	0.042628
TRIM45	-0.29178	0.007613	0.042628
ADAM9	0.348455	0.007614	0.042628
NOP58	-0.1736	0.00762	0.042644
GEMIN7	0.323782	0.007621	0.042644
UTP15	-0.13446	0.007626	0.042661
FLT3LG	0.100028	0.007631	0.042679
CA1	0.569049	0.007654	0.042795
CBX6	0.172998	0.00766	0.042818
BTBD3	-0.31189	0.007666	0.042841
SHC3	0.042594	0.007692	0.042975
BTN3A1	0.205876	0.007698	0.042999
PPP5D1	0.201427	0.007703	0.043009
LRRC28	0.143395	0.007704	0.043009
ATAD2B	0.279784	0.007708	0.043017
MMP8	0.772048	0.007717	0.04306
FRK	0.073404	0.007724	0.043083
TAZ	0.225207	0.007725	0.043083
CYB5R4	0.237309	0.007732	0.043109

C3orf17	-0.10033	0.007737	0.043116
AMZ1	-0.06175	0.007737	0.043116
CLGN	-0.56273	0.007754	0.043199
GPR156	-0.04116	0.007778	0.043323
METTL17	-0.24661	0.007788	0.043355
RRP36	0.203423	0.007788	0.043355
GMDS	-0.23748	0.007796	0.043388
TERF2IP	0.183338	0.007804	0.043423
CLK3	0.216333	0.00781	0.043446
KLC1	0.247907	0.007816	0.043467
LOC10013:	0.288032	0.007824	0.043494
CCDC134	0.151494	0.007825	0.043494
AHI1	-0.13777	0.007835	0.043537
NABP1	0.512434	0.007862	0.043677
CYB561D2	0.155585	0.007873	0.043722
TNRC6C-A	0.289253	0.007874	0.043722
CEP162	-0.22008	0.007879	0.043738
ITGB3BP	-0.25041	0.007894	0.043811
TEX10	-0.16167	0.007903	0.043849
NOTCH2N	-0.34144	0.007909	0.043869
GLT1D1	0.381381	0.007913	0.043879
ZNF518A	-0.35246	0.007922	0.04392
TKTL1	0.103689	0.007929	0.043948
ANXA3	0.673278	0.007949	0.044045
CD33	0.489523	0.007951	0.044045
HDAC7	0.166364	0.007953	0.044046
FLOT2	0.20587	0.007963	0.044092
RRAGA	0.181568	0.007966	0.044096
SARM1	0.273905	0.007977	0.044145
CYP3A5	-0.03732	0.007982	0.044146
MTFR1L	0.209328	0.007982	0.044146
UQCRFS1	0.194205	0.007983	0.044146
NPW	0.483397	0.007998	0.044219
SDAD1	0.315628	0.008015	0.044299
H2BFS	0.67779	0.008041	0.044431
HSD17B4	0.110851	0.008062	0.044534
INSIG1	0.380313	0.008067	0.044537
SNUPN	0.18967	0.008067	0.044537
LDLR	0.091995	0.008069	0.044537
VEZF1	-0.18392	0.00807	0.044537
SLC27A2	-0.43934	0.008079	0.04457
EIF5A2	0.069914	0.00808	0.04457
PWRN2	-0.07029	0.008092	0.044621
TTLL11	0.163341	0.008099	0.044651
SLC23A2	0.195865	0.008122	0.044763
DNAJC19	0.194711	0.008141	0.044857
SIGLEC15	0.190217	0.008143	0.044857
ZKSCAN8	-0.13533	0.008163	0.044956
AK056098	0.018489	0.008182	0.045052
COL4A1	0.33842	0.008191	0.045085
ANAPC4	-0.15756	0.008196	0.045102
PELI3	0.198551	0.008204	0.045136
GGCT	-0.20813	0.008211	0.045165
RAD52	0.150378	0.008215	0.045172
RASSF1	-0.27042	0.008222	0.045191
HILPDA	0.362674	0.008222	0.045191
RP11-435(	-0.08271	0.008235	0.045242
ARL16	0.284407	0.008236	0.045242

ADSS	-0.15618	0.008241	0.045258
POP5	0.258327	0.008247	0.045279
DDHD2	-0.21856	0.008253	0.045294
CD99	0.416538	0.008254	0.045294
COL4A2	0.294695	0.008261	0.045322
MPND	0.33491	0.008263	0.045322
ZNF12	-0.29563	0.00831	0.04557
LTV1	-0.2058	0.008329	0.045662
BC022892	0.347867	0.008333	0.045673
PDK2	0.143917	0.008358	0.045798
CECR1	0.662225	0.008369	0.045846
NOL10	0.153073	0.008377	0.04588
CCT5	0.246962	0.008382	0.045896
ARF1	-0.40303	0.008391	0.045933
NTPCR	0.217855	0.008395	0.045941
NBEAL2	0.152768	0.008403	0.045977
LOC101928	-0.05625	0.008418	0.046041
JMJD1C	-0.24057	0.008419	0.046041
MRPL38	0.171614	0.008433	0.046106
FAM83G	-0.15724	0.008489	0.046397
CCAR1	-0.19896	0.008501	0.046449
SAMD14	-0.03622	0.008503	0.046449
LOC101927	-0.3943	0.00851	0.04648
ATP6V1G1	0.178802	0.008518	0.046509
BEST1	0.068569	0.00853	0.046563
CCDC185	-0.05803	0.008538	0.046593
CCNDBP1	0.253573	0.008546	0.046628
RASIP1	0.095056	0.008553	0.046654
PQBP1	0.337752	0.00856	0.046683
FAXDC2	0.481773	0.008566	0.046701
MEA1	0.254871	0.008575	0.04674
TIMM23B	-0.29658	0.008581	0.046761
ANKRD10-	-0.30755	0.008591	0.046803
OCEL1	0.242468	0.008609	0.04689
TARDBP	-0.19481	0.008639	0.047033
ACTR10	0.156242	0.00864	0.047033
CBX3P2	-0.15494	0.008644	0.047042
OLA1	-0.16236	0.00865	0.047057
RNF170	0.266374	0.008653	0.047057
LOC100507	0.076102	0.008654	0.047057
TRMT44	0.103847	0.008655	0.047057
MALL	0.251605	0.008659	0.047068
HSPD1	-0.61788	0.00868	0.047156
LOC403323	-0.25243	0.00868	0.047156
CHUK	-0.28193	0.008707	0.047285
EXD3	0.05955	0.008708	0.047285
NFATC2	0.146702	0.008725	0.047355
EEF1D	-0.2579	0.008725	0.047355
EPX	0.503893	0.008729	0.047357
TSPY26P	-0.14653	0.00873	0.047357
HBB	0.720716	0.008746	0.047431
UTF1	0.026899	0.008766	0.047526
LOC100500	-0.21253	0.008795	0.047668
KRAS	-0.40829	0.008796	0.047668
CHST15	0.519323	0.008801	0.047684
JAK2	0.237008	0.008808	0.047709
TRPV6	-0.04996	0.008818	0.047734
ERV3-1	-0.30783	0.008819	0.047734

ZNF444	0.124893	0.008819	0.047734
RAB11A	-0.29083	0.008841	0.047837
TMEM169	0.025383	0.008847	0.047856
B3GNT6	-0.05334	0.00885	0.047856
PIEZO1	-0.12916	0.008851	0.047856
COX2	0.265561	0.008856	0.047867
DCTN6	0.190819	0.008857	0.047867
RFC3	0.432958	0.008862	0.047873
AP3M1	-0.17247	0.008863	0.047873
PAPD4	-0.36037	0.008865	0.047873
ALDH7A1	0.360038	0.008874	0.047911
RNF135	0.214331	0.008888	0.047973
EPHX1	0.203612	0.00891	0.048078
SNX8	-0.06883	0.008915	0.048097
APAF1	0.079865	0.008928	0.048154
KLC3	0.111666	0.008936	0.048181
C10orf54	0.307863	0.008938	0.048181
RARA-AS1	0.264266	0.00894	0.048181
PLEKHF2	0.250938	0.008954	0.048245
AK021537	-0.09345	0.008978	0.048361
RRP7A	0.443588	0.008985	0.048386
EU250746	-0.16229	0.008989	0.048401
WDR75	0.192137	0.008995	0.048418
NUDT17	0.117211	0.009045	0.048677
ALS2	-0.37908	0.009051	0.048696
ZNF175	-0.2002	0.009057	0.048715
PRDM1	0.251415	0.009062	0.048731
STMN4	-0.03288	0.009067	0.048744
KIAA0226L	0.799025	0.009086	0.048837
ADAMTS1(	-0.13047	0.0091	0.048899
CNPY2	0.273132	0.009106	0.048918
AC092620.	-0.36942	0.009117	0.048965
CLIC4	0.270389	0.009121	0.048975
LOC10013:	-0.09552	0.009136	0.049042
UBA1	0.161796	0.00915	0.049107
KCNQ1-AS	-0.08734	0.009176	0.049234
WTAP	0.248399	0.009188	0.049288
LINC00893	-0.32883	0.009192	0.049293
BIN2	0.287023	0.009201	0.049332
LOC10106(	0.048553	0.009211	0.049372
C1orf53	0.239197	0.009219	0.049401
TBC1D24	0.310253	0.009221	0.049401
TSC2	0.184993	0.009241	0.049497
DPM3	-0.24327	0.009251	0.049534
C1orf112	0.378268	0.009254	0.049534
MCL1	0.441611	0.009255	0.049534
GHDC	0.314432	0.009266	0.049572
STXBP5	-0.38155	0.009267	0.049572
ELP3	-0.20069	0.009274	0.049597
PTGFRN	0.130788	0.009303	0.049743
ZNF165	-0.46378	0.009326	0.049854
AFG3L1P	-0.21048	0.009344	0.049925
C6orf48	-0.2338	0.009344	0.049925
RAB2A	-0.15139	0.009355	0.049971
LOC44108:	0.233878	0.009362	0.049997
HACE1	-0.30616	0.009373	0.050044
RFC1	-0.17691	0.00938	0.050065
YWHAZ	0.219927	0.009397	0.050148

CASC4	-0.40639	0.00941	0.050203
PLEKHA1	0.291271	0.009415	0.050215
DQ570096	-0.19993	0.009433	0.0503
AK074476	-0.0582	0.009444	0.050337
CDC42EP3	0.424556	0.009446	0.050337
DHX32	-0.17628	0.009447	0.050337
FCHO1	0.143671	0.009451	0.050345
FARP1	0.151351	0.009457	0.050366
BAG4	0.328101	0.009469	0.05042
CDA	0.272275	0.009485	0.050476
XPOT	-0.14645	0.009487	0.050476
SUPV3L1	0.229286	0.009487	0.050476
ALDH1A3	0.238824	0.009493	0.050495
SNAI2	0.291527	0.009516	0.050604
OAZ3	0.116037	0.009527	0.050653
ALG12	0.157066	0.00953	0.050656
C9orf117	0.05618	0.009563	0.05082
RAB3IL1	-0.20165	0.009567	0.050821
LOC10192{	-0.30357	0.009568	0.050821
STX8	0.093718	0.009576	0.050849
MBNL3	-0.2569	0.009608	0.05101
LRRC40	-0.20698	0.009626	0.05109
KRT1	0.383861	0.009649	0.0512
ZNF229	-0.12409	0.009665	0.051271
ERLIN2	0.280124	0.009675	0.051312
ROCK1	-0.13395	0.009686	0.051359
PIGV	-0.24064	0.009699	0.051414
SLPI	0.351091	0.009707	0.051447
MLX	0.188709	0.009711	0.051452
PP12708	-0.40717	0.009714	0.051458
PGK1	0.187007	0.009723	0.051491
SLC27A1	0.175525	0.00975	0.051622
RRAD	0.240008	0.009753	0.051625
ZNF395	0.196275	0.009758	0.05164
HOXB6	0.267475	0.009773	0.051708
C14orf159	-0.13368	0.009779	0.051727
C16orf58	0.315538	0.009785	0.051742
MOGS	0.31723	0.009799	0.051804
SASS6	-0.19539	0.009809	0.051846
SIVA1	0.293077	0.009812	0.051849
FGFBP2	0.231804	0.009819	0.051874
NDUFS3	0.218012	0.009823	0.051882
ATL1	-0.40885	0.00984	0.051961
CSAD	0.153997	0.00985	0.051999
MPHOSPH	-0.23189	0.009856	0.052019
PGAP1	-0.40558	0.009883	0.052132
CLASP2	-0.22882	0.009884	0.052132
PDZRN3	0.20254	0.009885	0.052132
SLC16A1-/	0.074799	0.009887	0.052134
KIAA0907	-0.17714	0.009896	0.052168
FKBP8	0.112407	0.009903	0.05219
INPP5K	0.236906	0.009923	0.052282
LAD1	-0.02878	0.009925	0.052282
RET	-0.04028	0.009931	0.052297
PQLC3	0.124683	0.00995	0.052388
ZNF137P	-0.3461	0.009968	0.052467
PDLIM5	0.159516	0.009975	0.052481
DNASE1L3	0.510101	0.009976	0.052481

LOC10050°	-0.11951	0.009978	0.052481
RPRD1A	-0.17734	0.009989	0.052525
LOC10013:	0.167461	0.009991	0.052525
APOBEC3C	0.224164	0.010002	0.05257
SEPW1	0.261774	0.010033	0.052718
WDR13	-0.13394	0.010035	0.052718
FAM214B	0.256819	0.010057	0.052808
BTBD7	0.292155	0.010059	0.052808
PPP1R12B	0.143169	0.01006	0.052808
WBP11	0.314483	0.010062	0.052808
PHF21A	0.150351	0.010069	0.052832
RP3-406P2	0.226459	0.010072	0.052837
FBXL17	-0.3209	0.010086	0.052895
FLVCR2	0.173505	0.010096	0.052926
SEC31A	0.148661	0.010096	0.052926
MICAL2	0.322553	0.010104	0.052954
MLEC	0.2396	0.010112	0.052971
COX6B1	0.242873	0.010112	0.052971
SRGN	0.425941	0.010131	0.053057
FBXO22	0.254918	0.010139	0.053084
TAPBPL	0.302426	0.010158	0.053173
ELFN1-AS:	-0.10957	0.010171	0.053229
C17orf97	0.198247	0.010189	0.053307
TEFM	-0.22147	0.01022	0.053457
NUP107	-0.23391	0.010225	0.053469
FGFRL1	0.057557	0.010227	0.05347
PALB2	-0.25987	0.010236	0.053502
ZNF397	0.188528	0.010259	0.053612
RP11-403F	-0.23121	0.010263	0.053617
PKIA	-0.47656	0.010273	0.053656
DSCR10	0.021247	0.010282	0.053691
ID3	0.569196	0.010294	0.053743
GRAMD1B	-0.29149	0.010297	0.053744
STMN1	-0.20178	0.010329	0.053895
HPCAL4	-0.04723	0.010335	0.053916
TTC9B	-0.05637	0.010354	0.053993
SENP6	-0.26076	0.010355	0.053993
CYP2B6	0.044017	0.010371	0.054064
PRIM1	-0.29393	0.010407	0.054237
ERMP1	-0.28665	0.01041	0.05424
TMEM53	0.174184	0.01042	0.054283
15-Sep	-0.18525	0.010432	0.05433
TCTA	0.224119	0.010443	0.054373
SLC6A17	-0.04134	0.010454	0.054418
VMP1	-0.75483	0.010481	0.054546
CTDSP2	0.177312	0.010486	0.054558
CHD7	0.172279	0.010488	0.054558
PTPMT1	0.18832	0.01051	0.054659
CBX4	0.23103	0.010513	0.054661
ADCY6	0.227067	0.010519	0.054678
RP11-846E	-0.35936	0.010525	0.054691
NUDT5	-0.15587	0.010527	0.054691
UBLCP1	0.28047	0.010534	0.054718
GPBAR1	0.21705	0.01054	0.054724
MGME1	0.206974	0.010543	0.054724
CRYGS	0.162813	0.010544	0.054724
SREBF1	0.155198	0.010546	0.054724
ARNTL	0.210525	0.010575	0.054862

ST5	0.189656	0.010578	0.054866
KCTD13	0.135372	0.010587	0.0549
PDPK1	0.033949	0.010605	0.054982
CD46	0.195362	0.010618	0.055035
ZNF764	-0.19909	0.010644	0.055155
C17orf67	-0.15824	0.010662	0.055234
TNP1	-0.0778	0.010668	0.055252
MDN1	-0.40976	0.010684	0.055324
ZNF667-A:	-0.3883	0.010698	0.055381
RPL32P3	-0.22506	0.010715	0.055456
KANK3	0.216644	0.010733	0.055536
CD109	-0.41172	0.010736	0.055539
MGAM	0.580923	0.010742	0.055558
ATP8B1	-0.30687	0.010752	0.05559
UXT	0.214895	0.010754	0.05559
TBC1D2B	-0.37243	0.010759	0.055606
ZNF134	-0.26037	0.010774	0.055656
LOC10192	-0.29172	0.010774	0.055656
BCL2L1	0.157024	0.010808	0.055812
ENOPH1	-0.16833	0.010809	0.055812
FOXO4	-0.16599	0.010831	0.055907
SRRM2	0.216219	0.010833	0.055907
SLC35A3	-0.24596	0.010837	0.055917
BAIAP2L2	-0.06101	0.010875	0.056091
MRFAP1	-0.12261	0.010876	0.056091
11-Sep	-0.37205	0.010887	0.056135
APOL3	0.331853	0.010899	0.056181
UBE2B	0.278696	0.010914	0.056244
MED13	0.201035	0.01092	0.056264
ZNF423	0.320946	0.010929	0.056297
EFHC1	-0.36793	0.010961	0.056448
LINC01410	-0.48086	0.010968	0.056456
FADS2	0.102999	0.010972	0.056456
TLE2	0.101388	0.010972	0.056456
CYP2W1	-0.07528	0.010973	0.056456
SOCS7	-0.19379	0.01101	0.056634
CRABP2	-0.0641	0.01102	0.056669
ZNF770	-0.15875	0.011035	0.056736
ANAPC7	-0.13689	0.011041	0.056754
MTSS1	0.144242	0.011045	0.05676
TRNAU1AI	-0.18293	0.011078	0.056915
HSPA4	0.303017	0.0111	0.057012
EFTUD1	-0.21682	0.011103	0.057018
OCR1	0.007446	0.01111	0.057023
RPARP-AS	0.20928	0.011112	0.057023
CEP44	-0.2126	0.011112	0.057023
ZNF493	-0.25204	0.011115	0.057023
NRSN2	-0.03567	0.011119	0.057033
TMEM44-/	-0.28036	0.01113	0.057074
TP53I3	0.486348	0.011138	0.0571
LSM14A	-0.15961	0.011163	0.057213
EFHC2	-0.25606	0.011179	0.057285
BC041363	-0.75202	0.011197	0.057364
GPBP1	-0.12915	0.011218	0.057456
FIG4	0.37494	0.011248	0.057597
COX7C	0.264179	0.011251	0.057597
KLHL12	0.193535	0.011261	0.057635
PAXIP1-AS	-0.23572	0.011271	0.057675

NXNL1	-0.0448	0.011283	0.057722
LILRA1	0.213931	0.011287	0.057729
LAG3	0.37838	0.011301	0.057786
STAM	-0.2437	0.011321	0.057861
TMX3	-0.27497	0.011321	0.057861
CCNI	0.193352	0.011337	0.057927
SFT2D3	-0.13482	0.011345	0.057956
PNPLA2	-0.08842	0.011366	0.058024
ARID1B	-0.18754	0.011366	0.058024
TMA16	-0.41538	0.011368	0.058024
SMNDC1	-0.21217	0.011369	0.058024
GPR125	-0.30131	0.011376	0.058048
TBC1D9B	0.129724	0.01138	0.058051
PKNOX1	0.161142	0.0114	0.058142
PRPF8	0.218317	0.011403	0.058142
MCMDC2	0.020279	0.011428	0.058256
CCNT2	-0.19064	0.011436	0.058286
SMCO4	0.403989	0.011457	0.058356
ATF1	-0.24178	0.01146	0.058356
SPERT	-0.02219	0.011461	0.058356
TM9SF1	0.149812	0.011461	0.058356
SEC22C	0.184705	0.011464	0.058359
WDR3	-0.15938	0.011484	0.058435
PDCD5	0.277456	0.011487	0.058435
N4BP1	0.120977	0.011488	0.058435
MAGEE1	-0.24055	0.01149	0.058435
KIAA1652	-0.13804	0.011504	0.058491
PPP2R3C	-0.38421	0.011513	0.058526
DENND5B	0.309761	0.011526	0.058575
BEND6	-0.31375	0.011551	0.058688
APOOL	0.202959	0.011554	0.058692
OPA1	-0.1715	0.011573	0.05876
RP11-498(	-0.18329	0.011574	0.05876
SOCS4	-0.15754	0.011576	0.05876
GBP3	0.44741	0.011587	0.058795
ATP11C	-0.08785	0.011588	0.058795
ANAPC10	-0.19976	0.011605	0.058869
SMC2	-0.27797	0.011624	0.058948
INTS5	0.201014	0.011642	0.05903
RSL24D1	-0.20047	0.011647	0.059041
MT1X	0.785199	0.01168	0.059194
ADCY4	0.238367	0.011686	0.059207
SMOX	-0.11845	0.011695	0.059239
CBFA2T2	0.103612	0.011712	0.059315
CD3EAP	0.30798	0.011718	0.059328
ZNF223	-0.18884	0.011742	0.059436
HCFC1	0.08425	0.011744	0.059436
MTERF3	-0.22745	0.011771	0.059556
MEI1	0.22557	0.011805	0.059714
GLT8D2	0.092228	0.011847	0.059912
FUCA2	0.329617	0.011863	0.05998
METRN	0.106648	0.011868	0.059982
CTBP2	0.185848	0.011869	0.059982
SPATA7	0.319609	0.011904	0.060146
CTA-445C	-0.33131	0.011927	0.060247
SEC24A	0.409396	0.011943	0.060316
RPL41	-0.03397	0.011975	0.06046
ASCC1	-0.17226	0.011996	0.060551

COX10	-0.18891	0.012009	0.060606
SNRPC	0.227723	0.012065	0.060873
TBC1D10B	0.282321	0.012069	0.060879
TRAF5	-0.16746	0.012089	0.060965
MX2	0.515915	0.012104	0.061029
SDCBP	0.266673	0.012124	0.061112
ZNF516	0.419239	0.01213	0.061131
NEU4	-0.18483	0.012152	0.061225
KLF16	0.137064	0.012159	0.061247
NOM1	-0.2351	0.012164	0.061247
MATR3	-0.42922	0.012165	0.061247
MED25	0.072633	0.012185	0.061338
ITGAX	0.168026	0.012208	0.061436
ZNF114	0.272843	0.012241	0.061589
CHORDC1	-0.13972	0.012251	0.061627
IVD	0.230129	0.012267	0.061692
MTERF1	-0.23867	0.012289	0.061786
LINC00467	0.260064	0.012294	0.061795
HDAC5	0.15037	0.012298	0.061795
MUS81	0.173785	0.0123	0.061795
SAMD4B	0.115159	0.012302	0.061795
RAPGEFL1	0.022601	0.012324	0.06189
SLC33A1	0.229619	0.012334	0.061926
ARVCF	-0.09187	0.012386	0.062172
HSP90AB1	0.303329	0.012393	0.062196
KLF10	-0.46097	0.012402	0.062226
CCDC43	0.174374	0.012422	0.06231
L3MBTL1	0.035152	0.012428	0.062325
C2orf69	-0.25367	0.012453	0.062436
CREB3L4	-0.30574	0.012455	0.062436
SLC41A2	0.197317	0.012466	0.062476
ZNF140	0.379772	0.012483	0.062543
SPATA32	-0.08772	0.012493	0.062573
ZNF579	0.192074	0.012494	0.062573
MGC50722	-0.06323	0.012511	0.06264
SETDB2	-0.20387	0.012521	0.06267
SIAH2	0.286083	0.012522	0.06267
ZIK1	-0.26616	0.012534	0.062716
ABCA11P	-0.27615	0.012539	0.062726
LINC00595	-0.08547	0.012575	0.062889
IL22RA2	-0.03381	0.012584	0.062919
SNHG10	-0.11097	0.012609	0.063032
GRAMD1C	-0.4855	0.012615	0.063047
COMMD8	-0.29035	0.01263	0.063109
ST6GALNA	0.182748	0.012645	0.063164
TRIM27	-0.2346	0.012647	0.063164
TAOK2	0.09571	0.012652	0.06317
ULK2	0.229676	0.012675	0.063273
PUM2	-0.16436	0.012679	0.063279
ILF3-AS1	0.492485	0.012688	0.063309
LOC44014	0.167084	0.012697	0.063339
CUL4A	-0.17874	0.012708	0.063378
LOC15117	-0.23713	0.012713	0.063392
NAA30	0.096161	0.012718	0.0634
LGALS8	0.232692	0.012725	0.063421
ZC3H15	0.231366	0.012747	0.063513
LPAR6	0.342717	0.012758	0.063558
IPO13	0.213892	0.012762	0.063562

DHDH	0.091719	0.012817	0.0638
RPUSD3	0.41089	0.012817	0.0638
MTA1	0.172215	0.012819	0.0638
KDM6A	-0.33034	0.01283	0.063843
FLYWCH2	0.239734	0.012846	0.063905
THTPA	0.29705	0.012853	0.063927
SCML2	-0.139	0.012856	0.063928
TOMM5	0.230527	0.012861	0.063934
ACSF2	0.25031	0.012866	0.063947
SMYD3	-0.27474	0.01287	0.06395
SBF2-AS1	0.111471	0.012904	0.064103
RER1	0.210753	0.012906	0.064103
RAD51D	0.160432	0.012909	0.064103
KCNJ8	0.345398	0.012938	0.064231
NCOR1	0.190023	0.012943	0.064241
C2orf76	0.218451	0.012946	0.064241
GTF2H1	0.202571	0.012953	0.064261
RUNX3	0.438118	0.012973	0.064335
ZNF600	-0.23058	0.012974	0.064335
FKBP9	0.498614	0.012998	0.06444
SNRPG	-0.18231	0.013003	0.064442
EPHX3	0.082622	0.013004	0.064442
EIF1AX	-0.22878	0.01301	0.064456
SP140L	-0.19353	0.013018	0.064479
NEIL2	0.164641	0.013021	0.064479
SLC2A6	0.307313	0.013043	0.064564
SAV1	0.252931	0.013044	0.064564
ZNF830	-0.183	0.013058	0.064619
RAB11B-A:	0.121044	0.013079	0.064704
PID1	0.083324	0.013081	0.064704
PARPBP	-0.29341	0.013093	0.064748
PPP2R5E	0.168077	0.013104	0.064787
FBN1	0.406299	0.013128	0.064893
TMEM248	0.184544	0.013141	0.064936
EDEM3	0.362255	0.013143	0.064936
CRAMP1L	-0.10669	0.01315	0.064956
RPA2	-0.2308	0.013164	0.065012
HFE	0.068174	0.013169	0.065017
RP11-532F	-0.16528	0.013178	0.065044
TSSC4	0.208166	0.01318	0.065044
IL1R2	0.458012	0.013194	0.0651
MRPL44	0.182228	0.0132	0.065111
DNASE1	-0.22368	0.013224	0.065216
CCDC104	0.256926	0.013243	0.065289
TAF7	-0.23346	0.013245	0.065289
COG3	-0.13561	0.01326	0.065351
ZFP28	0.066488	0.013286	0.06546
MEF2A	0.312407	0.013293	0.065481
PCNX	-0.26945	0.013392	0.065956
C7orf50	-0.19904	0.013405	0.065999
PRKCB	0.24853	0.01341	0.065999
ZZZ3	-0.20511	0.01341	0.065999
LOC10050!	-0.03078	0.013417	0.066006
FABP2	-0.10855	0.013418	0.066006
MAPK1IP1I	0.159022	0.013432	0.066044
SDR39U1	-0.17908	0.013432	0.066044
RNH1	0.154231	0.013436	0.066044
EIF3K	0.224399	0.013438	0.066044

B3GAT3	0.197562	0.013442	0.066051
ARG2	0.459965	0.013448	0.066064
REC114	-0.05659	0.013456	0.066089
NOL8	0.227165	0.013495	0.066266
ESCO2	-0.18608	0.0135	0.066272
CMTM4	-0.36654	0.013502	0.066272
RXRB	0.086214	0.013547	0.06645
CAT	0.369746	0.013549	0.06645
CPPED1	0.341085	0.013552	0.06645
DCTN2	-0.19784	0.013553	0.06645
MAST2	0.114372	0.013554	0.06645
YIPF2	0.101245	0.013559	0.066459
CYP2J2	0.136106	0.013583	0.066565
LRRN4	0.087826	0.013613	0.066697
ZNF250	-0.10007	0.013617	0.066698
CTB-92J24	-0.2432	0.013743	0.067303
CMTM5	0.404903	0.013748	0.06731
RPS9	-0.23395	0.013779	0.067446
NFKB1	0.279945	0.013799	0.067527
LOC44033(	0.037847	0.013812	0.067578
ALKBH4	0.18969	0.013815	0.067578
TRIM9	-0.27733	0.013836	0.067634
UGP2	-0.25127	0.013838	0.067634
ZNF10	-0.24613	0.013839	0.067634
LCP2	0.205426	0.013839	0.067634
SLC14A1	0.410195	0.013843	0.067634
HSPA2	0.422107	0.013845	0.067634
EPB41L1	0.082863	0.013877	0.067774
RBL2	-0.17834	0.01389	0.067824
TPM1	0.332477	0.013898	0.067847
GBP1P1	0.119736	0.013917	0.067923
ACSL1	0.43144	0.013925	0.067948
CCDC141	0.19382	0.013937	0.067987
CEMP1	-0.09479	0.013942	0.067998
SSR3	0.181794	0.013959	0.068068
RP11-109D	0.013691	0.013964	0.068077
SFMBT2	0.228428	0.013968	0.068077
COPS6	-0.27359	0.013984	0.068137
DACT1	0.362466	0.013986	0.068137
LYSMD4	0.172696	0.014001	0.068194
OR52K3P	0.191029	0.014007	0.068208
ESRRA	0.112036	0.014031	0.068308
SIX5	0.07615	0.014039	0.068334
TMCO3	0.16295	0.014097	0.068597
LOC10013:	-0.1126	0.014099	0.068597
SMTN	0.040876	0.014123	0.068695
RPL11	0.258839	0.014134	0.068736
PTP4A2	0.17512	0.014141	0.068741
ZNF835	0.117385	0.014142	0.068741
HSBP1	0.128064	0.014152	0.068776
UTY	0.424609	0.014183	0.068911
C4orf33	0.046296	0.014188	0.068911
KIAA1804	-0.47499	0.014189	0.068911
POMZP3	0.429122	0.014196	0.068928
PAICS	-0.25338	0.014246	0.069152
POGLUT1	-0.17381	0.014268	0.069233
ZNF415	-0.55489	0.014269	0.069233
TRIM58	0.361511	0.014303	0.069353

SLC38A6	0.380216	0.014303	0.069353
PPP1R12A	0.27671	0.014303	0.069353
NAP1L4	0.15159	0.014328	0.069444
RNF103	-0.26276	0.014328	0.069444
CLPB	0.172733	0.014349	0.06953
NCBP1	0.168954	0.014356	0.069539
SIRPB1	0.083859	0.014357	0.069539
LOC10192!	0.113296	0.014362	0.069547
ARL13B	0.226696	0.014376	0.069599
RP11-378A	-0.26653	0.014414	0.069759
HPYR1	-0.03675	0.014416	0.069759
ARHGAP18	0.462683	0.014441	0.069864
MAD1L1	0.359341	0.014457	0.069914
CSE1L	-0.27826	0.014457	0.069914
GCFC2	-0.16859	0.014463	0.069926
ARFIP2	0.236952	0.014477	0.069965
BAALCOS	-0.12801	0.014478	0.069965
TRA2B	0.275434	0.014484	0.069974
NAIP	0.455756	0.014488	0.069974
LOC10192	-0.03683	0.014492	0.069974
COQ6	0.253281	0.014492	0.069974
PALMD	0.072759	0.014505	0.070015
COX19	-0.26457	0.014509	0.070015
DHX37	0.184313	0.014511	0.070015
POU2F2	0.164084	0.014514	0.070016
MUL1	-0.25641	0.014532	0.070089
NAPSB	0.665244	0.014541	0.070117
ZNF528	0.160326	0.01457	0.07024
MFI2-AS1	0.076241	0.014582	0.070283
CD38	0.447325	0.014622	0.070457
TAF4B	0.220154	0.01463	0.070481
IL33	0.636777	0.014668	0.07065
SLC35F2	-0.27011	0.014707	0.070819
NR2F2	0.161016	0.01471	0.070819
LOC10013	0.031719	0.014726	0.07087
CCDC85B	-0.04344	0.014729	0.07087
ACOT7	0.229852	0.014731	0.07087
LOC10192	-0.04483	0.014734	0.07087
CHID1	0.30126	0.014742	0.070894
SPG20OS	-0.20104	0.014749	0.070912
CEP57L1	-0.09505	0.014774	0.071018
SDF2L1	0.386402	0.014827	0.071256
C14orf93	-0.20405	0.014837	0.07129
ATP5F1	0.153323	0.014848	0.071323
CD44	0.468521	0.014888	0.0715
NINJ2	0.345717	0.014893	0.07151
ABHD17B	0.298182	0.014897	0.071511
SLBP	0.215155	0.014901	0.071518
LOC10013	-0.05261	0.014921	0.071597
SLC25A40	-0.17856	0.014934	0.071643
AF070581	0.006966	0.014955	0.07173
TCTEX1D2	-0.31537	0.014982	0.071843
RP11-138A	-0.52561	0.015022	0.072
RBM8A	0.18365	0.015023	0.072
ELFN1	-0.06133	0.015025	0.072
GIMAP1	0.452602	0.015055	0.072129
ABHD16A	0.142088	0.015063	0.072149
RBM43	0.175901	0.015092	0.072272

PPA1	-0.21401	0.015127	0.072423
C12orf80	-0.03655	0.015156	0.072547
PRODH2	-0.05612	0.015178	0.072638
BAD	0.119925	0.015195	0.072696
GRHL1	0.163188	0.015197	0.072696
C8orf60	-0.51176	0.015219	0.072782
CCKAR	-0.06662	0.015225	0.072798
DACT3	-0.02265	0.015244	0.072851
MYO5A	0.256355	0.015248	0.072851
CD99L2	0.078348	0.015249	0.072851
VPS51	-0.26542	0.01525	0.072851
CLEC4E	0.215112	0.015278	0.072972
NDUFAB1	0.225514	0.015286	0.072992
FUT2	-0.0288	0.015293	0.07301
FXR1	0.194388	0.015343	0.07323
EEF1E1	0.166114	0.015347	0.073234
SH3BP5L	0.134831	0.015366	0.073307
FAM132B	0.591691	0.015372	0.073307
SSBP2	-0.37204	0.015375	0.073307
MTF1	0.1776	0.015376	0.073307
TOR1B	0.187989	0.015379	0.073307
FAM63B	0.208252	0.015405	0.073412
TMC6	0.226272	0.015408	0.073412
ARL3	-0.1044	0.015426	0.07346
LSM3	0.131055	0.015426	0.07346
ROGDI	0.261261	0.015428	0.07346
TIE1	0.168669	0.015462	0.073602
FER	0.210642	0.015465	0.073602
SNCA	0.320701	0.015471	0.073617
PTDSS1	-0.20303	0.015484	0.073661
ZNF784	0.142375	0.01549	0.073676
LOC64677	-0.23233	0.015495	0.073681
CRBN	-0.16152	0.015517	0.073769
ZBTB33	-0.08796	0.015526	0.073785
GNL3L	0.138521	0.015527	0.073785
PHOSPHO	0.068622	0.015564	0.073943
RFPL1	0.071508	0.015572	0.073966
SERPINA9	-0.03905	0.015577	0.073974
ZNF627	-0.13325	0.015593	0.074034
FLJ10038	-0.27277	0.015627	0.07417
CLHC1	-0.37718	0.015629	0.07417
RAB22A	0.154069	0.015638	0.074198
NHP2L1	-0.26601	0.015671	0.07434
GABRB3	0.203852	0.015686	0.074394
ETS2	0.293166	0.015736	0.074616
NFU1	0.141169	0.015752	0.074674
ZNF581	0.336333	0.015759	0.074689
YARS	-0.22048	0.015782	0.074784
RP11-686D	0.213061	0.015793	0.074819
C8orf37	-0.19156	0.015798	0.074826
CSF2	-0.07157	0.01581	0.074867
TAL2	-0.04993	0.015841	0.074996
NUDT18	0.279923	0.015846	0.075004
TLR2	0.498394	0.015875	0.075123
SLC39A4	0.398745	0.015906	0.075257
MRPL22	0.326113	0.015917	0.075291
LOC10065	-0.07124	0.015922	0.075296
NDUFV1	-0.13266	0.015944	0.075384

MGST1	0.719226	0.015959	0.075438
DENR	-0.2131	0.015972	0.07548
BRAP	0.138033	0.015974	0.07548
SMIM4	-0.06827	0.015981	0.075496
STX7	0.228912	0.015992	0.075532
TNPO1	-0.16292	0.016013	0.075612
CPEB3	0.10604	0.016037	0.075711
SSRP1	0.219097	0.016041	0.075711
MT1F	0.581113	0.01605	0.075737
CRNKL1	-0.21542	0.016076	0.075844
VSIG1	0.00492	0.016092	0.075901
PAN3	-0.19729	0.016101	0.075931
PDRG1	-0.28521	0.016132	0.076035
LINC00851	-0.05929	0.016134	0.076035
AK055458	0.064794	0.016134	0.076035
SNTB1	-0.15443	0.016172	0.076197
IFT88	-0.17573	0.016182	0.076229
CYSLTR1	-0.34836	0.016201	0.076299
WFDC1	0.44385	0.016208	0.076316
C19orf26	-0.05344	0.016212	0.076318
MAGEF1	-0.20628	0.016216	0.076323
KCNS1	-0.09072	0.016224	0.076342
GRHPR	0.235445	0.016239	0.076399
DNMBP	0.261571	0.01625	0.076432
LOC10050	-0.14339	0.016269	0.076505
ARIH2OS	0.203066	0.016308	0.076673
LY6G6E	-0.02637	0.016332	0.076753
UROD	0.33125	0.016335	0.076753
CDK12	0.158545	0.016336	0.076753
IGFBP7-AS	-0.2366	0.016352	0.076812
PDCD2	-0.19687	0.016367	0.076867
ELTD1	0.814306	0.016382	0.076913
MFI2	0.118273	0.016388	0.076913
SPCS2	0.197086	0.016388	0.076913
IPO7	-0.16183	0.016398	0.076946
LOC39949	0.275984	0.016405	0.076961
MRPL36	0.233814	0.016414	0.076986
PTH2	-0.05488	0.016431	0.07705
LMO7	-0.03166	0.016435	0.07705
TFPI	-0.45813	0.016447	0.077091
ZSWIM1	0.09361	0.01647	0.077168
ICE1	-0.13336	0.016471	0.077168
MRPS21	-0.14559	0.016475	0.077173
ZNF26	-0.24384	0.016493	0.077238
RP11-365H	-0.12079	0.016496	0.077238
MTRF1L	-0.1259	0.016518	0.077325
LYN	0.23536	0.016545	0.077427
MARK2	0.076113	0.016547	0.077427
CTD-2269I	-0.03817	0.016552	0.077433
INSIG2	-0.22117	0.016559	0.077447
SMC6	-0.2072	0.016569	0.07748
DNAJC3	-0.34521	0.016576	0.07748
RRAGD	0.266298	0.016577	0.07748
BC038205	-0.05833	0.016582	0.07748
ZNF702P	0.122103	0.016584	0.07748
TAPT1	-0.29841	0.016617	0.077621
MED19	0.142171	0.016627	0.077651
CCZ1	-0.08853	0.016649	0.077734

RP11-489E	-0.2936	0.016693	0.07791
ALDH3A1	-0.10951	0.016694	0.07791
SLC22A25	0.021255	0.016697	0.07791
ZMYND19	0.249223	0.016722	0.07801
MDFIC	0.419701	0.016742	0.078086
MAPT	-0.02636	0.016755	0.078127
CDK8	-0.27518	0.01677	0.078161
ZNF703	0.134606	0.016773	0.078161
TPD52	0.289949	0.016774	0.078161
ZNF589	0.205191	0.016776	0.078161
MBIP	-0.31504	0.016792	0.078217
RP11-353N	-0.05232	0.016803	0.078242
CSNK2A2	0.179145	0.016805	0.078242
ADIPOR2	-0.22739	0.016855	0.078458
MAGT1	0.283398	0.016863	0.078467
ZNF324B	-0.04656	0.016864	0.078467
LY6E	0.370047	0.016877	0.078511
LRRC25	0.174086	0.016893	0.078567
CEL	0.132166	0.016915	0.078652
NACA2	0.118703	0.016921	0.078663
MTMR12	-0.15539	0.016949	0.078769
C21orf15	0.0367	0.01695	0.078769
LINC00998	-0.29312	0.016966	0.078825
DONSON	0.255277	0.016975	0.078851
CMPK2	0.340468	0.016984	0.078873
PPAT	-0.22295	0.017012	0.078989
ICT1	0.245778	0.017046	0.079129
LOC10192	-0.10783	0.01706	0.079174
MRPS2	0.29843	0.017063	0.079174
SLC25A48	-0.0398	0.017075	0.079205
PTRF	0.270424	0.017077	0.079205
TNF	0.494656	0.017085	0.079216
RAP1B	-0.14838	0.017087	0.079216
CNTD2	0.074363	0.017093	0.079227
ASS1	0.30507	0.017101	0.079247
FGF21	-0.04823	0.017117	0.079287
CENPBD1	0.293612	0.017119	0.079287
C1orf74	-0.18366	0.017121	0.079287
CSNK1D	0.28581	0.01714	0.079359
KCTD5	0.157449	0.017151	0.079396
ZNF343	-0.14884	0.017176	0.079482
LOC10050	0.088776	0.017182	0.079482
SLC44A3	0.206981	0.017183	0.079482
NME3	0.304738	0.017185	0.079482
RP13-270F	-0.23342	0.017197	0.07952
LYSMD3	-0.27796	0.017201	0.079522
ANKH	0.13686	0.017219	0.079592
PRLH	-0.11696	0.017241	0.079658
RP11-379H	-0.26417	0.017245	0.079658
POLR2K	0.196135	0.017247	0.079658
KLF2	0.413603	0.017249	0.079658
ITK	0.149974	0.017277	0.079771
DIAPH2	-0.2597	0.017317	0.079941
IGLV1-44	0.404827	0.01733	0.079984
RP11-342L	-0.26666	0.017341	0.080018
SLC12A4	-0.04932	0.017351	0.080045
MECR	0.312636	0.017363	0.080082
USP54	0.25785	0.017391	0.080179

HAUS5	-0.22176	0.017394	0.080179
CD244	-0.16691	0.017395	0.080179
C9orf66	0.150684	0.017408	0.080221
ISYNA1	-0.09676	0.017418	0.080251
TMEM18	-0.22568	0.017427	0.080278
FYCO1	-0.11664	0.017432	0.080282
CACNA1H	-0.04121	0.017437	0.080287
PPOX	0.232876	0.017445	0.080307
LOC10050(	0.13775	0.017455	0.080336
NR2C2	0.18361	0.017461	0.080349
LIMD2	0.076276	0.017468	0.080361
ETS1	0.291198	0.017475	0.080378
CEACAM1	0.156142	0.017481	0.080383
PRR7	0.127309	0.017483	0.080383
REEP4	0.150547	0.017497	0.080427
NIPAL2	-0.06917	0.017527	0.080547
ZNF347	0.137088	0.017531	0.080552
FAM186B	-0.04839	0.017547	0.080608
BRWD3	-0.1786	0.017583	0.080747
AP3B1	0.179792	0.017585	0.080747
MRPS35	-0.15395	0.01759	0.080753
ZGRF1	-0.13384	0.017594	0.080756
SYT17	-0.03608	0.017602	0.080772
EFCAB4B	-0.12177	0.017625	0.080843
GOLIM4	0.394007	0.017625	0.080843
RPS27	0.240318	0.017637	0.080876
FLJ35934	-0.16981	0.017639	0.080876
LINC00877	0.040889	0.017675	0.081025
LOC64479	0.089805	0.017683	0.081045
HMHA1	-0.12112	0.017708	0.081127
C1GALT1	-0.17102	0.017709	0.081127
ZNF580	0.222659	0.017757	0.081299
BC043356	-0.21721	0.017757	0.081299
DDX58	0.331404	0.017758	0.081299
ZNF77	-0.14822	0.017791	0.081435
HDAC3	0.219577	0.017814	0.081522
NABP2	0.244465	0.017864	0.081731
NANOG	-0.09619	0.017891	0.081842
FAT4	0.28633	0.017909	0.081904
KCTD10	-0.25075	0.017921	0.081942
TAF1L	-0.08286	0.017965	0.082127
ASB6	0.139391	0.017986	0.082203
MTMR3	0.092447	0.018	0.082252
MVD	0.115861	0.018007	0.082268
KCNA2	-0.01997	0.018023	0.082321
CD1D	0.440214	0.018028	0.082329
CCNC	0.190934	0.018054	0.082428
PPP1R13B	0.166545	0.018085	0.082554
SPATS2	0.181677	0.018095	0.082579
RPS21	-0.21453	0.018112	0.082634
BAGE	0.012773	0.018114	0.082634
DNAJC14	0.146133	0.018126	0.082671
RP11-143k	-0.02771	0.018136	0.082696
XKR8	0.174547	0.018147	0.08273
FAF2	0.164437	0.01817	0.082819
ZNF257	-0.3501	0.018185	0.082869
DDX6	-0.50902	0.018212	0.082977
DGCR11	-0.11583	0.01822	0.082994

LOC10050!	0.245089	0.018266	0.083188
RABGAP1L	0.201073	0.018272	0.083198
CTA-331P:	-0.33732	0.018302	0.083313
ANKRD19F	0.079589	0.018317	0.083368
NPRL3	0.241041	0.018325	0.083384
CNPPD1	0.131612	0.01833	0.083392
ETV6	0.247505	0.018334	0.083392
FBXL5	0.16199	0.01835	0.083413
BEND4	-0.23766	0.01835	0.083413
NPHP4	0.099174	0.018351	0.083413
ZC3H12B	-0.09575	0.018412	0.083673
CDKN1A	0.581395	0.018418	0.083685
RCOR3	0.19321	0.01846	0.083856
VPS36	-0.08092	0.018472	0.083893
HMGN4	-0.18878	0.018494	0.083976
C9orf156	-0.28614	0.018498	0.083979
CNNM3	0.264488	0.018506	0.083995
SRI	0.16909	0.018515	0.084021
MRPL15	0.203706	0.01856	0.084208
RP11-166F	-0.09699	0.018589	0.084319
RTF1	-0.2451	0.018595	0.084329
AKR1C3	-0.26399	0.018612	0.084391
RP11-235E	-0.22978	0.018652	0.084537
PSMB1	0.156088	0.018652	0.084537
ZMIZ2	0.0826	0.018664	0.084574
NUDCD2	0.198468	0.018708	0.084753
IGFBP5	0.034845	0.018729	0.084831
ZYX	0.507605	0.018733	0.084833
ATF5	0.107116	0.018749	0.084888
C4orf29	-0.2688	0.018757	0.084905
FAM8A1	-0.24002	0.018771	0.08495
NDC1	-0.24319	0.018795	0.085042
2-Sep	-0.25382	0.018846	0.085254
NAPRT	0.279881	0.018851	0.085256
UBE2D3	0.210409	0.018872	0.085323
SDHAF2	0.247461	0.018875	0.085323
DENND5A	0.401497	0.018877	0.085323
ICA1L	-0.08064	0.018899	0.085403
ANKRD16	0.213456	0.018958	0.085654
OR2L13	-0.18397	0.018966	0.08567
SPDL1	-0.24741	0.018971	0.085677
TCTN1	-0.12545	0.018995	0.085766
TNFAIP6	0.621156	0.019006	0.085799
FLJ42627	0.295502	0.019049	0.085975
BPHL	0.196658	0.019062	0.086012
KRI1	0.214231	0.019065	0.086012
C16orf54	-0.40919	0.019096	0.086132
PSMB9	0.276771	0.0191	0.086134
GALK2	0.278622	0.019156	0.086363
BAP1	0.168435	0.019159	0.086363
OR2F1	-0.04237	0.019168	0.086387
SMG7	0.209235	0.019186	0.086447
CASP10	0.058603	0.019192	0.086457
CMTR2	-0.39153	0.019233	0.086609
DCTPP1	-0.21918	0.019234	0.086609
LHPP	0.186593	0.019244	0.086639
NUP98	0.206898	0.01926	0.086691
MRPL2	0.236117	0.019277	0.086752

SERPINA12	-0.0701	0.019373	0.087164
TRIM37	-0.17688	0.019378	0.087169
ZNF219	0.068388	0.019408	0.087285
SORL1	-0.42399	0.019427	0.087354
SERINC1	0.292514	0.019457	0.08747
PIGW	-0.29933	0.019487	0.087585
LINC01366	-0.07884	0.019512	0.087681
STX5	0.241722	0.019534	0.08776
LINC00996	0.360122	0.019556	0.08784
LOC10192(	0.012798	0.019563	0.087857
MTHFR	-0.09642	0.019571	0.087872
HLA-DMB	0.297373	0.019615	0.088051
LINC01126	-0.30906	0.019625	0.088077
MBD3	0.199139	0.019644	0.088144
IMPA1	-0.24188	0.019655	0.088177
LRP5L	-0.30724	0.019678	0.08826
GLDN	0.264522	0.019706	0.088368
PGF	-0.10818	0.019714	0.088385
TMEM222	0.209905	0.019739	0.088472
NAA10	0.18947	0.019741	0.088472
RP11-330(	0.096017	0.019775	0.088603
SEC23IP	0.16967	0.019795	0.088675
MICB	-0.19591	0.019807	0.088714
NUDT13	-0.22286	0.019825	0.088775
LMAN2L	-0.22737	0.019832	0.088785
HS2ST1	-0.2148	0.019846	0.088831
SLC36A4	-0.23069	0.019879	0.088962
GPR37L1	-0.04167	0.019911	0.089085
CYC1	0.2556	0.019935	0.089174
PIGC	0.249994	0.019939	0.089174
SLC15A2	-0.46272	0.019959	0.089245
MRPL18	0.201073	0.019983	0.089333
ST6GALNA	0.218956	0.019988	0.089339
CDK18	0.07081	0.020013	0.08943
IRAK1BP1	-0.25228	0.020031	0.089494
DMAP1	0.164964	0.020039	0.089509
RPS16P5	-0.40907	0.020071	0.089633
CHN2	0.123136	0.020078	0.089648
MIR3682	-0.28488	0.020095	0.089704
STRIP1	-0.13096	0.020128	0.089835
PNN	0.151843	0.020137	0.089857
TREML2	0.063173	0.020149	0.089891
SNTB2	0.013152	0.020167	0.089951
LINC01016	-0.02227	0.02018	0.089985
KLHL10	-0.03187	0.020183	0.089985
SATB2	0.053749	0.020187	0.089985
KANK1	-0.10872	0.020234	0.090174
SCOC-AS1	-0.09287	0.020242	0.090193
FPR2	0.249258	0.020263	0.090267
PRKD2	0.298805	0.020267	0.090267
IGLC1	0.328477	0.020291	0.090341
KLHDC7A	-0.0209	0.020292	0.090341
CCT8	-0.17003	0.02033	0.090492
ARSB	0.124043	0.020358	0.090597
OSR2	0.125906	0.020372	0.09064
LOC44002{	-0.01217	0.020378	0.090651
DFFB	-0.23666	0.020398	0.090719
LINC01278	0.113991	0.020406	0.090737

HIST1H4E	-0.43425	0.020413	0.090752
DALRD3	0.177362	0.020424	0.09078
LOC10193(	0.398474	0.020448	0.090871
LOC10272	-0.12826	0.020481	0.090968
GPR97	0.16943	0.020483	0.090968
IRF2BP1	-0.10077	0.020483	0.090968
FBXO44	-0.06937	0.020499	0.091022
ANKRD13A	-0.1818	0.020507	0.091038
NYX	-0.08135	0.020522	0.091059
GPS1	0.217261	0.020524	0.091059
TMEM105	-0.04563	0.020525	0.091059
MUC19	-0.38185	0.020534	0.091071
GABBR1	0.25438	0.020536	0.091071
KDM5D	1.218066	0.020543	0.091071
TK1	0.344784	0.020544	0.091071
HYI	0.201947	0.020554	0.091086
RP11-255(	-0.04201	0.020556	0.091086
TRDV3	0.14747	0.020576	0.091155
HNRNPM	-0.33212	0.020595	0.091221
NOTCH3	0.066229	0.020614	0.091288
AIDA	0.216021	0.020687	0.09159
ORMDL3	-0.10563	0.020725	0.09174
GAR1	-0.25151	0.02074	0.091791
NR2F1	0.640494	0.02076	0.091861
LRFN4	0.107602	0.020772	0.091894
UBR5	0.195608	0.020839	0.092169
SPINK2	-0.48534	0.020894	0.092395
WDR83OS	0.166359	0.020899	0.092397
LRP10	0.204756	0.020965	0.092653
XRCC4	-0.22762	0.020965	0.092653
ZNF205-A	0.006249	0.020988	0.092728
KCNRG	-0.14434	0.020991	0.092728
EXTL1	-0.06692	0.021022	0.092848
RP11-1007	-0.10173	0.021047	0.092941
RP11-6I2.3	0.132313	0.021071	0.093025
LOC10192	-0.06648	0.021084	0.093065
BBS5	0.068466	0.021106	0.093139
RP11-548M	-0.14729	0.02111	0.093139
THEM6	0.276824	0.021117	0.093154
EPHB4	-0.2825	0.021136	0.093214
CRY1	-0.24149	0.021139	0.093214
EMC9	0.34381	0.021158	0.093276
CABP1	0.028655	0.021166	0.093294
COL27A1	0.066684	0.02119	0.093382
HLA-DPA1	0.350224	0.021211	0.093445
OXER1	0.066607	0.021213	0.093445
POLA2	-0.22932	0.021231	0.09348
LEPR	0.071796	0.021234	0.09348
NCDN	0.035704	0.021238	0.09348
C21orf91	-0.20233	0.021239	0.09348
GIPC3	0.056183	0.021262	0.093562
SDF4	0.164904	0.021303	0.093719
FAM185A	-0.17144	0.021306	0.093719
ZKSCAN5	-0.15169	0.021311	0.093722
INCENP	0.07768	0.021318	0.093736
RPS12	-0.04451	0.021336	0.093794
DCTN3	0.159793	0.021342	0.093802
CHERP	0.090239	0.021395	0.093953

ZNF571-A:	-0.20046	0.0214	0.093953
CTC-429P!	-0.19482	0.021401	0.093953
TMPRSS13	-0.06455	0.021403	0.093953
WDHD1	-0.30933	0.021404	0.093953
CTTNBP2N	0.292965	0.02141	0.093953
KCTD18	-0.16505	0.02141	0.093953
CEP290	-0.22049	0.021411	0.093953
LOC10028	0.165352	0.021418	0.093964
CDC14A	-0.26934	0.021442	0.094051
GOLGA2	0.143175	0.021456	0.094095
PTPRC	0.368206	0.021465	0.094114
ARL6IP1	-0.14222	0.021484	0.094166
RLF	-0.21384	0.021488	0.094166
ATRX	-0.20456	0.02149	0.094166
MT3	-0.07563	0.0215	0.094189
C10orf2	-0.29558	0.021508	0.094207
HDX	0.029265	0.02159	0.094528
STMN2	0.042246	0.021591	0.094528
LRRFIP2	-0.27869	0.021594	0.094528
ZNF681	-0.10059	0.021634	0.094678
ZEB1-AS1	-0.35164	0.021638	0.094678
CORO1C	0.233187	0.021647	0.094693
PRPF38B	-0.23821	0.02165	0.094693
SCAMP5	0.10236	0.02166	0.094718
HMGA1	0.191548	0.021673	0.094756
ATR	-0.19233	0.021693	0.094824
MORN3	-0.05523	0.021697	0.094825
ASL	0.30737	0.021744	0.095003
TNFAIP8	-0.25786	0.021747	0.095003
C15orf52	-0.33151	0.02178	0.095129
RP11-1008	0.069529	0.021791	0.095156
FAM13A-A	-0.27922	0.021796	0.095162
DIEXF	-0.24638	0.021843	0.095345
CIRBP	-0.27184	0.021861	0.095392
RHBDF2	0.184084	0.021862	0.095392
EEF1A2	-0.0673	0.021877	0.095434
ABHD14B	0.338186	0.021891	0.095461
DET1	-0.18026	0.021891	0.095461
TGM2	0.197361	0.021898	0.095469
IDH2	0.207242	0.021904	0.095472
CYP4F3	0.438133	0.021909	0.095472
MPST	0.307079	0.021912	0.095472
PSME1	0.165421	0.021918	0.095482
PTPLAD2	-0.10876	0.021936	0.095538
AP3M2	-0.17897	0.02195	0.09558
DSN1	0.292853	0.022033	0.095925
ALOX5	0.234882	0.022045	0.095958
WDR4	0.174805	0.022055	0.095983
B3GNT7	0.218965	0.02208	0.096071
SCLY	0.110484	0.022096	0.096122
BOLA3	0.338664	0.022145	0.096314
BCAS4	-0.13466	0.02217	0.096395
TBC1D2	0.188863	0.022172	0.096395
MFSD10	0.347752	0.022182	0.096414
THBD	0.476137	0.022188	0.096414
DNAJC3-A	-0.19958	0.02219	0.096414
CAMKV	-0.02999	0.0222	0.096419
ZNRF4	-0.08362	0.022205	0.096419

LOC72968:	-0.08055	0.022208	0.096419
WDR35	-0.20992	0.022209	0.096419
NFAM1	0.162255	0.022288	0.096743
RECK	0.191939	0.02232	0.096863
RP11-190/	-0.39813	0.02233	0.096886
SSR4P1	0.021782	0.022353	0.096967
PPM1G	0.271058	0.022375	0.097021
LINC01181	-0.18517	0.022379	0.097021
TACC2	0.100875	0.022381	0.097021
GOPC	-0.17353	0.022386	0.097021
TMEM101	-0.24334	0.022388	0.097021
GULP1	0.176427	0.022408	0.097087
ABHD4	0.227997	0.022432	0.097172
URGCP	0.130494	0.022456	0.097228
ARL4A	0.259225	0.022459	0.097228
NEURL3	-0.09691	0.022466	0.097228
CCT6B	-0.24999	0.022467	0.097228
SEPT7P9	0.136334	0.022467	0.097228
FTX	-0.59553	0.022486	0.097268
SNRNP200	-0.27068	0.022488	0.097268
TMEM180	0.174938	0.02249	0.097268
ARHGEF3	0.294473	0.022508	0.097328
SEC22A	-0.17043	0.022527	0.097388
HIST1H2AI	-0.14679	0.022542	0.097435
TRPM4	0.035786	0.022604	0.097683
HELB	-0.18301	0.022699	0.098072
RASGRP3	0.222058	0.022703	0.098072
AL833181	-0.51576	0.022708	0.098075
TMEM156	-0.27423	0.022774	0.098338
GPX2	-0.03707	0.022869	0.098708
RANBP3	0.137039	0.022872	0.098708
TSACC	-0.07309	0.022873	0.098708
COLCA1	0.125067	0.02289	0.098762
SF3B1	0.232968	0.022898	0.098778
CDSN	-0.01555	0.022924	0.098846
TXNL1	-0.35341	0.022925	0.098846
ASB8	0.152929	0.022928	0.098846
PIWIL1	0.114552	0.022932	0.098846
KIAA0753	-0.22392	0.022938	0.09885
LOC10028'	0.273239	0.022958	0.098915
PEPD	0.287209	0.022967	0.098937
ARHGAP26	0.201364	0.022979	0.09897
GRPEL2	0.165816	0.023004	0.099044
PEG3-AS1	-0.2441	0.023006	0.099044
GDPD3	0.180706	0.023045	0.099194
C1orf27	0.144659	0.023174	0.099728
SFR1	0.203378	0.023182	0.099743
LYRM4	0.196978	0.023214	0.099859
ZNF45	-0.14219	0.023238	0.099945
RWDD4	-0.16241	0.023338	0.100355
B3GALT4	0.120386	0.023345	0.100366
LOC10272	-0.29807	0.023356	0.100391
CIDEB	0.282947	0.023384	0.100494
TUBGCP3	0.233313	0.023408	0.100574
TSPAN17	0.148482	0.02342	0.100605
TTF1	0.178689	0.023482	0.100848
MYO1E	0.133681	0.023485	0.100848
C8orf82	0.337918	0.023491	0.10085

LINC01420	0.174949	0.023495	0.10085
LINC00957	0.060146	0.023533	0.10099
LOC10012	0.36216	0.023539	0.100999
HBD	0.697679	0.02363	0.101367
PRR35	0.058049	0.023644	0.101408
PSMF1	0.171594	0.023659	0.101454
PSMD9	0.145568	0.02375	0.101823
WDR90	-0.11507	0.023788	0.101967
FBXO16	0.155583	0.023822	0.102089
LOC10272	-0.05282	0.023831	0.102108
RBMXL2	0.004459	0.023838	0.10211
KIAA0586	-0.18565	0.023841	0.10211
IL18RAP	0.481874	0.023852	0.10214
METTL4	-0.19662	0.023865	0.102173
ANAPC15	0.260075	0.023917	0.102369
VPS16	-0.09252	0.02392	0.102369
DAPL1	-0.10233	0.023962	0.102505
DICER1	-0.27773	0.023965	0.102505
CELA2B	0.022697	0.023969	0.102505
LINC00173	-0.32203	0.023971	0.102505
UEVLD	0.124651	0.023981	0.10253
CDCA3	0.420969	0.024063	0.102859
RNF24	0.246121	0.024086	0.102937
MYCBP2	-0.17119	0.024119	0.103056
LOC10192	0.399293	0.024144	0.103134
UBAP2	-0.16418	0.024149	0.103134
GPATCH11	-0.14467	0.024156	0.103134
MGST2	-0.2035	0.024156	0.103134
PSIP1	0.190948	0.024168	0.103167
DDX3Y	0.562021	0.024217	0.103354
ALG10	-0.16582	0.024233	0.103403
PPP6R3	0.195415	0.024238	0.103405
DNM2	0.181432	0.024248	0.103423
MLYCD	-0.1238	0.024264	0.103474
PPA2	-0.35183	0.024294	0.103579
C2orf54	-0.08052	0.0243	0.103586
INPP5E	0.205792	0.024325	0.103673
C9orf116	0.035077	0.024362	0.10381
HLA-DQB2	-0.08911	0.024387	0.103895
LOC10050	-0.05496	0.024401	0.103935
GUSB	-0.11413	0.024442	0.104081
UNC5A	-0.04118	0.024445	0.104081
QRICH1	-0.16971	0.02445	0.104081
HSPA9	0.239659	0.024473	0.104158
TSPAN6	0.449774	0.024491	0.104214
LOC10063	0.097775	0.024504	0.104245
PPID	0.242107	0.024508	0.104245
PSMG3	0.206221	0.024561	0.10445
VPS9D1-A	-0.06256	0.024571	0.104475
COL11A1	0.164414	0.024582	0.104479
ELMO3	0.046915	0.024586	0.104479
IL34	-0.12364	0.024587	0.104479
FLJ11292	-0.01492	0.024606	0.104523
RP1-228H	-0.14273	0.024607	0.104523
LOC10272	-0.28824	0.024615	0.104536
SYNE3	-0.04628	0.024651	0.104653
RFC2	0.220429	0.024652	0.104653
LOC55310	0.044828	0.024669	0.104706

GTF3C2	-0.13782	0.024739	0.104984
RP3-508I1!	0.182003	0.024751	0.105013
TCOF1	0.219006	0.024758	0.105021
SOS1	0.478177	0.024767	0.105038
VWA1	-0.00386	0.024803	0.105172
SNX27	0.166287	0.024815	0.1052
RRAGC	0.183145	0.024842	0.105295
GBAS	-0.23565	0.024857	0.105339
AZIN1	-0.14252	0.024877	0.1054
AGPAT1	0.320632	0.024884	0.105411
MIF4GD	0.260294	0.024916	0.105526
LOC10050!	-0.07743	0.024927	0.105537
KMT2D	0.150807	0.024928	0.105537
DPYS	0.120455	0.024936	0.105549
HIC2	0.234628	0.024944	0.105561
FBXL18	0.033551	0.02495	0.105567
SORT1	0.315692	0.025035	0.105902
RADIL	-0.23803	0.025039	0.105902
LOC10192{	0.167557	0.025056	0.105946
LIPE	-0.05285	0.025059	0.105946
SPAG9	-0.24512	0.025134	0.106241
GALC	0.253036	0.025157	0.106317
FADD	-0.33355	0.025169	0.106347
SOCS6	-0.23797	0.025179	0.106358
AXIN1	0.130825	0.025182	0.106358
LOC22181·	-0.05767	0.025186	0.106358
BOLA2	0.129868	0.025218	0.106461
C16orf91	-0.2207	0.025224	0.106461
RTN4	-0.14272	0.02523	0.106461
FKBP2	-0.32284	0.025231	0.106461
SLAMF7	0.307201	0.025235	0.106461
TCF20	0.078786	0.02525	0.106503
FECH	0.387866	0.025295	0.106659
RP1-170O:	-0.10105	0.025297	0.106659
PPP1R16A	0.168963	0.025334	0.106796
SOD2	0.334595	0.025346	0.106824
SCARF1	0.19479	0.025351	0.106824
LINC01215	-0.49958	0.025419	0.10709
TMEM263	-0.24892	0.025459	0.107237
RPP25L	-0.20512	0.025512	0.107432
C12orf76	-0.18829	0.025519	0.107432
MMP13	0.057402	0.02552	0.107432
LYZL1	-0.02981	0.025571	0.107625
EDEM1	0.252834	0.025576	0.107625
RRP1B	-0.12893	0.025589	0.107662
FCHSD2	0.155287	0.025599	0.107672
SLC39A5	-0.03904	0.025602	0.107672
SLC6A6	0.034183	0.025607	0.107673
RP11-793H	-0.1701	0.025633	0.10776
AX746627	0.041696	0.025657	0.107843
TBCEL	0.183375	0.025674	0.107892
ALDH1A1	0.663204	0.025695	0.107958
ND2	0.195997	0.025712	0.108012
ITGB1BP2	0.119226	0.025732	0.108055
TRAM2	0.21328	0.025737	0.108055
BAMBI	0.504487	0.025738	0.108055
REL	0.342358	0.025757	0.108114
ABCB1	-0.48305	0.025779	0.108187

SLC36A1	0.149501	0.025796	0.108224
DDX1	-0.17377	0.025802	0.108224
HIST1H1D	-0.5137	0.025803	0.108224
ICA1	0.091608	0.025823	0.108258
ADSSL1	0.109984	0.025824	0.108258
SALL2	-0.32106	0.025826	0.108258
CDK5RAP2	0.158964	0.02584	0.108297
SERPINB2	0.685026	0.025853	0.108327
FLJ30064	0.014584	0.025922	0.1086
PFKL	0.185175	0.025928	0.1086
DNAJC5	0.259521	0.025939	0.108627
PANX1	0.164524	0.026011	0.108905
ZNF385C	0.133179	0.026019	0.108905
AGAP9	0.215916	0.026022	0.108905
CD97	0.377572	0.026028	0.108905
LOC10028	-0.2846	0.026031	0.108905
KLF3	0.287338	0.026049	0.10894
PHOSPHO	-0.28521	0.026053	0.10894
CNKSR1	0.154399	0.026057	0.10894
LARP7	-0.30505	0.026059	0.10894
LOC10192	-0.08498	0.026103	0.109102
KHNYN	0.181332	0.026117	0.109133
CXCL8	0.806673	0.02612	0.109133
PEX5	0.086631	0.026134	0.109169
PHLPP1	0.153996	0.026179	0.109336
AK096159	0.041741	0.026184	0.109337
CHKA	0.208828	0.02621	0.109424
FANCF	-0.20694	0.026239	0.109522
TMEM38B	-0.24245	0.026265	0.109592
DUS3L	-0.14792	0.026266	0.109592
YY1AP1	0.155254	0.026286	0.109657
ACHE	0.019953	0.0263	0.109695
MACC1	-0.32274	0.026345	0.109835
PSMC6	-0.13469	0.026345	0.109835
NEUROG3	0.025447	0.026349	0.109835
UBE2Q2	-0.28158	0.026378	0.109932
GPR75	-0.06544	0.026405	0.110026
GDPD5	0.140285	0.026427	0.110095
SCARNA2	-0.2047	0.026475	0.110259
HSPBP1	0.255234	0.026476	0.110259
TMED3	-0.14625	0.026554	0.110561
LRRFIP1	-0.37022	0.026562	0.110571
C11orf45	0.328571	0.026598	0.110701
SOCS1	0.048203	0.026611	0.110733
MAP1A	-0.12023	0.026616	0.110734
LINC00305	0.010987	0.026625	0.110751
RSBN1L	0.152331	0.02663	0.110751
PLCG2	-0.11603	0.026643	0.110768
ALAS1	0.214406	0.026645	0.110768
ELF2	-0.16215	0.026651	0.110771
ASTN2	0.085178	0.026664	0.110804
COMMD6	0.181332	0.026686	0.110875
UBXN11	-0.12036	0.026714	0.110953
ADAMTS2	0.039239	0.026718	0.110953
NXPH3	-0.04683	0.02672	0.110953
SNX13	0.058684	0.02674	0.111015
RBBP7	-0.11739	0.026802	0.11125
ZNF577	-0.12554	0.026813	0.111277

SLC16A11	-0.02818	0.026838	0.111345
CTD-2639I	-0.08544	0.02684	0.111345
KDM5A	0.162003	0.026858	0.111378
H2AFZ	-0.12465	0.026867	0.111378
ILF3	0.188898	0.026867	0.111378
TRO	-0.3223	0.026869	0.111378
UNC45A	-0.11407	0.026874	0.111381
USP25	-0.34767	0.026892	0.111433
NKX6-3	-0.05953	0.026906	0.111468
ARHGAP39	-0.07995	0.026931	0.111534
ZC3H7A	0.205308	0.026936	0.111534
IGJ	0.904251	0.026938	0.111534
PRKCQ-AS	-0.27074	0.026942	0.111534
LINC01192	-0.02996	0.026956	0.111569
RDH5	-0.31564	0.026983	0.111658
LYZL6	0.061947	0.026988	0.11166
C6orf47	-0.17001	0.02701	0.111718
SPRY3	0.071408	0.027013	0.111718
EXTL3-AS1	-0.12299	0.027018	0.111718
TENC1	0.070057	0.027044	0.111795
AVPI1	0.26821	0.027047	0.111795
ARMC1	0.184196	0.027052	0.111797
SLC22A13	0.008278	0.027077	0.111879
MDM2	0.121507	0.027086	0.111892
SDR42E1	-0.2078	0.027104	0.111947
MAPK8IP3	-0.08702	0.027117	0.111977
PQLC1	0.095274	0.027151	0.112096
FAM160A1	-0.04881	0.027177	0.112182
PAFAH2	0.090976	0.027201	0.11226
IARS2	-0.16328	0.027248	0.112434
LOC10272	-0.35547	0.027266	0.112488
SPG21	0.07562	0.027276	0.112505
NOXA1	0.101774	0.027299	0.11258
DHRSX	0.202863	0.027369	0.112817
FAM161A	0.208121	0.027371	0.112817
DND1	-0.23632	0.027372	0.112817
BCORL1	-0.04093	0.027386	0.112853
EFCAB13	-0.01661	0.02745	0.113097
DBH-AS1	0.142618	0.027482	0.113206
FNDC3B	-0.10381	0.027513	0.113311
DGCR9	-0.0351	0.027523	0.113315
CLSPN	0.082111	0.027524	0.113315
B4GALNT4	-0.02619	0.027542	0.113369
HMGB4	0.060732	0.027561	0.113424
POLR3B	-0.13832	0.02759	0.11352
NEDD4	-0.20428	0.027597	0.113529
CCDC59	0.175488	0.027629	0.113639
HIC1	0.050105	0.027659	0.113741
RP11-61L1	-0.23313	0.027676	0.113788
CIAPIN1	-0.20746	0.027714	0.113909
ESRRG	0.052365	0.027716	0.113909
LRRC8B	-0.31133	0.027784	0.114169
USP4	0.134002	0.02779	0.114169
CTD-3080I	0.012551	0.027815	0.114251
RPS6KA5	-0.28801	0.027849	0.114368
GSTCD	-0.20027	0.027897	0.114546
LOC10050	-0.01641	0.027923	0.114632
LINC01314	0.015785	0.027977	0.114831

CSTF3	-0.18867	0.028024	0.115
NR3C2	0.189511	0.028032	0.11501
PPP3CB-A:	-0.09878	0.028048	0.115056
PSMD3	0.214145	0.028068	0.115117
BAIAP2	0.075961	0.028075	0.115122
PROCA1	0.10949	0.028086	0.115146
TUBA1C	0.141195	0.028097	0.115171
MRPS14	-0.17251	0.02813	0.115277
DCBLD1	0.24028	0.028134	0.115277
TFB2M	-0.04036	0.028164	0.115372
MGC16275	-0.11367	0.028169	0.115372
GPR21	-0.17767	0.028173	0.115372
ZNF385D	0.314805	0.028214	0.115517
LPIN1	-0.22539	0.028224	0.115535
RP11-85A1	-0.0359	0.028258	0.115653
EIF1B	0.091386	0.028277	0.115708
C17orf59	0.13116	0.028308	0.115817
PPM1F	-0.15509	0.028334	0.1159
PIPOX	0.339788	0.028367	0.116013
MGC45922	-0.09275	0.028377	0.116033
CCNT2-AS	0.066365	0.028399	0.1161
RNF38	-0.12056	0.028422	0.116139
MMS19	0.193982	0.028423	0.116139
NLRP12	0.150241	0.028425	0.116139
WBSCR22	0.121291	0.028436	0.116161
RP11-314N	-0.21447	0.028497	0.11639
MYL6B	0.208903	0.028513	0.116425
BACE1	0.235218	0.028516	0.116425
C5orf55	0.139377	0.028596	0.116727
DNAJC30	-0.18041	0.028608	0.116749
BRAF	-0.19693	0.028612	0.116749
CCDC78	0.081158	0.028625	0.11678
DUSP1	0.408456	0.02867	0.116941
FAN1	-0.20003	0.028709	0.11708
ZNF786	0.308933	0.028756	0.117247
HMBS	0.439417	0.028784	0.117343
FBXO10	-0.02204	0.028804	0.117392
RBM12	-0.13883	0.028809	0.117392
GLRX3	0.144617	0.028813	0.117392
TBC1D14	0.170323	0.028833	0.117454
PPP1R35	0.206103	0.028886	0.11764
LINC00997	0.13242	0.02889	0.11764
NSL1	-0.13437	0.028912	0.117694
THG1L	-0.35009	0.028922	0.117694
PTPRH	0.052963	0.028922	0.117694
SSBP1	-0.28301	0.028928	0.117694
MED6	0.212776	0.02893	0.117694
CA9	-0.06559	0.028945	0.117731
PAK6	0.19824	0.028982	0.11786
LOC10192	0.219404	0.028992	0.11788
PCDHGA9	0.028412	0.029006	0.117913
CHMP5	0.198857	0.029021	0.117951
CDC42EP5	0.027163	0.029029	0.117961
CYP1A2	0.084878	0.02904	0.117985
RABGGTB	-0.17938	0.029057	0.118032
SUMO4	0.288416	0.029105	0.118194
KIAA1586	-0.2069	0.029111	0.118194
RRP1	-0.12408	0.029113	0.118194

LOC10192	0.020351	0.02913	0.118239
TOMM22	0.197653	0.029144	0.118275
ARSD	0.266754	0.029226	0.118555
EED	-0.21466	0.029229	0.118555
LY75	-0.42678	0.02923	0.118555
LOC43991	-0.2836	0.029243	0.118588
LSP1	0.314921	0.029255	0.118614
LOC28439	-0.06007	0.02927	0.118651
CELF2	-0.25889	0.029308	0.118786
ZNF8	-0.25235	0.029331	0.118854
ZNF621	-0.2402	0.029346	0.118893
L3MBTL2	0.169658	0.02936	0.118928
EXPH5	-0.19407	0.029398	0.119058
DUOXA2	-0.19471	0.029425	0.119147
ATP13A1	0.21681	0.029455	0.119213
MALT1	0.273144	0.029458	0.119213
HELZ	0.271789	0.029458	0.119213
SEMA4G	-0.02345	0.029478	0.119271
MNX1	-0.03388	0.0295	0.119338
KCNIP4	-0.01892	0.029507	0.119345
C4orf32	-0.16444	0.029541	0.119461
LSM5	-0.2265	0.029572	0.119559
BLZF1	-0.2571	0.029576	0.119559
MED17	-0.2292	0.029642	0.119765
FAM199X	0.263888	0.029642	0.119765
SREBF2	-0.19769	0.029644	0.119765
MMRN2	0.185428	0.029659	0.119801
AOC2	0.014774	0.02967	0.119827
TMEM245	0.178351	0.029709	0.119961
HIST1H4D	-0.1265	0.029749	0.120092
ZFPM1	0.161497	0.029755	0.120092
SH3GL3	0.085215	0.029758	0.120092
NPPB	-0.03672	0.029768	0.120097
ZFAT	0.058221	0.02977	0.120097
SELO	0.206087	0.029813	0.120246
LRRN1	0.49358	0.029908	0.120608
PARM1	0.140027	0.029966	0.120818
CFD	0.629553	0.029991	0.12088
PLB1	0.06226	0.029993	0.12088
LINC01003	-0.28733	0.030012	0.120934
GPC2	-0.19546	0.030031	0.120989
GLA	-0.20215	0.030048	0.121034
PTPRU	0.084656	0.030061	0.121065
BC045784	-0.06458	0.030066	0.121065
PSMB10	0.274018	0.030077	0.121069
HBZ	0.171089	0.030079	0.121069
LSM1	0.182809	0.030154	0.121305
TBC1D22B	-0.20944	0.030156	0.121305
CLEC4A	0.367118	0.03016	0.121305
FFAR4	0.020807	0.03016	0.121305
SLC35C2	0.145596	0.030168	0.121305
ZNF622	-0.18959	0.030171	0.121305
RP11-445H	-0.02942	0.030214	0.121455
TCN1	0.625117	0.030247	0.121566
CLDN12	-0.28128	0.030294	0.121733
PLAA	0.217973	0.030365	0.121994
HEXA	-0.2164	0.030376	0.122018
C15orf57	0.253013	0.030433	0.122222

LOC10013:	-0.03371	0.030467	0.122338
C12orf10	0.196912	0.030505	0.12246
AK2	0.200149	0.030509	0.12246
ZNF506	-0.22116	0.030555	0.12262
CLEC14A	0.136332	0.030604	0.122794
LSS	0.184844	0.030633	0.122891
ZNF442	0.13415	0.030641	0.122899
RP11-305E	-0.36426	0.030658	0.122944
TSPAN10	-0.04773	0.030664	0.122945
PCDHB17	-0.03061	0.030706	0.12309
KRTAP4-3	0.005895	0.030724	0.123139
GLTSCR2	-0.13214	0.030745	0.123202
SKP2	-0.28123	0.030813	0.123452
PIN1P1	-0.09296	0.030869	0.123652
COX7A2	-0.1093	0.03089	0.123715
HIST1H4A	-0.22745	0.030899	0.123727
ZFAS1	0.348546	0.030954	0.123926
ZNF292	-0.34354	0.030976	0.12399
RTCA	0.107038	0.030991	0.124028
SPRN	-0.04168	0.030999	0.124029
MAPKAP1	0.188874	0.031003	0.124029
TAS2R10	-0.09715	0.031022	0.124082
DSTN	0.334353	0.03105	0.12417
ABLIM3	0.318779	0.031071	0.124221
ZNF619	0.057765	0.031074	0.124221
MIEN1	0.19869	0.031097	0.124292
IFT74	0.243315	0.031129	0.124396
UCN2	-0.02231	0.031145	0.124438
ZMYND8	-0.23258	0.031157	0.12446
TCEAL4	-0.14827	0.031197	0.1246
ITPKB	0.234529	0.031204	0.124602
CYB561A3	0.238864	0.031259	0.124798
BCL7B	0.221272	0.031266	0.124805
RANBP1	0.146681	0.031275	0.124818
LOC10013:	0.005753	0.031283	0.124827
ZNF623	-0.27814	0.031306	0.124886
ADH5	-0.13606	0.031309	0.124886
PTCSC1	-0.05584	0.03134	0.124985
PUF60	0.184837	0.031377	0.125088
FGF13	0.71681	0.031378	0.125088
TM4SF1	0.577774	0.03145	0.125356
RAB13	-0.22138	0.031472	0.125418
SLC7A5	0.405496	0.031482	0.125434
RPL31	-0.33194	0.031495	0.125444
LOC28605	0.036084	0.031496	0.125444
DHPS	0.273276	0.031512	0.125474
ITIH6	0.013755	0.031515	0.125474
NETO1	0.003286	0.031536	0.125516
HBEGF	0.249315	0.031537	0.125516
ALG14	0.141792	0.031573	0.125635
LOC10334	-0.1186	0.031602	0.12573
RSG1	-0.08691	0.031608	0.12573
PCNXL4	-0.09407	0.031625	0.125766
A1BG	0.087423	0.031634	0.125766
SUCLG2	0.615077	0.031638	0.125766
LINC00887	0.010804	0.03164	0.125766
IRS4	-0.08541	0.03166	0.125806
FAM111B	0.241217	0.031662	0.125806

TBX6	-0.04417	0.031681	0.125853
MAPKAPK5	0.188088	0.031686	0.125853
MGC70870	-0.40179	0.031784	0.126221
FBXO15	-0.25761	0.031816	0.126323
NAA50	0.17619	0.031852	0.126444
FBXL15	0.170214	0.03187	0.126483
ATG10	0.206812	0.031873	0.126483
C14orf80	0.193575	0.031927	0.126673
SAP130	-0.17028	0.031939	0.126699
USP34	-0.17654	0.031962	0.126766
LOC38933	-0.04268	0.032009	0.126928
SSX1	-0.03732	0.03203	0.126967
TRDMT1	-0.17767	0.032038	0.126967
PLAGL2	0.221911	0.032038	0.126967
TRIAP1	-0.16154	0.032042	0.126967
FAM90A1	-0.09436	0.032069	0.127051
LRRC55	-0.02311	0.032076	0.127056
ATP9A	-0.29397	0.032098	0.127119
LOC38869	-0.16068	0.032112	0.127148
RAI1	-0.08204	0.032121	0.127162
EID3	-0.31427	0.032157	0.127281
LOC10192	-0.01717	0.032177	0.127331
ZSWIM3	0.202346	0.032181	0.127331
LINC00667	0.147478	0.03223	0.1275
PRB1	-0.13393	0.032244	0.127522
NDST1	-0.14468	0.032247	0.127522
LOC10192	-0.03645	0.032253	0.127522
RIC8B	-0.14471	0.032282	0.127612
NPRL2	-0.09729	0.032301	0.127652
SYTL2	-0.30708	0.032303	0.127652
AIP	0.184502	0.03231	0.127653
MRPL19	-0.28857	0.032347	0.127776
RBMY3AP	-0.05969	0.032368	0.127837
HAS3	-0.03618	0.03239	0.127902
SLC43A3	0.257768	0.032409	0.12795
SLC11A1	0.110028	0.032417	0.12796
AVL9	-0.1286	0.032428	0.127981
NFYC-AS1	-0.21546	0.032555	0.128458
FRMD1	-0.04482	0.032564	0.128472
NADK2-AS	0.013633	0.032582	0.128519
HEATR3	0.235948	0.032599	0.128561
LOC10013	-0.27354	0.032649	0.128736
KCNAB3	-0.03508	0.032673	0.128805
RPS8	0.036082	0.032719	0.128965
PADI4	0.172678	0.03275	0.12905
PPIEL	0.082776	0.032753	0.12905
LOC10192	0.009311	0.032797	0.129201
LBP	0.207717	0.032822	0.129261
C16orf89	0.029587	0.032824	0.129261
NDUFB3	0.126751	0.032857	0.129367
POLL	0.185663	0.032877	0.129414
SLC16A9	0.460448	0.032881	0.129414
DNPEP	0.250423	0.032908	0.129495
THADA	-0.08511	0.032952	0.129646
TIA1	-0.16339	0.032993	0.129748
BRD7	0.183789	0.032993	0.129748
EXTL3	0.059948	0.032996	0.129748
WNT5B	-0.05572	0.033103	0.13012

SLC41A1	0.144113	0.033104	0.13012
CYFIP1	0.199507	0.033108	0.13012
CLEC4G	0.610949	0.033125	0.130154
NRG1-IT1	0.013208	0.033134	0.130154
RP11-266A	0.074162	0.033135	0.130154
ZNF239	-0.27223	0.033179	0.130299
PPIL3	-0.20211	0.033189	0.130299
VILL	0.244307	0.03319	0.130299
RP11-212F	-0.14323	0.033222	0.130386
CEP192	-0.22173	0.033224	0.130386
CEP112	0.00707	0.033234	0.1304
NSMCE4A	0.323487	0.03324	0.1304
POLH	0.162374	0.033311	0.130646
DGAT2	0.227901	0.033315	0.130646
UBAP1	0.168731	0.033403	0.13097
EI24	0.146788	0.03343	0.131044
RP11-548H	0.08771	0.033434	0.131044
AMPD2	-0.18064	0.033451	0.131086
SMYD4	0.221541	0.033458	0.131089
KCNQ3	0.019912	0.033492	0.131201
ZNF79	0.182427	0.033507	0.131236
CTD-2587I	-0.02556	0.033519	0.131257
SLC2A1-AS	-0.03193	0.033527	0.131266
HNRNPU-A	-0.33951	0.033542	0.131298
CCM2L	0.086859	0.033551	0.131311
CNGA4	-0.04661	0.033558	0.131314
CORO7	0.154068	0.033612	0.131504
C16orf95	-0.15151	0.033642	0.131596
RAP1GAP2	-0.23524	0.033685	0.131741
ZNF281	-0.12887	0.033715	0.131813
SF3B4	0.235979	0.033716	0.131813
RP5-991G2	-0.18548	0.033759	0.131957
SLC39A11	0.237675	0.033854	0.132308
HOXB5	0.232985	0.033872	0.132352
LINC00202	-0.05842	0.033926	0.132517
TRPC4AP	0.183931	0.033929	0.132517
BTF3	-0.11406	0.033932	0.132517
ADARB2	-0.04551	0.033946	0.132546
NAIF1	0.079161	0.033993	0.132703
GPA33	-0.35624	0.033998	0.132703
CNBD1	-0.03243	0.034017	0.132753
DMTN	0.312607	0.034045	0.132836
NDUFS4	0.165806	0.034056	0.132856
ARHGEF2	0.218378	0.034086	0.132948
UPRT	-0.18542	0.034095	0.132961
LOC100507	-0.02471	0.034136	0.133098
RAB9B	-0.20806	0.034146	0.133098
CPSF2	0.169677	0.034149	0.133098
TFF3	0.171455	0.034165	0.133139
STK39	-0.19825	0.0342	0.13325
LOC283112	0.020929	0.034228	0.133336
DYRK2	0.173491	0.034274	0.133491
PPIL4	-0.30999	0.034284	0.133506
PLAGL1	-0.31329	0.034305	0.133564
CEBPZOS	0.138019	0.034311	0.133564
THY1	-0.12772	0.034351	0.133695
VPS13B	-0.11793	0.034421	0.133941
SLC22A17	0.092005	0.034438	0.133985

LOC10192	0.019226	0.034444	0.133985
PAQR4	0.227282	0.034502	0.134166
PRDM2	0.222389	0.034507	0.134166
PCBP3	0.103801	0.03451	0.134166
C2orf27A	-0.12492	0.034518	0.134174
TOP3A	0.162604	0.034553	0.134286
OSGEPL1	-0.17431	0.034609	0.134479
PSEN2	0.263472	0.034628	0.134514
LPCAT4	0.111942	0.03463	0.134514
TRAIP	-0.25288	0.034646	0.134537
PYGL	0.253937	0.034649	0.134537
SHISA5	0.17739	0.03466	0.134558
CCDC97	0.117301	0.034692	0.134658
AC017002.	0.407437	0.034707	0.134692
SLFN12	0.260683	0.034761	0.134879
DAZAP2	0.126335	0.034798	0.134995
NSFL1C	0.147718	0.034818	0.134995
KRT10	-0.124	0.034819	0.134995
ZNF750	0.007636	0.034819	0.134995
GGT1	-0.02976	0.034823	0.134995
POU5F1P4	0.00986	0.034839	0.135033
SLC35A2	0.071681	0.03485	0.135052
LAMB1	0.211805	0.034899	0.135217
ACSL3	0.202079	0.034922	0.135285
MRVI1	0.316275	0.034935	0.135309
LYZ	0.619505	0.034946	0.135326
RPL7L1	0.143334	0.034957	0.135326
ZNF626	-0.1384	0.034966	0.135326
EMC8	0.163939	0.034968	0.135326
ITFG1	-0.19209	0.03497	0.135326
ATP1B1	0.347448	0.035119	0.135878
U91328.20	-0.05137	0.035127	0.135885
CCNJ	0.217459	0.03516	0.135988
GINS4	-0.1206	0.035207	0.136143
LDOC1L	-0.17516	0.035257	0.136303
LPGAT1	-0.18486	0.035261	0.136303
IDH3G	0.134369	0.035274	0.136332
UBR1	-0.18431	0.035348	0.13659
HRSP12	0.153358	0.035372	0.13666
KB-1410C5	0.008651	0.03539	0.136705
FBXL19	0.133563	0.035402	0.136728
SLC19A2	-0.2978	0.035413	0.136747
KIF3B	0.127894	0.035423	0.136756
MPG	0.217792	0.035428	0.136756
SNX22	0.117451	0.035452	0.136806
DKK1	0.196121	0.035454	0.136806
OSCAR	0.228345	0.035469	0.136841
ADAMTS7	0.014734	0.035504	0.136949
LOC64277	0.020274	0.035517	0.136977
RPS23	0.212637	0.035552	0.137065
RP11-126C	-0.03785	0.035553	0.137065
HPS3	0.147521	0.035601	0.137227
KLLN	-0.05029	0.03563	0.137315
TNFRSF13E	-0.10781	0.035644	0.137342
RHOF	0.162382	0.03565	0.137342
RAB2B	0.169937	0.035682	0.137443
IL25	0.004231	0.03572	0.137565
PLSCR4	-0.39267	0.035776	0.137755

LOC10272	-0.14912	0.035818	0.137891
EIF2B5	-0.07825	0.035841	0.137957
ARHGAP1	-0.08001	0.035848	0.137958
USP28	0.184571	0.035867	0.137996
NUCKS1	0.158201	0.035871	0.137996
NUP155	-0.25892	0.035883	0.138019
MT2A	0.565512	0.035895	0.13804
YIF1B	0.17019	0.035924	0.138128
DCUN1D1	0.166831	0.035963	0.138253
EIF4EBP2	0.148825	0.03597	0.138257
MOCS1	0.091211	0.035997	0.138334
VWDE	-0.47452	0.036038	0.138458
DPY19L2P3	-0.09398	0.036042	0.138458
KANSL2	-0.19766	0.036085	0.138598
CCIN	-0.0218	0.036097	0.138619
EIF1AY	1.199591	0.036103	0.138619
LOC10192	0.122853	0.036124	0.138674
ADCK5	0.150712	0.036131	0.138676
RP11-539I	-0.1122	0.036147	0.138713
DEFB108B	0.013965	0.036153	0.138713
RASSF7	0.080194	0.03616	0.138714
HPSE	0.549842	0.036177	0.138756
TOMM20	0.161478	0.036197	0.138807
CAMK1D	0.164515	0.036208	0.138827
ZNF791	0.23556	0.036219	0.138841
LOC10050	0.184089	0.03625	0.138935
FTSJ2	0.209732	0.03627	0.138969
FAM76A	-0.12588	0.036271	0.138969
CNTN3	0.111191	0.036279	0.138973
RIMKLB	0.243425	0.036343	0.139195
MGAT5	-0.10228	0.036356	0.139222
RUFY2	0.069058	0.036376	0.13927
LDB2	0.2672	0.036393	0.139314
PARP4	0.207035	0.03641	0.139352
CCT4	-0.09109	0.036467	0.139548
TMEM251	0.193872	0.036504	0.139665
C11orf16	-0.09906	0.036517	0.139687
KPNA2	-0.20405	0.03653	0.139714
SMC3	0.275613	0.036539	0.139724
ZNF567	0.273667	0.03656	0.139777
SF3B3	-0.14428	0.03667	0.140176
LOC44124	-0.07123	0.036697	0.140253
ADPGK	0.186653	0.036754	0.140446
ZNF568	-0.06144	0.036799	0.140593
SLC9A5	-0.03102	0.036838	0.140705
TMEM62	-0.10804	0.036841	0.140705
LOC64676	0.173244	0.036888	0.14086
KIAA1551	0.262579	0.03694	0.14103
PTRH2	-0.20134	0.036955	0.14103
DIXDC1	0.10796	0.036958	0.14103
ILK	0.268889	0.036959	0.14103
SRR	0.243211	0.036966	0.141032
M1AP	0.009699	0.037045	0.141303
INHBC	0.034094	0.037052	0.141303
TRIM59	-0.27624	0.037057	0.141303
CYTH2	0.246197	0.037064	0.141307
CCL5	0.840696	0.037084	0.141335
AMOTL2	0.224925	0.037084	0.141335

TMEM147	0.168098	0.037099	0.141366
TELO2	0.169806	0.037141	0.141502
IRS2	0.25988	0.037185	0.141608
ANK1	0.489408	0.037185	0.141608
CCS	0.241563	0.037189	0.141608
TAF1A	-0.25113	0.037205	0.141645
SEMA3F	0.145613	0.037215	0.141651
EXOSC10	0.08277	0.03722	0.141651
VPS18	0.106545	0.037259	0.141753
ZHX3	-0.08243	0.03726	0.141753
C5orf45	0.099049	0.037306	0.141893
BCL2L11	0.331974	0.03731	0.141893
RP11-552M	-0.07758	0.03734	0.141984
DCPS	0.241306	0.037349	0.141994
BBS7	-0.23701	0.037358	0.142001
ZNF441	0.098882	0.037368	0.142014
UBFD1	0.176447	0.037386	0.142059
OGG1	0.216094	0.037411	0.142128
ZNF439	-0.33477	0.037418	0.14213
PLA2G4C	0.156245	0.037432	0.142157
RP11-422F	0.163922	0.03745	0.142204
NT5DC2	-0.06748	0.03748	0.142266
PSMC2	-0.17979	0.037483	0.142266
MLF1	0.440911	0.037486	0.142266
IDH3B	-0.21642	0.037516	0.142354
CECR6	0.066143	0.037552	0.142465
PPP4R1	-0.12667	0.037559	0.142466
TMEM206	0.181164	0.037596	0.142581
RGCC	0.372697	0.037617	0.142637
DDX31	-0.1659	0.037653	0.142747
SULT2B1	-0.05156	0.037678	0.142797
INO80	0.165492	0.037679	0.142797
HERC2	-0.16153	0.037695	0.142831
ARHGAP25	0.277327	0.037722	0.142908
SMAD6	-0.04012	0.037733	0.142925
LOC10192	-0.01604	0.037775	0.14306
PDK1	0.217877	0.037793	0.143101
RP11-533E	-0.22533	0.037801	0.143101
TCEB3B	-0.06548	0.037806	0.143101
FAM149B1	-0.21315	0.037828	0.14316
TMED4	0.281683	0.037872	0.143286
MMADHC	-0.12267	0.037879	0.143286
C7orf34	-0.02528	0.037884	0.143286
S100B	0.085772	0.037888	0.143286
CRYAB	0.009112	0.037914	0.14336
ELK1	0.060406	0.03795	0.143472
CUX1	0.234241	0.037975	0.143515
SOS2	0.101084	0.037975	0.143515
MCPH1	-0.16708	0.037994	0.143563
FAM83F	0.099362	0.038048	0.143741
RP11-753A	0.032237	0.038061	0.143767
ST8SIA6-A	-0.08436	0.038143	0.14405
SMARCD1	0.081477	0.038158	0.14407
PSD4	0.101583	0.038166	0.14407
RP3-522P1	-0.17451	0.038168	0.14407
PANK1	-0.37015	0.038204	0.144182
C1QTNF1	-0.04355	0.038215	0.144195
A1CF	-0.04573	0.038236	0.144243

RP1-202O8	-0.02782	0.038241	0.144243
PSMD6	0.170419	0.038336	0.144545
C5orf30	-0.23807	0.03834	0.144545
S100A10	0.414948	0.038341	0.144545
TUBBP5	-0.03404	0.038379	0.144664
LOC15793	-0.02094	0.038439	0.144866
HOXB9	0.024118	0.038507	0.145096
RP11-307C	0.005882	0.038535	0.145175
PARP16	-0.15746	0.038551	0.14521
LOC10050	-0.05411	0.038577	0.145284
HIST1H4G	-0.02432	0.038603	0.145357
LOC10192	-0.04299	0.038678	0.145614
LOC10192	0.013743	0.038702	0.145678
NHSL1	0.045379	0.038842	0.146182
LOC10050	-0.04443	0.038889	0.146332
PDCD11	-0.16464	0.038902	0.146348
CASP9	0.10721	0.038907	0.146348
SNORD114	0.190843	0.038928	0.146404
TLR1	0.421033	0.039023	0.146734
COL7A1	-0.0596	0.03904	0.146773
LOC10012	-0.08305	0.039064	0.146836
KIF1C	0.11348	0.03907	0.146836
IFT172	0.196561	0.039081	0.14685
ICOSLG	0.040594	0.039147	0.147064
SSFA2	-0.25451	0.039151	0.147064
USP5	-0.15536	0.039176	0.14713
DHX29	-0.12497	0.039183	0.147132
SPATA31C	-0.05827	0.039199	0.147165
NOSTRIN	0.213051	0.03923	0.147257
PITHD1	0.115805	0.03925	0.14729
NDUFB2	0.223851	0.039257	0.14729
LOC10050	-0.0797	0.039259	0.14729
WHSC1L1	-0.15355	0.039295	0.147397
WFS1	0.204655	0.039347	0.147551
AX747064	0.01375	0.039349	0.147551
SURF2	0.118253	0.039368	0.147595
TLCD1	0.113323	0.039379	0.147612
PIP	-0.02436	0.039438	0.147808
UBE2E3	0.16115	0.039446	0.147811
VRK3	0.114772	0.039467	0.147863
MAGED1	-0.1815	0.039489	0.147922
APP	0.349608	0.039501	0.147941
ABCE1	-0.10826	0.039614	0.148339
SLC4A1AP	0.088405	0.039622	0.148343
DECR1	0.158817	0.039648	0.148399
RP11-65J3	-0.08539	0.039651	0.148399
SLC16A14	-0.62343	0.039741	0.148712
CNST	-0.23927	0.039758	0.148748
ASAP1	0.252668	0.039782	0.148813
MCF2L2	0.025041	0.039791	0.14882
PIK3CG	0.235202	0.039896	0.149187
MGC24103	0.002811	0.039904	0.149191
CSNK1E	0.134222	0.039929	0.149261
HCRP1	-0.3386	0.040017	0.149562
WSCD2	-0.02037	0.040032	0.149592
DDX11L2	-0.40944	0.040058	0.149664
EMCN	0.381529	0.040069	0.149674
C16orf74	0.258936	0.040083	0.149674

MTFR1	0.293562	0.040083	0.149674
NEK2	0.274249	0.040088	0.149674
SNX29	-0.10472	0.040109	0.149727
AGPAT5	-0.21551	0.040133	0.149786
PLCH2	-0.03005	0.040139	0.149786
CD247	0.147825	0.040175	0.149896
ERAP1	-0.42731	0.040257	0.150157
ERVW-1	-0.05204	0.040259	0.150157
SLCO2A1	0.092435	0.040276	0.150193
LOC10063(	-0.24688	0.040293	0.150213
LETM2	-0.09985	0.040295	0.150213
NT5DC3	-0.10209	0.04032	0.150282
SNAP25	0.017817	0.040336	0.150315
LOC10028'	0.154968	0.040384	0.150467
SETD7	0.21687	0.040402	0.150507
PSG5	-0.03744	0.040426	0.150571
SPATA24	-0.10954	0.04048	0.150748
LILRA3	0.350663	0.040514	0.150849
SLC1A5	0.218909	0.04056	0.150993
LOC10099(	0.234825	0.040575	0.151022
PLRG1	0.137075	0.040636	0.151225
DRAM1	0.27726	0.040656	0.151272
MYCL	0.033679	0.04067	0.151298
GIGYF1	0.105389	0.040685	0.151328
LOC33980:	0.240439	0.040694	0.151337
RP5-1007H	-0.14898	0.040721	0.15141
TP73	-0.0104	0.04077	0.151568
TOMM70A	-0.13782	0.040796	0.151617
KCNMB2	0.03612	0.040802	0.151617
YTHDC2	0.218762	0.040805	0.151617
XBP1	-0.16563	0.040831	0.151687
P2RY1	-0.34115	0.040843	0.151697
NOXO1	-0.02159	0.040851	0.151697
ZFYVE1	0.149134	0.040854	0.151697
PDZK1IP1	0.559817	0.040889	0.151801
BTN3A2	0.263293	0.041083	0.152493
DPH2	-0.19023	0.041095	0.152513
FAM71F1	-0.04052	0.041111	0.152546
TTLL7-IT1	-0.06489	0.041142	0.152612
RP3-406A7	-0.2121	0.041143	0.152612
LOC10050(	-0.06304	0.041173	0.152699
ALG1	0.128114	0.041193	0.152745
FBF1	0.097922	0.041212	0.152791
GNB3	-0.09274	0.04124	0.152855
CADM3	-0.01617	0.041265	0.152855
TRMT5	0.151318	0.041271	0.152855
NCR2	-0.05704	0.041276	0.152855
FLRT3	0.201112	0.041276	0.152855
FYTTD1	0.11983	0.041277	0.152855
TAAR8	0.019507	0.041285	0.152855
LINC00924	0.011807	0.041286	0.152855
ACSL6	0.005347	0.041368	0.153131
FOXM1	0.38799	0.041415	0.153279
CREB1	0.186254	0.041423	0.153283
TRANK1	0.228159	0.041462	0.153402
NUTM2B	0.031103	0.041531	0.153631
CFL2	0.229812	0.041555	0.153692
STAMBPL1	0.238033	0.041595	0.153815

OLFML3	0.226237	0.041622	0.153887
CD177	0.25445	0.041675	0.154039
PNISR	-0.22893	0.041677	0.154039
PURA	0.274095	0.041691	0.154053
TMEM221	-0.0454	0.041695	0.154053
ATP6V0D2	0.586394	0.041721	0.154104
TFF2	-0.05945	0.041723	0.154104
WDR49	-0.27602	0.04173	0.154104
FZR1	0.071146	0.041738	0.154108
DMC1	-0.06441	0.041746	0.154108
KCTD1	0.100831	0.041763	0.154147
TSSK1B	-0.00954	0.041791	0.154203
MIER3	0.296059	0.041792	0.154203
FAM184B	-0.51021	0.041879	0.154498
KRT76	-0.0368	0.041898	0.154532
WRAP73	0.196881	0.041905	0.154532
MCTS1	-0.3208	0.04191	0.154532
PPP1R32	-0.03931	0.041999	0.154832
C11orf58	-0.14971	0.042016	0.154851
SPRYD4	0.226304	0.042018	0.154851
OR7E156P	0.066352	0.042033	0.154881
KIF3A	0.15242	0.042097	0.155065
DCDC2B	0.050088	0.042098	0.155065
FBXO36	0.026768	0.042119	0.155094
GRK6	0.142642	0.04212	0.155094
ATG4B	-0.15218	0.042156	0.1552
TTC39C	0.232456	0.042198	0.155327
LHFPL3-AS	-0.04069	0.042229	0.155383
BC039319	-0.02834	0.042232	0.155383
OGFOD2	-0.15015	0.042234	0.155383
SNX18	0.135248	0.042306	0.155608
ARPIN	0.141143	0.04231	0.155608
FLJ31945	0.008971	0.042322	0.155626
THEMIS	0.082604	0.042395	0.155841
SAR1B	0.323605	0.042401	0.155841
LOC155060	-0.19943	0.042402	0.155841
HAB1	-0.05546	0.042508	0.156204
C5orf15	-0.10798	0.042526	0.156243
MAP3K14	0.116739	0.042582	0.156424
LENEP	-0.04103	0.042673	0.156732
PXK	0.229663	0.042693	0.156779
CTD-3064I	-0.03029	0.042707	0.156804
MALRD1	-0.57295	0.042724	0.15684
HOMEZ	-0.05755	0.042758	0.156936
ZRANB2-A	0.027486	0.042785	0.15701
USP27X	-0.09127	0.042831	0.157105
LOC72806	-0.30552	0.042832	0.157105
LOC10106	-0.20045	0.042844	0.157105
LOC10050	-0.22195	0.042846	0.157105
SERPINI1	-0.4356	0.042848	0.157105
LINC00424	-0.02134	0.042855	0.157108
KLHL13	-0.43057	0.042874	0.157113
SYCP3	0.043999	0.042875	0.157113
LOC10050	-0.03969	0.042879	0.157113
PCDH17	0.384588	0.042933	0.15727
C14orf28	-0.20096	0.042943	0.15727
PNLIPRP3	0.011337	0.042943	0.15727
RP6-91H8.	-0.08267	0.04296	0.157294

ATP5I	0.215608	0.042964	0.157294
CDH17	0.012397	0.043058	0.157609
RNF41	-0.12829	0.0431	0.157739
FBL	-0.13888	0.043114	0.157762
APPBP2	-0.16399	0.043161	0.157879
LOC10192	0.125991	0.043166	0.157879
LANCL1	-0.11104	0.043168	0.157879
BC042374	0.011404	0.043202	0.15795
RHAG	0.506979	0.04321	0.15795
EXT2	0.150357	0.043214	0.15795
ZNF469	-0.21449	0.043217	0.15795
NRIP1	-0.29765	0.043224	0.15795
CLMN	0.130769	0.043263	0.158068
ANKRD36	-0.16483	0.043289	0.158135
DCAF4	-0.15537	0.043306	0.158171
RP11-313C	-0.07626	0.043327	0.158219
EDA2R	-0.07361	0.043371	0.158324
LINC00472	0.043459	0.043373	0.158324
WDSUB1	-0.13949	0.043379	0.158324
DLG5-AS1	-0.03811	0.043385	0.158324
TUBE1	-0.15784	0.043411	0.158393
APEX2	0.162873	0.043436	0.15845
ERN2	-0.06512	0.043444	0.15845
TRAK1	-0.19513	0.043455	0.15845
ABCA12	0.015457	0.043456	0.15845
STAMBP	-0.17368	0.043494	0.158563
PLA2G4A	0.204233	0.04351	0.158593
NR2C2AP	-0.23987	0.043524	0.158618
FLJ38379	-0.51055	0.043545	0.158668
C1orf216	-0.25548	0.043555	0.158679
ZMYND15	0.070466	0.043623	0.1589
AHNAK2	-0.02211	0.043635	0.158916
TIMM9	-0.13608	0.04367	0.159016
MEN1	0.253418	0.043681	0.159016
RP11-410E	-0.02627	0.043685	0.159016
LY9	0.161519	0.043707	0.159073
ZNF302	-0.30808	0.043724	0.159108
TSC22D3	0.199959	0.043765	0.15923
UBAC2	0.161913	0.043822	0.159388
L3MBTL3	-0.24978	0.043824	0.159388
LOC10050	0.09321	0.043842	0.159428
NUP62CL	0.180071	0.043887	0.159551
NKIRAS1	0.252184	0.04389	0.159551
LINC00032	-0.01774	0.043966	0.159799
SND1	-0.13437	0.043975	0.159806
SNX16	-0.24363	0.044021	0.159936
CEP83	-0.21508	0.044026	0.159936
UGGT1	0.270056	0.044041	0.159966
MRPL3	-0.12684	0.044057	0.159996
HIST1H1C	0.412088	0.044068	0.160001
RP1-190J2	-0.0193	0.044073	0.160001
OLFM4	0.469638	0.044096	0.160057
ZBTB47	0.080062	0.044106	0.160066
FAM110A	0.168738	0.044147	0.160174
SYNRG	0.14557	0.044152	0.160174
GPR116	0.303434	0.044164	0.160174
DHRS13	0.241795	0.044165	0.160174
PGBD1	0.224538	0.044185	0.160218

IRF1	0.336613	0.044247	0.160418
EFCAB12	-0.03303	0.04427	0.160475
DNAJB2	0.278389	0.044301	0.160536
IL27	-0.03027	0.044302	0.160536
KRTAP5-8	-0.0832	0.044351	0.160686
TOPORS-A	0.172684	0.044385	0.16077
KCNJ14	-0.04136	0.044389	0.16077
BIK	0.246556	0.044403	0.160796
FBXW2	0.173486	0.044428	0.160837
PIFO	-0.03738	0.04443	0.160837
HIPK1-AS1	-0.0955	0.044439	0.160844
SNRPD1	-0.14184	0.044474	0.160944
CACUL1	0.084989	0.044522	0.161091
HEATR1	0.150415	0.044586	0.161294
USP48	-0.18396	0.044616	0.161376
INTS2	-0.20325	0.044759	0.161852
MTBP	-0.01923	0.044769	0.161852
MTCP1	-0.15494	0.04477	0.161852
IER5L	0.085437	0.04482	0.162002
IFT52	0.125572	0.044826	0.162002
ANKRD26	-0.15688	0.044917	0.162301
TRPV2	-0.11694	0.045033	0.162688
VKORC1	0.209155	0.045041	0.162688
CLK4	-0.261	0.045046	0.162688
PTRH1	0.125748	0.045075	0.162765
TNKS2	-0.17172	0.045185	0.163135
SHBG	-0.05068	0.04527	0.163388
PAQR7	-0.19691	0.045277	0.163388
TNKS1BP1	0.073618	0.045278	0.163388
FAM43A	0.172172	0.045287	0.163395
GAS2L1	0.391956	0.045324	0.163501
INMT	0.118445	0.04534	0.163531
PTCD2	-0.1871	0.045365	0.163592
TMEM104	-0.26821	0.04541	0.163728
NGB	-0.03392	0.045423	0.163747
DHRS7B	0.220079	0.045496	0.163984
ZNF331	0.291036	0.045608	0.164339
CEACAM5	-0.02044	0.04561	0.164339
PAX6	-0.0186	0.045617	0.164339
MAG	-0.03244	0.045659	0.164445
IKBKAP	-0.09472	0.045681	0.164445
USP2-AS1	-0.07296	0.045686	0.164445
LOC28550(	0.09911	0.04569	0.164445
SGCG	0.239152	0.045691	0.164445
TOR1A	0.169566	0.045692	0.164445
SPTLC1	0.179848	0.045703	0.164456
LOC64551:	-0.17733	0.045729	0.164522
KLHL15	-0.2199	0.045753	0.164582
MAP3K4	-0.12296	0.045761	0.164583
JOSD1	0.18348	0.045824	0.164781
SUGP2	-0.17903	0.045867	0.164908
FGR	0.484263	0.045888	0.164957
DDC-AS1	0.012077	0.045903	0.164985
UBE2D4	0.123671	0.045911	0.164985
ZNF701	-0.08459	0.045989	0.165239
PTPN4	-0.12237	0.046017	0.165305
NCSTN	0.103691	0.046023	0.165305
KCNN2	0.013394	0.046057	0.165354

ZNF404	-0.39619	0.046061	0.165354
ALG2	-0.11732	0.046062	0.165354
SLC4A3	0.068667	0.046067	0.165354
XCL1	0.033424	0.04609	0.16541
RHNO1	0.173218	0.046137	0.165552
DHX40	-0.11247	0.04619	0.165714
CD160	0.092066	0.04621	0.165727
IDI1	-0.16038	0.046211	0.165727
AX747261	-0.04336	0.046217	0.165727
CWC27	0.146224	0.04627	0.165872
RP11-452L	-0.27433	0.046273	0.165872
DNTTIP2	-0.1352	0.0463	0.165943
CHI3L2	0.191175	0.046311	0.165956
IGFBP1	-0.00971	0.04634	0.166033
CA8	0.101037	0.046352	0.166036
RP11-305C	-0.48049	0.046357	0.166036
FAM65C	0.218913	0.046371	0.166059
C1orf233	-0.10569	0.046494	0.166446
TUG1	-0.18294	0.046501	0.166446
NUP50-AS	0.185577	0.046502	0.166446
ITGA9-AS1	-0.05608	0.04654	0.166557
STK3	-0.08041	0.046567	0.166623
AURKAIP1	-0.06121	0.046637	0.166847
GLTSCR1	-0.12473	0.046651	0.166871
MSH2	-0.17411	0.046668	0.166903
GYLTL1B	0.092327	0.04668	0.166909
LINC01115	-0.06525	0.046685	0.166909
ABCC4	0.338452	0.046735	0.167061
RAB12	0.204723	0.046749	0.167067
CASP8	0.185173	0.046752	0.167067
KIAA0895	-0.23251	0.046893	0.167542
LOC10192	-0.29091	0.046968	0.167782
RP4-593H1	-0.04926	0.047056	0.168068
ARPC3	-0.17767	0.047091	0.168158
CYHR1	0.094177	0.047097	0.168158
PREPL	0.212115	0.047147	0.168311
TMEM171	0.013258	0.047231	0.168581
MUC20	0.09991	0.047377	0.169056
WDR34	0.270645	0.047379	0.169056
SLC35B1	0.182777	0.047417	0.169136
RP11-998D	-0.02963	0.047424	0.169136
SRCAP	0.023319	0.047427	0.169136
RP11-355E	-0.32803	0.047441	0.169136
RNF126P1	0.104794	0.047441	0.169136
LINC00652	-0.04454	0.047525	0.169408
CCDC167	0.256424	0.047558	0.169499
DSCAM	0.028203	0.047625	0.169708
ZMIZ1-AS1	0.017787	0.047649	0.169768
VCX2	-0.0546	0.047704	0.169934
GDI1	0.187604	0.047732	0.170007
ERMARD	-0.16625	0.047783	0.170133
CDK15	-0.02614	0.047785	0.170133
MYO1A	0.025527	0.047791	0.170133
CXorf21	-0.26236	0.047863	0.170334
RASSF10	0.006036	0.047864	0.170334
GRIN3B	-0.01484	0.047878	0.170359
LOC10050	-0.04188	0.047965	0.170639
BGLT3	-0.08948	0.047981	0.170669

TIMP4	0.128789	0.048102	0.171069
TMC5	0.036085	0.048129	0.171136
EP400	0.186418	0.048136	0.171136
HCG22	-0.04496	0.048146	0.171144
CDH5	0.32747	0.048178	0.171211
RRBP1	0.05594	0.048182	0.171211
FGD5-AS1	0.078791	0.048189	0.171211
HSPB6	-0.05765	0.048216	0.17128
DDX28	-0.10373	0.048242	0.171345
NUBPL	-0.14456	0.048253	0.171353
VN1R1	0.049737	0.048265	0.171358
CCDC121	-0.13728	0.04827	0.171358
LINC01142	-0.02276	0.048295	0.171421
NTSR1	0.024705	0.048305	0.171426
MON1B	0.187778	0.048327	0.171476
ZDHHC24	0.196126	0.048348	0.17151
INVS	-0.15046	0.048355	0.17151
SNORA72	0.288356	0.04836	0.17151
CETN3	-0.25748	0.04844	0.171765
DPY19L3	-0.16439	0.048461	0.171812
OCIAD2	-0.25586	0.048504	0.171937
MLF2	0.181711	0.048514	0.171942
CCDC30	-0.03351	0.048523	0.171947
GINS3	-0.25178	0.04858	0.17212
TRMT11	-0.21611	0.048657	0.172361
1-Sep	0.193046	0.048664	0.172361
RNASEL	0.158509	0.048689	0.172396
ANKIB1	0.193015	0.04869	0.172396
PRG4	0.209906	0.048713	0.172449
PFDN1	0.155544	0.048751	0.172532
SNN	0.262441	0.048752	0.172532
IDH1-AS1	0.101667	0.048795	0.172655
ATXN10	0.125189	0.048806	0.172668
ZNF613	-0.18481	0.048867	0.172854
PGAM5	0.085586	0.04895	0.173121
ADK	-0.1994	0.048976	0.173185
PEX26	-0.07377	0.049033	0.173352
SLC25A6	-0.16563	0.04904	0.173352
PAGE5	0.034319	0.049064	0.173409
LOC10050!	-0.06043	0.04909	0.173474
TSPAN15	0.117908	0.049159	0.173666
CPNE6	-0.01829	0.049168	0.173666
TRAPPC2	-0.08102	0.049172	0.173666
FRAT2	0.204572	0.049177	0.173666
PTGR2	-0.23843	0.049195	0.173702
TMEM109	0.192828	0.049212	0.173735
CACNG1	-0.04	0.049224	0.173748
ASB12	-0.22367	0.049263	0.173856
AGPAT9	-0.50742	0.049309	0.173992
GOLGA8N	-0.20988	0.049362	0.174149
ACTR2	0.227896	0.049417	0.174315
LOC10192!	-0.03289	0.049495	0.174562
CLEC5A	0.341406	0.049508	0.17458
FOXP1	-0.16682	0.049522	0.174584
PBX3	0.311667	0.049525	0.174584
BRWD1-IT.	-0.09136	0.049536	0.174593
BEND2	-0.32574	0.049581	0.174723
LOC10012!	-0.2699	0.049613	0.174807

LOC10192!	-0.05153	0.049653	0.174921
PPP2R5C	0.177587	0.049661	0.174921
ZNF625	-0.06483	0.04971	0.175066
PACSIN2	0.167126	0.049737	0.17513
CDC25B	0.265353	0.049757	0.175174
ALG13	-0.25791	0.049784	0.175216
C14orf182	-0.02777	0.049792	0.175216
KCNN4	0.262826	0.049794	0.175216
LOC14869(	0.017202	0.049813	0.175257
CSF3	-0.06337	0.049845	0.17534
TM9SF4	0.174067	0.049934	0.175626
MRPL45P2	-0.03377	0.050031	0.175924
LOC10192	0.024844	0.050035	0.175924
RBM28	-0.14375	0.050059	0.175979
SEPSECS-/	-0.16861	0.05008	0.176025
TBL1XR1	0.182252	0.050096	0.176052
DHX9	-0.3079	0.050114	0.176086
WDFY4	0.10348	0.050171	0.176259
SSB	0.210689	0.05019	0.176282
QTRTD1	-0.08851	0.050194	0.176282
N4BP2L1	0.197493	0.050209	0.176306
TRIM4	0.187386	0.050221	0.176313
LHX4-AS1	-0.23947	0.050227	0.176313
ZNHIT6	-0.11373	0.050301	0.176543
RP11-480D	-0.04395	0.050327	0.176591
C1GALT1C	-0.12016	0.050338	0.176591
LINC00982	0.158112	0.050347	0.176591
RP11-457I.	-0.05063	0.050348	0.176591
SEMA3C	-0.28743	0.050357	0.176594
EMP3	0.408491	0.050371	0.176617
CHST3	0.019602	0.050384	0.176632
CRYGEP	-0.12475	0.050518	0.177058
FAM174B	0.242394	0.050521	0.177058
INHA	0.003131	0.050533	0.17707
PRR19	-0.08151	0.050558	0.177127
LOC10192	-0.0465	0.050575	0.177135
ZNF226	-0.10771	0.050584	0.177135
CDH3	0.021175	0.050585	0.177135
CASC7	-0.27102	0.050592	0.177135
NFIB	0.281123	0.050616	0.17719
GSDMD	0.155239	0.050625	0.177191
BCL9	0.092372	0.050686	0.177377
BLOC1S4	0.150375	0.050703	0.177408
LINC00886	-0.11907	0.050755	0.177561
TRIT1	-0.15085	0.050829	0.177791
NAA25	-0.19506	0.05096	0.178191
OTX2	0.007647	0.050961	0.178191
VWA5B2	-0.01614	0.050968	0.178191
RFX7	-0.15417	0.050981	0.178207
SZT2	0.142203	0.050999	0.178243
ARHGEF7-	0.011392	0.05101	0.17825
AP006216.	-0.04566	0.051025	0.178274
FBXW5	0.184039	0.051058	0.178361
MBD2	0.018593	0.051095	0.178462
TAF3	0.122041	0.051129	0.178551
TBPL1	0.191736	0.051143	0.178571
HCFC2	0.145082	0.051165	0.178619
LIN54	-0.0914	0.051189	0.178668

LAIR1	0.265644	0.051195	0.178668
UIMC1	-0.1398	0.051205	0.178672
NES	-0.0338	0.05126	0.178836
AP000462.	-0.01411	0.051313	0.178992
FNDC8	-0.03709	0.051372	0.179169
NECAP2	0.154273	0.051382	0.179171
SLC39A3	0.239551	0.051389	0.179171
LINC00968	0.091589	0.051411	0.179219
VGF	-0.04134	0.051465	0.179378
GCC2	-0.08289	0.051595	0.179803
C8orf58	0.084948	0.051608	0.179815
VWA3A	-0.06539	0.051615	0.179815
IREB2	-0.22585	0.051672	0.179983
FSD1L	-0.15383	0.051708	0.180081
COQ5	0.154408	0.051722	0.180099
EPM2AIP1	-0.24343	0.051766	0.180222
ZNF286A	-0.12494	0.051779	0.18024
TMEM126A	0.163004	0.051818	0.180319
VPS33B	-0.14173	0.05182	0.180319
RP4-539M	-0.0239	0.051833	0.180319
TCERG1	-0.41058	0.051835	0.180319
RP3-368A4	-0.39696	0.051851	0.180324
SMYD2	0.148163	0.051853	0.180324
ZNF330	0.094497	0.051879	0.180386
KIT	-0.31917	0.051906	0.180451
NHLH1	-0.04042	0.051931	0.180464
NBN	-0.17432	0.051941	0.180464
SGPP2	-0.02552	0.051944	0.180464
DTYMK	-0.1448	0.051949	0.180464
BRICD5	0.040763	0.051952	0.180464
SLC46A3	0.229639	0.051965	0.18048
HS3ST1	0.112611	0.051994	0.180554
RP11-384L	-0.06168	0.052008	0.180572
BTG1	0.263827	0.052046	0.180675
HIST1H2BN	0.026942	0.052077	0.180735
STRN3	0.183685	0.05208	0.180735
ISLR	0.037505	0.052103	0.180788
XCR1	-0.00605	0.052142	0.180892
DOK1	-0.14784	0.052174	0.180974
FRMD6-AS	-0.0873	0.052182	0.180974
OR2L1P	0.010559	0.052211	0.181033
ZNF256	-0.26664	0.052216	0.181033
MSS51	0.14469	0.052234	0.181068
RP11-343H	-0.07097	0.052251	0.181099
DAB2	0.137831	0.052272	0.181142
SETD5	0.190665	0.052285	0.181155
TPO	0.019176	0.052311	0.181177
TRIM62	-0.09739	0.052315	0.181177
CFI	0.393977	0.052316	0.181177
NSUN2	-0.13233	0.05238	0.181371
RP11-953B	-0.0407	0.052393	0.181388
ANKRD46	-0.283	0.05243	0.181485
RP11-77H9	0.030137	0.052439	0.181488
ACTR1B	0.125004	0.052496	0.181655
COPZ1	0.155025	0.052524	0.181712
LZIC	0.132898	0.052529	0.181712
ZNF211	-0.18669	0.052556	0.181772
TMEM178B	-0.02569	0.052568	0.181772

ZNF225	-0.12939	0.052572	0.181772
UFC1	-0.1138	0.052629	0.18194
DDX27	-0.15957	0.052653	0.181997
RP11-259(	0.005569	0.052681	0.182063
SCGB2B2	-0.09084	0.052755	0.182279
CRISPLD2	0.163978	0.052773	0.182279
SPP2	-0.03288	0.052777	0.182279
MRPL14	0.213617	0.052777	0.182279
OR7E104P	-0.04534	0.052799	0.182302
DCX	0.006726	0.052801	0.182302
ZNF552	0.054936	0.052818	0.18233
SLC25A44	0.099348	0.052826	0.18233
CARD11	-0.10632	0.052866	0.18244
DTX3	0.006908	0.052886	0.182462
SNX5	0.157792	0.052895	0.182462
TMEM208	0.232745	0.052898	0.182462
LDLRAD3	0.413001	0.05297	0.182683
KLK7	-0.04541	0.053067	0.182923
HCCS	0.187875	0.05307	0.182923
LOC10192!	-0.06774	0.053074	0.182923
MSANTD4	-0.04918	0.05308	0.182923
NUDT16L1	-0.18562	0.053082	0.182923
SLC30A5	0.133593	0.053103	0.182966
KLRC3	0.081577	0.053144	0.18308
RAN	0.099416	0.053188	0.183089
GGACT	0.074143	0.05319	0.183089
ERP29	-0.19184	0.053191	0.183089
SMURF2	-0.24272	0.053195	0.183089
LOC10192(	0.058656	0.053196	0.183089
FAM126B	0.193992	0.053198	0.183089
LOC28563!	0.02198	0.053242	0.183214
TOMM34	0.156455	0.053293	0.18336
DUSP10	0.365633	0.053345	0.183478
DLG3	-0.02597	0.053351	0.183478
ABHD2	0.15519	0.053357	0.183478
KCNQ1DN	-0.06992	0.053365	0.183478
EBLN2	-0.26018	0.053375	0.183478
REEP1	0.354408	0.053383	0.183478
RP11-664[	-0.0437	0.053387	0.183478
ASAP3	-0.03049	0.053396	0.183479
NAGPA	0.20024	0.053416	0.183518
TAF5L	0.123635	0.053461	0.183644
CHCHD7	0.230083	0.053479	0.183679
LINC00326	-0.0347	0.053542	0.183866
LOC25739(	-0.23767	0.053556	0.183883
ALDH16A1	0.202063	0.053572	0.183894
CPN2	-0.06297	0.053583	0.183894
FBXO30	0.153691	0.053584	0.183894
C11orf54	0.202472	0.05363	0.184023
AMT	-0.28	0.053642	0.184034
DIO2	0.008785	0.053652	0.184034
RP11-722E	-0.22758	0.053659	0.184034
LPAR4	-0.35375	0.053823	0.184568
RP11-549J	-0.17447	0.053853	0.184626
DEFA4	0.689015	0.05386	0.184626
AZI2	-0.15673	0.053866	0.184626
LOC91548	0.097671	0.053883	0.184656
DVL3	0.090967	0.053904	0.184698

TM9SF2	0.111771	0.053932	0.184765
GDPD2	-0.03805	0.053983	0.184909
KCNC3	0.040793	0.05402	0.185007
PAAF1	-0.15782	0.054049	0.185017
USP53	-0.18018	0.054054	0.185017
ZNF586	-0.09832	0.054057	0.185017
LRRC58	-0.08567	0.054064	0.185017
NR2F2-AS1	-0.01278	0.054066	0.185017
GABPA	-0.12774	0.054113	0.185148
ZNF585A	-0.17264	0.054121	0.185148
NKPD1	-0.04239	0.054182	0.185328
ECD	0.150281	0.054345	0.185855
UBE2T	0.382128	0.054493	0.186332
CA11	-0.13404	0.054565	0.18655
PTMS	0.075933	0.054582	0.186579
MPPE1	-0.14669	0.054628	0.186677
LOC10192	0.003924	0.054628	0.186677
VRK2	0.161763	0.054641	0.186692
LOC64621	-0.17332	0.054674	0.186776
TLE1	0.369231	0.054782	0.187086
LINC00919	0.010238	0.054784	0.187086
INF2	0.088248	0.054791	0.187086
NAA15	0.154724	0.054829	0.187187
GMPS	-0.19272	0.05495	0.187551
KRBOX4	-0.14368	0.054953	0.187551
MINK1	0.063235	0.054971	0.187583
TEAD1	0.013036	0.054992	0.187625
PRMT6	0.253895	0.055017	0.187681
IGSF10	-0.49442	0.055032	0.187702
SIAH3	0.068642	0.055059	0.187764
CNN2	0.167118	0.055083	0.187816
IGSF1	-0.02577	0.055227	0.188265
HTR1F	-0.28361	0.055234	0.188265
CPAMD8	0.121009	0.055241	0.188265
ASPDH	-0.12451	0.05528	0.188371
LIPG	0.040761	0.055305	0.188427
LOC28493	-0.03341	0.055323	0.188456
TEX22	-0.06566	0.055348	0.188512
SPRR4	-0.06457	0.05537	0.188558
COA1	-0.16521	0.055398	0.188622
AC005523.	-0.05901	0.055408	0.188629
RP4-539M	0.00714	0.055518	0.188972
C1orf54	0.434688	0.055533	0.188994
ITGA2B	0.488467	0.055547	0.189011
LOC10192	0.004901	0.055563	0.189038
GABRA1	0.045709	0.055595	0.189101
BEND5	0.144812	0.055599	0.189101
SLIT2	0.04191	0.05565	0.189243
GCHFR	0.212465	0.05571	0.18939
DPPA4	-0.44051	0.055711	0.18939
SGCE	0.152868	0.055726	0.189413
ILKAP	-0.18644	0.055743	0.189436
CLDN23	0.024457	0.05575	0.189436
GABBR2	-0.00832	0.055771	0.189465
VPS35	0.151683	0.055776	0.189465
PKMYT1	0.194764	0.055823	0.189592
TMEM239	-0.03175	0.055854	0.189669
TSC22D1	-0.19357	0.055889	0.18973

REEP5	0.272601	0.05589	0.18973
LGALS2	0.370049	0.055903	0.189746
MTMR4	-0.12659	0.055926	0.189795
PARD3-AS	0.020527	0.05594	0.189812
LOC44117	-0.01474	0.055978	0.189906
ANXA7	0.102951	0.055985	0.189906
PFKFB3	0.356854	0.056073	0.190174
LINC00280	-0.04087	0.056082	0.190174
PRPSAP2	-0.11224	0.056152	0.190377
RP11-298H	-0.016	0.056159	0.190377
BBS1	-0.02535	0.056186	0.190439
POLN	-0.04124	0.0562	0.190455
UBOX5	-0.10674	0.056217	0.190484
CSN2	-0.059	0.056248	0.19056
AC005162.	-0.05501	0.056273	0.190614
C15orf65	0.138333	0.05629	0.190643
SLC25A21-	-0.18971	0.056306	0.190667
OGFRL1	0.189934	0.056373	0.190864
ZC3H10	-0.10226	0.056485	0.191211
ACTR3B	-0.11033	0.056498	0.191226
RP11-805I	-0.09517	0.056597	0.191533
MITD1	0.241872	0.056624	0.191593
LINC01220	0.033453	0.056663	0.191683
TMEM207	-0.02299	0.056668	0.191683
HOTS	0.042108	0.0567	0.191727
PATE1	-0.031	0.056702	0.191727
NGDN	-0.11657	0.056715	0.191727
AC091633.	-0.03721	0.056717	0.191727
LOC73010	-0.31449	0.056739	0.191771
KANSL1L	-0.28221	0.056756	0.191799
CUTA	-0.1313	0.056869	0.192152
OLFM1	0.053234	0.056883	0.19217
ETHE1	0.239479	0.056934	0.19231
PSMB8-AS	0.109883	0.056952	0.192341
USP21	0.224961	0.056977	0.192395
SNX7	0.499834	0.056995	0.192426
TGM5	-0.07095	0.057014	0.192461
NTN3	-0.04034	0.057026	0.192471
WHSC1	-0.1944	0.05709	0.192657
NDC80	-0.28623	0.057114	0.19271
GBA2	0.105804	0.057123	0.19271
SURF6	-0.19674	0.057132	0.192711
LOC10192	-0.02213	0.05721	0.192944
TARBP2	0.174806	0.057268	0.193109
CCDC27	-0.01978	0.057291	0.193138
DOK5	0.097629	0.057309	0.193138
ODF4	-0.03974	0.057311	0.193138
PSMD2	-0.13605	0.057318	0.193138
AADACP1	0.019015	0.057322	0.193138
MTX2	0.177068	0.057351	0.193192
CACHD1	-0.19385	0.057355	0.193192
DDX25	-0.2024	0.057397	0.193302
SLC25A16	0.137439	0.057428	0.193375
ZNF268	0.219938	0.057441	0.193391
HIVEP2	-0.25957	0.05746	0.193424
LOC10050	-0.06272	0.057474	0.19344
IGFL2	0.036716	0.057483	0.193443
CES3	0.053181	0.0575	0.193468

EIF3J	-0.09536	0.05751	0.193473
GRK4	-0.10471	0.057525	0.193492
7-Mar	0.302319	0.057619	0.193762
GNAI1	-0.41403	0.057623	0.193762
SLC26A3	0.005174	0.057734	0.194107
HIST1H2BH	0.386667	0.057833	0.194407
SP4	-0.13297	0.057899	0.1946
XKR6	0.06812	0.057909	0.194602
MB21D1	-0.23457	0.057923	0.194621
LOC10192!	-0.04146	0.057941	0.19465
PP12719	-0.15503	0.057957	0.194674
CLDN19	-0.04792	0.058035	0.194907
PLA2G12A	-0.22041	0.058051	0.194927
CLDN7	0.057427	0.058081	0.194977
PRKACG	-0.04522	0.058083	0.194977
IRF2BPL	-0.19055	0.058102	0.19501
AIFM3	0.062002	0.058112	0.195013
ZKSCAN2	0.179021	0.058144	0.19509
ACTR3C	-0.08387	0.058157	0.195103
GADD45A	0.198961	0.058207	0.19524
RAMP1	0.295801	0.058296	0.195507
MAL	0.55414	0.058304	0.195507
FMR1	0.271756	0.058365	0.195679
C16orf80	-0.16824	0.058393	0.195731
NTMT1	0.243054	0.058409	0.195731
PRMT3	-0.1744	0.058414	0.195731
MIR100HG	0.222577	0.058416	0.195731
CCDC96	0.050094	0.058483	0.195925
MICU1	0.196768	0.05854	0.196085
CNP	0.074451	0.058571	0.196157
ZNF407	-0.02019	0.058593	0.196202
SLIT1	-0.04164	0.058629	0.196245
ZBTB45	0.08325	0.058635	0.196245
UNC5CL	-0.18948	0.058638	0.196245
UNC119B	-0.0763	0.058642	0.196245
C6orf203	0.198634	0.058667	0.196297
CALB2	-0.03356	0.058701	0.19638
C11orf80	-0.15893	0.058717	0.196404
CNEP1R1	0.169824	0.058766	0.196537
AP1S3	0.024323	0.05879	0.196584
VARS2	-0.18361	0.058798	0.196584
BRF1	-0.05082	0.058864	0.196774
CC2D2A	0.013193	0.058944	0.197012
CERS5	-0.0823	0.058975	0.197084
GADD45B	0.429085	0.058989	0.197099
SLC7A11	0.176887	0.059033	0.197214
NAE1	-0.17869	0.059051	0.197214
CNTNAP3F	0.027952	0.059054	0.197214
HNF1B	-0.05376	0.05906	0.197214
MAML2	0.20261	0.05907	0.197219
KCNE3	0.218898	0.059093	0.197248
LOC10192!	0.002905	0.059104	0.197248
CHRNE	-0.03082	0.059106	0.197248
SLC27A5	0.142262	0.059125	0.197272
MYO5C	-0.42745	0.059138	0.197272
LRCH3	-0.31442	0.059141	0.197272
LOC10013:	-0.15583	0.059159	0.197305
MYL6	0.12469	0.059208	0.197435

MGEA5	0.182694	0.059232	0.197484
C14orf64	-0.02844	0.059252	0.197521
TCF15	-0.2258	0.059275	0.197567
SLC2A3	0.395858	0.059304	0.197633
WNT10A	0.009117	0.059339	0.197721
LOC40132	0.005286	0.059362	0.197767
IGH	-0.06417	0.059389	0.197825
LOC37519	0.036764	0.059399	0.197828
OR7A10	-0.06697	0.059414	0.197848
LINC01087	-0.01803	0.059433	0.197882
WDR18	0.215809	0.059478	0.198
KLRG2	0.119556	0.059495	0.198028
AKTIP	0.144767	0.059546	0.198167
NAPSA	-0.04762	0.059569	0.198188
AC114730.	-0.01319	0.059571	0.198188
TTC30B	-0.14888	0.059594	0.198235
DUSP15	-0.04969	0.059639	0.198355
ADIRF	0.056996	0.059652	0.198368
NFS1	0.105051	0.059714	0.198534
NECAB2	-0.05867	0.059725	0.198534
FOXRED1	0.146648	0.05973	0.198534
CXCL11	0.246357	0.059782	0.198678
BCL2	-0.12501	0.059822	0.198778
CLN3	0.1887	0.059858	0.19887
CASS4	-0.11419	0.059887	0.198936
LOC10260	-0.18583	0.059917	0.199003
EMILIN1	0.369723	0.059934	0.199028
WDR5B	-0.25823	0.060022	0.19929
SNX3	0.090702	0.060054	0.199368
AMN1	-0.22999	0.060065	0.199374
HELQ	0.150286	0.060099	0.199455
ENOX1	0.107967	0.060167	0.19965
NGF	-0.06627	0.060181	0.199663
SCN2A	-0.16611	0.06019	0.199663
LOC28605	-0.25771	0.060198	0.199663
RUVBL2	0.229173	0.060267	0.199859
PLAC8L1	-0.07756	0.06028	0.199872
DEF6	0.152331	0.060306	0.199916
AZU1	0.734297	0.060316	0.199916
PGLYRP1	0.315897	0.060323	0.199916
LOC10013	-0.03265	0.06033	0.199916
TCF12	-0.16622	0.060382	0.200056
CIR1	0.15847	0.060391	0.200056
TOX	-0.25648	0.060409	0.200083
SLMAP	-0.10733	0.060417	0.200083
CROCC	-0.07393	0.060428	0.200087
NFATC2IP	0.119417	0.060449	0.200113
PRSS54	0.00928	0.060454	0.200113
LOC10192	0.033439	0.060549	0.200395
ZNF554	-0.06352	0.06057	0.200433
RP3-329A5	-0.09721	0.060614	0.20055
CXorf57	-0.26002	0.060672	0.20071
HTR6	-0.02283	0.060688	0.200735
RPS25	-0.10867	0.060718	0.200761
U91328.2	-0.16373	0.060723	0.200761
APOA4	-0.05518	0.060724	0.200761
GTPBP2	0.13349	0.06075	0.200817
MIRLET7DI	-0.20141	0.06081	0.200984

ZDHHC8P1	0.038508	0.06087	0.201131
NAT8L	-0.0774	0.060877	0.201131
GPR78	-0.04865	0.06089	0.201131
DXO	0.149135	0.060892	0.201131
LINC00348	-0.02483	0.060902	0.201133
RECQL5	0.073262	0.061002	0.201435
DPYSL5	-0.01566	0.061017	0.201453
GPN1	-0.12845	0.061074	0.201609
KIAA1045	-0.02452	0.061129	0.20176
MYO1F	0.218991	0.061159	0.201829
OIP5	0.304453	0.061172	0.201842
JPX	0.427594	0.061217	0.201959
MKL2	-0.04116	0.061257	0.202028
AP001189.	-0.19337	0.061266	0.202028
C15orf48	0.406151	0.061274	0.202028
DYM	-0.1902	0.061275	0.202028
F5	0.205348	0.061356	0.202238
CHST4	-0.06003	0.061358	0.202238
ZNF483	-0.01182	0.061401	0.20235
ANKRD53	-0.03467	0.061418	0.202373
DERA	0.145389	0.061487	0.202571
LOC10050(	0.02625	0.061529	0.202678
C3orf14	0.325948	0.061551	0.202696
SRGAP1	0.01408	0.061555	0.202696
C3orf38	-0.22339	0.061562	0.202696
HMGB2	-0.06174	0.061624	0.20287
PDP2	-0.13468	0.061702	0.203096
DDX55	0.207751	0.06175	0.20322
P4HTM	-0.17279	0.06177	0.203256
LDB1	0.225895	0.061795	0.203307
TRPM2	0.150609	0.061837	0.203413
SUOX	0.155178	0.061855	0.203442
SH3D21	-0.22801	0.061885	0.20351
TBL3	0.114781	0.061911	0.203564
TNFRSF17	0.37351	0.061933	0.203583
VCL	-0.13253	0.061935	0.203583
MLH1	-0.12814	0.061956	0.203622
SKIL	-0.31243	0.062026	0.20382
ZNF202	-0.15939	0.06208	0.203965
LINC00643	0.004098	0.062189	0.204274
RP11-506N	0.005289	0.062193	0.204274
CDH15	-0.02932	0.062203	0.204277
TPM2	0.052905	0.062261	0.204437
COL8A2	0.217383	0.06229	0.204477
RHOH	-0.31581	0.062304	0.204477
MCHR2	-0.03608	0.062309	0.204477
RP11-203E	-0.02295	0.062311	0.204477
FSIP1	-0.02599	0.062324	0.204487
ZNF136	-0.23543	0.062356	0.204564
CEND1	-0.03466	0.062371	0.204581
PLD2	0.103436	0.062403	0.204654
C14orf166	-0.07035	0.062457	0.204802
OAZ2	0.228276	0.062503	0.20492
INPPL1	0.110549	0.062514	0.204927
ZNF527	-0.06713	0.062565	0.205063
NSG1	0.053934	0.062619	0.205168
KNTC1	-0.21515	0.062627	0.205168
ZDHHC23	-0.3143	0.062632	0.205168

SLC28A3	-0.08183	0.062635	0.205168
FFAR2	0.149841	0.062675	0.205268
FOS	0.363655	0.062695	0.205301
HCG11	0.281889	0.062753	0.205462
SLC16A6	0.227136	0.062802	0.205565
UBTD2	-0.18927	0.062804	0.205565
TMEM184/	-0.03969	0.062841	0.205655
CDAN1	0.091046	0.062924	0.205895
MT1HL1	0.506283	0.063034	0.206224
DARS2	0.178418	0.063089	0.206373
CCDC7	-0.09722	0.063152	0.206492
SNIP1	0.123539	0.063153	0.206492
PMS2P4	-0.03741	0.063154	0.206492
DZANK1	-0.10686	0.063212	0.206651
LOC10099(	-0.37794	0.063221	0.206651
SPDEF	-0.02685	0.063246	0.2067
SEMA4A	0.147493	0.063291	0.206816
GAL3ST2	-0.04779	0.063314	0.206861
KLHL26	0.114186	0.063325	0.206864
RGAG1	-0.03897	0.063373	0.20699
TDH	0.012821	0.063385	0.206999
KRTAP19-:	0.004565	0.063432	0.20712
VEZT	-0.15923	0.063474	0.207195
SCAND2P	0.023541	0.063474	0.207195
RNF187	0.003689	0.063539	0.207375
CTCFL	-0.02703	0.063568	0.207428
HLA-DRB6	0.092515	0.063574	0.207428
TSFM	-0.19954	0.06364	0.207602
TRIM25	0.114724	0.063655	0.207602
A2ML1	-0.02404	0.063656	0.207602
PLAT	0.348502	0.063777	0.207964
PLCG1-AS.	0.024847	0.063823	0.208083
LINC01091	0.023668	0.063876	0.208226
LOC10012(	0.118744	0.063895	0.208226
CLEC4M	0.158367	0.063904	0.208226
APOL4	0.125788	0.063905	0.208226
SLC25A28	0.259613	0.063971	0.208408
FBLN1	0.080083	0.064033	0.208561
PIKFYVE	-0.17641	0.064037	0.208561
LOC10028:	0.004326	0.064059	0.2086
KAT6B	0.190481	0.064101	0.208706
LINC00566	-0.03554	0.064125	0.208722
MYL4	0.343521	0.064128	0.208722
PEX1	-0.14094	0.064135	0.208722
SPACA3	0.010523	0.064183	0.208849
LOC10050!	-0.03848	0.064196	0.208858
SLC25A5-/	-0.05351	0.06421	0.208867
CTSF	0.288708	0.064218	0.208867
TSPYL5	0.447006	0.06425	0.208911
SPP1	0.207784	0.064251	0.208911
IGLJ3	0.375807	0.064283	0.208985
DNAJB8	-0.02657	0.064304	0.209015
CXADR	0.389719	0.064311	0.209015
DDB2	-0.20095	0.064348	0.209102
EBF4	-0.04678	0.064375	0.209158
SPAG8	-0.05249	0.064395	0.20919
TM4SF18	0.400288	0.064513	0.209537
RNMTL1	-0.18251	0.064532	0.209537

BRI3BP	-0.18675	0.064539	0.209537
AGAP11	0.004501	0.06454	0.209537
RNF207	-0.04551	0.064553	0.20954
KLF12	-0.12036	0.06456	0.20954
MT1H	0.559935	0.064617	0.209694
METTL8	-0.20412	0.064658	0.209794
MIPEP	-0.26737	0.064672	0.209809
HDAC2	0.09514	0.06473	0.209966
PKHD1L1	0.217975	0.064757	0.21002
IL13	-0.03044	0.064782	0.21006
CIB2	0.086803	0.064788	0.21006
ITGB2	0.311168	0.064839	0.210193
RUNX1T1	0.115235	0.064866	0.210249
PELI1	0.334875	0.064882	0.210269
TCTN2	0.065823	0.064969	0.210496
RP11-220I	-0.01881	0.064971	0.210496
EGR3	0.586752	0.065038	0.210681
MAP2K7	-0.13701	0.065106	0.210868
RAPGEF5	0.026634	0.065128	0.210909
NPB	0.063224	0.065218	0.211148
KCNH3	0.048125	0.065221	0.211148
VANGL2	0.189326	0.065236	0.211165
MICU2	-0.1865	0.065257	0.211201
PDE4B	0.461665	0.065295	0.21129
TCEB1	-0.14013	0.065345	0.211421
PDCD2L	0.24129	0.065431	0.211669
ZNF234	-0.08959	0.065486	0.211814
RP11-629C	-0.14286	0.065517	0.211883
RP11-245J	-0.12322	0.065656	0.212301
LOC28555	-0.01084	0.065716	0.212465
RP11-708J	-0.11948	0.065804	0.212716
RRM2B	-0.19629	0.065826	0.212755
PPAPDC3	0.183023	0.065941	0.213094
TMCC1	0.193647	0.065958	0.213119
PLCE1	-0.0122	0.065989	0.213187
LRRC47	-0.1105	0.066007	0.213213
LRIF1	0.318359	0.066026	0.213243
MMD2	0.028696	0.066088	0.213406
RP11-400N	-0.04454	0.066096	0.213406
IGFBP3	0.131264	0.066136	0.213461
DNAJC9	-0.20103	0.06614	0.213461
PLXNC1	-0.12852	0.066143	0.213461
SLC17A8	0.05392	0.066153	0.213461
ZP2	-0.00839	0.066211	0.213618
ATP5L	0.140519	0.066247	0.213701
PCED1B-A	-0.03379	0.066307	0.213863
CDC16	-0.08958	0.066391	0.214086
VCAN	0.585537	0.066396	0.214086
FAM212B-	-0.27237	0.066413	0.214109
TXNDC17	0.11031	0.066451	0.214201
BAIAP3	-0.02867	0.066516	0.214368
RAB11B	-0.02219	0.06653	0.214368
LOC28488	-0.12991	0.066533	0.214368
THEM4	0.262915	0.066578	0.214481
CHURC1	0.215225	0.066633	0.214627
TFDP1	0.347246	0.06672	0.214874
ENO3	-0.09128	0.066761	0.214971
NME7	-0.22681	0.066769	0.214971

BC039487	-0.04256	0.06683	0.215108
CCDC84	-0.12939	0.066832	0.215108
RICTOR	0.212959	0.066855	0.215152
SLC10A1	-0.07399	0.066877	0.215177
WDR6	0.117265	0.066883	0.215177
SLX4IP	0.150852	0.066897	0.21519
NRXN3	0.040331	0.066918	0.215225
POLE2	-0.32684	0.066941	0.215267
ELP6	0.218017	0.067011	0.215461
RBPMS-AS	-0.08572	0.067024	0.21547
NAPG	0.137447	0.067153	0.215849
RP11-456(	-0.03391	0.067162	0.215849
RNF167	0.124422	0.06718	0.215875
LINC00838	0.014178	0.0672	0.215907
XPNPEP3	-0.16981	0.06721	0.215907
TNNI3	0.052147	0.067271	0.21607
LOC10192	-0.04333	0.067286	0.216074
ARFRP1	0.108177	0.067292	0.216074
GALE	-0.10175	0.067409	0.216417
FAM151A	0.002871	0.067429	0.21645
LOC10192	-0.01305	0.067464	0.216532
SYNJ2	0.113396	0.06749	0.216581
TGDS	0.121103	0.067542	0.216718
ZNF146	0.165366	0.06759	0.216839
GET4	-0.12739	0.067609	0.216867
LINC00685	0.102281	0.067626	0.216889
LINC00521	-0.00617	0.067639	0.216899
PPP1R17	-0.02568	0.06771	0.217094
MEF2D	0.143731	0.067743	0.217162
IFT122	-0.05576	0.067751	0.217162
SCYL3	-0.13879	0.067864	0.217492
RPRD1B	0.087253	0.067882	0.217519
CELSR1	0.004138	0.067932	0.217621
NMT2	-0.2142	0.067936	0.217621
REPS2	0.163942	0.067944	0.217621
CRYM	-0.35129	0.067988	0.2177
TTC9	-0.12272	0.067989	0.2177
FRMD3	0.48193	0.068047	0.217852
LRRC39	0.147707	0.068057	0.217854
ZNF41	0.034437	0.068146	0.218105
PSMA4	-0.09741	0.06816	0.21812
RP11-108k	-0.02309	0.068276	0.218456
CTSO	0.283074	0.068329	0.218593
MUC3B	-0.03889	0.068453	0.218958
MDS2	-0.01477	0.068464	0.218961
IL21R	0.04896	0.068481	0.218965
FAM205A	0.004788	0.068485	0.218965
TDRD12	0.104171	0.068501	0.218983
PPP1R3B	0.095116	0.068518	0.219004
NLE1	-0.24097	0.068603	0.219246
DEPDC1-A	-0.03962	0.068618	0.219259
CTB-181H	-0.2816	0.068633	0.219275
LOC10193(	-0.03671	0.068664	0.219342
FAM53B-A	0.013231	0.068701	0.219427
TSTD2	-0.02361	0.068748	0.219545
GAK	-0.10633	0.068773	0.219595
LOC14293	-0.30056	0.068794	0.219622
IRF5	0.320641	0.068802	0.219622

CXXC4	-0.01928	0.068832	0.219684
CRHBP	0.511093	0.068863	0.219748
PSMG1	-0.17536	0.068872	0.219748
ATP1B2	0.075802	0.068894	0.219786
PROKR2	0.008742	0.068954	0.21992
SHPRH	-0.27571	0.068957	0.21992
EIF2D	-0.14605	0.068983	0.219972
DNAH12	-0.07202	0.069033	0.2201
LDLRAP1	-0.19242	0.06915	0.22044
DBF4	-0.18651	0.069183	0.220498
RAVER2	0.125737	0.069189	0.220498
LOC10099(	-0.10556	0.069243	0.220638
PTP4A3	0.231614	0.069259	0.220657
LINC00174	-0.01621	0.069315	0.220784
ARHGAP5	-0.26719	0.069326	0.220784
LOC10099(	-0.04462	0.069329	0.220784
CAPG	0.315274	0.069407	0.220974
PLCG1	0.085863	0.069415	0.220974
MED1	0.115149	0.06942	0.220974
KIAA1522	0.219392	0.069437	0.220998
TAF15	-0.21416	0.069483	0.221112
ABCC1	0.180669	0.069495	0.221115
GYG2	0.084859	0.069535	0.221211
TMEM145	0.006868	0.069559	0.221253
LDHD	0.020831	0.069597	0.221342
SPAG5	0.288195	0.069641	0.221451
RP5-1074L	-0.32278	0.069658	0.221471
SLC45A3	0.105882	0.069702	0.22158
LOC72908:	0.031202	0.069748	0.221692
AX747826	-0.40585	0.069817	0.221841
DEGS1	0.13834	0.069824	0.221841
GS1-111G:	0.22013	0.069825	0.221841
IRAK2	-0.0394	0.06985	0.221867
SLC22A7	-0.00808	0.069862	0.221867
TGIF1	0.240454	0.069864	0.221867
FAIM2	-0.0254	0.069969	0.222165
AP000525.	0.404138	0.069979	0.222165
PIAS3	0.194177	0.070005	0.222216
CDKN2AIP	0.160179	0.070084	0.222436
STARD4	-0.30539	0.070194	0.22274
OR1C1	-0.02303	0.070205	0.22274
LOC10050!	-0.14986	0.070211	0.22274
RP4-794H:	0.015845	0.070244	0.22281
ARHGAP12	0.279269	0.070298	0.222934
ZNF667	-0.22882	0.070303	0.222934
SENP3	0.102306	0.070326	0.222975
OR51B4	0.008376	0.070521	0.223559
GJB6	0.161389	0.070605	0.223792
ABLIM1	-0.22249	0.070645	0.223887
RP1-39G2:	-0.16928	0.07068	0.223965
HMGN5	-0.17309	0.070725	0.224061
ZKSCAN4	0.202242	0.070731	0.224061
BLOC1S6	0.205066	0.070765	0.224136
ZNF562	0.061555	0.070824	0.22429
ZNRF1	0.22492	0.070851	0.224341
ZNF492	-0.27959	0.07087	0.224353
TRIM61	-0.10982	0.070875	0.224353
PRNP	0.156673	0.070895	0.224384

SEC11A	-0.11938	0.070936	0.224464
SOBP	0.002796	0.070941	0.224464
LOC72929:	-0.02518	0.070968	0.224515
SACS	-0.17294	0.070985	0.224537
GINS1	-0.25896	0.071003	0.224562
LOC10192	-0.02604	0.071169	0.225054
SGIP1	-0.23272	0.071301	0.225427
RP11-65L1	-0.04455	0.071308	0.225427
HPS4	-0.23063	0.071373	0.2256
GBP2	0.41147	0.071423	0.225724
LMAN2	0.085171	0.071433	0.225724
ARFGEF2	0.09287	0.071461	0.225779
RNF7	0.129268	0.071506	0.225889
RP1-155D2	0.00498	0.071547	0.225986
SPAST	-0.15039	0.07156	0.225993
GGCX	0.236006	0.071579	0.226019
C11orf71	0.117065	0.071596	0.226041
LOC14779:	0.013587	0.071657	0.226201
PLCZ1	0.006037	0.071682	0.226246
FRMPD4	-0.00865	0.071716	0.226313
POU5F2	-0.0095	0.071724	0.226313
LOC10028	-0.11769	0.071755	0.226377
FAM171A1	0.548774	0.071804	0.226498
NPC1	0.088894	0.071818	0.22651
RPH3AL	0.027588	0.071833	0.226515
PHYHD1	0.116285	0.07184	0.226515
RP11-644F	0.082178	0.071933	0.226775
VOPP1	0.192432	0.071953	0.226803
CDK17	-0.15998	0.072	0.226899
C11orf94	-0.05363	0.072004	0.226899
ALDH1L1	-0.03549	0.072019	0.226913
DDX50	-0.21631	0.072035	0.226931
RP1-58B11	-0.07035	0.072057	0.226968
BIRC6	0.149603	0.072188	0.227343
PCDHA5	-0.0516	0.072197	0.227343
ALDH1L1-,	0.030376	0.072208	0.227344
LOC10028	0.040621	0.072303	0.227608
HIF1A	-0.18026	0.072318	0.227624
AACSP1	-0.00972	0.072332	0.227633
HTATSF1P2	0.128619	0.072391	0.227787
SDC4	0.309095	0.072419	0.227792
KIR2DL1	-0.0544	0.07242	0.227792
BBX	0.131791	0.072424	0.227792
PRPF40B	0.046583	0.072467	0.227891
AKR1B10	0.02482	0.072477	0.227891
GRHL3	0.003565	0.07253	0.228025
ADRB1	-0.02311	0.072599	0.228209
XRN1	0.232582	0.072636	0.228291
FAM229B	0.177203	0.07266	0.228335
PCSK6	-0.01024	0.072672	0.228339
RP11-102M	-0.00849	0.072696	0.228363
IMP3	0.215552	0.072701	0.228363
SLC16A7	-0.24158	0.072718	0.228378
PPM1H	-0.15049	0.072727	0.228378
SEC61A1	0.160504	0.072759	0.228445
RBM33	-0.16749	0.072794	0.228523
LOC10192	0.007227	0.072881	0.228763
JMJD6	0.151956	0.072908	0.228809

N4BP2L2-I	-0.13059	0.072917	0.228809
ZNF507	-0.13092	0.07306	0.229226
KLHL29	0.005932	0.073113	0.229358
WDR19	-0.07766	0.07313	0.22938
SLAIN1	-0.42479	0.073149	0.229406
NR0B2	-0.01099	0.073207	0.229526
GZMH	0.307864	0.073209	0.229526
LPAR5	0.234417	0.073342	0.229912
PPIP5K2	-0.23771	0.073362	0.22994
ZNF354B	-0.24991	0.07341	0.230058
BC036209	0.014825	0.073444	0.230132
SH2D3A	0.03373	0.073466	0.230165
PRR12	0.069079	0.0735	0.230238
ELF1	0.120772	0.073558	0.230389
MESP1	-0.0507	0.073598	0.230449
LINC00922	0.034481	0.073599	0.230449
PLA1A	0.015656	0.073735	0.23084
FXYD1	0.005706	0.073768	0.230886
SUV420H2	-0.05347	0.07377	0.230886
CREB3L2	0.167273	0.073784	0.230896
LINC00238	0.016463	0.073839	0.231035
SPATA20	0.263318	0.073861	0.231068
HOXD12	0.006973	0.073882	0.231092
KDM4A	0.125018	0.073897	0.231092
GATA1	0.297093	0.0739	0.231092
LAMA2	0.158858	0.073914	0.2311
C19orf57	0.050007	0.07393	0.231118
PIK3C2B	-0.29707	0.074013	0.231322
SUV39H2	-0.12647	0.074027	0.231322
DUSP6	0.368212	0.074027	0.231322
FAM192A	-0.12438	0.074047	0.231343
RP1-199J3	-0.06279	0.074055	0.231343
POU4F2	0.003772	0.074111	0.231484
FLJ16779	-0.0105	0.074182	0.231672
PHOX2A	-0.05835	0.07427	0.231913
WDYHV1	-0.17602	0.074281	0.231913
FCAR	0.109501	0.074304	0.231951
GABPB2	-0.21935	0.074322	0.231974
ARRB1	-0.17005	0.074344	0.231974
COASY	0.12925	0.074347	0.231974
BUD13	0.172711	0.074356	0.231974
MRPL49	0.155128	0.074364	0.231974
COPS7B	0.162533	0.074378	0.231978
LOC10192	-0.06621	0.074387	0.231978
GYPE	0.163813	0.074464	0.232185
SKAP1	0.332848	0.074487	0.232221
CCDC14	-0.05821	0.074548	0.232378
PRAP1	0.004995	0.074581	0.232449
SYNDIG1L	0.021941	0.074607	0.232495
ATE1	-0.08313	0.074628	0.2325
EFS	0.110956	0.074632	0.2325
FAM134B	0.16585	0.074641	0.2325
ZBED3	0.164748	0.074655	0.232507
FBXW7	0.184572	0.074664	0.232507
SH3BP5-A	-0.20256	0.074686	0.232537
WNT9A	0.023936	0.074695	0.232537
BMX	0.280998	0.074831	0.232926
RP11-391N	-0.20129	0.074842	0.232926

MUC5B	0.017851	0.07492	0.233136
COX11	-0.10652	0.074988	0.233312
RP11-135A	-0.04461	0.074998	0.233312
GTPBP3	0.073668	0.075017	0.233339
PSG11	0.019074	0.075035	0.233361
PNPO	0.232841	0.075051	0.233376
SMCR8	0.123473	0.075071	0.233403
PHF11	0.126518	0.075083	0.233408
C8orf4	0.287616	0.075165	0.233612
RP11-120K	-0.0583	0.075185	0.233612
MSANTD3	0.181049	0.075191	0.233612
AQP2	-0.0307	0.075192	0.233612
CHD6	-0.10991	0.075216	0.233624
SLC25A51	0.173986	0.075225	0.233624
EMC2	-0.23253	0.075228	0.233624
SRPK1	-0.08377	0.075296	0.233803
LOC10192!	0.012228	0.075327	0.233866
HAUS6	0.273442	0.075341	0.233875
RELB	0.262352	0.075408	0.234034
TMEM175	0.116254	0.075414	0.234034
LINC00882	-0.01524	0.07545	0.234103
CHRM5	-0.06181	0.075463	0.234103
ZNF780A	0.113879	0.075469	0.234103
HECTD2	-0.0776	0.075544	0.234301
SLC25A41	-0.02726	0.075554	0.234301
TAF1C	-0.10918	0.075609	0.234433
KCTD20	0.257819	0.075626	0.234433
TMEM167E	-0.12861	0.075629	0.234433
FLNB	-0.22893	0.075688	0.234582
C5orf42	-0.08744	0.07571	0.234617
FEM1C	0.252269	0.075755	0.234693
LOC10272:	-0.02215	0.075756	0.234693
TIMM44	0.173807	0.075808	0.234811
NAALAD2	0.022291	0.075842	0.234811
NFKBIL1	0.057689	0.075861	0.234811
AP3S1	0.128543	0.075863	0.234811
LYPD3	0.049308	0.07588	0.234811
ST3GAL1	0.17506	0.075885	0.234811
VPS26A	-0.1129	0.07589	0.234811
VBP1	-0.09827	0.075892	0.234811
MTERF2	0.202453	0.075892	0.234811
RCAN3	-0.26922	0.075907	0.234822
LEMD2	0.106491	0.076008	0.235102
SLC17A6	-0.0059	0.07605	0.235188
MTERF4	-0.09236	0.076057	0.235188
RP11-672L	-0.25755	0.076083	0.235235
DCUN1D4	-0.2001	0.076105	0.235269
ABCA3	0.118431	0.076148	0.235366
CTD-2619J	-0.0237	0.076187	0.235433
OR10H1	-0.05775	0.076191	0.235433
TES	-0.24611	0.07623	0.235505
H2AFX	0.082357	0.076236	0.235505
LOC10192!	0.016613	0.076265	0.235561
HCG9	0.007156	0.076372	0.235843
MYO15B	0.28091	0.076378	0.235843
CYP7A1	0.006771	0.076405	0.235894
AC008746.	0.138153	0.076458	0.236024
PTAFR	0.251561	0.076492	0.236093

CCNL1	0.314079	0.07655	0.236199
PHKG1	-0.01941	0.076555	0.236199
SNAI3	0.056629	0.076559	0.236199
ABAT	-0.26049	0.076584	0.236216
MYOG	-0.06709	0.076594	0.236216
PDCL	-0.1056	0.076597	0.236216
TMPRSS11	-0.01461	0.076617	0.236244
KLF1	0.590166	0.076657	0.236335
LOC10192!	-0.21773	0.076837	0.236855
RP11-209D	-0.07842	0.076859	0.236884
PCDHA9	-0.0421	0.076868	0.236884
FAM135A	0.156299	0.076893	0.236901
GPR182	0.312824	0.076896	0.236901
FAM114A2	0.1803	0.076954	0.237029
AC092660.	-0.03658	0.076959	0.237029
AHCYL2	-0.08299	0.077005	0.237137
PANK4	0.183937	0.077029	0.237177
ZNF736	-0.16234	0.077052	0.237189
COPA	-0.12965	0.077055	0.237189
NR3C1	-0.21814	0.077071	0.237204
ABCC11	-0.01043	0.077095	0.237244
THBS2	0.029733	0.077129	0.237317
AK021933	-0.03955	0.077162	0.237383
CDC6	0.372034	0.07721	0.237499
PDSS2	-0.15417	0.077265	0.237634
KIAA0101	-0.19213	0.077334	0.237811
CLYBL-AS1	0.005078	0.077356	0.237844
POP4	0.100719	0.077384	0.237896
LOC10050!	0.006509	0.07742	0.237976
SGSM3	0.104643	0.077455	0.238047
LOC10272:	-0.01695	0.077488	0.238114
LINC00868	-0.05168	0.077508	0.238114
EPHX2	0.279908	0.077509	0.238114
AGO3	0.214068	0.077549	0.238201
AP000696.	0.014391	0.077564	0.238214
ASH2L	-0.16734	0.077599	0.238242
KIAA1211L	0.105879	0.077616	0.238242
LOC64535!	-0.01345	0.077618	0.238242
ICAM3	-0.26288	0.077619	0.238242
LOC14937:	-0.09176	0.077649	0.238242
CALB1	0.070931	0.077649	0.238242
CCDC71L	0.107607	0.07765	0.238242
TMEM43	0.156815	0.077661	0.238243
SMAD9	0.156062	0.077782	0.23858
TINF2	0.103907	0.077819	0.238641
PLEKHA3	0.1681	0.077824	0.238641
TMEM136	-0.21539	0.077841	0.238651
CCDC86	0.231853	0.077854	0.238651
ZNF362	-0.0827	0.077861	0.238651
CCDC50	0.350689	0.07789	0.238677
FLRT1	-0.02851	0.077891	0.238677
PPP1R14D	-0.03043	0.077941	0.238795
RP11-288H	0.061704	0.077978	0.238857
LTBR	0.034615	0.077983	0.238857
CLIP2	0.226153	0.077998	0.23887
HESX1	0.213709	0.078012	0.23888
PPP1CC	-0.11146	0.07804	0.238927
NXPE1	-0.02895	0.07805	0.238927

DQX1	0.006938	0.078096	0.239035
LOC10192	-0.06881	0.078176	0.239246
VKORC1L1	-0.10762	0.078329	0.23965
TMOD1	0.402359	0.078331	0.23965
SPCS1	0.138331	0.078355	0.23965
RP11-5N1	-0.03496	0.078356	0.23965
LINC00507	0.005968	0.078363	0.23965
HOXA7	-0.20578	0.078395	0.239713
RLIM	-0.15508	0.078454	0.239815
EPG5	-0.15075	0.078458	0.239815
IP6K2	-0.15707	0.078462	0.239815
DCLRE1B	0.164654	0.078486	0.239855
ZNF460	-0.02328	0.078574	0.240069
SOX12	0.113263	0.078578	0.240069
AKAP8L	0.146127	0.078684	0.240358
LOC64320	0.184353	0.078707	0.240395
RPS4Y1	1.487752	0.07874	0.240463
EPOR	0.338596	0.078757	0.24048
SSNA1	0.154862	0.078782	0.240522
CPQ	0.243335	0.0788	0.240543
HAPLN3	0.067529	0.078824	0.240582
LINC01231	-0.02405	0.078849	0.240624
SIRPG	0.036292	0.078902	0.240754
NPSA	-0.01573	0.078928	0.240797
NPTN	-0.19039	0.078954	0.240844
ARMC8	-0.12087	0.078973	0.240844
ACVR1C	0.014476	0.078976	0.240844
AMIGO1	0.096218	0.079249	0.241642
CELA1	-0.05156	0.079286	0.241719
LOC10050	0.052826	0.079297	0.241719
SLC9A1	-0.12964	0.07932	0.241755
CUL5	0.139837	0.079473	0.242188
MRPL41	-0.05151	0.079529	0.242323
CNIH3	-0.03419	0.079555	0.242344
RP11-96D	-0.01095	0.079558	0.242344
ATF7IP2	-0.25496	0.079652	0.242577
DDRGK1	0.134909	0.079657	0.242577
PMM1	0.108221	0.07971	0.242651
SEL1L	0.119539	0.079717	0.242651
CASP7	-0.16807	0.079735	0.242651
CBLC	-0.01834	0.07974	0.242651
MRGPRG-	-0.02627	0.079741	0.242651
ZNF16	-0.17373	0.079748	0.242651
FLAD1	0.141429	0.079919	0.243124
SPA17	0.268167	0.079926	0.243124
OR1F1	-0.02059	0.079986	0.243272
C6orf123	-0.00699	0.080068	0.243488
BZRAP1-A	-0.27174	0.080171	0.243767
LOC54944	-0.03961	0.080187	0.243781
LOC10192	0.009097	0.080228	0.24387
LSM6	0.153521	0.080303	0.244061
ZNF121	-0.15879	0.080313	0.244061
TMED10	0.14232	0.08034	0.24411
LOC10050	-0.01987	0.080367	0.244141
LOC28606	0.005743	0.080374	0.244141
TMEM155	0.032093	0.080384	0.244141
CSF3R	0.357305	0.080405	0.24417
KCNK1	0.151859	0.080483	0.244372

SCAF8	0.229743	0.080572	0.244605
MRPS22	-0.11295	0.080582	0.244605
HYPK	0.069551	0.080622	0.244691
NFKB2	0.075753	0.080696	0.244868
RP11-39H1	0.011894	0.080703	0.244868
C4orf17	-0.0363	0.080731	0.244919
GALNT11	-0.16162	0.080764	0.244984
C5orf51	0.078237	0.080824	0.245132
RP11-82L1	-0.03072	0.080886	0.245286
LMNA	0.241851	0.080906	0.245312
HGSNAT	-0.20354	0.080989	0.245529
AKAP6	-0.01871	0.08106	0.245689
PSME3	0.227199	0.081064	0.245689
RP1-118J2	-0.08542	0.08112	0.245823
MT1E	0.489986	0.081286	0.246293
POMC	0.085768	0.08132	0.24636
CNTNAP1	-0.02427	0.081332	0.246362
B4GALT3	0.080051	0.081363	0.246423
WIPF1	0.160479	0.081423	0.246568
SSBP3-AS1	-0.22806	0.081473	0.246669
LOC10192	-0.0043	0.081479	0.246669
PRKRA	-0.15846	0.081527	0.24678
RP11-568M	-0.34868	0.081601	0.24697
MSTN	0.016162	0.081742	0.247361
PDE2A	0.131848	0.08181	0.247533
ACAN	-0.03477	0.081861	0.247654
TNNC1	0.009311	0.082014	0.248082
DDX60L	0.152738	0.08204	0.248095
KIAA1107	-0.13394	0.082041	0.248095
OCRL	0.065414	0.082057	0.248106
PAF1	-0.15435	0.082108	0.248227
BBS12	0.272812	0.082125	0.248231
ZBTB46-AS	-0.0123	0.082142	0.248231
URI1	0.126916	0.082144	0.248231
LOC10050	0.004188	0.082157	0.248235
TXK	0.252572	0.082187	0.248293
ATP1A2	-0.02308	0.082209	0.248303
ELOF1	0.177035	0.082223	0.248303
TMEM159	0.269083	0.082225	0.248303
LOC10192	-0.14073	0.082323	0.248565
ULK3	0.178756	0.082341	0.248583
IZUMO4	-0.03327	0.082363	0.248616
TSC1	0.106018	0.082377	0.248624
GRM2	-0.03852	0.082417	0.248709
TRIM35	0.124568	0.082439	0.248741
ZNF81	-0.01856	0.082484	0.248841
VIPR2	0.020418	0.082529	0.248943
PDXDC1	-0.09654	0.082545	0.248956
ATL2	0.090981	0.082595	0.249073
KIFAP3	0.218931	0.082606	0.249073
LOC20202	0.239393	0.082663	0.24921
ENHO	-0.05267	0.082734	0.249374
ICMT	0.188004	0.082743	0.249374
SLC4A2	-0.27253	0.082759	0.249374
IQGAP1	0.267968	0.082764	0.249374
CTTNBP2	0.2052	0.082779	0.249385
LOC10050	-0.1197	0.082854	0.249577
PRR15L	0.022466	0.082893	0.24966

CTNNB1	0.198757	0.082907	0.249668
KIAA0232	0.147463	0.082943	0.24974
CTC-428G:	-0.17684	0.082984	0.249799
NFATC3	0.100172	0.082986	0.249799
CYP1A1	0.101633	0.08302	0.249866
CNN1	-0.18522	0.083047	0.249899
SLC4A9	-0.03466	0.083058	0.249899
OGDHL	0.127661	0.083065	0.249899
BRWD1	-0.15498	0.08309	0.249938
IL37	-0.04585	0.083139	0.250052
FAM104A	0.148861	0.083203	0.250211
ASIC1	-0.01668	0.083253	0.250316
UBAC1	0.224082	0.083262	0.250316
SPEF2	-0.09022	0.083313	0.250438
NCAPD2	0.248264	0.083362	0.250525
MYO1G	-0.22256	0.083366	0.250525
UPK2	-0.01397	0.083377	0.250525
LOC10192(	-0.04221	0.08343	0.250648
TCAIM	0.228061	0.083459	0.250685
NRN1	0.150425	0.083465	0.250685
PRR5	0.21341	0.083545	0.250891
GPR27	-0.24663	0.083565	0.250914
COX4I2	-0.05468	0.083582	0.250931
LCN15	-0.0596	0.08362	0.251012
SCFD2	-0.13345	0.083637	0.251027
RP11-496I:	0.003596	0.083649	0.251028
WBSCR27	0.029056	0.083702	0.251133
GCNT3	0.03832	0.083707	0.251133
CD36	0.565836	0.083788	0.25134
KIAA0513	0.168375	0.083869	0.251549
ZNF836	-0.09839	0.08397	0.251817
ADCYAP1F	-0.01817	0.08399	0.251841
RC3H1	-0.22225	0.084119	0.252195
RAB11FIP1	0.187107	0.084146	0.252241
FLNC	-0.06036	0.084199	0.252363
GBGT1	0.274982	0.08421	0.252363
RP11-635N	0.034191	0.084224	0.252369
ISM2	-0.04767	0.084282	0.252507
CWF19L1	0.155625	0.084339	0.252643
TEKT1	0.007288	0.084356	0.252661
CLEC4D	0.216097	0.084389	0.252724
EMR2	0.184386	0.084451	0.252874
HELZ2	0.016055	0.084493	0.252964
CRB1	0.0911	0.08452	0.252982
BAALC	-0.44763	0.084522	0.252982
GSN-AS1	0.015875	0.08459	0.253152
FOXN4	-0.01011	0.084637	0.253255
ABHD10	-0.14246	0.08467	0.253321
LAMA5-AS	0.02692	0.084695	0.25336
RP13-122E	0.022725	0.084728	0.253424
BMP10	0.032866	0.084744	0.253435
YIPF6	0.124155	0.084806	0.253588
IBA57	-0.18272	0.084819	0.253592
AK021804	0.250476	0.084838	0.253613
DENND6A	-0.12625	0.084878	0.253697
TTC7B	0.300151	0.085142	0.254431
ZNF665	-0.134	0.085147	0.254431
SCT	0.074303	0.08519	0.254523

KCNK17	-0.41323	0.085244	0.254649
PSRC1	0.266825	0.085283	0.254732
ZNF614	-0.10144	0.085325	0.254738
LOC14841:	-0.15203	0.085327	0.254738
ZNHIT3	0.175107	0.085328	0.254738
TLK2	0.133566	0.085338	0.254738
CYB5D2	0.15477	0.08535	0.254738
ADCY3	-0.17127	0.085369	0.254738
FAM195B	0.096316	0.085376	0.254738
EHHADH	-0.27545	0.085379	0.254738
LOC72987(	0.089311	0.085449	0.25491
L2HGDH	0.118961	0.085468	0.254932
MAST3	0.214779	0.085511	0.255009
ARPC5L	-0.16377	0.085529	0.255009
SMG8	-0.17549	0.085537	0.255009
PRR15	-0.2373	0.085541	0.255009
C10orf67	-0.01722	0.085581	0.255093
PPP2R5A	0.097968	0.085614	0.255137
PRB4	-0.04517	0.085619	0.255137
CCNG1	-0.12221	0.08565	0.255195
RAI14	0.238635	0.085672	0.255223
IDI2-AS1	0.062142	0.085744	0.2554
C2orf49	0.201475	0.085761	0.2554
MEF2BNB	0.131337	0.085766	0.2554
RCN1	0.153688	0.085835	0.255569
CHP2	-0.00323	0.085944	0.255836
PROSER3	0.049161	0.085948	0.255836
HIST1H1T	-0.0493	0.085966	0.255843
POU5F1B	-0.04352	0.085974	0.255843
OSR1	0.010436	0.086019	0.255942
B3GALT1	-0.01573	0.086062	0.256034
SLC30A9	-0.13397	0.086172	0.256327
IRX2	0.018385	0.086251	0.256525
PPP5C	0.053809	0.086303	0.256627
PI4KA	0.161617	0.086314	0.256627
TMEM59L	-0.03374	0.086321	0.256627
AKAP11	-0.19048	0.086372	0.256725
MAP10	-0.02504	0.086377	0.256725
PAQR8	-0.18468	0.086457	0.256909
NUDT15	0.157785	0.086463	0.256909
XDH	-0.04011	0.086475	0.256911
GNAS	0.131734	0.086495	0.256933
NDRG2	0.011591	0.08653	0.256983
CEP85L	-0.12067	0.086535	0.256983
PTGFR	-0.00674	0.086548	0.256984
LINC00629	0.039468	0.086575	0.257029
GCN1L1	0.136864	0.086586	0.257029
C19orf12	0.197538	0.086659	0.257209
OR5AK4P	-0.03313	0.086672	0.257213
CD6	-0.00825	0.086691	0.257233
C9orf135-/	-0.02895	0.086782	0.257463
ELANE	0.739748	0.086792	0.257463
LOC44166(	0.0852	0.086818	0.257506
CALCOCO:	-0.18502	0.086842	0.257514
RP11-366L	0.072521	0.086845	0.257514
FOXD2	-0.01659	0.086876	0.257572
C10orf88	-0.1515	0.086888	0.257572
CTC-338M	-0.14174	0.086923	0.25764

ABLIM2	-0.02995	0.086994	0.257816
TMEM108-	-0.03451	0.087129	0.258176
CD200R1	0.097569	0.08714	0.258176
FLJ34521	-0.02602	0.087171	0.258235
PROC	-0.15491	0.087201	0.258288
NANP	-0.21601	0.087234	0.25835
LOC10192	0.101292	0.087278	0.258441
TEDDM1	0.016107	0.087289	0.258441
STAG2	-0.2272	0.087357	0.258607
MRAP2	-0.18088	0.08738	0.25864
CCDC8	-0.17094	0.087416	0.25871
RIMKLA	0.02942	0.087489	0.258894
SGPP1	0.359229	0.087551	0.25904
SLFNL1	-0.07242	0.087579	0.259087
SNX6	0.180847	0.087615	0.259158
SPOPL	-0.14918	0.087655	0.259242
FNDC5	0.041444	0.087767	0.259538
TEX28	0.004933	0.087806	0.259617
PLEKHH3	0.019055	0.087824	0.259636
RPL19	0.12227	0.087843	0.259656
DPYSL4	0.013509	0.087894	0.259771
HLF	-0.46169	0.088022	0.260115
FAM224A	-0.03772	0.088084	0.260262
PCDH12	0.182572	0.088145	0.260406
TREM2	0.05038	0.088158	0.260408
AC012531.	-0.04021	0.088201	0.260501
ACBD5	0.157714	0.088216	0.260509
TRPC2	0.166207	0.088262	0.260585
ATG4C	-0.14873	0.088266	0.260585
RNMT	-0.1487	0.088278	0.260587
FUT8-AS1	-0.10699	0.088327	0.260696
ISOC1	-0.316	0.088349	0.260723
ADM	0.442889	0.088465	0.26103
KRT5	0.014992	0.088513	0.261138
PAX8	-0.01919	0.088535	0.26115
C10orf55	0.045842	0.088541	0.26115
LRRC45	0.093298	0.088577	0.261219
IL23A	-0.05193	0.088602	0.261259
LOC10013	0.026916	0.088647	0.261348
LOC10192	0.008528	0.088657	0.261348
SYPL1	-0.15624	0.088716	0.261486
RMND5B	0.102051	0.088762	0.261586
PDE4A	0.042676	0.088861	0.261805
RP11-774	-0.11783	0.088869	0.261805
C8orf44	-0.2414	0.088872	0.261805
NSUN6	0.21432	0.088918	0.261906
FAM81B	-0.29692	0.088952	0.261969
C12orf4	0.158988	0.088989	0.262043
CSPP1	0.208931	0.089071	0.262192
RPL23AP3	-0.19597	0.089079	0.262192
HIST1H2A	-0.38644	0.089088	0.262192
GCC1	-0.03295	0.089088	0.262192
HOTAIR	0.013503	0.089102	0.262197
CD82	0.188574	0.089121	0.262217
CA14	-0.06503	0.089173	0.262291
LINC00639	0.006106	0.089182	0.262291
C1orf177	0.003681	0.089183	0.262291
MND1	0.393462	0.089233	0.262314

SBF2	-0.34876	0.089237	0.262314
C5orf34	-0.20272	0.089238	0.262314
LOC33966(	-0.06192	0.089247	0.262314
AC005306.	-0.12872	0.089251	0.262314
LOC10013:	-0.29274	0.08943	0.262804
SNCG	-0.01253	0.089451	0.262829
MMP17	-0.02688	0.089503	0.262948
SORD	0.204679	0.089535	0.262968
BMP2	0.163706	0.089546	0.262968
RP11-63K6	-0.03518	0.089546	0.262968
SECISBP2L	-0.18205	0.08956	0.262974
AGTPBP1	-0.1769	0.089588	0.263018
MCTP2	-0.27342	0.089606	0.263036
RP1-68D18	-0.03974	0.089644	0.263111
AASS	0.307727	0.089686	0.263199
PHF7	0.095084	0.089737	0.263313
CA12	0.002256	0.089769	0.263371
LMAN1	-0.11988	0.089786	0.263386
DQ599616	0.02242	0.089823	0.26346
MYC	0.322347	0.089861	0.263513
ITGB5	0.257734	0.089866	0.263513
KLK8	-0.02622	0.089975	0.263777
H1FOO	-0.03076	0.089989	0.263777
NDRG4	0.016995	0.089992	0.263777
FAM71F2	0.006339	0.090043	0.263889
AOC3	0.371468	0.090061	0.263907
ECM1	0.100727	0.090083	0.263932
ATP2A2	-0.12075	0.090123	0.263932
CLCN7	0.122491	0.090138	0.263932
PCDH9	-0.42739	0.09014	0.263932
NAP1L2	-0.41466	0.09014	0.263932
TRIM16	-0.17405	0.090143	0.263932
TMED1	0.159002	0.090161	0.263949
TTC5	-0.11007	0.090249	0.264146
HCP5	0.308122	0.090252	0.264146
ZMYND10	-0.02169	0.090353	0.264404
CRYZL1	-0.21734	0.090394	0.264488
ZP4	-0.0039	0.090449	0.264615
IGDCC3	-0.0251	0.090473	0.264648
BLCAP	-0.17537	0.090506	0.26471
NOA1	-0.21608	0.090583	0.264868
ZNF777	0.035562	0.090585	0.264868
ABCG4	0.033468	0.09064	0.264993
RP11-304(	-0.02341	0.090673	0.265055
CKB	0.2497	0.090693	0.265079
RP4-740C4	-0.20011	0.090746	0.265198
FPGS	0.168797	0.090766	0.265218
IKBIP	-0.20149	0.090794	0.265242
PTBP3	-0.21204	0.090798	0.265242
ULBP1	-0.01679	0.090873	0.265425
PIH1D3	-0.02328	0.090891	0.265442
FAM76B	0.138209	0.090926	0.26549
HORMAD1	0.043091	0.090932	0.26549
RP11-58O:	0.037242	0.090976	0.265583
PTX3	0.540033	0.091013	0.265655
SLC12A8	0.018304	0.09113	0.265942
DIO3OS	0.010164	0.091136	0.265942
GTF3A	-0.08056	0.091168	0.265999

MAP3K7	0.165867	0.091306	0.266328
TPRKB	0.150254	0.091313	0.266328
FAM198B	0.215177	0.091318	0.266328
C11orf68	0.128538	0.09135	0.266386
SCAF1	-0.06477	0.091417	0.266541
TBRG1	0.168418	0.091444	0.266541
TRIM65	0.042182	0.091448	0.266541
GTF3C4	0.128579	0.091452	0.266541
DNASE2	0.094349	0.091483	0.266595
LOC10192(	0.010526	0.091595	0.266885
ELP2	-0.16597	0.091623	0.266908
SLC30A7	-0.15971	0.09163	0.266908
ZNF782	-0.14748	0.09164	0.266908
FAM71E2	-0.01312	0.091673	0.266967
MKL1	-0.04553	0.09172	0.267052
CNTN6	-0.0188	0.091726	0.267052
MEX3B	0.339491	0.091799	0.267218
UBE3B	-0.0878	0.09181	0.267218
SKIV2L	-0.15369	0.091831	0.267218
ZNF25	0.170724	0.091843	0.267218
RP11-285F	-0.20405	0.091845	0.267218
STEAP3	0.266205	0.091898	0.267335
PALM	0.076436	0.09192	0.267366
CNOT2	-0.10393	0.091949	0.267389
STOX1	-0.12932	0.091958	0.267389
UBE2D2	0.152037	0.091965	0.267389
LPIN3	-0.05512	0.091985	0.267403
TMEM260	-0.13517	0.091995	0.267403
WDR11-AS	-0.07336	0.09204	0.267465
LINC00337	0.032853	0.092041	0.267465
LOC10192{	-0.13922	0.092097	0.267593
DNAJB9	-0.25635	0.09211	0.267593
SLC20A2	0.075539	0.092192	0.267795
LOC28367	0.002827	0.092274	0.267998
NNAT	0.086242	0.092343	0.268141
PTRHD1	0.161578	0.092361	0.268141
TGM4	0.015384	0.092366	0.268141
BHLHE22	0.060035	0.092373	0.268141
GLI1	-0.01899	0.092422	0.268249
ZBED3-AS	0.090622	0.092517	0.268489
LYPD1	0.127114	0.09257	0.268606
TRIM44	0.120327	0.09263	0.268698
SLC39A8	0.066122	0.092633	0.268698
EML3	0.109968	0.092639	0.268698
CDKL2	0.023063	0.092708	0.268861
LOC10192(	-0.03021	0.09272	0.268862
TUBD1	0.200822	0.092779	0.268996
LPHN1	0.133042	0.092852	0.269171
RP5-1061H	-0.02974	0.092935	0.269375
TPRA1	0.20302	0.092992	0.269505
LIN7A	0.184521	0.093006	0.26951
LOC10192{	0.033145	0.093021	0.269517
ATG12	0.161682	0.093059	0.26959
HERC1	0.126558	0.093087	0.269637
KLHL24	-0.1997	0.093105	0.269652
RP11-440L	-0.03931	0.093215	0.269907
HIST1H3B	-0.28002	0.093218	0.269907
LOC10192	-0.0366	0.093252	0.26997

LINC00928	-0.01854	0.093336	0.270158
EDAR	-0.05739	0.093342	0.270158
ZNF792	-0.27013	0.0934	0.270291
ANKMY2	-0.11958	0.093442	0.270375
LOC10192	-0.04032	0.093499	0.270503
KRT25	-0.03158	0.093544	0.270578
RAPSN	0.025548	0.093549	0.270578
STAU1	0.135379	0.093567	0.270591
NREP	-0.25991	0.09366	0.270825
LDLRAD4	-0.29775	0.093683	0.270829
LIMCH1	0.257096	0.093686	0.270829
CASC15	-0.05064	0.093722	0.270863
FOXG1	0.010909	0.093726	0.270863
SLC12A2	-0.18658	0.093736	0.270863
SLC16A10	0.298023	0.093787	0.270975
NCBP2-AS	0.128556	0.093802	0.270983
OXA1L	-0.15113	0.093914	0.271269
CAPNS2	0.038663	0.09396	0.271367
TCF4	0.238531	0.094026	0.271457
DCLK1	0.093042	0.094027	0.271457
TPSG1	-0.03191	0.094029	0.271457
EMC3	0.156928	0.094095	0.271583
MSRB3	0.292304	0.094098	0.271583
SOCS5	-0.20113	0.094157	0.271717
ME3	-0.12552	0.094238	0.271888
ACVR2B	-0.17246	0.094243	0.271888
C7orf61	0.097057	0.094268	0.271888
DCBLD2	0.120211	0.094273	0.271888
THSD7B	0.027381	0.094279	0.271888
HLA-F-AS	0.126128	0.094329	0.271996
NOC3L	-0.14229	0.09435	0.27202
ACADM	-0.21716	0.094377	0.272052
JKAMP	-0.1053	0.094386	0.272052
TCEAL3	-0.17982	0.094406	0.272073
CIDEC	0.091024	0.094432	0.272113
ARV1	-0.08951	0.094483	0.272212
PTGDR2	-0.07514	0.094492	0.272212
CPSF4	-0.17641	0.094532	0.272291
VRK1	-0.15234	0.094592	0.27243
AFAP1-AS	-0.03307	0.094642	0.272499
LOC10192	-0.0255	0.094642	0.272499
AC016831.	-0.19572	0.094656	0.272503
HMGB3	0.223833	0.094698	0.27259
SEL1L3	0.236659	0.094716	0.272604
SUCNR1	0.461432	0.094748	0.27266
C14orf183	-0.02361	0.094764	0.272669
POM121L8	-0.05273	0.094791	0.27271
IL12A	0.234176	0.094822	0.272731
BET1L	0.105411	0.094828	0.272731
SMAD3	-0.069	0.094835	0.272731
RP11-421F	-0.00911	0.09488	0.272822
EGOT	0.050308	0.094907	0.272864
SNORA74A	-0.03382	0.094929	0.27289
SPATA9	0.025305	0.095014	0.273099
LPP	-0.23814	0.095031	0.273111
LYRM5	-0.15937	0.095043	0.273111
PDZD3	-0.016	0.09512	0.273296
CA4	0.102049	0.095169	0.2734

LIG4	-0.22053	0.0952	0.273446
MRPS25	-0.1228	0.09521	0.273446
THNSL2	0.074786	0.095236	0.273483
INTS8	-0.11458	0.09526	0.273515
CARS2	-0.05937	0.095415	0.273869
CHODL	0.023078	0.095421	0.273869
MIB1	0.1511	0.095427	0.273869
SLC28A1	-0.01259	0.095434	0.273869
CABP5	-0.1281	0.095464	0.273919
ITGA7	0.064408	0.095567	0.274097
FBXW8	0.074309	0.095569	0.274097
ADRA2C	-0.10058	0.09557	0.274097
SNRK	-0.28068	0.095576	0.274097
C17orf105	-0.02482	0.095665	0.274316
NODAL	-0.02734	0.095722	0.274442
ARHGAP21	-0.21151	0.095739	0.274456
WDR12	-0.19075	0.095776	0.274524
C2orf15	-0.03238	0.095805	0.27457
CTB-167B5	0.094832	0.095833	0.274615
PSMA3	-0.21597	0.095889	0.274726
LINC01089	-0.10848	0.095906	0.274726
LOC10192	-0.01441	0.09591	0.274726
ANO1	-0.02468	0.095946	0.274794
DIAPH2-A	-0.0049	0.096062	0.27509
FDFT1	0.173928	0.096117	0.275209
LOC10050	0.032851	0.096148	0.275262
ATP11A	0.026802	0.096179	0.275287
CFC1B	-0.01014	0.096184	0.275287
PPP2R4	0.08182	0.096195	0.275287
SOX10	-0.01505	0.096219	0.27532
KDR	0.358456	0.096244	0.275323
ZNF510	-0.15635	0.096263	0.275323
LINC01254	-0.0174	0.096263	0.275323
TECR	0.086353	0.096271	0.275323
PRF1	0.202145	0.096369	0.275567
KCNA3	-0.20525	0.096398	0.275614
FGFR3	0.131403	0.096416	0.275628
GPR3	-0.04921	0.096462	0.275725
AP4S1	0.117735	0.096493	0.275776
ERGIC3	-0.08816	0.096526	0.275788
ZNF425	0.004257	0.096533	0.275788
CA7	-0.02405	0.096535	0.275788
PTS	0.175482	0.096615	0.275948
KRBA1	-0.11152	0.096617	0.275948
HIST1H2AI	-0.14435	0.096687	0.276113
HDAC11	0.084969	0.096761	0.276241
RP11-521D	-0.03764	0.096764	0.276241
FNBP1	-0.20404	0.096776	0.276241
AP000347.	-0.16713	0.096783	0.276241
PFAS	0.182587	0.096807	0.276272
QSOX1	0.102689	0.096821	0.276277
LSM14B	0.039548	0.096854	0.276307
TTC33	-0.08707	0.096865	0.276307
STARD7	-0.12122	0.096896	0.276307
TCP10	-0.01335	0.0969	0.276307
MRPL32	0.137714	0.096903	0.276307
FAM228A	0.03307	0.096908	0.276307
TMC7	-0.02381	0.097	0.276532

TH	-0.01149	0.09705	0.276637
CAMSAP1	-0.17711	0.097078	0.276662
RP11-360/	-0.02871	0.097084	0.276662
NAT9	0.129491	0.097146	0.276773
CDKN1C	0.276959	0.097149	0.276773
OFD1	-0.15199	0.097209	0.276909
FBXO3	-0.10677	0.097371	0.277333
SLC51A	-0.01273	0.097411	0.27741
KLF8	-0.14687	0.097501	0.277631
CCDC120	0.024191	0.097533	0.277654
NOS3	-0.0107	0.097535	0.277654
KDELC1	-0.28906	0.097655	0.277913
WDR26	0.161068	0.097656	0.277913
IRAK4	0.122216	0.097694	0.277913
NPCDR1	-0.2513	0.097707	0.277913
LAMTOR3	0.127458	0.097719	0.277913
PNCK	-0.04654	0.097728	0.277913
TMTC1	0.074928	0.097733	0.277913
PHF20	0.201164	0.097739	0.277913
TXLNB	0.185444	0.097741	0.277913
FARP2	0.050718	0.097763	0.277939
CCDC122	-0.03591	0.097823	0.278074
BSPRY	-0.17861	0.097935	0.278353
DAK	0.099307	0.09798	0.278391
TMEM116	-0.07092	0.097995	0.278391
LOC10050(	-0.2308	0.097996	0.278391
TBC1D12	0.220287	0.097999	0.278391
ARHGDIB	0.244326	0.098042	0.278475
LQFBS-1	-0.01731	0.098206	0.278906
SNTG2	-0.01292	0.098226	0.278926
LOC15391(	-0.00981	0.098274	0.279026
GPHB5	0.009172	0.098304	0.279044
SLC4A11	-0.03111	0.098306	0.279044
EFCAB4A	0.066735	0.098371	0.279181
HIST1H2BI	0.343241	0.098381	0.279181
TMEM108	0.067551	0.09841	0.279229
LOC10192!	0.189079	0.098434	0.279259
PAX7	-0.01592	0.098472	0.279278
LOC10013(	-0.14593	0.098474	0.279278
TUSC8	-0.08406	0.098479	0.279278
NBPF4	-0.13141	0.098514	0.279324
ZNF451	-0.282	0.098521	0.279324
KIAA1257	-0.04343	0.098599	0.279507
HECTD1	0.200832	0.09862	0.279531
RP11-179E	0.005133	0.098634	0.279534
AGPAT2	0.188529	0.098674	0.279611
GNAT2	0.0237	0.098687	0.279612
PAM	-0.23832	0.098723	0.279678
HES5	-0.03141	0.098774	0.279738
PTPRS	-0.02419	0.098784	0.279738
GAS6-AS1	-0.27472	0.098795	0.279738
LAMA5	0.204526	0.098796	0.279738
SLC22A24	0.004792	0.09887	0.279908
ESD	-0.1108	0.098882	0.279908
PSMB6	0.159345	0.098909	0.279946
LEMD1	-0.02356	0.098944	0.280008
MXRA5	0.161792	0.098967	0.280039
PLA2G1B	-0.07923	0.098987	0.280059

EGFEM1P	-0.35989	0.09906	0.280206
TYW3	-0.15004	0.099071	0.280206
PARD6A	0.156505	0.099078	0.280206
LOC10272:	-0.00977	0.099101	0.280233
MAU2	0.036934	0.099118	0.280247
PFN2	-0.36361	0.099296	0.280713
TUBA4B	-0.12003	0.099331	0.280774
ADNP	-0.09976	0.099358	0.280813
ADORA2B	0.296337	0.099412	0.280931
DIS3L2	-0.15115	0.099471	0.28106
P2RY2	0.268679	0.099488	0.281071
ACKR2	0.007564	0.099603	0.281358
SCML1	0.164791	0.099707	0.281617
BC053951	-0.01861	0.099731	0.281647
RP11-194N	0.088488	0.099775	0.281716
KCNJ2-AS:	0.018037	0.099781	0.281716
KHK	-0.07571	0.099822	0.281794
RGS7BP	-0.00722	0.099835	0.281796
SH2D3C	0.185595	0.099924	0.282009
AC100830.	0.055033	0.099937	0.282009
TIRAP	0.026542	0.100023	0.282185
LINC00410	-0.04019	0.100026	0.282185
LOC10192	-0.03394	0.100056	0.282235
LOC40216	-0.01292	0.100083	0.282273
GLB1L	0.334788	0.100138	0.282392
AKAP1	0.155373	0.100162	0.282411
SNRK-AS1	-0.16919	0.100171	0.282411
C10orf82	-0.09987	0.100193	0.282433
TUSC1	-0.29678	0.100205	0.282433
POGK	-0.21217	0.100332	0.282738
NHLH2	-0.01759	0.100339	0.282738
CC2D2B	-0.04746	0.100379	0.282814
OR51I2	-0.03617	0.100411	0.282868
RP11-33O	-0.15463	0.100428	0.282878
LINC01442	0.012281	0.100446	0.28289
ASTE1	0.142563	0.100469	0.28289
CNGB3	-0.04597	0.100471	0.28289
NF1	-0.07574	0.10054	0.283028
LOC65227	-0.02841	0.100547	0.283028
C4BPB	0.041471	0.100594	0.283126
LINC00628	-0.02414	0.100624	0.283174
FAM117B	-0.1539	0.100639	0.283179
ZNF664	-0.07863	0.100666	0.283216
C5orf27	-0.02636	0.100882	0.283789
PIP4K2B	0.079183	0.100919	0.283852
NUP188	-0.08788	0.100931	0.283852
NRXN2	0.286912	0.100987	0.28396
TNPO2	0.152206	0.101003	0.28396
FGB	0.018164	0.10102	0.28396
MPZL1	0.109144	0.101022	0.28396
HP	0.453183	0.101064	0.284042
LOC10099	0.02837	0.10112	0.284161
LINC00563	-0.03283	0.101157	0.28423
DPH3P1	-0.01741	0.101231	0.284401
XRN2	0.169704	0.101262	0.284449
CXorf38	0.148749	0.101278	0.28446
NENF	-0.05464	0.101335	0.284581
C9orf78	0.083815	0.101361	0.284618

TPM3	-0.20587	0.101427	0.284753
URB1	-0.14093	0.101442	0.284753
DSCC1	-0.11465	0.101449	0.284753
ZNF197	-0.10915	0.101511	0.284887
C16orf78	-0.02103	0.101531	0.284887
KBTBD2	0.21276	0.101536	0.284887
SMCO3	-0.02553	0.101592	0.285009
XXYLT1	0.239279	0.101694	0.285258
MLLT3	-0.28445	0.101795	0.285503
MXRA8	0.085581	0.101814	0.285519
IL36RN	-0.04516	0.101924	0.285792
LOC10013:	-0.04972	0.101989	0.285936
RP11-631M	0.100778	0.102066	0.286115
SEC11C	0.110155	0.102104	0.286164
ITPKA	0.137822	0.10211	0.286164
DDIT4L	0.165782	0.102124	0.286167
FAM173A	0.189174	0.102161	0.286232
FAM43B	0.010698	0.102244	0.286428
LAMTOR5-	0.110531	0.102299	0.286545
DPP8	-0.09933	0.102352	0.286658
PPTC7	-0.16573	0.102449	0.286892
IL17RA	0.226179	0.102492	0.286975
B9D1	0.104305	0.102532	0.287051
C20orf194	0.187156	0.102645	0.287329
GJB1	0.070836	0.102711	0.28747
LOC10272:	-0.03078	0.102733	0.28747
NCL	0.049228	0.102735	0.28747
RNF144A	-0.07022	0.10276	0.287502
CPEB2	0.193246	0.102787	0.287542
LOC10192{	-0.02237	0.102909	0.287846
LOC10192{	-0.01826	0.10297	0.287967
GRIP1	0.128208	0.102979	0.287967
ANKRD45	0.075147	0.103036	0.288059
DYNC1LI2	0.151087	0.103039	0.288059
CCDC63	0.019076	0.103089	0.288162
MGC34796	-0.05467	0.10313	0.28824
TBC1D31	-0.09923	0.103152	0.288262
ACADS	0.069782	0.103165	0.288262
WDR77	0.169588	0.103242	0.28844
RP11-1017	0.052739	0.103258	0.288448
MAPK9	0.087014	0.103316	0.288564
RP11-999E	-0.26647	0.103333	0.288564
HMGCS2	0.016251	0.103339	0.288564
RP11-513M	-0.07454	0.103363	0.288594
RAB8B	0.235824	0.103418	0.288708
DYDC2	-0.0074	0.103539	0.289008
STARD3NL	-0.11239	0.103552	0.289008
TAF5	-0.17167	0.103581	0.289052
DOLK	0.198173	0.103596	0.289058
PPP1R12C	-0.03125	0.103645	0.289156
COA3	0.084609	0.103664	0.289173
ARAF	0.140829	0.103706	0.289253
RAB39B	0.14005	0.103776	0.289341
RNF185	0.145087	0.103781	0.289341
PRSS36	-0.04594	0.103787	0.289341
NAP1L3	-0.46037	0.103791	0.289341
LOC10192!	-0.00645	0.103815	0.289371
SNRNP48	-0.12927	0.103855	0.289406

PTAR1	-0.19046	0.103855	0.289406
LOC38983	0.143191	0.103879	0.289438
COPS8	0.089861	0.103991	0.289711
C21orf58	0.04326	0.104024	0.289757
MICALCL	0.097907	0.104046	0.289757
ZBTB39	0.080007	0.104048	0.289757
FURIN	0.153051	0.104112	0.289899
RP11-1078	-0.02493	0.104291	0.290361
RP4-635E1	-0.2101	0.104305	0.290363
LINC00314	-0.02806	0.104366	0.290494
PFDN2	0.122403	0.104387	0.290514
C1orf101	-0.04269	0.104496	0.290781
RAB6B	0.251762	0.104521	0.290786
MAP2K4	0.102725	0.104524	0.290786
MGC10814	-0.03354	0.10454	0.290791
CYSRT1	0.014827	0.104613	0.290941
CUL7	-0.0637	0.10462	0.290941
CXCL14	0.033476	0.104649	0.290983
NCK2	-0.15496	0.104684	0.291042
HSCB	0.159671	0.104713	0.291087
TRPV4	0.053967	0.104776	0.291223
MOB2	0.140535	0.104851	0.291355
SLC9A3R1	-0.14839	0.104866	0.291355
ERCC8	0.087932	0.104882	0.291355
HIST1H4L	-0.04403	0.104893	0.291355
UBE2U	-0.0129	0.104896	0.291355
EIF2B1	0.075327	0.104904	0.291355
RSPO3	0.051487	0.104962	0.29148
PIANP	-0.02673	0.105014	0.291548
LOC44145	-0.01687	0.105022	0.291548
NPHP3	-0.24127	0.105027	0.291548
FAM169B	-0.01837	0.105118	0.291738
C8orf49	-0.01394	0.105123	0.291738
PHTF2	0.073701	0.105224	0.291982
TBCCD1	0.195235	0.105295	0.292141
CS	0.161298	0.105308	0.292141
ZNF530	-0.07906	0.105334	0.292146
SAP30L	-0.1124	0.105337	0.292146
PIK3R5	0.069134	0.105367	0.292193
WWP2	-0.07123	0.105422	0.292306
LOC10192	0.007148	0.105503	0.292494
IFI16	0.159419	0.10557	0.292642
SPIN1	0.141338	0.105636	0.292786
CLEC10A	0.277955	0.10572	0.292982
RP4-613A2	-0.05944	0.105756	0.293044
ATG14	0.137813	0.105831	0.293215
LINC00582	0.03678	0.105863	0.293266
GNB1L	0.071584	0.105942	0.293448
LRRD1	0.013484	0.106023	0.293611
CRISP3	0.590218	0.106028	0.293611
FLYWCH1	0.13985	0.106042	0.293611
RP11-108F	-0.04218	0.106076	0.293669
ACOX2	0.195607	0.106093	0.293678
CAND1.11	0.018339	0.106178	0.293875
GCH1	0.294105	0.106287	0.29414
KMT2E-AS	-0.07742	0.106316	0.294181
LOC64335	-0.00633	0.106409	0.294338
ZNF160	-0.14791	0.10641	0.294338

PSMC3IP	0.148294	0.106413	0.294338
MMD	0.228728	0.106476	0.294475
C9orf72	-0.23933	0.106515	0.294546
POGZ	-0.1353	0.106551	0.294607
RNF186	-0.00864	0.106574	0.294632
DLEC1	-0.00995	0.106699	0.294931
SYT12	-0.04987	0.106709	0.294931
JTB	-0.0603	0.106738	0.294974
LCN12	-0.0408	0.106769	0.295023
MYH8	-0.01855	0.106821	0.295127
PLVAP	0.0634	0.106956	0.295463
MRPL24	0.190142	0.107	0.2955
LCOR	-0.1561	0.107002	0.2955
PLIN5	-0.01232	0.107015	0.2955
RPS6KA2	0.253924	0.107024	0.2955
AMZ2	0.11888	0.107052	0.29554
UTP11L	0.06389	0.107104	0.295646
TMEM55A	-0.22568	0.107161	0.295764
ZDHHC13	-0.11414	0.107199	0.295831
DTWD2	-0.12578	0.107411	0.296378
AX747652	-0.18765	0.10744	0.296421
RNF212B	-0.16203	0.10749	0.296506
LCMT1	-0.15034	0.107498	0.296506
PVRL4	-0.04658	0.107538	0.296578
TAF1A-AS1	0.090962	0.107565	0.296611
KRTAP7-1	-0.03385	0.107577	0.296611
EPN2-AS1	-0.02361	0.10761	0.296663
TRERF1	0.094466	0.107645	0.296721
FAM9B	0.003801	0.107667	0.296744
ZNF2	-0.15252	0.10775	0.296935
MCCC1	-0.10501	0.107781	0.296984
BC042366	-0.06564	0.107848	0.297121
TMEM132A	-0.0259	0.107861	0.297121
PREP	0.117702	0.107872	0.297121
SLC6A5	-0.01772	0.107914	0.297198
LINC00304	0.021604	0.108022	0.297458
IFT140	0.099812	0.108066	0.297482
CCL17	-0.04434	0.108071	0.297482
PF4	0.668905	0.108072	0.297482
LOC390705	-0.04929	0.108223	0.297852
OXCT1	-0.17469	0.108234	0.297852
FAM162A	0.143559	0.108342	0.298111
VPS25	0.144177	0.108357	0.298116
MS4A3	0.519806	0.108371	0.298117
YEATS4	-0.25119	0.108411	0.298188
HSD17B12	-0.14003	0.108441	0.298232
AC144652.	-0.20607	0.108524	0.298409
DYNLRB1	-0.04249	0.108533	0.298409
DCLK3	0.029395	0.108567	0.298467
ZNF674	-0.00741	0.108633	0.298608
PM20D1	0.030681	0.108668	0.298608
RNF157-AS	0.00293	0.108681	0.298608
FOXL2	0.027658	0.10871	0.298608
LINC00475	-0.0391	0.10871	0.298608
TMEM51	0.1913	0.108713	0.298608
RBM23	0.113437	0.108715	0.298608
CBR4	-0.15324	0.108788	0.29877
HGD	-0.27795	0.108847	0.298841

TUBGCP6	0.075359	0.10886	0.298841
TMEM79	-0.08795	0.108871	0.298841
TMEM120E	0.082773	0.108879	0.298841
LOC10012!	0.101603	0.108883	0.298841
RPTN	0.003374	0.108918	0.298901
NEO1	0.135688	0.109035	0.299164
ANTXR2	-0.15664	0.109042	0.299164
RITA1	0.201164	0.109056	0.299166
CSRNP1	0.265811	0.109077	0.299184
OLIG2	-0.01158	0.10911	0.299237
ITPA	0.146577	0.109136	0.29927
BC017209	-0.01345	0.109153	0.299279
HAP1	-0.01893	0.109224	0.299437
LRP8	0.204049	0.109263	0.299505
APC2	-0.01716	0.109312	0.299602
RP11-196C	-0.2019	0.109327	0.299606
MAP3K13	0.038656	0.109374	0.299696
LOC10027:	-0.29656	0.109403	0.299737
KCNH4	-0.01576	0.10946	0.299855
PLXNB1	0.043547	0.109483	0.299858
SLC38A5	0.228649	0.109489	0.299858
STK35	0.088305	0.109544	0.299935
LINC01350	-0.02239	0.109552	0.299935
ZNF445	-0.08137	0.109558	0.299935
ARL2	0.186753	0.109659	0.300173
AC073321.	0.017579	0.109766	0.300427
LRTM2	0.016661	0.109828	0.300539
LINC00476	-0.18141	0.109834	0.300539
C22orf24	0.011772	0.109872	0.300604
PTPN21	0.003393	0.109899	0.300622
RP11-439E	0.007681	0.109906	0.300622
AK090844	-0.10653	0.109957	0.300723
BCL2L13	0.133086	0.109999	0.3008
CCDC157	0.044089	0.110041	0.300854
LINC01304	0.011395	0.11006	0.300854
CCDC80	0.074181	0.110061	0.300854
NUP210	0.212749	0.110286	0.301433
C2orf16	0.033146	0.11034	0.301532
LOC10192!	-0.07723	0.110351	0.301532
KRT9	0.00427	0.110365	0.301532
TNFSF13	0.194349	0.110387	0.301532
MAP1LC3E	-0.01215	0.110392	0.301532
LINC01146	0.004764	0.11043	0.301559
KLHDC3	0.213859	0.11043	0.301559
DMD	-0.02092	0.110463	0.30161
NEK9	-0.01939	0.110479	0.301616
PCDHGC3	-0.01124	0.110537	0.301737
FAM13C	0.039593	0.110627	0.30194
GABRA2	0.13155	0.110639	0.30194
CCNA2	0.274835	0.110719	0.30212
SPOCD1	-0.07591	0.110796	0.302291
TMEM30B	-0.04771	0.110869	0.30245
BC047615	-0.00384	0.110882	0.30245
NAALADL2	-0.02082	0.110913	0.30249
PP7080	0.218343	0.110925	0.30249
EHD4	0.110863	0.111014	0.302696
CTD-2196E	-0.1158	0.11108	0.302788
ACD	0.176013	0.111107	0.302788

TET2	0.197799	0.111116	0.302788
RP11-454K	-0.00729	0.111116	0.302788
BABAM1	0.134641	0.111118	0.302788
DKFZp564I	0.006551	0.111189	0.302943
WNT2B	-0.01098	0.111266	0.30308
CHRDL2	-0.0235	0.111267	0.30308
RP11-320H	0.18726	0.111337	0.303233
FAM117A	0.216872	0.111381	0.303315
RNF26	0.114716	0.111423	0.303392
OR10A5	0.008907	0.111485	0.303522
PIM1	0.198947	0.111547	0.303652
EFHB	0.063385	0.111575	0.303683
UTP23	-0.10538	0.111586	0.303683
ANO9	0.100879	0.111699	0.303902
CITED1	0.029927	0.111704	0.303902
NGLY1	-0.17199	0.111709	0.303902
ANKRD28	-0.23619	0.111808	0.304133
TRIM15	-0.02206	0.111903	0.304353
LOC28542	-0.01686	0.111918	0.304356
ARSG	0.087244	0.112007	0.304558
CCDC173	-0.26486	0.112021	0.30456
TCEAL8	0.166776	0.112038	0.304568
LOC10050	-0.03767	0.112072	0.304591
ZNF671	-0.17151	0.112075	0.304591
RP11-109E	-0.01207	0.112112	0.304653
FAM160B2	0.05712	0.112188	0.304823
GLI4	0.005573	0.112205	0.304829
TOE1	-0.11809	0.112246	0.304902
LOC44093	0.16651	0.112513	0.30559
RP11-226L	-0.22243	0.112559	0.305676
HDGFL1	-0.0723	0.112622	0.305808
BRINP2	0.024465	0.112644	0.305831
CTD-2587H	0.013721	0.112684	0.305902
C1orf95	0.053864	0.112712	0.30594
GZMB	0.338421	0.112802	0.306127
CNTN2	0.006863	0.112818	0.306127
SMAP2	0.164035	0.112824	0.306127
FAM167A	-0.01659	0.112848	0.306154
MYRIP	0.146359	0.112908	0.306279
CTPS1	-0.21808	0.11295	0.30634
LINC00574	0.003397	0.112959	0.30634
CAP2	-0.00503	0.11304	0.306484
PAXIP1	-0.15112	0.11304	0.306484
PRDM12	-0.05138	0.113074	0.306538
LOC44102	-0.03636	0.113179	0.306783
ABCA7	0.152511	0.113241	0.306914
NCOA2	-0.13311	0.113267	0.306944
SCPEP1	0.168054	0.113309	0.307019
PAXBP1	-0.15726	0.113325	0.307026
GUSBP5	-0.19284	0.113365	0.307095
LOC10192	-0.01255	0.113387	0.307096
EGLN3	0.232343	0.113393	0.307096
TBC1D22A	0.127456	0.113436	0.307173
TM4SF20	-0.03311	0.11349	0.307206
ELOVL3	0.002805	0.113495	0.307206
DCK	-0.19474	0.113495	0.307206
HUNK	-0.03771	0.113512	0.307206
LOC10013	-0.28362	0.113519	0.307206

LOC101928	-0.02576	0.113691	0.30759
DLC1	-0.06262	0.113702	0.30759
LOC145678	0.018251	0.113711	0.30759
OPN5	-0.02235	0.113718	0.30759
ZNF273	-0.19958	0.113804	0.307755
PAXIP1OS	0.109038	0.113808	0.307755
LOC283745	-0.00313	0.113849	0.307829
GRHL2	-0.00913	0.113868	0.30783
ADAM30	-0.00684	0.113878	0.30783
NDUFB5	0.095606	0.113922	0.307879
IFT43	0.133565	0.113925	0.307879
SRP14	0.090734	0.114007	0.308064
TFCP2L1	0.04161	0.114065	0.308148
PPP1R27	0.084706	0.114067	0.308148
CDC42SE1	0.125433	0.114137	0.308298
BC033241	0.023031	0.114154	0.308301
FAM200B	-0.15638	0.114166	0.308301
IL4I1	0.092148	0.114249	0.308377
PSMD6-AS	-0.15782	0.114259	0.308377
FMR1NB	-0.01095	0.114262	0.308377
POTEM	0.005088	0.114263	0.308377
AK056982	0.016771	0.114266	0.308377
TSKU	-0.07487	0.114316	0.308467
HMOX2	0.125552	0.114327	0.308467
DNAJC7	0.148841	0.114369	0.308506
PPIL1	0.204355	0.11437	0.308506
CETN2	-0.13226	0.114515	0.308847
EDC3	-0.13934	0.114525	0.308847
KRT20	-0.02492	0.114545	0.308861
GABRR3	0.016687	0.114581	0.30892
FOSB	0.339833	0.1146	0.308934
DDHD1	0.155615	0.114623	0.308958
LOC101927	0.002269	0.114693	0.309108
AC013463.	0.031541	0.114744	0.309173
C11orf49	0.057716	0.114758	0.309173
NALCN-AS	-0.01834	0.114766	0.309173
PURB	0.013601	0.114774	0.309173
LOC101927	0.039155	0.114877	0.309377
DHTKD1	0.12942	0.114879	0.309377
ERF	0.065267	0.114935	0.30948
BTF3L4	-0.09387	0.114946	0.30948
SLC17A4	-0.02116	0.114976	0.309523
RP11-749H	0.02605	0.115024	0.309613
YWHAH	0.225864	0.115085	0.309739
RHOB	0.002285	0.115118	0.30979
CLSTN1	-0.16647	0.115151	0.309801
ZBTB14	0.071314	0.115151	0.309801
TNFRSF10A	0.127327	0.115246	0.31002
ARHGAP27	0.004589	0.115301	0.310114
OSBPL8	-0.16941	0.115319	0.310114
ZNF823	-0.1959	0.115328	0.310114
TMEM229E	-0.02373	0.115339	0.310114
RAD23B	0.140888	0.115467	0.310422
FLJ32955	-0.02974	0.115496	0.31046
SPATA19	-0.01522	0.115524	0.310484
GLYCTK	0.053359	0.115534	0.310484
SLC22A4	0.390476	0.115579	0.310567
PTGS2	0.461413	0.11565	0.310717

UBE3A	0.123679	0.115663	0.310717
PRKG2	-0.32005	0.115695	0.310764
UBE2W	-0.16563	0.115751	0.310828
LOC79999	-0.02708	0.115757	0.310828
HEPH	-0.0314	0.115774	0.310828
PRR24	0.164554	0.115777	0.310828
RPL23AP53	0.026902	0.115819	0.310903
LOC72844!	0.016509	0.115835	0.310907
LCN2	0.480221	0.115852	0.310914
CDC42BPG	-0.01179	0.115912	0.311039
MMP16	-0.02281	0.11594	0.311073
INTS6-AS1	-0.16281	0.11601	0.311225
NDOR1	0.035434	0.116046	0.31126
GPR55	-0.02278	0.116052	0.31126
LINC00663	-0.04363	0.11622	0.311671
LAMTOR5	0.071728	0.11626	0.311716
SYT13	-0.0222	0.116268	0.311716
CRABP1	0.043893	0.11628	0.311716
SLC8A1	0.012184	0.116301	0.311733
AFG3L2	-0.13751	0.116397	0.311952
C14orf2	-0.10338	0.116422	0.311982
GTF3C2-A!	-0.00698	0.116461	0.312048
PARP1	-0.13234	0.116483	0.312067
OR2C3	0.019445	0.116502	0.312078
SLC10A7	-0.0652	0.116537	0.312127
LOC10192!	-0.01316	0.116548	0.312127
MED14	-0.13898	0.116571	0.312149
TM7SF3	0.181906	0.116593	0.312169
PLA2G6	0.115996	0.116648	0.312278
KIR3DX1	-0.03772	0.116715	0.312417
DGCR6L	-0.02052	0.116897	0.312866
LOC10192	0.142622	0.11692	0.31289
SOX9	0.00925	0.116964	0.312968
GPR161	0.106435	0.117015	0.313067
LOC28388	0.052392	0.117061	0.313147
LOC10192!	0.090209	0.117079	0.313147
RP11-5C23	0.17408	0.117111	0.313147
RIBC2	-0.16678	0.117117	0.313147
IRAK3	-0.18779	0.117146	0.313147
UHMK1	0.245115	0.117155	0.313147
OLFM2	0.014361	0.117157	0.313147
IQCK	-0.0439	0.117174	0.313147
SPATA2	0.124866	0.117187	0.313147
EGFLAM-A	0.007328	0.11719	0.313147
SLC26A4	-0.01973	0.117253	0.313278
FHIT	0.24882	0.117338	0.313466
RP11-332H	-0.00882	0.11743	0.313674
LINC00587	-0.0232	0.117467	0.313696
LCTL	0.00344	0.117467	0.313696
CPT1C	0.05002	0.117505	0.313758
CTD-2251I	-0.01253	0.117547	0.313812
AC068039.	-0.14977	0.117555	0.313812
BC036311	-0.02314	0.117575	0.313829
C3orf80	-0.36704	0.117602	0.313863
CAMK2N2	-0.01549	0.117656	0.313966
LOC10192!	0.008931	0.1177	0.314045
LOC10192!	0.00216	0.117714	0.314045
ANAPC5	-0.13116	0.117753	0.314073

DCAF15	0.053595	0.117754	0.314073
TMEM132I	-0.01202	0.117778	0.314073
POLE3	-0.13867	0.117783	0.314073
MIRLET7BH	0.068397	0.117813	0.314114
RP11-502M	0.003738	0.117868	0.314209
STK11	0.059539	0.117877	0.314209
LOC10192	0.022353	0.117894	0.314214
NDNF	0.024872	0.117928	0.314267
LPPR1	0.01986	0.117971	0.314343
RTDR1	-0.01563	0.118059	0.314538
RP1-8B22.	0.040386	0.118088	0.314556
TAC3	0.026799	0.118108	0.314556
AIMP2	-0.14546	0.118109	0.314556
4-Sep	0.007857	0.118172	0.314685
DOCK7	-0.11451	0.118224	0.314772
LOC65316	0.0149	0.118234	0.314772
INSL5	0.008495	0.118346	0.315032
CADM2	0.005327	0.11838	0.315083
LINC0101C	-0.01558	0.118438	0.315171
OR2W1	0.002177	0.118442	0.315171
UNC79	-0.00642	0.118498	0.315273
ASXL1	-0.1368	0.118532	0.315273
CHCHD5	-0.04007	0.118534	0.315273
LOC10192	-0.0034	0.118539	0.315273
LOC10013	-0.14561	0.11858	0.315333
TRPM2-AS	-0.03486	0.118605	0.315333
COQ4	0.135132	0.118613	0.315333
NUMB	0.199263	0.11862	0.315333
TUBA1A	-0.14311	0.118641	0.315353
MINOS1	0.091557	0.118693	0.315451
RP11-286E	0.003555	0.118729	0.315469
TDO2	0.075499	0.118729	0.315469
LOC10192	-0.03138	0.118828	0.315692
HTR3B	-0.00671	0.11885	0.315714
LOC64732	0.002512	0.118879	0.315727
MIER1	-0.1401	0.118884	0.315727
RGS9BP	0.021286	0.118965	0.315904
AGER	0.025291	0.119054	0.316095
STPG2	-0.01219	0.119066	0.316095
KLHL30-AS	-0.02193	0.119098	0.316139
KIAA1456	-0.00874	0.119122	0.316145
PPP1R1A	-0.01324	0.119131	0.316145
IZUMO2	-0.0156	0.119147	0.316145
LOC64626	-0.02036	0.119159	0.316145
RBM26-AS	-0.12618	0.119185	0.316159
CTDSPL2	-0.11348	0.119193	0.316159
CNTFR-AS	-0.01685	0.119284	0.316361
AATK	-0.10814	0.119355	0.316465
LOC10192	0.022001	0.119364	0.316465
RP1-140C	0.017075	0.119367	0.316465
PRR18	-0.00513	0.119394	0.316499
WDR86	0.029104	0.119429	0.31653
Dbpht2	-0.01393	0.119459	0.31653
KIAA0930	0.161877	0.119465	0.31653
MGC57346	-0.09647	0.119476	0.31653
ALDOC	0.22619	0.119487	0.31653
TREML3P	-0.05213	0.119494	0.31653
RP11-305K	-0.1701	0.119559	0.316655

LOC10050!	-0.00452	0.11957	0.316655
CHEK1	-0.23124	0.119597	0.316689
DNAJC2	-0.07244	0.119657	0.316799
ZFR	0.165159	0.119668	0.316799
NFASC	0.009724	0.119684	0.316803
ARAP3	0.058866	0.119715	0.316823
ZMYM4	0.117409	0.119721	0.316823
KLB	0.00633	0.119743	0.316842
TMA7	0.088232	0.119781	0.316904
EIF5B	-0.14018	0.119868	0.317052
FAM69A	0.15465	0.11987	0.317052
RABEP2	0.122644	0.119886	0.317052
VAPA	0.166507	0.119896	0.317052
TROVE2	-0.06904	0.11993	0.317095
BTBD19	0.08459	0.119941	0.317095
FRG1B	-0.09581	0.119971	0.317136
AC092667.	-0.01878	0.12002	0.31717
LOC10192!	-0.09359	0.120038	0.31717
SIRT6	0.057557	0.120041	0.31717
ZNF449	-0.10357	0.120043	0.31717
BCKDHB	-0.09023	0.120132	0.31729
LOC10192!	-0.156	0.12014	0.31729
LOC10050	0.011069	0.120146	0.31729
LINC01137	-0.22119	0.120147	0.31729
CACFD1	-0.04944	0.12017	0.317312
LOC73020	0.056187	0.120189	0.317325
ALDH2	0.192548	0.120234	0.317365
SOX7	0.117393	0.120247	0.317365
LOC10013	0.057591	0.120249	0.317365
ITSN2	0.148243	0.120302	0.317455
LOC10050	-0.02463	0.120324	0.317455
MFSD2A	0.103181	0.120332	0.317455
PLXDC1	0.022062	0.12035	0.317455
S100A3	0.006465	0.120368	0.317455
SEC24D	0.183562	0.120371	0.317455
NUTM1	-0.02961	0.120396	0.317483
FANCB	0.097009	0.120447	0.31758
WDR74	-0.14415	0.12048	0.317627
GANC	-0.07827	0.120533	0.31773
NBPF20	0.105683	0.120652	0.318003
SLC44A1	0.159933	0.120702	0.318098
C2orf42	-0.18313	0.120838	0.318417
CCDC147	-0.04575	0.120869	0.318446
GLP1R	-0.02256	0.120886	0.318446
LOC15148	-0.01473	0.120893	0.318446
FUZ	0.054427	0.121064	0.318816
CENPO	-0.1549	0.121067	0.318816
TNFRSF18	-0.03231	0.121078	0.318816
MIR133A1I	0.027343	0.121116	0.318875
LOC10050!	-0.01735	0.12113	0.318875
APIP	-0.07226	0.121182	0.318975
C17orf74	0.003401	0.121232	0.319065
MTMR6	-0.17166	0.12134	0.319295
UTP18	-0.04172	0.121357	0.319295
ATP9B	0.031982	0.121363	0.319295
LOC10099	-0.01825	0.121477	0.319555
SLC16A2	0.188923	0.121511	0.319607
LOC10192!	-0.02529	0.121681	0.320016

SAG	-0.03287	0.121712	0.320057
LOC22007	0.008398	0.121804	0.320225
KIAA1656	-0.05467	0.121805	0.320225
HPS6	0.056403	0.121895	0.32042
LINC01242	0.115327	0.121923	0.32042
PARD6G-A	0.011888	0.121924	0.32042
JADE2	-0.21035	0.121941	0.320427
ATCAY	-0.05104	0.121975	0.320477
LOC10013	-0.0102	0.122007	0.320523
NLRP3	0.260163	0.122198	0.320985
CDH2	-0.23172	0.12224	0.32102
ALAD	0.245133	0.122241	0.32102
GPR135	0.02219	0.122286	0.321099
EFNA5	-0.01266	0.122369	0.321278
SENP5	0.151487	0.122395	0.321306
ZNF7	0.029974	0.122508	0.32156
C9orf24	-0.05515	0.122521	0.32156
RIOK2	-0.15671	0.122573	0.321657
CTB-12O2	-0.03286	0.122588	0.321657
WDR52	-0.17498	0.122609	0.321669
LOC10272	-0.05283	0.12263	0.321669
LOC10192	-0.01868	0.122637	0.321669
LINC00959	-0.2455	0.122654	0.32167
TNFSF14	0.085837	0.122667	0.32167
RP11-619L	-0.02386	0.122708	0.321694
ZBTB7B	0.024819	0.12271	0.321694
ELMSAN1	0.122244	0.122745	0.321694
KCTD21	0.160594	0.122748	0.321694
EFNB1	-0.02631	0.122763	0.321694
LOC10192	-0.02042	0.122765	0.321694
KIF5C	0.059271	0.12287	0.32193
ATP6V1C2	-0.00705	0.12299	0.322189
WARS2	0.134384	0.122999	0.322189
LGSN	0.123014	0.123023	0.322213
LCN1	-0.01546	0.123044	0.32223
LETM1	-0.09058	0.123061	0.322235
CST6	0.032335	0.123086	0.322235
SHB	-0.07848	0.123091	0.322235
LINC01049	-0.0309	0.123223	0.322542
GRPR	-0.05474	0.123288	0.322674
MIR4435-1	0.09778	0.123325	0.322724
DDX42	-0.19386	0.123337	0.322724
ADPRM	0.179681	0.12341	0.322877
ITGAE	-0.13272	0.123467	0.322951
TRUB1	0.028091	0.123477	0.322951
ACSS3	0.15721	0.123484	0.322951
SNAP91	0.00236	0.123656	0.323331
ZNF224	-0.19371	0.123659	0.323331
HIPK3	0.097601	0.123682	0.323352
PDE12	0.092795	0.123751	0.323463
CTC-459F4	-0.19589	0.123754	0.323463
ABCC2	0.038057	0.123775	0.323479
ACER3	0.156335	0.123867	0.323662
BPIFB2	0.027955	0.123875	0.323662
SLC25A22	0.10449	0.12389	0.323663
KIF20A	0.299821	0.123944	0.323698
KATNAL2	0.078825	0.123954	0.323698
RASGRP1	-0.36768	0.123956	0.323698

SDHAF1	-0.12085	0.123963	0.323698
FXR2	0.103479	0.124048	0.323876
OTX1	-0.01817	0.124062	0.323876
RPL13AP17	-0.01976	0.124193	0.32415
CNOT1	-0.17965	0.124196	0.32415
PLA2G3	-0.05701	0.124213	0.324154
OSMR	0.052515	0.124243	0.324195
IGHMBP2	0.051471	0.124326	0.32427
WDR86-AS	0.036586	0.124336	0.32427
HOXA3	-0.15874	0.124345	0.32427
LOC28408	0.004231	0.124347	0.32427
RNF25	0.091322	0.124347	0.32427
TMEM14B	0.144719	0.124452	0.324503
LYAR	0.170573	0.1245	0.324589
VPS13D	-0.14692	0.124558	0.324702
KLF3-AS1	-0.2253	0.124684	0.324993
LOC10192	0.023182	0.12472	0.325046
TMEM25	-0.03783	0.12489	0.325451
LINC01180	0.012503	0.124908	0.325458
WDR41	0.125393	0.124982	0.325581
CCT2	0.116625	0.124985	0.325581
TFIP11	0.112321	0.12502	0.325604
KCP	-0.0324	0.125024	0.325604
CTDP1	0.168422	0.125084	0.325714
FXYD7	0.018224	0.125107	0.325714
RP11-732A	0.086476	0.125111	0.325714
HEPACAM	0.159393	0.125128	0.325717
ABCG2	0.343157	0.125182	0.32579
CHKB	0.17204	0.125186	0.32579
MYL10	-0.04638	0.125288	0.326018
DQ583756	-0.02841	0.125376	0.326207
ZNF607	-0.12677	0.125421	0.326284
GFM1	0.120632	0.125602	0.326716
DISP1	0.065736	0.125621	0.326725
HOXC4	-0.096	0.125651	0.326736
SSR4	0.134931	0.125655	0.326736
PPP3CA	-0.08617	0.125696	0.326803
VIMP	0.153975	0.125741	0.326845
NVL	-0.11796	0.125742	0.326845
LMLN	0.101779	0.125773	0.326886
AAMDC	0.129712	0.125817	0.326962
NCAPG	0.257564	0.125865	0.327032
ZBTB32	0.005835	0.125875	0.327032
NEK4	-0.10936	0.125955	0.327195
FAT2	-0.02332	0.125967	0.327195
TDRD10	0.105071	0.126162	0.327635
RLN2	-0.23136	0.126167	0.327635
EIF2AK1	0.144235	0.126208	0.327669
CLDN10-A	0.010414	0.12621	0.327669
B3GNT4	-0.03661	0.126242	0.327704
PRLHR	0.006485	0.126264	0.327704
TAAR5	-0.02487	0.126269	0.327704
LYPD6B	0.089984	0.126335	0.327813
MIR663AH	0.00275	0.126342	0.327813
LOC10050	0.16083	0.126357	0.327814
TIMM8A	-0.08142	0.126411	0.327883
HNRNPU	0.153457	0.126414	0.327883
TUBGCP4	-0.11394	0.126496	0.328055

GDF5	-0.04256	0.126751	0.328678
WDR17	-0.17486	0.126815	0.328791
GEN1	0.185256	0.126856	0.328791
CDCA7	-0.20974	0.126857	0.328791
ADAMTS1	0.174704	0.126865	0.328791
RNF39	-0.02516	0.126872	0.328791
MEX3D	0.016668	0.126886	0.328791
GPR112	-0.00707	0.126972	0.328974
RAB33B	-0.19435	0.126997	0.328982
SRP72	-0.10205	0.127005	0.328982
TMPRSS5	-0.0214	0.127048	0.329056
ABL1	0.159981	0.127075	0.329085
MMRN1	-0.37556	0.127169	0.329288
LOC10012ⁱ	-0.03115	0.127209	0.329342
PTPN20B	-0.47728	0.127226	0.329342
LOC10050(	0.078051	0.127235	0.329342
KCTD3	-0.23086	0.127268	0.329386
ZBED6CL	-0.15728	0.127283	0.329386
FRMPD3	-0.05121	0.127372	0.329578
WNT6	-0.00614	0.127417	0.329645
PFKP	-0.08733	0.127438	0.329645
ZSCAN23	-0.03584	0.127455	0.329645
TLR6	0.09057	0.127466	0.329645
DMRT1	0.043246	0.127474	0.329645
PRORY	-0.02273	0.127507	0.329691
AX747031	0.002404	0.127543	0.329716
PUS3	0.121211	0.127547	0.329716
RGS10	-0.17821	0.127663	0.329938
LOC10192(	0.009118	0.127675	0.329938
YTHDF2	-0.08826	0.127678	0.329938
ORC6	-0.17367	0.127709	0.329976
SRRM2-AS	-0.02682	0.127728	0.329986
TCP10L	0.045881	0.127752	0.33001
SPDYE2	-0.23246	0.12788	0.330265
TMEM205	0.16988	0.127882	0.330265
SPTBN5	-0.01263	0.127905	0.330286
PPARA	0.085971	0.127941	0.330339
CDPF1	0.083817	0.128013	0.330487
OR4D1	-0.01924	0.128038	0.330502
PCDHB18	-0.01666	0.12805	0.330502
C14orf180	-0.02096	0.128114	0.330629
FAM84B	0.135699	0.128194	0.330795
ZBTB9	0.142395	0.128234	0.330859
CYR61	0.125576	0.12839	0.331222
KLF7	0.193202	0.128434	0.331265
INTS1	0.077482	0.128437	0.331265
SLC35A5	-0.13393	0.128488	0.331359
PSG9	-0.00761	0.128543	0.33146
LOC10192!	-0.01793	0.128627	0.331594
GNB5	0.162849	0.128646	0.331594
GSTT1	0.215437	0.128647	0.331594
PEBP4	-0.02179	0.128657	0.331594
FICD	0.108064	0.128684	0.331594
RP4-813F1	-0.13532	0.128687	0.331594
C1RL-AS1	-0.05758	0.128908	0.332124
C22orf39	0.093916	0.129045	0.332414
AQP3	0.063215	0.129051	0.332414
TTC13	-0.13694	0.129144	0.332612

C10orf53	-0.00799	0.129298	0.33297
TNFAIP8L1	0.138078	0.129458	0.333344
TIMMDC1	0.10678	0.129643	0.333727
ARHGAP24	0.078781	0.129652	0.333727
ZNF576	0.080892	0.129655	0.333727
CYP2A7	-0.02519	0.129686	0.333727
PDZK1	-0.03402	0.12969	0.333727
FAM150B	0.288486	0.129702	0.333727
HSPA1L	0.073798	0.129715	0.333727
RLTPR	0.078692	0.129731	0.333729
SMARCD3	0.145398	0.129753	0.333746
GRAMD4	0.097517	0.129779	0.333773
LOC38983	0.166076	0.129832	0.33387
LOC10192	0.008685	0.129937	0.334099
VAMP7	-0.08784	0.130009	0.334245
DSERG1	-0.25537	0.130038	0.334281
ACADSB	-0.1144	0.13011	0.334421
PSPC1	-0.14744	0.130124	0.334421
LOC64465	-0.21841	0.130159	0.334473
OXNAD1	0.168314	0.13019	0.334494
LOC72968	-0.32643	0.130199	0.334494
F3	0.251606	0.130234	0.334545
EFTUD2	-0.0698	0.130291	0.334618
VIPR1	-0.03829	0.130302	0.334618
LOC21968	-0.03729	0.130309	0.334618
CTNNAP1	-0.01255	0.13037	0.334735
RP11-16P6	-0.1224	0.13045	0.334878
SUSD3	0.100931	0.130456	0.334878
LOC10028	0.059797	0.130497	0.3349
BARX2	-0.0662	0.130519	0.3349
PLEKHG5	-0.07051	0.130535	0.3349
C2CD5	-0.12564	0.130536	0.3349
LOXL1	0.132498	0.130542	0.3349
PRKG1-AS	0.003867	0.130627	0.335078
TMEM163	-0.31391	0.130715	0.335173
SPAG5-AS	-0.05353	0.130722	0.335173
NEUROD6	-0.00382	0.130723	0.335173
LOC10192	0.170804	0.130757	0.335173
GPC3	0.017863	0.130765	0.335173
BMP8B	-0.02546	0.130767	0.335173
LOC43993	-0.00623	0.130772	0.335173
SERAC1	-0.1591	0.130817	0.335249
LOC10272	0.14783	0.130845	0.335279
CNTFR	0.05445	0.130873	0.335286
LOC10192	-0.00723	0.130893	0.335286
GVQW1	-0.05884	0.130894	0.335286
RWDD1	-0.10939	0.13096	0.335417
ATF6B	-0.09058	0.131019	0.335509
EGLN1	0.15882	0.131032	0.335509
ARHGEF17	-0.16158	0.131051	0.335509
C1orf94	-0.01921	0.131073	0.335509
PRDM5	-0.01817	0.131074	0.335509
POLR3G	-0.07404	0.131103	0.335544
AP006222.	0.117468	0.131139	0.335596
FAM179A	-0.11765	0.131174	0.335634
SART1	-0.14012	0.131201	0.335634
NIPSNAP3.	-0.21779	0.131206	0.335634
NKAIN3	-0.03875	0.131222	0.335634

MEP1A	0.02827	0.131231	0.335634
POLDIP2	0.131628	0.13125	0.335642
NYAP2	0.006305	0.131289	0.335703
LINC01289	-0.03352	0.131319	0.335711
ZNF696	-0.08589	0.131323	0.335711
RP4-633H1	-0.00578	0.131377	0.335798
THOP1	0.156416	0.131388	0.335798
SLC39A12	0.038073	0.131425	0.335852
LOC28333!	-0.02017	0.131458	0.335897
RP11-654A	0.013628	0.131492	0.335944
FLJ20712	-0.02911	0.131521	0.335967
C21orf91-(	0.018817	0.131532	0.335967
HNRNPA3I	-0.03087	0.131612	0.336131
CWH43	0.033174	0.131636	0.336152
HDAC4	-0.2315	0.131663	0.336182
PTTG1	0.232901	0.131679	0.336182
GLS	0.134682	0.131694	0.336182
RABGEF1	0.18781	0.131746	0.336239
PRMT5-AS	-0.00569	0.131747	0.336239
MAP1B	-0.0808	0.131813	0.33633
SYNJ1	-0.02003	0.131814	0.33633
IRS1	-0.08711	0.131843	0.336345
C5orf22	0.085663	0.131863	0.336345
LOC10192!	0.002545	0.131867	0.336345
TFG	-0.15616	0.131922	0.336447
XPNPEP2	0.067075	0.132009	0.336542
CCSER1	-0.03029	0.13201	0.336542
NPFF	0.086669	0.13202	0.336542
RAE1	0.155437	0.132028	0.336542
FAM124B	-0.25222	0.132037	0.336542
SCUBE1	-0.07801	0.132092	0.336614
FAM159A	0.005698	0.132096	0.336614
ST8SIA5	-0.03272	0.132128	0.336654
ZIC4	-0.00288	0.132177	0.336728
LOC40079	-0.02106	0.132208	0.336728
CACNA1G-	-0.04021	0.132211	0.336728
GABRR1	0.00256	0.132232	0.336728
KIF25-AS1	-0.02264	0.132234	0.336728
LINC00657	0.14699	0.132343	0.336925
RP11-389C	-0.07201	0.132343	0.336925
DERL1	0.102544	0.132394	0.336984
CAMTA2	0.079062	0.132403	0.336984
LOC10028!	-0.03368	0.132413	0.336984
TPST2	0.185797	0.132434	0.336999
RGS3	0.096988	0.132515	0.337164
SLC35B4	0.151332	0.13258	0.337285
C19orf81	-0.03981	0.132608	0.337285
KCNQ4	0.087043	0.132609	0.337285
TIGD4	-0.01532	0.132639	0.337322
PMS2L2	0.212998	0.132778	0.337607
ATG4A	0.135015	0.132784	0.337607
RETN	0.214689	0.132798	0.337607
IRF8	0.492246	0.13283	0.337619
GATA6-AS	-0.06552	0.132834	0.337619
PTPRG	0.003937	0.132886	0.3377
LOC10192	-0.03043	0.132911	0.3377
PRKCSH	0.141159	0.132915	0.3377
SLC25A19	0.136536	0.132928	0.3377

EPC2	-0.13873	0.132984	0.337795
C17orf62	0.159258	0.132996	0.337795
ADM5	0.117772	0.133027	0.337812
ERICH5	-0.03965	0.133034	0.337812
C11orf92	0.008333	0.13306	0.337837
RP11-16N1	-0.10985	0.13313	0.337949
LOC28483	0.136835	0.133135	0.337949
FBXO18	0.054282	0.133256	0.338217
UBL3	0.109516	0.133306	0.338302
SNRPD2	0.163317	0.133357	0.338392
NDUFB4	0.090167	0.133376	0.338402
PER4	0.003719	0.133406	0.338438
ATP11B	-0.11511	0.133463	0.338542
ACN9	-0.19201	0.133512	0.338627
CCDC88A	-0.1253	0.133559	0.338707
ATP13A3	-0.1458	0.133575	0.338707
C15orf61	0.132443	0.133651	0.338831
LOC10192	-0.04325	0.13366	0.338831
C1R	0.171801	0.133685	0.338831
DUSP27	-0.2027	0.13369	0.338831
RBP4	0.058851	0.133716	0.338831
PCMTD1	-0.1686	0.133729	0.338831
AKR7A2	-0.10796	0.133733	0.338831
SETD8	0.118157	0.133773	0.338892
LINGO1	-0.05983	0.133901	0.339176
PHLPP2	0.075693	0.133919	0.339182
NS3BP	-0.00353	0.133962	0.339251
ZFP14	-0.12231	0.134022	0.339336
LINC00324	0.112407	0.13403	0.339336
C16orf92	-0.03389	0.134042	0.339336
ZNF503	0.02581	0.134063	0.339336
C20orf144	0.010182	0.134078	0.339336
LHFPL1	-0.00465	0.134089	0.339336
RIC3	0.005633	0.134126	0.339347
FAM195A	0.160682	0.134131	0.339347
LMO3	0.00277	0.134154	0.339347
ADH7	-0.03146	0.134166	0.339347
TIAF1	0.079824	0.134172	0.339347
ZNF92	0.054521	0.134241	0.339482
KIR3DL3	0.021031	0.134267	0.339508
LOC10272	0.006895	0.134284	0.339511
LOC10272	-0.00684	0.134438	0.339863
LATS2	-0.2162	0.134512	0.339979
SHH	-0.01197	0.134516	0.339979
C1orf111	-0.01975	0.134543	0.340008
KIAA1614	0.010027	0.134588	0.340083
SLC25A34	0.080596	0.13462	0.340088
HACL1	-0.17338	0.134629	0.340088
TMEM14A	-0.1758	0.134638	0.340088
KRT6B	-0.02643	0.134697	0.340169
SYNPR	0.029391	0.134701	0.340169
SASH3	0.183836	0.134772	0.340308
LOC41430	0.002383	0.134804	0.340351
TSPAN33	0.238401	0.134825	0.340363
ZFP91	0.111063	0.134847	0.340379
DMGDH	-0.01112	0.134874	0.340408
CCDC85C	0.063089	0.134917	0.340476
SHOC2	0.106725	0.134964	0.340537

SERBP1	-0.10532	0.134972	0.340537
BCORP1	-0.02286	0.13501	0.340591
CMC2	0.10482	0.135131	0.340857
BICD2	0.16828	0.135182	0.340946
LOC10192	-0.01492	0.135205	0.340965
INA	-0.0238	0.135233	0.340996
RNFT1	-0.12854	0.135256	0.341013
AP000265.	-0.00811	0.135317	0.341098
AGO1	-0.09399	0.135336	0.341098
LOC10029	0.079912	0.135337	0.341098
CHADL	0.058105	0.135396	0.341209
TSG101	0.089193	0.135481	0.341353
HTRA4	-0.12167	0.135485	0.341353
LINC00950	-0.0024	0.135531	0.341424
RP11-945A	0.002112	0.135559	0.341424
C8orf88	0.267892	0.13556	0.341424
AADAT	0.150957	0.135602	0.341488
PGM2	0.101956	0.135622	0.3415
KIAA0430	0.067435	0.135655	0.341542
SEC14L4	0.122191	0.135672	0.341546
BRK1	0.063309	0.135702	0.341582
TMCO6	0.201143	0.13576	0.341688
TMED6	-0.19401	0.135795	0.341702
CENPV	0.306807	0.135808	0.341702
FGFR2	-0.00833	0.135813	0.341702
LINC00687	-0.01598	0.135854	0.341765
INPP5J	0.094039	0.135904	0.341829
NDUFB1	0.057348	0.135911	0.341829
RP11-209A	-0.14224	0.135955	0.341851
FAM110C	0.011682	0.135962	0.341851
TXNRD2	0.088789	0.135967	0.341851
AK000798	0.105691	0.135986	0.34186
C17orf98	-0.00514	0.136071	0.342033
LOC40074	-0.041	0.136089	0.342038
PCDHB14	0.168654	0.136121	0.34208
ZNF850	-0.28148	0.13628	0.342439
ADCY1	0.011555	0.136313	0.342483
CTD-2083I	-0.20692	0.136339	0.342509
HOXB4	-0.13248	0.13637	0.342548
CHRND	-0.04452	0.136413	0.342616
MGP	0.088817	0.136472	0.342701
HES7	-0.03621	0.136479	0.342701
SFXN3	0.192864	0.136606	0.342979
PROX1-AS	-0.00984	0.136629	0.342998
LOXL1-AS	-0.28532	0.136674	0.34307
TUT1	-0.09038	0.136689	0.34307
C22orf23	0.008278	0.136715	0.34309
RP11-543C	-0.01498	0.136729	0.34309
AC008088.	-0.10138	0.136802	0.343234
RP11-114H	-0.01549	0.136849	0.343311
SF3B2	0.043314	0.136897	0.343392
PRPS1L1	-0.0263	0.136924	0.343419
PNMA6A	0.184791	0.136943	0.343428
TRMU	-0.11865	0.136961	0.343433
TSPAN2	-0.37931	0.137031	0.343568
PPP1R7	0.123615	0.13719	0.343927
SIK1	0.197098	0.137235	0.344002
CIB4	-0.01403	0.137407	0.344392

SNRNP70	0.08521	0.137458	0.344475
C12orf40	-0.01484	0.137485	0.344475
CYP17A1	-0.00909	0.137498	0.344475
MAPK12	-0.06808	0.137503	0.344475
MRPS9	0.155011	0.137642	0.344746
LINC00301	0.002387	0.137651	0.344746
CAMK2D	0.198073	0.13769	0.344746
LOC10050!	0.032562	0.137696	0.344746
CES2	0.084731	0.137708	0.344746
LOC28616:	-0.15001	0.13771	0.344746
AOC1	0.073363	0.137723	0.344746
LLGL2	0.051779	0.13779	0.344873
RP11-673E	-0.01345	0.137817	0.3449
CDC123	-0.11339	0.137851	0.344945
C14orf132	0.061835	0.137898	0.344987
SLC5A9	-0.01154	0.137915	0.344987
LINC01365	-0.01676	0.137915	0.344987
CSMD2	0.003241	0.137944	0.34502
FBP2	0.021439	0.13797	0.345045
ATP1B3	0.047601	0.137996	0.345069
ALMS1P	-0.05319	0.138023	0.34507
GMFG	0.122929	0.138053	0.34507
IRF6	-0.02451	0.138062	0.34507
TNK2	0.077655	0.138069	0.34507
NHLRC4	-0.05754	0.138076	0.34507
SCO1	0.160897	0.138093	0.345073
LOC10192{	-0.01182	0.138162	0.345205
NLK	0.16619	0.138181	0.345214
LINC01133	0.123755	0.138206	0.345235
CCDC94	0.16636	0.13825	0.345307
S100A1	-0.00312	0.138429	0.345713
SLC3A2	0.141741	0.138517	0.345835
RSPO4	-0.0164	0.138541	0.345835
PPBP	0.622799	0.138543	0.345835
LSMEM2	-0.02357	0.138557	0.345835
SFSWAP	0.119654	0.138558	0.345835
ZNF431	-0.23987	0.138579	0.345848
CCDC153	0.03636	0.138612	0.345859
GRM6	-0.02696	0.138615	0.345859
TMEM259	0.035775	0.138656	0.345922
GTF2H5	-0.08601	0.138718	0.346036
HLA-DMA	0.147167	0.138757	0.346092
GALNTL6	-0.00622	0.138802	0.346116
LOC28489{	-0.01753	0.13881	0.346116
CDH11	0.071262	0.138814	0.346116
SLC2A11	0.101307	0.138859	0.346188
PSMB8	0.145142	0.138878	0.346188
LEP	-0.0402	0.138891	0.346188
KIF5A	-0.02569	0.138942	0.346268
LOC10272!	0.05845	0.138955	0.346268
RP11-461/	0.032455	0.138991	0.34629
NTN5	-0.01638	0.139009	0.34629
OR7D2	-0.1442	0.13903	0.34629
AQP8	0.00445	0.139039	0.34629
LOC10192	-0.01422	0.139047	0.34629
HIST1H4H	-0.42188	0.13906	0.34629
PAK7	0.003782	0.139086	0.346317
DMRTB1	-0.01944	0.139331	0.346887

HIST1H2BK	0.275696	0.139441	0.347064
SH3RF3	0.248288	0.13945	0.347064
ZNF573	-0.09563	0.139451	0.347064
COL15A1	0.093406	0.139481	0.347087
LOC10192	-0.02085	0.139496	0.347087
BAZ2B	-0.16532	0.139508	0.347087
RP11-164F	-0.0239	0.139528	0.347097
WDR48	0.127293	0.139587	0.347204
ZNF876P	-0.02574	0.139667	0.347325
FAM53B	0.060031	0.139668	0.347325
DHX36	0.09397	0.139727	0.347429
HS6ST1	-0.01972	0.139742	0.347429
PRKACB	-0.15912	0.139786	0.347492
EIF4H	-0.10199	0.139799	0.347492
TMEM177	-0.13358	0.139888	0.347674
STRADA	-0.07272	0.139927	0.34771
C21orf37	0.019364	0.139935	0.34771
STRN	-0.14604	0.139955	0.34772
BFSP2	0.039599	0.139997	0.347783
RGL2	0.148868	0.140044	0.347861
SEC23A	0.13582	0.140069	0.347883
UQCC2	0.029541	0.140099	0.347918
SH3GL2	0.248576	0.140117	0.347924
RP11-38L1	-0.03981	0.140182	0.348036
MEF2C-AS	-0.07076	0.140195	0.348036
SLC4A10	0.024939	0.140232	0.348088
C10orf99	-0.03487	0.140284	0.348177
LOC40191	-0.07557	0.140359	0.348234
CLOCK	0.093555	0.14036	0.348234
RP11-131L	-0.00953	0.14037	0.348234
ROR2	-0.02622	0.140371	0.348234
LOC40062	-0.01557	0.140404	0.34827
MB21D2	0.193238	0.140418	0.34827
CASP6	-0.10056	0.140466	0.348343
GYS1	0.173054	0.140488	0.348343
LYPLA1	-0.15137	0.140495	0.348343
DDC	-0.01452	0.140538	0.34841
FAM9C	-0.01465	0.140574	0.348459
ZNF28	-0.02127	0.140716	0.34877
MPHOSPH	-0.14005	0.140734	0.348774
LOC10050	0.012843	0.140756	0.348775
PRAMEF10	-0.01393	0.140784	0.348775
ZNF446	-0.03908	0.140793	0.348775
LINC00216	-0.00786	0.140799	0.348775
KDM1B	0.106543	0.140817	0.348781
LOC10050	-0.00433	0.140887	0.34891
C3orf43	-0.01666	0.140901	0.34891
OR10H3	-0.00415	0.140935	0.348955
LOC10192	-0.00801	0.140969	0.348966
BEX2	-0.32001	0.140972	0.348966
GNG3	-0.02828	0.141017	0.349037
GNPDA2	-0.15177	0.141057	0.349097
RP11-38C	-0.04245	0.141135	0.34925
CNOT8	0.12624	0.141174	0.349306
FOSL1	0.217749	0.141234	0.349332
VSIG10L	0.13524	0.141251	0.349332
SARS2	0.179929	0.141261	0.349332
DLEU2L	-0.0376	0.141269	0.349332

DZIP3	-0.1919	0.141283	0.349332
FAM86C1	0.031816	0.14129	0.349332
BTRC	-0.04411	0.141298	0.349332
FANCE	-0.144	0.141338	0.349391
CD2	0.272981	0.141355	0.349394
PRPF39	0.229543	0.141372	0.349395
PLEKHG6	0.038557	0.141461	0.349465
SETD6	-0.14141	0.141468	0.349465
AFF2	-0.00757	0.141489	0.349465
GABRA6	0.002915	0.141498	0.349465
TLE4	0.283891	0.141524	0.349465
CLEC3A	-0.01663	0.141549	0.349465
GZMM	-0.02584	0.14155	0.349465
ZNF461	0.016302	0.141561	0.349465
CHL1	0.110764	0.141572	0.349465
ANKRD36C	0.190604	0.141572	0.349465
MACF1	0.164425	0.14159	0.349465
RP11-1275	-0.17423	0.141594	0.349465
BLM	-0.16522	0.14163	0.349482
LOC10050	0.043159	0.141642	0.349482
MFAP5	0.062246	0.141661	0.349482
TBATA	-0.05637	0.141665	0.349482
ANO7	-0.01347	0.141764	0.349687
KIAA1024	-0.06803	0.141851	0.349783
WRAP53	-0.11722	0.141852	0.349783
TCEA2	0.101512	0.141863	0.349783
NOTCH4	0.068831	0.141881	0.349783
CDC42EP4	0.144439	0.14189	0.349783
MMP14	-0.01138	0.141916	0.349783
ORC3	-0.1005	0.141925	0.349783
RAX2	-0.02715	0.141932	0.349783
LINC01395	-0.00786	0.141992	0.349876
ZWILCH	-0.14132	0.142002	0.349876
TRIM28	0.195663	0.142026	0.349895
DNM1L	0.117093	0.14206	0.349938
C10orf113	-0.00907	0.142138	0.350091
NLRP14	0.014884	0.142173	0.350136
OR8D1	0.016368	0.142201	0.350167
FAM218A	-0.01983	0.142271	0.3503
ACTL6A	-0.23656	0.14231	0.350355
CYP27B1	0.135381	0.142333	0.350357
STARD7-A	0.085456	0.142343	0.350357
ILDR1	0.030644	0.142398	0.350453
SH3RF3-A	-0.0084	0.142432	0.350496
CPO	-0.01521	0.142496	0.350613
PIAS4	0.100319	0.142652	0.350955
LINC01352	0.006095	0.142667	0.350955
10-Mar	0.007092	0.142738	0.351076
NCAM2	-0.00548	0.142749	0.351076
PTGER4	-0.11529	0.142818	0.351207
FAHD2CP	-0.11874	0.142867	0.351286
RP11-285J	-0.12938	0.142919	0.351376
PARP3	0.144738	0.143013	0.351567
CREG2	-0.01542	0.143069	0.351581
CUEDC2	0.120398	0.14307	0.351581
NNT	-0.10497	0.143087	0.351581
ZNF383	-0.14038	0.143105	0.351581
IL17D	-0.13601	0.143105	0.351581

DMXL2	0.173823	0.143133	0.351581
NHEG1	-0.01581	0.143139	0.351581
LCN10	0.201407	0.143152	0.351581
CCDC25	-0.10117	0.143175	0.351581
LINC01021	0.137681	0.143181	0.351581
KIAA0485	-0.19722	0.143207	0.351584
FSCB	0.003082	0.143215	0.351584
ARMCX4	-0.02921	0.143255	0.351642
MGAT4C	0.009146	0.143296	0.351704
FAM131B	-0.10282	0.143324	0.351731
CLINT1	0.131607	0.143433	0.35196
YTHDC1	0.151419	0.143476	0.352026
C16orf62	0.048571	0.143579	0.352204
RP11-1012	-0.01376	0.143581	0.352204
DCAF11	0.111382	0.143604	0.352219
ZMYND12	0.159858	0.143625	0.352231
ILDR2	-0.14158	0.143663	0.352285
ZSCAN9	-0.11252	0.14368	0.352285
ATRNL1	-0.01076	0.143719	0.352308
TRBV27	-0.48316	0.143721	0.352308
ORM1	0.139571	0.1438	0.35246
LINC01102	0.007488	0.143825	0.352481
MEG3	0.204624	0.143845	0.352491
RP11-109M	-0.04202	0.143909	0.352608
LINC00567	-0.02111	0.143958	0.352688
PLEKHH2	0.0045	0.144014	0.352785
NOTUM	-0.0413	0.144047	0.352827
BC024169	0.013371	0.144187	0.353129
MPRIP	0.081654	0.144229	0.353193
LDB3	-0.01177	0.144324	0.353384
MAP3K19	-0.02621	0.144422	0.353559
LOC10192	0.004319	0.144428	0.353559
XPA	-0.12811	0.144477	0.35364
SLC22A31	0.03096	0.14453	0.353729
NAV2-AS4	-0.03298	0.144571	0.353789
ARHGEF7	-0.17482	0.144613	0.353854
FOSL2	0.34229	0.144672	0.353947
SIRT1	-0.16099	0.144688	0.353947
SLC22A18/	-0.04798	0.144701	0.353947
UMOD	-0.00933	0.144728	0.35395
GRAP2	-0.16225	0.144735	0.35395
FLJ31306	0.225082	0.144929	0.354386
LINC00910	-0.00937	0.145122	0.354787
LOC73142	0.117174	0.145134	0.354787
ACSL5	-0.10737	0.145143	0.354787
RASD1	-0.32165	0.145172	0.354819
ATG2A	0.144253	0.145415	0.355374
LOC40075	-0.07379	0.145487	0.355485
SAMD8	0.148301	0.145517	0.355485
LOC44070	-0.0057	0.145526	0.355485
TEX15	0.063987	0.145526	0.355485
MAD2L1BP	0.142427	0.145638	0.355718
COA5	-0.08397	0.145746	0.355941
ASB1	0.108653	0.145783	0.35599
ACAA2	0.151061	0.145807	0.356009
FOXC2	0.02085	0.145893	0.35615
RPL35	0.122748	0.145897	0.35615
MEOX2	0.005169	0.145968	0.356272

GBP4	0.207388	0.14598	0.356272
WIF1	-0.12488	0.146033	0.356331
TACR2	0.019113	0.146037	0.356331
LINC00934	-0.00524	0.146103	0.356434
SYN1	-0.01469	0.146113	0.356434
RBM48	0.134854	0.14615	0.356447
TOR3A	-0.09662	0.146161	0.356447
AX746823	-0.33284	0.146171	0.356447
RFX1	0.068438	0.1462	0.356447
LOC10192	0.003831	0.146209	0.356447
SP7	-0.01147	0.146225	0.356447
KIF7	-0.13566	0.146233	0.356447
IFNK	0.013369	0.146312	0.356557
CENPJ	0.217296	0.146316	0.356557
SFRP1	0.003605	0.146338	0.356557
RTP5	0.008334	0.146348	0.356557
U2SURP	-0.12072	0.146375	0.356557
HEATR9	-0.02883	0.146377	0.356557
CORO6	-0.08898	0.146401	0.356575
LTA	0.034552	0.146462	0.356684
GRB14	-0.24468	0.146556	0.356872
TYW1	-0.1184	0.146643	0.357045
BPIFA3	-0.0667	0.14669	0.357118
USE1	0.153081	0.146749	0.357223
GK3P	0.094447	0.146782	0.357223
RP11-461L	0.022142	0.146782	0.357223
PLAG1	-0.40222	0.146817	0.357268
PLK2	0.335406	0.146893	0.357411
TUBB6	0.34591	0.146936	0.357476
NYAP1	-0.0086	0.146962	0.3575
RAB1A	0.107301	0.147007	0.357569
RNLS	0.08827	0.147084	0.35771
STK17A	0.166394	0.147098	0.35771
C5orf49	0.013877	0.147151	0.357798
RP11-115A	0.023738	0.147195	0.357849
METTL14	0.10852	0.147219	0.357849
GPHN	0.162568	0.147234	0.357849
MOSPD2	0.14103	0.147238	0.357849
IFNAR2	0.105437	0.147258	0.357859
FGFBP3	0.095464	0.147348	0.358037
TEX37	-0.03067	0.147386	0.358087
MAP4K3	-0.26664	0.147411	0.358109
SMDT1	-0.15353	0.147444	0.358119
ARHGEF19	0.103755	0.147448	0.358119
HEXA-AS1	0.027026	0.147511	0.358232
ZNF3	-0.05968	0.147558	0.358245
ZNF417	-0.04491	0.147559	0.358245
CEACAM6	0.440335	0.147566	0.358245
ZFAND5	0.196994	0.147587	0.358255
AC018755.	0.071802	0.147641	0.358346
CLTCL1	0.037449	0.147672	0.358382
SPCS3	0.15627	0.147734	0.358492
LOC10192	-0.0106	0.147813	0.358643
SPINT2	-0.20286	0.147882	0.35871
CMPK1	-0.10874	0.147892	0.35871
MMP2	-0.02324	0.147899	0.35871
SLC2A4	0.004122	0.147922	0.35871
CCNT1	0.144749	0.147923	0.35871

LOC10272:	-0.07999	0.147949	0.358723
FAM206A	0.111177	0.147962	0.358723
CHST13	0.121258	0.148086	0.358984
STK19	-0.09345	0.14811	0.358984
SSPN	0.145573	0.148132	0.358984
KBTBD8	-0.22946	0.148136	0.358984
LOC55417	-0.02861	0.148157	0.358995
WFDC9	-0.02631	0.148198	0.359053
TTC28-AS:	0.08474	0.148238	0.359081
MSL3	-0.14178	0.148242	0.359081
FUT1	0.284464	0.148284	0.359143
MRPS15	0.111052	0.148432	0.35943
DLGAP1-A	0.014917	0.148436	0.35943
AMICA1	0.320517	0.14847	0.359471
ZBTB22	0.093348	0.148545	0.3596
SYDE2	-0.0929	0.148556	0.3596
RTP3	-0.01322	0.148609	0.359648
IL6R	0.261465	0.148626	0.359648
SNORA65	-0.0458	0.148641	0.359648
C11orf44	-0.00517	0.148642	0.359648
LOC28500	0.01026	0.148691	0.359726
XIST	-0.98897	0.148719	0.359753
TAF6	-0.16318	0.148754	0.359797
PACS1	0.071811	0.1488	0.359869
ZNF230	-0.20676	0.148911	0.360031
SPIN2A	-0.01929	0.148937	0.360031
GSG2	-0.15914	0.148946	0.360031
MC4R	0.005142	0.148949	0.360031
COX4I1	-0.13333	0.14895	0.360031
HAUS8	0.184804	0.148999	0.360083
VPS52	0.085637	0.14902	0.360083
TFB1M	-0.10095	0.149053	0.360083
LOC28451:	-0.18813	0.149061	0.360083
EHD2	0.194566	0.149068	0.360083
RCAN2	0.109407	0.149072	0.360083
AOC4P	-0.04463	0.14911	0.360135
GIGYF2	0.146652	0.149194	0.360299
ZNF280B	-0.17806	0.149248	0.360389
PNKP	0.145903	0.149289	0.360417
RAB27A	0.176802	0.149305	0.360417
CRTAM	0.196497	0.14931	0.360417
LOC38888:	0.021284	0.149333	0.360429
SLC25A43	-0.22002	0.149348	0.360429
BC027448	-0.06006	0.149448	0.360605
SLC30A10	0.00651	0.149454	0.360605
CADM2-A:	-0.008	0.149515	0.360684
SH3BP1	0.066461	0.149521	0.360684
LOC10050!	-0.00391	0.149537	0.360684
ST13	0.094135	0.149642	0.360877
TTTY11	-0.02283	0.149658	0.360877
LMTK3	-0.05601	0.149667	0.360877
CLUH	-0.17069	0.149696	0.360907
RP13-436F	-0.0161	0.149799	0.361095
TJP2	-0.08942	0.149807	0.361095
RIMBP2	0.024225	0.149851	0.361161
ROBO3	-0.17239	0.149876	0.361181
RP5-935K1	-0.09329	0.150044	0.361544
NGRN	0.12361	0.150089	0.361571

FLJ33544	-0.01887	0.150091	0.361571
MAN2C1	-0.13017	0.150105	0.361571
LOC10192[illegible]	-0.19769	0.150123	0.361575
LOC10246[illegible]	-0.01631	0.150171	0.361625
ARSI	0.004767	0.150177	0.361625
ATAD1	-0.14333	0.150288	0.361788
PLEKHA4	0.020179	0.150293	0.361788
BRD9	-0.1224	0.150295	0.361788
FOXK2	-0.11717	0.150345	0.361868
ZNF169	0.075675	0.150373	0.361896
MCM4	0.201372	0.150397	0.361913
KLHL8	0.160693	0.150416	0.361917
LOC44120[illegible]	-0.08237	0.150455	0.36197
CACNG5	-0.02659	0.150487	0.362008
LOC10192[illegible]	-0.02005	0.150577	0.362184
ZNF524	0.021454	0.150692	0.362381
FUNDC2	0.05151	0.150692	0.362381
APOA5	-0.0201	0.150802	0.362605
BCDIN3D-[illegible]	-0.02152	0.150827	0.362624
LTBP3	0.131723	0.150891	0.362738
KLK12	-0.03324	0.150961	0.362867
CSHL1	-0.00569	0.151013	0.36293
GTF3C3	-0.12229	0.151021	0.36293
CACNA1F	0.006373	0.151202	0.363308
FER1L4	0.022385	0.151212	0.363308
GNG8	0.094176	0.151386	0.363687
SDK1	-0.05727	0.151581	0.364114
TMEM179	-0.01595	0.151658	0.364244
LINC01004	-0.03023	0.151669	0.364244
HPDL	0.300509	0.151739	0.364373
LOC64658[illegible]	0.024311	0.15185	0.364491
SH2D1A	0.148178	0.151865	0.364491
CLPS	-0.02556	0.151866	0.364491
TXLNGY	0.741313	0.151875	0.364491
DSCR3	0.088857	0.151886	0.364491
SPAG4	0.147702	0.151889	0.364491
DNLZ	0.214442	0.152008	0.364734
RCC1	-0.17068	0.15203	0.364748
TRIM33	-0.0873	0.152136	0.364961
CLNK	-0.06558	0.152259	0.365215
C6orf52	-0.02114	0.152463	0.365664
CDK14	-0.07288	0.152518	0.365756
TAGAP	0.165883	0.152565	0.365827
MTFR2	-0.22703	0.15263	0.365942
TNFRSF4	0.022772	0.152665	0.365967
PAIP1	-0.1346	0.152674	0.365967
TMPRSS4	0.049159	0.152703	0.365997
CCDC135	-0.01865	0.15272	0.365998
MDC1	0.099578	0.152747	0.366021
LOC10050[illegible]	-0.01049	0.152933	0.366428
PSMC1	-0.11961	0.153043	0.366618
CAMKK2	0.121042	0.153047	0.366618
DDIT4	0.390181	0.153077	0.366629
FAM170A	0.00652	0.153085	0.366629
SPO11	0.018066	0.153248	0.366979
GTF2H2B	0.298255	0.153392	0.367215
CCNY	0.136076	0.153424	0.367215
ERCC6	-0.07788	0.15343	0.367215

LCE3D	-0.03964	0.153438	0.367215
LINC00623	-0.1445	0.153445	0.367215
ADAM5	0.008845	0.153449	0.367215
LOC101928	0.008073	0.153496	0.367235
CYCS	-0.24814	0.153506	0.367235
MGC39545	0.007848	0.153514	0.367235
C17orf80	0.117848	0.153525	0.367235
LINC01020	-0.00447	0.153834	0.367899
LOC100505	-0.21746	0.153836	0.367899
RP11-59H7	0.038136	0.153912	0.368039
LOC255654	0.001646	0.154036	0.368295
DAGLB	0.215389	0.154058	0.368308
BIN1	-0.23286	0.154086	0.368334
FAM179B	-0.1591	0.15414	0.368423
HOXD8	0.050935	0.15426	0.368607
ALPPL2	-0.02684	0.154263	0.368607
ZNF598	0.069471	0.154269	0.368607
FAM69B	0.076533	0.154296	0.368609
CABYR	-0.02149	0.154314	0.368609
MYH3	-0.19095	0.154321	0.368609
CSRP2	-0.2365	0.154339	0.368613
MZF1	0.143395	0.154361	0.368624
PDPN	0.003182	0.15438	0.36863
LRRC63	-0.03063	0.154475	0.368773
KIF27	0.103572	0.15448	0.368773
FAM163A	0.006275	0.154497	0.368773
CELSR3	0.179168	0.154508	0.368773
SYBU	0.187757	0.154552	0.368836
RBM3	0.136727	0.154717	0.369189
EFCAB6	-0.0049	0.154777	0.369293
LOC101928	-0.05856	0.154797	0.3693
FAM207A	0.099861	0.154891	0.369311
CR2	0.08329	0.154894	0.369311
DAP3	-0.07113	0.154896	0.369311
SLC5A3	0.100306	0.154897	0.369311
TSPAN7	0.336483	0.154897	0.369311
TEX33	0.014604	0.154904	0.369311
TMEM213	-0.00588	0.155141	0.369819
CEMIP	0.137685	0.155151	0.369819
SPATA31E1	0.013951	0.155194	0.36984
HAVCR1P1	-0.06511	0.155194	0.36984
HS3ST6	-0.00447	0.155247	0.369924
SYDE1	-0.02644	0.155304	0.370021
AHSA1	-0.14466	0.155339	0.370062
SPOCK3	-0.2353	0.155444	0.370273
SOX13	-0.01395	0.155526	0.370427
H1FX	0.187844	0.155552	0.370447
ANXA4	0.099902	0.155589	0.370496
TRAPPC6A	0.161272	0.155624	0.370538
TOX3	0.003125	0.15574	0.370774
MAN1A2	0.091479	0.155795	0.370862
REM1	-0.0687	0.15586	0.370966
B3GALNT1	-0.21734	0.155883	0.370966
FOXH1	-0.02132	0.15589	0.370966
G3BP1	-0.1561	0.155933	0.371029
SYT7	-0.00288	0.156017	0.37117
ARF6	0.106486	0.156027	0.37117
LOC645321	-0.01605	0.156107	0.371319

NR5A2	-0.1304	0.156128	0.371319
UHRF1BP1	0.102906	0.156141	0.371319
RBM12B	-0.1731	0.156171	0.371349
RP1L1	0.001529	0.156357	0.371654
CPNE4	-0.03799	0.156365	0.371654
LA16c-381	-0.01529	0.156367	0.371654
LOC10192!	-0.03325	0.156395	0.371654
ZNF826P	-0.15715	0.156397	0.371654
RAB30-AS!	-0.10462	0.156402	0.371654
RUVBL1	0.152448	0.15645	0.371692
SDR9C7	-0.01568	0.156452	0.371692
AQP10	-0.18727	0.156493	0.371747
RALYL	0.015814	0.156562	0.371871
MYBL2	0.250789	0.156644	0.372025
RP1-193H!	-0.09946	0.156677	0.372042
HID1	-0.02625	0.156686	0.372042
GGN	-0.00989	0.156723	0.37209
NOG	0.35903	0.156768	0.372157
SHMT2	0.169483	0.156806	0.372205
PON2	0.194393	0.15685	0.372269
SYCP2	0.22037	0.156869	0.372273
MAP4K1	-0.1738	0.157001	0.372507
SRRM4	-0.01159	0.157017	0.372507
ZDHHC8	0.036167	0.157019	0.372507
CNKSR3	-0.05142	0.157085	0.372615
RP11-177N	0.022585	0.157099	0.372615
SH3BGRL	0.090381	0.15726	0.372836
LOC40131!	-0.00327	0.157269	0.372836
TOLLIP-AS	-0.12176	0.157275	0.372836
LOC10192!	0.003352	0.157276	0.372836
PMP22	-0.06038	0.157299	0.372836
CASP4	0.161064	0.157309	0.372836
ANKRD1	-0.00567	0.157319	0.372836
RIMS4	-0.03461	0.15733	0.372836
PMS2P5	-0.26075	0.157408	0.37298
KANSL1	-0.13428	0.157468	0.373082
LINC01225	-0.04009	0.157612	0.373382
IRGQ	0.07788	0.157653	0.373438
ZNF428	0.02769	0.157698	0.3735
NCKAP1	0.142935	0.157728	0.3735
SOX30	-0.01726	0.157731	0.3735
CTH	0.123945	0.157762	0.373534
LOC64151!	0.013375	0.157934	0.373899
ADM2	-0.04882	0.158025	0.374075
TWF1	-0.01598	0.158155	0.37432
GATSL3	0.054776	0.158163	0.37432
ZNF700	-0.16191	0.158236	0.37437
RERE	0.014121	0.158237	0.37437
ZXDA	-0.21835	0.158239	0.37437
RP11-65D!	-0.0046	0.158254	0.37437
ABCC5	-0.16433	0.158297	0.374432
AC005838.	0.002533	0.158379	0.374585
LOC10192!	-0.04174	0.158473	0.374733
TMEM196	0.005677	0.158476	0.374733
ZBED8	-0.23603	0.158513	0.37478
MSL1	0.116375	0.158647	0.375054
FAM183CF	-0.0173	0.158676	0.375082
PAPOLB	-0.00642	0.158743	0.375199

PHF19	0.180437	0.158892	0.375511
C19orf83	-0.10927	0.158986	0.375691
C21orf119	0.115033	0.159027	0.375748
HBBP1	0.294317	0.159085	0.375796
C10orf32	-0.12322	0.159098	0.375796
ICOS	0.048502	0.159099	0.375796
LOC10050(	-0.09608	0.159224	0.37605
HN1L	0.101399	0.15927	0.376117
NDUFAF4	-0.12451	0.15946	0.376525
MAFG-AS1	0.034004	0.159527	0.376642
TMOD2	-0.1478	0.159547	0.376648
PPP2R2A	-0.18052	0.159586	0.376675
FAM19A1	0.008935	0.159593	0.376675
RBMS3	0.017632	0.159727	0.376949
MCUR1	0.126924	0.159805	0.377092
METTL18	-0.20861	0.15984	0.377103
TRIL	0.010609	0.159844	0.377103
STT3B	-0.19031	0.159939	0.377286
AC010524.	-0.02435	0.159966	0.377287
GABRA3	-0.00635	0.159975	0.377287
NDUFAF7	0.099912	0.160096	0.377533
CPXM2	-0.01536	0.160119	0.377545
GPR6	-0.01264	0.160181	0.377649
SLC2A9	0.063781	0.160264	0.377806
TGM1	-0.02485	0.160323	0.377904
RIMS1	0.036156	0.160361	0.377951
MAP7	0.16106	0.160395	0.377991
FOXD3	0.00503	0.160437	0.378049
GAL	0.158137	0.160491	0.378135
LOC10028(	-0.01	0.160566	0.378269
HEATR4	-0.03226	0.160629	0.378373
TRHDE	0.165906	0.160645	0.378373
LFNG	0.053868	0.160663	0.378375
AEBP1	0.22099	0.160684	0.378383
CHD2	0.128572	0.160701	0.378383
ABCB6	0.108122	0.160867	0.378708
RAB24	0.153719	0.160875	0.378708
TNFRSF11E	0.00996	0.161019	0.379007
LOC10192	0.002108	0.161041	0.379019
MRGPRX4	-0.04846	0.161093	0.379098
TEX2	-0.11474	0.161149	0.379164
FERMT1	0.230266	0.161168	0.379164
LOC10192(	-0.07623	0.161173	0.379164
AK3	-0.1313	0.16123	0.379256
LINC00598	-0.01624	0.161297	0.379273
GCAT	0.171128	0.161306	0.379273
KEL	0.221926	0.161306	0.379273
LOC10050(	-0.03414	0.161307	0.379273
ICE2	-0.10154	0.161375	0.379392
C1orf86	0.01299	0.161404	0.379418
MEDAG	-0.02847	0.161454	0.379494
RDH12	0.015115	0.161476	0.379506
GFRA4	0.008483	0.161541	0.379593
ZBTB48	0.161784	0.161557	0.379593
LOC10192(	0.005205	0.161566	0.379593
GABRG2	0.026108	0.161658	0.379768
AIM1L	0.031938	0.161714	0.379858
RP11-456F	0.140606	0.161871	0.380186

PDGFD	-0.19722	0.161917	0.380254
RP1-74M1	-0.0193	0.161944	0.380275
SNAPC3	0.107006	0.161996	0.380356
TMEM194E	-0.13849	0.162022	0.380375
LOC40076	-0.01522	0.162039	0.380375
LINC00485	0.025701	0.162086	0.380443
TFPI2	-0.15317	0.162146	0.380544
SVOP	-0.01701	0.162197	0.380621
CBLN4	-0.0082	0.162233	0.380665
PRTFDC1	-0.26059	0.162269	0.380683
TBCA	0.088493	0.16228	0.380683
DYX1C1	-0.14442	0.162294	0.380683
KIF13B	0.13168	0.16235	0.380774
LEFTY1	-0.08066	0.162437	0.380896
RPA1	0.134231	0.162444	0.380896
LOC10192	0.002413	0.162461	0.380896
LY6G5C	0.198384	0.162472	0.380896
FBLIM1	-0.03749	0.162496	0.38091
C5orf24	0.194591	0.162518	0.380922
FAM98A	-0.12538	0.162608	0.381064
CDH9	-0.41233	0.162614	0.381064
DKFZP434I	-0.01456	0.162655	0.381078
SCTR	0.006062	0.162655	0.381078
C16orf45	-0.0642	0.162705	0.381146
LIME1	0.104259	0.16272	0.381146
RAB40B	-0.09726	0.162789	0.381266
LOC15005	-0.05143	0.162842	0.381351
AX746710	-0.00498	0.162884	0.3814
UBXN8	0.118612	0.162899	0.3814
CDH13	-0.01097	0.16296	0.381503
POF1B	0.004434	0.163003	0.381562
A2M-AS1	-0.23811	0.16304	0.381588
RP3-507I1	-0.01015	0.163049	0.381588
DQ582785	-0.00472	0.163118	0.381694
EPM2A	0.126061	0.16313	0.381694
NT5C1A	-0.02873	0.163155	0.381711
MFSD11	-0.08524	0.163295	0.381967
UBL5	0.099577	0.163304	0.381967
GIPC1	0.093356	0.163317	0.381967
SLC25A30	-0.06371	0.163438	0.382195
CRTC2	0.119845	0.16345	0.382195
RP11-295N	-0.15127	0.163483	0.382208
TAX1BP1	0.105075	0.163498	0.382208
PLEKHG4B	0.137631	0.163508	0.382208
RP11-456H	-0.02767	0.163541	0.382243
GFOD1	-0.19762	0.163659	0.382476
HSD17B7	-0.08137	0.163715	0.382568
KPNA4	-0.10802	0.163787	0.382693
MBLAC1	-0.10641	0.163846	0.38279
EYA2	0.001623	0.163901	0.382878
RP11-340A	0.002225	0.163995	0.383057
PRMT5	-0.12307	0.164044	0.383119
ABRA	0.00916	0.164057	0.383119
TTC27	-0.13839	0.164078	0.383127
RAB4B	-0.04362	0.16411	0.38316
SLC39A14	-0.04361	0.164178	0.383277
GNA13	0.178209	0.164198	0.383283
RP11-495K	0.068095	0.16424	0.38332

STAG3L4	-0.1018	0.164274	0.38332
MYBPC2	-0.01496	0.164285	0.38332
TNFAIP3	0.33191	0.164285	0.38332
FCN2	0.118718	0.164347	0.383385
BC041025	0.264678	0.164349	0.383385
C18orf12	-0.03564	0.164396	0.383455
CRNDE	-0.28499	0.164423	0.383478
AGPS	-0.12403	0.164478	0.383534
PARP6	0.097368	0.164483	0.383534
FLJ21369	-0.02324	0.164702	0.384002
NKD2	0.058648	0.164742	0.384023
HEXB	-0.12743	0.164753	0.384023
ATXN3L	-0.0081	0.164774	0.384023
DENND1C	0.155297	0.164786	0.384023
RP11-862L	-0.02692	0.164804	0.384023
KLHL20	-0.08834	0.164852	0.384023
DUSP21	0.045622	0.164863	0.384023
LINC00622	-0.11919	0.164883	0.384023
HOXB8	0.038234	0.164899	0.384023
RHBDL2	-0.00721	0.164901	0.384023
AGAP1	0.085108	0.164906	0.384023
ADD1	-0.06609	0.164923	0.384023
ABCB9	-0.06216	0.164989	0.384135
PCOLCE2	0.345664	0.165027	0.384169
LOC10013(	0.004369	0.16505	0.384169
LOC28519:	-0.03309	0.165057	0.384169
FLJ38773	-0.02197	0.165253	0.384575
DSCR9	0.033168	0.165279	0.384575
CTBS	0.164212	0.165285	0.384575
NEIL3	-0.26745	0.16536	0.38471
ATOH1	-0.01055	0.165388	0.384732
MAP3K15	-0.0193	0.165458	0.384853
GGNBP2	0.117295	0.165475	0.384853
LIMD1	0.072944	0.16553	0.38492
RP11-6F2.!	-0.00889	0.165549	0.38492
C20orf203	-0.06773	0.165557	0.38492
PICK1	0.077665	0.165602	0.384948
TEX261	0.093355	0.165623	0.384948
ZNF662	-0.20601	0.165623	0.384948
ANP32B	-0.05247	0.165658	0.384989
CDC73	-0.12313	0.165682	0.385003
CASKIN1	-0.01086	0.165835	0.385318
GABRA4	0.020003	0.165878	0.385342
SMTNL1	-0.02114	0.165881	0.385342
WDR59	-0.08571	0.165972	0.385493
KRT2	0.003287	0.165982	0.385493
WDR70	0.130559	0.166016	0.385532
SOX3	-0.00943	0.166043	0.385552
SPAG11A	0.037436	0.166086	0.38561
CHGA	0.021206	0.166142	0.385699
TTC37	-0.23424	0.166165	0.385712
PRSS35	0.043445	0.166193	0.385735
AHSA2	-0.20281	0.166264	0.385763
SMA4	-0.17297	0.166268	0.385763
IGHV3-54	0.020465	0.166277	0.385763
NUDT4	0.023545	0.166284	0.385763
OR12D3	0.004767	0.166294	0.385763
TNFRSF10E	-0.05758	0.166357	0.385868

GPSM1	-0.07736	0.166419	0.38597
GHRL	-0.04474	0.166508	0.386062
KIAA1671	-0.01756	0.166509	0.386062
STX18-AS1	-0.06519	0.166512	0.386062
IGK	0.215628	0.166561	0.386134
RARS2	0.043866	0.166632	0.386225
LAMB2	0.24431	0.166636	0.386225
LINC00474	-0.01479	0.166713	0.386362
FTCD	-0.00417	0.166755	0.38642
LRFN3	0.022084	0.166825	0.386539
PRDM14	-0.02446	0.166998	0.386822
FAM209B	-0.07692	0.167001	0.386822
FSHB	-0.02022	0.16701	0.386822
ZNF335	-0.04238	0.167037	0.386822
C2orf43	-0.13159	0.167052	0.386822
AP001462.	0.106989	0.167054	0.386822
CLCA1	-0.02419	0.167175	0.387035
LINC00462	-0.00342	0.167182	0.387035
PROX2	-0.00962	0.167348	0.387373
OSBPL3	0.139256	0.167364	0.387373
C10orf71	-0.04204	0.167497	0.387641
PVRL1	0.030538	0.167517	0.387644
LOC64275	0.0114	0.167538	0.387652
BLACAT1	-0.01927	0.167626	0.387814
RP11-753D	0.006625	0.167663	0.387858
DDN	0.011736	0.167849	0.388203
RP11-218I	0.00983	0.16785	0.388203
LRRC75A	0.151578	0.167878	0.388203
LOC10192	0.005483	0.167884	0.388203
ZNF259P1	0.013264	0.16808	0.388615
TAF4	-0.06186	0.168143	0.38872
CCDC36	-0.00748	0.168233	0.388859
ZNF91	-0.08391	0.168253	0.388859
AQP11	0.027013	0.168257	0.388859
LOC64648	-0.05538	0.168297	0.388909
DHX16	-0.10485	0.168346	0.388982
RP11-4M2	-0.02365	0.168409	0.389086
DCP2	-0.17409	0.168545	0.389358
PAGE1	-0.02275	0.168639	0.38951
DOCK8	0.171232	0.168647	0.38951
KRT7	-0.00165	0.168805	0.389833
RP11-21G	-0.01086	0.168934	0.390089
GEM	0.275736	0.168963	0.390092
FDCSP	-0.00499	0.168992	0.390092
LOC10272	-0.00316	0.169007	0.390092
GSDMC	-0.02256	0.16901	0.390092
TGOLN2	0.145752	0.169025	0.390092
RPS19	-0.04737	0.169069	0.390152
LINC00608	-0.0324	0.169153	0.390267
TARP	-0.02879	0.169155	0.390267
IL1RAP	-0.22345	0.169225	0.390384
KLRB1	0.160016	0.169254	0.390384
RAB10	0.119256	0.169269	0.390384
LARGE	0.041619	0.169278	0.390384
PCAT19	0.081174	0.1693	0.390394
MIR7-3HG	-0.00988	0.169364	0.390475
BPTF	-0.10151	0.169371	0.390475
LOC10192	0.014261	0.169396	0.390487

ZFYVE16	-0.23716	0.169413	0.390487
SPTSSB	-0.0209	0.169483	0.390607
RP11-539L	0.18945	0.169583	0.390798
TMEM184(	0.100119	0.169792	0.391195
LOC10192!	0.004162	0.169792	0.391195
CDK5RAP1	-0.10964	0.169815	0.391206
SMYD5	0.15614	0.169848	0.391218
GPR61	0.00339	0.169856	0.391218
PTK6	0.010852	0.169888	0.39125
UG0898H0	0.022198	0.170101	0.391662
LOC10013:	0.006354	0.170111	0.391662
UGCG	0.304035	0.170121	0.391662
MYOT	-0.00569	0.170209	0.391756
KIAA0509	-0.00561	0.170234	0.391756
ZCCHC6	0.165655	0.170234	0.391756
HLA-DPB2	0.072917	0.170244	0.391756
NDP	0.011789	0.170253	0.391756
EYA1	0.091693	0.170293	0.391808
MET	0.004963	0.170363	0.391882
HABP4	-0.081	0.170392	0.391882
TCP11L2	0.030766	0.170409	0.391882
RP11-727F	0.01821	0.170418	0.391882
VSTM2L	0.038155	0.170428	0.391882
RIT1	-0.23194	0.170434	0.391882
NBAS	-0.16757	0.170464	0.391909
TRIM63	0.055368	0.17049	0.391927
STL	0.053606	0.170523	0.391961
ALG5	0.016864	0.170605	0.392109
SUPT3H	-0.14244	0.170646	0.392161
B3GNT3	-0.03389	0.170808	0.392492
ATP8B5P	-0.00534	0.170836	0.392515
SSTR5-AS1	-0.00755	0.170867	0.392544
LOC33962:	-0.00406	0.170959	0.392715
LINC00880	-0.01574	0.171076	0.392942
RRP8	-0.04598	0.171426	0.393697
LPPR2	0.038238	0.17145	0.393697
POPDC2	-0.05089	0.171459	0.393697
LOC10192!	-0.01084	0.171519	0.393792
POU6F1	-0.03087	0.171624	0.393962
BC042590	-0.11299	0.171641	0.393962
CLYBL-AS2	-0.02285	0.171667	0.393962
RPL38	-0.12767	0.171683	0.393962
KDM8	0.04162	0.171699	0.393962
C7orf57	-0.01859	0.171702	0.393962
BEST3	0.027612	0.171724	0.39397
AC010145.	-0.01222	0.171756	0.394001
USP39	0.115474	0.171869	0.394219
SMO	-0.10135	0.171929	0.394316
ELOVL5	-0.14764	0.172	0.394435
MMACHC	0.176176	0.172036	0.394478
KCNB2	-0.00687	0.172093	0.394565
RP1-217P2	-0.03662	0.172185	0.394648
LINC01212	-0.01073	0.172193	0.394648
TM2D3	-0.13531	0.172218	0.394648
UQCRB	-0.08763	0.172228	0.394648
SRF	-0.05275	0.17225	0.394648
HTR1B	-0.01106	0.172252	0.394648
LOC40109!	-0.01157	0.172256	0.394648

IL9	0.00279	0.172302	0.39471
LOC40056{	-0.01636	0.172322	0.394714
FLJ31356	-0.02521	0.17234	0.394714
WFDC5	-0.0079	0.17239	0.394752
EVA1C	-0.23615	0.172393	0.394752
TXNRD1	0.037864	0.172448	0.394762
PHC1	0.113884	0.17245	0.394762
BC040833	0.016045	0.172452	0.394762
TRAPPC3L	-0.12371	0.172477	0.394777
ORC4	-0.1075	0.172511	0.394814
HDAC6	-0.05191	0.172534	0.394824
PMM2	0.119987	0.172694	0.39506
ZNF304	-0.13363	0.172697	0.39506
ALPP	0.016096	0.172705	0.39506
DNHD1	-0.04549	0.17271	0.39506
TPT1-AS1	-0.13518	0.172826	0.395285
MKNK1	0.178593	0.172928	0.395463
MAS1	0.004299	0.172957	0.395463
C17orf96	-0.11377	0.172959	0.395463
LOC10050	0.079704	0.173002	0.39552
RGS13	-0.07753	0.173061	0.395613
CTD-2256I	-0.04619	0.173141	0.395748
WDR47	-0.11672	0.173157	0.395748
EPHB6	0.135695	0.173189	0.39578
SUGT1P1	-0.07566	0.17322	0.395808
CLIC5	0.011163	0.17324	0.395814
HEATR5B	-0.11769	0.173436	0.396192
SUGT1P3	-0.09133	0.173455	0.396192
RASL11A	-0.00222	0.173461	0.396192
OR1J4	-0.02831	0.173482	0.396198
SELL	-0.23407	0.173579	0.39637
SHISA4	-0.05764	0.173594	0.39637
BPIFA2	-0.01219	0.17364	0.396435
TMEM151I	-0.02214	0.17369	0.396506
RCHY1	-0.09456	0.173755	0.396613
BARX1-AS	-0.01077	0.173791	0.396654
PLCXD2	-0.14011	0.173968	0.397015
CMTM1	-0.03905	0.173994	0.397032
ADORA1	-0.03055	0.174044	0.397105
MFGE8	0.040857	0.174189	0.397395
SLC2A13	0.027258	0.174215	0.397412
KRTAP4-2	-0.01367	0.174245	0.397438
RDH16	-0.0462	0.174295	0.39751
HECA	0.199509	0.174335	0.397529
PTBP1	-0.03826	0.17435	0.397529
GPC5	0.206051	0.174358	0.397529
SOX21-AS	-0.01882	0.174382	0.39753
NOL3	0.04236	0.174396	0.39753
PYGO2	-0.03919	0.174437	0.397571
LOC10050(	0.039369	0.17445	0.397571
FAM172A	0.133089	0.174597	0.397825
N4BP2L2	-0.11951	0.174628	0.397825
CHRNA2	-0.06587	0.174632	0.397825
KLHL7	-0.21792	0.174642	0.397825
DHFRL1	-0.12861	0.174661	0.397825
RAP2C	-0.20476	0.174672	0.397825
FGFR1OP2	0.187402	0.174793	0.398007
OTOF	-0.00475	0.174798	0.398007

CDC20	0.297947	0.174807	0.398007
CCDC89	0.016154	0.174853	0.398069
LOC40117(	-0.01616	0.174951	0.398251
HS3ST3B1	-0.1469	0.175015	0.398319
CBX8	-0.0306	0.175033	0.398319
PGP	0.125955	0.175053	0.398319
PIP5KL1	0.050207	0.175055	0.398319
C7orf73	-0.13173	0.175099	0.398378
FAR2	-0.15137	0.175137	0.398423
PDK3	0.214868	0.175167	0.398451
ACOT9	0.136777	0.17534	0.398801
SDHD	0.063894	0.175361	0.398806
TMEM54	0.005986	0.175394	0.398815
R3HDM4	0.161549	0.175418	0.398815
TYRO3P	0.007005	0.17542	0.398815
LRRC10B	-0.0303	0.175549	0.399038
NPHP3-AS	0.001717	0.175555	0.399038
FBXL6	0.062584	0.17559	0.399047
LOC10028(	-0.16861	0.175595	0.399047
AIPL1	0.010439	0.175645	0.399118
RP11-339A	0.005399	0.175732	0.399273
GNAO1	-0.04666	0.17577	0.399317
PRR7-AS1	-0.00755	0.175806	0.399357
RAD17	0.113177	0.175899	0.399527
TMEM238	0.017046	0.175928	0.39953
PIDD1	0.07989	0.175941	0.39953
DGKI	0.067161	0.175956	0.39953
COL10A1	-0.13157	0.175974	0.39953
RPS6KA2-/	-0.0298	0.175998	0.399542
KIDINS220	-0.1758	0.176068	0.39959
KMT2E	0.143531	0.176079	0.39959
TNFSF9	0.104469	0.176097	0.39959
PYCRL	0.082283	0.176112	0.39959
LOC10272(	-0.0199	0.176114	0.39959
TENM1	-0.03527	0.17613	0.39959
MBD1	0.082044	0.176183	0.399669
EDIL3	0.001853	0.176206	0.399681
DNM1P46	0.019296	0.176254	0.399746
CHM	-0.11623	0.176272	0.399746
PRSS58	0.019749	0.17632	0.399812
NDUFA4L2	-0.01606	0.176383	0.399913
PACSIN3	0.046647	0.176427	0.399972
PLD6	-0.00625	0.176448	0.399978
ZNF502	-0.11055	0.176528	0.400117
RHCG	0.023445	0.17656	0.400146
LINC01343	-0.02049	0.176608	0.400148
LOC10192(	-0.01511	0.17661	0.400148
KAL1	0.166842	0.176616	0.400148
RP3-476K8	-0.00924	0.176743	0.400395
KLHDC8A	0.042866	0.176794	0.400469
CDK2AP1	0.095184	0.176858	0.400548
CLU	0.287091	0.176866	0.400548
FAM210A	-0.11816	0.177008	0.40077
TGFB1I1	0.306915	0.177011	0.40077
LOC10192(	-0.00475	0.17702	0.40077
PLAU	-0.19411	0.177162	0.401051
PGD	0.005796	0.177372	0.401484
TMEM27	-0.08088	0.177391	0.401484

NPAS2	0.003161	0.177428	0.401526
LOC10050(	-0.03451	0.177476	0.401592
CSTF2	0.113905	0.177538	0.40169
SIX4	0.010989	0.177609	0.40181
LOC10192	0.002421	0.177744	0.401996
BARX1	0.016062	0.17775	0.401996
GPR158	0.004501	0.177765	0.401996
CER1	-0.03335	0.177783	0.401996
PPM1A	-0.10393	0.177784	0.401996
RNF139	-0.09	0.177816	0.402027
LINC00525	0.00756	0.177892	0.402157
LIN9	-0.02591	0.177984	0.402322
OVOL3	-0.00436	0.178107	0.402559
MARVELD:	-0.06225	0.178208	0.402734
HS1BP3	0.019284	0.178222	0.402734
TPRG1	0.03277	0.178245	0.402744
DYNLL2	0.132402	0.178327	0.402887
LINC01310	-0.0221	0.178375	0.402923
OR5L2	-0.02052	0.17838	0.402923
MRPL1	0.136991	0.178455	0.403006
BDKRB1	-0.01953	0.178461	0.403006
LRRC52	-0.00393	0.178473	0.403006
PTPN14	-0.17605	0.178532	0.403097
KLHL2	0.131409	0.178612	0.403236
HIST1H1E	-0.21235	0.178669	0.403323
KRT31	-0.01906	0.178706	0.403348
AP000230.	-0.02605	0.178717	0.403348
FLJ45513	-0.12515	0.17884	0.403583
KIAA0125	-0.21202	0.178877	0.403623
AL022341.:	0.026013	0.178922	0.403684
TNFSF15	0.064285	0.178956	0.403718
ARMCX3	-0.12248	0.178992	0.403757
JHDM1D-/	0.111468	0.179029	0.403796
MLC1	0.18043	0.179046	0.403796
KIAA1432	0.078736	0.17911	0.403898
TCTN3	-0.10315	0.179136	0.403914
ARX	-0.01769	0.179175	0.403919
LINC01333	0.005004	0.179175	0.403919
PLCD3	0.028607	0.179233	0.404007
ATP2B1	0.276136	0.179292	0.404098
BOLA1	0.152159	0.179462	0.404411
RP11-48B3	-0.23993	0.179472	0.404411
PROSER1	0.089821	0.179487	0.404411
PROK1	0.007788	0.179543	0.404496
SNRPD3	-0.11095	0.17958	0.404537
RNF112	-0.02235	0.179599	0.404537
MGAT3	0.104876	0.179631	0.404567
DNAJB5	0.028071	0.17965	0.404567
HMX2	-0.02179	0.17975	0.40475
ZNF14	-0.15379	0.179828	0.404883
DEDD	0.116242	0.179881	0.404929
NUP93	0.063247	0.179885	0.404929
EPHA1	-0.01519	0.179928	0.404982
HEXIM1	0.139989	0.179964	0.405006
RP11-252E	0.087484	0.179994	0.405006
LINC01101	-0.01346	0.179994	0.405006
LOC10192	-0.00669	0.180032	0.405049
BPIFB4	0.011918	0.180231	0.405453

PDHX	0.076676	0.180256	0.405469
LOC10014	-0.12087	0.180302	0.405473
AP000473.	0.071403	0.180318	0.405473
PHF13	0.154285	0.180329	0.405473
IDS	-0.14901	0.180333	0.405473
NGFRAP1	-0.10574	0.18041	0.405604
LRRC71	-0.03201	0.180465	0.405686
MAS1L	-0.01113	0.180486	0.405691
NEU3	-0.01463	0.180529	0.405745
FARSA	0.148526	0.180551	0.405747
SCAF11	0.104734	0.180567	0.405747
GPR84	0.301017	0.180611	0.405804
LOC25405	-0.22863	0.18076	0.406072
SNORD8	0.045364	0.180778	0.406072
TBC1D16	0.120234	0.180787	0.406072
HS6ST3	-0.00352	0.180946	0.406388
LOC10192	0.095025	0.181144	0.406749
LOC10192	-0.00931	0.181145	0.406749
LRRC75B	0.02552	0.181186	0.406797
SHISA8	-0.01203	0.181203	0.406797
CRYBA4	0.004839	0.181234	0.406823
PPP1R8	0.099795	0.18128	0.406884
RP3-508D	-0.00989	0.181321	0.406903
OSTC	-0.09101	0.18133	0.406903
CHMP4C	-0.29848	0.181345	0.406903
FAM118A	0.223769	0.181423	0.407037
KCNIP2	0.004307	0.18145	0.407055
DGCR14	-0.04243	0.181494	0.40711
LOC10050	-0.00792	0.181552	0.407189
LAMP2	0.15647	0.181566	0.407189
UBE4B	0.09536	0.181603	0.407201
PPIL2	0.095585	0.181612	0.407201
CCDC64	-0.03039	0.181628	0.407201
PAN2	0.129563	0.181689	0.407277
NLGN1	-0.14546	0.1817	0.407277
UBASH3B	0.162494	0.181738	0.407322
TIFAB	0.238397	0.181758	0.407323
SLC5A11	-0.04852	0.181846	0.40748
ARC	0.007479	0.182005	0.407793
F2RL1	-0.12974	0.182049	0.407847
GTF3C5	0.092331	0.182092	0.407847
OXR1	0.162828	0.182092	0.407847
PWWP2B	-0.06244	0.182105	0.407847
KAT7	0.160069	0.182149	0.407893
MUC13	-0.02812	0.182163	0.407893
TNIP1	0.138601	0.182334	0.408235
DYNC2H1	-0.07796	0.182358	0.408247
RP3-359N	-0.03227	0.182381	0.408255
3-Mar	0.123364	0.182438	0.408308
POLD2	0.161106	0.182461	0.408308
KY	-0.00948	0.182477	0.408308
CRYAA	-0.00247	0.18248	0.408308
RMND5A	0.11056	0.182534	0.408349
IRG1	0.229856	0.18254	0.408349
LOC10192	-0.02295	0.182569	0.408349
DEFB122	0.006221	0.182574	0.408349
LINC00317	-0.01133	0.182596	0.408356
NLRP11	0.071061	0.182672	0.408484

ST3GAL3	-0.00724	0.182822	0.408778
EPHA5-AS	0.048674	0.182917	0.408948
MTX1	0.108673	0.182951	0.408982
SLCO4A1-	-0.01787	0.182973	0.408988
HCRTR2	0.007534	0.183402	0.409906
LINC00271	-0.04564	0.183447	0.409963
ESM1	0.101129	0.183499	0.410037
THAP4	0.103183	0.183579	0.410175
GUCY1B2	0.01156	0.183625	0.410233
TTC23	-0.01935	0.183666	0.410241
ZNF337	-0.14052	0.183714	0.410241
BEX5	-0.4212	0.183715	0.410241
ADH1A	-0.04877	0.183721	0.410241
HERPUD1	-0.1349	0.183737	0.410241
GPR114	-0.12677	0.183742	0.410241
NEU2	-0.02648	0.183778	0.41028
FAM46C	0.256012	0.183921	0.41051
ECHS1	0.098335	0.183933	0.41051
LOC10192	0.022605	0.183944	0.41051
CPLX2	-0.01658	0.183957	0.41051
CHST14	0.124262	0.184021	0.410611
METTL2B	0.086903	0.184306	0.411204
VWC2	0.009812	0.184326	0.411205
LINC00642	0.006864	0.184355	0.411209
TGIF2LY	-0.00922	0.184371	0.411209
RCBTB2	0.101194	0.184384	0.411209
PKP3	-0.01612	0.18444	0.411264
AAR2	-0.10879	0.184448	0.411264
TMEM102	0.032451	0.184466	0.411264
LOC10192	-0.03738	0.184496	0.41128
LOC28627	0.260922	0.184529	0.41128
FGFR1	0.102628	0.18453	0.41128
RP11-44F1	0.039489	0.184574	0.411336
LOC28319	-0.075	0.184599	0.411349
SPATS1	-0.02721	0.18463	0.411366
CTB-12A1	0.129823	0.184644	0.411366
FAM217B	0.159488	0.184695	0.411436
TXNDC2	-0.02724	0.184741	0.411495
C9orf172	-0.0285	0.184759	0.411495
SLC6A2	-0.00899	0.184804	0.411552
C6orf165	-0.03724	0.184999	0.411944
NRG2	-0.00882	0.185024	0.411958
LINC01448	0.006932	0.185052	0.411978
SEC61A2	-0.10928	0.185139	0.412085
TDRD5	0.018791	0.185156	0.412085
RP11-248J	-0.16556	0.185157	0.412085
LOC28338	-0.01071	0.185262	0.41221
DCAF4L2	-0.01216	0.185272	0.41221
FAM83H	0.070983	0.185282	0.41221
LINC01015	-0.03774	0.18529	0.41221
SYCP1	-0.06655	0.185395	0.412402
GSTT2	0.041649	0.18549	0.41257
GRIN2C	-0.00733	0.185552	0.412666
FLG-AS1	0.045968	0.185647	0.412788
KLHL1	-0.00979	0.185663	0.412788
NKX3-1	-0.01559	0.185664	0.412788
EEA1	0.200416	0.185734	0.412901
LOC10192	-0.05236	0.185837	0.413089

MGC39584	-0.02122	0.185911	0.413179
UBXN7-AS	-0.09633	0.185916	0.413179
KCTD16	-0.00978	0.186001	0.413324
PBX4	-0.09882	0.186106	0.413511
LOC72809	-0.03365	0.186125	0.413511
ZNF605	-0.0705	0.186163	0.413511
SEZ6	-0.02305	0.186178	0.413511
APOBEC3F	-0.05937	0.18618	0.413511
GCNT2	-0.10146	0.186202	0.413518
ROCK2	0.0663	0.186235	0.413548
NISCH	-0.07902	0.1863	0.413613
KRT23	0.073499	0.186323	0.413613
PIWIL2	-0.01735	0.186333	0.413613
ZDHHC2	-0.12073	0.186341	0.413613
SLC17A5	0.122973	0.18639	0.413657
ADIPOQ	0.019881	0.186399	0.413657
RASA2	-0.18775	0.18647	0.413773
PRAC1	0.060843	0.186553	0.413914
THOC2	-0.08347	0.186665	0.4141
HSPA12B	0.042895	0.186675	0.4141
TMEM151A	-0.01869	0.186731	0.414183
KAT2B	0.201564	0.186793	0.414275
RPL27	-0.03305	0.186811	0.414275
SLC25A29	-0.18812	0.186911	0.414405
LOC10192	-0.01937	0.186936	0.414405
CFHR3	0.034523	0.186943	0.414405
RP11-701I	0.016249	0.186946	0.414405
CDX4	-0.00195	0.18706	0.414615
SYPL2	-0.03611	0.18713	0.414728
IHH	-0.04007	0.187227	0.4149
TMEM255E	0.064271	0.187288	0.414978
CCDC172	-0.03075	0.187314	0.414978
OXSM	-0.13282	0.18732	0.414978
RNASE3	0.428206	0.187344	0.414988
LYRM1	-0.11337	0.187409	0.41506
STX2	0.11828	0.187414	0.41506
MTHFD1L	-0.02265	0.187467	0.415134
ZG16	-0.02031	0.187493	0.415149
YES1	0.223902	0.18752	0.415166
TMEM128	0.137819	0.187587	0.415272
LINS	-0.09902	0.18762	0.415281
ITPK1	-0.03365	0.187629	0.415281
LINC00883	-0.10393	0.187684	0.41536
DDX46	-0.10742	0.187746	0.415455
LOC10050	-0.25074	0.187779	0.415485
HR	-0.01987	0.187798	0.415485
LOC40089	0.026655	0.187905	0.41568
ARHGAP5-	-0.09001	0.187926	0.415683
HCN3	-0.05122	0.188055	0.415926
LOC1720	-0.01313	0.188144	0.416071
ZNF124	-0.19581	0.188159	0.416071
KSR2	-0.01324	0.188217	0.416152
KLF17	-0.01074	0.188257	0.416152
CCDC87	-0.01252	0.188286	0.416152
ARFGAP1	0.108539	0.188306	0.416152
EPHA5	0.107	0.18832	0.416152
BUB1B	-0.19521	0.188328	0.416152
PROP1	0.0091	0.188342	0.416152

MEP1B	-0.00663	0.188349	0.416152
DQ570835	-0.30099	0.18839	0.416182
COL25A1	0.013861	0.188402	0.416182
RP11-769(	-0.19005	0.188622	0.416626
ZNF692	0.144068	0.188668	0.416629
EMC3-AS1	0.141426	0.188673	0.416629
CORT	-0.09129	0.188696	0.416629
DACH1	0.213287	0.188726	0.416629
MRVI1-AS	0.018979	0.18874	0.416629
KRT77	0.003692	0.188756	0.416629
LOC15786(	-0.06576	0.188758	0.416629
TMX4	0.119453	0.188816	0.416716
ZNF83	-0.14709	0.188857	0.416735
YAP1	0.039015	0.188881	0.416735
LOC10192	-0.00732	0.188883	0.416735
LOC10028	0.059774	0.188907	0.416747
HSD3B7	0.212553	0.188929	0.416752
XAGE-4	-0.13196	0.189042	0.416959
LINC00936	0.263938	0.189066	0.41697
VCP	0.092511	0.189165	0.417146
WDR31	0.019218	0.189196	0.417171
RAB39A	0.092591	0.18925	0.417234
TNNI2	0.161902	0.189263	0.417234
GRB7	-0.01289	0.18953	0.417738
LOC73010	0.096985	0.18953	0.417738
AK091729	0.050769	0.189628	0.417912
MOV10L1	0.004163	0.189717	0.417965
FBXO40	-0.01402	0.189719	0.417965
LOC10192	-0.01533	0.189729	0.417965
IL36B	0.005402	0.18973	0.417965
ARRDC4	-0.22903	0.18978	0.418001
MIR210HG	-0.00634	0.189785	0.418001
KIAA1161	0.014232	0.189835	0.418004
KLHL36	0.113	0.189843	0.418004
TTC40	0.011672	0.189844	0.418004
MED22	0.151289	0.189866	0.41801
COBL	0.104476	0.189945	0.418141
NPY1R	0.05663	0.189974	0.418163
AC005606.	0.006849	0.190025	0.418234
LINC01204	0.005691	0.19008	0.418312
ZCCHC12	-0.00984	0.190173	0.418473
VIL1	-0.04779	0.190397	0.418923
USMG5	0.088377	0.19049	0.419086
QRICH2	-0.05609	0.190552	0.419173
POLR2F	-0.07802	0.190594	0.419173
CTC-428G	0.044486	0.1906	0.419173
NUFIP2	0.095971	0.190607	0.419173
CCL20	0.358591	0.190627	0.419174
PCAT18	0.204895	0.190734	0.419368
FLJ45482	-0.20195	0.190766	0.419394
PXMP2	-0.15551	0.190851	0.419538
TMEM242	-0.0865	0.190919	0.419591
LOC10272	0.007751	0.190929	0.419591
BAZ2A	0.06779	0.190933	0.419591
CYP1B1-A	-0.00806	0.191048	0.419801
NIPA1	0.075986	0.191111	0.419871
MBD3L2	0.003562	0.191125	0.419871
GNA14	-0.02163	0.191138	0.419871

CTXN3	0.001828	0.19121	0.419988
SLC12A6	-0.19753	0.191296	0.420103
MFSD1	0.11778	0.191302	0.420103
ELOVL2-AS	0.00439	0.191342	0.420141
LOC101928	0.006439	0.191358	0.420141
PRMT8	-0.01781	0.191394	0.420178
HCN1	0.037295	0.191414	0.420178
VARS	0.100633	0.191556	0.420419
AANAT	0.007962	0.191562	0.420419
KIRREL3-A	-0.02436	0.191632	0.420503
LOC101927	-0.00209	0.191639	0.420503
SPATA33	0.075564	0.191664	0.420514
ZCCHC3	0.053257	0.191812	0.420797
SGK223	0.319221	0.191831	0.420797
RTN4RL2	0.002637	0.191867	0.420833
RPS6KB1	-0.07497	0.191919	0.420904
LRRC2	0.133077	0.191947	0.420923
DPH7	-0.08227	0.192019	0.420981
LOC100506	-0.02124	0.192031	0.420981
CNTNAP4	0.002746	0.192048	0.420981
BC062753	-0.08455	0.192059	0.420981
IL9R	-0.03121	0.192071	0.420981
PRO2214	-0.00851	0.192124	0.42103
C17orf82	-0.01299	0.192132	0.42103
LOC102725	-0.01358	0.192237	0.421217
H2BFM	-0.03026	0.192264	0.421227
MYRF	0.017159	0.19228	0.421227
RP11-298D	0.006339	0.192374	0.421354
DYNC1I1	-0.04241	0.192377	0.421354
SLC12A1	-0.02582	0.192462	0.421496
RP11-109E	-0.01184	0.192577	0.421706
ZBED4	0.094582	0.192622	0.421761
LOC102723	-0.0156	0.192652	0.421771
TBC1D19	-0.1366	0.192665	0.421771
BC070118	-0.00852	0.192744	0.421888
SYNE1	0.024073	0.192772	0.421888
TEX26	-0.00765	0.19279	0.421888
DCP1B	0.126872	0.192796	0.421888
HSPBAP1	0.099808	0.192829	0.421917
LINC00917	0.005137	0.192872	0.421941
GJA3	-0.03351	0.192879	0.421941
SPRR1A	-0.02229	0.192915	0.421978
TET3	0.094587	0.192963	0.422025
RP11-433A	0.007258	0.192976	0.422025
RP11-334J	-0.00591	0.193169	0.422406
DPP10-AS1	-0.0049	0.193263	0.422568
CCDC92	0.180258	0.193309	0.422627
SLC25A33	-0.13669	0.193438	0.422864
AC012499.	-0.02203	0.193645	0.423244
OSGIN2	-0.07807	0.19365	0.423244
OVOL1-AS	-0.04494	0.193692	0.423292
CCM2	-0.1149	0.193762	0.423393
SMARCAL1	0.093437	0.193778	0.423393
TXNL4A	-0.129	0.193815	0.423432
ATP2A1	-0.01423	0.19384	0.423445
ISLR2	-0.01295	0.193875	0.42347
PDE4D	-0.09609	0.193891	0.42347
TEKT5	0.005034	0.19392	0.423491

ADCK2	0.140167	0.194025	0.423678
BOD1L1	0.201007	0.194051	0.423692
BC043291	-0.01153	0.194116	0.42375
LOC10013:	0.004757	0.194122	0.42375
KIAA0087	-0.29386	0.194136	0.42375
NMRAL1	-0.10788	0.194224	0.423898
MSRB2	-0.13265	0.19427	0.423953
MAGEA1	0.043472	0.194288	0.423953
RP11-300/	0.0115	0.194311	0.42396
ABCB11	0.018838	0.194388	0.424081
PKM	0.119577	0.194418	0.424081
SLC18A3	-0.01076	0.194441	0.424081
DCT	0.004557	0.194445	0.424081
STIM2	-0.10038	0.194475	0.424084
PCDHA10	0.031927	0.194499	0.424084
KLHDC4	0.07868	0.194505	0.424084
JAGN1	-0.11162	0.194545	0.424114
LGR4	-0.03877	0.194584	0.424114
MYH13	0.013864	0.194595	0.424114
PHGR1	0.013059	0.194597	0.424114
GPRIN3	0.097413	0.194623	0.424118
LINC00888	-0.09073	0.194649	0.424118
GUCY1A2	0.001992	0.194658	0.424118
LELP1	-0.02064	0.194709	0.424146
SCARB1	0.146065	0.19471	0.424146
SPATA12	0.005057	0.19484	0.424387
TMCC3	0.178796	0.194864	0.424397
LOC10254(	-0.00984	0.194896	0.424424
ZNF274	-0.13394	0.194967	0.424425
AP001171.	-0.25507	0.194968	0.424425
SCMH1	0.161104	0.194969	0.424425
BAAT	0.029499	0.194975	0.424425
GPX8	0.05404	0.19508	0.424611
TRIB3	0.108395	0.195144	0.424707
ABCB8	-0.02496	0.195212	0.424799
KRTAP4-9	0.005979	0.195225	0.424799
IFNA10	-0.00616	0.195261	0.424834
ZPBP2	-0.00999	0.195371	0.425029
PROL1	-0.04532	0.19539	0.425029
PHYHIPL	-0.01285	0.195436	0.425031
RP11-54K1	0.094546	0.195441	0.425031
NOB1	-0.14029	0.195456	0.425031
AGFG1	-0.16113	0.195473	0.425031
ZNF575	-0.06493	0.195498	0.425031
RNF6	-0.02633	0.195508	0.425031
ITPR1	0.158359	0.195547	0.425073
RP11-692F	0.046084	0.195588	0.425118
SLC26A7	0.004773	0.195679	0.425274
FGFR4	-0.01037	0.195719	0.425318
RIPPLY3	-0.02203	0.195768	0.425382
C8orf22	-0.00561	0.195915	0.425659
PHB2	0.126497	0.196002	0.425719
LINC00330	0.01923	0.19606	0.425719
KRT12	-0.00804	0.196074	0.425719
CXorf22	-0.0375	0.196091	0.425719
TRPC3	-0.02253	0.196103	0.425719
PRR34-AS:	0.111652	0.196124	0.425719
TDRG1	-0.06924	0.196129	0.425719

CLDN2	0.031974	0.196135	0.425719
LOC44117!	0.006458	0.196137	0.425719
PLD5	0.006098	0.196139	0.425719
HTR3A	-0.01091	0.196186	0.425749
PPP1R10	0.176671	0.196218	0.425749
LOC10050!	-0.01221	0.196236	0.425749
KIAA1467	-0.01745	0.196265	0.425749
LOC10192	-0.00427	0.196267	0.425749
TRMT61B	-0.09721	0.196271	0.425749
C6orf163	0.087007	0.196297	0.425763
LOC10192	0.020272	0.196349	0.425833
LOC28569	-0.00137	0.196443	0.425926
CPSF3L	0.058501	0.196446	0.425926
LOC10192!	-0.01214	0.196451	0.425926
RP11-79P5	-0.00888	0.196519	0.426031
HABP2	-0.01163	0.196546	0.426047
HAL	-0.28086	0.196624	0.426172
ZNF419	0.096506	0.196664	0.426218
OPTC	-0.05728	0.196701	0.426254
MIPOL1	-0.00865	0.196726	0.426266
JUP	0.260918	0.196762	0.426285
AOX2P	0.051092	0.196796	0.426285
MYOZ3	-0.00916	0.196803	0.426285
GAREML	0.067157	0.196814	0.426285
RGS6	-0.03982	0.196946	0.426496
SST	-0.00704	0.19699	0.426496
SLITRK6	0.145405	0.197001	0.426496
LOC10050!	-0.13989	0.197007	0.426496
PTPRD	-0.19235	0.19701	0.426496
POLR3C	0.118294	0.19708	0.426606
GS1-166A	-0.13217	0.197143	0.4267
LOC10192!	0.014376	0.197182	0.426741
FW340027	-0.03488	0.197276	0.426903
RP11-110!	-0.01409	0.197342	0.426976
SMIM6	0.07972	0.197359	0.426976
OR11A1	0.006481	0.197398	0.426976
LOC10192!	-0.00432	0.197433	0.426976
TIMM10	0.150023	0.197462	0.426976
IVL	-0.02428	0.197482	0.426976
GATC	-0.08596	0.197485	0.426976
GPR82	0.104062	0.197485	0.426976
CTBP1-AS	-0.02303	0.197506	0.426976
PRO1082	0.083555	0.197507	0.426976
CATSPER3	-0.06426	0.197604	0.427142
LOC72988	0.037417	0.19763	0.427157
RFX6	-0.00357	0.197679	0.42722
PARN	-0.08935	0.197755	0.427324
LINC00610	-0.04947	0.197767	0.427324
CAPN10	0.033324	0.197801	0.427342
ZDHHC22	-0.01476	0.197815	0.427342
GEMIN5	0.202594	0.197906	0.427455
DCST2	-0.01411	0.197907	0.427455
ITGA2	-0.15385	0.19793	0.427464
INSC	0.00706	0.197997	0.427566
VEPH1	-0.05562	0.198051	0.427638
HTR1E	0.017709	0.198119	0.4277
HFE2	-0.02963	0.198135	0.4277
LOC38976!	-0.14575	0.198138	0.4277

OBP2B	0.008195	0.198186	0.427759
GIMAP2	-0.18711	0.198239	0.427827
RP11-143I:	0.009921	0.198257	0.427827
LOC10192	0.037351	0.198474	0.428241
FGF16	-0.0027	0.198488	0.428241
WIZ	0.04472	0.198596	0.428431
LOC34488	-0.03427	0.19864	0.42844
DSP	0.022325	0.198657	0.42844
B9D2	0.100576	0.198659	0.42844
CACNG8	-0.04167	0.198704	0.428493
LOC10192	0.027005	0.198767	0.428588
PCGF6	-0.11901	0.198807	0.42863
FAM53A	-0.07871	0.198895	0.428777
LINC01165	0.020346	0.198987	0.428933
C10orf90	-0.01196	0.19902	0.428962
MSTO1	0.096171	0.199047	0.428977
CSNK1A1P	-0.00618	0.199089	0.429025
C21orf67	-0.00141	0.199183	0.429185
LARS2	0.091704	0.199219	0.42922
LOC10192	-0.00698	0.199264	0.429268
GUCA1A	-0.00699	0.199281	0.429268
LOC10013	-0.25416	0.199305	0.429276
RP1-187B2	0.002284	0.199337	0.429293
SLC7A14	-0.01287	0.199365	0.429293
IL1RN	0.047169	0.199379	0.429293
BPIFA4P	-0.00625	0.199392	0.429293
HCG26	-0.17037	0.199434	0.429342
PGLYRP4	-0.02818	0.199517	0.429474
PAPSS2	0.194574	0.199535	0.429474
7-Sep	-0.12713	0.199753	0.429859
LINC00313	0.008313	0.199754	0.429859
LINC01098	0.008889	0.199808	0.429887
AGPAT4-IT	0.193003	0.199819	0.429887
TMEM165	-0.08017	0.199827	0.429887
ABCA17P	0.188012	0.199934	0.430075
GNN	0.001878	0.200112	0.430416
FEN1	-0.18069	0.200136	0.430419
PER2	-0.07588	0.200153	0.430419
LMF1	-0.08778	0.200229	0.430516
ALMS1-IT1	-0.23624	0.20024	0.430516
RWDD3	-0.16258	0.200258	0.430516
OLIG3	-0.00419	0.200372	0.430695
CRLS1	-0.14782	0.200381	0.430695
ADAMDEC	0.083304	0.200406	0.430706
RP11-127E	-0.17341	0.200495	0.430853
C20orf78	0.004668	0.2006	0.431037
MARVELD2	-0.15662	0.200741	0.431297
C20orf173	-0.00433	0.200763	0.431302
PDE6A	-0.02968	0.200848	0.431441
NARF	0.074097	0.200876	0.431459
TCEANC2	-0.06428	0.200924	0.43152
ST3GAL4-/	-0.18221	0.200952	0.431537
CHRNB4	-0.0037	0.20103	0.431617
CILP	0.054274	0.201041	0.431617
CDK13	0.147751	0.201049	0.431617
ACKR4	0.026433	0.201116	0.431717
RELT	0.145516	0.201203	0.431861
LINC00302	-0.00657	0.201254	0.431929

RP11-544D	0.006265	0.201375	0.432146
BTBD2	0.056914	0.201422	0.432165
MGC32805	0.010162	0.201424	0.432165
EGFLAM-A	-0.0025	0.201479	0.43224
DLEU7-AS	0.006464	0.201527	0.432301
MSRA	-0.04827	0.201561	0.43233
SUV39H1	0.138992	0.2016	0.43237
IQCF3	0.004607	0.201693	0.432527
DDX21	-0.09451	0.201715	0.432533
ADRA2A	0.306493	0.201754	0.432536
PTGIR	0.059411	0.201757	0.432536
CAV2	0.303357	0.201851	0.432696
GPRC5A	0.001492	0.201912	0.432783
UNK	-0.17494	0.201968	0.432791
DHRS7	0.135724	0.201979	0.432791
C1orf56	-0.05386	0.202	0.432791
LOC10192	-0.01665	0.202007	0.432791
ZNF618	-0.15992	0.202016	0.432791
PDLIM1	-0.24831	0.202085	0.432897
LINC00626	-0.00446	0.20222	0.433125
CHIT1	0.075004	0.202232	0.433125
LOC10192	-0.01385	0.202291	0.433209
SLC26A9	-0.02865	0.202343	0.433278
CENPI	-0.05044	0.202387	0.433328
NEUROG2	-0.00413	0.202505	0.433538
SLC3A1	-0.02691	0.202591	0.433679
SLC5A5	-0.03189	0.202611	0.43368
NDUFAF1	-0.14223	0.202667	0.433718
PRRG2	-0.01187	0.20268	0.433718
EDDM3A	0.005016	0.202703	0.433718
E2F4	0.025654	0.202709	0.433718
CES1P1	-0.03275	0.202758	0.433759
LRRC37A4	0.194505	0.202768	0.433759
LINC00895	0.006967	0.202795	0.433774
DIO1	0.011776	0.20284	0.433777
MST4	-0.16841	0.202846	0.433777
INHBB	0.008773	0.202857	0.433777
LDHA	-0.05937	0.203026	0.434097
BPIFB1	0.040094	0.203087	0.434185
PLS1	-0.25768	0.203118	0.434207
DDX43	-0.30358	0.20315	0.434232
MAL2	0.01083	0.203263	0.434364
HAS2	0.042332	0.203269	0.434364
CRYZ	-0.14548	0.203276	0.434364
MKRN1	0.119546	0.203292	0.434364
LOC10012	-0.04258	0.203353	0.434452
NDUFB2-A	0.050737	0.203496	0.434715
BC039686	-0.03698	0.203545	0.434776
KIAA1468	0.167781	0.203577	0.434802
STRIP2	0.127977	0.203642	0.434899
LOC10192	-0.01485	0.203667	0.434908
CDC40	-0.05889	0.20375	0.435044
SLC5A10	-0.0635	0.203836	0.435183
RP11-214	0.064536	0.203906	0.435282
GSPT2	-0.16975	0.203922	0.435282
GABRG3	-0.00391	0.20399	0.435383
GLO1	-0.08431	0.204025	0.435415
LINC00458	0.008957	0.204114	0.435563

PUS10	-0.11667	0.204173	0.435645
TMSB4Y	0.094962	0.2042	0.435661
C11orf87	0.011464	0.204305	0.435719
LOC10050(	-0.00847	0.204322	0.435719
COL1A2	0.174863	0.204343	0.435719
RP11-258(	-0.18154	0.204352	0.435719
GPM6B	-0.23212	0.204353	0.435719
CDY1	-0.00753	0.204371	0.435719
TREH	0.003426	0.204387	0.435719
GCSAM	-0.16312	0.204389	0.435719
RGN	-0.0256	0.204495	0.435903
LOC10013:	0.008253	0.204597	0.436078
BZRAP1	-0.31629	0.204621	0.436085
EIF4A2	-0.00841	0.204714	0.43624
AMBP	-0.00637	0.204755	0.436286
TRMT13	-0.18884	0.204884	0.436517
HOXD-AS2	-0.00242	0.204957	0.436629
CDC42EP2	-0.02304	0.205089	0.436869
RP11-102(	-0.01829	0.205226	0.437118
ATG16L1	-0.12903	0.205291	0.437212
ZNF541	0.030247	0.205382	0.437364
BAI1	-0.04238	0.205428	0.437418
MAT2B	-0.04893	0.205484	0.437454
CHI3L1	0.215814	0.205485	0.437454
FAM81A	-0.01347	0.205598	0.437648
LOC10192!	-0.00439	0.205622	0.437648
LUZP1	-0.1239	0.205637	0.437648
CLCNKB	0.003432	0.205706	0.437751
DKK4	0.004541	0.205755	0.437813
MOS	-0.0055	0.205819	0.437855
LOC10192!	-0.01048	0.20584	0.437855
RP11-373D	0.315137	0.205853	0.437855
COL19A1	0.003476	0.205881	0.437855
C12orf49	-0.12575	0.205897	0.437855
ZFR2	-0.02235	0.205911	0.437855
AKR1E2	-0.14141	0.205916	0.437855
LONRF1	-0.21391	0.205955	0.437894
LINC00572	-0.00649	0.205991	0.437928
CEP170B	-0.0358	0.20606	0.437968
KIFC1	-0.16812	0.206091	0.437968
DANCR	0.201292	0.206096	0.437968
FRZB	0.073089	0.206119	0.437968
SEMA5B	-0.05154	0.206125	0.437968
AASDHPPT	-0.09873	0.206162	0.437968
CACNA2D4	0.086173	0.206169	0.437968
RTKN	-0.0753	0.206172	0.437968
CATX-1	-0.00343	0.206206	0.437993
GPCPD1	-0.13684	0.206224	0.437993
MEIS1	-0.21261	0.206271	0.438012
SCUBE3	-0.02937	0.206273	0.438012
NPAS3	0.004002	0.206298	0.438022
MAT2A	0.135258	0.206567	0.438517
SYNGR3	0.171322	0.206572	0.438517
ANKRD50	-0.16371	0.206634	0.438606
GJD3	0.009101	0.206665	0.438613
STARD13	0.054077	0.206678	0.438613
GAL3ST1	-0.0268	0.206703	0.438624
BMP8A	-0.01249	0.206728	0.438633

CLCN6	0.112825	0.206777	0.438694
PRO1483	-0.12093	0.206851	0.438808
MAMDC2-	-0.03299	0.20693	0.438868
BTBD17	-0.00739	0.206953	0.438868
NOL9	0.126744	0.20696	0.438868
POFUT1	-0.05451	0.20696	0.438868
PZP	0.01408	0.207042	0.438998
KLRG1	0.102611	0.207069	0.439013
LINC01159	-0.02614	0.207097	0.439029
MRC2	0.123547	0.207244	0.439277
LHX2	0.004828	0.207254	0.439277
RIBC1	-0.02073	0.207328	0.439391
CNR2	-0.02045	0.207387	0.439461
PARD6G	0.03359	0.207402	0.439461
IGHM	0.163794	0.207453	0.439484
KIR2DL3	-0.02449	0.207482	0.439484
GAST	0.036894	0.207482	0.439484
ZNF90	-0.0287	0.207501	0.439484
CH25H	0.097138	0.207514	0.439484
ITGAM	0.314355	0.207738	0.439897
SHISA7	0.025748	0.207771	0.439897
TMEM61	-0.01279	0.207778	0.439897
MRFAP1L1	0.126278	0.207791	0.439897
FLJ35700	-0.00861	0.207841	0.43996
RP11-397A	-0.01096	0.208008	0.440216
BIN3-IT1	-0.1064	0.208018	0.440216
TMEM203	0.101128	0.208023	0.440216
PRKAR1B	-0.03504	0.208177	0.440467
CLCN1	0.02596	0.208182	0.440467
ST14	0.190971	0.208232	0.44053
FAIM	-0.14254	0.208284	0.440565
FBXL22	-0.00236	0.208289	0.440565
PHIP	-0.14984	0.208328	0.440604
RALA	0.111536	0.208368	0.440645
LOC285095	0.007645	0.208429	0.440732
PANX2	-0.01243	0.208476	0.440788
LINC01093	0.055917	0.20856	0.440923
NT5DC1	0.099385	0.208599	0.440941
KCTD11	0.091387	0.208609	0.440941
ZFP64	-0.02064	0.20872	0.441092
ELAC1	0.109952	0.208728	0.441092
CTC-384G	-0.02421	0.208763	0.441092
CILP2	-0.02081	0.208763	0.441092
NUP205	0.081558	0.208802	0.441132
PNRC1	0.12283	0.209021	0.441513
DHX33	0.122801	0.209023	0.441513
RP11-847H	-0.01743	0.209085	0.441577
DAZL	-0.01009	0.209118	0.441577
SLC4A4	-0.01466	0.209165	0.441577
FBN3	-0.03439	0.209171	0.441577
PRR14L	-0.07273	0.209172	0.441577
TNP2	-0.00585	0.209198	0.441577
PNMA2	0.026786	0.209222	0.441577
NAT6	0.164069	0.209228	0.441577
ARHGEF11	0.012974	0.209237	0.441577
RP11-410C	-0.02043	0.209326	0.441722
PHF2P1	0.012155	0.209373	0.441778
LOC101928	-0.00969	0.209448	0.441893

EVI2A	-0.16848	0.209505	0.441959
LOC10192!	-0.04756	0.209531	0.441959
LOC33998!	-0.10561	0.20954	0.441959
RP11-1103	-0.01415	0.209784	0.442429
KIF26B	-0.03215	0.209839	0.442502
P2RX7	0.136812	0.209883	0.442552
LURAP1L	-0.10675	0.209932	0.442612
ST3GAL4	-0.0612	0.209953	0.442615
LINC01213	0.007522	0.210116	0.442915
AC079807.	0.003308	0.210163	0.442971
LOC10192	0.002577	0.210248	0.443107
ADAMTS5	0.11175	0.210287	0.443145
MYO16	0.10239	0.21034	0.443213
ENTHD1	-0.01047	0.210461	0.443392
BMS1P6	-0.18968	0.210465	0.443392
NEURL1B	-0.2268	0.210562	0.443553
FZD4	0.074989	0.210594	0.443567
RP11-63A1	0.006847	0.21061	0.443567
ZFYVE28	0.01437	0.210664	0.443621
LOC10050	-0.01771	0.210676	0.443621
ERV9-1	-0.02655	0.21076	0.443629
CHRM4	-0.00332	0.21076	0.443629
LOC28612	0.001759	0.210767	0.443629
SLC45A4	0.170791	0.210795	0.443629
MTMR10	0.066664	0.210805	0.443629
SNX17	-0.12031	0.210819	0.443629
CD52	-0.23893	0.21083	0.443629
LINC01279	0.061166	0.210844	0.443629
LIPH	-0.0653	0.210921	0.443737
STAC3	-0.12047	0.210943	0.443737
CCDC13-A	-0.0096	0.210957	0.443737
ATXN1L	0.081082	0.211135	0.444037
ZNF280A	0.001684	0.211142	0.444037
CDIPT-AS1	-0.02099	0.211161	0.444037
GDAP2	-0.08858	0.211206	0.444089
MS4A5	0.011602	0.211285	0.444177
OSBP	-0.13659	0.211305	0.444177
TTC32	-0.0993	0.211335	0.444177
LOC40069	-0.0255	0.211346	0.444177
XRCC6BP1	-0.07841	0.211351	0.444177
KIF20B	0.116055	0.211377	0.44419
NEGR1-IT1	0.010329	0.211444	0.444267
ERLIN1	-0.13688	0.211455	0.444267
MAP2K5	-0.12907	0.211497	0.444312
RP4-595K1	0.149606	0.211534	0.444345
CTNNBIP1	-0.10836	0.211568	0.444345
LOC72869	-0.01374	0.211574	0.444345
TRIM2	-0.05814	0.211597	0.444349
LOC10192	-0.01176	0.211749	0.444625
CRIP2	0.020467	0.211845	0.444784
GDF15	0.032912	0.211912	0.444881
SRBD1	0.104814	0.21196	0.444933
RP5-1103C	0.004175	0.211978	0.444933
CYP4F30P	-0.00225	0.21202	0.444978
GPN3	0.095121	0.212075	0.445014
AC007401.	-0.10165	0.212078	0.445014
LOC10050!	-0.004	0.212118	0.445021
CT62	0.025322	0.212122	0.445021

SLC7A2	-0.00266	0.212171	0.445081
BC048132	-0.04453	0.212244	0.445181
KXD1	0.026829	0.21226	0.445181
SLC25A32	-0.10531	0.21231	0.445241
KRT84	-0.00633	0.212494	0.445531
CRB3	-0.03651	0.212506	0.445531
LOC10192٤	-0.09917	0.212517	0.445531
LOC10192٤	0.145016	0.21253	0.445531
PRR9	-0.0029	0.212661	0.445762
SRY	0.004034	0.21274	0.445886
IAH1	-0.1032	0.212894	0.446082
RWDD2B	0.160899	0.212901	0.446082
MKLN1	0.119922	0.212908	0.446082
LOC10192!	-0.01011	0.212922	0.446082
GBAP1	0.114663	0.212937	0.446082
FLJ39080	-0.02938	0.213031	0.446197
STRBP	-0.1362	0.213033	0.446197
CHD3	0.122099	0.213068	0.446227
TRPC5	-0.00707	0.213119	0.44629
ARHGEF12	0.00278	0.213248	0.446495
CDHR1	-0.00547	0.213263	0.446495
EYA3	-0.07382	0.213278	0.446495
PCNA	-0.12588	0.213358	0.446584
MROH2A	0.009581	0.213382	0.446584
LOC10192!	-0.05558	0.213383	0.446584
DHX30	0.09068	0.213419	0.446595
LOC10013(	-0.01284	0.213452	0.446595
LOC10192٤	0.003432	0.213467	0.446595
SDCCAG8	0.105276	0.213471	0.446595
TECPR1	0.088759	0.213534	0.446648
LOC34001	0.001493	0.213537	0.446648
ZNF708	0.14501	0.213677	0.446897
PRKAB1	0.119041	0.2137	0.446903
DTD2	-0.10411	0.213739	0.44694
UBE2G1	-0.07601	0.21379	0.447001
KRTAP4-4	0.013266	0.213809	0.447001
LINC01260	0.024325	0.213921	0.44715
BC043227	-0.00678	0.213923	0.44715
LOC10050	0.079615	0.213942	0.44715
FAM122A	-0.12625	0.214015	0.447257
LOC33997٤	0.003594	0.214035	0.447257
LOC10027	0.019389	0.214058	0.447262
DDX60	0.226448	0.214126	0.447361
RP11-649/	-0.07081	0.214177	0.447395
OCA2	-0.06568	0.214183	0.447395
NEUROD2	-0.02501	0.214225	0.4474
CDKN2AIP	0.146197	0.21428	0.4474
RALGAPA1	-0.09608	0.21429	0.4474
P2RY14	-0.44853	0.214325	0.4474
MRPL51	0.083839	0.214327	0.4474
ALKBH6	-0.1095	0.214338	0.4474
DEFB132	0.010007	0.214346	0.4474
NXPH1	0.008718	0.214356	0.4474
SHC4	-0.0107	0.214372	0.4474
LINC00269	0.010583	0.214397	0.44741
LOC10192٤	0.004055	0.214462	0.447497
TSPY1	-0.01903	0.21448	0.447497
AMOT	-0.0227	0.214526	0.447547

HIST1H2BJ	-0.13509	0.214546	0.447547
C9orf173	-0.03369	0.214604	0.44759
C5orf66-A	0.003468	0.214628	0.44759
LEPREL4	-0.14077	0.214649	0.44759
KIAA1661	-0.00628	0.214662	0.44759
RP11-791N	-0.00668	0.214672	0.44759
LOC441124	-0.11678	0.214706	0.44759
LOC10192	-0.21783	0.214711	0.44759
REEP2	-0.03921	0.214766	0.447624
ZNF549	0.002681	0.21478	0.447624
FAM3B	0.129208	0.214789	0.447624
SDR16C5	0.014772	0.214863	0.447734
FKBP6	-0.01722	0.214901	0.447771
LOC10192	-0.03666	0.21494	0.447809
CLDN3	0.009718	0.215153	0.448153
AC024560.	0.094644	0.215163	0.448153
SEMA3A	0.022386	0.215167	0.448153
BC045789	-0.09625	0.215206	0.448153
SERPINA3	0.056513	0.215225	0.448153
CELF5	-0.02182	0.21523	0.448153
FMNL1	0.114575	0.215272	0.448199
LINC00943	-0.0117	0.215339	0.44827
RP11-587D	0.003084	0.215348	0.44827
CYP46A1	-0.0371	0.215393	0.448321
AKAP10	0.133269	0.21542	0.448334
TAS2R50	0.006243	0.215457	0.448369
OR52D1	0.016737	0.21551	0.448436
TMEM41B	-0.1145	0.215559	0.448493
BNC1	-0.00706	0.215579	0.448493
TSHZ1	0.11045	0.215681	0.448661
EMX1	-0.01607	0.215914	0.449054
PFN4	0.015919	0.215918	0.449054
RP11-18I14	-0.03802	0.215932	0.449054
TP53INP2	0.182632	0.215974	0.449099
LOR	0.001946	0.216025	0.449163
TIMM13	-0.06462	0.216252	0.449591
GSPT1	-0.10759	0.216275	0.449595
SPINK13	-0.00324	0.216514	0.449966
CKM	0.085001	0.216516	0.449966
KAT2A	-0.10279	0.216528	0.449966
SPOCK1	0.083706	0.216541	0.449966
LCMT2	-0.09808	0.216557	0.449966
KIAA1549	0.194845	0.216595	0.449989
LOC10013	-0.00248	0.21663	0.449989
LINC00308	-0.00845	0.216631	0.449989
RFXAP	-0.09663	0.216683	0.450054
FASTKD2	-0.1614	0.216786	0.450226
LINC00051	0.003524	0.216848	0.450311
LOC10159	-0.04864	0.216915	0.450381
SHROOM1	-0.0513	0.216923	0.450381
LOC61326	-0.00643	0.216964	0.450405
SCN10A	-0.02589	0.21698	0.450405
SNRPN	-0.0152	0.217017	0.450405
HMGA2	-0.00868	0.217018	0.450405
FBXO4	0.058417	0.217077	0.450484
EVX1	-0.0316	0.217147	0.45055
B3GALNT2	-0.03249	0.21715	0.45055
PKLR	0.106047	0.217218	0.450648

ZNF334	-0.18508	0.21726	0.45069
SYCN	0.00134	0.21734	0.450813
CCL19	-0.00775	0.217414	0.450924
RPP38	0.099858	0.217543	0.451149
PPP1R2P9	-0.01197	0.217663	0.451353
C1orf210	-0.02324	0.217708	0.451404
DBIL5P	0.049546	0.217749	0.451445
C3orf18	-0.11589	0.217854	0.45162
LOC10050!	-0.01037	0.217962	0.451771
RUNDC1	0.070922	0.217982	0.451771
POLR3D	-0.11216	0.217989	0.451771
OSCP1	-0.02622	0.218109	0.451977
PRO2958	0.001871	0.218151	0.45202
MYCNOS	-0.03638	0.218199	0.452076
C1orf127	-0.01914	0.218291	0.452157
SUSD4	-0.02267	0.218298	0.452157
FABP7	0.002471	0.2183	0.452157
IPO9	-0.09413	0.218366	0.452168
ABHD17C	0.237185	0.218369	0.452168
C19orf43	0.095461	0.218369	0.452168
ARR3	-0.03043	0.218435	0.452263
TRAV8-3	-0.00902	0.218497	0.452348
DLK1	0.384039	0.218622	0.452498
LOC10106(	0.124445	0.218624	0.452498
LINC01348	-0.01905	0.218632	0.452498
ASPN	0.008485	0.218681	0.452555
RP11-674F	0.003024	0.218744	0.452641
IL13RA2	0.197538	0.218783	0.452661
FGFBP1	-0.01366	0.218814	0.452661
MAP3K1	0.147674	0.218816	0.452661
GLIS3	0.064317	0.218946	0.452886
RP5-1119A	-0.05563	0.218978	0.452886
CTD-2373:	-0.0181	0.218987	0.452886
ASB14	-0.03066	0.219047	0.452966
BCKDHA	-0.11626	0.219146	0.453128
LINC00163	0.009816	0.219195	0.453185
ACTL9	0.005303	0.21931	0.453381
RRS1	0.119132	0.219351	0.453401
CA10	-0.0052	0.219379	0.453401
RP1-305G2	-0.0127	0.219386	0.453401
KCNN3	0.002077	0.219404	0.453401
GDAP1L1	-0.04204	0.219479	0.453513
FANCM	0.103583	0.219572	0.453594
RP5-1102E	0.021389	0.219573	0.453594
LOXHD1	0.004059	0.219581	0.453594
ST8SIA1	-0.04519	0.219607	0.453604
ZNF253	-0.09307	0.219697	0.453746
TULP2	0.018964	0.219797	0.453911
RP11-680(	-0.00946	0.219855	0.453932
RP11-642D	-0.10853	0.219863	0.453932
RAB21	0.101412	0.219886	0.453932
LOC10099(	-0.01121	0.219897	0.453932
C7orf33	-0.01284	0.219912	0.453932
DHRS9	0.325973	0.219982	0.454033
KIAA1328	-0.03947	0.220077	0.454185
USP35	0.027704	0.220115	0.45422
ETAA1	-0.08379	0.2202	0.454353
APLNR	0.065808	0.220226	0.454363

ZSCAN2	-0.08524	0.220338	0.454551
KIR2DS4	-0.0044	0.220465	0.45477
AHDC1	-0.02212	0.220548	0.454871
CHIAP2	0.003657	0.220556	0.454871
ZNF252P-/	0.005105	0.220603	0.454924
RPE65	0.010814	0.220647	0.454972
LOC40062:	-0.02045	0.220775	0.455192
LINC00486	0.003149	0.220807	0.455209
RPL39	-0.02491	0.220825	0.455209
MGC4294	-0.01002	0.2209	0.455315
LOC10272	-0.00849	0.220919	0.455315
AREL1	0.04968	0.220968	0.455358
DPM1	-0.08445	0.220992	0.455358
CEACAM3	0.04498	0.221003	0.455358
RRM1	0.123872	0.221029	0.455368
PLXNA2	0.030023	0.221112	0.455496
UBL4A	-0.13535	0.221134	0.455499
HIPK4	-0.01833	0.221326	0.455851
MMP21	-0.01879	0.221394	0.455947
ANKMY1	0.083694	0.221448	0.456014
CD300LG	-0.0186	0.221506	0.456026
CPSF1	-0.03425	0.221515	0.456026
ITGB1BP1	0.233682	0.221517	0.456026
EPHB1	0.031436	0.221582	0.456117
LOC10050	-0.0039	0.221642	0.456197
A4GNT	-0.00231	0.221757	0.456366
TRMT2A	0.130852	0.221766	0.456366
RASSF2	0.138072	0.221852	0.456499
TEAD3	-0.02552	0.221884	0.456522
CD34	-0.22942	0.221954	0.456623
THUMPD2	-0.08809	0.221993	0.456661
CT83	0.012981	0.222032	0.456696
KDM2A	0.109669	0.222117	0.456829
CSTA	0.350632	0.222162	0.45687
LOC10192	-0.03448	0.222179	0.45687
ZNF208	-0.04298	0.222208	0.456884
SETD4	-0.13182	0.222265	0.456952
PRELID2	-0.10709	0.222283	0.456952
BSN	-0.00494	0.222407	0.457164
GCKR	0.037162	0.222431	0.45717
A2MP1	-0.02358	0.22251	0.457289
SDF2	0.103301	0.222554	0.457313
KCNK16	0.002837	0.222564	0.457313
ZNF691	0.123364	0.222585	0.457313
LRRC32	0.049	0.222813	0.457712
LOC10192	-0.00348	0.222821	0.457712
LRRC3B	0.04668	0.222877	0.457742
CXCR1	-0.04891	0.222911	0.457742
ZBTB3	-0.10015	0.222916	0.457742
NLRC5	0.147848	0.222948	0.457742
TAGLN3	0.017782	0.222954	0.457742
CPLX1	0.103554	0.222963	0.457742
LINC00682	0.003	0.223098	0.457976
MT1G	0.372452	0.223196	0.458133
LOC10013	0.004909	0.223239	0.458151
CCDC178	-0.01768	0.223261	0.458151
HCN4	-0.02655	0.223268	0.458151
CCDC38	-0.04992	0.223291	0.458156

LRTM1	-0.0407	0.223322	0.458175
NCOR2	-0.04645	0.223346	0.45818
ABCA5	-0.11904	0.2234	0.458248
FASTKD3	-0.09687	0.223435	0.458276
ETNK2	-0.02989	0.223457	0.458279
RGS17	-0.06015	0.22348	0.458283
FEM1B	-0.12275	0.223514	0.458309
OR5P3	-0.01077	0.223566	0.458372
GRIK3	-0.00831	0.223642	0.458484
PCSK9	-0.01217	0.22371	0.458547
PXDNL	0.00914	0.223734	0.458547
LOC10272	-0.01337	0.223736	0.458547
NMUR2	0.013204	0.223865	0.458768
MS4A12	-0.00485	0.224075	0.459154
CTAGE5	-0.1763	0.224155	0.459274
HDAC1	-0.05963	0.224191	0.459283
TNN	-0.04556	0.224201	0.459283
CELF6	0.095786	0.224278	0.459306
ARTN	0.032025	0.224283	0.459306
LMO7DN	0.024023	0.224318	0.459306
KIAA0226	-0.04729	0.22432	0.459306
COPS5	0.08115	0.224336	0.459306
NEUROG1	-0.00604	0.224361	0.459306
IGHV1-69	-0.20596	0.224382	0.459306
KCNE1L	0.266103	0.224382	0.459306
PPEF1	0.003633	0.224411	0.459321
KRT19P2	0.001655	0.224513	0.459486
C1orf213	-0.11549	0.224681	0.459787
UPK3A	-0.02243	0.224726	0.459837
EMC6	-0.06346	0.224803	0.459951
LGALSL	0.290051	0.224842	0.459986
TNRC6B	-0.11047	0.224914	0.460077
RP4-621B1	-0.01945	0.224929	0.460077
DDX18	0.068491	0.22497	0.460118
PRSS50	-0.02818	0.225098	0.46032
PADI2	-0.01526	0.225111	0.46032
SLC32A1	0.020998	0.225151	0.46035
XPNPEP1	0.108624	0.225169	0.46035
NUP210L	-0.02751	0.225206	0.460376
NSUN3	0.078438	0.225242	0.460376
CACNA1S	0.030171	0.225245	0.460376
SLC1A7	-0.01067	0.225275	0.46039
LOC10192	-0.0048	0.225295	0.46039
RP11-539(	0.001823	0.225321	0.460401
SMR3B	-0.01891	0.225408	0.460534
TAGLN	0.033204	0.22543	0.460537
CA3	0.148013	0.225468	0.46057
ARHGEF18	0.122336	0.225507	0.460572
ETNK1	0.123169	0.22553	0.460572
ZNF512B	0.120437	0.225533	0.460572
SLC38A2	0.105579	0.225598	0.460657
RIIAD1	0.020393	0.225617	0.460657
GPR128	0.229919	0.225665	0.460687
AIG1	-0.13734	0.225674	0.460687
SF1	-0.24077	0.225697	0.46069
MST1L	-0.01474	0.225721	0.460696
ATL3	0.164995	0.225821	0.460857
CECR5-AS	0.01138	0.22596	0.461096

AC018755.	-0.00273	0.22599	0.461115
GNA14-AS	-0.02025	0.226024	0.461141
QSOX2	0.081938	0.226062	0.461174
SLC26A10	0.007606	0.226176	0.461365
CAPN2	0.09684	0.226235	0.461418
RP11-395I	-0.20201	0.226265	0.461418
PPP1R9A	-0.2082	0.226287	0.461418
RGS8	-0.01163	0.226288	0.461418
LOC10192	-0.0533	0.226469	0.461745
APOL2	0.076627	0.226497	0.461758
DEFB126	-0.00256	0.226602	0.461929
ZNF844	0.079592	0.226657	0.461997
LOC28636	-0.14558	0.226696	0.462002
ULK4	-0.05668	0.226702	0.462002
MRPS16	0.096587	0.226801	0.46216
C11orf31	-0.06375	0.226933	0.462318
GPR126	0.186252	0.226939	0.462318
DGCR10	-0.01336	0.226942	0.462318
ZNF275	-0.10949	0.226967	0.462322
AP4E1	-0.00897	0.226987	0.462322
RP1-155D	-0.01467	0.227009	0.462324
BOLL	-0.00358	0.227089	0.462428
CORO1A	0.187674	0.227124	0.462428
MEPE	-0.00565	0.227124	0.462428
RP11-37C	-0.01257	0.22717	0.462478
RP11-362	0.083495	0.22724	0.46257
FAM219B	0.119579	0.227258	0.46257
C15orf27	-0.01523	0.227306	0.46261
RP11-436	0.01006	0.227321	0.46261
UMPS	-0.09705	0.227376	0.462645
LINC00673	0.006225	0.22739	0.462645
BMP6	-0.01561	0.227402	0.462645
CD164	0.17969	0.227435	0.462668
KLHL34	-0.02188	0.227528	0.462813
TNFRSF8	-0.02966	0.227592	0.462901
GMFB	-0.11525	0.227622	0.462902
NUDT11	-0.32046	0.227635	0.462902
CEP57	-0.12618	0.227691	0.46296
STAP1	-0.27381	0.227717	0.46296
C1orf50	0.094068	0.227728	0.46296
HOOK2	0.057016	0.227753	0.462967
GALNT3	-0.12839	0.22779	0.462999
FAM193A	-0.08528	0.22782	0.463017
GBP6	0.006554	0.227932	0.463201
ING5	-0.09463	0.228016	0.463328
UHRF1	0.249594	0.228207	0.463672
RP11-378J	-0.01415	0.228236	0.463687
GGA3	0.058729	0.228263	0.463699
HSD17B3	-0.0269	0.228287	0.463705
MYO6	0.046244	0.228326	0.463707
IQSEC3	0.006947	0.228333	0.463707
FGF14-AS	-0.12823	0.228359	0.463707
ZNF358	0.019408	0.228374	0.463707
MED15P9	0.017937	0.22843	0.463744
SERP1	-0.09758	0.228445	0.463744
LOC28576	0.005098	0.228471	0.463744
METTL1	-0.1221	0.228494	0.463744
RTEL1	0.03603	0.228499	0.463744

LOC100288	0.076748	0.228565	0.463833
SIM2	-0.00515	0.228697	0.464016
CKAP2L	-0.26546	0.228698	0.464016
LOC102723	-0.00677	0.228868	0.464279
TMC1	0.003365	0.22887	0.464279
LPAL2	-0.00609	0.228901	0.464299
NMNAT1	0.073586	0.228939	0.464332
HRASLS2	-0.01924	0.229069	0.464553
ANKRD2	-0.05157	0.229126	0.464625
CCDC114	-0.10658	0.229248	0.464805
SUDS3	0.099333	0.229264	0.464805
ND4	0.056112	0.229279	0.464805
SSH2	0.047987	0.229324	0.464851
DERL3	0.122711	0.229373	0.464907
EFEMP2	0.098238	0.229405	0.464929
EXD1	0.008268	0.229523	0.465091
ADARB1	-0.04104	0.229528	0.465091
ELAVL3	0.005563	0.22955	0.465092
STX1A	0.025135	0.229586	0.465122
MED11	0.143051	0.229641	0.465175
FLJ25758	0.013559	0.229703	0.465175
UGT2B28	-0.06062	0.229707	0.465175
NCAPG2	-0.17807	0.229709	0.465175
LY6G6C	0.022193	0.22972	0.465175
BMP4	-0.04928	0.229833	0.465362
YDJC	0.101145	0.229868	0.465388
ATG9A	0.128523	0.22989	0.46539
SLC17A9	0.081499	0.229935	0.465436
LOC728175	0.003417	0.229982	0.465489
PKDCC	0.022303	0.230124	0.465667
ZNRD1	-0.0203	0.230126	0.465667
FAM91A1	-0.05746	0.230146	0.465667
FBXO45	-0.14955	0.230156	0.465667
TRIM51	-0.01697	0.230229	0.465735
LOC101928	0.001913	0.230233	0.465735
LOC101927	-0.03639	0.230354	0.465918
GAPDHS	0.003756	0.230366	0.465918
RUNX1-IT1	-0.19419	0.230466	0.466043
PSD3	0.262602	0.230471	0.466043
HADHB	0.054073	0.230553	0.466139
YOD1	0.103055	0.230565	0.466139
WBSCR17	0.013359	0.230583	0.466139
LIMS2	-0.03024	0.230618	0.466166
ENTPD2	0.0123	0.230863	0.466585
SYF2	-0.06589	0.230889	0.466585
RP11-748H	-0.01558	0.230915	0.466585
TTC31	0.101622	0.230951	0.466585
KRT33B	-0.01948	0.23097	0.466585
ITIH5	0.018359	0.230984	0.466585
ANXA13	-0.00895	0.230984	0.466585
VANGL1	-0.16503	0.231003	0.466585
PYHIN1	0.083657	0.231019	0.466585
C9orf152	-0.02056	0.231099	0.466655
CRYBB3	-0.04458	0.231108	0.466655
LOC100286	-0.00155	0.231159	0.466655
DOC2A	0.028853	0.231196	0.466655
RP1-31B8.1	-0.03703	0.231219	0.466655
RP11-474D	-0.01627	0.231222	0.466655

LINC01088	0.003805	0.231231	0.466655
ZNF684	-0.06279	0.231248	0.466655
ESR1	-0.00907	0.231282	0.466655
MTMR11	0.077648	0.231285	0.466655
BBS4	0.152091	0.231291	0.466655
RHBG	-0.0065	0.23139	0.466793
TOMM40	0.101783	0.23142	0.466793
ABHD15	0.215303	0.231442	0.466793
TM9SF3	0.091093	0.231475	0.466793
SRGAP2	0.219479	0.231482	0.466793
NXPH4	-0.02166	0.231489	0.466793
JSRP1	-0.06168	0.231569	0.466912
COL5A3	-0.00777	0.231679	0.467089
PRRX1	-0.00217	0.231734	0.467157
NEK6	0.098114	0.231777	0.4672
LOXL4	-0.10913	0.231845	0.467293
SRD5A1	-0.10759	0.231975	0.467462
FGL1	-0.01973	0.231978	0.467462
ART5	-0.02971	0.231993	0.467462
FRMD8P1	0.023979	0.232027	0.467486
TATDN2	-0.03925	0.232133	0.467656
ZFAND6	-0.09649	0.232178	0.467704
FAM230B	0.005039	0.232286	0.467878
TMEM225	-0.00969	0.232355	0.467935
ZFP69	-0.12007	0.232358	0.467935
HILS1	-0.02182	0.232532	0.468242
RP11-466A	-0.0101	0.232553	0.468242
RBM20	0.009562	0.232668	0.468428
LOC644135	0.003892	0.232689	0.468428
SPINT1	-0.05711	0.23273	0.468466
ZNF341	0.044676	0.232786	0.468504
PDCD4-AS	-0.14614	0.232793	0.468504
CTA-292E1	-0.14829	0.232813	0.468504
LOC101928	0.002018	0.23286	0.468529
KIF11	-0.20217	0.232881	0.468529
CTD-2130	-0.00831	0.23289	0.468529
LIN28A	-0.02493	0.232961	0.468627
PET112	0.111635	0.233026	0.468715
ELL	0.072524	0.233132	0.468846
LOC101928	0.021022	0.233171	0.468846
BDNF-AS	0.021611	0.233175	0.468846
KIF22	0.148931	0.233178	0.468846
PCDHGA4	-0.22624	0.233243	0.468891
CGREF1	0.132859	0.233253	0.468891
NKAP	-0.08307	0.233265	0.468891
PUS7L	0.100818	0.233395	0.469107
C17orf78	0.004106	0.233418	0.46911
FAM216B	0.003574	0.233489	0.469209
LACRT	-0.01501	0.233548	0.469231
LINGO3	0.038096	0.233565	0.469231
SOX17	0.005256	0.233565	0.469231
LOC101928	0.022702	0.233606	0.469256
INHBA-AS	-0.01139	0.233621	0.469256
S100A14	0.019418	0.233692	0.469339
HSPB2	0.02676	0.233705	0.469339
SLC25A4	0.05539	0.233753	0.469363
LINC00963	0.09085	0.23376	0.469363
PCNT	0.119192	0.233838	0.469427

BC040734	0.014825	0.23384	0.469427
KCNK6	0.126759	0.233879	0.469427
RP11-161D	-0.00236	0.233879	0.469427
CCDC40	0.007814	0.234181	0.469872
SKIDA1	0.058224	0.234202	0.469872
KRT8P12	0.048596	0.234211	0.469872
UBL7	0.123169	0.23423	0.469872
HIST2H2BE	-0.295	0.234251	0.469872
ABCA9	-0.01784	0.234256	0.469872
GPAA1	0.105984	0.234272	0.469872
LOC389199	-0.03895	0.234274	0.469872
RAI2	0.012365	0.234372	0.470024
NKAIN2	-0.19511	0.234466	0.470163
RTBDN	-0.00702	0.234485	0.470163
TTC25	0.130503	0.234528	0.470206
RP11-301C	-0.08256	0.234668	0.470404
MAMLD1	0.049894	0.23467	0.470404
PDYN	0.001136	0.234695	0.470412
PRPF19	-0.13839	0.234743	0.470463
CENPL	0.139782	0.234769	0.470473
GABARAPL	-0.0246	0.234822	0.470522
ANGPTL2	0.034578	0.234838	0.470522
TBC1D8B	-0.14945	0.234894	0.470591
RHOBTB1	0.225668	0.234943	0.470646
LOC101927	0.02027	0.234997	0.470711
MYH11	0.002631	0.235124	0.47089
C7orf69	-0.01783	0.235129	0.47089
LOC102724	-0.02303	0.235316	0.47122
PRDM11	-0.01151	0.235474	0.471431
LOC101929	-0.00345	0.235521	0.471431
TLX2	0.011364	0.235536	0.471431
GJC2	-0.00713	0.235593	0.471431
SLC6A1	0.033133	0.235614	0.471431
GTF2A2	-0.10605	0.235614	0.471431
RP11-804H	-0.13858	0.235618	0.471431
RCAN1	0.047703	0.235623	0.471431
DHRS1	0.133219	0.235634	0.471431
BC012193	0.054015	0.235656	0.471431
ORF1	-0.02864	0.235672	0.471431
RP11-503C	-0.03159	0.235693	0.471431
CD27	0.136082	0.235704	0.471431
C12orf56	0.001363	0.235764	0.471506
DNAJC5B	0.06664	0.235824	0.471564
NXN	0.209491	0.235837	0.471564
B3GALTL	0.101891	0.235864	0.471576
FIBIN	-0.00206	0.235975	0.471754
RNF183	0.020797	0.23612	0.472001
RP11-714L	-0.01303	0.236157	0.472032
KDELC2	0.132733	0.236195	0.472062
PLOD2	0.278085	0.236237	0.472078
RDH13	-0.12623	0.236253	0.472078
PROCR	0.139244	0.236288	0.472078
TECTA	-0.02892	0.236311	0.472078
PON1	-0.00598	0.236311	0.472078
RP11-324L	-0.00948	0.236337	0.472085
AREG	0.344755	0.23645	0.472267
GSK3A	0.037214	0.236476	0.472275
ADRA1D	0.008458	0.236515	0.47231

PRSS3P3	-0.0114	0.23659	0.472416
CCDC158	0.002704	0.236621	0.472435
BST2	-0.23462	0.236723	0.472576
METTL21EI	-0.01018	0.236753	0.472576
FLJ37035	-0.01251	0.236757	0.472576
C16orf59	-0.15623	0.236793	0.472604
EPPK1	-0.00549	0.236941	0.472856
SEPSECS	0.121669	0.237061	0.473052
LOC10192	-0.00409	0.237148	0.473118
DTD1	-0.07879	0.237159	0.473118
LOC10192	-0.01848	0.237166	0.473118
HSPB9	0.031302	0.237182	0.473118
MIR4755	0.036979	0.237218	0.473147
RP11-338I	-0.01376	0.237244	0.473155
KRTAP3-2	0.01494	0.237286	0.473196
UBAC2-AS	-0.05582	0.237418	0.473414
LOC72807	-0.0615	0.237503	0.473527
HTRA2	0.099601	0.237518	0.473527
ICAM4	0.225932	0.237594	0.473581
LOC10192	0.009648	0.237594	0.473581
GFOD2	0.066722	0.237633	0.473581
COLEC10	0.006837	0.237638	0.473581
YBX2	-0.04399	0.237686	0.473581
EMG1	-0.11621	0.237689	0.473581
CCDC37	0.017129	0.237698	0.473581
DDX26B	-0.12116	0.237734	0.47361
MOB1B	-0.1398	0.237818	0.473731
UNKL	0.07676	0.23784	0.473731
EZH2	-0.13864	0.237863	0.473731
LOC10192	-0.00675	0.237883	0.473731
RP5-991O	-0.00355	0.237976	0.473873
POU5F1P3	-0.01263	0.23813	0.474074
RP11-669I	-0.00396	0.238154	0.474074
LINC00561	0.001349	0.238161	0.474074
TTTY7	-0.00435	0.238164	0.474074
ZNF182	-0.09378	0.238248	0.474145
LOC10192	0.023999	0.238263	0.474145
GLRA2	-0.0132	0.238266	0.474145
GLMN	-0.09214	0.238342	0.474253
NNMT	0.013345	0.238478	0.474481
CAV1	0.257668	0.238532	0.474544
CHRNA10	0.001961	0.238562	0.474559
MTUS2-AS	-0.02997	0.238632	0.474622
CXorf23	0.118062	0.238637	0.474622
QRFPR	0.003766	0.238728	0.474726
SETD9	-0.07033	0.238733	0.474726
TGS1	-0.09495	0.238807	0.474775
NRDE2	0.052448	0.238821	0.474775
RP11-486	-0.15903	0.238824	0.474775
LOC10012	-0.01095	0.238847	0.474778
LOC28366	-0.02403	0.238883	0.474805
RBM4	-0.19669	0.238906	0.474808
PRIM2	0.139693	0.238956	0.474864
ANKRD30I	0.011267	0.239048	0.475003
C2orf88	0.274988	0.239101	0.475027
MANBA	-0.09915	0.239104	0.475027
UPB1	0.022984	0.239304	0.475365
LMNTD1	0.010485	0.239318	0.475365

GJA1	0.500489	0.239419	0.475522
UPK3B	-0.01721	0.239456	0.475551
MDGA2	-0.04053	0.239484	0.475563
SNHG18	-0.01948	0.239507	0.475566
CFTR	-0.00278	0.239529	0.475567
LOC10013:	-0.11569	0.239557	0.475568
LINC00658	0.005824	0.239574	0.475568
LOC10192!	-0.01179	0.239604	0.475572
LOC10192(	0.003556	0.239664	0.475572
TPM4	0.185588	0.239671	0.475572
LOC10192'	0.001236	0.239677	0.475572
TMC8	0.126601	0.239707	0.475572
RP11-171I:	-0.09388	0.23974	0.475572
HAPLN2	-0.01475	0.23974	0.475572
ARHGEF16	-0.0195	0.239752	0.475572
C9orf163	-0.02026	0.239807	0.475638
GZF1	-0.10807	0.23987	0.47572
RAD54L	-0.15098	0.239929	0.475794
ADH6	-0.01179	0.240055	0.475983
DISP2	0.014968	0.240111	0.475983
ZSCAN5A	-0.08458	0.240122	0.475983
HOXB13	-0.01258	0.240127	0.475983
TRIO	0.067112	0.240144	0.475983
JAM2	-0.19906	0.240156	0.475983
RNF175	0.356112	0.240276	0.476176
GAPDH	0.061189	0.240342	0.476235
PRRT2	-0.02844	0.240349	0.476235
RAD51C	0.118926	0.24041	0.476312
LOC10192'	-0.02508	0.240435	0.476317
GRIN1	-0.00642	0.240544	0.476482
NLRP6	0.001206	0.240562	0.476482
RP11-554I	-0.02729	0.240656	0.476608
EMILIN2	0.103285	0.24067	0.476608
CABP2	-0.01348	0.240692	0.476608
RP11-399E	-0.00687	0.240746	0.476671
KIN	-0.12762	0.240808	0.47675
TMEM217	0.152118	0.240882	0.476854
CLDN16	0.008369	0.24099	0.477023
SUPT16H	-0.11729	0.241069	0.477137
COMMD3	-0.08395	0.241147	0.477188
CTAGE11P	-0.01843	0.24117	0.477188
ZNF672	-0.00451	0.241178	0.477188
EGFR	0.012061	0.2412	0.477188
OPRD1	-0.02964	0.241205	0.477188
TRHDE-AS	0.095913	0.241462	0.477652
RP11-278J	0.01357	0.24165	0.477948
IKBKE	-0.05492	0.241656	0.477948
CNTNAP2	-0.02607	0.241694	0.477979
LOXL2	-0.00146	0.241724	0.477996
WNT2	0.031812	0.241751	0.478006
CLUHP3	0.091412	0.241783	0.478024
MUTYH	-0.0984	0.24195	0.478311
PRDM7	-0.02153	0.241996	0.47836
ERBB2IP	0.114757	0.242047	0.478416
UPP2	-0.00429	0.242094	0.478465
LOC10192!	0.006714	0.242363	0.478821
WASF3	0.320322	0.242368	0.478821
LOC28385(	0.001562	0.242411	0.478821

RP11-676J	0.003219	0.242429	0.478821
RP11-524C	-0.0535	0.242429	0.478821
LHCGR	0.001719	0.242441	0.478821
CST13P	0.007983	0.242442	0.478821
SIRT7	0.091202	0.242451	0.478821
LOC10192	-0.00849	0.242534	0.478941
SNRPA	0.157805	0.242624	0.479076
RP11-201A	-0.01375	0.242767	0.479314
INPP4B	-0.1843	0.242819	0.479372
OLFML1	-0.00297	0.242856	0.479403
LIMD1-AS1	0.056684	0.242893	0.479433
GRIK1	0.051854	0.24296	0.479511
LOC10192	0.001946	0.242977	0.479511
FXN	0.098444	0.243051	0.479546
SH3TC2	0.007048	0.243055	0.479546
EFCAB11	0.076208	0.243061	0.479546
PDE1C	-0.00942	0.243125	0.479628
SLC45A2	-0.00376	0.24326	0.47981
CYP26A1	0.022263	0.243262	0.47981
SULT1B1	0.142418	0.2435	0.480236
LRIG3	0.023453	0.243577	0.480344
NR2E1	-0.00693	0.243778	0.480683
GOLGA3	0.086531	0.243793	0.480683
LOC10192	0.003542	0.243918	0.480866
ALPL	0.033289	0.243931	0.480866
ACTA1	0.009358	0.243988	0.480937
MPP6	-0.14015	0.244047	0.481008
RP11-783K	-0.05933	0.244097	0.481062
RP11-353N	0.007964	0.244474	0.481757
TCF19	0.152629	0.244498	0.481757
EP400NL	-0.0853	0.244522	0.481757
IGHG1	0.002715	0.244538	0.481757
HP09025	0.020437	0.244583	0.481801
SPTY2D1-A	-0.02525	0.244627	0.481832
TRAV12-2	0.023215	0.244644	0.481832
LOC10192	-0.01061	0.244665	0.481832
SCG5	-0.07378	0.24473	0.481916
ERMN	-0.16643	0.244788	0.481962
MACROD1	0.079366	0.244798	0.481962
SFTA2	-0.01804	0.244835	0.481992
NOBOX	-0.00926	0.244935	0.482144
ETV3	0.21248	0.245027	0.482199
MAST1	-0.00349	0.245027	0.482199
ZCCHC7	-0.16918	0.24503	0.482199
HOXC11	-0.03458	0.245201	0.482491
PAGR1	-0.08213	0.245239	0.482523
MINOS1P1	0.219783	0.245339	0.482676
GGTLC2	-0.04251	0.245394	0.482737
LOC10192	0.010564	0.245415	0.482737
CYLC1	-0.0037	0.245513	0.482887
PPP2R5D	0.056869	0.245616	0.483046
C3orf65	0.001357	0.245845	0.483453
MYRFL	-0.05556	0.24593	0.483575
NKX2-1-A	0.005501	0.246069	0.483785
PRDM9	-0.0151	0.246081	0.483785
MT4	-0.00242	0.246215	0.484004
PDE1B	-0.01151	0.246257	0.484042
SERPINE2	0.145776	0.246382	0.484244

TUSC5	-0.0441	0.246418	0.484262
CDK5	0.142016	0.246436	0.484262
LOC10028	0.001404	0.246759	0.484823
ANKRD32	0.07794	0.246784	0.484823
LINC00202	-0.05804	0.246788	0.484823
LOC10013	-0.1651	0.246904	0.484883
B3GNT9	0.04278	0.246909	0.484883
ZNF521	-0.23005	0.246916	0.484883
GADD45G	0.139462	0.246926	0.484883
LINC00421	-0.00514	0.246931	0.484883
TRIM54	0.00879	0.24703	0.485033
COQ9	0.065282	0.247057	0.485042
EHD3	0.211576	0.247092	0.485067
SUPT7L	0.098653	0.247135	0.485108
EXTL2	-0.10138	0.247238	0.485215
ITGB3	-0.18477	0.247246	0.485215
CYP7B1	-0.18955	0.247257	0.485215
LINC00545	0.003291	0.247453	0.485556
LOC10192	-0.01137	0.247629	0.485823
KIF3C	0.095994	0.247634	0.485823
KLK10	0.003634	0.247671	0.485852
ZDHHC5	0.062143	0.247762	0.485987
CACNG3	0.015852	0.248039	0.486485
NRF1	0.092624	0.248097	0.486555
TMEM132	-0.0579	0.248156	0.486602
GFAP	0.001228	0.248166	0.486602
RSPH6A	0.016703	0.248281	0.486783
TRIM72	-0.03435	0.24846	0.487051
ZNHIT2	0.011005	0.248484	0.487051
COX15	-0.10688	0.248489	0.487051
VIT	-0.01121	0.248516	0.487051
HMGN3-A	0.027107	0.24853	0.487051
BC021061	-0.00153	0.248605	0.487154
PDGFRA	-0.03376	0.24891	0.487638
ESR2	0.020048	0.248921	0.487638
STAR	-0.24401	0.248987	0.487638
TAS2R19	-0.0142	0.248996	0.487638
TAF10	-0.0404	0.249	0.487638
SH2B2	-0.08832	0.249006	0.487638
L1TD1	-0.0321	0.249009	0.487638
ZNF260	-0.11242	0.249114	0.487799
OR7E24	-0.00584	0.249371	0.488222
LOC10192	-0.0222	0.249396	0.488222
CLECL1	0.141674	0.249398	0.488222
TAAR1	0.001616	0.249554	0.488483
APLP1	-0.01623	0.249766	0.488822
CBR3	0.149461	0.249785	0.488822
PRR26	0.002137	0.249799	0.488822
ATXN8OS	0.002252	0.249817	0.488822
PROM2	0.036998	0.25001	0.489155
LOC10050	-0.00185	0.250082	0.489233
WASF1	-0.2048	0.250113	0.489233
ZNF709	-0.0545	0.250117	0.489233
TGFA	0.019703	0.250175	0.489302
XIAP	0.145996	0.250211	0.489327
TXNDC11	0.10807	0.250255	0.489331
SH3D19	0.120308	0.250258	0.489331
RAB11FIP2	0.121749	0.250283	0.489336

GABRG1	0.116064	0.250341	0.489404
LOC10050!	-0.01218	0.250443	0.489559
LOC10272	-0.00773	0.250544	0.489713
SLC22A14	0.035457	0.25058	0.489739
NDUFAF2	0.117323	0.250659	0.489849
EIF2B3	0.139173	0.250721	0.489926
TMEM52	-0.03434	0.250768	0.489974
CARD8	-0.09005	0.250832	0.490026
RP1	0.018314	0.250852	0.490026
SLC34A2	-0.0145	0.250863	0.490026
TPGS1	0.055784	0.250946	0.490068
WDR16	-0.00995	0.250968	0.490068
FAM153A	-0.0162	0.250983	0.490068
DUSP5P1	-0.06699	0.250989	0.490068
LINC01255	-0.02958	0.251029	0.490068
ZNF706	0.069303	0.251041	0.490068
NECAP1	0.102242	0.251045	0.490068
TGM7	0.014422	0.251085	0.490068
MYPN	-0.02095	0.251088	0.490068
RAX	-0.01155	0.251227	0.490294
RPS10L	0.127801	0.251254	0.490294
PFKFB1	0.002808	0.251271	0.490294
AP4B1	-0.07371	0.251321	0.490346
PNLIPRP2	0.003097	0.251376	0.490406
PRR32	-0.02345	0.251397	0.490406
MAGI1-IT1	0.004501	0.25145	0.490466
LOC10192	0.002982	0.251527	0.490571
LOC10192	0.004912	0.251561	0.490594
B4GALT2	-0.10561	0.251702	0.490804
PPEF2	0.00618	0.251714	0.490804
C19orf40	0.056137	0.251738	0.490807
DCTD	0.087188	0.251803	0.490889
LINC00483	0.013919	0.251943	0.491117
SPACA7	0.005335	0.252084	0.491347
LOC39988	0.016652	0.252136	0.491364
PEX7	-0.10974	0.252149	0.491364
KCNE2	-0.01234	0.25216	0.491364
APOM	0.104193	0.252199	0.491384
LOC10012	0.003079	0.252216	0.491384
CDHR2	0.02853	0.25229	0.491415
RP11-195M	-0.00837	0.252305	0.491415
KHDC1	-0.09143	0.252308	0.491415
HAGHL	-0.11293	0.252336	0.491415
MAP2	-0.02655	0.252345	0.491415
MYH15	-0.00277	0.252381	0.491441
ZPR1	-0.10167	0.252406	0.491446
HCG27	-0.09124	0.2525	0.491513
THNSL1	-0.16704	0.252524	0.491513
C21orf62	0.012964	0.252537	0.491513
RPL12	-0.05772	0.25254	0.491513
CSK	0.092235	0.252554	0.491513
PYGM	0.011268	0.252592	0.491541
E2F8	-0.2212	0.252692	0.49167
PP2D1	-0.0121	0.252719	0.49167
C1orf167	0.016785	0.252726	0.49167
LOC69224	-0.01565	0.25278	0.491732
LOC10192	-0.01226	0.252902	0.491925
RPLP1	0.020099	0.252927	0.491929

MAGEB18	0.004976	0.252978	0.491984
SEL1L2	-0.003	0.253019	0.492019
GTSF1L	-0.03271	0.253049	0.492034
ZNF185	0.243523	0.253142	0.49217
LMNTD2	0.060895	0.253181	0.492202
MOGAT3	-0.01224	0.253246	0.492212
LOC10012ₜ	-0.03638	0.253253	0.492212
LINC01012	-0.00808	0.253254	0.492212
LOC10192ₜ	0.00977	0.253331	0.492316
MOSPD3	0.083837	0.253408	0.492417
RD3	0.002859	0.253428	0.492417
FMN1	0.003184	0.253485	0.492441
LINC00615	-0.00971	0.253486	0.492441
LINC01121	-0.03081	0.253744	0.492898
LOC10099ₜ	-0.00957	0.253775	0.492913
TOB1	0.196087	0.253802	0.492922
ISCA1	-0.1188	0.253875	0.493019
EBLN3	-0.13643	0.25394	0.493101
SCN1A	0.004572	0.254094	0.493356
HFM1	-0.06751	0.25419	0.493487
WDR44	-0.043	0.254217	0.493487
ASB16	-0.00289	0.25423	0.493487
DNAI2	-0.01401	0.254292	0.493563
RP11-389C	0.011113	0.254329	0.493563
SFRP2	0.002507	0.254337	0.493563
HAS1	0.188843	0.254367	0.493577
DNALI1	0.006308	0.254507	0.493719
POLR3H	0.08807	0.254507	0.493719
LHX3	0.029864	0.254509	0.493719
SLC35F6	-0.03538	0.254557	0.493733
TRPC7	-0.002	0.254562	0.493733
ZNF775	0.011959	0.254597	0.493758
CTSK	-0.16501	0.254626	0.49377
LOC14679ₜ	-0.00611	0.25467	0.493789
ZSCAN18	-0.16741	0.254681	0.493789
RP13-30Aₜ	-0.01307	0.254734	0.493813
LOC72811ₜ	-0.0186	0.25474	0.493813
LOC15257ₜ	-0.00314	0.254799	0.493885
FAM168B	0.036819	0.254861	0.49396
EPS15L1	0.08119	0.255024	0.494231
KCNC4	0.024491	0.255085	0.494306
MIRLET7D	-0.18814	0.25513	0.494329
RP5-856G1	-0.00289	0.255148	0.494329
ELAVL4	0.060158	0.255165	0.494329
RP11-740C	-0.00423	0.2552	0.494352
PKN3	0.135968	0.255265	0.494397
PPFIBP1	-0.19475	0.255269	0.494397
AQP6	-0.01775	0.255313	0.494435
GPR17	0.001527	0.255334	0.494435
TP53BP2	-0.12351	0.255392	0.494502
PDZRN4	-0.28981	0.255551	0.494728
MAMSTR	-0.03244	0.255554	0.494728
RP11-66N1	-0.00756	0.255743	0.495049
CCDC93	0.065489	0.255804	0.495124
ANKAR	-0.01344	0.255876	0.495218
BARHL1	-0.00727	0.255997	0.495409
PAX3	-0.00276	0.256021	0.495409
RSU1P2	-0.01383	0.256114	0.495528

ACADVL	0.102481	0.256128	0.495528
KIAA1751	0.006388	0.256263	0.49569
LOC78052!	-0.02389	0.256273	0.49569
DNER	-0.00397	0.256287	0.49569
LOC10192!	-0.02445	0.256325	0.49569
MAGEA10	-0.00366	0.256326	0.49569
KBTBD7	-0.17626	0.256356	0.495705
LOC10192	0.001732	0.256406	0.495756
C1orf100	-0.0107	0.256473	0.495831
LOC10192!	0.11401	0.25649	0.495831
KIAA1324L	-0.17484	0.256581	0.495962
DMPK	-0.01531	0.256612	0.495978
CTD-2619	-0.0483	0.256745	0.496191
AFF3	0.021969	0.256789	0.496231
LRRC3	-0.00748	0.256855	0.496261
LOC10028!	0.021021	0.256876	0.496261
PPL	0.019334	0.256885	0.496261
LMOD3	-0.00441	0.256896	0.496261
HSD17B1	0.049441	0.257139	0.496687
LOC10192!	0.019864	0.257202	0.496764
LOC10050	-0.01654	0.257231	0.496775
PHF10	0.086703	0.257259	0.496779
LOC10272	-0.01961	0.257279	0.496779
TRA2A	0.140332	0.257345	0.496852
FAM58A	0.070177	0.257363	0.496852
P2RY10	0.075198	0.257389	0.496858
TMEM26	0.022214	0.25744	0.4969
ITM2B	0.119469	0.257457	0.4969
STARD8	0.142748	0.257564	0.497064
RP11-796E	-0.07002	0.257634	0.497154
PTPRR	-0.00341	0.257692	0.497177
SLC22A11	-0.01258	0.257694	0.497177
PCDH19	-0.00851	0.257729	0.497177
C7orf26	0.116029	0.257738	0.497177
CCDC101	-0.10343	0.257936	0.497504
PRKAG3	-0.02106	0.257969	0.497504
GSTA3	-0.01789	0.257982	0.497504
RILP	0.117308	0.257999	0.497504
CECR9	-0.01106	0.258117	0.497654
GUCA2B	-0.00228	0.258123	0.497654
RAP2A	0.03195	0.258171	0.497671
THRB	0.304271	0.258178	0.497671
KCNJ13	-0.00664	0.258329	0.497918
OR8D2	0.021789	0.258396	0.498004
ADAMTS8	0.006923	0.258495	0.498123
ACSM2A	-0.00929	0.258504	0.498123
RP5-1092A	-0.25584	0.258536	0.498124
PLG	0.00199	0.258551	0.498124
LOC10099	-0.00224	0.258596	0.498166
AKR1C4	-0.02961	0.258692	0.498267
ZNF565	0.120925	0.258694	0.498267
ZCCHC13	-0.00342	0.258729	0.49829
HIGD1A	-0.12768	0.258833	0.498445
KCNJ3	-0.00268	0.258878	0.498488
ZNF630	0.071991	0.258901	0.498489
RP11-141M	-0.00208	0.258981	0.498599
MTL5	-0.06888	0.259098	0.498745
ATXN2L	-0.07543	0.259103	0.498745

CLIP1	0.110324	0.259167	0.498798
MST1	0.119143	0.25919	0.498798
KRTAP3-3	-0.00308	0.2592	0.498798
RP11-560/	-0.0113	0.259234	0.498818
RBP5	-0.00897	0.259283	0.498868
B3GAT1	-0.00191	0.259346	0.498947
HARBI1	0.05685	0.259418	0.49904
EDN3	-0.00481	0.259493	0.499139
TP53RK	0.149079	0.259548	0.499202
SEMA5A	0.006997	0.259599	0.499256
KCNC1	-0.01725	0.259635	0.49928
BC043540	-0.01752	0.259662	0.499288
FGF3	0.001156	0.259768	0.499448
PNP	0.156993	0.259888	0.499634
RP11-680F	0.004912	0.25994	0.49969
NUDT21	0.116242	0.260149	0.500028
DHX57	0.067778	0.260162	0.500028
LINC00669	0.089217	0.260265	0.50017
LRRC72	-0.01536	0.260282	0.50017
RPAIN	0.05991	0.260331	0.500209
TMX1	0.102716	0.260349	0.500209
TDRD1	-0.02273	0.260497	0.500421
RGS9	0.009302	0.260513	0.500421
NACA	-0.05156	0.260573	0.500421
MFSD9	-0.05806	0.260577	0.500421
GUF1	0.11793	0.260578	0.500421
EFNA4	0.067039	0.260598	0.500421
TMPRSS12	0.002337	0.260662	0.500468
ZNF22	-0.07735	0.260687	0.500468
ATP5E	0.085631	0.260692	0.500468
ETV2	-0.03922	0.260737	0.500499
IRF3	-0.10358	0.260754	0.500499
CCDC186	0.154711	0.260787	0.500518
MROH2B	0.001815	0.260828	0.500553
RBP3	-0.01811	0.260907	0.500637
PPP4R4	0.002204	0.260919	0.500637
NDST4	-0.06458	0.261074	0.500802
SMAGP	0.176459	0.261081	0.500802
SLC35E1	0.079147	0.261087	0.500802
N6AMT1	-0.03489	0.261097	0.500802
LOC10193(	-0.05372	0.261263	0.501077
C1orf234	-0.00945	0.26138	0.501215
USP9Y	0.414892	0.261382	0.501215
AGA	-0.13901	0.26147	0.50134
THOC1	0.087276	0.26151	0.501372
FAM3A	0.082436	0.261594	0.501488
LOC10106(	-0.01452	0.261656	0.501564
CDC42SE2	0.108784	0.261774	0.501744
ZNF252P	0.057732	0.261823	0.501794
SPTLC2	0.115595	0.261865	0.501826
ITGA1	0.009394	0.261907	0.501826
DCAKD	0.059089	0.261914	0.501826
LRRC4C	0.053822	0.261945	0.501826
FLJ13773	-0.09857	0.261959	0.501826
CENPE	0.248382	0.261986	0.501826
LINC00607	-0.00295	0.262002	0.501826
AR	-0.19921	0.262031	0.501838
AIMP1	-0.08797	0.262104	0.501871

LRRC16B	-0.06336	0.26214	0.501871
LAIR2	-0.09451	0.262146	0.501871
CDC14C	-0.00179	0.26215	0.501871
DLAT	0.142184	0.262164	0.501871
COX6B2	0.001181	0.262221	0.501877
PMF1	-0.10497	0.262224	0.501877
RIMS2	-0.01251	0.262256	0.501877
HIVEP3	0.115777	0.26226	0.501877
PTPRF	-0.01486	0.262303	0.501915
LINC01330	0.00467	0.262343	0.501932
RP11-1277	-0.06904	0.262358	0.501932
SGCB	0.097176	0.262405	0.501977
LIX1L	-0.0388	0.262489	0.50206
SOHLH2	0.005491	0.262495	0.50206
RP11-292D	-0.00284	0.262519	0.502062
CLEC4F	-0.00264	0.262652	0.502221
LOC100507	0.02225	0.262656	0.502221
RP11-83N9	-0.02709	0.262675	0.502221
KRT17P5	-0.00351	0.262695	0.502221
ATP13A4-A	-0.00467	0.262779	0.502336
NEFM	-0.00278	0.262801	0.502336
CTD-25540	-0.07033	0.262927	0.502425
LOC101929	0.020784	0.262938	0.502425
LINC00423	0.026582	0.262938	0.502425
ZBTB17	-0.05402	0.262946	0.502425
TMEM87A	0.067822	0.262964	0.502425
NUP210P1	-0.03487	0.262995	0.502439
PPP2R2D	-0.08246	0.263046	0.502475
SLC22A15	-0.23571	0.263063	0.502475
KRT24	-0.03345	0.263083	0.502475
MECOM	-0.11238	0.263186	0.502562
SKA3	-0.17223	0.26319	0.502562
SCGB2A2	-0.01375	0.263198	0.502562
ERI3	0.135892	0.263275	0.502629
LOC100129	-0.01034	0.26328	0.502629
ATP5G1	-0.10976	0.263324	0.502669
TNC	0.01207	0.263387	0.502716
NOS1	0.002075	0.263418	0.502716
PIGM	-0.13398	0.263433	0.502716
CKS1B	0.120508	0.263441	0.502716
LOC283177	-0.01539	0.263473	0.502732
STK25	0.105301	0.263525	0.502787
LINC01139	0.011346	0.263571	0.50283
LOC101928	0.007611	0.263634	0.502907
DMRTA2	-0.0111	0.263688	0.502965
HLX	0.260631	0.26372	0.502982
ANKRD55	0.105454	0.263849	0.503183
PAX9	0.005703	0.263991	0.503409
KIAA1683	0.069515	0.264088	0.503551
CNDP2	0.101173	0.264114	0.503556
SPSB3	-0.07329	0.264163	0.503605
ASIP	-0.00272	0.264247	0.503722
EPHB2	0.015546	0.264347	0.503842
DPYD	0.077206	0.264357	0.503842
CRYGN	-0.00709	0.264407	0.503859
NCLN	0.07599	0.264437	0.503859
SEPT5-GP1	-0.04899	0.264459	0.503859
LRRC27	0.031958	0.264472	0.503859

SULF1	0.014011	0.264482	0.503859
NEB	-0.04066	0.264703	0.504212
LYSMD1	0.030572	0.264714	0.504212
PRM1	-0.00661	0.264812	0.504354
AK131021	-0.0094	0.264918	0.504511
F11-AS1	-0.02138	0.265008	0.504549
GS1-164F2	-0.05033	0.26502	0.504549
ARL8B	0.069555	0.265042	0.504549
C8orf59	0.120218	0.265045	0.504549
CDYL2	-0.07206	0.265054	0.504549
EML1	0.014088	0.265406	0.505174
PPP1R26	0.158286	0.265658	0.50561
ITGA8	0.022885	0.265745	0.50573
IARS	0.067637	0.265785	0.505744
APPL1	0.110158	0.265827	0.505744
RPH3A	0.044526	0.265835	0.505744
SERPINA2	-0.00152	0.265845	0.505744
GARNL3	-0.01511	0.265944	0.505887
CCSER2	0.062749	0.26599	0.50593
PEX3	-0.1009	0.266083	0.505983
RP11-153K	0.004404	0.266084	0.505983
ZNF853	-0.04131	0.266088	0.505983
ZNF710	-0.06547	0.266125	0.506009
RP11-215E	-0.0286	0.266172	0.506054
LOC10192	-0.02745	0.266245	0.506148
PRO2852	-0.20268	0.266345	0.506294
LINC01018	-0.00896	0.266649	0.506818
ANXA2P3	-0.0203	0.266668	0.506818
LOC40068	-0.02784	0.266717	0.506868
TTC39A	0.00547	0.266746	0.506879
LOC10192	0.011346	0.266885	0.507096
LOC10192	-0.05368	0.266955	0.507096
CDC42BPA	-0.18431	0.266957	0.507096
LINC00565	0.014143	0.266965	0.507096
BFAR	0.08388	0.267019	0.507096
PRAME	-0.19454	0.26703	0.507096
ROM1	0.061092	0.267044	0.507096
FLJ37786	0.009923	0.26707	0.507096
MGC2889	-0.01056	0.267071	0.507096
LOC10050	-0.00303	0.267149	0.507198
GTDC1	0.050861	0.267292	0.507426
LOC14584	-0.01873	0.267328	0.50743
MUC4	-0.01004	0.267341	0.50743
GSX2	-0.00392	0.267397	0.507492
CTA-246H	-0.09872	0.267483	0.507611
TMEM174	0.017047	0.267558	0.507708
OTOP2	-0.01048	0.267647	0.507833
ZNF564	-0.11031	0.267851	0.508175
HJURP	0.176633	0.267899	0.508204
STX17	-0.09319	0.267913	0.508204
SIK3	0.117296	0.268242	0.508783
GINS2	0.029332	0.268276	0.508804
NFYB	0.106969	0.268382	0.508959
LGI2	0.05516	0.268405	0.508959
MSANTD1	-0.0054	0.268476	0.509049
LINC00319	0.006033	0.268579	0.509199
MVK	-0.04172	0.268761	0.5095
SMG6	0.024507	0.268887	0.509624

ZNF610	-0.1355	0.268905	0.509624
LINC01425	0.002098	0.268908	0.509624
TRIM46	-0.03344	0.268921	0.509624
UBL7-AS1	0.085528	0.269176	0.510062
LINC00664	-0.0299	0.269204	0.510062
ATPAF2	0.075964	0.269222	0.510062
GKAP1	0.142314	0.269262	0.510092
FGD5	-0.12903	0.269363	0.510179
SPOP	-0.11782	0.26937	0.510179
TBX1	-0.01275	0.269378	0.510179
KCNH6	0.004224	0.269484	0.510335
SERPINA5	0.050765	0.269641	0.510587
MPPED2	0.235774	0.269693	0.510597
PRPSAP1	0.032218	0.269693	0.510597
LOC10192	0.001374	0.269815	0.510783
COL1A1	0.057148	0.26989	0.51088
CBY3	-0.00381	0.269939	0.510929
C6orf211	-0.16875	0.270011	0.510986
LOC10192	-0.03309	0.27005	0.510986
PRSS3P2	-0.16691	0.270072	0.510986
ARL5B	-0.15984	0.270104	0.510986
RP11-52A	0.003302	0.270118	0.510986
CGA	-0.00637	0.270128	0.510986
SEMA7A	0.047078	0.270134	0.510986
DAOA	-0.01004	0.270161	0.510992
EDN2	-0.00639	0.270295	0.511201
HAT1	-0.07671	0.270362	0.511282
RP3-388M	0.00306	0.270468	0.511423
TACR3	0.014883	0.270484	0.511423
CISD2	-0.18914	0.270513	0.511434
DUS4L	-0.10308	0.270586	0.511527
CTRB2	-0.02477	0.270706	0.511709
SYNM	-0.19408	0.270771	0.511787
RNF5	-0.02452	0.270796	0.511787
OSBPL5	0.113557	0.270818	0.511787
ABCD4	0.038045	0.270854	0.511801
PRG3	0.127108	0.270876	0.511801
WDR93	-0.02239	0.270896	0.511801
MZB1	0.275719	0.271067	0.51202
FLJ33534	-0.03019	0.271074	0.51202
KCNA4	0.014168	0.271083	0.51202
MIR4313	-0.00919	0.271149	0.512068
ITM2C	-0.15162	0.271176	0.512068
RPS17	-0.03954	0.27118	0.512068
RP3-496C	-0.0257	0.271299	0.512213
CEP135	-0.12639	0.271322	0.512213
TRIM7	-0.06112	0.271329	0.512213
USH1C	0.038111	0.271351	0.512213
CACTIN	0.059706	0.271448	0.512351
NXPE3	0.098064	0.271475	0.512353
CYP2A7P1	-0.00764	0.271519	0.512353
TTL	0.060215	0.271564	0.512353
ALX3	-0.01685	0.271564	0.512353
COL13A1	-0.02845	0.271567	0.512353
ICK	-0.05303	0.271621	0.512409
PRRT3-AS	-0.14809	0.271795	0.512693
TIPIN	-0.13067	0.27184	0.512706
ACSM3	-0.22569	0.271849	0.512706

LOC10099(	0.01261	0.271939	0.512831
ADCY10	-0.00922	0.272061	0.513016
WNT9B	-0.0038	0.272087	0.513021
POLB	-0.1025	0.272191	0.513172
AXIN2	0.00174	0.272359	0.513444
GGA1	-0.09455	0.272444	0.513532
LOC10192!	-0.00779	0.272467	0.513532
SLC30A6	-0.11241	0.272477	0.513532
TBX21	-0.02147	0.272516	0.513561
SLC38A11	-0.00236	0.272582	0.513641
GALR3	-0.00319	0.272665	0.513735
SPTBN1	-0.01054	0.272679	0.513735
ENKUR	-0.08357	0.272798	0.513875
LOC10106(	0.061081	0.272818	0.513875
SEZ6L	-0.00456	0.272847	0.513875
SLC22A2	-0.00433	0.272849	0.513875
RPN2	0.075045	0.272872	0.513875
NEURL1	0.015779	0.27292	0.51392
MNT	-0.03351	0.272949	0.513927
CAB39L	0.136877	0.272971	0.513927
GTSF1	-0.48499	0.273016	0.513933
ELAC2	0.050714	0.273022	0.513933
NBPF3	0.09146	0.273104	0.514023
ANKRD37	0.066978	0.273117	0.514023
IRX5	0.058829	0.273209	0.514139
PKD1L1	0.00379	0.273226	0.514139
LOC14651:	0.020928	0.273337	0.514302
GPR113	0.029266	0.273362	0.514306
FAM99B	-0.05116	0.273433	0.514354
WWTR1	0.055498	0.273435	0.514354
ANXA2P1	-0.04754	0.273502	0.514434
GPATCH2	0.119408	0.273661	0.514606
POLE	0.136003	0.273663	0.514606
ACTRT2	-0.01267	0.273664	0.514606
RP4-593C1	0.004055	0.273755	0.514712
LOC10050	0.028211	0.273768	0.514712
AMBN	-0.00417	0.273996	0.515065
MRGPRX1	0.006194	0.274003	0.515065
STK4-AS1	-0.04053	0.274028	0.515065
TNNI3K	0.097483	0.274131	0.515214
DNAL1	-0.04554	0.274249	0.515344
AF131215.!	-0.09525	0.274262	0.515344
MAP4	0.074394	0.274292	0.515344
RPL34-AS1	0.011381	0.274295	0.515344
RPL3L	-0.02765	0.274337	0.515379
ANKRD34C	-0.00279	0.274368	0.515392
MMP24	-0.00738	0.274399	0.515406
DCAF4L1	0.028128	0.274503	0.515547
MYO3A	0.002238	0.274522	0.515547
HOXD9	-0.01829	0.274685	0.515766
SLC35F5	-0.14953	0.274686	0.515766
CDHR3	-0.00267	0.274743	0.515828
PSG7	0.001632	0.274785	0.515833
LOC10192!	-0.05902	0.274793	0.515833
GATA3-AS	-0.00323	0.274879	0.515949
FCRLA	0.065052	0.274927	0.515956
RNF128	-0.00694	0.274931	0.515956
CPED1	-0.13602	0.275016	0.516039

LOC10099(	0.005092	0.275045	0.516039
PCLO	-0.0222	0.275054	0.516039
RASGRF1	0.00161	0.27507	0.516039
FLG2	0.021035	0.275163	0.516147
C7orf71	-0.00572	0.275176	0.516147
RASGEF1B	0.176347	0.275199	0.516147
PLEKHG1	0.187898	0.275379	0.51644
ERICH3	-0.01807	0.275411	0.516456
LOC28495(	0.004499	0.275578	0.516724
SULT1E1	0.005963	0.275621	0.516759
LOC10050(	-0.00223	0.275749	0.516828
LOC10049!	-0.0458	0.275765	0.516828
SHMT1	0.022251	0.275768	0.516828
PWAR5	-0.05622	0.275784	0.516828
LOC10192!	-0.00717	0.275786	0.516828
MYL12A	0.128147	0.275803	0.516828
EN2	-0.00187	0.275847	0.516828
LINC00661	-0.01171	0.275851	0.516828
PCOLCE	0.060182	0.275872	0.516828
TMED9	-0.12615	0.27594	0.516911
LOC10192!	-0.00776	0.276002	0.516982
C6orf99	0.008867	0.276102	0.517123
ADRA1A	0.001061	0.276272	0.517397
BTN2A3P	-0.0242	0.276324	0.51745
UGT2B15	-0.01247	0.276348	0.51745
LOC10192!	-0.01146	0.276416	0.517534
KNDC1	-0.00143	0.276489	0.517581
PHF20L1	0.096359	0.276489	0.517581
SLN	0.003201	0.27652	0.517594
TRIML2	-0.00531	0.276633	0.517761
LINC00839	0.094979	0.276665	0.517775
VIPAS39	-0.08025	0.276718	0.517796
MYOCD	0.002061	0.276729	0.517796
ZNF596	0.044391	0.276747	0.517796
ABCB7	0.172388	0.276772	0.517797
HOXC12	-0.02497	0.276895	0.517981
TRAPPC11	0.091725	0.277057	0.518241
GFRA1	0.013304	0.277155	0.51833
C1QTNF7	-0.00326	0.277169	0.51833
RP5-1085F	0.073742	0.2772	0.51833
GOLGB1	-0.08565	0.277208	0.51833
PSORS1C1	0.004974	0.277238	0.51833
SARNP	-0.06102	0.277248	0.51833
IL12B	-0.02881	0.277311	0.518402
LINC00529	0.001281	0.277395	0.518514
LAGE3	0.099377	0.277472	0.518614
LOC10050(	-0.12789	0.277537	0.518691
RSPO2	0.0028	0.277563	0.518694
MOXD1	0.028912	0.277684	0.518875
ZNF200	0.126539	0.277725	0.518907
CCDC144C	-0.00626	0.277867	0.519127
RP11-467D	-0.03599	0.27794	0.51922
PPP1R3A	-0.0029	0.277972	0.519223
PDIA3	0.091681	0.27799	0.519223
OGFRP1	-0.0068	0.278162	0.5195
ACBD4	0.049184	0.278308	0.519658
NIPBL	0.09259	0.27832	0.519658
LOC40095!	-0.00601	0.278392	0.519658

EVPLL	-0.01786	0.2784	0.519658
FRA10AC1	-0.09411	0.278407	0.519658
LOC10065:	0.038886	0.278434	0.519658
SCARF2	-0.00158	0.278453	0.519658
RIPK1	0.068609	0.278483	0.519658
CLRN1-AS	0.017821	0.278485	0.519658
LOC10192!	0.01672	0.278487	0.519658
CENPW	0.145636	0.278564	0.519723
CXCL13	0.004481	0.278595	0.519723
LINC00662	0.073085	0.278607	0.519723
LOC10272	0.17686	0.278643	0.519723
CCR5	0.145293	0.278645	0.519723
LOC28576	0.001177	0.278669	0.519723
LOC10050!	-0.00455	0.27869	0.519723
TBX10	0.017673	0.278727	0.519749
PCAT4	0.004947	0.278777	0.519796
LOC33866	0.001586	0.278871	0.519881
ZNF284	-0.07742	0.278873	0.519881
ZDHHC20	-0.06381	0.278894	0.519881
OBSCN	0.035797	0.27898	0.519993
BMS1P20	-0.09881	0.279003	0.519993
GPC1	-0.02033	0.279027	0.519993
TMEM45A	-0.28464	0.279059	0.520009
LOC10192	-0.01606	0.279271	0.520273
RABIF	-0.07216	0.279277	0.520273
PACS2	0.065906	0.279283	0.520273
HEIH	0.071791	0.279351	0.520273
PSME4	-0.08181	0.279368	0.520273
PDCL2	0.011838	0.279369	0.520273
GSTM2	0.196463	0.27938	0.520273
KIF12	-0.02484	0.279393	0.520273
COMMD7	0.034788	0.279452	0.520338
PRIMA1	-0.03103	0.279499	0.520382
OR4D2	-0.01905	0.279533	0.520399
LOC34018	-0.01658	0.279628	0.520467
C2orf50	0.004395	0.279632	0.520467
ACRC	0.149192	0.279642	0.520467
MISP	-0.02858	0.279682	0.520498
MIR3916	0.168399	0.279718	0.52052
RP11-884k	0.066492	0.279805	0.520638
ZNF155	-0.06316	0.279863	0.520676
CACNG2	-0.00161	0.279874	0.520676
LRMP	-0.07986	0.280032	0.520884
VPS41	0.172566	0.280034	0.520884
LOC10050!	-0.00526	0.280112	0.520961
UROC1	-0.00511	0.28015	0.520961
ANKRD65	0.036025	0.280154	0.520961
FAM69C	0.019778	0.280174	0.520961
CORO2B	0.102399	0.280196	0.520961
MS4A15	-0.00284	0.280289	0.52109
SOGA1	-0.03315	0.280378	0.521211
JMY	-0.12994	0.280424	0.521252
LRP4	-0.12535	0.280489	0.521328
SLC10A3	0.116444	0.280514	0.52133
ASAH2B	0.028367	0.280568	0.521386
EAF1	0.106341	0.280597	0.521394
CHKB-AS1	0.044801	0.280674	0.521467
LOC28369:	-0.01852	0.280726	0.521467

TREM1	0.209651	0.280743	0.521467
NEBL-AS1	0.012465	0.280747	0.521467
RELN	0.048984	0.280756	0.521467
C7orf13	-0.11685	0.280921	0.521672
HORMAD2	0.006389	0.280934	0.521672
APTX	0.108508	0.280939	0.521672
BMS1P5	-0.10298	0.280995	0.521688
OR7E12P	0.067221	0.280996	0.521688
INTS10	-0.13002	0.281029	0.521694
IFNL1	-0.01166	0.281047	0.521694
SRP19	0.09679	0.281108	0.521714
TMEM150A	0.118592	0.281127	0.521714
RNF208	0.001563	0.28113	0.521714
STYXL1	0.121722	0.281201	0.5218
CHRNB3	0.001203	0.28128	0.521902
FAM124A	-0.01386	0.281321	0.521934
MALAT1	-0.12954	0.281365	0.521963
LOC28429	0.001523	0.281385	0.521963
LRRC4B	-0.00261	0.281447	0.522032
BC113958	-0.12611	0.281475	0.52204
LMAN1L	0.008217	0.281538	0.522109
PLCD4	-0.0317	0.281561	0.522109
RIMS3	0.147513	0.281636	0.522115
FBXL19-AS	-0.00986	0.28165	0.522115
LPCAT2	-0.13449	0.281658	0.522115
POU3F1	-0.02333	0.281666	0.522115
PDS5A	-0.08911	0.281684	0.522115
ZNF683	-0.05391	0.281712	0.522121
PTGDR	0.031861	0.281757	0.522162
RP11-96K1	-0.08475	0.281872	0.522322
MTA2	0.032693	0.281893	0.522322
HMBOX1	0.101808	0.281916	0.522322
RP5-855D2	0.016005	0.282038	0.522503
LOC72917	-0.01235	0.282153	0.522672
ADRB2	0.311486	0.282225	0.522759
KIZ	-0.10813	0.282296	0.522847
XK	0.356631	0.282348	0.522852
DIRC1	-0.0079	0.282353	0.522852
C7orf63	0.032221	0.282451	0.522852
BTBD11	-0.14861	0.282458	0.522852
LINC00242	-0.00804	0.282461	0.522852
TLR10	-0.03482	0.282466	0.522852
ZNF674-A	-0.13642	0.282468	0.522852
RAB26	-0.00642	0.282524	0.522864
FASTKD5	-0.07623	0.282527	0.522864
COG2	-0.04948	0.282547	0.522864
HAUS2	0.097295	0.282592	0.522879
PAEP	0.001055	0.282603	0.522879
C11orf84	0.038247	0.282647	0.522916
MYF5	-0.00866	0.282681	0.522934
CRYBB2P1	-0.04223	0.282788	0.523073
CPA4	-0.02036	0.282816	0.523073
LOC10192	-0.01085	0.282829	0.523073
FAM154A	-0.01157	0.282854	0.523074
CCNB3	0.031942	0.2829	0.523115
PBXIP1	0.160432	0.282957	0.523176
ACTRT3	0.057225	0.282999	0.523209
RP11-540	-0.03746	0.283032	0.523225

RXRG	-0.00449	0.283146	0.523392
BC048420	0.019824	0.283213	0.523428
RBFOX3	0.02219	0.283215	0.523428
STARD10	0.154696	0.283274	0.523494
RP11-385F	-0.06025	0.283672	0.524184
C14orf23	-0.01258	0.283768	0.524274
LRRN3	-0.01615	0.283777	0.524274
TSIX	0.190862	0.283799	0.524274
LOC93432	0.00248	0.283855	0.524274
LOC10192	0.136362	0.283864	0.524274
SNORD89	-0.12856	0.283887	0.524274
HTR7	-0.01423	0.283904	0.524274
TRIM36	0.001121	0.283914	0.524274
LOC10192	-0.01089	0.283974	0.524313
LOC10265	-0.01226	0.283984	0.524313
BBS10	-0.14062	0.284014	0.524324
DLX3	0.014653	0.284042	0.524332
OR1A1	-0.01293	0.284073	0.524343
KCNC2	0.021181	0.284173	0.524438
RP11-118C	0.018724	0.284173	0.524438
IL16	-0.17254	0.284205	0.524454
CASD1	0.011081	0.2843	0.524585
ARL14EP	-0.08358	0.284349	0.524593
PP13	-0.038	0.284354	0.524593
ALPK2	0.005769	0.284379	0.524596
HRH4	-0.01028	0.284448	0.524671
SLC29A1	0.12158	0.284482	0.524671
GEMIN4	-0.12744	0.284508	0.524671
HCG4	0.251169	0.284517	0.524671
RP11-403F	-0.1803	0.284585	0.524753
DRD3	-0.002	0.284619	0.524771
TPRXL	-0.00807	0.284716	0.524905
ZNF282	-0.11183	0.284769	0.524957
ILVBL	-0.12379	0.284823	0.525012
OTULIN	0.083069	0.284933	0.525171
RGS7	0.007001	0.28499	0.52523
MED9	-0.094	0.285176	0.525529
CERK	-0.12296	0.285214	0.525554
MUC17	-0.01845	0.285239	0.525554
LOC28391	-0.02809	0.285299	0.525621
RDH8	-0.01551	0.285347	0.525666
CUZD1	0.074872	0.285417	0.525749
CCDC170	0.02218	0.285449	0.525764
FBLN5	0.150481	0.285517	0.525844
LOC10050	0.053685	0.285565	0.525844
HEATR5A	0.192968	0.285566	0.525844
LOC10192	-0.02066	0.28565	0.525899
RB1CC1	-0.12236	0.285671	0.525899
ACVR2B-A	0.013921	0.285674	0.525899
MOP-1	0.108948	0.285693	0.525899
STOML2	0.097505	0.285747	0.525954
FGD1	-0.00899	0.285872	0.526119
RGS12	-0.01303	0.285885	0.526119
SIKE1	-0.10052	0.286134	0.526533
ROBO1	0.377839	0.286219	0.526643
SLC39A13	0.038765	0.286312	0.52673
POU6F2	-0.01111	0.286326	0.52673
ARID5A	0.116748	0.286339	0.52673

OR2F2	-0.00502	0.286372	0.526746
PSTPIP1	0.113561	0.286501	0.526895
USP32P2	-0.20444	0.286502	0.526895
IDI2	0.003481	0.286601	0.527034
ERVMER61	0.001067	0.286657	0.527047
TMEM185/	-0.10529	0.286657	0.527047
CPNE3	-0.09984	0.286722	0.527121
BCL11A	0.150265	0.286767	0.52716
ARHGEF7-	-0.00276	0.286846	0.52722
KISS1	-0.00249	0.286873	0.52722
TAAR3	-0.00213	0.286885	0.52722
KNOP1	-0.10859	0.286897	0.52722
DLGAP1	-0.00604	0.287059	0.527473
MICALL2	0.074284	0.28712	0.527524
KITLG	0.012385	0.287149	0.527524
RP1-181J2	-0.01435	0.28716	0.527524
CTD-2313	0.001862	0.287216	0.527551
P2RY12	0.225648	0.287224	0.527551
LOC10192	0.00154	0.287319	0.527682
RP11-634E	-0.00931	0.287374	0.527739
KRTAP4-8	-0.03077	0.287424	0.527785
GABRQ	0.006734	0.287458	0.527802
TMEM237	-0.10353	0.287529	0.527864
ZKSCAN1	0.139785	0.28754	0.527864
LOC10028	-0.10857	0.287572	0.527878
PAGE4	-0.00479	0.287728	0.528119
DNAH14	0.018591	0.287819	0.528242
KCNIP1	0.001125	0.287847	0.528249
SERPINH1	0.174386	0.287892	0.528262
MAGEE2	-0.02386	0.287909	0.528262
GNGT1	-0.02606	0.287945	0.528262
ATP7A	0.105627	0.287968	0.528262
CCDC130	0.083385	0.288002	0.528262
CRYGD	-0.22725	0.288027	0.528262
ABTB1	0.006417	0.288039	0.528262
LOC10192	-0.01184	0.28805	0.528262
KIF15	-0.20216	0.288092	0.528295
VSTM2A	0.001493	0.288271	0.528578
LOC10050	-0.03847	0.288336	0.528648
LINC00889	-0.02958	0.288358	0.528648
RASSF6	-0.15756	0.288423	0.528723
ZNF316	-0.03307	0.288605	0.528956
GSTM1	0.174296	0.288626	0.528956
PCDHB7	-0.01367	0.288666	0.528956
TMPO-AS	0.051828	0.288667	0.528956
UBE2E1	0.064435	0.288715	0.528956
COL17A1	0.00329	0.28872	0.528956
CCDC65	-0.008	0.288721	0.528956
COL23A1	0.182555	0.288767	0.528967
AC007680.	-0.00437	0.288776	0.528967
ALG6	-0.10458	0.288828	0.529017
PKHD1	-0.00335	0.288916	0.529094
RERG	-0.0143	0.288919	0.529094
CTSG	0.352646	0.289057	0.529223
SPINT3	0.001015	0.289062	0.529223
HIST1H2BE	0.191489	0.289126	0.529223
C17orf77	-0.01906	0.289127	0.529223
OR1Q1	-0.00435	0.289139	0.529223

CEP120	-0.07911	0.289161	0.529223
C1orf64	-0.03174	0.289186	0.529223
ATG3	0.095271	0.289188	0.529223
ADRB3	-0.01092	0.289209	0.529223
FLJ30403	-0.00901	0.289362	0.529396
WASL	0.092312	0.289363	0.529396
BHMT	-0.01796	0.289377	0.529396
LOC10192	-0.03467	0.289413	0.529417
LOC10012	0.010001	0.289475	0.529486
PADI3	-0.01011	0.289548	0.52956
LOC10192	0.002491	0.289593	0.52956
IGSF3	-0.00477	0.289602	0.52956
PMCHL2	0.006638	0.289641	0.52956
LOC10050	0.102608	0.289649	0.52956
TBX20	-0.00311	0.289663	0.52956
PAFAH1B3	0.116202	0.289687	0.52956
FLJ26850	0.014029	0.289847	0.529808
OR7E19P	0.003636	0.28993	0.529883
AKR1C1	0.062009	0.28994	0.529883
RP11-432J	-0.03009	0.290007	0.529883
PCCA	-0.04907	0.290027	0.529883
LOC64869	0.00228	0.290045	0.529883
FAM189A1	-0.01845	0.29005	0.529883
CMTM6	0.062019	0.290059	0.529883
ITGA3	-0.02397	0.290097	0.529908
PAX2	-0.00463	0.290194	0.530003
TMEM187	-0.13152	0.290209	0.530003
RBPJ	0.126824	0.290223	0.530003
BAI3	-0.07745	0.290254	0.530016
C19orf73	0.011757	0.290408	0.530251
HECTD4	0.056025	0.290451	0.530285
ATXN2	0.11604	0.290536	0.530396
RAB9BP1	0.001784	0.290598	0.530464
DNAH5	-0.05438	0.29064	0.530469
ZNF594	0.065008	0.29065	0.530469
ZNF839	-0.045	0.290769	0.530643
NXF1	0.093874	0.290824	0.530676
TNR	-0.00115	0.290836	0.530676
BLK	0.070968	0.290979	0.530892
C15orf56	0.027288	0.291025	0.53093
RP11-333C	-0.0063	0.291072	0.530972
TFAP2E	-0.09579	0.291258	0.531267
OR51B5	-0.00378	0.291443	0.531559
FNDC1	0.010985	0.291529	0.531671
RCOR2	-0.03532	0.291609	0.531748
METAP1D	-0.01047	0.291625	0.531748
HTT	-0.08692	0.291649	0.531748
TSPYL6	-0.01179	0.291684	0.531748
ZNF29P	-0.00493	0.291694	0.531748
RYR3	-0.34681	0.291747	0.531801
RP11-506E	-0.00479	0.291785	0.531825
IDH3A	-0.10364	0.291845	0.531888
ZSCAN32	0.047389	0.291901	0.531908
CPNE5	-0.1277	0.291905	0.531908
CCL21	-0.01265	0.291955	0.53191
LOC15258	0.001872	0.291955	0.53191
NUBP1	0.101405	0.291996	0.53194
SGMS2	0.099725	0.292045	0.531984

ATP13A4	-0.00829	0.292091	0.532024
KCND3	-0.00716	0.292134	0.532058
KIR2DS5	0.028357	0.292285	0.532239
NXPE4	-0.00689	0.292293	0.532239
LEAP2	-0.16092	0.292307	0.532239
LOC38989!	0.047857	0.292459	0.532471
ZNF814	-0.11816	0.292504	0.532506
FLCN	-0.05287	0.292636	0.532702
CD1C	0.214556	0.292702	0.532778
ZNF141	-0.09184	0.292755	0.532824
RNF212	-0.02042	0.29278	0.532824
SDC1	0.122621	0.292801	0.532824
SCART1	-0.0276	0.292885	0.532855
RYR2	0.045224	0.292887	0.532855
MMP27	-0.0184	0.292922	0.532855
PKD2L2	-0.02368	0.29296	0.532855
CYB5R2	-0.02806	0.292962	0.532855
EML6	-0.08219	0.292965	0.532855
ADAMTS12	-0.01789	0.293065	0.532991
PSMA2	-0.06052	0.293129	0.533018
ZNF503-A!	-0.04017	0.293139	0.533018
TNKS	0.020363	0.293154	0.533018
OSBPL2	0.074379	0.293207	0.533036
PGAP2	0.088075	0.29324	0.533036
SLC12A7	0.092759	0.293285	0.533036
RP11-443(	-0.02378	0.293312	0.533036
CDC37L1	0.117665	0.293364	0.533036
LINC01119	0.021472	0.293386	0.533036
LOC10192	-0.01288	0.293423	0.533036
FBXO9	0.119999	0.293461	0.533036
RPL39L	0.188841	0.29347	0.533036
N4BP3	-0.02249	0.293473	0.533036
TMEM253	-0.00267	0.293473	0.533036
TCEB3-AS1	-0.12985	0.293478	0.533036
SYCE1	-0.03413	0.293484	0.533036
PSG1	0.001169	0.293552	0.533116
C17orf50	-0.02174	0.293609	0.533174
CCL11	-0.01744	0.293722	0.533249
AMER3	0.001134	0.293737	0.533249
RP11-932(	-0.00177	0.293749	0.533249
AS3MT	-0.07057	0.293752	0.533249
TRPC4	0.002593	0.293784	0.533249
HSPB7	-0.00943	0.293798	0.533249
CDKN2B-A	-0.00225	0.293861	0.533319
SNX31	-0.00785	0.294025	0.533556
SLC25A21	0.245369	0.294041	0.533556
LINC01111	-0.00511	0.294087	0.533595
MAP2K1	0.092256	0.294163	0.533635
MYLK2	0.009688	0.294197	0.533635
IMP4	0.109733	0.294227	0.533635
ANAPC13	-0.03301	0.294228	0.533635
LOC72973:	0.04531	0.294232	0.533635
CSN1S2AP	0.012517	0.294304	0.53372
AEN	-0.11143	0.294435	0.533914
ZAK	0.107093	0.294556	0.533985
LOC10192(	-0.00392	0.294564	0.533985
AMDHD1	-0.08563	0.294568	0.533985
LIG3	0.113503	0.294573	0.533985

LRRC37BP1	-0.00712	0.294617	0.53402
IL17RC	-0.02353	0.294682	0.534039
GLS2	0.011623	0.294689	0.534039
LOC10050!	0.102228	0.294712	0.534039
SEC61B	0.09531	0.294726	0.534039
PTPN11	0.052507	0.294819	0.534155
LOC10012{	-0.03755	0.294857	0.534155
BCAN	-0.00361	0.294864	0.534155
LOC28460(	0.059506	0.295068	0.534438
LINC00477	0.004656	0.295074	0.534438
KLHL41	0.005908	0.295096	0.534438
MYOM3	-0.0165	0.295119	0.534438
TRG-AS1	-0.31604	0.295147	0.534444
AFP	-0.01648	0.295172	0.534445
RHEBL1	-0.09182	0.295244	0.53453
FENDRR	-0.02435	0.295293	0.534551
ERV3-2	0.193539	0.295305	0.534551
VPREB1	0.394266	0.295419	0.534712
CLDN1	0.011239	0.295489	0.534795
PRKCA	-0.03094	0.295522	0.53481
MAGEA11	0.001144	0.295638	0.534938
LOC10050?	-0.01557	0.295664	0.534938
LOC34007	-0.0055	0.295708	0.534938
PINX1	-0.10382	0.295727	0.534938
CCL16	-0.01202	0.29573	0.534938
ZBED6	0.098213	0.295741	0.534938
LOC10192{	-0.00425	0.295782	0.534962
BNIPL	-0.00395	0.295804	0.534962
PRCP	0.043244	0.295904	0.53505
TNK2-AS1	-0.02207	0.295928	0.53505
DQ576800	-0.0095	0.29594	0.53505
RP11-138I	-0.01422	0.295951	0.53505
FER1L5	-0.00806	0.296137	0.535341
CXCL2	0.160967	0.296218	0.535392
LINC01207	0.00147	0.296235	0.535392
CCDC108	0.001292	0.29624	0.535392
GPR143	-0.00543	0.296397	0.535633
PDZRN3-A	-0.03162	0.296534	0.535835
ZC3H6	-0.08244	0.296602	0.535912
ARSK	0.067046	0.296637	0.535931
SLC6A7	-0.00972	0.296749	0.536009
DAGLA	-0.01084	0.296752	0.536009
LINC00938	-0.18135	0.296769	0.536009
PKNOX2	-0.00842	0.296779	0.536009
LOC33896	-0.02604	0.296844	0.536081
TGIF2	-0.10621	0.297208	0.536695
FOLH1	-0.01654	0.297255	0.536735
GJB3	-0.01383	0.297328	0.536822
DNAH11	-0.00294	0.297387	0.536883
GUSBP11	-0.03482	0.297632	0.537196
IQCF5	0.011624	0.297648	0.537196
LTK	0.057645	0.297664	0.537196
ASGR1	0.06519	0.29771	0.537196
AMOTL1	-0.04431	0.297733	0.537196
HSD17B6	-0.0674	0.297745	0.537196
LINC00210	0.004308	0.297754	0.537196
DLEU7	-0.00161	0.297759	0.537196
SMLR1	0.010177	0.297791	0.53721

GTF2E1	0.104905	0.297838	0.53725
IFNA17	0.002972	0.298048	0.537583
MIOS	-0.083	0.298154	0.537668
HTR1A	-0.01344	0.298166	0.537668
ICAM2	0.099849	0.298169	0.537668
SLC47A2	-0.00818	0.298197	0.537674
BTBD6	0.164632	0.29832	0.537799
UBE2NL	-0.04296	0.298338	0.537799
VPS13A-A:	-0.02527	0.298341	0.537799
ZNF519	-0.12984	0.298373	0.537811
FUBP3	0.075643	0.298501	0.537997
STEAP4	0.110656	0.298597	0.538126
EXO1	0.181561	0.29866	0.538195
TMEM185E	0.080391	0.298861	0.538512
DKFZp547.	-0.00346	0.298887	0.538513
NR1I2	-0.08055	0.299172	0.538982
RP11-403N	-0.02866	0.299521	0.53955
SLC26A4-/	-0.00396	0.299545	0.53955
ANKHD1	-0.15395	0.299627	0.53955
GATS	0.042074	0.29963	0.53955
FAM135B	-0.00153	0.299635	0.53955
CXXC5	0.095707	0.299637	0.53955
HNRNPA2I	-0.09279	0.29987	0.539926
LOC28542:	-0.00321	0.299902	0.539939
LOC10192{	-0.00443	0.299931	0.539945
NDE1	0.065463	0.300063	0.540139
LOC10192	-0.00734	0.30013	0.540172
AADAC	-0.01378	0.300145	0.540172
EXOC5	0.151798	0.300157	0.540172
RXFP2	-0.00194	0.300192	0.540191
ASAP1-IT2	0.142777	0.300253	0.540257
OR6W1P	0.010122	0.300351	0.540387
LDLRAD4-.	-0.00427	0.300483	0.54058
DLL1	0.097192	0.300696	0.540891
AC104667.	0.012535	0.300706	0.540891
ARHGEF25	-0.03314	0.300731	0.540891
DAOA-AS:	0.005309	0.300888	0.541096
RP11-629E	-0.01713	0.300895	0.541096
KBTBD3	0.081399	0.300961	0.54117
UQCRC2	-0.07999	0.300996	0.541187
CPA2	-0.03002	0.301028	0.541201
TONSL	-0.03542	0.301081	0.541251
TUBB7P	0.013739	0.301133	0.5413
NEDD4L	0.158173	0.301179	0.541316
SWAP70	-0.10441	0.301192	0.541316
TMEM135	-0.04225	0.301264	0.541399
TM4SF5	-0.00704	0.301419	0.541633
LOC10099(	0.011673	0.301502	0.541738
LOC10050!	-0.00307	0.301557	0.541758
BCAT2	-0.05542	0.301563	0.541758
FADS3	0.133096	0.301607	0.541792
FANCC	0.023381	0.301731	0.541922
TMEM161E	-0.0812	0.30175	0.541922
LOC10192	0.017928	0.301755	0.541922
IQCF2	0.008581	0.30179	0.541941
ARNTL2-A	0.137488	0.301866	0.542033
NXNL2	-0.01667	0.301976	0.542143
LOC10192!	0.001842	0.301978	0.542143

ZNF569	-0.07327	0.302018	0.542159
RHO	-0.02138	0.302037	0.542159
LINC01125	-0.06453	0.302107	0.542239
FLJ46026	-0.00729	0.302174	0.542315
MKKS	-0.06845	0.302199	0.542316
SCAF4	-0.13126	0.30228	0.542417
SDS	0.018284	0.302591	0.542907
RP5-944M	0.001053	0.302604	0.542907
FBLN7	-0.04073	0.302816	0.543242
GIF	-0.01572	0.302848	0.543255
ADCY9	-0.03912	0.302879	0.543266
LOC28585	-0.02077	0.303048	0.543474
FTHL17	-0.01313	0.30306	0.543474
C3orf55	0.036841	0.30307	0.543474
GRAMD2	-0.01569	0.303154	0.543578
TTTY13	0.003969	0.303226	0.543663
TMEM63C	0.021373	0.303282	0.543718
LTB	-0.16242	0.303326	0.543726
RFXANK	0.090538	0.30334	0.543726
RP11-589F	0.143785	0.303382	0.543726
PKD2	-0.13372	0.303387	0.543726
RP11-426C	-0.05083	0.303462	0.543802
UBAP1L	-0.0245	0.303479	0.543802
PHKB	-0.06931	0.303574	0.543926
GSX1	0.005066	0.303632	0.543985
GALNT15	0.059261	0.303744	0.544141
IL2RB	0.121912	0.303851	0.544221
VAT1L	0.067567	0.303878	0.544221
LOC10050	-0.01532	0.303916	0.544221
C11orf74	-0.09888	0.303932	0.544221
LOC10272	0.001459	0.303952	0.544221
LOC10192	-0.00833	0.30396	0.544221
IFNA1	0.002726	0.303965	0.544221
ARNTL2	0.119679	0.304078	0.54438
AC003973.	-0.00198	0.304127	0.544423
GK2	-0.01369	0.304213	0.544501
TIAM1	0.101639	0.304221	0.544501
RP11-53O	-0.11951	0.304271	0.544545
ADORA2A	-0.00294	0.304384	0.544693
SSX3	-0.00844	0.304404	0.544693
KCNK10	0.005139	0.304454	0.544737
NRTN	0.00298	0.304563	0.544859
RP11-274H	-0.0218	0.304573	0.544859
NDUFS6	-0.09645	0.304636	0.544889
PRSS53	0.030927	0.304685	0.544889
CHDH	-0.01407	0.304685	0.544889
LOC10192	0.001548	0.30469	0.544889
SPINK7	0.001357	0.304769	0.544986
PITX2	-0.11291	0.304863	0.545109
GRASPOS	-0.00466	0.304983	0.545276
EHBP1	-0.0596	0.305007	0.545276
RP1-28O1	0.008161	0.305103	0.545403
TRAPPC8	0.091793	0.305145	0.545424
FGF7	-0.00326	0.305181	0.545424
RP11-157F	0.016545	0.3052	0.545424
LOC10042	-0.01498	0.305216	0.545424
DNAJB14	0.115835	0.305385	0.545681
GPR110	-0.03829	0.305426	0.545709

GPATCH1	0.0845	0.305504	0.545804
LOC10192!	0.001837	0.305644	0.546009
BZW2	-0.0686	0.305709	0.54608
LAMC2	-0.00645	0.305766	0.546133
OR5K1	0.003422	0.305789	0.546133
LOC10192	-0.02651	0.305839	0.546178
LOC10192	0.015336	0.305914	0.546206
ZNF677	0.083726	0.306034	0.546206
AF289551	-0.09875	0.306039	0.546206
CD2AP	-0.04196	0.306042	0.546206
LOC10192	-0.01781	0.306096	0.546206
LOC34009	0.00154	0.306109	0.546206
C10orf76	0.077696	0.306115	0.546206
CAPS	0.03385	0.306116	0.546206
LOC10192	-0.00798	0.306119	0.546206
RP4-730D	-0.00528	0.306127	0.546206
IGHV5-78	-0.2665	0.306133	0.546206
OR2S2	0.003935	0.306184	0.546252
TMEM60	-0.10163	0.306256	0.546322
ING4	0.08209	0.30632	0.546322
ILF2	0.079301	0.306342	0.546322
DOT1L	0.12073	0.306345	0.546322
KRBOX1-A	-0.01863	0.306374	0.546322
CXorf24	0.12023	0.306374	0.546322
HMCN1	0.057656	0.306507	0.546466
AC079741.	-0.0124	0.306528	0.546466
KNG1	-0.00641	0.306531	0.546466
LOC10028	-0.01828	0.306612	0.546546
ZEB2	-0.16231	0.306626	0.546546
LOC10192	-0.00444	0.306731	0.546553
LINC00161	-0.01227	0.30678	0.546553
CA5A	-0.05959	0.306824	0.546553
LOC10050	-0.02063	0.306836	0.546553
IL7	-0.2125	0.306842	0.546553
DYRK1A	0.006281	0.306854	0.546553
ZNF818P	0.120564	0.30687	0.546553
LOC10192	-0.013	0.306887	0.546553
LOC10013	0.024371	0.306896	0.546553
SYT6	-0.01543	0.306902	0.546553
GAGE1	0.002707	0.306942	0.546553
GPATCH8	-0.07796	0.306962	0.546553
CCL25	0.01397	0.306973	0.546553
CTC-436K	0.003189	0.307027	0.546553
SMG7-AS1	-0.01408	0.307055	0.546553
STGC3	0.003343	0.30708	0.546553
ERCC6L2	-0.08054	0.307093	0.546553
TAS1R1	-0.00399	0.307129	0.546553
SLC9A8	0.066983	0.307131	0.546553
VPS37D	-0.0322	0.307135	0.546553
C1orf21	-0.11781	0.307196	0.546617
LOC10192	0.018527	0.307226	0.546625
NDUFA2	0.014532	0.307251	0.546625
AC008753.	-0.05791	0.307305	0.546676
ZNF333	-0.05458	0.307332	0.546679
CREB3L1	-0.04989	0.307424	0.546777
DEGS2	0.084514	0.307437	0.546777
TRIM38	-0.08826	0.307505	0.546852
LOC55420	-0.00243	0.307574	0.546916

ATP6V0A2	-0.11933	0.307592	0.546916
AGFG2	-0.00274	0.307703	0.546992
TMEM134	-0.01066	0.307709	0.546992
ARHGEF39	0.046306	0.30771	0.546992
TMEM173	-0.15099	0.307735	0.546992
LINC00896	-0.02381	0.307822	0.547062
CALR3	-0.00557	0.307825	0.547062
ORC5	0.137624	0.307892	0.547096
LINC00488	-0.00706	0.307965	0.547096
GALNT18	0.037032	0.307979	0.547096
TMEM56	0.279449	0.308005	0.547096
CAPZA3	-0.00243	0.30802	0.547096
OR2K2	-0.01204	0.30803	0.547096
C18orf25	-0.08346	0.308039	0.547096
ZNF71	-0.0131	0.308046	0.547096
ZNF213	0.064178	0.308105	0.547111
LINC00478	0.115088	0.308127	0.547111
LINC00656	0.014429	0.308131	0.547111
RP5-1184F	-0.01768	0.308196	0.547182
LA16c-395	0.004475	0.308283	0.547292
SLC35F3	0.036695	0.308359	0.547333
SYNE2	-0.00501	0.308408	0.547333
AF007147	-0.14458	0.308415	0.547333
SCN5A	-0.02154	0.308416	0.547333
BRMS1L	-0.04815	0.308438	0.547333
PSAPL1	-0.04213	0.308457	0.547333
RMDN2	-0.0524	0.3085	0.547341
LOC10192	-0.00597	0.308537	0.547341
GRAP	0.036201	0.308546	0.547341
PDE3B	-0.18355	0.308563	0.547341
TMEM114	-0.0061	0.308606	0.547372
ACOT8	-0.08715	0.308662	0.547378
TMEM72-A	-0.01452	0.308668	0.547378
CWC25	-0.09418	0.308685	0.547378
MEIG1	0.03173	0.308806	0.547548
KRTAP9-9	0.002378	0.308852	0.547563
AC005224.	0.021265	0.308866	0.547563
AHSG	-0.02239	0.308945	0.54766
MASP2	-0.00784	0.30904	0.547784
PGS1	0.040119	0.309072	0.547794
NOP14-AS	-0.00965	0.309128	0.547845
ALOX15	-0.02674	0.309176	0.547845
LINC00710	0.001757	0.309176	0.547845
RP11-402C	-0.00252	0.309247	0.547925
PPAPDC1B	-0.07451	0.309326	0.548021
ZNF74	0.071515	0.309393	0.548095
LOC10192	-0.0047	0.30947	0.548186
C11orf85	-0.0103	0.309545	0.548264
RP4-584D1	-0.0018	0.309565	0.548264
AXDND1	0.005252	0.3097	0.548434
FOXP4	0.033654	0.309711	0.548434
ATF2	0.104011	0.309753	0.548464
FAM19A2	-0.2624	0.309934	0.548739
RP11-218C	0.00509	0.310006	0.548821
CPEB1	0.049978	0.310101	0.548945
CTNS	0.135144	0.310176	0.549029
C15orf62	-0.0015	0.31022	0.549029
OR14J1	0.001316	0.310225	0.549029

PTPLB	0.090083	0.310385	0.549268
CDKL1	-0.0947	0.310457	0.54935
LOC10013:	-0.02266	0.310529	0.549432
MACROD2	0.046273	0.310587	0.54945
GNAL	-0.01313	0.31059	0.54945
LBX1-AS1	0.01272	0.310631	0.549479
SLC10A4	-0.37386	0.310664	0.549492
LINC00520	-0.10958	0.310717	0.549541
ACTC1	0.031762	0.310833	0.549687
LOC10192	-0.03132	0.310873	0.549687
COL5A2	0.112378	0.310891	0.549687
GZMA	0.214265	0.310901	0.549687
CPD	-0.12612	0.31099	0.549754
GAB1	-0.10188	0.31099	0.549754
CCT8L2	-0.00459	0.311034	0.549764
MSI2	0.156877	0.311072	0.549764
LMX1A	-0.00939	0.311086	0.549764
ACAA1	0.076085	0.311097	0.549764
FGF19	0.004753	0.311152	0.549816
HIST1H2B(	-0.08127	0.311244	0.549934
ZHX1	0.078032	0.311298	0.54994
BRS3	0.007645	0.311298	0.54994
LOC10050	-0.03512	0.311344	0.549951
MLK7-AS1	-0.00665	0.311355	0.549951
SLC44A5	0.047801	0.311399	0.549983
RP11-360k	-0.00349	0.311485	0.550091
PRAF2	0.118584	0.311808	0.550617
AVPR2	-0.02126	0.31186	0.550627
PNPLA8	0.115229	0.311877	0.550627
FOXD1	0.011614	0.311891	0.550627
IPO5P1	-0.01315	0.311946	0.55064
CD276	0.017982	0.311949	0.55064
ZCCHC14	-0.06655	0.312009	0.550701
IMMT	0.066538	0.312078	0.550758
NID2	0.218463	0.312092	0.550758
CXorf40A	-0.06854	0.31212	0.550762
CHML	-0.03733	0.312285	0.551008
CCL1	-0.00702	0.312445	0.55124
LOC33987	-0.00625	0.312467	0.55124
ARHGAP2	-0.08516	0.312547	0.551298
CERCAM	0.020211	0.312551	0.551298
LOC10192	-0.04599	0.312671	0.551466
YTHDF3-A	-0.05077	0.312728	0.551521
RASGEF1A	-0.10845	0.312759	0.55153
LOC10192	-0.01057	0.312795	0.551548
TPRN	0.030842	0.312873	0.551642
LINC00284	-0.00385	0.312964	0.551757
TMEM100	-0.02323	0.313095	0.551926
XYLT2	-0.0495	0.31311	0.551926
BPY2	-0.01684	0.313156	0.55196
IFFO2	-0.11946	0.313243	0.55207
CTB-43E1	-0.01282	0.313359	0.552194
CNIH2	0.012573	0.313365	0.552194
LOC10192	0.002028	0.31342	0.552206
LOC10192	-0.1548	0.313466	0.552206
CTD-2083	-0.02167	0.313468	0.552206
HHIPL2	-0.01009	0.31349	0.552206
NRSN1	-0.02143	0.313512	0.552206

CLEC12B	0.086965	0.313524	0.552206
FSD2	-0.00174	0.313614	0.552308
PSMB7	-0.08725	0.313634	0.552308
KRT79	-0.01985	0.313696	0.552331
RNF133	0.01012	0.313698	0.552331
KIAA0556	-0.08298	0.313728	0.552341
LOC10099(	-0.02015	0.313757	0.552345
DDB1	0.061823	0.313794	0.552366
PCDHB5	0.189443	0.313886	0.552483
LOC10099(	-0.0693	0.313945	0.552543
SCAMP1	0.081671	0.314063	0.552704
CCDC85A	-0.01486	0.314137	0.55279
KREMEN2	-0.014	0.314216	0.552884
C14orf178	-0.00839	0.314296	0.55298
ZNF222	-0.12277	0.314448	0.553078
FNIP1	-0.10088	0.314452	0.553078
CNPY1	0.007598	0.314452	0.553078
LOC28466(	-0.03294	0.314454	0.553078
EPHA8	-0.02009	0.314545	0.553194
TBX2	-0.00651	0.314619	0.553226
RPF2	-0.09197	0.31464	0.553226
RP11-510(	0.00118	0.31464	0.553226
MEX3C	0.180054	0.314728	0.553335
PRSS12	-0.00413	0.314824	0.55346
LOC10192(	0.010253	0.314884	0.553521
FAM95A	-0.01813	0.31504	0.553627
COL3A1	0.08948	0.315055	0.553627
C6orf201	0.010962	0.315062	0.553627
MBNL1	0.14978	0.315073	0.553627
RP11-348E	0.002237	0.315073	0.553627
TPPP2	-0.00251	0.315291	0.553965
APCDD1L	-0.0343	0.315364	0.554049
NPNT	0.00356	0.315406	0.554052
RP4-614O	-0.13588	0.315417	0.554052
ARCN1	0.068125	0.315539	0.554221
NUDT7	0.104472	0.315602	0.554287
LOC10272	-0.19546	0.315676	0.554373
MMP7	-0.18134	0.315778	0.554506
SLC22A9	0.00758	0.315939	0.554744
TVP23A	0.089283	0.316002	0.554806
SHROOM2	0.050857	0.316025	0.554806
CYP3A4	-0.00555	0.316302	0.555246
RSPH3	0.068284	0.31635	0.555285
CTD-2287(	-0.04766	0.316392	0.55529
DNAJB6	0.133196	0.316412	0.55529
EIF2B2	-0.08635	0.316429	0.55529
DOCK1	-0.05214	0.316485	0.555331
CADM1	0.006543	0.316535	0.555331
ZNF879	-0.13466	0.316554	0.555331
PDX1	-0.00165	0.316556	0.555331
RP11-474F	-0.0222	0.316664	0.555477
DKFZP761(	-0.01427	0.316725	0.555539
LOC72835	-0.00984	0.316877	0.555759
BTN2A2	0.068862	0.316912	0.555777
SYT4	-0.05498	0.31712	0.556054
LOC10050	-0.10118	0.317121	0.556054
FLJ20021	0.089066	0.317239	0.556091
EPB41L4A-	-0.01665	0.317277	0.556091

KLK5	-0.00209	0.317303	0.556091
BMPER	0.016556	0.317324	0.556091
DPH6	0.119991	0.317325	0.556091
LOC10050(	-0.00357	0.317333	0.556091
GPLD1	0.007675	0.317345	0.556091
LRRC19	0.002138	0.317348	0.556091
LINC01356	-0.06285	0.317422	0.556175
PDILT	-0.00408	0.317455	0.556189
LOC10028!	0.038542	0.317495	0.556214
SLC25A26	-0.09468	0.317533	0.556236
RNF32	0.001889	0.317639	0.556369
CCDC60	-0.02877	0.317661	0.556369
MBOAT7	0.108003	0.317689	0.556373
PDCD10	-0.09119	0.317839	0.556591
LOC10192!	0.001382	0.31788	0.556618
PPP2R3A	0.106807	0.317945	0.556684
BLNK	0.444554	0.317969	0.556684
TRPS1	0.147121	0.318092	0.556853
FAM78B	-0.00844	0.318184	0.556905
TRUB2	0.095243	0.318201	0.556905
FAM26D	0.002883	0.318215	0.556905
FAM219A	-0.05338	0.318235	0.556905
TBX18	-0.00527	0.31825	0.556905
SCNN1G	-0.00695	0.318385	0.557096
HSPA5	0.112932	0.318421	0.557115
DNASE1L2	-0.0238	0.318448	0.557117
SGCA	0.005418	0.3185	0.557163
ELOVL2	-0.03557	0.318531	0.557172
AKR1D1	0.001141	0.318572	0.557199
DUSP12	0.051762	0.318631	0.557256
HYDIN2	-0.00227	0.318688	0.557283
TLR8-AS1	-0.00175	0.318697	0.557283
XKRX	-0.00427	0.318725	0.557287
C6orf118	0.005365	0.318757	0.557297
EDN1	0.031315	0.318784	0.557299
GPRC5D	0.0065	0.318901	0.557411
HSPC081	-0.0274	0.318917	0.557411
HMP19	-0.00984	0.318948	0.557411
RIPK2	0.104105	0.318959	0.557411
LMO2	-0.13535	0.318989	0.557411
LOC28345	-0.0163	0.319002	0.557411
FAM168A	0.073794	0.319065	0.557475
RP11-388N	-0.13159	0.319172	0.557618
ERVH48-1	-0.01217	0.319271	0.557672
MCEE	0.078342	0.319273	0.557672
GALR1	0.016958	0.319281	0.557672
ASB17	-0.00199	0.319327	0.557708
ALDH9A1	0.071876	0.319366	0.557731
DPF1	-0.02561	0.319435	0.557767
RP11-217E	0.007455	0.319478	0.557767
SLITRK1	0.00396	0.319497	0.557767
CEBPZ	0.070056	0.31951	0.557767
RAPGEF4	0.141407	0.319515	0.557767
LOC72930	0.001498	0.319647	0.557879
SPPL2B	-0.07491	0.31965	0.557879
SLC30A1	0.42249	0.319657	0.557879
LOC10192!	-0.00223	0.319697	0.557905
THAP6	0.088967	0.319741	0.557936

PPME1	0.096918	0.319923	0.558182
TREML5P	-0.00307	0.319939	0.558182
LINC00944	-0.00451	0.319959	0.558182
ACVR1	-0.13005	0.320125	0.558427
MIS18A	-0.0899	0.32018	0.558477
CCDC169	0.039232	0.320232	0.558523
LOC10192!	-0.08897	0.320283	0.558567
LOC10192	-0.00684	0.320357	0.558652
GPR137	-0.01473	0.320407	0.558693
BTBD10	0.065893	0.320452	0.558727
ITM2A	-0.15403	0.320517	0.558796
CACNA1B	0.030792	0.320615	0.558921
TWIST1	-0.17163	0.320651	0.558939
CEP152	0.089769	0.320743	0.559054
PLA2G12B	0.010736	0.32078	0.559073
LOC10192	0.020482	0.320816	0.559078
FOXO3	-0.08169	0.320834	0.559078
ENKD1	-0.09115	0.320908	0.559161
EMX2	-0.00258	0.320957	0.559203
MAN1A1	-0.14886	0.321054	0.559327
RP11-468E	-0.17431	0.321103	0.559367
GDAP1	-0.1159	0.321178	0.559373
SMC1A	-0.04982	0.321182	0.559373
LOC44079	0.012408	0.321184	0.559373
DNAJC13	0.120492	0.32127	0.559474
FMOD	0.04782	0.321294	0.559474
POLR2A	0.018955	0.321328	0.559489
BC041998	-0.01155	0.321398	0.559565
LOC10050	-0.02265	0.321552	0.559764
CDH18	-0.00653	0.321565	0.559764
SNX20	0.061949	0.32159	0.559764
ZNF546	0.064232	0.321749	0.559926
RP11-108E	-0.00422	0.321755	0.559926
RP11-554J	-0.11341	0.321778	0.559926
CTD-2124I	-0.11438	0.321786	0.559926
LOC10192	0.03294	0.321938	0.560145
C11orf1	-0.11889	0.322019	0.560242
UTS2B	-0.00212	0.322101	0.560338
LY6K	0.036763	0.322149	0.560338
PKIG	0.179388	0.322153	0.560338
DLG2	0.012828	0.322252	0.560432
NFAT5	-0.1468	0.322275	0.560432
RFPL3	-0.02007	0.322284	0.560432
PEG3	-0.13	0.322422	0.560627
GNG7	0.136542	0.3225	0.560718
KCNH7	-0.00457	0.322534	0.560732
RP1-149C7	-0.00393	0.32263	0.560854
RP11-231E	-0.01258	0.322707	0.560942
KCNS3	-0.06624	0.322893	0.561183
GOT1L1	0.019578	0.322897	0.561183
C16orf82	0.002191	0.322995	0.561231
ITGB7	-0.16993	0.322997	0.561231
EDARADD	-0.00697	0.323003	0.561231
ZADH2	0.086184	0.323148	0.561438
LOC15222!	-0.01907	0.323258	0.561585
SLC25A3P1	-0.00141	0.323341	0.561684
KIRREL3-A	-0.00471	0.323486	0.56189
UCHL5	-0.05787	0.323609	0.561991

FGA	0.003895	0.323619	0.561991
CLDN17	-0.00285	0.323622	0.561991
LOC10192	0.032914	0.323648	0.561991
DAD1	0.104958	0.323722	0.562076
ARHGAP22	0.005212	0.323792	0.562152
HNRNPA3	-0.06349	0.323841	0.562187
CGRRF1	-0.06809	0.323874	0.562187
BC042029	-0.06827	0.323913	0.562187
DSEL	0.006689	0.323916	0.562187
LINC01120	-0.00786	0.32395	0.5622
ACTA2-AS	-0.00721	0.324053	0.562335
NOVA1	-0.05752	0.324097	0.562366
AC141928.	0.011025	0.32419	0.56244
LOC10192	0.006664	0.324192	0.56244
SCNN1B	0.019312	0.324223	0.562448
TNNC2	-0.04933	0.324309	0.562538
LOC10192	-0.00923	0.324375	0.562538
RP11-324J	0.010933	0.324378	0.562538
ETFDH	-0.08258	0.32439	0.562538
JADE1	0.073922	0.324483	0.562538
CHTF18	-0.10613	0.324521	0.562538
USP30-AS	0.070601	0.324564	0.562538
LOC10192	0.002017	0.324568	0.562538
GTF3C6	0.067541	0.324588	0.562538
LINC00923	-0.04	0.32459	0.562538
SPSB2	0.104235	0.324607	0.562538
TKTL2	0.001213	0.324623	0.562538
SCN4B	-0.01183	0.324631	0.562538
JUN	0.250026	0.324638	0.562538
LOC44105	-0.05573	0.324723	0.562641
CLVS2	0.010536	0.324771	0.562679
FAM222A	0.015614	0.324902	0.562829
FOXE1	0.007085	0.32491	0.562829
RNF217	-0.12269	0.325054	0.56295
LGALS8-A	-0.01314	0.325076	0.56295
CNRIP1	0.337959	0.325096	0.56295
GPR39	0.002293	0.325097	0.56295
TPPP3	0.025116	0.32511	0.56295
HOXB1	-0.00927	0.325206	0.563071
T-18	0.00708	0.325325	0.563212
ZFP30	0.090556	0.325388	0.563212
TRIM5	-0.04702	0.32539	0.563212
4-Mar	0.01718	0.325391	0.563212
TTN-AS1	-0.09469	0.325442	0.563255
TCL1B	-0.04185	0.32553	0.563341
INPP5F	0.062953	0.325544	0.563341
RILPL2	-0.09376	0.3256	0.563361
PC	0.054382	0.325607	0.563361
ORC1	-0.16857	0.325705	0.563377
TESPA1	0.141046	0.325715	0.563377
GJA8	-0.01225	0.325717	0.563377
LOC28348	0.00439	0.325721	0.563377
LINC00900	0.0232	0.325776	0.563386
ZFPL1	-0.07055	0.325778	0.563386
CR936796	-0.21104	0.326113	0.563921
HOOK1	-0.2183	0.326293	0.564188
SLC35E3	-0.04618	0.326398	0.564324
CTA-268H	-0.02185	0.326435	0.564335

DSTYK	0.049435	0.326473	0.564335
LOC21969	-0.00344	0.326509	0.564335
MYBPH	-0.00589	0.326531	0.564335
PGC	-0.0038	0.326571	0.564335
LOC28437	-0.02634	0.326592	0.564335
RPGRIP1	0.01506	0.326643	0.564335
GPR162	-0.07611	0.326662	0.564335
SNORA71A	0.006847	0.326682	0.564335
CXCL5	-0.0893	0.326711	0.564335
RGR	-0.00174	0.326754	0.564335
LCORL	0.113672	0.326794	0.564335
ARMCX5	-0.05863	0.326819	0.564335
RP11-1152	-0.00397	0.32682	0.564335
MORN4	-0.04626	0.326844	0.564335
LYZL4	-0.01108	0.326866	0.564335
DQ576994	-0.04733	0.326873	0.564335
GAS1	-0.09512	0.326874	0.564335
EMB	-0.09824	0.32697	0.564444
C8orf76	0.075601	0.327065	0.564444
RP11-381F	-0.01755	0.327072	0.564444
GRP	0.00287	0.327075	0.564444
MAOA	0.096768	0.327109	0.564444
SESTD1	0.152783	0.327115	0.564444
LINC00969	-0.12428	0.327119	0.564444
RASA3	0.016294	0.327195	0.564499
PCSK1	0.009707	0.327203	0.564499
TTLL12	0.117091	0.327326	0.56466
MCM3AP-	0.091476	0.327349	0.56466
PI15	0.005274	0.327385	0.564676
LOC10192	0.01411	0.327455	0.564678
TMEM243	0.13646	0.327458	0.564678
STAM-AS1	0.02556	0.327486	0.564678
OTOGL	0.002806	0.32749	0.564678
VWA5A	0.130036	0.32752	0.564686
LINC00282	0.135781	0.327649	0.564798
ERI2	-0.0417	0.327672	0.564798
KRTAP4-1	-0.00771	0.327676	0.564798
FXYD3	-0.03247	0.32769	0.564798
ZNF800	-0.05543	0.327719	0.564804
GNA15	-0.11602	0.327759	0.564827
DGKB	-0.00386	0.327793	0.56484
ZBTB25	0.108206	0.327964	0.565049
BACH1	0.012544	0.327966	0.565049
ARL14	0.00552	0.328122	0.565245
C1QTNF9B	-0.03148	0.328132	0.565245
FAM217A	-0.01607	0.328216	0.565345
FABP1	-0.00445	0.328292	0.565421
TRPM7	-0.06804	0.328312	0.565421
DESI2	-0.06026	0.32838	0.565492
LOC10192	-0.00888	0.328425	0.565525
ZFHX4	0.001203	0.328457	0.565535
LOC10050	-0.0549	0.328538	0.565561
METTL10	-0.12731	0.328556	0.565561
SSTR2	-0.00748	0.328583	0.565561
UBR3	-0.05569	0.328601	0.565561
FAM73B	0.077916	0.328603	0.565561
RP11-84D	-0.01201	0.328677	0.565644
LINC00857	-0.00321	0.328727	0.565679

CYP2D6	0.022552	0.328749	0.565679
MAGEC2	-0.01127	0.328856	0.565817
MROH6	0.102675	0.328949	0.56592
C9	-0.01562	0.328974	0.56592
RRN3P2	-0.19836	0.328994	0.56592
TNFSF13B	-0.14766	0.32915	0.566144
KIAA1407	0.108421	0.329341	0.566427
LRRC36	-0.04133	0.329422	0.566522
LOC10050!	0.004791	0.329451	0.566527
EID2	0.08675	0.329482	0.566534
PROZ	-0.07669	0.329532	0.566576
OPCML	0.005705	0.329747	0.566856
DDX41	-0.08476	0.329761	0.566856
STAT5A	-0.08083	0.329774	0.566856
LOC10013(	0.001196	0.329803	0.56686
METRNL	0.246884	0.329907	0.566994
RORC	0.006441	0.330003	0.567115
SAMD11	0.013466	0.330104	0.56724
LOC10192ℓ	0.030127	0.330128	0.56724
PSMD14	0.074165	0.33016	0.567249
FAM131A	-0.02	0.330194	0.567253
CROT	-0.09673	0.330214	0.567253
PIGN	-0.09367	0.330279	0.567319
PWP1	-0.07634	0.330376	0.567372
MFN1	0.079637	0.330393	0.567372
C2CD4B	-0.02033	0.330406	0.567372
DQ594366	-0.02218	0.330414	0.567372
TTN	-0.02561	0.330501	0.567455
LOC10192ˀ	-0.00331	0.330516	0.567455
POMT1	-0.12784	0.330613	0.567574
FOCAD	-0.06337	0.330637	0.567574
PPY2	-0.00235	0.330695	0.567629
LINC00675	-0.02226	0.330758	0.567658
MTNR1B	0.001272	0.330765	0.567658
ATP6V0E2	0.111089	0.330828	0.567722
AVPR1A	-0.0227	0.3309	0.567792
CDC14B	-0.0631	0.330928	0.567792
GZMK	0.126931	0.330948	0.567792
LOC10192!	-0.03785	0.330995	0.567794
SUGT1	-0.07956	0.331001	0.567794
HIST1H2AI	-0.10797	0.331077	0.567879
LOC10192!	-0.00892	0.33114	0.567943
TPD52L2	0.084777	0.331183	0.56797
MBD4	-0.0513	0.331217	0.567984
VCPIP1	-0.07938	0.331355	0.568093
FAM222A-	0.002277	0.331356	0.568093
CLRN3	0.001123	0.331359	0.568093
FOLR1	-0.0212	0.331428	0.568131
FZD5	0.085545	0.331434	0.568131
LOC10272ʌ	-0.06607	0.331584	0.568344
DCAF17	0.097275	0.331613	0.568348
CLCF1	-0.0533	0.331651	0.568368
DKFZP434I	-0.00649	0.331689	0.568388
COL20A1	0.041002	0.331795	0.568525
HOXC10	-0.0107	0.331823	0.568525
CCDC51	-0.10509	0.331848	0.568525
LINC01293	-0.01738	0.331976	0.568625
ENTPD7	0.073842	0.331998	0.568625

VGLL3	0.070067	0.332039	0.568625
LRP6	-0.05635	0.332047	0.568625
RBM18	-0.08285	0.332075	0.568625
RP3-333B1	-0.00206	0.332078	0.568625
MPI	0.063477	0.332101	0.568625
CLNS1A	-0.09044	0.332116	0.568625
YJEFN3	0.039209	0.332175	0.568681
TNFRSF10(	0.044628	0.332258	0.568779
GPAT2	-0.37761	0.332407	0.568966
CNGA3	-0.00273	0.33242	0.568966
THAP2	-0.08618	0.332455	0.568981
SLC39A9	-0.01807	0.332503	0.568983
IQCJ-SCHI	-0.01189	0.332517	0.568983
AK5	-0.14607	0.332555	0.568983
ZSCAN10	-0.00116	0.332562	0.568983
RP11-359E	-0.00379	0.332591	0.568989
OTUD7B	0.018428	0.332667	0.569019
RP11-803D	-0.12857	0.33268	0.569019
DNM1	0.167298	0.332688	0.569019
TLL1	-0.00116	0.332743	0.569068
HDAC9	0.054361	0.332914	0.569316
LINC00208	-0.01957	0.332982	0.569387
HYAL3	0.134363	0.333045	0.569451
STK24-AS1	-0.02148	0.333111	0.569469
NOLC1	0.113344	0.333131	0.569469
ANLN	0.167281	0.333159	0.569469
TWIST2	-0.01319	0.333161	0.569469
NOX3	0.005094	0.33332	0.569696
CHRNG	-0.03561	0.333412	0.56976
RP11-75C9	0.072635	0.333426	0.56976
EIF4G2	-0.0508	0.333436	0.56976
C11orf88	0.005987	0.333543	0.569897
TFDP3	-0.01738	0.333623	0.569988
LOC10050!	0.007059	0.333654	0.569996
TBC1D28	-0.00371	0.333694	0.57002
ITIH1	0.00712	0.333728	0.570033
EML5	-0.0122	0.333805	0.570095
TRPC1	-0.21685	0.333834	0.570095
DCLRE1A	-0.10976	0.333843	0.570095
SGSM2	-0.12581	0.333988	0.570296
ARHGEF10	0.098471	0.334233	0.570598
CIART	0.043658	0.334243	0.570598
RP11-354I	0.001027	0.334243	0.570598
ARHGEF28	-0.00559	0.334294	0.570632
LPO	0.00699	0.334316	0.570632
SLC5A7	-0.00839	0.334436	0.570792
DPT	0.010797	0.334648	0.571068
LOC10012	0.111232	0.334651	0.571068
SPATA13	-0.07568	0.334695	0.571098
REG3A	0.010143	0.334746	0.571137
PAPPA	0.018711	0.334781	0.571137
GEMIN6	-0.07478	0.334799	0.571137
HRASLS5	-0.01362	0.334838	0.571137
PDGFRB	-0.07218	0.334871	0.571137
RAB3C	-0.02807	0.334875	0.571137
GK5	-0.08105	0.3351	0.571476
ASNSD1	-0.05784	0.335154	0.571522
ANKRD44-	0.020391	0.335203	0.57156

C19orf47	0.006888	0.335265	0.571622
MYCN	-0.00441	0.335297	0.571631
ADAMTSL4	0.026979	0.335335	0.57165
PTPRCAP	0.158833	0.335372	0.571669
F9	0.001634	0.335418	0.571703
FBXO31	0.083025	0.335465	0.571737
SLC27A3	0.117054	0.33561	0.571939
DAPP1	0.166964	0.335656	0.571972
CIDECP	-0.02443	0.335822	0.57221
LINC00559	0.009003	0.335877	0.57226
PPP1R26-A	-0.03011	0.336088	0.572574
CETN1	-0.01534	0.336216	0.572732
CELA3B	-0.01267	0.336234	0.572732
LOC10272	-0.01201	0.336286	0.572742
LMX1B	-0.01414	0.336293	0.572742
THUMPD3	-0.0454	0.336332	0.572742
CENPN	0.110628	0.336345	0.572742
CTD-2542I	0.014267	0.33652	0.572995
VASN	0.02453	0.336589	0.573066
PHYKPL	0.109039	0.336794	0.573371
STYX	-0.08962	0.336881	0.573473
KIAA0319	-0.00387	0.336948	0.573543
C11orf83	0.097446	0.337131	0.573805
TMC2	0.007957	0.337155	0.573805
TM7SF2	0.103342	0.337199	0.573834
HAGLROS	0.007023	0.33726	0.573837
KLF14	-0.01323	0.337293	0.573837
GCM2	-0.00125	0.337297	0.573837
HMGB3P1	0.088418	0.337306	0.573837
LL22NC03-	0.247482	0.337411	0.573935
CTD-2537I	-0.0131	0.337451	0.573935
MRTO4	0.112462	0.337462	0.573935
BDH1	0.058043	0.33747	0.573935
VENTX	0.06518	0.337518	0.573972
EXOC2	0.082557	0.337594	0.574028
TAMM41	0.065402	0.337646	0.574028
GPR68	-0.02142	0.337678	0.574028
EXOSC4	0.112101	0.337704	0.574028
S100A16	0.165702	0.337712	0.574028
PLCH1	-0.01005	0.337725	0.574028
SLC25A24	-0.05215	0.337737	0.574028
FAM83A	0.151292	0.337775	0.574049
NRG1	-0.02434	0.337872	0.574152
LINC00853	-0.08262	0.337898	0.574152
PPAPDC1A	0.142823	0.33792	0.574152
CCR7	-0.06329	0.337942	0.574152
RGPD4-AS	-0.00263	0.338115	0.5744
MORF4L2	0.148803	0.338162	0.574435
SLC26A1	0.012377	0.338279	0.574589
GLOD5	-0.04325	0.338325	0.574603
DOCK9	-0.01173	0.338343	0.574603
ZCWPW2	0.011796	0.338367	0.574603
HENMT1	-0.07396	0.338629	0.574915
LOC10099	-0.01852	0.338629	0.574915
LOC10050	0.011169	0.33863	0.574915
SLC45A1	0.024187	0.338777	0.575118
CD79B	-0.05412	0.338868	0.575229
ZNF117	-0.10708	0.338959	0.575338

SEC14L1P1	-0.12795	0.339001	0.575364
CYB5R3	0.064926	0.339093	0.575468
DTX4	0.097033	0.339137	0.575468
MICA	-0.07808	0.339168	0.575468
RBBP6	0.086357	0.339169	0.575468
FILIP1L	0.067547	0.339294	0.575635
FEV	-0.01662	0.339375	0.57569
SGK2	-0.01236	0.339379	0.57569
CCNYL2	-0.00144	0.339463	0.575787
CDKN1B	0.119501	0.339536	0.575866
RP11-560(	0.015555	0.339674	0.57604
NINL	0.154091	0.339692	0.57604
ESF1	-0.09431	0.33975	0.576093
C12orf66	-0.1156	0.339831	0.576186
CDNF	0.002616	0.339962	0.576362
GSC2	-0.00243	0.340138	0.576616
KIAA2018	0.029516	0.340214	0.576699
DIP2A	0.057608	0.340375	0.576927
TRAF4	-0.05072	0.340533	0.577149
ANKRD60	-0.00139	0.340628	0.577265
AARS	0.095831	0.340741	0.577354
VDR	0.066395	0.340758	0.577354
SAA3P	0.026358	0.340772	0.577354
SLC6A4	-0.08476	0.340787	0.577354
PTGIS	-0.00394	0.340897	0.577458
LOC10192:	0.00451	0.340902	0.577458
GPX5	0.006042	0.341026	0.577623
CYP11B1	-0.00672	0.341108	0.577659
ALDH3B2	-0.00276	0.341123	0.577659
PCBP2	0.081058	0.341127	0.577659
CD9	-0.025	0.34121	0.577755
MSLN	0.017775	0.341365	0.577914
HAAO	-0.03348	0.341374	0.577914
RHPN1	0.0368	0.341401	0.577914
LRRC15	-0.01793	0.341411	0.577914
SP2	-0.00537	0.341486	0.577995
C3orf36	-0.01487	0.341573	0.578098
RNF214	0.068305	0.341631	0.578147
IST1	-0.067	0.341719	0.578147
KIF2B	-0.0085	0.341747	0.578147
LOC10192	-0.00356	0.34175	0.578147
ING1	0.068771	0.341754	0.578147
TMEM50A	-0.07803	0.341762	0.578147
LOC28486!	-0.02008	0.341797	0.578161
LOC10050	-0.06661	0.341833	0.578172
ICAM5	-0.014	0.341867	0.578172
TSEN34	-0.06784	0.341886	0.578172
DHRS12	-0.04477	0.341915	0.578172
MESDC2	0.079816	0.341939	0.578172
TBX5	0.002267	0.341964	0.578172
VGLL4	0.062948	0.341991	0.578173
C12orf55	-0.00321	0.342059	0.578243
ARMCX2	0.19234	0.342102	0.578271
UCA1	-0.20144	0.34213	0.578273
GHET1	-0.00384	0.342199	0.578344
BBS9	-0.07972	0.342226	0.578345
NKX3-2	-0.00787	0.342256	0.57835
MFAP1	-0.09333	0.342421	0.578565

IL20RA	-0.00301	0.342467	0.578565
JMJD8	0.072295	0.342479	0.578565
LINC01227	-0.00908	0.342507	0.578565
SLC8A2	-0.00179	0.342531	0.578565
GATAD2A	0.039183	0.342544	0.578565
HIST1H3E	0.04131	0.342582	0.578567
LOC10050	-0.04147	0.342598	0.578567
SATB2-AS	0.006525	0.34266	0.578582
PTOV1-AS	-0.00956	0.342666	0.578582
FOXI1	-0.00537	0.342687	0.578582
SALL3	0.006398	0.342824	0.578742
GPR88	0.121149	0.342836	0.578742
BAZ1A	0.105595	0.342951	0.578855
ARL9	0.173373	0.342983	0.578855
ABCD3	0.082651	0.343018	0.578855
NOX5	-0.01544	0.343029	0.578855
ZNF398	0.093588	0.343036	0.578855
ECEL1	0.001125	0.34308	0.578867
RP11-266L	-0.00209	0.34312	0.578867
EHMT2	0.06387	0.343123	0.578867
OIT3	-0.00314	0.34321	0.578903
USP38	0.077009	0.343212	0.578903
NSA2	-0.06032	0.343225	0.578903
RP11-536C	-0.03428	0.343356	0.579058
GPKOW	-0.10819	0.343374	0.579058
ARID5B	-0.00924	0.343397	0.579058
NHP2	0.072562	0.343436	0.579078
PPP1R3D	0.059152	0.343486	0.579106
EPHA6	-0.00127	0.343506	0.579106
RFESD	0.239962	0.343596	0.579179
CFC1	0.007572	0.343623	0.579179
RP1-101G	-0.02444	0.343629	0.579179
PDE1A	-0.0197	0.343777	0.579336
ERVH-1	-0.05297	0.343804	0.579336
RP13-20L1	-0.00197	0.343823	0.579336
LINC00266	0.071277	0.343855	0.579336
RP11-12M	-0.00563	0.343868	0.579336
RP4-675G	0.011674	0.343883	0.579336
KLHL35	-0.02217	0.343935	0.579379
ZNF837	-0.01551	0.344014	0.579444
LOC10192	-0.04006	0.344053	0.579444
LOC10050	0.003084	0.344054	0.579444
DES	0.014897	0.344201	0.579647
CDCP1	-0.03043	0.344285	0.579743
GRK7	-0.0096	0.344373	0.579817
SMCR5	-0.00705	0.344383	0.579817
LOC15165	9.97E-04	0.34445	0.579886
8-Sep	0.211112	0.344491	0.57991
DHCR7	0.130054	0.344519	0.579912
XRCC3	-0.04507	0.344632	0.580056
PIAS1	0.079247	0.344692	0.580113
PPCDC	0.11003	0.344776	0.580137
ERVK3-2	0.017447	0.344787	0.580137
RAPGEF6	-0.13067	0.344795	0.580137
ZNF783	-0.08265	0.344814	0.580137
CABP7	-0.01464	0.344856	0.58016
CTCF	0.06292	0.344893	0.58016
FREM2	0.02733	0.344908	0.58016

RP4-561L2	-0.00155	0.344961	0.580204
MIR4296	-0.03962	0.345072	0.580345
TTLL13	-0.00636	0.34516	0.580416
SLC22A16	-0.11449	0.345167	0.580416
ARSF	-0.01289	0.34528	0.580561
ACACA	0.068833	0.345427	0.580658
RPS10	0.05653	0.34543	0.580658
AC124997.	-0.00864	0.345496	0.580658
LOC10272:	-0.10071	0.345515	0.580658
MGAT2	0.090923	0.345522	0.580658
ADAM32	-0.12281	0.345537	0.580658
ANKRD12	-0.07321	0.345546	0.580658
ST3GAL2	-0.07053	0.345553	0.580658
AF131215.	0.046203	0.345624	0.580733
SRRM5	0.027458	0.345654	0.580739
VAX2	-0.01155	0.345739	0.580802
CHRM2	-0.00163	0.345745	0.580802
CRMP1	-0.11354	0.34581	0.580836
CTD-2118I	-0.00585	0.345819	0.580836
UROS	0.127136	0.345964	0.581012
NTRK3	-0.00701	0.345978	0.581012
PITRM1-AS	-0.01719	0.346181	0.581225
TYMSOS	0.037367	0.346184	0.581225
FCGR1A	-0.00209	0.346185	0.581225
CCDC79	-0.00194	0.346223	0.581244
STK24	-0.08202	0.346295	0.581319
COPS3	0.06951	0.346391	0.581435
NCS1	0.013031	0.346418	0.581436
LOC72874:	0.007625	0.346512	0.58148
ATP5J	0.059108	0.346521	0.58148
RP11-66N:	-0.04402	0.346528	0.58148
ANK2	0.001037	0.346552	0.58148
LINC01122	-0.09525	0.346625	0.581527
LOC28335:	-0.0068	0.346652	0.581527
FAM71D	-0.01265	0.34666	0.581527
CTNND2	0.001121	0.346793	0.581704
SLC5A6	0.105941	0.34688	0.581745
FLJ30679	-0.00101	0.346896	0.581745
B3GNT1	-0.04507	0.346901	0.581745
LOC10192	0.002272	0.346974	0.581745
NKX6-2	-0.0096	0.346977	0.581745
ZNF214	-0.00901	0.34699	0.581745
BTBD9	-0.01539	0.347005	0.581745
CHD1	-0.10919	0.347049	0.581768
MRI1	-0.0588	0.347088	0.581768
GPR1-AS	-0.09462	0.347102	0.581768
IFNL2	0.00524	0.347127	0.581768
RBM46	0.003814	0.347172	0.581784
TMEM31	-0.01072	0.34719	0.581784
PCTP	-0.11797	0.347227	0.581801
EXOG	0.026472	0.347304	0.581847
LOC10192	-0.01221	0.347326	0.581847
RP11-384F	-0.06902	0.347335	0.581847
UBE2J2	0.077062	0.347378	0.581874
LOC28559:	-0.00778	0.34757	0.58215
FBXL20	-0.09475	0.347638	0.582219
FCER1A	0.125935	0.347677	0.582241
ELOVL4	0.014699	0.347777	0.582331

ACSS2	0.088102	0.347787	0.582331
RP4-676L2	0.002717	0.347812	0.582331
HMGN1	0.053851	0.347984	0.582544
USP13	-0.07639	0.347993	0.582544
AX748292	0.00976	0.348118	0.582708
LOC10013	0.023604	0.348195	0.582745
LOC10192	-0.03593	0.348214	0.582745
TEX11	0.003201	0.34822	0.582745
LOC10028	0.087664	0.348334	0.582866
CCDC125	-0.08728	0.348347	0.582866
CRELD2	0.105998	0.348403	0.582914
IL2	-0.00316	0.348467	0.582937
DYNC1I2	-0.1354	0.34847	0.582937
C6orf57	0.116775	0.348515	0.582968
SRL	-0.03177	0.348698	0.583228
PVALB	-0.10739	0.348821	0.583304
DTX1	0.079532	0.348844	0.583304
TRMT112	0.081834	0.348889	0.583304
LOC33997	-0.02711	0.348892	0.583304
RP11-457K	-0.00469	0.3489	0.583304
UVRAG	-0.09375	0.348905	0.583304
NKAPL	-0.01565	0.348986	0.583353
IL18	0.126465	0.349	0.583353
LOC10192	-0.0013	0.349034	0.583353
AP4M1	0.067091	0.349042	0.583353
FGF17	0.016991	0.349075	0.583363
TMEM178A	0.115499	0.349109	0.583375
HES1	-0.20224	0.349136	0.583375
GIP	-0.00971	0.349197	0.583408
FAM178B	-0.06334	0.349209	0.583408
C20orf166-	-0.01941	0.349292	0.583428
FAM19A5	-0.06495	0.349341	0.583428
GTF2A1	0.053701	0.349346	0.583428
LRRC8E	0.026596	0.349349	0.583428
AWAT1	-0.00998	0.349356	0.583428
CCKBR	-0.01711	0.349426	0.583473
NRM	-0.11004	0.349437	0.583473
SERPINB10	0.194522	0.349541	0.583601
NUP85	-0.0963	0.349746	0.583886
SLC7A6	-0.13664	0.349775	0.583886
DYRK1B	0.020895	0.349792	0.583886
MUC15	-0.00467	0.35014	0.584421
FGD4	0.080236	0.350292	0.58463
BSDC1	0.068813	0.350426	0.584761
CSRP3	0.006082	0.350428	0.584761
RASSF9	0.061363	0.35047	0.584761
ZNF471	0.040401	0.350491	0.584761
TEP1	0.0949	0.350513	0.584761
LOC64526	-0.00554	0.350539	0.584761
RP11-806L	0.006897	0.350559	0.584761
CALM1	-0.12542	0.350712	0.584951
MSX1	0.034777	0.350733	0.584951
RBAK	-0.11481	0.350754	0.584951
JAKMIP1	0.007112	0.350785	0.584956
RNF139-AS	0.063104	0.350817	0.584965
SLC17A2	-0.01022	0.350981	0.585115
PDZD4	0.010058	0.350988	0.585115
LAX1	-0.13021	0.350999	0.585115

CPT1A	-0.09478	0.351015	0.585115
CYP4F8	-0.01701	0.351058	0.585142
USP14	0.060678	0.351129	0.585216
WNT7A	-0.01177	0.351257	0.585383
EP300-AS1	0.091185	0.351401	0.585562
C12orf57	0.119436	0.351431	0.585562
LOC28348	-0.00627	0.351446	0.585562
RAB33A	-0.17518	0.351558	0.585664
LOC10192	-0.00413	0.351561	0.585664
LOC10012	0.005173	0.351613	0.585706
RP3-406C	-0.00702	0.351663	0.585744
RTP1	-0.01246	0.351727	0.585764
MYH10	0.232908	0.351729	0.585764
LOC49415	0.086145	0.351883	0.585976
CCDC24	0.011909	0.351919	0.585991
CERS3	-0.00357	0.35198	0.586037
DYRK3	0.048465	0.352001	0.586037
CBLN1	-0.00404	0.352101	0.586138
NOL4L	0.072121	0.352116	0.586138
KIAA1919	0.0544	0.352331	0.586451
MFAP4	0.167471	0.352404	0.586527
FLJ35816	-0.00259	0.352543	0.586714
RALGAPA2	0.131343	0.352727	0.586946
MPPED1	-0.01245	0.352737	0.586946
RP1-39J2.1	-0.00275	0.352786	0.586983
ZNF217	-0.14027	0.352926	0.58717
PIK3R4	-0.02686	0.353065	0.587357
ZNF107	-0.09292	0.353101	0.587371
LARP4	-0.07193	0.353183	0.587429
PMS2P1	-0.06616	0.353231	0.587429
KHDRBS3	0.119359	0.353245	0.587429
COX18	0.03039	0.353269	0.587429
CELF2-AS1	-0.01299	0.353271	0.587429
FBXW9	0.086496	0.353338	0.587496
F10	0.009423	0.353419	0.587538
CDC27	0.080116	0.353431	0.587538
CAMK2B	-0.00153	0.353446	0.587538
BNIP1	0.09716	0.353473	0.587538
GTPBP6	0.075677	0.35362	0.587738
ZBTB46	0.043156	0.353697	0.587821
CBLL1	-0.14368	0.353836	0.588008
PRO1804	-0.04279	0.353967	0.588179
ST7-OT4	-0.00223	0.354103	0.58836
AGXT	-0.00223	0.354197	0.588471
KIRREL3	-0.01025	0.354603	0.589101
GLOD4	-0.06803	0.354673	0.589172
AP1M2	0.001034	0.354738	0.589235
HPCA	-0.02452	0.354765	0.589235
TMEM231	-0.13119	0.354839	0.589312
TAS2R38	-0.01486	0.354874	0.589326
PRAMEF12	-0.02752	0.35496	0.589423
ZNF474	-0.01853	0.355004	0.58945
PLLP	0.008819	0.355106	0.589575
AK098263	0.014067	0.355172	0.589594
RGS1	0.292619	0.355198	0.589594
REXO1L1P	-0.00257	0.355199	0.589594
FBXL3	-0.04444	0.355231	0.589602
RP11-315F	0.016404	0.355324	0.589642

RP11-1109	-0.00175	0.35533	0.589642
ALDH6A1	0.087194	0.355337	0.589642
USP10	0.089315	0.355385	0.589663
CDR2L	-0.03551	0.355404	0.589663
CHN1	0.079215	0.355475	0.589735
TBCC	0.072849	0.355526	0.589746
GAL3ST4	-0.04609	0.355557	0.589746
SPN	-0.01467	0.355563	0.589746
CCNE2	0.094538	0.355674	0.589885
CYP4A11	-0.01465	0.355811	0.590066
HOXA11	0.001274	0.355889	0.590151
ZHX2	0.092408	0.356022	0.590268
ATP5B	0.062984	0.356052	0.590268
OCM2	0.005162	0.356123	0.590268
TXNDC9	-0.08504	0.356134	0.590268
LOC101928	-0.00293	0.356139	0.590268
ODAM	-0.00513	0.356173	0.590268
MAPRE1	0.070643	0.356177	0.590268
CHDC2	-0.01169	0.356202	0.590268
HIST1H2AE	-0.21248	0.356205	0.590268
LOC101928	-0.19826	0.356256	0.590307
EXOC3L1	0.006919	0.35644	0.590568
LOC101929	-0.01976	0.356663	0.590849
PIK3C3	-0.08581	0.356665	0.590849
C22orf31	-0.02569	0.356716	0.590889
LINC00702	-0.00681	0.356789	0.590965
LOC101060	0.013931	0.356905	0.591111
C1orf198	0.139726	0.357176	0.591398
TBC1D15	-0.06151	0.357208	0.591398
C6orf25	-0.00514	0.357228	0.591398
ENDOG	0.133063	0.357235	0.591398
HNRNPK	0.051497	0.357248	0.591398
RP6-149D1	-0.00515	0.357254	0.591398
CBLN2	-0.05705	0.357269	0.591398
RNF165	-0.03073	0.35736	0.591472
USP9X	-0.05917	0.357369	0.591472
H2AFY2	-0.17035	0.357422	0.591514
SYNPO	-0.03871	0.357539	0.591646
OR10A3	0.006137	0.357556	0.591646
MC2R	-0.0011	0.357641	0.591742
FAM133A	0.156384	0.357719	0.591747
UCHL1	0.152809	0.357739	0.591747
AJUBA	-0.03394	0.35774	0.591747
TUBG2	-0.06657	0.357784	0.591747
H2AFV	0.079658	0.357807	0.591747
LOC100500	-0.02163	0.357808	0.591747
INO80C	0.069842	0.358019	0.592025
11-Mar	0.018814	0.358031	0.592025
AC002059.	0.029133	0.358141	0.592162
GPC6	-0.14244	0.358302	0.592383
STXBP5-AS	-0.00181	0.358344	0.592407
GCSAML-A	-0.2444	0.358511	0.592623
FAM60A	0.090105	0.358562	0.592623
IKZF3	0.03528	0.35858	0.592623
LRG1	0.145276	0.358584	0.592623
SYT2	0.002575	0.358679	0.592734
CDX1	-0.01499	0.358829	0.592881
LOC101928	-0.02449	0.358857	0.592881

CAPZA1	-0.06157	0.35887	0.592881
LINC00514	-0.00782	0.3589	0.592881
OR3A3	-0.01194	0.358915	0.592881
KRT82	-0.02643	0.358935	0.592881
ST8SIA2	0.003728	0.359	0.592881
PLEKHM3	-0.07199	0.359003	0.592881
LOC10192[illegible]	-0.08784	0.359014	0.592881
TSPYL1	-0.06531	0.359347	0.593386
LOC28466[illegible]	-0.00667	0.359422	0.593464
TTC23L	-0.01087	0.359715	0.593886
TEX35	-0.02083	0.359733	0.593886
AK055981	-0.12277	0.359794	0.593942
FAM118B	0.107655	0.359885	0.594048
PCDH15	-0.00271	0.360004	0.594198
TLDC2	-0.00987	0.36007	0.594226
ARMC6	0.082623	0.360088	0.594226
NR1I3	-0.00254	0.360103	0.594226
KIFC2	-0.02341	0.36021	0.594315
TBX4	-0.01378	0.360212	0.594315
CCDC113	-0.16586	0.360265	0.594357
TTTY5	-0.02643	0.360334	0.594426
METTL15	0.005812	0.360418	0.59452
ATF3	0.099227	0.360643	0.594845
ASB5	-0.00143	0.360707	0.594906
PTCHD1	-0.001	0.360799	0.594973
LINC01243	-0.00166	0.360804	0.594973
STAC	-0.18511	0.3609	0.594973
C12orf43	0.083356	0.360931	0.594973
LOC10012[illegible]	-0.00154	0.360961	0.594973
TSSK4	-0.01789	0.360966	0.594973
ZDHHC11	-0.07096	0.360969	0.594973
FCF1	-0.06875	0.360989	0.594973
MYO3B	-0.00566	0.360995	0.594973
SLC2A12	-0.01398	0.361034	0.594991
AC017104.[illegible]	-0.02346	0.361079	0.595021
FGF1	9.36E-04	0.361119	0.595041
MTHFD1	0.080162	0.361211	0.595126
MUM1L1	0.00733	0.361225	0.595126
CTA-384D	0.012342	0.361318	0.595233
PDE6C	-0.00241	0.361431	0.595375
FAM228B	0.070719	0.361462	0.59538
RP11-327J	0.001036	0.361703	0.595731
CASC17	-0.00178	0.361824	0.595862
TRH	-0.17658	0.361852	0.595862
APOO	0.106296	0.361922	0.595862
MRGPRX3	-0.00781	0.36194	0.595862
SLC25A2	0.008977	0.361944	0.595862
RPP40	-0.08413	0.361947	0.595862
AF086184	-0.01624	0.361995	0.595874
AMPD1	0.111957	0.362009	0.595874
CCDC155	-0.01309	0.362063	0.595917
C9orf3	0.084062	0.362208	0.59608
TEX30	-0.08187	0.362223	0.59608
CLMP	0.026714	0.362245	0.59608
PHLDA3	-0.01734	0.362344	0.596198
ARHGEF26	0.058483	0.362418	0.596274
LINC01144	0.004869	0.362486	0.59634
AX747405	-0.03408	0.362622	0.596518

ADAM18	-0.00253	0.36299	0.59708
MTR	-0.06512	0.363018	0.59708
MFSD7	0.023084	0.363047	0.597083
PLGLB2	0.005757	0.363119	0.597141
RCCD1	0.054837	0.363138	0.597141
CASC1	0.033902	0.36336	0.597461
CALML3	0.004942	0.363408	0.597494
WDR7	0.042473	0.363762	0.598032
MAK16	-0.08547	0.363825	0.598048
KDM4D	-0.01023	0.363827	0.598048
FZD10	-0.00139	0.363902	0.598125
TECRL	-0.00642	0.363982	0.598211
B4GALT5	0.103037	0.364103	0.598365
C1orf228	-0.04248	0.364242	0.598547
KIF17	0.050679	0.364287	0.598576
ALPI	0.003208	0.364324	0.598591
MSH4	0.001186	0.364404	0.598655
LEPREL2	0.025935	0.364418	0.598655
PLOD3	0.085921	0.364479	0.598666
LOC10050(	-0.10636	0.364507	0.598666
C10orf10	0.138335	0.364507	0.598666
WT1-AS	-0.12183	0.364653	0.598859
MSI1	-0.02887	0.364685	0.598867
FA2H	-0.0103	0.364762	0.598948
ZNF414	-0.01033	0.364829	0.599012
UCHL3	0.075694	0.364998	0.599244
MCM8	-0.08663	0.365039	0.599266
KLRD1	0.016391	0.365107	0.599332
RBBP8	0.091425	0.365167	0.599341
PGM3	0.095997	0.365168	0.599341
OSTM1-AS	-0.00358	0.365231	0.599377
RFK	-0.08227	0.365245	0.599377
RP11-182J	-0.00285	0.365273	0.599377
LINC00927	-0.0072	0.365385	0.599516
LOC65484:	0.002488	0.365499	0.599648
CCDC3	0.15811	0.365521	0.599648
PCA3	-0.00778	0.365598	0.599729
ABTB2	0.058962	0.365656	0.599779
COL18A1-.	-0.00294	0.365712	0.599803
MAGEC1	9.31E-04	0.365726	0.599803
SMAD1-AS	0.006562	0.365774	0.599836
MDH2	0.07194	0.365866	0.59988
PCDHGA1(	-0.00836	0.36587	0.59988
SMARCA5	0.059605	0.365884	0.59988
RASGRF2	0.024196	0.365944	0.599933
LGALS14	-0.00883	0.366013	0.599939
DPCR1	-0.00495	0.366052	0.599939
F11	-0.00296	0.366076	0.599939
ARHGEF15	-0.02187	0.366083	0.599939
LOC10192!	0.002727	0.366086	0.599939
CCDC110	-0.0048	0.366254	0.60017
DCAF12L1	-0.0506	0.366312	0.600218
RP11-764E	-0.0077	0.366612	0.600665
DRD5	0.004128	0.366715	0.600787
GRIK1-AS1	-0.00761	0.36677	0.600832
FAM46B	-0.00461	0.366829	0.600883
CCDC148	0.06306	0.366884	0.600898
CDH16	0.001018	0.366963	0.600898

C15orf43	-0.00375	0.366986	0.600898
BTG4	-0.00197	0.366996	0.600898
ZNF514	0.154009	0.367056	0.600898
FAM47C	0.015359	0.367057	0.600898
LOC10192	-0.01913	0.36706	0.600898
REM2	0.055474	0.36706	0.600898
SLC6A9	0.002909	0.367105	0.600927
FAH	0.050316	0.367233	0.601048
MED27	-0.08377	0.367234	0.601048
LOC10013	0.046679	0.367312	0.601123
LOC28574	-0.0033	0.367341	0.601123
NAT2	-0.0079	0.367364	0.601123
ADAM20	-0.01386	0.367437	0.601164
PHEX	-0.0273	0.367444	0.601164
CMYA5	-0.01628	0.367704	0.601516
LOC44146	0.049658	0.367737	0.601516
TRHR	9.22E-04	0.367764	0.601516
CCDC112	-0.10783	0.367777	0.601516
ABCG1	-0.14518	0.367822	0.601516
ERBB2	-0.00755	0.367826	0.601516
KRBOX1	0.104623	0.367913	0.601576
UPF3A	-0.05474	0.367918	0.601576
RP11-573	0.001511	0.367987	0.601643
LINC0070	-0.00599	0.368099	0.601745
MIR124-2	8.66E-04	0.368105	0.601745
LOC44017	-0.02731	0.368294	0.602
DPAGT1	-0.09536	0.368317	0.602
TUSC7	-0.00892	0.368448	0.602169
TCAP	-0.05005	0.368568	0.60232
LINC00466	-0.00553	0.3686	0.602327
SIGLEC8	0.005571	0.368675	0.602404
LOC10272	-0.03057	0.368803	0.602532
LRGUK	0.010646	0.368864	0.602532
PAPPA2	-0.01661	0.368876	0.602532
NOSIP	-0.079	0.368888	0.602532
CADM3-A	-0.00129	0.368909	0.602532
CLIC6	-0.11259	0.36892	0.602532
LGR6	-0.05879	0.369031	0.602647
USH1G	0.016378	0.369047	0.602647
AHRR	-0.15178	0.369074	0.602647
SAMD10	-0.02203	0.369114	0.602666
PENK	0.011425	0.369215	0.602786
AL832163	-0.0244	0.369321	0.602892
ATP2B4	-0.11294	0.369336	0.602892
ZDHHC19	0.008183	0.369571	0.603224
TAF6L	-0.0161	0.369595	0.603224
DNM1P41	-0.02544	0.3697	0.603333
REC8	0.070942	0.369717	0.603333
PRDM13	-0.00487	0.369783	0.60338
MYEOV	0.024599	0.369842	0.60338
VDAC2	0.055013	0.369854	0.60338
ENGASE	-0.1062	0.369858	0.60338
ARGLU1	0.099889	0.369886	0.60338
CDRT15	-0.0327	0.369916	0.603385
KCNA6	0.003879	0.370012	0.603433
RFTN2	0.011554	0.370071	0.603433
MS4A6E	0.01034	0.370073	0.603433
INTS4L1	-0.00951	0.370089	0.603433

AL133493.:	-0.32943	0.37011	0.603433
H1FNT	-0.00119	0.370143	0.603433
AGBL1	0.002863	0.37016	0.603433
FHL1	0.114722	0.370169	0.603433
SCN8A	-0.0048	0.370259	0.603493
TSSK6	-0.00389	0.370261	0.603493
LINC00856	0.009404	0.370299	0.603509
LOC10050!	-0.00795	0.370364	0.60357
PYROXD2	-0.04488	0.370544	0.603817
C9orf84	0.001957	0.370674	0.603949
WNT8A	0.012774	0.370695	0.603949
EPRS	0.076614	0.370708	0.603949
FITM1	-0.02499	0.370774	0.60401
TRMT1L	-0.08859	0.370876	0.604131
PNMAL1	-0.30867	0.370914	0.604148
EIF6	0.084721	0.370986	0.604185
CYP4F22	0.01149	0.370993	0.604185
LOC10192ł	-0.00157	0.371148	0.604393
CA5BP1	0.079133	0.371197	0.604427
FLJ31104	-0.01636	0.371292	0.604519
SPATC1	-0.02043	0.371317	0.604519
ZNF34	-0.08573	0.371365	0.604519
C2orf82	0.027071	0.371406	0.604519
PACRG-AS	-0.00545	0.371429	0.604519
CAMKK1	0.039818	0.371455	0.604519
FBXO5	0.110602	0.371456	0.604519
ZNF326	-0.08726	0.371477	0.604519
RP11-279(	-0.00614	0.371578	0.604637
CTB-78F1.:	-0.00252	0.371652	0.604714
IPP	0.048628	0.371688	0.604726
DEPDC7	0.180909	0.371921	0.60506
UBE2N	0.06794	0.371983	0.605116
LOC10192ł	-0.00498	0.372027	0.605141
LOC57399	-0.00676	0.372087	0.605194
RPL32	-0.03887	0.372199	0.605331
RP11-341N	0.012307	0.372258	0.605344
AP1S2	0.107951	0.372271	0.605344
AMIGO2	-0.2223	0.372292	0.605344
ATAT1	-0.05337	0.372552	0.605548
SULT2A1	-0.00274	0.372612	0.605548
LRRC18	-0.01149	0.372613	0.605548
RNF17	-0.00323	0.372621	0.605548
POLD3	-0.05382	0.372639	0.605548
LOC10192ł	-0.00751	0.372643	0.605548
TMEM106E	-0.10133	0.372645	0.605548
ARG1	-0.01057	0.372665	0.605548
RAB3GAP1	0.053371	0.372668	0.605548
PRAMEF11	0.016738	0.372716	0.605566
EEF2	0.069675	0.372744	0.605566
VGLL2	-0.02763	0.372765	0.605566
CHCHD4	-0.10583	0.3728	0.605566
SLC1A2	-0.00132	0.372819	0.605566
ASB15	0.005232	0.372904	0.605658
SLFN11	0.091936	0.373012	0.60578
SLC11A2	0.094073	0.373055	0.60578
DHX8	0.076391	0.373082	0.60578
RP11-157E	-0.00125	0.373099	0.60578
SYCP2L	-0.08853	0.373119	0.60578

PRRT3	-0.06521	0.373254	0.605954
KIF16B	0.087251	0.373433	0.606171
FLJ22763	0.003391	0.373453	0.606171
ZNF215	-0.06419	0.373472	0.606171
HPR	-0.00788	0.373717	0.606366
DCAF13	0.070631	0.373722	0.606366
RP4-742J2	-0.03296	0.373756	0.606366
LOC10192	-0.00868	0.37377	0.606366
FAM73A	0.083285	0.373784	0.606366
TMCO1	-0.04212	0.373786	0.606366
LLNLR-24	0.0136	0.373788	0.606366
LINC00312	0.017823	0.373905	0.606461
PSMD13	0.074833	0.373925	0.606461
PSORS1C2	-0.00337	0.373975	0.606461
GPR26	0.008778	0.373975	0.606461
LOC10019	-0.11592	0.373986	0.606461
CD101	-0.03208	0.374037	0.606497
JAG1	0.071821	0.374074	0.606512
SERPINA4	9.89E-04	0.374345	0.606906
SORCS3	-0.00151	0.374612	0.607293
PHPT1	0.090781	0.374658	0.607323
RP11-410	-0.00861	0.374909	0.607684
C3orf79	-0.00414	0.375036	0.607844
DPYSL2	-0.1573	0.375074	0.607857
PTPRG-AS	-0.03613	0.3751	0.607857
LOC10192	-0.00295	0.37514	0.607876
RASSF8-A	0.043227	0.375257	0.608004
TTC1	0.091064	0.375275	0.608004
RP11-164	-0.00689	0.375434	0.608182
RBM24	-0.00214	0.375441	0.608182
PKDREJ	-0.01159	0.375495	0.608224
FAM222B	0.073814	0.375538	0.608248
RASAL2	0.013069	0.375595	0.608295
MMP1	-0.11127	0.375672	0.608374
ZNF254	-0.10303	0.375769	0.608486
SCCPDH	0.055783	0.375811	0.608508
CST11	-0.00414	0.375891	0.608577
TEAD2	-0.0141	0.37591	0.608577
PRKCI	0.077905	0.375938	0.608577
SBNO2	0.028038	0.376004	0.608638
OR5I1	-0.00502	0.376057	0.608679
NKX2-3	0.244461	0.376102	0.608707
MED31	-0.13273	0.376208	0.608819
TIGIT	0.021642	0.376229	0.608819
TLX1	-0.0063	0.376261	0.608819
ATP6V1B1	-0.00828	0.376284	0.608819
AMELX	-0.00372	0.376397	0.608931
ATP5J2	0.029471	0.376424	0.608931
UBXN7	-0.08985	0.376451	0.608931
TARS	0.04659	0.376466	0.608931
NAT1	0.07772	0.376519	0.608955
SSR2	0.068343	0.376537	0.608955
9-Mar	-0.02974	0.376867	0.609443
SLC20A1	0.089413	0.376988	0.609593
C8B	-0.00201	0.377041	0.609621
NUDT12	-0.07589	0.377061	0.609621
LILRA4	-0.16396	0.377258	0.609839
IGKC	0.075917	0.377274	0.609839

IQCD	-0.02728	0.377281	0.609839
TCP11	-0.00624	0.377351	0.609906
HOGA1	-0.00236	0.377425	0.609982
RP11-305(	0.006708	0.377589	0.610201
PPP1R14C	-0.12208	0.377657	0.610229
NUDT9	-0.08152	0.377663	0.610229
DNAJC1	0.086097	0.377709	0.610258
MEGF6	0.112204	0.377745	0.61027
COMP	-0.0088	0.37784	0.610378
KATNB1	0.103	0.377913	0.61045
PPY	-0.00272	0.377952	0.610469
SCGB3A2	-0.02063	0.377988	0.61048
NCR3	-0.0044	0.378038	0.610516
ZFP92	-0.07859	0.378086	0.610533
RP11-319E	-0.04539	0.378105	0.610533
LINC01197	9.39E-04	0.378249	0.61072
LOC10192!	-0.00463	0.378302	0.610739
AFF1	0.061297	0.378357	0.610739
RP11-876N	-0.06206	0.378358	0.610739
HAO2	0.007401	0.378373	0.610739
SLC17A3	-9.97E-04	0.378443	0.610806
OR51I1	-0.00694	0.378515	0.610837
PNPLA1	-0.01392	0.378519	0.610837
KRTAP2-1	-0.00194	0.378629	0.61097
ZNF319	-0.03879	0.378665	0.610981
MAST4	-0.12146	0.37874	0.611058
RP11-274E	-0.0027	0.37892	0.611281
KIF1B	0.142342	0.378935	0.611281
HMHB1	0.17096	0.379083	0.611475
FSTL1	0.116898	0.379131	0.611506
NSUN5	0.083177	0.379218	0.611601
PRRC1	0.078779	0.379357	0.611643
TPSAB1	0.194288	0.379393	0.611643
LINC00551	0.002724	0.379397	0.611643
ESYT3	-0.00309	0.379411	0.611643
EFCAB2	-0.13138	0.379412	0.611643
XG	0.010847	0.379427	0.611643
LOC10192!	-0.00272	0.379442	0.611643
CHMP6	0.103094	0.379744	0.612084
LINC01398	8.72E-04	0.379888	0.612271
PNMT	-0.1545	0.379958	0.612338
PLD4	0.088358	0.380011	0.612378
MIR143HG	0.013932	0.38007	0.612404
LINC00310	-0.01064	0.380084	0.612404
LOC10050(	-0.01634	0.38012	0.612418
UQCRC1	0.105767	0.380224	0.612498
NAPEPLD	0.095526	0.380227	0.612498
B3GNTL1	-0.0693	0.380322	0.612606
BC047644	-0.00585	0.380437	0.612745
HGC6.3	-0.04873	0.380475	0.612761
ABCC6P1	0.027441	0.380509	0.61277
STEAP1	0.120146	0.380639	0.612922
COX6A2	-0.00325	0.38066	0.612922
ZNF503-A!	-0.00571	0.380726	0.612983
CDH7	0.05995	0.380923	0.613169
LINC00593	-0.00214	0.38094	0.613169
TOX4	-0.0791	0.380943	0.613169
KLHL5	-0.09211	0.380955	0.613169

NDUFA6	-0.07974	0.380985	0.613172
SLC17A1	9.67E-04	0.381013	0.613172
SYT15	0.014084	0.381095	0.613243
ARHGEF33	-0.01015	0.381114	0.613243
LY86-AS1	-0.01467	0.381211	0.613311
FAM126A	0.034266	0.381213	0.613311
DEFB129	0.002297	0.381386	0.613528
NSUN7	-0.0898	0.381404	0.613528
MTTP	-0.00758	0.381523	0.613602
CDK5RAP3	0.081901	0.381526	0.613602
TFF1	-0.02805	0.381536	0.613602
CFH	0.231428	0.38158	0.613602
ARHGAP33	-0.02583	0.381592	0.613602
ZPBP	-0.0039	0.381701	0.613705
WDFY2	0.103921	0.381738	0.613705
ANGPTL3	0.001135	0.381753	0.613705
SPATA25	0.018649	0.38177	0.613705
POFUT2	-0.01214	0.381827	0.613753
CST1	-0.00935	0.381896	0.613779
GOLPH3L	-0.07205	0.381901	0.613779
WBP2NL	-0.05026	0.382032	0.61389
PNPLA4	-0.0719	0.382035	0.61389
C10orf95	-0.05295	0.382055	0.61389
ACTR8	-0.06256	0.382176	0.614039
AGGF1	0.069358	0.382376	0.614315
USP24	0.075916	0.382461	0.614406
SIGLEC17P	-0.14198	0.382538	0.614485
KLF5	0.121534	0.382627	0.614554
NOS2	-0.00595	0.382639	0.614554
ADCY5	-0.00627	0.382698	0.614591
LOC10192	-0.01404	0.382718	0.614591
WNK4	-0.00361	0.382758	0.61461
DKFZp434I	0.004959	0.382876	0.614753
NIPAL3	0.084015	0.382927	0.614791
WHAMM	-0.07726	0.382973	0.614809
DEFB118	-0.02565	0.382995	0.614809
KLK3	-0.04429	0.38314	0.614994
SNORA68	0.052616	0.383315	0.615162
PLEKHG2	0.040904	0.383323	0.615162
DMRTA1	0.00132	0.383329	0.615162
PNLIPRP1	-0.00225	0.383396	0.61519
PIK3CA	0.078156	0.383431	0.61519
AGBL2	-0.05858	0.38344	0.61519
PCDHAC1	0.002181	0.383475	0.61519
RARRES1	0.08544	0.383489	0.61519
CIB3	-0.02514	0.383579	0.615246
SLC24A2	-0.00207	0.383581	0.615246
FZD2	0.098606	0.38365	0.61529
LPHN3	-0.09106	0.383665	0.61529
AC007389.	-0.05223	0.383703	0.615305
SERGEF	0.095208	0.383742	0.615309
ACSS1	-0.01214	0.383782	0.615309
FGF18	-0.00824	0.38379	0.615309
FANCD2	0.038992	0.383831	0.615328
RNF115	0.048877	0.383965	0.615498
TRPV5	-0.00905	0.383993	0.615498
AC079305.	-0.11708	0.3842	0.615784
MMAA	-0.0704	0.384268	0.615847

IP6K3	0.027728	0.384305	0.615857
VSIG2	-0.0276	0.384331	0.615857
3-Sep	-0.02673	0.384454	0.616008
C17orf58	0.137489	0.384511	0.616047
LOC10192[illegible]	-0.00382	0.384535	0.616047
LINC01095	-0.00167	0.38463	0.616153
SLC38A4	9.50E-04	0.384666	0.616166
CRISP2	0.117119	0.384806	0.616337
TTC21A	-0.01857	0.38483	0.616337
FAM181A	-0.00123	0.384899	0.616367
AOX1	-0.08845	0.384906	0.616367
SEC14L3	-0.00101	0.385188	0.616773
RP11-257I[illegible]	-0.00178	0.385233	0.6168
SLC19A1	-0.00704	0.385301	0.616851
ITGBL1	0.066101	0.385322	0.616851
CRTC1	0.026677	0.385403	0.616935
SPACA1	-0.03118	0.385538	0.617106
LINC00334	0.001869	0.385629	0.617205
PPM1D	0.072641	0.385688	0.617255
TMEM158	-0.18917	0.386025	0.617748
LINC01222	-0.02557	0.386086	0.6178
FAM129A	0.116851	0.386134	0.617802
ZNF174	0.050225	0.386152	0.617802
SLC13A5	-0.01123	0.386176	0.617802
LOC10050[illegible]	-0.03485	0.386201	0.617802
LINC00894	-0.02334	0.386294	0.617906
GLIS1	-0.01179	0.386464	0.618131
RP11-161I[illegible]	-0.03049	0.38659	0.618287
CCDC66	-0.05157	0.386713	0.618438
PLEKHF1	-0.04398	0.3868	0.618483
APOBEC4	-0.00672	0.386825	0.618483
SPARCL1	0.182197	0.386827	0.618483
LOC10050[illegible]	0.014239	0.38705	0.618794
KALRN	-0.0081	0.387085	0.618804
OR3A1	0.015824	0.387238	0.618854
LOC10013[illegible]	-0.01153	0.387253	0.618854
mir-223	-0.18776	0.387281	0.618854
PLIN4	-0.04374	0.38731	0.618854
PACRG	0.014338	0.387314	0.618854
LOC10192[illegible]	-0.11688	0.387329	0.618854
MATN1-A[illegible]	-0.06535	0.387364	0.618854
GRM8	-0.0082	0.387382	0.618854
ARSE	0.035754	0.387428	0.618854
ADPRH	0.026893	0.387428	0.618854
DUOX2	0.013839	0.38743	0.618854
LOC10192[illegible]	0.004017	0.387631	0.619128
CCT7	-0.06835	0.387759	0.619287
KISS1R	-0.04561	0.387845	0.61935
IGHD	0.095336	0.387855	0.61935
GABRB1	0.013028	0.387902	0.619379
MUCL1	-0.00945	0.387938	0.61939
ISPD-AS1	-0.00244	0.387996	0.619399
LOC10050[illegible]	-9.99E-04	0.38801	0.619399
LOC38894[illegible]	-0.00634	0.388055	0.619399
LOC10192[illegible]	9.78E-04	0.388057	0.619399
FBXO32	-0.02984	0.388114	0.619444
RP11-329E[illegible]	-0.02299	0.388151	0.619456
DFNB31	-0.00431	0.388285	0.619625

RP11-123K	-0.02263	0.388337	0.619662
IFNA8	-0.0018	0.388393	0.619706
RBAKDN	-0.04377	0.388464	0.619774
MIR4500H	-0.0017	0.388549	0.619863
THAP10	-0.10089	0.388658	0.619992
HCCAT5	-0.00359	0.388701	0.620015
LLPH	-0.14719	0.388928	0.620331
ACSBG1	-0.08085	0.388975	0.620335
MDM4	-0.07967	0.388988	0.620335
ACOX3	0.079474	0.389098	0.62045
LOC10192	0.005856	0.389136	0.62045
REV1	-0.05671	0.389146	0.62045
KLHDC8B	0.158751	0.389217	0.620518
TMEM38A	-0.01169	0.389388	0.620744
RP4-781K5	-0.04155	0.389491	0.620843
TMEM220	0.08459	0.389507	0.620843
RBM47	-0.02575	0.389594	0.620936
PCSK1N	-0.03531	0.38964	0.620964
PEX13	-0.07799	0.389738	0.621047
GFER	0.041381	0.389757	0.621047
TACR1	0.003757	0.389778	0.621047
OXTR	-0.0938	0.389865	0.621094
ERG	0.117424	0.389867	0.621094
LOC10028	7.54E-04	0.389917	0.621094
MYBPC1	-0.00947	0.38998	0.621094
CSPG4P1Y	0.015444	0.389987	0.621094
LOC10192	-0.00774	0.389987	0.621094
RP11-167H	-0.01095	0.390009	0.621094
GMPR2	-0.09453	0.390088	0.621154
PGM2L1	0.065733	0.390104	0.621154
DSCR8	-0.01277	0.390211	0.621279
GPR107	0.054331	0.390277	0.621298
AIF1L	-0.11042	0.39028	0.621298
ZNF300	-0.23507	0.390474	0.62147
MTUS2	-0.04628	0.390481	0.62147
TSHZ2	-0.00113	0.390489	0.62147
COL18A1	0.101955	0.390503	0.62147
PTPRJ	-0.0027	0.390563	0.621519
CCDC136	-0.06204	0.390681	0.621661
CTD-2194I	0.003098	0.390822	0.621803
LOC28378	-0.08248	0.390827	0.621803
UBE2V2	-0.10283	0.391025	0.622072
PCDHB11	-0.00333	0.391114	0.622167
SPRR3	-0.00964	0.391144	0.62217
PLEC	0.015183	0.391319	0.622403
DISC1	-0.00309	0.391368	0.622434
CDX2	-0.02002	0.391401	0.622442
FIGNL1	-0.10222	0.391535	0.62261
SUN5	-0.00999	0.3916	0.622667
RP11-347D	-0.00119	0.39164	0.622684
APOBEC3D	-0.01896	0.391737	0.622793
ZNF628	0.112935	0.391826	0.622883
MRS2	0.077223	0.391851	0.622883
CCNB1	-0.1366	0.391967	0.623021
AGMAT	0.068144	0.391997	0.623024
GPRC5B	0.011643	0.392088	0.623054
LOC10272	0.100897	0.392108	0.623054
ERVMER34	0.027471	0.392135	0.623054

RPS27L	0.10888	0.392157	0.623054
MAP6D1	-0.05778	0.39216	0.623054
SUCLG1	0.031234	0.392191	0.623058
SIM1	-0.01806	0.392247	0.6231
LOC10050(	0.127862	0.392297	0.623118
TIMM23	-0.00252	0.392315	0.623118
PIK3R2	-0.01659	0.392564	0.623467
NCR1	0.009539	0.392623	0.623515
LOC64409(	-0.00858	0.392783	0.623689
SEMA4C	-0.08483	0.39279	0.623689
PRSS23	0.05907	0.392885	0.623794
CD3D	0.139486	0.39302	0.623962
ZC3H4	0.026507	0.393146	0.624105
PPP1R3F	-0.0181	0.393168	0.624105
CALCOCO.	0.091534	0.393201	0.624112
RP11-1072	0.021788	0.393233	0.624118
FOXS1	-0.00518	0.393289	0.624161
FHL3	-0.06273	0.39337	0.624244
XKR7	-0.00242	0.393493	0.624393
SVIP	-0.08161	0.393617	0.624544
AQPEP	0.002161	0.393653	0.624555
SLITRK5	-0.20993	0.393721	0.624593
RAPGEF2	-0.13607	0.393734	0.624593
COL6A1	-0.00104	0.393787	0.624631
UBE2DNL	-0.00353	0.393963	0.624864
LOC10192!	-0.00346	0.394058	0.62497
LINC00905	-0.00694	0.394088	0.624971
PRMT9	-0.07638	0.394159	0.624998
KCNV2	0.002511	0.394179	0.624998
AC007362.	8.97E-04	0.394192	0.624998
LOC28581:	-0.12655	0.394339	0.625112
TMEM262	0.0178	0.39435	0.625112
C5orf47	-0.0019	0.39435	0.625112
C9orf131	-0.02002	0.394435	0.6252
PDE6H	0.004622	0.394521	0.625236
MPP7	0.062104	0.394541	0.625236
TANC1	-0.04132	0.394544	0.625236
APOBEC1	-0.0152	0.394641	0.625343
BOK	0.032759	0.394692	0.625378
HECW1	-0.00837	0.394899	0.62566
TYW5	0.071144	0.394969	0.62571
IL2RA	-0.09072	0.394987	0.62571
VSX1	0.001163	0.395356	0.626248
MEF2C	-0.09848	0.395511	0.62641
SPRR1B	-0.00207	0.395534	0.62641
MAP3K10	-0.026	0.395545	0.62641
IFT57	0.06579	0.395624	0.626452
LOC10192!	0.00215	0.395629	0.626452
PALD1	0.145786	0.39585	0.626692
CCR9	-0.04633	0.395888	0.626692
LOC10192!	-0.00416	0.395895	0.626692
LOC10192!	0.004092	0.395897	0.626692
POLR2J4	0.085665	0.396077	0.626887
GRIA1	-0.01317	0.396131	0.626887
ABCA4	-0.0043	0.396145	0.626887
GLIPR1	-0.1664	0.396155	0.626887
MAPKAPK:	0.06697	0.396165	0.626887
LINC00621	-0.05041	0.396252	0.626927

FIRRE	-0.10599	0.396264	0.626927
LINC00115	-0.09576	0.396277	0.626927
RBM5	-0.08379	0.396368	0.627026
LOC34058:	0.001663	0.396486	0.627166
NIM1K	-0.01239	0.396522	0.627178
PITX3	-0.03617	0.396575	0.627216
ISCA2	0.087776	0.396654	0.627294
RBBP8NL	-0.02626	0.396698	0.627319
ZBTB1	0.059292	0.396806	0.627443
FAM154B	-0.0967	0.39686	0.627482
RAB38	-0.16283	0.396933	0.627553
EIF2S1	0.076237	0.397012	0.627595
PRKAA2	-0.0368	0.397018	0.627595
STX18	0.076517	0.397059	0.627613
HEMGN	0.176828	0.397127	0.627657
HLA-DOB	0.084554	0.397144	0.627657
LINC01153	-0.00113	0.397423	0.628021
RP11-304L	-0.00148	0.397432	0.628021
RP11-410L	0.075847	0.397488	0.628034
BRINP3	-0.00573	0.397499	0.628034
CENPC	-0.08325	0.397592	0.628136
DTL	-0.11914	0.397633	0.628154
CBS	0.01979	0.39768	0.628184
MGC45800	-0.0156	0.397781	0.628258
RALBP1	0.042568	0.397793	0.628258
JPH1	0.065205	0.397815	0.628258
PPP1R2	-0.07253	0.397849	0.628264
MICAL1	0.147693	0.39793	0.628264
STXBP6	-0.11958	0.397955	0.628264
NLGN4Y	0.018558	0.397957	0.628264
FRMD4B	-0.01608	0.397976	0.628264
SHISA3	-0.02765	0.398028	0.628264
NFKBIE	0.13076	0.398042	0.628264
PTER	0.092995	0.398068	0.628264
RPL18	-0.04513	0.398114	0.628264
GRM3	0.006283	0.398116	0.628264
TRIM71	-0.20538	0.398138	0.628264
B4GALNT2	0.026089	0.398182	0.628288
AX746699	-0.01291	0.398341	0.628491
PIK3IP1	0.069445	0.398368	0.628491
KIAA1211	-0.16937	0.398546	0.628715
GAS5-AS1	-0.01613	0.398577	0.628715
TSEN2	0.054266	0.398597	0.628715
LOC10013:	-0.02476	0.398754	0.628915
RFT1	0.062416	0.398812	0.628962
WDR36	0.065175	0.398844	0.628966
HES4	-0.04461	0.398899	0.628976
ANHX	-0.01428	0.398908	0.628976
FAM71C	-0.00142	0.399012	0.629094
ROPN1	-0.00319	0.399068	0.629128
KCNV1	0.004693	0.399092	0.629128
UBE2Q2L	0.05949	0.399121	0.629129
KCNJ11	-0.00531	0.399256	0.629295
UBR2	0.047869	0.399287	0.629295
PWWP2A	0.087919	0.399314	0.629295
MIR31HG	0.002095	0.399412	0.629404
PTBP2	0.07647	0.39949	0.629481
LOC15840:	0.121316	0.399545	0.629522

LPCAT3	0.107901	0.399819	0.629907
LYPLAL1	0.104077	0.399882	0.629961
SLC19A3	0.002499	0.39999	0.630078
MAP2K6	-0.04575	0.400014	0.630078
RP11-1081	7.23E-04	0.400062	0.630099
TSPAN12	0.02032	0.400086	0.630099
PRO2949	-0.00236	0.400245	0.630304
STH	-0.00914	0.400383	0.630476
LOC10192	-0.01355	0.40047	0.630567
TUBAL3	0.141202	0.400605	0.630733
LOC28456	0.04665	0.400672	0.630738
MIAT	0.106454	0.400693	0.630738
LOC10050	0.073796	0.400696	0.630738
CERS4	0.049116	0.400791	0.630842
OR10C1	-8.50E-04	0.400889	0.630951
ZNF318	0.087037	0.40099	0.631032
ZNF366	-0.01026	0.400999	0.631032
LCN8	0.005787	0.401052	0.631069
FGF10-AS	0.001119	0.401128	0.631144
APH1B	0.062772	0.40123	0.631258
PDE5A	-0.04437	0.401267	0.631271
LOC28638	-0.00991	0.401342	0.631342
BSND	-0.0053	0.401397	0.631384
OR51B2	-0.01282	0.401429	0.631387
PROM1	-0.21118	0.401568	0.631561
RIPPLY1	0.014729	0.401804	0.631842
COG1	-0.02047	0.401829	0.631842
LOC10106	8.05E-04	0.401918	0.631842
MELK	-0.13242	0.401919	0.631842
TMEM170	0.162232	0.401921	0.631842
FREM1	-0.23956	0.401922	0.631842
ZNF204P	-0.16821	0.402069	0.632014
BBS2	-0.08231	0.402124	0.632014
ZNF711	-0.15058	0.402137	0.632014
LOC38878	0.029098	0.402154	0.632014
RASA1	0.088474	0.402177	0.632014
RNF220	-0.06953	0.402247	0.632077
TARBP1	-0.08653	0.40234	0.632177
RP11-284	0.001928	0.402504	0.632389
LINC01362	0.006032	0.402546	0.632409
ECHDC2	0.163528	0.402612	0.632451
SHANK2	-0.07156	0.402659	0.632451
TMEM121	0.060189	0.40266	0.632451
GDPGP1	-0.03687	0.402703	0.632473
SBSN	0.001547	0.402828	0.632623
FAM196A	0.006772	0.403039	0.632865
GPR101	0.001489	0.403041	0.632865
LOC10192	0.008886	0.403094	0.632904
LINC0130	-0.01772	0.4032	0.633003
BTNL3	-0.06271	0.403221	0.633003
SLAMF9	0.010695	0.403245	0.633003
WFDC13	0.00472	0.403332	0.633093
MYH4	0.024247	0.403367	0.633102
MIR205	-0.00969	0.403411	0.633126
TDRD9	0.218211	0.403696	0.633527
C1orf145	0.007945	0.403729	0.633532
FAM201A	-0.0994	0.403951	0.633815
LINC0056	-0.00307	0.403967	0.633815

MYADML2	-0.00931	0.404018	0.633849
YARS2	-0.05362	0.404075	0.63387
RAB3GAP2	-0.02736	0.404133	0.63387
RBL1	0.048326	0.404139	0.63387
GBA3	0.018181	0.404169	0.63387
ZNF497	-0.00731	0.404178	0.63387
TIMELESS	-0.05828	0.40422	0.63389
EVI5L	0.00517	0.404261	0.633908
DDAH2	0.151674	0.404297	0.633919
CEP83-AS1	-0.01433	0.404434	0.634022
AGR2	-0.05175	0.404446	0.634022
LOC28368:	0.014115	0.404451	0.634022
HOXA5	-0.1676	0.404554	0.634097
RP11-29P2	0.019257	0.404557	0.634097
SH2D1B	0.015758	0.404603	0.634123
SPRY4	-0.00638	0.404639	0.634134
LURAP1	0.030959	0.404812	0.634359
LAPTM4A	0.056188	0.40492	0.634482
LOC10192	-0.01148	0.405105	0.634704
LPP-AS2	0.005298	0.40512	0.634704
PARP2	-0.06147	0.405163	0.634726
RP11-504A	-0.00228	0.40522	0.634768
ELAVL2	0.005603	0.405265	0.634794
FAM64A	-0.15011	0.405406	0.634968
HMGCLL1	9.43E-04	0.405512	0.635042
CFLAR-AS1	-0.00953	0.405518	0.635042
AX747630	-0.00465	0.405541	0.635042
FBXO33	-0.07848	0.405616	0.635113
LOC33956	-0.01039	0.405716	0.635199
KANK4	-0.03926	0.40573	0.635199
ARID2	-0.07729	0.4059	0.63542
IGF2BP3	-0.08975	0.406018	0.635552
MUC5AC	0.004344	0.406064	0.635552
GPR20	-0.00697	0.406073	0.635552
FBN2	-0.04507	0.406112	0.635555
LIG1	0.107484	0.406133	0.635555
TFCP2	0.073826	0.406178	0.635579
RAD9B	-0.03896	0.406292	0.635712
ZNF805	0.075576	0.406403	0.63584
TRIM69	0.020845	0.406525	0.635984
FAM9A	-0.01734	0.406557	0.635988
CCDC184	-0.00367	0.406595	0.635991
RP4-758J2	-0.08777	0.406655	0.635991
LOC28617	-0.00862	0.4067	0.635991
LOC43993	-0.0457	0.406736	0.635991
WNT4	-0.00369	0.406776	0.635991
OTUB2	-0.00611	0.406787	0.635991
RP11-307F	-0.01382	0.406812	0.635991
C9orf50	-0.02158	0.406825	0.635991
SLC39A6	0.06669	0.406844	0.635991
ZNF620	0.020872	0.406869	0.635991
HIST1H1A	-0.03786	0.406882	0.635991
FZD6	-0.18745	0.406948	0.636049
LRRC73	-0.01624	0.407014	0.636107
PPP3CB	-0.05429	0.407063	0.636136
DDX11-AS	-0.02005	0.407145	0.636218
HDHD1	0.11927	0.407182	0.636224
KRT33A	-0.0019	0.407207	0.636224

CTD-2350J	-0.00254	0.407292	0.636304
LHFPL5	-0.0018	0.407317	0.636304
ZFP37	-0.03966	0.407354	0.636316
CECR7	-0.01719	0.407489	0.636481
NUDT19	0.112102	0.407664	0.636695
SLC6A11	-0.02106	0.407685	0.636695
GREB1L	-0.00834	0.407729	0.636718
RP11-44N1	-0.0151	0.407912	0.636945
CYP2C9	-0.05617	0.407933	0.636945
CTC-471C1	-0.00203	0.407987	0.636952
TRAPPC9	-0.02556	0.408	0.636952
LOC10192	0.001471	0.408025	0.636952
TMEM234	-0.04519	0.408217	0.637204
BCL11B	0.067509	0.408506	0.637596
RPA4	-0.12027	0.408557	0.637596
OR52A1	-0.00317	0.408568	0.637596
GS1-259H1	0.045207	0.408596	0.637596
GAB4	0.004106	0.408615	0.637596
E2F7	-0.0909	0.408762	0.637779
KLHL31	-0.02316	0.408866	0.637882
LRR1	0.057327	0.408886	0.637882
LOC10192	-0.00441	0.409012	0.638032
SCAP	0.066382	0.409045	0.638038
KDM5C	0.056624	0.409092	0.638065
DCST1	-0.0015	0.409157	0.638077
LOC10192	-0.00175	0.409159	0.638077
GTPBP10	-9.94E-04	0.40932	0.638265
KLK15	0.012503	0.409347	0.638265
TAB1	0.048096	0.409368	0.638265
PARVA	0.006213	0.409437	0.638327
AP3B2	-0.01404	0.40955	0.638428
RP11-2E11	-0.12546	0.409561	0.638428
TBRG4	0.063863	0.40966	0.638536
LOC64690	-0.00479	0.40991	0.638857
OPRM1	-0.00197	0.409959	0.638857
ACE	0.013199	0.409965	0.638857
FAM71E1	-0.02167	0.409988	0.638857
RP11-356E	-0.02301	0.410029	0.638857
PSMA8	-0.11333	0.410043	0.638857
POLI	-0.09295	0.410077	0.638865
HAPLN4	8.65E-04	0.410155	0.63892
KIAA1958	0.094324	0.410172	0.63892
OBSL1	-0.04891	0.410312	0.639093
RP11-498E	-0.07378	0.410438	0.63919
LINC00911	-0.00878	0.410438	0.63919
NUDC	0.081017	0.410463	0.63919
FAM47B	0.00928	0.411232	0.640289
CDKN2D	0.053349	0.411238	0.640289
COL5A1	-0.18162	0.411257	0.640289
ZNF713	0.044051	0.411429	0.640511
TEX14	0.05647	0.411521	0.640607
FLJ41170	-0.00598	0.411606	0.640694
C1orf123	-0.06051	0.411749	0.640871
PDZD7	0.013252	0.411847	0.640977
ITGA4	0.091621	0.41192	0.641009
LOC10272	0.211155	0.411927	0.641009
TTBK2	-0.0568	0.41219	0.641336
RP5-963E2	0.001243	0.412202	0.641336

RP3-497J2	-0.00786	0.412238	0.641336
RASAL3	-0.08612	0.412256	0.641336
RP11-254F	0.002088	0.412471	0.641571
ANO5	-0.17154	0.412485	0.641571
CREB5	0.163992	0.412496	0.641571
CXorf58	-0.00638	0.412532	0.641581
RP11-250E	0.107464	0.412604	0.641647
BROX	-0.10029	0.412665	0.641696
PATL2	-0.06788	0.412763	0.641803
LINC00884	0.041782	0.412888	0.641943
LGALS4	0.01651	0.41293	0.641943
SLCO5A1	-0.15302	0.412981	0.641943
NAPA-AS1	0.019752	0.413024	0.641943
CPNE1	-0.11276	0.41303	0.641943
SLC12A5	-0.03648	0.413031	0.641943
LINC00403	0.001172	0.413151	0.642084
PRSS30P	-0.01816	0.413199	0.642112
GNG4	0.004634	0.413313	0.642198
NLRP9	-0.00439	0.413337	0.642198
MBNL1-AS	-0.07009	0.413344	0.642198
LOC340178	-0.00217	0.413395	0.642233
LOC284825	-0.00102	0.413458	0.642262
DCD	-0.00561	0.4135	0.642262
MEGF10	-0.01771	0.413516	0.642262
HTR2A	-0.03227	0.413533	0.642262
DENND2C	0.059406	0.413727	0.642437
BTC	-0.04952	0.413729	0.642437
CYP11A1	-0.00208	0.413817	0.642437
LOC10192	-0.00149	0.413884	0.642437
EFR3A	0.088997	0.41389	0.642437
SRP9	0.06307	0.413907	0.642437
GNRH1	-0.03006	0.414009	0.642437
SKA2	0.064815	0.414014	0.642437
LOC10013	-0.01642	0.41405	0.642437
SYTL5	-9.49E-04	0.41406	0.642437
TMEM131	0.046457	0.414063	0.642437
FAM24B	0.197073	0.41407	0.642437
GNAT3	-0.00547	0.414077	0.642437
JPH3	0.001698	0.414078	0.642437
PSAT1	0.151024	0.414093	0.642437
SPATA6L	-0.00121	0.414121	0.642437
IFNE	-0.00283	0.414233	0.642566
SYCE2	0.056529	0.414394	0.642769
NPM3	0.156095	0.414542	0.642953
FOXJ1	-0.03658	0.414589	0.642963
RP2	0.070033	0.414608	0.642963
LOC643659	0.001326	0.414741	0.643096
LINC00589	-0.00499	0.414782	0.643096
FAM83E	-0.00154	0.414783	0.643096
RP5-1170D	-0.0132	0.414821	0.643109
KRTAP17-1	-0.00904	0.415002	0.643344
RP11-475A	-0.00114	0.415204	0.643611
ZMYM5	0.111212	0.415382	0.64384
CHST1	-0.00371	0.415419	0.643851
FOXQ1	-0.00222	0.415606	0.644096
RP11-320N	0.006174	0.415679	0.644163
HTATIP2	0.111569	0.415809	0.644318
RP11-24P1	-0.00105	0.415957	0.644502

FLJ32154	8.47E-04	0.416033	0.644549
CTAG2	-0.0712	0.416048	0.644549
MAPK10	-0.01769	0.416124	0.644621
RP11-25I1!	-0.01765	0.416289	0.644832
PSMG3-AS	-0.07399	0.416378	0.64487
TERF1	0.060172	0.416382	0.64487
ANGPTL7	-0.00573	0.416403	0.64487
ANKS4B	-0.00451	0.416519	0.644941
AMELY	-0.01117	0.416522	0.644941
LOC10050(	0.050303	0.416538	0.644941
CERS6	-0.0789	0.416686	0.645124
LOC33929!	-0.00686	0.416798	0.645156
DCSTAMP	0.026066	0.416839	0.645156
CRLF1	-0.02493	0.416854	0.645156
APOC3	-0.01083	0.416855	0.645156
LOC10024(	0.008602	0.416856	0.645156
DPYSL3	-0.01571	0.416977	0.645296
THEG5	0.007732	0.417092	0.645428
CALML5	-0.02224	0.417187	0.645501
CLUL1	0.008666	0.417201	0.645501
HTR4	-0.0074	0.417253	0.645501
TRIM40	-0.0104	0.417282	0.645501
RP11-95O:	-0.00351	0.417315	0.645501
GSTTP1	0.003383	0.417318	0.645501
WDR65	-0.01104	0.41737	0.645536
POU4F1	0.111111	0.41746	0.645628
GXYLT1	0.072089	0.417562	0.645706
C2orf47	-0.04762	0.417569	0.645706
GCA	0.104134	0.417624	0.645743
LOC15843	-0.00234	0.417767	0.64592
IGDCC4	-0.00365	0.417838	0.645982
RPL30	0.019991	0.417908	0.646045
CRCT1	0.001098	0.418001	0.646143
ODF3L1	-0.00527	0.418314	0.646573
SP8	-0.04	0.418368	0.646573
FETUB	-0.00152	0.418369	0.646573
FGF4	0.007109	0.418516	0.646736
NIPA2	0.056894	0.418537	0.646736
ADAMTS3	-0.19456	0.41859	0.646736
LOC10065:	-0.00962	0.418594	0.646736
FZD9	-0.0182	0.418632	0.646749
GDF11	0.091497	0.418672	0.646762
NIPSNAP3	-0.10846	0.418701	0.646762
LHX1	-0.01419	0.418786	0.646849
LRRC7	-0.04515	0.418919	0.647008
TRY2P	-0.004	0.418998	0.64706
FBXL7	0.061645	0.419013	0.64706
RP11-151E	-0.00333	0.419296	0.647422
ANKRD10	-0.04698	0.419307	0.647422
ELOVL7	-0.18707	0.419473	0.647592
SLC30A2	-0.01868	0.419477	0.647592
TMEM70	0.068984	0.419533	0.647632
FAM122C	-0.04262	0.419662	0.647745
CTNNA3	-0.04112	0.41968	0.647745
PLEKHA6	-0.05911	0.419696	0.647745
PPP1R3E	0.111281	0.419762	0.647801
TTC9C	-0.06287	0.419836	0.647869
TAPT1-AS:	-0.16694	0.419917	0.647948

PNMA1	-0.10421	0.420054	0.648114
SRPR	0.064965	0.420185	0.648269
RP1-28C2(	-0.00203	0.420235	0.648293
PMS1	0.058743	0.420273	0.648293
LOC10192	-0.01276	0.420291	0.648293
CRADD	0.083447	0.420378	0.648381
FABP6	7.29E-04	0.420416	0.648394
SHPK	-0.04674	0.420616	0.648657
WFDC12	-0.01266	0.420713	0.64876
S100A2	-0.00981	0.420918	0.648999
FLJ31715	-0.0248	0.420928	0.648999
LINC01305	0.005843	0.421014	0.649019
HNRNPUL2	-0.04401	0.421031	0.649019
SMC1B	-0.00886	0.421031	0.649019
OIP5-AS1	0.066497	0.421154	0.649163
OR5P2	-0.00152	0.421197	0.649183
PRICKLE1	0.113124	0.421249	0.649217
LOC10013(	-0.06749	0.421315	0.649272
C15orf54	-0.07788	0.421353	0.649284
SYNE4	0.002724	0.421437	0.649289
LOC10192	-0.01136	0.421445	0.649289
ATG4D	0.073574	0.421463	0.649289
CKMT2	0.034818	0.421475	0.649289
SUGCT	0.06449	0.421532	0.64933
THEM5	0.001244	0.42161	0.649332
KHDC3L	0.004616	0.421622	0.649332
TNRC6C	-0.01792	0.421623	0.649332
SYNCRIP	0.058964	0.421764	0.64946
ZFP82	-0.06485	0.421767	0.64946
ARHGAP31	0.027748	0.42184	0.649526
RP11-747H	-0.08455	0.421899	0.649571
CCSAP	0.025841	0.421947	0.649579
LOC10012	-0.00734	0.421964	0.649579
P2RX2	-9.83E-04	0.421994	0.649579
RP11-416N	-0.08883	0.42207	0.64963
MDH1B	-0.01413	0.422087	0.64963
LINC01085	0.01158	0.422125	0.649643
LINC00937	-0.02777	0.422172	0.649668
DPY19L2P4	-0.01852	0.422281	0.64979
LOC10099(	8.29E-04	0.422311	0.64979
POM121L1	0.009262	0.422385	0.649858
TSPAN3	-0.02577	0.422434	0.649887
FDX1	-0.05061	0.422659	0.650148
PRM3	-0.00565	0.422684	0.650148
CBL	-0.17276	0.422711	0.650148
ACAP2	-0.08908	0.422727	0.650148
APOB	0.019115	0.422754	0.650148
EIF2AK4	0.043602	0.422902	0.650255
LOC10050(	-0.01226	0.42292	0.650255
KRT4	-0.00323	0.422939	0.650255
CASC16	-0.00946	0.422962	0.650255
KIAA0355	-0.12678	0.422977	0.650255
CCDC171	-0.07103	0.423004	0.650255
DNAH17-A	7.98E-04	0.423163	0.650454
HSF4	-0.04139	0.423441	0.650783
LRFN1	-0.06896	0.423515	0.650783
RP11-1007	-0.00722	0.423518	0.650783
LINC00708	0.00999	0.423518	0.650783

RP1-151F1	-0.0651	0.423527	0.650783
IL24	0.021473	0.423671	0.650957
FOXF2	-0.00523	0.423744	0.651003
NTM	-0.00323	0.42376	0.651003
RAET1E	0.001883	0.423905	0.651179
TMEM51-/	-0.00558	0.423967	0.651228
SLC22A6	0.007	0.424102	0.651389
AF067845.:	-0.00242	0.42424	0.651555
GRAPL	0.002685	0.424282	0.651572
LTBP1	0.211679	0.424317	0.65158
NKX6-1	-0.01258	0.42455	0.651891
C14orf79	0.029832	0.424739	0.652115
LRRC34	-0.11464	0.424777	0.652115
LOC10272:	0.022249	0.424801	0.652115
RAB6A	0.059185	0.424815	0.652115
FAM186A	-0.00466	0.424886	0.652176
GJA5	0.037511	0.424953	0.652233
COLCA2	-0.00549	0.425019	0.652252
RSRC1	-0.08733	0.425032	0.652252
KRTAP5-9	0.018367	0.425084	0.652252
CCNA1	0.223684	0.425086	0.652252
SLC13A4	-0.00627	0.425155	0.652313
LOC10012{	-0.18266	0.425221	0.652367
LOC10099(	-0.0025	0.425293	0.652407
LOC10063:	0.002356	0.425307	0.652407
PI3	0.049001	0.425411	0.652476
HINT1	0.064215	0.425413	0.652476
POMGNT1	-0.0081	0.425482	0.652537
HOXA-AS2	0.002911	0.425546	0.652543
NXT2	-0.06668	0.425581	0.652543
LOC10272!	-0.00163	0.425582	0.652543
UNC5B	0.035117	0.425629	0.652543
OR5J2	-0.0015	0.425675	0.652543
C21orf88	0.001388	0.425691	0.652543
CHL1-AS1	-0.00387	0.425698	0.652543
ZNF563	0.050377	0.425786	0.652628
A4GALT	0.031321	0.425869	0.652628
C14orf37	0.043522	0.425871	0.652628
KCNN1	-0.01905	0.425874	0.652628
SORBS3	0.026566	0.425908	0.652628
MID2	-0.02369	0.425975	0.652628
KCNK7	-0.00539	0.42598	0.652628
SUGP1	-0.05639	0.425994	0.652628
CD81-AS1	-0.00689	0.426144	0.652798
RP11-330N	0.002307	0.426176	0.652798
GPR31	8.89E-04	0.426195	0.652798
SUV420H1	0.068554	0.426247	0.652831
LOC10192{	0.005849	0.426337	0.652923
SERPINF2	0.013602	0.426369	0.652926
LINC00899	-0.08794	0.426438	0.652986
ANKRD6	0.124388	0.426583	0.653161
KAT8	-0.05496	0.426672	0.65325
PPM1L	0.052225	0.426857	0.653488
CTSE	0.113511	0.427012	0.653599
LOC10192	-0.02104	0.427021	0.653599
RP11-333I.	-0.00166	0.427022	0.653599
IL17B	0.004251	0.42705	0.653599
EFCAB5	0.003249	0.427189	0.653766

KAZALD1	-0.02038	0.427286	0.653779
IMMP1L	-0.08349	0.427313	0.653779
SLC22A8	-0.01729	0.427314	0.653779
RP11-508N	-0.01162	0.427372	0.653779
POLR2D	-0.06363	0.427386	0.653779
CHAD	-0.02256	0.427405	0.653779
LOC10099(	-0.01173	0.427409	0.653779
FAM104B	0.032131	0.427491	0.653858
CCDC37-A	-0.00607	0.427564	0.653923
TRAF3IP2-	-0.03017	0.427621	0.653937
GS1-24F4.:	-0.03028	0.427634	0.653937
TRIM55	-0.00273	0.427897	0.654294
ATN1	0.003744	0.427966	0.654314
FAM105A	0.145054	0.427981	0.654314
LOC10192!	-0.03472	0.428001	0.654314
TTC28	-0.10185	0.428136	0.654475
TCP11L1	-0.03769	0.428287	0.654659
SCGB2A1	8.12E-04	0.428364	0.654683
LINC00948	-0.00279	0.428391	0.654683
FUT7	-0.0414	0.428394	0.654683
COL6A2	-0.04056	0.428499	0.654799
DPH6-AS1	-0.00197	0.428602	0.65491
KRTAP1-1	-0.00462	0.428912	0.655271
STK4	-0.10335	0.428964	0.655271
BC045791	0.006636	0.428971	0.655271
ERCC2	0.035967	0.428984	0.655271
OTC	-0.01244	0.42899	0.655271
LOC10099(	-0.01404	0.429028	0.655283
TLDC1	-0.04982	0.429098	0.655344
MLNR	-0.00149	0.429227	0.655479
TTC22	-0.00178	0.429247	0.655479
FAM160B1	-0.03974	0.429294	0.655504
OXCT2	0.034182	0.429405	0.655627
TLX3	0.007474	0.429683	0.655965
C22orf46	0.037765	0.429689	0.655965
C15orf26	-0.09869	0.429717	0.655965
DUSP4	0.063954	0.429759	0.655983
C17orf99	0.191689	0.42989	0.656137
CTD-2302I	-0.00971	0.430024	0.656255
OR7E2P	-0.00181	0.43006	0.656255
MMP12	-0.00229	0.430084	0.656255
MPO	0.235611	0.430089	0.656255
GABPB1-A	-0.17452	0.430126	0.656266
TRADD	0.060055	0.430286	0.656464
GALNT1	-0.04193	0.430366	0.656533
XIRP2	-0.14114	0.430392	0.656533
PP14571	-0.00917	0.430588	0.656785
LOC10050!	-0.01579	0.430722	0.656912
TPPP	-0.00517	0.430733	0.656912
LOC10050(	0.00957	0.430762	0.656912
TRNP1	-0.00878	0.430865	0.656988
HPGDS	0.230448	0.430872	0.656988
HMCN2	-0.00589	0.430906	0.656993
LINC00487	0.040064	0.430995	0.657083
ID4	-0.00545	0.431166	0.657296
BPIFB6	-0.00273	0.431486	0.657738
ACCS	-0.08089	0.431562	0.657807
LOC72986(	0.010749	0.43169	0.657912

LOC10192	-0.00533	0.431691	0.657912
RP11-874J	-0.0827	0.431801	0.658033
LOC10192	0.03184	0.431866	0.658085
LOC10028	-0.00642	0.432041	0.658298
NAA35	-0.05027	0.432094	0.658298
KPTN	-0.06682	0.432096	0.658298
SCYL2	-0.08179	0.432129	0.658302
LOC28635	-0.01095	0.432237	0.65842
TMEM261	0.063447	0.432323	0.658437
HIST1H3C	-0.04755	0.432344	0.658437
RPL29	0.067852	0.432346	0.658437
NPAP1	-0.00874	0.432379	0.658437
LOC14970	-0.0047	0.432401	0.658437
IFNA2	0.001838	0.432496	0.658536
ADD3-AS1	-0.02823	0.432606	0.658594
TIGD3	-0.06035	0.432619	0.658594
CAPN6	-0.01005	0.432665	0.658594
DMTF1	-0.0916	0.432681	0.658594
CTSW	-0.15473	0.43271	0.658594
KIAA1210	-0.00215	0.432717	0.658594
ADCY10P1	-0.06634	0.432772	0.658602
RPUSD2	-0.08754	0.432783	0.658602
BCMO1	0.018804	0.432918	0.658613
USP2	0.016154	0.432947	0.658613
NME9	-0.00324	0.432956	0.658613
LOC10192	-0.02987	0.432959	0.658613
CD48	0.117912	0.432965	0.658613
CABLES2	0.072871	0.432973	0.658613
CD1B	-0.02789	0.43301	0.658616
RAB40A	-0.01828	0.433035	0.658616
BANF2	-0.00248	0.433083	0.658643
WDR89	0.0797	0.43325	0.65885
ARHGEF38	0.003715	0.433406	0.659033
ZNF440	-0.05654	0.433431	0.659033
CCDC150	-0.08384	0.433802	0.659533
TIPARP-AS	-0.03438	0.433836	0.659533
U47924.27	-0.00764	0.433858	0.659533
LOC10192	0.008183	0.433882	0.659533
PTK2B	0.064229	0.434039	0.659726
BRIP1	-0.10411	0.434099	0.65973
BCL2L14	-0.00238	0.434103	0.65973
PLEKHA7	0.09234	0.434198	0.659791
GPR22	-0.03451	0.434204	0.659791
LTB4R2	-0.03362	0.434317	0.659892
LINC00113	0.001177	0.434348	0.659892
C14orf105	-0.00334	0.434361	0.659892
MYLIP	0.101643	0.434511	0.660029
MCF2L	-0.00238	0.434536	0.660029
BCAR1	0.016374	0.434544	0.660029
RUFY1	0.077774	0.434599	0.660065
WDR5	0.06125	0.434628	0.660065
LOC10013	-0.00288	0.434714	0.660149
BC033164	0.023394	0.434791	0.660181
FAM83D	-0.20593	0.434796	0.660181
ATP8B3	-0.01699	0.434875	0.66024
TCRBV15S	-0.00126	0.434896	0.66024
SCUBE2	-0.01295	0.434963	0.660289
LINC00901	-0.00251	0.434989	0.660289

TNNT3	-0.0377	0.435019	0.660289
RND2	-0.01371	0.435138	0.660415
CLSTN2	-0.05908	0.435174	0.660415
CXCL6	0.092484	0.435194	0.660415
CYP2S1	0.006249	0.435301	0.660531
RP11-981(	-0.07462	0.435336	0.660533
ZC2HC1A	-0.12699	0.435363	0.660533
SAMD5	0.00323	0.435484	0.66067
SHCBP1L	-0.01421	0.435521	0.66068
IRGM	0.043919	0.435765	0.660964
NDEL1	0.030906	0.435779	0.660964
MAGEA8	-0.0022	0.4358	0.660964
RP1-6P5.2	-0.03	0.435868	0.66102
RP6-99M1	-0.16102	0.435905	0.661032
LOC10192	-0.00171	0.435978	0.661096
LINC00644	-8.33E-04	0.436076	0.661197
MYL7	-0.00581	0.436172	0.661297
UNC13C	-0.03136	0.436365	0.66149
TNFRSF25	0.065578	0.436385	0.66149
CLDN4	-0.00636	0.436391	0.66149
SPATA3-A	-0.00318	0.436501	0.66161
LOC10192	-0.02007	0.436536	0.661617
UBE2J1	0.073704	0.436678	0.661786
SOX8	-0.02021	0.436962	0.66213
TEX36-AS1	-0.00113	0.436966	0.66213
AF279780	0.005052	0.43728	0.66256
FOLH1B	-0.04396	0.437339	0.66256
CDH12	-0.02849	0.437366	0.66256
DRD1	0.003148	0.437378	0.66256
SEC31B	-0.12588	0.437403	0.66256
NIP7	0.070801	0.437554	0.662701
LINC00619	-0.03976	0.437626	0.662701
IL23R	-0.00356	0.437684	0.662701
ITGB4	-0.00875	0.437694	0.662701
LIFR	0.062979	0.437715	0.662701
LOC10029	0.027893	0.437752	0.662701
FAM120B	-0.06454	0.437756	0.662701
ITGB8	-0.00348	0.437823	0.662701
LINC00471	-0.02712	0.437846	0.662701
ZNF430	-0.08268	0.437882	0.662701
AK093205	-0.00507	0.437886	0.662701
MROH8	-0.02504	0.437918	0.662701
SCRT1	0.009483	0.43792	0.662701
AGBL3	-0.01486	0.437924	0.662701
LOC10192	-0.01013	0.438057	0.662856
OR1A2	-0.00164	0.438232	0.663073
RAPGEF4-/	0.004983	0.438298	0.663084
KBTBD11	-0.17892	0.438348	0.663084
RBM11	0.098927	0.438368	0.663084
RP11-677(	-0.0039	0.438377	0.663084
CST5	-0.02147	0.438406	0.663084
MGC15885	-8.17E-04	0.438422	0.663084
LOC10192	-0.00427	0.438463	0.663099
HLCS	0.019431	0.438552	0.663177
RP11-585F	-0.05654	0.438576	0.663177
LOC10272	8.30E-04	0.438692	0.663306
MOCOS	0.025696	0.438808	0.663435
GALNT9	0.008647	0.438907	0.663539

LTN1	-0.09007	0.438975	0.663596
VN1R10P	7.48E-04	0.439125	0.663776
LOC10192	-0.03239	0.439166	0.663784
KRTAP9-3	-0.0033	0.439191	0.663784
CD300LB	-0.01798	0.439352	0.663944
CCDC116	-0.00198	0.439359	0.663944
RP11-263K	-0.02759	0.439436	0.663953
APLN	-0.03423	0.439453	0.663953
CASP3	-0.10672	0.439457	0.663953
ADPRHL1	-0.00656	0.439571	0.664079
FBXO38	-0.06804	0.439678	0.664194
TBC1D7	0.045874	0.439743	0.664216
MAGEA12	-0.00424	0.439754	0.664216
PDE10A	-0.01383	0.439821	0.664272
AC016999.	-0.01351	0.439875	0.664307
ZYG11A	-0.01237	0.44001	0.664465
NTN4	0.120594	0.440048	0.664475
LOC10065	0.007971	0.440207	0.664668
ANXA11	-0.05984	0.440269	0.66471
RP11-55K1	-0.00653	0.44031	0.66471
LNX1	-0.10898	0.440326	0.66471
CENPM	0.116349	0.440379	0.664744
SCGN	-0.0133	0.440453	0.664809
ADIRF-AS1	-0.00911	0.440571	0.664941
ANKLE2	0.063909	0.440798	0.665236
RP11-546C	-0.00167	0.440913	0.665364
HADH	0.071762	0.44099	0.665433
OLR1	0.104429	0.44118	0.665643
LRP11	-0.16885	0.441237	0.665643
PIWIL4	0.139166	0.441254	0.665643
FLJ13224	0.014821	0.441282	0.665643
DKK3	7.68E-04	0.441311	0.665643
SSTR1	0.008294	0.441313	0.665643
NT5C3A	0.084622	0.441367	0.665679
SMPDL3B	0.045614	0.441471	0.665714
KIAA1841	-0.05439	0.441496	0.665714
LINC00645	7.60E-04	0.441505	0.665714
DDI1	0.00123	0.441514	0.665714
DPP4	0.037506	0.441549	0.665721
ACADL	-0.0179	0.441625	0.665788
FAM131C	-0.01826	0.441705	0.665863
RP11-471E	-0.02801	0.44186	0.665975
TBL1Y	-0.024	0.441908	0.665975
DSC1	-0.00685	0.44193	0.665975
SRD5A3-A	0.025944	0.441939	0.665975
TRBC1	-0.17669	0.441968	0.665975
MAPKBP1	-0.04636	0.442001	0.665975
RP11-112L	0.006996	0.442003	0.665975
LOC10192	0.003683	0.442028	0.665975
CNTNAP5	-0.01474	0.442072	0.665975
GALR2	-0.00279	0.442103	0.665975
MANEAL	0.131462	0.442118	0.665975
CYB5B	-0.0597	0.442244	0.666119
ZNF778	-0.02565	0.442415	0.66633
LOC10192	-0.00755	0.44245	0.666337
SCP2	-0.05521	0.442554	0.666447
EMC10	0.043541	0.4427	0.666553
ACO2	0.044871	0.442706	0.666553

LCE2B	-0.0115	0.442717	0.666553
ERICH1	-0.08783	0.442767	0.666583
TINCR	-0.0087	0.442814	0.666592
P2RX1	-0.1089	0.442879	0.666592
EMR3	0.054762	0.442927	0.666592
CTC-527H	-0.01228	0.442929	0.666592
PCK1	-0.02584	0.44294	0.666592
LINC01241	0.003594	0.442958	0.666592
PRLR	-0.00176	0.443007	0.666619
KDM1A	0.058965	0.443055	0.666645
LOC10192	-0.00257	0.443122	0.666655
LINC00452	-0.01821	0.443123	0.666655
LOC10192	0.042434	0.443184	0.6667
DTHD1	-9.23E-04	0.44323	0.666723
C11orf42	-0.00655	0.443348	0.666767
C15orf45	-0.00437	0.44335	0.666767
LOC10192	-0.00674	0.443384	0.666767
SENP7	-0.07062	0.443387	0.666767
ZBTB20	-0.11244	0.443451	0.666767
CAPSL	-0.07344	0.443456	0.666767
CACNB4	-0.08437	0.443501	0.666767
ODC1	-0.09819	0.443507	0.666767
CTA-280A	0.016865	0.443536	0.666767
SLC1A1	0.014286	0.443588	0.666798
GOT2	-0.08121	0.443626	0.666809
PITPNM2	-0.00569	0.443741	0.666932
TRAF1	0.015976	0.443769	0.666932
H1F0	0.128069	0.443895	0.667042
PNPLA5	-0.00103	0.443904	0.667042
NCAPH2	0.050252	0.443974	0.667077
LOC28373	-0.00579	0.443989	0.667077
C20orf26	-0.00446	0.444066	0.667146
RP11-1024	-0.00473	0.444203	0.667258
DIRAS2	7.20E-04	0.444213	0.667258
DFNB59	-0.08487	0.444284	0.667258
EIF1B-AS1	0.039352	0.444292	0.667258
LINC00260	0.057507	0.444295	0.667258
LOC28618	-0.00284	0.444426	0.667365
LOC10192	-0.00287	0.444445	0.667365
DGAT1	0.095145	0.444458	0.667365
LOC10192	-0.06295	0.444489	0.667365
LOC10192	0.00501	0.444575	0.667448
HINT3	0.10355	0.444628	0.667466
BEGAIN	-0.01849	0.444649	0.667466
SPACA4	-0.00994	0.444725	0.667535
ARFGAP3	-0.09811	0.444834	0.6676
AC009120.	0.086013	0.444893	0.6676
THPO	-0.00717	0.444906	0.6676
BOC	-0.01883	0.444907	0.6676
RAB3A	0.026392	0.444949	0.6676
HIST1H2B	-0.18504	0.444954	0.6676
MOG	9.26E-04	0.444996	0.667618
AC006538.	0.045667	0.445046	0.667638
DBN1	0.089157	0.445071	0.667638
LRTOMT	0.007226	0.445186	0.667764
LOC10050	0.003247	0.445355	0.66797
BPESC1	-0.0011	0.445504	0.66807
IKZF1	0.134607	0.445512	0.66807

TMEM98	-0.23393	0.445514	0.66807
PVRL3	-0.00985	0.445585	0.668131
CCL27	-0.01079	0.44585	0.668482
SLC7A4	-0.00303	0.445963	0.668575
LOC10192	-0.00692	0.445992	0.668575
CT55	-0.00491	0.446005	0.668575
GRID1-AS	-0.00174	0.446075	0.668634
RP11-216L	-0.05534	0.446135	0.668678
GOLGA4	0.068206	0.446186	0.668708
PRND	0.003872	0.446233	0.668732
RND1	0.041887	0.446278	0.668753
KCTD4	-0.00373	0.446389	0.668838
SMAD5-AS	9.57E-04	0.4464	0.668838
HERC4	-0.06303	0.446427	0.668838
GFI1B	0.128111	0.446488	0.668883
LOC40106	-0.01498	0.446579	0.668967
DLX1	0.021596	0.446606	0.668967
LOC44160	-0.01682	0.446646	0.668981
SORCS3-A	0.002982	0.446792	0.669154
LIPF	-0.0086	0.447068	0.66949
PHACTR1	-0.08923	0.447079	0.66949
C2orf44	0.093107	0.447121	0.669507
ZNF277	0.075731	0.447281	0.669668
RP1-272E8	-0.00707	0.447291	0.669668
SEC1P	0.012104	0.447365	0.669732
PLA2R1	-0.01174	0.447434	0.66979
C15orf32	-0.00113	0.447475	0.669805
GPT	0.019607	0.44753	0.669839
BVES	0.007024	0.447585	0.669839
PERP	-0.0098	0.44759	0.669839
XIRP1	-0.04141	0.447633	0.669856
AMMECR1	0.13175	0.447812	0.670077
ACTG2	0.020895	0.447846	0.670082
RP11-646J	-0.00141	0.447922	0.670142
APCS	-0.01817	0.447996	0.670142
PRR34	0.010271	0.448002	0.670142
ZBTB2	0.07796	0.448035	0.670142
RP6-91H8.	-0.00288	0.44804	0.670142
NBPF8	-0.01034	0.448111	0.670194
C1orf52	-0.05071	0.448137	0.670194
CIDEA	-0.00541	0.448253	0.670322
GH2	-0.00656	0.4483	0.670344
FHL5	-0.00158	0.44836	0.670388
KRTCAP3	0.095384	0.448436	0.670456
TTLL11-IT1	-0.00519	0.44849	0.670461
ANKRD24	-0.00406	0.448501	0.670461
C15orf40	0.042824	0.448564	0.67049
LOC72782	0.065881	0.448583	0.67049
ZNF426	-0.0824	0.448768	0.670679
ABCD1	0.017937	0.448797	0.670679
GATA3	0.091308	0.448802	0.670679
CTRC	0.017382	0.44887	0.670734
REG3G	-0.00974	0.448987	0.670748
LINC01186	-0.00594	0.449005	0.670748
CARD14	-0.006	0.449016	0.670748
GLI2	-0.02096	0.449018	0.670748
LOC14569	-0.00761	0.449034	0.670748
PLAC9	-0.00871	0.449174	0.67091

LINC00294	0.077372	0.449232	0.670952
FSCN3	-0.00298	0.449354	0.67108
CHRNA5	0.009824	0.449384	0.67108
AK054988	-0.01083	0.449411	0.67108
SNTG1	-7.91E-04	0.449735	0.671483
FANCA	-0.08763	0.449804	0.671483
HLA-DQB1	-0.44701	0.449824	0.671483
TMEM115	0.082983	0.449836	0.671483
EFNA3	0.030375	0.449862	0.671483
QTRT1	-0.10567	0.449885	0.671483
WRB	-0.06387	0.449956	0.671483
PAX1	-0.00269	0.449997	0.671483
VWF	0.102134	0.450035	0.671483
KDF1	0.004309	0.450041	0.671483
KCNAB2	0.080561	0.450101	0.671483
ERICH3-AS	-0.01036	0.450105	0.671483
OBFC1	-0.04729	0.450107	0.671483
NOP2	-0.06422	0.450115	0.671483
COL8A1	0.001855	0.450185	0.671536
RP1-86D1.	-0.02901	0.450213	0.671536
AC106801.	-0.01549	0.450262	0.671563
RASAL1	0.010194	0.450304	0.671567
BNC2	-0.00144	0.45037	0.671567
PP12613	-0.00657	0.450385	0.671567
CTLA4	-0.00128	0.450389	0.671567
ESRP1	-0.01387	0.450463	0.671584
C9orf57	-0.00331	0.450507	0.671584
FSTL4	-0.00293	0.450515	0.671584
CHAT	-0.0021	0.450524	0.671584
TNNT2	-0.01347	0.450611	0.671667
SAPCD2	0.120703	0.450713	0.671765
GALNT13	-0.00339	0.450738	0.671765
RABAC1	-0.10527	0.450833	0.671844
ATP2A3	0.036816	0.450854	0.671844
C5orf17	-0.02884	0.450909	0.671845
RHBDL1	-0.003	0.450935	0.671845
DNMT3L	-0.00335	0.450947	0.671845
DGCR12	-0.01055	0.451001	0.671879
LOC10192	-0.00101	0.451117	0.672004
HIPK2	0.068212	0.451151	0.672009
MYEF2	-0.13448	0.451247	0.672064
MAP3K8	0.140092	0.45125	0.672064
EFNB2	0.138338	0.451397	0.672226
FRAT1	-0.07397	0.451452	0.672226
TM4SF4	-0.00324	0.451481	0.672226
BC048997	-7.37E-04	0.451483	0.672226
BHLHE40	0.104054	0.451628	0.672397
SLC25A10	-0.04205	0.451738	0.672514
FCN3	-0.03696	0.451959	0.672781
PEX11B	0.065219	0.452008	0.672781
RAD51	-0.07459	0.452011	0.672781
RP11-118(	0.002838	0.452132	0.672876
AHR	0.129563	0.452136	0.672876
BAI2	-0.00308	0.452233	0.672967
TLK1	0.063642	0.45226	0.672967
CNTLN	0.059183	0.452452	0.673075
LOC10050(	-0.00384	0.45246	0.673075
KCNH5	8.39E-04	0.45247	0.673075

ZAR1	-0.00477	0.452474	0.673075
POLD1	0.096615	0.452518	0.673075
WIPI2	-0.06863	0.452519	0.673075
SLC12A3	-0.00159	0.452723	0.673332
SPAG7	0.051816	0.452796	0.673395
ALKBH3	0.064092	0.452878	0.673427
MXD3	0.071029	0.45288	0.673427
TTC29	0.042029	0.45292	0.673441
KLF11	0.088401	0.452955	0.673446
DPF3	0.017286	0.453101	0.673617
LRP12	-0.10659	0.453147	0.673638
ARL5C	-0.00292	0.453331	0.673867
MEIS3	-0.00335	0.453636	0.674229
NMNAT3	-0.06468	0.453664	0.674229
CREBBP	-0.03906	0.453669	0.674229
LOC10192(	-0.00281	0.453836	0.674432
CDC42	-0.08767	0.453968	0.674581
LINC00955	8.29E-04	0.454017	0.674608
TTC39B	0.106909	0.454111	0.674672
ZNF132	-0.01314	0.454122	0.674672
PDIA2	0.002875	0.454186	0.67472
UCMA	0.007167	0.454521	0.675172
LINC00221	-0.00112	0.45462	0.675272
C2orf70	0.001164	0.454689	0.675328
FAM187B	6.95E-04	0.454753	0.675377
MCAT	0.083115	0.454802	0.675382
LARS	-0.06062	0.454819	0.675382
LRP2	-0.01743	0.454907	0.675467
HAGLR	0.011115	0.455013	0.675553
AVIL	0.039926	0.455039	0.675553
PSEN1	-0.05818	0.455059	0.675553
CES5A	-0.00701	0.455173	0.675676
ABI3BP	-0.00611	0.455241	0.675725
DNAH7	-0.00374	0.455268	0.675725
TMEM190	-0.00652	0.45532	0.675756
GRIA3	7.39E-04	0.455379	0.675798
LOC64298(	0.015994	0.455452	0.675829
PTPRB	-0.01512	0.455463	0.675829
LOC10192(	0.002733	0.455513	0.675858
CCDC102B	0.043713	0.455601	0.675913
SFT2D1	0.085327	0.455613	0.675913
LOC10050	-0.00559	0.455679	0.675953
DLGAP1-A	-0.02059	0.455705	0.675953
PDLIM4	0.005629	0.455736	0.675953
BRCA2	0.086627	0.455765	0.675953
TSSK3	0.003853	0.455874	0.676068
LINC00935	-0.00313	0.456038	0.676204
LINC01057	0.001894	0.45605	0.676204
CD24	0.262241	0.456068	0.676204
RNF150	0.09612	0.456091	0.676204
MIR3663H(	-0.00107	0.456155	0.676253
HMX1	-0.03064	0.456342	0.67648
SAMD15	-0.00266	0.456394	0.67648
LAMA3	-8.63E-04	0.45645	0.67648
NPAS4	-0.00207	0.456479	0.67648
RP11-505k	0.00254	0.4565	0.67648
CTD-2002:	-0.01067	0.456522	0.67648
ZFHX3	0.022365	0.456526	0.67648

RP11-352E	-0.00632	0.456615	0.676543
SUB1	-0.05592	0.456632	0.676543
FANK1	0.061932	0.456707	0.676609
LOC14594	-0.01928	0.456844	0.676728
LOC10099	0.002449	0.45685	0.676728
GOLGA5	-0.0446	0.457047	0.676973
C9orf89	0.047852	0.457104	0.677011
C1QL1	-0.00848	0.457193	0.677098
LOC10192	-0.00319	0.457462	0.67742
ZNF843	0.010855	0.457493	0.67742
CEP41	-0.03407	0.457505	0.67742
PPP1R21	-0.03642	0.457833	0.67786
SIMC1	0.086234	0.458031	0.678042
LINC01138	-0.01719	0.458045	0.678042
ARMC7	0.045111	0.458054	0.678042
FANCI	0.091744	0.458134	0.678042
GCM1	-0.00478	0.458179	0.678042
APBA2	-0.12011	0.458186	0.678042
LINC01360	8.84E-04	0.458204	0.678042
RP1-102H	0.090409	0.458207	0.678042
ITGA10	0.010012	0.458346	0.678186
TBK1	0.062379	0.458393	0.678186
PKP2	0.093783	0.458398	0.678186
DNAJC22	0.005156	0.458572	0.678252
LINC00698	0.015494	0.458576	0.678252
FLVCR1-A	0.124552	0.458582	0.678252
LOC10013	0.025635	0.458592	0.678252
SLC9C2	0.003315	0.458599	0.678252
DDX39A	-0.06671	0.45879	0.678488
LGR5	-0.01546	0.459089	0.678883
LEO1	-0.07498	0.459121	0.678884
DSE	0.006302	0.459253	0.679003
ANKRD30A	-0.00375	0.459264	0.679003
PRG2	0.362492	0.459299	0.679008
SFTA3	-0.00276	0.459467	0.679211
MBD3L1	-0.00745	0.459539	0.679234
LOC22112	-0.00993	0.459575	0.679234
RCVRN	0.003903	0.459577	0.679234
MUC6	-0.0144	0.460009	0.679826
MSMO1	0.100383	0.46006	0.679832
FAM110D	-0.02087	0.460075	0.679832
LOC28307	0.104453	0.46012	0.679851
STX10	0.055975	0.460233	0.679943
SQLE	-0.08207	0.460245	0.679943
LINC01405	0.013733	0.460325	0.680006
SERPINB4	6.75E-04	0.46035	0.680006
PRR30	0.003671	0.460398	0.680006
WDR87	-0.01558	0.460413	0.680006
GSTM3	0.126323	0.460476	0.680052
HDHD2	0.042902	0.460559	0.68011
KRTAP5-A	0.004501	0.460596	0.68011
CD207	0.001554	0.460609	0.68011
TCTE1	-0.00836	0.460678	0.680165
TMEM72	-0.05609	0.460866	0.680372
LOC10028	-0.00106	0.460881	0.680372
ST20	-0.07347	0.460952	0.680423
MIR205HG	0.00266	0.460978	0.680423
MNX1-AS	-0.00179	0.46102	0.680438

IL1RL2	-9.88E-04	0.461091	0.680496
HAUS1	0.070891	0.461139	0.680521
PCDH18	0.052441	0.461259	0.680652
WWC1	0.006352	0.461349	0.680723
GNG13	0.001252	0.46137	0.680723
LOC40065!	-0.01602	0.461433	0.680769
WDR88	9.16E-04	0.461474	0.680781
ATP8A2	-0.02409	0.461504	0.680781
HSD17B10	-0.07415	0.461694	0.680895
RNPC3	0.107985	0.461699	0.680895
PHGDH	-0.1571	0.461707	0.680895
EPHA10	0.003393	0.461707	0.680895
SCIN	-0.01107	0.461747	0.680898
LDOC1	0.116746	0.461772	0.680898
KCNA1	-0.00269	0.461837	0.680937
LOC10192!	-0.01312	0.461861	0.680937
SLC24A4	0.084198	0.462006	0.681014
RP4-714D!	-0.03431	0.462043	0.681014
MLANA	-0.00164	0.46213	0.681014
COX17	-0.05272	0.462148	0.681014
ERICH1-AS	-0.006	0.462171	0.681014
HTR2C	-0.00173	0.462175	0.681014
HAND2-AS	-0.00555	0.462181	0.681014
HAO1	-0.00216	0.462188	0.681014
NUDT6	-0.08256	0.462196	0.681014
C10orf107	-0.00153	0.462335	0.681171
PMCH	-0.02529	0.462426	0.68126
LOC10050!	-0.00267	0.46266	0.681523
LOC15227	-0.00163	0.462695	0.681523
RNF121	-0.06573	0.462716	0.681523
SDSL	0.083405	0.462731	0.681523
PCYOX1L	0.092722	0.462791	0.681565
ATP2B3	-8.41E-04	0.462886	0.681659
LHX4	-0.00369	0.463007	0.681731
TTC6	-6.96E-04	0.463023	0.681731
C9orf91	0.0863	0.463029	0.681731
LOC10192	0.002368	0.463109	0.68177
CEP78	-0.07603	0.463135	0.68177
NIN	-0.08042	0.46315	0.68177
KDELR3	0.028219	0.463384	0.682068
TAS2R5	-0.02451	0.463492	0.68218
MUC1	-0.11485	0.463558	0.682231
PTTG3P	-0.06933	0.463608	0.682258
UGGT2	0.067232	0.463773	0.682427
BFSP2-AS1	-0.00434	0.463785	0.682427
FZD1	0.025604	0.463895	0.682542
STEAP2	-0.10387	0.46408	0.682733
LCN6	-0.05999	0.46411	0.682733
DOCK2	0.033568	0.464119	0.682733
COL2A1	-0.08687	0.464165	0.682753
UNC80	-0.00158	0.464232	0.682805
SPAM1	9.07E-04	0.464386	0.682986
LOC10013	-0.18606	0.464557	0.683176
VWA7	0.001689	0.464606	0.683176
AL590762.	-0.03769	0.464629	0.683176
TPTE2P5	-0.02801	0.464642	0.683176
LOC10272	-0.00686	0.464701	0.683212
DLX5	-0.00299	0.464729	0.683212

ADAM23	-0.00809	0.464832	0.683276
ADRA2B	0.016838	0.464836	0.683276
IQCF4	0.006339	0.465138	0.683587
CASC6	-0.00123	0.465152	0.683587
HDGFRP2	-0.04781	0.465156	0.683587
MAGIX	-0.00213	0.46521	0.683587
MGC15705	-0.00842	0.465211	0.683587
PLA2G4F	-0.02101	0.465243	0.683587
PRDM15	0.060399	0.465268	0.683587
LINC00960	-0.12542	0.465309	0.683599
TTC36	0.027443	0.465375	0.683613
C15orf59	-0.01704	0.465381	0.683613
AGAP2	0.029442	0.46544	0.683653
MCAM	-0.03246	0.46551	0.68371
SEMA6B	0.017154	0.465557	0.683733
MRPL16	0.061166	0.465901	0.684157
TEK	-0.08147	0.465909	0.684157
COX8C	-0.002	0.466113	0.684264
RHBDD1	0.039241	0.466114	0.684264
LINC00086	-0.00406	0.466138	0.684264
SLCO6A1	-7.77E-04	0.466179	0.684264
RGS22	-0.01671	0.466192	0.684264
PIK3C2G	-0.00144	0.466204	0.684264
FLJ31713	-0.00461	0.466217	0.684264
PDE3A	0.039653	0.466249	0.684264
BCAM	-0.01167	0.466266	0.684264
XKR4	-0.00251	0.466505	0.684544
EGLN2	0.001843	0.466563	0.684544
FMN2	0.008419	0.466567	0.684544
B3GALT6	0.078068	0.466583	0.684544
EPS8L2	0.005167	0.466666	0.684595
GPRC6A	-0.00516	0.466682	0.684595
LOC28584	-0.00109	0.466767	0.684621
APTR	0.039228	0.466794	0.684621
ATP2C2	-0.00843	0.466825	0.684621
LOC15774	-0.0182	0.466826	0.684621
CDK5R1	0.048027	0.466891	0.68467
LOC10192	-7.63E-04	0.466947	0.684689
SLC43A2	0.058137	0.466967	0.684689
C1orf110	-0.00139	0.4671	0.684762
ZNF33A	0.044253	0.467106	0.684762
LOC10192	-0.00122	0.467112	0.684762
BC045805	0.003495	0.467216	0.684868
LOC64354	6.89E-04	0.467261	0.684888
RPL37	0.01643	0.467293	0.684889
LIN28B	-0.04743	0.467387	0.68498
RP11-352	-0.00268	0.467544	0.685164
GUCY2F	7.26E-04	0.467588	0.685181
BZW1	-0.04209	0.467637	0.685194
GHR	0.074331	0.467673	0.685194
CARHSP1	-0.03906	0.467691	0.685194
IFNA21	-0.00184	0.467733	0.685209
LINC00537	-0.01521	0.467921	0.685437
COQ3	0.082653	0.46798	0.685478
FAM189B	-0.02315	0.468184	0.685704
METTL21C	-0.00456	0.468198	0.685704
GNL1	-0.04137	0.468264	0.685715
RP11-63A	0.001052	0.468291	0.685715

LINC00536	0.00316	0.468331	0.685715
OR2B6	-0.01661	0.468347	0.685715
CACNA1G	0.009257	0.468434	0.685715
ANAPC16	0.03803	0.468453	0.685715
UBXN2B	0.046614	0.468475	0.685715
G6PC	-0.00357	0.468482	0.685715
IL20	-0.00634	0.468554	0.685715
WNT16	0.002219	0.468577	0.685715
USP43	-0.06392	0.468579	0.685715
C10orf62	0.010902	0.468592	0.685715
MCEMP1	0.185548	0.468617	0.685715
FGD3	0.042585	0.468664	0.685738
VSTM4	-0.01342	0.46873	0.685787
PPP1R15B	0.076387	0.468847	0.685879
GATSL2	0.058255	0.468856	0.685879
RP11-399(	-0.00236	0.468887	0.685879
FRMPD1	-0.00667	0.468979	0.685962
ACVR2A	0.062802	0.469014	0.685962
SSUH2	0.001107	0.469039	0.685962
IFFO1	0.073277	0.469104	0.68601
FHOD3	-0.03401	0.469183	0.686081
KLK11	0.006085	0.469244	0.686123
PIK3CB	-0.07622	0.469498	0.686384
TMEM107	-0.0842	0.469502	0.686384
RP11-506(	-0.04974	0.469549	0.686384
LOC10192!	0.029358	0.469549	0.686384
HGFAC	-0.00381	0.469699	0.686551
CADM4	0.005324	0.469727	0.686551
SYT8	-0.00795	0.469799	0.68661
LINC00564	6.48E-04	0.469906	0.686682
RNF185-A!	-0.00321	0.469924	0.686682
CDIPT	0.042775	0.469973	0.686682
TUBB1	0.227247	0.470005	0.686682
KIF4A	0.133122	0.470007	0.686682
LOC73009!	-0.01634	0.470039	0.686682
LOC10050	0.001054	0.470144	0.68679
DARS	0.071718	0.470244	0.686863
TG	0.009346	0.470258	0.686863
MYO5B	0.015706	0.470339	0.686936
LRRC56	-0.04251	0.470644	0.687335
GRID2	-0.00204	0.470701	0.68735
LINC00527	-0.00528	0.470718	0.68735
ARL6IP5	0.068228	0.470844	0.687461
EIF4B	0.067589	0.470857	0.687461
GMNN	-0.08906	0.470987	0.687604
BIN3	0.042106	0.471188	0.687831
LOC10050!	0.025105	0.471206	0.687831
INPP5D	-0.10639	0.471302	0.687925
RASL11B	0.00612	0.471385	0.687999
LINC00659	0.001212	0.471443	0.688038
LOC10028!	-0.01852	0.471507	0.688084
C19orf54	0.065866	0.471592	0.688163
RP11-742E	-0.00247	0.471712	0.688253
RP11-18F1	0.001858	0.471718	0.688253
NSAP11	-0.00601	0.471896	0.688467
TFAP4	-0.02927	0.472009	0.688586
MON2	-0.0687	0.472048	0.688596
TMEM42	0.060644	0.47211	0.688639

GDNF	0.005711	0.47227	0.68873
PANX3	-0.00371	0.472281	0.68873
NKX2-5	-0.0108	0.472301	0.68873
HHLA1	0.015376	0.472312	0.68873
CSTF3-AS1	-0.01315	0.472366	0.68873
LOC10106(	0.010855	0.472367	0.68873
GNRH2	-0.01691	0.472395	0.68873
PCGEM1	0.003446	0.47246	0.688779
DNAJC18	-0.02699	0.472595	0.688929
ANKS3	-0.05387	0.47269	0.688983
SEC16A	-0.04423	0.472696	0.688983
LOC25357:	0.001645	0.472732	0.68899
LATS1	0.022744	0.472879	0.68914
NKX2-1	-0.00372	0.472899	0.68914
RFPL2	-0.02288	0.472947	0.689165
SLC38A3	-0.01356	0.47311	0.689355
KLHL11	-0.00786	0.473186	0.689399
DDX5	-0.0653	0.473203	0.689399
APOBEC3E	-0.15235	0.473256	0.689429
ZNF599	-0.04736	0.473336	0.689498
CD8B	-0.00164	0.473391	0.689533
6-Sep	0.081657	0.473504	0.689651
DCDC2	-0.02779	0.473577	0.689711
RP11-429E	-0.00292	0.473697	0.689818
LIN7C	0.058164	0.473714	0.689818
AC007787.	-0.00165	0.473753	0.689828
CCPG1	0.055926	0.473869	0.68995
RP11-152L	-0.00145	0.473936	0.689974
MMP20	7.85E-04	0.473949	0.689974
LXN	0.110075	0.473999	0.690001
AFM	-0.00187	0.47409	0.690057
TNXB	-0.00152	0.47412	0.690057
ADRBK1	0.013003	0.474133	0.690057
FW339973	0.038831	0.474252	0.690183
LOC51145	-0.0043	0.474342	0.690254
FAM132A	-0.02649	0.474365	0.690254
LOC10028	0.010827	0.474495	0.690398
LBX1	-9.08E-04	0.474545	0.690424
LOC10050(	0.002516	0.474702	0.690601
CAD	-0.04523	0.47473	0.690601
MBD5	0.055968	0.47479	0.690641
NRIP3	-0.11036	0.474912	0.690764
GLRX2	0.063915	0.474938	0.690764
CLEC1B	0.261478	0.475027	0.690847
C3orf33	0.044144	0.475121	0.690937
IL17F	0.001394	0.475376	0.691259
RP1-20C7.	-0.02198	0.475449	0.691259
TPSB2	0.160012	0.475477	0.691259
LCNL1	0.002245	0.475502	0.691259
PRKCG	-0.00648	0.475502	0.691259
LOC64242(	0.004602	0.475538	0.691264
LOC64898	0.058249	0.475588	0.691291
VLDLR	0.094445	0.475891	0.691676
BEST2	-0.00678	0.475917	0.691676
FAM3C	-0.12427	0.47603	0.691765
SIRT3	-0.02367	0.476056	0.691765
EIF3C	0.113098	0.476074	0.691765
STAG3	0.127404	0.476105	0.691765

KANSL1-A	0.087193	0.476187	0.691836
KHDC1L	-6.96E-04	0.476345	0.691978
PRM2	-0.00164	0.476351	0.691978
RUNDC3B	-0.0768	0.47638	0.691978
MMP11	-0.00204	0.47667	0.692335
CLEC2B	0.122627	0.47669	0.692335
UVSSA	0.014692	0.476764	0.692355
ADAM3A	-0.01663	0.476768	0.692355
SGPL1	0.10032	0.476799	0.692355
TMEM200A	0.237427	0.476936	0.692493
RP11-255F	-0.00494	0.476958	0.692493
LOC10012	0.02291	0.477085	0.692631
LINC00606	7.53E-04	0.477239	0.692807
ACSBG2	-0.01425	0.477326	0.692878
RP11-465I	-0.00564	0.477351	0.692878
C20orf166	0.022862	0.477384	0.692879
THRSP	-0.00702	0.47745	0.69292
RP11-214N	-0.01193	0.477476	0.69292
PCNXL2	-0.04958	0.47765	0.693126
ZNF462	-0.10102	0.47775	0.69318
SPACA6P	0.0159	0.477752	0.69318
LOC10192	-0.01892	0.47788	0.693211
JAKMIP2	-0.08445	0.477918	0.693211
ZFP42	-8.62E-04	0.477924	0.693211
ZFY	0.139175	0.478002	0.693211
PLA2G2F	-0.00683	0.478027	0.693211
CARD6	0.082831	0.478047	0.693211
NOS1AP	0.011577	0.478053	0.693211
CNGA1	-0.02661	0.478133	0.693211
TMEM198	-0.01704	0.478152	0.693211
ALX4	-0.00406	0.478163	0.693211
MB	0.03219	0.478172	0.693211
PTPRN2	0.055845	0.478192	0.693211
TRMT1	-0.08522	0.478216	0.693211
GPR12	-0.0135	0.478221	0.693211
PRSS27	-0.00274	0.478293	0.693269
GARS	0.067112	0.478394	0.693326
KIR3DS1	-0.00151	0.478417	0.693326
SERPINC1	-0.00119	0.478456	0.693326
LOC10050	-0.00204	0.478474	0.693326
CTD-2076I	-0.00241	0.478494	0.693326
FRRS1	-0.00108	0.478528	0.693326
PSG4	-0.00686	0.478584	0.693326
LOC10192	-0.00608	0.478619	0.693326
BPIFC	-0.00803	0.478676	0.693326
EME2	0.04134	0.478683	0.693326
SLC25A38	0.062356	0.478685	0.693326
GAS8	0.051989	0.478818	0.693473
KRCC1	0.084728	0.478854	0.693479
DLGAP1-A	-0.04981	0.47891	0.693513
CDK19	0.068818	0.479006	0.693606
LOC10192	0.012006	0.47914	0.693753
UBP1	-0.0552	0.479365	0.694033
RP11-483I	0.057616	0.47943	0.69408
RP1-142L7	-0.00155	0.47948	0.694107
KLHL25	-0.0177	0.479634	0.694284
ART1	-0.00803	0.479841	0.694401
DEPDC4	0.011759	0.479843	0.694401

GPR115	0.003369	0.479855	0.694401
LOC10050(	0.003608	0.479866	0.694401
LOC10106(	0.044268	0.479876	0.694401
LOC80154	-0.03709	0.479974	0.694496
LNPEP	-0.09711	0.480073	0.694594
ULBP2	0.017507	0.480153	0.694642
RIPPLY2	-0.00118	0.48017	0.694642
GPR83	-0.01234	0.480273	0.694744
ANKRD20A	0.010863	0.480311	0.694752
PTPN1	0.006892	0.480472	0.694926
IL4	0.008986	0.480503	0.694926
LOC10192	-7.25E-04	0.480528	0.694926
IFRD1	-0.09559	0.480647	0.695041
INADL	0.014454	0.480702	0.695041
PTEN	-0.04891	0.480703	0.695041
DLX6-AS1	-0.00981	0.480807	0.695145
EDRF1	-0.06098	0.480881	0.695205
LOC10192	-0.00118	0.480923	0.695219
GKN2	-0.00766	0.481013	0.695236
THAP1	-0.04958	0.481043	0.695236
RPRML	-0.04026	0.481094	0.695236
RP11-388M	0.001339	0.481094	0.695236
CTD-2366I	-0.01856	0.481095	0.695236
PBLD	-0.03649	0.481199	0.695311
C20orf181	-0.01109	0.481211	0.695311
CKLF	0.051585	0.481438	0.695593
EPO	-0.01632	0.481473	0.695597
LINC00632	-0.0012	0.48151	0.695603
C4orf47	0.004952	0.481589	0.695638
SULT1C4	0.067569	0.481598	0.695638
PMCHL1	-0.00108	0.481649	0.695665
SCRG1	-0.00144	0.481695	0.695685
ZGPAT	0.076742	0.481731	0.695692
SLFN13	0.120294	0.481796	0.695738
LOC10028	-0.0063	0.481905	0.69585
SPANXB1	-0.08252	0.481969	0.695895
PDCD6IP	0.039522	0.482014	0.695903
ITPRIPL1	-0.0875	0.482038	0.695903
LOC10192	-0.00943	0.482152	0.696021
LOC10105	6.76E-04	0.482207	0.696054
AMBRA1	0.020942	0.482309	0.696154
CATSPER1	-0.00944	0.482443	0.696302
GRTP1-AS	-0.00242	0.482559	0.696397
GPATCH4	0.012328	0.482639	0.696397
TCRBV12S	0.003425	0.482655	0.696397
CDCA2	0.099484	0.482665	0.696397
LOC10192	-0.00221	0.482772	0.696397
LOC10192	-0.00448	0.482787	0.696397
C12orf60	0.046348	0.482789	0.696397
GS1-279B7	-0.01131	0.482805	0.696397
LDHAL6B	6.27E-04	0.48283	0.696397
DKFZP434I	0.084422	0.482878	0.696397
WDR37	0.086568	0.482895	0.696397
GNG11	0.151078	0.482921	0.696397
MANF	-0.06354	0.482937	0.696397
RP11-753A	-0.0046	0.48296	0.696397
CTD-3126I	0.01538	0.483197	0.696694
CRIPAK	-0.05642	0.483246	0.696717

CXXC1P1	-0.00132	0.483316	0.696731
MROH1	-0.04549	0.48332	0.696731
LDHAL6A	-0.05462	0.483496	0.696882
EXOC3	-0.0553	0.483544	0.696882
CBX5	-0.07608	0.483606	0.696882
LINC00592	0.00615	0.483613	0.696882
TMEM198E	0.071508	0.483636	0.696882
LOC10050?	0.039924	0.483697	0.696882
CTD-3028I	-0.02344	0.483704	0.696882
PARD3B	0.003417	0.483718	0.696882
SLC29A2	0.007587	0.48374	0.696882
GPC4	0.031587	0.483747	0.696882
TAB3	0.0558	0.483799	0.696911
GLYATL1	0.044291	0.483911	0.697025
SLC44A4	-0.01077	0.483942	0.697025
DGKA	0.0565	0.484009	0.697074
KIAA0196	-0.09887	0.484058	0.6971
SLC5A1	0.005092	0.48419	0.697242
PRSS8	6.75E-04	0.484242	0.697263
KTN1	-0.08364	0.484268	0.697263
MED28	-0.05494	0.484633	0.697741
EXOC3L4	0.008273	0.484707	0.697775
COX5A	-0.05461	0.484733	0.697775
CYP27C1	0.009322	0.484769	0.697775
LOC10192!	-0.02254	0.484785	0.697775
SNCB	-0.01207	0.484881	0.697847
C6orf226	-0.049	0.4849	0.697847
TMCO5B	-0.0103	0.485048	0.697998
CDC23	-0.06454	0.485069	0.697998
CASQ1	-0.00139	0.485118	0.698022
SPIB	0.104046	0.485156	0.69803
LOC10272	-0.02071	0.485263	0.698138
MC1R	-0.03068	0.485376	0.698254
SERPINB3	0.001158	0.48542	0.69826
CNNM2	0.031072	0.485445	0.69826
SPHK2	0.033186	0.485557	0.698305
DEFB127	7.58E-04	0.485565	0.698305
TMC4	-0.0133	0.485593	0.698305
KRT36	-0.00114	0.485619	0.698305
ANKRD33	-0.00972	0.485637	0.698305
PLAC4	0.011571	0.485704	0.698355
ZNF470	0.032768	0.48587	0.698527
LOC10192!	-0.00152	0.485888	0.698527
LOC10050(	-0.01309	0.485938	0.698552
EXO5	0.043507	0.485997	0.698591
FGG	0.002044	0.486061	0.698611
DPPA2	-0.02749	0.486076	0.698611
EDC4	0.056658	0.486113	0.698618
DUSP19	-0.03171	0.486159	0.698634
NR2E3	-0.00702	0.486205	0.698634
MBTD1	-0.08732	0.486256	0.698634
TBX15	0.019391	0.486264	0.698634
BMP1	-0.00808	0.486301	0.698634
NAA11	-0.00258	0.486318	0.698634
ENTHD2	-0.02176	0.486525	0.698847
LOC38964	-0.03468	0.48653	0.698847
DNAJB13	0.015906	0.486633	0.698863
LOC10050?	-0.00411	0.486654	0.698863

SETX	0.08146	0.48666	0.698863
PTCRA	-0.07179	0.486684	0.698863
BRCA1	-0.0758	0.486722	0.698863
TTC30A	0.042153	0.486735	0.698863
LPXN	-0.08723	0.486809	0.698915
SNHG22	0.018959	0.486836	0.698915
CTD-2021I	-0.01816	0.486887	0.698942
TNFSF8	-0.07201	0.486972	0.699016
BCOR	-0.08467	0.487003	0.699016
P2RX6	-0.00456	0.487036	0.699016
ANGPT2	-0.06471	0.487255	0.699266
RHBDF1	-0.05188	0.487274	0.699266
RIPK4	-0.01347	0.487335	0.699278
C10orf40	0.00145	0.487347	0.699278
LOC10050(	-0.0131	0.487401	0.699309
BLID	-8.73E-04	0.487579	0.699519
ZGLP1	-0.03216	0.487643	0.699539
COLEC11	-0.04497	0.487658	0.699539
SCAI	-0.08214	0.487745	0.699574
CPNE9	-0.02604	0.487747	0.699574
RGS11	0.001606	0.487793	0.699593
TDRD6	0.087263	0.487875	0.699664
CAMK4	0.003999	0.487944	0.699685
RP1-100J1	0.023687	0.487954	0.699685
PAQR6	-0.06007	0.487997	0.6997
ZFPM2	-0.1386	0.488305	0.700075
FPGT	-0.08234	0.488323	0.700075
SMAD4	-0.10003	0.488477	0.700193
MDH1	0.045871	0.48848	0.700193
BGN	0.026738	0.488502	0.700193
HEXIM2	-0.07723	0.48861	0.700247
LOC10192(	-8.42E-04	0.488618	0.700247
MAGEB3	-0.00505	0.488637	0.700247
SIT1	0.024353	0.488775	0.700399
RNASE6	0.193491	0.488809	0.7004
TDRD3	0.063092	0.488956	0.700565
LZTS3	-0.07444	0.489214	0.700888
GABRB2	-0.01656	0.489281	0.700937
LINC01272	0.018653	0.489356	0.700999
ZBTB6	0.014672	0.489436	0.701068
LINC01355	0.049984	0.489495	0.701106
DYNLRB2	0.022578	0.489568	0.701139
LOC10192(	-0.00284	0.489594	0.701139
C9orf62	-0.01131	0.489615	0.701139
SCAMPER	-0.00186	0.489723	0.701247
LOC64648-	-0.00104	0.489759	0.701252
RP11-476I	-8.53E-04	0.489804	0.70127
ACTL10	-0.02817	0.489847	0.701286
RBBP4	-0.05303	0.489953	0.701359
USP45	-0.08492	0.489964	0.701359
ATP8B4	0.180462	0.490052	0.701439
CACNA1C	0.008003	0.490086	0.701441
SNX33	0.03202	0.490147	0.701441
LOC10192	0.004191	0.490171	0.701441
MBOAT2	-0.10421	0.490183	0.701441
RP11-329E	-0.00412	0.49025	0.701492
FGF11	0.010328	0.490361	0.701551
RP11-222K	-0.0016	0.490368	0.701551

AMHR2	0.0371	0.490395	0.701551
KLHL9	-0.02815	0.490422	0.701551
PRDX4	0.095334	0.490573	0.701712
MAPT-AS1	-0.01752	0.490599	0.701712
NUCB2	-0.06813	0.490862	0.702014
XAGE3	0.010288	0.490875	0.702014
LOC28343!	0.001498	0.490927	0.702042
LHFPL3	-0.00289	0.491058	0.702145
CATSPERG	-0.00224	0.491064	0.702145
LOC10013:	-0.00699	0.49111	0.702165
DLX2	0.015596	0.491161	0.702191
NR4A3	0.178489	0.491278	0.702313
RCOR1	-0.02857	0.491383	0.702416
COA7	0.077298	0.491538	0.702558
AHNAK	-0.02682	0.491567	0.702558
PHB	-0.0678	0.491579	0.702558
FAM181A-	-0.00181	0.491655	0.702558
LINC01214	-0.00778	0.491676	0.702558
ABCA6	-0.07322	0.491689	0.702558
ZFP1	0.005864	0.49172	0.702558
DACH2	0.003389	0.491742	0.702558
LOC10192!	0.001487	0.49179	0.702581
MAGI3	-0.0215	0.492201	0.703121
ACER1	-0.00766	0.49231	0.70323
ATP1A1-A	-0.0347	0.492379	0.703245
FAM227B	-0.02149	0.492385	0.703245
JARID2-AS	-0.00962	0.49246	0.703305
CEP76	-0.06602	0.492622	0.703381
NPFFR2	-0.00117	0.492646	0.703381
SERINC5	-0.10228	0.49267	0.703381
LOC14583	-0.00543	0.49268	0.703381
LOC10028!	0.0447	0.492696	0.703381
SMCP	-0.00466	0.492708	0.703381
PPIP5K1	-0.04504	0.492812	0.703449
PTTG2	0.017083	0.49282	0.703449
ANKRD35	-0.12537	0.492865	0.703466
ASPHD1	-0.0119	0.492898	0.703467
WNK2	0.009548	0.493048	0.703589
CREBRF	0.09353	0.493066	0.703589
STRADB	0.101915	0.493144	0.703589
C9orf64	-0.06129	0.493145	0.703589
SGK494	-0.08871	0.493146	0.703589
PRL	-0.01131	0.493218	0.703645
GNAZ	-0.13784	0.493269	0.703672
C8orf31	0.005339	0.493394	0.703801
USP37	-0.06141	0.493424	0.703801
RP5-1180E	0.030387	0.493572	0.703866
LOC57453!	-0.00139	0.493591	0.703866
GS1-304P7	-0.0033	0.4936	0.703866
LINC00641	0.008923	0.4936	0.703866
IL1F10	-0.0254	0.493965	0.70432
NKAIN4	-0.00207	0.494011	0.70432
FOXO1	-0.12768	0.494034	0.70432
LOC10192!	-0.00403	0.494048	0.70432
CCNH	0.055753	0.494217	0.704421
SLC25A47	-0.00915	0.494229	0.704421
EID2B	0.086169	0.494255	0.704421
GLYAT	-0.00123	0.494292	0.704421

OR51E1	-0.00605	0.494329	0.704421
POU1F1	-0.00748	0.494341	0.704421
CNTN4	0.029073	0.494347	0.704421
FOXE3	0.002251	0.49445	0.70444
KMT2C	0.100721	0.494458	0.70444
ACAD10	0.022864	0.494458	0.70444
RP11-669I	0.00325	0.494613	0.704604
DDX17	-0.10117	0.494638	0.704604
ZNF133	0.070578	0.494732	0.70469
LOC10012	0.037808	0.494764	0.70469
CRYBG3	0.11847	0.494862	0.704784
PHYH	-0.07416	0.494945	0.704855
NDST3	0.046817	0.495211	0.705028
RRM2	0.121749	0.495245	0.705028
CTB-31O2	-0.00166	0.495272	0.705028
BFSP1	-0.06103	0.495336	0.705028
RAB1B	0.061262	0.495341	0.705028
CALN1	-0.14929	0.495356	0.705028
ZNF93	0.041941	0.495381	0.705028
TRAC	-0.04389	0.495391	0.705028
ACMSD	-0.00262	0.495393	0.705028
NPY5R	-0.00278	0.495398	0.705028
IFT46	0.055077	0.49543	0.705028
ZNF365	-0.0047	0.495457	0.705028
RP11-73K	-0.03573	0.495666	0.70528
RBPJL	0.006446	0.495752	0.705318
STAC2	-0.01103	0.495758	0.705318
ZNF536	-8.32E-04	0.495861	0.705351
FIGN	0.206637	0.495873	0.705351
UBTD1	0.024654	0.49588	0.705351
CCDC18	-0.10435	0.495922	0.705366
LOC10192	-0.00204	0.495969	0.705386
HRH2	-0.01313	0.496047	0.70545
GABRP	-0.00186	0.496121	0.705472
AURKA	0.133238	0.496127	0.705472
MAP6	0.002714	0.496204	0.705535
CPEB2-AS	-0.00661	0.496276	0.705591
HIST1H2BL	-9.29E-04	0.496369	0.705676
RP5-890E1	0.005123	0.496473	0.705779
SLC5A4	6.38E-04	0.496588	0.705856
NKRF	0.030774	0.496593	0.705856
DLGAP3	-0.00878	0.496693	0.705874
RAG2	-0.20297	0.496696	0.705874
NAALADL1	0.104598	0.496711	0.705874
RPL23A	-0.02329	0.496736	0.705874
AP001063.	0.003338	0.497013	0.706222
MYH16	0.008154	0.49706	0.706242
SORBS2	-0.00119	0.497094	0.706244
BRE-AS1	0.211393	0.497215	0.706369
WDR25	0.066577	0.497328	0.706484
HEXDC	0.031073	0.497372	0.7065
NFKBIA	0.078314	0.497449	0.706563
CORIN	-0.00118	0.497561	0.706575
FAM26E	0.040693	0.497574	0.706575
GS1-124K	-0.00117	0.497595	0.706575
ANKEF1	-0.04208	0.497637	0.706575
OR7A5	0.116537	0.497669	0.706575
KCNF1	-0.03332	0.497711	0.706575

FBXO8	0.078097	0.497712	0.706575
PDZD2	-0.10631	0.497719	0.706575
CHST6	-0.00259	0.497895	0.706779
MRAP	-0.00151	0.497961	0.706811
SNX19	0.025101	0.497983	0.706811
USP27X-A:	-0.02397	0.498032	0.706814
SPRNP1	0.008534	0.498083	0.706814
TBCE	-0.04912	0.498101	0.706814
HIF3A	-0.0105	0.498151	0.706814
TBC1D27	0.010071	0.498167	0.706814
NLRP2	0.132176	0.498194	0.706814
CHTOP	-0.04237	0.498213	0.706814
CSH1	-0.01324	0.498348	0.706958
LOC25402ε	-0.00362	0.498381	0.706959
CITED4	0.034425	0.498454	0.707017
SKIV2L2	-0.04964	0.498585	0.707156
NTS	-0.01435	0.49863	0.707174
CYFIP2	0.108415	0.498855	0.707446
COMTD1	0.067689	0.499001	0.707607
STON1	-0.06079	0.499064	0.70765
NELFB	-0.06205	0.499136	0.707706
HNF4A	0.001098	0.49918	0.707722
CHST5	-0.00298	0.499246	0.707729
CCDC88C	0.079059	0.499251	0.707729
BCAS3	-0.06992	0.499351	0.707821
LSAMP	5.94E-04	0.499408	0.707821
HMGCS1	-0.04763	0.499413	0.707821
RGAG4	-0.0525	0.499446	0.707822
IL1B	0.119786	0.499519	0.707877
ZNF154	0.032788	0.499595	0.70794
LOC10192!	-0.00116	0.499739	0.708097
CD53	-0.10892	0.499866	0.708231
LOC10012ε	-0.01651	0.499986	0.708311
NFATC4	0.005208	0.499988	0.708311
PHF21B	0.001313	0.500049	0.70835
OAT	0.072623	0.500123	0.708377
ZNF396	0.002189	0.500133	0.708377
SH3BGR	0.065963	0.500245	0.708488
FGF12	-7.58E-04	0.500276	0.708488
FAM198A	0.001695	0.500439	0.708584
CLEC2D	0.048838	0.500443	0.708584
ZCCHC4	0.013376	0.500466	0.708584
EPN1	-0.04848	0.500475	0.708584
COG4	-0.05845	0.500659	0.708762
LINC00176	0.01083	0.500667	0.708762
LIPT1	-0.05874	0.500721	0.708793
ELN	-0.01449	0.500765	0.708793
RP11-1137	-0.00761	0.500795	0.708793
CGN	-0.00804	0.500843	0.708793
TRIML1	-0.00114	0.500853	0.708793
ZBTB11	-0.08121	0.500912	0.708793
RP11-930F	-0.01761	0.500918	0.708793
C15orf39	0.07476	0.500988	0.708847
NUTM2A-/	0.093227	0.501046	0.708848
CAPN10-A	-0.02075	0.501055	0.708848
C3orf67	-0.0346	0.501169	0.708923
PP13439	-0.06478	0.501185	0.708923
LEPROT	-0.06519	0.501207	0.708923

ZNF500	-0.08194	0.501238	0.708923
COLGALT2	-0.05457	0.501418	0.70913
RP11-515C	-0.01028	0.501559	0.709238
CHAC2	-0.1195	0.501573	0.709238
PPP2CA	-0.09544	0.501637	0.709238
LINC00240	0.016595	0.501722	0.709238
FBXO39	-0.01104	0.501733	0.709238
CPNE7	0.041696	0.501741	0.709238
GBX2	-0.00231	0.501783	0.709238
VRTN	0.024821	0.501786	0.709238
RP11-669C	-0.02092	0.501801	0.709238
TSR2	0.06579	0.501822	0.709238
DCN	0.059582	0.501866	0.709255
SSX2B	-0.00294	0.501995	0.709346
RTFDC1	0.048681	0.501996	0.709346
SPATA4	6.19E-04	0.502048	0.709348
LINC01007	-0.00671	0.502089	0.709348
INPP5A	0.032796	0.50216	0.709348
RP11-787E	-0.00647	0.502165	0.709348
LINC00605	0.004634	0.50218	0.709348
DUSP2	0.159005	0.502201	0.709348
STAU2	0.111431	0.502259	0.709348
PRCD	-0.04506	0.502261	0.709348
TTLL1	0.065845	0.502346	0.709348
RNF151	-0.02332	0.502348	0.709348
UBL4B	-0.00614	0.502358	0.709348
GPR111	-0.00112	0.502621	0.709673
PRDX6	0.025788	0.50274	0.709795
MSN	0.073114	0.502781	0.709807
NALCN	-0.00515	0.502931	0.709972
TTPA	-8.21E-04	0.502964	0.709972
SERPINB9	-0.14369	0.503019	0.710004
STX3	-0.10045	0.503054	0.710006
LOC440602	-0.01542	0.50309	0.710011
FOXN1	-0.00233	0.50317	0.710042
FAM92A1	0.119755	0.503177	0.710042
HDLBP	-0.04179	0.503307	0.710178
CTNNAL1	0.113717	0.503375	0.710228
ITPR1-AS1	0.052193	0.503482	0.710333
LINC00557	8.79E-04	0.503582	0.710393
MACROD2	-0.00119	0.503611	0.710393
LINC00441	-0.00843	0.503623	0.710393
KRT78	0.016404	0.503828	0.710623
CTD-2547I	-0.02081	0.503872	0.710623
LOC28617	-0.00961	0.503939	0.710623
GSTM5	-0.19198	0.503944	0.710623
RP11-263K	-0.00683	0.50395	0.710623
LRRC17	-0.02479	0.504032	0.710692
DQ586822	0.0059	0.504255	0.710895
HEY2	0.010835	0.50426	0.710895
C2orf40	-0.09781	0.504274	0.710895
HNRNPAB	0.051859	0.504312	0.710902
CDIP1	0.05997	0.504422	0.711009
HNF4G	-0.00794	0.504453	0.711009
GOLPH3	0.065657	0.504511	0.711044
TEX36	8.27E-04	0.504629	0.711121
ZNF880	0.075599	0.504631	0.711121
ZNF322	-0.07586	0.504709	0.711184

12-Sep	-0.00815	0.504838	0.71132
LOC10192	0.001261	0.504951	0.711433
C2orf73	-0.0112	0.505009	0.711467
SNRPB2	-0.03088	0.505164	0.71164
R3HDML	0.00128	0.505421	0.711914
LOC10050	0.002444	0.505509	0.711914
TP53BP1	-0.04761	0.505544	0.711914
C9orf135	0.001929	0.505556	0.711914
KCNK4	-0.00109	0.505587	0.711914
RP11-533E	0.024922	0.505596	0.711914
SLC38A10	-0.03276	0.505598	0.711914
FAU	0.038353	0.505622	0.711914
DCTN1-AS	0.00412	0.50572	0.712006
MARVELD3	-0.00464	0.506009	0.712367
IRX4	-0.00439	0.506067	0.712397
ASB9	-0.10527	0.506108	0.712397
PCDHGC5	-0.00104	0.506135	0.712397
NT5DC4	-9.91E-04	0.506192	0.712397
SELE	0.07211	0.506195	0.712397
AADACL2	-0.00219	0.506253	0.712432
CNNM4	0.045976	0.506313	0.71247
DIRAS1	0.018534	0.506511	0.712661
PLIN2	-0.0544	0.506515	0.712661
UBE2E2-AS	-0.002	0.506547	0.712661
DEFB1	-0.03305	0.506596	0.712683
RP3-452M	6.07E-04	0.506633	0.712689
C11orf70	-0.00283	0.506697	0.7127
AC087501.	0.007259	0.506707	0.7127
LINC01107	-0.00523	0.506848	0.712853
ANKRD13C	0.073186	0.507042	0.713079
ABRACL	-0.05993	0.507151	0.713154
NOVA2	-0.00957	0.507161	0.713154
FILIP1	0.00648	0.507331	0.713347
NEGR1	0.152846	0.507418	0.713423
OCLN	-0.10191	0.507591	0.713483
COL4A5	0.152506	0.507604	0.713483
NOP9	-0.04159	0.507618	0.713483
LOC10192	-0.01336	0.507677	0.713483
ENOX2	0.041219	0.507677	0.713483
GCGR	-0.00733	0.50769	0.713483
SLCO3A1	0.071539	0.507734	0.713483
HOXB2	0.100473	0.507746	0.713483
PXN-AS1	0.067169	0.50779	0.713483
RFPL1S	0.097927	0.507815	0.713483
SYTL4	0.0448	0.507823	0.713483
RP11-414H	0.001854	0.508046	0.71355
MMEL1	-0.07881	0.508049	0.71355
CTD-2561I	-0.06225	0.508057	0.71355
Igk	0.004653	0.508118	0.71355
EPB41L5	-0.04247	0.508143	0.71355
LINC00879	-0.00311	0.508169	0.71355
CD300LF	0.09688	0.508172	0.71355
ABI1	-0.05494	0.508173	0.71355
LOC10192	-8.08E-04	0.508192	0.71355
MADCAM1	0.021726	0.5082	0.71355
U2AF1	-0.09285	0.508282	0.713619
LCE1B	-0.00663	0.508393	0.713689
TSPAN18	-0.05667	0.508398	0.713689

STAP2	-0.0201	0.508554	0.713861
EAF2	0.025396	0.50873	0.714062
LOC10004!	0.054482	0.508775	0.714078
CYP4F2	-0.10016	0.508808	0.714079
VPS37B	0.09029	0.508922	0.714192
MKI67	0.120844	0.508969	0.714212
AGT	-0.0221	0.509041	0.714268
LOC10192!	-0.00375	0.509102	0.714307
EXOC4	-0.09074	0.509298	0.714535
GEMIN8	-0.06007	0.509374	0.714577
LINC01097	0.005998	0.509394	0.714577
ENTPD3	-0.0049	0.509437	0.714582
IGFL1	6.23E-04	0.509463	0.714582
CXCL3	0.181598	0.509751	0.714939
DLGAP2	-0.01489	0.509928	0.715059
LOC64285	0.060714	0.509953	0.715059
ARHGAP11	-0.09542	0.509963	0.715059
TMEM218	-0.04344	0.509969	0.715059
AP1G2	-0.03807	0.510135	0.715179
LOC10272	-0.08188	0.510149	0.715179
ACRBP	0.148817	0.510153	0.715179
KCNMB1	0.02893	0.510241	0.715242
LOC10013	-0.00217	0.510264	0.715242
PEMT	0.076525	0.51035	0.715316
CCDC105	-0.01173	0.510418	0.715365
NRD1	-0.04786	0.510463	0.715371
FOXA1	0.005784	0.510488	0.715371
WDR38	8.22E-04	0.51056	0.715424
CHRM3	-0.11278	0.510624	0.715468
ENO1-AS1	-0.04886	0.510791	0.715596
SMIM15	-0.0469	0.510797	0.715596
IYD	-0.00581	0.510814	0.715596
WFIKKN1	-0.00945	0.510954	0.715724
RELL2	-0.0228	0.510972	0.715724
ZNF512	-0.06052	0.511014	0.715738
LOC28351	-0.00524	0.511184	0.715929
SPATA22	0.118408	0.511308	0.716051
F13B	-0.004	0.511337	0.716051
LOC10013	0.009107	0.511517	0.716257
TP53INP1	-0.02522	0.511594	0.716318
TEF	-0.00884	0.511666	0.716371
FATE1	0.0183	0.511816	0.716536
SMPD1	-0.0117	0.511982	0.716722
RP11-846E	0.10627	0.51209	0.716827
RP11-375I	0.00119	0.512172	0.716881
ZNF436	-0.04853	0.512195	0.716881
CTAGE1	-0.0039	0.512285	0.716951
IP6K1	0.054084	0.512337	0.716951
WWC2-AS	0.006759	0.512344	0.716951
ARID4B	-0.04928	0.512486	0.717103
LOC10192	0.004502	0.512565	0.717167
LOC15112	-0.00137	0.51264	0.717226
ATOH7	-0.00222	0.512741	0.717252
CXXC1	-0.04984	0.512746	0.717252
C2orf57	0.006838	0.512785	0.717252
GPR150	-0.00764	0.512836	0.717252
ZNF35	0.076515	0.512918	0.717252
DPPA5	-0.00363	0.512923	0.717252

EIF1AD	0.053069	0.512977	0.717252
TOP1P2	-0.00112	0.513015	0.717252
BTF3P11	0.007578	0.513022	0.717252
LINC01359	0.005346	0.513073	0.717252
GPR87	-0.00116	0.513106	0.717252
LINC00029	-0.0062	0.513148	0.717252
RASL10B	0.024558	0.513173	0.717252
EDNRB-AS	0.004985	0.513181	0.717252
LGI4	-0.01268	0.513193	0.717252
KIR2DL5A	0.023582	0.513216	0.717252
LOC10192	-0.01239	0.513244	0.717252
KANSL3	-0.02095	0.513255	0.717252
CCBE1	-0.00126	0.513322	0.717295
TIGD5	0.0499	0.513368	0.717295
TIGD1	-0.09453	0.513385	0.717295
KRT38	-0.00749	0.513453	0.717299
CYB5D1	0.065686	0.513454	0.717299
PBK	0.189039	0.51366	0.717541
ROPN1B	0.01138	0.513758	0.717631
IQCG	0.045301	0.513864	0.717667
SHANK1	-0.00352	0.513894	0.717667
LOC10013	0.007254	0.513911	0.717667
KRTAP19-	-0.00545	0.513939	0.717667
SNRPB	0.067616	0.513971	0.717667
PGLS	0.045957	0.513983	0.717667
RHPN1-AS	-0.032	0.514118	0.717791
ZSWIM2	6.62E-04	0.514156	0.717791
CCDC182	-0.00788	0.514171	0.717791
LINC00920	-0.0256	0.514212	0.717802
LOC10192	-0.0028	0.514479	0.718129
TTTY15	0.107846	0.514545	0.718174
C6orf164	0.044173	0.514632	0.71825
SEMA3B	-0.01556	0.514873	0.718515
NCAPD3	0.059473	0.514889	0.718515
TRIB1	-0.00183	0.515052	0.718697
LOC10192	-0.00148	0.515109	0.71873
LINC00842	-0.00297	0.51525	0.718763
RARG	-0.00899	0.51525	0.718763
SPG7	-0.00714	0.515299	0.718763
AC007292.	-0.01356	0.515324	0.718763
SPTBN2	-0.0122	0.515335	0.718763
EFHD1	-0.00435	0.515371	0.718763
LOC10192	0.002386	0.515394	0.718763
EPCAM	-0.30277	0.515398	0.718763
SLC51B	-0.00745	0.515542	0.718881
C2CD4A	0.001304	0.515561	0.718881
NCOA1	0.045394	0.515582	0.718881
LOC14870	-0.0058	0.515688	0.718981
WAC-AS1	-0.05916	0.515731	0.718995
BRINP1	0.003651	0.515964	0.719274
CHRNA4	-7.53E-04	0.516112	0.719433
PLEKHN1	-0.01735	0.516173	0.719438
TSNAX	0.050116	0.516181	0.719438
TSNARE1	0.036249	0.516246	0.719482
SLC23A3	-0.01872	0.516547	0.71977
GSC	-0.00344	0.516556	0.71977
TSSK2	-0.00455	0.516575	0.71977
C3orf35	0.008905	0.516586	0.71977

PTGES2	0.083486	0.516828	0.720036
DSG3	-0.00243	0.516848	0.720036
MAFF	0.176438	0.516876	0.720036
PNOC	0.087642	0.517021	0.720116
SMIM22	-0.0055	0.517039	0.720116
GNAI3	0.063257	0.517061	0.720116
IL1A	0.061701	0.517089	0.720116
C22orf15	-0.01516	0.5171	0.720116
FLJ21408	-0.00435	0.51716	0.720118
RP11-1277	-0.00896	0.517168	0.720118
DEPDC1B	-0.10641	0.517203	0.720121
KRTAP3-1	0.00588	0.517414	0.720368
COL12A1	5.75E-04	0.517537	0.720454
CAPN8	0.008254	0.517543	0.720454
PAIP2B	0.077042	0.517582	0.720462
PCIF1	-0.08419	0.517645	0.720504
LOC102540	-0.00548	0.517792	0.720663
LY6H	0.01963	0.517912	0.720782
HPRT1	-0.02216	0.517993	0.72085
OR2C1	-0.00298	0.518097	0.720877
MAGI1	-0.00302	0.518115	0.720877
MC5R	0.001393	0.518132	0.720877
TRAP1	-0.04445	0.518146	0.720877
HMGN2P4	-0.00874	0.518385	0.721155
BC023201	-0.00116	0.518413	0.721155
MCM6	-0.05499	0.518493	0.72122
LOC100130	-0.05234	0.518531	0.721227
ALPK3	0.068992	0.51857	0.721235
MID1	0.10322	0.518627	0.721268
ATAD2	0.076512	0.518805	0.721469
POU3F3	-8.20E-04	0.518873	0.721518
TPP2	-0.08245	0.519134	0.721834
MEGF11	8.03E-04	0.519205	0.721886
DHRS11	0.107699	0.519294	0.721931
LOC100500	-0.00107	0.519398	0.721931
FGF20	-0.01878	0.5194	0.721931
VDAC3	0.053285	0.51942	0.721931
LOC100291	0.06332	0.519428	0.721931
ALOX12	0.164859	0.519437	0.721931
ITIH3	-0.00503	0.519485	0.721952
ZNF616	-0.06583	0.51961	0.722021
LRRC57	0.030703	0.519642	0.722021
HIST1H4J	-0.09114	0.519703	0.722021
CEP85	-0.03966	0.519709	0.722021
LLGL1	0.032319	0.519734	0.722021
LOC100507	0.004871	0.519802	0.722021
OR1G1	8.59E-04	0.519852	0.722021
LOC101930	-0.0204	0.519862	0.722021
FAM162B	-0.06406	0.519865	0.722021
WDR83	0.069448	0.519869	0.722021
KIF9	-0.03911	0.519938	0.722057
MAGEB6	0.004341	0.519966	0.722057
LINC00342	0.079975	0.519994	0.722057
PLCH1-AS	-0.00105	0.52006	0.722089
LOC102723	0.00509	0.520084	0.722089
DSCAM-AS	-0.0154	0.52028	0.722315
PSMD12	-0.04891	0.52038	0.722382
C20orf62	-0.00253	0.520395	0.722382

GIT1	-0.0569	0.520484	0.722387
DST	0.021103	0.520513	0.722387
LSMEM1	-0.07064	0.520538	0.722387
TSPAN8	-0.01649	0.520546	0.722387
BC015159	6.21E-04	0.520565	0.722387
PECR	0.031727	0.520776	0.722558
LINC00184	-0.01604	0.5208	0.722558
CD226	-0.14308	0.520823	0.722558
ETFA	0.052861	0.520854	0.722558
C1orf68	0.004783	0.520856	0.722558
RBSG3	-0.00212	0.521028	0.722751
NMUR1	-0.00897	0.521121	0.722833
RPF1	-0.08122	0.521302	0.723038
NR2F1-AS.	0.012319	0.521387	0.72311
PNLIP	-1.00E-03	0.521655	0.723417
COMMD5	0.033848	0.521676	0.723417
COLQ	0.057158	0.521783	0.72352
LOC10192!	0.055721	0.521854	0.723572
LOC10193(	-0.01359	0.521997	0.72367
SMTNL2	0.003706	0.522012	0.72367
ZNF416	-0.06011	0.522029	0.72367
LOC10272!	-0.01499	0.522059	0.72367
FSIP2	-0.00321	0.522219	0.723715
TMEM182	-0.03469	0.522234	0.723715
LOC10050	-0.05026	0.522252	0.723715
PLCB4	-0.14567	0.522284	0.723715
LRRC49	-0.06941	0.522285	0.723715
OR2A4	-0.00496	0.522307	0.723715
STX1B	-0.00345	0.522342	0.723715
TNFRSF12/	0.050771	0.522389	0.723715
VWA8-AS:	-0.00593	0.522392	0.723715
RP11-489(	-0.0064	0.522443	0.72374
TSPAN32	-0.08361	0.522649	0.723979
WBP5	0.087695	0.522689	0.723985
ADAMTS1:	-0.00859	0.522756	0.723985
GALNT12	0.080364	0.522781	0.723985
MLKL	0.067243	0.522787	0.723985
LOC28588!	-0.00233	0.522944	0.724156
ASB11	0.001301	0.523079	0.724274
MADD	-0.05423	0.523117	0.724274
GAP43	-8.80E-04	0.523135	0.724274
SH3PXD2A	-0.00353	0.523163	0.724274
LINC01431	0.001798	0.523303	0.724422
PAX5	-0.13977	0.523421	0.724494
KCNK9	-0.00545	0.523453	0.724494
ANKRD30E	-0.00532	0.523456	0.724494
TYR	-7.54E-04	0.523553	0.724582
KRT15	-0.00962	0.523672	0.724642
EXOSC7	-0.05502	0.523711	0.724642
UQCC1	-0.04805	0.523721	0.724642
IGF2-AS	0.006751	0.52373	0.724642
IRGC	-0.00453	0.523816	0.724672
LYG2	-0.00573	0.523836	0.724672
PP2672	5.94E-04	0.523852	0.724672
AC068138.	-6.70E-04	0.523966	0.724783
ARHGAP1!	0.072649	0.524035	0.724833
DICER1-AS	-0.02859	0.524086	0.724856
CASQ2	-0.00773	0.52428	0.725079

ALK	-0.01531	0.524406	0.725182
C3orf56	-0.0132	0.524421	0.725182
ANKRD23	-0.06527	0.52448	0.725217
CTA-29F1:	-0.09213	0.524606	0.725344
CCDC17	0.010742	0.524698	0.725425
ZDHHC7	0.05025	0.52475	0.725451
IRX1	-0.16056	0.525008	0.725761
WDR92	0.063626	0.525211	0.725968
PRSS33	-0.01257	0.525229	0.725968
MOGAT2	0.003842	0.525258	0.725968
TMEM8B	0.017423	0.525495	0.726215
CCR8	-0.00833	0.525515	0.726215
MATN3	-0.0052	0.525568	0.726215
DGCR5	0.00174	0.525571	0.726215
OR7E14P	-0.00614	0.52562	0.726236
MAP3K5	0.04746	0.525699	0.7263
HSH2D	0.101207	0.52602	0.726696
LOC10050(	7.19E-04	0.526195	0.72684
PARS2	-0.07275	0.526206	0.72684
PAPD7	-0.06431	0.526256	0.72684
ST7L	0.052465	0.526258	0.72684
TAS2R41	5.96E-04	0.526364	0.72694
KRTAP11-:	9.60E-04	0.526432	0.726987
KIAA0408	0.001145	0.526473	0.726997
MIR622	0.002914	0.526555	0.727065
DPEP3	0.204724	0.526685	0.727161
LOC44057(	0.010046	0.526714	0.727161
ZNF484	-0.00481	0.526732	0.727161
AMPH	0.008278	0.526759	0.727161
LEKR1	-0.02852	0.52694	0.727263
ACTR5	0.060864	0.526948	0.727263
ZNF584	-0.063	0.526968	0.727263
OVGP1	-0.06062	0.526968	0.727263
ASCL2	0.058412	0.527001	0.727263
ACTN3	-0.00278	0.527169	0.727449
L1CAM	0.004177	0.527203	0.727449
ARID3B	0.066624	0.527564	0.72789
SLCO4C1	-0.04919	0.52759	0.72789
MRPL46	-0.05667	0.527748	0.728036
BC047364	-0.01276	0.527763	0.728036
HLA-DOA	0.021173	0.527836	0.728079
SH3GLB2	-0.02905	0.527862	0.728079
RAB7B	-0.13025	0.527933	0.728102
PTPN7	0.064224	0.527947	0.728102
SLC22A1	0.020364	0.527979	0.728102
LINC01268	0.163864	0.528049	0.728153
LOC10192!	5.87E-04	0.52811	0.72819
RP11-480/	-0.07679	0.528334	0.728413
CST4	0.01691	0.528339	0.728413
LOC73096:	-0.01347	0.528425	0.728484
HCP5B	-0.00916	0.528542	0.728597
FAM200A	0.048822	0.528573	0.728597
METTL20	0.007682	0.528807	0.728873
LOC10013(	-0.02756	0.528887	0.728883
UNC93A	-0.00386	0.528917	0.728883
LOC10013:	-8.54E-04	0.528945	0.728883
NLGN2	0.004074	0.52895	0.728883
ADAMTSL!	0.013278	0.529031	0.728949

HIGD1B	0.005201	0.529089	0.728957
MMP26	-0.00645	0.529104	0.728957
ANKZF1	-0.05316	0.529234	0.729078
ANKRD18E	-0.01314	0.529265	0.729078
MYL12B	0.047775	0.529293	0.729078
ONECUT1	-0.00227	0.529332	0.729086
PEG10	0.023328	0.529439	0.729131
ZNF704	0.028666	0.529465	0.729131
TEX101	-0.01973	0.529466	0.729131
CA6	-0.00627	0.529532	0.729176
CRLF3	0.035988	0.529665	0.729288
LOC10050(	0.002785	0.529685	0.729288
SV2B	0.010885	0.529714	0.729288
UPK1A	-0.01734	0.529831	0.729402
HMGXB4	0.048432	0.530086	0.729706
NPTX2	-0.09282	0.530248	0.729883
RP11-31K2	-0.0035	0.530283	0.729883
CDKL5	0.00405	0.530316	0.729883
TPCN2	-0.04992	0.53064	0.730147
NOL6	-0.01701	0.530681	0.730147
TAF1B	0.056217	0.530701	0.730147
CPA3	-0.16505	0.530732	0.730147
C8orf12	-8.85E-04	0.530755	0.730147
ALX1	-0.00974	0.530768	0.730147
HMMR	-0.12086	0.530769	0.730147
LOC10013	-0.00952	0.530777	0.730147
FOXN3-AS	-0.00403	0.53086	0.730215
ANKRD13D	0.066799	0.53099	0.730347
GAS5	0.044193	0.531102	0.73044
TNFSF18	-0.00389	0.531125	0.73044
SIAH1	-0.04847	0.531304	0.73064
ATP6V0E2-	0.024255	0.531427	0.73076
KCNT2	0.038847	0.5315	0.73076
CASC10	0.057755	0.531513	0.73076
RP11-1109	-0.0298	0.531527	0.73076
TBX5-AS1	5.46E-04	0.531615	0.730794
NAF1	0.094558	0.531618	0.730794
SRD5A3	-0.06698	0.531668	0.730796
CARKD	-0.0685	0.531687	0.730796
LIMA1	0.067314	0.531772	0.730865
ATP6V1G2	-0.04543	0.531849	0.730926
C10orf85	0.012492	0.531937	0.73097
IRX3	-0.1431	0.531972	0.73097
DECR2	-0.06022	0.531983	0.73097
ZNF578	-0.08894	0.532064	0.730992
NFE4	0.013189	0.532081	0.730992
SLC6A13	0.029373	0.5321	0.730992
RP11-297L	0.021993	0.532213	0.73106
APOL5	0.003406	0.532231	0.73106
LINC00112	-0.00117	0.532256	0.73106
ROR1-AS1	-0.01313	0.532285	0.73106
SNORA37	-0.00764	0.532423	0.731204
LINC-PINT	-0.0634	0.532521	0.731292
RNF113A	-0.05607	0.532581	0.731327
PCDP1	-6.40E-04	0.532704	0.731418
ARL10	-0.01647	0.532734	0.731418
EPHA2	-0.01038	0.532748	0.731418
LOC10192	-0.01127	0.533086	0.7318

PARP15	0.023938	0.533109	0.7318
CNTROB	0.019538	0.533128	0.7318
RFX5	-0.03706	0.533273	0.731953
FAM32A	0.044313	0.53364	0.73241
ACKR3	-0.07653	0.533747	0.732427
CCDC148-	5.48E-04	0.533779	0.732427
LOC10192!	-7.45E-04	0.53378	0.732427
SGTA	0.044938	0.533787	0.732427
FAM50B	-0.08375	0.533979	0.732644
AK024936	0.039978	0.534047	0.732691
C17orf89	0.025939	0.534565	0.733354
C11orf63	-0.02585	0.534631	0.733386
KRTAP4-5	-0.01059	0.534655	0.733386
AGPAT3	0.083995	0.534809	0.73355
PTGER4P2-	-6.72E-04	0.534927	0.733666
SLC25A27	0.064232	0.535058	0.733799
LINC00323	0.002603	0.535105	0.733817
DGKK	-0.01131	0.53518	0.733873
IFNG	7.79E-04	0.535239	0.733907
GAGE3	-7.43E-04	0.535344	0.734004
CTC-360P!	0.012091	0.535538	0.734181
CTC-462L7	0.016333	0.535567	0.734181
ERVH-6	-0.01181	0.535575	0.734181
TRIM13	-0.06512	0.535642	0.734181
CHST8	-0.0167	0.535655	0.734181
SHF	0.038809	0.535731	0.734181
LOC10013:	-0.00247	0.53575	0.734181
EFNA1	0.089224	0.535766	0.734181
WDR60	-0.03409	0.53581	0.734181
USP16	0.040765	0.535844	0.734181
RP11-493L	-0.00253	0.535847	0.734181
CNGB1	-0.00897	0.535881	0.734181
ADAM22	-0.00515	0.535913	0.734181
CSNK1G3	0.062964	0.536023	0.734286
LACE1	-0.01066	0.536117	0.734367
RP11-79P5	-0.01375	0.536165	0.734387
PLK3	-0.06843	0.536304	0.73453
LINC01206	-0.00954	0.536352	0.73455
DIMT1	0.047019	0.536448	0.734635
LOC28421!	0.049362	0.536602	0.73473
RASGEF1C	-0.00656	0.536614	0.73473
CTD-2534I	0.007739	0.536637	0.73473
WNT7B	-0.02088	0.536653	0.73473
IL12RB2	0.109513	0.537393	0.735697
RP11-138I	0.005845	0.537712	0.736087
DAPK2	-0.01721	0.537751	0.736094
MNS1	-0.08452	0.537927	0.736288
PRKAR2B	0.129787	0.538021	0.736314
AJAP1	-8.41E-04	0.538038	0.736314
HTR1D	-7.60E-04	0.538048	0.736314
DUX1	-0.01754	0.538095	0.736331
RBM27	0.050286	0.538202	0.7364
IGSF5	5.53E-04	0.538213	0.7364
TMEM154	0.086989	0.538335	0.736521
WEE2-AS1	-0.0095	0.538402	0.736534
NFX1	0.051827	0.538441	0.736534
OR51B6	-0.00129	0.538447	0.736534
ENDOU	0.004505	0.53859	0.736646

LINC00355	0.03517	0.538622	0.736646
CCDC28B	0.045648	0.538631	0.736646
DNAH2	0.029728	0.538819	0.736783
ADAL	-0.04288	0.538834	0.736783
KCND2	0.014272	0.538837	0.736783
LOC10192ε	-0.01075	0.538867	0.736783
SIL1	0.079523	0.538953	0.736796
RP11-359K	-0.00105	0.538969	0.736796
NASP	-0.05861	0.538979	0.736796
PTCH1	9.09E-04	0.53913	0.736956
AA06	-0.0212	0.53917	0.736964
ZNF32-AS:	-8.77E-04	0.539224	0.736992
MBP	-0.01913	0.539372	0.737147
GOSR1	0.049237	0.53941	0.737153
AIM1	-0.13649	0.539478	0.737162
MATK	0.091351	0.539509	0.737162
RFX4	0.002129	0.539519	0.737162
UBN1	0.04178	0.539595	0.737197
PCGF3	0.032387	0.539625	0.737197
ADD2	-0.09882	0.539668	0.737197
SIRT4	-0.01953	0.539693	0.737197
KCNH2	0.141454	0.53972	0.737197
CARTPT	0.00405	0.53978	0.737197
DACT3-AS	0.003002	0.539782	0.737197
IGFLR1	-0.076	0.539884	0.737221
MC3R	-0.00264	0.539926	0.737221
SLC35D1	0.069816	0.539931	0.737221
IL21	-7.30E-04	0.539936	0.737221
ATG101	0.068414	0.539985	0.737241
TAS2R39	5.98E-04	0.540257	0.737566
LOC10192(	0.018372	0.540322	0.737608
PODXL2	0.010848	0.540468	0.737761
AGO4	0.055451	0.540778	0.738114
GPR180	-0.04671	0.540795	0.738114
MYH1	-0.00202	0.540946	0.738273
INTU	-0.10677	0.541185	0.738511
LOC10192ε	0.013348	0.541188	0.738511
IL20RB	0.028737	0.541264	0.738567
PGLYRP2	0.020623	0.541298	0.738567
SIAE	-0.01206	0.541348	0.738568
GJB4	-0.0088	0.541366	0.738568
SCNN1D	0.009392	0.541438	0.738619
SENCR	0.037656	0.541486	0.738638
RP11-102L	-0.01642	0.541602	0.738727
C6orf120	0.042739	0.54162	0.738727
SNX32	0.016814	0.541716	0.738812
KRT13	0.078024	0.541836	0.738929
ZNF157	-0.00974	0.541873	0.738933
ZNF718	-0.06517	0.54233	0.73951
SOX11	-8.11E-04	0.542388	0.739543
ARHGAP3(	7.24E-04	0.542469	0.739604
DOC2B	-0.01963	0.542502	0.739604
DUSP7	0.033707	0.542552	0.739627
LINC00958	-0.01537	0.542628	0.739665
HARS2	0.06815	0.542649	0.739665
ANO2	-0.0072	0.542798	0.739799
LYST	-0.07148	0.542815	0.739799
RP3-412A(	-0.0115	0.542928	0.739907

GADD45GI	-0.00145	0.543008	0.739968
C5AR2	0.020052	0.543135	0.740095
FRS2	-0.03215	0.543294	0.740219
RNF43	0.041306	0.543325	0.740219
LINC01426	0.002931	0.543329	0.740219
LINC00482	-0.02887	0.543595	0.740506
CALHM2	-0.08544	0.543608	0.740506
UBQLNL	0.030247	0.543703	0.740585
CCDC28A	0.036724	0.543734	0.740585
SLC6A18	-0.01042	0.543962	0.740849
ASXL2	0.07799	0.544175	0.741092
MAOB	7.63E-04	0.544212	0.741097
TPST1	-0.0673	0.544384	0.741267
RBMS3-AS	9.61E-04	0.544406	0.741267
ZDHHC6	-0.03644	0.544519	0.741374
ACSM2B	0.001669	0.544589	0.741424
SCG2	0.032386	0.544697	0.741486
C9orf40	0.090716	0.544706	0.741486
CHIA	-0.00445	0.544738	0.741486
RAD1	0.0564	0.54519	0.742054
ARFGAP2	0.028235	0.545254	0.742095
LOC10050(	-0.00492	0.545395	0.742241
SLC35G1	0.05438	0.545467	0.742251
ENDOD1	-0.08554	0.545542	0.742251
LRRC61	-0.05262	0.545543	0.742251
TTLL2	0.001554	0.545553	0.742251
CPOX	0.090779	0.545606	0.742251
CLCN3	0.076681	0.545608	0.742251
ALLC	-0.00941	0.545804	0.74247
SEMA6D	0.002129	0.546009	0.742702
LIMK1	0.00718	0.546059	0.742724
EYS	0.001975	0.546202	0.742871
RP11-471(	0.009852	0.546271	0.742918
GS1-600G8	-0.00295	0.54636	0.742993
NLRP13	0.005864	0.546495	0.74313
ZNRD1-AS	-0.06711	0.54653	0.743131
LOC10192	-0.0115	0.546588	0.743163
SHOX	0.009875	0.546676	0.743236
DSG2	-0.10236	0.546814	0.743338
DOCK4-AS	-0.00846	0.546819	0.743338
LA16c-358	-0.03612	0.546871	0.743361
LMO1	-0.02403	0.546917	0.743373
MRPS17	0.083927	0.546948	0.743373
RUSC1-AS	-0.05245	0.547084	0.743511
RECQL4	0.080603	0.547139	0.743538
NCK1	0.034545	0.547249	0.743621
SEMA6C	-0.02334	0.547268	0.743621
AF213884.:	-0.03549	0.547443	0.743812
MTG1	-0.06929	0.547509	0.743855
TAS2R4	-0.00281	0.547575	0.743899
NAGS	0.007281	0.547705	0.744028
RP11-413E	-0.00189	0.547798	0.744081
TTC7A	0.089001	0.547813	0.744081
PCDHB13	-0.00864	0.547855	0.744088
LOC93444	-0.01381	0.547886	0.744088
AC007365.	-0.01437	0.547925	0.744093
ANKFN1	0.003835	0.548085	0.744264
CTB-174D:	0.001003	0.548209	0.744386

POP1	-0.07659	0.548257	0.744404
KIAA1109	-0.01097	0.548294	0.744407
LOC10272	0.002096	0.548349	0.744437
TNMD	-0.00925	0.548536	0.744643
ZNF717	-0.02979	0.548625	0.74466
TAS2R8	-0.00187	0.548657	0.74466
RP11-171I	-0.00148	0.548709	0.74466
GRIA2	-0.00151	0.548715	0.74466
NUBP2	-0.05422	0.548788	0.74466
METTL23	0.034467	0.548807	0.74466
B4GALT7	-0.04407	0.548819	0.74466
CPA6	-0.0059	0.548824	0.74466
GPR64	-0.00352	0.548932	0.74474
MCF2L-AS	0.019935	0.548951	0.74474
TTLL5	-0.02867	0.549162	0.744979
STK17B	0.124143	0.549208	0.744995
TPSD1	-0.01692	0.549272	0.745035
FUBP1	0.058198	0.549405	0.745169
CRYBA2	-6.43E-04	0.549753	0.745594
YPEL1	0.046711	0.54982	0.745638
MAML3	0.012442	0.549897	0.745694
LOC10192	-0.01494	0.54993	0.745694
MON1A	0.066855	0.549997	0.745738
ST6GALNA	-0.15322	0.550171	0.745927
FGF10	-0.00116	0.550265	0.746008
ANG	0.052731	0.550372	0.746107
FAM101B	0.071039	0.550445	0.746129
LOC10050	0.072218	0.550458	0.746129
FAM171A2	-6.34E-04	0.550521	0.746168
C2orf71	-7.73E-04	0.550657	0.746279
MAGEB2	0.002555	0.550672	0.746279
FAM175A	0.073858	0.550762	0.746355
INTS6	0.040247	0.550796	0.746355
ZNF271	-0.05618	0.550968	0.746541
COL6A3	0.134021	0.55112	0.7467
STS	0.020695	0.551252	0.746794
SLC22A23	-0.01665	0.551259	0.746794
ANKUB1	-0.00106	0.551364	0.74689
WDR20	0.067187	0.551421	0.746921
LOC10272	-0.00174	0.551472	0.746943
IGSF9B	0.005914	0.55169	0.747191
DDX11	0.053323	0.551875	0.747381
GUCA1B	-0.02959	0.551905	0.747381
MAFK	0.046508	0.551934	0.747381
PANK3	0.032412	0.551971	0.747386
LOC44152	0.049465	0.55205	0.747445
LOC64652	0.002254	0.552131	0.747477
LOC10192	-0.00807	0.552142	0.747477
BORA	-0.07499	0.55221	0.747522
ZRANB3	0.02769	0.552266	0.747546
PTGER1	-0.00558	0.552297	0.747546
LHX5	-0.00219	0.552351	0.747572
FAM46A	-0.08129	0.552408	0.747603
CATSPERD	-5.84E-04	0.552545	0.747705
POLR2J2	0.037086	0.552552	0.747705
DBR1	-0.06766	0.552697	0.747853
RNF215	-0.00154	0.552993	0.748208
ZCCHC17	0.042077	0.553036	0.748219

LRFN2	0.004795	0.553075	0.748226
PABPC3	-0.0308	0.553129	0.748229
CDK20	0.040089	0.553147	0.748229
CCER1	0.005667	0.553234	0.7483
ARPP21	0.071602	0.553583	0.748726
CYP39A1	-0.04604	0.553649	0.748751
WBSCR28	-5.99E-04	0.553706	0.748751
C9orf37	-0.0454	0.553706	0.748751
SEMA6A	0.025789	0.553988	0.749086
ZNF781	-0.0277	0.554073	0.749115
RRP9	-0.07032	0.554079	0.749115
LRRC59	-0.00227	0.554123	0.749129
GOLGA6A	-0.00543	0.554261	0.749236
FAS-AS1	0.003716	0.554272	0.749236
YWHAEP7	-0.00196	0.554321	0.749255
LOC10028(	-0.04219	0.55446	0.749397
LOC10192(	0.002413	0.554532	0.749447
KIAA1033	-0.07401	0.554945	0.749958
PON3	0.004624	0.555029	0.750025
AFAP1	-0.03517	0.55507	0.750034
FAAH2	-0.04016	0.55513	0.750068
MAGEB4	0.00528	0.555254	0.750189
CEP70	-0.07746	0.555398	0.750337
KLHL6	0.058917	0.555508	0.750438
SACS-AS1	-0.00928	0.55567	0.750551
SGMS1	-0.10946	0.55573	0.750551
CDC45	0.09505	0.555755	0.750551
LOC10050(	-0.00869	0.55576	0.750551
SRSF12	0.030426	0.555765	0.750551
KRT8	-0.06236	0.555908	0.750698
H2AFJ	0.054614	0.556056	0.75085
KIF18B	0.096261	0.556147	0.750919
PTK2	0.074665	0.556211	0.750919
HRCT1	-0.01123	0.556211	0.750919
DRP2	-0.00317	0.556277	0.750962
SFRP5	0.054264	0.55633	0.750986
CHST11	-0.05604	0.556462	0.7511
LOC64624(	5.99E-04	0.556483	0.7511
RN7SKP15(	-7.45E-04	0.556553	0.751147
CCR3	0.014051	0.55667	0.751258
WNT8B	-0.02054	0.556727	0.751289
TMEM132E	-5.31E-04	0.556778	0.75131
AC013733.	0.00111	0.556874	0.751393
TEX38	-0.00922	0.556914	0.7514
LOC10013(	-0.02316	0.557014	0.751488
LOC10050(	0.001571	0.557142	0.751584
ALDH18A1	-0.05169	0.557155	0.751584
DNAJC6	0.072596	0.557191	0.751587
LIM2	0.005963	0.557294	0.751592
DERL2	0.043558	0.557376	0.751592
CD19	0.095179	0.557388	0.751592
ALCAM	0.085282	0.557395	0.751592
C3orf30	0.003145	0.557418	0.751592
FAM83C	0.00952	0.557431	0.751592
GPATCH3	0.063374	0.557464	0.751592
SYN3	-0.00481	0.55749	0.751592
NCAPH	0.102875	0.557508	0.751592
ST7-AS1	0.067203	0.557576	0.751637

KIF18A	0.088521	0.557776	0.751816
KIF2C	0.102495	0.557778	0.751816
ZBED2	-7.39E-04	0.55797	0.752028
LOC64373:	0.026904	0.558017	0.752044
UMODL1	0.159597	0.558165	0.752163
C14orf39	-0.02443	0.558182	0.752163
ZMPSTE24	0.035455	0.558208	0.752163
ULBP3	0.00591	0.558468	0.752407
IGFALS	-0.03368	0.558474	0.752407
MAPK11	-0.01032	0.558501	0.752407
C20orf202	0.011986	0.558529	0.752407
LYRM7	-0.0593	0.558599	0.752455
LOC40122(	-7.41E-04	0.558638	0.752461
VN1R3	-5.63E-04	0.55873	0.752515
RP11-210M	0.014034	0.558748	0.752515
LOC10192'	-0.0355	0.558829	0.752577
PCDHB4	-0.01536	0.558988	0.752715
CPS1	0.049608	0.559025	0.752715
RP11-473I.	0.066353	0.559036	0.752715
CBX1	0.050846	0.55916	0.752836
COL4A6	-0.00618	0.559205	0.752836
LINC01281	-0.00234	0.55923	0.752836
HIRIP3	0.047931	0.559296	0.752878
DQ592442	-0.10762	0.559368	0.752929
SPSB1	-0.02843	0.559537	0.753095
ELMOD2	0.030366	0.559562	0.753095
TMEM74B	0.002682	0.559607	0.75311
IGF2BP1	5.25E-04	0.559678	0.753158
LOC40105:	-0.01367	0.559724	0.753173
PRSS55	-0.00262	0.559765	0.753182
SOSTDC1	0.078025	0.559899	0.753296
DDX4	5.48E-04	0.559953	0.753296
OR8G1	-0.00281	0.559954	0.753296
LOC28424(	-5.74E-04	0.560251	0.753649
CSMD3	0.053976	0.56039	0.753747
UGT1A6	-6.70E-04	0.560394	0.753747
LOC10192'	-0.00559	0.560543	0.753867
ZNRF3	0.085845	0.560584	0.753867
MAF1	0.062907	0.560588	0.753867
CCR4	6.21E-04	0.56066	0.753917
ANP32D	-0.00543	0.560706	0.753933
FBXL13	0.025915	0.561092	0.75438
SRRD	0.072818	0.561108	0.75438
SOD3	-0.02552	0.561169	0.754414
DTX2P1-U	0.024052	0.561212	0.754426
RP11-700H	-0.00652	0.56129	0.754484
SETD3	-0.02671	0.561396	0.754579
CMAHP	-0.09299	0.561456	0.754613
MARK3	-0.03783	0.56149	0.754613
LEPROTL1	0.046545	0.561714	0.754866
PTPN9	0.022692	0.561882	0.755045
PGM5	0.027962	0.562052	0.755227
RTN4RL1	-0.01598	0.562327	0.755447
TIAL1	0.036069	0.562333	0.755447
RMDN1	-0.04272	0.56235	0.755447
ERO1LB	-0.07505	0.562376	0.755447
RP3-525N:	-0.11898	0.56239	0.755447
RHBDD3	0.035212	0.562483	0.755525

ATG9B	-0.01149	0.562577	0.755604
ATP12A	-0.00395	0.563057	0.756201
MORC1	-0.03758	0.563123	0.756201
SCML4	-0.03419	0.563126	0.756201
DNAH10	-0.00454	0.563164	0.756205
AC090627.	-0.00628	0.563246	0.756269
WISP1	0.019202	0.563369	0.756343
GTSE1-AS1	0.004029	0.563371	0.756343
LOC28493(	-7.97E-04	0.563528	0.756507
C6orf141	-0.07843	0.56361	0.756525
SLC16A8	-0.02605	0.563693	0.756525
GDF3	-0.016	0.563714	0.756525
LOC10013	0.002038	0.563718	0.756525
CTD-2611(	-0.00343	0.563722	0.756525
LOC10192!	5.55E-04	0.563752	0.756525
HIST1H3A	0.02475	0.563862	0.756604
LOC14700	-0.00269	0.563911	0.756604
CYP3A7-C	-5.14E-04	0.563915	0.756604
ARHGAP9	-0.05558	0.563971	0.756631
HERC2P7	0.004854	0.564163	0.756763
NKAPP1	-0.04687	0.564188	0.756763
AGPAT4	-0.06098	0.564225	0.756763
LOC10192	0.005569	0.564227	0.756763
AP000253.	-0.01659	0.564274	0.756763
LOC15727	-0.00197	0.564279	0.756763
C8orf48	-0.0308	0.564382	0.756855
DHX34	-0.01807	0.564432	0.756876
EXD2	-0.06443	0.564719	0.757213
ZSCAN12	-0.01238	0.56478	0.757237
DUS2	-0.04264	0.564839	0.757237
MYLK3	-9.65E-04	0.564869	0.757237
SPRED2	-0.00778	0.564876	0.757237
ZNF697	-0.04048	0.564943	0.757274
GLTSCR1L	0.045778	0.56498	0.757274
KLHDC9	-0.0261	0.565009	0.757274
RARS	0.03727	0.565162	0.757431
VSIG10	-0.02005	0.565259	0.757488
RP11-285E	-0.00682	0.565274	0.757488
MIP	-0.01545	0.565367	0.757566
LOC10192	-6.79E-04	0.565455	0.757637
SNRNP25	0.074944	0.565511	0.757665
HOTAIRM1	0.051352	0.565618	0.757684
MRPL45	0.04839	0.565684	0.757684
CD1E	0.028243	0.565694	0.757684
KRT81	-0.00475	0.565701	0.757684
TIMM17A	0.043702	0.565731	0.757684
LOC10192	-0.00213	0.565841	0.757684
ELP4	-0.05343	0.56586	0.757684
DDAH1	-0.0486	0.565887	0.757684
SLC35D2	-0.05146	0.565891	0.757684
C18orf61	-0.01206	0.565897	0.757684
GRWD1	-0.04722	0.56591	0.757684
RP11-400N	-0.00563	0.565956	0.757699
OR2J3	-7.62E-04	0.566038	0.757755
GPM6A	-0.0993	0.566083	0.757755
PCDHB15	-0.03523	0.566133	0.757755
ARHGAP2(	0.016747	0.566184	0.757755
HSPA4L	0.089936	0.566203	0.757755

RSPH9	-0.01489	0.566237	0.757755
TRPV3	-0.00787	0.566262	0.757755
HNRNPDL	0.05567	0.566336	0.757755
MAT1A	0.00691	0.566363	0.757755
KCNMB3	0.047117	0.566369	0.757755
NDUFV2-A	-0.05156	0.566385	0.757755
OR2J2	0.002321	0.566418	0.757755
CRYGC	0.004732	0.566487	0.7578
LOC10192	0.006655	0.566609	0.757917
SFMBT1	0.052148	0.566843	0.758181
NOL7	0.03757	0.566877	0.758181
MBOAT1	0.068954	0.567019	0.758325
USP36	-0.05336	0.567091	0.758329
ZNF518B	0.043991	0.567092	0.758329
HNRNPD	-0.04557	0.567176	0.758394
KIR2DS2	0.004363	0.567237	0.758429
STAU2-AS	-5.56E-04	0.567411	0.75861
RSRC2	0.04209	0.567447	0.75861
NCAM1	-7.95E-04	0.567477	0.75861
SIX3	0.002377	0.567633	0.758772
PNLDC1	-0.03215	0.567767	0.758896
PGR	0.017594	0.567796	0.758896
TOB1-AS1	-0.02868	0.56791	0.759001
JAKMIP3	-0.00421	0.56805	0.759141
RNF40	-0.04555	0.568121	0.759189
RP11-394I	-0.01253	0.568183	0.759226
LOC10192	0.006175	0.568224	0.759233
OVCH1-AS	-0.014	0.568385	0.759401
TMEM39B	0.065391	0.568601	0.759644
RP5-1068E	-0.0165	0.568739	0.759781
KAAG1	-0.00131	0.568781	0.75979
LINC00965	-0.08901	0.568941	0.75993
WNT1	-0.00352	0.568956	0.75993
HSD17B7P	0.01646	0.569095	0.760068
C12orf77	5.13E-04	0.569162	0.760112
CYYR1	-0.12793	0.569232	0.760157
S100Z	0.116146	0.569275	0.760168
RP11-140I	-0.09321	0.569398	0.760286
CAPN15	-0.03121	0.569523	0.7604
LINC01351	7.78E-04	0.569554	0.7604
PYDC1	-0.00251	0.56959	0.760402
C3P1	-0.00233	0.569716	0.760523
ITGAV	-0.0815	0.569763	0.760538
WDR66	-0.00284	0.569965	0.760727
POU3F4	-0.0028	0.569974	0.760727
UBXN10-A	0.034197	0.570156	0.760923
EVI2B	-0.06966	0.570234	0.76098
FNBP1L	-0.08014	0.570496	0.761099
REG1A	-0.00951	0.570499	0.761099
MED24	-6.86E-04	0.570572	0.761099
CPLX3	-0.01094	0.57061	0.761099
ACPT	-0.02722	0.570614	0.761099
HHEX	0.064414	0.570615	0.761099
LOC10050	-0.00298	0.570637	0.761099
RMI1	-0.06774	0.570644	0.761099
SERTAD4	-6.47E-04	0.57075	0.761099
CTD-2310I	0.005324	0.570765	0.761099
ZNF517	-0.00491	0.570805	0.761099

GKN1	0.002146	0.570805	0.761099
TPTE	-0.00112	0.570815	0.761099
CSTL1	-7.66E-04	0.570816	0.761099
TFAP2D	-0.00726	0.570942	0.761196
LOC10028!	-0.00644	0.570959	0.761196
MFSD4	-0.01079	0.571016	0.761226
C10orf91	0.005443	0.571058	0.761235
MEGF8	0.051789	0.571129	0.761283
IL5RA	0.013028	0.571261	0.761412
PODN	-0.00396	0.571423	0.761576
RP5-1031E	0.011557	0.571455	0.761576
LOC10192	-0.03759	0.571511	0.761605
IL22	0.0024	0.57168	0.761702
LOC10192	8.17E-04	0.571686	0.761702
CELF3	-0.01506	0.57169	0.761702
ACE2	-0.01678	0.571933	0.761909
NR4A1	0.09046	0.571945	0.761909
USP46	-0.05192	0.571951	0.761909
RP11-108F	-0.00803	0.572038	0.761978
LOC10272	-0.01343	0.572182	0.762089
TAAR2	-6.79E-04	0.572191	0.762089
LOC10013	-0.06139	0.572233	0.762097
VAV2	0.026852	0.572279	0.762112
LOC10099	4.82E-04	0.572517	0.762308
ADH1B	0.007563	0.572537	0.762308
C9orf47	0.014473	0.572552	0.762308
CD200	-0.11498	0.572567	0.762308
ABCC8	0.003064	0.572871	0.762609
DHH	0.002176	0.572922	0.762609
AGTR1	0.018821	0.572974	0.762609
KNSTRN	0.093574	0.573004	0.762609
LEMD1-AS	-0.00259	0.573054	0.762609
RP11-1L12	0.003781	0.573057	0.762609
THSD7A	-0.14525	0.573062	0.762609
SETD1B	0.038034	0.573075	0.762609
ARHGEF26	0.052031	0.573142	0.762651
KRTAP9-8	-5.30E-04	0.573218	0.762705
DMBX1	0.002751	0.573269	0.762727
TMEM87B	-0.05074	0.573321	0.762749
MTSS1L	0.011969	0.573402	0.762777
FAM204A	-0.034	0.573412	0.762777
SLURP1	-8.44E-04	0.573701	0.763114
CLDN14	-0.01503	0.573771	0.76316
CHRNA3	-7.45E-04	0.573838	0.763202
GPR132	0.035751	0.573877	0.763207
ESX1	0.004879	0.574034	0.763369
LOC10192	-5.84E-04	0.574158	0.763488
ALDH4A1	0.029322	0.574261	0.763577
RP11-1017	0.003187	0.5743	0.763581
PXYLP1	-0.0528	0.574334	0.763581
ZC2HC1B	-6.49E-04	0.574422	0.763609
DNAH17	-0.00375	0.574452	0.763609
PGAM2	-0.00466	0.574473	0.763609
SMARCE1	-0.04332	0.574496	0.763609
LOC10192	-0.00562	0.574816	0.763987
PRSS22	-0.009	0.57488	0.764025
LINC01019	-5.86E-04	0.574929	0.764044
NSD1	-0.03379	0.575188	0.76434

TCAM1P	-0.12643	0.575351	0.764491
NBEAP1	0.001401	0.575416	0.764491
SLC37A4	0.055643	0.575425	0.764491
NDUFA9	0.052873	0.575461	0.764491
HHATL	-0.00149	0.57549	0.764491
LRRIQ1	0.003532	0.575513	0.764491
MYH2	-0.01101	0.575611	0.76455
WDR76	0.063206	0.575628	0.76455
RP11-203E	0.009874	0.57574	0.764652
TMEM30A	-0.06849	0.575826	0.76472
CD96	-0.11065	0.575982	0.764879
FGF8	-0.00318	0.57608	0.764962
STEAP1B	-0.02947	0.576218	0.765081
LAT2	-0.04052	0.576245	0.765081
IQCH-AS1	-0.05437	0.576283	0.765081
CYTIP	-0.09615	0.57631	0.765081
EPS8L3	-0.01563	0.576543	0.765304
COL28A1	-0.01016	0.576586	0.765304
NCOA6	0.026326	0.576613	0.765304
TMEM67	-0.04778	0.576635	0.765304
NDUFA8	0.058418	0.576655	0.765304
ASB7	0.038974	0.576806	0.765457
DGKQ	-0.02839	0.576846	0.765464
GRIP2	0.010936	0.576927	0.765501
CCDC168	-7.19E-04	0.576945	0.765501
MAPK8IP1	-0.00653	0.577045	0.765587
SPATA17	-0.00614	0.577118	0.765637
IL1RAPL1	-0.00315	0.577241	0.765744
LZTS1-AS1	-0.00549	0.57727	0.765744
SLC35D3	-0.14528	0.577708	0.766233
HOTTIP	-0.00918	0.577739	0.766233
TMEM214	0.049431	0.577745	0.766233
LOC646626	-0.02755	0.578025	0.766558
GABRA5	-0.00117	0.578141	0.766666
C7orf55	0.056986	0.578197	0.766671
LINC00494	0.010279	0.578216	0.766671
P2RY4	-0.00369	0.578312	0.766751
COPG2	-0.02819	0.578451	0.766889
LOC338694	-0.00286	0.578588	0.767024
PPFIA3	0.03294	0.57888	0.767364
ALKBH1	0.035434	0.578998	0.767473
CACNA1D	-0.03571	0.57909	0.767502
MAP3K6	-0.00119	0.579091	0.767502
C1orf159	0.018894	0.57917	0.76756
DUXAP10	0.080751	0.579237	0.767568
H3F3A	-0.05411	0.579258	0.767568
FUT3	-0.00269	0.579311	0.767568
C9orf85	-0.04436	0.579318	0.767568
SORCS1	-0.01555	0.579447	0.767692
PPP1R36	-0.02577	0.57959	0.767834
ITGA6	0.091121	0.579648	0.767865
GART	-0.05407	0.579791	0.767963
DPEP1	0.008538	0.579793	0.767963
PTGER3	0.028901	0.579999	0.768188
OR5V1	-5.43E-04	0.580226	0.768325
CNKSR2	-0.00806	0.580232	0.768325
MCHR1	-6.57E-04	0.580262	0.768325
C12orf54	-0.00298	0.580268	0.768325

KLK14	-0.01365	0.58028	0.768325
DNAJB7	-0.01853	0.580405	0.768444
SLC2A5	0.075014	0.58062	0.768681
METTL22	0.033317	0.580766	0.768827
ERVFH21-1	-0.01204	0.580833	0.768869
RPL26L1	0.040617	0.580895	0.768885
CXorf30	-0.00195	0.580915	0.768885
INTS4	-0.01894	0.580991	0.768894
CAPN14	-0.04737	0.581021	0.768894
HPGD	-0.13402	0.581052	0.768894
MGC12488	0.081348	0.581064	0.768894
LINC00672	-0.04398	0.581183	0.769004
CTA-254O	-5.93E-04	0.581317	0.769037
TUBB4A	-0.01492	0.581331	0.769037
SERP2	9.29E-04	0.581343	0.769037
MTMR7	-5.20E-04	0.58135	0.769037
MPC2	0.052843	0.5814	0.769056
C1QTNF2	-6.40E-04	0.581537	0.769191
LOC10192	-0.03639	0.581574	0.769192
XRRA1	0.013743	0.581683	0.769289
KIAA0040	-0.04366	0.581755	0.769321
PACSIN1	-0.0501	0.581829	0.769321
GPR56	0.052681	0.581847	0.769321
ZNF232	-0.08736	0.581894	0.769321
NDUFB6	0.036895	0.581894	0.769321
NAPB	-0.04652	0.58192	0.769321
LOC10012	-0.011	0.582019	0.769405
ELSPBP1	0.001664	0.582072	0.769427
NFKBIZ	0.074542	0.58225	0.769616
CRCP	-0.03333	0.582329	0.769674
CXCL17	-0.00962	0.582407	0.769729
KCNS2	-8.15E-04	0.58267	0.77003
C1RL	0.058249	0.582985	0.7704
MMP28	-0.06251	0.583088	0.77045
SYTL3	-0.03451	0.583156	0.77045
ODF1	0.011762	0.583164	0.77045
COL14A1	-0.00621	0.583177	0.77045
GUCA1C	-4.93E-04	0.583249	0.77045
GPR18	0.095467	0.58325	0.77045
LOC37444	-0.0644	0.583294	0.77045
LOC44041	-5.21E-04	0.583443	0.77045
TAOK1	-0.03424	0.583444	0.77045
TRPA1	-6.42E-04	0.583458	0.77045
LIPI	0.002568	0.583526	0.77045
CACNG6	-0.00813	0.583577	0.77045
RPS6KB2	0.065685	0.583599	0.77045
CCDC42B	0.016556	0.583605	0.77045
SERPINA7	-6.52E-04	0.583612	0.77045
SWT1	-0.05505	0.583622	0.77045
UGT1A1	-5.22E-04	0.583703	0.77045
UBA52	0.035783	0.583705	0.77045
FLG	-0.00304	0.583729	0.77045
TRIB2	0.159595	0.583734	0.77045
RP11-91I8.	-5.38E-04	0.583882	0.770585
OR6B1	4.93E-04	0.583908	0.770585
LOC10028	0.099096	0.583979	0.770632
GLRA3	5.59E-04	0.584052	0.770652
KAT6A	0.04444	0.584099	0.770652

NPY	-0.12428	0.584101	0.770652
SMIM17	-0.05409	0.584171	0.770654
LOC10192	0.016093	0.584174	0.770654
ATP11A-A	-5.35E-04	0.584334	0.770818
C10orf126	-0.01062	0.58445	0.770925
FIGLA	-5.38E-04	0.584533	0.770987
CACNB2	0.067956	0.584598	0.771025
LOC10192	-6.88E-04	0.584644	0.771039
SRD5A2	0.006987	0.584726	0.771101
RC3H2	0.048449	0.584816	0.771172
SERPINB5	-0.00273	0.584938	0.771287
RP4-665N	-0.0049	0.584981	0.771296
CHD5	0.011493	0.585021	0.771302
MMAB	-0.05119	0.585121	0.771365
DUSP9	-0.01396	0.58514	0.771365
THAP7-AS	0.01073	0.585236	0.771445
PRDX2	-0.03608	0.585301	0.771482
LINC00343	7.68E-04	0.585426	0.771601
NKX2-2	-7.34E-04	0.585685	0.771896
SLC7A6OS	-0.03345	0.585737	0.771917
TBC1D21	-0.00453	0.585813	0.77193
VAC14	-0.02417	0.585819	0.77193
MLIP	-5.41E-04	0.585916	0.772011
PHACTR3	-0.03184	0.586014	0.772094
CYS1	-0.00317	0.586133	0.772203
SNTA1	0.027714	0.586316	0.772397
LOC28562	5.08E-04	0.586686	0.772838
PTDSS2	0.045211	0.586749	0.772874
LOC10192	0.001983	0.586912	0.773042
TMEM200	-0.00257	0.587209	0.773386
PCDHGC4	-0.00207	0.587283	0.773436
MPP2	-0.00976	0.587355	0.773473
RNASEH1-	-0.05942	0.587382	0.773473
HYLS1	0.08788	0.587527	0.773616
ARPC1B	-0.07198	0.587618	0.773689
POMT2	0.022094	0.587698	0.773748
ALS2CL	0.023479	0.587763	0.773786
LOC10192	0.001907	0.587798	0.773786
LOC10050	5.56E-04	0.588022	0.774034
LOC10050	9.40E-04	0.588168	0.774131
FAAH	0.075693	0.58818	0.774131
DEFB124	-0.02285	0.588204	0.774131
ABCF1	-0.04492	0.58842	0.774369
LINC00577	-0.01761	0.588557	0.7745
C12orf79	8.77E-04	0.588591	0.7745
AY927499	0.03344	0.588674	0.774515
ZBTB37	-0.02714	0.588708	0.774515
NPR1	0.022947	0.58871	0.774515
LINC00824	-0.01137	0.588866	0.774626
LOC10192	-5.97E-04	0.588866	0.774626
FLJ32790	-0.00264	0.588957	0.774689
NEURL2	-0.06223	0.588985	0.774689
NOV	-0.07409	0.589093	0.774783
MED12L	-0.02847	0.589197	0.774873
FZD3	-0.1243	0.589266	0.774917
MKNK1-AS	-5.06E-04	0.589423	0.775038
MT1M	0.05053	0.58943	0.775038
STX4	0.049136	0.589597	0.77521

SLITRK3	-0.00141	0.589774	0.775397
ZNF468	-0.06141	0.58997	0.775585
P4HA3	-0.00975	0.590016	0.775585
IGFBP7	0.075156	0.590057	0.775585
RP11-464F	0.022306	0.59006	0.775585
IER2	-0.0827	0.590139	0.775641
LOC10192!	-0.06081	0.590244	0.775679
ZNF649	0.017137	0.590248	0.775679
SEC23B	0.043083	0.590287	0.775679
SH2D4A	-0.0312	0.590319	0.775679
CDC42EP1	-0.00957	0.590347	0.775679
ECE2	0.00914	0.590424	0.775734
DBF4B	0.02525	0.590586	0.775818
HOXB3	0.065727	0.590613	0.775818
PHKG2	0.006857	0.590629	0.775818
ZSCAN1	-9.86E-04	0.590646	0.775818
LOC10192!	0.014431	0.590684	0.775818
RASD2	-0.00111	0.590704	0.775818
LANCL3	-0.0977	0.590763	0.775825
FGF9	-0.05885	0.590781	0.775825
EIF4E	-0.07255	0.590996	0.776061
CEACAM4	-0.03485	0.591229	0.776319
TC2N	-0.08686	0.591381	0.776472
POLQ	-0.09788	0.591514	0.776538
MIR646HG	0.100638	0.591533	0.776538
ATP5S	0.041691	0.591538	0.776538
SGSM1	-0.00515	0.591672	0.776638
WFIKKN2	0.003288	0.591704	0.776638
FOXA3	-0.04408	0.591804	0.776638
GPR157	0.028768	0.591814	0.776638
SFXN2	-0.04649	0.591818	0.776638
LMOD2	0.006283	0.59183	0.776638
LOC10192	0.00434	0.592005	0.776821
[truncated: 341,626 more chars]
