# Supplementary figures and images for: Comprehensive analysis of a necroptosis-associated diagnostic signature for myelodysplastic syndromes based on single-cell RNA-seq and bulk RNA-seq
Source: Hereditas. 2024 Oct 15;161:38. doi: 10.1186/s41065-024-00335-x (PMC11481600; doi:10.1186/s41065-024-00335-x)

**Supplementary Figure S1 Consensus clustering of the 13 DE-NRGs for k from 2 to 5.**


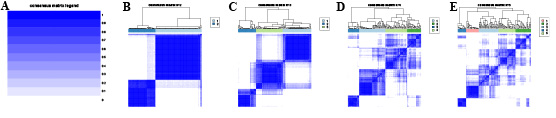

Supplement: Supplementary file 2 — Supplementary Material 2: Supplementary Figure S1. Consensus clustering of the 13 DE-NRGs for k from 2 to 5. [file 41065_2024_335_MOESM2_ESM.docx]
